# Identification of potential biomarkers of head and neck squamous cell carcinoma using iTRAQ based quantitative proteomic approach

**DOI:** 10.1016/j.dib.2018.05.100

**Published:** 2018-05-24

**Authors:** Niraj Babu, Sonali Mohan, Vishalakshi Nanjappa, Sandip Chavan, Jayshree Advani, Aafaque Ahmed Khan, Santosh Renuse, Aneesha Radhakrishnan, T.S. Keshava Prasad, Rekha V. Kumar, Jay Gopal Ray, Manjusha Biswas, Saravanan Thiyagarajan, Joseph A. Califano, David Sidransky, Harsha Gowda, Aditi Chatterjee

**Affiliations:** a Institute of Bioinformatics, International Tech Park, Bangalore 560066, India; b Manipal Academy of Higher Education, Manipal, Karnataka 576104, India; c School of Biotechnology, Kalinga Institute of Industrial Technology, Bhubaneswar 751024, India; d Department of Pathology, Kidwai Memorial Institute of Oncology, Bangalore 560029, India; e Department of Oral Pathology, Burdwan Dental College and Hospital, Burdwan, West Bengal 713101, India; f Division of Molecular Pathology, Mitra Biotech, Bangalore 560099, India; g Division of Cancer Biology, Mitra Biotech, Bangalore, Karnataka 560099, India; h Department of Otolaryngology-Head and Neck Surgery, Johns Hopkins University School of Medicine, Baltimore, MD 21231, USA; i Department of Surgery, UC San Diego, Moores Cancer Center, La Jolla, CA 92093, USA

**Keywords:** HNSCC, iTRAQ, Parallel reaction monitoring, Mass spectrometry, OKF6/TERT1

## Abstract

Head and neck squamous cell carcinoma (HNSCC) is one of the most common cancers in India. Despite improvements in treatment strategy, the survival rates of HNSCC patients remain poor. Thus, it is necessary to identify biomarkers that can be used for early detection of disease. In this study, we employed iTRAQ-based quantitative mass spectrometry analysis to identify dysregulated proteins from a panel of head and neck squamous cell carcinoma (HNSCC) cell lines. We identified 2468 proteins, of which 496 proteins were found to be dysregulated in at least two out of three HNSCC cell lines compared to immortalized normal oral keratinocytes. We detected increased expression of replication protein A1 (RPA1) and heat shock protein family H (Hsp110) member 1 (HSPH1), in HNSCC cell lines compared to control. The differentially expressed proteins were further validated using parallel reaction monitoring (PRM) and western blot analysis in HNSCC cell lines. Immunohistochemistry-based validation using HNSCC tissue microarrays revealed overexpression of RPA1 and HSPH1 in 15.7% and 32.2% of the tested cases, respectively. Our study illustrates quantitative proteomics as a robust approach for identification of potential HNSCC biomarkers. The proteomic data has been submitted to ProteomeXchange Consortium (http://www.proteomecentral.proteomexchange.org) via the PRIDE public data repository accessible using the data identifier - PXD009241.

**Specifications Table**

**Table:** 

Subject area	Biology
More specific subject area	Cancer proteomics
Type of data	Mass spectrometry raw files, tables, figures
How data was acquired	LTQ Orbitrap Velos mass spectrometer (Thermo Scientific, Bremen, Germany)
	Proteome Discoverer (Version 1.4) software suite (Thermo Scientific, Bremen, Germany)
	SEQUEST and Mascot search engines
	NCBI Human RefSeq protein database (Version 81)
Data format	Analysed output data
Experimental factors	Quantitative proteomic analysis of head and neck squamous cell carcinoma (HNSCC) cell lines
Experimental features	iTRAQ-based quantitative proteomic analysis of HNSCC cell lines, CAL 27, FaDu and JHU-O28 compared to immortalized normal oral keratinocytes, OKF6/TERT1. Validation of candidate proteins using parallel reaction monitoring (PRM), western blotting and immunohistochemistry (IHC)
Data source location	Bangalore, India
Data accessibility	Data is available here and via a web application (ProteomeXchange Consortium - http://www.proteomecentral.proteomexchange.org) via the PRIDE public data repository accessible using the data identifier - PXD009241

**Value of the data**

• This data set provides insights into proteomic alterations in HNSCC cell lines compared to normal oral keratinocyte cell line and validates candidate biomarkers using targeted proteomics approach using PRM and IHC.
• The data provides a useful resource to study proteins altered in HNSCC and will aid in identification of early detection biomarkers.

## 1 Data

The data represents iTRAQ-based quantitative proteomic analysis of HNSCC cell lines - CAL 27, FaDu and JHU-O28, compared to immortalized normal oral keratinocytes - OKF6/TERT1. A representative work flow of the study is depicted in Supplementary Fig. 1**A**. The study led to the identification of 2468 proteins, among which 496 proteins were dysregulated (fold change ≥ 2) in at least two out of three HNSCC cell lines. The complete list of proteins and peptides identified in this study is provided in Supplementary Table 1. Overexpression of RPA1 and HSPH1 identified in HNSCC cell lines in comparison to OKF6/TERT1 was validated using PRM and western blot. Further, the relative abundance of RPA1 and HSPH1 were assessed in HNSCC primary tissue compared to normal head and neck tissue samples using IHC.

## 2 Experimental design

### 2.1 Cell culture

FaDu and CAL 27 cell lines were purchased from ATCC (Manassas, VA). Immortalized, non-transformed normal oral keratinocytes OKF6/TERT1 was a kind gift from Dr. James Rheinwald at Brigham and Women׳s Hospital in Boston, MA [1]. As described earlier, FaDu and JHU-O28 were cultured in RPMI-1640 culture media supplemented with 10% fetal bovine serum and 1% penicillin/streptomycin solution [2]. CAL 27 and OKF6/TERT1 were cultured in keratinocyte serum-free media (KSFM) (Life Technologies, Grand Island, NY) supplemented with bovine pituitary extract (25 mg/ml), calcium chloride (0.4 mM), epidermal growth factor (0.2 ng/ml) and 1% penicillin/streptomycin solution. All the cell lines were grown and maintained at 37 °C in a humidified incubator with 5% CO_2_.

### 2.2 Sample preparation for LC–MS/MS analysis

Sample preparation for mass spectrometry analysis was performed as described previously [3]. Each cell line was grown to 70% confluency. The cells were then maintained in serum-free growth media for 12 h prior to harvesting for protein extraction. Cells were lysed using lysis buffer (2% SDS, 5 mM sodium fluoride, 1 mM β-glycerophosphate and 1 mM sodium orthovanadate in 50 mM triethyl ammonium bicarbonate (TEABC)). Protein concentration was determined using bicinchoninic acid (BCA) assay (Pierce, Waltham, MA) [4]. Filter-assisted sample preparation (FASP) was used to reduce SDS concentration in the sample as described earlier [5]. Briefly, equal amounts of protein from each cell line was reduced using tris(2-carboxyethyl)phosphine (TCEP) at 60 °C for 1 h and alkylated using methyl methanethiosulfonate (MMTS) for 20 min at room temperature. The samples were then digested using trypsin (Promega, San Luis Obispo, CA) overnight at 37 °C.

### 2.3 iTRAQ labeling and SCX fractionation

Peptides from each cell line were labelled using iTRAQ 8-plex kit (AB SCIEX, Washington, D.C.) in two technical replicates as per manufacturer׳s instructions. OKF6/TERT1 was labelled using iTRAQ label 113 and 114, CAL 27 with 115 and 116, FaDu with 117 and 118, and JHU-O28 with 119 and 121. The iTRAQ-labeled samples were pooled, dried and subjected to SCX fractionation, as described earlier [6]. Briefly, the samples were reconstituted in 25% acetonitrile and, adjusted to pH 2.8 using orthophosphoric acid. Sample fractionation was carried out on polysulfoethyl A column (200 × 4.6 mm, 5 μm, 200Å, PolyLC Inc., Columbia, MD) on Agilent 1260 HPLC platform. Peptide fractionation was carried out using a linear gradient of 0–60% of solvent B (10 mM KH_2_PO_4_, 350 mM KCl, 25% acetonitrile) over a period of 50 min at a constant flow rate of 350 μl/min. The samples were concatenated and pooled into 25 fractions, reconstituted in 0.1% formic acid and desalted using C18 Stage Tips (3 M Empore, St. Paul, MN).

### 2.4 LC–MS/MS

iTRAQ based LC–MS/MS analysis was carried out using LTQ-Orbitrap Velos mass spectrometer (Thermo Scientific, Bremen, Germany) interfaced with Easy-nLC II system (Thermo Scientific, Bremen, Germany). Samples were loaded on a trap column (75 μm × 2 cm, Magic C18Aq, 5 μm, 100Å) and washed using Solvent A (0.1% formic acid) at a flow rate of 3 μl/min. Samples were then resolved on an analytical column (75 μm × 12 cm, Magic C18 Aq, 3 μm, 100Å) at 350 nl/min flow rate using a linear gradient of 7–30% of Solvent B (0.1% formic acid in 95% acetonitrile) over 75 min. MS and MS/MS scans were at a mass resolution of 60,000 at 400 *m/z* and 15,000 at 400 *m/z*, respectively. Full MS scans were acquired in *m/z* range of 350–1800 *m/z*. For each cycle, twenty most abundant precursor ions with charge state ≥ 2 were sequentially isolated. Higher energy collision dissociation was used as the activation method with 40% normalized collision energy. Isolation width used was 2 *m/z*. Precursor ions with single charge or unassigned charges were rejected.

## 3 Data analysis

Proteome Discoverer (Version 1.4) software suite (Thermo Scientific, Bremen, Germany) was used for protein identification and quantification. Mass spectrometry data was queried against NCBI Human RefSeq protein database (Version 81) using Sequest and Mascot (version 2.4) search algorithms. Oxidation of methionine was set to dynamic modification in the search parameters while static modifications included - carbamidomethylation at cysteine and iTRAQ 8-plex modification at N-terminus of the peptide and lysine. Trypsin was specified as protease with one missed cleavage allowed. The precursor mass tolerance was set to 10 ppm and fragment mass tolerance was set to 0.1 Da. Decoy database searches were carried out to calculate the false discovery rate (FDR). 1% FDR cut-off at PSM, peptide and protein levels were considered for protein identification. The protein abundance ratios in HNSCC cell lines was obtained as follows: CAL 27 (115+116)/OKF6/TERT1 (113+114), FaDu (117+118)/OKF6/TERT1 (113+114) and JHU-O28 (119+121)/OKF6/TERT1 (113+114). The representative spectra of two proteins, RPA1 and HSPH1 is provided in Supplementary Fig. 1B.

## 4 Parallel reaction monitoring (PRM)

**Fig. 1:**
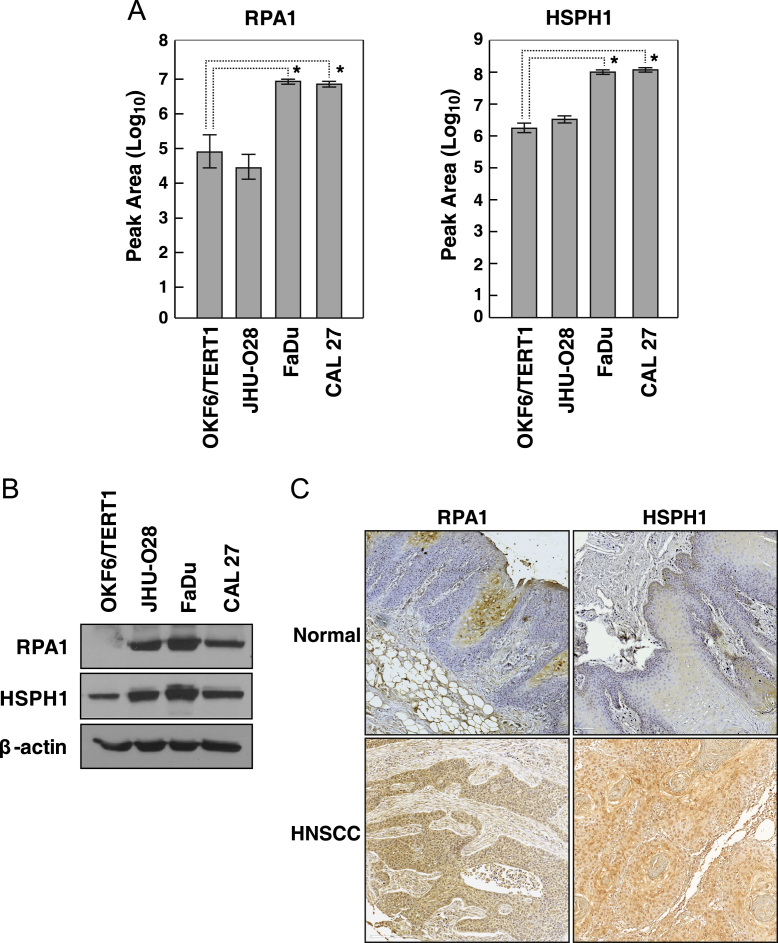
**A.** Bar graph depicting the relative abundance of RPA1 and HSPH1 in HNSCC cell lines (JHU-O28, FaDu and CAL27) and normal oral keratinocytes OKF6/TERT1 using parallel reaction monitoring (*p*-value ≤ 0.05). **B.** Western blot analysis to identify the relative expression of RPA1 and HSPH1 in HNSCC cell lines (JHU-O28, FaDu and CAL27), compared to normal oral keratinocyte (OKF6/TERT1). **C.** Representative sections from HNSCC tissues and normal head and neck tissue probed with anti-RPA1 and anti-HSPH1 antibodies.

Select proteins identified from the mass spectrometry-based proteomic analysis were validated using PRM on a Q-Exactive mass spectrometer (Thermo Fisher Scientific, Bremen, Germany). The full scan event was carried out between 120.0 and 1240.0 mass selection with ion injection time of 120 ms and a resolution of 17,500. The PRM scans were carried out in MS2 mode with 1 microscan set to a resolution of 17,500. The target AGC was set to 2 × 10^5^ with an isolation window of 0.7 *m/z*. The data was acquired using an isolation offset of 0.2 *m/z* with a first fixed mass of 120.0 *m/z*. The normalized collision energy was set to 27%. The data was acquired in triplicate and analysed on Skyline software (version 3.5) [7] (Fig. 1A, Table 1). Statistical significance (*p*-value) for the fold changes was calculated using two-sample *t*-test.

**Table 1:** List of peptides of RPA1 and HSPH1 used to study the relative abundance in HNSCC cell lines using parallel reaction monitoring.

**Protein name**	**Peptide sequence**	**Charge**	***m/z***	**Fold change**	***p*****-value**	**Fold change**	***p*****-value**	**Fold change**	***p*****-value**
				**(O28/OKF6/TERT1)**		**(FaDu/OKF6/TERT1)**		**(CAL 27/OKF6/TERT1)**	
RPA1	LFSLELVDESGEIR	3	536.2	0.2	0.31	50.2	5.3E−07	48.7	3.7E−05
HSPH1	AGGIETIANEFSDR	2	740.3	1.4	0.12	48.0	4.7E−04	52.4	2.5E−04

## 5 Western blot

Western blot was carried out as described previously [8] to study the expression of proteins - RPA1 and HSPH1, across HNSCC cell lines (CAL 27, FaDu and JHU-O28) compared to human normal oral keratinocyte (OKF6/TERT1) (Fig. 1B). Cell lines were cultured to 70% confluence and harvested in modified RIPA buffer containing 50 mM TrisHCl, 1% NP-40, 0.25% sodium deoxycholate, 150 mM NaCl, 2 mM EDTA, and 50 mM NaF, supplemented with protease inhibitor (Roche, Indianapolis, IN) and phosphatase inhibitor cocktail (Thermo Scientific, Bremen, Germany). Protein concentration was determined using BCA assay (Pierce, Waltham, MA) and 30 μg equivalent protein from each cell lysate was resolved on SDS-PAGE. Subsequently, the resolved proteins were transferred on a nitrocellulose membrane and probed using mouse monoclonal anti-RPA1 antibody (Cat # sc-48425; Santa Cruz Biotechnology, Dallas, TX) and rabbit polyclonal anti-HSPH1 antibody (Cat # HPA028675; Sigma, Darmstadt, Germany).

## 6 Immunohistochemistry (IHC)

Immunohistochemistry on commercially procured tissue microarrays (TMA) was performed as described previously [3]. Commercially available HNSCC TMAs were purchased from US Biomax (Cat # HN242a and HN483; US Biomax Inc., Derwood, MD). Briefly, the formalin fixed paraffin embedded tissue sections were deparaffinised, followed by antigen retrieval using citrate buffer (0.01 M Trisodium citrate buffer, pH 6) for 20 min. To inhibit the activity of endogenous peroxidases, the sections were quenched with blocking solution (methanol: water mixed in the ratio of 3:1) for 20 min. The sections were then washed using phosphate buffered saline and treated with 5% goat serum to block non-specific binding of antibodies. The sections were incubated in mouse monoclonal anti-RPA1 antibody (Cat # sc-48425; Santa Cruz Biotechnology) and polyclonal rabbit anti-HSPH1 antibody (Cat # HPA028675; Sigma) overnight at 4 °C. Following this, the sections were treated with secondary antibody conjugated with horseradish peroxidase (Bangalore Genei, India) before the addition of DAB chromogen (Dako, Glostrup, Denmark). Once the colored substrate was formed, the reaction was quenched using water and counterstained using hematoxylin solution. The tissue sections were examined under the microscope by two expert pathologists independently and scored based on the expression of proteins in the tissue sections. The tissue expression of RPA1 and HSPH1 were scored on a scale of 0–3, with 0 representing absence of staining, + 1 representing weak staining, + 2 representing moderate staining, and + 3 representing intense staining (Fig. 1C; Table 2A, Table 2B). Two-tailed Chi-square test was carried out to evaluate the *p*-value significance of RPA1 and HSPH1 expression in HNSCC.

**Table 2A:** Summary of immunohistochemical validation of RPA1 in HNSCC and normal tissues.

**Staining intensity**	**Normal**	**HNSCC**
0–1+(Negative–Weak)	11	48
2+–3+(Moderate–Strong)	1	9
*p*-value	5.04E−01	
Subcellular location of staining	Predominantly cytoplasmic

**Table 2B:** Summary of immunohistochemical validation of HSPH1 in HNSCC and normal tissues.

**Staining intensity**	**Normal**	**HNSCC**
0–1+(Negative–Weak)	11	40
2+–3+(Moderate–Strong)	1	19
*p*-value	9.3E−02	
Subcellular location of staining	Predominantly cytoplasmic	

## Appendix A Supplementary material

Supplementary material Fig. 1 **A.** Workflow of the iTRAQ-based quantitative proteomic analysis of OKF6/TERT1 and HNSCC cell lines. **B.** Representative MS/MS spectra of RPA1 and HSPH1. The peaks represent the *m/z* values and the relative intensities of fragment ions derived from tryptic peptide of the proteins. Inset, the peaks show the intensities of the different iTRAQ labels, representing relative abundance of the proteins identified in each cell line.
