# Supplementary material for: Identification of potential biomarkers of head and neck squamous cell carcinoma using iTRAQ based quantitative proteomic approach
Source: Data Brief. 2018 May 24;19:1124–30. doi: 10.1016/j.dib.2018.05.100 (PMC6139602; doi:10.1016/j.dib.2018.05.100)

Supplementary Figure 1

A

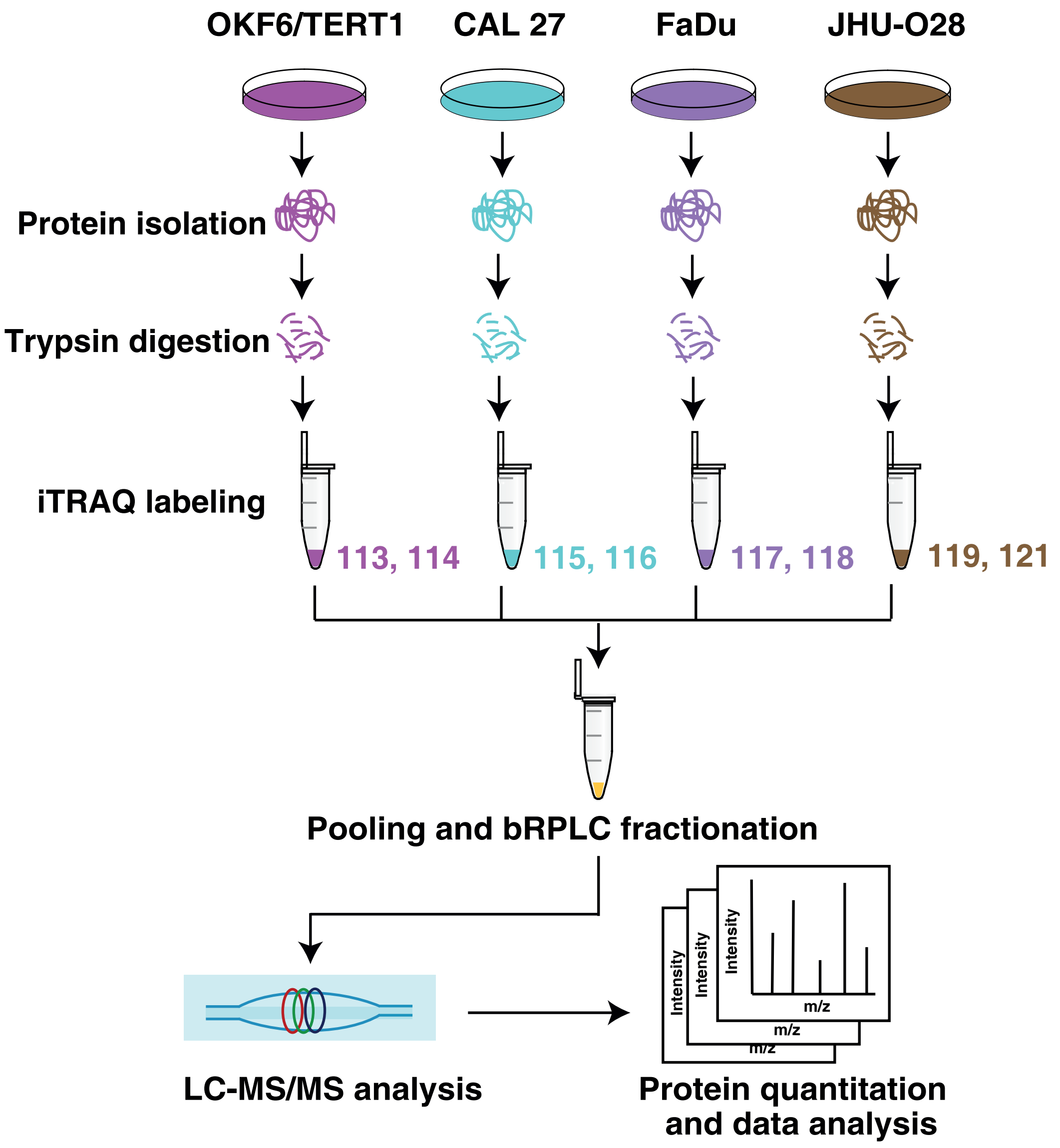

B

Replication protein A 70 kDa DNA-binding subunit (RPA1)

GWFDAEGQALDGVSISDLK

Heat shock protein 105 kDa (HSPH1)

SVLDAAQIVGLNCLR

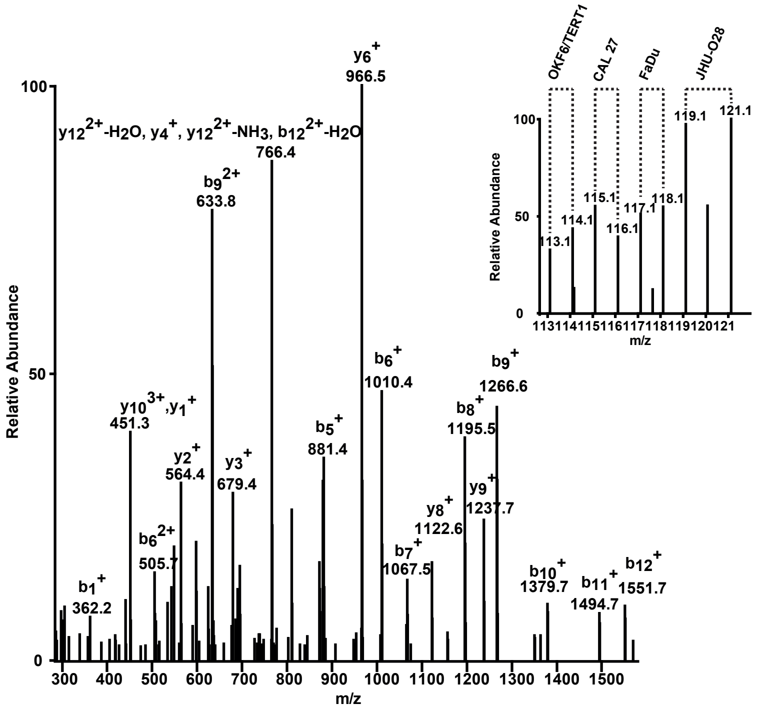

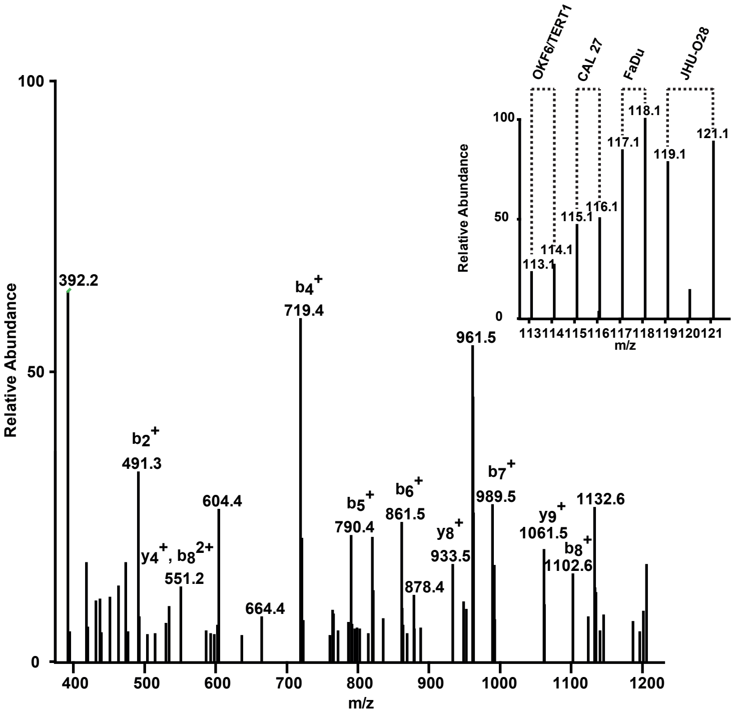

Supplement: Supplementary file 2 — Supplementary material Fig. 1 A. Workflow of the iTRAQ-based quantitative proteomic analysis of OKF6/TERT1 and HNSCC cell lines. B. Representative MS/MS spectra of RPA1 and HSPH1. The peaks represent the m/z values and the relative intensities of fragment ions derived from tryptic peptide of the proteins. Inset, the peaks show the intensities of the different iTRAQ labels, representing relative abundance of the proteins identified in each cell line. [file mmc2.pdf]
