# Supplementary material for: Identification of potential biomarkers of head and neck squamous cell carcinoma using iTRAQ based quantitative proteomic approach
Source: Data Brief. 2018 May 24;19:1124–30. doi: 10.1016/j.dib.2018.05.100 (PMC6139602; doi:10.1016/j.dib.2018.05.100)
Supplement: Supplementary file 3 — Supplementary material [file mmc3.pdf]

Babu *et al.*, 2018. Identification of potential biomarkers of head and neck squamous cell carcinoma using iTRAQ based quantitative proteomic approach

Supplementary Table 1: List of proteins identified and quantified in HNSCC cell lines using iTRAQ-based quantitative proteomics approach

Accesion	Gene_Symbol	Description	Refseq_Accession	ΣCoverage	Σ# Unique Peptides	Σ# Peptides	Σ# PSMs	# AAs	MW [kDa]	calc. pI	CAL 27/OKF6_TERT1	FaDu/OKF6_TERT1	JHU-O28/OKF6_TERT1	Sequence	# PSMs	# Proteins	# Protein Groups	Modifications	CAL 27/OKF6_TERT1	FaDu/OKF6_TERT1	JHU-O28/OKF6_TERT1	XCorr	Ionscore	# Missed Cleavages	MH+ [Da]	Charge	m/z [Da]	ΔM [ppm]	RT [min]
4501881	ACTA1	actin, alpha skeletal muscle	NP_001091.1	32.63	1	12	617	377	42.0	5.39	0.873	0.803	1.269																
														YPIEHGIITNWDDMEK	44	4	1	N-Term(iTRAQ8plex); K16(iTRAQ8plex)	0.824	0.781	1.27	4.27	31	0	2569.30185	4	719.133728	-4.878721698	36.11628167
														YPIEHGIITNWDDMEK	23	4	1	N-Term(iTRAQ8plex); M14(Oxidation); K16(iTRAQ8plex)	0.984	0.91	1.269	3.93	-	0	2585.30985	4	719.133728	-4.878721698	36.11628167
														YPIEHGIITNWDDMEK	1	4	1	N-Term(iTRAQ8plex); Y1(iTRAQ8plex); K16(iTRAQ8plex)	-	-	-	-	37	0	2873.51308	4	719.133728	-4.878721698	36.11628167
														IWHHTFYNELR	59	23	3	N-Term(iTRAQ8plex)	-	-	-	5.27	56	0	1819.94927	2	910.4796143	-1.428281869	32.213985
														IWHHTFYNELR	4	23	3	N-Term(iTRAQ8plex); Y7(iTRAQ8plex)	-	-	-	-	50	0	2124.14629	2	910.4796143	-1.428281869	32.213985
														AGFAGDDAPR	34	24	3	N-Term(iTRAQ8plex)	-	-	-	4.35	73	0	1280.65605	3	427.5570984	2.366454583	19.34597667
														AVFPSIVGRPR	138	20	3	N-Term(iTRAQ8plex)	-	-	-	4.24	42	0	1502.91629	3	501.639679	-4.290149398	23.05970333
														GILTLK	1	23	3	N-Term(iTRAQ8plex); K6(iTRAQ8plex)	-	-	-	-	26	0	1252.84558	3	418.2867126	0.553451648	23.60428667
														ILTER	2	27	5	N-Term(iTRAQ8plex)	-	-	-	-	28	0	935.57616	2	468.2917175	-7.036157716	17.75416
														DSYVGDEAQSK	34	6	2	N-Term(iTRAQ8plex); K11(iTRAQ8plex)	-	-	-	4.93	59	0	1806.92607	2	903.9666748	-3.834307739	16.92340333
														EITALAPSTMK	20	7	2	N-Term(iTRAQ8plex); M10(Oxidation); K11(iTRAQ8plex)	-	-	-	4.57	40	0	1786.0087	2	885.5181885	-0.04009362	30.370905
														EITALAPSTMK	32	7	2	N-Term(iTRAQ8plex); K11(iTRAQ8plex)	-	-	-	4.52	50	0	1770.0291	2	885.5181885	-0.04009362	30.370905
														DLTDYLMK	15	8	3	N-Term(iTRAQ8plex); K8(iTRAQ8plex)	-	-	-	3.55	36	0	1606.88591	2	803.9469604	-6.488959006	34.11513833
														IIAPPER	20	8	3	N-Term(iTRAQ8plex)	-	-	-	3.2	50	0	1099.67974	3	367.2290039	-4.785817094	19.22718
														DLTDYLMK	11	8	3	N-Term(iTRAQ8plex); M7(Oxidation); K8(iTRAQ8plex)	-	-	-	2.96	35	0	1622.8792	2	803.9469604	-6.488959006	34.11513833
														SYELPDGQVITIGNER	114	16	4	N-Term(iTRAQ8plex)	-	-	-	5.5	87	0	2095.09258	2	1048.049927	-2.278851268	43.30549667
														SYELPDGQVITIGNER	10	16	4	N-Term(iTRAQ8plex); Y2(iTRAQ8plex)	-	-	-	-	61	0	2399.29204	2	1048.049927	-2.278851268	43.30549667
														HQGVMVGMGQK	10	7	3	N-Term(iTRAQ8plex); K11(iTRAQ8plex)	-	-	-	6.08	64	0	1779.96953	2	890.4884033	-6.922185558	14.76235333
														HQGVMVGMGQK	15	7	3	N-Term(iTRAQ8plex); M5(Oxidation); K11(iTRAQ8plex)	-	-	-	4.94	41	0	1795.96048	2	890.4884033	-6.922185558	14.76235333
														HQGVMVGMGQK	30	7	3	N-Term(iTRAQ8plex); M5(Oxidation); M8(Oxidation); K11(iTRAQ8plex)	-	-	-	4.79	44	0	1811.96592	2	890.4884033	-6.922185558	14.76235333
4501891	ACTN1	alpha-actinin-1 isoform b	NP_001093.1	63.12	26	44	291	892	103.0	5.41	0.506	1.066	1.443																
														ETADTDTADQVMASFK	2	15	1	N-Term(iTRAQ8plex); K16(iTRAQ8plex)	0.386	0.971	1.387	6.52	84	0	2338.17974	2	1169.593506	4.44952909	34.71389333
														ISIEMHGTLEDQLSHLR	6	15	1	N-Term(iTRAQ8plex)	0.556	1.056	0.776	5.51	32	0	2283.19619	4	571.5545044	-4.705509729	37.793875
														ICDQWDNLGALTQK	12	15	1	N-Term(iTRAQ8plex); C2(Methylthio); K14(iTRAQ8plex)	0.289	1.21	1.588	5.42	69	0	2259.15898	2	1130.085449	-3.811358512	41.36717
														LLETIDQLYLEYAK	26	15	1	N-Term(iTRAQ8plex); K14(iTRAQ8plex)	0.201	1.15	1.614	5.37	78	0	2320.31284	2	1160.670532	3.334232844	63.20242833
														IDQLEGDHQLIQEALIFDNK	10	15	1	N-Term(iTRAQ8plex); K20(iTRAQ8plex)	0.944	0.931	1.787	5.25	62	0	2947.60215	3	983.2055664	1.205952695	51.00244833
														DDPLTNLNTAFDVAEK	2	15	1	N-Term(iTRAQ8plex); K16(iTRAQ8plex)	0.995	1.168	1.271	5.2	37	0	2371.24985	3	791.0881348	-4.360676341	34.48756333
														MVSDINNAWGCLEQVEK	4	15	1	N-Term(iTRAQ8plex); C11(Methylthio); K17(iTRAQ8plex)	0.247	1.387	2.058	5.17	78	0	2590.29912	2	1295.653198	2.574051902	55.435365
														LAILGIHNEVSK	9	15	1	N-Term(iTRAQ8plex); K12(iTRAQ8plex)	0.365	1.074	1.4	5.09	48	0	1902.16037	2	951.583252	-2.136526581	35.33993833
														DGLGFCALIHR	4	15	1	N-Term(iTRAQ8plex); C6(Methylthio)	1.447	0.962	1.375	4.99	66	0	1551.80838	3	517.940979	0.38758762	43.31566333
														VLAVNQENEQLMEDYEK	4	15	1	N-Term(iTRAQ8plex); K17(iTRAQ8plex)	0.671	0.682	0.799	4.89	44	0	2660.34977	3	887.4547729	-7.538746447	31.07963167
														LVSIGAEEIVDGNVK	4	17	1	N-Term(iTRAQ8plex); K15(iTRAQ8plex)	0.824	1.232	1.595	4.66	56	0	2151.2305	3	717.7483521	-8.213158763	33.63561333
														AGTQIENIEEDFRDGLK	4	15	1	N-Term(iTRAQ8plex); K17(iTRAQ8plex)	0.857	0.689	2.793	4.51	44	1	2543.34682	4	636.5921631	-3.699391749	41.18906833
														KDDPLTNLNTAFDVAEK	2	15	1	N-Term(iTRAQ8plex); K1(iTRAQ8plex); K17(iTRAQ8plex)	-	-	2.049	4.45	27	1	2803.54653	4	701.6420898	-4.99075184	38.32603
														IVQTYHVNMAGTNPYTTITPQEINGK	1	15	1	N-Term(iTRAQ8plex); K26(iTRAQ8plex)	-	-	-	4.14	-	0	3498.84121	4	875.4657593	-2.850678277	36.83329667
														ILAGDKNYITMDELR	6	15	1	N-Term(iTRAQ8plex); K6(iTRAQ8plex)	0.21	1.091	1.467	4.07	32	1	2360.2997	4	590.8324585	-1.036811984	34.91075167
														GISQEQMNEFR	4	15	1	N-Term(iTRAQ8plex)	0.428	1.024	1.332	3.87	51	0	1642.80632	2	821.9067993	-5.952793739	18.50795167
														MLDAEDIVGTARPDEK	1	15	1	N-Term(iTRAQ8plex); K16(iTRAQ8plex)	-	1.756	1.41	3.85	-	0	2368.25527	4	592.8192749	-3.653141085	32.26612167
														ACLISLGYDIGNDPQGEAEFAR	2	3	1	N-Term(iTRAQ8plex); C2(Methylthio)	0.598	0.34	0.564	3.78	25	0	2689.27585	3	897.0968018	-5.421636665	54.01129667
														ATLPDADKER	3	15	1	N-Term(iTRAQ8plex); K8(iTRAQ8plex)	0.382	0.881	1.341	3.74	27	1	1723.9666	2	862.4869385	-7.72112805	18.06540167
														ASFNHFDRDHSGTLGPEEFK	1	10	1	N-Term(iTRAQ8plex); K20(iTRAQ8plex)	-	-	-	3.7	-	1	2899.44472	4	725.6166382	-4.717608691	27.99860667
														QKDYETATLSEIK	1	15	1	N-Term(iTRAQ8plex); K2(iTRAQ8plex); K13(iTRAQ8plex)	1.92	-	1.964	3.2	-	1	2438.36757	4	610.3473511	-9.427160764	22.73990667
														VEQIAAIAQELNELDYYDSPSVNAR	2	15	1	N-Term(iTRAQ8plex)	-	2.445	1.038	3.08	43	0	3112.58457	4	854.9520264	1.991429432	61.73927333
														NYITMDELR	5	15	1	N-Term(iTRAQ8plex)	0.669	0.927	1.305	2.95	31	0	1458.74346	2	729.8753662	-8.901838136	22.40008833
														ASFNHFDR	2	20	1	N-Term(iTRAQ8plex)	0.413	0.48	0.563	2.92	39	0	1297.65305	3	433.2225342	-4.663812828	14.64217167
														RDQALTEEHAR	2	15	1	N-Term(iTRAQ8plex)	1.058	1.092	1.282	2.85	31	1	1629.85744	4	408.2198181	-2.226402732	9.840508333
														HRPELIDYGK	2	15	1	N-Term(iTRAQ8plex); K10(iTRAQ8plex)	0.39	1.071	1.393	2.73	26	0	1836.06276	4	459.7711487	2.15327419	17.07063
														VEQIAAIAQELNELDYYDSPSVNAR	1	15	1	N-Term(iTRAQ8plex); Y17(iTRAQ8plex)	-	-	-	-	47	0	3416.78628	4	854.9520264	1.991429432	61.73927333
														TINEVENQILTR	9	20	2	N-Term(iTRAQ8plex)	-	-	-	6.26	67	0	1733.97672	3	578.6637573	3.902833633	35.70732833
														AIMTYVSSFYHAFSGAQK	4	18	2	N-Term(iTRAQ8plex); K18(iTRAQ8plex)	-	-	-	5.81	38	0	2616.37685	4	654.8496704	1.055069962	63.23410333
														FAIQDISVEETSAK	8	24	2	N-Term(iTRAQ8plex); K14(iTRAQ8plex)	-	-	-	5.39	56	0	2146.17844	3	716.0623169	-5.975226604	27.650155
														KTFTAWCNSHLR	18	14	2	N-Term(iTRAQ8plex); K1(iTRAQ8plex); C7(Methylthio)	-	-	-	5.01	46	1	2118.12612	4	530.2869873	3.020140629	35.549485
														GYEEWLLNEIR	40	23	2	N-Term(iTRAQ8plex)	-	-	-	4.99	61	0	1725.90422	2	863.4557495	-4.142116619	40.94413667
														KHEAFESDLAAHQDR	4	23	2	N-Term(iTRAQ8plex); K1(iTRAQ8plex)	-	-	-	4.9	50	1	2362.22239	3	788.0789795	-5.776794565	17.487465
														LMLLLEVISGER	4	24	2	N-Term(iTRAQ8plex)	-	-	-	4.72	58	0	1677.00309	3	559.6725464	6.436845732	66.21151667
														TFTAWCNSHLR	4	17	2	N-Term(iTRAQ8plex); C6(Methylthio)	-	-	-	4.56	47	0	1685.82273	3	562.6124268	1.978098518	38.61585
														VGWEQLLTTIAR	15	20	2	N-Term(iTRAQ8plex)	-	-	-	4.5	49	0	1690.97367	3	564.3294067	-3.391143971	57.81293
														CQLEINFNTLQTK	6	25	2	N-Term(iTRAQ8plex); C1(Methylthio); K13(iTRAQ8plex)	-	-	-	4.38	40	0	2206.16709	3	736.0605469	-6.783223757	35.175255
														ELPPDQAEYCIAR	16	20	2	N-Term(iTRAQ8plex); C10(Methylthio)	-	-	-	4.2	44	0	1854.89629	2	927.9517822	-3.727683817	31.54306
														LSNRPAFMPSEGK	1	5	2	N-Term(iTRAQ8plex); K13(iTRAQ8plex)	-	-	-	3.88	-	0	2042.12563	4	511.2868652	-2.806018598	21.50268
														EGLLLWCQR	20	24	2	N-Term(iTRAQ8plex); C7(Methylthio)	-	-	-	3.87	47	0	1467.76616	3	489.9292908	-1.426129989	46.98549167
														ASIHEAWTDGK	1	14	2	N-Term(iTRAQ8plex); K11(iTRAQ8plex)	-	-	-	3.81	-	0	1822.97861	3	608.3310547	-6.687125909	25.401765
														DLLLDPAWEK	2	14	2	N-Term(iTRAQ8plex); K10(iTRAQ8plex)	-	-	-	3.76	36	0	1808.02384	3	603.3461304	-9.741816645	60.69756333
														LASDLLEWIR	13	20	2	N-Term(iTRAQ8plex)	-	-	-	3.7	79	0	1519.87745	3	507.2973328	-0.773916995	55.214405
														HTNYTMEHIR	3	23	2	N-Term(iTRAQ8plex); M6(Oxidation)	-	-	-	3.41	32	0	1621.79715	3	541.2705688	-5.353941468	10.12486833
														ALDFIASK	1	22	2	N-Term(iTRAQ8plex); K8(iTRAQ8plex)	-	-	-	-	32	0	1472.89515	3	491.6365662	1.24512348	34.84575667
														LDHLAEK	1	20	2	N-Term(iTRAQ8plex); K7(iTRAQ8plex)	-	-	-	-	32	0	1433.84697	2	717.427124	-7.169435323	20.96574333
4502027	ALB	serum albumin preproprotein	NP_000468.1	9.52	2	3	5	609	69.3	6.28	0.973	1.345	0.949																
														RPCFSALEVDETYVPK	2	1	1	N-Term(iTRAQ8plex); C3(Methylthio); K16(iTRAQ8plex)	0.973	1.345	0.949	3.62	29	0	2508.30118	3	836.7719116	-3.010279138	39.08045333
														SHCIAEVENDEMPADLPSLAADFVESK	1	1	1	N-Term(iTRAQ8plex); C3(Methylthio); M12(Oxidation); K27(iTRAQ8plex)	-	-	-	3.11	-	0	3587.7206	4	897.6856079	1.135668319	54.16814833
														KVPQVSTPTLVEVSR	2	2	2	N-Term(iTRAQ8plex); K1(iTRAQ8plex)	-	-	-	2.92	27	1	2248.33719	3	750.1172485	-5.047705731	25.92453833
4502049	AKR1B1	aldose reductase	NP_001619.1	45.25	12	12	63	316	35.8	6.98	0.877	1.693	4.612																
														AIGISNFNHLQVEMILNKPGLK	1	2	1	N-Term(iTRAQ8plex); K18(iTRAQ8plex); K22(iTRAQ8plex)	-	-	-	5.62	-	0	3348.9311	5	670.592041	-8.43932916	46.89262667
														VCALLSCTSHK	28	2	1	N-Term(iTRAQ8plex); C2(Methylthio); C7(Methylthio); K11(iTRAQ8plex)	0.977	2.489	6.388	4.73	51	0	1861.94785	4	466.2428589	-6.4865521	49.1221
														VFDFELSSQDMTTLLSYNR	2	2	1	N-Term(iTRAQ8plex)	0.71	0.427	1.049	4.7	64	0	2570.28421	3	857.4329224	3.557015133	63.17490667
														MPILGLGTWK	4	1	1	N-Term(iTRAQ8plex); K10(iTRAQ8plex)	0.712	1.366	4.084	4.23	45	0	1724.02182	2	862.5145264	-9.96227882	37.10459333
														TTAQVLIR	5	4	1	N-Term(iTRAQ8plex)	0.552	0.701	1.932	4.16	33	0	1205.75241	2	603.3756714	-6.53135834	20.46227833
														HIDCAHVYQNENEVGVAIQEK	1	1	1	N-Term(iTRAQ8plex); C4(Methylthio); K21(iTRAQ8plex)	-	-	-	3.89	-	0	3050.50844	4	763.3825684	-6.562428156	32.606255
														SPPGQVTEAVK	2	1	1	N-Term(iTRAQ8plex); K11(iTRAQ8plex)	-	-	-	3.87	51	0	1720.99125	3	574.3352661	-8.219803672	15.12675333
														MPILGLGTWK	2	1	1	N-Term(iTRAQ8plex); M1(Oxidation); K10(iTRAQ8plex)	1.475	1.751	2.247	3.22	27	0	1740.01847	2	862.5145264	-9.96227882	37.10459333
														LWCTYHEK	2	1	1	N-Term(iTRAQ8plex); C3(Methylthio); K8(iTRAQ8plex)	0.647	1.4	4.3	3.2	-	0	1733.8978	3	578.6374512	0.840860185	29.33524167
														LIQYCQSK	6	2	1	N-Term(iTRAQ8plex); C5(Methylthio); K8(iTRAQ8plex)	1.021	1.771	3.97	3.08	41	0	1636.88523	3	546.2999268	-9.692357351	55.89469333
														REELFIVSK	5	1	1	N-Term(iTRAQ8plex); K9(iTRAQ8plex)	1.063	1.85	7.31	3.04	29	1	1729.03122	3	577.0152588	-9.044476816	30.661195
														VAIDVGYR	2	1	1	N-Term(iTRAQ8plex)	0.9	3.439	13.159	-	29	0	1196.68657	2	598.8469238	-6.28423844	24.95606667
														DYPFHEEF	3	2	1	N-Term(iTRAQ8plex)	0.877	1.218	1.874	-	24	0	1387.63884	2	694.3230591	-6.017239996	29.75197333
4502101	ANXA1	annexin A1	NP_000691.1	59.54	19	19	141	346	38.7	7.02	1.737	4.254	2.695																
														QAWFIENEEQEYVQTVK	8	2	1	N-Term(iTRAQ8plex); K17(iTRAQ8plex)	1.484	3.943	2.326	7.22	70	0	2749.41777	3	917.1411133	-7.486957323	68.04704333
														GGPGSAVSPYPTFNPSSDVAALHK	15	2	1	N-Term(iTRAQ8plex); K24(iTRAQ8plex)	1.26	6.209	3.928	6.98	81	0	2964.56174	2	1482.783813	-2.452629063	34.370695
														MYGISLCQAILDETKGDYEK	13	2	1	N-Term(iTRAQ8plex); C7(Methylthio); K15(iTRAQ8plex); K20(iTRAQ8plex)	2.024	8.171	5.23	6.17	41	1	3235.68081	4	809.6756592	-0.258202616	63.38810833
														GLGTDEDTLIEILASR	12	2	1	N-Term(iTRAQ8plex)	2.027	4.647	2.553	5.84	91	0	2007.0927	2	1004.049988	0.736222068	58.60724
														MYGISLCQAILDETK	2	2	1	N-Term(iTRAQ8plex); M1(Oxidation); C7(Methylthio); K15(iTRAQ8plex)	-	-	-	5.42	41	0	2355.21585	3	785.7434692	-2.561478662	51.76613167
														GTDVNVFNTILTTR	5	2	1	N-Term(iTRAQ8plex)	1.841	4.518	2.863	5.17	51	0	1855.02536	2	928.0128174	-2.352081507	46.51056333
														GDRSEDFGVNEDLADSDAR	8	2	1	N-Term(iTRAQ8plex)	0.957	1.586	1.492	5.16	53	1	2372.08152	3	791.3653564	-3.775255615	20.38991667
														MYGISLCQAILDETK	4	2	1	N-Term(iTRAQ8plex); C7(Methylthio); K15(iTRAQ8plex)	1.834	6.31	5.981	5.11	40	0	2339.21134	3	785.7434692	-2.561478662	51.76613167
														GVDEATIIDILTK	17	2	1	N-Term(iTRAQ8plex); K13(iTRAQ8plex)	1.563	4.254	2.783	5	86	0	1996.16789	2	998.5875854	-5.403864872	42.95194833
														SEDFGVNEDLADSDAR	4	2	1	N-Term(iTRAQ8plex)	1.268	3.321	2.391	4.97	92	0	2043.93205	2	1022.469666	-4.343033465	27.344355
														GVDEATIIDILTKR	2	2	1	N-Term(iTRAQ8plex); K13(iTRAQ8plex)	-	-	-	4.74	34	1	2152.27505	4	538.8242188	-2.20983838	47.36934
														ALTGHLEEVVLALLK	17	2	1	N-Term(iTRAQ8plex); K15(iTRAQ8plex)	1.36	3.158	1.843	4.61	84	0	2214.36528	2	1107.687378	-0.335111779	66.48755667
														KGTDVNVFNTILTTR	9	2	1	N-Term(iTRAQ8plex); K1(iTRAQ8plex)	1.391	2.829	1.998	4.27	37	1	2287.31302	3	763.1091919	-4.384562282	38.44976
														NALLSLAK	2	2	1	N-Term(iTRAQ8plex); K8(iTRAQ8plex)	1.542	3.777	2.596	3.83	31	0	1437.91094	2	719.4591064	-9.739844814	26.636025
														TPAQFDADELR	6	2	1	N-Term(iTRAQ8plex)	1.891	5.136	3	3.72	48	0	1566.80425	2	783.9057617	-1.489964368	27.35150667
														DITSDTSGDFR	10	2	1	N-Term(iTRAQ8plex)	1.859	5.35	3.153	3.63	35	0	1517.74114	2	759.3742065	1.703298326	23.58094833
														SEIDMNDIK	2	2	1	N-Term(iTRAQ8plex); K9(iTRAQ8plex)	1.924	3.842	2.19	3.37	45	0	1672.89067	2	836.9489746	-7.746292966	19.96682667
														VLDLELK	2	2	1	N-Term(iTRAQ8plex); K7(iTRAQ8plex)	0.628	1.25	1.017	3.14	31	0	1437.91655	2	719.4619141	1.975222413	48.88600833
														CATSKPAFFAEK	1	2	1	N-Term(iTRAQ8plex); C1(Methylthio); K5(iTRAQ8plex); K12(iTRAQ8plex)	1.689	4.9	2.94	3.09	-	0	2258.22672	3	753.4137573	-7.675747169	25.78528333
														ALYEAGER	2	2	1	N-Term(iTRAQ8plex)	1.622	3.462	2.179	3.01	29	0	1212.64849	2	606.8278809	-3.410882297	17.13725
4502171	APRT	adenine phosphoribosyltransferase isoform a	NP_000476.1	30.00	4	4	8	180	19.6	6.02	0.669	1.440	1.767																
														LQAEVLECVSLVELTSLK	2	1	1	N-Term(iTRAQ8plex); C8(Methylthio); K18(iTRAQ8plex)	-	-	-	3.88	61	0	2628.4863	3	876.8336182	1.905637547	69.48242667
														DISPVLKDPASFR	1	2	1	N-Term(iTRAQ8plex); K7(iTRAQ8plex)	-	-	-	3.74	-	1	2053.17446	4	514.0490723	-7.69156144	29.22509667
														SFPDFPTPGVVFR	3	2	1	N-Term(iTRAQ8plex)	0.727	1.543	1.956	3.61	28	0	1769.94597	2	885.4766235	-3.893254259	48.39591333
														IDYIAGLDSR	2	2	1	N-Term(iTRAQ8plex)	0.482	0.976	1.18	3.14	41	0	1426.778	2	713.8926392	-4.464324289	37.954575

4502201	ARF1	ADP-ribosylation factor 1	NP_001649.1	58.56	6	9	45	181	20.7	6.80	0.847	1.808	1.788																
														QDLPNAMNAAEITDK	4	2	1	N-Term(iTRAQ8plex); K15(iTRAQ8plex)	0.794	1.535	1.471	5.6	59	0	2239.16837	3	747.0609741	-7.392113495	40.562095
														NISFTVWDVGGQDK	8	4	1	N-Term(iTRAQ8plex); K14(iTRAQ8plex)	0.893	1.971	1.842	5.43	71	0	2174.15508	2	1087.581177	-6.974849465	63.32097333
														QDLPNAMNAAEITDKLGLHSLR	2	2	1	N-Term(iTRAQ8plex); K15(iTRAQ8plex)	-	-	-	4.43	40	1	3015.64785	4	754.6674194	-0.932948993	41.91797667
														MLAEDELRDAVLLVFANK	5	4	1	N-Term(iTRAQ8plex); K18(iTRAQ8plex)	0.847	3.037	2.144	3.95	24	1	2655.50551	4	668.626709	-3.739583968	57.7521
														MLAEDELRDAVLLVFANK	7	4	1	N-Term(iTRAQ8plex); M1(Oxidation); K18(iTRAQ8plex)	-	-	-	3.8	38	1	2671.48501	4	668.626709	-3.739583968	57.7521
														MLAEDELR	1	4	1	N-Term(iTRAQ8plex)	0.717	0.98	0.994	3.1	-	0	1280.67827	2	648.8392944	-4.499253402	20.69607333
														LGLHSLR	2	2	1	N-Term(iTRAQ8plex)	1.138	1.702	2.051	3.04	35	0	1099.68006	3	367.2315369	-8.078671826	20.23064667
														MLAEDELR	1	4	1	N-Term(iTRAQ8plex); M1(Oxidation)	0.847	1.239	1.311	-	30	0	1296.67131	2	648.8392944	-4.499253402	20.69607333
														ILMVGLDAAGK	10	6	2	N-Term(iTRAQ8plex); K11(iTRAQ8plex)	-	-	-	4.97	49	0	1696.01639	2	848.5135498	-5.287857196	34.75682667
														HYFQNTQGLIFVVDSNDRER	2	6	2	N-Term(iTRAQ8plex)	-	-	-	4.4	30	1	2742.38369	4	686.3513794	-2.371514207	36.87686833
														LGEIVTTIPTIGFNVETVEYK	2	6	2	N-Term(iTRAQ8plex); K21(iTRAQ8plex)	-	-	-	3.76	41	0	2931.63919	3	977.8845825	-5.040521847	54.47209833
														HYFQNTQGLIFVVDSNDRER	1	6	2	N-Term(iTRAQ8plex); Y2(iTRAQ8plex)	-	-	-	-	28	1	3046.58491	4	686.3513794	-2.371514207	36.87686833
4502205	ARF4	ADP-ribosylation factor 4	NP_001651.1	42.22	2	5	26	180	20.5	7.14	0.771	1.229	1.531																
														NICFTVWDVGGQDR	7	1	1	N-Term(iTRAQ8plex); C3(Methylthio)	0.818	1.308	1.515	5.23	67	0	1959.93985	2	980.4726563	1.083493438	48.176595
														IQEVADELQK	4	1	1	N-Term(iTRAQ8plex); K10(iTRAQ8plex)	0.548	1.088	1.576	3.65	48	0	1781.01761	3	594.3440552	-5.002209184	56.14126833
														ILMVGLDAAGK	10	6	2	N-Term(iTRAQ8plex); K11(iTRAQ8plex)	-	-	-	4.97	49	0	1696.01639	2	848.5135498	-5.287857196	34.75682667
														HYFQNTQGLIFVVDSNDRER	2	6	2	N-Term(iTRAQ8plex)	-	-	-	4.4	30	1	2742.38369	4	686.3513794	-2.371514207	36.87686833
														LGEIVTTIPTIGFNVETVEYK	2	6	2	N-Term(iTRAQ8plex); K21(iTRAQ8plex)	-	-	-	3.76	41	0	2931.63919	3	977.8845825	-5.040521847	54.47209833
														HYFQNTQGLIFVVDSNDRER	1	6	2	N-Term(iTRAQ8plex); Y2(iTRAQ8plex)	-	-	-	-	28	1	3046.58491	4	686.3513794	-2.371514207	36.87686833
4502211	ARF6	ADP-ribosylation factor 6	NP_001654.1	15.43	1	1	1	175	20.1	8.95	-	-	-																
														NWYVQPSCATSGDGLYEGLTWLTSNYK	1	1	1	N-Term(iTRAQ8plex); C8(Methylthio); K27(iTRAQ8plex)	-	-	-	-	32	0	3707.82346	3	1236.612671	7.111219183	61.84274333
4502277	ATP1B1	sodium/potassium-transporting ATPase subunit beta 1	NP_001668.1	8.91	3	3	11	303	35.0	8.53	1.613	0.524	0.508																
														SYEAYVLNIVR	6	1	1	N-Term(iTRAQ8plex)	1.477	0.545	0.638	4.01	64	0	1630.90354	3	544.3060303	-4.34343879	47.299425
														EGKPCIIIK	2	1	1	N-Term(iTRAQ8plex); K3(iTRAQ8plex); C5(Methylthio); K9(iTRAQ8plex)	1.518	0.4	0.352	3.64	-	0	1959.19719	2	980.1022339	3.761159658	22.49049333
														FIWNSEK	3	1	1	N-Term(iTRAQ8plex); K7(iTRAQ8plex)	1.742	0.526	0.455	2.75	26	0	1531.87029	2	766.4407349	0.843285022	25.29393167
4502281	ATP1B3	sodium/potassium-transporting ATPase subunit beta 3	NP_001670.1	27.24	7	7	19	279	31.5	8.35	1.283	1.117	0.601																
														SLNQSLAEWK	2	1	1	N-Term(iTRAQ8plex); K10(iTRAQ8plex)	1.248	1.157	0.691	4.7	58	0	1784.0053	2	892.5062866	-6.157312332	28.24873333
														YRDQIPSPGLMVFPKPVTALEYTFSR	2	1	1	N-Term(iTRAQ8plex); K15(iTRAQ8plex)	-	-	-	4.38	-	1	3620.97181	5	725.0001831	-1.163301699	54.66134833
														LFIYNPTTGEFLGR	8	1	1	N-Term(iTRAQ8plex)	1.224	1.011	0.674	3.66	63	0	1932.04648	3	644.6870117	-3.524446132	50.98631
														DQIPSPGLMVFPKPVTALEYTFSR	1	1	1	N-Term(iTRAQ8plex); K13(iTRAQ8plex)	-	-	-	3.48	-	0	3301.81777	4	826.2098999	1.874249219	51.554065
														IIGLKPEGVPR	2	1	1	N-Term(iTRAQ8plex); K5(iTRAQ8plex)	1.363	1.104	0.501	3.25	25	0	1787.12834	2	894.0678101	-4.486416906	25.649715
														IDGSANLK	1	1	1	N-Term(iTRAQ8plex); K8(iTRAQ8plex)	1.298	1.187	0.661	3.17	-	0	1425.85832	2	713.4328003	4.315061205	13.85751167
														DQIPSPGLMVFPKPVTALEYTFSR	1	1	1	N-Term(iTRAQ8plex); M9(Oxidation); K13(iTRAQ8plex)	1.514	1.763	0.496	3.1	-	0	3317.8198	4	826.2098999	1.874249219	51.554065
														EVTVECK	2	1	1	N-Term(iTRAQ8plex); C6(Methylthio); K7(iTRAQ8plex)	1.286	1.056	0.451	3	28	0	1461.78996	2	731.3986206	-0.135251347	29.95464667
4502297	ATP5D	ATP synthase subunit delta, mitochondrial precursor	NP_001678.1	13.69	2	2	12	168	17.5	5.49	0.922	0.424	0.455																
														AQAELVGTADEATR	8	1	1	N-Term(iTRAQ8plex)	0.922	0.577	0.635	5.41	88	0	1735.91216	2	868.4597168	-0.399977756	24.775835
														IEANEALVK	4	1	1	N-Term(iTRAQ8plex); K9(iTRAQ8plex)	0.847	0.383	0.413	3.9	53	0	1594.95952	2	797.9833984	-1.843962159	31.35373667
4502303	ATP5O	ATP synthase subunit O, mitochondrial precursor	NP_001688.1	28.17	5	5	14	213	23.3	9.96	0.696	0.692	0.605																
														FSPLTTNLINLLAENGR	2	1	1	N-Term(iTRAQ8plex)	0.49	-	0.301	5.09	77	0	2177.2291	3	726.4145508	2.695074098	64.13625333
														YATALYSAASK	3	1	1	N-Term(iTRAQ8plex); K11(iTRAQ8plex)	0.617	0.507	0.486	4.38	51	0	1753.98198	2	877.4946289	-7.11529972	28.53187333
														LVRPPVQVYGIEGR	4	1	1	N-Term(iTRAQ8plex)	0.988	0.766	0.807	4.09	34	0	1887.10417	2	944.0557251	-4.052624542	28.00650333
														VAASVLNPYVK	3	1	1	N-Term(iTRAQ8plex); K11(iTRAQ8plex)	0.734	0.89	0.628	4.02	33	0	1769.07566	3	590.3634033	-1.41010875	44.63434
														YVDMSVK	2	1	1	N-Term(iTRAQ8plex); K7(iTRAQ8plex)	0.606	0.412	0.381	3.06	-	0	1449.82427	2	725.4157715	0.755297573	30.99609
4502419	BLVRB	flavin reductase (NADPH)	NP_000704.1	35.92	5	5	34	206	22.1	7.65	0.877	1.249	1.568																
														YVAVMPPHIGDQPLTGAYTVTLDGR	1	1	1	N-Term(iTRAQ8plex); M5(Oxidation)	-	2.767	8.645	5.07	-	0	2991.54325	3	997.8526001	-4.007716433	39.97387833
														HDLGHFMLR	7	1	1	N-Term(iTRAQ8plex)	0.712	1.444	2.287	5.02	57	0	1429.75881	3	477.2582703	-5.150345273	20.29777167
														TVAGQDAVIVLLGTR	2	1	1	N-Term(iTRAQ8plex)	0.896	1.223	3.686	4.99	59	0	1817.06987	2	909.0385742	-5.497577531	48.69772667
														YVAVMPPHIGDQPLTGAYTVTLDGR	1	1	1	N-Term(iTRAQ8plex)	1.122	3.131	8.07	4.9	-	0	2975.55338	3	997.8526001	-4.007716433	39.97387833
														VVACTSAFLLWDPTK	2	1	1	N-Term(iTRAQ8plex); C4(Methylthio); K15(iTRAQ8plex)	0.767	0.617	1.253	4.68	30	0	2305.25516	3	769.0899048	0.281446271	64.27999
														LQAVTDDHIR	13	1	1	N-Term(iTRAQ8plex)	0.912	1.223	1.489	4.31	58	0	1471.8097	3	491.274231	-6.068507617	19.86042
														HDLGHFMLR	8	1	1	N-Term(iTRAQ8plex); M7(Oxidation)	0.401	0.729	1.535	3.15	30	0	1445.75443	3	477.2582703	-5.150345273	20.29777167
4502491	C1QBP	complement component 1 Q subcomponent-binding protein, mitochondrial precursor	NP_001203.1	24.47	4	4	77	282	31.3	4.84	1.610	0.632	0.651																
														ALVLDCHYPEDEVGQEDEAESDIFSIR	49	1	1	N-Term(iTRAQ8plex); C6(Methylthio)	2.248	0.783	0.57	8.91	71	0	3429.56174	3	1143.858765	-4.426689424	73.77200667
														AFVDFLSDEIKEER	21	1	1	N-Term(iTRAQ8plex); K11(iTRAQ8plex)	1.573	0.595	0.633	4.18	87	1	2306.24956	2	1153.628784	0.602900466	47.78149667
														EVSFQSTGESEWK	2	1	1	N-Term(iTRAQ8plex); K13(iTRAQ8plex)	1.265	0.596	0.714	4.14	45	0	2122.07444	3	708.0296631	-7.952262136	38.59076333
														MSGGWELELNGTEAK	2	1	1	N-Term(iTRAQ8plex); K15(iTRAQ8plex)	1.347	0.757	1.012	4.14	41	0	2230.15372	3	744.0560913	-4.367691845	53.16321167
														ALVLDCHYPEDEVGQEDEAESDIFSIR	3	1	1	N-Term(iTRAQ8plex); C6(Methylthio); Y8(iTRAQ8plex)	0.96	0.242	-	-	30	0	3733.77968	3	1143.858765	-4.426689424	73.77200667
4502549	CALM2	calmodulin isoform 2	NP_001734.1	76.51	8	8	27	149	16.8	4.22	0.904	1.229	1.249																
														EADIDGDGQVNYEEFVQMMTAK	2	4	1	N-Term(iTRAQ8plex); M18(Oxidation); K22(iTRAQ8plex)	2.57	3.238	1.933	7.65	54	0	3114.50644	3	1033.498413	-3.258142687	43.837595
														EADIDGDGQVNYEEFVQMMTAK	2	4	1	N-Term(iTRAQ8plex); K22(iTRAQ8plex)	1.025	1.229	0.772	5.8	67	0	3098.48069	3	1033.498413	-3.258142687	43.837595
														EADIDGDGQVNYEEFVQMMTAK	2	4	1	N-Term(iTRAQ8plex); M18(Oxidation); M19(Oxidation); K22(iTRAQ8plex)	-	-	-	5.23	59	0	3130.48496	3	1033.498413	-3.258142687	43.837595
														EAFSLFDKDGDGTITTK	2	3	1	N-Term(iTRAQ8plex); K8(iTRAQ8plex); K17(iTRAQ8plex)	0.937	1.129	1.34	4.44	-	1	2757.48989	4	690.1279297	-6.358112162	35.8014
														VFDKDGNGYISAAELR	5	4	1	N-Term(iTRAQ8plex); K4(iTRAQ8plex)	0.853	1.272	1.175	4.25	38	1	2363.26943	4	591.5728149	-5.146012647	32.70620667
														DTDSEEEIREAFR	5	4	1	N-Term(iTRAQ8plex)	1.132	1.002	1.587	3.89	25	1	1900.91179	2	950.9595337	-3.824763055	23.57641833
														MKDTDSEEEIR	3	4	1	N-Term(iTRAQ8plex); M1(Oxidation); K2(iTRAQ8plex)	0.656	0.989	1.078	3.53	31	1	1976.99013	4	495.0029907	-7.796341073	14.70337333
														DTDSEEEIR	4	4	1	N-Term(iTRAQ8plex)	0.752	1.005	1.207	2.88	38	0	1397.66509	2	699.3361816	-3.373224956	12.005675
														SLGQNPTEAELQDMINEVDADGNGTIDFPEFLTMMAR	1	4	1	N-Term(iTRAQ8plex)	-	-	-	-	38	0	4374.0593	3	1458.691284	1.677615359	70.10985667
														ELGTVMR	1	6	1	N-Term(iTRAQ8plex)	1.024	1.274	1.263	-	22	0	1109.62358	2	555.3154297	-4.937300437	21.31791333
4502551	CALU	calumenin isoform a precursor	NP_001210.1	26.67	6	6	11	315	37.1	4.64	0.831	0.430	0.921																
														GHDLNEDGLVSWEEYK	2	3	1	N-Term(iTRAQ8plex); K16(iTRAQ8plex)	0.807	0.685	1.057	3.91	35	0	2499.24973	3	833.7547607	-4.606311768	31.643695
														EEIVDKYDLFVGSQATDFGEALVR	1	5	1	N-Term(iTRAQ8plex); K6(iTRAQ8plex)	0.935	0.236	0.668	3.7	-	1	3309.76462	3	1103.926392	5.505000135	58.31876833
														TFDQLTPEESKER	1	5	1	N-Term(iTRAQ8plex); K11(iTRAQ8plex)	0.504	0.268	0.664	3.24	-	1	2188.1572	3	730.057251	-6.138325123	21.30716167
														WIYEDVER	5	3	1	N-Term(iTRAQ8plex)	0.774	0.374	0.847	3.08	30	0	1413.72563	2	707.3664551	-4.221302641	41.82825667
														EEFTAFLHPEEYDYMK	1	6	1	N-Term(iTRAQ8plex); K16(iTRAQ8plex)	0.855	0.656	1.01	2.84	-	0	2657.30381	3	886.4394531	-0.604061529	45.344225
														EQFVEFR	1	5	1	N-Term(iTRAQ8plex)	0.971	0.791	1.168	-	22	0	1258.66594	2	629.8366089	-5.895246959	20.84353667
4502565	CAPNS1	calpain small subunit 1 isoform 1	NP_001740.1	32.84	4	4	7	268	28.3	5.20	0.879	1.063	0.827																
														SGTICSSELPGAFEAAGFHLNEHLYNMIIR	1	4	1	N-Term(iTRAQ8plex); C5(Methylthio)	-	-	-	4.19	-	0	3627.78823	4	907.7025146	4.996546823	62.25150333
														THYSNIEANESEEVR	4	3	1	N-Term(iTRAQ8plex)	0.822	0.943	0.787	3.94	57	0	2081.99485	3	694.6697998	-4.474139768	20.40723167
														ILGGVISAISEAAAQYNPEPPPPR	1	2	1	N-Term(iTRAQ8plex)	-	-	-	-	33	0	2751.50571	3	917.8400879	2.663676782	69.93821333
														YSDESGNMDFDNFISCLVR	1	4	1	N-Term(iTRAQ8plex); C16(Methylthio)	1.537	2.68	1.368	-	25	0	2562.1276	3	854.7140503	0.888062292	65.45573333
4502599	CBR1	carbonyl reductase [NADPH] 1 isoform 1	NP_001748.1	19.86	4	4	12	277	30.4	8.32	0.268	1.190	2.036																
														ILLNACCPGWVR	5	1	1	N-Term(iTRAQ8plex); C6(Methylthio); C7(Methylthio)	0.805	2.178	5.091	3.97	53	0	1740.87212	2	870.9396973	-0.152445862	58.83044333
														DVCTELLPLIKPQGR	2	1	1	N-Term(iTRAQ8plex); C3(Methylthio); K11(iTRAQ8plex)	0.229	1.735	3.808	3.92	-	0	2336.31791	3	779.4441528	-4.784046675	51.503185
														FHQLDIDDLQSIR	4	4	1	N-Term(iTRAQ8plex)	0.156	0.188	0.626	3.42	30	0	1904.01395	3	635.3428345	-2.111923619	36.81553167
														SETITEEELVGLMNK	1	1	1	N-Term(iTRAQ8plex); M13(Oxidation); K15(iTRAQ8plex)	0.314	0.816	1.089	2.75	-	0	2317.2407	3	773.085083	-0.461983325	34.52758333
4502643	CCT6A	T-complex protein 1 subunit zeta isoform a	NP_001753.1	41.43	17	17	61	531	58.0	6.68	1.215	2.049	1.387																
														AQLGVQAFADALLIIPK	9	2	1	N-Term(iTRAQ8plex); K17(iTRAQ8plex)	2.025	1.515	1.445	5.95	43	0	2376.45517	3	792.8232422	3.207303292	64.57445667
														VHAELADVLTEAVVDSILAIK	8	2	1	N-Term(iTRAQ8plex); K21(iTRAQ8plex)	-	-	-	4.97	55	0	2814.63297	3	938.8825073	-3.834452827	71.95422167
														VLAQNSGFDLQETLVK	2	2	1	N-Term(iTRAQ8plex); K16(iTRAQ8plex)	1.444	2.237	1.378	4.92	54	0	2370.34348	3	790.7860107	-2.304443843	39.33282333
														NAIDDGCVVPGAGAVEVAMAEALIK	2	2	1	N-Term(iTRAQ8plex); C7(Methylthio); K25(iTRAQ8plex)	0.663	0.855	1.174	4.91	47	0	3067.63144	3	1023.215332	7.393503481	63.36095667
														AQAALAVNISAAR	2	2	1	N-Term(iTRAQ8plex)	1.395	3.971	2.462	4.59	48	0	1559.91557	2	780.4614258	-1.004129663	28.82039833
														QADLYISEGLHPR	4	4	1	N-Term(iTRAQ8plex)	1.38	2.049	1.557	4.55	62	0	1802.96042	3	601.6583252	-5.468208808	26.189295
														DGNVLLHEMQIQHPTASLIAK	1	1	1	N-Term(iTRAQ8plex); K21(iTRAQ8plex)	-	-	-	4.5	-	0	2923.61684	4	731.659668	-3.970870886	37.69019833
														GIDPFSLDALSK	5	2	1	N-Term(iTRAQ8plex); K12(iTRAQ8plex)	1.616	2.326	1.632	4.08	42	0	1871.06352	2	936.0314331	-9.562361034	33.92905
														ALQFLEEVK	6	2	1	N-Term(iTRAQ8plex); K9(iTRAQ8plex)	2.109	4.784	2.301	4.05	48	0	1684.99431	2	843.0007935	-8.96205159	71.05674
														HKSETDTSLIR	1	2	1	N-Term(iTRAQ8plex); K2(iTRAQ8plex)	1.068	2.44	1.321	3.92	-	1	1895.07387	4	474.5239258	-3.587224274	16.51031333
														EMDRETLIDVAR	8	2	1	N-Term(iTRAQ8plex)	0.686	1.775	1.303	3.75	41	1	1751.92143	2	876.4643555	-2.835162812	24.980985
														GLVLDHGAR	4	2	1	N-Term(iTRAQ8plex)	0.889	1.444	1.004	3.64	47	0	1241.71744	3	414.5773315	-7.537883243	14.43957167
														KQDEPIDLFMIEIMEMK	2	2	1	N-Term(iTRAQ8plex); K1(iTRAQ8plex); M16(Oxidation); K17(iTRAQ8plex)	-	-	-	3.11	31	1	3038.64052	4	760.4155884	0.821859514	62.46330333
														MLVSGAGDIK	2	5	1	N-Term(iTRAQ8plex); K10(iTRAQ8plex)	0.965	1.51	0.431	2.84	39	0	1598.94065	3	533.6517334	0.635198883	34.27887833
														AGMSSLK	1	8	1	N-Term(iTRAQ8plex); K7(iTRAQ8plex)	1.325	1.629	1.358	-	34	0	1301.75956	3	434.5913696	-8.600407988	11.732045
														GLQDVLR	2	5	1	N-Term(iTRAQ8plex)	0.998	2.931	2.098	-	31	0	1104.66094	2	552.8341064	-6.287023599	19.203705
														EMDRETLIDVAR	1	2	1	N-Term(iTRAQ8plex); M2(Oxidation)	1.123	1.518	1.381	-	28	1	1767.91069	2	876.4643555	-2.835162812	24.980985
														SETDTSLIR	1	2	1	N-Term(iTRAQ8plex)	0.766	1.87	1.318	-	22	0	1325.72124	2	663.3642578	-0.145049347	17.80205667
4502735	CDK4	cyclin-dependent kinase 4	NP_000066.1	4.29	1	1	2	303	33.7	7.01	0.668	1.043	1.667																
														VTLVFEHVDQDLR	2	1	1	N-Term(iTRAQ8plex)	0.668	1.043	1.667	4.15	32	0	1875.02561	3	625.6800537	-1.175139784	46.10335833
4502877	CLDN4	claudin-4	NP_001296.1	7.66	1	1	1	209	22.1	8.05	1.435	1.203	1.106																
														VYDSLLALPQDLQAAR	1	4	1	N-Term(iTRAQ8plex)	1.435	1.203	1.106	-	29	0	2077.15921	3	693.0579224	-0.167276612	55.32005167
4502985	COX6B1	cytochrome c oxidase subunit 6B1	NP_001854.1	47.67	3	3	31	86	10.2	7.05	0.956	0.936	0.932																
														VYQSLCPTSWVTDWDEQR	7	1	1	N-Term(iTRAQ8plex); C6(Methylthio)	0.836	0.932	1.282	5.96	69	0	2563.20041	3	855.0716553	4.080941257	58.983905
														NCWQNYLDFHR	16	1	1	N-Term(iTRAQ8plex); C2(Methylthio)	0.955	1.034	1.013	4.6	45	0	1845.84079	3	615.9580688	7.03790938	39.78703333
														GGDISVCEWYQR	6	1	1	N-Term(iTRAQ8plex); C7(Methylthio)	1.405	0.945	0.865	4.44	35	0	1762.81523	2	881.9087524	-5.23824802	38.70276
														VYQSLCPTSWVTDWDEQR	1	1	1	N-Term(iTRAQ8plex); Y2(iTRAQ8plex); C6(Methylthio)	1.367	1.088	0.926	-	41	0	2867.39042	3	855.0716553	4.080941257	58.983905
														NCWQNYLDFHR	1	1	1	N-Term(iTRAQ8plex); C2(Methylthio); Y6(iTRAQ8plex)	0.528	0.928	0.326	-	31	0	2150.06533	3	615.9580688	7.03790938	39.78703333

4503117	CSTB	cystatin-B	NP_000091.1	45.92	3	3	26	98	11.1	7.56	1.221	1.481	1.578																
														VHVGDEDFVHLR	22	1	1	N-Term(iTRAQ8plex)	1.265	1.632	1.591	5.17	51	0	1726.91189	3	576.3137817	5.171420633	25.28613833
														SQVVAGTNYFIK	3	1	1	N-Term(iTRAQ8plex); K12(iTRAQ8plex)	1.174	1.04	1.151	4.12	34	0	1935.10324	3	645.7059326	-6.58708959	40.70484
														VFQSLPHENKPLTLSNYQTNK	1	1	1	N-Term(iTRAQ8plex); K10(iTRAQ8plex); K21(iTRAQ8plex)	-	-	-	2.87	-	0	3370.86439	5	674.9786987	-7.198200482	32.45451167
4503143	CTSD	cathepsin D preproprotein	NP_001900.1	31.07	9	9	44	412	44.5	6.54	0.715	0.407	0.576																
														AIGAVPLIQGEYMIPCEK	5	1	1	N-Term(iTRAQ8plex); C16(Methylthio); K18(iTRAQ8plex)	0.59	0.395	0.566	6.05	104	0	2586.41606	2	1293.71167	7.965046322	48.41291833
														DPDAQPGGELMLGGTDSK	4	1	1	N-Term(iTRAQ8plex); K18(iTRAQ8plex)	0.667	0.386	0.603	5.89	58	0	2396.23296	2	1198.620117	4.384571611	28.23884667
														LVDQNIFSFYLSR	13	1	1	N-Term(iTRAQ8plex)	0.74	0.419	0.668	5.23	68	0	1906.04404	3	636.0195313	3.362566585	53.59532667
														ISVNNVLPVFDNLMQQK	3	1	1	N-Term(iTRAQ8plex); M14(Oxidation); K17(iTRAQ8plex)	0.737	1.163	1.106	4.98	32	0	2583.44052	3	856.4868164	-0.672002963	47.99560833
														ISVNNVLPVFDNLMQQK	4	1	1	N-Term(iTRAQ8plex); K17(iTRAQ8plex)	0.795	0.378	0.654	4.83	50	0	2567.4459	3	856.4868164	-0.672002963	47.99560833
														VSTLPAITLK	5	1	1	N-Term(iTRAQ8plex); K10(iTRAQ8plex)	0.711	0.446	0.525	4.22	42	0	1651.06162	2	826.0297241	-5.61440636	27.09187833
														FDGILGMAYPR	4	1	1	N-Term(iTRAQ8plex)	0.558	0.41	0.592	4.13	41	0	1543.82488	2	772.4160767	0.256117335	47.48528667
														QPGITFIAAK	2	1	1	N-Term(iTRAQ8plex); K10(iTRAQ8plex)	0.756	0.41	0.566	4.03	36	0	1654.01291	3	552.0091553	-1.160045168	36.59596833
														AYWQVHLDQVEVASGLTLCK	1	1	1	N-Term(iTRAQ8plex); C19(Methylthio); K20(iTRAQ8plex)	0.549	-	0.481	3.87	-	0	2914.53164	4	729.3883667	-3.415774596	54.31340667
														LLDIACWIHHK	2	1	1	N-Term(iTRAQ8plex); C6(Methylthio); K11(iTRAQ8plex)	0.445	0.122	0.379	3.72	33	0	2003.12551	4	501.5368347	3.78243258	51.52332667
														AIGAVPLIQGEYMIPCEK	1	1	1	N-Term(iTRAQ8plex); M13(Oxidation); C16(Methylthio); K18(iTRAQ8plex)	-	-	-	3.01	-	0	2602.39638	2	1293.71167	7.965046322	48.41291833
4503183	CYB5A	cytochrome b5 isoform 2	NP_001905.1	9.18	1	1	1	98	11.3	5.14	0.227	0.636	1.013																
														STWLILHHK	1	4	1	N-Term(iTRAQ8plex); K9(iTRAQ8plex)	0.227	0.636	1.013	-	26	0	1743.05251	4	436.5185852	-0.046102849	24.34000667
4503253	DAD1	dolichyl-diphosphooligosaccharide--protein glycosyltransferase subunit DAD1	NP_001335.1	19.47	2	2	6	113	12.5	7.08	0.926	0.483	0.573																
														FLEEYLSSTPQR	2	1	1	N-Term(iTRAQ8plex)	0.767	0.416	0.477	4.43	36	0	1773.93328	2	887.4702759	0.441864316	41.91642667
														ADFQGISPER	4	1	1	N-Term(iTRAQ8plex)	0.943	0.494	0.584	2.86	47	0	1423.74101	2	712.3741455	-5.139210222	16.74465
4503363	DPM1	dolichol-phosphate mannosyltransferase subunit 1 isoform 3	NP_003850.1	8.46	1	1	1	260	29.6	9.57	-	-	-																
														GANFLTQILLRPGASDLTGSFR	1	3	1	N-Term(iTRAQ8plex)	-	-	-	3.8	-	0	2638.46767	4	660.3723755	2.175798167	56.853685
4503471	EEF1A1	elongation factor 1-alpha 1	NP_001393.1	48.92	10	18	457	462	50.1	9.01	1.141	2.890	1.723																
														VETGVLKPGMVVTFAPVNVTTEVK	18	1	1	N-Term(iTRAQ8plex); K7(iTRAQ8plex); M10(Oxidation); K24(iTRAQ8plex)	4.012	15.521	6.131	6.23	35	0	3443.98634	2	1714.498657	-2.976515907	45.70123833
														DGNASGTTLLEALDCILPPTRPTDKPLR	19	1	1	N-Term(iTRAQ8plex); C15(Methylthio); K25(iTRAQ8plex)	-	-	-	5.58	36	0	3618.93903	4	905.4907837	-0.880758793	56.188755
														SGDAAIVDMVPGKPMCVESFSDYPPLGR	56	1	1	N-Term(iTRAQ8plex); M9(Oxidation); K13(iTRAQ8plex); C16(Methylthio)	1.529	7.703	4.511	5.56	39	0	3608.76821	4	906.9448242	-2.575428862	40.21384
														KDGNASGTTLLEALDCILPPTRPTDKPLR	2	1	1	N-Term(iTRAQ8plex); K1(iTRAQ8plex); C16(Methylthio); K26(iTRAQ8plex)	-	-	-	5.43	-	1	4051.22785	5	811.0513916	-4.189051519	53.79789333
														VETGVLKPGMVVTFAPVNVTTEVK	27	1	1	N-Term(iTRAQ8plex); K7(iTRAQ8plex); K24(iTRAQ8plex)	1.77	12.142	4.567	5.42	57	0	3428.01162	2	1714.498657	-2.976515907	45.70123833
														SGDAAIVDMVPGKPMCVESFSDYPPLGR	22	1	1	N-Term(iTRAQ8plex); M9(Oxidation); K13(iTRAQ8plex); M15(Oxidation); C16(Methylthio)	-	-	-	5.26	44	0	3624.75429	4	906.9448242	-2.575428862	40.21384
														YYVTIIDAPGHR	73	1	1	N-Term(iTRAQ8plex)	0.994	2.064	1.454	5.25	61	0	1708.92899	3	570.3145142	-2.008316058	35.49489333
														YEEIVKEVSTYIK	2	1	1	N-Term(iTRAQ8plex); K6(iTRAQ8plex); K13(iTRAQ8plex)	-	-	-	5.12	26	1	2513.45058	4	629.118103	-4.937065687	57.13264667
														SGDAAIVDMVPGKPMCVESFSDYPPLGR	70	1	1	N-Term(iTRAQ8plex); K13(iTRAQ8plex); C16(Methylthio)	0.848	10.462	3.85	4.88	40	0	3592.75747	4	906.9448242	-2.575428862	40.21384
														IGYNPDTVAFVPISGWNGDNMLEPSANMPWFK	9	1	1	N-Term(iTRAQ8plex); K32(iTRAQ8plex)	-	-	-	4.41	52	0	4176.0812	3	1392.703979	3.667042492	61.593155
														IGYNPDTVAFVPISGWNGDNMLEPSANMPWFK	5	1	1	N-Term(iTRAQ8plex); M21(Oxidation); K32(iTRAQ8plex)	-	-	-	3.52	48	0	4192.10776	3	1392.703979	3.667042492	61.593155
														EVSTYIK	5	1	1	N-Term(iTRAQ8plex); K7(iTRAQ8plex)	1.253	4.626	2.455	3.1	28	0	1447.8477	2	724.4277954	-9.211669176	16.26334
														KLEDGPK	1	1	1	N-Term(iTRAQ8plex); K1(iTRAQ8plex); K7(iTRAQ8plex)	1.423	3.856	1.956	2.93	-	1	1699.05276	4	425.5186462	0.608445462	17.91177167
														YYVTIIDAPGHR	8	1	1	N-Term(iTRAQ8plex); Y2(iTRAQ8plex)	1.043	3.051	1.864	-	43	0	2013.12517	3	570.3145142	-2.008316058	35.49489333
														KIGYNPDTVAFVPISGWNGDNMLEPSANMPWFK	1	1	1	N-Term(iTRAQ8plex); K1(iTRAQ8plex); K33(iTRAQ8plex)	-	-	-	-	23	1	4608.39028	4	1152.853027	1.709532944	60.011175
														IGYNPDTVAFVPISGWNGDNMLEPSANMPWFK	1	1	1	N-Term(iTRAQ8plex); Y3(iTRAQ8plex); K32(iTRAQ8plex)	-	-	-	-	22	0	4480.27326	3	1392.703979	3.667042492	61.593155
														EHALLAYTLGVK	99	2	2	N-Term(iTRAQ8plex); K12(iTRAQ8plex)	-	-	-	5.93	66	0	1923.14055	2	962.0739136	-6.151797672	33.90791833
														THINIVVIGHVDSGK	6	2	2	N-Term(iTRAQ8plex); K15(iTRAQ8plex)	-	-	-	5.56	35	0	2197.2808	4	550.0750732	-5.865533871	23.91521667
														QTVAVGVIK	4	2	2	N-Term(iTRAQ8plex); K9(iTRAQ8plex)	-	-	-	4.23	53	0	1522.97319	2	761.9902344	-2.967333912	17.33712167
														QLIVGVNK	6	2	2	N-Term(iTRAQ8plex); K8(iTRAQ8plex)	-	-	-	3.81	43	0	1478.93698	3	493.6505127	-9.808710047	31.30948833
														STTTGHLIYK	5	2	2	N-Term(iTRAQ8plex); K10(iTRAQ8plex)	-	-	-	3.75	43	0	1728.99748	2	865.0023804	-7.494040871	22.702905
														EAAEMGK	2	2	2	N-Term(iTRAQ8plex); K7(iTRAQ8plex)	-	-	-	3.73	-	0	1343.7331	3	448.58255	-8.814550704	12.39407
														IGGIGTVPVGR	15	2	2	N-Term(iTRAQ8plex)	-	-	-	3.2	46	0	1329.80962	2	665.4084473	-4.521992223	27.41090333
														LPLQDVYK	1	2	2	N-Term(iTRAQ8plex); K8(iTRAQ8plex)	-	-	-	3.08	-	0	1583.94585	2	792.4765625	-10.01481207	29.80285333
4503475	EEF1A2	elongation factor 1-alpha 2	NP_001949.1	37.15	4	12	172	463	50.4	9.03	1.006	1.822	2.431																
														VETGILRPGMVVTFAPVNITTEVK	10	1	1	N-Term(iTRAQ8plex); K24(iTRAQ8plex)	-	2.055	3.099	5.59	21	0	3179.80874	4	795.7125854	-1.201128649	43.130915
														EGNASGVSLLEALDTILPPTRPTDKPLR	3	1	1	N-Term(iTRAQ8plex); K25(iTRAQ8plex)	-	-	-	5	38	0	3569.00478	4	893.0066528	-3.143692026	57.03036
														YYITIIDAPGHR	9	1	1	N-Term(iTRAQ8plex)	1.006	1.615	2.431	4.45	51	0	1722.93729	3	574.9839478	-6.257504753	32.30403333
														SGDAAIVEMVPGKPMCVESFSQYPPLGR	5	1	1	N-Term(iTRAQ8plex); K13(iTRAQ8plex); M15(Oxidation); C16(Methylthio)	-	-	-	4.34	-	0	3635.81728	4	909.7097778	-0.518687197	41.06900167
														VETGILRPGMVVTFAPVNITTEVK	1	1	1	N-Term(iTRAQ8plex); M10(Oxidation); K24(iTRAQ8plex)	-	-	-	3.87	-	0	3195.81972	4	795.7125854	-1.201128649	43.130915
														SGDAAIVEMVPGKPMCVESFSQYPPLGR	4	1	1	N-Term(iTRAQ8plex); K13(iTRAQ8plex); C16(Methylthio)	-	-	-	3.62	-	0	3619.8163	4	909.7097778	-0.518687197	41.06900167
														YYITIIDAPGHR	2	1	1	N-Term(iTRAQ8plex); Y2(iTRAQ8plex)	-	-	1.648	-	29	0	2027.13625	3	574.9839478	-6.257504753	32.30403333
														EHALLAYTLGVK	99	2	2	N-Term(iTRAQ8plex); K12(iTRAQ8plex)	-	-	-	5.93	66	0	1923.14055	2	962.0739136	-6.151797672	33.90791833
														THINIVVIGHVDSGK	6	2	2	N-Term(iTRAQ8plex); K15(iTRAQ8plex)	-	-	-	5.56	35	0	2197.2808	4	550.0750732	-5.865533871	23.91521667
														QTVAVGVIK	4	2	2	N-Term(iTRAQ8plex); K9(iTRAQ8plex)	-	-	-	4.23	53	0	1522.97319	2	761.9902344	-2.967333912	17.33712167
														QLIVGVNK	6	2	2	N-Term(iTRAQ8plex); K8(iTRAQ8plex)	-	-	-	3.81	43	0	1478.93698	3	493.6505127	-9.808710047	31.30948833
														STTTGHLIYK	5	2	2	N-Term(iTRAQ8plex); K10(iTRAQ8plex)	-	-	-	3.75	43	0	1728.99748	2	865.0023804	-7.494040871	22.702905
														EAAEMGK	2	2	2	N-Term(iTRAQ8plex); K7(iTRAQ8plex)	-	-	-	3.73	-	0	1343.7331	3	448.58255	-8.814550704	12.39407
														IGGIGTVPVGR	15	2	2	N-Term(iTRAQ8plex)	-	-	-	3.2	46	0	1329.80962	2	665.4084473	-4.521992223	27.41090333
														LPLQDVYK	1	2	2	N-Term(iTRAQ8plex); K8(iTRAQ8plex)	-	-	-	3.08	-	0	1583.94585	2	792.4765625	-10.01481207	29.80285333
4503481	EEF1G	elongation factor 1-gamma	NP_001395.1	38.22	11	11	106	437	50.1	6.67	1.282	3.703	2.260																
														GSTPEAAAQVVQWVSFADSDIVPPASTWVFPTLGIMHHNK	2	1	1	N-Term(iTRAQ8plex); K40(iTRAQ8plex)	-	-	-	5.39	35	0	4899.53774	4	1225.639893	-0.259385184	67.7233
														GSTPEAAAQVVQWVSFADSDIVPPASTWVFPTLGIMHHNK	2	1	1	N-Term(iTRAQ8plex); M36(Oxidation); K40(iTRAQ8plex)	-	-	-	5.39	35	0	4915.53774	4	1225.639893	-0.259385184	67.7233
														WFLTCINQPQFR	8	1	1	N-Term(iTRAQ8plex); C5(Methylthio)	0.689	2.013	1.297	5.27	44	0	1902.97098	3	634.9951782	2.579712469	54.90914167
														GQELAFPLSPDWQVDYESYTWR	4	1	1	N-Term(iTRAQ8plex)	-	-	-	4.95	72	0	2991.43906	3	997.8178711	-2.557721751	59.33691667
														QAFPNTNRWFLTCINQPQFR	10	1	1	N-Term(iTRAQ8plex); C13(Methylthio)	1.52	5.045	2.64	4.83	26	1	2831.42397	4	708.6114502	2.242193792	53.34971333
														ILGLLDAYLK	34	1	1	N-Term(iTRAQ8plex); K10(iTRAQ8plex)	2.232	7.416	4.132	4.69	57	0	1727.08122	2	864.0442505	-6.668443426	85.34799833
														VLSAPPHFHFGQTNR	15	1	1	N-Term(iTRAQ8plex)	0.99	1.877	1.261	4.64	49	0	2012.06925	3	671.3621826	-2.398869648	25.908225
														EYFSWEGAFQHVGK	12	1	1	N-Term(iTRAQ8plex); K14(iTRAQ8plex)	1.183	4.086	2.476	4.61	41	0	2293.17588	3	765.0634766	-4.506790171	40.709385
														KAAAPAPEEEMDECEQALAAEPK	2	1	1	N-Term(iTRAQ8plex); K1(iTRAQ8plex); C14(Methylthio); K23(iTRAQ8plex)	-	-	-	4.46	32	1	3386.69594	4	847.4294434	-2.562598664	39.81004667
														ALIAAQYSGAQVR	6	1	1	N-Term(iTRAQ8plex)	1.188	2.705	1.979	4.15	47	0	1651.93974	2	826.4735107	-2.177098551	28.78820167
														STFVLDEFKR	5	1	1	N-Term(iTRAQ8plex); K9(iTRAQ8plex)	1.43	4.693	2.557	3.88	42	1	1850.05094	2	925.5300903	-5.587546126	30.067595
														STFVLDEFK	4	1	1	N-Term(iTRAQ8plex); K9(iTRAQ8plex)	1.488	3.812	2.434	3.85	43	0	1693.94927	2	847.4782715	-7.588746313	58.11391833
														ILGLLDAYLK	2	1	1	N-Term(iTRAQ8plex); Y8(iTRAQ8plex); K10(iTRAQ8plex)	-	-	-	-	26	0	2031.29665	2	864.0442505	-6.668443426	85.34799833
4503483	EEF2	elongation factor 2	NP_001952.1	48.95	31	32	249	858	95.3	6.83	1.040	2.974	2.699																
														STAISLFYELSENDLNFIK	39	1	1	N-Term(iTRAQ8plex); K19(iTRAQ8plex)	1.347	3.33	3.123	8.18	81	0	2812.5311	2	1406.766113	0.714177664	64.62649833
														CELLYEGPPDDEAAMGIK	12	1	1	N-Term(iTRAQ8plex); C1(Methylthio); K18(iTRAQ8plex)	0.913	1.621	1.625	7.58	76	0	2605.29717	2	1303.152222	6.246672964	45.7533
														WLPAGDALLQMITIHLPSPVTAQK	19	1	1	N-Term(iTRAQ8plex); K24(iTRAQ8plex)	-	-	-	6.96	47	0	3208.84292	4	802.9661865	1.612703982	69.29142667
														GHVFEESQVAGTPMFVVK	12	1	1	N-Term(iTRAQ8plex); K18(iTRAQ8plex)	0.627	2.07	2.115	6.28	59	0	2570.38164	2	1285.694458	-3.167585797	35.46586333
														IWCFGPDGTGPNILTDITK	2	1	1	N-Term(iTRAQ8plex); C3(Methylthio); K19(iTRAQ8plex)	0.5	3.003	3.856	6.24	64	0	2702.42868	3	901.4810791	5.329189545	59.500855
														ETVSEESNVLCLSK	8	1	1	N-Term(iTRAQ8plex); C11(Methylthio); K14(iTRAQ8plex)	1.21	2.875	3.064	5.69	35	0	2192.14011	3	731.3848877	0.085549248	32.67864667
														YVEPIEDVPCGNIVGLVGVDQFLVK	5	1	1	N-Term(iTRAQ8plex); C10(Methylthio); K25(iTRAQ8plex)	-	-	-	5.16	58	0	3356.80039	3	1119.60498	-2.727044415	67.63885333
														RWLPAGDALLQMITIHLPSPVTAQK	3	1	1	N-Term(iTRAQ8plex); K25(iTRAQ8plex)	-	-	-	5.1	26	1	3364.94306	5	673.7944336	1.248210943	64.30801
														VFSGLVSTGLK	2	1	1	N-Term(iTRAQ8plex); K11(iTRAQ8plex)	0.918	3.25	2.885	4.92	59	0	1716.03679	2	858.5220337	-8.636491518	32.40650833
														CLYASVLTAQPR	18	1	1	N-Term(iTRAQ8plex); C1(Methylthio)	1.096	3.018	2.51	4.71	63	0	1671.88236	2	836.4446411	-2.635768208	40.92513333
														WLPAGDALLQMITIHLPSPVTAQK	2	1	1	N-Term(iTRAQ8plex); M11(Oxidation); K24(iTRAQ8plex)	-	-	-	4.65	30	0	3224.84145	4	802.9661865	1.612703982	69.29142667
														EGALCEENMR	6	1	1	N-Term(iTRAQ8plex); C5(Methylthio)	0.687	2.076	2.029	4.64	37	0	1501.67815	2	751.3375854	-4.813328959	19.04133667
														EGIPALDNFLDKL	25	1	1	N-Term(iTRAQ8plex); K12(iTRAQ8plex)	1.211	4.059	2.741	4.63	57	1	2053.16489	3	685.0598145	-6.882637967	79.89853333
														NMSVIAHVDHGK	5	1	1	N-Term(iTRAQ8plex); K12(iTRAQ8plex)	1.258	1.93	2.274	4.6	-	0	1916.04763	4	479.7673645	-8.159072853	24.52094667
														DGAGFLINLIDSPGHVDFSSEVTAALR	2	1	1	N-Term(iTRAQ8plex)	-	-	-	4.59	30	0	3105.624	3	1035.879517	2.590543703	64.423495
														AYLPVNESFGFTADLR	19	1	1	N-Term(iTRAQ8plex)	0.997	2.694	2.695	4.57	84	0	2104.09844	2	1052.553467	-0.976344558	46.59738833
														GHVFEESQVAGTPMFVVK	3	1	1	N-Term(iTRAQ8plex); M14(Oxidation); K18(iTRAQ8plex)	1.074	1.437	1.816	4.55	33	0	2586.36977	2	1285.694458	-3.167585797	35.46586333
														ARPFPDGLAEDIDKGEVSAR	3	1	1	N-Term(iTRAQ8plex); K14(iTRAQ8plex)	0.694	3.737	3.817	4.47	-	1	2751.46619	5	551.0990601	-8.169287008	30.20978
														TGTITTFEHAHNMR	6	1	1	N-Term(iTRAQ8plex)	1.378	3.475	3.426	4.31	73	0	1919.98186	2	960.4945679	6.191632557	22.05672
														STLTDSLVCK	7	1	1	N-Term(iTRAQ8plex); C9(Methylthio); K10(iTRAQ8plex)	0.99	2.943	2.921	4.31	54	0	1720.92705	2	860.9671631	-9.48485767	42.91208
														DLEEDHACIPIKK	2	1	1	N-Term(iTRAQ8plex); C8(Methylthio); K12(iTRAQ8plex); K13(iTRAQ8plex)	1.573	4.823	4.643	4.02	-	1	2469.33817	3	823.7842407	-9.198165856	24.269615
														GVQYLNEIKDSVVAGFQWATK	2	1	1	N-Term(iTRAQ8plex); K9(iTRAQ8plex); K21(iTRAQ8plex)	-	-	-	3.95	-	1	3265.8312	4	817.2132568	-1.118807443	66.453545
														CELLYEGPPDDEAAMGIK	5	1	1	N-Term(iTRAQ8plex); C1(Methylthio); M15(Oxidation); K18(iTRAQ8plex)	0.57	2.201	1.869	3.83	60	0	2621.28691	2	1303.152222	6.246672964	45.7533
														YEWDVAEAR	10	1	1	N-Term(iTRAQ8plex)	1.058	1.921	1.873	3.71	33	0	1442.71868	2	721.8629761	-2.145790177	28.800665
														NMSVIAHVDHGK	1	1	1	N-Term(iTRAQ8plex); M2(Oxidation); K12(iTRAQ8plex)	0.765	1.248	1.343	3.58	-	0	1932.04043	4	479.7673645	-8.159072853	24.52094667
														TGTITTFEHAHNMR	1	1	1	N-Term(iTRAQ8plex); M13(Oxidation)	1.224	1.464	1.29	3.57	-	0	1935.95981	2	960.4945679	6.191632557	22.05672
														IMGPNYTPGK	2	1	1	N-Term(iTRAQ8plex); K10(iTRAQ8plex)	1.039	1.809	1.715	3.37	29	0	1685.94475	2	843.4760132	-3.417403099	19.68010667
														EDLYLKPIQR	2	1	1	N-Term(iTRAQ8plex); K6(iTRAQ8plex)	1.246	3.509	3.074	3.28	26	0	1883.10759	2	942.0574341	-7.163560913	22.38916667
														GPLMMYISK	5	1	1	N-Term(iTRAQ8plex); K9(iTRAQ8plex)	1.091	5.656	4.994	3.16	38	0	1647.92595	2	824.4666138	-9.897520534	55.43708833
														YLAEKYEWDVAEAR	2	1	1	N-Term(iTRAQ8plex); K5(iTRAQ8plex)	1.216	2.833	2.007	3.07	-	1	2351.23954	3	784.4180298	-4.114933584	34.24463667
														FSVSPVVR	2	1	1	N-Term(iTRAQ8plex)	0.993	3.49	2.498	2.96	41	0	1194.7074	3	398.9073181	-6.219266986	26.26046333
														GEGQLGPAER	6	1	1	N-Term(iTRAQ8plex)	0.95	3.429	3.364	2.89	38	0	1317.70183	2	659.3543701	-3.800318785	12.12759
														AGIIASAR	2	1	1	N-Term(iTRAQ8plex)	1.09	4.764	4.48	2.72	28	0	1062.65386	2	531.8305664	-3.260805463	23.23128833
														AYLPVNESFGFTADLR	2	1	1	N-Term(iTRAQ8plex); Y2(iTRAQ8plex)	0.506	2.036	2.636	-	53	0	2408.2952	2	1052.553467	-0.976344558	46.59738833
														VNFTVDQIR	3	1	1	N-Term(iTRAQ8plex)	1.098	2.661	2.704	-	40	0	1395.78211	3	465.9322205	-5.50456479	30.159515
														STAISLFYELSENDLNFIK	1	1	1	N-Term(iTRAQ8plex); Y8(iTRAQ8plex); K19(iTRAQ8plex)	-	-	-	-	25	0	3116.71461	2	1406.766113	0.714177664	64.62649833

														SDPVVSYR	1	1	1	N-Term(iTRAQ8plex)	1.152	3.549	3.083	-	23	0	1226.66094	2	613.8341064	-5.971519234	16.56696333
														TILMMGR	1	1	1	N-Term(iTRAQ8plex)	0.879	4.145	3.97	-	20	0	1125.63542	2	563.3213501	-6.385481957	28.26393833
														GGGQIIPTAR	1	4	2	N-Term(iTRAQ8plex)	-	-	-	2.95	-	0	1273.74748	2	637.3773804	-4.347155054	17.59469667
4503507	EIF2S3	eukaryotic translation initiation factor 2 subunit 3	NP_001406.1	15.68	5	5	18	472	51.1	8.40	1.501	2.813	2.120																
														QATINIGTIGHVAHGK	4	1	1	N-Term(iTRAQ8plex); K16(iTRAQ8plex)	1.832	2.249	1.294	5.16	32	0	2225.28293	3	742.4324951	-6.547121819	22.789835
														SCGSSTPDEFPTDIPGTK	2	1	1	N-Term(iTRAQ8plex); C2(Methylthio); K18(iTRAQ8plex)	1.617	3.911	3	4.87	63	0	2493.21538	2	1247.111328	2.232992001	31.72884667
														SFDVNKPGCEVDDLK	1	1	1	N-Term(iTRAQ8plex); K6(iTRAQ8plex); C9(Methylthio); K15(iTRAQ8plex)	1.537	3.927	2.962	4.29	-	0	2624.35672	3	875.4570923	-9.918123667	30.062275
														LTPLSHEVISR	9	1	1	N-Term(iTRAQ8plex)	1.298	2.736	1.942	3.79	46	0	1555.90647	3	519.3044434	-7.837574048	21.794335
														VGQEIEVRPGIVSK	2	1	1	N-Term(iTRAQ8plex); K14(iTRAQ8plex)	1.762	3.36	2.499	3.55	44	0	2119.26006	2	1060.133667	-4.489272372	24.80785833
														ILVIR	1	2	1	N-Term(iTRAQ8plex)	2.022	3.866	2.769	-	24	0	917.63628	2	459.3217773	-9.45171056	27.010125
4503509	EIF3A	eukaryotic translation initiation factor 3 subunit A	NP_003741.1	9.19	10	10	18	1382	166.5	6.79	0.952	2.394	2.096																
														DIDIEDLEELDPDFIMAK	2	1	1	N-Term(iTRAQ8plex); K18(iTRAQ8plex)	1.145	4.331	3.727	6.12	57	0	2729.42447	3	910.4796753	7.045928216	55.350255
														EDAPIGPHLQSMPSEQIR	2	1	1	N-Term(iTRAQ8plex)	0.992	2.229	2.107	4.93	36	0	2309.17527	3	770.3966064	-4.739880174	26.104995
														LTSLVPFVDAFQLER	2	1	1	N-Term(iTRAQ8plex)	0.952	3.637	3.314	4.48	42	0	2039.15434	2	1020.080811	3.140112355	66.141155
														LVQLDSAISMELWQEAFK	2	1	1	N-Term(iTRAQ8plex); K18(iTRAQ8plex)	-	-	-	4.02	37	0	2716.50125	3	906.171936	6.321939089	62.16716667
														LLDMDGIIVEK	2	1	1	N-Term(iTRAQ8plex); K11(iTRAQ8plex)	1.027	2.394	2.096	3.73	28	0	1854.06877	2	927.5380249	-9.66960642	68.96616833
														VLLATLSIPITPER	2	1	1	N-Term(iTRAQ8plex)	0.858	1.936	1.589	3.51	70	0	1827.11833	2	914.0628052	-4.054235733	54.71265833
														VSTVFWK	1	1	1	N-Term(iTRAQ8plex); K7(iTRAQ8plex)	0.504	0.763	0.991	2.9	-	0	1474.89191	3	492.3021545	2.772456082	29.80890333
														RLEEIPLIK	1	1	1	N-Term(iTRAQ8plex); K9(iTRAQ8plex)	0.82	1.651	1.802	2.87	-	1	1719.09079	3	573.7017822	-4.716371202	36.715275
														LLDMDGIIVEK	1	1	1	N-Term(iTRAQ8plex); M4(Oxidation); K11(iTRAQ8plex)	8.819	3.901	1.016	2.75	-	0	1870.0772	2	927.5380249	-9.66960642	68.96616833
														ELEIEER	2	1	1	N-Term(iTRAQ8plex)	1.055	2.784	2.538	-	25	0	1221.65911	2	611.3331909	-3.074576243	26.50035167
														GLDDDRGPWR	1	1	1	N-Term(iTRAQ8plex)	0.903	1.488	1.839	-	24	1	1490.75603	2	745.8816528	-6.280206657	16.33768667
4503513	EIF3I	eukaryotic translation initiation factor 3 subunit I	NP_003748.1	9.85	3	3	14	325	36.5	5.64	0.963	2.388	2.314																
														FFHLAFEEEFGR	8	2	1	N-Term(iTRAQ8plex)	0.952	1.836	1.574	3.64	37	0	1832.92765	3	611.6459961	-2.14753583	48.14403667
														LWDCETGK	4	1	1	N-Term(iTRAQ8plex); C4(Methylthio); K8(iTRAQ8plex)	0.902	2.565	2.763	3.6	26	0	1605.82036	2	803.4138184	-1.352705012	29.66715
														SYSSGGEDGYVR	2	2	1	N-Term(iTRAQ8plex)	1.228	2.588	2.314	2.91	40	0	1580.73918	2	790.87323	-6.483017041	13.404075
4503515	EIF3H	eukaryotic translation initiation factor 3 subunit H	NP_003747.1	30.40	6	6	17	352	39.9	6.54	1.292	4.278	3.379																
														LEITNCFPFPQHTEDDADFDEVQYQMEMMR	2	1	1	N-Term(iTRAQ8plex); C6(Methylthio)	-	4.278	4.002	6.43	40	0	4029.74485	3	1343.9198	0.787609187	58.24348833
														ANITFEYMFEEVPIVIK	4	1	1	N-Term(iTRAQ8plex); K17(iTRAQ8plex)	1.95	4.265	2.904	5.08	63	0	2651.47368	2	1326.240479	4.578250706	59.67059
														TAQGSLSLK	4	1	1	N-Term(iTRAQ8plex); K9(iTRAQ8plex)	1.047	4.377	3.726	4.04	39	0	1512.90727	2	756.9572754	-8.795739817	26.41921167
														NSHLINVLMWELEK	4	1	1	N-Term(iTRAQ8plex); K14(iTRAQ8plex)	-	-	-	4.03	29	0	2334.29165	4	584.3319702	-1.713708716	51.93252
														NLQLLMDRVDEMSQDIVK	2	1	1	N-Term(iTRAQ8plex); K18(iTRAQ8plex)	-	-	-	3.89	45	1	2755.475	4	689.6242065	-7.0205711	60.80387167
														EGTGSTATSSSSTAGAAGK	1	1	1	N-Term(iTRAQ8plex); K19(iTRAQ8plex)	-	-	-	-	33	0	2236.14109	3	746.0518799	-4.594925439	13.70400833
4503519	EIF3F	eukaryotic translation initiation factor 3 subunit F	NP_003745.1	15.41	3	3	20	357	37.5	5.45	0.891	1.344	1.201																
														LHPVILASIVDSYER	16	1	1	N-Term(iTRAQ8plex)	0.916	1.305	1.125	4.96	77	0	2016.14348	2	1008.575378	0.153221207	44.369635
														AYVSTLMGVPGR	2	1	1	N-Term(iTRAQ8plex)	0.792	1.385	1.423	3.41	30	0	1554.85295	2	777.9301147	-5.555784421	35.210405
														HSVEVTNCFSVPHNESEDEVAVDMEFAK	2	1	1	N-Term(iTRAQ8plex); C8(Methylthio); K28(iTRAQ8plex)	-	-	-	3.34	23	0	3803.78432	4	951.7015381	0.809762986	41.95532667
4503521	EIF3E	eukaryotic translation initiation factor 3 subunit E	NP_001559.1	12.81	5	5	15	445	52.2	6.04	0.862	1.855	1.878																
														NALSSLWGK	3	2	1	N-Term(iTRAQ8plex); K9(iTRAQ8plex)	1.222	1.482	1.572	4.37	34	0	1583.92363	2	792.4654541	-8.169407045	30.41518
														LASEILMQNWDAAMEDLTR	2	2	1	N-Term(iTRAQ8plex)	-	-	-	4.21	72	0	2511.25614	3	837.756897	1.4082068	68.315095
														HLVFPLLEFLSVK	4	2	1	N-Term(iTRAQ8plex); K13(iTRAQ8plex)	-	1.447	1.878	3.8	25	0	2150.31003	3	717.4415283	-4.54333976	65.82981
														MLFDYLADK	2	2	1	N-Term(iTRAQ8plex); K9(iTRAQ8plex)	0.911	2.443	2.506	3.73	37	0	1723.94365	2	862.4754639	-6.543244971	69.83763167
														IAHFLDR	4	2	1	N-Term(iTRAQ8plex)	0.816	2.362	2.039	3.05	32	0	1175.67459	3	392.5630493	-7.881943544	26.87742667
4503523	EIF3D	eukaryotic translation initiation factor 3 subunit D	NP_003744.1	15.33	5	5	9	548	63.9	6.05	1.097	2.013	2.172																
														NLAMEATYINHNFSQQCLR	2	1	1	N-Term(iTRAQ8plex); C17(Methylthio)	0.899	1.768	1.962	4.61	39	0	2603.2421	3	868.4188843	-1.917884614	46.46662333
														YNFPNPNPFVEDDMDKNEIASVAYR	2	1	1	N-Term(iTRAQ8plex); K16(iTRAQ8plex)	-	-	-	4.53	-	1	3553.74502	4	889.1917114	-1.934713055	43.85960167
														WTCCALLAGSEYLK	2	1	1	N-Term(iTRAQ8plex); C3(Methylthio); C4(Methylthio); K14(iTRAQ8plex)	-	4.698	4.711	4.5	28	0	2258.11704	3	753.3771973	-5.838826288	84.01576167
														SDWEVKEEMDFPQLMK	1	1	1	N-Term(iTRAQ8plex); K6(iTRAQ8plex); K16(iTRAQ8plex)	1.097	2.173	2.299	3.41	-	1	2924.52408	3	975.5128784	-2.082149411	42.18547
														YNFPNPNPFVEDDMDKNEIASVAYR	1	1	1	N-Term(iTRAQ8plex); M14(Oxidation); K16(iTRAQ8plex)	-	-	-	3.39	-	1	3569.7416	4	889.1917114	-1.934713055	43.85960167
														LGDDIDLIVR	1	1	1	N-Term(iTRAQ8plex)	1.198	1.866	2.052	3.33	-	0	1432.8272	2	716.9172363	-2.893066073	43.55226833
4503529	EIF4A1	eukaryotic initiation factor 4A-I isoform 1	NP_001407.1	32.51	5	10	67	406	46.1	5.48	0.977	1.821	2.425																
														LQMEAPHIIVGTPGR	2	2	1	N-Term(iTRAQ8plex)	0.761	2.925	4.042	4.72	53	0	1923.07305	2	962.0401611	-3.00334633	29.616325
														LQMEAPHIIVGTPGR	5	2	1	N-Term(iTRAQ8plex); M3(Oxidation)	1.288	1.129	1.601	4.27	48	0	1939.06583	2	962.0401611	-3.00334633	29.616325
														DFTVSAMHGDMDQK	2	2	1	N-Term(iTRAQ8plex); K14(iTRAQ8plex)	0.968	1.275	1.522	4.11	-	0	2190.05967	3	730.6914063	-8.376849402	23.50810833
														GFKDQIYDIFQK	4	2	1	N-Term(iTRAQ8plex); K3(iTRAQ8plex); K12(iTRAQ8plex)	0.871	2.603	4.281	3.27	27	1	2414.3624	3	805.4584351	-9.915408954	41.04181833
														KEELTLEGIR	1	2	1	N-Term(iTRAQ8plex); K1(iTRAQ8plex)	0.783	1.756	2.652	3.26	-	1	1796.06169	2	898.5344849	-6.747091976	22.150185
														DIETFYNTSIEEMPLNVADLI	1	1	1	N-Term(iTRAQ8plex); Y6(iTRAQ8plex)	-	-	-	-	24	0	3035.58854	3	1012.534363	4.652748139	56.37042667
														GYDVIAQAQSGTGK	6	3	2	N-Term(iTRAQ8plex); K14(iTRAQ8plex)	-	-	-	5.39	72	0	2003.09026	2	1002.048767	-5.767957918	33.57006667
														MFVLDEADEMLSR	16	3	2	N-Term(iTRAQ8plex)	-	-	-	4.67	75	0	1859.91391	3	620.6419067	-3.970262283	58.32259167
														MFVLDEADEMLSR	12	3	2	N-Term(iTRAQ8plex); M1(Oxidation)	-	-	-	3.85	58	0	1875.90799	3	620.6419067	-3.970262283	58.32259167
														VLITTDLLAR	8	2	2	N-Term(iTRAQ8plex)	-	-	-	3.36	41	0	1418.88054	2	709.9452515	-3.682466757	44.96066
														GIYAYGFEKPSAIQQR	9	5	3	N-Term(iTRAQ8plex); K9(iTRAQ8plex)	-	-	-	4.36	33	0	2436.33755	4	609.8397827	-5.044589113	29.05811333
														VDWLTEK	1	5	3	N-Term(iTRAQ8plex); K7(iTRAQ8plex)	-	-	-	3.27	-	0	1498.85893	2	749.9331055	-9.098242795	30.08201167
4503535	EIF4E	eukaryotic translation initiation factor 4E isoform 1	NP_001959.1	16.59	4	4	9	217	25.1	6.15	0.874	3.008	2.820																
														IAIWTTECENR	2	4	1	N-Term(iTRAQ8plex); C8(Methylthio)	0.872	2.531	2.135	3.76	57	0	1685.84014	2	843.4237061	6.425988531	42.15341833
														WLITLNK	3	4	1	N-Term(iTRAQ8plex); K7(iTRAQ8plex)	0.895	3.008	3.063	3.58	25	0	1495.9334	2	748.4703369	-8.204972158	35.41540667
														DGIEPMWEDEK	2	4	1	N-Term(iTRAQ8plex); K11(iTRAQ8plex)	0.932	1.869	1.287	3.38	32	0	1956.96457	3	652.993042	-9.600875611	37.56747667
														WALWFFK	2	5	1	N-Term(iTRAQ8plex); K7(iTRAQ8plex)	0.532	3.745	2.938	-	25	0	1605.93161	3	535.9820557	-5.34739944	53.81456333
4503571	ENO1	alpha-enolase isoform 1	NP_001419.1	75.81	31	31	388	434	47.1	7.39	1.228	4.162	5.731																
														FTASAGIQVVGDDLTVTNPK	6	3	1	N-Term(iTRAQ8plex); K20(iTRAQ8plex)	1.37	7.471	9.181	7.84	91	0	2641.47734	2	1321.24231	4.376800982	38.503005
														SGETEDTFIADLVVGLCTGQIK	54	7	1	N-Term(iTRAQ8plex); C17(Methylthio); K22(iTRAQ8plex)	-	4.114	4.332	7.23	62	0	2950.51371	3	984.1799927	-3.673082281	69.29130667
														DATNVGDEGGFAPNILENK	4	3	1	N-Term(iTRAQ8plex); K19(iTRAQ8plex)	1.138	4.192	7.059	6.36	61	0	2569.31961	3	857.1113892	-6.183376114	26.8568
														LAQANGWGVMVSHR	12	3	1	N-Term(iTRAQ8plex)	1.008	3.052	3.854	6.01	75	0	1829.96738	2	915.4873657	-3.948963501	30.40861333
														LAMQEFMILPVGAANFR	10	3	1	N-Term(iTRAQ8plex)	0.86	3.602	4.807	5.9	89	0	2212.19034	2	1106.596802	-2.791086944	60.90711667
														VNQIGSVTESIQACK	24	3	1	N-Term(iTRAQ8plex); C14(Methylthio); K15(iTRAQ8plex)	1.135	3.901	6.544	5.89	81	0	2231.17803	2	1116.094727	-7.290213758	41.192115
														DYPVVSIEDPFDQDDWGAWQK	22	3	1	N-Term(iTRAQ8plex); K21(iTRAQ8plex)	2.366	1.592	1.368	5.73	63	0	3118.50473	3	1040.178955	-1.019494269	55.62278333
														AAVPSGASTGIYEALELR	16	6	1	N-Term(iTRAQ8plex)	1.496	2.521	3.343	5.66	87	0	2109.14263	3	703.718811	-3.566115966	44.63147667
														YISPDQLADLYK	50	3	1	N-Term(iTRAQ8plex); K12(iTRAQ8plex)	1.238	4.788	7.089	5.64	62	0	2034.12261	2	1017.562805	-9.069073383	39.60770667
														FGANAILGVSLAVCK	50	7	1	N-Term(iTRAQ8plex); C14(Methylthio); K15(iTRAQ8plex)	1.136	4.349	6.958	5.36	74	0	2117.19541	2	1059.103149	-3.84946894	52.52376667
														VVIGMDVAASEFFR	51	3	1	N-Term(iTRAQ8plex)	1.457	5.505	7.515	5.03	74	0	1844.9948	2	923.0010376	3.532408502	59.49072833
														LAQANGWGVMVSHR	6	3	1	N-Term(iTRAQ8plex); M10(Oxidation)	0.9	1.207	1.889	4.9	49	0	1845.96292	2	915.4873657	-3.948963501	30.40861333
														IDKLMIEMDGTENK	2	2	1	N-Term(iTRAQ8plex); K3(iTRAQ8plex); K14(iTRAQ8plex)	-	-	-	4.9	40	1	2549.38393	4	638.1014404	-9.53934829	37.329595
														HIADLAGNSEVILPVPAFNVINGGSHAGNK	7	3	1	N-Term(iTRAQ8plex); K30(iTRAQ8plex)	-	-	-	4.89	32	0	3619.9852	3	1207.333252	1.268322691	44.00858
														LMIEMDGTENK	10	2	1	N-Term(iTRAQ8plex); K11(iTRAQ8plex)	1.074	3.508	4.644	4.87	50	0	1888.98113	3	635.6626587	-9.655502571	31.11239167
														GNPTVEVDLFTSK	24	2	1	N-Term(iTRAQ8plex); K13(iTRAQ8plex)	1.89	4.488	6.124	4.86	72	0	2015.10917	2	1008.057922	-9.132606416	49.490455
														IGAEVYHNLK	4	3	1	N-Term(iTRAQ8plex); K10(iTRAQ8plex)	1.234	2.753	3.878	4.67	46	0	1752.01079	2	876.5090332	-8.933457397	26.74979667
														SKFGANAILGVSLAVCK	11	6	1	N-Term(iTRAQ8plex); K2(iTRAQ8plex); C16(Methylthio); K17(iTRAQ8plex)	2.971	17.452	22.714	4.64	57	1	2636.53434	2	1318.770264	-2.382513571	52.27445833
														YGKDATNVGDEGGFAPNILENK	1	3	1	N-Term(iTRAQ8plex); K3(iTRAQ8plex); K22(iTRAQ8plex)	-	-	-	4.15	-	1	3221.70864	4	806.1826172	-3.71927798	32.51925667
														SFIKDYPVVSIEDPFDQDDWGAWQK	6	3	1	N-Term(iTRAQ8plex); K4(iTRAQ8plex); K25(iTRAQ8plex)	0.981	1.154	3.012	4.09	29	1	3898.00153	3	1300.003906	-3.380146121	48.76827333
														IEEELGSK	6	3	1	N-Term(iTRAQ8plex); K8(iTRAQ8plex)	0.992	3.524	4.609	3.69	29	0	1512.85906	2	756.9334717	-8.794312465	20.179085
														LAMQEFMILPVGAANFR	3	3	1	N-Term(iTRAQ8plex); M7(Oxidation)	1.049	3.069	4.94	3.61	71	0	2228.179	2	1106.596802	-2.791086944	60.90711667
														LNVTEQEK	6	2	1	N-Term(iTRAQ8plex); K8(iTRAQ8plex)	1.09	1.752	3.252	3.54	41	0	1568.89725	3	523.6372681	-8.38828086	13.96593167
														VVIGMDVAASEFFR	4	3	1	N-Term(iTRAQ8plex); M5(Oxidation)	1.103	2.456	3.62	3.47	64	0	1860.97478	2	923.0010376	3.532408502	59.49072833
														TIAPALVSK	2	1	1	N-Term(iTRAQ8plex); K9(iTRAQ8plex)	1.305	3.421	4.601	3.46	35	0	1507.95256	2	754.4799194	-9.449271263	19.421515
														LMIEMDGTENK	2	2	1	N-Term(iTRAQ8plex); M5(Oxidation); K11(iTRAQ8plex)	0.848	4.301	7.023	3.43	61	0	1904.97342	3	635.6626587	-9.655502571	31.11239167
														AVEHINK	4	1	1	N-Term(iTRAQ8plex); K7(iTRAQ8plex)	1.639	6.15	7.805	2.89	40	0	1418.84416	2	709.9257202	-9.456634248	17.15810167
														SCNCLLLK	3	3	1	N-Term(iTRAQ8plex); C2(Methylthio); C4(Methylthio); K8(iTRAQ8plex)	2.852	18.002	28.679	2.85	39	0	1593.83489	2	797.4210815	-6.046318941	37.57337333
														KLNVTEQEK	1	1	1	N-Term(iTRAQ8plex); K1(iTRAQ8plex); K9(iTRAQ8plex)	1.361	9.139	13.488	2.82	-	1	2001.19267	2	1001.099976	-9.027990335	18.78342667
														YDLDFKSPDDPSR	1	3	1	N-Term(iTRAQ8plex); K6(iTRAQ8plex)	1.354	5.653	6.695	2.79	-	1	2163.10581	3	721.7067871	-5.572183215	22.9578
														SGKYDLDFK	1	3	1	N-Term(iTRAQ8plex); K3(iTRAQ8plex); K9(iTRAQ8plex)	-	5.912	8.119	2.71	-	1	1985.12807	4	497.0374756	-9.570584646	29.88313667
														DYPVVSIEDPFDQDDWGAWQK	2	3	1	N-Term(iTRAQ8plex); Y2(iTRAQ8plex); K21(iTRAQ8plex)	-	-	-	-	53	0	3422.72988	3	1040.178955	-1.019494269	55.62278333
														AGAVEK	1	3	1	N-Term(iTRAQ8plex); K6(iTRAQ8plex)	1.485	4.699	5.465	-	32	0	1182.71921	3	394.9112549	-9.342793671	11.85829833
														YISPDQLADLYK	1	3	1	N-Term(iTRAQ8plex); Y1(iTRAQ8plex); K12(iTRAQ8plex)	1.431	4.055	5.925	-	31	0	2338.3198	2	1017.562805	-9.069073383	39.60770667
														YNQLLR	1	3	1	N-Term(iTRAQ8plex)	0.685	3.446	4.682	-	26	0	1110.64934	2	555.8283081	-7.15951361	30.16015833
														EIFDSR	1	2	1	N-Term(iTRAQ8plex)	1.105	2.78	4.098	-	23	0	1070.57036	2	535.7888184	-7.501796752	15.49253667
														GVPLYR	1	6	1	N-Term(iTRAQ8plex)	1.072	2.912	3.626	-	21	0	1008.60576	2	504.8065186	-8.537340319	15.35636667
														SPDDPSR	2	3	1	N-Term(iTRAQ8plex)	1.193	9.171	12.481	-	19	0	1077.54155	2	539.2749023	-4.912318876	7.224821667
4503607	ETFA	electron transfer flavoprotein subunit alpha, mitochondrial isoform a	NP_000117.1	52.55	11	11	52	333	35.1	8.38	0.777	0.798	0.772																
														AAVDAGFVPNDMQVGQTGK	2	2	1	N-Term(iTRAQ8plex); K19(iTRAQ8plex)	0.83	0.706	0.604	7	53	0	2513.33384	3	838.4494629	2.353098419	30.858495
														LLYDLADQLHAAVGASR	25	2	1	N-Term(iTRAQ8plex)	0.778	0.788	0.919	6.43	61	0	2117.16739	3	706.3943481	1.313019538	60.714315
														VVPEMTEILK	4	2	1	N-Term(iTRAQ8plex); K10(iTRAQ8plex)	0.856	1.295	0.867	4.78	49	0	1767.0604	3	589.6916504	3.246420938	38.36208167
														GLLPEELTPLILATQK	8	2	1	N-Term(iTRAQ8plex); K16(iTRAQ8plex)	0.497	0.589	0.474	4.72	42	0	2344.42765	3	782.1473999	-1.524825462	52.26554667
														LEVAPISDIIAIK	4	2	1	N-Term(iTRAQ8plex); K13(iTRAQ8plex)	0.828	0.905	0.811	4.38	37	0	1990.23612	3	664.0835571	-2.398289951	51.91147833
														GTSFDAAATSGGSASSEK	2	2	1	N-Term(iTRAQ8plex); K18(iTRAQ8plex)	0.443	0.27	0.837	3.79	54	0	2239.12259	3	747.0457153	-3.264025797	15.05578333
														IVAPELYIAVGISGAIQHLAGMKDSK	1	2	1	N-Term(iTRAQ8plex); K23(iTRAQ8plex); K26(iTRAQ8plex)	-	-	-	3.6	-	1	3594.07656	4	899.2745972	-2.543771112	69.39491667

														LGGEVSCLVAGTK	1	1	1	N-Term(iTRAQ8plex); C7(Methylthio); K13(iTRAQ8plex)	0.987	1.241	0.724	3.47	-	0	1888.03489	3	630.0164795	-7.614834636	32.65224833
														IVAPELYIAVGISGAIQHLAGMK	2	2	1	N-Term(iTRAQ8plex); K23(iTRAQ8plex)	-	-	-	3.39	33	0	2959.72573	4	740.6868896	-0.223867449	73.043185
														TIVAINKDPEAPIFQVADYGIVADLFK	2	2	1	N-Term(iTRAQ8plex); K7(iTRAQ8plex); K27(iTRAQ8plex)	0.344	0.994	0.302	3.34	31	1	3860.20127	3	1287.405273	0.908181273	65.16921333
														QFNYTHICAGASAFGK	1	2	1	N-Term(iTRAQ8plex); C8(Methylthio); K16(iTRAQ8plex)	0.79	0.995	0.719	3.33	-	0	2369.18918	3	790.4012451	-4.18717024	39.195785
4503609	ETFB	electron transfer flavoprotein subunit beta isoform 1	NP_001976.1	42.35	10	10	60	255	27.8	8.10	0.797	0.716	0.673																
														HSMNPFCEIAVEEAVR	2	2	1	N-Term(iTRAQ8plex); M3(Oxidation); C7(Methylthio)	0.925	1.147	0.873	4.99	44	0	2198.03189	3	728.017395	-0.967211278	46.265045
														HSMNPFCEIAVEEAVR	14	2	1	N-Term(iTRAQ8plex); C7(Methylthio)	1.34	1.017	0.814	4.61	57	0	2182.04148	3	728.017395	-0.967211278	46.265045
														GIHVEVPPAEAER	19	2	1	N-Term(iTRAQ8plex)	0.754	0.638	0.703	4.18	47	0	1707.92813	3	569.980896	-2.9606408	18.25151333
														VETTEDLVAK	2	2	1	N-Term(iTRAQ8plex); K10(iTRAQ8plex)	0.952	0.682	0.575	4.17	49	0	1712.98845	2	856.9978638	-0.362162942	32.99271333
														EKVDLVLLGK	4	2	1	N-Term(iTRAQ8plex); K2(iTRAQ8plex); K10(iTRAQ8plex)	0.791	0.746	0.769	4.13	28	1	2026.29814	2	1013.65271	-2.856439534	28.896945
														LKLPAVVTADLR	6	2	1	N-Term(iTRAQ8plex); K2(iTRAQ8plex)	0.859	0.729	0.826	4.12	43	1	1904.21218	4	476.8094482	0.326532684	35.73266
														IEVIKPGDLGVDLTSK	2	2	1	N-Term(iTRAQ8plex); K5(iTRAQ8plex); K16(iTRAQ8plex)	0.643	0.336	0.401	4.06	26	0	2596.57387	4	649.8989258	1.914809762	36.63239833
														LSVISVEDPPQR	2	2	1	N-Term(iTRAQ8plex)	0.562	0.56	0.673	3.77	25	0	1643.92583	2	822.4665527	-0.731451036	31.994285
														EIDGGLETLR	6	2	1	N-Term(iTRAQ8plex)	0.785	0.621	0.567	3.66	38	0	1406.77849	2	703.8928833	-0.576720815	28.75288
														VDLVLLGK	2	2	1	N-Term(iTRAQ8plex); K8(iTRAQ8plex)	0.541	0.484	0.564	3.04	33	0	1464.96155	3	488.9920349	0.375563664	59.61841667
														TALAMGADR	1	2	1	N-Term(iTRAQ8plex)	0.674	0.675	0.754	-	23	0	1209.65288	2	605.3300781	-2.869972087	18.82674333
4503641	F3	tissue factor isoform 1 precursor	NP_001984.1	8.47	2	2	6	295	33.0	7.03	6.049	1.919	0.859																
														CFYTTDTECDLTDEIVK	4	2	1	N-Term(iTRAQ8plex); C1(Methylthio); C9(Methylthio); K17(iTRAQ8plex)	4.946	1.678	0.757	4.97	67	0	2696.26078	3	899.4251099	6.806848954	48.48340167
														AGVGQSWK	2	1	1	N-Term(iTRAQ8plex); K8(iTRAQ8plex)	6.737	2.593	1.148	3.35	34	0	1440.8451	3	480.9532166	2.189762252	16.177285
4503659	FAU	ubiquitin-like protein fubi and ribosomal protein S30 precursor	NP_001988.1	8.27	2	2	5	133	14.4	10.17	1.430	3.058	2.929																
														FVNVVPTFGK	4	1	1	N-Term(iTRAQ8plex); K10(iTRAQ8plex)	1.284	2.425	2.548	3.5	34	0	1716.01518	2	858.5112305	-8.914246587	61.77959833
														FVNVVPTFGKK	1	1	1	N-Term(iTRAQ8plex); K10(iTRAQ8plex); K11(iTRAQ8plex)	1.89	3.869	2.929	2.93	-	1	2148.3107	3	716.7750854	-9.360006593	31.52334167
4503727	FKBP3	peptidyl-prolyl cis-trans isomerase FKBP3	NP_002004.1	8.48	2	2	5	224	25.2	9.28	1.174	3.013	2.442																
														GWDEALLTMSK	4	4	1	N-Term(iTRAQ8plex); K11(iTRAQ8plex)	0.944	2.295	1.915	4.67	40	0	1859.0064	2	930.0068359	-6.963959367	64.49871167
														AWTVEQLR	1	2	1	N-Term(iTRAQ8plex)	1.38	3.67	3.137	-	26	0	1306.73711	2	653.8721924	-3.82873827	22.07933167
4503771	FNTA	protein farnesyltransferase/geranylgeranyltransferase type-1 subunit alpha	NP_002018.1	8.44	2	2	5	379	44.4	5.08	0.829	1.441	2.247																
														DPSQELEFIADILNQDAK	2	1	1	N-Term(iTRAQ8plex); K18(iTRAQ8plex)	0.933	2.778	3.75	5.8	76	0	2654.42942	3	885.4813232	6.029610908	61.89583333
														LWDNELQYVDQLLK	3	1	1	N-Term(iTRAQ8plex); K14(iTRAQ8plex)	0.737	1.248	1.877	3.36	35	0	2385.30649	3	795.7785034	-2.726801219	54.379865
4503841	XRCC6	X-ray repair cross-complementing protein 6 isoform 1	NP_001460.1	39.41	16	16	98	609	69.8	6.64	1.442	2.012	1.840																
														NLEALALDLMEPEQAVDLTLPK	4	3	1	N-Term(iTRAQ8plex); K22(iTRAQ8plex)	1.909	2.957	2.641	5.88	66	0	3031.68901	2	1516.348145	1.435220592	64.873615
														IQVTPPGFQLVFLPFADDKR	17	3	1	N-Term(iTRAQ8plex); K19(iTRAQ8plex)	1.822	2.417	2.06	5.48	36	1	2896.65561	3	966.2233887	0.349183942	58.76339667
														SDSFENPVLQQHFR	18	3	1	N-Term(iTRAQ8plex)	0.835	1.288	1.227	5.25	58	0	2008.0125	2	1004.509888	-3.253231431	26.676575
														NIYVLQELDNPGAK	8	2	1	N-Term(iTRAQ8plex); K14(iTRAQ8plex)	1.873	2.221	1.831	5.22	68	0	2182.2144	2	1091.613037	-6.430932254	34.14847167
														TFNTSTGGLLLPSDTKR	6	3	1	N-Term(iTRAQ8plex); K16(iTRAQ8plex)	1.621	2.228	2.024	4.86	31	1	2416.35146	3	806.1244507	-2.835878191	27.19206333
														DSLIFLVDASK	4	2	1	N-Term(iTRAQ8plex); K11(iTRAQ8plex)	1.219	1.827	1.876	4.65	48	0	1816.05107	2	908.5291748	-9.134275391	68.16837167
														DIISIAEDEDLR	5	3	1	N-Term(iTRAQ8plex)	1.387	1.608	1.661	4.65	58	0	1692.89226	2	846.9497681	-2.092151563	39.49111
														DTGIFLDLMHLK	17	3	1	N-Term(iTRAQ8plex); K12(iTRAQ8plex)	1.518	2.209	1.957	4.61	43	0	2011.14482	2	1006.07605	-2.917895598	54.31679333
														IISSDRDLLAVVFYGTEK	4	2	1	N-Term(iTRAQ8plex); K18(iTRAQ8plex)	2.646	2.648	3.432	4.41	26	1	2634.49692	3	878.8371582	0.220178422	57.07504333
														IMLFTNEDNPHGNDSAK	4	3	1	N-Term(iTRAQ8plex); K17(iTRAQ8plex)	1.675	2.03	1.926	3.89	31	0	2511.26291	3	837.7591553	-5.156262063	28.89997667
														ELVYPPDYNPEGK	2	3	1	N-Term(iTRAQ8plex); K13(iTRAQ8plex)	0.958	1.283	1.214	3.83	39	0	2129.11832	3	710.3776245	-9.018293939	22.81486667
														LGSLVDEFK	2	3	1	N-Term(iTRAQ8plex); K9(iTRAQ8plex)	1.487	1.927	1.864	3.76	35	0	1615.93559	2	808.4714355	-9.880855589	68.55371167
														ILELDQFK	3	3	1	N-Term(iTRAQ8plex); K8(iTRAQ8plex)	1.905	2.055	1.648	3.74	38	0	1613.95647	2	807.4818726	-9.803735802	65.51401833
														VHFEESSKLEDLLR	1	3	1	N-Term(iTRAQ8plex); K8(iTRAQ8plex)	-	1.659	2.646	3.32	-	1	2310.27529	4	578.3242798	-6.982734677	37.59057333
														DTGIFLDLMHLK	1	3	1	N-Term(iTRAQ8plex); M9(Oxidation); K12(iTRAQ8plex)	0.955	1.302	1.397	3.12	-	0	2027.14634	2	1006.07605	-2.917895598	54.31679333
														FDDPGLMLMGFKPLVLLKK	1	3	1	N-Term(iTRAQ8plex); K12(iTRAQ8plex); K18(iTRAQ8plex); K19(iTRAQ8plex)	1.04	2.587	1.708	2.76	-	1	3379.03269	3	1127.015747	-0.146000086	58.31728167
														NIPPYFVALVPQEEELDDQK	1	3	1	N-Term(iTRAQ8plex); K20(iTRAQ8plex)	1.841	2.471	2.436	-	27	0	2952.59282	2	1476.800049	3.820281676	46.63993
4503971	GDI1	rab GDP dissociation inhibitor alpha	NP_001484.1	19.02	2	7	30	447	50.6	5.14	0.397	1.980	2.599																
														FLVFVANFDENDPK	6	1	1	N-Term(iTRAQ8plex); K14(iTRAQ8plex)	0.409	2.402	3.29	5.18	61	0	2263.20133	3	755.0719604	-9.112402899	79.58795667
														QLICDPSYIPDR	1	1	1	N-Term(iTRAQ8plex); C4(Methylthio)	0.073	0.28	0.316	3.54	-	0	1769.89421	2	885.4507446	4.181231714	36.2434
														FDLGQDVIDFTGHALALYR	2	4	2	N-Term(iTRAQ8plex)	-	-	-	5.7	59	0	2455.29032	3	819.1016235	-0.840611196	68.32359333
														SPYLYPLYGLGELPQGFAR	4	4	2	N-Term(iTRAQ8plex)	-	-	-	4.33	76	0	2445.30669	2	1223.156982	-2.18503318	58.673125
														KFDLGQDVIDFTGHALALYR	11	4	2	N-Term(iTRAQ8plex); K1(iTRAQ8plex)	-	-	-	4.16	46	1	2887.59145	4	722.6541748	0.747348173	59.09888833
														GRDWNVDLIPK	1	4	2	N-Term(iTRAQ8plex); K11(iTRAQ8plex)	-	-	-	2.71	-	1	1921.09348	3	641.0360107	-9.433337646	28.10433833
														FDLGQDVIDFTGHALALYR	2	4	2	N-Term(iTRAQ8plex); Y18(iTRAQ8plex)	-	-	-	-	34	0	2759.50088	3	819.1016235	-0.840611196	68.32359333
														MLLYTEVTR	2	3	2	N-Term(iTRAQ8plex)	-	-	-	3.43	31	0	1429.79424	2	715.4007568	-5.891930396	38.35435667
														MLLYTEVTR	1	3	2	N-Term(iTRAQ8plex); M1(Oxidation)	-	-	-	-	23	0	1445.7918	2	715.4007568	-5.891930396	38.35435667
4503987	GGH	gamma-glutamyl hydrolase precursor	NP_003869.1	12.58	3	3	11	318	35.9	7.11	0.635	0.505	0.866																
														TAFYLAEFFVNEAR	6	1	1	N-Term(iTRAQ8plex)	0.824	1.093	1.737	4.96	59	0	1982.05071	2	991.5289917	9.154901222	62.05900833
														NLDGISHAPNAVK	3	1	1	N-Term(iTRAQ8plex); K13(iTRAQ8plex)	0.441	0.408	0.821	3.9	27	0	1944.0994	3	648.7068481	-3.256055192	18.17666
														LDLTEKDYEILFK	2	2	1	N-Term(iTRAQ8plex); K6(iTRAQ8plex); K13(iTRAQ8plex)	0.69	0.646	1.178	3.61	23	1	2539.45986	4	635.6204224	-7.403829127	45.97441
4504009	GLA	alpha-galactosidase A precursor	NP_000160.1	3.96	1	1	3	429	48.7	5.60	0.805	0.657	1.391																
														FDGCYCDSLENLADGYK	3	1	1	N-Term(iTRAQ8plex); C4(Methylthio); C6(Methylthio); K17(iTRAQ8plex)	0.805	0.657	1.391	4.59	40	0	2613.15177	3	871.7221069	-2.776627561	46.69657167
4504061	GNS	N-acetylglucosamine-6-sulfatase precursor	NP_002067.1	13.04	5	5	11	552	62.0	8.31	0.540	0.553	1.256																
														WQTLLSVDDLVEK	4	1	1	N-Term(iTRAQ8plex); K13(iTRAQ8plex)	0.604	0.624	1.418	5.5	42	0	2154.21128	3	718.7419434	-7.155506073	75.94404833
														YLNEYGAPDAGGLEHVPLGWSYWYALEK	2	1	1	N-Term(iTRAQ8plex); K28(iTRAQ8plex)	-	-	-	4.37	27	0	3806.92739	4	952.4873047	-1.093383082	57.07971333
														SDVLVEYQGEGR	2	1	1	N-Term(iTRAQ8plex)	0.54	0.541	1.307	4.35	29	0	1655.85637	2	828.4318237	1.280027045	27.37151667
														LMMLQSCSGPTCR	2	1	1	N-Term(iTRAQ8plex); C7(Methylthio); C12(Methylthio)	0.758	0.674	1.16	3.66	22	0	1822.82195	2	911.9146118	5.542896183	46.30405333
														VPLLVR	1	1	1	N-Term(iTRAQ8plex)	0.429	0.321	1.068	-	27	0	1000.67729	2	500.8422852	-4.774373847	21.23839667
4504067	GOT1	aspartate aminotransferase, cytoplasmic	NP_002070.1	11.62	3	3	5	413	46.2	7.01	0.721	2.547	2.615																
														IANDNSLNHEYLPILGLAEFR	2	1	1	N-Term(iTRAQ8plex)	0.721	3.085	3.715	4.25	36	0	2703.4343	3	901.8162842	-2.419889005	54.98286
														TDDCHPWVLPVVK	1	1	1	N-Term(iTRAQ8plex); C4(Methylthio); K13(iTRAQ8plex)	0.842	2.547	2.615	3.92	-	0	2163.14042	3	721.7183228	-6.799044657	36.05558667
														VGGVQSLGGTGALR	2	1	1	N-Term(iTRAQ8plex)	0.672	1.61	1.913	3.12	25	0	1575.90727	2	788.4572754	-3.037703314	26.59735167
4504151	GRN	granulins precursor	NP_002078.1	10.46	5	5	14	593	63.5	6.83	0.421	0.284	0.543																
														VHCCPHGAFCDLVHTR	2	1	1	N-Term(iTRAQ8plex); C3(Methylthio); C4(Methylthio); C10(Methylthio)	0.725	1.834	2.07	5.43	52	0	2236.96206	4	559.9959717	-2.256391088	39.30674
														CPDGSTCCELPSGK	4	1	1	N-Term(iTRAQ8plex); C1(Methylthio); C7(Methylthio); C8(Methylthio); [illegible]	0.387	0.267	0.514	4.8	34	0	2142.91484	2	1071.96106	-6.188663736	30.15487
														CDMEVSCPDGYTCCR	5	1	1	N-Term(iTRAQ8plex); C1(Methylthio); C7(Methylthio); C13(Methylthio); [illegible]	0.245	0.223	0.367	4.7	58	0	2169.74126	2	1085.374268	3.043328887	46.76189833
														QGWACCPYR	1	1	1	N-Term(iTRAQ8plex); C5(Methylthio); C6(Methylthio)	0.531	0.436	0.472	2.99	-	0	1479.6259	2	740.3165894	-3.258397312	53.40118833
														HCCPAGFR	2	1	1	N-Term(iTRAQ8plex); C2(Methylthio); C3(Methylthio)	0.473	0.3	0.573	-	33	0	1286.5542	3	429.5229187	-2.044567169	20.90717667
4504183	GSTP1	glutathione S-transferase P	NP_000843.1	52.38	10	10	92	210	23.3	5.64	0.975	6.799	1.538																
														DQQEAALVDMVNDGVEDLR	4	2	1	N-Term(iTRAQ8plex)	0.86	10.035	2.143	6.77	95	0	2421.18389	2	1211.095581	-1.295155913	53.86928167
														MLLADQGQSWKEEVVTVETWQEGSLK	2	2	1	N-Term(iTRAQ8plex); K11(iTRAQ8plex); K26(iTRAQ8plex)	-	-	-	5.64	-	1	3904.10024	4	976.7805176	1.84399274	56.04338167
														MLLADQGQSWKEEVVTVETWQEGSLK	1	2	1	N-Term(iTRAQ8plex); M1(Oxidation); K11(iTRAQ8plex); K26(iTRAQ8plex)	-	-	-	5.38	-	1	3920.07998	4	976.7805176	1.84399274	56.04338167
														FQDGDLTLYQSNTILR	16	2	1	N-Term(iTRAQ8plex)	1.131	3.88	1.145	5.31	88	0	2188.14946	2	1094.578369	-2.618486008	43.5362
														ALPGQLKPFETLLSQNQGGK	10	2	1	N-Term(iTRAQ8plex); K7(iTRAQ8plex); K20(iTRAQ8plex)	-	4.433	0.766	5.27	22	0	3038.76333	4	760.4552612	7.43837953	44.49283333
														TLGLYGKDQQEAALVDMVNDGVEDLR	4	2	1	N-Term(iTRAQ8plex); K7(iTRAQ8plex)	-	-	-	4.93	44	1	3457.80613	3	1153.27356	-0.956051017	54.38341167
														PPYTVVYFPVR	37	2	1	N-Term(iTRAQ8plex)	1.021	4.937	1.298	4.74	53	0	1641.92667	3	547.9801636	-2.858732685	38.88628833
														MLLADQGQSWK	2	2	1	N-Term(iTRAQ8plex); K11(iTRAQ8plex)	0.472	2.454	0.626	4.7	54	0	1885.03887	2	943.0230713	-3.901957677	44.28248333
														ASCLYGQLPK	21	2	1	N-Term(iTRAQ8plex); C3(Methylthio); K10(iTRAQ8plex)	0.931	7.38	1.576	3.88	53	0	1733.94219	2	867.4747314	-6.739572673	46.92385667
														DQQEAALVDMVNDGVEDLR	2	2	1	N-Term(iTRAQ8plex); M10(Oxidation)	0.984	7.599	1.538	3.62	82	0	2437.17363	2	1211.095581	-1.295155913	53.86928167
														MPPYTVVYFPVR	2	1	1	N-Term(iTRAQ8plex)	0.841	3.36	0.753	3.12	27	0	1772.96538	2	886.9863281	-3.244096059	46.61675
														TLGLYGK	2	2	1	N-Term(iTRAQ8plex); K7(iTRAQ8plex)	0.848	7.147	1.552	2.7	29	0	1359.84856	2	680.4279175	2.167183191	19.40165
														FQDGDLTLYQSNTILR	1	2	1	N-Term(iTRAQ8plex); Y9(iTRAQ8plex)	1.43	5.764	1.307	-	43	0	2492.35991	2	1094.578369	-2.618486008	43.5362
														PPYTVVYFPVR	1	2	1	N-Term(iTRAQ8plex); Y7(iTRAQ8plex)	0.3	6.139	1.116	-	32	0	1946.13517	3	547.9801636	-2.858732685	38.88628833
4504245	HIST1H2AC	histone H2A type 1-C	NP_003503.1	40.77	1	7	189	130	14.1	11.05	1.807	1.270	0.791																
														NDEELNKLLGR	3	3	1	N-Term(iTRAQ8plex); K7(iTRAQ8plex)	1.807	1.27	0.791	4.03	28	1	1909.08379	2	955.0455322	-6.575650034	27.06728667
														VTIAQGGVLPNIQAVLLPK	39	10	2	N-Term(iTRAQ8plex); K19(iTRAQ8plex)	-	-	-	7.02	76	0	2539.56987	2	1270.292358	-0.862790435	49.553205
														HLQLAIRNDEELNK	3	12	2	N-Term(iTRAQ8plex); K14(iTRAQ8plex)	-	-	-	3.42	28	1	2301.30117	4	576.0807495	-5.380926798	26.793805
														NDEELNK	7	13	3	N-Term(iTRAQ8plex); K7(iTRAQ8plex)	-	-	-	3.69	35	0	1469.80754	2	735.4074097	1.314060434	12.628885
														KGNYAER	1	9	3	N-Term(iTRAQ8plex); K1(iTRAQ8plex)	-	-	-	2.94	-	1	1445.81789	4	362.2099304	-9.821084933	10.03500667
														AGLQFPVGR	34	17	4	N-Term(iTRAQ8plex)	-	-	-	3.56	51	0	1248.73304	3	416.91745	0.925177022	28.85471333
														HLQLAIR	102	17	3	N-Term(iTRAQ8plex)	-	-	-	3.4	39	0	1154.72541	3	385.5799866	-4.966404282	30.15787333
4504255	H2AFZ	histone H2A.Z	NP_002097.1	31.25	2	4	168	128	13.5	10.58	1.087	0.823	0.639																
														ATIAGGGVIPHIHK	25	4	1	N-Term(iTRAQ8plex); K14(iTRAQ8plex)	1.122	0.844	0.707	5.88	66	0	1979.19817	2	990.1027222	-1.472103254	24.064745
														GDEELDSLIK	7	5	1	N-Term(iTRAQ8plex); K10(iTRAQ8plex)	0.885	0.736	0.621	4.02	57	0	1726.96123	2	863.9829102	-5.673331034	39.96444
														AGLQFPVGR	34	17	4	N-Term(iTRAQ8plex)	-	-	-	3.56	51	0	1248.73304	3	416.91745	0.925177022	28.85471333
														HLQLAIR	102	17	3	N-Term(iTRAQ8plex)	-	-	-	3.4	39	0	1154.72541	3	385.5799866	-4.966404282	30.15787333
4504279	H3F3A	histone H3.3	NP_002098.1	44.85	1	5	63	136	15.3	11.27	-	-	-																
														FQSAAIGALQEASEAYLVGLFEDTNLCAIHAK	5	1	1	N-Term(iTRAQ8plex); C27(Methylthio); K32(iTRAQ8plex)	-	-	-	8.03	36	0	4035.05727	4	1009.527527	1.627037979	73.66081167
														EIAQDFK	26	4	2	N-Term(iTRAQ8plex); K7(iTRAQ8plex)	-	-	-	3.43	35	0	1458.84208	2	729.9260864	2.484795726	28.75155667
														STELLIR	30	5	2	N-Term(iTRAQ8plex)	-	-	-	3.14	41	0	1135.69231	2	568.3497925	-5.752402314	28.27890167
														VTIMPK	1	5	2	N-Term(iTRAQ8plex); M4(Oxidation); K6(iTRAQ8plex)	-	-	-	-	31	0	1312.81675	3	438.2770996	3.701413881	23.42421
														YRPGTVALR	1	5	2	N-Term(iTRAQ8plex)	-	-	-	-	26	0	1336.79551	3	446.2700195	-3.580170812	20.97728333
4504319	HIST1H4G	histone H4-like protein type G	NP_003538.1	13.27	1	1	1	98	11.0	11.06	1.373	0.653	0.554																
														TVTAMAVVYVLKR	1	1	1	N-Term(iTRAQ8plex); M5(Oxidation); K12(iTRAQ8plex)	1.373	0.653	0.554	2.78	-	1	2075.23623	4	519.5645142	-6.991753126	45.567165
4504323	HIST2H4A	histone H4	NP_003539.1	63.11	9	9	724	103	11.4	11.36	1.193	0.593	0.645																

														TVTAMDVVYALK	162	1	1	N-Term(iTRAQ8plex); K12(iTRAQ8plex)	1.107	0.586	0.624	6.03	80	0	1919.11333	2	960.0603027	0.050830114	43.63068333
														TVTAMDVVYALKR	11	1	1	N-Term(iTRAQ8plex); M5(Oxidation); K12(iTRAQ8plex)	1.927	1.005	1.053	5.23	43	1	2091.19423	2	1038.110962	0.142591575	46.23053167
														TVTAMDVVYALKR	83	1	1	N-Term(iTRAQ8plex); K12(iTRAQ8plex)	1.797	0.968	0.847	5.01	62	1	2075.21465	2	1038.110962	0.142591575	46.23053167
														KTVTAMDVVYALK	22	1	1	N-Term(iTRAQ8plex); K1(iTRAQ8plex); K13(iTRAQ8plex)	1.112	0.586	0.604	4.93	67	1	2351.42521	3	784.4799194	4.951784457	40.72185333
														TVTAMDVVYALK	51	1	1	N-Term(iTRAQ8plex); M5(Oxidation); K12(iTRAQ8plex)	1.218	0.58	0.629	4.53	60	0	1935.10649	2	960.0603027	0.050830114	43.63068333
														ISGLIYEETR	188	1	1	N-Term(iTRAQ8plex)	1.142	0.594	0.68	4.42	88	0	1484.82245	3	495.6123352	-2.545041503	37.736325
														GVLKVFLENVIR	22	1	1	N-Term(iTRAQ8plex); K4(iTRAQ8plex)	1.204	0.761	0.576	4.41	34	1	1995.26699	4	499.5722046	4.734794606	61.40579333
														DAVTYTEHAK	60	1	1	N-Term(iTRAQ8plex); K10(iTRAQ8plex)	1.206	0.609	0.651	4.28	58	0	1742.95098	2	871.979126	-1.351711499	15.80964667
														KTVTAMDVVYALK	9	1	1	N-Term(iTRAQ8plex); K1(iTRAQ8plex); M6(Oxidation); K13(iTRAQ8plex)	0.979	0.977	0.815	4.11	49	1	2367.40116	3	784.4799194	4.951784457	40.72185333
														VFLENVIR	89	1	1	N-Term(iTRAQ8plex)	1.191	0.488	0.589	3.94	56	0	1293.78312	3	431.9325562	-0.10518994	31.82575833
														DNIQGITKPAIR	17	1	1	N-Term(iTRAQ8plex); K8(iTRAQ8plex)	1.3	0.547	0.629	3.92	45	0	1934.15654	2	967.5825806	-3.348634173	20.68426
														ISGLIYEETR	3	1	1	N-Term(iTRAQ8plex); Y6(iTRAQ8plex)	1.023	0.492	0.629	-	33	0	1789.02861	3	495.6123352	-2.545041503	37.736325
														TVTAMDVVYALK	2	1	1	N-Term(iTRAQ8plex); Y9(iTRAQ8plex); K12(iTRAQ8plex)	1.516	0.476	0.912	-	31	0	2223.32192	2	960.0603027	0.050830114	43.63068333
														TVTAMDVVYALK	1	1	1	N-Term(iTRAQ8plex); M5(Oxidation); Y9(iTRAQ8plex); K12(iTRAQ8plex)	1.093	0.418	0.521	-	26	0	2239.31679	2	960.0603027	0.050830114	43.63068333
														TLYGFGG	4	1	1	N-Term(iTRAQ8plex)	1.596	5.776	1.096	-	26	0	1018.54967	2	509.7784729	-1.406037685	26.83687333
4504425	HMGB1	high mobility group protein B1	NP_002119.1	36.74	5	7	35	215	24.9	5.74	2.431	10.152	7.245																
														LGEMWNNTAADDKQPYEK	1	1	1	N-Term(iTRAQ8plex); K13(iTRAQ8plex); K18(iTRAQ8plex)	-	-	-	5.28	-	1	3022.55019	4	756.3930054	-6.808719234	29.805655
														IKGEHPGLSIGDVAK	2	1	1	N-Term(iTRAQ8plex); K2(iTRAQ8plex); K15(iTRAQ8plex)	2.698	16.39	10.912	4.49	43	1	2433.43852	2	1217.2229	-8.52996225	22.73820333
														RPPSAFFLFCSEYRPK	5	1	1	N-Term(iTRAQ8plex); C10(Methylthio); K16(iTRAQ8plex)	3.545	10.166	7.907	3.5	-	0	2599.36356	3	867.1260376	-5.333135098	53.10655
														KHPDASVNFSEFSK	2	1	1	N-Term(iTRAQ8plex); K1(iTRAQ8plex); K14(iTRAQ8plex)	1.536	4.366	3.432	3.21	-	1	2505.36802	3	835.7941895	-7.374641375	22.12145
														HPDASVNFSEFSK	1	1	1	N-Term(iTRAQ8plex); K13(iTRAQ8plex)	0.859	1.356	1.408	3.15	-	0	2073.08242	4	519.026062	-1.805588187	31.61338833
														MSSYAFFVQTCR	16	2	2	N-Term(iTRAQ8plex); C11(Methylthio)	-	-	-	5.35	59	0	1789.83025	2	895.4187622	-4.192859042	41.095185
														MSSYAFFVQTCREEHK	1	2	2	N-Term(iTRAQ8plex); C11(Methylthio); K16(iTRAQ8plex)	-	-	-	4.02	-	1	2617.26967	4	655.072876	-4.782951438	39.27769667
														MSSYAFFVQTCR	7	2	2	N-Term(iTRAQ8plex); M1(Oxidation); C11(Methylthio)	-	-	-	3.22	32	0	1805.82842	2	895.4187622	-4.192859042	41.095185
4504437	HMOX1	heme oxygenase 1	NP_002124.1	10.07	2	2	12	288	32.8	8.25	0.618	0.813	1.288																
														ESPVFAPVYFPEELHR	2	1	1	N-Term(iTRAQ8plex)	1.287	0.852	1.482	5.03	42	0	2221.15079	3	741.0551147	-3.948544041	40.229245
														EVHTQAENAEFMR	6	1	1	N-Term(iTRAQ8plex)	0.539	0.565	1.341	4.66	26	0	1865.90549	3	627.9728394	-1.462074391	14.87004167
														EVHTQAENAEFMR	4	1	1	N-Term(iTRAQ8plex); M12(Oxidation)	0.716	0.813	1.184	3.97	28	0	1881.90396	3	627.9728394	-1.462074391	14.87004167
4504483	HPRT1	hypoxanthine-guanine phosphoribosyltransferase	NP_000185.1	30.73	4	4	29	218	24.6	6.68	0.768	2.053	2.280																
														SPGVVISDDEPGYDLDLFCIPNHYAEDLER	4	1	1	N-Term(iTRAQ8plex); C19(Methylthio)	-	-	-	6.08	56	0	3728.74509	3	1243.588623	2.953694063	59.64885
														NVLIVEDIIDTGK	6	1	1	N-Term(iTRAQ8plex); K13(iTRAQ8plex)	0.733	2.886	2.523	5.65	59	0	2037.1967	2	1019.10199	-4.186354612	39.85794333
														DLNHVCVISETGK	6	1	1	N-Term(iTRAQ8plex); C6(Methylthio); K13(iTRAQ8plex)	0.604	1.537	1.355	4.46	57	0	2069.08243	2	1035.046509	-5.92113784	27.00473833
														VFIPHGLIMDR	8	1	1	N-Term(iTRAQ8plex)	1.14	6.845	4.222	3.44	41	0	1601.9075	3	534.6389771	-7.237268523	35.80932
														VFIPHGLIMDR	3	1	1	N-Term(iTRAQ8plex); M9(Oxidation)	0.934	2.053	1.231	2.79	35	0	1617.89756	3	534.6389771	-7.237268523	35.80932
														SPGVVISDDEPGYDLDLFCIPNHYAEDLER	2	1	1	N-Term(iTRAQ8plex); C19(Methylthio); Y24(iTRAQ8plex)	-	-	-	-	30	0	4032.93227	3	1243.588623	2.953694063	59.64885
4504505	HSD17B4	peroxisomal multifunctional enzyme type 2 isoform 2	NP_000405.1	17.53	10	10	36	736	79.6	8.84	0.717	0.469	0.521																
														ATSTATSGFAGAIGQK	2	5	1	N-Term(iTRAQ8plex); K16(iTRAQ8plex)	0.557	0.327	0.328	7.95	65	0	2076.16202	3	692.7255249	3.578794408	27.47591167
														LGLLGLANSLAIEGR	5	5	1	N-Term(iTRAQ8plex)	0.73	0.447	0.477	5.68	71	0	1801.08231	3	601.0322876	-1.466760857	60.87674167
														GALVVVNDLGGDFK	4	1	1	N-Term(iTRAQ8plex); K14(iTRAQ8plex)	0.62	0.423	0.51	5.67	65	0	2012.17314	2	1006.59021	4.687470314	40.77597667
														LQSTFVFEEIGR	2	5	1	N-Term(iTRAQ8plex)	0.581	0.318	0.386	3.99	69	0	1729.95781	2	865.4825439	8.745861041	42.85182
														ISDEDWDIIHR	13	4	1	N-Term(iTRAQ8plex)	0.856	0.86	0.761	3.83	46	0	1702.86673	3	568.2927856	-3.780464646	27.72646833
														ICDFENASKPQSIQESTGSIIEVLSK	1	5	1	N-Term(iTRAQ8plex); C2(Methylthio); K9(iTRAQ8plex); K26(iTRAQ8plex)	-	-	-	3.73	-	0	3782.03042	4	946.2630615	4.87143458	57.30945333
														VVLVTGAGAGLGR	4	1	1	N-Term(iTRAQ8plex)	0.912	0.48	0.528	3.69	39	0	1473.90862	2	737.4579468	2.100289429	31.91634333
														LQSTFVFEEIGRR	1	5	1	N-Term(iTRAQ8plex)	0.512	0.407	0.458	3.26	-	1	1886.04087	3	629.3518066	-1.555972714	38.35329167
														IDVVVNNAGILR	2	4	1	N-Term(iTRAQ8plex)	0.717	1.073	0.746	3.17	29	0	1586.9489	2	793.9780884	-2.704032995	43.623915
														AYALAFAER	2	1	1	N-Term(iTRAQ8plex)	0.633	0.373	0.428	2.85	29	0	1315.73039	2	658.3688354	-0.620964269	33.20525167
4504511	DNAJA1	dnaJ homolog subfamily A member 1 isoform 1	NP_001530.1	32.75	7	7	19	397	44.8	7.08	1.445	1.531	1.314																
														IHQIGPGMVQQIQSVCMECQGHGER	4	2	1	N-Term(iTRAQ8plex); C16(Methylthio); C19(Methylthio)	-	-	-	7.53	50	0	3161.46328	4	791.1212769	2.683643975	55.77624833
														NVVHQLSVTLEDLYNGATR	3	1	1	N-Term(iTRAQ8plex)	0.268	0.574	0.749	5.26	52	0	2433.30997	3	811.7748413	2.457301311	62.48194167
														TIVITSHPGQIVK	2	2	1	N-Term(iTRAQ8plex); K13(iTRAQ8plex)	1.94	1.994	1.925	4.96	40	0	2001.22624	2	1001.11676	-2.727034089	23.872825
														CVLNEGMPIYR	3	2	1	N-Term(iTRAQ8plex); C1(Methylthio)	1.301	1.499	1.314	3.82	25	0	1644.81926	2	822.913269	-1.289402497	38.61830833
														QISQAYEVLSDAK	2	1	1	N-Term(iTRAQ8plex); K13(iTRAQ8plex)	1.514	1.933	1.855	3.72	42	0	2060.13333	3	687.3826294	-7.323004851	30.103115
														ITFHGEGDQEPGLEPGDIIIVLDQK	2	2	1	N-Term(iTRAQ8plex); K25(iTRAQ8plex)	-	-	0.914	3.66	34	0	3328.77729	5	666.5612793	-3.401046282	51.18204667
														EGGAGGGFGSPMDIFDMFFGGGGR	2	1	1	N-Term(iTRAQ8plex)	-	-	-	2.85	34	0	2627.2009	3	881.7347412	-0.543251664	66.8818
														EGGAGGGFGSPMDIFDMFFGGGGR	1	1	1	N-Term(iTRAQ8plex); M12(Oxidation)	-	-	-	-	51	0	2643.18967	3	881.7347412	-0.543251664	66.8818
4504517	HSPB1	heat shock protein beta-1	NP_001531.1	80.00	10	10	67	205	22.8	6.40	0.681	1.932	2.106																
														KYTLPPGVDPTQVSSSLSPEGTLTVEAPMPK	1	1	1	N-Term(iTRAQ8plex); K1(iTRAQ8plex); K31(iTRAQ8plex)	-	-	-	5.41	-	1	4139.25112	4	1035.568237	-4.899910797	41.80695167
														LATQSNEITIPVTFESR	4	1	1	N-Term(iTRAQ8plex)	0.544	2.04	1.552	4.99	77	0	2210.19145	2	1105.599365	-2.541612963	41.92121833
														VSLDVNHFAPDELTVK	7	1	1	N-Term(iTRAQ8plex); K16(iTRAQ8plex)	0.678	2.45	2.504	4.4	42	0	2392.32181	3	798.1129761	-3.727056179	34.582105
														LPEEWSQWLGGSSWPGYVRPLPPAAIESPAVAAPAYSR	1	1	1	N-Term(iTRAQ8plex)	-	-	-	4.4	-	0	4397.30922	4	1100.082764	9.781779107	57.96490167
														LFDQAFGLPR	15	1	1	N-Term(iTRAQ8plex)	0.626	2.021	2.354	3.95	56	0	1467.81755	2	734.4124146	-5.88548517	36.74956833
														QLSSGVSEIR	8	1	1	N-Term(iTRAQ8plex)	0.753	1.98	2.148	3.56	51	0	1379.77056	2	690.388916	-6.570577053	16.10489
														GPSWDPFRDWYPHSR	3	1	1	N-Term(iTRAQ8plex)	-	1.869	2.707	3.52	20	1	2207.08999	4	552.5228271	-1.355909399	39.88336833
														AQLGGPEAAK	2	1	1	N-Term(iTRAQ8plex); K10(iTRAQ8plex)	1.126	2.849	3.86	3.51	47	0	1549.90097	3	517.3051758	-9.598810826	20.274235
														RVPFSLLR	24	1	1	N-Term(iTRAQ8plex)	0.69	1.884	1.691	3.49	45	1	1291.80503	3	431.273468	-7.253796892	25.602105
														DGVVEITGK	2	1	1	N-Term(iTRAQ8plex); K9(iTRAQ8plex)	0.738	1.411	1.509	3.4	31	0	1525.89067	2	763.4489746	-9.134796811	29.71596
4504523	HSPE1	10 kDa heat shock protein, mitochondrial	NP_002148.1	57.84	7	7	64	102	10.9	8.92	2.002	0.871	0.658																
														VLQATVVAVGSGSK	17	2	1	N-Term(iTRAQ8plex); K14(iTRAQ8plex)	1.629	0.871	0.753	6.01	85	0	1924.15703	2	962.5821533	-6.107235112	28.59508667
														VVLDDKDYFLFR	27	1	1	N-Term(iTRAQ8plex); K6(iTRAQ8plex)	2.211	0.923	0.669	4.07	41	1	2138.20561	2	1069.606567	-2.236994327	43.48143667
														GGEIQPVSVK	4	1	1	N-Term(iTRAQ8plex); K10(iTRAQ8plex)	2.969	1.493	1.171	3.87	54	0	1621.96526	2	811.4862671	-4.996247777	15.79919833
														VLLPEYGGTK	4	1	1	N-Term(iTRAQ8plex); K10(iTRAQ8plex)	1.841	0.676	0.516	3.36	40	0	1685.01029	2	843.0044556	-4.60936478	22.34254333
														FLPLFDR	4	2	1	N-Term(iTRAQ8plex)	1.811	0.754	0.564	2.81	34	0	1211.69959	3	404.5713806	-7.784688867	36.39031
														KFLPLFDR	6	2	1	N-Term(iTRAQ8plex); K1(iTRAQ8plex)	2.004	0.888	0.446	2.71	36	1	1644.0104	4	411.7581177	0.784376386	36.69097333
														VLVER	2	2	1	N-Term(iTRAQ8plex)	1.898	0.784	0.664	-	24	0	919.58611	2	460.2966919	-1.874898853	21.50167667
4504747	ITGA3	integrin alpha-3 preproprotein	NP_002195.1	16.75	15	15	42	1051	116.5	6.77	0.507	0.363	0.755																
														EAGNPGSLFGYSVALHR	2	2	1	N-Term(iTRAQ8plex)	0.951	1.44	1.516	5.59	50	0	2079.08768	3	693.7007446	-2.337882777	33.25700333
														HMGAVFLLSQEAGGDLR	6	1	1	N-Term(iTRAQ8plex)	0.617	0.647	0.836	5.29	65	0	2105.10697	3	702.3738403	-2.196351053	39.80643167
														YTQVLWSGSEDQR	2	2	1	N-Term(iTRAQ8plex)	0.415	0.189	0.652	5.14	43	0	1872.93889	2	936.9730835	-0.256777098	31.25475833
														TGAVYLCPLTAHKDDCER	3	2	1	N-Term(iTRAQ8plex); C7(Methylthio); K13(iTRAQ8plex); C16(Methylthio)	0.492	0.288	0.388	5.08	39	1	2692.30643	3	898.1069946	-4.149848306	34.948435
														TGAVYLCPLTAHK	2	2	1	N-Term(iTRAQ8plex); C7(Methylthio); K13(iTRAQ8plex)	0.415	0.243	0.675	4.75	47	0	2028.11661	3	676.7103882	-3.18571387	32.561335
														NDPGHHIIEDMWLGVTVASQGPAGR	7	2	1	N-Term(iTRAQ8plex)	0.454	0.287	0.753	4.59	35	0	2961.49601	3	987.836853	0.545174476	46.22986833
														LQSFFGGTVMGESGMK	2	2	1	N-Term(iTRAQ8plex); K16(iTRAQ8plex)	0.64	0.468	0.79	4.15	30	0	2284.18705	3	762.0671997	-2.458392764	39.14065
														VNGWATLFLR	4	2	1	N-Term(iTRAQ8plex)	0.441	0.258	0.692	3.8	41	0	1480.8532	2	740.9302368	-3.122718171	75.37739667
														QPQQVIHGEK	1	2	1	N-Term(iTRAQ8plex); K10(iTRAQ8plex)	0.53	0.411	0.899	3.19	-	0	1772.02384	3	591.3461304	-2.073017665	18.02695167
														VLVCAHR	2	2	1	N-Term(iTRAQ8plex); C4(Methylthio)	0.301	0.374	0.765	2.99	33	0	1147.63373	3	383.216095	-3.869385997	27.48672667
														YLLLAGAPR	5	2	1	N-Term(iTRAQ8plex)	0.458	0.319	0.745	2.9	35	0	1277.78533	2	639.3963013	-2.352023361	36.62213333
														SETVLTCATGR	2	2	1	N-Term(iTRAQ8plex); C7(Methylthio)	0.516	0.264	0.652	2.81	40	0	1487.7437	2	744.3754883	-4.222741794	22.15313667
														ARPVINIVHK	2	2	1	N-Term(iTRAQ8plex); K10(iTRAQ8plex)	0.517	0.351	0.784	2.72	20	0	1755.11916	4	439.5352478	-1.253458518	21.35805333
														CESNLQMR	1	2	1	N-Term(iTRAQ8plex); C1(Methylthio)	0.616	0.363	0.828	-	21	0	1330.6165	2	665.8118896	-4.088047243	29.412865
														FLVVK	1	2	1	N-Term(iTRAQ8plex); K5(iTRAQ8plex)	0.497	0.379	0.691	-	15	0	1213.81487	2	607.4110718	1.65233986	24.50537167
4504901	KPNA4	importin subunit alpha-3	NP_002259.1	9.60	3	3	6	521	57.9	4.96	1.032	1.514	1.339																
														MAEDEAETIGNLIEECGGLEK	2	1	1	N-Term(iTRAQ8plex); M1(Oxidation); C16(Methylthio); K21(iTRAQ8plex)	1.301	1.643	2.912	6.08	45	0	2921.42044	3	974.4783325	5.646913517	61.55755
														NVPHEDICEDSDIDGDYR	2	1	1	N-Term(iTRAQ8plex); C8(Methylthio)	0.87	1.401	1.339	5.99	54	0	2442.04941	3	814.6879883	0.097988906	27.716145
														NVTWVMVNLCR	2	1	1	N-Term(iTRAQ8plex); C10(Methylthio)	1.032	1.514	1.086	3.11	32	0	1684.86138	2	842.9343262	-1.511445563	49.22661167
4504957	LAMP2	lysosome-associated membrane glycoprotein 2 isoform A precursor	NP_002285.1	2.20	1	1	6	410	44.9	5.63	0.524	0.357	0.423																
														YLDFVFAVK	6	3	1	N-Term(iTRAQ8plex); K9(iTRAQ8plex)	0.524	0.357	0.423	4.22	60	0	1709.99785	2	855.5025635	-6.328058759	75.333255
4504981	LGALS1	galectin-1	NP_002296.1	77.78	10	10	221	135	14.7	5.50	0.146	0.213	0.527																
														LNLEAINYMAADGDFK	69	1	1	N-Term(iTRAQ8plex); K16(iTRAQ8plex)	0.148	0.167	0.489	6.64	105	0	2393.26152	2	1197.138184	2.473297935	44.73061667
														LNLEAINYMAADGDFK	12	1	1	N-Term(iTRAQ8plex); M9(Oxidation); K16(iTRAQ8plex)	0.039	0.091	0.382	6.57	77	0	2409.25454	2	1197.138184	2.473297935	44.73061667
														FNAHGDANTIVCNSK	16	1	1	N-Term(iTRAQ8plex); C12(Methylthio); K15(iTRAQ8plex)	0.101	0.21	0.46	5.47	65	0	2245.12065	2	1123.063965	-4.796727733	21.40657
														SFVLNLGKDSNNLCLHFNPR	3	1	1	N-Term(iTRAQ8plex); K8(iTRAQ8plex); C14(Methylthio)	0.33	0.232	0.823	5.27	-	1	2942.55263	4	736.3936157	-2.157748787	47.78893667
														DSNNLCLHFNPR	69	1	1	N-Term(iTRAQ8plex); C6(Methylthio)	0.204	0.339	0.558	4.96	66	0	1779.8532	2	890.4302368	-2.272820699	29.03754333
														EAVFPFQPGSVAEVCITFDQANLTVK	16	1	1	N-Term(iTRAQ8plex); C15(Methylthio); K26(iTRAQ8plex)	-	-	0.444	4.72	55	0	3464.80515	3	1155.605713	-0.847632167	67.50783167
														SFVLNLGK	16	1	1	N-Term(iTRAQ8plex); K8(iTRAQ8plex)	0.083	0.142	0.572	3.53	50	0	1485.92522	2	743.4662476	0.186548545	43.93697
														DGGAWGTEQR	2	1	1	N-Term(iTRAQ8plex)	0.17	0.197	0.415	3.28	30	0	1380.67961	2	690.8434448	-0.991013085	18.32344
														LPDGYEFK	10	1	1	N-Term(iTRAQ8plex); K8(iTRAQ8plex)	0.072	0.131	0.465	3.15	28	0	1576.88066	3	526.2987061	-0.993344061	29.08191667
														GEVAPDAK	5	1	1	N-Term(iTRAQ8plex); K8(iTRAQ8plex)	0.172	0.148	0.579	3.08	29	0	1394.81293	3	465.6091614	2.114678459	12.71831833
														VRGEVAPDAK	2	1	1	N-Term(iTRAQ8plex); K10(iTRAQ8plex)	0.323	0.309	0.691	2.79	-	1	1649.97397	3	550.6628418	-3.363179946	14.25302667
														LNLEAINYMAADGDFK	1	1	1	N-Term(iTRAQ8plex); Y8(iTRAQ8plex); K16(iTRAQ8plex)	-	0.22	1.36	-	30	0	2697.47402	2	1197.138184	2.473297935	44.73061667
4505021	LRPAP1	alpha-2-macroglobulin receptor-associated protein precursor	NP_002328.1	4.20	2	2	3	357	41.4	8.78	0.931	0.726	0.678																
														LNQLWEK	2	1	1	N-Term(iTRAQ8plex); K7(iTRAQ8plex)	0.939	0.725	0.655	3.18	35	0	1538.91289	2	769.960083	-1.437316167	29.01602333
														NLNVILAK	1	1	1	N-Term(iTRAQ8plex); K8(iTRAQ8plex)	0.924	0.728	0.702	-	31	0	1492.96854	3	498.3276978	0.936866171	39.02640667
4505061	M6PR	cation-dependent mannose-6-phosphate receptor isoform 1 precursor	NP_002346.1	15.88	3	3	8	277	31.0	5.83	0.861	0.762	0.797																
														HTLADNFNPVSEER	4	1	1	N-Term(iTRAQ8plex)	0.94	0.762	0.683	5.59	46	0	1932.9675	3	644.9940186	-2.198115557	22.52242667
														AVVMISCNR	2	2	1	N-Term(iTRAQ8plex); C7(Methylthio)	0.879	1.18	0.827	3.04	20	0	1342.6939	2	671.8505859	-0.615959455	31.20353667

														GVGDDQLGEESEERDDHLLPM	2	2	1	N-Term(iTRAQ8plex)	0.784	0.612	0.769	2.86	34	1	2645.22855	3	882.4143677	-0.681046126	32.25830333
4505087	MAGOH	protein mago nashi homolog	NP_002361.1	23.97	2	2	4	146	17.2	6.11	1.063	2.119	2.021																
														IIDDSEITKEDDALWPPPDR	1	3	1	N-Term(iTRAQ8plex); K9(iTRAQ8plex)	-	-	-	4.17	-	1	2933.52724	4	734.1372681	-2.760811032	40.06211
														FGHEFLEFEFRPDGK	3	2	1	N-Term(iTRAQ8plex); K15(iTRAQ8plex)	1.063	2.119	2.021	3.34	28	0	2463.27968	4	616.5753784	-4.907079048	39.49218333
4505185	MIF	macrophage migration inhibitory factor	NP_002406.1	17.39	2	2	48	115	12.5	7.88	0.681	6.152	5.294																
														LLCGLLAER	44	1	1	N-Term(iTRAQ8plex); C3(Methylthio)	0.634	6.471	6.603	3.65	74	0	1337.75479	3	446.5897827	-2.920868222	55.76919833
														PMFIVNTNVPR	4	1	1	N-Term(iTRAQ8plex)	0.904	4.713	3.857	-	52	0	1591.90024	3	531.3049316	4.403775872	33.83145667
4505229	FADD	FAS-associated death domain protein	NP_003815.1	15.87	1	1	1	208	23.3	5.69	-	-	-																
														RVDDFEAGAAAGAAPGEEDLCAAFNVICDNVGK	1	1	1	N-Term(iTRAQ8plex); C21(Methylthio); C28(Methylthio); K33(iTRAQ8plex)	-	-	-	-	25	1	3995.90493	4	999.7316895	4.791063062	66.95572667
4505257	MSN	moesin	NP_002435.1	46.97	15	24	120	577	67.8	6.40	0.728	0.957	2.027																
														FYPEDVSEELIQDITQR	8	4	1	N-Term(iTRAQ8plex)	0.645	1.156	2.711	5.87	74	0	2386.20609	3	796.0756836	1.871682114	63.68634333
														IAQDLEMYGVNYFSIK	12	4	1	N-Term(iTRAQ8plex); K16(iTRAQ8plex)	0.729	0.9	1.932	5.72	50	0	2499.35538	3	833.789978	5.59325502	55.36038667
														TAMSTPHVAEPAENEQDEQDENGAEASADLR	2	4	1	N-Term(iTRAQ8plex)	-	-	-	5.13	44	0	3616.63401	3	1206.216187	2.534129883	25.77431167
														EVWFFGLQYQDTK	4	4	1	N-Term(iTRAQ8plex); K13(iTRAQ8plex)	0.483	0.986	2.273	4.83	47	0	2269.20206	3	757.0722046	-4.098700874	50.911025
														KTQEQLALEMAELTAR	10	4	1	N-Term(iTRAQ8plex); K1(iTRAQ8plex)	0.711	1.156	2.705	4.6	83	1	2440.35723	2	1220.682251	-4.841900502	39.00849167
														TQEQLALEMAELTAR	2	4	1	N-Term(iTRAQ8plex)	0.546	0.928	2.027	4.56	67	0	2008.06182	2	1004.534546	-3.434218545	46.93410667
														IAQDLEMYGVNYFSIK	1	4	1	N-Term(iTRAQ8plex); M7(Oxidation); K16(iTRAQ8plex)	0.336	0.341	0.582	4.01	-	0	2515.32804	3	833.789978	5.59325502	55.36038667
														AQMVQEDLEK	2	4	1	N-Term(iTRAQ8plex); K10(iTRAQ8plex)	0.728	1.336	2.941	3.85	67	0	1798.96733	2	899.9873047	-8.681950314	37.80815667
														IQVWHEEHR	4	4	1	N-Term(iTRAQ8plex)	0.954	0.927	1.554	3.71	42	0	1537.81023	2	769.4087524	-4.879039721	16.13039
														TANDMIHAENMR	4	4	1	N-Term(iTRAQ8plex); M5(Oxidation)	0.535	0.82	1.739	3.71	26	0	1722.81443	3	574.9431763	-3.233511134	12.03379833
														EGILNDDIYCPPETAVLLASYAVQSK	4	4	1	N-Term(iTRAQ8plex); C10(Methylthio); K26(iTRAQ8plex)	-	-	1.382	3.6	42	0	3463.79221	3	1155.268921	-0.80341271	66.40445833
														TANDMIHAENMR	1	4	1	N-Term(iTRAQ8plex); M5(Oxidation); M11(Oxidation)	0.713	0.55	1.157	3.14	-	0	1738.81162	3	574.9431763	-3.233511134	12.03379833
														SGYLAGDK	4	4	1	N-Term(iTRAQ8plex); K8(iTRAQ8plex)	0.897	1.152	2.148	3.12	38	0	1418.79692	2	709.9020996	-9.189970067	13.30239833
														ISQLEMAR	2	4	1	N-Term(iTRAQ8plex)	1.141	1.078	1.754	2.98	-	0	1251.69768	2	626.352478	-4.483403145	26.79554667
														TANDMIHAENMR	2	4	1	N-Term(iTRAQ8plex)	0.425	0.542	1.783	2.93	25	0	1706.81961	3	574.9431763	-3.233511134	12.03379833
														GFSTWLK	3	4	1	N-Term(iTRAQ8plex); K7(iTRAQ8plex)	0.703	1.041	2.87	2.87	25	0	1446.84368	2	723.9249878	-9.553446323	29.79685333
														KESPLLFK	1	4	1	N-Term(iTRAQ8plex); K1(iTRAQ8plex); K8(iTRAQ8plex)	0.569	0.741	2.589	2.79	-	1	1874.17226	4	469.2985229	-8.302002671	27.54069167
														ALTSELANAR	1	4	1	N-Term(iTRAQ8plex)	1.337	1.41	1.974	2.79	-	0	1349.76335	2	675.3853149	-4.228916998	25.62976833
														KTQEQLALEMAELTAR	1	4	1	N-Term(iTRAQ8plex); K1(iTRAQ8plex); M10(Oxidation)	0.814	0.836	1.587	2.79	-	1	2456.35434	2	1220.682251	-4.841900502	39.00849167
														FYPEDVSEELIQDITQR	3	4	1	N-Term(iTRAQ8plex); Y2(iTRAQ8plex)	0.959	1.027	2.706	-	49	0	2690.40689	3	796.0756836	1.871682114	63.68634333
														VTTMDAELEFAIQPNTTGK	4	7	3	N-Term(iTRAQ8plex); M4(Oxidation); K19(iTRAQ8plex)	-	-	-	5.01	62	0	2690.42168	2	1345.714478	1.818361101	34.020625
														APDFVFYAPR	7	9	3	N-Term(iTRAQ8plex)	-	-	-	3.69	38	0	1486.79473	2	743.906311	3.843240361	35.09177167
														KPDTIEVQQMK	2	9	3	N-Term(iTRAQ8plex); K1(iTRAQ8plex); K11(iTRAQ8plex)	-	-	-	3.47	34	0	2229.28287	3	743.7658081	-9.482974937	17.16221
														RKPDTIEVQQMK	1	9	3	N-Term(iTRAQ8plex); K2(iTRAQ8plex); K12(iTRAQ8plex)	-	-	-	3.34	-	1	2385.38759	4	597.102356	-7.351992573	15.829755
														QLFDQVVK	2	7	3	N-Term(iTRAQ8plex); K8(iTRAQ8plex)	-	-	-	3.26	37	0	1584.94194	2	792.9746094	-9.482473522	25.558705
														IGFPWSEIR	11	9	3	N-Term(iTRAQ8plex)	-	-	-	3.21	41	0	1408.79267	3	470.2690735	2.552475654	39.48728
														LFFLQVK	3	8	3	N-Term(iTRAQ8plex); K7(iTRAQ8plex)	-	-	-	3.06	28	0	1502.9406	2	751.973938	-9.921844845	69.32707
														KAPDFVFYAPR	7	9	3	N-Term(iTRAQ8plex); K1(iTRAQ8plex)	-	-	-	2.98	29	1	1919.08756	3	640.3673706	-6.46282841	28.32321167
														ILALCMGNHELYMR	10	7	2	N-Term(iTRAQ8plex); C5(Methylthio)	-	-	-	5.97	58	0	2013.99936	3	672.0046387	-2.730653483	59.78209667
														ILALCMGNHELYMR	2	7	2	N-Term(iTRAQ8plex); C5(Methylthio); M13(Oxidation)	-	-	-	3.61	26	0	2029.99253	3	672.0046387	-2.730653483	59.78209667
4505357	NDUFA4	cytochrome c oxidase subunit NDUFA4	NP_002480.1	27.16	2	2	17	81	9.4	9.38	1.691	0.778	0.688																
														LALFNPDVCWDR	13	1	1	N-Term(iTRAQ8plex); C9(Methylthio)	1.604	0.778	0.737	4.47	57	0	1798.88957	2	899.9484253	-1.488881864	61.038055
														FYSVNVDYSK	4	1	1	N-Term(iTRAQ8plex); K10(iTRAQ8plex)	1.891	0.727	0.63	4.07	42	0	1829.98331	3	610.6659546	-3.3119321	40.24984667
4505409	NME2	nucleoside diphosphate kinase B isoform a	NP_002503.1	59.87	4	7	68	152	17.3	8.41	1.231	3.928	3.152																
														YMNSGPVVAMVWEGLNVVK	14	2	1	N-Term(iTRAQ8plex); K19(iTRAQ8plex)	1.178	3.423	2.621	7.04	69	0	2701.48038	2	1351.243774	5.038314832	62.67860333
														EISLWFKPEELVDYK	12	2	1	N-Term(iTRAQ8plex); K7(iTRAQ8plex); K15(iTRAQ8plex)	1.409	6.618	4.676	5.45	41	0	2808.58076	2	1404.795654	-3.941248018	51.38888833
														DRPFFPGLVK	6	3	1	N-Term(iTRAQ8plex); K10(iTRAQ8plex)	1.151	3.828	3.328	3.76	-	0	1784.05039	3	595.3549805	-9.838928243	28.42036
														SCAHDWVYE	1	2	1	N-Term(iTRAQ8plex); C2(Methylthio)	1.285	4.205	2.959	-	22	0	1459.62346	2	730.3153687	-3.645882281	28.75044167
														VMLGETNPADSKPGTIR	3	4	2	N-Term(iTRAQ8plex); M2(Oxidation); K12(iTRAQ8plex)	-	-	-	5.76	45	0	2410.31394	2	1197.662354	-4.072512953	23.83921333
														VMLGETNPADSKPGTIR	11	4	2	N-Term(iTRAQ8plex); K12(iTRAQ8plex)	-	-	-	4.94	87	0	2394.31743	2	1197.662354	-4.072512953	23.83921333
														TFIAIKPDGVQR	13	5	2	N-Term(iTRAQ8plex); K6(iTRAQ8plex)	-	-	-	3.88	39	0	1953.16631	2	977.086792	-4.041609562	25.15961667
														GDFCIQVGR	8	4	2	N-Term(iTRAQ8plex); C4(Methylthio)	-	-	-	3.68	28	0	1344.67644	2	672.8418579	4.333310336	31.69768333
4505571	SQSTM1	sequestosome-1 isoform 1	NP_003891.1	27.27	7	7	31	440	47.7	5.22	1.105	0.630	1.436																
														SSSQPSSCCSDPSKPGGNVEGATQSLAEQMR	3	2	1	N-Term(iTRAQ8plex); C8(Methylthio); C9(Methylthio); K14(iTRAQ8plex)	2.985	-	3.647	6.42	32	0	3825.72451	4	957.1865845	-4.876276202	35.09728333
														NMVHPNVICDGCNGPVVGTR	2	2	1	N-Term(iTRAQ8plex); C9(Methylthio); C12(Methylthio)	0.684	0.619	0.982	5.18	58	0	2478.1406	3	826.7183838	-3.25856524	37.0298
														NMVHPNVICDGCNGPVVGTR	2	2	1	N-Term(iTRAQ8plex); M2(Oxidation); C9(Methylthio); C12(Methylthio)	2.01	0.889	1.436	4.78	53	0	2494.13852	3	826.7183838	-3.25856524	37.0298
														CSVCPDYDLCSVCEGK	14	2	1	N-Term(iTRAQ8plex); C1(Methylthio); C4(Methylthio); C10(Methylthio); [illegible]	1.265	0.648	1.784	4.64	56	0	2513.03208	3	838.3508301	3.076628594	54.65871167
														EAALYPHLPPEADPR	4	2	1	N-Term(iTRAQ8plex)	0.91	0.794	1.234	4.57	39	0	1980.043	3	660.6858521	-3.170839041	28.33273167
														LTPVSPESSSTEEK	2	2	1	N-Term(iTRAQ8plex); K14(iTRAQ8plex)	0.483	0.121	0.339	3.97	43	0	2099.11484	3	700.3764648	-8.57505192	16.01713
														VAALFPALRPGGFQAHYR	3	1	1	N-Term(iTRAQ8plex)	0.585	-	0.872	3.61	-	0	2275.27236	4	569.5735474	-1.86866722	43.30961167
														AYLLGK	1	1	1	N-Term(iTRAQ8plex); K6(iTRAQ8plex)	0.533	0.242	0.771	2.88	-	0	1272.81424	3	424.9429321	0.519979033	27.14599
4505621	PEBP1	phosphatidylethanolamine-binding protein 1	NP_002558.1	50.80	7	7	29	187	21.0	7.53	0.854	2.140	2.043																
														APVAGTCYQAEWDDYVPK	6	1	1	N-Term(iTRAQ8plex); C7(Methylthio); K18(iTRAQ8plex)	0.931	1.882	2.041	6.86	64	0	2667.31963	2	1334.163452	5.719365882	39.213715
														GNDISSGTVLSDYVGSGPPK	2	1	1	N-Term(iTRAQ8plex); K20(iTRAQ8plex)	1.344	2.699	3.129	4.64	47	0	2558.34409	3	853.4528809	-4.609220442	26.617975
														YVWLVYEQDRPLK	7	1	1	N-Term(iTRAQ8plex); K13(iTRAQ8plex)	0.581	2.089	2.869	3.96	36	0	2317.30117	4	580.0807495	-6.612491003	40.955445
														YREWHHFLVVNMK	3	1	1	N-Term(iTRAQ8plex); K13(iTRAQ8plex)	1.379	2.201	2.269	3.91	25	1	2367.28781	3	789.7674561	-5.346365515	37.41475
														LYTLVLTDPDAPSR	6	1	1	N-Term(iTRAQ8plex)	0.911	2.105	2.046	3.4	52	0	1865.02764	2	933.0174561	-2.458843257	43.29229333
														LYEQLSGK	2	1	1	N-Term(iTRAQ8plex); K8(iTRAQ8plex)	0.707	2.632	2.007	3.28	35	0	1545.89849	2	773.4528809	-7.236051628	23.48362167
														CDEPILSNR	3	1	1	N-Term(iTRAQ8plex); C1(Methylthio)	0.725	1.369	0.927	2.99	20	0	1396.67502	3	466.2298584	-8.327173111	26.60484167
4505753	PGAM1	phosphoglycerate mutase 1 isoform 1	NP_002620.1	58.66	11	11	103	254	28.8	7.18	0.758	2.393	2.900																
														FSGWYDADLSPAGHEEAKR	2	1	1	N-Term(iTRAQ8plex); K18(iTRAQ8plex)	-	3.97	4.138	6.08	41	1	2744.37392	4	686.848938	-5.465445781	32.07671
														YADLTEDQLPSCESLKDTIAR	12	2	1	N-Term(iTRAQ8plex); C12(Methylthio); K16(iTRAQ8plex)	0.722	3.247	3.742	5.44	27	1	3022.52799	3	1008.180847	-1.400829408	50.42589667
														FSGWYDADLSPAGHEEAK	4	1	1	N-Term(iTRAQ8plex); K18(iTRAQ8plex)	1.462	4.043	4.965	5.28	41	0	2588.26938	3	863.4279785	-7.1163393	33.290385
														SYDVPPPPMEPDHPFYSNISK	10	3	1	N-Term(iTRAQ8plex); K21(iTRAQ8plex)	0.65	2.929	3.6	5.11	35	0	3025.50418	3	1009.172913	-6.097085281	33.25415
														ALPFWNEEIVPQIK	23	4	1	N-Term(iTRAQ8plex); K14(iTRAQ8plex)	0.614	2.132	2.037	5.03	51	0	2292.32022	3	764.776123	-3.252351106	46.7071
														HLEGLSEEAIMELNLPTGIPIVYELDK	5	2	1	N-Term(iTRAQ8plex); K27(iTRAQ8plex)	-	-	-	4.97	38	0	3631.97597	4	908.7494507	0.149107367	62.86321
														YADLTEDQLPSCESLK	6	2	1	N-Term(iTRAQ8plex); C12(Methylthio); K16(iTRAQ8plex)	0.52	1.869	2.281	4.91	52	0	2466.22428	3	822.7462769	-4.462280132	43.70928667
														VLIAAHGNSLR	11	3	1	N-Term(iTRAQ8plex)	0.839	0.946	1.287	4.68	51	0	1454.86621	3	485.6269226	-5.716489703	21.29481167
														DAGYEFDICFTSVQK	22	1	1	N-Term(iTRAQ8plex); C9(Methylthio); K15(iTRAQ8plex)	0.83	2.208	2.531	4.63	71	0	2377.15262	2	1189.080078	-5.718046204	41.438575
														HYGGLTGLNK	4	4	1	N-Term(iTRAQ8plex); K10(iTRAQ8plex)	0.823	1.863	2.078	4.58	37	0	1667.97246	2	834.4898682	2.121382823	19.58371167
														HGESAWNLENR	3	1	1	N-Term(iTRAQ8plex)	1.443	2.645	2.597	4.39	56	0	1616.79947	3	539.6046753	-5.46058509	15.92057333
														SYDVPPPPMEPDHPFYSNISK	1	3	1	N-Term(iTRAQ8plex); M9(Oxidation); K21(iTRAQ8plex)	-	-	-	2.72	-	0	3041.51723	3	1009.172913	-6.097085281	33.25415
														IVLIR	3	3	1	N-Term(iTRAQ8plex)	0.964	2.689	3.093	-	19	0	917.63823	2	459.3227539	-7.323264519	29.26145333
4505763	PGK1	phosphoglycerate kinase 1	NP_000282.1	51.32	13	13	53	417	44.6	8.10	0.875	1.880	2.350																
														VLNNMEIGTSLFDEEGAK	6	1	1	N-Term(iTRAQ8plex); K18(iTRAQ8plex)	1.058	3.855	5.695	6.61	94	0	2575.35576	2	1288.181519	0.892765734	43.98564
														VLNNMEIGTSLFDEEGAK	5	1	1	N-Term(iTRAQ8plex); M5(Oxidation); K18(iTRAQ8plex)	-	-	-	5.85	74	0	2591.33402	2	1288.181519	0.892765734	43.98564
														ACANPAAGSVILLENLR	2	1	1	N-Term(iTRAQ8plex); C2(Methylthio)	1.49	5.745	11.706	5.48	92	0	2062.10283	2	1031.555054	-3.060709694	53.85866333
														GCITIIGGGDTATCCAK	6	1	1	N-Term(iTRAQ8plex); C2(Methylthio); C14(Methylthio); C15(Methylthio); [illegible]	0.483	1.908	2.457	5.15	60	0	2330.08097	3	777.3651733	-6.702569864	37.001515
														QIVWNGPVGVFEWEAFAR	10	1	1	N-Term(iTRAQ8plex)	0.868	2.101	2.792	5.1	76	0	2409.26493	3	803.7598267	-0.358851997	63.936025
														ITLPVDFVTADKFDENAK	1	1	1	N-Term(iTRAQ8plex); K12(iTRAQ8plex); K18(iTRAQ8plex)	-	-	-	4.84	-	1	2935.64272	4	734.6661377	-3.989040855	42.03202167
														VSHVSTGGGASLELLEGK	2	2	1	N-Term(iTRAQ8plex); K18(iTRAQ8plex)	1.21	1.254	1.298	4.81	34	0	2349.30997	3	783.7748413	-5.742377288	28.38307333
														LGDVYVNDAFGTAHR	5	2	1	N-Term(iTRAQ8plex)	0.784	1.144	1.602	4.7	64	0	1938.99236	2	969.9998169	-2.689093084	29.80635667
														SVVLMSHLGRPDGVPMPDKYSLEPVAVELK	1	1	1	N-Term(iTRAQ8plex); K19(iTRAQ8plex); K30(iTRAQ8plex)	-	-	-	4.52	-	1	4176.31666	5	836.0691528	-3.894256872	44.93135667
														TGQATVASGIPAGWMGLDCGPESSKK	1	1	1	N-Term(iTRAQ8plex); M15(Oxidation); C19(Methylthio); K25(iTRAQ8plex); [illegible]	-	-	-	4.02	-	1	3522.8102	4	881.4580078	-1.616871098	34.11965167
														ALESPERPFLAILGGAK	3	1	1	N-Term(iTRAQ8plex); K17(iTRAQ8plex)	-	-	-	3.99	30	0	2377.3937	4	595.1040649	-5.043931383	43.70254167
														AHSSMVGVNLPQK	2	1	1	N-Term(iTRAQ8plex); K13(iTRAQ8plex)	1.192	1.768	1.449	3.95	37	0	1976.10605	3	659.3735352	-7.453588452	19.90361
														AHSSMVGVNLPQK	1	1	1	N-Term(iTRAQ8plex); M5(Oxidation); K13(iTRAQ8plex)	1.214	1.576	2.046	3.64	-	0	1992.09665	3	659.3735352	-7.453588452	19.90361
														SVVLMSHLGRPDGVPMPDK	2	1	1	N-Term(iTRAQ8plex); K19(iTRAQ8plex)	1.162	4.042	5.852	3.16	-	0	2643.43851	3	881.817688	-7.059209087	32.83633833
														ELNYFAK	4	1	1	N-Term(iTRAQ8plex); K7(iTRAQ8plex)	0.828	3.169	5.497	3.08	31	0	1492.8488	2	746.9280396	-8.834664579	23.453615
														LGDVYVNDAFGTAHR	2	2	1	N-Term(iTRAQ8plex); Y5(iTRAQ8plex)	0.541	1.564	1.741	-	35	0	2243.18984	2	969.9998169	-2.689093084	29.80635667
4505775	SLC25A3	phosphate carrier protein, mitochondrial isoform b precursor	NP_002626.1	29.64	11	11	47	361	39.9	9.36	0.512	0.697	0.673																
														GWAPTFLGYSMQGLCK	14	2	1	N-Term(iTRAQ8plex); C15(Methylthio); K16(iTRAQ8plex)	0.48	0.729	0.69	5.93	56	0	2413.25479	3	805.0813599	-1.328493672	50.154965
														GIFNGFSVTLK	2	2	1	N-Term(iTRAQ8plex); K11(iTRAQ8plex)	0.427	0.586	0.64	3.9	51	0	1791.05425	2	896.0307617	-4.614311663	67.3691
														IQTQPGYANTLR	9	2	1	N-Term(iTRAQ8plex)	0.527	0.705	0.727	3.68	53	0	1665.91789	2	833.4633789	-1.866901453	22.12664667
														LPRPPPPEMPESLK	3	2	1	N-Term(iTRAQ8plex); K14(iTRAQ8plex)	0.39	0.407	0.564	3.43	-	0	2196.26235	4	549.8210449	-2.181657624	27.46470333
														FGFYEVFK	2	2	1	N-Term(iTRAQ8plex); K8(iTRAQ8plex)	0.445	0.431	0.557	3.3	31	0	1644.9196	2	822.9634399	-3.038468979	68.83552167
														TVEALYK	4	2	1	N-Term(iTRAQ8plex); K7(iTRAQ8plex)	0.475	0.542	0.561	3.23	31	0	1431.86772	2	716.4375	0.685873411	30.41657333
														MYKEEGLK	1	2	1	N-Term(iTRAQ8plex); M1(Oxidation); K3(iTRAQ8plex); K8(iTRAQ8plex)	0.605	0.83	0.656	3.17	-	1	1926.11928	4	482.2852783	3.08760797	17.927745
														LPRPPPPEMPESLK	1	2	1	N-Term(iTRAQ8plex); M9(Oxidation); K14(iTRAQ8plex)	0.745	0.642	0.62	3.09	-	0	2212.25534	4	549.8210449	-2.181657624	27.46470333
														QIPYTMMK	2	2	1	N-Term(iTRAQ8plex); K8(iTRAQ8plex)	0.489	0.751	0.76	3.01	26	0	1619.91318	3	540.6425781	1.376508955	35.99723667
														GSSASLVLK	2	2	1	N-Term(iTRAQ8plex); K9(iTRAQ8plex)	0.679	0.583	0.661	2.91	29	0	1469.9157	2	735.4614868	0.629772724	29.38606833
														GVAPLWMR	2	2	1	N-Term(iTRAQ8plex)	0.487	0.616	0.601	-	34	0	1233.70511	3	411.9065552	-2.333639236	24.25409667
														FACFER	5	2	1	N-Term(iTRAQ8plex); C3(Methylthio)	0.513	0.82	0.731	-	30	0	1122.53362	2	561.7704468	-3.719590442	47.24497333
4505813	DYNLL1	dynein light chain 1, cytoplasmic	NP_003737.1	37.08	2	2	20	89	10.4	7.40	0.984	1.389	1.356																
														NADMSEEMQQDSVECATQALEK	2	1	1	N-Term(iTRAQ8plex); M4(Oxidation); M8(Oxidation); C15(Methylthio); [illegible]	-	-	-	4.83	45	0	3143.40371	4	786.6063843	-1.948547242	31.08935

														YNPTWHCIVGR	17	2	1	N-Term(iTRAQ8plex); C7(Methylthio)	1.04	1.378	1.372	3.9	42	0	1695.83457	3	565.949707	-3.275352652	31.97643
														YNPTWHCIVGR	1	2	1	N-Term(iTRAQ8plex); Y1(iTRAQ8plex); C7(Methylthio)	0.424	2.136	1.356	-	26	0	2000.03579	3	565.949707	-3.275352652	31.97643
4505823	PIR	pirin	NP_003653.1	4.14	1	1	6	290	32.1	6.92	1.077	8.897	10.715																
														NLDPFLLFDEFK	6	1	1	N-Term(iTRAQ8plex); K12(iTRAQ8plex)	1.077	8.897	10.715	3.88	41	0	2106.15305	2	1053.580933	-8.837283704	54.77771333
4505891	PLOD3	procollagen-lysine,2-oxoglutarate 5-dioxygenase 3 precursor	NP_001075.1	8.40	5	5	9	738	84.7	6.05	0.740	0.427	0.749																
														LWSNFWGALSPDEYYAR	2	1	1	N-Term(iTRAQ8plex)	0.822	0.246	0.582	5.15	78	0	2379.19267	2	1190.099976	9.03384249	60.98103167
														QVGYEDQWLQLLR	2	1	1	N-Term(iTRAQ8plex)	0.689	0.494	0.824	3.84	32	0	1952.05718	2	976.5322266	1.447296234	48.59476667
														LVGPEEALSPGEAR	2	1	1	N-Term(iTRAQ8plex)	0.74	0.37	0.621	3.63	54	0	1728.94182	2	864.9745483	-0.931922806	29.08117667
														AVMNFVVR	1	1	1	N-Term(iTRAQ8plex)	0.653	0.427	0.749	3.22	-	0	1239.72759	2	620.3674316	7.288182781	32.35499333
														KGWALLHPGR	2	1	1	N-Term(iTRAQ8plex); K1(iTRAQ8plex)	0.779	0.477	0.759	2.81	29	1	1743.06081	4	436.5206604	-1.743816634	22.81033333
4505893	PLP2	proteolipid protein 2	NP_002659.1	18.42	2	2	4	152	16.7	7.24	0.884	0.577	0.530																
														HTAAPTDPADGPV	2	1	1	N-Term(iTRAQ8plex)	0.489	0.441	0.405	3.76	59	0	1552.7896	2	776.8984375	-0.858403483	16.40564333
														LSAPGCWAACTNFSR	2	1	1	N-Term(iTRAQ8plex); C6(Methylthio); C10(Methylthio)	1.598	0.754	0.693	3.39	39	0	1979.89726	2	990.4522705	3.556293426	51.90192333
4505939	POLR2A	DNA-directed RNA polymerase II subunit RPB1	NP_000928.1	3.35	4	4	5	1970	217.1	7.37	0.887	0.961	0.997																
														HMCDGDIVIFNR	2	1	1	N-Term(iTRAQ8plex); C3(Methylthio)	0.984	1.043	0.997	3.7	31	0	1769.83994	3	590.6181641	-2.250690818	34.59939
														AHNNELEPTPGNTLR	1	1	1	N-Term(iTRAQ8plex)	0.817	0.665	0.802	3.16	-	0	1967.01798	3	656.3441772	-3.494025878	16.69741
														YARPEWMIVTVLPVPPLSVRPAVVMQGSAR	1	1	1	N-Term(iTRAQ8plex)	-	-	-	-	33	0	3624.02505	4	906.7617188	0.693654147	59.01007833
														LTHVYDLCK	1	1	1	N-Term(iTRAQ8plex); C8(Methylthio); K9(iTRAQ8plex)	0.887	0.961	1.04	-	29	0	1745.94229	3	582.6522827	-6.625308008	37.17502
4505991	PPIC	peptidyl-prolyl cis-trans isomerase C precursor	NP_000934.1	6.13	1	1	2	212	22.7	8.40	0.842	0.344	0.683																
														TVENFVALATGEK	2	1	1	N-Term(iTRAQ8plex); K13(iTRAQ8plex)	0.842	0.344	0.683	4.06	37	0	1987.13211	3	663.0488892	0.026636613	37.117845
4506003	PPP1CA	serine/threonine-protein phosphatase PP1-alpha catalytic subunit isoform 1	NP_002699.1	34.85	2	9	59	330	37.5	6.33	0.972	1.012	1.075																
														NVQLTENEIR	2	2	1	N-Term(iTRAQ8plex)	0.992	1.159	1.304	3.37	33	0	1519.82927	2	760.4182739	-5.875704993	16.65892667
														LNLDSIIGR	2	3	1	N-Term(iTRAQ8plex)	0.953	0.884	0.887	2.74	31	0	1304.7824	2	652.8948364	-1.214814395	39.94918167
														AHQVVEDGYEFFAK	16	8	3	N-Term(iTRAQ8plex); K14(iTRAQ8plex)	-	-	-	6.73	82	0	2248.17412	2	1124.592163	-3.928404183	37.17953833
														IFCCHGGLSPDLQSMEQIR	15	8	3	N-Term(iTRAQ8plex); C3(Methylthio); C4(Methylthio)	-	-	-	6.06	56	0	2530.16782	3	844.0611572	0.073168904	51.22169167
														EIFLSQPILLELEAPLK	4	7	3	N-Term(iTRAQ8plex); K17(iTRAQ8plex)	-	-	-	5.49	46	0	2561.54331	3	854.5192871	0.701443586	59.77000833
														TFTDCFNCLPIAAIVDEK	6	8	3	N-Term(iTRAQ8plex); C5(Methylthio); C8(Methylthio); K18(iTRAQ8plex)	-	-	-	5.38	60	0	2700.35978	3	900.7914429	8.573695806	64.00521167
														IFCCHGGLSPDLQSMEQIR	2	8	3	N-Term(iTRAQ8plex); C3(Methylthio); C4(Methylthio); M15(Oxidation)	-	-	-	4.52	25	0	2546.163	3	844.0611572	0.073168904	51.22169167
														IKYPENFFLLR	5	8	3	N-Term(iTRAQ8plex); K2(iTRAQ8plex)	-	-	-	4.1	46	1	2048.19716	4	512.8047485	-8.864888445	43.35610167
														IYGFYDECK	6	8	3	N-Term(iTRAQ8plex); C8(Methylthio); K9(iTRAQ8plex)	-	-	-	4.08	43	0	1791.87297	2	896.4437256	-5.813864271	41.88330333
														HDLDLICR	1	8	3	N-Term(iTRAQ8plex); C7(Methylthio)	-	-	-	-	21	0	1334.68022	2	667.84375	-4.517126945	25.74778667
4506005	PPP1CB	serine/threonine-protein phosphatase PP1-beta catalytic subunit isoform 1	NP_002700.1	40.67	2	9	59	327	37.2	6.19	-	-	-																
														IMRPTDVPDTGLLCDLLWSDPDKDVQGWGENDR	2	1	1	N-Term(iTRAQ8plex); M2(Oxidation); C14(Methylthio); K23(iTRAQ8plex)	-	-	-	4.65	22	1	4427.17359	5	886.2405396	3.793378227	60.45222167
														IMRPTDVPDTGLLCDLLWSDPDKDVQGWGENDR	1	1	1	N-Term(iTRAQ8plex); C14(Methylthio); K23(iTRAQ8plex)	-	-	-	4.08	-	1	4411.15858	5	886.2405396	3.793378227	60.45222167
														FLNRHDLDLICR	1	1	1	N-Term(iTRAQ8plex); C11(Methylthio)	-	-	-	3.6	-	1	1864.99538	4	467.004303	6.750544363	42.36679333
														AHQVVEDGYEFFAK	16	8	3	N-Term(iTRAQ8plex); K14(iTRAQ8plex)	-	-	-	6.73	82	0	2248.17412	2	1124.592163	-3.928404183	37.17953833
														IFCCHGGLSPDLQSMEQIR	15	8	3	N-Term(iTRAQ8plex); C3(Methylthio); C4(Methylthio)	-	-	-	6.06	56	0	2530.16782	3	844.0611572	0.073168904	51.22169167
														EIFLSQPILLELEAPLK	4	7	3	N-Term(iTRAQ8plex); K17(iTRAQ8plex)	-	-	-	5.49	46	0	2561.54331	3	854.5192871	0.701443586	59.77000833
														TFTDCFNCLPIAAIVDEK	6	8	3	N-Term(iTRAQ8plex); C5(Methylthio); C8(Methylthio); K18(iTRAQ8plex)	-	-	-	5.38	60	0	2700.35978	3	900.7914429	8.573695806	64.00521167
														IFCCHGGLSPDLQSMEQIR	2	8	3	N-Term(iTRAQ8plex); C3(Methylthio); C4(Methylthio); M15(Oxidation)	-	-	-	4.52	25	0	2546.163	3	844.0611572	0.073168904	51.22169167
														IKYPENFFLLR	5	8	3	N-Term(iTRAQ8plex); K2(iTRAQ8plex)	-	-	-	4.1	46	1	2048.19716	4	512.8047485	-8.864888445	43.35610167
														IYGFYDECK	6	8	3	N-Term(iTRAQ8plex); C8(Methylthio); K9(iTRAQ8plex)	-	-	-	4.08	43	0	1791.87297	2	896.4437256	-5.813864271	41.88330333
														HDLDLICR	1	8	3	N-Term(iTRAQ8plex); C7(Methylthio)	-	-	-	-	21	0	1334.68022	2	667.84375	-4.517126945	25.74778667
4506007	PPP1CC	serine/threonine-protein phosphatase PP1-gamma catalytic subunit isoform 1	NP_002701.1	39.94	1	8	56	323	37.0	6.54	-	-	-																
														IMRPTDVPDQGLLCDLLWSDPDKDVLGWGENDR	1	4	1	N-Term(iTRAQ8plex); M2(Oxidation); C14(Methylthio); K23(iTRAQ8plex)	-	-	-	-	21	1	4439.19546	4	1110.554321	0.510898039	62.86764833
														AHQVVEDGYEFFAK	16	8	3	N-Term(iTRAQ8plex); K14(iTRAQ8plex)	-	-	-	6.73	82	0	2248.17412	2	1124.592163	-3.928404183	37.17953833
														IFCCHGGLSPDLQSMEQIR	15	8	3	N-Term(iTRAQ8plex); C3(Methylthio); C4(Methylthio)	-	-	-	6.06	56	0	2530.16782	3	844.0611572	0.073168904	51.22169167
														EIFLSQPILLELEAPLK	4	7	3	N-Term(iTRAQ8plex); K17(iTRAQ8plex)	-	-	-	5.49	46	0	2561.54331	3	854.5192871	0.701443586	59.77000833
														TFTDCFNCLPIAAIVDEK	6	8	3	N-Term(iTRAQ8plex); C5(Methylthio); C8(Methylthio); K18(iTRAQ8plex)	-	-	-	5.38	60	0	2700.35978	3	900.7914429	8.573695806	64.00521167
														IFCCHGGLSPDLQSMEQIR	2	8	3	N-Term(iTRAQ8plex); C3(Methylthio); C4(Methylthio); M15(Oxidation)	-	-	-	4.52	25	0	2546.163	3	844.0611572	0.073168904	51.22169167
														IKYPENFFLLR	5	8	3	N-Term(iTRAQ8plex); K2(iTRAQ8plex)	-	-	-	4.1	46	1	2048.19716	4	512.8047485	-8.864888445	43.35610167
														IYGFYDECK	6	8	3	N-Term(iTRAQ8plex); C8(Methylthio); K9(iTRAQ8plex)	-	-	-	4.08	43	0	1791.87297	2	896.4437256	-5.813864271	41.88330333
														HDLDLICR	1	8	3	N-Term(iTRAQ8plex); C7(Methylthio)	-	-	-	-	21	0	1334.68022	2	667.84375	-4.517126945	25.74778667
4506017	PPP2CA	serine/threonine-protein phosphatase 2A catalytic subunit alpha isoform	NP_002706.1	31.72	5	6	26	309	35.6	5.54	0.938	2.344	2.272																
														LQEVPHEGPMCDLLWSDPDDR	6	2	1	N-Term(iTRAQ8plex); C11(Methylthio)	0.938	2.788	2.36	7.1	70	0	2802.27005	3	934.7615356	-4.970476128	73.66212
														LQEVPHEGPMCDLLWSDPDDR	6	2	1	N-Term(iTRAQ8plex); M10(Oxidation); C11(Methylthio)	0.888	2.567	2.86	5.12	32	0	2818.28189	3	934.7615356	-4.970476128	73.66212
														ELDQWIEQLNECK	4	1	1	N-Term(iTRAQ8plex); C12(Methylthio); K13(iTRAQ8plex)	1.121	2.372	2.663	4.97	52	0	2302.14566	3	768.0534058	-9.186327857	77.93595833
														NVVTIFSAPNYCYR	2	2	1	N-Term(iTRAQ8plex); C12(Methylthio)	0.668	1.516	1.756	3.64	45	0	1996.99016	2	998.9987183	-1.253084073	48.31290667
														YSFLQFDPAPR	5	2	1	N-Term(iTRAQ8plex)	0.977	1.877	2.002	3.52	54	0	1644.8737	2	822.9404907	2.999333808	39.24414
														GAGYTFGQDISETFNHANGLTLVSR	1	2	1	N-Term(iTRAQ8plex)	0.846	3.072	4.737	2.77	-	0	2959.49338	3	987.1693115	2.753856577	42.40912667
														QITQVYGFYDECLR	2	5	2	N-Term(iTRAQ8plex); C12(Methylthio)	-	-	-	4.82	52	0	2085.02764	2	1043.017456	9.07574991	48.04100833
4506025	PPP3R1	calcineurin subunit B type 1	NP_000936.1	17.06	1	1	2	170	19.3	4.81	-	-	-																
														KLDLDNSGSLSVEEFMSLPELQQNPLVQR	2	1	1	N-Term(iTRAQ8plex); K1(iTRAQ8plex)	-	-	-	3.42	23	1	3895.0917	4	974.5283813	4.732676935	57.38066167
4506127	PRPS1	ribose-phosphate pyrophosphokinase 1 isoform 1	NP_002755.1	18.24	2	4	12	318	34.8	6.98	0.834	1.334	2.400																
														FSNQETCVEIGESVR	2	1	1	N-Term(iTRAQ8plex); C7(Methylthio)	0.812	1.747	3.931	5.89	54	0	2047.98198	2	1024.494629	4.345200584	36.16284
														LNVDFALIHK	5	1	1	N-Term(iTRAQ8plex); K10(iTRAQ8plex)	0.857	1.3	2.092	4.34	31	0	1778.06504	3	593.359375	-8.390897962	34.59401167
														VAILVDDMADTCGTICHAADK	2	4	2	N-Term(iTRAQ8plex); C12(Methylthio); C16(Methylthio); K21(iTRAQ8plex)	-	-	-	4.55	49	0	2862.36269	4	716.3461304	-5.670733367	72.50452833
														VTAVIPCFPYAR	3	4	2	N-Term(iTRAQ8plex); C7(Methylthio)	-	-	-	3.28	32	0	1686.89481	3	562.9697876	-3.87265228	53.19228
4506129	PRPS2	ribose-phosphate pyrophosphokinase 2 isoform 2	NP_002756.1	15.09	1	3	7	318	34.7	6.61	1.318	2.482	1.269																
														FSNQETSVEIGESVR	2	2	1	N-Term(iTRAQ8plex)	1.318	2.482	1.269	3.43	34	0	1986.00664	2	993.506958	-0.78645476	28.80694
														VAILVDDMADTCGTICHAADK	2	4	2	N-Term(iTRAQ8plex); C12(Methylthio); C16(Methylthio); K21(iTRAQ8plex)	-	-	-	4.55	49	0	2862.36269	4	716.3461304	-5.670733367	72.50452833
														VTAVIPCFPYAR	3	4	2	N-Term(iTRAQ8plex); C7(Methylthio)	-	-	-	3.28	32	0	1686.89481	3	562.9697876	-3.87265228	53.19228
4506141	HTRA1	serine protease HTRA1 precursor	NP_002766.1	2.50	1	1	2	480	51.3	7.83	0.156	0.167	0.215																
														IAPAVVHIELFR	2	1	1	N-Term(iTRAQ8plex)	0.156	0.167	0.215	5.03	42	0	1669.00705	3	557.0072021	-1.952121581	37.88744333
4506145	PRSS1	trypsin-1 preproprotein	NP_002760.1	8.10	1	1	1	247	26.5	6.51	1.060	0.985	0.855																
														LGEHNIEVLEGNEQFINAAK	1	4	1	N-Term(iTRAQ8plex); K20(iTRAQ8plex)	1.06	0.985	0.855	2.76	-	0	2833.52799	3	945.1808472	-0.890425683	38.96585833
4506179	PSMA1	proteasome subunit alpha type-1 isoform 2	NP_002777.1	23.95	4	4	13	263	29.5	6.61	0.814	1.621	1.622																
														ILHVDNHIGISIAGLTADAR	3	3	1	N-Term(iTRAQ8plex)	0.95	1.329	1.562	5.33	36	0	2390.3395	4	598.340332	-2.64525395	40.888705
														DLEFTIYDDDDVSPFLEGLEERPQR	8	2	1	N-Term(iTRAQ8plex)	0.452	2.398	2.263	4.4	39	0	3302.60483	3	1101.539795	1.235453781	54.43859333
														FVFDRPLPVSR	1	3	1	N-Term(iTRAQ8plex)	0.854	1.091	1.182	2.77	-	0	1636.94072	2	818.973999	-4.270083231	29.66665667
														LLCNFMR	1	3	1	N-Term(iTRAQ8plex); C3(Methylthio)	0.776	2.866	2.834	-	21	0	1246.63359	2	623.8204346	-6.120306256	71.69616833
4506181	PSMA2	proteasome subunit alpha type-2	NP_002778.1	38.46	5	5	26	234	25.9	7.43	0.935	1.332	1.439																
														YNEDLELEDAIHTAILTLK	9	1	1	N-Term(iTRAQ8plex); K19(iTRAQ8plex)	-	2.825	1.786	6.09	33	0	2809.53261	4	703.1386108	-4.199211131	64.16890833
														GYSFSLTTFSPSGK	5	1	1	N-Term(iTRAQ8plex); K14(iTRAQ8plex)	0.9	1.523	1.559	5.07	27	0	2087.10971	3	696.3747559	-8.258776123	59.02491
														HIGLVYSGMGPDYR	9	1	1	N-Term(iTRAQ8plex)	0.972	1.072	1.319	4.85	54	0	1868.9531	3	623.657959	-2.015879547	27.03638333
														LVQIEYALAAVAGGAPSVGIK	2	1	1	N-Term(iTRAQ8plex); K21(iTRAQ8plex)	-	-	-	3.43	38	0	2635.55381	3	879.1894531	-4.012505825	63.69769167
														KLAQQYYLVYQEPIPTAQLVQR	1	1	1	N-Term(iTRAQ8plex); K1(iTRAQ8plex)	-	-	-	3.28	-	1	3257.84829	4	815.2175293	-0.737908057	38.62674333
4506189	PSMA7	proteasome subunit alpha type-7	NP_002783.1	32.26	6	6	13	248	27.9	8.46	0.883	1.651	1.823																
														KICALDDNVCMAFAGLTADAR	2	1	1	K1(iTRAQ8plex); N-Term(iTRAQ8plex); C3(Methylthio); C10(Methylthio)	0.676	3.555	3.577	4.77	70	1	2898.431	3	966.8151855	1.535344681	65.015775
														LTVEDPVTVEYITR	4	6	1	N-Term(iTRAQ8plex)	0.968	2.036	2.419	3.9	52	0	1939.0634	2	970.0353394	-2.887584313	35.89577833
														GRDIVVLGVEK	3	1	1	N-Term(iTRAQ8plex); K11(iTRAQ8plex)	0.778	1.016	1.181	3.8	30	1	1793.10532	3	598.373291	-2.911989088	21.69444333
														NYTDEAIETDDLTIK	2	1	1	N-Term(iTRAQ8plex); K15(iTRAQ8plex)	0.887	1.052	1.279	3.31	28	0	2349.21567	3	783.7434082	-5.333669776	30.525565
														ALLEVVQSGGK	1	6	1	N-Term(iTRAQ8plex); K11(iTRAQ8plex)	1.288	0.832	0.903	2.75	-	0	1709.0286	3	570.3477173	-7.713559938	30.54584167
														NIELAVMR	1	1	1	N-Term(iTRAQ8plex)	0.863	1.802	2.035	-	25	0	1249.71575	2	625.3615112	-6.629874906	35.78811667
4506193	PSMB1	proteasome subunit beta type-1	NP_002784.1	39.00	7	7	32	241	26.5	8.13	0.892	2.224	2.015																
														NMQNVEHVPLSLDR	2	1	1	N-Term(iTRAQ8plex); M2(Oxidation)	0.915	1.359	1.207	4.91	29	0	1972.01914	3	652.677124	-5.461343473	27.33891333
														RFFPYYVYNIIGGLDEEGK	8	1	1	N-Term(iTRAQ8plex); K19(iTRAQ8plex)	1.14	2.903	2.242	4.77	46	1	2888.5519	3	963.5246582	5.220615946	61.999915
														NMQNVEHVPLSLDR	8	1	1	N-Term(iTRAQ8plex)	0.799	2.678	2.299	4.62	49	0	1956.01682	3	652.677124	-5.461343473	27.33891333
														GAVYSFDPVGSYQR	5	1	1	N-Term(iTRAQ8plex)	0.922	2.174	2.019	3.88	50	0	1849.94158	2	925.4692383	-4.022504999	29.36013833
														AMTTGAIAAMLSTILYSR	2	1	1	N-Term(iTRAQ8plex)	-	-	-	3.62	68	0	2175.18711	3	725.7338867	2.360995169	70.28129667
														DVFISAAER	3	1	1	N-Term(iTRAQ8plex)	0.988	2.173	1.799	2.86	31	0	1311.71672	2	656.3619995	-3.300176394	26.65784167
														DVYTGDALR	2	1	1	N-Term(iTRAQ8plex)	0.857	2.281	1.822	2.84	21	0	1313.69304	2	657.3501587	-5.526814075	20.07442833
														EGIREETVSLR	2	1	1	N-Term(iTRAQ8plex)	0.803	1.483	1.487	2.72	39	1	1592.88213	2	796.9447021	-5.558241235	17.60167333
4506201	PSMB5	proteasome subunit beta type-5 isoform 1	NP_002788.1	8.37	2	2	6	263	28.5	6.92	0.759	1.892	2.074																
														VSSDNVADLHEK	4	2	1	N-Term(iTRAQ8plex); K12(iTRAQ8plex)	0.392	1.995	2.112	4.27	28	0	1922.02585	3	641.3468018	-9.421973458	16.82323667
														HGVIVAADSR	2	3	1	N-Term(iTRAQ8plex)	0.893	1.059	1.713	3.54	48	0	1328.75187	3	443.5888062	-5.242583774	13.58224833
4506209	PSMC2	26S protease regulatory subunit 7 isoform 1	NP_002794.1	11.55	4	4	11	433	48.6	5.95	1.083	1.198	2.073																
														EVVETPLLHPER	2	1	1	N-Term(iTRAQ8plex)	-	0.613	1.532	4.98	63	0	1722.96366	3	574.9927368	-3.235181867	23.72711333
														TMLELINQLDGFDPR	3	1	1	N-Term(iTRAQ8plex)	2.099	1.042	1.575	4	26	0	2066.09598	3	689.3701782	3.160607265	60.44426
														ALDEGDIALLK	2	2	1	N-Term(iTRAQ8plex); K11(iTRAQ8plex)	1.021	1.198	1.346	3.48	35	0	1766.03501	3	589.3498535	-9.634012381	52.92672333
														KIEFSLPDLEGR	4	1	1	N-Term(iTRAQ8plex); K1(iTRAQ8plex)	0.996	2.004	2.301	3.11	34	1	2012.14699	4	503.7922058	-8.303537399	37.25689667
4506221	PSMD12	26S proteasome non-ATPase regulatory subunit 12 isoform 1	NP_002807.1	14.47	5	5	14	456	52.9	7.65	0.693	1.351	1.866																
														MEVDYSATVDQR	4	2	1	N-Term(iTRAQ8plex); M1(Oxidation)	0.734	1.275	1.611	5.82	67	0	1733.84452	3	578.6196899	7.330688593	21.66536333
														LQEVIETLLSLEK	2	1	1	N-Term(iTRAQ8plex); K13(iTRAQ8plex)	0.624	1.638	2.496	4.92	38	0	2123.26664	3	708.427063	-5.536556247	86.07570667
														AIYDTPCIQAESEK	2	3	1	N-Term(iTRAQ8plex); C7(Methylthio); K14(iTRAQ8plex)	0.693	1.121	1.778	4.81	64	0	2222.13555	2	1111.571411	2.787114241	32.84938167

														WSTLVEDYGMELR	3	3	1	N-Term(iTRAQ8plex)	0.657	1.267	1.667	4.57	67	0	1902.95952	2	951.9833984	1.139779894	51.34889333
														EWDLLNENIMLLSK	3	3	1	N-Term(iTRAQ8plex); K14(iTRAQ8plex)	0.638	1.351	1.965	4.52	38	0	2326.27951	3	776.0980225	-6.115845968	77.523835
4506289	PTPN1	tyrosine-protein phosphatase non-receptor type 1 isoform 1	NP_002818.1	31.03	11	11	21	435	49.9	6.27	1.009	0.588	0.811																
														QLELENLTTQETR	2	2	1	N-Term(iTRAQ8plex)	0.925	0.276	0.626	5.06	61	0	1879.01628	2	940.0117798	4.694823772	31.04131667
														SYILTQGPLPNTCGHFWEMVWEQK	1	2	1	N-Term(iTRAQ8plex); C13(Methylthio); K24(iTRAQ8plex)	0.465	-	0.511	4.78	-	0	3518.76088	4	880.4456787	2.518688834	65.49624333
														FIMGDSSVQDQWK	2	2	1	N-Term(iTRAQ8plex); K13(iTRAQ8plex)	1.009	0.421	0.707	4.67	60	0	2149.11161	3	717.0420532	-4.30172263	54.53597833
														SGSWAAIYQDIR	4	1	1	N-Term(iTRAQ8plex)	0.935	0.482	0.658	4.61	45	0	1670.872	2	835.9396362	-5.030582285	32.08324833
														MGLIQTADQLR	1	2	1	N-Term(iTRAQ8plex)	0.757	0.588	0.768	4.07	-	0	1549.86662	2	775.4369507	-0.507497097	35.59867
														LTLISEDIK	3	2	1	N-Term(iTRAQ8plex); K9(iTRAQ8plex)	1.064	0.609	0.904	4.02	31	0	1640.00749	2	820.5073853	-0.961832305	63.41437667
														EMIFEDTNLK	4	2	1	N-Term(iTRAQ8plex); K10(iTRAQ8plex)	1.216	0.661	1.117	3.82	49	0	1847.98789	3	616.6674805	-8.36755687	49.74192833
														FSYLAVIEGAK	1	2	1	N-Term(iTRAQ8plex); K11(iTRAQ8plex)	0.922	1.047	1.129	3.33	-	0	1806.056	3	602.6901855	-3.41512631	38.22304833
														ELSHEDLEPPPEHIPPPPRPPKR	1	2	1	N-Term(iTRAQ8plex); K22(iTRAQ8plex)	-	-	-	3.17	-	1	3272.78608	5	655.3630371	-4.287558913	22.75760167
														ELSHEDLEPPPEHIPPPPRPPK	1	2	1	N-Term(iTRAQ8plex); K22(iTRAQ8plex)	-	-	-	3.01	-	0	3116.69141	5	624.144104	-2.431357315	24.33125
														HEASDFPCR	1	1	1	N-Term(iTRAQ8plex); C8(Methylthio)	1.347	0.742	0.947	-	34	0	1411.63034	3	471.2149658	-6.863049595	15.66928
4506357	MPZL1	myelin protein zero-like protein 1 isoform a precursor	NP_003944.1	8.55	2	2	5	269	29.1	8.72	1.108	0.651	0.827																
														NPPDIVVQPGHIR	3	2	1	N-Term(iTRAQ8plex)	1.088	0.795	0.874	5	47	0	1745.99503	3	582.6698608	-0.814452759	21.35544
														SESVVYADIR	2	2	1	N-Term(iTRAQ8plex)	1.433	0.488	0.394	4.19	38	0	1442.78374	2	721.8955078	3.089655698	26.85073
4506363	RAB13	ras-related protein Rab-13 isoform 1	NP_002861.1	14.29	2	2	6	203	22.8	9.19	0.765	0.669	0.882																
														SSMNVDEAFSSLAR	4	2	1	N-Term(iTRAQ8plex)	0.742	0.741	0.915	5.6	54	0	1817.90813	2	909.4577026	4.157170166	35.34173333
														GAMGIILVYDITDEK	2	1	1	N-Term(iTRAQ8plex); K15(iTRAQ8plex)	1	0.669	0.792	3.53	28	0	2246.24961	3	749.4213867	-2.975801856	46.88125167
														LLIIGDSGVGK	22	2	5	N-Term(iTRAQ8plex); K11(iTRAQ8plex)	-	-	-	4.71	52	0	1680.04277	3	560.6833496	-9.598441174	57.819735
4506365	RAB2A	ras-related protein Rab-2A isoform a	NP_002856.1	49.06	8	8	27	212	23.5	6.54	0.887	0.801	0.710																
														TASNVEEAFINTAK	2	2	1	N-Term(iTRAQ8plex); K14(iTRAQ8plex)	1.502	1.262	0.957	5.13	36	0	2103.14591	3	701.7201538	-3.966973564	48.59986833
														EHGLIFMETSAK	2	7	1	N-Term(iTRAQ8plex); K12(iTRAQ8plex)	1.203	1.188	1.467	4.92	36	0	1971.069	3	657.694519	-7.101436361	24.09183667
														LQIWDTAGQESFR	6	4	1	N-Term(iTRAQ8plex)	0.805	0.748	0.752	4.88	49	0	1854.95915	2	927.9832153	-3.265438985	32.77628333
														FQPVHDLTIGVEFGAR	2	2	1	N-Term(iTRAQ8plex)	1.009	0.772	0.71	4.48	32	0	2090.13535	3	697.3833008	0.793151387	39.64801
														GAAGALLVYDITR	6	6	1	N-Term(iTRAQ8plex)	0.866	0.628	0.479	4.4	80	0	1623.95049	2	812.4788818	8.181131125	40.88572333
														DTFNHLTTWLEDAR	2	2	1	N-Term(iTRAQ8plex)	1.223	1.104	1.406	3.87	-	0	2023.01566	3	675.0100708	-1.503768714	46.406775
														YIIIGDTGVGK	4	2	1	N-Term(iTRAQ8plex); K11(iTRAQ8plex)	0.878	0.948	0.688	3.85	48	0	1744.04276	3	582.019104	-2.157365846	43.22480833
														SCLLLQFTDKR	3	2	1	N-Term(iTRAQ8plex); C2(Methylthio); K10(iTRAQ8plex)	0.877	0.727	0.73	3.61	33	1	1978.10988	4	495.2829285	1.239408997	49.61116333
4506403	GPRC5A	retinoic acid-induced protein 3	NP_003970.1	3.64	1	1	2	357	40.2	8.15	1.477	1.662	1.613																
														TNVNVFSELSAPR	2	1	1	N-Term(iTRAQ8plex)	1.477	1.662	1.613	3.65	41	0	1737.93682	2	869.4720459	-3.985256003	37.08445167
4506439	RBBP7	histone-binding protein RBBP7 isoform 2	NP_002884.1	20.94	3	7	14	425	47.8	5.05	0.967	1.922	1.130																
														IGEEQSAEDAEDGPPELLFIHGGHTAK	1	2	1	N-Term(iTRAQ8plex); K27(iTRAQ8plex)	-	-	-	4.98	-	0	3455.74049	5	691.9539185	-3.916823297	33.24136167
														HPAKPDPSGECNPDLR	2	2	1	N-Term(iTRAQ8plex); K4(iTRAQ8plex); C11(Methylthio)	-	-	-	4.88	27	0	2387.19106	4	597.5532227	-6.120815062	17.59096167
														EMFEDTVEER	2	1	1	N-Term(iTRAQ8plex)	0.967	1.922	1.13	3.55	35	0	1588.74126	2	794.8742676	-3.419539664	27.43989
														TPSSDVLVFDYTK	2	5	2	N-Term(iTRAQ8plex); K13(iTRAQ8plex)	-	-	-	3.97	29	0	2080.13047	3	694.0483398	-5.675133816	27.72360667
														TVALWDLR	4	5	2	N-Term(iTRAQ8plex)	-	-	-	3.49	31	0	1277.74541	2	639.3763428	-5.120247805	29.29529
														LNVWDLSK	2	5	2	N-Term(iTRAQ8plex); K8(iTRAQ8plex)	-	-	-	3.05	29	0	1582.93888	3	528.3178101	-1.544200003	32.90782667
														LMIWDTR	1	5	2	N-Term(iTRAQ8plex)	-	-	-	-	25	0	1238.68205	2	619.8446655	-3.92099433	51.24503
4506455	RCN1	reticulocalbin-1 precursor	NP_002892.1	22.96	6	6	18	331	38.9	5.00	1.481	0.853	1.298																
														IDNDGDGFVTTEELK	2	1	1	N-Term(iTRAQ8plex); K15(iTRAQ8plex)	1.168	0.57	1.134	6.8	73	0	2261.1895	2	1131.098389	6.062925618	31.429535
														HWILPQDYDHAQAEAR	4	1	1	N-Term(iTRAQ8plex)	0.976	0.734	0.946	5.35	48	0	2254.12644	3	752.0469971	-1.895784988	23.26549167
														EEFTAFLHPEEFEHMK	5	1	1	N-Term(iTRAQ8plex); K16(iTRAQ8plex)	2.061	0.759	1.698	4.68	23	0	2629.31093	4	658.0831909	-4.11419889	42.51971833
														IVDRIDNDGDGFVTTEELK	2	1	1	N-Term(iTRAQ8plex); K19(iTRAQ8plex)	1.832	2.282	2.595	4.66	36	1	2744.45304	3	915.4891968	-1.207606005	35.52398667
														EEFTAFLHPEEFEHMK	1	1	1	N-Term(iTRAQ8plex); M15(Oxidation); K16(iTRAQ8plex)	0.853	-	1.568	3.92	-	0	2645.29873	4	658.0831909	-4.11419889	42.51971833
														AADLNGDLTATR	2	1	1	N-Term(iTRAQ8plex)	1.61	0.844	1.387	3.74	62	0	1521.82585	2	761.4165649	5.50126205	25.9001
														TFDQLTPDESKER	2	1	1	N-Term(iTRAQ8plex); K11(iTRAQ8plex)	1.195	0.876	0.916	3.15	-	1	2174.13901	3	725.3845215	-7.345481035	16.825515
4506457	RCN2	reticulocalbin-2 isoform a precursor	NP_002893.1	19.87	5	5	10	317	36.9	4.40	1.193	1.275	0.632																
														WDPTANEDPEWILVEK	4	2	1	N-Term(iTRAQ8plex); K16(iTRAQ8plex)	1.745	1.371	0.633	4.98	33	0	2550.35886	2	1275.679077	6.734907352	43.50619167
														EALLGVQEDVDEYVK	2	2	1	N-Term(iTRAQ8plex); K15(iTRAQ8plex)	0.951	0.593	0.482	4.94	75	0	2315.24631	3	772.4202881	-5.533014836	36.01254667
														VIDFDENTALDDAEEESFR	2	2	1	N-Term(iTRAQ8plex)	0.556	0.45	0.631	4.17	52	0	2519.16648	3	840.3936768	-2.510463925	47.25950333
														VIDFDENTALDDAEEESFRK	1	2	1	N-Term(iTRAQ8plex); K20(iTRAQ8plex)	-	-	-	3.12	-	1	2951.46743	4	738.6223145	-1.932383683	38.47713
														QLHDDYFYHDEL	1	2	1	N-Term(iTRAQ8plex)	0.907	1.363	1.117	2.84	-	0	1898.87748	3	633.6306763	-4.621541684	29.75661667
4506467	RDX	radixin isoform 2	NP_002897.1	22.30	4	13	57	583	68.5	6.37	0.922	1.355	2.123																
														FFPEDVSEELIQEITQR	2	3	1	N-Term(iTRAQ8plex)	-	-	-	5.25	68	0	2384.22434	3	795.4129639	-1.863477512	66.664285
														AFAAQEDLEK	4	4	1	N-Term(iTRAQ8plex); K10(iTRAQ8plex)	0.793	2.672	3.695	4.14	52	0	1729.94597	2	865.4766235	-7.01805137	32.38261667
														AQLENEK	1	5	1	N-Term(iTRAQ8plex); K7(iTRAQ8plex)	0.903	0.878	0.984	2.7	-	0	1439.8209	3	480.6118164	-7.317112343	13.48685667
														IAQDLEMYGVNYFEIK	1	3	1	N-Term(iTRAQ8plex); K16(iTRAQ8plex)	1.173	0.914	1.566	-	47	0	2541.35172	3	847.7887573	-0.099387008	60.04881667
														VTTMDAELEFAIQPNTTGK	4	7	3	N-Term(iTRAQ8plex); M4(Oxidation); K19(iTRAQ8plex)	-	-	-	5.01	62	0	2690.42168	2	1345.714478	1.818361101	34.020625
														APDFVFYAPR	7	9	3	N-Term(iTRAQ8plex)	-	-	-	3.69	38	0	1486.79473	2	743.906311	3.843240361	35.09177167
														KPDTIEVQQMK	2	9	3	N-Term(iTRAQ8plex); K1(iTRAQ8plex); K11(iTRAQ8plex)	-	-	-	3.47	34	0	2229.28287	3	743.7658081	-9.482974937	17.16221
														RKPDTIEVQQMK	1	9	3	N-Term(iTRAQ8plex); K2(iTRAQ8plex); K12(iTRAQ8plex)	-	-	-	3.34	-	1	2385.38759	4	597.102356	-7.351992573	15.829755
														QLFDQVVK	2	7	3	N-Term(iTRAQ8plex); K8(iTRAQ8plex)	-	-	-	3.26	37	0	1584.94194	2	792.9746094	-9.482473522	25.558705
														IGFPWSEIR	11	9	3	N-Term(iTRAQ8plex)	-	-	-	3.21	41	0	1408.79267	3	470.2690735	2.552475654	39.48728
														LFFLQVK	3	8	3	N-Term(iTRAQ8plex); K7(iTRAQ8plex)	-	-	-	3.06	28	0	1502.9406	2	751.973938	-9.921844845	69.32707
														KAPDFVFYAPR	7	9	3	N-Term(iTRAQ8plex); K1(iTRAQ8plex)	-	-	-	2.98	29	1	1919.08756	3	640.3673706	-6.46282841	28.32321167
														ILALCMGNHELYMR	10	7	2	N-Term(iTRAQ8plex); C5(Methylthio)	-	-	-	5.97	58	0	2013.99936	3	672.0046387	-2.730653483	59.78209667
														ILALCMGNHELYMR	2	7	2	N-Term(iTRAQ8plex); C5(Methylthio); M13(Oxidation)	-	-	-	3.61	26	0	2029.99253	3	672.0046387	-2.730653483	59.78209667
4506505	RGS10	regulator of G-protein signaling 10 isoform b	NP_002916.1	7.19	1	1	2	167	19.6	5.87	1.324	10.600	10.029																
														ASSQVNVEGQSR	2	2	1	N-Term(iTRAQ8plex)	1.324	10.6	10.029	3.56	48	0	1565.81438	2	783.4108276	-2.652068227	14.271935
4506583	RPA1	replication protein A 70 kDa DNA-binding subunit	NP_002936.1	8.12	3	3	6	616	68.1	7.21	2.246	1.380	2.076																
														GWFDAEGQALDGVSISDLK	2	1	1	N-Term(iTRAQ8plex); K19(iTRAQ8plex)	1.244	1.334	1.775	6.2	72	0	2616.37113	3	872.7952271	-2.107268956	41.04566667
														DKNEQAFEEVFQNANFR	2	1	1	N-Term(iTRAQ8plex); K2(iTRAQ8plex)	2.844	5.307	4.665	4.14	34	1	2694.35794	3	898.7908325	-5.688550565	37.53029833
														LFSLELVDESGEIR	2	1	1	N-Term(iTRAQ8plex)	2.246	1.38	2.076	3.83	58	0	1911.02872	3	637.6810913	-4.709831999	52.970175
4506585	RPA2	replication protein A 32 kDa subunit isoform 1	NP_002937.1	8.52	1	1	2	270	29.2	6.15	-	-	-																
														AQHIVPCTISQLLSATLVDEVFR	2	2	1	N-Term(iTRAQ8plex); C7(Methylthio)	-	-	-	5.51	56	0	2890.569	3	964.194519	7.504259154	69.16306833
4506587	RPA3	replication protein A 14 kDa subunit	NP_002938.1	14.05	1	1	4	121	13.6	5.08	1.950	2.445	2.472																
														IIHDFPQFYPLGIVQHD	4	1	1	N-Term(iTRAQ8plex)	1.95	2.445	2.472	5.92	47	0	2343.23819	3	781.7509155	-2.457269596	52.35031167
4506597	RPL12	60S ribosomal protein L12	NP_000967.1	54.55	6	6	111	165	17.8	9.42	1.303	1.584	1.536																
														EILGTAQSVGCNVDGR	8	1	1	N-Term(iTRAQ8plex); C11(Methylthio)	1.216	1.675	1.242	6.15	87	0	1968.98735	2	984.9973145	4.489610479	34.52707
														CTGGEVGATSALAPK	10	1	1	N-Term(iTRAQ8plex); C1(Methylthio); K15(iTRAQ8plex)	1.267	1.819	1.455	5.78	63	0	2016.06462	2	1008.53363	-5.693371181	20.69680167
														HSGNITFDEIVNIAR	53	1	1	N-Term(iTRAQ8plex)	1.303	1.426	1.554	5.44	90	0	1990.06438	2	995.5358276	-0.810348941	36.23967667
														HPHDIIDDINSGAVECPAS	34	1	1	N-Term(iTRAQ8plex); C16(Methylthio)	1.25	1.505	1.282	4.84	50	0	2340.08457	2	1170.547363	-1.197543921	32.77379167
														QAQIEVVPSASALIIK	4	1	1	N-Term(iTRAQ8plex); K16(iTRAQ8plex)	2.386	2.455	2.829	4.61	37	0	2275.37308	3	759.1292114	-5.063025819	38.134065
														IGPLGLSPK	2	1	1	N-Term(iTRAQ8plex); K9(iTRAQ8plex)	1.902	1.794	1.438	2.83	23	0	1489.94145	2	745.4743652	-9.931565574	37.352195
4506605	RPL23	60S ribosomal protein L23	NP_000969.1	55.00	6	6	43	140	14.9	10.51	0.988	1.413	1.350																
														ISLGLPVGAVINCADNTGAK	9	1	1	N-Term(iTRAQ8plex); C13(Methylthio); K20(iTRAQ8plex)	0.82	1.096	1.015	6.54	64	0	2567.40598	3	856.4735107	-3.366558896	53.59105333
														LPAAGVGDMVMATVK	4	1	1	N-Term(iTRAQ8plex); K15(iTRAQ8plex)	1.481	1.788	1.653	6.17	60	0	2068.17534	2	1034.591309	-0.106728137	40.44661833
														DGVFLYFEDNAGVIVNNK	5	1	1	N-Term(iTRAQ8plex); K18(iTRAQ8plex)	0.986	0.72	1.228	4.5	42	0	2622.39658	3	874.8037109	-2.235259741	48.58908833
														LPAAGVGDMVMATVK	5	1	1	N-Term(iTRAQ8plex); M9(Oxidation); M11(Oxidation); K15(iTRAQ8plex)	1.323	1.125	1.375	4.11	58	0	2100.15726	2	1034.591309	-0.106728137	40.44661833
														LPAAGVGDMVMATVK	3	1	1	N-Term(iTRAQ8plex); M11(Oxidation); K15(iTRAQ8plex)	0.859	0.934	1.022	3.86	54	0	2084.17363	2	1034.591309	-0.106728137	40.44661833
														NLYIISVK	2	1	1	N-Term(iTRAQ8plex); K8(iTRAQ8plex)	1.104	1.416	1.797	3.74	23	0	1557.98362	3	519.9993896	0.753791825	30.97896667
														VHPAVVIR	11	1	1	N-Term(iTRAQ8plex)	0.756	1.528	1.326	2.96	48	0	1194.75711	3	398.9232483	-6.076288267	15.20318
														ECADLWPR	4	1	1	N-Term(iTRAQ8plex); C2(Methylthio)	1.09	1.516	1.292	-	27	0	1339.63884	2	670.3230591	-3.897136882	52.20253
4506613	RPL22	60S ribosomal protein L22	NP_000974.1	50.78	4	4	31	128	14.8	9.19	2.047	2.105	1.759																
														FTLDCTHPVEDGIMDAANFEQFLQER	12	1	1	N-Term(iTRAQ8plex); C5(Methylthio)	2.492	3.024	2.637	7.62	69	0	3376.54099	3	1126.202026	9.599488512	57.748125
														AGNLGGGVVTIER	10	1	1	N-Term(iTRAQ8plex)	2.012	1.302	1.177	4.89	66	0	1546.89189	2	773.942627	-4.870464161	19.11918167
														ITVTSEVPFSK	7	1	1	N-Term(iTRAQ8plex); K11(iTRAQ8plex)	1.506	1.069	1.044	4.11	33	0	1816.05045	3	606.0216675	-9.472263616	28.13627833
														YFQINQDEEEEEDED	2	1	1	N-Term(iTRAQ8plex); Y1(iTRAQ8plex)	1.434	1.684	1.361	-	54	0	2540.13407	3	847.3828735	-2.694324138	24.5289
4506619	RPL24	60S ribosomal protein L24	NP_000977.1	31.85	6	6	45	157	17.8	11.25	1.570	1.985	1.982																
														AITGASLADIMAK	12	1	1	N-Term(iTRAQ8plex); K13(iTRAQ8plex)	1.82	2.143	1.924	6.38	76	0	1870.106	2	935.5566406	7.033467604	37.06882833
														VELCSFSGYK	4	1	1	N-Term(iTRAQ8plex); C4(Methylthio); K10(iTRAQ8plex)	1.086	1.705	1.654	4.66	37	0	1786.93137	3	596.3153076	-0.801764669	66.64735333
														VFQFLNAK	6	1	1	N-Term(iTRAQ8plex); K8(iTRAQ8plex)	1.67	2.841	2.44	3.88	42	0	1574.9406	2	787.973938	-6.915780469	39.95959167
														QINWTVLYR	19	1	1	N-Term(iTRAQ8plex)	1.526	1.887	1.946	3.86	37	0	1496.84709	2	748.9274902	-3.34533115	30.93207333
														MKVELCSFSGYK	2	1	1	N-Term(iTRAQ8plex); K2(iTRAQ8plex); C6(Methylthio); K12(iTRAQ8plex)	1.265	2.923	2.195	3.6	37	1	2350.2526	3	784.0890503	-8.949961853	46.71537833
														CESAFLSK	2	1	1	N-Term(iTRAQ8plex); C1(Methylthio); K8(iTRAQ8plex)	1.554	2.876	2.447	3.31	34	0	1538.80327	2	769.9052734	-8.735388896	27.6759
4506621	RPL26	60S ribosomal protein L26	NP_000978.1	16.55	3	3	7	145	17.2	10.55	1.575	1.908	1.771																
														YKEETIEK	1	1	1	N-Term(iTRAQ8plex); K2(iTRAQ8plex); K8(iTRAQ8plex)	0.971	1.821	1.578	3.41	-	1	1952.12856	4	488.7875977	-9.308072924	19.54163333
														YVIYIER	5	3	1	N-Term(iTRAQ8plex)	1.712	2.173	1.902	2.84	28	0	1259.72771	2	630.3674927	-1.915150727	31.17389333
														FNPFVTSDR	1	3	1	N-Term(iTRAQ8plex)	0.486	0.814	0.633	-	25	0	1386.72368	2	693.8654785	-5.957126763	23.50851833
4506623	RPL27	60S ribosomal protein L27	NP_000979.1	42.65	5	5	27	136	15.8	10.56	1.445	1.566	1.336																
														NIDDGTSDRPYSHALVAGIDR	5	1	1	N-Term(iTRAQ8plex)	1.453	1.803	1.363	4.14	-	0	2576.29165	4	644.8283691	-3.52235645	22.67060333
														VYNYNHLMPTR	12	1	1	N-Term(iTRAQ8plex)	1.338	1.26	0.986	3.82	48	0	1711.87918	3	571.2979126	-5.833797806	23.06872
														VYNYNHLMPTR	3	1	1	N-Term(iTRAQ8plex); M8(Oxidation)	1.755	1.864	1.464	3.17	25	0	1727.87784	3	571.2979126	-5.833797806	23.06872

														DVFRDPALK	1	1	1	N-Term(iTRAQ8plex); K9(iTRAQ8plex)	1.282	1.225	1.175	2.78	-	1	1668.98052	2	834.9938965	-5.293621563	19.18958667
														VVLVLAGR	5	1	1	N-Term(iTRAQ8plex)	1.693	1.894	1.776	2.7	29	0	1130.76323	2	565.8782349	-6.295112551	27.54121
														YSVDIPLDK	1	1	1	N-Term(iTRAQ8plex); K9(iTRAQ8plex)	1.9	1.746	2.016	-	33	0	1657.9462	3	553.3202515	-9.596303823	40.49610833
4506625	RPL27A	60S ribosomal protein L27a	NP_000981.1	22.30	2	2	19	148	16.6	11.00	1.745	2.852	2.518																
														NQSFCPTVNLDKLWTLVS EQTR	13	1	1	N-Term(iTRAQ8plex); C5(Methylthio); K12(iTRAQ8plex)	2.51	4.26	3.507	5.06	37	1	3233.68984	4	809.1773071	-1.049471998	58.56279333
														TGAAPIIDVVR	6	1	1	N-Term(iTRAQ8plex)	1.232	1.655	1.541	3.73	50	0	1415.84722	3	472.6210327	-2.730804423	30.347475
4506629	RPL29	60S ribosomal protein L29	NP_000983.1	9.43	1	1	6	159	17.7	11.66	1.481	1.846	1.682																
														AQAAAPASVPAQAPK	6	1	1	N-Term(iTRAQ8plex); K15(iTRAQ8plex)	1.481	1.846	1.682	4.34	84	0	1986.15122	2	993.5780029	-5.317859871	14.93597
4506631	RPL30	60S ribosomal protein L30	NP_000980.1	40.00	5	6	30	115	12.8	9.63	1.314	1.967	1.579																
														KSEIEYYAMLAK	2	1	1	N-Term(iTRAQ8plex); K1(iTRAQ8plex); M9(Oxidation); K12(iTRAQ8plex)	1.333	1.818	1.75	4.8	30	1	2374.33005	3	786.7810059	-9.390961526	37.07194667
														LVILANNCPALR	11	1	1	N-Term(iTRAQ8plex); C8(Methylthio)	1.662	2.087	1.724	4.37	63	0	1646.94321	3	549.6525879	2.676547587	55.610685
														KSEIEYYAMLAK	8	1	1	N-Term(iTRAQ8plex); K1(iTRAQ8plex); K12(iTRAQ8plex)	1.16	1.992	1.595	4	41	1	2358.32792	3	786.7810059	-9.390961526	37.07194667
														SEIEYYAMLAK	3	1	1	N-Term(iTRAQ8plex); K11(iTRAQ8plex)	1.101	0.512	1.174	3.95	41	0	1926.03696	3	642.6838379	-6.916211059	63.994175
														VCTLAIIDPGDSDIIR	3	1	1	N-Term(iTRAQ8plex); C2(Methylthio)	0.712	0.78	1.115	3.54	46	0	2051.07346	3	684.3626709	-4.126061233	55.49564167
														LVILANNCPALRK	1	1	1	N-Term(iTRAQ8plex); C8(Methylthio); K13(iTRAQ8plex)	-	-	-	3.51	-	1	2079.22817	4	520.5625	-5.272235225	46.99086667
														LQLVMK	2	1	2	N-Term(iTRAQ8plex); K6(iTRAQ8plex)	-	-	-	2.88	-	0	1339.84668	3	447.2870789	-9.320452773	28.80433667
4506635	RPL32	60S ribosomal protein L32	NP_000985.1	29.63	4	4	15	135	15.8	11.33	1.510	2.050	1.859																
														SYCAEIAHNVSSK	10	1	1	N-Term(iTRAQ8plex); C3(Methylthio); K13(iTRAQ8plex)	1.452	1.842	1.567	5.14	29	0	2063.03958	3	688.3513794	-5.543939384	32.70953667
														ELEVLLMCNK	2	1	1	N-Term(iTRAQ8plex); C8(Methylthio); K10(iTRAQ8plex)	1.223	1.585	1.859	4.2	59	0	1845.99858	2	923.5029297	-6.023593465	74.01896
														AAQLAIR	2	1	1	N-Term(iTRAQ8plex)	1.777	2.347	2.137	2.69	26	0	1046.6668	2	523.8370361	4.19841887	23.01729
														AALRPLVKPK	1	1	1	N-Term(iTRAQ8plex); K8(iTRAQ8plex); K10(iTRAQ8plex)	3.056	5.342	3.916	-	15	0	2005.33037	2	1003.168823	-5.461162804	16.70185167
4506643	RPL37A	60S ribosomal protein L37a	NP_000989.1	21.74	2	2	28	92	10.3	10.43	1.431	2.141	1.793																
														AVGIWHCGSCMK	15	1	1	N-Term(iTRAQ8plex); C7(Methylthio); C10(Methylthio); K12(iTRAQ8plex)	1.365	1.991	1.8	4.01	38	0	1991.95549	2	996.4813843	-2.59612493	39.87628667
														YTCSFCGK	10	1	1	N-Term(iTRAQ8plex); C3(Methylthio); C6(Methylthio); K8(iTRAQ8plex)	1.516	2.266	1.793	3.56	25	0	1608.74004	2	804.8736572	-6.362411971	33.527975
														AVGIWHCGSCMK	3	1	1	N-Term(iTRAQ8plex); C7(Methylthio); C10(Methylthio); M11(Oxidation); K12(iTRAQ8plex)	1.835	2.52	2.562	2.99	17	0	2007.93601	2	996.4813843	-2.59612493	39.87628667
4506645	RPL38	60S ribosomal protein L38	NP_000990.1	17.14	1	1	5	70	8.2	10.10	1.264	2.657	2.487																
														YLYTLVITDKEK	5	1	1	N-Term(iTRAQ8plex); K10(iTRAQ8plex); K12(iTRAQ8plex)	1.264	2.657	2.487	4.68	32	1	2398.4238	3	800.1461182	-5.103375728	39.81333167
4506647	RPL39	60S ribosomal protein L39	NP_000991.1	19.61	1	1	1	51	6.4	12.56	0.776	1.023	1.174																
														QNRPIPQWIR	1	1	1	N-Term(iTRAQ8plex)	0.776	1.023	1.174	-	25	0	1611.92827	2	806.4677734	-6.365695788	22.24726
4506649	RPL3	60S ribosomal protein L3 isoform a	NP_000958.1	30.52	12	12	74	403	46.1	10.18	1.239	1.678	1.542																
														LEQQVPVNQVFGQDEMID VIGVTK	4	2	1	N-Term(iTRAQ8plex); K24(iTRAQ8plex)	1.215	1.214	0.594	6.36	66	0	3294.79538	3	1098.936646	2.695758736	48.66538667
														SINPLGGFVHYGEVTNDFV MLK	10	2	1	N-Term(iTRAQ8plex); K22(iTRAQ8plex)	2.009	2.522	2.123	6.3	57	0	3045.63662	3	1015.884644	2.136206312	54.21434
														SINPLGGFVHYGEVTNDFV MLK	7	2	1	N-Term(iTRAQ8plex); M20(Oxidation); K22(iTRAQ8plex)	1.474	1.05	1.054	4.92	37	0	3061.62998	3	1015.884644	2.136206312	54.21434
														VACIGAWHPAR	20	4	1	N-Term(iTRAQ8plex); C3(Methylthio)	1.109	1.537	1.542	4.33	75	0	1530.79108	3	510.9352112	-4.227882669	34.06441667
														AGMTHIVR	2	2	1	N-Term(iTRAQ8plex)	1.083	0.885	0.835	3.56	35	0	1188.67389	3	402.2287598	-4.71518755	9.985395
														TVFAEHISDECK	7	2	1	N-Term(iTRAQ8plex); C11(Methylthio); K12(iTRAQ8plex)	1.367	1.613	1.399	3.31	25	0	2033.00991	3	678.3433838	-6.711302572	33.08256667
														TVFAEHISDECKR	2	2	1	N-Term(iTRAQ8plex); C11(Methylthio); K12(iTRAQ8plex)	2.81	3.131	2.174	3.18	40	1	2189.11416	4	548.0339966	-7.402926808	30.68983833
														NNASTDYDLSDK	2	2	1	N-Term(iTRAQ8plex); K12(iTRAQ8plex)	0.692	1.553	1.428	3.14	49	0	1950.97153	3	650.9953613	-7.668264446	24.547525
														HGSLGFLPR	8	2	1	N-Term(iTRAQ8plex)	1.809	2.613	2.09	3.07	40	0	1287.75226	3	429.9191895	-3.507058918	21.20356667
														IGQGYLIK	2	2	1	N-Term(iTRAQ8plex); K8(iTRAQ8plex)	1.668	2.227	2.11	2.89	26	0	1499.92558	2	750.4664307	-9.998223931	23.55173833
														VIAHTQMR	3	1	1	N-Term(iTRAQ8plex)	0.946	1.217	1.25	2.88	33	0	1259.7179	3	420.5774841	-1.351221113	14.64108
														AGMTHIVR	4	2	1	N-Term(iTRAQ8plex); M3(Oxidation)	1.142	1.508	1.146	-	36	0	1204.67173	3	402.2287598	-4.71518755	9.985395
														KVACIGAWHPAR	1	4	1	N-Term(iTRAQ8plex); K1(iTRAQ8plex); C4(Methylthio)	-	-	-	-	30	1	1963.08925	4	491.527771	-4.39521311	25.06973333
														VITLR	1	8	1	N-Term(iTRAQ8plex)	1.57	1.953	1.714	-	24	0	905.60027	2	453.303772	-9.15853825	22.95823167
														VAFSVAR	2	2	1	N-Term(iTRAQ8plex)	1.493	2.078	1.598	-	22	0	1053.62969	2	527.3184814	-5.851229759	21.760375
4506651	RPL36AL	60S ribosomal protein L36a-like	NP_000992.1	15.09	2	2	10	106	12.5	10.65	1.469	2.499	2.059																
														LECVEPNCR	8	3	1	N-Term(iTRAQ8plex); C3(Methylthio); C8(Methylthio)	1.616	2.288	2.059	3.3	29	0	1458.63982	2	729.8258057	-4.93512483	29.02941
														KGQVIQF	2	2	1	N-Term(iTRAQ8plex); K1(iTRAQ8plex)	0.671	3.34	1.976	-	28	1	1427.87006	3	476.6282043	-9.112532896	25.33691667
4506661	RPL7A	60S ribosomal protein L7a	NP_000963.1	32.71	8	8	40	266	30.0	10.61	1.561	1.975	1.468																
														TCTTVAFTQVNSEDKGAL AK	2	1	1	N-Term(iTRAQ8plex); C2(Methylthio); K15(iTRAQ8plex); K20(iTRAQ8plex)	-	-	-	6.3	32	1	3042.61391	4	761.4089355	-7.503414221	29.83913667
														AGVNTVTTLVENK	7	1	1	N-Term(iTRAQ8plex); K13(iTRAQ8plex)	1.607	1.576	1.402	6.25	73	0	1954.12908	2	977.5681763	-7.100544743	46.11211
														LKVPPAINQFTQALDR	15	1	1	N-Term(iTRAQ8plex); K2(iTRAQ8plex)	1.171	1.561	1.434	4.49	50	1	2419.42275	3	807.1466064	-1.213186497	42.19366833
														HWGGNVLGPK	3	1	1	N-Term(iTRAQ8plex); K10(iTRAQ8plex)	1.494	2.505	2.206	3.85	31	0	1672.95989	2	836.9835815	-8.652230206	19.71343167
														RHWGGNVLGPK	1	1	1	N-Term(iTRAQ8plex); K11(iTRAQ8plex)	0.825	1.846	1.261	3.81	-	1	1829.05991	3	610.3581543	-8.513476947	17.06000333
														VAPAPAVVK	2	1	1	N-Term(iTRAQ8plex); K9(iTRAQ8plex)	1.084	1.289	1.383	3.78	32	0	1459.95938	3	487.324646	9.379328236	22.76912
														MGVPYCIIK	5	1	1	N-Term(iTRAQ8plex); C6(Methylthio); K9(iTRAQ8plex)	2.107	2.922	2.326	3.55	26	0	1677.92209	3	559.9788818	-7.729823838	65.559845
														KVVNPLFEK	5	1	1	N-Term(iTRAQ8plex); K1(iTRAQ8plex); K9(iTRAQ8plex)	2.241	2.819	2.504	3.43	24	1	1986.23343	3	662.7493286	-9.091937463	28.34745167
4506669	RPLP1	60S acidic ribosomal protein P1 isoform 1	NP_000994.1	28.95	1	2	39	114	11.5	4.32	1.815	3.194	2.951																
														AAGVNVEPFWPGLFAK	34	1	1	N-Term(iTRAQ8plex); K16(iTRAQ8plex)	1.815	3.194	2.951	5.65	66	0	2311.29302	2	1156.150146	-5.605066583	43.74410833
														KEESEESDDDMGFGLFD	5	3	2	N-Term(iTRAQ8plex); K1(iTRAQ8plex)	-	-	-	2.83	38	1	2558.15311	3	853.3892212	-6.65752793	63.779365
4506671	RPLP2	60S acidic ribosomal protein P2	NP_000995.1	57.39	5	6	72	115	11.7	4.54	1.359	2.118	2.154																
														MRYVASYLLAALGGNSSP SAK	17	1	1	N-Term(iTRAQ8plex); K21(iTRAQ8plex)	1.095	1.843	1.521	6.2	59	1	2764.53896	3	922.1790161	-1.868753163	64.92575333
														YVASYLLAALGGNSSPSAK	20	1	1	N-Term(iTRAQ8plex); K19(iTRAQ8plex)	0.936	1.511	1.279	5.91	73	0	2477.38639	2	1239.205811	7.388044584	53.786595
														NIEDVIAQGIGK	16	1	1	N-Term(iTRAQ8plex); K12(iTRAQ8plex)	1.494	2.7	2.351	5.9	79	0	1865.08122	2	933.0457153	-5.966087864	36.16898667
														ILDSVGIEADDDRLNK	9	1	1	N-Term(iTRAQ8plex); K16(iTRAQ8plex)	1.884	2.929	2.535	5.36	71	1	2381.30278	2	1191.155029	-4.417466913	32.09907833
														MRYVASYLLAALGGNSSP SAK	1	1	1	N-Term(iTRAQ8plex); M1(Oxidation); K21(iTRAQ8plex)	-	3.356	2.984	4.54	-	1	2780.50747	3	922.1790161	-1.868753163	64.92575333
														ILDSVGIEADDDR	4	1	1	N-Term(iTRAQ8plex)	1.12	1.146	1.23	4.08	56	0	1721.87969	2	861.4434814	-3.65009787	36.24223
														KEESEESDDDMGFGLFD	5	3	2	N-Term(iTRAQ8plex); K1(iTRAQ8plex)	-	-	-	2.83	38	1	2558.15311	3	853.3892212	-6.65752793	63.779365
4506675	RPN1	dolichyl-diphosphooligosaccharide--protein glycosyltransferase subunit 1 precursor	NP_002941.1	48.43	22	22	121	607	68.5	6.38	0.973	0.596	0.689																
														THYIVGYNLPSYEYLYNLG DQYALK	8	1	1	N-Term(iTRAQ8plex); K25(iTRAQ8plex)	-	0.878	0.762	6.75	61	0	3605.86704	3	1202.627197	-2.945963623	53.52748
														VTAEVVLAHLGGGSTSR	10	1	1	N-Term(iTRAQ8plex)	1.141	0.656	0.84	5.78	67	0	1958.09148	2	979.5493774	-2.973833067	34.508325
														QFVVFEGNHYFYSPYPTK	4	1	1	N-Term(iTRAQ8plex); K18(iTRAQ8plex)	0.989	0.664	0.609	5.35	30	0	2831.46439	3	944.492981	-0.332576638	40.80904167
														VHYENNSPFLTITSMTR	14	1	1	N-Term(iTRAQ8plex)	0.885	1.002	0.968	5.19	65	0	2314.17405	3	772.0628662	-2.72923171	38.40045833
														GEDEEENNLEVR	8	1	1	N-Term(iTRAQ8plex)	1.076	0.572	0.611	5.17	69	0	1736.82456	3	579.6130371	0.268753757	20.60335
														ATSFLLALEPELEAR	6	1	1	N-Term(iTRAQ8plex)	0.998	0.613	0.786	5.17	75	0	1964.11235	2	982.5598145	5.952300151	60.605425
														FVDHVFDEQVIDSLTVK	2	1	1	N-Term(iTRAQ8plex); K17(iTRAQ8plex)	1.162	0.716	0.664	5.04	47	0	2599.42746	3	867.1473389	1.778103689	50.29382
														NLVEQHIQDIVVHYTFNK	4	1	1	N-Term(iTRAQ8plex); K18(iTRAQ8plex)	1.113	0.639	0.661	4.8	-	0	2805.55036	5	561.9158936	-0.160409369	47.388385
														NIEIDSPYEISR	6	1	1	N-Term(iTRAQ8plex)	1.04	0.39	0.449	4.68	53	0	1739.91802	2	870.4626465	3.59502499	29.93773833
														ALTSEIALLQSR	4	1	1	N-Term(iTRAQ8plex)	0.755	0.378	0.571	4.47	55	0	1605.9437	3	535.986084	-2.529725593	44.645635
														ISVIVETVYTHVLHPYPTQI TQSEK	1	1	1	N-Term(iTRAQ8plex); K25(iTRAQ8plex)	-	-	-	4.23	-	0	3490.96998	5	698.9998169	8.406449452	65.99312667
														FPLFGGWK	6	1	1	N-Term(iTRAQ8plex); K8(iTRAQ8plex)	1.041	0.551	0.561	4.22	32	0	1559.91501	3	520.6431885	-2.85856619	69.92265
														VACITEQVLTLVNKR	9	1	1	N-Term(iTRAQ8plex); C3(Methylthio); K14(iTRAQ8plex)	0.945	0.547	0.636	4.09	81	1	2341.35927	2	1171.180786	-0.566618379	64.40786667
														SEDLLDYGPFR	13	1	1	N-Term(iTRAQ8plex)	0.948	0.576	0.666	4.07	48	0	1615.82024	2	808.4160156	-1.371850501	36.407595
														VACITEQVLTLVNK	6	1	1	N-Term(iTRAQ8plex); C3(Methylthio); K14(iTRAQ8plex)	0.788	0.449	0.686	4.06	67	0	2185.25884	2	1093.133057	1.983454398	59.35337167
														LDFSITKDPAAEAR	2	1	1	N-Term(iTRAQ8plex); K7(iTRAQ8plex)	1.276	-	0.813	3.98	32	1	2142.19814	4	536.3049927	-1.592045483	29.89498167
														LAHLGVQVKGEDEEENNL EVR	1	1	1	N-Term(iTRAQ8plex); K9(iTRAQ8plex)	-	-	-	3.91	-	1	2986.60537	4	747.4067993	-0.038141757	28.75375833
														TVDLSSHLAK	5	1	1	N-Term(iTRAQ8plex); K10(iTRAQ8plex)	1.087	0.524	0.625	3.78	49	0	1678.99088	2	839.9996338	-1.679782804	19.16539333
														DTYIENEK	3	1	1	N-Term(iTRAQ8plex); K8(iTRAQ8plex)	1.129	0.729	0.769	3.51	37	0	1619.87784	2	810.4381104	-2.924444531	14.12417
														TILPAAAQDVYYR	4	1	1	N-Term(iTRAQ8plex)	0.931	0.608	0.679	3.41	44	0	1784.97978	2	892.9935303	-2.844498952	34.702155
														LPVALDPGAK	2	1	1	N-Term(iTRAQ8plex); K10(iTRAQ8plex)	0.875	0.435	0.521	3	52	0	1588.98003	2	794.9936523	-5.200337546	23.60928167
														IDHILDAL	3	1	1	N-Term(iTRAQ8plex)	0.788	0.768	0.848	-	36	0	1213.70928	2	607.3582764	-0.111382213	43.193475
4506681	RPS11	40S ribosomal protein S11	NP_001006.1	41.77	7	7	23	158	18.4	10.30	1.399	2.221	1.968																
														DVQIGDIVTVGECRPLSK	6	1	1	N-Term(iTRAQ8plex); C13(Methylthio); K18(iTRAQ8plex)	1.506	2.504	2.061	4.94	37	0	2583.40127	4	646.6057739	-3.199842388	41.80302
														EAIEGTYIDK	2	1	1	N-Term(iTRAQ8plex); K10(iTRAQ8plex)	1.032	1.686	1.508	4.05	51	0	1746.95903	2	873.9831543	-8.231060939	32.60684
														NMSVHLSPCFR	2	1	1	N-Term(iTRAQ8plex); C9(Methylthio)	0.969	0.612	1.149	4.01	27	0	1640.79435	3	547.6029663	-4.245885072	38.37233667
														EAIEGTYIDKK	1	1	1	N-Term(iTRAQ8plex); K10(iTRAQ8plex); K11(iTRAQ8plex)	1.2	2.081	1.954	3.98	-	1	2179.25371	2	1090.130493	-9.191950339	19.41214333
														QPTIFQNK	3	1	1	N-Term(iTRAQ8plex); K8(iTRAQ8plex)	1.52	2.463	2.226	3.49	39	0	1583.92754	2	792.4674072	-5.69689374	16.19957333
														CPFTGNVSIR	5	1	1	N-Term(iTRAQ8plex); C1(Methylthio)	1.455	2.454	2.201	3.08	34	0	1443.73174	2	722.3695068	-5.046123139	35.77070333
														RDYLHYIR	3	1	1	N-Term(iTRAQ8plex)	-	-	-	2.86	31	1	1439.80068	4	360.7056274	-3.751501475	23.80258333
														NMSVHLSPCFR	1	1	1	N-Term(iTRAQ8plex); M2(Oxidation); C9(Methylthio)	1.123	1.145	1.034	2.85	-	0	1656.78568	3	547.6029663	-4.245885072	38.37233667
4506685	RPS13	40S ribosomal protein S13	NP_001008.1	43.71	7	7	45	151	17.2	10.54	1.303	2.116	1.946																
														GLAPDLPEDLYHLIK	15	1	1	N-Term(iTRAQ8plex); K15(iTRAQ8plex)	1.517	2.266	2.013	5.53	58	0	2302.32657	2	1151.66333	-3.195804979	52.12417833
														LTSDDVKEQIYK	1	1	1	N-Term(iTRAQ8plex); K7(iTRAQ8plex); K12(iTRAQ8plex)	-	-	-	4.21	-	1	2351.33584	4	588.5894165	-9.652030146	24.25873333
														KGLTPSQIGVILR	8	1	1	N-Term(iTRAQ8plex); K1(iTRAQ8plex)	1.302	2.268	1.953	4.14	54	1	1990.2482	4	498.3182678	-6.084604137	30.50044833
														GLTPSQIGVILR	2	1	1	N-Term(iTRAQ8plex)	0.824	1.069	1.224	3.4	28	0	1557.95793	2	779.482605	-3.265787191	39.300935
														DSHGVAQVR	5	1	1	N-Term(iTRAQ8plex)	0.9	2.09	2.076	3.21	30	0	1272.68955	3	424.9013672	-5.243217019	9.953193333
														GLSQSALPYR	9	1	1	N-Term(iTRAQ8plex)	1.241	1.346	1.236	2.87	35	0	1395.78606	2	698.3966675	-2.66728489	24.34518
														LILIESR	5	1	1	N-Term(iTRAQ8plex)	1.346	2.241	2.017	-	25	0	1147.73064	2	574.3689575	-4.00170631	34.962895
4506687	RPS15	40S ribosomal protein S15 isoform 2	NP_001009.1	48.97	4	5	13	145	17.0	10.39	1.256	2.949	2.938																
														DMIILPEMVGSMVGVYNG K	6	2	1	N-Term(iTRAQ8plex); K19(iTRAQ8plex)	1.157	2.654	2.859	6.72	54	0	2661.42459	2	1331.227905	7.898941726	59.84766333
														GVDLDQLLDMSYEQLMQ LYSAR	4	2	1	N-Term(iTRAQ8plex)	-	-	-	5.04	115	0	2892.44474	3	964.8197632	0.748502039	70.66444
														EAPPMEKPEVVK	1	2	1	N-Term(iTRAQ8plex); K7(iTRAQ8plex); K12(iTRAQ8plex)	1.364	3.473	3.018	3.77	-	0	2266.30449	2	1133.655884	-8.798615523	21.907735
														HGRPGIGATHSSR	1	2	1	N-Term(iTRAQ8plex)	-	-	-	2.77	-	0	1636.88796	4	409.9774475	-3.305749122	7.313933333
														FIPLK	1	2	2	N-Term(iTRAQ8plex); K5(iTRAQ8plex)	-	-	-	-	21	0	1225.80924	3	409.2745972	-2.955636147	30.547225
4506691	RPS16	40S ribosomal protein S16 isoform 1	NP_001011.1	45.89	8	8	58	146	16.4	10.21	1.390	2.524	2.199																

														LLEPVLLLGK	30	3	1	N-Term(iTRAQ8plex); K10(iTRAQ8plex)	1.448	3.113	2.691	4.09	41	0	1703.11418	2	852.06073	-8.782710642	45.88657833
														ALVAYYQK	2	2	1	N-Term(iTRAQ8plex); K8(iTRAQ8plex)	1.151	1.743	1.917	3.64	31	0	1563.91997	2	782.463623	-9.918555011	24.19971667
														GGGHVAQIYAIR	10	2	1	N-Term(iTRAQ8plex)	1.147	1.961	1.651	3.62	51	0	1545.87602	2	773.4416504	-2.792681096	20.47073833
														EIKDILIQYDR	7	2	1	N-Term(iTRAQ8plex); K3(iTRAQ8plex)	1.234	2.537	2.241	3.39	39	1	2014.16543	4	504.296814	-6.903762941	46.55777
														GPLQSVQVFGR	2	3	1	N-Term(iTRAQ8plex)	1.57	2.524	1.976	3.03	44	0	1491.85051	2	746.428894	-5.389168687	25.47747
														DILIQYDR	4	2	1	N-Term(iTRAQ8plex)	1.425	2.331	1.831	2.81	28	0	1339.75456	2	670.3809204	1.666648782	29.78888167
														PSKGPLQSVQVFGR	2	3	1	N-Term(iTRAQ8plex); K3(iTRAQ8plex)	2.124	4.134	3.548	-	39	1	2108.23185	3	703.4154663	-5.614005045	26.36376333
														VNGRPLEMIEPR	1	2	1	N-Term(iTRAQ8plex)	1.133	1.928	2.199	-	24	0	1714.95261	3	572.3223877	-2.933879358	24.83847333
4506693	RPS17	40S ribosomal protein S17	NP_001012.1	56.30	5	5	24	135	15.5	9.85	1.301	2.777	2.462																
														LLDFGSLSNLQVTQPTVGMNFK	4	1	1	N-Term(iTRAQ8plex); K22(iTRAQ8plex)	1.779	4.255	2.479	6.58	56	0	3017.67827	2	1509.342773	6.355533388	54.83693833
														RDNYVPEVSALDQEIIEVDPDTK	4	1	1	N-Term(iTRAQ8plex); K23(iTRAQ8plex)	-	-	-	4.85	32	1	3253.69204	4	814.1784668	-3.959806998	41.36100167
														IAGYVTHLMK	6	1	1	N-Term(iTRAQ8plex); K10(iTRAQ8plex)	1.112	2.161	2.008	3.94	31	0	1741.01731	2	879.0081787	-8.494832661	26.89335667
														VCEEIAIIPSK	4	1	1	N-Term(iTRAQ8plex); C2(Methylthio); K11(iTRAQ8plex)	1.106	2.879	2.616	3.3	35	0	1856.03594	2	928.5305176	2.99896284	42.17353
														LLDFGSLSNLQVTQPTVGMNFK	2	1	1	N-Term(iTRAQ8plex); M19(Oxidation); K22(iTRAQ8plex)	-	-	-	3.2	47	0	3033.65055	2	1509.342773	6.355533388	54.83693833
														IAGYVTHLMK	2	1	1	N-Term(iTRAQ8plex); M9(Oxidation); K10(iTRAQ8plex)	1.344	1.832	1.708	3.17	33	0	1757.00908	2	879.0081787	-8.494832661	26.89335667
														LGNDFHTNKR	2	1	1	N-Term(iTRAQ8plex); K9(iTRAQ8plex)	1.67	2.955	2.731	2.92	-	1	1810.02004	4	453.2604675	1.120947361	13.19861833
4506697	RPS20	40S ribosomal protein S20 isoform 2	NP_001014.1	25.21	3	3	21	119	13.4	9.94	1.237	2.693	2.303																
														LIDLHSPSEIVK	7	2	1	N-Term(iTRAQ8plex); K12(iTRAQ8plex)	1.081	1.796	1.477	5.22	46	0	1959.16045	2	980.0846558	-5.862912999	29.44712667
														TPVEPEVAIHR	8	2	1	N-Term(iTRAQ8plex)	1.132	2.473	2.303	3.47	35	0	1551.87407	3	517.9633179	-2.758556981	18.43694333
														VCADLIR	6	2	1	N-Term(iTRAQ8plex); C2(Methylthio)	1.33	3.202	2.725	-	41	0	1139.61418	2	570.31073	-6.738209453	40.42308333
4506699	RPS21	40S ribosomal protein S21	NP_001015.1	44.58	4	4	18	83	9.1	8.50	1.285	3.363	2.927																
														DHASIQMNVAEVDK	2	1	1	N-Term(iTRAQ8plex); K14(iTRAQ8plex)	0.565	1.085	0.803	4.8	40	0	2165.14341	3	722.3859863	-2.182564726	20.998745
														DHASIQMNVAEVDK	1	1	1	N-Term(iTRAQ8plex); M7(Oxidation); K14(iTRAQ8plex)	0.887	0.729	0.967	3.62	-	0	2181.1276	3	722.3859863	-2.182564726	20.998745
														TYAICGAIR	11	1	1	N-Term(iTRAQ8plex); C5(Methylthio)	1.352	4.005	3.386	3.56	35	0	1317.68657	2	659.3482056	-5.272705834	36.158845
														DHASIQMNVAEVDKVTGR	1	1	1	N-Term(iTRAQ8plex); K14(iTRAQ8plex)	1.268	2.497	2.485	2.79	-	1	2578.37308	3	860.1292114	-5.329047513	27.73580667
														MGESDDSILR	2	1	1	N-Term(iTRAQ8plex)	0.889	2.628	2.292	2.7	30	0	1426.70683	2	721.855835	-3.821294335	17.12496667
														MGESDDSILR	1	1	1	N-Term(iTRAQ8plex); M1(Oxidation)	1.299	3.363	3.067	-	32	0	1442.70439	2	721.855835	-3.821294335	17.12496667
4506701	RPS23	40S ribosomal protein S23	NP_001016.1	49.65	6	6	33	143	15.8	10.49	1.199	2.116	1.876																
														KITAFVPNDGCLNFIEENDEVLVAGFGR	4	1	1	N-Term(iTRAQ8plex); K1(iTRAQ8plex); C11(Methylthio)	-	-	-	5.57	54	1	3721.91445	4	931.2340698	-0.941150898	65.56103
														VANVSLLALYK	14	1	1	N-Term(iTRAQ8plex); K11(iTRAQ8plex)	1.299	2.387	2.031	5.38	54	0	1799.10906	2	900.0581665	-8.918254361	67.040705
														ANPFGGASHAK	4	1	1	N-Term(iTRAQ8plex); K11(iTRAQ8plex)	1.114	1.797	1.437	3.6	45	0	1664.92034	2	832.9638062	-7.54127092	19.72946
														GHAVGDIPGVR	4	1	1	N-Term(iTRAQ8plex)	0.94	1.31	1.33	3.45	60	0	1381.77793	3	461.2641602	-5.40179511	15.990825
														KGHAVGDIPGVR	5	1	1	N-Term(iTRAQ8plex); K1(iTRAQ8plex)	0.939	2.427	2.072	3.09	30	1	1814.08573	3	605.3667603	0.003424159	14.412325
														KAHLGTALK	2	1	1	N-Term(iTRAQ8plex); K1(iTRAQ8plex); K9(iTRAQ8plex)	1.74	2.958	2.69	3.06	17	1	1851.18729	3	617.7339478	-3.787454851	13.73560667
4506707	RPS25	40S ribosomal protein S25	NP_001019.1	15.20	2	2	9	125	13.7	10.11	1.616	3.966	3.488																
														AALQELLSK	4	1	1	N-Term(iTRAQ8plex); K9(iTRAQ8plex)	2.212	4.292	4.504	3.84	49	0	1580.97429	2	790.9907837	-5.629779839	34.08427833
														LITPAVVSER	5	1	1	N-Term(iTRAQ8plex)	1.321	3.231	2.928	3.04	40	0	1388.8355	2	694.9213867	-4.323393601	28.85422833
4506711	RPS27	40S ribosomal protein S27	NP_001021.1	38.10	1	3	22	84	9.5	9.45	1.674	2.006	1.593																
														DLLHPSPEEEK	6	1	1	N-Term(iTRAQ8plex); K11(iTRAQ8plex)	1.674	2.006	1.593	3.45	36	0	1902.02739	2	951.517334	-8.154431067	16.90736833
														LVQSPNSYFMDVK	12	2	2	N-Term(iTRAQ8plex); K13(iTRAQ8plex)	-	-	-	5.65	57	0	2136.15166	2	1068.579468	-4.827547277	36.80544333
														LTEGCSFR	4	2	2	N-Term(iTRAQ8plex); C5(Methylthio)	-	-	-	-	30	0	1262.60991	2	631.8085938	-6.012482505	23.43498833
4506715	RPS28	40S ribosomal protein S28	NP_001022.1	46.38	3	3	19	69	7.8	10.70	1.409	2.423	2.260																
														EGDVLTLLESER	10	1	1	N-Term(iTRAQ8plex)	1.686	1.59	1.946	5.16	62	0	1664.91313	2	832.9602051	7.359084389	41.67244667
														VEFMDDTSR	5	1	1	N-Term(iTRAQ8plex)	1.278	1.867	1.644	2.74	28	0	1403.67497	2	710.3348389	-7.315250173	14.86116667
														VEFMDDTSR	3	1	1	N-Term(iTRAQ8plex); M4(Oxidation)	1.409	2.792	3.127	-	32	0	1419.6624	2	710.3348389	-7.315250173	14.86116667
														TGSQGQCTQVR	1	1	1	N-Term(iTRAQ8plex); C7(Methylthio)	1.414	3.527	3.233	-	29	0	1514.73125	2	757.8692627	-2.953348575	16.31469167
4506717	RPS29	40S ribosomal protein S29 isoform 1	NP_001023.1	32.14	2	2	5	56	6.7	10.13	1.094	1.699	1.334																
														GHQQLYWSHPR	4	2	1	N-Term(iTRAQ8plex)	1.223	1.434	1.299	-	31	0	1712.88296	4	428.9761963	-5.444635736	13.35408
														YGLNMCR	1	2	1	N-Term(iTRAQ8plex); C6(Methylthio)	1.094	2.292	1.93	-	19	0	1206.56548	2	603.786377	-6.667697611	29.35593667
4506723	RPS3A	40S ribosomal protein S3a isoform 1	NP_000997.1	27.27	6	6	40	264	29.9	9.73	1.113	1.727	1.676																
														ACQSIYPLHDVFVR	21	1	1	N-Term(iTRAQ8plex); C2(Methylthio)	1.22	1.727	1.536	5.19	51	0	1998.02195	3	666.6781006	-2.284157697	40.22900333
														VFEVSLADLQNDEVAFR	2	1	1	N-Term(iTRAQ8plex)	1.635	2.925	2.59	4.53	78	0	2256.18047	2	1128.593872	-0.431605268	60.073445
														NCLTNFHGMDLTR	10	1	1	N-Term(iTRAQ8plex); C2(Methylthio)	0.927	1.615	1.914	4.32	33	0	1871.88091	2	936.4440918	-3.170781981	35.10529333
														NCLTNFHGMDLTR	3	1	1	N-Term(iTRAQ8plex); C2(Methylthio); M9(Oxidation)	1.018	1.036	1.007	3.45	22	0	1887.87375	2	936.4440918	-3.170781981	35.10529333
														LMELHGEGSSSGK	2	1	1	N-Term(iTRAQ8plex); K13(iTRAQ8plex)	1.661	2.227	1.86	3.29	35	0	1940.01944	3	647.3446655	-8.937007321	17.69107667
														VVDPFSKK	1	2	1	N-Term(iTRAQ8plex); K7(iTRAQ8plex); K8(iTRAQ8plex)	1.154	2.621	1.62	2.71	-	1	1832.12417	4	458.786499	-9.117878885	26.249605
														MMEIMTR	1	1	1	N-Term(iTRAQ8plex)	1.073	2.106	1.647	-	20	0	1215.61125	2	608.3092651	-7.335936169	23.98254167
4506725	RPS4X	40S ribosomal protein S4, X isoform X isoform	NP_000998.1	37.26	9	9	47	263	29.6	10.15	1.263	1.661	1.520																
														TDITYPAGFMDVISIDK	6	1	1	N-Term(iTRAQ8plex); K17(iTRAQ8plex)	1.488	2.271	2.748	6.62	96	0	2494.35649	2	1247.681885	8.215189492	47.24266333
														FDTGNLCMVTGGANLGR	4	1	1	N-Term(iTRAQ8plex); C7(Methylthio)	1.061	1.178	1.058	5.8	54	0	2076.01323	2	1046.498413	-1.552468618	42.67968167
														VNDTIQIDLETGK	12	1	1	N-Term(iTRAQ8plex); K13(iTRAQ8plex)	1.216	1.515	2.079	5.01	74	0	2054.14824	2	1027.580078	-2.980143328	25.721895
														ERHPGSFDVVHVK	1	3	1	N-Term(iTRAQ8plex); K13(iTRAQ8plex)	0.836	1.453	1.477	4.65	-	1	2115.17255	5	423.840332	-9.180174788	19.253935
														GIPHLVTHDAR	13	4	1	N-Term(iTRAQ8plex)	1.329	1.572	1.387	4.02	45	0	1519.85439	2	760.4404907	5.930443618	13.89330167
														HPGSFDVVHVK	2	3	1	N-Term(iTRAQ8plex); K11(iTRAQ8plex)	1.172	2.036	1.645	4	-	0	1830.03165	3	610.6820679	-9.071393227	16.45215167
														FDTGNLCMVTGGANLGR	3	1	1	N-Term(iTRAQ8plex); C7(Methylthio); M8(Oxidation)	1.086	1.872	1.331	3.47	57	0	2091.98955	2	1046.498413	-1.552468618	42.67968167
														ECLPLIIFLR	3	1	1	N-Term(iTRAQ8plex); C2(Methylthio)	1.13	1.204	1.384	3.35	33	0	1566.89939	3	522.9713135	-3.819722161	65.19382833
														GNKPWISLPR	1	1	1	N-Term(iTRAQ8plex); K3(iTRAQ8plex)	1.482	2.752	2.193	2.68	-	0	1776.06536	2	888.5363159	-4.913154821	24.17113
														LTIAEER	1	2	1	N-Term(iTRAQ8plex)	1.556	2.381	2.482	-	31	0	1135.65691	2	568.3320923	-4.889988912	21.417575
														TDITYPAGFMDVISIDK	1	1	1	N-Term(iTRAQ8plex); Y5(iTRAQ8plex); K17(iTRAQ8plex)	-	-	-	-	27	0	2798.52163	2	1247.681885	8.215189492	47.24266333
4506741	RPS7	40S ribosomal protein S7	NP_001002.1	47.42	7	7	67	194	22.1	10.10	1.406	1.839	1.842																
														TLTAVHDAILEDLVFPSEIVGK	32	1	1	N-Term(iTRAQ8plex); K22(iTRAQ8plex)	1.366	1.869	1.558	5.62	106	0	2975.69682	2	1488.352051	1.809203967	62.82862667
														KAIIIFVPVPQLK	8	1	1	N-Term(iTRAQ8plex); K1(iTRAQ8plex); K13(iTRAQ8plex)	1.78	1.8	2.311	5.24	49	1	2378.55864	2	1189.782959	-3.363360949	47.28367833
														AQQNNVEHK	4	1	1	N-Term(iTRAQ8plex); K9(iTRAQ8plex)	1.296	1.441	1.454	4.46	34	0	1675.92038	3	559.3116455	-7.887025359	8.75525
														AIIIFVPVPQLK	10	1	1	N-Term(iTRAQ8plex); K12(iTRAQ8plex)	1.483	2.595	2.609	4.06	54	0	1946.25286	2	973.6300659	-6.913841656	43.70400833
														HVVFIAQR	11	1	1	N-Term(iTRAQ8plex)	1.315	1.83	1.769	3.32	45	0	1273.75907	3	425.2588501	-4.917917983	15.60095333
														VETFSGVYKK	1	1	1	N-Term(iTRAQ8plex); K9(iTRAQ8plex); K10(iTRAQ8plex)	1.406	2.104	1.737	3.29	-	1	2070.21645	4	518.3095703	-9.549692249	30.25932333
														IVKPNGEKPDEFESGISQALLELEMNSDLK	1	1	1	N-Term(iTRAQ8plex); K3(iTRAQ8plex); K8(iTRAQ8plex); K30(iTRAQ8plex)	-	-	-	3.19	-	0	4547.45578	3	1516.490112	-9.499297628	56.08181167
4506743	RPS8	40S ribosomal protein S8	NP_001003.1	48.08	10	10	89	208	24.2	10.32	1.287	1.809	1.744																
														ISSLLEEQFQQGKLLACIASRPGQCGR	2	1	1	N-Term(iTRAQ8plex); K13(iTRAQ8plex); C17(Methylthio); C25(Methylthio)	-	-	-	6.92	-	1	3632.90113	5	727.3860474	0.458863687	62.78255333
														LDVGNFSWGSECCTR	14	1	1	N-Term(iTRAQ8plex); C12(Methylthio); C13(Methylthio)	1.475	2.424	2.077	5.77	71	0	2069.90288	2	1035.455078	8.380262751	48.02082
														ISSLLEEQFQQGK	4	1	1	N-Term(iTRAQ8plex); K13(iTRAQ8plex)	1.369	1.856	1.569	5.16	60	0	2115.18509	3	705.7332153	-2.618472457	39.00320667
														NCIVLIDSTPYR	13	1	1	N-Term(iTRAQ8plex); C2(Methylthio)	0.948	1.421	1.582	4.54	42	0	1743.91733	3	581.9772949	5.613348436	41.31654
														IIDVVYNASNNELVR	22	1	1	N-Term(iTRAQ8plex)	1.487	2.314	2.158	4.48	69	0	2023.10893	2	1012.058105	-1.812548221	52.79822667
														NCIVLIDSTPYRQWYESHYALPLGR	4	1	1	N-Term(iTRAQ8plex); C2(Methylthio)	-	-	-	4.44	-	1	3344.67617	4	836.9244995	-3.011990584	57.34775833
														QWYESHYALPLGR	11	1	1	N-Term(iTRAQ8plex)	1.115	1.326	1.508	4.28	55	0	1923.99797	2	962.5026245	-2.042048182	33.097685
														LTPEEEEILNK	12	1	1	N-Term(iTRAQ8plex); K11(iTRAQ8plex)	1.142	2.132	1.721	4.26	45	0	1923.07431	3	641.6962891	-7.907839229	47.322335
														LLACIASRPGQCGR	2	1	1	N-Term(iTRAQ8plex); C4(Methylthio); C12(Methylthio)	1.228	1.697	1.663	2.94	-	0	1840.93071	2	920.9689941	-0.717918791	41.935545
														ELEFYLR	4	1	1	N-Term(iTRAQ8plex)	1.049	1.589	1.561	2.92	-	0	1273.70183	2	637.3545532	-5.94446401	31.85153167
														IIDVVYNASNNELVR	1	1	1	N-Term(iTRAQ8plex); Y6(iTRAQ8plex)	0.727	1.866	2.297	-	32	0	2327.30947	2	1012.058105	-1.812548221	52.79822667
4506753	RUVBL1	ruvB-like 1 isoform 1	NP_003698.1	36.62	12	12	35	456	50.2	6.42	1.012	1.286	1.314																
														VPFCPMVGSEVYSTEIK	4	8	1	N-Term(iTRAQ8plex); C4(Methylthio); K17(iTRAQ8plex)	0.96	0.963	0.904	6.29	44	0	2540.30596	2	1270.656616	-0.002558009	47.65873833
														TALALAIAQELGSK	2	8	1	N-Term(iTRAQ8plex); K14(iTRAQ8plex)	1.352	1.575	2.339	5.09	39	0	1994.20877	3	665.4077759	-0.941181939	78.70303167
														ALESSIAPIVIFASNR	4	8	1	N-Term(iTRAQ8plex)	1.021	1.094	1.146	5.02	64	0	1992.14884	3	664.7211304	2.83440834	55.68024667
														AQTEGINISEEALNHLGEIGTK	2	4	1	N-Term(iTRAQ8plex); K22(iTRAQ8plex)	-	-	-	4.85	38	0	2932.57143	4	733.8983154	-4.173627662	43.39814
														TISHVIIGLK	4	8	1	N-Term(iTRAQ8plex); K10(iTRAQ8plex)	0.964	1.178	1.18	4.38	36	0	1689.07596	2	845.0402832	-8.89926674	27.48334167
														YSVQLLTPANLLAK	2	2	1	N-Term(iTRAQ8plex); K14(iTRAQ8plex)	1.609	1.367	1.357	4.36	32	0	2139.29166	3	713.7687378	-3.796196304	48.068985
														LDPSIFESLQK	2	8	1	N-Term(iTRAQ8plex); K11(iTRAQ8plex)	0.929	1.379	1.329	4.23	47	0	1885.07329	2	943.0402832	-8.398354502	33.96933833
														EACGVIVELIK	5	5	1	N-Term(iTRAQ8plex); C3(Methylthio); K11(iTRAQ8plex)	1.117	1.304	1.329	4.13	41	0	1828.04367	3	610.0194092	-5.255897916	46.96835167
														GLGLDESGLAK	2	5	1	N-Term(iTRAQ8plex); K11(iTRAQ8plex)	0.965	1.56	1.665	3.65	47	0	1667.96428	2	834.4857788	-8.735380594	33.63473667
														GTEDITSPHGIPLDLLDR	6	7	1	N-Term(iTRAQ8plex)	1.021	1.253	1.217	3.64	38	0	2253.20194	3	751.7388306	-0.42732556	38.39320833
														KTEVLMENFR	1	8	1	N-Term(iTRAQ8plex); K1(iTRAQ8plex)	0.791	1.194	1.028	2.84	-	1	1875.0452	3	625.6865845	-8.891056082	27.43179
														QAASGLVGQENAR	1	5	1	N-Term(iTRAQ8plex)	0.854	1.287	1.633	-	23	0	1604.86101	2	802.9341431	-3.004488961	19.66960667
4506761	S100A10	protein S100-A10	NP_002957.1	45.36	3	3	16	97	11.2	7.37	0.304	0.398	0.621																
														EFPGFLENQKDPLAVDK	6	1	1	N-Term(iTRAQ8plex); K10(iTRAQ8plex); K17(iTRAQ8plex)	0.393	0.63	0.663	6.14	29	1	2859.59305	4	715.6538696	-2.920927027	38.38213167
														FAGDKGYLTK	3	1	1	N-Term(iTRAQ8plex); K5(iTRAQ8plex); K10(iTRAQ8plex)	0.394	0.626	0.645	3.49	34	1	2012.19524	2	1006.601257	0.435707441	21.31821833
														PSQMEHAMETMMFTFHK	7	1	1	N-Term(iTRAQ8plex); K17(iTRAQ8plex)	0.284	0.373	0.593	-	60	0	2691.32792	3	897.7808228	9.907269779	53.13893833
4506787	IQGAP1	ras GTPase-activating-like protein IQGAP1	NP_003861.1	25.53	30	30	132	1657	189.1	6.48	0.893	1.524	1.796																
														NVIFEISPTEEVGDFEVK	6	1	1	N-Term(iTRAQ8plex); K18(iTRAQ8plex)	0.833	1.787	1.876	7.85	76	0	2660.4188	3	887.4817505	1.013713624	45.52756833
														LEGVLAEVAQHYQDTLIR	20	1	1	N-Term(iTRAQ8plex)	0.99	1.468	1.727	7.41	66	0	2359.2808	3	787.0984497	-4.906337951	83.24403167
														FALGIFAINEAVESGDVGK	8	1	1	N-Term(iTRAQ8plex); K19(iTRAQ8plex)	1.109	2.568	3.105	7.16	59	0	2545.42545	3	849.1389771	-3.898557549	59.00003833
														ILAIGLINEALDEGDAQK	6	1	1	N-Term(iTRAQ8plex); K18(iTRAQ8plex)	-	-	-	6.59	75	0	2491.44301	3	831.1430054	-3.370951258	57.28811667
														FQPGETLTEILETPATSEQEAEHQR	8	1	1	N-Term(iTRAQ8plex)	0.572	0.643	0.368	5.94	57	0	3145.54026	3	1049.184937	-6.022806304	70.87475667
														HTDNVIQWLNAMDEIGLPK	14	1	1	N-Term(iTRAQ8plex); K19(iTRAQ8plex)	0.635	2.036	2.266	5.93	57	0	2802.51432	3	934.8429565	2.63159366	60.958965
														SPDVGLYGVIPECGETYHSDLAEAK	6	1	1	N-Term(iTRAQ8plex); C13(Methylthio); K25(iTRAQ8plex)	0.422	2.014	1.406	5.65	43	0	3304.63987	3	1102.214233	-1.369399063	44.457395
														GVLLEIEDLQVNQFK	5	1	1	N-Term(iTRAQ8plex); K15(iTRAQ8plex)	0.628	1.242	1.509	5.37	84	0	2353.34616	2	1177.182251	-0.661169014	44.35800833

														EKLEAYQHLFYLLQTNPTYLAK	6	1	1	N-Term(iTRAQ8plex); K2(iTRAQ8plex); K22(iTRAQ8plex)	0.705	2.872	2.549	5.21	48	1	3596.0167	3	1199.341553	-5.299302927	53.48586833
														EEIQSSISGVTAAYNR	2	1	1	N-Term(iTRAQ8plex)	0.641	0.667	0.581	4.96	38	0	2029.03965	3	677.0180664	-5.295771954	31.69489333
														LAAVALINAAIQK	2	1	1	N-Term(iTRAQ8plex); K13(iTRAQ8plex)	1.141	1.191	0.918	4.81	29	0	1904.2031	3	635.4058838	-6.429557238	45.82233667
														EQLWLANEGLITR	2	1	1	N-Term(iTRAQ8plex)	1.164	1.461	1.851	4.77	27	0	1847.03398	2	924.0206299	0.585720477	39.39235833
														SKVDQIQEIVTGNPTVIK	2	1	1	N-Term(iTRAQ8plex); K2(iTRAQ8plex); K18(iTRAQ8plex)	1.031	1.709	1.616	4.76	27	1	2881.69736	3	961.2373047	-5.288718971	37.103965
														TLQALQIPAAK	2	1	1	N-Term(iTRAQ8plex); K11(iTRAQ8plex)	0.894	2.131	2.166	4.75	41	0	1762.09819	2	881.5527344	-3.699663293	24.76108833
														VNVNLLIFLLNK	2	7	1	N-Term(iTRAQ8plex); K12(iTRAQ8plex)	0.295	1.225	1.479	4.48	31	0	2008.26761	3	670.0940552	-5.138270713	83.19914833
														ITLQDVVSHSK	6	1	1	N-Term(iTRAQ8plex); K11(iTRAQ8plex)	0.926	1.662	1.928	4.48	67	0	1835.06902	2	918.038147	-8.541354325	28.573185
														KFVHLLDQSDQDFQEELDLMK	2	1	1	N-Term(iTRAQ8plex); K1(iTRAQ8plex); K21(iTRAQ8plex)	0.962	1.631	2.292	4.29	-	1	3490.8531	5	698.9764404	-3.576679173	57.19726667
														RLAAVALINAAIQK	6	1	1	N-Term(iTRAQ8plex); K14(iTRAQ8plex)	0.59	1.572	1.927	4.25	32	1	2060.30124	3	687.4385986	-7.386614757	44.112065
														SNQQLENDLNLMDIK	1	1	1	N-Term(iTRAQ8plex); K15(iTRAQ8plex)	0.685	1.248	1.717	3.84	-	0	2383.25754	3	795.0906982	-7.237918327	33.52831
														ALESGDVNTVWK	2	1	1	N-Term(iTRAQ8plex); K12(iTRAQ8plex)	0.704	1.267	1.613	3.79	49	0	1927.05887	3	643.0244751	-8.131177132	38.92388333
														LPYDVTPEQALAHEEVK	3	1	1	N-Term(iTRAQ8plex); K17(iTRAQ8plex)	0.987	1.917	2.265	3.78	36	0	2547.38193	3	849.7988281	-3.768643752	35.14275167
														HTDNVIQWLNAMDEIGLPK	2	1	1	N-Term(iTRAQ8plex); M12(Oxidation); K19(iTRAQ8plex)	1.9	2.213	2.489	3.57	27	0	2818.49697	3	934.8429565	2.63159366	60.958965
														LGNFFSPK	4	1	1	N-Term(iTRAQ8plex); K8(iTRAQ8plex)	0.785	1.623	1.738	3.48	29	0	1517.87895	3	506.6316528	-8.720090108	31.222575
														RIPADTFAALK	1	1	1	N-Term(iTRAQ8plex); K11(iTRAQ8plex)	0.908	1.091	1.205	3.23	-	1	1811.08866	3	604.3677368	-6.247126962	34.958245
														FDVPGDENAEMDAR	2	1	1	N-Term(iTRAQ8plex)	0.629	0.919	0.989	3.21	22	0	1869.85393	2	935.430603	-2.776615451	28.43856667
														MREEVITLIR	3	1	1	N-Term(iTRAQ8plex)	0.921	0.965	1.202	3.19	32	1	1563.91202	3	521.9755249	-4.750806226	31.72707167
														IFYPETTDIYDRK	1	1	1	N-Term(iTRAQ8plex); K13(iTRAQ8plex)	1.152	2.223	2.495	3.03	-	1	2269.22055	3	757.0783691	-5.256046224	33.62306
														LTELGTVDPK	2	1	1	N-Term(iTRAQ8plex); K10(iTRAQ8plex)	0.734	1.32	1.343	2.96	32	0	1680.98857	2	840.9979248	-6.346450414	24.04261333
														IFYPETTDIYDR	2	1	1	N-Term(iTRAQ8plex)	0.823	1.463	1.548	2.84	30	0	1836.92607	2	918.9666748	-3.308957674	39.03820667
														EQLSDMMMINK	1	1	1	N-Term(iTRAQ8plex); K11(iTRAQ8plex)	0.956	1.332	1.242	2.78	-	0	1947.9979	3	650.0041504	-9.426795658	52.23368
														LEGVLAEVAQHYQDTLIR	1	1	1	N-Term(iTRAQ8plex); Y12(iTRAQ8plex)	-	-	-	-	32	0	2663.49184	3	787.0984497	-4.906337951	83.24403167
														LIVDVIR	2	1	1	N-Term(iTRAQ8plex)	1.124	1.986	2.247	-	21	0	1131.73271	2	566.3706665	-5.535675234	27.867165
4506901	SRSF3	serine/arginine-rich splicing factor 3	NP_003008.1	23.17	4	5	26	164	19.3	11.65	0.897	1.004	0.830																
														NPPGFAFVEFEDPRDAADAVR	16	1	1	N-Term(iTRAQ8plex)	0.945	1.004	0.76	4.84	45	1	2624.30239	4	656.8310547	-0.91164418	42.20958667
														DSCPLDCK	3	1	1	N-Term(iTRAQ8plex); C3(Methylthio); C7(Methylthio); K8(iTRAQ8plex)	1.099	1.161	1.119	3.45	34	0	1580.73454	2	790.8709106	-3.529138372	25.91686
														AFGYYGPLR	4	1	1	N-Term(iTRAQ8plex)	0.838	0.88	0.83	3.07	28	0	1347.731	2	674.3691406	-3.915224909	30.25033
														DAADAVR	1	1	1	N-Term(iTRAQ8plex)	0.845	0.79	0.768	-	23	0	1021.55608	2	511.2816772	-1.882841142	12.31067833
														NPPGFAFVEFEDPR	2	5	2	N-Term(iTRAQ8plex)	-	-	-	3.22	51	0	1925.96697	2	963.4871216	-1.565320688	45.065485
4506903	SRSF9	serine/arginine-rich splicing factor 9	NP_003760.1	31.22	5	6	29	221	25.5	8.65	1.313	1.442	1.108																
														EAGDVCYADVQK	4	1	1	N-Term(iTRAQ8plex); C6(Methylthio); K12(iTRAQ8plex)	1.742	1.867	1.371	4.71	78	0	1951.95732	2	976.4822998	-7.207796226	34.60054833
														HGLVPFAFVR	16	1	1	N-Term(iTRAQ8plex)	1.126	1.315	0.866	4.07	53	0	1446.84967	3	482.9541626	-3.045763778	33.089465
														VLVSGLPPSGSWQDLK	2	1	1	N-Term(iTRAQ8plex); K16(iTRAQ8plex)	0.978	1.011	1.056	3.73	31	0	2291.3071	3	764.4405518	-6.502522752	34.61236833
														DGVGMVEYLR	1	1	1	N-Term(iTRAQ8plex)	1.71	1.457	1.512	3.26	-	0	1442.75957	2	721.8834229	-1.366975425	32.38741167
														FEDPRDAEDAIYGR	3	1	1	N-Term(iTRAQ8plex)	1.413	1.961	1.623	3.24	21	1	1957.95354	2	979.4821777	0.662705411	27.28369833
														LRVEFPR	3	3	2	N-Term(iTRAQ8plex)	-	-	-	-	28	1	1220.73337	3	407.5826416	-6.83994188	22.63803833
4506921	SGTA	small glutamine-rich tetratricopeptide repeat-containing protein alpha	NP_003012.1	4.15	1	1	4	313	34.0	4.87	1.165	2.312	3.024																
														TPPSEEDSAEAER	4	1	1	N-Term(iTRAQ8plex)	1.165	2.312	3.024	3.19	51	0	1721.8073	2	861.4072876	-3.422406614	13.60870667
4507015	SLC31A1	high affinity copper uptake protein 1	NP_001850.1	5.79	1	1	2	190	21.1	7.42	0.979	0.826	1.557																
														AVVVDITEHCH	2	1	1	N-Term(iTRAQ8plex); C10(Methylthio)	0.979	0.826	1.557	3.2	47	0	1572.77597	3	524.9301758	-3.584875623	33.02072833
4507115	FSCN1	fascin	NP_003079.1	24.75	9	9	48	493	54.5	7.24	1.290	1.948	1.668																
														VGKDELFALEQSCAQVVLQAANER	15	1	1	N-Term(iTRAQ8plex); K3(iTRAQ8plex); C13(Methylthio)	-	-	-	6.49	76	1	3272.7396	3	1091.584717	5.116459813	67.92173833
														YLTAEAFGFK	4	1	1	N-Term(iTRAQ8plex); K10(iTRAQ8plex)	1.326	2.567	1.958	5.22	62	0	1754.97942	2	877.9933472	-8.167380151	65.28405667
														SSYDVFQLEFNDGAYNIK	2	1	1	N-Term(iTRAQ8plex); K18(iTRAQ8plex)	3.628	6.825	5.747	5.02	50	0	2718.38285	3	906.7991333	-1.587211184	47.82923
														LSCFAQTVSPAEK	2	1	1	N-Term(iTRAQ8plex); C3(Methylthio); K13(iTRAQ8plex)	0.852	1.093	1.36	4.27	37	0	2035.06437	3	679.0263062	-8.312804871	31.80604
														LVARPEPATGYTLEFR	3	1	1	N-Term(iTRAQ8plex)	0.857	1.211	0.948	4.09	35	0	2124.16298	4	531.7962036	-5.916084108	33.33941
														FLIVAHDDGR	16	1	1	N-Term(iTRAQ8plex)	1.392	2.266	1.419	3.97	69	0	1446.7919	3	482.9354248	-6.206930691	23.55412167
														YLAADKDGNVTCER	3	1	1	N-Term(iTRAQ8plex); K6(iTRAQ8plex); C12(Methylthio)	1.128	1.981	1.729	3.49	26	1	2209.10227	4	553.0304565	-9.142188293	21.75627167
														VTGTLDANR	2	1	1	N-Term(iTRAQ8plex)	1.373	2.845	2.474	3.03	28	0	1250.70659	2	625.8569336	4.756725154	20.44899833
														EVPGPDCR	1	1	1	N-Term(iTRAQ8plex); C7(Methylthio)	0.963	1.668	1.61	-	17	0	1222.58013	2	611.7937012	-4.978468177	19.1343
4507125	SNRPB	small nuclear ribonucleoprotein-associated proteins B and B' isoform B	NP_003082.1	16.45	5	5	40	231	23.6	10.90	0.940	1.033	0.885																
														GENLVSMTVEGPPPK	2	3	1	N-Term(iTRAQ8plex); K15(iTRAQ8plex)	0.855	0.868	0.903	5.96	77	0	2163.20049	2	1082.103882	2.984352081	31.11617167
														HMNLILCDCDEFRK	9	3	1	N-Term(iTRAQ8plex); C7(Methylthio); C9(Methylthio); K14(iTRAQ8plex)	1.623	1.427	1.828	4.11	43	1	2437.16958	4	610.0478516	-3.433052046	43.95879333
														HMNLILCDCDEFR	4	3	1	N-Term(iTRAQ8plex); M2(Oxidation); C7(Methylthio); C9(Methylthio)	1.351	1.456	1.064	3.92	73	0	2020.86618	3	674.2935791	-3.139369327	44.89506667
														RVLGLVLLR	10	3	1	N-Term(iTRAQ8plex)	1.076	1.038	1.215	3.5	48	1	1342.91864	3	448.3092957	-4.987356177	34.055595
														HMNLILCDCDEFR	2	3	1	N-Term(iTRAQ8plex); C7(Methylthio); C9(Methylthio)	0.992	1.028	0.609	3.31	30	0	2004.87522	3	674.2935791	-3.139369327	44.89506667
														VLGLVLLR	13	3	1	N-Term(iTRAQ8plex)	0.792	0.781	0.801	3.12	52	0	1186.81272	3	396.2757568	-5.21905613	42.52096167
4507127	SNRPC	U1 small nuclear ribonucleoprotein C	NP_003084.1	11.32	1	1	3	159	17.4	9.67	1.073	1.238	1.105																
														FYCDYCDTYLTHDSPSVR	2	1	1	N-Term(iTRAQ8plex); C3(Methylthio); C6(Methylthio)	0.751	1.076	1.001	6.09	47	0	2581.07663	3	861.0303955	-1.677604805	51.51687
														FYCDYCDTYLTHDSPSVR	1	1	1	N-Term(iTRAQ8plex); C3(Methylthio); C6(Methylthio); Y9(iTRAQ8plex)	1.534	1.425	1.22	-	14	0	2885.31093	3	861.0303955	-1.677604805	51.51687
4507129	SNRPE	small nuclear ribonucleoprotein E isoform 1	NP_003085.1	25.00	2	2	20	92	10.8	9.44	1.001	0.914	0.965																
														IQVWLYEQVNMR	11	2	1	N-Term(iTRAQ8plex)	1.001	1.07	1.068	4.25	50	0	1883.01494	2	942.0111084	-0.101498464	45.65185167
														VMVQPINLIFR	6	1	1	N-Term(iTRAQ8plex)	1.019	0.782	0.91	3.89	41	0	1633.97074	3	545.3284302	-3.570457244	53.662425
														IQVWLYEQVNMR	3	2	1	N-Term(iTRAQ8plex); M11(Oxidation)	0.731	0.817	0.975	3.02	46	0	1899.00418	2	942.0111084	-0.101498464	45.65185167
4507131	SNRPF	small nuclear ribonucleoprotein F	NP_003086.1	24.42	2	2	16	86	9.7	4.67	0.873	0.763	0.940																
														GVEEEEDGEMRE	8	1	1	N-Term(iTRAQ8plex)	0.911	0.664	0.66	5.39	63	1	1841.81559	3	614.6100464	7.739433994	20.72849667
														GVEEEEDGEMRE	4	1	1	N-Term(iTRAQ8plex); M11(Oxidation)	0.967	0.742	0.961	4.23	50	1	1857.79428	3	614.6100464	7.739433994	20.72849667
														CNNVLYIR	4	1	1	N-Term(iTRAQ8plex); C1(Methylthio)	0.759	0.969	0.987	3.1	22	0	1344.71135	2	672.8533936	-5.549054318	25.46844667
4507185	SPR	sepiapterin reductase	NP_003115.1	4.21	1	1	2	261	28.0	8.05	-	2.705	4.671																
														LLSLLEKDEFK	2	1	1	N-Term(iTRAQ8plex); K7(iTRAQ8plex); K11(iTRAQ8plex)	-	2.705	4.671	4.76	-	1	2247.35366	4	562.5938721	-8.491275362	50.20155
4507217	SRP9	signal recognition particle 9 kDa protein isoform 2	NP_003124.1	25.58	2	2	8	86	10.1	7.97	1.177	1.514	1.420																
														VTDDLVCLVYK	4	1	1	N-Term(iTRAQ8plex); C7(Methylthio); K11(iTRAQ8plex)	0.812	1.055	0.914	3.9	48	0	1922.05661	3	641.3570557	-1.107437626	46.29404
														PQYQTWEEFSR	4	2	1	N-Term(iTRAQ8plex)	1.184	1.983	1.561	-	53	0	1774.87663	2	887.9419556	3.613328773	32.07660333
4507241	SSRP1	FACT complex subunit SSRP1	NP_003137.1	14.53	9	9	24	709	81.0	6.87	1.454	2.242	1.426																
														VDNIQAGELTEGIWR	2	3	1	N-Term(iTRAQ8plex)	1.815	2.929	1.914	5.21	48	0	2005.08061	2	1003.043945	7.452421063	39.52111167
														GFIYVHKPPVHIR	1	3	1	N-Term(iTRAQ8plex); K7(iTRAQ8plex)	-	-	-	4.3	-	0	2171.28904	5	435.0636292	-7.904087266	27.610805
														FDEISFVNFAR	6	3	1	N-Term(iTRAQ8plex)	1.496	2.254	1.472	4.15	42	0	1648.86601	2	824.9366455	1.402840258	45.66393167
														RPMSAYMLWLNASR	3	2	1	N-Term(iTRAQ8plex)	1.183	1.011	0.821	4.04	-	0	2000.05167	3	667.3554077	0.224291471	40.59715
														GWNWGTVK	2	3	1	N-Term(iTRAQ8plex); K8(iTRAQ8plex)	1.576	2.619	1.755	3.89	29	0	1555.87236	2	778.4398193	-7.570831909	29.154945
														ASSGLLYPLER	4	3	1	N-Term(iTRAQ8plex)	0.685	1.036	0.615	3.81	31	0	1509.85161	2	755.4294434	-4.146898416	29.04640167
														IYPTFLHLHGK	2	3	1	N-Term(iTRAQ8plex); K11(iTRAQ8plex)	2.146	4.334	2.697	3.79	-	0	1934.13784	4	484.289917	-4.851366363	32.44731833
														QMFFVISLDPPIK	3	3	1	N-Term(iTRAQ8plex); K13(iTRAQ8plex)	1.271	1.768	1.686	3.54	29	0	2143.23429	3	715.0829468	-4.812454724	45.47843
														LFLLPHK	1	3	1	N-Term(iTRAQ8plex); K7(iTRAQ8plex)	2.15	3.099	1.83	-	24	0	1475.95557	3	492.6567078	-0.183785332	31.27594333
4507357	TAGLN2	transgelin-2 isoform b	NP_003555.1	34.67	5	5	34	199	22.4	8.25	0.694	3.129	2.201																
														YGINTTDIFQTVDLWEGK	19	2	1	N-Term(iTRAQ8plex); K18(iTRAQ8plex)	0.519	6.319	4.714	7.12	60	0	2708.44626	3	903.4869385	2.603293182	55.63007333
														QMEQISQFLQAAER	4	2	1	N-Term(iTRAQ8plex)	1.2	2.6	1.707	6.95	65	0	1983.04117	3	661.6852417	7.060139676	47.262935
														TLMNLGGLAVAR	6	2	1	N-Term(iTRAQ8plex)	0.662	3.041	2.201	3.77	67	0	1519.88896	3	507.3005676	-4.007974633	40.65756833
														NFSDNQLQEGK	2	2	1	N-Term(iTRAQ8plex); K11(iTRAQ8plex)	1.307	1.214	1.137	3.63	30	0	1887.99479	3	630.0031128	-3.8698047	19.714775
														DDGLFSGDPNWFPK	2	2	1	N-Term(iTRAQ8plex); K14(iTRAQ8plex)	0.504	1.4	1.125	2.71	27	0	2203.11802	3	735.0441895	-4.555448485	39.13971
														QMEQISQFLQAAER	1	2	1	N-Term(iTRAQ8plex); M2(Oxidation)	0.551	1.521	1.158	-	21	0	1999.01909	3	661.6852417	7.060139676	47.262935
4507401	TFAM	transcription factor A, mitochondrial isoform 1 precursor	NP_003192.1	14.63	3	3	10	246	29.1	9.72	1.194	0.927	0.619																
														FKEQLTPSQIMSLEK	2	4	1	N-Term(iTRAQ8plex); K2(iTRAQ8plex); M11(Oxidation); K15(iTRAQ8plex)	1.755	1.027	0.646	4.28	33	1	2707.53861	3	903.1843872	-3.016735895	30.29072333
														SWEEQMIEVGR	6	2	1	N-Term(iTRAQ8plex)	1.116	0.875	0.552	4.1	60	0	1667.82915	2	834.416626	-6.317185984	26.47934833
														SAYNVYVAER	2	1	1	N-Term(iTRAQ8plex)	1.337	0.927	0.619	3.73	36	0	1475.7741	2	738.390686	-3.730821475	17.71983833
4507445	TFAP2C	transcription factor AP-2 gamma	NP_003213.1	1.56	1	1	3	450	49.1	7.83	1.601	9.048	3.990																
														TLEKMEK	3	1	1	N-Term(iTRAQ8plex); K4(iTRAQ8plex); M5(Oxidation); K7(iTRAQ8plex)	1.601	9.048	3.99	2.91	-	1	1807.08278	4	452.5261536	3.629096503	16.82705833
4507467	TGFBI	transforming growth factor-beta-induced protein ig-h3 precursor	NP_000349.1	11.13	6	6	19	683	74.6	7.71	0.377	0.254	0.356																
														EGVYTVFAPTNEAFR	2	1	1	N-Term(iTRAQ8plex)	0.288	0.109	0.088	4.33	43	0	2005.03386	2	1003.020569	0.284172414	41.33873833
														GDELADSALEIFK	8	1	1	N-Term(iTRAQ8plex); K13(iTRAQ8plex)	0.389	0.333	0.36	4.22	35	0	2016.10343	3	672.7059937	-3.75654982	70.80941
														LTLLAPLNSVFK	2	1	1	N-Term(iTRAQ8plex); K12(iTRAQ8plex)	0.209	0.046	-	4.04	36	0	1924.21909	3	642.0778809	5.149116429	54.766635
														SPYQLVLQHSR	1	1	1	N-Term(iTRAQ8plex)	0.474	0.492	0.38	3.57	-	0	1631.91098	3	544.6418457	-3.748636222	26.11994167
														DGTPPIDAHTR	4	1	1	N-Term(iTRAQ8plex)	0.239	0.058	0.144	3.55	34	0	1483.78525	2	742.3936157	-0.496350342	11.14138167
														YLYHGQTLETLGGK	2	1	1	N-Term(iTRAQ8plex); K14(iTRAQ8plex)	0.602	0.503	0.496	3.45	28	0	2188.2222	3	730.0789185	-0.017904446	29.030595
4507555	TMPO	thymopoietin isoform alpha	NP_003267.1	26.51	6	12	47	694	75.4	7.66	1.447	1.247	1.720																
														AYEAAASALQIATHTAFVAK	4	2	1	N-Term(iTRAQ8plex); K20(iTRAQ8plex)	-	-	-	6.49	40	0	2642.47573	4	661.3743896	-0.205332539	57.57963667
														TYDAASYICEAAFDEVK	4	2	1	N-Term(iTRAQ8plex); C9(Methylthio); K17(iTRAQ8plex)	4.695	4.51	6.77	4.09	65	0	2550.24468	2	1275.625977	3.683648509	59.13067667
														VIEEEWQQVDR	2	2	1	N-Term(iTRAQ8plex)	1.613	1.692	1.964	3.27	27	0	1734.88176	2	867.944519	-8.473104271	30.82577167
														DIVENICGR	3	2	1	N-Term(iTRAQ8plex); C7(Methylthio)	1.442	1.92	2.118	3.09	26	0	1368.69536	2	684.8466797	-4.135020492	30.79130833
														MAAHTMGNATVGR	3	2	1	N-Term(iTRAQ8plex); M6(Oxidation)	1.269	0.93	1.531	2.99	31	0	1636.81748	3	546.2773438	-1.636285069	10.46363
														SGIQPLCPER	1	2	1	N-Term(iTRAQ8plex); C7(Methylthio)	0.902	1.011	1.358	-	21	0	1449.74443	2	725.3758545	-3.559203615	26.91410333
														DVYVQLYLQHLTAR	16	5	2	N-Term(iTRAQ8plex)	-	-	-	6.06	73	0	2023.12387	3	675.0466919	-1.141237348	45.605305
														QEDKDDLDVTELTNEDLLDQLVK	2	5	2	N-Term(iTRAQ8plex); K4(iTRAQ8plex); K23(iTRAQ8plex)	-	-	-	5.39	76	1	3600.92685	3	1200.980469	0.236522439	44.28536167
														SSTPLPTISSSAENTR	2	5	2	N-Term(iTRAQ8plex)	-	-	-	3.95	56	0	1952.01531	2	976.5112915	-4.372359253	22.43312333
														GPPDFSSDEEREPTPVLGSGAAAAGR	3	5	2	N-Term(iTRAQ8plex)	-	-	-	3.72	24	1	2874.42423	3	958.8129272	2.423855259	27.84742

														YGVNPGPIVGTTR	6	5	2	N-Term(iTRAQ8plex)	-	-	-	3.21	42	0	1634.91057	2	817.9589233	-3.805248083	20.54155333
														PEFLEDPSVLTK	1	5	2	N-Term(iTRAQ8plex); K12(iTRAQ8plex)	-	-	-	-	41	0	1983.10893	2	992.0581055	-8.560791697	31.588345
4507645	TPI1	triosephosphate isomerase isoform 1	NP_000356.1	81.53	15	15	212	249	26.7	6.90	0.931	3.561	3.131																
														ELASQPDVDGFLVGGASLKPEFVDIINAK	18	3	1	N-Term(iTRAQ8plex); K19(iTRAQ8plex); K29(iTRAQ8plex)	-	6.76	4.827	6.49	35	0	3942.21084	4	986.3018188	-3.499528261	62.60362833
														VVLAYEPVWAIGTGK	33	3	1	N-Term(iTRAQ8plex); K15(iTRAQ8plex)	0.836	3.643	3.9	6.43	76	0	2211.28911	2	1106.149658	-3.509945141	46.302235
														VAHALAEGLGVIACIGEK	33	3	1	N-Term(iTRAQ8plex); C14(Methylthio); K18(iTRAQ8plex)	1.748	25.196	22.981	5.91	122	0	2405.34282	2	1203.175049	-3.218361924	65.10098333
														DCGATWVVLGHSER	38	3	1	N-Term(iTRAQ8plex); C2(Methylthio)	0.941	2.361	2.822	5.66	74	0	1879.90129	2	940.4542847	-4.468098456	30.87100333
														IIYGGSVTGATCK	14	3	1	N-Term(iTRAQ8plex); C12(Methylthio); K13(iTRAQ8plex)	0.779	3.61	3.565	5.61	81	0	1924.03276	2	962.5200195	-8.565268649	29.44871833
														HVFGESDELIGQK	12	3	1	N-Term(iTRAQ8plex); K13(iTRAQ8plex)	0.725	2.14	2.195	5.23	67	0	2067.12334	2	1034.065308	-4.732511816	24.61684667
														TATPQQAQEVHEK	6	3	1	N-Term(iTRAQ8plex); K13(iTRAQ8plex)	1.067	2.688	2.715	4.97	36	0	2075.12253	3	692.3790283	-5.609109286	13.92380167
														QSLGELIGTLNAAK	6	2	1	N-Term(iTRAQ8plex); K14(iTRAQ8plex)	0.59	1.382	1.487	4.74	51	0	2023.18137	3	675.0653076	-9.609468894	68.82613833
														IAVAAQNCYK	8	2	1	N-Term(iTRAQ8plex); C8(Methylthio); K10(iTRAQ8plex)	0.967	2.105	2.133	4.63	52	0	1734.94158	2	867.9744263	-4.349645569	26.50609
														VTNGAFTGEISPGMIK	2	2	1	N-Term(iTRAQ8plex); K16(iTRAQ8plex)	-	10.191	9.449	4.61	35	0	2230.2222	3	744.0789185	-6.290484819	27.91531
														SNVSDAVAQSTR	12	3	1	N-Term(iTRAQ8plex)	0.933	4.461	3.202	4.59	70	0	1538.8029	2	769.9050903	-3.072034713	16.4493
														KQSLGELIGTLNAAK	4	2	1	N-Term(iTRAQ8plex); K1(iTRAQ8plex); K15(iTRAQ8plex)	1.823	6.117	4.482	4.54	53	1	2455.49283	3	819.1691284	-3.383962676	49.20341667
														VPADTEVVCAPPTAYIDFAR	13	2	1	N-Term(iTRAQ8plex); C9(Methylthio)	1.538	5.251	4.886	4.23	59	0	2485.23589	2	1243.121582	-2.037978578	52.81257
														FFVGGNWK	10	2	1	N-Term(iTRAQ8plex); K8(iTRAQ8plex)	1.129	3.801	3.526	3.34	35	0	1562.87986	3	521.6326904	-6.69484374	36.17890667
														VIADNVK	1	3	1	N-Term(iTRAQ8plex); K7(iTRAQ8plex)	1.055	3.02	3.04	-	32	0	1366.83843	2	683.9228516	-9.521824049	26.44532667
														VPADTEVVCAPPTAYIDFAR	1	2	1	N-Term(iTRAQ8plex); C9(Methylthio); Y15(iTRAQ8plex)	-	3.882	3.748	-	30	0	2789.43692	2	1243.121582	-2.037978578	52.81257
														VVLAYEPVWAIGTGK	1	3	1	N-Term(iTRAQ8plex); Y5(iTRAQ8plex); K15(iTRAQ8plex)	1.102	3.251	3.135	-	23	0	2515.49045	2	1106.149658	-3.509945141	46.302235
4507651	TPM4	tropomyosin alpha-4 chain isoform Tpm4.2cy	NP_003281.1	29.44	3	10	75	248	28.5	4.69	1.063	1.384	1.800																
														CGDLEEELKNVTNNLK	3	7	1	N-Term(iTRAQ8plex); C1(Methylthio); K9(iTRAQ8plex); K16(iTRAQ8plex)	-	-	-	5.24	26	1	2777.47695	4	695.1246948	-6.172106353	56.88946
														IQALQQQADEAEDR	4	2	1	N-Term(iTRAQ8plex)	0.862	0.868	1.329	5.19	75	0	1918.98747	2	959.9973755	5.332529107	31.35937333
														LVILEGELERAEER	1	7	1	N-Term(iTRAQ8plex)	1.126	2.007	1.8	2.86	-	1	1960.09769	3	654.0374146	-2.061579724	51.82589
														LVILEGELER	4	13	2	N-Term(iTRAQ8plex)	-	-	-	3.79	38	0	1474.87944	2	737.9433594	0.780287505	44.75856667
														KLVILEGELER	10	13	2	N-Term(iTRAQ8plex); K1(iTRAQ8plex)	-	-	-	3.44	36	1	1907.16794	3	636.3933105	-6.943765614	47.02894833
														CGDLEEELK	1	9	2	N-Term(iTRAQ8plex); C1(Methylthio); K9(iTRAQ8plex)	-	-	-	-	34	0	1689.84783	2	845.4275513	-10.04610041	51.86537167
														HIAEEADR	6	16	2	N-Term(iTRAQ8plex)	-	-	-	2.8	46	0	1244.6453	3	415.5538025	-5.493188107	10.93135
														IQLVEEELDR	29	47	4	N-Term(iTRAQ8plex)	-	-	-	3.76	44	0	1547.85076	2	774.4290161	-4.862027822	39.66570333
														IQLVEEELDRAQER	14	47	4	N-Term(iTRAQ8plex)	-	-	-	3.33	49	1	2032.09641	2	1016.549255	-3.182523355	46.83701
														LEEAEK	3	49	5	N-Term(iTRAQ8plex); K6(iTRAQ8plex)	-	-	-	2.83	28	0	1326.76068	2	663.8832397	-10.03831576	13.15593833
4507669	TPT1	translationally-controlled tumor protein isoform 2	NP_003286.1	15.70	2	2	20	172	19.6	4.93	1.289	2.898	2.533																
														DLISHDEMFSDIYK	10	2	1	N-Term(iTRAQ8plex); K14(iTRAQ8plex)	1.207	2.649	1.803	5.59	63	0	2321.19048	2	1161.098877	-1.69051777	36.20710333
														EIADGLCLEVEGK	8	2	1	N-Term(iTRAQ8plex); C7(Methylthio); K13(iTRAQ8plex)	1.24	3.345	2.634	5.56	73	0	2030.07341	2	1015.540344	-1.216923202	42.65891333
														DLISHDEMFSDIYK	2	2	1	N-Term(iTRAQ8plex); M8(Oxidation); K14(iTRAQ8plex)	1.43	3.057	2.651	4.13	31	0	2337.17759	2	1161.098877	-1.69051777	36.20710333
4507677	HSP90B1	endoplasmin precursor	NP_003290.1	41.59	30	32	202	803	92.4	4.84	0.862	0.709	0.705																
														LISLTDENALSGNEELTVK	4	1	1	N-Term(iTRAQ8plex); K19(iTRAQ8plex)	0.943	1.387	1.108	5.52	57	0	2654.45839	4	664.3700562	-4.719068361	41.87760333
														LTESPCALVASQYGWSGNMER	6	1	1	N-Term(iTRAQ8plex); C6(Methylthio)	1.367	1.234	0.876	5.48	72	0	2649.23027	2	1325.118774	-4.186156255	56.31245167
														DISTNYYASQK	6	1	1	N-Term(iTRAQ8plex); K11(iTRAQ8plex)	0.805	0.972	0.809	5.21	43	0	1898.0114	2	949.5093384	-0.084911075	17.69473333
														VFITDDFHDMMPK	10	1	1	N-Term(iTRAQ8plex); K13(iTRAQ8plex)	0.801	0.682	0.704	4.99	53	0	2204.12505	2	1102.566162	-4.094006037	39.76363
														LGVIEDHSNR	16	1	1	N-Term(iTRAQ8plex)	0.976	0.825	0.761	4.8	52	0	1443.77973	3	481.9343262	1.838195666	25.00531667
														GYEVIYLTEPVDEYCIQALPEFDGKR	13	1	1	N-Term(iTRAQ8plex); C15(Methylthio); K25(iTRAQ8plex)	0.947	0.426	0.8	4.6	37	1	3701.87832	4	926.2250366	2.456672964	65.35690833
														TDDEVVQREEEAIQLDGLNASQIR	10	1	1	N-Term(iTRAQ8plex)	0.706	0.649	0.595	4.59	66	1	3032.53642	2	1516.771851	-2.465509751	40.40364
														YSQFINFPIYVWSSK	5	1	1	N-Term(iTRAQ8plex); K15(iTRAQ8plex)	1.149	0.824	0.941	4.54	34	0	2487.35392	3	829.7894897	0.263206726	51.92092167
														EFEPLLNWMK	6	1	1	N-Term(iTRAQ8plex); K10(iTRAQ8plex)	0.647	0.481	0.477	4.47	60	0	1915.05242	2	958.0340576	0.014262928	44.257
														GVVDSDDLPLNVSR	7	1	1	N-Term(iTRAQ8plex)	0.798	0.66	0.624	4.47	56	0	1789.9605	2	895.4838867	0.388565838	30.48391667
														TVWDWELMNDIKPIWQRPSK	1	1	1	N-Term(iTRAQ8plex); M8(Oxidation); K12(iTRAQ8plex); K20(iTRAQ8plex)	0.937	0.372	0.29	4.34	-	0	3470.89948	5	694.9857178	-0.796318887	44.66209333
														FAFQAEVNR	12	1	1	N-Term(iTRAQ8plex)	0.715	0.552	0.595	4.2	59	0	1385.74272	3	462.5882874	1.71568466	29.12918
														NLLHVTDTGVGMTR	18	1	1	N-Term(iTRAQ8plex)	0.855	0.775	0.777	4.06	57	0	1817.98064	2	909.4939575	-2.163323885	26.608765
														EAVEKEFEPLLNWMK	7	1	1	N-Term(iTRAQ8plex); K5(iTRAQ8plex); K15(iTRAQ8plex)	1.021	0.831	0.782	3.88	52	1	2775.54668	2	1388.276978	-1.874528688	56.82816833
														SGTSEFLNK	2	1	1	N-Term(iTRAQ8plex); K9(iTRAQ8plex)	0.982	0.766	0.71	3.83	41	0	1590.89629	2	795.9517822	0.959542829	16.02359667
														LIINSLYK	3	1	1	N-Term(iTRAQ8plex); K8(iTRAQ8plex)	0.933	0.492	0.576	3.8	35	0	1571.99138	2	786.4993286	-4.268912362	50.09686667
														NLLHVTDTGVGMTR	2	1	1	N-Term(iTRAQ8plex); M12(Oxidation)	0.593	0.74	0.555	3.79	-	0	1833.97739	2	909.4939575	-2.163323885	26.608765
														SGYLLPDTK	4	1	1	N-Term(iTRAQ8plex); K9(iTRAQ8plex)	0.709	0.572	0.569	3.75	37	0	1601.92876	2	801.4680176	-4.452609838	21.12270833
														EVEEDEYK	5	1	1	N-Term(iTRAQ8plex); K8(iTRAQ8plex)	0.876	0.698	0.633	3.58	52	0	1648.84722	2	824.9272461	-3.278492057	14.77191667
														KYSQFINFPIYVWSSK	1	1	1	N-Term(iTRAQ8plex); K1(iTRAQ8plex); K16(iTRAQ8plex)	-	-	-	3.39	-	1	2919.65688	4	730.6696777	1.126761683	56.31914833
														NLGTIAK	3	1	1	N-Term(iTRAQ8plex); K7(iTRAQ8plex)	0.966	0.782	0.647	3.33	26	0	1324.84441	2	662.9258423	2.669483519	19.690395
														GYEVIYLTEPVDEYCIQALPEFDGK	2	1	1	N-Term(iTRAQ8plex); C15(Methylthio); K25(iTRAQ8plex)	-	-	-	3.31	26	0	3545.78391	3	1182.599487	4.457791132	67.21458833
														TVWDWELMNDIKPIWQRPSK	1	1	1	N-Term(iTRAQ8plex); K12(iTRAQ8plex); K20(iTRAQ8plex)	0.856	0.899	0.852	3.28	-	0	3454.89951	5	694.9857178	-0.796318887	44.66209333
														EFGTNIK	7	1	1	N-Term(iTRAQ8plex); K7(iTRAQ8plex)	0.956	0.867	0.746	3.25	35	0	1416.83265	2	708.9199829	1.403059322	18.01490833
														IYFMAGSSR	1	1	1	N-Term(iTRAQ8plex)	0.726	0.655	0.645	3.22	-	0	1335.70256	3	451.2400208	5.409984505	26.49952333
														KEAESSPFVER	3	1	1	N-Term(iTRAQ8plex); K1(iTRAQ8plex)	0.802	0.721	0.637	3.16	24	1	1887.03971	3	629.6844482	-2.35809855	15.48246167
														YLNFVK	6	1	1	N-Term(iTRAQ8plex); K6(iTRAQ8plex)	0.964	0.774	0.776	3.05	24	0	1391.85491	2	696.4302979	1.887974177	27.66688167
														SILFVPTSAPR	4	1	1	N-Term(iTRAQ8plex)	0.724	0.758	0.765	2.99	32	0	1491.88483	3	497.9664612	0.756757425	33.73997333
														EAESSPFVER	2	1	1	N-Term(iTRAQ8plex)	0.862	0.618	0.609	2.91	26	0	1454.74004	2	727.8736572	-1.975244236	19.14019833
														ETLQQHK	1	1	1	N-Term(iTRAQ8plex); K7(iTRAQ8plex)	1.273	0.732	0.71	2.84	-	0	1491.8754	3	497.9633179	0.971303154	11.24284
														IYFMAGSSR	2	1	1	N-Term(iTRAQ8plex); M4(Oxidation)	0.864	0.561	0.586	2.81	26	0	1351.70551	3	451.2400208	5.409984505	26.49952333
														TFEINPR	1	1	1	N-Term(iTRAQ8plex)	0.68	0.523	0.55	-	25	0	1180.66472	2	590.8359985	1.633952704	21.222295
														GTTITLVLK	1	1	1	N-Term(iTRAQ8plex); K9(iTRAQ8plex)	0.754	0.709	0.744	-	25	0	1554.01023	3	518.6749268	1.001102445	40.40028
														VFITDDFHDMMPK	1	1	1	N-Term(iTRAQ8plex); M10(Oxidation); K13(iTRAQ8plex)	-	-	-	-	24	0	2220.12417	2	1102.566162	-4.094006037	39.76363
														ELISNASDALDK	8	4	2	N-Term(iTRAQ8plex); K12(iTRAQ8plex)	-	-	-	5.22	89	0	1884.04155	2	942.5244141	-6.32671556	24.70796833
														ELISNASDALDKIR	21	4	2	N-Term(iTRAQ8plex); K12(iTRAQ8plex)	-	-	-	5.02	66	1	2153.23979	2	1077.123535	0.525901715	38.22399833
4507709	TSTA3	GDP-L-fucose synthase isoform 2	NP_003304.1	9.03	2	2	3	321	35.9	6.60	1.021	2.397	1.871																
														NVHMNDNVLHSAFEVGAR	1	2	1	N-Term(iTRAQ8plex)	1.167	1.305	1.302	3.37	-	0	2314.15775	3	772.0574341	-3.769105157	27.26689333
														DADLTDTAQTR	2	2	1	N-Term(iTRAQ8plex)	0.894	4.401	2.689	3.27	38	0	1510.76055	2	755.8839111	-3.015470842	18.60779833
4507711	TTC1	tetratricopeptide repeat protein 1	NP_003305.1	13.01	2	2	4	292	33.5	4.84	0.542	0.739	0.590																
														VTDTQEAECAGPPVPDPK	2	1	1	N-Term(iTRAQ8plex); C9(Methylthio); K18(iTRAQ8plex)	0.338	0.406	0.342	5.62	49	0	2508.26822	3	836.7609253	4.429481985	28.97029167
														SNEDVNSSELDEEYLIELEK	2	1	1	N-Term(iTRAQ8plex); K20(iTRAQ8plex)	0.868	1.347	1.019	5.59	28	0	2963.48856	3	988.5010376	1.875574296	39.40280667
4507729	TUBB2A	tubulin beta-2A chain isoform 1	NP_001060.1	48.99	1	17	424	445	49.9	4.89	0.426	1.314	1.498																
														INVYYNEAAGNK	2	1	1	N-Term(iTRAQ8plex); K12(iTRAQ8plex)	0.426	1.314	1.498	3.93	31	0	1964.05204	3	655.3555298	-9.02480902	35.63777333
														AILVDLEPGTMDSVR	10	7	3	N-Term(iTRAQ8plex)	-	-	-	4.45	57	0	1920.02982	2	960.5213623	-3.121458321	44.53172
														AILVDLEPGTMDSVR	4	7	3	N-Term(iTRAQ8plex); M11(Oxidation)	-	-	-	2.95	45	0	1936.028	2	960.5213623	-3.121458321	44.53172
														EIVHIQAGQCGNQIGAK	24	9	4	N-Term(iTRAQ8plex); C10(Methylthio); K17(iTRAQ8plex)	-	-	-	6.74	73	0	2420.28691	2	1210.647095	-5.366911999	28.04771167
														LHFFMPGFAPLTSR	105	21	4	N-Term(iTRAQ8plex)	-	-	-	5.26	73	0	1925.03666	3	642.350647	-1.861590873	50.37672667
														LHFFMPGFAPLTSR	9	21	4	N-Term(iTRAQ8plex); M5(Oxidation)	-	-	-	4.68	37	0	1941.02982	3	642.350647	-1.861590873	50.37672667
														NSSYFVEWIPNNVK	31	20	5	N-Term(iTRAQ8plex); K14(iTRAQ8plex)	-	-	-	4.24	49	0	2305.22641	2	1153.123413	-1.809680962	42.78834
														FPGQLNADLR	9	25	5	N-Term(iTRAQ8plex)	-	-	-	4.12	55	0	1434.7963	3	478.9369507	-3.068379269	31.15012333
														LAVNMVPFPR	13	25	5	N-Term(iTRAQ8plex)	-	-	-	4.01	51	0	1447.83405	3	483.2828674	-3.931565971	41.77579833
														LAVNMVPFPR	4	25	5	N-Term(iTRAQ8plex); M5(Oxidation)	-	-	-	3.45	44	0	1463.82685	3	483.2828674	-3.931565971	41.77579833
														KLAVNMVPFPR	15	25	5	N-Term(iTRAQ8plex); K1(iTRAQ8plex)	-	-	-	3.44	37	1	1880.12295	4	470.7861938	-9.108866745	31.42844
														FPGQLNADLRK	1	25	5	N-Term(iTRAQ8plex); K11(iTRAQ8plex)	-	-	-	3.07	-	1	1867.09755	4	467.5298462	-1.861935048	33.46670167
														NMMAACDPR	3	23	5	N-Term(iTRAQ8plex); C6(Methylthio)	-	-	-	-	55	0	1358.59062	2	679.7989502	-6.255401168	21.43194333
														NMMAACDPR	5	23	5	N-Term(iTRAQ8plex); M3(Oxidation); C6(Methylthio)	-	-	-	-	47	0	1374.58525	2	679.7989502	-6.255401168	21.43194333
														NMMAACDPR	1	23	5	N-Term(iTRAQ8plex); M2(Oxidation); M3(Oxidation); C6(Methylthio)	-	-	-	-	34	0	1390.5822	2	679.7989502	-6.255401168	21.43194333
														LTTPTYGDLNHLVSATMSGVTTCLR	95	12	3	N-Term(iTRAQ8plex); C23(Methylthio)	-	-	-	7.13	75	0	3001.51689	2	1501.262085	2.319161318	66.15877833
														SGPFGQIFRPDNFVFGQSGAGNNWAK	9	12	3	N-Term(iTRAQ8plex); K26(iTRAQ8plex)	-	-	-	5.17	30	0	3406.74965	4	852.442749	-1.486779919	46.41363167
														LTTPTYGDLNHLVSATMSGVTTCLR	14	12	3	N-Term(iTRAQ8plex); M17(Oxidation); C23(Methylthio)	-	-	-	5	31	0	3017.50541	2	1501.262085	2.319161318	66.15877833
														EVDEQMLNVQNK	4	9	3	N-Term(iTRAQ8plex); K12(iTRAQ8plex)	-	-	-	4.36	65	0	2055.08745	2	1028.047363	-6.160982347	33.78303333
														EVDEQMLNVQNK	2	9	3	N-Term(iTRAQ8plex); M6(Oxidation); K12(iTRAQ8plex)	-	-	-	4.18	46	0	2071.07792	2	1028.047363	-6.160982347	33.78303333
														TAVCDIPPR	12	17	3	N-Term(iTRAQ8plex); C4(Methylthio)	-	-	-	2.88	36	0	1321.68877	2	661.3473511	-2.713782013	26.90244667
														IREEYPDR	3	13	3	N-Term(iTRAQ8plex)	-	-	-	-	29	1	1381.73252	3	461.2490234	-3.788169878	16.81976833
														GHYTEGAELVDSVLDVVR	51	11	4	N-Term(iTRAQ8plex)	-	-	-	5.05	98	0	2263.18999	2	1132.098633	1.214084992	52.94817667
														GHYTEGAELVDSVLDVVR	10	11	4	N-Term(iTRAQ8plex); Y3(iTRAQ8plex)	-	-	-	-	64	0	2567.37212	2	1132.098633	1.214084992	52.94817667
														MSATFIGNSTAIQELFKR	2	4	2	N-Term(iTRAQ8plex); K17(iTRAQ8plex)	-	-	-	4.35	29	1	2622.45424	4	656.3690186	0.306216496	50.822585
4507793	UBE2N	ubiquitin-conjugating enzyme E2 N	NP_003339.1	30.26	3	3	17	152	17.1	6.57	0.993	1.883	2.404																
														YFHVVIAGPQDSPFEGGTFK	5	1	1	N-Term(iTRAQ8plex); K20(iTRAQ8plex)	0.981	2.255	2.925	6.97	61	0	2804.46889	3	935.4934082	-7.548653864	43.01445
														LELFLPEEYPMAAPK	4	1	1	N-Term(iTRAQ8plex); K15(iTRAQ8plex)	0.931	2.747	2.897	3.99	55	0	2356.32207	2	1178.664673	5.830180562	52.97861167
														TNEAQAIETAR	3	1	1	N-Term(iTRAQ8plex)	0.913	2.109	2.167	3.59	43	0	1507.7935	2	754.4003906	-5.515788451	15.07875667
														LELFLPEEYPMAAPK	5	1	1	N-Term(iTRAQ8plex); M11(Oxidation); K15(iTRAQ8plex)	1.237	1.442	2.478	3.16	34	0	2372.30289	2	1178.664673	5.830180562	52.97861167
4507835	UMPS	uridine 5'-monophosphate synthase	NP_000364.1	2.08	1	1	2	480	52.2	7.24	1.826	3.753	1.708																
														CHEFLIFEDR	2	1	1	N-Term(iTRAQ8plex); C1(Methylthio)	1.826	3.753	1.708	3.75	45	0	1658.7916	3	553.6020508	-3.420083785	41.35623333
4507873	VBP1	prefoldin subunit 3 isoform 1	NP_003363.1	25.89	4	4	10	197	22.6	7.11	1.104	3.265	3.655																
														LHLGIPEAVFVEDVDSFMK	4	2	1	N-Term(iTRAQ8plex); K19(iTRAQ8plex)	-	4.32	3.659	5.46	57	0	2754.50791	3	918.8408203	2.964735544	63.93540333
														NLDNLKEDLDFLR	2	4	1	N-Term(iTRAQ8plex)	-	-	-	4.27	64	0	[illegible]	3	[illegible]	[illegible]	[illegible]
														FLLADNLYCK	2	4	1	N-Term(iTRAQ8plex); C9(Methylthio); K10(iTRAQ8plex)	0.773	1.854	2.06	3.95	35	0	[illegible]	3	[illegible]	[illegible]	[illegible]

														FMELNLAQK	2	4	1	N-Term(iTRAQ8plex); K9(iTRAQ8plex)	1.581	3.265	3.655	3.46	30	0	1701.97281	3	567.9957886	-5.293167598	55.529875
4507877	VCL	vinculin isoform VCL	NP_003364.1	16.51	12	12	36	1066	116.6	6.09	0.875	1.815	2.161																
														MQEAMTQEVSDVFSDTT TPIK	1	2	1	N-Term(iTRAQ8plex); K21(iTRAQ8plex)	7.152	3.658	2.199	4.83	-	0	2966.51603	3	1000.167114	0.723105883	33.43702833
														VLQLTSWDEDAWASK	2	2	1	N-Term(iTRAQ8plex); K15(iTRAQ8plex)	0.744	1.697	1.93	4.72	55	0	2357.24631	3	786.4202881	-5.718660848	40.774975
														MQEAMTQEVSDVFSDTT TPIK	2	2	1	N-Term(iTRAQ8plex); M1(Oxidation); M5(Oxidation); K21(iTRAQ8plex)	-	-	-	4.34	58	0	2998.48679	3	1000.167114	0.723105883	33.43702833
														CDRVDQLTAQLADLAAR	8	2	1	N-Term(iTRAQ8plex); C1(Methylthio)	2.055	2.955	4.108	4.24	41	1	2209.13578	3	737.0501099	-0.622849972	52.08884167
														TNISDEESEQATEMLVHN AQNLMQSVK	1	2	1	N-Term(iTRAQ8plex); K27(iTRAQ8plex)	-	-	-	4.16	-	0	3654.83095	4	914.4631958	2.980171522	51.20886
														LLAVAATAPPDAPNREEV FDER	5	2	1	N-Term(iTRAQ8plex)	0.907	3.502	2.446	4.1	26	1	2685.41452	3	895.8104248	0.618102974	38.60052
														MTGLVDEAIDTK	2	2	1	N-Term(iTRAQ8plex); K12(iTRAQ8plex)	0.713	0.833	1.314	3.74	38	0	1901.03464	3	634.3497314	-8.6269938	32.49356333
														NQWIDNVEK	6	2	1	N-Term(iTRAQ8plex); K9(iTRAQ8plex)	0.714	0.923	1.018	3.61	38	0	1753.95536	3	585.3233032	-7.96763839	27.17297
														NQGIEEALK	2	2	1	N-Term(iTRAQ8plex); K9(iTRAQ8plex)	1.086	2.368	3.235	3.45	45	0	1609.9262	2	805.4667358	-6.693623811	16.69119167
														NPGNQAAYEHFETMK	2	2	1	N-Term(iTRAQ8plex); K15(iTRAQ8plex)	0.901	1.894	2.161	3.35	25	0	2345.16428	3	782.3929443	-6.908489238	22.48503833
														MSAEINEIIR	2	2	1	N-Term(iTRAQ8plex)	0.9	1.283	2.209	3.29	31	0	1479.81267	2	740.4099731	-1.109350508	40.2134
														ELTPQVVSAAR	2	2	1	N-Term(iTRAQ8plex)	0.597	1.239	1.979	2.95	27	0	1474.84465	2	737.9259644	-5.749221825	23.08039667
														TDAGFTLR	1	2	1	N-Term(iTRAQ8plex)	0.83	1.236	1.61	-	30	0	1184.64922	2	592.8282471	-7.169868725	16.59213
4507947	YARS	tyrosine--tRNA ligase, cytoplasmic	NP_003671.1	6.44	2	2	4	528	59.1	7.05	0.551	1.954	2.058																
														QVEHPLLSGLLYPGLQALD EEYLK	2	1	1	N-Term(iTRAQ8plex); K24(iTRAQ8plex)	-	-	-	4.65	43	0	3333.87192	3	1111.962158	4.916223467	60.591105
														ISEECIAQWK	2	2	1	N-Term(iTRAQ8plex); C5(Methylthio); K10(iTRAQ8plex)	0.551	1.954	2.058	3.22	42	0	1860.96292	3	620.9924927	-9.622718657	65.56559667
4507951	YWHAH	14-3-3 protein eta	NP_003396.1	25.20	3	6	54	246	28.2	4.84	0.914	1.348	2.359																
														NSVVEASEAAYK	2	1	1	N-Term(iTRAQ8plex); K12(iTRAQ8plex)	1.326	1.442	2.589	4.97	39	0	1876.00998	3	626.0081787	-9.199533768	31.05072167
														AVTELNEPLSNEDR	2	1	1	N-Term(iTRAQ8plex)	0.914	1.348	2.359	4.62	50	0	1890.97038	2	945.9888306	-0.37376957	26.02634333
														EQMQPTHPIR	4	1	1	N-Term(iTRAQ8plex); M3(Oxidation)	0.327	0.5	1.032	3.29	31	0	1556.81046	2	770.9118042	-2.887768951	11.97116667
														EQMQPTHPIR	2	1	1	N-Term(iTRAQ8plex)	1.042	1.515	2.648	3.28	33	0	1540.81633	2	770.9118042	-2.887768951	11.97116667
														DSTLIMQLLR	24	9	7	N-Term(iTRAQ8plex)	-	-	-	4.73	54	0	1493.87629	3	498.6302795	6.656557269	46.36715833
														NLLSVAYK	16	10	7	N-Term(iTRAQ8plex); K8(iTRAQ8plex)	-	-	-	4.01	39	0	1515.92546	2	758.4644165	-9.192370231	25.08285833
														DSTLIMQLLR	2	9	7	N-Term(iTRAQ8plex); M6(Oxidation)	-	-	-	3.14	48	0	1509.85791	3	498.6302795	6.656557269	46.36715833
														VISSIEQK	2	5	5	N-Term(iTRAQ8plex); K8(iTRAQ8plex)	-	-	-	3.44	29	0	1511.91423	2	756.4607544	-7.341111487	22.45532333
4557237	ACAT1	acetyl-CoA acetyltransferase, mitochondrial precursor	NP_000010.1	16.16	5	5	11	427	45.2	8.85	0.830	0.285	0.794																
														TPIGSFLGSLSLLPATK	3	1	1	N-Term(iTRAQ8plex); K17(iTRAQ8plex)	0.968	0.288	0.829	4.77	35	0	2310.38266	3	770.7990723	-2.894651709	57.78016333
														IVAFADAAVEPIDFPIAPV YAASMVLK	4	3	1	N-Term(iTRAQ8plex); K27(iTRAQ8plex)	-	-	-	4.23	40	0	3426.92026	3	1142.978271	-0.166358372	70.63110833
														EVVIVSATR	2	1	1	N-Term(iTRAQ8plex)	0.66	0.217	0.998	3.6	54	0	1277.77704	3	426.5971985	3.099843598	26.22014333
														DGLTDVYNK	1	3	1	N-Term(iTRAQ8plex); K9(iTRAQ8plex)	0.83	0.526	0.794	3.45	-	0	1632.90373	2	816.9555054	-0.971997172	17.251285
														LEDLIVK	1	3	1	N-Term(iTRAQ8plex); K7(iTRAQ8plex)	0.515	0.281	0.693	-	23	0	1437.91477	3	479.9764404	0.734910779	53.99307333
4557491	CSTF1	cleavage stimulation factor subunit 1	NP_001315.1	8.58	2	2	4	431	48.3	6.58	0.844	0.917	1.295																
														CIVHSPTNPGFMTCSDDF R	2	1	1	N-Term(iTRAQ8plex); C1(Methylthio); C14(Methylthio)	0.788	1.047	1.243	5.41	40	0	2523.09336	3	841.7026367	1.273677108	42.29749167
														TQAVFNHTEDYVLLPDER	2	1	1	N-Term(iTRAQ8plex)	0.903	0.802	1.35	5.28	39	0	2451.23783	3	817.7507935	-3.257202878	36.53681
4557495	CSTF3	cleavage stimulation factor subunit 3 isoform 1	NP_001317.1	7.67	4	4	8	717	82.9	8.12	1.154	0.989	1.007																
														FLAFESNIGDLASILK	3	1	1	N-Term(iTRAQ8plex); K16(iTRAQ8plex)	1.212	0.911	1.125	5.1	60	0	2346.37003	3	782.7948608	7.269940086	62.21131
														GCVNPMINIEQLWR	2	1	1	N-Term(iTRAQ8plex); C2(Methylthio)	1.001	1.116	1.249	4.98	61	0	2023.03203	2	1012.019653	4.482242738	56.27159333
														KLEENPYDLDAWSILIR	2	3	1	N-Term(iTRAQ8plex); K1(iTRAQ8plex)	1.594	0.798	0.854	4.33	43	1	2683.48917	3	895.1679077	-0.902334727	52.66929667
														VLHIDLWK	1	1	1	N-Term(iTRAQ8plex); K8(iTRAQ8plex)	0.98	0.989	1.007	3.39	-	0	1632.0051	3	544.6732178	-2.598784579	36.78499833
4557541	TOR1A	torsin-1A precursor	NP_000104.1	3.31	1	1	2	332	37.8	6.99	1.071	0.657	0.570																
														GYEIDEDIVSR	2	1	1	N-Term(iTRAQ8plex)	1.071	0.657	0.57	4.26	31	0	1599.825	2	800.4161377	5.12397958	29.26867667
4557553	EMD	emerin	NP_000108.1	9.84	2	2	6	254	29.0	5.50	1.021	0.688	0.986																
														TYGEPESAGPSR	3	1	1	N-Term(iTRAQ8plex)	1.059	0.327	0.898	3.43	39	0	1554.76738	2	777.8873291	-1.800730391	14.37241167
														YNIPHGPVVGSTR	3	1	1	N-Term(iTRAQ8plex)	1.009	0.764	1.098	2.92	37	0	1700.93071	2	850.9689941	-4.626666269	21.23719333
4557581	FABP5	fatty acid-binding protein, epidermal	NP_001435.1	30.37	3	3	5	135	15.2	7.01	1.023	4.890	2.351																
														TQTVCNFTDGALVQHQE WDGK	2	1	1	N-Term(iTRAQ8plex); C5(Methylthio); K21(iTRAQ8plex)	-	-	-	5.06	33	0	3031.48232	4	758.6260376	-1.303583184	38.62003667
														TTQFSCTLGEK	2	1	1	N-Term(iTRAQ8plex); C6(Methylthio); K11(iTRAQ8plex)	0.841	3.853	1.967	4.06	37	0	1868.95493	3	623.6564941	-8.412791491	40.62718
														ELGVGIALR	1	1	1	N-Term(iTRAQ8plex)	1.245	6.208	2.809	-	37	0	1231.76396	2	616.3856201	-2.961351146	31.22443
4557701	KRT17	keratin, type I cytoskeletal 17	NP_000413.1	66.20	17	38	470	432	48.1	5.02	0.546	0.229	0.149																
														TMQALEIELQSQLSMK	10	1	1	N-Term(iTRAQ8plex); M15(Oxidation); K16(iTRAQ8plex)	0.796	0.397	0.437	7.46	45	0	2474.34116	2	1229.68103	1.697324935	51.24807333
														ADLEMQIENLKEELAYLK	16	1	1	N-Term(iTRAQ8plex); K11(iTRAQ8plex); K18(iTRAQ8plex)	0.497	0.135	0.079	6.67	40	1	3062.74819	4	766.4425049	8.834664025	62.12190333
														TMQALEIELQSQLSMK	36	1	1	N-Term(iTRAQ8plex); K16(iTRAQ8plex)	0.545	0.229	0.177	6.61	76	0	2458.35478	2	1229.68103	1.697324935	51.24807333
														ILTATVDNANILLQIDNAR	6	1	1	N-Term(iTRAQ8plex)	0.516	0.123	0.041	6.26	111	0	2372.35845	2	1186.682861	5.604016658	61.74064833
														ASLEGNLAETENR	18	1	1	N-Term(iTRAQ8plex)	0.557	0.202	0.105	6.2	67	0	1707.87688	2	854.4417725	-3.087459099	17.01053167
														ADLEMQIENLKEELAYLK	3	1	1	N-Term(iTRAQ8plex); M5(Oxidation); K11(iTRAQ8plex); K18(iTRAQ8plex)	0.462	0.284	-	5.31	-	1	3078.73354	4	766.4425049	8.834664025	62.12190333
														TMQALEIELQSQLSMK	4	1	1	N-Term(iTRAQ8plex); M2(Oxidation); M15(Oxidation); K16(iTRAQ8plex)	0.722	0.625	0.281	4.95	65	0	2490.33291	2	1229.68103	1.697324935	51.24807333
														ALEEANTELEVK	16	1	1	N-Term(iTRAQ8plex); K12(iTRAQ8plex)	0.54	0.301	0.172	4.87	86	0	1954.09183	2	977.5526123	1.333960448	25.53173833
														LSVEADINGLR	11	1	1	N-Term(iTRAQ8plex)	0.482	0.292	0.312	4.78	54	0	1490.84937	3	497.6213074	0.883770801	35.01416833
														ADLEMQIENLK	4	1	1	N-Term(iTRAQ8plex); K11(iTRAQ8plex)	0.638	0.193	0.164	4.42	50	0	1912.06235	3	638.0256348	-2.442521355	62.63486833
														TIVEEVQDGK	9	1	1	N-Term(iTRAQ8plex); K10(iTRAQ8plex)	0.598	0.216	0.206	4.23	53	0	1725.98308	2	863.4951782	-0.719285549	33.09785
														LSGGLGAGSCR	6	1	1	N-Term(iTRAQ8plex); C10(Methylthio)	0.476	0.177	0.045	4.15	58	0	1327.67951	3	443.2313538	2.316620297	27.39212667
														LLEGEDAHLTQYK	5	1	1	N-Term(iTRAQ8plex); K13(iTRAQ8plex)	0.581	0.304	0.185	4.05	25	0	2125.17641	3	709.0615234	-2.331219366	28.24747167
														TIEELQNK	10	1	1	N-Term(iTRAQ8plex); K8(iTRAQ8plex)	0.546	0.261	0.159	3.97	37	0	1582.92241	2	791.9648438	-2.30612693	30.09455333
														LSVEADINGLRR	5	1	1	N-Term(iTRAQ8plex)	0.67	0.358	0.293	3.96	36	1	1646.94646	2	823.9768677	-1.647028914	26.318025
														DAEDWFFSK	27	1	1	N-Term(iTRAQ8plex); K9(iTRAQ8plex)	0.583	0.161	0.123	3.82	39	0	1752.89946	2	876.9570313	0.82410543	34.49825
														QFTSSSSIK	9	1	1	N-Term(iTRAQ8plex); K9(iTRAQ8plex)	0.522	0.283	0.149	3.31	47	0	1592.90666	2	796.9569702	-2.346333528	16.31156667
														ATMQNLNDR	2	2	1	N-Term(iTRAQ8plex)	0.498	0.16	0.049	3.19	42	0	1366.70329	2	683.8552856	-1.307255894	14.00686
														ATMQNLNDR	2	2	1	N-Term(iTRAQ8plex); M3(Oxidation)	0.603	0.082	-	-	29	0	1382.69941	2	683.8552856	-1.307255894	14.00686
														KDAEDWFFSK	1	1	1	N-Term(iTRAQ8plex); K1(iTRAQ8plex); K10(iTRAQ8plex)	0.695	0.223	0.195	-	28	1	2185.20591	3	729.0734863	0.10728251	33.78587833
														DYSQYYR	3	1	1	N-Term(iTRAQ8plex)	0.602	0.28	0.136	-	22	0	1298.6281	2	649.817688	-2.881458856	17.01732667
														LAADDFR	10	31	7	N-Term(iTRAQ8plex)	-	-	-	-	39	0	1111.60307	2	556.3051758	-1.68797437	21.86801333
														TRLEQEIATYR	39	9	4	N-Term(iTRAQ8plex)	-	-	-	3.84	45	1	1683.92827	2	842.4677734	-2.90454474	28.813565
														LEQEIATYR	22	11	5	N-Term(iTRAQ8plex)	-	-	-	3.33	37	0	1426.78179	2	713.894104	-2.403951519	24.59227833
														LASYLDKVR	18	11	6	N-Term(iTRAQ8plex); K7(iTRAQ8plex)	-	-	-	3.04	34	1	1673.01726	4	419.0100403	-1.374788993	40.70226667
														VLDELTLAR	35	7	5	N-Term(iTRAQ8plex)	-	-	-	3.26	41	0	1333.80168	2	667.40448	1.785903382	36.03089333
														LASYLDK	45	13	7	N-Term(iTRAQ8plex); K7(iTRAQ8plex)	-	-	-	3.56	36	0	1417.84687	3	473.2871399	-2.979780374	31.776525
														EVATNSELVQSGK	15	2	2	N-Term(iTRAQ8plex); K13(iTRAQ8plex)	-	-	-	4.99	56	0	1970.09818	2	985.5529175	-1.483661338	20.27982833
														CEMEQQNQEYK	6	2	2	N-Term(iTRAQ8plex); C1(Methylthio); K11(iTRAQ8plex)	-	-	-	4.53	51	0	2083.96611	2	1042.486694	-2.217045708	29.56338833
														NHEEEMNALR	15	2	2	N-Term(iTRAQ8plex)	-	-	-	3.49	46	0	1546.75431	2	773.8811035	-2.360353717	12.883705
														NHEEEMNALR	7	2	2	N-Term(iTRAQ8plex); M6(Oxidation)	-	-	-	3.36	26	0	1562.75334	2	773.8811035	-2.360353717	12.883705
														CEMEQQNQEYK	6	2	2	N-Term(iTRAQ8plex); C1(Methylthio); M3(Oxidation); K11(iTRAQ8plex)	-	-	-	3.34	44	0	2099.96445	2	1042.486694	-2.217045708	29.56338833
														SEISELR	9	2	2	N-Term(iTRAQ8plex)	-	-	-	3.01	34	0	1137.64849	2	569.3234253	-1.887534604	15.25243333
														ILNEMRDQYEK	1	2	2	N-Term(iTRAQ8plex); K11(iTRAQ8plex)	-	-	-	2.98	-	1	2047.10805	4	512.5324707	-1.083659212	36.977935
														KNHEEEMNALR	2	2	2	N-Term(iTRAQ8plex); K1(iTRAQ8plex)	-	-	-	2.96	26	1	1979.05901	2	990.0331421	0.048639119	11.96262333
														TEELNREVATNSELVQSGK	1	2	2	N-Term(iTRAQ8plex); K19(iTRAQ8plex)	-	-	-	2.88	-	1	2712.45229	4	678.8685303	-3.756779901	28.37277167
														SEISELRR	2	2	2	N-Term(iTRAQ8plex)	-	-	-	2.84	-	1	1293.74082	3	431.918457	-1.564035255	14.41779667
														ILLDVK	16	2	2	N-Term(iTRAQ8plex); K6(iTRAQ8plex)	-	-	-	2.76	25	0	1308.86919	2	654.9382324	-1.469265429	39.06651667
														ILNEMR	2	3	3	N-Term(iTRAQ8plex)	-	-	-	-	25	0	1079.61772	2	540.3125	-0.711288805	26.970265
														IRDWYQR	1	4	3	N-Term(iTRAQ8plex)	-	-	-	-	23	1	1340.73529	3	447.5832825	-1.780469196	20.12547667
														TKFETEQALR	6	2	2	N-Term(iTRAQ8plex); K2(iTRAQ8plex)	-	-	-	2.8	48	1	1831.05312	4	458.5187378	-0.152921901	24.78654833
														FETEQALR	2	2	2	N-Term(iTRAQ8plex)	-	-	-	-	29	0	1297.70098	2	649.354126	-3.402915586	16.7415
														EELAYLKK	2	3	3	N-Term(iTRAQ8plex); K7(iTRAQ8plex); K8(iTRAQ8plex)	-	-	-	3.68	25	1	1906.17815	2	953.5927124	0.265916744	21.585135
														EELAYLK	5	3	3	N-Term(iTRAQ8plex); K7(iTRAQ8plex)	-	-	-	3.16	33	0	1473.87773	2	737.4425049	0.286214803	35.20986333
4557797	NME1	nucleoside diphosphate kinase A isoform b	NP_000260.1	34.21	1	4	37	152	17.1	6.19	0.590	1.301	1.632																
														NIIHGSDSVESAEK	2	2	1	N-Term(iTRAQ8plex); K14(iTRAQ8plex)	0.59	1.301	1.632	4.5	29	0	2094.11625	3	698.7102661	-5.97684593	15.48867333
														VMLGETNPADSKPGTIR	3	4	2	N-Term(iTRAQ8plex); M2(Oxidation); K12(iTRAQ8plex)	-	-	-	5.76	45	0	2410.31394	2	1197.662354	-4.072512953	23.83921333
														VMLGETNPADSKPGTIR	11	4	2	N-Term(iTRAQ8plex); K12(iTRAQ8plex)	-	-	-	4.94	87	0	2394.31743	2	1197.662354	-4.072512953	23.83921333
														TFIAIKPDGVQR	13	5	2	N-Term(iTRAQ8plex); K6(iTRAQ8plex)	-	-	-	3.88	39	0	1953.16631	2	977.086792	-4.041609562	25.15961667
														GDFCIQVGR	8	4	2	N-Term(iTRAQ8plex); C4(Methylthio)	-	-	-	3.68	28	0	1344.67644	2	672.8418579	4.333310336	31.69768333
4557817	OXCT1	succinyl-CoA:3-ketoacid coenzyme A transferase 1, mitochondrial precursor	NP_000427.1	4.04	2	2	3	520	56.1	7.46	0.838	0.563	0.279																
														GMGGAMDLVSSAK	1	1	1	N-Term(iTRAQ8plex); K13(iTRAQ8plex)	1.032	0.544	0.323	3.4	-	0	1831.98227	3	611.3322754	-2.406343222	41.32887167
														NFNLPMCK	2	2	1	N-Term(iTRAQ8plex); C7(Methylthio); K8(iTRAQ8plex)	0.681	0.584	0.241	2.88	26	0	1620.85361	3	540.9560547	0.955368511	33.97982667
4757714	ACP1	low molecular weight phosphotyrosine protein phosphatase isoform c	NP_004291.1	15.19	1	1	2	158	18.0	6.74	-	1.082	0.770																
														QLIIEDPYYGNDSDFETVY QQCVR	2	2	1	N-Term(iTRAQ8plex); C22(Methylthio)	-	1.082	0.77	3.15	37	0	3245.50632	3	1082.506958	-0.311669879	57.04539833
4757764	RHOB	rho-related GTP-binding protein RhoB precursor	NP_004031.1	25.51	1	3	16	196	22.1	5.24	-	-	-																
														DEFPEVYVPTVFENYVADI EVDGK	2	1	1	N-Term(iTRAQ8plex); K24(iTRAQ8plex)	-	-	-	2.81	27	0	3382.73232	4	846.4385376	3.887156706	66.14976667
														QVELALWDTAGQEDYDR	10	4	2	N-Term(iTRAQ8plex)	-	-	-	5.56	85	0	2313.14653	2	1157.076904	7.094524071	39.93450167
														TCLLIVFSK	4	7	2	N-Term(iTRAQ8plex); C2(Methylthio); K9(iTRAQ8plex)	-	-	-	3.65	34	0	1677.97356	2	839.4904175	-9.323126091	75.76786333
4757810	ATP5A1	ATP synthase subunit alpha, mitochondrial isoform a precursor	NP_004037.1	56.78	26	26	226	553	59.7	9.13	0.732	0.475	0.501																
														EVAAFAQFGSDLDAATQ QLLSR	4	4	1	N-Term(iTRAQ8plex)	0.846	0.385	0.406	6.88	107	0	2642.37851	3	881.4643555	2.146496508	68.65815667
														GMSLNLEPDNVGVVVFGN DK	7	4	1	N-Term(iTRAQ8plex); M2(Oxidation); K20(iTRAQ8plex)	0.67	0.377	0.401	6.29	79	0	2728.46123	2	1364.734253	6.429609183	39.23391
														NVQAEEMVEFSSGLK	17	4	1	N-Term(iTRAQ8plex); K15(iTRAQ8plex)	0.855	0.56	0.538	6.11	51	0	2276.19651	3	759.4036865	-3.872679561	62.00892333
														GMSLNLEPDNVGVVVFGN DK	6	4	1	N-Term(iTRAQ8plex); K20(iTRAQ8plex)	0.519	0.422	0.479	6.01	56	0	2712.43668	2	1364.734253	6.429609183	39.23391

														TGTAEMSSILEER	4	3	1	N-Term(iTRAQ8plex)	0.729	0.37	0.439	5.97	67	0	1727.88848	2	864.447876	5.616122373	30.97903
														FENAFLSHVVSQHQALLGTIR	23	4	1	N-Term(iTRAQ8plex)	0.845	0.604	0.584	5.64	50	0	2671.45493	3	891.1589966	0.07306713	48.24451833
														HALIIYDDLSK	35	4	1	N-Term(iTRAQ8plex); K11(iTRAQ8plex)	0.749	0.568	0.519	5.32	57	0	1896.1027	2	948.5525513	-3.831754268	29.42782667
														VVDALGNAIDGK	13	3	1	N-Term(iTRAQ8plex); K12(iTRAQ8plex)	0.691	0.411	0.398	5.3	63	0	1780.03777	2	890.522522	-2.67071563	31.94733
														TGTAEMSSILEER	3	3	1	N-Term(iTRAQ8plex); M6(Oxidation)	0.825	0.363	0.434	5.23	47	0	1743.87998	2	864.447876	5.616122373	30.97903
														TSIAIDTIINQK	7	4	1	N-Term(iTRAQ8plex); K12(iTRAQ8plex)	0.766	0.474	0.512	5.2	43	0	1925.14688	2	963.0733032	-6.987273326	32.878985
														LYCIYVAIGQK	4	4	1	N-Term(iTRAQ8plex); C3(Methylthio); K11(iTRAQ8plex)	0.413	0.377	0.408	5.09	39	0	1925.0925	3	642.3690186	3.958310602	53.24919
														EAYPGDVFYLHSR	39	4	1	N-Term(iTRAQ8plex)	0.795	0.776	0.694	4.71	70	0	1857.93967	2	929.4723511	-3.388581056	28.61605167
														ILGADTSVDLEETGR	10	4	1	N-Term(iTRAQ8plex)	0.824	0.457	0.47	4.63	84	0	1879.98711	2	940.4971924	-2.336800266	34.11064333
														TGAIVDVPVGEELLGR	6	3	1	N-Term(iTRAQ8plex)	0.755	0.647	0.858	4.27	57	0	1929.09294	2	965.0501099	-1.543830974	45.39366
														KLYCIYVAIGQK	3	4	1	N-Term(iTRAQ8plex); K1(iTRAQ8plex); C4(Methylthio); K12(iTRAQ8plex)	0.328	0.371	0.478	3.94	29	1	2357.38906	4	590.1027222	1.631674611	47.82218
														ITKFENAFLSHVVSQHQALLGTIR	5	4	1	N-Term(iTRAQ8plex); K3(iTRAQ8plex)	0.719	1.051	-	3.8	-	1	3317.89106	5	664.3840332	-0.983092668	58.27653833
														GIRPAINVGLSVSR	8	4	1	N-Term(iTRAQ8plex)	0.868	0.515	0.539	3.41	56	0	1743.0457	2	872.0288086	-2.278036617	25.63806833
														AVDSLVPIGR	5	4	1	N-Term(iTRAQ8plex)	0.645	0.481	0.479	2.98	38	0	1330.79607	2	665.9016724	-2.685004505	31.49088833
														FNDGSDEK	4	4	1	N-Term(iTRAQ8plex); K8(iTRAQ8plex)	0.518	0.468	0.464	2.89	25	0	1519.7885	2	760.3988647	3.666013415	13.46453167
														QVAGTMK	1	4	1	N-Term(iTRAQ8plex); M6(Oxidation); K7(iTRAQ8plex)	0.67	1.064	0.884	2.78	-	0	1358.79444	3	453.6029968	1.634484551	10.35431833
														VLSIGDGIAR	4	4	1	N-Term(iTRAQ8plex)	0.707	0.443	0.455	2.72	35	0	1304.77873	2	652.8930054	-4.029171901	24.69646333
														ELIIGDR	5	4	1	N-Term(iTRAQ8plex)	0.731	0.423	0.422	2.7	31	0	1119.66533	2	560.3363037	-1.983180209	22.74218833
														LTELLK	5	4	1	N-Term(iTRAQ8plex); K6(iTRAQ8plex)	0.636	0.388	0.372	2.68	24	0	1324.8676	2	662.937439	1.192579308	45.90041333
														EAYPGDVFYLHSR	1	4	1	N-Term(iTRAQ8plex); Y3(iTRAQ8plex)	0.511	0.3	0.237	-	35	0	2162.1415	2	929.4723511	-3.388581056	28.61605167
														LKEIVTNFLAGFEA	1	3	1	N-Term(iTRAQ8plex); K2(iTRAQ8plex)	0.766	0.433	0.429	-	32	1	2160.23979	2	1080.623535	-5.891756803	86.23027667
														QMSLLLR	2	4	1	N-Term(iTRAQ8plex)	0.679	0.429	0.454	-	26	0	1164.70293	2	582.8551025	-4.04638965	23.27556667
														YTIVVSATASDAAPLQYLAPYSGCSMGEYFR	1	4	1	N-Term(iTRAQ8plex); C24(Methylthio); M26(Oxidation); Y29(iTRAQ8plex)	-	-	-	-	26	0	4001.98354	4	1001.251343	6.270369054	56.354105
														ISEQSDAK	1	3	1	N-Term(iTRAQ8plex); K8(iTRAQ8plex)	0.752	0.574	0.568	-	25	0	1485.83415	2	743.4207153	-1.855695624	11.162535
														APGIIPR	2	4	1	N-Term(iTRAQ8plex)	0.669	0.394	0.445	-	19	0	1027.65422	2	514.3307495	-2.305149447	15.91154167
4757826	B2M	beta-2-microglobulin precursor	NP_004039.1	8.40	1	1	3	119	13.7	6.52	2.583	1.410	0.871																
														VEHSDLSFSK	3	2	1	N-Term(iTRAQ8plex); K10(iTRAQ8plex)	2.583	1.41	0.871	4.06	41	0	1756.9592	3	586.324585	-5.571937686	25.72865
4757834	BAG2	BAG family molecular chaperone regulator 2	NP_004273.1	8.53	2	2	5	211	23.8	6.70	2.031	1.620	1.378																
														LLESLDQLELR	2	1	1	N-Term(iTRAQ8plex)	2.031	1.62	1.378	3.95	29	0	1632.95073	2	816.9790039	2.020815715	50.437385
														RLETLLR	3	2	1	N-Term(iTRAQ8plex)	-	-	-	2.84	30	1	1204.76328	3	402.2592773	-3.861770052	23.74667167
4757886	PTTG1IP	pituitary tumor-transforming gene 1 protein-interacting protein isoform 1 precursor	NP_004330.1	10.00	1	1	1	180	20.3	8.79	0.907	0.415	0.496																
														ACLDYPVTSVLPPASLCK	1	1	1	N-Term(iTRAQ8plex); C2(Methylthio); C17(Methylthio); K18(iTRAQ8plex)	0.907	0.415	0.496	3.97	-	0	2577.33176	3	859.7821045	-3.570775629	57.29643167
4757900	CALR	calreticulin precursor	NP_004334.1	64.99	18	18	176	417	48.1	4.44	0.818	0.548	0.738																
														SGTIFDNFLITNDEAYAEEFGNETWGVTK	10	1	1	N-Term(iTRAQ8plex); K29(iTRAQ8plex)	1.371	0.365	0.772	7.63	43	0	3876.90598	4	969.9907227	8.901204444	60.897305
														GTWIHPEIDNPEYSPDPSIYAYDNFGVLGLDLWQVK	3	1	1	N-Term(iTRAQ8plex); K36(iTRAQ8plex)	-	-	-	6	45	0	4757.39077	4	1190.108887	2.35541739	65.70086833
														IDNSQVESGSLEDDWDFLPPK	6	1	1	N-Term(iTRAQ8plex); K21(iTRAQ8plex)	0.795	0.473	0.647	5.96	65	0	2999.51566	3	1000.510071	2.049615019	44.24113333
														IDNSQVESGSLEDDWDFLPPKK	8	1	1	N-Term(iTRAQ8plex); K21(iTRAQ8plex); K22(iTRAQ8plex)	1.188	0.588	0.783	5.62	49	1	3431.79922	4	858.7052612	-3.096410209	39.90019167
														HEQNIDCGGGYVK	16	1	1	N-Term(iTRAQ8plex); C7(Methylthio); K13(iTRAQ8plex)	0.841	0.807	0.767	5.38	71	0	2074.02481	2	1037.515991	-2.857486754	16.42392
														LFPNSLDQTDMHGDSEYNIMFGPDICGPGTK	14	1	1	N-Term(iTRAQ8plex); C26(Methylthio); K31(iTRAQ8plex)	1.222	0.9	1.008	5.24	31	0	4053.91074	3	1362.631592	-1.187053734	49.27461333
														FYALSASFEPFSNK	30	1	1	N-Term(iTRAQ8plex); K14(iTRAQ8plex)	0.773	0.43	0.726	5.22	79	0	2216.17032	2	1108.588135	-7.137717747	74.655275
														LFPNSLDQTDMHGDSEYNIMFGPDICGPGTK	13	1	1	N-Term(iTRAQ8plex); M11(Oxidation); C26(Methylthio); K31(iTRAQ8plex)	0.717	0.37	0.518	4.7	28	0	4069.90369	3	1362.631592	-1.187053734	49.27461333
														GQTLVVQFTVK	18	1	1	N-Term(iTRAQ8plex); K11(iTRAQ8plex)	0.697	0.5	0.657	4.65	63	0	1828.11199	2	914.5618286	0.606803032	30.188525
														LFPNSLDQTDMHGDSEYNIMFGPDICGPGTK	3	1	1	N-Term(iTRAQ8plex); M11(Oxidation); M20(Oxidation); C26(Methylthio); [illegible]	1.659	0.441	0.752	4.41	33	0	4085.88906	3	1362.631592	-1.187053734	49.27461333
														EQFLDGDGWTSR	20	1	1	N-Term(iTRAQ8plex)	0.812	0.546	0.734	4.16	50	0	1714.83927	3	572.2846069	3.152628286	33.14791333
														QIDNPDYK	2	1	1	N-Term(iTRAQ8plex); K8(iTRAQ8plex)	0.969	0.544	0.808	4.01	30	0	1600.87944	2	800.9433594	0.212901728	16.70709667
														VHVIFNYK	8	1	1	N-Term(iTRAQ8plex); K8(iTRAQ8plex)	0.626	0.729	0.913	3.75	42	0	1627.98172	2	814.4885254	-5.065904098	28.31012667
														KPEDWDEEMDGEWEPPVIQNPEYK	2	1	1	N-Term(iTRAQ8plex); K1(iTRAQ8plex); K24(iTRAQ8plex)	-	-	-	3.71	-	0	3872.90932	4	968.9827881	0.000300485	37.78701167
														KVHVIFNYK	4	1	1	N-Term(iTRAQ8plex); K1(iTRAQ8plex); K9(iTRAQ8plex)	1.676	0.979	0.901	3.69	19	1	2060.28505	3	687.4332275	3.289868519	30.14526667
														FYGDEEKDK	1	1	1	N-Term(iTRAQ8plex); K7(iTRAQ8plex); K9(iTRAQ8plex)	1.01	0.513	0.666	3.5	-	1	2043.12343	4	511.5363159	3.554602603	22.34874333
														CKDDEFTHLYTLIVRPDNTYEVK	6	1	1	N-Term(iTRAQ8plex); C1(Methylthio); K2(iTRAQ8plex); K23(iTRAQ8plex)	0.902	-	0.448	3.47	-	1	3757.96064	5	752.3979492	-2.411604889	50.52588833
														IKDPDASKPEDWDER	1	1	1	N-Term(iTRAQ8plex); K2(iTRAQ8plex); K8(iTRAQ8plex)	0.66	0.267	0.57	3.22	-	1	2713.45053	3	905.1550293	-2.036764372	20.043615
														NVLINK	3	1	1	N-Term(iTRAQ8plex); K6(iTRAQ8plex)	0.917	0.68	0.792	2.94	27	0	1308.85002	3	436.9547729	2.914236421	14.85437167
														GLQTSQDAR	4	1	1	N-Term(iTRAQ8plex)	0.775	0.408	0.598	2.77	35	0	1279.68767	2	640.3474731	-2.447183802	12.34416
														FYALSASFEPFSNK	3	1	1	N-Term(iTRAQ8plex); Y2(iTRAQ8plex); K14(iTRAQ8plex)	0.82	0.452	0.654	-	38	0	2520.37241	2	1108.588135	-7.137717747	74.655275
														SGTIFDNFLITNDEAYAEEFGNETWGVTK	1	1	1	N-Term(iTRAQ8plex); Y16(iTRAQ8plex); K29(iTRAQ8plex)	-	-	-	-	28	0	4181.13881	4	969.9907227	8.901204444	60.897305
4757902	CETN2	centrin-2	NP_004335.1	7.56	1	1	2	172	19.7	5.00	0.582	0.626	1.169																
														MNFGDFLTVMTQK	2	1	1	N-Term(iTRAQ8plex); K13(iTRAQ8plex)	0.582	0.626	1.169	4.17	30	0	2140.13504	3	714.0498657	-1.919216286	46.86243167
4757952	CDC42	cell division control protein 42 homolog isoform 1 precursor	NP_001782.1	36.65	5	6	27	191	21.2	6.55	1.028	0.997	0.966																
														TPFLLVGTQIDLRDDPSTIEK	2	2	1	N-Term(iTRAQ8plex); K21(iTRAQ8plex)	-	-	-	5.7	28	1	2966.66396	4	742.4214478	-0.670842536	44.72865167
														NVFDEAILAALEPPEPK	10	1	1	N-Term(iTRAQ8plex); K17(iTRAQ8plex)	1.036	1.081	0.845	4.72	74	0	2461.36631	2	1231.190674	-2.380891153	60.77684
														YVECSALTQK	4	1	1	N-Term(iTRAQ8plex); C4(Methylthio); K10(iTRAQ8plex)	1.132	1.142	1.015	4.44	32	0	1795.95224	3	599.3222656	-1.124013741	45.390895
														QKPITPETAEK	1	2	1	N-Term(iTRAQ8plex); K2(iTRAQ8plex); K11(iTRAQ8plex)	1.082	1.205	0.964	3.96	-	0	2154.29506	4	539.3292236	2.475682806	19.31798833
														TPFLLVGTQIDLR	4	2	1	N-Term(iTRAQ8plex)	0.977	0.647	1.024	3.42	39	0	1777.05027	3	593.0216064	-1.297213763	51.02731
														CVVVGDGAVGK	6	16	3	N-Term(iTRAQ8plex); C1(Methylthio); K11(iTRAQ8plex)	-	-	-	4.67	48	0	1657.90886	2	829.4603271	-5.563995833	21.63299833
4758012	CLTC	clathrin heavy chain 1 isoform 1	NP_004850.1	35.94	48	48	317	1675	191.5	5.69	1.037	1.057	1.267																
														LAELEEFINGPNNAHIQQVGDR	19	3	1	N-Term(iTRAQ8plex)	1.078	1.14	1.728	7.42	81	0	2768.43601	3	923.4824219	2.062927148	54.738615
														ISGETIFVTAPHEATAGIIGVNR	10	3	1	N-Term(iTRAQ8plex)	0.855	0.755	1.296	7.11	60	0	2657.45469	3	886.4897461	-0.687224794	36.50632333
														VIQCFAETGQVQK	12	3	1	N-Term(iTRAQ8plex); C4(Methylthio); K13(iTRAQ8plex)	1.108	1.096	1.379	6.8	72	0	2105.12651	2	1053.066895	-3.738424961	33.40001333
														GQCDLELINVCNENSLFK	8	3	1	N-Term(iTRAQ8plex); C3(Methylthio); C11(Methylthio); K18(iTRAQ8plex)	0.952	1.249	1.264	6.46	61	0	2739.36284	2	1370.185059	7.06268889	57.11008333
														HSSLAGCQIINYR	6	3	1	N-Term(iTRAQ8plex); C7(Methylthio)	1.23	0.898	0.903	5.89	80	0	1811.91281	3	604.6424561	-3.872915893	37.43693833
														NLQNLLILTAIK	14	11	1	N-Term(iTRAQ8plex); K12(iTRAQ8plex)	1.053	0.904	1.229	5.65	70	0	1962.24724	2	981.6272583	-5.071388679	74.69727833
														LASTLVHLGEYQAAVDGAR	7	3	1	N-Term(iTRAQ8plex)	0.961	1.045	1.298	5.51	64	0	2275.22312	3	759.0792236	-5.161538432	46.35177
														LHIIEVGTPPTGNQPFPK	4	3	1	N-Term(iTRAQ8plex); K18(iTRAQ8plex)	0.385	1.254	1.728	5.4	32	0	2553.45737	3	851.8239746	-2.980555682	33.240255
														TLQIFNIEMK	6	10	1	N-Term(iTRAQ8plex); K10(iTRAQ8plex)	0.892	0.885	1.063	5.32	42	0	1845.0584	2	923.0328369	-9.785105936	67.37879667
														GQFSTDELVAEVEKR	5	11	1	N-Term(iTRAQ8plex); K14(iTRAQ8plex)	1.218	1.279	1.877	5.16	47	1	2316.25014	4	579.8179932	-6.678261326	35.333215
														KFDVNTSAVQVLIEHIGNLDR	14	3	1	N-Term(iTRAQ8plex); K1(iTRAQ8plex)	0.761	0.758	0.819	5.14	67	1	2976.66996	3	992.8962402	0.472390677	56.205475
														AHTMTDDVTFWK	7	3	1	N-Term(iTRAQ8plex); K12(iTRAQ8plex)	1.057	1.254	1.161	5.13	40	0	2060.05936	3	687.3566284	-8.654765635	29.909745
														NNLAGAEELFAR	7	3	1	N-Term(iTRAQ8plex)	1.007	0.84	1.212	5.01	44	0	1608.85735	2	804.932312	-4.614390813	35.46288167
														AHTMTDDVTFWK	4	3	1	N-Term(iTRAQ8plex); M4(Oxidation); K12(iTRAQ8plex)	1.098	1.382	1.86	5.01	34	0	2076.05692	3	687.3566284	-8.654765635	29.909745
														IVLDNSVFSEHR	13	11	1	N-Term(iTRAQ8plex)	1.055	1.378	1.559	4.91	61	0	1719.92521	3	573.9799194	-4.631741657	30.64619167
														ESYVETELIFALAK	12	3	1	N-Term(iTRAQ8plex); K14(iTRAQ8plex)	1.129	0.887	1.198	4.87	50	0	2221.25278	3	741.0891113	-2.192711099	58.18359
														LLEMNLMHAPQVADAILGNQMFTHYDR	4	3	1	N-Term(iTRAQ8plex)	-	-	-	4.85	38	0	3432.73037	4	858.9380493	3.908284132	64.46444
														RPLIDQVVQTALSETQDPEEVSVTVK	8	3	1	N-Term(iTRAQ8plex); K26(iTRAQ8plex)	-	-	-	4.84	52	0	3489.93271	3	1163.982422	1.877135045	57.844965
														IHEGCEEPATHNALAK	3	3	1	N-Term(iTRAQ8plex); C5(Methylthio); K16(iTRAQ8plex)	1.808	2.504	3.061	4.82	24	0	2374.19863	4	594.3051147	-4.963020145	18.383165
														GQFSTDELVAEVEK	4	11	1	N-Term(iTRAQ8plex); K14(iTRAQ8plex)	1.062	1.271	1.134	4.72	40	0	2160.1492	3	720.7212524	-7.076930497	57.91777667
														VVGAMQLYSVDR	8	10	1	N-Term(iTRAQ8plex)	0.97	1.014	1.278	4.69	54	0	1641.898	2	821.4526367	2.66491433	35.29242667
														RPISADSAIMNPASK	2	3	1	N-Term(iTRAQ8plex); M10(Oxidation); K15(iTRAQ8plex)	0.709	0.517	1.022	4.66	27	0	2182.19913	3	728.071228	-5.474050374	18.61274833
														VGEQAQVVIIDMNDPSNPIR	2	3	1	N-Term(iTRAQ8plex)	0.608	0.467	1.002	4.62	30	0	2499.31821	3	833.7775879	0.095674842	44.39913333
														AFMTADLPNELIELLEK	6	11	1	N-Term(iTRAQ8plex); K17(iTRAQ8plex)	2.126	2.042	3.019	4.53	44	0	2555.4177	2	1278.214966	-0.984682268	62.761285
														KDPELWGSVLLESNPYR	4	3	1	N-Term(iTRAQ8plex); K1(iTRAQ8plex)	1.186	1.218	1.704	4.49	44	1	2611.42527	3	871.1466064	-3.371803964	43.96518167
														KVGYTPDWIFLLR	15	10	1	N-Term(iTRAQ8plex); K1(iTRAQ8plex)	1.198	0.957	1.455	4.28	47	1	2216.2936	4	554.8288574	-5.239149046	52.968825
														TSIDAYDNFDNISLAQR	2	3	1	N-Term(iTRAQ8plex)	0.34	1.258	1.19	4.27	48	0	2247.11899	3	749.7111816	-0.244592354	37.63081833
														AFMTADLPNELIELLEK	6	11	1	N-Term(iTRAQ8plex); M3(Oxidation); K17(iTRAQ8plex)	1.468	0.902	1.323	4.25	30	0	2571.42368	2	1278.214966	-0.984682268	62.761285
														VGYTPDWIFLLR	14	10	1	N-Term(iTRAQ8plex)	1.066	0.822	1.168	4.17	61	0	1784.01238	2	892.5098267	4.201428427	61.181765
														CNEPAVWSQLAK	8	3	1	N-Term(iTRAQ8plex); C1(Methylthio); K12(iTRAQ8plex)	0.918	1.284	1.477	4.09	54	0	2000.03569	2	1000.521484	-9.854909129	58.26055
														RPISADSAIMNPASK	3	3	1	N-Term(iTRAQ8plex); K15(iTRAQ8plex)	0.843	0.851	0.886	4.02	-	0	2166.20597	3	728.071228	-5.474050374	18.61274833
														WLLLTGISAQQNR	4	3	1	N-Term(iTRAQ8plex)	0.913	0.912	1.271	4.01	44	0	1804.04924	2	902.5282593	6.058944753	46.60973167
														EVCFACVDGK	2	3	1	N-Term(iTRAQ8plex); C3(Methylthio); C6(Methylthio); K10(iTRAQ8plex)	0.846	0.781	1.105	3.82	45	0	1770.84148	2	885.9243774	-5.237643968	66.51825333
														LKLLLPWLEAR	10	3	1	N-Term(iTRAQ8plex); K2(iTRAQ8plex)	1.342	1.421	1.771	3.79	27	1	1960.2604	4	490.8205566	1.829033789	63.92221167
														LLYNNVSNFGR	6	3	1	N-Term(iTRAQ8plex)	1.079	1.114	1.416	3.69	37	0	1600.86809	2	800.9363403	-5.938822785	26.71806
														EHLELFWSR	7	3	1	N-Term(iTRAQ8plex)	0.776	0.995	1.114	3.54	37	0	1520.8165	3	507.6075745	-5.372907306	31.81119333
														MREHLELFWSR	3	3	1	N-Term(iTRAQ8plex)	1.292	1.019	1.286	3.53	32	1	1807.97466	2	904.4909668	9.234462267	34.6353
														IYIDSNNNPER	7	3	1	N-Term(iTRAQ8plex)	1.23	1.074	1.45	3.33	53	0	1638.83257	2	819.9199219	-3.877253923	20.78305
														DPHLACVAYER	7	11	1	N-Term(iTRAQ8plex); C6(Methylthio)	0.922	0.95	1.171	3.29	38	0	1623.78581	2	812.3965454	-4.124989656	25.00684667
														LEKHELIEFR	2	3	1	N-Term(iTRAQ8plex); K3(iTRAQ8plex)	1.417	1.383	1.797	3.23	35	1	1922.12676	2	961.5670166	-2.72339837	24.29152167
														HDVVFLITK	1	3	1	N-Term(iTRAQ8plex); K9(iTRAQ8plex)	0.845	1.142	2.017	3.21	-	0	1680.02707	3	560.680542	-2.02292778	30.29286
														LLLPWLEAR	8	3	1	N-Term(iTRAQ8plex)	0.916	0.954	1.288	3.09	43	0	1414.86772	3	472.2946777	-2.071814338	46.09994833
														AHMGMFTELAILYSK	2	10	1	N-Term(iTRAQ8plex); K15(iTRAQ8plex)	0.543	0.626	0.911	3.04	-	0	2320.25722	4	580.8197632	-3.529111145	51.89733667
														IAAYLFK	6	3	1	N-Term(iTRAQ8plex); K7(iTRAQ8plex)	1.185	1.099	1.485	2.92	26	0	1433.9021	3	478.6326904	-9.856631142	42.89216333
														ISGETIFVTAPHEATAGIIGVNRK	1	3	1	N-Term(iTRAQ8plex); K24(iTRAQ8plex)	-	-	-	2.88	-	1	3089.75503	4	773.1942139	-0.587655408	36.74372333
														QLPLVKPYLR	2	3	1	N-Term(iTRAQ8plex); K6(iTRAQ8plex)	0.883	0.928	1.018	2.86	-	0	1835.15923	2	918.083252	-7.353047926	29.04763667
														MREHLELFWSR	2	3	1	N-Term(iTRAQ8plex); M1(Oxidation)	-	-	-	2.8	23	1	1823.94607	2	904.4909668	9.234462267	34.6353
														HELIEFR	5	3	1	N-Term(iTRAQ8plex)	1.119	1.169	1.281	2.76	37	0	1247.69886	3	416.5711365	-4.917152999	25.563365
														KGQVLSVCVEEENIIPYITNVLQNPDLALR	1	3	1	N-Term(iTRAQ8plex); K1(iTRAQ8plex); C8(Methylthio)	-	-	-	2.71	-	1	4022.21889	4	1006.310181	6.787416841	68.75247
														NLILVVR	2	3	1	N-Term(iTRAQ8plex)	0.944	0.737	1.124	-	32	0	1130.75176	2	565.8795166	-4.019199483	31.43966167

														RDPHLACVAYER	1	11	1	N-Term(iTRAQ8plex); C7(Methylthio)	1.106	0.87	1.244	-	30	1	1779.90227	2	890.4547729	4.852501504	20.12132333
														ESNCYDPER	1	3	1	N-Term(iTRAQ8plex); C4(Methylthio)	0.965	1.069	1.093	-	29	0	1462.61553	2	731.8114014	-6.089218572	14.58316
4758152	TIMM8A	mitochondrial import inner membrane translocase subunit Tim8 A isoform 1	NP_004076.1	11.34	1	1	2	97	11.0	5.16	0.827	0.530	0.964																
														AEACFVNCVER	2	1	1	N-Term(iTRAQ8plex); C4(Methylthio); C8(Methylthio)	0.827	0.53	0.964	3.71	38	0	1636.72075	2	818.8640137	-3.068682454	37.79525667
4758208	DUSP3	dual specificity protein phosphatase 3	NP_004081.1	7.03	1	1	4	185	20.5	7.80	-	1.302	3.831																
														LGITHVLNAAEGR	4	1	1	N-Term(iTRAQ8plex)	-	1.302	3.831	3.28	36	0	1654.94675	3	552.3204346	-4.53237487	31.04621333
4758220	FAM50A	protein FAM50A	NP_004690.1	4.72	1	1	1	339	40.2	6.83	0.889	1.386	3.184																
														LLSDATVEKDESHAGK	1	1	1	N-Term(iTRAQ8plex); K9(iTRAQ8plex); K16(iTRAQ8plex)	0.889	1.386	3.184	3.11	-	1	2612.44497	4	653.8666992	-8.005644239	21.58681667
4758256	EIF2S1	eukaryotic translation initiation factor 2 subunit 1	NP_004085.1	40.63	10	10	28	315	36.1	5.08	1.492	2.879	2.330																
														HVAEVLEYTKDEQLESLFQR	5	1	1	N-Term(iTRAQ8plex); K10(iTRAQ8plex)	1.755	2.35	2.418	6.1	33	1	3042.63076	4	761.413147	-1.621389438	47.230725
														HAVSDPSILDSLDLNEDEREVLINNINR	5	1	1	N-Term(iTRAQ8plex)	-	-	-	5.84	30	1	3494.80019	4	874.4580078	2.065940519	45.59533833
														ENAEVDGDDDAEEMEAK	2	1	1	N-Term(iTRAQ8plex); M14(Oxidation); K17(iTRAQ8plex)	1.641	2.806	1.92	5.79	63	0	2491.11673	3	831.0437622	-2.887431492	18.77035333
														VVTDTDETELAR	2	1	1	N-Term(iTRAQ8plex)	1.853	3.529	2.585	3.88	59	0	1652.85893	2	826.9331055	-3.362042812	22.03386
														AGLNCSTENMPIK	2	1	1	N-Term(iTRAQ8plex); C5(Methylthio); K13(iTRAQ8plex)	1.515	1.959	1.904	3.74	55	0	2032.03342	3	678.0159912	-7.476214237	41.19128
														NECVVVIR	4	1	1	N-Term(iTRAQ8plex); C3(Methylthio)	1.471	3.291	2.649	3.53	32	0	1281.68877	2	641.3480225	-5.714334327	35.84056
														HAVSDPSILDSLDLNEDER	2	1	1	N-Term(iTRAQ8plex)	1.327	1.393	1.444	3.36	25	0	2429.2042	3	810.40625	-2.323621047	34.72999
														YVMTTTTLER	3	1	1	N-Term(iTRAQ8plex)	1.694	3.284	2.347	3.35	26	0	1518.80889	2	759.9080811	-3.335383848	26.66353833
														FPEVEDVVMVNVR	2	1	1	N-Term(iTRAQ8plex)	1.469	2.879	2.332	3.19	47	0	1836.97771	2	918.9924927	-2.984559046	49.79933833
														TVYSILR	1	1	1	N-Term(iTRAQ8plex)	1.037	2.272	1.678	-	23	0	1155.69939	2	578.3533325	-3.913544459	30.35908667
4758302	ERH	enhancer of rudimentary homolog	NP_004441.1	23.08	2	2	25	104	12.3	5.92	1.383	1.039	0.839																
														TYADYESVNECMEGVCK	20	1	1	N-Term(iTRAQ8plex); C11(Methylthio); C16(Methylthio); K17(iTRAQ8plex)	1.365	1.12	0.834	5.29	78	0	2641.15958	2	1321.09375	8.674182775	47.25273
														TYADYESVNECMEGVCK	2	1	1	N-Term(iTRAQ8plex); C11(Methylthio); M12(Oxidation); C16(Methylthio); K17(iTRAQ8plex)	1.721	0.971	0.839	2.93	26	0	2657.14945	2	1321.09375	8.674182775	47.25273
														TYADYESVNECMEGVCK	1	1	1	N-Term(iTRAQ8plex); Y5(iTRAQ8plex); C11(Methylthio); C16(Methylthio); K17(iTRAQ8plex)	1.364	2.556	2.583	-	27	0	2945.35732	2	1321.09375	8.674182775	47.25273
														IYVLLRR	2	1	1	N-Term(iTRAQ8plex)	1.347	0.745	0.558	-	25	1	1236.80399	3	412.9395142	-4.367765287	35.46763833
4758304	PDIA4	protein disulfide-isomerase A4 precursor	NP_004902.1	28.22	15	15	67	645	72.9	5.07	0.826	0.732	1.005																
														YGIVDYMIEQSGPPSK	12	2	1	N-Term(iTRAQ8plex); K16(iTRAQ8plex)	0.575	0.678	0.917	6.68	78	0	2392.27324	2	1196.640259	2.232043578	43.05361833
														GESDPAYQQYQDAANNLR	4	2	1	N-Term(iTRAQ8plex)	0.58	0.526	0.761	6.56	93	0	2344.12358	2	1172.56543	5.469615636	29.22646667
														EKYGIVDYMIEQSGPPSK	2	2	1	N-Term(iTRAQ8plex); K2(iTRAQ8plex); K18(iTRAQ8plex)	-	0.786	0.73	4.84	37	1	2953.61464	4	739.1591187	1.291105927	35.59116333
														FAMEPEEFDSDTLR	6	2	1	N-Term(iTRAQ8plex)	0.855	0.679	1.167	4.69	72	0	1990.93279	2	995.9700317	-2.131886855	39.99798333
														VDATAETDLAK	4	2	1	N-Term(iTRAQ8plex); K11(iTRAQ8plex)	0.782	0.696	1.03	4.67	55	0	1741.96794	2	871.4876099	-6.485879078	29.47463333
														KDVLIEFYAPWCGHCK	5	2	1	N-Term(iTRAQ8plex); K1(iTRAQ8plex); C12(Methylthio); C15(Methylthio); K16(iTRAQ8plex)	0.97	1.591	1.415	4.46	37	1	2913.49575	4	729.1293945	-3.217802877	55.21284333
														DFPEYTFAIADEEDYAGEVK	4	2	1	N-Term(iTRAQ8plex); K20(iTRAQ8plex)	0.809	0.672	0.989	4.45	56	0	2917.43277	3	973.1491089	2.996507833	46.82241167
														GESDPAYQQYQDAANNLREDYK	4	2	1	N-Term(iTRAQ8plex); K22(iTRAQ8plex)	1.12	0.974	1.038	3.9	23	1	3183.53098	3	1061.848511	-4.077963596	30.79816333
														FDVSGYPTIK	5	2	1	N-Term(iTRAQ8plex); K10(iTRAQ8plex)	0.838	0.795	1.085	3.8	44	0	1734.981	2	867.9941406	-4.413114599	43.52280833
														MDATANDVPSDR	4	2	1	N-Term(iTRAQ8plex)	0.896	0.761	1.03	3.79	63	0	1595.75859	2	798.3829346	-3.22654028	14.597135
														MDATANDVPSDR	4	2	1	N-Term(iTRAQ8plex); M1(Oxidation)	0.964	0.769	1.224	3.72	41	0	1611.75566	2	798.3829346	-3.22654028	14.597135
														FAMEPEEFDSDTLR	2	2	1	N-Term(iTRAQ8plex); M3(Oxidation)	0.611	0.499	0.508	3.47	37	0	2006.92856	2	995.9700317	-2.131886855	39.99798333
														RFDVSGYPTLK	2	2	1	N-Term(iTRAQ8plex); K11(iTRAQ8plex)	0.873	0.735	0.993	3.19	-	1	1891.07737	3	631.0306396	-6.565360526	28.40322333
														YALPLVGHR	3	2	1	N-Term(iTRAQ8plex)	0.894	1.179	1.325	2.99	59	0	1329.78797	3	443.9341736	-4.890853434	24.93019167
														IDATSASVLASR	4	2	1	N-Term(iTRAQ8plex)	0.863	0.641	0.901	2.98	59	0	1494.83879	2	747.9230347	-2.788651246	26.643305
														QVQEFLKDGDDVIIIGVFK	1	2	1	N-Term(iTRAQ8plex); K7(iTRAQ8plex); K19(iTRAQ8plex)	-	-	-	2.91	-	1	3075.78188	4	769.7009277	-1.271300323	57.80267
														SPPIPLAK	1	2	1	N-Term(iTRAQ8plex); K8(iTRAQ8plex)	0.808	0.665	0.855	-	30	0	1430.91973	2	715.963501	0.415140776	18.73073833
4758332	ACSL4	long-chain-fatty-acid--CoA ligase 4 isoform 1	NP_004449.1	10.60	2	4	8	670	74.4	8.03	0.728	0.553	1.273																
														GVEGTWVDICNNPAMEAEILK	2	2	1	N-Term(iTRAQ8plex); C10(Methylthio); K21(iTRAQ8plex)	0.73	0.512	1.037	6.97	61	0	2943.50022	3	981.8382568	4.302686216	55.83035333
														WFCTGDIGEFHPDGCLQIIDR	2	2	1	N-Term(iTRAQ8plex); C3(Methylthio); C15(Methylthio)	0.726	0.598	1.564	4.91	37	0	2818.28666	3	940.1004028	3.644748513	63.23123833
														LSPEPWTPETGLVTDAFK	2	3	2	N-Term(iTRAQ8plex); K18(iTRAQ8plex)	-	-	-	5.89	55	0	2596.43022	2	1298.71875	7.037385344	44.65474
														LQAGEYVSLGK	2	3	2	N-Term(iTRAQ8plex); K11(iTRAQ8plex)	-	-	-	3.04	28	0	1773.03073	3	591.6817627	-3.353841502	39.63769
4758340	FARSA	phenylalanine--tRNA ligase alpha subunit	NP_004452.1	5.31	2	2	4	508	57.5	7.80	0.810	1.525	0.926																
														SLQALGEVIEAELR	2	1	1	N-Term(iTRAQ8plex)	-	-	-	5.59	68	0	1832.04728	3	611.3539429	2.25652653	61.141675
														HWELTAEGEEIAR	2	1	1	N-Term(iTRAQ8plex)	0.81	1.525	0.926	3.1	24	0	1844.93625	3	615.6502686	-4.460254888	22.91022333
4758356	FEN1	flap endonuclease 1	NP_004102.1	15.79	4	4	9	380	42.6	8.62	1.240	1.409	1.445																
														QGGDVLQNEEGETTSHLMGMFYR	4	1	1	N-Term(iTRAQ8plex)	1.361	1.366	1.428	6.14	54	0	2903.35233	3	968.4556274	-2.862975223	46.44458333
														EAHQLFLEPEVLDPESVELK	1	1	1	N-Term(iTRAQ8plex); K20(iTRAQ8plex)	-	-	-	3.93	-	0	2930.58779	4	733.4024048	-3.211655835	44.24080667
														LIADVAPSAIR	2	1	1	N-Term(iTRAQ8plex)	0.768	1.149	1.074	3.69	30	0	1429.86186	2	715.4345703	-4.334191404	36.06357
														LDDFFK	2	1	1	N-Term(iTRAQ8plex); K6(iTRAQ8plex)	1.291	4.341	3.256	2.72	28	0	1392.78472	2	696.8959961	-9.783085891	34.35989167
4758442	GMFB	glia maturation factor beta	NP_004115.1	16.20	1	1	1	142	16.7	5.29	-	-	-																
														LVVLDEELEGISPDELKDELPER	1	1	1	N-Term(iTRAQ8plex); K17(iTRAQ8plex)	-	-	-	-	28	1	3245.75185	4	812.1934204	-2.942165578	52.70511833
4758516	HDGF	hepatoma-derived growth factor isoform a	NP_004485.1	29.17	4	4	7	240	26.8	4.73	1.033	2.416	1.349																
														SCVEEPEPEPEAAEGDGDK	2	5	1	N-Term(iTRAQ8plex); C2(Methylthio); K19(iTRAQ8plex)	0.898	2.416	1.349	5.93	51	0	2642.21299	3	881.4091797	2.688470815	25.45219333
														EAENPEGEEKEAATLEVERPLPMEVEK	1	5	1	N-Term(iTRAQ8plex); K10(iTRAQ8plex); M23(Oxidation); K27(iTRAQ8plex)	-	-	-	4.13	-	1	3982.04633	5	797.2150879	-6.728539709	27.61964833
														GFSEGLWEIENNPTVK	2	5	1	N-Term(iTRAQ8plex); K16(iTRAQ8plex)	1.033	1.682	1.33	3.93	39	0	2428.29874	3	810.1044312	0.753163934	37.16707
														CGDLVFAK	2	1	1	N-Term(iTRAQ8plex); C1(Methylthio); K8(iTRAQ8plex)	1.061	3.893	3.15	2.69	27	0	1506.81426	2	753.9107666	-8.385716302	33.20121333
4758638	PRDX6	peroxiredoxin-6	NP_004896.1	46.88	10	10	33	224	25.0	6.38	0.810	3.689	3.505																
														FHDFLGDSWGILFSHPR	3	1	1	N-Term(iTRAQ8plex)	0.722	2.756	2.08	5.29	37	0	2335.18471	4	584.5516357	-3.37798171	59.97822
														ELAILLGMLDPAEKDEK	6	1	1	N-Term(iTRAQ8plex); K14(iTRAQ8plex); K17(iTRAQ8plex)	-	-	-	5.18	34	1	2797.61074	4	700.1568604	-3.317041441	64.13870333
														DINAYNCEEPTEKLPFPIIDDR	3	1	1	N-Term(iTRAQ8plex); C7(Methylthio); K13(iTRAQ8plex)	-	12.498	6.153	5.04	35	1	3246.62246	3	1082.879272	-1.213004056	45.95520333
														DINAYNCEEPTEK	2	1	1	N-Term(iTRAQ8plex); C7(Methylthio); K13(iTRAQ8plex)	1.109	2.209	2.279	4.35	21	0	2180.03098	3	727.3485107	-6.895921931	34.209335
														LIALSIDSVEDHLAWSK	4	1	1	N-Term(iTRAQ8plex); K17(iTRAQ8plex)	0.68	4.159	3.505	4.32	44	0	2505.41958	4	627.1103516	0.884111396	64.42124
														DFTPVCTTELGR	4	1	1	N-Term(iTRAQ8plex); C6(Methylthio)	0.944	5.677	5.744	3.94	39	0	1688.83672	2	844.9219971	4.586127566	34.70786667
														LSILYPATTGR	4	1	1	N-Term(iTRAQ8plex)	0.895	3.162	3.122	3.51	37	0	1495.87639	2	748.4418335	-1.478015046	35.57941167
														LKLSILYPATTGR	2	1	1	N-Term(iTRAQ8plex); K2(iTRAQ8plex)	0.892	3.476	3.26	3.31	-	1	2041.24692	3	681.0871582	-7.877508819	33.56768167
														LPFPIIDDR	4	1	1	N-Term(iTRAQ8plex)	0.675	7.501	6.533	2.9	51	0	1389.79822	3	463.9375916	-4.439415522	44.57651333
														NFDEILR	1	1	1	N-Term(iTRAQ8plex)	0.912	3.242	3.015	2.72	-	0	1210.66557	2	605.8364258	-6.431468492	30.81309667
4758714	MGST3	microsomal glutathione S-transferase 3	NP_004519.1	15.79	2	2	9	152	16.5	9.38	0.301	0.257	0.315																
														VEYPIMYSTDPENGHIFNCIQR	8	1	1	N-Term(iTRAQ8plex); C19(Methylthio)	0.301	0.253	0.315	6.08	82	0	2976.39695	3	992.803833	-0.905970074	46.90904833
														YKVEYPIMYSTDPENGHIFNCIQR	1	1	1	N-Term(iTRAQ8plex); K2(iTRAQ8plex); C21(Methylthio)	-	0.359	-	3.88	-	1	3571.76015	4	893.6954956	-0.879050661	44.91281333
4758774	NDUFB10	NADH dehydrogenase [ubiquinone] 1 beta subcomplex subunit 10	NP_004539.1	29.07	5	5	11	172	20.8	8.48	1.112	0.714	0.582																
														VDQEIINIMQDR	2	1	1	N-Term(iTRAQ8plex)	0.877	0.473	0.344	4.01	57	0	1777.94634	2	889.4768066	2.41591164	44.73840167
														DYKVDQEIINIMQDR	4	1	1	N-Term(iTRAQ8plex); K3(iTRAQ8plex)	-	0.225	0.398	3.92	42	1	2488.32461	4	622.8366089	-3.229334753	47.26175
														EVEQFTQVAK	2	1	1	N-Term(iTRAQ8plex); K10(iTRAQ8plex)	1.241	0.739	0.682	3.8	42	0	1787.01286	2	894.0100708	-1.727082383	21.56973167
														YQDLGAYSSAR	2	1	1	N-Term(iTRAQ8plex)	1.236	0.766	0.582	3.32	43	0	1534.77532	2	767.8912964	-3.26767476	20.52236333
														AFDLIVDRPVTLVR	1	1	1	N-Term(iTRAQ8plex)	1	0.714	0.61	2.76	-	0	1918.14292	3	640.0524902	0.06055499	42.33076833
4758788	NDUFS3	NADH dehydrogenase [ubiquinone] iron-sulfur protein 3, mitochondrial precursor	NP_004542.1	27.65	5	5	17	264	30.2	7.50	1.117	0.670	0.639																
														KFDLNSPWEAFPVYR	5	1	1	N-Term(iTRAQ8plex); K1(iTRAQ8plex)	1.053	0.76	0.726	4.89	50	1	2477.34739	3	826.4508057	-2.392712718	44.59902167
														ILTDYGFEGHPFR	2	1	1	N-Term(iTRAQ8plex)	0.987	0.564	0.548	4.79	57	0	1855.9581	3	619.3242188	-3.431273578	39.05143833
														VVAEPVELAQEFR	6	1	1	N-Term(iTRAQ8plex)	1.117	0.626	0.565	4.46	61	0	1790.99419	2	896.0007324	-0.710820983	46.87590833
														QLSAFGEYVAEILPK	2	1	1	N-Term(iTRAQ8plex); K15(iTRAQ8plex)	1.323	1.295	1.727	4.36	37	0	2273.30509	3	758.4398804	2.153940229	55.859785
														TYTDELTPIESAVSVFK	2	1	1	N-Term(iTRAQ8plex); K17(iTRAQ8plex)	1.608	0.938	0.774	3.24	24	0	2508.36582	3	836.793457	-1.424615271	49.42136167
4758792	NDUFS6	NADH dehydrogenase [ubiquinone] iron-sulfur protein 6, mitochondrial precursor	NP_004544.1	29.03	2	2	3	124	13.7	8.28	0.927	0.646	0.512																
														QKEVNENFAIDLIAEQPVSEVETR	1	1	1	N-Term(iTRAQ8plex); K2(iTRAQ8plex)	-	-	-	4.26	-	1	3366.79384	4	842.4539185	-1.897161564	40.90567333
														TGTCGYCGLQFR	2	1	1	N-Term(iTRAQ8plex); C4(Methylthio); C7(Methylthio)	0.927	0.646	0.512	2.92	22	0	1701.74639	2	851.3768311	-3.477492695	34.05659167
4758796	DRG1	developmentally-regulated GTP-binding protein 1	NP_004138.1	3.81	1	1	4	367	40.5	8.90	1.301	2.641	2.024																
														IQLLDLPGIIEGAK	4	1	1	N-Term(iTRAQ8plex); K14(iTRAQ8plex)	1.301	2.641	2.024	4.22	47	0	2088.27756	3	696.7640381	-5.435241176	49.944555
4758874	TM9SF2	transmembrane 9 superfamily member 2 precursor	NP_004791.1	7.99	3	3	11	663	75.7	7.44	1.278	0.894	0.607																
														FCNPGFPIGCYITDK	4	1	1	N-Term(iTRAQ8plex); C2(Methylthio); C10(Methylthio); K15(iTRAQ8plex)	1.257	0.764	0.561	4.59	43	0	2375.1489	3	792.3878174	-1.176516371	48.97854167
														LDSVESVLPYEYTAFDFCQASEGK	4	1	1	N-Term(iTRAQ8plex); C18(Methylthio); K24(iTRAQ8plex)	1.637	-	0.493	4.44	55	0	3352.65158	3	1118.222046	8.99352186	63.340765
														RPSENLGQVLFGER	3	1	1	N-Term(iTRAQ8plex)	1.208	1.034	0.784	2.76	32	0	1906.03983	3	636.0181274	-2.645393713	29.21984667
4758876	PABPN1	polyadenylate-binding protein 2	NP_004634.1	19.61	4	4	8	306	32.7	5.06	1.208	1.201	0.848																
														SIYVGNVDYGATAEELEAHFHGCGSVNR	4	2	1	N-Term(iTRAQ8plex); C23(Methylthio)	-	-	-	6.25	45	0	3345.54726	4	837.1422729	-2.941337352	48.13534833
														TSLALDESLFR	2	2	1	N-Term(iTRAQ8plex)	1.306	1.123	0.731	3.92	36	0	1555.86052	2	778.4338989	-1.825400413	40.98395667
														VTILCDK	1	4	1	N-Term(iTRAQ8plex); C5(Methylthio); K7(iTRAQ8plex)	1.208	1.282	0.996	3.43	-	0	1445.83344	3	482.6159973	1.249378601	49.019015
														GFAYIEFSDKESVR	1	2	1	N-Term(iTRAQ8plex); K10(iTRAQ8plex)	1.092	1.201	0.848	2.77	-	1	2256.20414	3	752.739563	-3.530525022	32.04809333
4758898	PEX16	peroxisomal biogenesis factor 16 isoform 1	NP_004804.1	3.87	1	1	2	336	38.6	9.85	1.514	0.777	0.593																
														HPAATAQLETAVR	2	2	1	N-Term(iTRAQ8plex)	1.514	0.777	0.593	4.01	27	0	1668.93052	3	556.9816895	-1.795275836	16.14940833
4758950	PPIB	peptidyl-prolyl cis-trans isomerase B precursor	NP_000933.1	54.63	13	13	103	216	23.7	9.41	0.776	0.674	0.767																
														TVDNFVALATGEK	16	1	1	N-Term(iTRAQ8plex); K13(iTRAQ8plex)	0.738	0.625	0.709	6.61	75	0	1973.10515	2	987.0562134	-5.707329985	66.243035
														IEVEKPFAIAKE	1	1	1	N-Term(iTRAQ8plex); K5(iTRAQ8plex); K11(iTRAQ8plex)	0.542	0.491	0.6	4.87	-	1	2286.37661	4	572.3496094	-3.080717313	31.52102333
														HYGPGWVSMANAGK	3	1	1	N-Term(iTRAQ8plex); K14(iTRAQ8plex)	0.617	0.924	0.933	4.64	37	0	2083.11289	3	695.0424805	6.003926298	25.91933
														DTNGSQFFITTVK	5	1	1	N-Term(iTRAQ8plex); K13(iTRAQ8plex)	0.71	0.603	0.71	4.3	46	0	2066.1229	2	1033.568848	-3.602196373	25.63005
														VIKDFMIQGGDFTR	28	1	1	N-Term(iTRAQ8plex); K3(iTRAQ8plex)	0.877	0.758	0.928	4.04	54	1	2235.24468	2	1118.125977	1.35341112	44.78206
														VIFGLFGK	8	1	1	N-Term(iTRAQ8plex); K8(iTRAQ8plex)	0.798	0.57	0.715	3.69	29	0	1488.92998	2	744.9730835	-0.658809988	71.36337167
														DVIIADCGK	6	1	1	N-Term(iTRAQ8plex); C7(Methylthio); K9(iTRAQ8plex)	0.701	1.061	1.235	3.61	33	0	1587.85466	2	794.4309692	-9.33992499	41.56650167
														DFMIQGGDFTR	4	1	1	N-Term(iTRAQ8plex)	0.655	0.469	0.66	3.35	35	0	1590.78569	2	795.8947144	-4.198694997	25.29669833
														VLEGMEVVR	12	1	1	N-Term(iTRAQ8plex)	0.769	0.723	0.744	3.3	67	0	1335.75591	3	445.9254456	0.720278072	34.61002
														VLEGMEVVR	2	1	1	N-Term(iTRAQ8plex); M5(Oxidation)	0.877	0.593	0.708	3.14	38	0	1351.76053	3	445.9254456	0.720278072	34.61002

														TAWLDGK	3	1	1	N-Term(iTRAQ8plex); K7(iTRAQ8plex)	0.786	0.741	0.818	3.13	-	0	1398.82121	2	699.9142456	0.767282428	27.69483
														DFMIQGGDFTR	3	1	1	N-Term(iTRAQ8plex); M3(Oxidation)	0.737	0.594	0.712	3.11	27	0	1606.77739	2	795.8947144	-4.198694997	25.29669833
														VIKDFMIQGGDFTR	2	1	1	N-Term(iTRAQ8plex); K3(iTRAQ8plex); M6(Oxidation)	1.047	0.78	0.785	3	-	1	2251.22788	2	1118.125977	1.35341112	44.78206
														DKPLKDVIIADCGK	1	1	1	N-Term(iTRAQ8plex); K2(iTRAQ8plex); K5(iTRAQ8plex); C12(Methylthio); K14(iTRAQ8plex)	1.64	0.956	0.882	2.79	-	1	2777.62856	4	695.1625977	-1.937463877	34.87305833
														VYFDLR	5	1	1	N-Term(iTRAQ8plex)	0.804	0.668	0.846	-	34	0	1116.63078	2	558.8190308	-4.223774435	36.590105
														IGDEDVGR	3	1	1	N-Term(iTRAQ8plex)	0.776	0.652	0.718	-	31	0	1164.61406	2	582.8106689	-1.880476501	15.59237333
														HYGPGWVSMANAGK	1	1	1	N-Term(iTRAQ8plex); Y2(iTRAQ8plex); K14(iTRAQ8plex)	0.752	0.679	0.693	-	31	0	2387.31571	3	695.0424805	6.003926298	25.91933
4758970	PSMB8	proteasome subunit beta type-8 isoform E1 precursor	NP_004150.1	4.41	1	1	2	272	29.8	5.82	1.184	2.498	1.008																
														FQHGVIAAVDSR	2	2	1	N-Term(iTRAQ8plex)	1.184	2.498	1.008	3.74	39	0	1603.88376	3	535.2994385	-1.290068745	24.67744
4759156	SNRPA	U1 small nuclear ribonucleoprotein A	NP_004587.1	9.22	2	2	12	282	31.3	9.83	0.693	0.968	0.877																
														HDIAFVEFDNEVQAGAAR	10	1	1	N-Term(iTRAQ8plex)	0.652	1.027	1.004	6.26	67	0	2293.14731	3	765.0539551	-1.844327926	34.76757333
														GQAFVIFK	2	2	1	N-Term(iTRAQ8plex); K8(iTRAQ8plex)	0.85	0.817	0.721	3.47	28	0	1517.93076	3	506.6484375	0.479567329	44.40900333
4759160	SNRPD3	small nuclear ribonucleoprotein Sm D3	NP_004166.1	31.75	3	3	51	126	13.9	10.32	0.916	0.910	0.882																
														VLHEAEGHIVTCETNTGEVYR	21	1	1	N-Term(iTRAQ8plex); C12(Methylthio)	0.731	0.951	0.886	6.09	48	0	2707.30288	3	903.105896	-3.349296797	35.08874667
														VAQLEQVYIR	10	1	1	N-Term(iTRAQ8plex)	0.916	0.76	0.84	4.11	41	0	1522.88481	2	761.9460449	-3.07841815	34.84338167
														FLILPDMLK	20	1	1	N-Term(iTRAQ8plex); K9(iTRAQ8plex)	0.978	0.948	0.909	3.46	42	0	1698.04717	2	849.5272217	-0.7564929	47.15713667
4759202	SYNGR2	synaptogyrin-2 isoform 1	NP_004701.1	4.46	1	1	1	224	24.8	4.94	0.956	1.399	0.947																
														DVLVGADSVR	1	2	1	N-Term(iTRAQ8plex)	0.956	1.399	0.947	-	25	0	1334.75176	2	667.8795166	-4.805925029	17.13620333
4759212	TBCA	tubulin-specific chaperone A isoform 2	NP_004598.1	18.52	2	2	7	108	12.8	5.29	1.190	1.540	1.450																
														RLEAAYLDLQR	6	2	1	N-Term(iTRAQ8plex)	1.209	1.473	1.412	3.72	39	1	1651.93503	3	551.3165283	-5.030215416	28.49949167
														QAEILQESR	1	2	1	N-Term(iTRAQ8plex)	0.916	1.54	1.614	2.97	-	0	1377.75701	2	689.3821411	-5.055821461	19.05122667
4759224	PDCD5	programmed cell death protein 5	NP_004699.1	29.60	3	3	4	125	14.3	6.04	0.942	1.227	1.931																
														NSILAQVLDQSAR	2	2	1	N-Term(iTRAQ8plex)	0.834	1.874	3.207	3.99	37	0	1718.96977	2	859.9885254	-0.300842259	40.75574167
														HGDPGDAAQQEAK	1	2	1	N-Term(iTRAQ8plex); K13(iTRAQ8plex)	0.942	0.952	1.402	3.7	-	0	1931.98661	3	644.6670532	-8.574014942	9.943938333
														VSEQGLIEILK	1	1	1	N-Term(iTRAQ8plex); K11(iTRAQ8plex)	0.965	1.227	1.931	3.48	-	0	1837.11325	3	613.0426025	-6.671843874	34.84196333
4759264	COPS2	COP9 signalosome complex subunit 2 isoform 1	NP_004227.1	2.26	1	1	2	443	51.6	5.53	0.574	1.182	1.002																
														AHTDFFEAFK	2	2	1	N-Term(iTRAQ8plex); K10(iTRAQ8plex)	0.574	1.182	1.002	4.78	33	0	1820.96244	3	607.6589966	-9.19008738	32.426025
4759270	TSN	translin isoform 1	NP_004613.1	5.26	1	1	2	228	26.2	6.44	1.178	1.965	1.925																
														KVEEVVYDLSIR	2	1	1	N-Term(iTRAQ8plex); K1(iTRAQ8plex)	1.178	1.965	1.925	3.71	-	1	2058.19059	3	686.7350464	-7.271139003	45.13909833
4759276	RRP9	U3 small nucleolar RNA-interacting protein 2	NP_004695.1	7.79	2	2	3	475	51.8	7.85	1.407	0.785	0.786																
														VWNVAENSYVETLFGHQDAVAALDALSR	2	1	1	N-Term(iTRAQ8plex)	-	-	-	4.48	43	0	3379.73764	3	1127.250732	4.471564724	56.843455
														ECCVTAGGR	1	1	1	N-Term(iTRAQ8plex); C2(Methylthio); C3(Methylthio)	1.407	0.785	0.786	-	21	0	1291.5479	2	646.2775879	-6.97158716	30.18259
4759300	VAMP3	vesicle-associated membrane protein 3	NP_004772.1	17.00	1	1	2	100	11.3	8.79	0.608	0.521	0.275																
														ADALQAGASQFETSAAK	2	4	1	N-Term(iTRAQ8plex); K17(iTRAQ8plex)	0.608	0.521	0.275	3.31	25	0	2274.21036	3	758.7416382	-3.649618065	25.61186
4759302	VAPB	vesicle-associated membrane protein-associated protein B/C isoform 1	NP_004729.1	21.81	3	4	8	243	27.2	7.30	2.387	1.249	1.241																
														FMVQSMFAPTDTSDMEAVWK	2	1	1	N-Term(iTRAQ8plex); K20(iTRAQ8plex)	3.398	1.473	1.428	6.58	61	0	2929.44577	3	977.1534424	2.127569144	52.66791833
														CVFELPAENDKPHDVEINK	2	1	1	N-Term(iTRAQ8plex); C1(Methylthio); K11(iTRAQ8plex); K19(iTRAQ8plex)	3.232	-	1.033	4.08	-	0	3155.64174	4	789.6658936	-6.831561691	35.15727833
														FRGPFTDVVTTNLK	2	2	1	N-Term(iTRAQ8plex); K14(iTRAQ8plex)	1.68	1.091	1.268	3.74	-	1	2203.26908	3	735.0945435	-0.219754778	36.349815
														GPFTDVVTTNLK	2	4	2	N-Term(iTRAQ8plex); K12(iTRAQ8plex)	-	-	-	3.86	51	0	1900.09343	2	950.550354	-3.468204289	26.99260833
4826649	MRPL49	39S ribosomal protein L49, mitochondrial	NP_004918.1	10.84	2	2	5	166	19.2	9.45	0.945	0.535	0.466																
														DVEDFLSPLLGK	4	1	1	N-Term(iTRAQ8plex); K12(iTRAQ8plex)	0.897	0.488	0.44	4.68	55	0	1941.10747	2	971.057373	-4.060479069	44.366945
														AWLLEK	1	1	1	N-Term(iTRAQ8plex); K6(iTRAQ8plex)	1.012	0.637	0.658	-	28	0	1367.8521	2	684.4296875	1.014057003	27.79849833
4826686	DDX1	ATP-dependent RNA helicase DDX1	NP_004930.1	12.84	6	6	14	740	82.4	7.23	1.072	1.286	1.528																
														GHVDILAPTVQELAALEK	2	1	1	N-Term(iTRAQ8plex); K18(iTRAQ8plex)	1.1	1.297	1.484	5.19	29	0	2512.44521	4	628.8667603	-5.715612946	44.74517333
														GSAFAIGSDGLCCQSR	2	1	1	N-Term(iTRAQ8plex); C12(Methylthio); C13(Methylthio)	1.078	1.276	1.596	5.04	48	0	1967.88896	2	984.4481201	7.109300694	38.90227
														GIDIHGVPYVINVTLPDEK	2	1	1	N-Term(iTRAQ8plex); K19(iTRAQ8plex)	0.803	1.134	0.997	4.87	27	0	2687.51346	3	896.5093384	-3.508601321	43.246385
														ELAEQTLNNIK	2	1	1	N-Term(iTRAQ8plex); K11(iTRAQ8plex)	1.027	0.939	1.215	4.17	47	0	1881.07285	3	627.6958008	-9.214000541	42.80029333
														FLICTDVAAR	4	1	1	N-Term(iTRAQ8plex); C4(Methylthio)	1.079	1.388	1.606	3.7	32	0	1458.77727	2	729.8922729	1.499185985	44.005865
														FLVLDEADGLLSQGYSDFINR	2	1	1	N-Term(iTRAQ8plex)	-	-	-	2.96	74	0	2676.39127	3	892.8019409	3.339647685	69.686695
4826754	FGFBP1	fibroblast growth factor-binding protein 1 precursor	NP_005121.1	5.56	1	1	2	234	26.2	9.10	0.917	0.780	0.509																
														WAATEQEEGISLK	2	1	1	N-Term(iTRAQ8plex); K13(iTRAQ8plex)	0.917	0.78	0.509	4.11	45	0	2070.12925	3	690.7145996	-1.712802175	43.88158
4826774	ISG15	ubiquitin-like protein ISG15 precursor	NP_005092.1	5.45	1	1	1	165	17.9	7.44	1.124	2.082	0.546																
														LTQTVAHLK	1	1	1	N-Term(iTRAQ8plex); K9(iTRAQ8plex)	1.124	2.082	0.546	3.21	-	0	1619.00168	3	540.3387451	-5.175534115	22.44862667
4826834	MMP14	matrix metalloproteinase-14 preproprotein	NP_004986.1	7.22	3	3	6	582	65.9	7.77	0.541	0.513	0.414																
														GSFMGSDEVFTYFYK	4	1	1	N-Term(iTRAQ8plex); K15(iTRAQ8plex)	0.383	0.615	0.476	3.47	36	0	2386.1876	3	796.0673828	-0.413258581	51.53552333
														NNQVMDGYPMPIGQFWR	1	1	1	N-Term(iTRAQ8plex)	0.718	0.661	0.569	3.35	-	0	2357.15549	3	786.3900146	3.465785496	46.03238833
														VWEGIPESPR	1	1	1	N-Term(iTRAQ8plex)	0.61	0.394	0.246	-	29	0	1473.79729	2	737.4022827	-2.091529368	23.63272667
4826852	NDUFAB1	acyl carrier protein, mitochondrial precursor	NP_004994.1	13.46	3	3	13	156	17.4	4.93	1.238	0.746	0.369																
														LMCPQEIVDYIADKK	4	2	1	N-Term(iTRAQ8plex); M2(Oxidation); C3(Methylthio); K14(iTRAQ8plex); K15(iTRAQ8plex)	1.125	0.896	0.136	5.73	53	1	2740.50429	4	681.8804321	3.56261185	61.44283667
														LMCPQEIVDYIADK	2	2	1	N-Term(iTRAQ8plex); C3(Methylthio); K14(iTRAQ8plex)	0.966	0.573	0.334	5.47	42	0	2292.20157	3	764.7387085	5.108598835	61.04137167
														LMCPQEIVDYIADKK	6	2	1	N-Term(iTRAQ8plex); C3(Methylthio); K14(iTRAQ8plex); K15(iTRAQ8plex)	1.79	1.214	0.997	4.74	57	1	2724.4999	4	681.8804321	3.56261185	61.44283667
														VLYVLK	1	2	1	N-Term(iTRAQ8plex); K6(iTRAQ8plex)	1.023	0.811	0.369	-	25	0	1342.89347	3	448.3026733	1.217869899	35.63449167
4826898	PFN1	profilin-1	NP_005013.1	62.14	9	9	142	140	15.0	8.27	0.884	2.110	3.294																
														DSLLQDGEFSMDLR	10	2	1	N-Term(iTRAQ8plex); M11(Oxidation)	0.842	1.563	2.33	5.77	57	0	1945.94036	3	643.993103	6.085360883	40.19545667
														DSLLQDGEFSMDLR	27	2	1	N-Term(iTRAQ8plex)	1.023	1.972	2.765	5.71	73	0	1929.96476	3	643.993103	6.085360883	40.19545667
														TFVNITPAEVGVLVGK	31	2	1	N-Term(iTRAQ8plex); K16(iTRAQ8plex)	0.889	2.775	4.936	5.49	76	0	2252.33054	2	1126.67395	-3.049063406	50.34444667
														STGGAPTFNVTVTK	15	2	1	N-Term(iTRAQ8plex); K14(iTRAQ8plex)	0.826	4.333	8.326	5.06	59	0	1988.1104	2	994.5588379	-8.501609748	34.67853
														CYEMASHLR	31	1	1	N-Term(iTRAQ8plex); C1(Methylthio)	0.753	2.288	4.278	4.54	59	0	1459.67136	3	487.234314	5.896014929	31.833585
														CYEMASHLR	6	1	1	N-Term(iTRAQ8plex); C1(Methylthio); M4(Oxidation)	1.343	1.139	1.753	3.79	36	0	1475.66544	3	487.234314	5.896014929	31.833585
														KCYEMASHLR	2	1	1	N-Term(iTRAQ8plex); K1(iTRAQ8plex); C2(Methylthio)	-	1.015	4.586	3.77	30	1	1891.98781	4	473.7524109	4.070520239	29.54551167
														DSPSVWAAVPGK	6	2	1	N-Term(iTRAQ8plex); K12(iTRAQ8plex)	0.605	2.149	3.757	3.66	50	0	1822.01702	2	911.512146	-8.197468896	37.21600333
														EGVHGGLINK	4	1	1	N-Term(iTRAQ8plex); K10(iTRAQ8plex)	0.54	1.338	2.965	3.33	57	0	1631.96147	2	816.484375	-4.576039399	19.033475
														TDKTLVLLMGK	3	1	1	N-Term(iTRAQ8plex); K3(iTRAQ8plex); K11(iTRAQ8plex)	0.919	4.884	6.597	3.07	29	1	2131.30753	3	711.1073608	-9.966666085	42.38340667
														TLVLLMGK	2	1	1	N-Term(iTRAQ8plex); K8(iTRAQ8plex)	1.184	2.329	3.461	2.71	22	0	1482.94315	3	494.9859009	-7.189697923	36.3774
														CYEMASHLR	3	1	1	N-Term(iTRAQ8plex); C1(Methylthio); Y2(iTRAQ8plex)	-	-	-	-	31	0	1763.88393	3	487.234314	5.896014929	31.833585
														CYEMASHLR	2	1	1	N-Term(iTRAQ8plex); C1(Methylthio); Y2(iTRAQ8plex); M4(Oxidation)	0.778	1.601	3.087	-	26	0	1779.86635	3	487.234314	5.896014929	31.833585
4826972	RBM8A	RNA-binding protein 8A	NP_005096.1	41.38	6	6	22	174	19.9	5.72	1.261	1.248	1.058																
														MREDYDSVEQDGDEPGPQR	4	1	1	N-Term(iTRAQ8plex)	1.911	0.964	1.638	4.98	63	1	2527.12461	3	843.0463867	-2.510518766	19.41065
														GYTLVEYETYK	10	1	1	N-Term(iTRAQ8plex); K11(iTRAQ8plex)	1.282	1.217	0.934	4.3	50	0	1974.05124	2	987.5352173	-2.458558465	30.90556167
														SVEGWILFVTGVHEEATEEDIHDK	1	1	1	N-Term(iTRAQ8plex); K24(iTRAQ8plex)	-	-	-	3.6	-	0	3348.7394	4	837.9403076	5.521268316	60.78268833
														NIHLNLDRR	2	1	1	N-Term(iTRAQ8plex)	1.344	2.676	2.088	3.01	-	1	1454.84157	4	364.4658508	-5.35993735	15.94385333
														MREDYDSVEQDGDEPGPQR	2	1	1	N-Term(iTRAQ8plex); M1(Oxidation)	1.341	-	1.612	2.99	27	1	2543.11814	3	843.0463867	-2.510518766	19.41065
														NIHLNLDR	2	1	1	N-Term(iTRAQ8plex)	1.154	1.331	1.246	2.85	-	0	1298.74308	3	433.5858765	-3.984741527	22.79123
														GFGSEEGSR	1	1	1	N-Term(iTRAQ8plex)	1.24	1.012	0.84	-	20	0	1229.60246	2	615.3048706	-3.209743943	12.15674333
4885063	ALDOC	fructose-bisphosphate aldolase C	NP_005156.1	16.76	1	4	21	364	39.4	6.87	-	-	-																
														VDKGVVPLAGTDGETTTQGLDGLSER	2	1	1	N-Term(iTRAQ8plex); K3(iTRAQ8plex)	-	-	-	3.72	40	1	3223.72719	4	806.6872559	0.140290252	31.76995667
														YASICQQNGIVPIVEPEILPDGDHDLK	4	4	2	N-Term(iTRAQ8plex); C5(Methylthio); K27(iTRAQ8plex)	-	-	-	5.14	43	0	3617.88205	3	1206.632202	0.436967068	45.759295
														YASICQQNGIVPIVEPEILPDGDHDLKR	13	4	2	N-Term(iTRAQ8plex); C5(Methylthio); K27(iTRAQ8plex)	-	-	-	5.07	34	1	3773.97573	4	944.2493896	-1.553689444	48.23959167
														DGADFAK	2	4	2	N-Term(iTRAQ8plex); K7(iTRAQ8plex)	-	-	-	2.98	24	0	1331.72998	3	444.5815125	-8.693351222	16.29871167
4885243	FOSL1	fos-related antigen 1 isoform 1	NP_005429.1	4.80	1	1	1	271	29.4	8.02	0.797	1.195	1.075																
														RPCEQISPEEEER	1	1	1	N-Term(iTRAQ8plex); C3(Methylthio)	0.797	1.195	1.075	2.97	-	0	1951.91013	3	651.3082275	-2.788516095	19.43014167
4885281	GLUD1	glutamate dehydrogenase 1, mitochondrial isoform a precursor	NP_005262.1	42.65	18	18	82	558	61.4	7.80	0.871	0.497	0.590																
														IIAEGANGPTTPEADKIFLER	4	5	1	N-Term(iTRAQ8plex); K16(iTRAQ8plex)	1.234	0.536	0.639	5.51	27	1	2850.57932	3	950.864624	-1.021715013	37.67926833
														KGFIGPGIDVPAPDMSTGER	4	3	1	N-Term(iTRAQ8plex); K1(iTRAQ8plex)	0.932	0.647	0.604	5.48	48	1	2652.42057	3	884.8117065	-2.671115276	31.772835
														CIAVGESDGSIWNPDGIDPK	4	4	1	N-Term(iTRAQ8plex); C1(Methylthio); K20(iTRAQ8plex)	0.855	0.55	0.862	5.43	67	0	2727.37847	2	1364.192871	7.539572435	42.38870833
														DDGSWEVIEGYR	10	3	1	N-Term(iTRAQ8plex)	0.878	0.455	0.502	4.88	45	0	1729.84201	3	577.2855225	4.909600266	32.783945
														HGGTIPIVPTAEFQDR	16	4	1	N-Term(iTRAQ8plex)	0.838	0.51	0.746	4.73	60	0	2042.09001	3	681.3695068	-1.632658556	26.55274333
														AKPYEGSILEADCDILIPAASEK	1	3	1	N-Term(iTRAQ8plex); K2(iTRAQ8plex); C13(Methylthio); K23(iTRAQ8plex)	-	-	-	4.58	-	0	3391.8124	4	848.7085571	-3.919339519	52.059425
														GASIVEDKLVEDLR	3	1	1	N-Term(iTRAQ8plex); K8(iTRAQ8plex)	1.182	0.387	0.878	4.56	26	1	2152.23549	4	538.8143311	-3.683129133	42.59685833
														YSTDVSVDEVK	4	4	1	N-Term(iTRAQ8plex); K11(iTRAQ8plex)	0.828	0.831	0.752	4.42	50	0	1849.99797	2	925.5026245	-1.285891443	20.60816833
														ALASLMTYK	8	4	1	N-Term(iTRAQ8plex); K9(iTRAQ8plex)	0.875	0.474	0.492	4.32	38	0	1605.94809	2	803.4765625	-2.237260991	61.11342167
														DSNYHLLMSVQESLER	2	4	1	N-Term(iTRAQ8plex)	0.884	0.649	0.883	4.2	33	0	2225.10807	3	742.3742065	-4.208748885	41.68495167
														LQHGSILGFPK	1	4	1	N-Term(iTRAQ8plex); K11(iTRAQ8plex)	0.753	0.698	0.911	4.09	-	0	1805.07969	3	602.3647461	-5.377196824	30.24431833
														CAVVDVPFGGAK	6	5	1	N-Term(iTRAQ8plex); C1(Methylthio); K12(iTRAQ8plex)	0.909	0.46	0.568	4.08	48	0	1816.97612	2	908.9916992	-8.200703532	61.30188833
														KGFIGPGIDVPAPDMSTGER	1	3	1	N-Term(iTRAQ8plex); K1(iTRAQ8plex); M15(Oxidation)	0.702	0.479	0.731	3.8	-	1	2668.41849	3	884.8117065	-2.671115276	31.772835
														DIVHSGLAYTMER	4	4	1	N-Term(iTRAQ8plex)	0.913	0.904	0.747	3.64	41	0	1795.92766	2	898.4674683	-2.11667577	27.50355333
														IIKPCNHVLSLSFPIR	1	3	1	N-Term(iTRAQ8plex); K3(iTRAQ8plex); C5(Methylthio)	0.304	0.711	0.634	3.38	-	0	2491.44106	4	623.6157227	-3.665169987	47.12326667
														FTMELAK	3	5	1	N-Term(iTRAQ8plex); K7(iTRAQ8plex)	0.994	0.407	0.507	3.13	28	0	1447.84394	3	483.2861633	0.010664295	37.98710333
														YNLGLDLR	2	5	1	N-Term(iTRAQ8plex)	0.828	0.473	0.486	3.07	30	0	1267.73418	2	634.3707275	2.348408819	31.929145
														IIKPCNHVLSLSFPIRR	2	3	1	N-Term(iTRAQ8plex); K3(iTRAQ8plex); C5(Methylthio)	0.407	-	0.317	2.91	23	1	2647.54163	5	530.3141479	-3.655868442	47.18858333
														DIVHSGLAYTMER	1	4	1	N-Term(iTRAQ8plex); M11(Oxidation)	0.985	0.92	0.912	2.8	-	0	1811.92673	2	898.4674683	-2.11667577	27.50355333
														DSNYHLLMSVQESLER	1	4	1	N-Term(iTRAQ8plex); Y4(iTRAQ8plex)	-	-	-	-	25	0	2529.31045	3	742.3742065	-4.208748885	41.68495167
														MVEGFFDR	4	3	1	N-Term(iTRAQ8plex)	0.851	0.481	0.541	-	25	0	1304.6541	2	652.8306885	-5.366147911	55.78598667
4885371	H1F0	histone H1.0	NP_005309.1	16.49	3	3	19	194	20.9	10.84	0.466	1.052	0.697																
														YSDMIVAAIQAEK	14	1	1	N-Term(iTRAQ8plex); K13(iTRAQ8plex)	0.324	1.045	0.697	5.24	59	0	2047.12822	2	1024.067749	-3.521945059	70.64127333

														YSDMIVAAIQAEK	2	1	1	N-Term(iTRAQ8plex); M4(Oxidation); K13(iTRAQ8plex)	0.466	1.012	1.048	4.11	25	0	2063.12308	2	1024.067749	-3.521945059	70.64127333
														GVGASGSFR	2	1	1	N-Term(iTRAQ8plex)	0.531	1.061	0.515	-	37	0	1141.62541	2	571.3163452	-1.171566695	17.450545
														VGENADSQIK	1	1	1	N-Term(iTRAQ8plex); K10(iTRAQ8plex)	0.744	1.288	0.851	-	32	0	1668.94242	3	556.9856567	2.825540771	12.960125
4885379	HIST1H1E	histone H1.4	NP_005312.1	33.79	6	10	59	219	21.9	11.03	1.201	0.632	0.863																
														ASGPPVSELITK	11	3	1	N-Term(iTRAQ8plex); K12(iTRAQ8plex)	1.229	0.609	0.833	5.34	62	0	1807.07573	2	904.0415039	-1.566125565	24.092075
														ALAAAGYDVEK	26	5	1	N-Term(iTRAQ8plex); K11(iTRAQ8plex)	1.207	0.635	0.861	4.91	70	0	1715.97014	2	858.4887085	-5.070558985	33.45456
														SGVSLAALK	4	3	1	N-Term(iTRAQ8plex); K9(iTRAQ8plex)	1.408	0.678	1.042	3.66	35	0	1453.92176	3	485.3121033	1.303553265	27.239625
														SGVSLAALKK	2	3	1	N-Term(iTRAQ8plex); K9(iTRAQ8plex); K10(iTRAQ8plex)	0.919	0.639	0.872	3.63	20	1	1886.22585	4	472.3119202	3.000427579	30.77497667
														KASGPPVSELITK	2	3	1	N-Term(iTRAQ8plex); K1(iTRAQ8plex); K13(iTRAQ8plex)	2.032	1.038	1.04	3.54	27	1	2239.38444	5	448.6827087	2.476524834	30.61276167
														KPAAAAGAK	2	1	1	N-Term(iTRAQ8plex); K1(iTRAQ8plex); K9(iTRAQ8plex)	1.129	0.242	0.574	3.42	30	0	1697.08755	4	425.0273438	2.264950922	12.799285
														GTGASGSFK	6	6	2	N-Term(iTRAQ8plex); K9(iTRAQ8plex)	-	-	-	3.78	39	0	1419.80811	2	710.406189	-0.084511819	11.25244167
														AVAASK	3	4	2	N-Term(iTRAQ8plex); K6(iTRAQ8plex)	-	-	-	3.2	29	0	1154.73731	3	385.583313	0.038463222	10.99039833
														AASGEAKPK	1	3	2	N-Term(iTRAQ8plex); K7(iTRAQ8plex); K9(iTRAQ8plex)	-	-	-	3.04	-	0	1771.08413	4	443.5264893	0.020242545	10.262005
														GTLVQTK	2	5	2	N-Term(iTRAQ8plex); K7(iTRAQ8plex)	-	-	-	2.95	39	0	1354.85332	2	677.9302979	1.393348711	14.35407833
4885381	HIST1H1B	histone H1.5	NP_005313.1	38.94	9	13	58	226	22.6	10.92	0.817	0.688	0.205																
														NGLSLAALKK	6	1	1	N-Term(iTRAQ8plex); K9(iTRAQ8plex); K10(iTRAQ8plex)	0.834	0.82	0.165	4.8	31	1	1927.24636	2	964.125	-2.084802268	23.75391167
														KATGPPVSELITK	5	1	1	N-Term(iTRAQ8plex); K1(iTRAQ8plex); K13(iTRAQ8plex)	0.856	0.813	0.426	4.79	35	1	2253.39492	2	1127.202881	1.750155943	23.730875
														ALAAGGYDVEK	12	1	1	N-Term(iTRAQ8plex); K11(iTRAQ8plex)	0.689	0.628	0.144	4.38	62	0	1701.95989	2	851.4848022	-0.507313116	18.62211667
														ATGPPVSELITK	7	1	1	N-Term(iTRAQ8plex); K12(iTRAQ8plex)	0.547	0.51	0.121	4.31	63	0	1821.0905	2	911.0488892	-2.037021051	23.29235167
														NGLSLAALK	2	1	1	N-Term(iTRAQ8plex); K9(iTRAQ8plex)	0.788	0.608	0.309	4	36	0	1494.94846	3	498.9876709	1.370044097	26.21394667
														AAGAGAAK	5	1	1	N-Term(iTRAQ8plex); K8(iTRAQ8plex)	1.269	1.164	0.38	3.72	43	0	1224.75162	3	408.9220581	-0.367612164	9.254918333
														KPAAAGVK	5	1	1	N-Term(iTRAQ8plex); K1(iTRAQ8plex); K8(iTRAQ8plex)	0.82	0.6	0.11	2.93	22	0	1654.08169	4	414.2758789	2.300034535	14.23585833
														KAAGAGAAK	3	1	1	N-Term(iTRAQ8plex); K1(iTRAQ8plex); K9(iTRAQ8plex)	1.092	1.312	0.277	2.75	27	1	1657.06264	4	415.0211182	6.180500354	11.38665333
														KAAGAK	1	1	1	N-Term(iTRAQ8plex); K1(iTRAQ8plex); K6(iTRAQ8plex)	0.893	0.662	0.23	-	23	1	1457.9562	4	365.2445068	-0.338718166	10.24203833
														GTGASGSFK	6	6	2	N-Term(iTRAQ8plex); K9(iTRAQ8plex)	-	-	-	3.78	39	0	1419.80811	2	710.406189	-0.084511819	11.25244167
														AVAASK	3	4	2	N-Term(iTRAQ8plex); K6(iTRAQ8plex)	-	-	-	3.2	29	0	1154.73731	3	385.583313	0.038463222	10.99039833
														AASGEAKPK	1	3	2	N-Term(iTRAQ8plex); K7(iTRAQ8plex); K9(iTRAQ8plex)	-	-	-	3.04	-	0	1771.08413	4	443.5264893	0.020242545	10.262005
														GTLVQTK	2	5	2	N-Term(iTRAQ8plex); K7(iTRAQ8plex)	-	-	-	2.95	39	0	1354.85332	2	677.9302979	1.393348711	14.35407833
4885399	SMC3	structural maintenance of chromosomes protein 3	NP_005436.1	6.16	5	5	15	1217	141.5	7.18	1.075	1.628	1.595																
														CDPAPFYLFDEIDQALDAQHR	8	1	1	N-Term(iTRAQ8plex); C1(Methylthio)	0.848	1.508	1.684	6.96	67	0	2814.33139	3	938.7819824	5.124384305	65.89232333
														DTAYPETNDAIPMISK	3	1	1	N-Term(iTRAQ8plex); K16(iTRAQ8plex)	1.185	1.694	1.083	4.3	40	0	2374.252	2	1187.629639	4.173703443	31.81616667
														MNLPGEVTFLPLNK	1	1	1	N-Term(iTRAQ8plex); K14(iTRAQ8plex)	1.145	1.746	1.792	3.88	-	0	2181.24515	3	727.7532349	-5.079649185	37.19850833
														LALLHEGTGPR	2	1	1	N-Term(iTRAQ8plex)	1.272	1.714	1.587	3.66	38	0	1467.85343	3	489.9559937	-3.490534458	28.23473333
														RLDQVEQELNELR	1	1	1	N-Term(iTRAQ8plex)	0.987	1.547	1.206	3.63	-	1	1946.05735	3	649.3572998	-1.83197887	39.49739
4885413	HINT1	histidine triad nucleotide-binding protein 1	NP_005331.1	47.62	4	4	18	126	13.8	6.95	0.876	2.692	2.488																
														CLAFHDISPQAPTHFLVIPK	9	2	1	N-Term(iTRAQ8plex); C1(Methylthio); K20(iTRAQ8plex)	-	7.755	9.008	5.92	45	0	2888.57022	3	963.5282593	-2.550645266	46.84128167
														CAADLGLNK	5	1	1	N-Term(iTRAQ8plex); C1(Methylthio); K9(iTRAQ8plex)	0.811	2.419	2.147	3.97	29	0	1558.84123	2	779.9242554	-8.299868857	25.92332333
														MVVNEGSDGGQSVYHVHLHVLGGR	2	1	1	N-Term(iTRAQ8plex)	-	-	-	3.47	44	0	2851.44399	4	713.6164551	-4.766310524	31.56991
														IIFEDDR	2	2	1	N-Term(iTRAQ8plex)	0.906	2.692	2.488	-	25	0	1211.65471	2	606.3309937	-2.204105913	26.83101667
4885495	DNAJB6	dnaJ homolog subfamily B member 6 isoform b	NP_005485.1	8.71	1	2	5	241	26.9	7.55	1.130	0.915	1.139																
														VDYYEVLGVQR	1	5	1	N-Term(iTRAQ8plex)	1.13	0.915	1.139	-	30	0	1644.88286	2	822.9450684	-4.274590866	33.23005667
														VEVEEDGQLK	4	7	2	N-Term(iTRAQ8plex); K10(iTRAQ8plex)	-	-	-	4.05	52	0	1753.9625	3	585.3256836	-9.546252428	29.382285
4885585	SAE1	SUMO-activating enzyme subunit 1 isoform a	NP_005491.1	21.68	4	4	8	346	38.4	5.30	1.091	1.838	1.513																
														NDVLDSLGISPDLLPEDFVR	2	3	1	N-Term(iTRAQ8plex)	1.091	3.023	3.799	4.31	70	0	2518.34087	2	1259.674072	2.595602261	62.47365833
														GLTMLDHEQVTPEDPGAQFLIR	2	6	1	N-Term(iTRAQ8plex); M4(Oxidation)	-	-	-	3.9	43	0	2787.42844	3	929.8143311	-0.196017924	34.37382833
														TTSDYFLLQVLLK	2	3	1	N-Term(iTRAQ8plex); K13(iTRAQ8plex)	1.568	1.838	1.882	3.42	48	0	2149.26249	3	717.0923462	-4.841649567	62.69298333
														GLTMLDHEQVTPEDPGAQFLIR	1	6	1	N-Term(iTRAQ8plex)	-	-	1.064	2.9	-	0	2771.44057	3	929.8143311	-0.196017924	34.37382833
														VEKEEAGGGISEEEAAQYDR	1	3	1	N-Term(iTRAQ8plex); K3(iTRAQ8plex)	0.146	0.865	1.217	-	32	1	2775.37937	3	925.7979736	-3.611632946	21.88447
5031569	ACTR1A	alpha-centractin	NP_005727.1	4.79	1	1	1	376	42.6	6.64	-	-	-																
														ACYLSINPQKDETLETEK	1	1	1	N-Term(iTRAQ8plex); C2(Methylthio); K10(iTRAQ8plex); K18(iTRAQ8plex)	-	-	-	3.86	-	1	3040.58291	4	760.9011841	-8.858778959	31.91961833
5031571	ACTR2	actin-related protein 2 isoform b	NP_005713.1	26.14	7	7	17	394	44.7	6.74	0.907	1.305	1.421																
														DLMVGDEASELR	2	1	1	N-Term(iTRAQ8plex)	0.856	1.664	1.824	4.95	43	0	1638.83342	2	819.9203491	1.415837728	29.80022667
														HLWDYTFGPEK	4	2	1	N-Term(iTRAQ8plex); K11(iTRAQ8plex)	0.907	1.566	1.814	4.89	37	0	2001.05436	3	667.6896362	-7.339060308	33.48945
														ILLTEPPMNPTK	2	2	1	N-Term(iTRAQ8plex); K12(iTRAQ8plex)	0.826	1.314	1.477	4.35	34	0	1962.15618	2	981.5817261	0.374194698	32.80063
														LALETTVLVESYTLPDGR	2	2	1	N-Term(iTRAQ8plex)	-	-	-	3.98	47	0	2281.26853	3	761.0943604	4.026928253	62.13868667
														GYAFNHSADFETVR	5	2	1	N-Term(iTRAQ8plex)	1.09	1.297	1.367	3.71	30	0	1917.93143	3	639.9819946	-4.317497043	25.07727167
														LCYVGYNIEQEQK	1	2	1	N-Term(iTRAQ8plex); C2(Methylthio); K13(iTRAQ8plex)	0.956	1.185	1.114	3.31	-	0	2241.14133	3	747.7186279	-4.047830815	41.22063167
														CGYAGSNFPEHIFPALVGRPIIR	1	2	1	N-Term(iTRAQ8plex); C1(Methylthio)	-	-	-	2.74	-	0	2864.49209	4	716.878479	-2.986974346	50.78250167
5031573	ACTR3	actin-related protein 3 isoform 1	NP_005712.1	31.82	9	9	29	418	47.3	5.88	0.858	1.377	1.560																
														AEPEDHYFLLTEPPLNTPENR	7	2	1	N-Term(iTRAQ8plex)	1.037	1.402	1.807	6.22	49	0	2786.38108	3	929.46521	-4.620569417	36.67789333
														HGIVEDWDLMER	11	2	1	N-Term(iTRAQ8plex)	1.043	1.278	1.452	4.99	58	0	1803.89133	3	601.9697266	-3.079371943	32.722305
														DREVGIPPEQSLETAK	1	2	1	N-Term(iTRAQ8plex); K16(iTRAQ8plex)	0.262	0.471	0.7	4.07	-	1	2377.30295	3	793.105835	-6.490408459	23.15113
														TLTGTVIDSGDGVTHVIPVAEGYVIGSCIK	2	2	1	N-Term(iTRAQ8plex); C28(Methylthio); K30(iTRAQ8plex)	-	-	-	3.95	-	0	3655.95156	4	914.7433472	-0.569894894	58.37957167
														LPACVVDCGTGYTK	2	1	1	N-Term(iTRAQ8plex); C4(Methylthio); C8(Methylthio); K14(iTRAQ8plex)	0.8	1.041	0.688	3.77	34	0	2127.04502	3	709.6865234	-5.503521273	41.42233667
														FMEQVIFK	2	2	1	N-Term(iTRAQ8plex); K8(iTRAQ8plex)	0.72	1.42	1.725	3.58	31	0	1649.95383	3	550.6561279	-0.430776258	66.19994667
														YSYVCPDLVK	2	2	1	N-Term(iTRAQ8plex); C5(Methylthio); K10(iTRAQ8plex)	0.802	1.624	1.701	3.51	40	0	1840.96184	2	920.9845581	-9.724649733	32.50533167
														NIVLSGGSTMFR	1	2	1	N-Term(iTRAQ8plex)	1.003	1.826	1.583	2.82	-	0	1585.86601	2	793.4366455	-0.880846403	34.53068667
														EFSIDVGYER	1	2	1	N-Term(iTRAQ8plex)	0.603	1.725	1.593	-	25	0	1518.76836	2	759.8878174	-3.84729794	31.20261333
5031593	ARPC5	actin-related protein 2/3 complex subunit 5 isoform 1	NP_005708.1	7.95	1	1	1	151	16.3	5.67	0.672	1.145	1.804																
														ALAAGGVGSIVR	1	2	1	N-Term(iTRAQ8plex)	0.672	1.145	1.804	3.48	-	0	1374.83989	2	687.923584	2.029362668	28.96201
5031595	ARPC4	actin-related protein 2/3 complex subunit 4 isoform a	NP_005709.1	17.86	3	3	7	168	19.7	8.43	0.616	1.269	1.386																
														VLIEGSINSVR	2	3	1	N-Term(iTRAQ8plex)	0.658	1.027	1.254	3.43	27	0	1490.88298	2	745.9451294	-0.971995089	31.19138333
														ELLLQPVTISR	3	3	1	N-Term(iTRAQ8plex)	0.504	1.667	1.553	3.25	36	0	1572.95322	3	524.9892578	-6.014872139	37.66054167
														AENFFILR	2	4	1	N-Term(iTRAQ8plex)	0.616	1.479	1.533	2.98	35	0	1313.74736	2	657.3773193	-3.493250116	41.78193833
5031601	ARPC1B	actin-related protein 2/3 complex subunit 1B	NP_005711.1	11.29	2	2	3	372	40.9	8.35	0.768	2.861	3.589																
														CSQFCTTGMDGGMSIWDVK	2	1	1	N-Term(iTRAQ8plex); C1(Methylthio); C5(Methylthio); K19(iTRAQ8plex)	0.954	1.462	1.819	4.76	54	0	2766.22354	3	922.7460327	-4.102490375	50.21826667
														STVLSLDWHPNNVLLAAGSCDFK	1	3	1	N-Term(iTRAQ8plex); C20(Methylthio); K23(iTRAQ8plex)	0.618	5.6	7.082	3.36	-	0	3141.60825	3	1047.874268	-7.630746332	55.41308833
5031611	BET1	BET1 homolog isoform 1	NP_005859.1	15.25	1	1	4	118	13.3	9.06	0.789	0.582	0.778																
														LLAEMDSQFDSTTGFLGK	4	1	1	N-Term(iTRAQ8plex); K18(iTRAQ8plex)	0.789	0.582	0.778	5.42	64	0	2568.34531	3	856.7866211	-0.907692879	49.57758833
5031635	CFL1	cofilin-1	NP_005498.1	66.27	11	12	126	166	18.5	8.09	1.312	5.225	2.507																
														EILVGDVGQTVDDPYATFVK	2	1	1	N-Term(iTRAQ8plex); K20(iTRAQ8plex)	0.314	1.618	0.964	6.03	71	0	2774.50064	3	925.5050659	-2.402881436	44.03620333
														EDLVFIFWAPESAPLK	4	3	1	N-Term(iTRAQ8plex); K16(iTRAQ8plex)	1.625	6.981	3.587	5.68	53	0	2470.39604	2	1235.70166	4.761338786	60.40918167
														NIILEEGKEILVGDVGQTVDDPYATFVK	23	1	1	N-Term(iTRAQ8plex); K8(iTRAQ8plex); K28(iTRAQ8plex)	-	-	-	5.6	44	1	3975.19619	4	994.5551147	-2.722428898	57.82600167
														YALYDATYETK	28	3	1	N-Term(iTRAQ8plex); K11(iTRAQ8plex)	1.312	4.398	2.308	5.6	54	0	1946.0186	2	973.5168457	-5.291045215	26.570125
														KEDLVFIFWAPESAPLK	16	3	1	N-Term(iTRAQ8plex); K1(iTRAQ8plex); K17(iTRAQ8plex)	2.353	22.221	9.915	5.48	52	1	2902.67485	2	1451.841064	-3.362076101	57.608365
														LGGSAVISLEGKPL	26	1	1	N-Term(iTRAQ8plex); K12(iTRAQ8plex)	1.259	6.804	2.332	5.33	73	0	1949.18169	2	975.0933228	-5.039255158	38.42393
														AVLFCLSEDKK	6	1	1	N-Term(iTRAQ8plex); C5(Methylthio); K10(iTRAQ8plex); K11(iTRAQ8plex)	2.194	9.139	3.206	4.32	37	1	2211.24697	4	553.5679321	-6.581646147	39.40452667
														AVLFCLSEDK	3	1	1	N-Term(iTRAQ8plex); C5(Methylthio); K10(iTRAQ8plex)	0.946	2.025	1.151	3.72	47	0	1778.95122	2	889.979248	-7.253052309	69.915755
														HELQANCYEEVKDR	7	1	1	N-Term(iTRAQ8plex); C7(Methylthio); K12(iTRAQ8plex)	1.378	2.312	1.657	3.37	32	1	2388.17544	4	597.7993164	-5.957081204	23.94088333
														NIILEEGK	4	1	1	N-Term(iTRAQ8plex); K8(iTRAQ8plex)	0.866	7.132	3.531	3.01	42	0	1523.91144	3	508.6419983	-9.121052952	19.14526167
														MLPDKDCR	2	1	1	N-Term(iTRAQ8plex); K5(iTRAQ8plex); C7(Methylthio)	0.901	5.617	2.249	2.76	23	1	1631.84343	2	816.425354	-5.742531426	23.95153167
														MIYASSK	4	5	2	N-Term(iTRAQ8plex); M1(Oxidation); K7(iTRAQ8plex)	-	-	-	3.65	43	0	1423.79551	3	475.2700195	-8.435871728	15.36087
														MIYASSK	1	5	2	N-Term(iTRAQ8plex); K7(iTRAQ8plex)	-	-	-	-	26	0	1407.79914	3	475.2700195	-8.435871728	15.36087
5031653	BCAS2	pre-mRNA-splicing factor SPF27	NP_005863.1	16.00	2	2	6	225	26.1	5.66	1.609	1.062	1.170																
														NDITAWQECVNNSMAQLEHQAVR	4	1	1	N-Term(iTRAQ8plex); C9(Methylthio)	1.524	0.827	1.323	7.08	105	0	3007.41886	3	1003.14447	2.049962052	61.66055667
														TIVQLENEIYQIK	2	1	1	N-Term(iTRAQ8plex); K13(iTRAQ8plex)	1.609	1.365	1.034	4.33	27	0	2199.27939	3	733.7646484	-2.332235512	43.87133333
5031741	DNAJA2	dnaJ homolog subfamily A member 2	NP_005871.1	17.48	4	4	6	412	45.7	6.48	0.965	2.162	1.979																
														QLAPGMVQQMQSVCSDCNGEGEVINEKDR	1	1	1	N-Term(iTRAQ8plex); C14(Methylthio); C17(Methylthio); K27(iTRAQ8plex)	-	-	-	4.11	-	1	3865.8102	4	975.1990967	-2.151602441	32.86973
														QLAPGMVQQMQSVCSDCNGEGEVINEKDR	2	1	1	N-Term(iTRAQ8plex); M6(Oxidation); M10(Oxidation); C14(Methylthio); [illegible]	-	-	-	3.36	22	1	3897.77456	4	975.1990967	-2.151602441	32.86973
														EISFAYEVLSNPEKR	1	1	1	N-Term(iTRAQ8plex); K14(iTRAQ8plex)	1.154	2.222	1.979	2.82	-	1	2390.30875	3	797.4411011	-3.715556555	41.3753
														VSLEDLYNGK	1	1	1	N-Term(iTRAQ8plex); K10(iTRAQ8plex)	0.965	1.594	1.208	-	30	0	1745.97287	3	582.6624756	-9.460509059	45.04457333
														LYDILGVPPGASENELKK	1	1	1	N-Term(iTRAQ8plex); K17(iTRAQ8plex); K18(iTRAQ8plex)	0.957	2.162	1.983	-	27	1	2855.63999	3	952.5515137	-8.615768963	40.24708333
5031749	HMGN2	non-histone chromosomal protein HMG-17	NP_005508.1	20.00	2	2	3	90	9.4	9.99	1.704	0.584	0.606																
														EGNNPAENGDAK	2	1	1	N-Term(iTRAQ8plex); K12(iTRAQ8plex)	1.415	0.66	0.709	4.18	30	0	1823.91959	3	608.6447144	-8.13165431	10.02963333
														EGNNPAENGDAKTDQAQK	1	1	1	N-Term(iTRAQ8plex); K12(iTRAQ8plex); K18(iTRAQ8plex)	2.051	0.517	0.519	2.93	-	1	2799.43924	3	933.8179321	-8.722519462	11.42497
5031755	HNRNPR	heterogeneous nuclear ribonucleoprotein R isoform 2	NP_005817.1	25.91	10	15	97	633	70.9	8.13	1.076	0.786	0.748																
														EFNEEGALSVLQQFK	4	6	1	N-Term(iTRAQ8plex); K15(iTRAQ8plex)	1.131	1.168	0.879	5.67	65	0	2347.2625	2	1174.134888	-5.513882398	42.66710667
														VTEGLVDVILYHQPDDK	6	6	1	N-Term(iTRAQ8plex); K17(iTRAQ8plex)	0.975	0.53	0.682	5.64	42	0	2549.40151	4	638.105835	-2.224575544	48.80243
														VQESTKGPDEAK	1	12	1	N-Term(iTRAQ8plex); K6(iTRAQ8plex); K12(iTRAQ8plex)	0.847	0.611	0.555	5.28	-	1	2201.2582	4	551.0700073	1.870801379	16.394395
														NLATTVTEEILEK	6	12	1	N-Term(iTRAQ8plex); K13(iTRAQ8plex)	1.291	0.817	0.741	4.75	41	0	2069.19797	2	1035.101929	0.739176688	39.07322333
														GYAFITFCGK	7	8	1	N-Term(iTRAQ8plex); C8(Methylthio); K10(iTRAQ8plex)	1.023	0.812	0.729	4.14	53	0	1760.92168	2	880.9644775	-6.095581653	71.45922333
														LKDYAFVHFEDR	5	12	1	N-Term(iTRAQ8plex); K2(iTRAQ8plex)	1.073	0.918	0.818	3.83	27	1	2148.16239	3	716.725647	-3.46511608	33.70593333
														DLYEDELVPLFEK	12	8	1	N-Term(iTRAQ8plex); K13(iTRAQ8plex)	1.314	0.783	0.812	3.75	52	0	2218.20098	2	1109.604126	-4.235850123	39.78618167
														ENILEEFSK	3	12	1	N-Term(iTRAQ8plex); K9(iTRAQ8plex)	0.992	0.843	0.609	3.5	35	0	1716.94919	3	572.987915	-7.955641831	59.01226
														SFSEFGK	5	12	1	N-Term(iTRAQ8plex); K7(iTRAQ8plex)	1.061	0.639	0.628	3.16	30	0	1409.79265	2	705.3999634	2.942870934	24.487865

														LMMDPLSGQNR	1	8	1	N-Term(iTRAQ8plex)	1.063	0.99	0.914	-	23	0	1565.80364	2	783.4054565	-2.908943342	33.62657333
														TGYTLDVTTGQR	11	18	2	N-Term(iTRAQ8plex)	-	-	-	5.07	51	0	1615.86875	3	539.2944336	5.833537696	25.62625833
														GFCFLEYEDHK	30	23	2	N-Term(iTRAQ8plex); C3(Methylthio); K11(iTRAQ8plex)	-	-	-	4.94	46	0	2041.99399	3	681.3337402	-5.159116385	40.79938167
														HIGVCISVANNR	2	11	2	N-Term(iTRAQ8plex); C5(Methylthio)	-	-	-	3.46	76	0	1632.85489	3	544.9564819	-4.109213408	22.9882
														AGPIWDLR	2	19	2	N-Term(iTRAQ8plex)	-	-	-	3.28	29	0	1231.71318	2	616.3556519	-4.92343041	25.28796333
														SAFLCGVMK	2	13	2	N-Term(iTRAQ8plex); C5(Methylthio); K9(iTRAQ8plex)	-	-	-	2.88	29	0	1609.85954	2	805.4334106	-8.03028499	65.452195
														VLFVR	2	23	2	N-Term(iTRAQ8plex)	-	-	-	-	26	0	937.61131	2	469.3092957	-2.4920896	19.740415
5031815	KARS	lysine--tRNA ligase isoform 2	NP_005539.1	12.73	6	6	22	597	68.0	6.35	1.017	1.846	1.930															
														VTYHPDGPEGQAYDVDFTPPFR	6	3	1	N-Term(iTRAQ8plex)	1.066	1.445	1.405	6.58	65	0	2812.34745	3	938.1206665	-1.648103006	33.63926833
														FHVDISLTDFIQK	3	2	1	N-Term(iTRAQ8plex); K13(iTRAQ8plex)	0.814	1.805	1.928	5.6	46	0	2171.22507	3	724.413208	-3.238045957	48.851825
														EICNAYTELNDPMR	4	3	1	N-Term(iTRAQ8plex); C3(Methylthio)	0.957	1.652	1.562	5.24	63	0	2018.94304	2	1009.975159	7.067293393	39.94457167
														ILDDICVAK	2	3	1	N-Term(iTRAQ8plex); C6(Methylthio); K9(iTRAQ8plex)	1.253	2.734	2.462	4.04	49	0	1643.92302	2	822.4651489	-5.517939904	70.16959167
														YLDLILNDFVR	2	3	1	N-Term(iTRAQ8plex)	0.97	2.313	2.335	2.89	41	0	1684.95745	2	842.9823608	-0.086791695	60.70125
														LIFYDLR	5	2	1	N-Term(iTRAQ8plex)	1.379	2.861	2.559	2.77	27	0	1243.73015	2	622.3687134	-4.077381405	41.39747667
5031839	KRT6A	keratin, type II cytoskeletal 6C	NP_005545.1	59.04	4	38	872	564	60.0	8.00	0.212	0.141	0.136															
														QCANLQAAIADAEQR	26	2	1	N-Term(iTRAQ8plex); C2(Methylthio)	0.425	0.235	0.198	6.74	86	0	1951.95976	2	976.4849243	-0.317776168	40.39710167
														SGFSSVSVSR	22	1	1	N-Term(iTRAQ8plex)	0.17	0.104	0.13	4.72	53	0	1316.72368	2	658.8654785	9.477395627	22.64378
														GMQDLVEDFK	19	1	1	N-Term(iTRAQ8plex); K10(iTRAQ8plex)	0.172	0.085	0.103	3.89	71	0	1789.95659	2	895.4819336	-2.737905783	62.80277667
														KQCANLQAAIADAEQR	4	2	1	N-Term(iTRAQ8plex); K1(iTRAQ8plex); C3(Methylthio)	0.293	0.332	0.28	3.86	54	1	2384.26554	3	795.4266968	0.845032569	33.75028167
														LALDVEIATYR	117	10	7	N-Term(iTRAQ8plex)	-	-	-	4.83	67	0	1567.90409	3	523.3062134	2.771589637	39.12087
														LALDVEIATYR	8	10	7	N-Term(iTRAQ8plex); Y10(iTRAQ8plex)	-	-	-	-	60	0	1872.106	3	523.3062134	2.771589637	39.12087
														TAAENEFVTLK	10	4	3	N-Term(iTRAQ8plex); K11(iTRAQ8plex)	-	-	-	4.72	51	0	1831.03826	2	916.0227661	-2.1385099	38.95932833
														TAAENEFVTLKK	2	4	3	N-Term(iTRAQ8plex); K11(iTRAQ8plex); K12(iTRAQ8plex)	-	-	-	4.09	34	1	2263.34414	4	566.5914917	0.722654964	26.116015
														FLEQQNK	13	31	8	N-Term(iTRAQ8plex); K7(iTRAQ8plex)	-	-	-	-	33	0	1514.87885	3	505.631134	0.090799082	15.97476833
														GELAIK	4	2	6	N-Term(iTRAQ8plex); K6(iTRAQ8plex)	-	-	-	-	28	0	1238.78995	3	413.6025085	0.089671776	17.83946167
														EYQELMNVK	14	5	3	N-Term(iTRAQ8plex); K9(iTRAQ8plex)	-	-	-	4.08	56	0	1761.96379	2	881.4855347	-1.565630391	28.72846833
														FASFIDKVR	16	27	8	N-Term(iTRAQ8plex); K7(iTRAQ8plex)	-	-	-	3.33	31	1	1691.01076	4	423.5080261	0.114317571	39.05999667
														FASFIDK	57	28	8	N-Term(iTRAQ8plex); K7(iTRAQ8plex)	-	-	-	3.23	35	0	1435.83818	2	718.4240723	0.227470368	33.40336167
														NKYEDEINK	1	9	5	N-Term(iTRAQ8plex); K2(iTRAQ8plex); K9(iTRAQ8plex)	-	-	-	-	27	1	2065.16616	4	517.0469971	-1.50249351	14.99920833
														QNLEPLFEQYINNLR	50	4	3	N-Term(iTRAQ8plex)	-	-	-	6.54	76	0	2195.17461	2	1098.090942	-0.753085595	58.40959667
														WTLLQEQGTK	50	4	3	N-Term(iTRAQ8plex); K10(iTRAQ8plex)	-	-	-	4.48	49	0	1812.04447	2	906.5276489	0.242781404	32.60840167
														QLDSIVGER	26	3	3	N-Term(iTRAQ8plex)	-	-	-	3.72	66	0	1320.74237	3	440.9189758	-0.103774792	25.30154833
														QNLEPLFEQYINNLRR	5	4	3	N-Term(iTRAQ8plex)	-	-	-	3.15	27	1	2351.28554	4	588.5768433	3.471235937	52.94203
														NLDLDSIIAEVK	64	8	5	N-Term(iTRAQ8plex); K12(iTRAQ8plex)	-	-	-	5.73	89	0	1938.13909	2	969.5731812	1.173488892	50.31108
														KLLEGEECR	13	13	6	N-Term(iTRAQ8plex); K1(iTRAQ8plex); C8(Methylthio)	-	-	-	2.79	31	1	1730.93911	3	577.6514282	0.43855829	29.74912
														LLEGEECR	16	16	6	N-Term(iTRAQ8plex); C7(Methylthio)	-	-	-	2.75	36	0	1298.63898	3	433.551178	0.260569375	30.10390833
														AEAESWYQTK	19	4	3	N-Term(iTRAQ8plex); K10(iTRAQ8plex)	-	-	-	4.56	66	0	1820.96013	2	910.9831543	-2.67404492	21.62443167
														YEELQVTAGR	43	3	3	N-Term(iTRAQ8plex)	-	-	-	4.27	46	0	1469.78899	2	735.4003296	2.178033814	27.29477167
														YEELQVTAGR	3	3	3	N-Term(iTRAQ8plex); Y1(iTRAQ8plex)	-	-	-	-	32	0	1773.99126	2	735.4003296	2.178033814	27.29477167
														GEMALK	1	4	3	N-Term(iTRAQ8plex); M3(Oxidation); K6(iTRAQ8plex)	-	-	-	-	30	0	1272.74594	3	424.920166	1.365941791	12.0309
														ALYDAELSQMQTHISDTSVVLSMDNNR	37	3	2	N-Term(iTRAQ8plex)	-	-	-	8.02	71	0	3342.63169	3	1114.88208	2.042009116	47.06352
														GSGGLGGACGGAGFGSR	22	3	2	N-Term(iTRAQ8plex); C9(Methylthio)	-	-	-	7.23	107	0	1717.81766	2	859.4060059	-0.301082678	27.15323167
														AIGGGLSSVGGGSSTIK	29	2	2	N-Term(iTRAQ8plex); K17(iTRAQ8plex)	-	-	-	7.14	100	0	2056.18364	2	1028.595459	-1.099099908	27.14610667
														ADTLTDEINFLR	22	3	2	N-Term(iTRAQ8plex)	-	-	-	6.31	70	0	1711.91397	3	571.3095703	-1.579292097	42.44845667
														ISIGGGSCAISGGYGSR	40	3	2	N-Term(iTRAQ8plex); C8(Methylthio)	-	-	-	5.5	76	0	1891.93132	2	946.4692993	0.26412841	34.20610167
														ALYDAELSQMQTHISDTSVVLSMDNNR	15	3	2	N-Term(iTRAQ8plex); M23(Oxidation)	-	-	-	5.22	44	0	3358.62156	3	1114.88208	2.042009116	47.06352
														SLYGLGGSK	26	3	2	N-Term(iTRAQ8plex); K9(iTRAQ8plex)	-	-	-	4.12	48	0	1489.88294	3	497.2991638	-0.351098001	22.94869333
														NKLEGLEDALQK	4	3	2	N-Term(iTRAQ8plex); K2(iTRAQ8plex); K12(iTRAQ8plex)	-	-	-	4.07	33	1	2270.34477	2	1135.676025	-1.562378891	31.82945
														DVDAAYMNKVELQAK	1	9	2	N-Term(iTRAQ8plex); M7(Oxidation); K9(iTRAQ8plex); K15(iTRAQ8plex)	-	-	-	4.03	-	1	2623.44716	4	656.6172485	-2.177302998	24.033175
														LLKEYQELMNVK	1	3	2	N-Term(iTRAQ8plex); K3(iTRAQ8plex); M9(Oxidation); K12(iTRAQ8plex)	-	-	-	3.34	-	1	2436.42789	3	807.4837036	0.634716014	54.63243167
														QEIAEINR	9	3	2	N-Term(iTRAQ8plex)	-	-	-	3.24	37	0	1276.72034	3	426.2434082	-0.48557454	23.91620167
														LLKEYQELMNVK	6	3	2	N-Term(iTRAQ8plex); K3(iTRAQ8plex); K12(iTRAQ8plex)	-	-	-	3.22	29	1	2420.44155	3	807.4837036	0.634716014	54.63243167
														LPGVSR	2	3	2	N-Term(iTRAQ8plex)	-	-	-	-	16	0	932.58293	2	466.795105	-0.158655126	16.53337333
														VELQAK	1	16	4	N-Term(iTRAQ8plex); K6(iTRAQ8plex)	-	-	-	2.75	-	0	1295.81623	3	432.61026	1.470485751	22.94674667
														LDSELR	1	16	3	N-Term(iTRAQ8plex)	-	-	-	-	26	0	1036.59331	2	518.800293	-0.706142412	18.550555
														DVDAAYMNK	25	11	4	N-Term(iTRAQ8plex); K9(iTRAQ8plex)	-	-	-	4.2	50	0	1634.86492	2	817.9366455	-0.50579336	22.28227833
														DVDAAYMNK	13	11	4	N-Term(iTRAQ8plex); M7(Oxidation); K9(iTRAQ8plex)	-	-	-	3.52	47	0	1650.86186	2	817.9366455	-0.50579336	22.28227833
														KDVDAAYMNK	3	11	4	N-Term(iTRAQ8plex); K1(iTRAQ8plex); K10(iTRAQ8plex)	-	-	-	3	31	1	2067.16056	3	689.7250366	-3.199890157	21.25677833
														AQYEEIAQR	16	7	4	N-Term(iTRAQ8plex)	-	-	-	3.65	42	0	1411.75541	3	471.2566528	5.024911587	22.42886333
5031857	LDHA	L-lactate dehydrogenase A chain isoform 1	NP_005557.1	54.52	15	17	205	332	36.7	8.27	1.090	2.047	5.069															
														LIIVSNPVDILTYVAWK	25	1	1	N-Term(iTRAQ8plex); K17(iTRAQ8plex)	1.484	4.911	22.888	6.49	73	0	2552.5261	2	1276.772949	2.883606711	63.60595
														GLYGIKDDVFLSVPCILGQNGISDLVK	23	3	1	N-Term(iTRAQ8plex); K6(iTRAQ8plex); C15(Methylthio); K27(iTRAQ8plex)	-	-	-	5.39	48	1	3822.15085	3	1274.721802	5.106999748	66.44452833
														QVVESAYEVIK	35	3	1	N-Term(iTRAQ8plex); K11(iTRAQ8plex)	1.083	1.689	4.179	5.24	83	0	1873.07073	2	937.0400391	-8.707507251	27.26170167
														DLADELALVDVIEDK	16	5	1	N-Term(iTRAQ8plex); K15(iTRAQ8plex)	0.8	1.922	5.881	5.14	83	0	2266.26152	2	1133.634399	-1.045374442	58.13165833
														GYTSWAIGLSVADLAESIMK	13	3	1	N-Term(iTRAQ8plex); K20(iTRAQ8plex)	-	3.549	12.836	4.73	68	0	2720.48801	3	907.5008545	3.315187028	65.21080833
														VTLTSEEEAR	23	3	1	N-Term(iTRAQ8plex)	1.252	2.911	7.584	4.56	73	0	1438.76722	3	480.2605896	-1.324572281	22.82921333
														GEMMDLQHGSLFLR	8	5	1	N-Term(iTRAQ8plex)	1.312	1.662	5.684	4.34	50	0	1937.98442	2	969.4958496	-1.825969914	38.06756333
														SADTLWGIQK	9	3	1	N-Term(iTRAQ8plex); K10(iTRAQ8plex)	0.581	0.999	1.859	4.26	54	0	1726.98357	2	863.9954224	-6.516097573	26.09129
														DQLIYNLLK	6	5	1	N-Term(iTRAQ8plex); K9(iTRAQ8plex)	1.074	1.222	5.069	4.16	56	0	1728.03447	2	864.520874	-9.907136349	70.40733667
														LVIITAGAR	10	4	1	N-Term(iTRAQ8plex)	1.419	3.764	12.504	4.05	40	0	1217.78089	3	406.6019287	2.374448702	31.47006667
														LKGEMMDLQHGSLFLR	2	5	1	N-Term(iTRAQ8plex); K2(iTRAQ8plex)	-	-	-	4.01	-	1	2483.38255	5	497.4823303	4.100778331	44.06111
														DYNVTANSK	6	4	1	N-Term(iTRAQ8plex); K9(iTRAQ8plex)	1.053	2.257	6.499	3.98	39	0	1619.87578	2	810.4415283	-5.637024448	15.32675333
														VHPVSTMIK	9	3	1	N-Term(iTRAQ8plex); K9(iTRAQ8plex)	0.791	2.78	5.139	3.63	57	0	1619.96244	3	540.6585693	-9.36846056	16.48706
														FIIPNVVK	7	4	1	N-Term(iTRAQ8plex); K8(iTRAQ8plex)	1.528	1.614	3.944	3.21	35	0	1537.9777	3	513.3307495	-9.711463885	53.93235333
														GEMMDLQHGSLFLR	2	5	1	N-Term(iTRAQ8plex); M4(Oxidation)	0.888	1.41	2.396	3.04	24	0	1953.97373	2	969.4958496	-1.825969914	38.06756333
														LKGYTSWAIGLSVADLAESIMK	1	3	1	N-Term(iTRAQ8plex); K2(iTRAQ8plex); K22(iTRAQ8plex)	-	-	-	2.85	-	1	3265.86904	4	817.2227173	1.729389806	68.75882
														DQLIYNLLKEEQTPQNK	1	5	1	N-Term(iTRAQ8plex); K9(iTRAQ8plex); K17(iTRAQ8plex)	-	3.188	17.412	2.76	-	1	2986.67942	3	996.2313232	-6.10873177	43.21719167
														LIIVSNPVDILTYVAWK	2	1	1	N-Term(iTRAQ8plex); Y13(iTRAQ8plex); K17(iTRAQ8plex)	-	-	-	-	34	0	2856.7145	2	1276.772949	2.883606711	63.60595
														VIGSGCNLDSAR	32	14	2	N-Term(iTRAQ8plex); C6(Methylthio)	-	-	-	5.17	104	0	1541.77402	3	514.5961914	1.445522314	30.11560667
														LNLVQR	2	8	2	N-Term(iTRAQ8plex)	-	-	-	-	18	0	1046.65569	2	523.8314819	-6.405213189	23.27319
5031863	LGALS3BP	galectin-3-binding protein precursor	NP_005558.1	12.31	5	5	26	585	65.3	5.27	0.881	1.044	0.319															
														TLQALEFHTVPFQLLAR	17	1	1	N-Term(iTRAQ8plex)	0.88	1.018	0.305	5.56	63	0	2288.3123	2	1144.65979	2.360760862	53.185985
														ELSEALGQIFDSQR	2	1	1	N-Term(iTRAQ8plex)	0.923	0.913	0.204	5.19	53	0	1897.01396	2	949.0106201	8.989029444	50.60697
														ASHEEVEGLVEK	4	1	1	N-Term(iTRAQ8plex); K12(iTRAQ8plex)	0.684	1.044	0.242	4.69	69	0	1935.05254	2	968.0299072	-6.115320718	16.36156667
														STSSFPCPAGHFNGFR	2	1	1	N-Term(iTRAQ8plex); C7(Methylthio)	0.882	1.136	0.653	4.66	38	0	2061.9523	3	687.9889526	-2.617732637	35.98430667
														SDLAVPSELALLK	1	1	1	N-Term(iTRAQ8plex); K13(iTRAQ8plex)	0.971	1.07	0.657	3.63	-	0	1964.18277	3	655.3991089	-3.09436063	35.18106167
5031873	LMAN1	protein ERGIC-53 precursor	NP_005561.1	9.41	2	2	7	510	57.5	6.77	1.436	0.877	0.869															
														GHPDLQGQPAEEIFESVGDR	2	1	1	N-Term(iTRAQ8plex)	0.759	0.877	0.869	6.24	55	0	2485.23081	3	829.0817871	1.8657334	35.79962
														GPHLVQSDGTVPFWAHAGNAIPSSDQIR	5	1	1	N-Term(iTRAQ8plex)	2.717	-	-	5.92	34	0	3261.66323	4	816.1712646	-2.312496944	34.670545
5031875	LMNA	lamin isoform C	NP_005563.1	64.51	1	34	421	572	65.1	6.84	0.745	-	0.313															
														SVTVVEDDEDEDGDDLLHHHHVSGSR	4	2	1	N-Term(iTRAQ8plex)	0.745	-	0.313	6.18	41	0	3203.47409	3	1068.496216	-1.216519612	24.77108667
														AGQVVTIWAAGAGATHSPPTDLVWK	31	8	2	N-Term(iTRAQ8plex); K25(iTRAQ8plex)	-	-	-	6.34	57	0	3141.72915	4	786.1877441	-0.472547896	43.57742333
														IDSLSAQLSQLQK	6	7	2	N-Term(iTRAQ8plex); K13(iTRAQ8plex)	-	-	-	6.17	59	0	2039.19097	2	1020.099121	-2.327262419	33.64147833
														NSNLVGAAHEELQQSR	27	7	2	N-Term(iTRAQ8plex)	-	-	-	5.79	85	0	2057.06499	2	1029.036133	-1.357417006	22.37199667
														MQQQLDEYQELLDIK	16	8	2	N-Term(iTRAQ8plex); K15(iTRAQ8plex)	-	-	-	5.67	86	0	2502.32378	2	1251.665527	-5.304362535	73.416185
														ITESEEVVSR	13	4	2	N-Term(iTRAQ8plex)	-	-	-	5.34	67	0	1452.78806	3	484.9342041	2.263065605	24.38489333
														TLEGELHDLR	49	7	2	N-Term(iTRAQ8plex)	-	-	-	5.33	72	0	1486.81171	3	496.2755127	-3.199102584	23.654935
														LALDMEIHAYR	30	8	2	N-Term(iTRAQ8plex)	-	-	-	5.13	76	0	1635.87687	3	545.9638672	-3.669583546	32.87996833
														TALINSTGEEVAMR	10	7	2	N-Term(iTRAQ8plex)	-	-	-	5.03	62	0	1795.95865	3	604.651123	-4.807231513	29.81118
														MQQQLDEYQELLDIK	15	8	2	N-Term(iTRAQ8plex); M1(Oxidation); K15(iTRAQ8plex)	-	-	-	4.91	50	0	2518.33315	2	1251.665527	-5.304362535	73.416185
														VAVEEVDEEGK	6	8	2	N-Term(iTRAQ8plex); K11(iTRAQ8plex)	-	-	-	4.88	59	0	1811.98064	2	906.4939575	-2.25878845	29.31310667
														LQTMKEELDFQK	3	7	2	N-Term(iTRAQ8plex); K5(iTRAQ8plex); K12(iTRAQ8plex)	-	-	-	4.8	44	1	2422.37758	2	1211.690552	-1.686116457	36.74179333
														EAALSTALSEK	11	7	2	N-Term(iTRAQ8plex); K11(iTRAQ8plex)	-	-	-	4.77	51	0	1727.99419	2	864.5007324	-3.346698901	42.82938833
														LKDLEALLNSK	14	7	2	N-Term(iTRAQ8plex); K2(iTRAQ8plex); K11(iTRAQ8plex)	-	-	-	4.73	37	1	2156.34316	2	1078.674072	-0.418945243	50.611525
														LEAALGEAK	14	7	2	N-Term(iTRAQ8plex); K9(iTRAQ8plex)	-	-	-	4.38	51	0	1509.91047	3	503.9750061	0.50583168	38.65092667
														TALINSTGEEVAMR	5	7	2	N-Term(iTRAQ8plex); M13(Oxidation)	-	-	-	4.37	64	0	1811.93882	3	604.651123	-4.807231513	29.81118
														AAYEAELGDAR	16	4	2	N-Term(iTRAQ8plex)	-	-	-	4.34	58	0	1469.76225	2	735.3789673	-2.145499762	22.40932833
														SGAQASSTPLSPTR	6	4	2	N-Term(iTRAQ8plex)	-	-	-	4.33	54	0	1663.89067	2	832.4489746	-0.618250506	16.90638167
														LALDMEIHAYR	10	8	2	N-Term(iTRAQ8plex); M5(Oxidation)	-	-	-	4.31	55	0	1651.87461	3	545.9638672	-3.669583546	32.87996833
														QNGDDPLLTYR	2	8	2	N-Term(iTRAQ8plex)	-	-	-	3.7	42	0	1595.82915	2	798.4182129	-2.48280886	19.22656833
														LRDLEDSLAR	42	8	2	N-Term(iTRAQ8plex)	-	-	-	3.62	64	1	1491.84006	3	497.9511414	-2.969809824	38.74856833
														LQEKEDLQELNDR	4	4	2	N-Term(iTRAQ8plex); K4(iTRAQ8plex)	-	-	-	3.59	29	1	2238.20717	4	560.3076782	-4.364333217	22.5758
														LQLELSK	13	4	2	N-Term(iTRAQ8plex); K7(iTRAQ8plex)	-	-	-	3.55	35	0	1438.90959	2	719.9574585	-0.911057649	35.984405
														EGDLIAAQAR	8	7	2	N-Term(iTRAQ8plex)	-	-	-	3.54	49	0	1347.74952	3	449.9213562	-2.898124941	15.81620333
														SLETENAGLR	5	4	2	N-Term(iTRAQ8plex)	-	-	-	3.46	43	0	1393.75559	3	465.2567139	-2.369818973	17.995785

														LADALQELR	17	7	2	N-Term(iTRAQ8plex)	-	-	-	3.25	40	0	1332.77532	2	666.8912964	-2.689985716	44.88191167
														SNEDQSMGNWQIK	2	8	2	N-Term(iTRAQ8plex); K13(iTRAQ8plex)	-	-	-	3.19	26	0	2145.08622	3	715.7002563	0.323759933	21.69900833
														AQNTWGCGNSLR	3	8	2	N-Term(iTRAQ8plex); C7(Methylthio)	-	-	-	3.14	43	0	1656.78179	2	828.8945313	-4.253076694	20.70826167
														QLQDEMLR	1	7	2	N-Term(iTRAQ8plex)	-	-	-	2.96	-	0	1336.71367	2	668.8604736	-4.489109753	22.99892167
														LAVYIDR	10	4	2	N-Term(iTRAQ8plex)	-	-	-	2.89	62	0	1153.68875	3	385.234436	0.419138582	32.518365
														NIYSEELR	8	7	2	N-Term(iTRAQ8plex)	-	-	-	2.86	32	0	1327.70964	2	664.3584595	-4.744241125	16.52615333
														DLEDSLAR	3	8	2	N-Term(iTRAQ8plex)	-	-	-	2.8	31	0	1222.65495	2	611.8303833	-3.779745018	16.11086167
														VAVEEVDEEGKFVR	2	8	2	N-Term(iTRAQ8plex); K11(iTRAQ8plex)	-	-	-	2.71	35	1	2214.22353	4	554.3113403	0.379432389	26.61114167
														LALDMEIHAYR	5	8	2	N-Term(iTRAQ8plex); Y10(iTRAQ8plex)	-	-	-	-	51	0	1940.07561	3	545.9638672	-3.669583546	32.87996833
														AAYEAELGDAR	1	4	2	N-Term(iTRAQ8plex); Y3(iTRAQ8plex)	-	-	-	-	39	0	1773.95232	2	735.3789673	-2.145499762	22.40932833
														RVDAENR	1	7	2	N-Term(iTRAQ8plex)	-	-	-	-	38	1	1163.63999	3	388.5515137	-2.985059307	7.35876
														LVEIDNGK	1	7	2	N-Term(iTRAQ8plex); K8(iTRAQ8plex)	-	-	-	-	28	0	1495.89751	2	748.4523926	2.317830086	18.8729
														LALDMEIHAYR	1	8	2	N-Term(iTRAQ8plex); M5(Oxidation); Y10(iTRAQ8plex)	-	-	-	-	24	0	1956.07424	3	545.9638672	-3.669583546	32.87996833
														EMAEMR	1	8	2	N-Term(iTRAQ8plex)	-	-	-	-	17	0	1070.52471	2	535.7659912	-2.742111517	7.418713333
														LLEGEEER	5	11	4	N-Term(iTRAQ8plex)	-	-	-	2.96	37	0	1278.68534	3	426.8999634	0.786422343	21.17209167
5031877	LMNB1	lamin-B1 isoform 1	NP_005564.1	40.27	19	20	78	586	66.4	5.16	1.125	0.684	0.659																
														SLETENSALQLQVTER	2	1	1	N-Term(iTRAQ8plex)	1.095	0.649	0.726	6.42	90	0	2122.13628	2	1061.571777	3.249917346	30.18935
														IESLSSQLSNLQK	5	2	1	N-Term(iTRAQ8plex); K13(iTRAQ8plex)	1.281	0.804	0.789	5.94	71	0	2055.17923	2	1028.093994	-4.821784622	33.19262167
														TTIPEEEEEEEEAAGVVVEEELFHQQGTPR	2	2	1	N-Term(iTRAQ8plex)	-	-	-	5.69	28	0	3715.74892	4	929.692688	-7.441091272	73.86604667
														DAALATALGDKK	2	1	1	N-Term(iTRAQ8plex); K11(iTRAQ8plex); K12(iTRAQ8plex)	1.711	0.95	0.816	4.93	27	1	2086.2643	4	522.3215332	0.369863252	23.43632
														EMAEIRDQMQQQLNDYEQLLDVK	2	2	1	N-Term(iTRAQ8plex); K23(iTRAQ8plex)	-	-	-	4.84	27	1	3445.76259	4	862.196106	2.125116843	62.54652667
														NQNSWGTGEDVK	2	2	1	N-Term(iTRAQ8plex); K12(iTRAQ8plex)	1.262	0.737	0.651	4.73	57	0	1943.00359	2	972.0054321	-2.225246631	13.86932333
														LAQALHEMR	12	2	1	N-Term(iTRAQ8plex)	1.303	0.869	0.682	4.7	64	0	1372.76053	3	458.2594299	-2.589487895	26.50404
														CQSLTEDLEFR	6	1	1	N-Term(iTRAQ8plex); C1(Methylthio)	1.068	0.672	0.561	4.32	53	0	1690.80339	2	845.9053345	-2.862529558	36.76732
														LSSEMNTSTVNSAR	2	2	1	N-Term(iTRAQ8plex)	1.237	0.682	0.568	4.3	64	0	1800.90459	2	900.9559326	-0.989883438	21.16491667
														IQELEDLLAK	9	2	1	N-Term(iTRAQ8plex); K10(iTRAQ8plex)	0.898	0.58	0.502	4.28	59	0	1780.05998	2	890.5336304	-4.312875904	78.136575
														CKAEHDQLLLNYAK	2	1	1	N-Term(iTRAQ8plex); C1(Methylthio); K2(iTRAQ8plex); K14(iTRAQ8plex)	1.871	0.937	0.938	4	29	1	2604.43741	3	868.8173218	-1.190359943	26.92446167
														ALYETELADAR	6	1	1	N-Term(iTRAQ8plex)	1.106	0.684	0.835	3.86	49	0	1555.8239	2	778.4155884	-1.973978024	29.36049
														LAQALHEMR	2	2	1	N-Term(iTRAQ8plex); M8(Oxidation)	1.366	0.319	0.488	3.77	34	0	1388.75754	3	458.2594299	-2.589487895	26.50404
														SMYEEEINETR	2	1	1	N-Term(iTRAQ8plex)	1.156	0.603	0.993	3.74	33	0	1704.79863	2	852.9029541	-3.882990491	17.72981833
														LALDMEISAYR	6	2	1	N-Term(iTRAQ8plex)	1.025	0.711	0.675	3.33	39	0	1585.85356	2	793.4304199	-1.644588322	42.66062
														LAVYIDK	5	1	1	N-Term(iTRAQ8plex); K7(iTRAQ8plex)	1.073	0.65	0.551	3.31	30	0	1429.88848	2	715.447876	0.695182276	41.77502333
														SLETENSALQLQVTEREEVR	1	1	1	N-Term(iTRAQ8plex)	1.555	0.952	0.702	3.2	-	1	2635.37528	3	879.1299438	-3.355502005	30.75250333
														DQMQQQLNDYEQLLDVK	1	2	1	N-Term(iTRAQ8plex); M3(Oxidation); K17(iTRAQ8plex)	-	-	-	3.16	-	0	2732.39297	4	683.8536987	-3.371132279	38.45599333
														AGGPTTPLSPTR	2	1	1	N-Term(iTRAQ8plex)	1.112	0.665	0.617	2.72	27	0	1458.81816	2	729.9127197	-2.514684125	16.87899333
														LVEVDSGR	1	2	1	N-Term(iTRAQ8plex)	1.092	0.616	0.566	-	25	0	1178.6624	2	589.8348389	-4.988863325	19.325185
														LSSEMNTSTVNSAR	1	2	1	N-Term(iTRAQ8plex); M5(Oxidation)	0.735	1.221	1.876	-	24	0	1816.89878	2	900.9559326	-0.989883438	21.16491667
														LQIEIGK	8	1	2	N-Term(iTRAQ8plex); K7(iTRAQ8plex)	-	-	-	3.45	35	0	1408.90134	3	470.3052979	2.09312363	28.58012833
														LLEGEEER	5	11	4	N-Term(iTRAQ8plex)	-	-	-	2.96	37	0	1278.68534	3	426.8999634	0.786422343	21.17209167
5031977	NAMPT	nicotinamide phosphoribosyltransferase precursor	NP_005737.1	9.78	3	3	8	491	55.5	7.15	1.257	3.550	2.552																
														VIQGDGVDINTLQEIVEGMK	4	1	1	N-Term(iTRAQ8plex); K20(iTRAQ8plex)	1.34	8.022	5.248	6.66	76	0	2766.5308	3	922.8484497	5.040453956	57.08231333
														NAQLNIELEAAHH	2	1	1	N-Term(iTRAQ8plex)	0.355	0.922	0.797	4.9	66	0	1763.93021	3	588.6482544	-2.279776367	22.43616167
														YLLETSGNLDGLEYK	2	1	1	N-Term(iTRAQ8plex); K15(iTRAQ8plex)	1.843	2.637	1.994	4.58	58	0	2323.24381	3	775.0861206	-8.769089804	33.061185
5031981	PSMD14	26S proteasome non-ATPase regulatory subunit 14	NP_005796.1	4.19	1	1	4	310	34.6	6.52	0.933	1.653	1.507																
														AVAVVVDPIQSVK	4	1	1	N-Term(iTRAQ8plex); K13(iTRAQ8plex)	0.933	1.653	1.507	5.93	53	0	1933.18987	2	967.0985718	-2.278289054	34.14602333
5031987	PPIF	peptidyl-prolyl cis-trans isomerase F, mitochondrial precursor	NP_005720.1	43.48	6	6	15	207	22.0	9.38	2.181	0.978	0.831																
														HVGPGVLSMANAGPNTNGSQFFICTIK	2	2	1	N-Term(iTRAQ8plex); C24(Methylthio); K27(iTRAQ8plex)	-	-	-	5.1	54	0	3414.74404	4	854.4414673	-4.144685286	50.23434167
														GSGDPSSSSSSGNPLVYLDVDANGKPLGR	4	2	1	N-Term(iTRAQ8plex); K25(iTRAQ8plex)	3.022	0.884	0.989	4.65	-	0	3441.75144	3	1147.921997	-5.602769232	37.04044667
														HVVFGHVK	2	1	1	N-Term(iTRAQ8plex); K8(iTRAQ8plex)	2.223	1.051	0.854	3.49	42	0	1530.93474	2	765.9710083	-1.150485642	13.90467333
														VVLELK	2	3	1	N-Term(iTRAQ8plex); K6(iTRAQ8plex)	2.734	0.92	0.75	2.79	26	0	1308.87082	3	436.961792	-0.220475109	36.73129
														KIVITDCGQLS	4	1	1	N-Term(iTRAQ8plex); K1(iTRAQ8plex); C7(Methylthio)	1.812	0.963	0.795	-	37	1	1831.02445	3	611.0130005	-1.820885111	55.98094667
														FPDENFTLK	1	2	1	N-Term(iTRAQ8plex); K9(iTRAQ8plex)	3.417	1.605	1.183	-	29	0	1718.95932	3	573.657959	1.135805309	47.02204167
5032051	RPS14	40S ribosomal protein S14	NP_005608.1	39.74	7	7	36	151	16.3	10.05	1.279	1.777	1.847																
														ADRDESSPYAAMLAAQDVAQR	6	2	1	N-Term(iTRAQ8plex); M12(Oxidation)	1.021	0.292	0.727	6.33	89	1	2585.25034	3	862.4216309	-2.509223875	23.06576167
														ADRDESSPYAAMLAAQDVAQR	4	2	1	N-Term(iTRAQ8plex)	1.076	1.231	1.426	6.31	76	1	2569.25772	3	862.4216309	-2.509223875	23.06576167
														ELGITALHIK	11	2	1	N-Term(iTRAQ8plex); K10(iTRAQ8plex)	1.248	1.902	1.866	4.36	41	0	1703.05535	2	852.031311	-7.190517748	28.78106167
														CKELGITALHIK	2	2	1	N-Term(iTRAQ8plex); C1(Methylthio); K2(iTRAQ8plex); K12(iTRAQ8plex)	1.565	3.184	3.063	4.11	30	1	2284.34214	3	762.1188965	-9.935506821	34.90617333
														TKTPGPGAQSALR	3	2	1	N-Term(iTRAQ8plex); K2(iTRAQ8plex)	1.261	1.38	0.876	3.79	25	1	1892.10769	4	473.7816162	-6.752446991	12.83867333
														IEDVTPIPSDSTR	4	1	1	N-Term(iTRAQ8plex)	1.294	1.637	1.587	3.74	51	0	1733.91692	2	867.4620972	-3.127937681	28.00025167
														IEDVTPIPSDSTRR	2	1	1	N-Term(iTRAQ8plex)	0.832	0.88	1.276	3.54	32	1	1890.01645	3	630.677002	-3.708269637	23.06916833
														TPGPGAQSALR	4	2	1	N-Term(iTRAQ8plex)	1.519	2.108	2.12	3.21	43	0	1358.76596	3	453.5935059	-2.5294804	15.46060333
5032057	S100A11	protein S100-A11	NP_005611.1	53.33	6	6	99	105	11.7	7.12	0.971	1.762	2.388																
														TEFLSFMNTELAAFTK	54	1	1	N-Term(iTRAQ8plex); K16(iTRAQ8plex)	1.101	1.817	2.258	6.38	65	0	2458.32475	2	1229.666016	4.020351657	61.52979167
														CIESLIAVFQK	36	1	1	N-Term(iTRAQ8plex); C1(Methylthio); K11(iTRAQ8plex)	0.877	1.626	2.712	4.7	69	0	1905.07939	2	953.0404053	-3.300855853	59.1824
														CIESLIAVFQKYAGK	1	1	1	N-Term(iTRAQ8plex); C1(Methylthio); K11(iTRAQ8plex); K15(iTRAQ8plex)	-	-	-	4.09	-	1	2628.51162	4	657.8833618	3.635169638	69.66159333
														DGYNYTLSK	4	1	1	N-Term(iTRAQ8plex); K9(iTRAQ8plex)	0.799	1.239	1.758	3.97	34	0	1668.89336	2	834.9486694	-9.131250259	18.074965
														ISSPTETER	3	1	1	N-Term(iTRAQ8plex)	0.967	1.634	1.952	2.77	34	0	1323.69878	2	662.3555908	-1.41744643	12.98358
														DPGVLDR	1	1	1	N-Term(iTRAQ8plex)	1.003	1.832	2.096	-	23	0	1075.59917	2	538.3032227	-5.37617327	15.99083167
5032069	SF3B4	splicing factor 3B subunit 4	NP_005841.1	8.73	2	2	14	424	44.4	8.56	0.928	1.546	0.985																
														VSEPLLWELFLQAGPVVNTHMPK	8	1	1	N-Term(iTRAQ8plex); K23(iTRAQ8plex)	1.123	-	0.711	6.94	66	0	3213.79941	3	1071.937988	1.204273588	64.92813167
														NQDATVYVGGLDEK	2	1	1	N-Term(iTRAQ8plex); K14(iTRAQ8plex)	0.767	1.546	1.366	4.62	37	0	2117.11686	3	706.3771362	-7.867236308	33.19617167
														VSEPLLWELFLQAGPVVNTHMPK	4	1	1	N-Term(iTRAQ8plex); M21(Oxidation); K23(iTRAQ8plex)	-	-	-	4.05	32	0	3229.79116	3	1071.937988	1.204273588	64.92813167
5032087	SF3A1	splicing factor 3A subunit 1	NP_005868.1	9.58	5	5	8	793	88.8	5.22	0.955	0.809	0.705																
														ASKPLPPAPAPDEYLVSPITGEK	2	1	1	N-Term(iTRAQ8plex); K3(iTRAQ8plex); K23(iTRAQ8plex)	-	-	-	5.1	38	0	3289.87197	4	823.2234497	-2.791097028	33.38909167
														VMQQQQQTTQQQLPQK	2	1	1	N-Term(iTRAQ8plex); K16(iTRAQ8plex)	0.753	0.639	0.562	4.87	44	0	2550.40812	3	850.8075562	6.36914027	23.82893833
														EDSAPSKPVVGIIYPPPEVR	2	1	1	N-Term(iTRAQ8plex); K7(iTRAQ8plex)	0.721	0.457	0.521	4.11	30	0	2758.55454	3	920.1896973	-1.987545669	32.78662333
														EVLDQVCYR	1	1	1	N-Term(iTRAQ8plex); C7(Methylthio)	1.211	1.109	1.22	2.81	-	0	1474.72783	2	737.8675537	-3.906821316	35.78591
														LTAQFVAR	1	1	1	N-Term(iTRAQ8plex)	1.247	1.025	0.884	2.79	-	0	1209.72038	2	605.3638306	-4.419853758	26.24182167
5032093	SLC1A5	neutral amino acid transporter B(0) isoform 1	NP_005619.1	10.54	4	4	14	541	56.6	5.48	1.855	0.511	0.748																
														SELPLDPLPVPTEEGNPLLK	2	3	1	N-Term(iTRAQ8plex); K20(iTRAQ8plex)	4.093	2.035	0.782	4.41	43	0	2766.56925	3	922.8612671	-2.083186775	50.189255
														EVLDSFLDLAR	6	2	1	N-Term(iTRAQ8plex)	1.788	0.614	0.82	4.38	58	0	1581.87529	2	791.4412842	-2.35768309	55.84335333
														LSAFVFPGELLLR	2	2	1	N-Term(iTRAQ8plex)	1.855	0.511	0.73	4.37	47	0	1766.05242	2	883.5298462	0.31961838	65.90877333
														NIFPSNLVSAAFR	4	2	1	N-Term(iTRAQ8plex)	1.194	0.377	0.595	3.02	43	0	1739.97617	3	580.6635742	0.872486806	45.38273667
5032137	VAMP7	vesicle-associated membrane protein 7 isoform 1	NP_005629.1	8.18	1	1	1	220	24.9	8.60	1.401	0.903	1.383																
														HAWCGGNFLEVTEQILAK	1	2	1	N-Term(iTRAQ8plex); C4(Methylthio); K18(iTRAQ8plex)	1.401	0.903	1.383	3.71	-	0	2670.39402	3	890.8028564	-1.975818561	62.63436833
5032179	TRIM28	transcription intermediary factor 1-beta	NP_005753.1	11.74	9	9	18	835	88.5	5.77	0.995	1.063	1.027																
														FQWDLNAWTK	2	1	1	N-Term(iTRAQ8plex); K10(iTRAQ8plex)	0.9	0.931	0.994	4.6	38	0	1917.04082	2	959.0240479	-3.704781246	69.28187833
														FASWALESDNNTALLLSK	2	1	1	N-Term(iTRAQ8plex); K18(iTRAQ8plex)	1.029	1.411	1.196	4.3	42	0	2588.40268	3	863.4724121	-5.952791649	43.98525167
														DHQYQFLEDAVR	2	1	1	N-Term(iTRAQ8plex)	2.105	1.811	1.586	3.77	30	0	1824.90781	3	608.9741211	-5.716052445	25.53991
														DIVENYFMR	3	1	1	N-Term(iTRAQ8plex)	1.007	0.8	0.9	3.67	28	0	1490.76164	2	745.8844604	0.075785715	36.78919667
														MIVDPVEPHGEMK	2	1	1	N-Term(iTRAQ8plex); M1(Oxidation); K13(iTRAQ8plex)	0.965	0.839	0.898	3.55	-	0	2106.11478	2	1045.563721	-1.601110695	24.63309167
														MIVDPVEPHGEMK	2	1	1	N-Term(iTRAQ8plex); K13(iTRAQ8plex)	0.925	1.063	1.027	3.41	45	0	2090.12016	2	1045.563721	-1.601110695	24.63309167
														KLIYFQLHR	2	1	1	N-Term(iTRAQ8plex); K1(iTRAQ8plex)	-	-	-	3.12	40	1	1826.11916	4	457.2852478	-3.794916131	27.31676
														SGEGEVSGLMR	1	1	1	N-Term(iTRAQ8plex)	0.724	0.541	0.623	-	34	0	1425.72441	2	713.3658447	-4.607108369	16.46818833
														LTEDKADVQSIIGLQR	1	1	1	N-Term(iTRAQ8plex); K5(iTRAQ8plex)	1.02	1.527	1.345	-	30	1	2394.37479	3	798.7964478	-2.723587096	37.637115
														LIYFQLHR	1	1	1	N-Term(iTRAQ8plex); Y3(iTRAQ8plex)	0.722	1.644	1.815	-	28	0	1698.02859	4	425.2626038	-1.493579649	31.366225
5032181	TIMM17B	mitochondrial import inner membrane translocase subunit Tim17-B isoform 2	NP_005825.1	12.79	1	1	2	172	18.3	9.03	1.029	1.364	1.216																
														GKEDPWNSITSGALTGAVLAAR	2	2	1	N-Term(iTRAQ8plex); K2(iTRAQ8plex)	1.029	1.364	1.216	5.64	43	1	2822.55667	3	941.5237427	-1.943813105	45.43887333
5174419	CLPP	ATP-dependent Clp protease proteolytic subunit, mitochondrial precursor	NP_006003.1	8.30	2	2	2	277	30.2	8.09	0.722	0.704	0.667																
														QSLQVIESAMER	1	1	1	N-Term(iTRAQ8plex)	0.747	0.775	0.741	3.44	-	0	1694.91313	2	847.9602051	4.84522545	36.22332833
														IMIHQPSGGAR	1	1	1	N-Term(iTRAQ8plex)	0.698	0.639	0.6	3.11	-	0	1470.8071	3	490.9405518	-5.581477375	15.085505
5174447	RACK1	receptor of activated protein C kinase 1	NP_006089.1	68.77	15	15	66	317	35.1	7.69	1.229	2.598	2.677																
														IIVDELKQEVISTSSK	6	1	1	N-Term(iTRAQ8plex); K7(iTRAQ8plex); K16(iTRAQ8plex)	-	-	-	5.47	50	1	2701.60024	5	541.1251221	-5.543928716	47.36304167
														DGQAMLWDLNEGK	6	1	1	N-Term(iTRAQ8plex); K13(iTRAQ8plex)	1.495	3.133	3.06	5.42	51	0	2085.07427	2	1043.041992	-6.164542989	49.62758667
														YTVQDESHSEWVSCVR	10	1	1	N-Term(iTRAQ8plex); C14(Methylthio)	1.457	2.079	2.003	4.92	53	0	2275.03598	3	759.0168457	-2.882785447	31.69356833
														HLYTLDGGDIINALCFSPNR	[illegible]	[illegible]	[illegible]	[illegible]	[illegible]	[illegible]	[illegible]	[illegible]	[illegible]	[illegible]	[illegible]	[illegible]	[illegible]	[illegible]	[illegible]
														GHNGWVTQIATTPQFPDMILSASR	[illegible]	[illegible]	[illegible]	[illegible]	[illegible]	[illegible]	[illegible]	[illegible]	[illegible]	[illegible]	[illegible]	[illegible]	[illegible]	[illegible]	[illegible]
														DVLSVAFSSDNR	[illegible]	[illegible]	[illegible]	[illegible]	[illegible]	[illegible]	[illegible]	[illegible]	[illegible]	[illegible]	[illegible]	[illegible]	[illegible]	[illegible]	[illegible]
														[illegible]	[illegible]	[illegible]	[illegible]	[illegible]	[illegible]	[illegible]	[illegible]	[illegible]	[illegible]	[illegible]	[illegible]	[illegible]	[illegible]	[illegible]	[illegible]
														LWNTLGVCK	[illegible]	[illegible]	[illegible]	[illegible]	[illegible]	[illegible]	[illegible]	[illegible]	[illegible]	[illegible]	[illegible]	[illegible]	[illegible]	[illegible]	[illegible]
														VWNLANCK	[illegible]	[illegible]	[illegible]	[illegible]	[illegible]	[illegible]	[illegible]	[illegible]	[illegible]	[illegible]	[illegible]	[illegible]	[illegible]	[illegible]	[illegible]
														LWDLTTGTTTR	[illegible]	[illegible]	[illegible]	[illegible]	[illegible]	[illegible]	[illegible]	[illegible]	[illegible]	[illegible]	[illegible]	[illegible]	[illegible]	[illegible]	[illegible]
														VWQVTIGTR	[illegible]	[illegible]	[illegible]	[illegible]	[illegible]	[illegible]	[illegible]	[illegible]	[illegible]	[illegible]	[illegible]	[illegible]	[illegible]	[illegible]	[illegible]
														[illegible]	[illegible]	[illegible]	[illegible]	[illegible]	[illegible]	[illegible]	[illegible]	[illegible]	[illegible]	[illegible]	[illegible]	[illegible]	[illegible]	[illegible]	[illegible]
														DRTIREWK	[illegible]	[illegible]	[illegible]	[illegible]	[illegible]	[illegible]	[illegible]	[illegible]	[illegible]	[illegible]	[illegible]	[illegible]	[illegible]	[illegible]	[illegible]

														IWDLEGK	5	1	1	N-Term(iTRAQ8plex); K7(iTRAQ8plex)	1.213	4.168	3.476	2.81	29	0	1468.85124	2	734.9292603	-7.330498737	30.39168667
														TNHIGHTGYLNTVTVSPDGSLCASGGK	1	1	1	N-Term(iTRAQ8plex); Y9(iTRAQ8plex); C22(Methylthio); K27(iTRAQ8plex)	-	-	-	-	32	0	3644.90322	4	911.9812622	2.844149077	29.14714333
														AEPPQCTSLAWSADGQTLFAGYTDNLVR	1	1	1	N-Term(iTRAQ8plex); C6(Methylthio)	-	-	-	-	27	0	3361.61972	3	1121.211426	1.830008822	60.01747
														HLYTLDGGDIINALCFSPNR	1	1	1	N-Term(iTRAQ8plex); Y3(iTRAQ8plex); C15(Methylthio)	1.698	2.829	3.193	-	25	0	2873.4827	3	857.0979004	-2.095676575	58.23341833
5174449	H1FX	histone H1x	NP_006017.1	7.04	1	1	4	213	22.5	10.76	0.842	0.549	1.175																
														GAPAAATAPAPTAHK	4	1	1	N-Term(iTRAQ8plex); K15(iTRAQ8plex)	0.842	0.549	1.175	6	60	0	1940.11479	2	970.5610352	-1.354356716	12.82092
5174529	MAT2A	S-adenosylmethionine synthase isoform type-2	NP_005902.1	8.10	2	2	8	395	43.6	6.48	0.886	2.276	2.371																
														ICDQISDAVLDAHLQQDPDAK	2	1	1	N-Term(iTRAQ8plex); C2(Methylthio); K21(iTRAQ8plex)	-	1.045	1.834	6.26	26	0	2949.47988	4	738.1254272	-3.669526855	52.02696
														TQVTVQYMQDR	4	1	1	N-Term(iTRAQ8plex); M8(Oxidation)	0.801	2.276	2.268	5.37	45	0	1688.86136	3	563.6253052	2.022651131	20.84691167
														TQVTVQYMQDR	2	1	1	N-Term(iTRAQ8plex)	1.061	4.356	4.965	4.46	23	0	1672.86296	3	563.6253052	2.022651131	20.84691167
5174539	MDH1	malate dehydrogenase, cytoplasmic isoform MDH1	NP_005908.1	24.85	6	6	17	334	36.4	7.36	0.930	3.841	3.332																
														VIVVGNPANTNCLTASK	2	4	1	N-Term(iTRAQ8plex); C12(Methylthio); K17(iTRAQ8plex)	0.949	2.725	3.156	6.8	52	0	2355.30521	3	785.7732544	2.847882969	33.76092333
														EVGVYEALKDDSWLK	3	4	1	N-Term(iTRAQ8plex); K9(iTRAQ8plex); K15(iTRAQ8plex)	0.77	2.994	3.518	6.55	31	1	2664.47988	4	666.8754272	-8.002385678	44.89073333
														GEFVTTVQQR	4	4	1	N-Term(iTRAQ8plex)	0.863	4.241	4.722	4.88	40	0	1468.81382	3	490.276123	5.204601524	24.86382
														FVEGLPINDFSR	4	4	1	N-Term(iTRAQ8plex)	1.128	5.485	6.103	4.39	41	0	1697.90972	2	849.4584961	-3.978852608	46.03885667
														DLDVAILVGSMPR	2	3	1	N-Term(iTRAQ8plex)	0.744	2.175	2.603	4.12	69	0	1689.9428	2	845.4750366	-4.949681036	50.45844167
														ELTEEKESAFEFLSSA	2	3	1	N-Term(iTRAQ8plex); K6(iTRAQ8plex)	2.146	6.673	2.284	3.42	26	1	2425.25351	3	809.0893555	-2.477326068	74.667835
5174613	NAP1L4	nucleosome assembly protein 1-like 4	NP_005960.1	20.80	5	6	46	375	42.8	4.69	0.733	1.568	1.928																
														QVPNESFFNFFNPLK	5	1	1	N-Term(iTRAQ8plex); K15(iTRAQ8plex)	0.685	1.398	1.928	4.47	49	0	2436.31363	3	812.776062	-1.481137238	48.75015333
														LDNVPHTPSSYIETLPK	2	1	1	N-Term(iTRAQ8plex); K17(iTRAQ8plex)	0.946	2.816	1.691	4.02	25	0	2519.38456	3	840.4663696	-4.781192793	34.273385
														NVDMLSELVQEYDEPILK	2	1	1	N-Term(iTRAQ8plex); K18(iTRAQ8plex)	0.59	1.732	3.21	3.8	75	0	2743.47783	2	1372.242554	3.407901376	56.68298167
														GIPEFWFTIFR	2	1	1	N-Term(iTRAQ8plex)	0.667	2.07	3.588	3.64	35	0	1716.95439	2	858.980835	7.473699899	65.33216
														NASNTEK	1	1	1	N-Term(iTRAQ8plex); K7(iTRAQ8plex)	1.122	1.332	1.014	2.88	-	0	1371.78202	3	457.9321899	9.619075086	13.13314167
														FYEEVHDLER	33	4	2	N-Term(iTRAQ8plex)	-	-	-	4.07	45	0	1640.81559	3	547.6083984	-7.050927879	38.88224833
														FYEEVHDLER	1	4	2	N-Term(iTRAQ8plex); Y2(iTRAQ8plex)	-	-	-	-	37	0	1945.04119	3	547.6083984	-7.050927879	38.88224833
5174661	S100A2	protein S100-A2	NP_005969.1	26.80	3	3	9	97	11.0	4.78	0.346	1.102	0.141																
														ELPSFVGEKVDEEGLK	2	1	1	N-Term(iTRAQ8plex); K9(iTRAQ8plex); K16(iTRAQ8plex)	0.405	1.194	0.077	5.09	-	1	2688.51674	4	672.8846436	-2.085522161	36.80313167
														YSCQEGDKFK	2	1	1	N-Term(iTRAQ8plex); C3(Methylthio); K8(iTRAQ8plex); K10(iTRAQ8plex)	0.279	1.076	0.201	4.54	27	1	2163.13515	4	541.5392456	0.467026702	25.04240667
														ELPSFVGEK	5	1	1	N-Term(iTRAQ8plex); K9(iTRAQ8plex)	0.341	1.13	0.141	3.24	37	0	1613.93584	2	807.4715576	-0.045036254	38.14236
														EIIHK	1	2	2	N-Term(iTRAQ8plex); K5(iTRAQ8plex)	-	-	-	-	20	0	1247.79375	2	624.4005127	0.454779186	17.22253333
5174735	TUBB4B	tubulin beta-4B chain	NP_006079.1	70.34	3	22	525	445	49.8	4.89	1.075	3.133	2.367																
														INVYYNEATGGK	12	1	1	N-Term(iTRAQ8plex); K12(iTRAQ8plex)	0.953	2.79	2.091	4.99	62	0	1937.04888	2	969.0280762	-5.155046099	28.32962
														FWEVISDEHGIDPTGTYHGDSDLQLER	9	4	1	N-Term(iTRAQ8plex)	-	-	-	4.73	29	0	3420.63198	3	1140.877319	-3.296639423	48.54329
														AVLVDLEPGTMDSVR	6	5	1	N-Term(iTRAQ8plex)	1.302	4.081	2.927	4.57	68	0	1906.01933	2	953.5133057	-3.387474491	40.95937167
														AVLVDLEPGTMDSVR	4	5	1	N-Term(iTRAQ8plex); M11(Oxidation)	1.073	2.748	2.061	3.25	40	0	1922.01775	2	953.5133057	-3.387474491	40.95937167
														EIVHIQAGQCGNQIGAK	24	9	4	N-Term(iTRAQ8plex); C10(Methylthio); K17(iTRAQ8plex)	-	-	-	6.74	73	0	2420.28691	2	1210.647095	-5.366911999	28.04771167
														EAESCDCLQGFQLTHSLGGGTGSGMGTLLISK	9	12	2	N-Term(iTRAQ8plex); C5(Methylthio); C7(Methylthio); K32(iTRAQ8plex)	-	-	-	5.55	34	0	3897.87046	3	1299.96167	-1.802723937	57.84804
														YLTVAAVFR	48	9	2	N-Term(iTRAQ8plex)	-	-	-	3.6	59	0	1343.80103	3	448.6051941	1.590804996	40.796475
														LHFFMPGFAPLTSR	105	21	4	N-Term(iTRAQ8plex)	-	-	-	5.26	73	0	1925.03666	3	642.350647	-1.861590873	50.37672667
														LHFFMPGFAPLTSR	9	21	4	N-Term(iTRAQ8plex); M5(Oxidation)	-	-	-	4.68	37	0	1941.02982	3	642.350647	-1.861590873	50.37672667
														NSSYFVEWIPNNVK	31	20	5	N-Term(iTRAQ8plex); K14(iTRAQ8plex)	-	-	-	4.24	49	0	2305.22641	2	1153.123413	-1.809680962	42.78834
														FPGQLNADLR	9	25	5	N-Term(iTRAQ8plex)	-	-	-	4.12	55	0	1434.7963	3	478.9369507	-3.068379269	31.15012333
														LAVNMVPFPR	13	25	5	N-Term(iTRAQ8plex)	-	-	-	4.01	51	0	1447.83405	3	483.2828674	-3.931565971	41.77579833
														LAVNMVPFPR	4	25	5	N-Term(iTRAQ8plex); M5(Oxidation)	-	-	-	3.45	44	0	1463.82685	3	483.2828674	-3.931565971	41.77579833
														KLAVNMVPFPR	15	25	5	N-Term(iTRAQ8plex); K1(iTRAQ8plex)	-	-	-	3.44	37	1	1880.12295	4	470.7861938	-9.108866745	31.42844
														FPGQLNADLRK	1	25	5	N-Term(iTRAQ8plex); K11(iTRAQ8plex)	-	-	-	3.07	-	1	1867.09755	4	467.5298462	-1.861935048	33.46670167
														NMMAACDPR	3	23	5	N-Term(iTRAQ8plex); C6(Methylthio)	-	-	-	-	55	0	1358.59062	2	679.7989502	-6.255401168	21.43194333
														NMMAACDPR	5	23	5	N-Term(iTRAQ8plex); M3(Oxidation); C6(Methylthio)	-	-	-	-	47	0	1374.58525	2	679.7989502	-6.255401168	21.43194333
														NMMAACDPR	1	23	5	N-Term(iTRAQ8plex); M2(Oxidation); M3(Oxidation); C6(Methylthio)	-	-	-	-	34	0	1390.5822	2	679.7989502	-6.255401168	21.43194333
														LTTPTYGDLNHLVSATMSGVTTCLR	95	12	3	N-Term(iTRAQ8plex); C23(Methylthio)	-	-	-	7.13	75	0	3001.51689	2	1501.262085	2.319161318	66.15877833
														SGPFGQIFRPDNFVFGQSGAGNNWAK	9	12	3	N-Term(iTRAQ8plex); K26(iTRAQ8plex)	-	-	-	5.17	30	0	3406.74965	4	852.442749	-1.486779919	46.41363167
														LTTPTYGDLNHLVSATMSGVTTCLR	14	12	3	N-Term(iTRAQ8plex); M17(Oxidation); C23(Methylthio)	-	-	-	5	31	0	3017.50541	2	1501.262085	2.319161318	66.15877833
														EVDEQMLNVQNK	4	9	3	N-Term(iTRAQ8plex); K12(iTRAQ8plex)	-	-	-	4.36	65	0	2055.08745	2	1028.047363	-6.160982347	33.78303333
														EVDEQMLNVQNK	2	9	3	N-Term(iTRAQ8plex); M6(Oxidation); K12(iTRAQ8plex)	-	-	-	4.18	46	0	2071.07792	2	1028.047363	-6.160982347	33.78303333
														TAVCDIPPR	12	17	3	N-Term(iTRAQ8plex); C4(Methylthio)	-	-	-	2.88	36	0	1321.68877	2	661.3473511	-2.713782013	26.90244667
														IREEYPDR	3	13	3	N-Term(iTRAQ8plex)	-	-	-	-	29	1	1381.73252	3	461.2490234	-3.788169878	16.81976833
														GHYTEGAELVDSVLDVVR	51	11	4	N-Term(iTRAQ8plex)	-	-	-	5.05	98	0	2263.18999	2	1132.098633	1.214084992	52.94817667
														GHYTEGAELVDSVLDVVR	10	11	4	N-Term(iTRAQ8plex); Y3(iTRAQ8plex)	-	-	-	-	64	0	2567.37212	2	1132.098633	1.214084992	52.94817667
														IMNTFSVVPSPK	18	11	3	N-Term(iTRAQ8plex); K12(iTRAQ8plex)	-	-	-	4.54	51	0	1928.10552	2	964.553772	-6.900042966	31.209235
														IMNTFSVVPSPK	2	11	3	N-Term(iTRAQ8plex); M2(Oxidation); K12(iTRAQ8plex)	-	-	-	3.64	37	0	1944.09331	2	964.553772	-6.900042966	31.209235
														EAESCDCLQGFQLTHSLGGGTGSGMGTLLLSK	5	12	2	N-Term(iTRAQ8plex); C5(Methylthio); C7(Methylthio); M25(Oxidation); [illegible]	-	-	-	4.96	30	0	3913.86538	4	975.2184448	-6.55075756	57.66156667
														MSATFIGNSTAIQELFKR	2	4	2	N-Term(iTRAQ8plex); K17(iTRAQ8plex)	-	-	-	4.35	29	1	2622.45424	4	656.3690186	0.306216496	50.822585
														ALTVPELTQQMFDAK	10	7	2	N-Term(iTRAQ8plex); K15(iTRAQ8plex)	-	-	-	5.83	52	0	2300.27726	3	772.7599487	-3.33008426	35.96597833
														ALTVPELTQQMFDAK	2	7	2	N-Term(iTRAQ8plex); M11(Oxidation); K15(iTRAQ8plex)	-	-	-	4.01	60	0	2316.26529	3	772.7599487	-3.33008426	35.96597833
5453555	RAN	GTP-binding nuclear protein Ran isoform 1	NP_006316.1	32.87	7	7	90	216	24.4	7.49	1.024	3.075	2.014																
														FNVWDTAGQEK	6	2	1	N-Term(iTRAQ8plex); K11(iTRAQ8plex)	1.334	2.962	2.152	5.31	55	0	1903.00102	2	952.0041504	-8.406269123	41.40875167
														SNYNFEKPFLWLAR	12	4	1	N-Term(iTRAQ8plex); K7(iTRAQ8plex)	1.096	4.073	2.711	5.02	33	0	2393.32158	2	1197.164429	-0.447285536	48.39871333
														VCENIPIVLCGNK	40	4	1	N-Term(iTRAQ8plex); C2(Methylthio); C10(Methylthio); K13(iTRAQ8plex)	0.853	3.59	2.496	4.93	62	0	2102.09868	2	1051.552979	-4.958325148	46.22422333
														YVATLGVEVHPLVFHTNR	18	2	1	N-Term(iTRAQ8plex)	1.34	2.285	1.664	4.76	52	0	2356.3013	3	786.1134033	7.513991492	39.28428833
														KYVATLGVEVHPLVFHTNR	4	2	1	N-Term(iTRAQ8plex); K1(iTRAQ8plex)	-	-	-	4.65	35	1	2788.62989	3	930.2144775	7.386449785	34.57085667
														VCENIPIVLCGNKVDIK	2	4	1	N-Term(iTRAQ8plex); C2(Methylthio); C10(Methylthio); K13(iTRAQ8plex); [illegible]	-	-	-	4.43	-	1	2861.57509	4	716.149231	-4.815303259	56.30526333
														NLQYYDISAK	5	4	1	N-Term(iTRAQ8plex); K10(iTRAQ8plex)	1.001	1.922	1.427	4.31	46	0	1823.00493	2	912.0061035	-6.028995625	25.01388833
														YVATLGVEVHPLVFHTNR	3	2	1	N-Term(iTRAQ8plex); Y1(iTRAQ8plex)	2.039	5.186	2.207	-	44	0	2660.52976	3	786.1134033	7.513991492	39.28428833
5453565	BAIAP2	brain-specific angiogenesis inhibitor 1-associated protein 2 isoform 3	NP_006331.1	2.69	1	1	2	520	57.4	8.79	1.046	0.445	0.605																
														ELGDVLFQMAEVHR	2	13	1	N-Term(iTRAQ8plex)	1.046	0.445	0.605	4.68	66	0	1948.02866	3	650.0144043	1.133628406	48.82423167
5453597	CAPZA1	F-actin-capping protein subunit alpha-1	NP_006126.1	37.41	6	7	23	286	32.9	5.69	0.743	1.094	1.024																
														DVQDSLTVSNEAQTAK	1	2	1	N-Term(iTRAQ8plex); K16(iTRAQ8plex)	0.866	1.142	0.849	5.87	-	0	2314.2396	3	772.0847168	2.11984992	26.35993
														IEGYEDQVLITEHGDLGNSR	2	3	1	N-Term(iTRAQ8plex)	0.743	0.958	1.178	5.54	54	0	2549.27671	3	850.4304199	-0.735418156	36.45241167
														FTITPPTAQVVGVLK	2	3	1	N-Term(iTRAQ8plex); K15(iTRAQ8plex)	1.321	1.171	1.239	4.83	24	0	2179.3295	3	727.1146851	-0.733883618	46.89744667
														FITHAPPGEFNEVFNDVR	8	3	1	N-Term(iTRAQ8plex)	0.549	1.087	0.977	4.79	41	0	2393.21372	3	798.4108887	-0.463198222	41.65565833
														TIDGQQTIIACIESHQFQPK	5	3	1	N-Term(iTRAQ8plex); C11(Methylthio); K20(iTRAQ8plex)	-	-	-	4.33	27	0	2911.53066	4	728.6381226	1.369744935	49.26085667
														ESCDSALR	1	3	1	N-Term(iTRAQ8plex); C3(Methylthio)	0.77	1.094	1.083	-	20	0	1230.57073	2	615.7890015	-4.311812559	19.43231833
														LLLNNDNLLR	4	4	2	N-Term(iTRAQ8plex)	-	-	-	3.34	49	0	1501.89202	2	751.449646	-5.590234002	29.13142667
5453599	CAPZA2	F-actin-capping protein subunit alpha-2	NP_006127.1	9.79	1	2	16	286	32.9	5.85	0.435	0.902	1.546																
														FIIHAPPGEFNEVFNDVR	12	1	1	N-Term(iTRAQ8plex)	0.435	0.902	1.546	4.84	50	0	2405.24979	3	802.4214478	-2.420242715	44.94949
														LLLNNDNLLR	4	4	2	N-Term(iTRAQ8plex)	-	-	-	3.34	49	0	1501.89202	2	751.449646	-5.590234002	29.13142667
5453601	CRTAP	cartilage-associated protein precursor	NP_006362.1	2.74	1	1	1	401	46.5	5.73	0.749	0.860	0.860																
														AIAAAHTFLLK	1	1	1	N-Term(iTRAQ8plex); K11(iTRAQ8plex)	0.749	0.86	0.86	3.75	-	0	1764.09043	3	588.7016602	-4.990719928	34.696445
5453603	CCT2	T-complex protein 1 subunit beta isoform 1	NP_006422.1	48.04	19	19	112	535	57.5	6.46	0.986	2.315	1.909																
														TVYGGGCSEMLMAHAVTQLANR	14	2	1	N-Term(iTRAQ8plex); C7(Methylthio)	0.916	1.795	1.265	6.52	57	0	2659.27512	3	887.0965576	-0.598922024	49.34856833
														LTSFIGAIAIGDLVK	14	1	1	N-Term(iTRAQ8plex); K15(iTRAQ8plex)	1.364	5.609	3.238	6.45	66	0	2126.30234	2	1063.659546	3.411483125	63.04494167
														LALVTGGEIASTFDHPELVK	14	2	1	N-Term(iTRAQ8plex); K20(iTRAQ8plex)	0.986	2.938	1.951	6.32	43	0	2705.52993	3	902.515564	-0.492566859	52.12627833
														EGTIGDMAILGITESFQVK	2	2	1	N-Term(iTRAQ8plex); K19(iTRAQ8plex)	0.421	0.747	0.806	6.15	40	0	2617.44266	3	878.4807129	-1.568631751	47.58656333
														AAHSEGNTTAGLDMR	11	2	1	N-Term(iTRAQ8plex); M14(Oxidation)	0.939	3.178	2.247	5.77	60	0	1850.89139	2	917.9528198	-1.966469443	14.14578833
														GATQQILDEAER	6	2	1	N-Term(iTRAQ8plex)	0.955	3.014	1.927	5.53	72	0	1634.8698	2	817.9385376	2.836479894	28.220425
														QVLLSAAEAAEVILR	4	2	1	N-Term(iTRAQ8plex)	1.675	5.521	3.528	5.48	81	0	1887.12192	3	629.7121582	0.100713882	65.96403667
														AAHSEGNTTAGLDMR	14	2	1	N-Term(iTRAQ8plex)	1.031	2.124	1.584	5.48	72	0	1834.89676	2	917.9528198	-1.966469443	14.14578833
														SLHDALCVLAQTVK	8	2	1	N-Term(iTRAQ8plex); C7(Methylthio); K14(iTRAQ8plex)	0.98	3.902	2.27	5.47	45	0	2152.20371	3	718.0727539	-1.939329086	50.79119
														EGTIGDMAILGITESFQVK	2	2	1	N-Term(iTRAQ8plex); M7(Oxidation); K19(iTRAQ8plex)	0.854	2.526	2.228	5.02	56	0	2633.42759	3	878.4807129	-1.568631751	47.58656333
														FRQDLMNIAGTTLSSK	5	2	1	N-Term(iTRAQ8plex); K16(iTRAQ8plex)	0.713	2.2	1.891	4.67	35	1	2390.32834	3	797.4476318	-1.635357837	44.22086667
														VQDDEVGDGTTSVTVLAAELLR	2	2	1	N-Term(iTRAQ8plex)	1.331	4.388	3.383	4.57	75	0	2592.35356	2	1296.68042	-5.218453394	59.36673333
														DASLMVTNDGATILK	2	2	1	N-Term(iTRAQ8plex); K15(iTRAQ8plex)	1.18	3.051	2	4.37	33	0	2157.20683	2	1079.107056	1.039959678	32.71544167
														HGINCFINR	3	2	1	N-Term(iTRAQ8plex); C5(Methylthio)	0.791	1.488	1.703	3.46	30	0	1423.71677	3	475.2437744	-5.100949828	23.5527
														QDLMNIAGTTLSSK	1	2	1	N-Term(iTRAQ8plex); K14(iTRAQ8plex)	1.173	2.27	1.574	3.28	-	0	2087.14725	3	696.3872681	-7.407407778	61.79834667
														ILIANTGMDTDKIK	1	2	1	N-Term(iTRAQ8plex); M8(Oxidation); K12(iTRAQ8plex); K14(iTRAQ8plex)	1.296	4.081	2.406	3.02	-	1	2461.43003	3	821.1481934	-6.626014836	26.89986167
														KIHPQTIIAGWR	1	2	1	N-Term(iTRAQ8plex); K1(iTRAQ8plex)	1.131	1.948	1.653	3	-	1	2028.22208	3	676.7455444	-5.241659345	25.99037667
														IHPQTIIAGWR	2	2	1	N-Term(iTRAQ8plex)	0.975	1.333	0.734	2.91	30	0	1595.92142	3	532.6453247	-6.867774793	28.25293167
														MLPTIIADNAGYDSADLVAQLR	1	2	1	N-Term(iTRAQ8plex)	0.336	1.129	1.268	2.72	-	0	2651.40909	4	739.6591797	2.648372773	55.28640667
														LAVEAVLR	3	2	1	N-Term(iTRAQ8plex)	1.272	3.282	2.239	2.69	23	0	1174.73796	2	587.8726196	-6.962116189	25.58269167
														MLPTIIADNAGYDSADLVAQLR	1	2	1	N-Term(iTRAQ8plex); Y12(iTRAQ8plex)	-	-	-	-	28	0	2955.61489	4	739.6591797	2.648372773	55.28640667
														VLVDMSR	1	2	1	N-Term(iTRAQ8plex)	1.206	1.796	1.072	-	21	0	1123.63872	2	562.322998	-5.332520272	26.06623333
5453678	NPC2	epididymal secretory protein E1 precursor	NP_006423.1	31.79	3	3	36	151	16.6	7.65	0.658	0.763	0.990																
														AVVHGILMGVPVPFPIPEPDGCK	23	2	1	N-Term(iTRAQ8plex); C22(Methylthio); K23(iTRAQ8plex)	-	0.953	1.213	5.7	65	0	3026.65312	2	1513.8302	1.331989646	59.35656833
														AVVHGILMGVPVPFPIPEPDGCK	7	2	1	N-Term(iTRAQ8plex); M8(Oxidation); C22(Methylthio); K23(iTRAQ8plex)	-	-	1.994	4.79	37	0	3042.65403	2	1513.8302	1.331989646	59.35656833
														EVNVSPCPTQPCQLSK	5	2	1	N-Term(iTRAQ8plex); C7(Methylthio); C12(Methylthio); K16(iTRAQ8plex)	0.66	0.646	0.887	3.99	34	0	2430.20542	3	810.7399902	-2.298508116	35.317125

														DKTYSYLNK	1	2	1	N-Term(iTRAQ8plex); K2(iTRAQ8plex); K9(iTRAQ8plex)	0.655	0.756	1.002	2.8	-	1	2044.1713	3	682.0619507	-6.292280508	16.02266667
5453682	HEXIM1	protein HEXIM1	NP_006451.1	3.90	1	1	4	359	40.6	4.89	0.722	0.785	0.792																
														AENLQLLTENELHR	4	1	1	N-Term(iTRAQ8plex)	0.722	0.785	0.792	4.41	47	0	1984.07407	3	662.029541	-1.25071093	30.53508667
5453690	DNAJB1	dnaJ homolog subfamily B member 1 isoform 1	NP_006136.1	7.94	2	2	4	340	38.0	8.63	1.576	3.288	2.626																
														VSLEEIYSGCTK	2	5	1	N-Term(iTRAQ8plex); C10(Methylthio); K12(iTRAQ8plex)	0.95	2.642	2.508	3.89	34	0	1983.02085	3	661.6784668	-9.019148437	67.184695
														FKEIAEAYDVLSDPR	2	1	1	N-Term(iTRAQ8plex); K2(iTRAQ8plex)	2.614	4.091	2.75	3.65	48	1	2361.28946	3	787.7680054	-0.689638752	53.84284667
5453704	ARL6IP5	PRA1 family protein 3	NP_006398.1	16.49	2	2	15	188	21.6	9.77	0.408	0.414	0.527																
														RTPMGIVLDALEQQEEGINR	6	1	1	N-Term(iTRAQ8plex)	1.409	1.225	1.477	5.58	68	1	2573.37467	3	858.4630737	3.371697683	60.57332667
														AWDDFFPGSDR	9	1	1	N-Term(iTRAQ8plex)	0.401	0.301	0.363	3.84	30	0	1616.75859	2	808.8829346	-3.790782003	65.073085
5453710	LASP1	LIM and SH3 domain protein 1 isoform a	NP_006139.1	8.43	2	2	4	261	29.7	7.05	1.525	3.081	3.631																
														GFSVVADTPELQR	2	2	1	N-Term(iTRAQ8plex)	0.959	1.673	2.303	4.74	51	0	1722.9395	2	861.9733887	3.859348368	31.33255
														ACFHCETCK	2	1	1	N-Term(iTRAQ8plex); C2(Methylthio); C5(Methylthio); C8(Methylthio); K9(iTRAQ8plex)	2.426	5.674	5.723	2.89	17	0	1787.75241	3	596.5889893	-9.274089873	28.99847
5453740	MYL12A	myosin regulatory light chain 12A isoform 1	NP_006462.1	38.60	5	5	17	171	19.8	4.81	0.705	1.175	1.138																
														ATSNVFAMFDQSQIQEFK	4	5	1	N-Term(iTRAQ8plex); K18(iTRAQ8plex)	1.554	1.173	1.034	5.85	65	0	2699.38053	3	900.4650269	-5.725011141	42.26953833
														FTDEEVDELYR	4	3	1	N-Term(iTRAQ8plex)	0.662	1.162	1.138	4.4	57	0	1719.84282	2	860.4250488	2.843129727	33.12307167
														DGFIDKEDLHDMLASLGK	1	4	1	N-Term(iTRAQ8plex); K6(iTRAQ8plex); K18(iTRAQ8plex)	-	-	0.929	3.61	-	1	2916.58242	4	729.901062	-2.7577253	46.885885
														GNFNYIEFTR	7	3	1	N-Term(iTRAQ8plex)	0.671	1.252	1.436	3.49	44	0	1564.79778	2	782.9024048	-5.514035845	26.1912
														ELLTTMGDR	1	5	1	N-Term(iTRAQ8plex)	0.708	0.955	1.011	-	31	0	1339.71172	2	670.3594971	-5.69061185	20.51190167
5453760	NEDD8	NEDD8 precursor	NP_006147.1	34.57	2	2	3	81	9.1	8.43	1.050	1.328	1.724																
														ILGGSVLHLVLALR	2	1	1	N-Term(iTRAQ8plex)	-	-	-	2.85	22	0	1765.13871	3	589.0510864	1.20371693	63.56455833
														EIEIDIEPTDKVER	1	2	1	N-Term(iTRAQ8plex); K11(iTRAQ8plex)	1.05	1.328	1.724	2.7	-	1	2294.25821	3	765.4242554	-5.151310629	27.602005
5453790	NNMT	nicotinamide N-methyltransferase	NP_006160.1	22.35	4	4	12	264	29.6	5.74	0.765	1.245	2.510																
														KEPEAFDWSPVVTYVCDLEGNR	6	1	1	N-Term(iTRAQ8plex); K1(iTRAQ8plex); C16(Methylthio)	-	-	-	5.16	49	1	3208.5864	3	1070.200317	-1.253948332	63.60854833
														EAVEAAVK	2	1	1	N-Term(iTRAQ8plex); K8(iTRAQ8plex)	0.427	0.496	1.621	3.44	31	0	1424.84314	3	475.6192322	-9.676866441	26.20224
														NLGSLLKPGGFLVIMDALK	2	1	1	N-Term(iTRAQ8plex); K7(iTRAQ8plex); K19(iTRAQ8plex)	-	-	-	3.19	18	0	2898.75258	4	725.4436035	-3.190033379	65.80144167
														DTYLSHFNPR	1	1	1	N-Term(iTRAQ8plex)	1.373	3.128	3.887	2.69	-	0	1553.79398	4	465.2583313	2.547893447	21.85472
														DTYLSHFNPR	1	1	1	N-Term(iTRAQ8plex); Y3(iTRAQ8plex)	-	-	-	-	28	0	1858.0115	4	465.2583313	2.547893447	21.85472
5453880	ANP32A	acidic leucine-rich nuclear phosphoprotein 32 family member A	NP_006296.1	8.84	2	2	12	249	28.6	4.09	0.910	5.448	4.355																
														SLDLFNCEVTNLNDYR	7	2	1	N-Term(iTRAQ8plex); C7(Methylthio)	0.908	2.617	2.175	6.04	86	0	2266.06792	2	1133.537598	-4.714601629	53.75836833
														IHLELR	5	2	1	N-Term(iTRAQ8plex)	1.021	8.01	5.018	-	29	0	1084.67044	3	362.2283325	-7.003962767	28.18122
5453930	POLR2I	DNA-directed RNA polymerase II subunit RPB9	NP_006224.1	18.40	1	1	2	125	14.5	5.14	-	-	-																
														ITHEVDELTQIIADVSQDPTLPR	2	1	1	N-Term(iTRAQ8plex)	-	-	-	4.31	44	0	2894.54111	3	965.5185547	-0.086537897	66.89018167
5453994	RAD21	double-strand-break repair protein rad21 homolog	NP_006256.1	10.46	3	3	7	631	71.6	4.65	1.309	1.255	0.945																
														EAAYNAITLPEEFHDFDQPLPDLDDIDVAQQFSLNQSR	2	1	1	N-Term(iTRAQ8plex)	-	-	-	6.12	57	0	4666.27622	3	1556.096924	5.005476162	58.03576833
														TGAESISLLELCR	4	1	1	N-Term(iTRAQ8plex); C12(Methylthio)	1.243	1.208	0.933	4.37	54	0	1741.91509	2	871.4611816	1.173628534	53.68447167
														MAFRPGVVDLPEENR	1	1	1	N-Term(iTRAQ8plex)	1.467	1.372	0.945	2.91	-	0	2034.07029	3	678.6949463	-2.062702967	28.92621167
5453998	IPO7	importin-7	NP_006382.1	12.04	9	9	18	1038	119.4	4.82	0.947	1.947	2.270																
														NPVWYQALTHGLNEEQR	2	1	1	N-Term(iTRAQ8plex)	0.573	1.181	1.988	5.15	40	0	2359.20298	3	787.0725098	-2.846407534	38.90296167
														DVPNETLQVEEDDRPELPWWK	1	1	1	N-Term(iTRAQ8plex); K21(iTRAQ8plex)	-	-	0.735	5.05	-	0	3203.64248	4	801.6660767	-1.418553852	39.55925333
														QLQDIATLADQR	2	1	1	N-Term(iTRAQ8plex)	0.931	1.608	2.072	4.61	45	0	1675.9334	2	838.4703369	3.165942116	33.35783
														VLQQTLNYINQGVSHALTWK	2	1	1	N-Term(iTRAQ8plex); K20(iTRAQ8plex)	-	-	-	4.46	40	0	2921.6415	4	731.1658325	-1.465307046	68.48409833
														ENIVEAIIHSPELIR	2	1	1	N-Term(iTRAQ8plex)	0.165	0.617	0.564	3.74	29	0	2037.16843	3	679.7276611	1.854951403	56.94757
														FKSDQNLQTALELTR	2	1	1	N-Term(iTRAQ8plex); K2(iTRAQ8plex)	0.947	2.089	2.452	3.49	-	1	2372.33347	3	791.4493408	-2.513174956	34.86769833
														WALHILAR	4	1	1	N-Term(iTRAQ8plex)	1.884	3.099	3.927	3.37	49	0	1283.78235	3	428.5989685	-5.179252083	33.86832167
														GIDQCIPLFVEAALER	2	1	1	N-Term(iTRAQ8plex); C5(Methylthio)	-	-	-	3.31	43	0	2124.12027	3	708.7116089	3.159477474	68.98991167
														KQLQDIATLADQR	1	1	1	N-Term(iTRAQ8plex); K1(iTRAQ8plex)	1.131	2.08	2.27	2.88	-	1	2108.21555	3	703.4100342	-6.105642728	28.119395
5454052	SFN	14-3-3 protein sigma	NP_006133.1	39.11	6	8	65	248	27.8	4.74	0.527	2.064	1.047																
														TTFDEAMADLHTLSEDSYK	7	1	1	N-Term(iTRAQ8plex); K19(iTRAQ8plex)	0.438	2.064	1.191	6.12	39	0	2782.381	3	928.1295166	1.36044065	50.29688667
														GAVEKGEELSCEER	2	1	1	N-Term(iTRAQ8plex); K5(iTRAQ8plex); C11(Methylthio)	0.868	2.426	1.11	5.76	25	1	2190.09145	4	548.2783203	-3.511693539	23.98222333
														LGLALNFSVFHYEIANSPEEAISLAK	1	1	1	N-Term(iTRAQ8plex); K26(iTRAQ8plex)	-	1.908	0.89	4.46	-	0	3441.90268	3	1249.363159	-4.897662569	56.294415
														SAYQEAMDISK	2	1	1	N-Term(iTRAQ8plex); K11(iTRAQ8plex)	0.441	1.376	0.828	4.32	72	0	1850.96965	2	925.9884644	-4.429683141	34.92858333
														YEDMAAFMK	9	1	1	N-Term(iTRAQ8plex); K9(iTRAQ8plex)	0.545	2.215	1.047	4.25	49	0	1713.86405	2	857.4399414	-4.349860937	56.13789
														GEELSCEER	1	1	1	N-Term(iTRAQ8plex); C6(Methylthio)	0.396	1.377	0.743	-	41	0	1401.6237	2	701.3154907	-3.922218687	26.489335
														LGLALNFSVFHYEIANSPEEAISLAK	1	1	1	N-Term(iTRAQ8plex); Y12(iTRAQ8plex); K26(iTRAQ8plex)	1.017	7.024	2.814	-	28	0	3746.07492	3	1249.363159	-4.897662569	56.294415
														DSTLIMQLLR	24	9	7	N-Term(iTRAQ8plex)	-	-	-	4.73	54	0	1493.87629	3	498.6302795	6.656557269	46.36715833
														NLLSVAYK	16	10	7	N-Term(iTRAQ8plex); K8(iTRAQ8plex)	-	-	-	4.01	39	0	1515.92546	2	758.4644165	-9.192370231	25.08285833
														DSTLIMQLLR	2	9	7	N-Term(iTRAQ8plex); M6(Oxidation)	-	-	-	3.14	48	0	1509.85791	3	498.6302795	6.656557269	46.36715833
														VISSIEQK	2	5	5	N-Term(iTRAQ8plex); K8(iTRAQ8plex)	-	-	-	3.44	29	0	1511.91423	2	756.4607544	-7.341111487	22.45532333
5454064	RBM14	RNA-binding protein 14 isoform 1	NP_006319.1	18.83	9	9	25	669	69.4	9.67	1.079	0.792	0.691																
														AQPSASLGVGYR	4	1	1	N-Term(iTRAQ8plex)	0.911	0.831	0.598	4.96	67	0	1509.83402	3	503.9495239	0.859185399	23.44517667
														QFAFVHMR	2	5	1	N-Term(iTRAQ8plex)	1.123	0.839	0.679	4.6	39	0	1339.71567	3	447.2434082	-6.732727561	24.738185
														IFVGNVSAACTSQELR	2	5	1	N-Term(iTRAQ8plex); C10(Methylthio)	0.627	0.641	0.494	4.56	63	0	2045.04105	3	682.3518677	-2.514630529	46.44533
														AIEALHGHELRPGR	4	5	1	N-Term(iTRAQ8plex)	1.17	0.926	0.844	3.9	26	0	1860.04567	4	465.7668762	-2.659234262	23.56376833
														YSGSYNDYLR	4	1	1	N-Term(iTRAQ8plex)	0.953	0.604	0.653	3.61	29	0	1541.74663	2	771.3771362	-4.388177231	22.33103333
														KPGAGDTAFPGTGGFSATFDYQQAFGNSTGGFDGQAR	4	1	1	N-Term(iTRAQ8plex); K1(iTRAQ8plex)	-	-	-	3.31	41	0	4294.07876	4	1074.275146	2.332224924	44.67355667
														INVELSTK	2	2	1	N-Term(iTRAQ8plex); K8(iTRAQ8plex)	1.12	0.717	0.705	3.23	38	0	1511.92375	2	756.4655151	-1.043469658	31.95667833
														AQPSVSLGAAYR	2	1	1	N-Term(iTRAQ8plex)	0.401	0.338	0.225	2.98	26	0	1523.85588	2	762.4315796	4.929114212	25.23778667
														LAELSDYRR	1	1	1	N-Term(iTRAQ8plex)	1.78	0.971	0.875	-	27	1	1426.79203	3	476.2688599	-2.50559303	22.31276167
5454090	SSR4	translocon-associated protein subunit delta isoform 2 precursor	NP_006271.1	24.86	3	3	21	173	19.0	6.15	0.893	0.742	0.736																
														VQNMALYADVGGK	2	3	1	N-Term(iTRAQ8plex); K13(iTRAQ8plex)	1.298	0.739	0.691	5.37	76	0	1974.08293	2	987.545105	-5.555976842	44.07720833
														NNEDISIIPPLFTVSVDHR	7	4	1	N-Term(iTRAQ8plex)	0.959	0.949	0.926	4.61	50	0	2470.32053	3	824.1122437	-0.904170715	49.36495333
														VQNMALYADVGGK	2	3	1	N-Term(iTRAQ8plex); M4(Oxidation); K13(iTRAQ8plex)	1.064	0.78	0.713	4.31	39	0	1990.07425	2	987.545105	-5.555976842	44.07720833
														FFDEESYSLLR	10	4	1	N-Term(iTRAQ8plex)	0.829	0.719	0.685	4.22	52	0	1709.87139	2	855.4393311	1.49379035	43.25262167
5454120	TIMM17A	mitochondrial import inner membrane translocase subunit Tim17-A	NP_006326.1	12.87	1	1	4	171	18.0	7.87	1.708	1.496	1.136																
														GKEDPWNSITSGALTGAILAAR	4	1	1	N-Term(iTRAQ8plex); K2(iTRAQ8plex)	1.708	1.496	1.136	4.28	36	1	2836.57889	3	946.1978149	0.380837009	49.81438167
5454158	VARS	valine--tRNA ligase	NP_006286.1	8.07	5	5	12	1264	140.4	7.59	0.829	1.070	1.137																
														VQGSDSDEEVVVATTR	4	2	1	N-Term(iTRAQ8plex)	0.76	0.866	0.866	4.98	72	0	1996.02556	2	998.5164185	5.946313664	25.32136
														HGLEAISIMDSR	4	3	1	N-Term(iTRAQ8plex)	0.755	0.914	0.957	4.59	58	0	1632.86203	3	544.9588623	-3.740448251	25.695085
														SLGNVIDPLDVIYGISLQGLHNQLLNSNLDPSEVEK	2	3	1	N-Term(iTRAQ8plex); K36(iTRAQ8plex)	-	-	-	4.13	29	0	4512.4601	4	1128.870483	3.408875291	63.460515
														GETTLWNPGCDHAGIATQVVVEK	1	2	1	N-Term(iTRAQ8plex); C10(Methylthio); K23(iTRAQ8plex)	-	-	-	3.98	-	0	3079.57705	4	770.6497192	-1.021317807	37.94699
														SSAQDPQAVLGALGR	1	2	1	N-Term(iTRAQ8plex)	0.994	2.089	1.782	2.7	-	0	1773.98491	2	887.4960938	4.954709562	31.806105
5454166	VTI1B	vesicle transport through interaction with t-SNAREs homolog 1B	NP_006361.1	7.76	2	2	3	232	26.7	9.04	0.527	0.686	1.104																
														GLHEDLQGVPER	2	1	1	N-Term(iTRAQ8plex)	0.602	0.601	1.092	3.07	41	0	1653.87778	3	551.9641113	-5.10954662	17.33038333
														LHEIFR	1	1	1	N-Term(iTRAQ8plex)	0.461	0.783	1.115	-	21	0	1118.65482	3	373.5564575	-6.768836352	23.753095
5729770	TPP1	tripeptidyl-peptidase 1 preproprotein	NP_000382.3	4.97	2	2	7	563	61.2	6.48	0.444	0.392	0.719																
														LFGGNFAHQASVAR	4	1	1	N-Term(iTRAQ8plex)	0.406	0.615	0.968	3.65	30	0	1778.95584	3	593.6567993	-2.561390602	25.930245
														LYQQHGAGLFDVTR	3	1	1	N-Term(iTRAQ8plex)	0.444	0.384	0.638	3.65	34	0	1909.01932	3	637.0119019	-1.167605498	29.91951333
5729840	TUBGCP2	gamma-tubulin complex component 2 isoform 2	NP_006650.1	1.44	1	1	1	902	102.5	6.84	1.148	1.237	1.428																
														IHHDVNELLSLLR	1	2	1	N-Term(iTRAQ8plex)	1.148	1.237	1.428	2.74	-	0	1863.06992	3	621.6948242	-2.953133263	53.00634667
5729850	GNAI3	guanine nucleotide-binding protein G(k) subunit alpha	NP_006487.1	16.67	2	4	26	354	40.5	5.69	0.948	1.058	0.969																
														ISQSNYIPTQQDVLR	1	1	1	N-Term(iTRAQ8plex)	1.099	1.207	1.219	3.31	-	0	2066.12383	2	1033.565552	2.625977957	30.92582333
														EYQLNDSASYYLNDLDR	2	1	1	N-Term(iTRAQ8plex)	0.818	0.928	0.77	3.31	52	0	2383.13175	3	795.0487671	-1.600113158	41.11741167
														NVQFVFDAVTDVIIK	20	9	2	N-Term(iTRAQ8plex); K15(iTRAQ8plex)	-	-	-	5.33	59	0	2316.35263	3	772.7890625	4.417699555	59.933485
														TTGIVETHFTFK	3	12	2	N-Term(iTRAQ8plex); K12(iTRAQ8plex)	-	-	-	3.76	-	0	1989.12284	3	663.7124634	-1.872389785	30.03967333
														ILLLGAGESGK	8	4	4	N-Term(iTRAQ8plex); K11(iTRAQ8plex)	-	-	-	5.53	59	0	1666.02866	3	556.0167236	-0.21227131	53.50589833
5729875	PGRMC1	membrane-associated progesterone receptor component 1 isoform 1	NP_006658.1	16.41	3	4	19	195	21.7	4.70	1.263	1.442	1.158																
														FYGPEGPYGVFAGR	10	1	1	N-Term(iTRAQ8plex)	1.292	1.865	1.195	4.58	35	0	1820.9218	2	910.9645386	-3.053822279	39.06366667
														KFYGPEGPYGVFAGR	6	1	1	N-Term(iTRAQ8plex); K1(iTRAQ8plex)	1.306	1.238	1.107	4.31	33	1	2253.2186	3	751.7443848	-4.034341418	31.29986167
														DFTPAELR	1	1	1	N-Term(iTRAQ8plex)	1.137	1.18	0.919	-	23	0	1252.67912	2	626.8432007	-3.837018369	25.29020667
														GLATFCLDK	2	2	2	N-Term(iTRAQ8plex); C6(Methylthio); K9(iTRAQ8plex)	-	-	-	3.75	44	0	1621.87712	2	811.4421997	-8.076756095	66.02182333
5729877	HSPA8	heat shock cognate 71 kDa protein isoform 1	NP_006588.1	60.99	26	31	432	646	70.9	5.52	1.050	2.031	2.538																
														GVPQIEVTFDIDANGILNVSAVDK	4	1	1	N-Term(iTRAQ8plex); K24(iTRAQ8plex)	0.907	3.527	4.929	6.53	96	0	3122.73447	3	1041.583008	4.806328442	66.28720833
														GPAVGIDLGTTYSCVGVFQHGK	12	2	1	N-Term(iTRAQ8plex); C14(Methylthio); K22(iTRAQ8plex)	0.85	1.721	2.137	5.92	63	0	2860.49247	3	954.1671143	-2.748754719	47.43735833
														TVTNAVVTVPAYFNDSQR	6	2	1	N-Term(iTRAQ8plex)	0.733	1.215	1.995	5.66	77	0	2286.20098	2	1143.604126	-0.97802726	38.090715
														LLQDFFNGKELNK	4	4	1	N-Term(iTRAQ8plex); K9(iTRAQ8plex); K13(iTRAQ8plex)	2.179	4.389	4.115	5.58	26	1	2478.43349	4	620.3638306	-6.003354305	46.82712833
														IINEPTAAAIAYGLDKK	13	3	1	N-Term(iTRAQ8plex); K16(iTRAQ8plex); K17(iTRAQ8plex)	1.425	2.553	3.293	5.39	47	1	2700.58486	4	675.9014893	-8.225682928	47.25636667
														RFDDAVVQSDMK	9	2	1	N-Term(iTRAQ8plex); K12(iTRAQ8plex)	0.854	1.164	1.884	5.35	38	1	2019.06168	3	673.6920776	-8.574101534	18.24360333
														QTQTFTTYSDNQPGVLIQVYEGER	6	2	1	N-Term(iTRAQ8plex)	-	2.718	1.539	5.28	65	0	3078.52512	3	1026.846558	-2.306850898	38.86992333
														SFYPEEVSSMVLTK	4	2	1	N-Term(iTRAQ8plex); M10(Oxidation); K14(iTRAQ8plex)	0.792	1.137	1.165	5.27	42	0	2241.18473	3	742.4000244	-5.797857166	42.86117333
														NSLESYAFNMK	18	1	1	N-Term(iTRAQ8plex); K11(iTRAQ8plex)	1.306	2.312	3.204	5.25	76	0	1911.99443	2	956.5003052	-8.445410427	49.34239
														STAGDTHLGGEDFDNR	33	3	1	N-Term(iTRAQ8plex)	1.053	2.074	2.608	5.06	87	0	1995.92191	2	998.4640503	-5.109087465	15.47553667
														NQVAMNPTNTVFDAK	10	2	1	N-Term(iTRAQ8plex); K15(iTRAQ8plex)	1.016	2.42	2.396	5.01	61	0	2258.18755	2	1129.597412	-8.163051501	22.64661
														MVNHFIAEFK	35	2	1	N-Term(iTRAQ8plex); K10(iTRAQ8plex)	1.2	3.129	3.733	5.01	70	0	1844.02226	2	922.5202026	-0.972133599	49.60001333
														SQIHDIVLVGGSTR	42	2	1	N-Term(iTRAQ8plex)	1.05	1.504	1.803	4.99	56	0	1786.00664	2	893.506958	-3.276530023	24.83389
														NSLESYAFNMK	2	1	1	N-Term(iTRAQ8plex); M10(Oxidation); K11(iTRAQ8plex)	0.831	1.329	1.679	4.88	43	0	1927.99436	2	956.5003052	-8.445410427	49.34239

														SFYPEEVSSMVLTK	15	2	1	N-Term(iTRAQ8plex); K14(iTRAQ8plex)	1.374	1.84	3.1	4.8	51	0	2225.18804	3	742.4000244	-5.797857166	42.86117333
														HWPFMVVNDAGRPK	13	2	1	N-Term(iTRAQ8plex); K14(iTRAQ8plex)	1.253	2.569	3.102	4.78	29	0	2262.22817	4	566.3125	-6.401290635	33.67827167
														FEELNADLFR	42	3	1	N-Term(iTRAQ8plex)	0.984	2.126	2.636	4.75	67	0	1557.81369	3	519.9457397	0.747638551	44.320205
														RFDDAVVQSDMK	8	2	1	N-Term(iTRAQ8plex); M11(Oxidation); K12(iTRAQ8plex)	0.866	0.893	1.123	4.43	32	1	2035.05979	3	673.6920776	-8.574101534	18.24360333
														LDKSQIHDIVLVGGSTR	7	2	1	N-Term(iTRAQ8plex); K3(iTRAQ8plex)	1.536	1.57	1.953	4.4	30	1	2446.40934	3	816.1412964	-5.929257067	31.18658167
														NQVAMNPTNTVFDAK	2	2	1	N-Term(iTRAQ8plex); M5(Oxidation); K15(iTRAQ8plex)	0.651	1.613	2.883	4.33	46	0	2274.18784	2	1129.597412	-8.163051501	22.64661
														DAGTIAGLNVLR	8	2	1	N-Term(iTRAQ8plex)	1.331	3.185	3.649	4.25	51	0	1503.8743	3	501.9629517	-3.583789899	34.19468167
														FDDAVVQSDMK	2	2	1	N-Term(iTRAQ8plex); K11(iTRAQ8plex)	1.307	1.12	1.855	4.17	39	0	1862.96262	3	621.6590576	-8.186797887	35.46563167
														LLQDFFNGK	4	4	1	N-Term(iTRAQ8plex); K9(iTRAQ8plex)	1.028	3.083	4.632	4.1	38	0	1689.97373	3	563.9960938	-2.788978267	44.04329833
														MVNHFIAEFK	32	2	1	N-Term(iTRAQ8plex); M1(Oxidation); K10(iTRAQ8plex)	1.094	1.931	2.596	3.97	41	0	1860.01665	2	922.5202026	-0.972133599	49.60001333
														CNEIINWLDK	15	1	1	N-Term(iTRAQ8plex); C1(Methylthio); K10(iTRAQ8plex)	0.799	1.36	1.795	3.95	47	0	1901.99027	2	951.4994507	-8.284764563	77.717735
														VQVEYKGETK	1	3	1	N-Term(iTRAQ8plex); K6(iTRAQ8plex); K10(iTRAQ8plex)	0.883	2.227	2.134	3.89	-	1	2093.2206	4	524.0606079	-7.810710138	19.68643333
														HWPFMVVNDAGRPK	3	2	1	N-Term(iTRAQ8plex); M5(Oxidation); K14(iTRAQ8plex)	1.255	1.377	2.139	3.84	-	0	2278.22475	4	566.3125	-6.401290635	33.67827167
														MKEIAEAYLGK	2	2	1	N-Term(iTRAQ8plex); K2(iTRAQ8plex); K11(iTRAQ8plex)	1.375	2.717	3.477	3.51	30	1	2165.25583	3	722.4234619	-9.651888474	47.824545
														VCNPIITK	3	1	1	N-Term(iTRAQ8plex); C2(Methylthio); K8(iTRAQ8plex)	1.31	3.357	3.655	3.36	41	0	1541.88555	2	771.4464111	-9.622409664	26.32446833
														SINPDEAVAYGAAVQAAILSGDK	2	2	1	N-Term(iTRAQ8plex); K23(iTRAQ8plex)	-	-	-	3.28	38	0	2868.55002	3	956.8548584	-2.223895222	51.69586833
														GTLDPVEK	3	2	1	N-Term(iTRAQ8plex); K8(iTRAQ8plex)	1.427	2.592	3.004	3.23	26	0	1466.85283	2	733.9300537	-9.99452557	14.16397667
														QTQTFTTYSDNQPGVLIQVYEGER	1	2	1	N-Term(iTRAQ8plex); Y20(iTRAQ8plex)	-	-	-	-	34	0	3382.73208	3	1026.846558	-2.306850898	38.86992333
														NSLESYAFNMK	1	1	1	N-Term(iTRAQ8plex); Y6(iTRAQ8plex); K11(iTRAQ8plex)	0.967	2.445	3.073	-	33	0	2216.19365	2	956.5003052	-8.445410427	49.34239
														LYQSAGGMPGGMPGGFPGGGAPPSGGASSGPTIEEVD	1	1	1	N-Term(iTRAQ8plex); Y2(iTRAQ8plex); M8(Oxidation)	-	-	-	-	29	0	3970.93393	3	1324.316162	7.3047748	34.60940667
														IINEPTAAAIAYGLDK	12	5	2	N-Term(iTRAQ8plex); K16(iTRAQ8plex)	-	-	-	5.68	53	0	2268.30278	2	1134.655029	-1.423675126	44.57258167
														VEIIANDQGNR	24	6	3	N-Term(iTRAQ8plex)	-	-	-	4.22	66	0	1532.82414	3	511.616333	0.64225686	26.62443333
														TTPSYVAFTDTER	27	6	2	N-Term(iTRAQ8plex)	-	-	-	4.46	84	0	1791.8958	2	896.4515381	-6.067176522	26.16447333
														MVQEAEK	2	2	2	N-Term(iTRAQ8plex); K7(iTRAQ8plex)	-	-	-	3.4	27	0	1442.79975	3	486.9385986	-4.814673416	11.32049167
														MVQEAEK	2	2	2	N-Term(iTRAQ8plex); M1(Oxidation); K7(iTRAQ8plex)	-	-	-	3.39	35	0	1458.80124	3	486.9385986	-4.814673416	11.32049167
														TTPSYVAFTDTER	1	6	2	N-Term(iTRAQ8plex); Y5(iTRAQ8plex)	-	-	-	-	52	0	2096.106	2	896.4515381	-6.067176522	26.16447333
														ITITNDK	1	5	2	N-Term(iTRAQ8plex); K7(iTRAQ8plex)	-	-	-	-	27	0	1412.84452	3	471.6196899	-8.772887346	14.269095
5802974	PRDX3	thioredoxin-dependent peroxide reductase, mitochondrial isoform a precursor	NP_006784.1	31.25	9	9	51	256	27.7	7.78	1.104	0.959	0.642																
														GTAVVNGEFKDLSLDDFK	1	2	1	N-Term(iTRAQ8plex); K10(iTRAQ8plex); K18(iTRAQ8plex)	2.663	3.2	2.41	6.29	-	1	2867.57607	4	717.6494751	-5.496206199	41.55834
														DYGVLLEGSGLALR	12	2	1	N-Term(iTRAQ8plex)	0.891	0.859	0.823	5.52	57	0	1766.99211	3	589.6680298	-3.347611522	45.97150667
														GLFIIDPNGVIK	15	1	1	N-Term(iTRAQ8plex); K12(iTRAQ8plex)	1.234	1.028	0.644	4.82	62	0	1894.15398	2	947.5806274	-4.362389374	35.49678
														HLSVNDLPVGR	6	1	1	N-Term(iTRAQ8plex)	1.099	0.964	0.713	4.24	60	0	1510.87571	3	504.2967529	7.508386707	23.939845
														GTAVVNGEFK	4	2	1	N-Term(iTRAQ8plex); K10(iTRAQ8plex)	1.148	0.79	0.468	4	53	0	1629.93865	2	815.4729614	-2.095212875	28.99363667
														DLSLDDFK	4	2	1	N-Term(iTRAQ8plex); K8(iTRAQ8plex)	0.92	0.742	0.535	3.52	33	0	1560.87151	2	780.9393921	-0.937771178	42.96414333
														NGGLGHMNIALLSDLTK	2	2	1	N-Term(iTRAQ8plex); K17(iTRAQ8plex)	0.609	0.756	0.576	3.39	30	0	2362.32895	3	788.114502	-3.551025616	44.01980833
														SVEETLR	5	1	1	N-Term(iTRAQ8plex)	1.295	1.078	0.534	3.09	34	0	1137.64048	3	379.8850098	-1.094592845	20.67377833
														KNGGLGHMNIALLSDLTK	1	2	1	N-Term(iTRAQ8plex); K1(iTRAQ8plex); K18(iTRAQ8plex)	1.661	1.992	1.437	3.04	-	1	2794.61294	4	699.4086914	-8.851155981	39.63248833
														DYGVLLEGSGLALR	1	2	1	N-Term(iTRAQ8plex); Y2(iTRAQ8plex)	1.03	1.01	0.72	-	32	0	2071.19748	3	589.6680298	-3.347611522	45.97150667
5803013	ERP29	endoplasmic reticulum resident protein 29 isoform 1 precursor	NP_006808.1	39.46	6	6	16	261	29.0	7.31	0.826	0.713	0.748																
														LAENSASSDDLLVAEVGISDYGDKLNMELSEK	2	1	1	N-Term(iTRAQ8plex); K24(iTRAQ8plex); K32(iTRAQ8plex)	-	-	-	5.82	41	1	4325.22565	5	865.8509521	-5.039521531	57.50724833
														GQGVYLGMPGCLPVYDALAGEFIR	6	2	1	N-Term(iTRAQ8plex); C11(Methylthio)	0.687	0.507	0.663	4.99	75	0	2876.45981	3	959.4914551	5.089479893	68.93854
														SLNILTAFQK	2	2	1	N-Term(iTRAQ8plex); K10(iTRAQ8plex)	0.695	0.657	0.565	4.23	26	0	1743.04959	3	581.6880493	-7.404609037	34.34255833
														DGDFENPVPYTGAVK	2	2	1	N-Term(iTRAQ8plex); K15(iTRAQ8plex)	0.982	0.713	0.765	4.23	46	0	2217.14873	2	1109.078003	-7.257997812	23.38992
														ILDQGEDFPASEMTR	2	2	1	N-Term(iTRAQ8plex)	1.128	0.771	0.868	4.22	31	0	2012.9904	2	1006.99884	0.135491983	38.01369667
														GQGVYLGMPGCLPVYDALAGEFIR	1	2	1	N-Term(iTRAQ8plex); M8(Oxidation); C11(Methylthio)	0.439	-	0.732	3.33	-	0	2892.44675	3	959.4914551	5.089479893	68.93854
														WAEQYLK	1	2	1	N-Term(iTRAQ8plex); K7(iTRAQ8plex)	1.938	1.706	1.138	2.91	-	0	1545.89298	3	515.9691772	2.870307546	32.72195667
5803023	LMAN2	vesicular integral-membrane protein VIP36 precursor	NP_006807.1	38.48	9	9	48	356	40.2	6.95	0.633	0.644	0.621																
														DNFHGLAIFLDTYPNDETTER	15	1	1	N-Term(iTRAQ8plex)	0.664	0.836	0.862	5.29	59	0	2772.34439	3	924.7857056	0.235233996	44.36323833
														EHSLIKPYQGVGSSSMPLWDFQGSTMLTSQYVR	2	1	1	N-Term(iTRAQ8plex); K6(iTRAQ8plex)	-	-	-	5	33	0	4338.2184	4	1085.310059	0.821349842	48.293245
														LFQLMVEHTPDEESIDWTK	1	1	1	N-Term(iTRAQ8plex); M5(Oxidation); K19(iTRAQ8plex)	0.632	0.519	1.203	4.59	-	0	2942.50185	4	736.3809204	-1.636206065	42.55997333
														NLHGDGIALWYTR	10	1	1	N-Term(iTRAQ8plex)	0.863	0.783	0.789	4.48	45	0	1819.96872	3	607.3279419	-3.532403818	28.41044167
														WTELAGCTADFR	11	1	1	N-Term(iTRAQ8plex); C7(Methylthio)	0.628	0.628	0.607	4.18	39	0	1719.81865	2	860.4129639	2.900779319	49.65449667
														LFQLMVEHTPDEESIDWTK	2	1	1	N-Term(iTRAQ8plex); K19(iTRAQ8plex)	0.931	-	-	3.71	-	0	2926.51919	4	736.3809204	-1.636206065	42.55997333
														IEPSVNFLK	2	1	1	N-Term(iTRAQ8plex); K9(iTRAQ8plex)	0.629	0.575	0.512	3.54	31	0	1654.99114	2	827.9992065	-4.655513793	46.66480333
														LVPGPVFGSK	1	1	1	N-Term(iTRAQ8plex); K10(iTRAQ8plex)	0.582	0.453	0.475	-	33	0	1608.99089	2	804.9990845	-1.540701504	24.53478833
														DNVDDPTGNFR	2	1	1	N-Term(iTRAQ8plex)	0.532	0.588	0.638	-	32	0	1553.74431	2	777.3757935	-3.527611269	15.050065
														NCIDITGVR	2	1	1	N-Term(iTRAQ8plex); C2(Methylthio)	0.614	0.517	0.517	-	29	0	1340.6917	2	670.8494873	-3.822139447	22.468465
5803036	HNRNPA0	heterogeneous nuclear ribonucleoprotein A0	NP_006796.1	27.21	4	4	10	305	30.8	9.29	0.885	0.844	0.735																
														CFGFVTYSNVEEADAAMAASPHAVDGNTVELK	2	1	1	N-Term(iTRAQ8plex); C1(Methylthio); K32(iTRAQ8plex)	-	-	-	6.26	49	0	3997.93813	4	1000.23999	3.741618057	53.90617333
														GDVAEGDLIEHFSQFGTVEK	2	1	1	N-Term(iTRAQ8plex); K20(iTRAQ8plex)	0.963	1.012	0.741	5.82	44	0	2786.43711	3	929.4838867	-3.113077172	42.695955
														GFGFVYFQNHDAADK	4	1	1	N-Term(iTRAQ8plex); K15(iTRAQ8plex)	0.885	0.858	0.742	5.12	45	0	2324.1846	3	775.3997192	-3.195458374	34.21464
														LFIGGLNVQTSESGLR	2	1	1	N-Term(iTRAQ8plex)	0.48	0.389	0.34	4.2	54	0	1995.11504	2	998.0611572	-1.334994135	44.474865
														IFVGGIK	9	9	4	N-Term(iTRAQ8plex); K7(iTRAQ8plex)	-	-	-	3.12	27	0	1341.87183	3	447.9621277	0.282073617	31.44851833
														KLFVGGIK	9	9	4	N-Term(iTRAQ8plex); K1(iTRAQ8plex); K8(iTRAQ8plex)	-	-	-	3.5	30	1	1774.17019	2	887.5895996	0.079642084	21.37640667
5803048	KDELR1	ER lumen protein-retaining receptor 1	NP_006792.1	17.45	2	3	12	212	24.5	8.62	0.423	0.526	0.772																
														TGEAETITSHYLFALGVYR	2	1	1	N-Term(iTRAQ8plex)	0.325	0.633	0.772	5.48	54	0	2432.26975	3	811.4281006	-2.722885564	53.43322667
														TLYLFNWIWR	6	1	1	N-Term(iTRAQ8plex)	0.454	0.52	0.75	3.99	48	0	1715.95671	3	572.6566162	-1.297188332	67.51441333
														TLYLFNWIWR	1	1	1	N-Term(iTRAQ8plex); Y3(iTRAQ8plex)	-	-	-	-	32	0	2020.17019	3	572.6566162	-1.297188332	67.51441333
														SCAGISGK	3	3	2	N-Term(iTRAQ8plex); C2(Methylthio); K8(iTRAQ8plex)	-	-	-	3.35	25	0	1376.7515	3	459.5886841	2.082217521	19.46175667
5803050	KDELR2	ER lumen protein-retaining receptor 2 isoform 1	NP_006845.1	16.51	2	3	15	212	24.4	8.72	1.180	0.485	0.605																
														SQILFALVFTTR	6	2	1	N-Term(iTRAQ8plex)	1.214	0.473	0.691	4.57	47	0	1700.01384	2	850.5105591	5.270688887	62.92644167
														ALYLVNWIWR	6	1	1	N-Term(iTRAQ8plex)	1.18	0.485	0.617	4	40	0	1637.94559	3	546.6533813	-0.836723172	59.36435833
														YLDLFTSFISLYNTSMK	4	2	1	N-Term(iTRAQ8plex); K17(iTRAQ8plex)	-	-	-	3.52	30	0	2651.42978	3	889.8190918	8.504715185	65.44502167
														YLDLFTSFISLYNTSMK	2	2	1	N-Term(iTRAQ8plex); M16(Oxidation); K17(iTRAQ8plex)	-	-	0.385	3.18	34	0	2667.44272	3	889.8190918	8.504715185	65.44502167
														SCAGISGK	3	3	2	N-Term(iTRAQ8plex); C2(Methylthio); K8(iTRAQ8plex)	-	-	-	3.35	25	0	1376.7515	3	459.5886841	2.082217521	19.46175667
5803133	RAB32	ras-related protein Rab-32	NP_006825.1	9.33	1	2	6	225	25.0	6.54	0.699	0.235	0.480																
														DNINIEEAAR	2	1	1	N-Term(iTRAQ8plex)	0.699	0.235	0.48	4.08	31	0	1448.76909	2	724.8881836	3.022750505	23.99478167
														LQLWDIAGQER	4	4	2	N-Term(iTRAQ8plex)	-	-	-	4.27	45	0	1632.89592	2	816.9515991	-3.202701252	33.955305
5803137	RBM3	RNA-binding protein 3	NP_006734.1	31.85	2	2	9	157	17.2	8.91	1.078	1.942	2.109																
														GFGFITFTNPEHASVAMR	6	1	1	N-Term(iTRAQ8plex)	1.078	1.834	1.706	6.22	53	0	2286.15726	3	762.723938	-3.101529925	41.30963833
														LFVGGLNFNTDEQALEDHFSSFGPISEVVVVK	3	1	1	N-Term(iTRAQ8plex); K32(iTRAQ8plex)	0.76	2.007	2.176	3.71	-	0	4103.18442	3	1368.399658	6.265879555	65.11474667
5803149	TMED2	transmembrane emp24 domain-containing protein 2 isoform 1 precursor	NP_006806.1	24.88	4	4	13	201	22.7	5.17	0.830	0.799	0.566																
														IVMFTIDIGEAPK	6	2	1	N-Term(iTRAQ8plex); K13(iTRAQ8plex)	0.898	0.799	0.672	5.67	69	0	2042.17266	3	681.3970947	-2.41916884	43.12556833
														LEEMINELAVAMTAVK	5	2	1	N-Term(iTRAQ8plex); K16(iTRAQ8plex)	0.622	0.366	0.479	4.61	41	0	2370.32096	3	790.7785034	-1.010114722	69.036935
														YTFAAHMDGTYK	1	2	1	N-Term(iTRAQ8plex); K12(iTRAQ8plex)	0.863	1.216	0.72	2.84	-	0	2013.02579	3	671.6801147	-5.082056953	25.717395
														AINDNTNSR	1	2	1	N-Term(iTRAQ8plex)	0.815	0.799	0.566	-	32	0	1308.67912	2	654.8432007	-1.403363726	9.161868333
5803165	SEC61B	protein transport protein Sec61 subunit beta	NP_006799.1	15.63	1	1	1	96	10.0	11.56	0.962	0.487	0.917																
														PGPTPSGTNVGSSGR	1	1	1	N-Term(iTRAQ8plex)	0.962	0.487	0.917	-	30	0	1674.86899	3	558.9611816	-1.384960935	13.86663333
5803167	SF3A3	splicing factor 3A subunit 3 isoform 1	NP_006793.1	9.18	3	3	8	501	58.8	5.38	1.230	1.009	0.921																
														CLGIPNTAHFANVTQIEDAVSLWAK	6	2	1	N-Term(iTRAQ8plex); C1(Methylthio); K25(iTRAQ8plex)	-	1.38	1.218	5.27	35	0	3352.76992	3	1118.262817	2.859303588	63.08781667
														SLESLDTSLFAK	1	2	1	N-Term(iTRAQ8plex); K12(iTRAQ8plex)	1.167	0.916	0.84	3.72	-	0	1919.0817	3	640.3654175	-6.723063744	32.89712167
														YMEVSGNLR	1	1	1	N-Term(iTRAQ8plex)	1.296	1.009	0.921	2.83	-	0	1372.71428	2	686.8607788	-3.919462984	32.16658167
5803177	SNTB2	beta-2-syntrophin	NP_006741.1	1.85	1	1	2	540	57.9	8.82	0.561	0.525	0.463																
														LIELHSPDSR	2	1	1	N-Term(iTRAQ8plex)	0.561	0.525	0.463	2.96	41	0	1470.81488	3	490.9431458	-4.716633083	21.98209333
5803181	STIP1	stress-induced-phosphoprotein 1 isoform b	NP_006810.1	14.92	6	7	28	543	62.6	6.80	1.845	2.451	2.800																
														ALSVGNIDDALQCYSEAIK	4	3	1	N-Term(iTRAQ8plex); C13(Methylthio); K19(iTRAQ8plex)	2.55	3.074	3.597	6.2	99	0	2664.38138	3	888.7959595	-3.767164284	51.94191667
														DCEECIQLEPTFIK	9	3	1	N-Term(iTRAQ8plex); C2(Methylthio); C5(Methylthio); K14(iTRAQ8plex)	1.469	1.918	2.556	5.34	56	0	2368.16899	2	1184.588135	7.275499047	48.43304
														LAYINPDLALEEK	8	3	1	N-Term(iTRAQ8plex); K13(iTRAQ8plex)	1.84	1.995	2.034	4.75	57	0	2097.18682	2	1049.097046	-8.776577743	34.61893667
														LMDVGLIAIR	2	3	1	N-Term(iTRAQ8plex)	2.373	4.369	5.441	3.49	44	0	1404.85527	2	702.9312744	0.150128009	45.08492
														AYEDGCK	2	3	1	N-Term(iTRAQ8plex); C6(Methylthio); K7(iTRAQ8plex)	1.205	2.444	2.799	3.09	23	0	1439.69776	3	480.5707703	-9.82481143	15.01509833
														KAAALEFLNR	2	2	1	N-Term(iTRAQ8plex); K1(iTRAQ8plex)	2.685	4.125	3.865	2.75	26	1	1741.0499	3	581.0214844	-4.710620299	31.01467
														MEQVNELK	1	2	1	N-Term(iTRAQ8plex); K8(iTRAQ8plex)	-	-	-	-	33	0	1598.90622	3	533.6402588	1.87116489	26.02221833
5803187	TALDO1	transaldolase	NP_006746.1	15.13	5	5	28	337	37.5	6.81	1.047	1.931	3.126																
														ALAGCDFLTISPK	19	1	1	N-Term(iTRAQ8plex); C5(Methylthio); K13(iTRAQ8plex)	0.891	1.902	2.557	4.72	59	0	1990.08391	2	995.5455933	-6.181848163	40.737045
														WLHNEDQMAVEK	3	1	1	N-Term(iTRAQ8plex); K12(iTRAQ8plex)	1.271	1.441	2.14	4.46	50	0	2108.08976	3	703.368103	-7.483299952	23.01543167
														MESALDQLK	2	1	1	N-Term(iTRAQ8plex); K9(iTRAQ8plex)	1.251	3.651	5.739	3.16	49	0	1642.91533	2	821.9613037	-8.594781399	45.81446833
														TIVMGASFR	2	1	1	N-Term(iTRAQ8plex)	1.123	4.375	8.578	3.05	29	0	1285.72173	2	643.364502	-1.792000322	29.07949667
														VSTEVDAR	2	1	1	N-Term(iTRAQ8plex)	0.902	4.082	8.609	-	33	0	1180.64311	2	590.8251953	-3.749901175	16.53049667
5803221	WDR3	WD repeat-containing protein 3	NP_006775.1	6.79	6	6	11	943	106.0	6.64	1.813	1.046	0.864																
														FWDFELVKDENSTQK	2	1	1	N-Term(iTRAQ8plex); K8(iTRAQ8plex); K15(iTRAQ8plex)	2.247	0.951	0.634	5.68	27	1	2798.49404	4	700.3789673	-6.71505428	40.63254667
														VWDIAYLQEIEDPEEPDPK	2	1	1	N-Term(iTRAQ8plex); K19(iTRAQ8plex)	1.762	1.329	1.265	5.13	58	0	2894.47739	3	965.4973145	-5.068683789	44.10371833
														VWDIAYLQEIEDPEEPDPKK	2	1	1	N-Term(iTRAQ8plex); K19(iTRAQ8plex); K20(iTRAQ8plex)	2.664	1.371	1.4	5.1	30	1	3326.78603	4	832.4519653	-1.911710526	47.29493167
														WWDLDTQHCFK	2	1	1	N-Term(iTRAQ8plex); C9(Methylthio); K11(iTRAQ8plex)	1.583	0.973	0.796	3.77	23	0	2133.04007	3	711.6848755	-4.954939801	44.04310833
														IMEAIELYR	1	1	1	N-Term(iTRAQ8plex)	1.866	1.124	0.937	3.28	-	0	1441.80962	2	721.4084473	4.817980934	46.25370333
														GNIVFVTLR	2	1	1	N-Term(iTRAQ8plex)	1.303	0.666	0.516	2.89	37	0	1322.80339	2	661.9053345	-4.844994143	27.27360667

5803227	YWHAQ	14-3-3 protein theta	NP_006817.1	32.65	3	7	84	245	27.7	4.78	0.936	1.874	2.230																
														AVTEQGAELSNEER	6	1	1	N-Term(iTRAQ8plex)	0.977	1.956	2.753	5.38	63	0	1836.93633	2	918.9718018	6.633651612	21.70452333
														TAFDEAIAELDTLNEDSYK	4	1	1	N-Term(iTRAQ8plex); K19(iTRAQ8plex)	0.363	1.874	1.683	5.34	59	0	2753.3906	3	918.4683838	-2.619745566	48.095115
														YLAEVACGDDRK	1	1	1	N-Term(iTRAQ8plex); C7(Methylthio); K12(iTRAQ8plex)	0.503	1.045	1.367	2.73	-	1	1994.01139	3	665.34198	-9.125127728	27.917855
														DSTLIMQLLR	24	9	7	N-Term(iTRAQ8plex)	-	-	-	4.73	54	0	1493.87629	3	498.6302795	6.656557269	46.36715833
														NLLSVAYK	16	10	7	N-Term(iTRAQ8plex); K8(iTRAQ8plex)	-	-	-	4.01	39	0	1515.92546	2	758.4644165	-9.192370231	25.08285833
														DSTLIMQLLR	2	9	7	N-Term(iTRAQ8plex); M6(Oxidation)	-	-	-	3.14	48	0	1509.85791	3	498.6302795	6.656557269	46.36715833
														EMQPTHPIR	13	3	3	N-Term(iTRAQ8plex)	-	-	-	3.75	37	0	1412.75782	3	476.9222107	-3.525783092	8.55931
														EMQPTHPIR	16	3	3	N-Term(iTRAQ8plex); M2(Oxidation)	-	-	-	3.14	48	0	1428.75373	3	476.9222107	-3.525783092	8.55931
														VISSIEQK	2	5	5	N-Term(iTRAQ8plex); K8(iTRAQ8plex)	-	-	-	3.44	29	0	1511.91423	2	756.4607544	-7.341111487	22.45532333
5901896	ATP5E	ATP synthase subunit epsilon, mitochondrial	NP_008817.1	13.73	1	1	2	51	5.8	9.92	0.987	0.782	0.673																
														YSQICAK	2	1	1	N-Term(iTRAQ8plex); C5(Methylthio); K7(iTRAQ8plex)	0.987	0.782	0.673	2.97	-	0	1466.79956	3	489.6047058	2.728308447	26.62390333
5901922	CDC37	hsp90 co-chaperone Cdc37	NP_008996.1	3.17	1	1	3	378	44.4	5.25	1.235	2.678	2.163																
														CIDSGLWVPNSK	3	2	1	N-Term(iTRAQ8plex); C1(Methylthio); K12(iTRAQ8plex)	1.235	2.678	2.163	3.25	30	0	1973.0302	2	987.0187378	-7.249424804	61.31728833
5901926	NUDT21	cleavage and polyadenylation specificity factor subunit 5	NP_008937.1	28.19	4	4	10	227	26.2	8.82	1.005	0.885	0.604																
														LPHVLLLQLGTTFFK	4	1	1	N-Term(iTRAQ8plex); K15(iTRAQ8plex)	0.761	0.31	0.335	4.93	40	0	2335.42875	3	779.1477661	-3.205497137	62.28302167
														LVAAPLFELYDNAPGYGPI ISSLPQLLSR	2	1	1	N-Term(iTRAQ8plex)	-	-	-	4.7	45	0	3418.8834	3	1140.299316	-2.777319419	85.75467333
														GVTQFGNK	2	1	1	N-Term(iTRAQ8plex); K8(iTRAQ8plex)	1.189	1.102	0.934	3.09	29	0	1458.84034	3	486.9516296	-8.339249805	14.82987
														TVEGVLIVHEHR	2	1	1	N-Term(iTRAQ8plex)	1.1	0.885	0.604	3.01	45	0	1692.96421	3	564.9929199	-3.357888107	18.00914833
5902076	SRSF1	serine/arginine-rich splicing factor 1 isoform 1	NP_008855.1	42.74	9	10	47	248	27.7	10.36	0.784	0.825	0.767																
														EAGDVCYADVYR	10	2	1	N-Term(iTRAQ8plex); C6(Methylthio)	0.817	0.809	0.768	5.02	64	0	1710.773	3	570.930542	0.087928793	31.21555833
														VVVSGLPPSGSWQDLK	3	2	1	N-Term(iTRAQ8plex); K16(iTRAQ8plex)	0.686	0.696	0.49	4.93	31	0	2277.31839	3	759.7776489	5.287897985	36.98815833
														GGPPFAFVEFEDPRDAED AVYGR	18	2	1	N-Term(iTRAQ8plex)	0.842	0.939	0.743	4.54	32	1	2845.37856	4	712.0986938	-0.226961241	46.237445
														IYVGNLPPDIR	4	2	1	N-Term(iTRAQ8plex)	0.678	0.912	0.972	3.83	31	0	1560.90215	2	780.9547119	-1.930993484	34.98319667
														DGTGVVEFVR	3	2	1	N-Term(iTRAQ8plex)	0.738	0.784	0.945	3.19	25	0	1382.75444	2	691.8808594	-2.696914135	26.55281833
														GGPPFAFVEFEDPR	1	2	1	N-Term(iTRAQ8plex)	0.978	0.771	0.804	2.9	-	0	1868.96135	2	934.984314	6.864780633	42.47946167
														DGYDYDGYR	3	2	1	N-Term(iTRAQ8plex)	0.926	0.813	0.725	2.82	22	0	1427.63213	2	714.3197021	-4.163273584	16.108815
														DIEDVFYK	1	2	1	N-Term(iTRAQ8plex); K8(iTRAQ8plex)	0.615	0.656	0.691	2.72	-	0	1636.90335	3	546.3059692	-0.553994317	45.402435
														SHEGETAYIR	1	1	1	N-Term(iTRAQ8plex)	0.841	1.099	0.992	-	34	0	1466.74866	3	489.587738	-3.722818242	15.294685
														LRVEFPR	3	3	2	N-Term(iTRAQ8plex)	-	-	-	-	28	1	1220.73337	3	407.5826416	-6.83994188	22.63803833
5902102	SNRPD1	small nuclear ribonucleoprotein Sm D1 isoform 1	NP_008869.1	36.97	3	3	36	119	13.3	11.56	0.923	0.939	0.951																
														LSHETVTIELK	3	2	1	N-Term(iTRAQ8plex); K11(iTRAQ8plex)	0.716	0.986	0.908	5.01	58	0	1878.10491	2	939.5560913	-5.726678408	27.14542167
														YFILPDSLPLDTLLVDVEPK	13	1	1	N-Term(iTRAQ8plex); K20(iTRAQ8plex)	0.993	0.732	0.876	4.78	47	0	2895.65696	4	724.6691284	-1.145684996	62.41326167
														NREPVQLETLSIR	20	1	1	N-Term(iTRAQ8plex)	0.91	1.035	0.973	4.23	46	1	1859.06119	2	930.0326538	-3.889229218	27.05533333
6005717	ATP5I	ATP synthase subunit e, mitochondrial	NP_009031.1	52.17	3	3	14	69	7.9	9.35	0.740	0.741	0.747																
														YSALFLGVAYGATR	9	1	1	N-Term(iTRAQ8plex)	0.817	0.779	0.827	4.51	49	0	1792.99126	2	896.9992676	0.729081218	51.792185
														ELAEDDSILK	2	1	1	N-Term(iTRAQ8plex); K10(iTRAQ8plex)	0.622	0.368	0.417	4.3	59	0	1740.97869	2	870.992981	-3.047714474	21.55092667
														MVPPVQVSPLIK	1	1	1	N-Term(iTRAQ8plex); K12(iTRAQ8plex)	0.41	0.432	0.424	2.81	-	0	1916.19231	2	958.5997925	3.108790757	35.01794
														YSALFLGVAYGATR	2	1	1	N-Term(iTRAQ8plex); Y1(iTRAQ8plex)	0.742	0.718	0.755	-	31	0	2097.1893	2	896.9992676	0.729081218	51.792185
6005719	C14orf1	probable ergosterol biosynthetic protein 28	NP_009107.1	6.43	1	1	1	140	15.9	9.83	0.953	0.702	0.528																
														YLEVEPVSR	1	1	1	N-Term(iTRAQ8plex)	0.953	0.702	0.528	2.87	-	0	1395.77507	2	698.3911743	-2.492727702	26.07832167
6005731	CHP1	calcineurin B homologous protein 1	NP_009167.1	14.87	3	3	6	195	22.4	5.10	0.759	0.542	0.505																
														ISRDELLQVLR	2	1	1	N-Term(iTRAQ8plex)	0.865	0.679	0.766	2.73	25	1	1645.98319	3	549.333252	-3.097652772	34.52118333
														LHFAFR	3	1	1	N-Term(iTRAQ8plex)	0.431	0.436	0.467	2.71	28	0	1094.63156	3	365.5497437	-6.014420247	24.16023333
														IPELAINPLGDR	1	2	1	N-Term(iTRAQ8plex)	0.985	0.542	0.611	-	48	0	1611.93096	2	806.4691162	-3.880748181	44.46006
6005830	PKP3	plakophilin-3 isoform PKP3a	NP_009114.1	3.01	2	2	4	797	87.0	9.32	1.199	0.952	0.507																
														HNGAAEPEPEAETAR	2	2	1	N-Term(iTRAQ8plex)	1.221	0.858	0.341	3.63	29	0	1882.91617	3	628.3102417	-1.890393421	9.601551667
														DLLYFDGLR	2	2	1	N-Term(iTRAQ8plex)	1.178	1.056	0.753	3.28	24	0	1415.78179	2	708.3945313	-1.310526819	43.65751667
6005846	TWF2	twinfilin-2	NP_009215.1	4.87	1	1	1	349	39.5	6.84	-	-	1.435																
														AVLPLLDAQQPCYLLYR	1	1	1	N-Term(iTRAQ8plex); C12(Methylthio)	-	-	1.435	-	37	0	2326.25974	3	776.0914307	-0.341130356	63.831755
														IDLER	1	2	3	N-Term(iTRAQ8plex)	-	-	-	-	26	0	949.56273	2	475.2850037	0.757616317	23.49304
6005860	RPL35	60S ribosomal protein L35	NP_009140.1	10.57	1	1	6	123	14.5	11.05	2.134	3.501	2.881																
														QLDDLKVELSQLR	6	1	1	N-Term(iTRAQ8plex); K6(iTRAQ8plex)	2.134	3.501	2.881	4.52	46	1	2165.27324	2	1083.140259	-0.831461748	44.7147
6005862	MRPL3	39S ribosomal protein L3, mitochondrial	NP_009139.1	3.45	1	1	1	348	38.6	9.48	0.981	0.583	0.582																
														RPGAVATGDIGR	1	1	1	N-Term(iTRAQ8plex)	0.981	0.583	0.582	-	34	0	1473.84162	3	491.9520569	-1.603900769	14.233015
6005884	SSR3	translocon-associated protein subunit gamma isoform 2	NP_009038.1	7.57	1	1	4	185	21.1	9.61	1.007	0.783	0.843																
														QQSEEDLLLQDFSR	4	2	1	N-Term(iTRAQ8plex)	1.007	0.783	0.843	6.86	70	0	2012.03874	2	1006.52301	7.40174008	38.89213
6005942	VCP	transitional endoplasmic reticulum ATPase	NP_009057.1	50.87	28	28	134	806	89.3	5.26	1.032	1.015	1.386																
														AVANETGAFFFLINGPEIM SK	6	1	1	N-Term(iTRAQ8plex); K21(iTRAQ8plex)	1.351	1.148	1.494	7.07	77	0	2864.56704	4	716.8972168	6.729117262	60.29520167
														LADDVDLEQVANETHGH VGADLAALCSEAALQAIR	7	1	1	N-Term(iTRAQ8plex); C26(Methylthio)	2.068	0.918	1.888	6.06	56	0	3965.9758	4	992.2476196	1.163986918	65.422105
														VINQILTEMDGMSTK	4	1	1	N-Term(iTRAQ8plex); K15(iTRAQ8plex)	1.097	1.026	1.317	5.9	67	0	2288.23441	3	763.4163208	-4.663922373	51.46416833
														ELQELVQYPVEHPDK	4	1	1	N-Term(iTRAQ8plex); K15(iTRAQ8plex)	1.337	1.041	1.242	5.26	39	0	2432.319	3	811.444519	-3.777227033	34.43424167
														EAVCIVLSDDTCSDEK	10	1	1	N-Term(iTRAQ8plex); C4(Methylthio); C12(Methylthio); K16(iTRAQ8plex)	1.037	1.307	1.591	5.23	61	0	2427.13675	3	809.7149658	-2.842488076	44.456175
														AIANECQANFISIK	4	1	1	N-Term(iTRAQ8plex); C6(Methylthio); K14(iTRAQ8plex)	0.964	1.107	1.442	4.97	67	0	2176.15752	2	1088.582397	-6.426004555	34.50021
														KMDLIDLEDETIDAEVMN SLAVTMDDFR	2	1	1	N-Term(iTRAQ8plex); K1(iTRAQ8plex)	-	-	-	4.94	65	1	3837.90342	3	1279.972656	-0.626852669	69.03140667
														LDQLIYIPLPDEK	6	1	1	N-Term(iTRAQ8plex); K13(iTRAQ8plex)	1.018	0.864	1.295	4.85	59	0	2165.26763	2	1083.137451	-0.090324285	48.68557667
														NAPAIIFIDELDAIAPK	18	1	1	N-Term(iTRAQ8plex); K17(iTRAQ8plex)	1.019	0.86	1.534	4.73	61	0	2419.40654	2	1210.206909	0.32670796	56.29496167
														ELQELVQYPVEHPDKFLK	1	1	1	N-Term(iTRAQ8plex); K15(iTRAQ8plex); K18(iTRAQ8plex)	0.9	1.079	0.936	4.64	-	1	3124.75722	4	781.9447632	-7.612894036	43.20116833
														ETVVEVPQVTWEDIGGLE DVKR	9	1	1	N-Term(iTRAQ8plex); K21(iTRAQ8plex)	0.841	1.618	2.262	4.37	49	1	3106.6915	3	1036.235352	1.075142961	48.17083
														QAAPCVLFFDELDSIAK	15	1	1	N-Term(iTRAQ8plex); C5(Methylthio); K17(iTRAQ8plex)	1.082	0.67	1.464	4.26	51	0	2521.33371	3	841.112854	-2.040014795	65.29795
														LGDVISIQPCPDVK	6	1	1	N-Term(iTRAQ8plex); C10(Methylthio); K14(iTRAQ8plex)	0.983	0.892	1.428	4.1	43	0	2138.16501	3	713.3931885	-7.483108283	37.26163167
														WALSQSNPSALR	3	1	1	N-Term(iTRAQ8plex)	0.995	0.941	1.147	4.08	34	0	1633.90032	2	817.4473267	-5.517271151	22.27286667
														EMVELPLRHPALFK	1	1	1	N-Term(iTRAQ8plex); K14(iTRAQ8plex)	1.908	4.037	2.045	3.98	-	1	2288.32461	4	572.8366089	-7.151797033	33.15337667
														AHVIVMAATNRPNSIDPA LR	2	1	1	N-Term(iTRAQ8plex)	0.773	-	2.399	3.95	-	0	2450.35463	4	613.3441162	-2.369590168	29.66579333
														LIVDEAINEDNSVVSLSQPK	2	1	1	N-Term(iTRAQ8plex); K20(iTRAQ8plex)	-	-	-	3.9	31	0	2778.52456	4	695.3865967	-3.61143515	41.27481833
														EVDIGIPDATGR	4	1	1	N-Term(iTRAQ8plex)	1.022	0.858	1.198	3.78	43	0	1546.83415	2	773.9207153	-2.416067636	28.44699
														KYEMFAQTLQQSR	4	1	1	N-Term(iTRAQ8plex); K1(iTRAQ8plex)	0.943	1.167	1.76	3.69	40	1	2238.19992	3	746.7390747	-6.013945511	31.51929167
														IVSQLLTLMDGLK	3	1	1	N-Term(iTRAQ8plex); K13(iTRAQ8plex)	1.193	0.758	1.386	3.64	43	0	2039.24797	2	1020.127625	4.149129263	66.76754333
														MTNGFSGADLTEICQR	2	1	1	N-Term(iTRAQ8plex); C14(Methylthio)	1.297	1.125	1.236	3.58	62	0	2092.99321	2	1047.000244	7.840140694	43.214015
														DVDLEFLAK	4	1	1	N-Term(iTRAQ8plex); K9(iTRAQ8plex)	1.066	0.996	1.412	3.49	47	0	1657.94646	2	829.4768677	-9.45300034	54.58588
														LEILQIHTK	2	1	1	N-Term(iTRAQ8plex); K9(iTRAQ8plex)	1.002	0.955	0.936	3.46	-	0	1703.05568	4	426.5193787	-6.985403747	31.14997833
														GPELLTMWFGESEANVR	2	1	1	N-Term(iTRAQ8plex)	-	2.083	0.631	3.45	48	0	2240.13645	2	1128.565186	-1.866842399	58.79115167
														GVLFYGPPGCGK	2	1	1	N-Term(iTRAQ8plex); C10(Methylthio); K12(iTRAQ8plex)	1.062	0.912	1.307	3.45	39	0	1848.98124	3	616.9985962	-8.035222585	55.229215
														GPELLTMWFGESEANVR	3	1	1	N-Term(iTRAQ8plex); M7(Oxidation)	0.643	1.039	1.327	3.45	49	0	2256.12309	2	1128.565186	-1.866842399	58.79115167
														KMDLIDLEDETIDAEVMN SLAVTMDDFR	2	1	1	N-Term(iTRAQ8plex); K1(iTRAQ8plex); M2(Oxidation)	-	-	-	3.43	33	1	3853.90757	3	1279.972656	-0.626852669	69.03140667
														FDREVDIGIPDATGR	1	1	1	N-Term(iTRAQ8plex)	0.75	0.848	1.338	3.41	-	1	1965.03147	3	655.6820068	-1.482949375	28.84659
														KYEMFAQTLQQSR	1	1	1	N-Term(iTRAQ8plex); K1(iTRAQ8plex); M4(Oxidation)	0.773	0.596	0.795	3.39	-	1	2254.20829	3	746.7390747	-6.013945511	31.51929167
														LAGESESNLR	2	1	1	N-Term(iTRAQ8plex)	0.858	0.733	1.041	3.32	28	0	1379.7415	2	690.3743896	-1.260107869	21.84387833
														AHVIVMAATNRPNSIDPA LR	1	1	1	N-Term(iTRAQ8plex); M6(Oxidation)	-	0.368	0.741	3.19	-	0	2466.33681	4	613.3441162	-2.369590168	29.66579333
														EMVELPLR	1	1	1	N-Term(iTRAQ8plex)	0.936	0.769	1.19	2.79	-	0	1290.73418	2	645.8707275	-4.007674052	32.34541
6466450	COMT	catechol O-methyltransferase isoform S-COMT	NP_009294.1	37.10	5	5	22	221	24.4	5.33	0.850	0.554	0.474																
														GTVLLADNVICPGAPDFL AHVR	8	3	1	N-Term(iTRAQ8plex); C11(Methylthio)	0.563	0.971	0.61	6.14	54	0	2628.39713	3	876.807373	4.98917702	49.47932333
														KGTVLLADNVICPGAPDF LAHVR	4	3	1	N-Term(iTRAQ8plex); K1(iTRAQ8plex); C12(Methylthio)	1.239	-	-	5.73	33	1	3060.69863	4	765.9301147	1.256838664	49.43401667
														YLPDTLLLEECGLLR	6	3	1	N-Term(iTRAQ8plex); C11(Methylthio)	0.85	0.549	0.436	4.41	60	0	2098.12735	3	700.0473022	2.054779122	66.966515
														ILNHVLQHAEPGNAQSVL EAIDTYCEQK	1	3	1	N-Term(iTRAQ8plex); C25(Methylthio); K28(iTRAQ8plex)	-	-	-	4.08	-	0	3774.94306	5	755.7944336	0.696476399	63.38914333
														LITIEINPDCAAITQR	2	3	1	N-Term(iTRAQ8plex); C10(Methylthio)	0.915	2.709	0.792	4.07	45	0	2121.13108	3	707.71521	-1.855456851	51.796355
														YLPDTLLLEECGLLR	1	3	1	N-Term(iTRAQ8plex); Y1(iTRAQ8plex); C11(Methylthio)	0.557	0.542	0.472	-	34	0	2402.32614	3	700.0473022	2.054779122	66.966515
6679189	TMED3	transmembrane emp24 domain-containing protein 3 isoform a precursor	NP_031390.1	8.76	1	1	2	217	24.8	5.60	1.553	0.911	-																
														VTALTQMESACVTIHEAL K	2	1	1	N-Term(iTRAQ8plex); C11(Methylthio); K19(iTRAQ8plex)	1.553	0.911	-	4.34	42	0	2699.4396	4	675.6153564	0.179148758	63.09876833
6681764	NDUFA9	NADH dehydrogenase [ubiquinone] 1 alpha subcomplex subunit 9, mitochondrial precursor	NP_004993.1	18.83	5	5	24	377	42.5	9.80	0.994	0.667	0.497																
														LPHLPGLEDLGIQATPLEL K	10	1	1	N-Term(iTRAQ8plex); K20(iTRAQ8plex)	1.432	0.569	0.942	5.91	39	0	2762.62351	3	921.5513306	4.244907626	53.39956
														FGPIPLGSLGWK	6	1	1	N-Term(iTRAQ8plex); K12(iTRAQ8plex)	1.032	0.668	0.482	4.81	47	0	1880.11797	2	940.5611572	-5.544058031	39.86610667
														VFEISPFEPWITR	6	1	1	N-Term(iTRAQ8plex)	0.92	0.634	0.462	4.63	55	0	1925.05681	3	642.3534546	-0.873111933	60.76815667
														DAFPEAIIVKPSDIFGREDR	1	1	1	N-Term(iTRAQ8plex); K10(iTRAQ8plex)	-	-	-	3.82	-	1	2883.57656	4	721.6495972	-2.085087045	41.99404
														AIEVLR	1	1	1	N-Term(iTRAQ8plex)	0.994	0.712	0.577	-	24	0	1004.63756	2	502.8224182	-3.027454219	22.85486667
6912238	PRDX5	peroxiredoxin-5, mitochondrial isoform a precursor	NP_036226.1	44.86	8	8	36	214	22.0	8.57	0.646	0.748	0.841																
														THLPGFVEQAEALK	11	2	1	N-Term(iTRAQ8plex); K14(iTRAQ8plex)	0.599	1.179	1.005	6.1	50	0	2148.22409	3	716.7462158	-1.52083252	34.36365667
														GVLFGVPGAFTPGCSK	6	2	1	N-Term(iTRAQ8plex); C14(Methylthio); K16(iTRAQ8plex)	0.627	0.713	0.739	5.56	54	0	2191.19034	2	1096.098022	1.061525099	42.95429833
														ETDLLLDDSLVSIFGNR	2	4	1	N-Term(iTRAQ8plex)	-	-	-	5.17	67	0	2211.18601	3	737.7335205	2.219159048	63.50818333
														FSMVVQDGIVK	8	4	1	N-Term(iTRAQ8plex); K11(iTRAQ8plex)	0.716	0.756	0.854	4.65	61	0	1831.04753	2	916.0321655	-2.051746724	60.038475
														VGDAIPAVEVFEGEPGNK	4	2	1	N-Term(iTRAQ8plex); K18(iTRAQ8plex)	0.706	0.599	0.832	4.26	39	0	2436.31308	3	812.7758789	-4.132404541	40.60618667
														LLADPTGAFGK	2	4	1	N-Term(iTRAQ8plex); K11(iTRAQ8plex)	0.633	0.595	0.802	3.6	42	0	1697.99004	2	849.4986572	-8.618111148	27.41986833
														VNLAELFK	2	2	1	N-Term(iTRAQ8plex); K8(iTRAQ8plex)	0.585	0.718	0.721	3.58	36	0	1541.95053	3	514.6550293	-0.406406925	67.76999167
														ETDLLLDDSLVSIFGNRR	1	4	1	N-Term(iTRAQ8plex)	0.561	0.347	0.522	3.16	-	1	2367.28324	3	789.7659302	0.429174392	56.821665
6912280	AHSA1	activator of 90 kDa heat shock protein ATPase homolog 1 isoform 1	NP_036243.1	21.30	5	5	26	338	38.3	5.53	1.074	4.561	2.588																

														VFTTQELVQAFTHAPATLEADR	16	2	1	N-Term(iTRAQ8plex)	1.658	4.14	2.737	7.94	56	0	2749.43607	3	917.1574097	4.126069663	52.60039
														FHMVDGNVSGEFTDLVPEK	2	2	1	N-Term(iTRAQ8plex); K19(iTRAQ8plex)	-	8.334	4.105	5.52	52	0	2729.39371	3	910.4694214	-4.708148111	41.11538
														LDGEASINNR	3	1	1	N-Term(iTRAQ8plex)	0.68	4.148	2.285	5.15	48	0	1392.74338	3	464.9193115	3.511070581	20.93060167
														ETFLTSPEELYR	4	2	1	N-Term(iTRAQ8plex)	1.12	5.822	2.649	4.89	47	0	1788.92668	2	894.96698	-3.062148445	38.67920667
														GIPAPEEER	1	2	1	N-Term(iTRAQ8plex)	1.069	3.899	2.098	-	24	0	1301.69487	2	651.3510742	-4.186451539	16.66154167
6912486	LSM4	U6 snRNA-associated Sm-like protein LSm4 isoform 1	NP_036453.1	20.14	2	2	4	139	15.3	9.99	1.097	1.861	1.638																
														IPDEIIDMVKEEVVAK	2	2	1	N-Term(iTRAQ8plex); K10(iTRAQ8plex); K16(iTRAQ8plex)	1.125	1.748	1.587	6.96	27	1	2740.58901	4	685.90271	-1.609630716	63.95011667
														TAQNHPMLVELK	2	1	1	N-Term(iTRAQ8plex); K12(iTRAQ8plex)	1.071	1.981	1.691	3.82	54	0	1989.12493	2	995.0661011	-8.172323092	24.45492667
6912494	MAPRE1	microtubule-associated protein RP/EB family member 1	NP_036457.1	8.96	2	2	2	268	30.0	5.14	0.767	1.631	1.881																
														LEHEYIQNFK	1	1	1	N-Term(iTRAQ8plex); K10(iTRAQ8plex)	1.074	1.303	1.474	3.98	-	0	1929.05326	3	643.68927	-8.177322918	27.00170667
														GKFQDNFEFVQWFK	1	1	1	N-Term(iTRAQ8plex); K2(iTRAQ8plex); K14(iTRAQ8plex)	0.548	2.041	2.402	3.9	-	1	2732.47939	4	683.8753052	-6.221630411	49.19745333
6912586	PGLS	6-phosphogluconolactonase	NP_036220.1	22.09	4	4	7	258	27.5	6.05	0.779	1.828	2.265																
														ILEDQEENPLPAALVQPHTGK	2	2	1	N-Term(iTRAQ8plex); K21(iTRAQ8plex)	0.618	0.855	1.347	5.75	45	0	2907.60166	3	969.8720703	-0.695973087	37.37189833
														LCWFLDEAAAR	2	2	1	N-Term(iTRAQ8plex); C2(Methylthio)	0.743	2.303	3.23	3.22	50	0	1644.81572	2	822.911499	-1.398863772	58.18508167
														FALGLSGGSLVSMLAR	2	1	1	N-Term(iTRAQ8plex)	1.064	2.123	2.293	3.08	29	0	1883.07768	2	942.0424805	2.656830115	60.6875
														WTLGFCDER	1	1	1	N-Term(iTRAQ8plex); C6(Methylthio)	0.818	1.574	2.236	-	33	0	1476.68437	2	738.8458252	-4.989914911	67.98819667
6912594	PITPNB	phosphatidylinositol transfer protein beta isoform isoform 1	NP_036531.1	10.33	3	3	4	271	31.5	6.87	0.765	1.425	1.504																
														FKWWGLQSK	1	4	1	N-Term(iTRAQ8plex); K2(iTRAQ8plex); K9(iTRAQ8plex)	0.765	1.807	1.504	3.83	-	1	2092.2499	4	523.8179321	1.351337442	34.96604667
														TVEIVHIDIADR	1	5	1	N-Term(iTRAQ8plex)	1.112	1.1	0.913	3.62	-	0	1684.95096	3	562.3218384	-1.554593184	30.179045
														IFTNFHR	2	4	1	N-Term(iTRAQ8plex)	0.666	1.425	1.675	2.69	30	0	1238.68668	3	413.5670776	-6.516288563	24.51372667
6912634	RPL13A	60S ribosomal protein L13a isoform 1	NP_036555.1	30.54	8	8	55	203	23.6	10.93	1.397	1.920	1.749																
														YQAVTATLEEK	10	2	1	N-Term(iTRAQ8plex); K11(iTRAQ8plex)	1.506	1.983	2.113	5.26	50	0	1861.04093	2	931.0224609	-8.100881268	49.686945
														CEGINISGNFYR	26	1	1	N-Term(iTRAQ8plex); C1(Methylthio)	1.418	2.025	1.795	4.84	50	0	1722.8178	2	861.9125366	-3.915491136	31.21515167
														LAHEVGWK	4	2	1	N-Term(iTRAQ8plex); K8(iTRAQ8plex)	0.975	1.145	1.035	3.84	26	0	1547.90036	3	516.6383057	-9.740651379	29.735575
														VFDGIPPPYDKK	1	2	1	N-Term(iTRAQ8plex); K11(iTRAQ8plex); K12(iTRAQ8plex)	1.399	2.01	1.891	3.38	-	1	2288.32102	3	763.4451904	-9.069208723	27.95639667
														VFDGIPPPYDK	4	2	1	N-Term(iTRAQ8plex); K11(iTRAQ8plex)	0.828	1.412	1.308	3.22	32	0	1856.03435	2	928.520813	-3.821019498	26.82252167
														GQAALDR	1	2	1	N-Term(iTRAQ8plex)	1.778	1.952	1.621	-	36	0	1034.58306	2	517.795166	-6.356076214	10.41285
														CEGINISGNFYR	1	1	1	N-Term(iTRAQ8plex); C1(Methylthio); Y11(iTRAQ8plex)	1.395	2.363	2.1	-	29	0	2027.01848	2	861.9125366	-3.915491136	31.21515167
														GPYHFR	1	2	1	N-Term(iTRAQ8plex)	1.03	0.628	0.559	-	21	0	1080.5806	3	360.8650513	-7.968624832	17.69470333
														YLAFLR	7	1	1	N-Term(iTRAQ8plex)	1.497	1.84	1.728	-	18	0	1086.65325	2	543.8372803	5.48707683	38.56344333
6912676	SNW1	SNW domain-containing protein 1 isoform 2	NP_036377.1	7.46	2	2	7	536	61.5	9.52	0.615	0.490	0.471																
														LLEDFGDGGAFPEIHVAQYPLDMGR	5	3	1	N-Term(iTRAQ8plex)	0.79	1.058	0.649	5.28	48	0	3051.52292	3	1017.845825	1.324268583	57.10271167
														EGPVQFEEDPFGLDK	2	1	1	N-Term(iTRAQ8plex); K15(iTRAQ8plex)	0.305	0.227	0.213	3.11	25	0	2315.18259	3	772.3990479	-8.220083976	32.51300167
7019415	SEC61A1	protein transport protein Sec61 subunit alpha isoform 1	NP_037468.1	11.55	4	4	10	476	52.2	8.06	1.153	0.626	0.610																
														TWIEVSGSSAK	2	4	1	N-Term(iTRAQ8plex); K11(iTRAQ8plex)	1.249	1.013	0.917	4.49	30	0	1772.99906	3	591.6712036	-0.701472542	32.93388333
														FSGNLLVSLLGTWSDTSSGGPAR	2	1	1	N-Term(iTRAQ8plex)	1.153	0.503	0.396	4.07	61	0	2626.39067	2	1313.698975	4.855065502	67.35327833
														ETSMVHELNR	3	1	1	N-Term(iTRAQ8plex)	1.197	0.68	0.618	2.96	42	0	1519.79578	3	507.2701111	7.710708312	16.88883333
														AFSPTTVNTGR	2	1	1	N-Term(iTRAQ8plex)	0.988	0.511	0.515	-	32	0	1454.78508	2	727.8961792	-3.739055088	19.48481
														ETSMVHELNR	1	1	1	N-Term(iTRAQ8plex); M4(Oxidation)	1.049	0.662	0.745	-	21	0	1535.77567	3	507.2701111	7.710708312	16.88883333
7019545	NENF	neudesin precursor	NP_037481.1	5.81	1	1	2	172	18.8	5.69	0.900	1.009	0.923																
														GVVFDVTSGK	2	1	1	N-Term(iTRAQ8plex); K10(iTRAQ8plex)	0.9	1.009	0.923	3.8	38	0	1616.95098	2	808.979126	2.575237709	35.60325
7305503	STOML2	stomatin-like protein 2, mitochondrial isoform a	NP_038470.1	34.55	10	10	28	356	38.5	7.39	0.799	0.502	0.376																
														AEQINQAAGEASAVLAK	2	4	1	N-Term(iTRAQ8plex); K17(iTRAQ8plex)	0.716	0.394	0.355	6.33	98	0	2279.29131	2	1140.149292	4.258369408	35.80784333
														ESMQMQVEAER	3	3	1	N-Term(iTRAQ8plex)	0.742	0.406	0.338	4.96	57	0	1641.7918	2	821.3995361	2.402463964	23.418915
														ERESLNASIVDAINQAADCWGIR	2	3	1	N-Term(iTRAQ8plex); C19(Methylthio)	-	-	-	4.19	55	1	2881.43534	3	961.1499634	3.961423869	67.04596
														DVQGTDASLDEELDRVK	3	4	1	N-Term(iTRAQ8plex); K17(iTRAQ8plex)	0.917	0.666	0.342	3.81	30	1	2498.30776	4	625.3323975	-4.703718428	28.62479667
														DVQGTDASLDEELDR	1	4	1	N-Term(iTRAQ8plex)	0.35	0.753	1.057	3.65	-	0	1966.95371	3	656.3227539	1.498341693	27.92354333
														ESLNASIVDAINQAADCWGIR	3	3	1	N-Term(iTRAQ8plex); C17(Methylthio)	0.547	0.507	0.316	3.61	59	0	2596.28885	3	866.1011353	3.331760143	69.38751333
														NTVVLFVPQQEAWVVER	1	2	1	N-Term(iTRAQ8plex)	0.739	0.307	0.429	3.57	-	0	2318.27488	3	773.4298096	-2.68123471	48.77752333
														ILEPGLNILIPVLDR	5	3	1	N-Term(iTRAQ8plex)	0.977	0.46	0.422	3.33	58	0	1979.22068	2	990.116394	2.42022241	65.81669
														ESMQMQVEAER	2	3	1	N-Term(iTRAQ8plex); M3(Oxidation)	0.799	0.44	0.376	3.09	43	0	1657.78191	2	821.3995361	2.402463964	23.418915
														APVPGTPDSLSSGSSR	4	4	1	N-Term(iTRAQ8plex)	0.892	0.599	0.444	2.95	53	0	1818.94536	2	909.9763184	-2.529598388	17.61495833
														DIHVPPR	2	3	1	N-Term(iTRAQ8plex)	0.935	0.827	0.922	2.87	22	0	1137.67014	3	379.8948975	1.694544619	14.18729833
7330335	CLIC4	chloride intracellular channel protein 4	NP_039234.1	14.23	4	4	12	253	28.8	5.59	0.784	0.892	3.124																
														KFLDGNEMTLADCNLLPK	6	1	1	N-Term(iTRAQ8plex); K1(iTRAQ8plex); C13(Methylthio); K18(iTRAQ8plex)	1.165	1.042	3.295	5.91	42	1	2980.61277	3	994.199707	-7.651001248	47.66217833
														IEEFLEEVLCPPK	2	1	1	N-Term(iTRAQ8plex); C10(Methylthio); K13(iTRAQ8plex)	-	-	-	3.62	44	0	2200.19107	3	734.0685425	2.564273382	67.09720333
														TDVNKIEEFLEEVLCPPK	3	1	1	N-Term(iTRAQ8plex); K5(iTRAQ8plex); C15(Methylthio); K18(iTRAQ8plex)	-	-	-	3.5	33	1	3061.65957	4	766.1703491	-3.976828697	69.52943333
														FLDGNEMTLADCNLLPK	1	1	1	N-Term(iTRAQ8plex); C12(Methylthio); K17(iTRAQ8plex)	0.696	0.852	2.962	-	38	0	2548.31279	2	1274.660034	2.256174907	54.542545
7427519	MCM6	DNA replication licensing factor MCM6	NP_005906.2	9.87	7	7	15	821	92.8	5.41	1.184	1.600	1.428																
														DQVAIHEAMEQQTISITK	2	1	1	N-Term(iTRAQ8plex); K18(iTRAQ8plex)	1.184	1.588	1.687	5.36	28	0	2650.42636	3	884.1469727	-2.537924214	29.182655
														GVLLMLFGGVPK	3	1	1	N-Term(iTRAQ8plex); K12(iTRAQ8plex)	0.907	4.208	1.837	4.46	35	0	1839.13401	3	613.7161865	-2.541946194	51.257175
														ISNLIVLHLR	4	1	1	N-Term(iTRAQ8plex)	0.671	1.517	1.386	4.34	56	0	1481.94101	3	494.6518555	-4.01375203	42.805795
														VSGVDGYETEGIR	2	1	1	N-Term(iTRAQ8plex)	1.391	2.167	2.01	3.9	41	0	1685.86296	2	843.4351196	-1.102498975	26.65936667
														ESEDFIVEQYK	2	1	1	N-Term(iTRAQ8plex); K11(iTRAQ8plex)	0.554	0.927	0.942	2.97	32	0	1995.03769	3	665.684082	-7.733415917	40.13655667
														MHCCDEVQPK	1	1	1	N-Term(iTRAQ8plex); C3(Methylthio); C4(Methylthio); K10(iTRAQ8plex)	0.999	1.376	1.144	2.91	-	0	1889.84745	3	630.6206665	-9.861662812	25.02743
														SLEVILR	1	1	1	N-Term(iTRAQ8plex)	1.284	4.59	4.083	-	20	0	1133.71489	2	567.361084	-4.136799548	31.60939833
7524346	AK2	adenylate kinase 2, mitochondrial isoform b	NP_037543.1	43.10	7	7	47	232	25.6	7.88	0.607	0.991	0.894																
														LAENFCVCHLATGDMLR	4	5	1	N-Term(iTRAQ8plex); C6(Methylthio); C8(Methylthio)	0.467	1.297	1.237	5.6	57	0	2289.06534	3	763.6932983	1.25311824	56.906045
														LVSDEMVVELIEK	11	7	1	N-Term(iTRAQ8plex); K13(iTRAQ8plex)	0.551	0.992	0.797	5.08	72	0	2112.20391	2	1056.613647	5.556713682	50.322845
														EKLDSVIEFSIPDSLLIR	7	6	1	N-Term(iTRAQ8plex); K2(iTRAQ8plex)	0.811	1.152	0.997	4.53	54	1	2682.55698	3	894.8571777	1.158839551	57.22625167
														LQAYHTQTTPLIEYYR	5	7	1	N-Term(iTRAQ8plex)	0.889	1.046	0.895	4.38	31	0	2301.21018	3	767.7415771	-3.438670992	34.51386
														GIHSAIDASQTPDVVFASILAAFSK	15	7	1	N-Term(iTRAQ8plex); K25(iTRAQ8plex)	-	-	-	4.36	39	0	3153.74941	4	789.1928101	2.818567114	67.87202333
														LDSVIEFSIPDSLLIR	3	6	1	N-Term(iTRAQ8plex)	0.566	0.688	0.513	4.33	57	0	2121.21367	2	1061.110474	1.286690569	65.76134167
														QAEMLDDLMEK	2	7	1	N-Term(iTRAQ8plex); K11(iTRAQ8plex)	0.593	0.905	0.892	3.73	42	0	1930.99417	3	644.3362427	-6.885842966	62.92962333
														IIHPK	1	0	1	N-Term(iTRAQ8plex); K5(iTRAQ8plex)	-	-	-	-	17	0	1215.80692	3	405.9404907	2.934362285	17.55837667
7657015	RTCB	tRNA-splicing ligase RtcB homolog	NP_055121.1	1.39	1	1	3	505	55.2	7.23	1.000	1.404	1.745																
														LMFEELR	3	1	1	N-Term(iTRAQ8plex)	1	1.404	1.745	2.79	33	0	1241.67839	2	621.3428345	-6.595085119	27.48723333
7657257	TOMM20	mitochondrial import receptor subunit TOM20 homolog	NP_055580.1	13.79	1	1	2	145	16.3	8.60	-	-	-																
														FFLEEIQLGEELLAQGEYEK	2	1	1	N-Term(iTRAQ8plex); K20(iTRAQ8plex)	-	-	-	2.9	36	0	2993.62021	3	998.5449219	7.800279709	65.51142667
7657345	MTCH1	mitochondrial carrier homolog 1 PSAP-LS	NP_055156.1	5.65	1	1	1	372	39.9	9.48	0.956	1.465	0.992																
														LLIQVGHEPMPPTLGTNVLGR	1	4	1	N-Term(iTRAQ8plex)	0.956	1.465	0.992	3.72	-	0	2546.45414	3	849.489563	4.32932001	39.41646833
7657369	NDUFA8	NADH dehydrogenase [ubiquinone] 1 alpha subcomplex subunit 8 isoform 1	NP_055037.1	25.00	4	4	7	172	20.1	7.65	0.888	0.475	0.322																
														FYFWTK	2	1	1	N-Term(iTRAQ8plex); K6(iTRAQ8plex)	0.861	0.418	0.207	2.96	27	0	1499.85413	3	500.6228943	2.292430484	38.039225
														FDECVLDK	2	2	1	N-Term(iTRAQ8plex); C4(Methylthio); K8(iTRAQ8plex)	0.91	0.723	0.423	2.89	29	0	1622.83977	2	811.9235229	1.181249359	54.84643167
														CALDFFR	2	2	1	N-Term(iTRAQ8plex); C1(Methylthio)	0.973	0.475	0.312	-	25	0	1221.60198	2	611.3046265	-3.467569957	68.498995
														HCAEPFTEYWTCIDYTGQQLFR	1	2	1	N-Term(iTRAQ8plex); C2(Methylthio); C12(Methylthio)	0.84	-	0.322	-	23	0	3104.38437	3	1035.466309	4.081076867	66.81794333
7657381	PRPF19	pre-mRNA-processing factor 19	NP_055317.1	14.29	7	7	16	504	55.1	6.61	1.311	0.885	0.852																
														VTSVVFHPSQDLVFSASPDATIR	2	1	1	N-Term(iTRAQ8plex)	0.699	0.554	0.939	5.49	44	0	2777.47995	3	926.4981689	0.836112583	42.52324667
														IWSVPNASCVQVVR	5	1	1	N-Term(iTRAQ8plex); C9(Methylthio)	1.581	1.316	0.969	4.08	45	0	1908.00858	3	636.6743774	-2.716563142	47.86226667
														SSEQILATLK	4	1	1	N-Term(iTRAQ8plex); K10(iTRAQ8plex)	1.029	0.73	0.745	3.93	31	0	1698.01933	2	849.5133057	-3.796536202	41.12569667
														KVTSVVFHPSQDLVFSASPDATIR	1	1	1	N-Term(iTRAQ8plex); K1(iTRAQ8plex)	-	-	-	3.5	-	1	3209.76528	4	803.1967773	-3.951006973	39.12659833
														NVVVFDK	1	1	1	N-Term(iTRAQ8plex); K7(iTRAQ8plex)	1.285	0.86	0.865	3.36	-	0	1428.87236	2	714.9398193	3.688749265	19.648135
														ATVLTTER	1	1	1	N-Term(iTRAQ8plex)	1.419	1.038	0.839	-	36	0	1194.69585	2	597.8515625	-3.116207364	17.867385
														FIASTGMDR	2	1	1	N-Term(iTRAQ8plex)	1.221	0.865	0.604	-	36	0	1301.6779	2	651.3425903	-3.577886605	19.05293333
														IWDIR	1	4	3	N-Term(iTRAQ8plex)	-	-	-	-	33	0	1006.59648	2	503.8018799	-2.233421647	28.65469833
7657441	PDAP1	28 kDa heat- and acid-stable phosphoprotein	NP_055706.1	7.18	1	1	2	181	20.6	8.87	1.733	1.980	2.093																
														GVEGLIDIENPNR	2	1	1	N-Term(iTRAQ8plex)	1.733	1.98	2.093	4.55	46	0	1729.94109	2	865.4741821	1.396774624	34.10132167
7661532	NOB1	RNA-binding protein NOB1	NP_054781.1	3.64	1	1	1	412	46.6	7.18	1.025	1.000	1.402																
														APVEHVVADAGAFLR	1	1	1	N-Term(iTRAQ8plex)	1.025	1	1.402	-	28	0	1856.03226	3	619.348938	-0.533711174	42.21416
7661672	POLDIP2	polymerase delta-interacting protein 2 isoform 1	NP_056399.1	2.72	1	1	1	368	42.0	8.63	1.369	1.174	1.093																
														GVVLFPWQAR	1	1	1	N-Term(iTRAQ8plex)	1.369	1.174	1.093	-	34	0	1476.85588	2	738.9315796	-4.759259448	34.56487167
7661714	FAM3C	protein FAM3C precursor	NP_055703.1	5.73	1	1	4	227	24.7	8.29	1.392	1.123	0.599																
														ICLEDNVLMSGVK	4	1	1	N-Term(iTRAQ8plex); C2(Methylthio); K13(iTRAQ8plex)	1.392	1.123	0.599	3.66	34	0	2075.10788	3	692.3741455	-3.898123383	69.56832167
7661728	LAMTOR2	ragulator complex protein LAMTOR2 isoform 1	NP_054736.1	16.80	2	2	6	125	13.5	5.40	0.588	0.582	0.447																
														VANLLLCMYAK	4	1	1	N-Term(iTRAQ8plex); C7(Methylthio); K11(iTRAQ8plex)	0.619	0.609	0.524	4.5	40	0	1893.05802	3	631.6908569	-2.1366219	80.26216667
														FILMDCMEGR	2	1	1	N-Term(iTRAQ8plex); C6(Methylthio)	0.399	0.485	0.37	3.57	40	0	1564.73345	2	782.8703613	2.322271732	52.39977667
7661730	MRPS28	28S ribosomal protein S28, mitochondrial	NP_054737.1	13.37	3	3	4	187	20.8	9.10	1.234	0.819	0.723																
														LLDLELTSR	1	1	1	N-Term(iTRAQ8plex)	1.501	0.681	0.623	3.48	-	0	1363.8112	2	682.4092407	0.985125586	38.688425
														NVESFASMLR	1	1	1	N-Term(iTRAQ8plex)	1.312	0.793	0.721	3.33	-	0	1457.78276	2	729.3950195	7.080027811	37.00826333
														FHCVCR	2	1	1	N-Term(iTRAQ8plex); C3(Methylthio); C5(Methylthio)	1.158	0.965	0.743	-	25	0	1160.50806	3	387.5075378	-5.026268071	25.70697
7661806	MRPL15	39S ribosomal protein L15, mitochondrial	NP_054894.1	10.81	2	2	3	296	33.4	10.01	0.999	0.446	0.562																
														QIFFGLAPGWVVNMADKK	2	1	1	N-Term(iTRAQ8plex); K17(iTRAQ8plex); K18(iTRAQ8plex)	-	-	-	4.41	44	1	2933.68789	4	734.1774292	1.355160136	56.29232
														SLDIVCKPVPFFLR	1	1	1	N-Term(iTRAQ8plex); C6(Methylthio); K7(iTRAQ8plex)	0.999	0.446	0.562	3.76	-	0	2288.30703	4	572.8322144	-2.16088054	56.750645
7661822	DYNLRB1	dynein light chain roadblock-type 1 isoform a	NP_054902.1	43.75	2	2	4	96	10.9	7.25	0.633	-	1.151																
														STMDNPTTTQYASLMHSFILK	2	3	1	N-Term(iTRAQ8plex); K21(iTRAQ8plex)	-	-	-	4.26	27	0	2994.55117	4	749.3932495	-0.461883283	47.11035167

														NEIMVAPDKDYFLIVIQNPTE	2	3	1	N-Term(iTRAQ8plex); K9(iTRAQ8plex)	0.633	-	1.151	3.84	27	1	3057.64981	3	1019.888123	2.303444217	51.687195
7661910	EMC2	ER membrane protein complex subunit 2	NP_055488.1	6.40	1	1	2	297	34.8	6.57	0.754	1.411	0.406																
														NSEQIVEVGEELINEYASK	2	1	1	N-Term(iTRAQ8plex); K19(iTRAQ8plex)	0.754	1.411	0.406	4.51	48	0	2759.46695	3	920.4938354	3.972433025	53.08564167
7661914	PSMD6	26S proteasome non-ATPase regulatory subunit 6 isoform 2	NP_055629.1	25.45	8	8	23	389	45.5	5.62	0.660	1.119	1.239																
														YSVFFQSLAVVEQEMK	6	6	1	N-Term(iTRAQ8plex); K16(iTRAQ8plex)	0.66	1.33	1.34	7.39	52	0	2513.35984	3	838.4581299	1.104138929	65.42079167
														GAEILEVLHSLPAVR	4	7	1	N-Term(iTRAQ8plex)	-	-	-	4.08	49	0	1908.1287	3	636.7144165	3.477208708	56.96959833
														IHAYSQLLESYR	4	6	1	N-Term(iTRAQ8plex)	0.83	0.977	0.997	3.79	41	0	1783.95554	3	595.3233643	-4.989909426	38.45952833
														KDWLFAPHYR	2	6	1	N-Term(iTRAQ8plex); K1(iTRAQ8plex)	-	1.844	1.795	3.6	27	1	1941.09511	4	486.0292358	-0.215249949	30.70915333
														RLDEELEDAEK	2	7	1	N-Term(iTRAQ8plex); K11(iTRAQ8plex)	0.843	1.087	1.251	3.36	30	1	1955.04233	3	652.3522949	-6.076484475	20.33108167
														FLLSLPEHR	3	3	1	N-Term(iTRAQ8plex)	0.429	0.651	0.955	2.89	52	0	1415.82557	3	472.6133728	-4.02319462	29.93990833
														GDAAVRDELMAAVR	1	3	1	N-Term(iTRAQ8plex)	0.618	1.741	1.74	-	32	1	1777.94455	3	593.3197021	-4.920347303	28.90324667
														SLIEEGGDWDRR	1	7	1	N-Term(iTRAQ8plex)	0.722	1.102	1.051	-	27	1	1736.87766	3	579.6307373	-5.361710936	21.67258833
7661920	EIF4A3	eukaryotic initiation factor 4A-III	NP_055555.1	30.66	10	12	44	411	46.8	6.73	1.309	1.268	0.900																
														YLPPATQVVLISATLPHEILEMTNK	2	2	1	N-Term(iTRAQ8plex); K25(iTRAQ8plex)	-	-	-	7	48	0	3386.89956	4	847.4803467	-6.58255755	85.41206833
														MLVLDEADEMLNK	10	2	1	N-Term(iTRAQ8plex); K13(iTRAQ8plex)	1.471	1.221	0.877	5.96	65	0	2129.14775	2	1065.077515	1.616316609	50.17885167
														MLVLDEADEMLNK	2	2	1	N-Term(iTRAQ8plex); M1(Oxidation); K13(iTRAQ8plex)	1.279	1.149	0.829	4.91	54	0	2145.11991	2	1065.077515	1.616316609	50.17885167
														KLDYGQHVVAGTPGR	4	2	1	N-Term(iTRAQ8plex); K1(iTRAQ8plex)	1.825	1.604	1.251	4.27	39	1	2206.23868	3	736.0844116	-7.533041124	18.52711333
														ETQALILAPTR	2	1	1	N-Term(iTRAQ8plex)	1.379	1.55	1.211	3.67	38	0	1516.89433	2	758.9508057	-3.788392997	27.802255
														LDYGQHVVAGTPGR	3	2	1	N-Term(iTRAQ8plex)	1.272	0.858	0.719	3.62	25	0	1773.95084	3	591.9884644	-2.328972991	21.10281
														ELAVQIQK	2	1	1	N-Term(iTRAQ8plex); K8(iTRAQ8plex)	1.649	1.536	1.234	3.43	50	0	1536.96123	2	768.9842529	2.770362254	32.10022667
														EQIYDVYR	4	2	1	N-Term(iTRAQ8plex)	1.184	0.969	0.879	2.69	30	0	1389.73113	2	695.3692017	-0.32711341	26.26644167
														VFDMIR	3	2	1	N-Term(iTRAQ8plex)	1.224	1.373	0.882	-	34	0	1084.60784	2	542.8075562	-4.467619681	28.85468667
														VLISTDVWAR	1	2	1	N-Term(iTRAQ8plex)	1.555	1.525	1.206	-	27	0	1463.84673	2	732.427002	-3.876098258	39.56957
														FMTDPIR	1	2	1	N-Term(iTRAQ8plex)	1.223	1.52	1.013	-	21	0	1183.63506	2	592.321167	-8.156175812	21.74880667
														GIYAYGFEKPSAIQQR	9	5	3	N-Term(iTRAQ8plex); K9(iTRAQ8plex)	-	-	-	4.36	33	0	2436.33755	4	609.8397827	-5.044589113	29.05811333
														VDWLTEK	1	5	3	N-Term(iTRAQ8plex); K7(iTRAQ8plex)	-	-	-	3.27	-	0	1498.85893	2	749.9331055	-9.098242795	30.08201167
7661922	RAB21	ras-related protein Rab-21	NP_055814.1	32.44	7	7	20	225	24.3	7.94	1.644	1.242	0.916																
														VNLAIWDTAGQER	2	1	1	N-Term(iTRAQ8plex)	1.591	1.139	0.887	4.98	47	0	1776.96172	2	888.9844971	3.976574484	35.88282167
														HVSIQEAESYAESVGAK	2	1	1	N-Term(iTRAQ8plex); K17(iTRAQ8plex)	0.899	0.887	1.125	4.77	46	0	2413.27311	3	805.0958862	-3.673789996	36.14526833
														VVLLGEGCVGK	4	1	1	N-Term(iTRAQ8plex); C8(Methylthio); K11(iTRAQ8plex)	1.432	1.052	0.964	4.58	43	0	1727.99932	2	864.5032959	-0.889539198	33.66919333
														GIEELFLDLCK	7	1	1	N-Term(iTRAQ8plex); C10(Methylthio); K11(iTRAQ8plex)	2.043	1.241	0.782	4.33	78	0	1934.05753	2	967.5334473	0.442391352	63.032615
														FHALGPIYYR	2	1	1	N-Term(iTRAQ8plex)	1.48	2.468	1.924	3.23	35	0	1540.86856	3	514.2943726	6.991592943	32.36035833
														GIEELFLDLCKR	1	1	1	N-Term(iTRAQ8plex); C10(Methylthio); K11(iTRAQ8plex)	3.001	2.029	1.022	2.91	-	1	2090.15842	3	697.3909912	-0.699428892	65.57838167
														MIETAQVDER	2	1	1	N-Term(iTRAQ8plex); M1(Oxidation)	2.368	1.495	1.051	2.73	42	0	1511.76763	2	756.3874512	-0.086373228	19.15052833
7662502	MCTS1	malignant T-cell-amplified sequence 1 isoform 1	NP_054779.1	19.34	2	2	3	181	20.5	8.82	-	4.647	1.960																
														LYPAAVDTIVAIMAEGK	2	2	1	N-Term(iTRAQ8plex); K17(iTRAQ8plex)	-	-	-	4.27	43	0	2370.35923	3	790.7912598	1.21278954	67.05708
														GIGIENIHYLNDGLWHMK	1	2	1	N-Term(iTRAQ8plex); K18(iTRAQ8plex)	-	4.647	1.96	4.15	-	0	2718.46035	4	680.3705444	-1.58672376	41.777835
7662645	MRPS18B	28S ribosomal protein S18b, mitochondrial	NP_054765.1	15.12	2	2	3	258	29.4	9.38	0.523	0.571	0.531																
														DLDFSTSHGAVSATPPAPTLVSGDPWYPWYNWK	1	1	1	N-Term(iTRAQ8plex); K33(iTRAQ8plex)	-	-	-	4.24	-	0	4271.14223	4	1068.541016	2.037234896	56.16543833
														LHVDFR	2	1	1	N-Term(iTRAQ8plex)	0.523	0.571	0.531	-	33	0	1090.62915	3	364.2145691	-1.77629087	18.60762667
7662677	NSA2	ribosome biogenesis protein NSA2 homolog isoform 1	NP_055701.1	4.23	1	1	4	260	30.0	10.27	1.991	0.869	1.022																
														ATFCLPILGVK	4	2	1	N-Term(iTRAQ8plex); C4(Methylthio); K11(iTRAQ8plex)	1.991	0.869	1.022	4.73	48	0	1816.06145	2	908.5343628	-3.900382372	45.61167167
7669494	GCDH	glutaryl-CoA dehydrogenase, mitochondrial isoform b precursor	NP_039663.1	6.07	1	1	2	428	47.3	8.31	1.205	0.391	-																
														SSRPEFDWQDPLVLEEQLTTDEILIR	2	5	1	N-Term(iTRAQ8plex)	1.205	0.391	-	5.71	86	0	3433.78159	3	1145.265381	0.640118793	63.37412167
7705425	MRPS17	28S ribosomal protein S17, mitochondrial	NP_057053.1	6.92	1	1	2	130	14.5	9.85	1.112	0.628	0.712																
														HELAEIVFK	2	1	1	N-Term(iTRAQ8plex); K9(iTRAQ8plex)	1.112	0.628	0.712	3.93	37	0	1694.00237	2	847.5048218	-4.353220194	30.613515
7705626	MRPS16	28S ribosomal protein S16, mitochondrial	NP_057149.1	14.60	1	1	2	137	15.3	9.50	0.719	0.282	0.523																
														LLGLAGFFPLHPMMITNAER	2	1	1	N-Term(iTRAQ8plex)	0.719	0.282	0.523	4.85	41	0	2532.38211	3	844.7988892	1.880810341	65.20584833
7705630	MRPS18C	28S ribosomal protein S18c, mitochondrial isoform 1	NP_057151.1	13.38	1	1	1	142	15.8	9.55	-	-	-																
														NVQLLSQFVSPFTGCIYGR	1	1	1	N-Term(iTRAQ8plex); C15(Methylthio)	-	-	-	3.69	-	0	2479.28427	3	827.0996094	2.538061111	66.41381833
7705636	GOLT1B	vesicle transport protein GOT1B	NP_057156.1	10.87	1	1	10	138	15.4	10.36	1.356	1.066	1.229																
														RVPVLGSLLNLPGIR	10	2	1	N-Term(iTRAQ8plex)	1.356	1.066	1.229	5.11	62	1	1908.2067	3	636.7404175	0.3290479	52.40224333
7705696	TXNDC12	thioredoxin domain-containing protein 12 precursor	NP_056997.1	8.72	1	1	2	172	19.2	5.40	1.030	1.633	1.177																
														EAAASGLPLMVIIHK	2	1	1	N-Term(iTRAQ8plex); K15(iTRAQ8plex)	1.03	1.633	1.177	5.54	25	0	2158.28189	3	720.0988159	-2.769237934	40.24062
7705706	RPS27L	40S ribosomal protein S27-like	NP_057004.1	38.10	1	3	20	84	9.5	9.45	0.971	1.001	1.577																
														DLLHPSLEEEK	4	1	1	N-Term(iTRAQ8plex); K11(iTRAQ8plex)	0.971	1.001	1.577	3.47	29	0	1918.05741	3	640.0239868	-8.755786306	23.10117667
														LVQSPNSYFMDVK	12	2	2	N-Term(iTRAQ8plex); K13(iTRAQ8plex)	-	-	-	5.65	57	0	2136.15166	2	1068.579468	-4.827547277	36.80544333
														LTEGCSFR	4	2	2	N-Term(iTRAQ8plex); C5(Methylthio)	-	-	-	-	30	0	1262.60991	2	631.8085938	-6.012482505	23.43498833
7705805	MRPS2	28S ribosomal protein S2, mitochondrial	NP_057118.1	2.70	1	1	1	296	33.2	9.26	1.322	0.673	0.511																
														GIILFISR	1	2	1	N-Term(iTRAQ8plex)	1.322	0.673	0.511	-	22	0	1222.77886	2	611.8930664	-2.997359238	34.01780833
7705855	HSD17B12	very-long-chain 3-oxoacyl-CoA reductase	NP_057226.1	22.76	7	7	29	312	34.3	9.32	0.696	0.774	0.406																
														SKGVFVQSVLPYFVATK	2	3	1	N-Term(iTRAQ8plex); K2(iTRAQ8plex); K17(iTRAQ8plex)	0.747	1.075	0.736	5.65	39	1	2782.65744	3	928.223999	-2.158900191	45.82002
														GVFVQSVLPYFVATK	2	3	1	N-Term(iTRAQ8plex); K15(iTRAQ8plex)	0.752	0.447	0.31	4.39	31	0	2263.33371	3	755.1160889	1.158490923	51.97251
														MININILSVCK	8	3	1	N-Term(iTRAQ8plex); C10(Methylthio); K11(iTRAQ8plex)	0.624	0.682	0.359	4.38	54	0	1902.08512	2	951.5444336	-1.020846061	47.381675
														MTQLVLPGMVER	4	3	1	N-Term(iTRAQ8plex)	0.822	0.905	0.533	4.18	38	0	1677.9301	2	839.468689	-1.966703089	42.50626667
														ISYSLFTALR	10	1	1	N-Term(iTRAQ8plex)	0.709	0.745	0.363	3.15	34	0	1474.85637	2	737.9315796	-0.846686329	48.50589
														TFVDFFSQCLHEEYR	2	3	1	N-Term(iTRAQ8plex); C9(Methylthio); Y14(iTRAQ8plex)	0.248	1.116	0.344	-	45	0	2575.26211	4	644.5709839	1.977805235	57.90717333
														VVLISR	1	3	1	N-Term(iTRAQ8plex)	0.809	0.681	0.399	-	19	0	990.65953	2	495.8334045	-1.815798374	22.75909167
7705927	ATPIF1	ATPase inhibitor, mitochondrial isoform 1 precursor	NP_057395.1	9.43	1	1	2	106	12.2	9.35	0.891	0.743	0.502																
														HHEEEIVHHK	2	1	1	N-Term(iTRAQ8plex); K10(iTRAQ8plex)	0.891	0.743	0.502	4.24	-	0	1903.02628	3	635.0136108	-6.92687779	8.704855
7706057	MRPL27	39S ribosomal protein L27, mitochondrial	NP_057588.1	14.86	2	2	3	148	16.1	10.42	0.867	0.767	0.691																
														WHPGAHVGVGK	1	1	1	N-Term(iTRAQ8plex); K11(iTRAQ8plex)	0.674	0.596	0.641	3.17	-	0	1753.02811	3	585.0142212	9.297639965	16.70548
														CLYALEEGIVR	2	1	1	N-Term(iTRAQ8plex); C1(Methylthio)	1.116	0.988	0.745	3.04	23	0	1615.85002	2	808.4286499	0.650360289	51.747635
7706254	NOP58	nucleolar protein 58	NP_057018.1	27.60	10	11	52	529	59.5	8.92	1.284	0.829	0.761																
														YDAFGEDSSSAMGVENR	2	1	1	N-Term(iTRAQ8plex)	1.327	0.695	0.795	8.43	71	0	2138.96921	3	713.6612549	4.174954174	29.53177
														EMAAMCLGLAHSLSR	12	1	1	N-Term(iTRAQ8plex); C6(Methylthio)	1.206	0.835	0.768	6.17	66	0	1939.95444	3	647.3234253	1.484739508	59.08615167
														LSELLPEEVEAEVK	10	1	1	N-Term(iTRAQ8plex); K14(iTRAQ8plex)	1.663	0.909	0.823	5.89	45	0	2193.25357	3	731.7560425	2.768896163	45.53615833
														HAASTVQILGAEK	6	1	1	N-Term(iTRAQ8plex); K13(iTRAQ8plex)	1.474	0.894	0.815	5.34	60	0	1933.12615	2	967.0667725	-3.338115172	17.11180333
														EMAAMCLGLAHSLSR	2	1	1	N-Term(iTRAQ8plex); M2(Oxidation); C6(Methylthio)	1.17	0.919	1.257	5.14	54	0	1955.94284	3	647.3234253	1.484739508	59.08615167
														EAHEPLAVADAK	4	1	1	N-Term(iTRAQ8plex); K12(iTRAQ8plex)	0.992	0.605	0.546	4.92	57	0	1859.04082	2	930.0240479	-4.040910128	16.84067333
														FQDTAEALAAFTALMEGK	4	1	1	N-Term(iTRAQ8plex); K18(iTRAQ8plex)	1.318	0.582	0.599	4.9	93	0	2522.34453	2	1261.675903	0.935130232	64.06013333
														SQMDGLIPGVEPR	2	1	1	N-Term(iTRAQ8plex)	1.5	0.764	0.845	4.6	24	0	1702.91741	2	851.9623413	4.336497567	31.76590167
														LNLSCIHSPVVNELMR	6	1	1	N-Term(iTRAQ8plex); C5(Methylthio)	1.077	0.612	0.744	4.31	34	0	2175.13675	3	725.7171021	-1.057127858	49.331955
														IISDNLTYCK	2	1	1	N-Term(iTRAQ8plex); C9(Methylthio); K10(iTRAQ8plex)	0.943	0.665	0.504	3.15	30	0	1823.98221	3	608.6655884	-1.8368673	56.31981833
														LIAHAGSLLNLAK	1	1	1	N-Term(iTRAQ8plex); K13(iTRAQ8plex)	-	-	-	-	26	0	1929.21218	4	483.0585022	0.828114751	41.06674333
														MLAAK	1	8	2	N-Term(iTRAQ8plex); M1(Oxidation); K5(iTRAQ8plex)	-	-	-	-	22	0	1157.719	3	386.5778503	1.504229778	12.19833333
7706278	ERGIC3	endoplasmic reticulum-Golgi intermediate compartment protein 3 isoform b	NP_057050.1	3.66	1	1	2	383	43.2	6.06	0.754	0.793	0.664																
														LDKDGIPVSSEAER	2	2	1	N-Term(iTRAQ8plex); K3(iTRAQ8plex)	0.754	0.793	0.664	3.29	25	1	2124.17337	3	708.7293091	-1.109751179	21.53746833
7706322	C14orf166	UPF0568 protein C14orf166	NP_057123.1	15.57	3	3	16	244	28.1	6.65	0.804	1.458	1.633																
														HILGFDTGDAVLNEAAQILR	4	1	1	N-Term(iTRAQ8plex)	0.804	1.944	1.098	6.25	77	0	2457.34421	3	819.7862549	1.546424966	59.28499333
														NFIVWLEDQK	6	1	1	N-Term(iTRAQ8plex); K10(iTRAQ8plex)	1.043	1.824	1.889	5.14	42	0	1900.06034	3	634.0249634	-9.765170569	73.48268
														LLHIEELR	6	1	1	N-Term(iTRAQ8plex)	0.741	1.213	1.548	3.29	55	0	1326.80555	3	442.940033	0.628823836	29.83946667
7706326	SF3B6	splicing factor 3B subunit 6	NP_057131.1	32.00	3	3	11	125	14.6	9.38	1.245	1.082	1.244																
														ITAEEMYDIFGK	6	1	1	N-Term(iTRAQ8plex); K12(iTRAQ8plex)	1.258	1.027	1.082	4.86	56	0	2025.06694	2	1013.037109	-7.599338906	70.79629333
														GTAYVVYEDIFDAK	4	1	1	N-Term(iTRAQ8plex); K14(iTRAQ8plex)	1.175	1.022	1.244	4.78	54	0	2199.17356	3	733.7293701	-2.65290496	50.72651333
														NACDHLSGFNVCNR	1	1	1	N-Term(iTRAQ8plex); C3(Methylthio); C12(Methylthio)	1.233	1.455	1.528	-	21	0	1945.8367	3	649.2837524	-3.915655289	36.74189833
7706339	PPIL1	peptidyl-prolyl cis-trans isomerase-like 1	NP_057143.1	6.63	1	1	1	166	18.2	7.99	1.480	1.601	1.526																
														QFEDELHPDLK	1	1	1	N-Term(iTRAQ8plex); K11(iTRAQ8plex)	1.48	1.601	1.526	3.4	-	0	1979.0643	3	660.3596191	-2.600788723	24.57227167
7706343	FAM96B	mitotic spindle-associated MMXD complex subunit MIP18	NP_057146.1	12.88	1	1	2	163	17.7	5.19	-	1.175	1.675																
														VAAALENTHLLEVVNQCLSAR	2	1	1	N-Term(iTRAQ8plex); C17(Methylthio)	-	1.175	1.675	4.62	43	0	2601.38028	3	867.7982788	0.296333368	66.611515
7706423	LSM7	U6 snRNA-associated Sm-like protein LSm7	NP_057283.1	24.27	1	1	2	103	11.6	5.27	-	-	-																
														GFDPLLNLVLDGTIEYMRDPDDQYK	2	2	1	N-Term(iTRAQ8plex); K25(iTRAQ8plex)	-	-	-	4.43	35	1	3535.8229	4	884.7111816	-0.312407474	68.05503833
7706425	LSM8	LSM8 homolog, U6 small nuclear RNA associated	NP_057284.1	27.08	2	2	9	96	10.4	4.48	0.949	1.436	1.396																
														GFDQTINLILDESHER	8	1	1	N-Term(iTRAQ8plex)	0.988	1.406	1.518	5.71	57	0	2191.129	3	731.0478516	-0.32853497	49.78660667
														TVAVITSDGR	1	1	1	N-Term(iTRAQ8plex)	0.912	1.466	1.156	-	24	0	1322.75505	2	661.8811646	-2.350260112	20.3562
7706441	VPS29	vacuolar protein sorting-associated protein 29 isoform 1	NP_057310.1	20.33	3	3	20	182	20.5	6.79	1.417	2.803	2.682																
														IGLIHGHQVIPWGDMASLALLQR	10	4	1	N-Term(iTRAQ8plex)	1.417	2.803	2.682	5.51	39	0	2829.58371	3	943.8657837	-1.284103008	54.34829
														LVLVLGDLHIPHR	8	6	1	N-Term(iTRAQ8plex)	0.828	1.55	1.423	5.47	56	0	1786.09275	3	596.0357666	-4.352934822	52.43962167
														MLVLVLGDLHIPHR	6	2	1	N-Term(iTRAQ8plex)	-	-	-	3.68	45	0	1917.13222	4	480.0385132	-4.589378615	59.28482833
7706495	DNAJB11	dnaJ homolog subfamily B member 11 precursor	NP_057390.1	12.57	3	3	10	358	40.5	6.18	1.097	0.748	0.712																
														DGMEYPFIGEGEPHVDGEPGDLR	6	1	1	N-Term(iTRAQ8plex)	1	0.817	0.61	6.3	56	0	2820.29862	3	940.7710571	-3.660308598	58.877425
														FQMTQEVVCDECPNVK	3	1	1	N-Term(iTRAQ8plex); C9(Methylthio); C12(Methylthio); K16(iTRAQ8plex)	0.951	0.803	0.787	5.81	38	0	2570.19999	3	857.4048462	-1.648889505	41.70387167
														ILGVPR	1	1	1	N-Term(iTRAQ8plex)	1.312	0.675	0.684	-	15	0	958.63481	2	479.8210449	-0.321369771	23.56795
7706497	CMPK1	UMP-CMP kinase isoform a	NP_057392.1	12.72	3	3	8	228	25.8	7.97	1.656	5.070	2.472																
														SVDEVFDEVVQIFDK	2	2	1	N-Term(iTRAQ8plex); K15(iTRAQ8plex)	-	-	-	4.71	55	0	2377.27494	3	793.0964966	0.065283191	62.63629

														SVDEVFDEVVQIFDKEG	4	2	1	N-Term(iTRAQ8plex); K15(iTRAQ8plex)	1.656	5.07	2.472	4.63	86	1	2563.34648	2	1282.17688	2.977297714	63.90148833
														IVPVEITISLLK	2	1	1	N-Term(iTRAQ8plex); K12(iTRAQ8plex)	-	-	-	3.65	38	0	1933.24784	3	645.0874634	-4.119706294	57.62414333
7706735	GTF2A1	transcription initiation factor IIA subunit 1 isoform 1	NP_056943.1	2.93	1	1	2	376	41.5	4.55	1.051	3.956	2.993															
														SVIEDVINDVR	2	1	1	N-Term(iTRAQ8plex)	1.051	3.956	2.993	3.9	43	0	1562.87688	2	781.9420776	4.92511825	43.871455
7710145	MBD2	methyl-CpG-binding domain protein 2 testis-specific isoform	NP_056647.1	5.30	1	1	1	302	31.7	10.78	0.813	0.793	0.628															
														REPVPFPSGSAGPGPR	1	2	1	N-Term(iTRAQ8plex)	0.813	0.793	0.628	3.13	-	1	1912.03104	3	638.0151978	-1.716127099	20.55192333
8392875	CFAP20	cilia- and flagella-associated protein 20	NP_037374.1	11.92	2	2	2	193	22.8	9.76	0.856	0.908	0.964															
														AYGTNYIETLR	1	1	1	N-Term(iTRAQ8plex)	0.926	1.032	1.108	2.96	-	0	1604.86772	2	802.9375	5.696469477	30.77792333
														LYSEDELPAEFK	1	1	1	N-Term(iTRAQ8plex); K12(iTRAQ8plex)	0.791	0.8	0.839	-	29	0	2049.08146	3	683.6986694	-9.090048213	28.87950833
8393638	F11R	junctional adhesion molecule A precursor	NP_058642.1	20.74	4	4	11	299	32.6	7.90	1.055	0.502	0.309															
														VTFLPTGITFK	5	1	1	N-Term(iTRAQ8plex); K11(iTRAQ8plex)	0.937	0.502	0.309	3.98	46	0	1832.11924	3	611.3779297	2.742459335	40.79319
														AVLTCSEQDGSPPSEYTWFK	3	1	1	N-Term(iTRAQ8plex); C5(Methylthio); K20(iTRAQ8plex)	0.952	0.777	0.482	3.68	36	0	2899.41704	2	1450.212158	2.32373824	42.63857333
														LIVLVPPSKPTVNIPSSATIGNR	2	1	1	N-Term(iTRAQ8plex); K9(iTRAQ8plex)	1.377	0.384	0.169	3.23	21	0	2981.78519	3	994.5999146	-4.041504296	39.38152667
														ITASYEDR	1	1	1	N-Term(iTRAQ8plex)	1.055	0.451	0.216	-	25	0	1258.65703	2	629.8321533	-0.843183925	17.38566167
8922601	ARL8B	ADP-ribosylation factor-like protein 8B	NP_060654.1	8.60	1	1	2	186	21.5	8.43	-	-	-															
														GVNAIVYMIDAADREK	2	1	1	N-Term(iTRAQ8plex); K16(iTRAQ8plex)	-	-	-	3.12	35	1	2373.30849	4	594.0825806	1.171818006	54.15461167
8922794	IMP3	U3 small nucleolar ribonucleoprotein protein IMP3	NP_060755.1	36.41	4	4	9	184	21.8	9.50	1.300	0.868	0.591															
														QVDFLNWEVTDHNLHELR	3	1	1	N-Term(iTRAQ8plex)	0.71	1.252	0.47	6.17	39	0	2569.31284	3	857.1091309	1.046205073	39.96127333
														ASAALLDKLYALGLVPTR	2	1	1	N-Term(iTRAQ8plex); K8(iTRAQ8plex)	-	-	-	4.8	28	1	2480.50698	4	620.8822021	0.345660689	60.01572333
														MAQHLQAAVAFVEQGHVR	3	1	1	N-Term(iTRAQ8plex)	-	-	0.481	4.55	54	0	2296.23037	4	574.8130493	0.734284177	44.65879167
														SMEDFVTWVDSSK	1	1	1	N-Term(iTRAQ8plex); K13(iTRAQ8plex)	1.363	0.601	0.726	3.06	-	0	2139.0842	3	713.699585	-2.187696578	71.84748667
8923001	ABHD10	mycophenolic acid acyl-glucuronide esterase, mitochondrial isoform 1 precursor	NP_060864.1	15.03	3	3	8	306	33.9	8.57	1.234	0.795	1.080															
														EAEHHCLLHSPIPVNCPIR	4	1	1	N-Term(iTRAQ8plex); C6(Methylthio); C16(Methylthio)	1.07	0.898	1.104	5.01	35	0	2561.27797	3	854.4208984	-2.741334346	37.48440333
														VLSTDVDVILR	2	1	1	N-Term(iTRAQ8plex)	1.513	0.703	0.725	3.15	38	0	1533.90947	2	767.458374	-3.865855995	46.077615
														ADIQLLVYTIDDLIDK	2	1	1	N-Term(iTRAQ8plex); K16(iTRAQ8plex)	-	-	-	3.11	32	0	2456.42301	3	819.479187	4.940714656	67.713375
8923417	ADPRHL2	poly(ADP-ribose) glycohydrolase ARH3	NP_060295.1	4.41	1	1	1	363	38.9	5.07	1.091	0.955	0.832															
														HVQSLEPDPGTPGSER	1	1	1	N-Term(iTRAQ8plex)	1.091	0.955	0.832	4.34	-	0	2010.01773	3	670.6774292	-0.849222529	16.53343
8923421	SARS2	serine--tRNA ligase, mitochondrial isoform b precursor	NP_060297.1	3.09	1	1	2	518	58.2	8.13	0.567	0.588	0.648															
														ALLANQDSGEVQQDPK	2	1	1	N-Term(iTRAQ8plex); K16(iTRAQ8plex)	0.567	0.588	0.648	4.75	45	0	2321.26664	3	774.427063	4.680382114	24.9613
														IDIIR	1	0	2	N-Term(iTRAQ8plex)	-	-	-	-	22	0	933.601	2	467.3041382	-2.658044296	28.15645333
8923444	NHP2	H/ACA ribonucleoprotein complex subunit 2 isoform a	NP_060308.1	18.95	3	3	6	153	17.2	8.22	1.395	0.905	0.682															
														ADPDGPEAQAEACSGER	2	2	1	N-Term(iTRAQ8plex); C13(Methylthio)	0.9	0.658	0.641	6.04	45	0	2052.90195	3	684.972168	5.576669771	25.50254667
														IKADPDGPEAQAEACSGER	2	2	1	N-Term(iTRAQ8plex); K2(iTRAQ8plex); C15(Methylthio)	1.473	0.905	0.682	4.12	33	1	2598.26383	3	866.7594604	-4.262154683	24.53792167
														NLPYVYIPSK	2	1	1	N-Term(iTRAQ8plex); K10(iTRAQ8plex)	1.395	1.131	0.989	3.56	33	0	1802.06511	2	901.5361938	-1.176552764	32.908335
8923857	EMC3	ER membrane protein complex subunit 3	NP_060917.1	9.58	1	1	1	261	29.9	6.81	0.874	0.818	0.474															
														TEWEALELTDHQWALDDVEEELMAK	1	1	1	N-Term(iTRAQ8plex); K25(iTRAQ8plex)	0.874	0.818	0.474	4.7	-	0	3609.77773	4	903.1998901	-2.85353667	60.55224333
8923900	CMAS	N-acylneuraminate cytidylyltransferase	NP_061156.1	4.84	2	2	11	434	48.3	7.93	1.001	1.020	1.384															
														HLAGVPLIGWVLR	8	1	1	N-Term(iTRAQ8plex)	0.766	1.02	1.8	4.43	60	0	1735.06803	3	579.0275269	-0.27317868	55.48098667
														MEVSVSDK	3	1	1	N-Term(iTRAQ8plex); K8(iTRAQ8plex)	1.143	0.873	1.182	2.84	37	0	1502.81963	2	751.9134521	-9.883836258	27.28288333
8923920	H2AFY2	core histone macro-H2A.2	NP_061119.1	10.22	1	3	23	372	40.0	9.69	0.200	0.608	0.529															
														GVTIASGGVLPR	1	1	1	N-Term(iTRAQ8plex)	0.2	0.608	0.529	-	42	0	1430.85906	2	715.9331665	-2.973676665	27.63815167
														HILLAVANDEELNQLLK	20	15	2	N-Term(iTRAQ8plex); K17(iTRAQ8plex)	-	-	-	7.21	100	0	2541.47806	2	1271.244873	-1.43295352	47.19058333
														AGVIFPVGR	2	15	2	N-Term(iTRAQ8plex)	-	-	-	-	31	0	1219.74358	2	610.3754272	-2.380359171	30.74489
8923930	CISD1	CDGSH iron-sulfur domain-containing protein 1	NP_060934.1	25.93	2	2	7	108	12.2	9.09	1.660	1.382	1.016															
														IVHAFDMEDLGDK	4	2	1	N-Term(iTRAQ8plex); K13(iTRAQ8plex)	1.84	1.382	0.892	6.26	55	0	2098.10355	3	700.0393677	-3.055865692	34.26038333
														IVHAFDMEDLGDK	2	2	1	N-Term(iTRAQ8plex); M7(Oxidation); K13(iTRAQ8plex)	1.55	1.247	1.006	4.75	53	0	2114.10276	3	700.0393677	-3.055865692	34.26038333
														HNEETGDNVGPLIIK	1	2	1	N-Term(iTRAQ8plex); K15(iTRAQ8plex)	1.449	1.661	1.264	3.66	-	0	2244.23636	3	748.7503052	-3.613265872	19.81759
8923942	NOP10	H/ACA ribonucleoprotein complex subunit 3	NP_061118.1	20.31	1	1	6	64	7.7	9.99	1.470	1.175	0.892															
														MFLQYYLNEQGDR	6	1	1	N-Term(iTRAQ8plex)	1.47	1.175	0.892	4.64	58	0	1980.99114	2	990.9992065	6.060181551	41.79509833
9257257	WDR1	WD repeat-containing protein 1 isoform 1	NP_059830.1	17.16	5	5	14	606	66.2	6.65	0.894	1.250	1.976															
														CFSIDNPGYEPEVVAVHPGGDTVAIGGVDGNVR	2	3	1	N-Term(iTRAQ8plex); C1(Methylthio)	0.894	1.279	3.157	7.68	37	0	3690.8115	3	1230.942017	7.576722548	41.75463667
														NIDNPALADIYTEHAHQVVAK	2	2	1	N-Term(iTRAQ8plex); K22(iTRAQ8plex)	-	1.182	2.494	6.72	41	0	3026.63833	4	757.4150391	-4.52149498	33.31913
														LATGSDDNCAAFFEGPPFK	6	2	1	N-Term(iTRAQ8plex); C9(Methylthio); K19(iTRAQ8plex)	0.833	1.841	1.566	5.78	52	0	2641.27182	2	1321.150024	1.521947195	49.67474667
														LYSILGTTLKDEGK	2	3	1	N-Term(iTRAQ8plex); K10(iTRAQ8plex); K14(iTRAQ8plex)	-	-	-	4.52	23	1	2450.4645	4	613.371582	0.472132937	46.97412333
														YAPSGFYIASGDVSGK	2	2	1	N-Term(iTRAQ8plex); K16(iTRAQ8plex)	0.897	0.728	0.779	2.69	39	0	2227.17038	3	743.0616455	-6.805957315	26.02976
9506859	TOMM7	mitochondrial import receptor subunit TOM7 homolog	NP_061932.1	10.91	1	1	8	55	6.2	10.29	1.689	0.705	0.674															
														LQQLFK	8	1	1	N-Term(iTRAQ8plex); K6(iTRAQ8plex)	1.689	0.705	0.674	3.26	30	0	1384.87772	3	462.2974243	0.338224243	34.53798333
9845502	RPSA	40S ribosomal protein SA isoform 1	NP_002286.2	44.75	10	10	76	295	32.8	4.87	1.067	2.124	2.066															
														ADHQPLTEASYVNLPTIALCNTDSPLR	31	2	1	N-Term(iTRAQ8plex); C20(Methylthio)	0.756	1.03	1.586	6.73	54	0	3289.6373	3	1097.22522	3.393550074	49.67952833
														FLAAGTHLGGTNLDFQMEQYIYK	5	2	1	N-Term(iTRAQ8plex); K23(iTRAQ8plex)	-	-	-	6.56	54	0	3225.68479	3	1075.89978	-0.484774487	51.57431167
														AIVAIENPADVSVISSR	8	2	1	N-Term(iTRAQ8plex)	1.138	1.575	1.466	4.85	77	0	2045.14507	2	1023.076172	-4.608069203	39.963365
														GAHSVGLMWWMLAR	5	2	1	N-Term(iTRAQ8plex); M8(Oxidation)	1.113	1.419	1.388	4.52	50	0	1934.9943	2	968.0046387	-0.803914544	41.58840833
														FTPGTFTNQIQAAFR	2	2	1	N-Term(iTRAQ8plex)	1.329	2.124	2.145	4.38	59	0	2003.07927	2	1002.043274	6.994197266	45.44575667
														GAHSVGLMWWMLAR	4	2	1	N-Term(iTRAQ8plex); M8(Oxidation); M11(Oxidation)	1.107	2.252	1.894	4.29	27	0	1950.99112	2	968.0046387	-0.803914544	41.58840833
														FLAAGTHLGGTNLDFQMEQYIYK	2	2	1	N-Term(iTRAQ8plex); M17(Oxidation); K23(iTRAQ8plex)	-	-	-	3.89	32	0	3241.68569	3	1075.89978	-0.484774487	51.57431167
														FAAATGATPIAGR	4	2	1	N-Term(iTRAQ8plex)	1.016	2.169	2.343	3.88	47	0	1507.8488	2	754.4280396	-3.102975862	26.072055
														YVDIAIPCNNK	8	2	1	N-Term(iTRAQ8plex); C8(Methylthio); K11(iTRAQ8plex)	1.04	2.744	2.708	3.75	42	0	1904.00589	2	952.5065308	-9.053226991	30.148715
														FTPGTFTNQIQAAFREPR	1	2	1	N-Term(iTRAQ8plex)	-	-	-	3.22	-	1	2385.25649	4	597.0695801	-2.205577271	46.07594667
														FLAAGTHLGGTNLDFQMEQYIYKR	1	2	1	N-Term(iTRAQ8plex); K23(iTRAQ8plex)	1.067	2.689	2.287	3.15	-	1	3381.78945	4	846.2028198	0.584932003	47.05558167
														LLVVTDPR	3	2	1	N-Term(iTRAQ8plex)	1.131	3.082	2.915	-	54	0	1216.75054	2	608.8789063	-5.067119198	30.41884
														ADHQPLTEASYVNLPTIALCNTDSPLR	2	2	1	N-Term(iTRAQ8plex); Y11(iTRAQ8plex); C20(Methylthio)	-	-	-	-	28	0	3593.84829	3	1097.22522	3.393550074	49.67952833
														ILLAAR	2	1	1	N-Term(iTRAQ8plex)	0.865	2.036	1.674	-	24	0	960.64195	2	480.8246155	-9.178198942	19.83922833
9845511	RAC1	ras-related C3 botulinum toxin substrate 1 isoform Rac1	NP_008839.2	29.69	4	5	36	192	21.4	8.50	2.430	1.186	1.244															
														LTPITYPQGLAMAK	2	2	1	N-Term(iTRAQ8plex); K14(iTRAQ8plex)	2.943	0.861	1.314	5.6	48	0	2112.23545	3	704.75	0.329248333	35.51804667
														HHCPNTPIILVGTK	4	2	1	N-Term(iTRAQ8plex); C3(Methylthio); K14(iTRAQ8plex)	2.896	2.026	1.836	5.3	42	0	2184.20391	2	1092.605591	-9.28818016	24.015855
														YLECSALTQR	20	4	1	N-Term(iTRAQ8plex); C4(Methylthio)	2.139	1.25	1.248	5.04	56	0	1533.76325	3	511.9304199	3.91466085	35.14545833
														TVFDEAIR	4	4	1	N-Term(iTRAQ8plex)	2.935	1.032	1.122	-	35	0	1254.69524	2	627.8512573	-3.461614766	27.50658833
														CVVVGDGAVGK	6	16	3	N-Term(iTRAQ8plex); C1(Methylthio); K11(iTRAQ8plex)	-	-	-	4.67	48	0	1657.90886	2	829.4603271	-5.563995833	21.63299833
9910346	EMC7	ER membrane protein complex subunit 7 precursor	NP_064539.1	11.98	2	2	3	242	26.5	9.25	1.360	0.711	0.675															
														VLVDGEEHVGFLK	1	1	1	N-Term(iTRAQ8plex); K13(iTRAQ8plex)	1.386	0.824	0.83	3.22	-	0	2050.17569	3	684.0634155	-1.784221704	32.24715167
														AVVPGVKPQDWISAAR	2	1	1	N-Term(iTRAQ8plex); K7(iTRAQ8plex)	1.333	0.613	0.549	2.94	27	0	2302.34292	4	576.3411865	-2.742895598	31.82949167
9910382	TOMM22	mitochondrial import receptor subunit TOM22 homolog	NP_064628.1	33.80	3	3	19	142	15.5	4.34	1.807	0.756	0.570															
														LQMEQQQQLQQR	2	1	1	N-Term(iTRAQ8plex)	1.855	0.652	0.441	4.85	61	0	1861.98918	2	931.49823	1.913029877	23.628685
														LWGLTEMFPER	16	1	1	N-Term(iTRAQ8plex)	1.797	0.785	0.586	4.68	44	0	1682.88799	2	841.9493408	2.129502007	53.85597
														GDAEKPEEELEEDDDEELDETLSER	1	1	1	N-Term(iTRAQ8plex); K5(iTRAQ8plex)	-	-	-	3.13	-	0	3529.62319	4	883.1612549	-1.555873943	33.51450833
9966764	LYPLA2	acyl-protein thioesterase 2	NP_009191.1	11.26	2	2	5	231	24.7	7.23	0.867	1.886	1.525															
														DLAILQCHGELDPMVPVR	4	3	1	N-Term(iTRAQ8plex); C7(Methylthio)	0.867	1.301	1.193	4.83	41	0	2356.21671	3	786.0770874	1.585871544	46.14923667
														YICPHAPR	1	3	1	N-Term(iTRAQ8plex); Y1(iTRAQ8plex); C3(Methylthio)	-	2.232	1.948	-	35	0	1610.87563	4	403.4743652	0.048891112	18.91417667
9966799	UTP3	something about silencing protein 10	NP_065101.1	5.43	2	2	4	479	54.5	5.62	1.620	0.722	0.970															
														RVPAHGHPVIER	2	1	1	N-Term(iTRAQ8plex)	1.62	0.722	0.97	3.13	28	1	1671.96659	3	557.9937134	-2.580442286	10.80667667
														KESPELLELIEDLK	2	1	1	N-Term(iTRAQ8plex); K1(iTRAQ8plex); K14(iTRAQ8plex)	-	-	-	2.76	27	1	2568.53725	4	642.8897705	4.240098617	56.06668
9966805	DDX24	ATP-dependent RNA helicase DDX24	NP_065147.1	10.24	6	6	16	859	96.3	9.06	2.003	1.682	1.355															
														VLDIMPLTLHACMHQK	1	1	1	N-Term(iTRAQ8plex); C12(Methylthio); K16(iTRAQ8plex)	-	1.741	2.052	4.92	-	0	2504.34121	4	626.8407593	-2.337968408	54.78481333
														LEDCVLLATDVAAR	2	1	1	N-Term(iTRAQ8plex); C4(Methylthio)	2.003	1.661	1.355	4.41	58	0	1838.96269	2	919.9849854	-1.701076854	66.87079167
														RPLLGLVLTPTR	6	1	1	N-Term(iTRAQ8plex)	3.172	2.197	2.807	3.96	44	0	1640.04599	3	547.3535156	-3.97408062	35.94009167
														TLAFAIPMIHAVLQWQKR	4	1	1	N-Term(iTRAQ8plex); K17(iTRAQ8plex)	-	1.663	0.951	3.82	26	1	2731.59658	4	683.6546021	-3.255596725	63.55903333
														GHFAELSQLLEMLNDSQYNPK	2	1	1	N-Term(iTRAQ8plex); K21(iTRAQ8plex)	-	-	-	3.11	38	0	3042.57749	3	1014.864014	-1.335556577	60.877125
														LDLLMQK	1	1	1	N-Term(iTRAQ8plex); K7(iTRAQ8plex)	1.753	1.682	1.214	-	30	0	1468.90409	3	490.3062134	1.5764043	62.439525
9966849	TIGAR	fructose-2,6-bisphosphatase TIGAR	NP_065108.1	10.37	2	2	4	270	30.0	7.69	0.583	2.038	3.207															
														IIQGQGVDEPLSETGFK	2	1	1	N-Term(iTRAQ8plex); K17(iTRAQ8plex)	0.404	2.085	3.119	4.94	83	0	2426.34331	2	1213.675293	1.86618995	34.01117167
														SLFDYFLTDLK	2	1	1	N-Term(iTRAQ8plex); K11(iTRAQ8plex)	0.842	1.992	3.297	4.5	29	0	1970.10312	3	657.3725586	-3.24286007	52.16215833
10047140	PNO1	RNA-binding protein PNO1	NP_064528.1	11.90	2	2	4	252	27.9	9.73	1.205	0.819	1.017															
														QAEQLSAAGEGGDAGR	2	1	1	N-Term(iTRAQ8plex)	1.222	0.658	0.837	5.33	64	0	1820.9011	3	607.6385498	-1.644864701	18.65035167
														TAICNLILGNPPSK	2	1	1	N-Term(iTRAQ8plex); C4(Methylthio); K14(iTRAQ8plex)	1.187	1.019	1.237	4.66	55	0	2095.18595	3	699.0668335	-0.226739645	44.49013333
10092657	NDUFA12	NADH dehydrogenase [ubiquinone] 1 alpha subcomplex subunit 12 isoform a	NP_061326.1	11.72	1	1	2	145	17.1	9.63	1.384	1.704	1.394															
														NTFWDVDGSMVPPEWHR	2	1	1	N-Term(iTRAQ8plex)	1.384	1.704	1.394	4.97	58	0	2377.1301	3	793.0482178	-1.54860253	42.64854
10190712	S100A14	protein S100-A14	NP_065723.1	44.23	3	3	9	104	11.7	5.24	0.230	0.787	0.057															
														SANAEDAQEFSDVER	3	1	1	N-Term(iTRAQ8plex)	0.236	0.654	0.069	5.89	75	0	1971.93035	2	986.468811	5.35219225	24.48769
														SFWELIGEAAK	4	1	1	N-Term(iTRAQ8plex); K11(iTRAQ8plex)	0.221	0.787	0.045	5	71	0	1859.04729	2	930.0272827	-2.723163818	72.97836
														DLVTQQLPHLMPSNCGLEEK	2	1	1	N-Term(iTRAQ8plex); M11(Oxidation); C15(Methylthio); K20(iTRAQ8plex)	0.457	0.964	0.194	4.14	-	0	2922.49428	4	731.3790283	-1.4179322	37.67023667
10190714	RAB22A	ras-related protein Rab-22A	NP_065724.1	5.67	1	1	2	194	21.8	8.15	0.996	0.567	0.528															
														FLIWDTAGQER	2	4	1	N-Term(iTRAQ8plex)	0.996	0.567	0.528	3.85	25	0	1639.88017	2	820.4437256	3.398572628	41.12259167
10334859	CKMT1B	creatine kinase U-type, mitochondrial precursor	NP_066270.1	27.58	8	8	23	417	47.0	8.34	0.293	0.375	0.379															
														TTPTGWTLDQCIQTGVDNPGHPFIK	3	4	1	N-Term(iTRAQ8plex); C11(Methylthio); K25(iTRAQ8plex)	0.159	-	-	5.1	40	0	3380.72898	3	1127.581177	1.817573511	49.26512
														GTGGVDTAATGGVFDISNLDR	2	4	1	N-Term(iTRAQ8plex)	0.582	0.532	0.456	3.87	32	0	2327.17398	3	776.3961792	-1.794860364	40.03806833

														SFLIWVNEEDHTR	8	4	1	N-Term(iTRAQ8plex)	0.552	0.733	0.604	3.86	46	0	1950.00041	2	975.5038452	-0.978199953	39.08578
														VVVDALSGLK	2	4	1	N-Term(iTRAQ8plex); K10(iTRAQ8plex)	0.24	0.161	-	3.66	39	0	1609.01053	3	537.0083618	-2.466796023	33.315445
														LYPPSAEYPDLR	1	1	1	N-Term(iTRAQ8plex)	0.433	0.646	0.701	3.48	-	0	1724.91716	2	862.9622192	0.609047545	29.93129667
														LGYILTCPSNLGTGLR	4	5	1	N-Term(iTRAQ8plex); C7(Methylthio)	0.174	0.077	0.112	3.47	50	0	2028.09233	2	1014.549805	-0.034290845	58.99410667
														IPTPVIHTK	1	4	1	N-Term(iTRAQ8plex); K9(iTRAQ8plex)	0.293	0.097	0.093	3.11	-	0	1614.01828	3	538.6776123	-1.002241396	22.284065
														GWEFMWNER	2	5	1	N-Term(iTRAQ8plex)	0.234	0.143	0.087	3.03	28	0	1558.73711	2	779.8721924	-2.805575329	64.89739333
10716563	CANX	calnexin precursor	NP_001737.1	35.81	20	20	152	592	67.5	4.60	1.261	0.680	0.716																
														IVDDWANDGWGLK	4	2	1	N-Term(iTRAQ8plex); K13(iTRAQ8plex)	1.384	0.553	0.667	6.11	73	0	2097.10942	2	1049.05835	-6.269916633	69.70336
														IVDDWANDGWGLKK	10	2	1	N-Term(iTRAQ8plex); K13(iTRAQ8plex); K14(iTRAQ8plex)	1.817	0.708	0.742	5.23	38	1	2529.41672	3	843.8057861	-7.945018295	38.88826167
														TPELNLDQFHDK	16	2	1	N-Term(iTRAQ8plex); K12(iTRAQ8plex)	1.198	0.48	0.551	5.07	51	0	2065.11553	2	1033.058105	-4.129071526	26.38298
														KIPNPDFFEDLEPFR	53	2	1	N-Term(iTRAQ8plex); K1(iTRAQ8plex)	1.436	0.736	0.677	4.9	50	1	2472.33584	4	618.8394165	-1.033502139	51.73066
														CESAPGCGVWQRPVIDNPNYK	4	2	1	N-Term(iTRAQ8plex); C1(Methylthio); C7(Methylthio); K21(iTRAQ8plex)	1.71	1.114	0.69	4.86	-	0	3033.46132	4	759.1207886	-1.677582992	36.411065
														TPYTIMFGPDK	4	2	1	N-Term(iTRAQ8plex); K11(iTRAQ8plex)	1.286	0.682	0.814	4.76	85	0	1878.02263	2	939.5149536	-3.47743064	51.63770833
														GTLSGWILSK	10	2	1	N-Term(iTRAQ8plex); K10(iTRAQ8plex)	1.268	0.656	0.717	4.26	54	0	1670.00701	2	835.5071411	-1.638132911	35.71943667
														SDAEEDGGTVSQEEEDRKPK	1	2	1	N-Term(iTRAQ8plex); K18(iTRAQ8plex); K20(iTRAQ8plex)	1.288	-	1.958	4	-	1	3118.58442	5	624.5227051	-1.918312468	14.11883
														APVPTGEVYFADSFDR	12	2	1	N-Term(iTRAQ8plex)	1.606	0.687	0.827	3.95	78	0	2075.03226	2	1038.023804	0.746733378	41.23584333
														LNKPFLFDTKPLIVQYEVNFQNGIECGGAYVK	1	2	1	N-Term(iTRAQ8plex); K3(iTRAQ8plex); K10(iTRAQ8plex); C26(Methylthio); [illegible]	-	-	-	3.84	-	0	4907.69985	4	1227.68042	1.709050626	62.90185833
														CGEDYKLHFIFR	2	3	1	N-Term(iTRAQ8plex); C1(Methylthio); K6(iTRAQ8plex)	4.116	2.542	2.103	3.5	-	1	2182.131	4	546.288208	-4.028240624	38.34699167
														WEVEEMK	3	2	1	N-Term(iTRAQ8plex); K7(iTRAQ8plex)	1.176	0.611	0.509	3.05	27	0	1558.84201	3	520.2855225	1.567081712	23.36690667
														TYFTDKK	1	2	1	N-Term(iTRAQ8plex); K6(iTRAQ8plex); K7(iTRAQ8plex)	1.934	0.79	0.842	3.04	-	1	1815.07976	2	908.0435181	1.018254324	16.47638833
														TPELNLDQFHDKTPYTIMFGPDK	1	2	1	N-Term(iTRAQ8plex); K12(iTRAQ8plex); K23(iTRAQ8plex)	-	-	-	3.03	-	1	3619.91621	5	724.7890625	-1.993225696	45.314855
														EIEDPEDRKPEDWDERPK	1	2	1	N-Term(iTRAQ8plex); K9(iTRAQ8plex); K18(iTRAQ8plex)	2.707	0.486	0.704	2.75	-	1	3195.68662	3	1065.900391	5.63801372	21.12521167
														LHFIFR	24	3	1	N-Term(iTRAQ8plex)	0.878	0.73	0.801	-	33	0	1136.68155	3	379.5653687	-5.857922963	33.097085
														IPNPDFFEDLEPFR	1	2	1	N-Term(iTRAQ8plex)	1.07	1.119	0.618	-	30	0	2040.04228	2	1020.52478	2.069872232	58.84669
														EIEDPEDR	2	2	1	N-Term(iTRAQ8plex)	1.146	0.457	0.582	-	29	0	1306.63774	2	653.8225098	-3.917220957	13.82333
														KDDTDDEIAK	1	2	1	N-Term(iTRAQ8plex); K1(iTRAQ8plex); K10(iTRAQ8plex)	1.255	0.577	0.553	-	27	1	2062.15464	3	688.0563965	5.579069059	16.46206833
														GLVLMSR	1	2	1	N-Term(iTRAQ8plex)	1.203	0.719	0.78	-	26	0	1079.65288	2	540.3300781	-1.853994036	25.983925
10835049	RHOA	transforming protein RhoA isoform 1 precursor	NP_001655.1	37.31	5	7	33	193	21.8	6.10	0.873	1.515	1.359																
														HFCPNVPIILVGNKK	8	5	1	N-Term(iTRAQ8plex); C3(Methylthio); K14(iTRAQ8plex); K15(iTRAQ8plex)	0.884	1.738	1.451	5.11	29	1	2637.53614	4	660.1397705	-4.841694175	35.10593667
														IGAFGYMECSAK	2	4	1	N-Term(iTRAQ8plex); C9(Methylthio); K12(iTRAQ8plex)	0.87	1.92	1.318	4.58	56	0	1930.95146	2	965.9793701	-8.855057181	66.96553333
														LVIVGDGACGK	2	6	1	N-Term(iTRAQ8plex); C9(Methylthio); K11(iTRAQ8plex)	0.873	1.434	1.359	4.04	44	0	1685.94524	2	843.4762573	-5.139118025	47.27924
														HFCPNVPIILVGNK	1	5	1	N-Term(iTRAQ8plex); C3(Methylthio); K14(iTRAQ8plex)	0.847	1.36	1.1	2.89	-	0	2205.23427	4	552.0640259	-6.991848693	40.29661833
														EVFEMATR	5	5	1	N-Term(iTRAQ8plex)	0.929	1.29	1.205	2.83	25	0	1286.66545	2	651.8347168	-3.392534454	20.55433833
														EVFEMATR	1	5	1	N-Term(iTRAQ8plex); M5(Oxidation)	0.93	1.286	1.141	-	25	0	1302.66216	2	651.8347168	-3.392534454	20.55433833
														QVELALWDTAGQEDYDR	10	4	2	N-Term(iTRAQ8plex)	-	-	-	5.56	85	0	2313.14653	2	1157.076904	7.094524071	39.93450167
														TCLLIVFSK	4	7	2	N-Term(iTRAQ8plex); C2(Methylthio); K9(iTRAQ8plex)	-	-	-	3.65	34	0	1677.97356	2	839.4904175	-9.323126091	75.76786333
10835067	SSB	lupus La protein	NP_003133.1	19.12	6	6	34	408	46.8	7.12	0.814	3.042	3.007																
														GSIFVVFDSIESAK	5	1	1	N-Term(iTRAQ8plex); K14(iTRAQ8plex)	0.669	4.376	4.311	4.79	59	0	2107.17734	3	703.0639648	-5.808727396	76.20962667
														LTTDFNVIVEALSK	18	1	1	N-Term(iTRAQ8plex); K14(iTRAQ8plex)	0.814	3.112	3.064	4.36	50	0	2158.25395	2	1079.630615	-1.870702585	56.457905
														NKEVTWEVLEGEVEK	2	1	1	N-Term(iTRAQ8plex); K2(iTRAQ8plex); K15(iTRAQ8plex)	0.785	2.35	2.05	4.35	49	1	2701.49082	3	901.168457	-9.91062271	34.69063333
														LDEGWVPLEIMIK	6	1	1	N-Term(iTRAQ8plex); K13(iTRAQ8plex)	1.351	3.145	3.095	4.2	65	0	2151.24419	2	1076.125732	4.530830951	56.17096167
														EQIKLDEGWVPLEIMIK	2	1	1	N-Term(iTRAQ8plex); K4(iTRAQ8plex); K17(iTRAQ8plex)	0.7	2.166	1.898	3.68	33	1	2953.71219	3	985.2422485	-2.650544136	52.992845
														GFPTDATLDDIKEWLEDK	1	1	1	N-Term(iTRAQ8plex); K12(iTRAQ8plex); K18(iTRAQ8plex)	0.87	4.023	3.775	2.99	-	1	3005.60959	3	1002.541382	-4.637675573	60.75406
10835073	NMT1	glycylpeptide N-tetradecanoyltransferase 1	NP_066565.1	11.29	3	3	7	496	56.8	7.80	0.548	1.037	1.186																
														AAYSFYNVHTQTPLLDLMSDALVLAK	2	2	1	N-Term(iTRAQ8plex); K26(iTRAQ8plex)	-	-	-	4.91	23	0	3489.91372	4	873.2338867	6.43159024	67.97955
														DIPVVHQLLTR	4	2	1	N-Term(iTRAQ8plex)	0.548	1.037	1.186	4.58	54	0	1594.95371	3	532.3227539	-2.854522502	31.38864333
														LGEVVNTHGPVEPDKDNIR	1	2	1	N-Term(iTRAQ8plex); K15(iTRAQ8plex)	-	-	-	3.19	-	1	2697.46303	4	675.1212158	-5.578537523	25.39579833
10835159	SERPINE1	plasminogen activator inhibitor 1 precursor	NP_000593.1	10.95	3	3	5	402	45.0	7.20	0.427	0.519	1.137																
														EVPLSALTNILSAQLISHWK	2	2	1	N-Term(iTRAQ8plex); K20(iTRAQ8plex)	-	-	-	5.16	38	0	2828.65371	4	707.9188843	1.490436728	66.198635
														LVQGFMPHFFR	2	2	1	N-Term(iTRAQ8plex); M6(Oxidation)	0.487	0.61	1.105	3.24	35	0	1698.90195	3	566.972168	-4.276670438	36.69347333
														KPLENLGMTDMFR	1	2	1	N-Term(iTRAQ8plex); K1(iTRAQ8plex)	0.374	0.441	1.169	2.96	-	0	2160.16294	3	720.7258301	-6.330224166	36.19235833
10835165	CD59	CD59 glycoprotein preproprotein	NP_000602.1	27.34	5	5	48	128	14.2	6.48	0.706	0.391	0.450																
														ENELTYYCCK	2	1	1	N-Term(iTRAQ8plex); C8(Methylthio); C9(Methylthio); K10(iTRAQ8plex)	0.666	0.266	0.27	3.99	30	0	1965.90268	3	655.9724121	-0.605432754	67.00515667
														FEHCNFNDVTTR	25	1	1	N-Term(iTRAQ8plex); C4(Methylthio)	0.539	0.413	0.478	3.96	51	0	1832.8333	2	916.9197388	-2.166800068	26.80942
														LRENELTYYCCK	15	1	1	N-Term(iTRAQ8plex); C10(Methylthio); C11(Methylthio); K12(iTRAQ8plex)	0.863	0.265	0.282	3.83	28	1	2235.08841	3	745.6998901	-1.76554343	42.054905
														AGLQVYNK	4	1	1	N-Term(iTRAQ8plex); K8(iTRAQ8plex)	0.961	0.337	0.605	3.46	32	0	1500.90241	3	500.9723206	1.97689064	20.39787833
														CWKFEHCNFNDVTTR	2	1	1	N-Term(iTRAQ8plex); C1(Methylthio); K3(iTRAQ8plex); C7(Methylthio)	1.633	0.398	0.45	2.99	-	1	2600.21474	4	650.8091431	0.772661424	44.99800167
10835230	MT1G	metallothionein-1G isoform 1	NP_005941.1	32.79	2	2	28	61	6.1	7.96	0.499	2.408	2.556																
														SCCSCCPVGCAK	17	6	1	N-Term(iTRAQ8plex); C2(Methylthio); C3(Methylthio); C5(Methylthio); [illegible]	0.391	2.114	2.373	4.51	80	0	1998.73406	2	999.8724365	-7.16946194	41.51840667
														CAQGCICK	11	6	1	N-Term(iTRAQ8plex); C1(Methylthio); C5(Methylthio); C7(Methylthio); [illegible]	0.566	2.483	2.993	3.71	44	0	1571.70415	2	786.3557129	-7.224730402	29.25328333
10863945	XRCC5	X-ray repair cross-complementing protein 5	NP_066964.1	40.16	19	19	169	732	82.7	5.81	1.388	2.010	2.018																
														ETVYCLNDDDETEVLKEDIIQGFR	15	1	1	N-Term(iTRAQ8plex); C5(Methylthio); K16(iTRAQ8plex)	2.284	2.975	2.678	5.66	42	1	3498.72324	4	875.4349976	-1.372487124	61.695135
														ANPQVGVAFPHIK	15	1	1	N-Term(iTRAQ8plex); K13(iTRAQ8plex)	1.305	1.885	1.719	5.52	70	0	1986.15996	2	993.5836182	-7.336565116	28.10070167
														YAPTEAQLNAVDALIDSMSLAK	4	1	1	N-Term(iTRAQ8plex); K22(iTRAQ8plex)	-	-	-	5.48	75	0	2929.57633	3	977.1969604	-1.311862847	64.48988833
														EPLPPIQQHIWNMLNPPAEVTTK	13	1	1	N-Term(iTRAQ8plex); K23(iTRAQ8plex)	2.38	4.931	3.371	5.13	35	0	3261.79245	3	1087.935669	0.288885927	51.14169167
														IQPGSQQADFLDALIVSMDVIQHETIGK	6	1	1	N-Term(iTRAQ8plex); K28(iTRAQ8plex)	-	-	-	4.92	81	0	3661.96718	4	916.2472534	-1.337398147	70.27807
														ASFEEASNQLINHIEQFLDTNETPYFMK	4	1	1	N-Term(iTRAQ8plex); K28(iTRAQ8plex)	-	-	-	4.85	41	0	3924.9697	3	1308.994751	3.100248057	69.74418
														HLMLPDFDLLEDIESK	18	1	1	N-Term(iTRAQ8plex); K16(iTRAQ8plex)	1.165	2.94	2.597	4.81	65	0	2523.35869	2	1262.182983	-1.534115281	58.02843
														DQVTAQEIFQDNHEDGPTAK	3	1	1	N-Term(iTRAQ8plex); K20(iTRAQ8plex)	1.59	1.635	1.618	4.76	41	0	2851.42179	3	951.1448364	-4.19274356	25.17000667
														TDTLEDLFPTTK	6	1	1	N-Term(iTRAQ8plex); K12(iTRAQ8plex)	1.138	1.705	1.481	4.71	47	0	1989.08867	2	995.0479736	-5.736624286	32.97777333
														HIEIFTDLSSR	22	1	1	N-Term(iTRAQ8plex)	1.474	1.745	2.022	4.14	53	0	1621.879	2	811.4429932	-3.971685328	27.027775
														LGGHGPSFPLK	2	1	1	N-Term(iTRAQ8plex); K11(iTRAQ8plex)	1.099	1.945	1.633	3.95	28	0	1718.00516	3	573.3399048	-9.208467407	24.61372333
														HLMLPDFDLLEDIESK	7	1	1	N-Term(iTRAQ8plex); M3(Oxidation); K16(iTRAQ8plex)	2.123	2.381	2.172	3.92	37	0	2539.3544	2	1262.182983	-1.534115281	58.02843
														DKPSGDTAAVFEEGGDVDDLLDMI	3	1	1	N-Term(iTRAQ8plex); K2(iTRAQ8plex); M23(Oxidation)	-	1.046	2.438	3.91	68	0	3133.52634	3	1039.855225	3.71743002	53.96969667
														CFSVLGFCK	6	1	1	N-Term(iTRAQ8plex); C1(Methylthio); C8(Methylthio); K9(iTRAQ8plex)	1.346	2.88	2.513	3.89	38	0	1703.85495	3	568.6231689	-3.069342288	81.7221
														LFQCLLHR	29	1	1	N-Term(iTRAQ8plex); C4(Methylthio)	1.298	2.03	2.103	3.87	54	0	1379.75571	3	460.5894165	-4.099310114	55.123965
														DKPSGDTAAVFEEGGDVDDLLDMI	6	1	1	N-Term(iTRAQ8plex); K2(iTRAQ8plex)	2.521	5.363	2.567	3.64	77	0	3117.55112	3	1039.855225	3.71743002	53.96969667
														SQLDIIIHSLK	1	1	1	N-Term(iTRAQ8plex); K11(iTRAQ8plex)	0.877	0.994	1.257	3.61	-	0	1875.13565	3	625.7167358	-8.920437533	39.17679333
														SQLDIIIHSLKK	1	1	1	N-Term(iTRAQ8plex); K11(iTRAQ8plex); K12(iTRAQ8plex)	1.123	1.372	1.157	3.51	-	1	2307.43203	4	577.6134644	-8.963972883	35.89715833
														EPLPPIQQHIWNMLNPPAEVTTK	3	1	1	N-Term(iTRAQ8plex); M13(Oxidation); K23(iTRAQ8plex)	-	-	-	3.5	-	0	3277.79043	3	1087.935669	0.288885927	51.14169167
														TLFPLIEAK	2	1	1	N-Term(iTRAQ8plex); K9(iTRAQ8plex)	1.387	1.721	1.218	3.35	35	0	1640.01194	3	547.3421631	-7.558049984	37.32217833
														RFFMGNQVLK	1	1	1	N-Term(iTRAQ8plex); K10(iTRAQ8plex)	2.203	2.858	2.534	3.17	-	1	1848.05912	3	616.6912231	-9.926693495	25.67121333
														EEASGSSVTAEEAK	2	1	1	N-Term(iTRAQ8plex); K14(iTRAQ8plex)	1.388	2.292	2.443	-	66	0	2003.03203	2	1002.019653	-3.455401247	18.18199
10864011	SQRDL	sulfide:quinone oxidoreductase, mitochondrial	NP_067022.1	47.78	19	19	76	450	49.9	9.11	0.737	0.212	0.250																
														ANIIFNTSLGAIFGVKK	6	1	1	N-Term(iTRAQ8plex); K16(iTRAQ8plex); K17(iTRAQ8plex)	1.093	-	0.285	6.06	59	1	2705.65639	4	677.1695557	3.046017326	56.004855
														ANIIFNTSLGAIFGVK	2	1	1	N-Term(iTRAQ8plex); K16(iTRAQ8plex)	0.737	-	0.171	5.9	53	0	2273.3516	3	758.4553833	1.660698833	54.285155
														HFYQPIWTLVGAGAK	6	1	1	N-Term(iTRAQ8plex); K15(iTRAQ8plex)	0.946	0.42	0.563	5.83	30	0	2296.30239	3	766.1036377	-4.316282617	45.52289667
														VGAENVAIVEPSER	4	1	1	N-Term(iTRAQ8plex)	0.697	0.107	0.217	5.66	67	0	1773.96428	2	887.4857788	-0.344033315	25.21477167
														AEPLETFPFDQSK	2	1	1	N-Term(iTRAQ8plex); K13(iTRAQ8plex)	0.62	0.074	0.119	5.56	52	0	2117.15264	2	1059.079956	7.129476223	34.79141667
														RYPNVFGIGDCTNLPTSK	10	1	1	N-Term(iTRAQ8plex); C11(Methylthio); K18(iTRAQ8plex)	0.628	0.134	0.269	5.51	49	1	2636.38149	3	879.4615479	-3.205921408	40.11193333
														TSPVADAAGWVDVDKETLQHR	5	1	1	N-Term(iTRAQ8plex); K15(iTRAQ8plex)	0.757	0.581	0.416	5.19	34	1	2903.53823	4	726.6400146	-3.100557162	35.959355
														EGNAIFTFPNTPVK	3	1	1	N-Term(iTRAQ8plex); K14(iTRAQ8plex)	0.719	0.264	0.312	4.73	46	0	2143.19023	2	1072.098755	-4.935704825	29.08582333
														YADALQEIIQER	6	1	1	N-Term(iTRAQ8plex)	0.626	0.168	0.273	4.69	67	0	1752.94145	3	584.9873657	2.363229854	45.10503667
														VILAEFDYK	7	1	1	N-Term(iTRAQ8plex); K9(iTRAQ8plex)	0.65	0.233	0.232	4.33	31	0	1705.99443	3	569.3366089	-1.892086041	33.18628833
														IMYLSEAYFR	4	1	1	N-Term(iTRAQ8plex)	0.647	0.11	0.203	4.06	39	0	1596.83721	2	798.9311523	9.554259612	49.58538333
														KVGAENVAIVEPSER	2	1	1	N-Term(iTRAQ8plex); K1(iTRAQ8plex)	0.777	-	0.195	4.05	53	1	2206.2601	3	736.0915527	-2.319013458	21.87126167
														TAAAVAAQSGILDR	3	1	1	N-Term(iTRAQ8plex)	0.845	0.336	0.379	3.84	61	0	1647.93169	2	824.4694824	-0.912098639	30.21654833
														GLPEGFAHPK	4	1	1	N-Term(iTRAQ8plex); K10(iTRAQ8plex)	0.628	0.13	0.192	3.69	42	0	1660.96195	3	554.3255005	-0.712289139	18.989975
														KYDGYTSCPLVTGYNR	2	1	1	N-Term(iTRAQ8plex); K1(iTRAQ8plex); C8(Methylthio)	0.817	0.266	0.332	3.48	28	1	2491.25168	3	831.0887451	-2.131538325	32.12472667
														TISVIMK	1	1	1	N-Term(iTRAQ8plex); K7(iTRAQ8plex)	0.698	0.081	0.136	3.32	-	0	1399.87871	2	700.4429932	-1.13694984	34.07528333
														NLTVNYK	3	1	1	N-Term(iTRAQ8plex); K7(iTRAQ8plex)	0.808	0.234	0.286	3.1	25	0	1459.86811	3	487.29422	-3.270595384	20.40454167
														LSMYLMK	2	1	1	N-Term(iTRAQ8plex); K7(iTRAQ8plex)	0.743	0.075	0.146	3.04	28	0	1493.86969	3	498.6280823	1.11942573	52.99432167
														GYWGGPAFLR	3	1	1	N-Term(iTRAQ8plex)	0.711	0.226	0.288	2.83	27	0	1427.76885	2	714.3880615	-3.435341188	53.39103
														HFYQPIWTLVGAGAK	1	1	1	N-Term(iTRAQ8plex); Y3(iTRAQ8plex); K15(iTRAQ8plex)	1.168	0.442	0.43	-	25	0	2600.50943	3	766.1036377	-4.316282617	45.52289667
10947135	ABCF1	ATP-binding cassette sub-family F member 1 isoform b	NP_001081.1	3.22	2	2	7	807	91.6	7.68	0.829	0.995	1.139																
														GAVIVVSHDAR	6	2	1	N-Term(iTRAQ8plex)	0.837	0.826	1.126	4.68	61	0	1427.8219	3	476.6121521	-3.74574109	15.07281667
														EVLEALGEVMVSRPR	1	2	1	N-Term(iTRAQ8plex)	0.733	1.554	1.172	2.8	-	0	1989.11148	3	663.7086792	0.479132348	49.90731333
11024700	TIMM13	mitochondrial import inner membrane translocase subunit Tim13	NP_036590.1	18.95	2	2	4	95	10.5	8.18	0.763	0.604	0.926																
														YMDAWNTVSR	3	1	1	N-Term(iTRAQ8plex)	0.724	0.638	0.919	3.11	22	0	1546.75432	2	773.8807983	-5.34748163	43.29812167
														CIAMCMDR	1	1	1	N-Term(iTRAQ8plex); C1(Methylthio); C5(Methylthio)	0.763	0.596	0.99	-	18	0	1338.53826	2	669.7727661	-6.744441856	69.48261333
11038643	AP2S1	AP-2 complex subunit sigma isoform AP17delta	NP_067586.1	35.58	3	3	9	104	12.4	5.85	1.447	0.521	1.175																
														LIEEVHAVVTVR	6	7	1	N-Term(iTRAQ8plex)	1.552	0.737	1.466	4.5	56	0	1668.99112	3	557.0018921	-2.353685658	42.489185
														VYTVVDEMFLAGEIR	2	7	1	N-Term(iTRAQ8plex)	-	-	-	4.03	60	0	2046.09299	3	682.7025146	2.252778996	65.82777333
														FILIQNRAGK	1	7	1	N-Term(iTRAQ8plex); K10(iTRAQ8plex)	1.349	0.521	0.61	2.82	-	1	1768.09586	3	590.0368042	-5.385427659	49.02890333
11056036	TTLL12	tubulin--tyrosine ligase-like protein 12	NP_055955.1	12.11	6	6	16	644	74.4	5.53	1.066	5.361	1.398																

														FTLTQSEADADILFNFSHFK	3	1	1	N-Term(iTRAQ8plex); K20(iTRAQ8plex)	-	-	-	5.9	43	0	2939.54873	4	735.6426392	2.968299367	59.21844
														AFALNDLDDYEK	2	1	1	N-Term(iTRAQ8plex); K12(iTRAQ8plex)	0.925	4.6	1.321	4.76	54	0	2022.05564	3	674.6900635	-4.151280587	57.39717833
														LPLDINPVVHPHGHIFK	4	1	1	N-Term(iTRAQ8plex); K17(iTRAQ8plex)	1.332	6.58	1.955	3.92	23	0	2541.5019	3	847.8405151	6.110886259	34.20405
														MPVWYIMDEFGSR	4	1	1	N-Term(iTRAQ8plex)	0.755	3.928	1.283	3.81	42	0	1934.96172	2	967.9844971	8.799254513	57.72037
														YIESPVLFLR	2	1	1	N-Term(iTRAQ8plex)	1.229	6.248	1.479	3.29	26	0	1540.8991	2	770.953186	-3.242165371	48.99302667
														YIVLLR	1	1	1	N-Term(iTRAQ8plex)	0.89	1.662	0.849	-	17	0	1080.70525	2	540.8562622	-2.797715606	27.29764167
11056044	PPA1	inorganic pyrophosphatase	NP_066952.1	16.61	3	3	23	289	32.6	5.86	1.264	4.443	2.648																
														VLGILAMIDEGETDWK	10	1	1	N-Term(iTRAQ8plex); K16(iTRAQ8plex)	1.042	12.575	4.973	5.9	71	0	2398.32309	3	805.4450073	4.416324508	63.46465833
														VLGILAMIDEGETDWK	4	1	1	N-Term(iTRAQ8plex); M7(Oxidation); K16(iTRAQ8plex)	1.95	3.059	2.357	5.51	72	0	2414.32047	3	805.4450073	4.416324508	63.46465833
														HTGCCGDNDPIDVCEIGSK	8	1	1	N-Term(iTRAQ8plex); C4(Methylthio); C5(Methylthio); C14(Methylthio); [illegible]	1.264	4.745	2.408	5	39	0	2709.16591	3	903.7244263	-5.270544421	40.94376833
														MEIATKDPLNPIK	1	1	1	N-Term(iTRAQ8plex); K6(iTRAQ8plex); K13(iTRAQ8plex)	-	-	-	3.55	-	1	2382.39858	4	596.3551025	-8.735324107	29.41565333
11056056	MRPS12	28S ribosomal protein S12, mitochondrial precursor	NP_066930.1	18.12	1	1	2	138	15.2	10.29	0.357	-	0.317																
														EAVCFIPGEGHTLQEHQIVLVEGGR	2	1	1	N-Term(iTRAQ8plex); C4(Methylthio)	0.357	-	0.317	4.79	-	0	3068.55092	4	767.8931885	-2.965006602	39.999775
11067747	CDC5L	cell division cycle 5-like protein	NP_001244.1	8.23	5	5	12	802	92.2	8.18	1.596	0.925	0.831																
														ILQEAQNLMALTNVDTPLK	4	1	1	N-Term(iTRAQ8plex); K19(iTRAQ8plex)	1.619	0.679	0.652	6.52	63	0	2720.56968	3	907.5280762	8.062269393	63.67101167
														QLNDLWDQIEQAHLELR	2	1	1	N-Term(iTRAQ8plex)	1.127	0.437	0.615	5.58	52	0	2425.27658	3	809.0970459	-0.49761528	54.62185167
														TAAQCLEHYEFLLDK	3	1	1	N-Term(iTRAQ8plex); C5(Methylthio); K15(iTRAQ8plex)	2.131	1.706	1.729	4.13	23	0	2435.2562	3	812.423584	0.101181603	50.93349833
														AAGIEIQK	2	1	1	N-Term(iTRAQ8plex); K8(iTRAQ8plex)	1.715	1.403	1.058	3.72	42	0	1437.8917	3	479.96875	2.180588432	29.14711833
														IASLLHR	1	1	1	N-Term(iTRAQ8plex)	1.2	0.662	0.5	-	47	0	1113.70722	3	371.9072571	2.35851056	28.41123667
11136628	EEF1B2	elongation factor 1-beta	NP_066944.1	41.33	6	6	29	225	24.7	4.67	1.582	3.064	1.602																
														SYIEGYVPSQADVAVFEAVSSPPPADLCHALR	10	1	1	N-Term(iTRAQ8plex); C28(Methylthio)	-	0.461	0.179	6.22	52	0	3738.82102	3	1246.94519	-6.425624768	79.85939
														SPAGLQVLNDYLADK	10	1	1	N-Term(iTRAQ8plex); K15(iTRAQ8plex)	1.582	3.235	1.602	5.78	71	0	2212.22807	2	1106.617676	-6.928018713	37.19143333
														SIQADGLVWGSSK	5	1	1	N-Term(iTRAQ8plex); K13(iTRAQ8plex)	1.889	2.925	1.564	4.89	55	0	1956.08342	2	978.5453491	-9.033192356	40.645195
														SSILLDVKPWDDETDMAK	1	1	1	N-Term(iTRAQ8plex); K8(iTRAQ8plex); K18(iTRAQ8plex)	-	5.438	1.868	4.12	-	0	2975.59919	5	595.9256592	-5.76418747	38.84859167
														SSILLDVKPWDDETDMAKLEECVR	1	1	1	N-Term(iTRAQ8plex); K8(iTRAQ8plex); K18(iTRAQ8plex); C22(Methylthio)	-	-	-	3.58	-	1	3750.93763	5	750.9933472	-3.847829951	53.66109667
														LVPVGYGIK	2	1	1	N-Term(iTRAQ8plex); K9(iTRAQ8plex)	0.926	3.064	1.83	3.56	31	0	1553.97307	2	777.4901733	-9.312413832	24.7196
11225260	TOP1	DNA topoisomerase 1	NP_003277.1	27.97	18	18	53	765	90.7	9.31	2.257	1.163	1.072																
														GPVFAPPYEPLPENVK	3	1	1	N-Term(iTRAQ8plex); K16(iTRAQ8plex)	1.465	0.66	0.724	4.99	38	0	2362.33183	2	1181.669556	2.151860314	34.93525833
														VEHINLHPELDGQEYVVEFDFLGK	1	1	1	N-Term(iTRAQ8plex); K24(iTRAQ8plex)	-	-	-	4.88	-	0	3435.80654	4	859.7070923	0.572779812	49.02223333
														AEEVATFFAK	2	1	1	N-Term(iTRAQ8plex); K10(iTRAQ8plex)	2.716	1.203	1.122	4.68	59	0	1720.96526	2	860.9862671	-4.517081491	66.01805833
														IMPEDIIINCSK	6	1	1	N-Term(iTRAQ8plex); C10(Methylthio); K12(iTRAQ8plex)	2.646	1.591	1.417	4.46	45	0	2030.08733	2	1015.547302	-3.534048863	39.92104667
														VTWLVSWTENIQGSIK	6	1	1	N-Term(iTRAQ8plex); K16(iTRAQ8plex)	2.413	1.3	2.141	4.43	57	0	2469.39328	3	823.8026123	-1.189618501	59.47871167
														AVALYFIDKLALR	5	4	1	N-Term(iTRAQ8plex); K9(iTRAQ8plex)	3.039	1.833	1.504	4.29	33	1	2101.29911	4	526.0792236	-2.063798094	61.22258
														AVALYFIDK	4	4	1	N-Term(iTRAQ8plex); K9(iTRAQ8plex)	2.576	1.792	1.491	4.28	35	0	1647.97673	3	550.0023193	-0.374117129	33.872465
														CDFTQMSQYFK	4	1	1	N-Term(iTRAQ8plex); C1(Methylthio); K11(iTRAQ8plex)	2.214	1.203	1.055	4.28	71	0	2051.97026	2	1026.48877	-7.144283603	70.35014333
														TYNASITLQQQLK	2	1	1	N-Term(iTRAQ8plex); K13(iTRAQ8plex)	1.446	0.506	0.737	4.25	32	0	2116.21689	3	706.0771484	-2.527735942	38.04724667
														AVAILCNHQR	6	1	1	N-Term(iTRAQ8plex); C6(Methylthio)	1.199	0.963	1.012	4.17	57	0	1474.78626	3	492.2669373	-4.199783112	30.33864167
														NLQLFMENKQPEDDLFDR	1	1	1	N-Term(iTRAQ8plex); K9(iTRAQ8plex)	0.462	-	0.616	4.06	-	1	2860.46255	4	715.8710938	-4.718180989	42.53159667
														EYGFCIMDNHK	2	1	1	N-Term(iTRAQ8plex); C5(Methylthio); K11(iTRAQ8plex)	1.072	0.868	0.762	3.91	29	0	2010.96054	3	670.9916992	-4.50923504	32.889095
														AGNEKEEGETADTVGCCSLR	3	1	1	N-Term(iTRAQ8plex); K5(iTRAQ8plex); C16(Methylthio); C17(Methylthio)	3.52	1.425	1.362	3.89	32	1	2769.26797	4	693.0724487	-3.364881349	29.432405
														ITVAWCK	3	1	1	N-Term(iTRAQ8plex); C6(Methylthio); K7(iTRAQ8plex)	2.792	1.091	1.032	3.43	36	0	1474.83432	3	492.282959	-1.850103996	53.85077667
														HLQDLMEGLTAK	1	1	1	N-Term(iTRAQ8plex); K12(iTRAQ8plex)	1.105	0.717	0.809	3.34	-	0	1964.09946	3	655.3713379	-5.137661472	35.23155833
														WWEEERYPEGIK	1	1	1	N-Term(iTRAQ8plex); K12(iTRAQ8plex)	2.818	1.163	0.974	3.3	-	1	2230.1625	4	558.2960815	-5.750705676	31.81786833
														NLQLFMENK	1	4	1	N-Term(iTRAQ8plex); K9(iTRAQ8plex)	1.621	0.759	0.598	3.11	-	0	1744.99125	3	582.3352661	2.070950129	30.95861833
														YIMLNPSSR	2	1	1	N-Term(iTRAQ8plex)	1.405	0.698	0.78	2.8	-	0	1384.75286	2	692.8800659	-2.300832275	27.93600167
11321591	HMGB2	high mobility group protein B2	NP_002120.1	21.53	2	4	27	209	24.0	7.81	1.078	3.320	1.832																
														SEHPGLSIGDTAK	2	1	1	N-Term(iTRAQ8plex); K13(iTRAQ8plex)	1.078	3.32	1.832	4.34	44	0	1920.05271	3	640.6890869	-6.246603422	15.23670167
														RPPSAFFLFCSEHRPK	1	1	1	N-Term(iTRAQ8plex); C10(Methylthio); K16(iTRAQ8plex)	-	-	-	3.8	-	0	2573.36378	5	515.4785767	-3.588885995	43.17617667
														MSSYAFFVQTCR	16	2	2	N-Term(iTRAQ8plex); C11(Methylthio)	-	-	-	5.35	59	0	1789.83025	2	895.4187622	-4.192859042	41.095185
														MSSYAFFVQTCREEHK	1	2	2	N-Term(iTRAQ8plex); C11(Methylthio); K16(iTRAQ8plex)	-	-	-	4.02	-	1	2617.26967	4	655.072876	-4.782951438	39.27769667
														MSSYAFFVQTCR	7	2	2	N-Term(iTRAQ8plex); M1(Oxidation); C11(Methylthio)	-	-	-	3.22	32	0	1805.82842	2	895.4187622	-4.192859042	41.095185
11321601	PFKP	ATP-dependent 6-phosphofructokinase, platelet type isoform 1	NP_002618.1	40.43	23	27	199	784	85.5	7.55	0.803	1.555	3.432																
														NESCSENYTTDFIYQLYSEEGK	10	11	1	N-Term(iTRAQ8plex); C4(Methylthio); K22(iTRAQ8plex)	0.68	1.361	4.488	7.34	61	0	3274.51242	3	1092.175659	3.283538943	49.97305667
														IIEVVDAIMTTAQSHQR	42	9	1	N-Term(iTRAQ8plex)	1.088	1.186	2.371	7.04	97	0	2216.20127	3	739.4052734	0.065771328	68.947075
														IIEVVDAIMTTAQSHQR	14	9	1	N-Term(iTRAQ8plex); M9(Oxidation)	-	0.652	2.45	6.07	82	0	2232.18894	3	739.4052734	0.065771328	68.947075
														LPLMECVQMTQDVQK	14	11	1	N-Term(iTRAQ8plex); C6(Methylthio); K15(iTRAQ8plex)	0.941	1.724	2.825	5.05	63	0	2417.24118	3	806.4185791	-4.541642508	47.35636167
														NVLGHMQQGGAPSPFDR	5	11	1	N-Term(iTRAQ8plex)	0.383	1.034	1.449	4.79	36	0	2115.06058	3	705.692627	-3.524098086	25.62081667
														MLAIYDGFDGFAK	16	11	1	N-Term(iTRAQ8plex); K13(iTRAQ8plex)	0.93	2.271	6.24	4.73	71	0	2056.08281	2	1028.545044	-10.01906599	74.48873167
														IKELVVTQLGYDTR	8	11	1	N-Term(iTRAQ8plex); K2(iTRAQ8plex)	0.694	2.718	7.283	4.67	46	1	2243.30875	3	748.4411011	-5.898130315	43.118755
														DLQSNVEHLTEK	5	11	1	N-Term(iTRAQ8plex); K12(iTRAQ8plex)	0.767	1.44	5.769	4.67	53	0	2021.09661	2	1011.051941	-7.800757283	26.55229667
														VTILGHVQR	12	11	1	N-Term(iTRAQ8plex)	0.866	0.966	2.082	4.65	55	0	1326.80545	3	442.9407959	-6.110044331	17.08745
														GITNLCVIGGDGSLTGANLFR	2	8	1	N-Term(iTRAQ8plex); C6(Methylthio)	0.108	0.535	2.063	4.51	52	0	2428.25884	2	1214.633057	-1.752547416	60.08123333
														KNVLGHMQQGGAPSPFDR	3	11	1	N-Term(iTRAQ8plex); K1(iTRAQ8plex)	0.603	1.483	2.283	4.42	26	1	2547.35575	3	849.7901001	-6.031453223	24.44278
														NVLGHMQQGGAPSPFDR	6	11	1	N-Term(iTRAQ8plex); M6(Oxidation)	0.287	1.648	2.852	4.28	35	0	2131.05649	3	705.692627	-3.524098086	25.62081667
														FTTDDSICVLGISK	9	11	1	N-Term(iTRAQ8plex); C8(Methylthio); K14(iTRAQ8plex)	0.849	1.353	3.051	4.12	68	0	2153.13506	2	1077.071167	-4.284420336	40.930705
														NVIFQPVAELK	3	11	1	N-Term(iTRAQ8plex); K11(iTRAQ8plex)	0.886	1.264	4.505	3.97	41	0	1866.11345	2	933.5603638	-9.367265923	29.77823667
														GITNLCVIGGDGSLTGANLFRK	2	8	1	N-Term(iTRAQ8plex); C6(Methylthio); K22(iTRAQ8plex)	-	-	-	3.87	28	1	2860.56435	4	715.8965454	0.324922117	51.43505333
														YLEEIATQMR	6	11	1	N-Term(iTRAQ8plex)	0.626	1.565	4.111	3.84	36	0	1557.8272	2	779.4172363	1.498713244	41.09238833
														MLAIYDGFDGFAK	2	11	1	N-Term(iTRAQ8plex); M1(Oxidation); K13(iTRAQ8plex)	0.322	0.51	1.216	3.79	47	0	2072.08445	2	1028.545044	-10.01906599	74.48873167
														KEWSGLLEELAR	5	8	1	N-Term(iTRAQ8plex); K1(iTRAQ8plex)	1.431	2.88	9.005	3.7	51	1	2039.16245	4	510.5466003	-4.918589187	46.46217333
														EWSGLLEELAR	11	8	1	N-Term(iTRAQ8plex)	0.737	2.069	7.459	3.68	59	0	1606.86618	3	536.2935791	-5.03293018	55.178345
														FLEHLSGAGK	2	4	1	N-Term(iTRAQ8plex); K10(iTRAQ8plex)	0.869	1.029	2.246	3.48	31	0	1666.95879	2	833.9830322	-8.940513097	28.05634167
														ELVVTQLGYDTR	4	11	1	N-Term(iTRAQ8plex)	1.37	1.237	2.333	3.48	36	0	1697.93535	2	849.4713135	-1.313929223	32.28787833
														AMEWITAK	2	11	1	N-Term(iTRAQ8plex); K8(iTRAQ8plex)	0.362	0.712	2.706	3.16	28	0	1557.87632	3	519.963623	-10.02994218	48.30336
														KNVLGHMQQGGAPSPFDR	1	11	1	N-Term(iTRAQ8plex); K1(iTRAQ8plex); M7(Oxidation)	-	3.113	4.178	2.87	-	1	2563.34463	3	849.7901001	-6.031453223	24.44278
														ASYDVSDSGQLEHVQPWSV	1	11	1	N-Term(iTRAQ8plex); Y3(iTRAQ8plex)	0.705	1.697	3.617	-	28	0	2712.35739	3	904.7906494	-5.598933179	31.28191667
														AACNLLQR	2	8	1	N-Term(iTRAQ8plex); C3(Methylthio)	0.836	1.594	5.026	-	24	0	1238.65691	2	619.8320923	-6.631658561	31.20066
														NESCSENYTTDFIYQLYSEEGK	1	11	1	N-Term(iTRAQ8plex); C4(Methylthio); Y14(iTRAQ8plex); K22(iTRAQ8plex)	-	-	-	-	22	0	3578.69546	3	1092.175659	3.283538943	49.97305667
														LAIKLPDDQIPK	1	11	1	N-Term(iTRAQ8plex); K4(iTRAQ8plex); K12(iTRAQ8plex)	-	-	-	-	21	1	2263.39272	4	566.6036377	-9.967514052	32.59216167
														EKHEEFCVPMVMVPATVSNNVPGSDFSIGADTALNTI[illegible]	1	6	1	N-Term(iTRAQ8plex); K2(iTRAQ8plex); C7(Methylthio); C41(Methylthio)	-	-	-	-	21	1	5311.5084	3	1771.174316	1.388055558	65.08779667
														MGIYVGAK	2	13	2	N-Term(iTRAQ8plex); K8(iTRAQ8plex)	-	-	-	3.06	35	0	1446.84555	3	482.9533691	-9.916143491	32.84326333
														GVFDCR	2	18	2	N-Term(iTRAQ8plex); C5(Methylthio)	-	-	-	-	18	0	1046.4998	2	523.7539063	-5.691750549	19.51380333
														EQWWLK	2	16	2	N-Term(iTRAQ8plex); K6(iTRAQ8plex)	-	-	-	2.74	-	0	1497.85271	2	749.4299927	-9.822440052	32.76727167
														TFVLEVMGR	3	20	3	N-Term(iTRAQ8plex)	-	-	-	3.75	27	0	1355.75701	2	678.3821411	-6.561421701	43.95479333
11345462	SPCS3	signal peptidase complex subunit 3	NP_068747.1	11.67	2	2	3	180	20.3	8.62	0.972	0.573	0.349																
														NNALNQVVLWDK	2	1	1	N-Term(iTRAQ8plex); K12(iTRAQ8plex)	0.861	0.49	0.311	4.83	39	0	2022.14719	3	674.7205811	-5.974533406	55.32296833
														NVEDFTGPR	1	1	1	N-Term(iTRAQ8plex)	1.097	0.671	0.392	-	34	0	1338.68962	2	669.8484497	-4.436058842	16.21459167
11386147	PSAP	prosaposin isoform a preproprotein	NP_002769.1	9.54	5	5	17	524	58.1	5.17	0.493	0.569	0.905																
														GCSFLPDPYQK	5	3	1	N-Term(iTRAQ8plex); C2(Methylthio); K11(iTRAQ8plex)	0.493	0.492	0.788	4.1	40	0	1908.9708	2	954.9855347	-8.920852312	60.77685333
														EICALVGFCDEVK	4	3	1	N-Term(iTRAQ8plex); C3(Methylthio); C9(Methylthio); K13(iTRAQ8plex)	0.718	0.917	1.344	3.62	53	0	2126.05954	3	709.3580322	-0.917128741	59.84619167
														QEILAALEK	2	3	1	N-Term(iTRAQ8plex); K9(iTRAQ8plex)	0.69	0.881	0.905	3.44	33	0	1622.99485	3	541.6697998	0.668433461	62.43273
														SLPCDICK	2	3	1	N-Term(iTRAQ8plex); C4(Methylthio); C7(Methylthio); K8(iTRAQ8plex)	0.318	0.326	0.609	3.09	22	0	1578.79473	2	789.901001	-1.594866536	63.015445
														DGGFCEVCK	4	3	1	N-Term(iTRAQ8plex); C5(Methylthio); C8(Methylthio); K9(iTRAQ8plex)	0.455	0.535	1.151	2.83	31	0	1657.76145	3	553.258667	-3.158218841	53.46817833
11415026	RPL18A	60S ribosomal protein L18a	NP_000971.1	35.23	6	6	27	176	20.7	10.71	1.157	1.581	1.821																
														SSGEIVYCGQVFEK	8	1	1	N-Term(iTRAQ8plex); C8(Methylthio); K14(iTRAQ8plex)	1.458	1.568	1.498	5.2	73	0	2200.10576	2	1100.556519	-8.232245445	64.63858
														DLTTAGAVTQCYR	9	1	1	N-Term(iTRAQ8plex); C11(Methylthio)	1.004	1.515	1.375	4.56	39	0	1748.85588	2	874.9313965	-3.319857584	28.49326167
														IFAPNHVVAK	6	1	1	N-Term(iTRAQ8plex); K10(iTRAQ8plex)	1.684	2.265	1.914	3.68	47	0	1704.02663	2	852.5210571	-4.033756843	29.79085667
														VKNFGIWLR	2	1	1	N-Term(iTRAQ8plex); K2(iTRAQ8plex)	1.682	2.381	1.848	2.85	34	1	1741.05913	4	436.0227051	-2.504706892	38.53538
														AHSIQIMK	1	1	1	N-Term(iTRAQ8plex); M7(Oxidation); K8(iTRAQ8plex)	1.041	1.47	1.373	2.79	-	0	1551.89975	3	517.9714355	-9.003744826	20.92812667
														IKFPLPHR	1	1	1	N-Term(iTRAQ8plex); K2(iTRAQ8plex)	-	-	-	-	29	1	1616.02382	4	404.7614136	-1.136668344	23.226955
11496277	LAMTOR3	ragulator complex protein LAMTOR3 isoform 1	NP_068805.1	30.65	2	2	3	124	13.6	7.34	0.552	0.446	0.385																
														VANDNAPEHALRPGFLSTFALATDQGSK	1	1	1	N-Term(iTRAQ8plex); K28(iTRAQ8plex)	-	-	-	4.06	-	0	3535.86294	4	884.7211914	-3.545260443	38.36831167
														ELAPLFEELR	2	2	1	N-Term(iTRAQ8plex)	0.552	0.446	0.385	3.07	36	0	1520.8582	2	760.9327393	-2.91901268	50.96781333
11559929	COPG1	coatomer subunit gamma-1	NP_057212.1	10.41	7	7	15	874	97.7	5.47	1.095	1.511	2.005																
														SIATLAITTLLK	4	4	1	N-Term(iTRAQ8plex); K12(iTRAQ8plex)	0.831	1.667	2.069	4.62	58	0	1853.18132	2	927.0942993	-6.404816974	47.482685
														EMSCIAEDVIIVTSSLTK	2	2	1	N-Term(iTRAQ8plex); C4(Methylthio); K18(iTRAQ8plex)	-	-	-	4.25	53	0	2593.39652	3	865.1370239	8.38137446	66.53735833
														VVLEHEEVR	4	2	1	N-Term(iTRAQ8plex)	1.041	2.02	2.155	4	41	0	1413.79502	3	471.9365234	-3.78006531	18.25095667
														ATFYLNVLEQK	1	1	1	N-Term(iTRAQ8plex); K11(iTRAQ8plex)	1.133	1.067	1.063	2.93	-	0	1934.10514	3	645.37323	-8.068128406	69.19184
														NTHTLLLAGVFR	2	1	1	N-Term(iTRAQ8plex)	1.841	1.026	0.772	2.89	25	0	1645.95774	3	549.3240967	-6.940950355	43.14539333
														FGAQNEEMLPSILVLLK	1	2	1	N-Term(iTRAQ8plex); K17(iTRAQ8plex)	0.672	2.426	2.354	-	44	0	2510.46025	2	1255.733765	3.55766512	61.67139167
														CVMDDDNEVRDR	1	1	1	N-Term(iTRAQ8plex); C1(Methylthio)	1.545	1.481	2.006	-	17	1	1816.78562	3	606.2667236	-4.075688122	17.66065333
11596859	MRPL17	39S ribosomal protein L17, mitochondrial	NP_071344.1	5.14	1	1	2	175	20.0	10.11	0.795	0.575	0.533																
														MADFWLTEK	2	1	1	N-Term(iTRAQ8plex); K9(iTRAQ8plex)	0.795	0.575	0.533	3.27	42	0	1748.94646	2	874.9768677	-2.134181077	65.98113667
11761621	GMPPB	mannose-1-phosphate guanyltransferase beta isoform 2	NP_068806.1	5.56	1	1	2	360	39.8	6.79	0.987	0.911	0.759																
														ISMSHEEEPLGTAGPLALAR	2	2	1	N-Term(iTRAQ8plex)	0.987	0.911	0.759	4.8	32	0	2383.25534	3	795.0899658	-1.698134894	35.105195

11863154	ARCN1	coatomer subunit delta isoform 1	NP_001646.2	15.85	7	7	10	511	57.2	6.21	0.958	2.039	2.438																
														NTLEWCLPVIDAK	2	2	1	N-Term(iTRAQ8plex); C6(Methylthio); K13(iTRAQ8plex)	0.958	1.656	1.926	4.22	34	0	2156.15439	3	719.3896484	-7.443918468	45.28733167
														LFTAESLIGLK	2	3	1	N-Term(iTRAQ8plex); K11(iTRAQ8plex)	1.231	2.644	3.577	4.17	45	0	1800.09391	3	600.7028198	-8.457935051	44.55783333
														NSNILEDLETLR	2	2	1	N-Term(iTRAQ8plex)	0.772	1.264	1.733	3.94	40	0	1720.93083	2	860.9690552	-4.350882842	39.45532833
														TRIEGLLAAFPK	1	2	1	N-Term(iTRAQ8plex); K12(iTRAQ8plex)	1.035	1.638	1.87	3.85	-	1	1924.17331	3	642.0626221	-5.57555756	39.4433
														QHTFVETESVR	1	2	1	N-Term(iTRAQ8plex)	0.614	2.514	3.295	3.32	-	0	1636.85307	2	818.9301758	-4.023792728	14.92420833
														VAPAPARPSGPSK	1	3	1	N-Term(iTRAQ8plex); K13(iTRAQ8plex)	0.504	2.131	2.986	3.16	-	0	1843.08293	2	922.045105	-9.824820541	13.91285667
														ENVNLAQIR	1	3	1	N-Term(iTRAQ8plex)	1.372	2.039	2.438	2.7	-	0	1360.778	2	680.8926392	-5.17321685	17.15883667
11968009	SIL1	nucleotide exchange factor SIL1 precursor	NP_071909.1	2.60	1	1	2	461	52.1	5.36	0.261	0.500	0.525																
														VVTLLYDLVTEK	2	2	1	N-Term(iTRAQ8plex); K12(iTRAQ8plex)	0.261	0.5	0.525	4.15	39	0	2001.20212	3	667.7388916	-3.552737245	66.20595167
11968182	RPS18	40S ribosomal protein S18	NP_072045.1	46.71	9	9	28	152	17.7	10.99	1.074	2.295	2.054																
														AGELTEDEVER	8	1	1	N-Term(iTRAQ8plex)	1.022	1.729	1.802	4.61	40	0	1551.78081	2	776.3898926	-5.10562193	15.78813167
														YSQVLANGLDNK	2	1	1	N-Term(iTRAQ8plex); K12(iTRAQ8plex)	0.646	0.899	0.877	3.99	38	0	1930.06968	3	644.0280762	-8.15827251	30.33024333
														YAHVVLR	4	1	1	N-Term(iTRAQ8plex)	1.083	2.394	1.957	3.23	36	0	1161.70136	4	367.2323608	-1.587972259	16.58061833
														VLNTNIDGR	2	1	1	N-Term(iTRAQ8plex)	1.146	2.195	1.911	3.11	35	0	1305.73711	2	653.3721924	-4.390741704	21.74313167
														FQHILR	2	1	1	N-Term(iTRAQ8plex)	0.99	3.472	3.272	2.97	23	0	1117.66742	3	373.2273254	-9.795607723	18.764995
														IPDWFLNR	2	1	1	N-Term(iTRAQ8plex)	1.264	2.78	2.242	2.8	38	0	1364.75287	3	455.5891418	-7.311955954	36.108565
														HFWGLR	3	1	1	N-Term(iTRAQ8plex)	1.489	3.475	3.695	2.74	20	0	1119.62885	3	373.881134	-6.843741476	26.12721333
														QYKIPDWFLNR	1	1	1	N-Term(iTRAQ8plex); K3(iTRAQ8plex)	1.11	2.805	1.736	2.7	-	1	2088.17063	3	696.7283936	-6.92179353	39.649435
														YAHVVLR	3	1	1	N-Term(iTRAQ8plex); Y1(iTRAQ8plex)	0.572	2.417	2.381	-	39	0	1465.90761	4	367.2323608	-1.587972259	16.58061833
														VITIMQNPR	1	1	1	N-Term(iTRAQ8plex)	1.363	2.283	2.078	-	27	0	1375.79875	2	688.4030151	-3.334533158	30.54023167
12025678	ACTN4	alpha-actinin-4 isoform 1	NP_004915.2	61.36	25	44	256	911	104.8	5.44	0.625	0.741	1.368																
														VEQIAAIAQELNELDYYDSHNVNTR	8	5	1	N-Term(iTRAQ8plex)	2.035	1.004	2.398	8.74	99	0	3209.62168	3	1070.536621	-1.994416159	63.86347333
														QFASQANVVGPWIQTK	2	5	1	N-Term(iTRAQ8plex); K16(iTRAQ8plex)	0.883	1.222	1.106	6.35	38	0	2382.33414	3	794.7828979	-2.057101168	35.37067667
														ICDQWDALGSLTHSR	10	5	1	N-Term(iTRAQ8plex); C2(Methylthio)	0.773	0.969	1.185	5.62	73	0	2051.98862	3	684.6681519	-2.231971799	54.63362333
														LVSIGAEEIVDGNAK	2	5	1	N-Term(iTRAQ8plex); K15(iTRAQ8plex)	0.668	0.59	1.379	5.47	69	0	2123.20566	3	708.4067383	-5.27961396	38.05282833
														DGLAFNALIHR	10	10	1	N-Term(iTRAQ8plex)	0.726	0.773	1.219	5.43	71	0	1530.8667	3	510.9604187	-1.795353233	31.29268667
														ETTDTDTADQVIASFK	2	5	1	N-Term(iTRAQ8plex); K16(iTRAQ8plex)	0.64	0.886	1.908	5.3	87	0	2350.20244	2	1175.604858	-8.944388264	30.54452667
														QLEAIDQLHLEYAK	7	5	1	N-Term(iTRAQ8plex); K14(iTRAQ8plex)	0.62	0.537	0.877	4.96	34	0	2279.27285	4	570.5736694	-5.591013659	33.51476667
														MVSDINNGWQHLEQAEK	1	5	1	N-Term(iTRAQ8plex); K17(iTRAQ8plex)	0.494	0.511	1.219	4.59	-	0	2607.33249	3	869.7823486	-4.64791184	38.10501833
														ACLISLGYDVENDR	2	3	1	N-Term(iTRAQ8plex); C2(Methylthio)	0.808	0.913	1.312	4.59	46	0	1917.95146	2	959.4793701	8.462838963	45.46594667
														VLAGDKNFITAEELR	4	5	1	N-Term(iTRAQ8plex); K6(iTRAQ8plex)	0.646	1.139	1.713	4.49	54	1	2284.30058	2	1142.653931	-5.072271116	36.06779167
														AGTQIENIDEDFRDGLK	5	5	1	N-Term(iTRAQ8plex); K17(iTRAQ8plex)	0.536	0.528	1.241	4.43	45	1	2529.32656	4	633.0886841	-3.034338365	33.57330667
														MLDAEDIVNTARPDEK	3	3	1	N-Term(iTRAQ8plex); K16(iTRAQ8plex)	0.747	0.778	1.902	4.27	41	0	2425.27944	4	607.0753174	-2.449882126	33.159595
														KDDPVTNLNNAFEVAEK	2	5	1	N-Term(iTRAQ8plex); K1(iTRAQ8plex); K17(iTRAQ8plex)	-	1.204	2.756	3.99	25	1	2816.54311	4	704.8912354	-4.494791398	32.19572
														DYETATLSDIK	2	5	1	N-Term(iTRAQ8plex); K11(iTRAQ8plex)	0.59	0.564	0.975	3.96	66	0	1864.01116	2	932.5092163	-2.599395823	36.00975333
														GISQEQMQEFR	4	5	1	N-Term(iTRAQ8plex)	0.605	0.782	1.555	3.7	51	0	1656.82341	2	828.9153442	-5.033393045	18.59903833
														TIPWLEDRVPQK	1	5	1	N-Term(iTRAQ8plex); K12(iTRAQ8plex)	0.605	0.367	1.481	3.57	-	1	2090.21963	4	523.3103638	-1.079361158	32.82581167
														NVNVQNFHISWK	3	5	1	N-Term(iTRAQ8plex); K12(iTRAQ8plex)	0.724	0.656	1.339	3.56	30	0	2094.15598	3	698.7235107	-6.929915242	34.97721667
														ASFNHFDKDHGGALGPEEFK	2	3	1	N-Term(iTRAQ8plex); K8(iTRAQ8plex); K20(iTRAQ8plex)	0.519	0.845	1.399	3.46	-	1	3115.63833	4	779.6650391	0.588986849	28.80101167
														SIVDYKPNLDLLEQQHQLIQEALIFDNK	1	5	1	N-Term(iTRAQ8plex); K6(iTRAQ8plex); K28(iTRAQ8plex)	-	-	-	3.38	-	0	4237.36036	5	848.2778931	-0.774651597	57.202915
														STLPDADRER	6	5	1	N-Term(iTRAQ8plex)	0.595	0.708	1.29	3.34	24	1	1463.77027	3	488.5949402	-3.650841583	13.62778833
														ACLISLGYDVENDRQGEAEFNR	1	2	1	N-Term(iTRAQ8plex); C2(Methylthio)	0.468	1.274	1.626	3.25	-	1	2849.35581	3	950.4567871	2.013008733	45.77723333
														RDHALLEEQSK	3	5	1	N-Term(iTRAQ8plex); K11(iTRAQ8plex)	0.376	0.896	1.866	3.21	41	1	1934.07866	2	967.5429688	-6.680400472	11.57798667
														HRPELIEYDK	2	5	1	N-Term(iTRAQ8plex); K10(iTRAQ8plex)	0.451	0.685	1.48	3.06	30	0	1908.08611	4	477.7758789	0.91424021	16.93280667
														EAILAIHK	2	5	1	N-Term(iTRAQ8plex); K8(iTRAQ8plex)	0.714	0.803	1.346	3.01	27	0	1502.93877	2	751.9730225	-8.465413491	20.69476333
														NFITAEELR	1	5	1	N-Term(iTRAQ8plex)	0.41	0.729	1.037	2.71	-	0	1396.76763	2	698.8874512	-4.428493427	29.88282333
														TINEVENQILTR	9	20	2	N-Term(iTRAQ8plex)	-	-	-	6.26	67	0	1733.97672	3	578.6637573	3.902833633	35.70732833
														AIMTYVSSFYHAFSGAQK	4	18	2	N-Term(iTRAQ8plex); K18(iTRAQ8plex)	-	-	-	5.81	38	0	2616.37685	4	654.8496704	1.055069962	63.23410333
														FAIQDISVEETSAK	8	24	2	N-Term(iTRAQ8plex); K14(iTRAQ8plex)	-	-	-	5.39	56	0	2146.17844	3	716.0623169	-5.975226604	27.650155
														KTFTAWCNSHLR	18	14	2	N-Term(iTRAQ8plex); K1(iTRAQ8plex); C7(Methylthio)	-	-	-	5.01	46	1	2118.12612	4	530.2869873	3.020140629	35.549485
														GYEEWLLNEIR	40	23	2	N-Term(iTRAQ8plex)	-	-	-	4.99	61	0	1725.90422	2	863.4557495	-4.142116619	40.94413667
														KHEAFESDLAAHQDR	4	23	2	N-Term(iTRAQ8plex); K1(iTRAQ8plex)	-	-	-	4.9	50	1	2362.22239	3	788.0789795	-5.776794565	17.487465
														LMLLLEVISGER	4	24	2	N-Term(iTRAQ8plex)	-	-	-	4.72	58	0	1677.00309	3	559.6725464	6.436845732	66.21151667
														TFTAWCNSHLR	4	17	2	N-Term(iTRAQ8plex); C6(Methylthio)	-	-	-	4.56	47	0	1685.82273	3	562.6124268	1.978098518	38.61585
														VGWEQLLTTIAR	15	20	2	N-Term(iTRAQ8plex)	-	-	-	4.5	49	0	1690.97367	3	564.3294067	-3.391143971	57.81293
														CQLEINFNTLQTK	6	25	2	N-Term(iTRAQ8plex); C1(Methylthio); K13(iTRAQ8plex)	-	-	-	4.38	40	0	2206.16709	3	736.0605469	-6.783223757	35.175255
														ELPPDQAEYCIAR	16	20	2	N-Term(iTRAQ8plex); C10(Methylthio)	-	-	-	4.2	44	0	1854.89629	2	927.9517822	-3.727683817	31.54306
														LSNRPAFMPSEGK	1	5	2	N-Term(iTRAQ8plex); K13(iTRAQ8plex)	-	-	-	3.88	-	0	2042.12563	4	511.2868652	-2.806018598	21.50268
														EGLLLWCQR	20	24	2	N-Term(iTRAQ8plex); C7(Methylthio)	-	-	-	3.87	47	0	1467.76616	3	489.9292908	-1.426129989	46.98549167
														ASIHEAWTDGK	1	14	2	N-Term(iTRAQ8plex); K11(iTRAQ8plex)	-	-	-	3.81	-	0	1822.97861	3	608.3310547	-6.687125909	25.401765
														DLLLDPAWEK	2	14	2	N-Term(iTRAQ8plex); K10(iTRAQ8plex)	-	-	-	3.76	36	0	1808.02384	3	603.3461304	-9.741816645	60.69756333
														LASDLLEWIR	13	20	2	N-Term(iTRAQ8plex)	-	-	-	3.7	79	0	1519.87745	3	507.2973328	-0.773916995	55.214405
														HTNYTMEHIR	3	23	2	N-Term(iTRAQ8plex); M6(Oxidation)	-	-	-	3.41	32	0	1621.79715	3	541.2705688	-5.353941468	10.12486833
														ALDFIASK	1	22	2	N-Term(iTRAQ8plex); K8(iTRAQ8plex)	-	-	-	-	32	0	1472.89515	3	491.6365662	1.24512348	34.84575667
														LDHLAEK	1	20	2	N-Term(iTRAQ8plex); K7(iTRAQ8plex)	-	-	-	-	32	0	1433.84697	2	717.427124	-7.169435323	20.96574333
12232389	METTL17	methyltransferase-like protein 17, mitochondrial isoform 2 precursor	NP_073571.1	2.41	1	1	1	456	50.7	9.33	0.619	0.843	0.784																
														HLPVEPEELQR	1	2	1	N-Term(iTRAQ8plex)	0.619	0.843	0.784	3.14	-	0	1650.90946	3	550.9746704	-1.364895888	22.14213667
12408656	CAPN1	calpain-1 catalytic subunit	NP_005177.2	9.94	5	5	9	714	81.8	5.67	0.907	1.321	0.974																
														QLAGEDMEISVK	2	1	1	N-Term(iTRAQ8plex); K12(iTRAQ8plex)	0.329	0.534	0.367	3.63	36	0	1928.0557	3	643.3567505	-3.238021164	36.692015
														NYPATFWVNPQFK	2	1	1	N-Term(iTRAQ8plex); K13(iTRAQ8plex)	0.935	1.822	1.599	3.42	29	0	2220.19425	3	740.7362671	-5.388393597	65.777785
														WNTTLYEGTWR	2	1	1	N-Term(iTRAQ8plex)	0.998	1.171	0.906	3.16	29	0	1730.86736	2	865.9373169	-7.53040427	54.79122667
														LVFVHSAEGNEFWSALLEK	1	1	1	N-Term(iTRAQ8plex); K19(iTRAQ8plex)	-	-	-	3	-	0	2784.52993	4	696.8879395	4.22930721	59.11092333
														TLDTDLDGVVTFDLFK	2	1	1	N-Term(iTRAQ8plex); K16(iTRAQ8plex)	0.88	1.49	1.048	2.94	51	0	2407.32549	2	1204.166382	1.560134637	55.25675
12408675	PFDN2	prefoldin subunit 2	NP_036526.2	35.06	4	4	6	154	16.6	6.58	1.265	1.698	2.320																
														GAVSAEQVIAGFNR	2	1	1	N-Term(iTRAQ8plex)	1.265	2.215	2.804	4.75	38	0	1722.95122	2	861.979248	4.137188372	34.77130833
														IIETLTQQLQAK	2	2	1	N-Term(iTRAQ8plex); K12(iTRAQ8plex)	1.309	1.698	2.32	4.5	38	0	1994.202	3	665.4055176	-4.328456008	74.78598667
														AAELEMELNEHSLVIDTLK	1	1	1	N-Term(iTRAQ8plex); K19(iTRAQ8plex)	-	-	-	3.23	-	0	2763.5021	4	691.6309814	-1.391494783	50.39851167
														MVGGVLVER	1	2	1	N-Term(iTRAQ8plex)	1.002	1.649	2.063	-	30	0	1263.73418	2	632.3707275	-4.362342943	28.833325
12408677	PFDN4	prefoldin subunit 4	NP_002614.2	8.96	1	1	2	134	15.3	4.53	1.117	2.201	2.698																
														NLQEEIDALESR	2	2	1	N-Term(iTRAQ8plex)	1.117	2.201	2.698	3.8	54	0	1720.8947	2	860.9509888	-4.20733525	32.658265
13027378	GNPDA1	glucosamine-6-phosphate isomerase 1	NP_005462.1	13.49	2	2	4	289	32.6	6.92	1.173	3.630	4.812																
														AAGGIELFVGGIGPDGHIAFNEPGSSLVSR	2	2	1	N-Term(iTRAQ8plex)	1.377	4.516	4.626	6.06	69	0	3228.67929	3	1076.897949	-5.056833541	74.37419667
														LVDPLYSIK	2	2	1	N-Term(iTRAQ8plex); K9(iTRAQ8plex)	0.999	2.918	5.006	3.62	49	0	1656.00359	2	828.5054321	-9.446635445	30.80375833
13027602	DDRGK1	DDRGK domain-containing protein 1	NP_076424.1	6.05	1	1	4	314	35.6	5.12	1.090	0.819	0.777																
														AASAGQEPLHNEELAGAGR	4	1	1	N-Term(iTRAQ8plex)	1.09	0.819	0.777	4.09	55	0	2182.11014	3	728.0415649	-2.451629585	18.35508833
13027604	MRPS34	28S ribosomal protein S34, mitochondrial isoform 2	NP_076425.1	12.39	1	1	1	218	25.6	9.98	-	-	-																
														HEEEAFTAFTPAPEDSLASVPYPPLLR	1	3	1	N-Term(iTRAQ8plex)	-	-	-	3.86	-	0	3288.68442	3	1096.899658	3.43604248	53.19699667
13128860	HDAC1	histone deacetylase 1	NP_004955.2	10.37	1	4	12	482	55.1	5.48	1.135	1.124	1.480																
														QQTDIAVNWAGGLHHAK	2	1	1	N-Term(iTRAQ8plex); K17(iTRAQ8plex)	1.135	1.124	1.48	4.6	27	0	2454.35293	4	614.343689	2.719527133	23.91835
														MTHNLLLNYGLYR	4	3	2	N-Term(iTRAQ8plex)	-	-	-	4.3	49	0	1912.0336	3	638.0160522	-4.214262607	35.758585
														YGEYFPGTGDLR	4	4	2	N-Term(iTRAQ8plex)	-	-	-	4.1	36	0	1678.84184	2	839.9245605	2.372572356	32.61322833
														VMTVSFHK	2	4	2	N-Term(iTRAQ8plex); K8(iTRAQ8plex)	-	-	-	3.31	35	0	1556.89409	2	778.9506836	-8.877117964	22.64419667
13129092	TMEM109	transmembrane protein 109 precursor	NP_076997.1	4.94	1	1	4	243	26.2	10.48	0.870	0.615	0.627																
														EAPVDVLTQIGR	4	1	1	N-Term(iTRAQ8plex)	0.87	0.615	0.627	4.08	60	0	1601.91423	2	801.4607544	-1.397784533	37.85435
13129100	DCTPP1	dCTP pyrophosphatase 1	NP_077001.1	8.24	1	1	1	170	18.7	5.03	0.677	1.605	0.689																
														FSFSPEPTLEDIRR	1	1	1	N-Term(iTRAQ8plex)	0.677	1.605	0.689	2.76	-	1	1998.05637	3	666.6903076	-1.742542648	36.287465
13236587	TMEM43	transmembrane protein 43	NP_077310.1	33.25	9	9	43	400	44.8	8.13	0.552	0.345	0.358																
														TATSLAEGLSLVVSPDSIHSVAPENEGR	2	2	1	N-Term(iTRAQ8plex)	0.549	-	0.268	7.27	49	0	3140.64145	3	1047.552002	1.16259988	46.73276333
														VSFSYAGLSGDDPDLGPAHVVTVIAR	4	2	1	N-Term(iTRAQ8plex)	0.732	0.663	0.72	6.93	78	0	2947.5474	3	983.1873169	0.205639982	45.324085
														SGDTLLLLHHGDFSAEEVFHR	4	2	1	N-Term(iTRAQ8plex)	-	-	0.203	5.77	41	0	2684.39802	5	537.6854248	9.136578584	37.268755
														FFLSSGLIDKVDNFK	3	2	1	N-Term(iTRAQ8plex); K10(iTRAQ8plex); K15(iTRAQ8plex)	0.304	-	0.207	5.18	38	1	2642.53432	4	661.3890381	0.836758763	54.41365833
														HVEMYQWVETEESR	6	2	1	N-Term(iTRAQ8plex)	0.806	0.642	0.734	4.03	65	0	2127.00364	3	709.6727295	-3.886252614	32.10775
														FFLSSGLIDK	2	2	1	N-Term(iTRAQ8plex); K10(iTRAQ8plex)	0.62	0.271	0.274	3.77	48	0	1735.02239	2	868.0148315	-1.54167121	67.87232167
														LEDPHVDIIR	5	2	1	N-Term(iTRAQ8plex)	0.555	0.345	0.378	3.45	31	0	1510.85271	2	755.9272461	-3.915506769	23.52458333
														LVHIIGALR	16	2	1	N-Term(iTRAQ8plex)	0.297	0.282	0.281	3.06	50	0	1295.84314	3	432.6190796	-2.960066458	34.07895667
														TSSQPGFLER	1	1	1	N-Term(iTRAQ8plex)	0.619	0.526	0.442	2.79	-	0	1425.76055	2	713.3839111	-2.40268563	19.956185
13259531	SMN2	survival motor neuron protein isoform c	NP_075015.1	16.00	2	2	4	250	27.1	5.25	1.702	3.303	2.991																
														CSAIWSEDGCIYPATIASIDFK	2	6	1	N-Term(iTRAQ8plex); C1(Methylthio); C10(Methylthio); K22(iTRAQ8plex)	-	-	-	5.76	73	0	3090.5101	3	1030.841553	6.323115752	61.52732833
														GTGQSDDSDIWDDTALIK	2	10	1	N-Term(iTRAQ8plex); K18(iTRAQ8plex)	1.702	3.303	2.991	4.52	43	0	2545.28702	3	849.1005249	-0.333888855	36.85585333
13375616	FADS3	fatty acid desaturase 3	NP_068373.1	3.37	1	1	2	445	51.1	7.77	1.264	0.675	1.027																
														LIGHHGAEDATDAFR	2	2	1	N-Term(iTRAQ8plex)	1.264	0.675	1.027	3.47	29	0	1913.97013	3	638.6615601	-3.685728486	18.24506833
13375618	DHCR24	delta(24)-sterol reductase precursor	NP_055577.1	4.84	2	2	3	516	60.1	8.16	0.925	0.985	0.367																
														NIMINLMDILEVDTKK	2	1	1	N-Term(iTRAQ8plex); K15(iTRAQ8plex); K16(iTRAQ8plex)	1	1.223	-	5.33	29	1	2802.63271	4	701.4136353	3.225479953	64.533015
														EGLEYIPLR	1	1	1	N-Term(iTRAQ8plex)	0.855	0.793	0.367	-	31	0	1393.79851	2	697.4028931	-0.56090402	33.70376667

13375983	SLC25A22	mitochondrial glutamate carrier 1	NP_078974.1	15.17	3	3	4	323	34.4	9.29	1.773	0.932	0.627																
														GVNEDTYSGILDCAR	2	1	1	N-Term(iTRAQ8plex); C13(Methylthio)	1.768	1.028	0.529	5.3	29	0	1962.93389	2	981.9705811	6.914746072	36.54336333
														DVPFSVVYFPLFANLNQLGRPASEEK	1	1	1	N-Term(iTRAQ8plex); K26(iTRAQ8plex)	-	-	-	3.82	-	0	3545.9269	4	887.2371826	0.431280394	57.91563
														GLGATLLR	1	5	1	N-Term(iTRAQ8plex)	1.778	0.845	0.743	-	28	0	1104.70378	2	552.8555298	-0.442063922	27.45941167
13376617	PTGES2	prostaglandin E synthase 2 isoform 1	NP_079348.1	18.04	5	5	14	377	41.9	9.16	1.376	0.759	0.831																
														AFLDFHALPYQVVEVNPVR	6	1	1	N-Term(iTRAQ8plex)	1.039	0.351	0.831	5.49	39	0	2518.38572	3	840.1334229	3.83738068	44.11177833
														LQLTLYQYK	2	1	1	N-Term(iTRAQ8plex); K9(iTRAQ8plex)	1.572	0.866	1.103	4.59	40	0	1778.05643	3	593.3569946	-6.068796859	57.54885
														TPTEALASFDYIVR	2	2	1	N-Term(iTRAQ8plex)	2.124	0.829	0.81	4.25	70	0	1887.01006	2	944.008667	-3.451987762	49.106965
														QWADDWLVHLISPNVYR	2	2	1	N-Term(iTRAQ8plex)	3.017	1.188	1.177	4.2	38	0	2416.27658	3	806.0970459	2.074739848	61.0606
														AITEASPAH	2	2	1	N-Term(iTRAQ8plex)	1.376	0.663	0.712	3.39	53	0	1200.64812	2	600.8276978	-3.749984793	16.10729167
13449277	CYP2S1	cytochrome P450 2S1 precursor	NP_085125.1	3.17	1	1	2	504	55.8	8.62	0.786	0.469	0.381																
														QVQQHQGNLDASGPAR	2	1	1	N-Term(iTRAQ8plex)	0.786	0.469	0.381	3.32	33	0	2010.04081	3	670.6851196	-0.545095044	11.45717
13514831	DDX10	probable ATP-dependent RNA helicase DDX10	NP_004389.2	1.71	1	1	2	875	100.8	8.63	0.863	0.578	0.609																
														SNSEVEDVGPTSHNR	2	1	1	N-Term(iTRAQ8plex)	0.863	0.578	0.609	6.12	56	0	1931.9282	3	644.647583	-4.086863363	11.305625
13540555	PTDSS2	phosphatidylserine synthase 2	NP_110410.1	5.54	2	2	2	487	56.2	6.25	1.144	0.569	0.827																
														IAFQFTPYSWVR	1	4	1	N-Term(iTRAQ8plex)	1.49	0.621	0.959	3.27	-	0	1818.98613	2	909.9967041	0.912751942	48.98158333
														DAGGPRPESPVPAGR	1	2	1	N-Term(iTRAQ8plex)	0.879	0.521	0.714	2.78	-	0	1766.94083	3	589.6517944	-2.456929595	13.13650333
13559514	PI4K2A	phosphatidylinositol 4-kinase type 2-alpha	NP_060895.1	7.93	3	3	4	479	54.0	8.29	0.485	0.389	0.625																
														AYPFYWAWLPQAK	1	1	1	N-Term(iTRAQ8plex); K13(iTRAQ8plex)	0.836	0.602	0.834	3.5	-	0	2249.22654	3	750.4136963	-4.555202711	47.39253167
														ERNEFPEDPEFEAVVR	1	1	1	N-Term(iTRAQ8plex)	0.404	0.389	0.625	3.11	-	1	2267.11814	3	756.3775635	-2.877715195	31.78390167
														LVVLDYIIR	2	1	1	N-Term(iTRAQ8plex)	0.485	0.255	0.399	2.87	24	0	1407.89507	2	704.4511719	5.224700457	56.71330833
13569962	RAB1B	ras-related protein Rab-1B	NP_112243.1	49.25	3	8	47	201	22.2	5.73	2.052	1.439	0.652																
														NATNVEQAFMTMAAEIK	10	2	1	N-Term(iTRAQ8plex); K17(iTRAQ8plex)	2.005	1.583	0.573	6.13	86	0	2477.29117	2	1239.151978	-0.905358609	50.10906667
														NATNVEQAFMTMAAEIK	2	2	1	N-Term(iTRAQ8plex); M10(Oxidation); K17(iTRAQ8plex)	-	-	-	5.13	50	0	2493.28505	2	1239.151978	-0.905358609	50.10906667
														MGPGAASGGERPNLK	4	2	1	N-Term(iTRAQ8plex); K15(iTRAQ8plex)	2.197	1.49	0.71	4.03	54	0	2050.12798	2	1025.567627	-2.17743175	16.090265
														MGPGAASGGERPNLK	1	2	1	N-Term(iTRAQ8plex); M1(Oxidation); K15(iTRAQ8plex)	2.25	1.202	0.796	3.97	-	0	2066.12539	2	1025.567627	-2.17743175	16.090265
														KVVDNTTAK	1	2	1	N-Term(iTRAQ8plex); K1(iTRAQ8plex); K9(iTRAQ8plex)	1.722	1.39	0.619	3.04	-	1	1888.16709	3	630.0605469	2.132266274	13.84216
														IQIWDTAGQER	14	3	9	N-Term(iTRAQ8plex)	-	-	-	3.41	41	0	1620.85991	2	810.9328003	-3.971558752	26.96297167
														LLIIGDSGVGK	22	2	5	N-Term(iTRAQ8plex); K11(iTRAQ8plex)	-	-	-	4.71	52	0	1680.04277	3	560.6833496	-9.598441174	57.819735
														FADDTYTESYISTIGVDFK	7	5	2	N-Term(iTRAQ8plex); K19(iTRAQ8plex)	-	-	-	5.83	34	0	2780.43198	3	927.4765625	0.871434618	51.39833167
														EFADSLGIPFLETSAK	11	3	2	N-Term(iTRAQ8plex); K16(iTRAQ8plex)	-	-	-	5	58	0	2333.27524	2	1167.139893	-5.332752049	48.565745
														TIELDGK	2	9	2	N-Term(iTRAQ8plex); K7(iTRAQ8plex)	-	-	-	3.18	28	0	1383.83265	2	692.4209595	3.086369702	15.34975333
														QWLQEIDR	4	4	2	N-Term(iTRAQ8plex)	-	-	-	2.96	31	0	1391.75395	2	696.3806152	-3.260239525	41.98017333
														YASENVNK	5	4	2	N-Term(iTRAQ8plex); K8(iTRAQ8plex)	-	-	-	2.81	40	0	1532.85015	2	766.9287109	-1.784939649	13.45385667
13654237	PRKDC	DNA-dependent protein kinase catalytic subunit isoform 1	NP_008835.5	15.55	49	49	163	4128	468.8	7.12	1.074	1.250	0.969																
														LAGANPAVITCDELLLGHEK	17	2	1	N-Term(iTRAQ8plex); C11(Methylthio); K20(iTRAQ8plex)	1.035	1.479	0.938	7.45	58	0	2718.48026	2	1359.746948	3.188378531	57.66669333
														SLEYCSTASIDSENPPDLNK	2	2	1	N-Term(iTRAQ8plex); C5(Methylthio); K20(iTRAQ8plex)	0.559	0.592	0.366	6.72	38	0	2837.39096	3	946.4685059	4.078404517	35.31220333
														NLDLAVLELMQSSVDNTK	4	2	1	N-Term(iTRAQ8plex); K18(iTRAQ8plex)	0.749	1.104	1.388	6.17	55	0	2598.42899	3	866.8145142	0.784293308	56.645755
														DPTVHDDVLELEMDELNR	2	2	1	N-Term(iTRAQ8plex)	0.812	1.212	0.791	5.8	58	0	2444.18845	3	815.401001	-1.354895656	39.72103167
														LLLQGEADQSLLTFIDK	4	2	1	N-Term(iTRAQ8plex); K17(iTRAQ8plex)	1.098	1.129	0.937	5.8	83	0	2512.4666	2	1256.736938	7.271353626	55.73383333
														SLGTIQQCCDAIDHLCR	6	2	1	N-Term(iTRAQ8plex); C8(Methylthio); C9(Methylthio); C16(Methylthio)	1.571	1.022	0.93	5.55	80	0	2318.0156	3	773.3433838	-1.728281214	70.88703833
														IMEFTTTLLNTSPEGWK	4	2	1	N-Term(iTRAQ8plex); K17(iTRAQ8plex)	1.034	1.155	0.727	5.54	46	0	2576.38419	3	859.4662476	-1.906499131	56.88704
														QLFSSLFSGILK	2	2	1	N-Term(iTRAQ8plex); K12(iTRAQ8plex)	1.458	1.19	1.029	5.43	41	0	1948.15427	3	650.0562744	-9.507105314	80.001165
														MDPMNIWDDIITNR	5	2	1	N-Term(iTRAQ8plex)	1.055	1.222	0.863	5.31	66	0	2038.02115	3	680.0119019	8.410617518	59.235175
														SDPGLLTNTMDVFVK	2	2	1	N-Term(iTRAQ8plex); K15(iTRAQ8plex)	0.955	0.718	0.946	4.95	42	0	2245.23868	3	749.0844116	1.242769111	42.59839
														AQEPESGLSEETQVK	2	2	1	N-Term(iTRAQ8plex); K15(iTRAQ8plex)	1.118	1.176	0.777	4.81	67	0	2240.19585	2	1120.601563	4.092088648	23.80877333
														NLLIFENLIDLK	14	2	1	N-Term(iTRAQ8plex); K12(iTRAQ8plex)	1.27	1.921	1.237	4.8	62	0	2053.2396	2	1027.123291	-6.076198092	81.52495167
														LLALNSLYSPK	2	2	1	N-Term(iTRAQ8plex); K11(iTRAQ8plex)	1.384	1.106	1.01	4.77	53	0	1827.10307	2	914.0551758	-9.269513982	67.73943833
														LSDFNDITNMLLLK	9	2	1	N-Term(iTRAQ8plex); K14(iTRAQ8plex)	0.83	1.377	0.982	4.74	78	0	2245.273	2	1123.14563	5.216197912	59.92098667
														AVAFFLESIAMHDIIAAEK	2	2	1	N-Term(iTRAQ8plex); K19(iTRAQ8plex)	-	-	-	4.68	37	0	2684.49502	4	671.8792114	0.281067061	67.26319667
														YNFPVEVEVPMER	2	2	1	N-Term(iTRAQ8plex)	1.168	1.53	0.991	4.65	42	0	1912.96794	2	956.9876099	-5.304946941	42.70768333
														GGSWIQEINVAEK	2	2	1	N-Term(iTRAQ8plex); K13(iTRAQ8plex)	1.135	2.488	1.674	4.57	27	0	2039.12729	3	680.3806152	-5.354666634	54.71802333
														CGAALAGHQLIR	10	2	1	N-Term(iTRAQ8plex); C1(Methylthio)	0.999	1.29	1.032	4.44	70	0	1559.83756	3	520.6177979	-4.097856232	26.5157
														NELEIPGQYDGR	3	2	1	N-Term(iTRAQ8plex)	1.053	1.072	0.744	4.35	23	0	1694.86894	2	847.9318848	-5.108626474	20.05801333
														DLLLNTMSQEEK	2	1	1	N-Term(iTRAQ8plex); K12(iTRAQ8plex)	0.942	1.192	1.007	4.21	37	0	2029.09	3	677.0348511	-9.665085011	47.40240667
														CLMDQATDPNILGR	4	2	1	N-Term(iTRAQ8plex); C1(Methylthio)	1.248	1.228	0.947	4.14	30	0	1896.92082	2	948.9640503	-3.989799662	40.719175
														TVGALQVLGTEAQSSLLK	1	2	1	N-Term(iTRAQ8plex); K18(iTRAQ8plex)	0.818	0.726	1	4.13	-	0	2423.42173	3	808.4787598	-4.656743399	46.90287667
														NCISTVVHQGLIR	3	2	1	N-Term(iTRAQ8plex); C2(Methylthio)	2.359	1.573	0.989	4.06	41	0	1789.96213	3	597.3255615	-5.447589933	37.61924333
														APPCEYKDWLTK	1	2	1	N-Term(iTRAQ8plex); C4(Methylthio); K7(iTRAQ8plex); K12(iTRAQ8plex)	1.795	1.563	0.993	4.04	-	1	2409.30385	4	603.0814209	-1.460416146	34.5805
														TRDDWLVSWVQSYCR	8	2	1	N-Term(iTRAQ8plex); C14(Methylthio)	0.648	1.626	1.241	4.02	53	1	2264.10422	3	755.3729248	6.527466326	64.63067167
														ETGLMYSIMVHALR	2	2	1	N-Term(iTRAQ8plex)	0.923	0.766	1.075	3.88	32	0	1925.04068	3	642.3517456	6.027024003	53.97227
														ATQQQHDFTLTQTADGR	4	2	1	N-Term(iTRAQ8plex)	2.147	1.972	1.448	3.88	39	0	2222.10257	3	741.3739014	-1.449276843	18.67957667
														LGASLAFNNIYR	2	2	1	N-Term(iTRAQ8plex)	1.146	2.056	1.324	3.79	28	0	1642.91057	2	821.9589233	-6.878792138	31.661875
														KNILEESLCELVAK	5	2	1	N-Term(iTRAQ8plex); K1(iTRAQ8plex); C9(Methylthio); K14(iTRAQ8plex)	-	0.506	0.362	3.73	37	1	2547.44228	4	637.6160278	-9.056476782	60.17918167
														NKLQYFMEQFYGIIR	2	2	1	N-Term(iTRAQ8plex); K2(iTRAQ8plex)	1.137	1.142	0.965	3.59	28	1	2558.42099	3	853.4785156	6.250857478	64.22644333
														LQSVQALTEIQEFISFISK	2	2	1	N-Term(iTRAQ8plex); K19(iTRAQ8plex)	-	-	-	3.57	46	0	2789.59047	4	698.1530762	-0.177980426	68.55272
														ECSPWMSDFKVEFLR	2	2	1	N-Term(iTRAQ8plex); C2(Methylthio); K10(iTRAQ8plex)	0.743	1.386	1.095	3.53	-	1	2528.26552	4	632.8218384	2.306456964	56.14755333
														SLGPPQGEEDSVPR	2	2	1	N-Term(iTRAQ8plex)	1.366	1.573	0.961	3.44	55	0	1771.90935	2	886.458313	-1.976367154	20.81472
														MDPMNIWDDIITNR	2	2	1	N-Term(iTRAQ8plex); M1(Oxidation)	1.307	1.907	1.449	3.32	55	0	2054.00273	3	680.0119019	8.410617518	59.235175
														GYGLFAGPCK	2	2	1	N-Term(iTRAQ8plex); C9(Methylthio); K10(iTRAQ8plex)	0.801	1.029	1.068	3.26	34	0	1666.89347	3	556.3026733	1.748443791	36.039075
														LGNPIVPLNIR	1	2	1	N-Term(iTRAQ8plex)	1.007	0.787	0.594	3.21	-	0	1509.94597	2	755.4766235	2.701520712	38.72197167
														EIFNFVLK	2	2	1	N-Term(iTRAQ8plex); K8(iTRAQ8plex)	1.341	1.551	1.059	3.16	43	0	1617.97441	2	809.4908447	-4.974409244	69.33913167
														AALSALESFLK	2	2	1	N-Term(iTRAQ8plex); K11(iTRAQ8plex)	0.815	0.364	0.281	3.11	32	0	1758.056	3	586.6901855	-3.51405721	48.05555833
														SIGEYDVLR	2	2	1	N-Term(iTRAQ8plex)	1.087	1.137	0.835	3.04	36	0	1355.74443	2	678.3758545	-2.079252961	29.49820167
														LLEEALLR	1	2	1	N-Term(iTRAQ8plex)	0.705	1.353	0.987	3	-	0	1260.77837	2	630.8928223	-3.61156954	46.78632667
														LCYDAFTENMAGENQLLER	1	2	1	N-Term(iTRAQ8plex); C2(Methylthio)	1.303	1.158	1.152	2.97	-	0	2567.18369	3	856.3994141	-1.781470691	55.06946667
														GIFTSEIGTK	2	2	1	N-Term(iTRAQ8plex); K10(iTRAQ8plex)	0.757	1.252	0.798	2.95	27	0	1660.97513	3	554.329895	1.270644306	36.73710667
														LACDVDQVTR	4	2	1	N-Term(iTRAQ8plex); C3(Methylthio)	1.045	1.147	0.9	2.93	40	0	1469.73418	2	735.3707275	-3.567884555	31.54599333
														CFFLSK	1	2	1	N-Term(iTRAQ8plex); C1(Methylthio); K6(iTRAQ8plex)	1.191	1.569	1.025	2.92	-	0	1398.75994	2	699.883606	-9.613519517	37.39197167
														DQNILLGTTYR	1	2	1	N-Term(iTRAQ8plex)	0.925	1.2	1.206	2.85	-	0	1597.89079	2	799.4490356	3.53801967	29.495485
														DLPSWMK	2	2	1	N-Term(iTRAQ8plex); K7(iTRAQ8plex)	0.969	1.115	0.891	2.81	27	0	1484.8383	2	742.9227905	-0.583811254	27.21506667
														YNFPVEVEVPMER	1	2	1	N-Term(iTRAQ8plex); M11(Oxidation)	0.866	0.726	0.821	2.71	-	0	1928.9738	2	956.9876099	-5.304946941	42.70768333
														LLQIIER	2	2	1	N-Term(iTRAQ8plex)	1.171	1.222	0.939	-	38	0	1188.75884	2	594.8830566	-2.477069915	46.99809833
														FYGELALK	1	2	1	N-Term(iTRAQ8plex); K8(iTRAQ8plex)	1.181	1.338	1.17	-	32	0	1548.92777	3	516.9807739	2.044786986	48.75563333
														ELSIAIR	1	2	1	N-Term(iTRAQ8plex)	1.173	1.128	0.968	-	30	0	1105.68144	2	553.3443604	-6.183713141	26.99265833
														LPPDVLR	1	2	1	N-Term(iTRAQ8plex)	0.823	0.909	0.726	-	19	0	1113.69109	2	557.3491821	-2.050534671	27.36317
13654274	FAHD1	acylpyruvase FAHD1, mitochondrial isoform 2	NP_112485.1	6.25	1	1	1	224	24.8	7.39	1.169	0.990	0.814																
														SFTASCPVSAFVPK	1	3	1	N-Term(iTRAQ8plex); C6(Methylthio); K14(iTRAQ8plex)	1.169	0.99	0.814	3.93	-	0	2095.11362	3	699.0427246	-1.933891239	37.03949
13775198	SH3BGRL3	SH3 domain-binding glutamic acid-rich-like protein 3	NP_112576.1	31.18	2	2	6	93	10.4	4.93	0.483	1.143	2.056																
														IQYQLVDISQDNALRDEMR	4	1	1	N-Term(iTRAQ8plex)	0.516	1.428	2.125	3.59	-	1	2611.34323	3	871.1192627	-0.764535292	41.21404333
														VYSTSVTGSR	1	1	1	N-Term(iTRAQ8plex)	0.385	1.156	2.49	2.91	-	0	1360.73955	2	680.8734131	1.57873847	20.15668333
														IQYQLVDISQDNALRDEMR	1	1	1	N-Term(iTRAQ8plex); M18(Oxidation)	0.53	0.913	1.698	2.8	-	1	2627.33292	3	871.1192627	-0.764535292	41.21404333
13899231	MRPL9	39S ribosomal protein L9, mitochondrial isoform 1	NP_113608.1	4.49	1	1	4	267	30.2	10.08	0.726	0.619	0.563																
														NLGVVVAPHTLK	4	1	1	N-Term(iTRAQ8plex); K12(iTRAQ8plex)	0.726	0.619	0.563	4.15	31	0	1856.14377	3	619.3861084	-7.563721571	25.50549
13904866	RPL28	60S ribosomal protein L28 isoform 2	NP_000982.2	27.01	4	4	10	137	15.7	12.02	1.260	1.308	1.370																
														QTYSTEPNNLK	1	5	1	N-Term(iTRAQ8plex); K11(iTRAQ8plex)	1.635	1.349	1.226	3.16	-	0	1903.02298	3	635.0125122	-7.954674767	14.93515
														NCSSFLIK	5	5	1	N-Term(iTRAQ8plex); C2(Methylthio); K8(iTRAQ8plex)	0.989	1.652	1.781	3.08	33	0	1565.85202	2	783.4301758	-6.972146796	30.70292833
														TVGVEPAADGK	3	5	1	N-Term(iTRAQ8plex); K11(iTRAQ8plex)	1.955	1.443	2.038	2.69	35	0	1651.93338	3	551.315979	-8.576337533	14.61741667
														ATLSSIR	1	3	1	N-Term(iTRAQ8plex)	1.263	1.269	1.174	-	25	0	1051.63762	2	526.3224487	-3.518758733	17.86163667
13904870	RPS5	40S ribosomal protein S5	NP_001000.2	37.25	7	7	106	204	22.9	9.72	1.516	2.672	2.279																
														WSTDDVQINDISLQDYIAVK	7	1	1	N-Term(iTRAQ8plex); K20(iTRAQ8plex)	1.66	2.323	2.297	6.62	73	0	2931.58354	2	1466.29541	9.381411817	47.78878667
														TIAECLADELINAAK	33	1	1	N-Term(iTRAQ8plex); C5(Methylthio); K15(iTRAQ8plex)	1.811	3.415	2.951	5.87	74	0	2229.19406	2	1115.108643	0.91791991	55.253515
														VNQAIWLLCTGAR	30	1	1	N-Term(iTRAQ8plex); C9(Methylthio)	1.479	2.951	2.521	5.47	62	0	1794.96306	2	897.9840088	-2.975743008	60.08391667
														RVNQAIWLLCTGAR	28	1	1	N-Term(iTRAQ8plex); C10(Methylthio)	1.529	2.678	2.087	3.92	60	1	1951.06656	3	651.0270386	-0.327937219	53.83198333
														QAVDVSPLRR	3	1	1	N-Term(iTRAQ8plex)	0.988	1.543	1.252	3.06	35	1	1444.84624	2	722.9267578	-5.233979088	15.60296
														AQCPIVER	3	1	1	N-Term(iTRAQ8plex); C3(Methylthio)	1.364	1.799	1.389	2.75	23	0	1265.65837	2	633.3319092	-6.518799021	17.45254167
														WSTDDVQINDISLQDYIAVK	1	1	1	N-Term(iTRAQ8plex); Y16(iTRAQ8plex); K20(iTRAQ8plex)	-	-	-	-	33	0	3235.7643	2	1466.29541	9.381411817	47.78878667
														LTNSMMMHGR	1	1	1	N-Term(iTRAQ8plex)	0.805	1.354	1.172	-	31	0	1481.74028	2	741.3737793	4.974335813	19.09569833
13994151	PDLIM1	PDZ and LIM domain protein 1	NP_066272.1	25.84	5	5	20	329	36.0	7.02	0.675	1.208	1.002																
														AALANLCIGDVITAIDGENTSNMTHLEAQNR	8	1	1	N-Term(iTRAQ8plex); C7(Methylthio)	-	-	-	5.09	51	0	3605.80075	3	1202.605103	9.501930229	66.97540167
														MNLASEPQEVLHIGSAHNR	1	2	1	N-Term(iTRAQ8plex)	0.478	0.965	0.948	4.99	-	0	2407.23256	4	602.5635986	-5.353304429	32.66080167
														GHFFVEDQIYCEK	2	2	1	N-Term(iTRAQ8plex); C11(Methylthio); K13(iTRAQ8plex)	0.855	0.92	1.002	4.34	23	0	2269.10868	3	757.0410767	-6.842144535	33.53290167
														GCTDNLTLTVAR	4	1	1	N-Term(iTRAQ8plex); C2(Methylthio)	0.782	1.328	1.092	4.21	46	0	1613.8328	3	538.6157837	2.165976688	30.70745167
														AALANLCIGDVITAIDGENTSNMTHLEAQNR	3	1	1	N-Term(iTRAQ8plex); C7(Methylthio); M23(Oxidation)	-	-	-	3.77	35	0	3621.7726	3	1202.605103	9.501930229	66.97540167
														DFEQPLAISR	2	1	1	N-Term(iTRAQ8plex)	0.675	1.208	0.969	2.77	31	0	1479.80718	2	740.4072266	-2.537354231	28.954695

13994257	MRPS21	28S ribosomal protein S21, mitochondrial	NP_114107.1	29.89	2	2	6	87	10.7	10.21	1.213	0.489	0.593																
														TVMVQEGNVESAYR	2	1	1	N-Term(iTRAQ8plex)	1.275	0.654	0.621	5.21	32	0	1886.9655	2	943.9863892	3.757567537	27.15954333
														ILTMDGLIEDIK	4	1	1	N-Term(iTRAQ8plex); K12(iTRAQ8plex)	1.206	0.435	0.591	4.26	54	0	1969.15079	3	657.0551147	0.385794874	47.34897
13994261	MRPL32	39S ribosomal protein L32, mitochondrial precursor	NP_114109.1	12.23	1	1	2	188	21.4	9.73	1.150	0.554	0.454																
														APTIETVVLYTGETPSEQDQGKR	2	1	1	N-Term(iTRAQ8plex); K22(iTRAQ8plex)	1.15	0.554	0.454	5.13	44	1	3127.66513	3	1043.226563	-2.582453573	35.34614333
14028877	MRPS25	28S ribosomal protein S25, mitochondrial	NP_071942.1	9.83	1	1	2	173	20.1	8.82	1.010	1.023	0.785																
														VMTVNYNTHGELGEGAR	2	1	1	N-Term(iTRAQ8plex)	1.01	1.023	0.785	4.83	37	0	2152.07389	3	718.02948	-0.940770949	23.01790167
14043022	MARS	methionine--tRNA ligase, cytoplasmic	NP_004981.2	5.78	3	3	4	900	101.1	6.16	2.717	5.210	4.093																
														GLESLPPLRPQQNPVLPVAGER	1	2	1	N-Term(iTRAQ8plex)	3.753	7.847	6.277	3.85	-	0	2671.51224	3	891.1755981	-2.826158267	32.73792
														RLEEWLGR	2	2	1	N-Term(iTRAQ8plex)	1.967	3.46	2.669	2.76	29	1	1362.77076	3	454.9284363	-6.46896191	29.14208667
														AEVLISTVGPEDCVVPFLTRPK	1	1	1	N-Term(iTRAQ8plex); C13(Methylthio); K22(iTRAQ8plex)	-	-	-	2.71	-	0	3024.68447	4	756.9265747	4.019145881	54.85652667
14043044	CCR9	C-C chemokine receptor type 9 isoform B	NP_006632.2	1.68	1	1	1	357	40.7	8.29	1.214	3.646	3.396																
														REGSLK	1	2	1	N-Term(iTRAQ8plex); K6(iTRAQ8plex)	1.214	3.646	3.396	2.82	-	1	1297.79221	3	433.2689209	-9.717930649	26.52862167
14043072	HNRNPA2B1	heterogeneous nuclear ribonucleoproteins A2/B1 isoform B1	NP_112533.1	64.87	18	21	232	353	37.4	8.95	1.453	0.652	0.670																
														GFGDGYNGYGGGPGGGNFGGSPGYGGGR	6	2	1	N-Term(iTRAQ8plex)	1.501	0.292	0.591	7.46	142	0	2799.236	2	1400.134521	5.981951724	29.70147167
														GFGFVTFSSMAEVDAAMAARPHSIDGR	9	2	1	N-Term(iTRAQ8plex); M17(Oxidation)	-	-	0.732	5.85	40	0	3147.51821	4	787.6367188	-1.411007041	45.03397833
														DYFEEYGKIDTIEIITDR	20	2	1	N-Term(iTRAQ8plex); K8(iTRAQ8plex)	2.143	0.947	0.891	5.85	37	1	2828.48178	3	943.4955444	-3.320255231	48.27002833
														KLFIGGLSFETTEESLR	17	2	1	N-Term(iTRAQ8plex); K1(iTRAQ8plex)	1.258	0.624	0.607	5.76	62	1	2535.42422	3	845.8137207	-0.521585054	43.82644
														GFGFVTFSSMAEVDAAMAARPHSIDGR	13	2	1	N-Term(iTRAQ8plex)	0.625	1.376	0.706	5.71	34	0	3131.52749	4	787.6367188	-1.411007041	45.03397833
														LFIGGLSFETTEESLR	8	2	1	N-Term(iTRAQ8plex)	1.308	0.68	0.695	5.67	84	0	2103.12174	2	1052.064697	-2.607261225	53.25587667
														NMGGPYGGGNYGPGGSGGSGGYGGR	4	2	1	N-Term(iTRAQ8plex)	1.249	0.582	0.846	5.56	68	0	2494.10721	3	837.37146	-2.354355442	20.67226667
														NMGGPYGGGNYGPGGSGGSGGYGGR	8	2	1	N-Term(iTRAQ8plex); M2(Oxidation)	0.736	0.339	0.331	5.46	86	0	2510.09983	3	837.37146	-2.354355442	20.67226667
														GFGFVTFDDHDPVDK	31	2	1	N-Term(iTRAQ8plex); K15(iTRAQ8plex)	1.539	0.723	0.7	4.88	70	0	2304.16484	2	1152.58606	-4.725694609	36.27978667
														GGGGNFGPGPGSNFR	19	2	1	N-Term(iTRAQ8plex)	1.403	0.594	0.661	4.87	76	0	1681.84037	2	841.4180298	-3.626022904	16.00587167
														QEMQEVQSSR	8	2	1	N-Term(iTRAQ8plex)	1.514	0.641	0.582	4.7	45	0	1525.75486	3	514.5877686	-2.856422573	8.452671667
														IDTIEIITDR	17	2	1	N-Term(iTRAQ8plex)	1.409	0.606	0.611	4.48	57	0	1492.84687	3	498.2871399	-3.747787125	43.16993
														YHTINGHNAEVR	12	2	1	N-Term(iTRAQ8plex)	1.899	0.58	1.316	4.38	53	0	1714.89127	2	857.9483032	-1.954338761	10.47706
														LTDCVVMRDPASK	4	2	1	N-Term(iTRAQ8plex); C4(Methylthio); K13(iTRAQ8plex)	1.143	0.751	0.656	3.88	45	1	2089.09218	2	1045.05481	-1.976820039	30.283905
														NYYEQWGK	6	2	1	N-Term(iTRAQ8plex); K8(iTRAQ8plex)	1.498	0.719	0.753	3.77	33	0	1695.89031	2	848.4470825	-4.825133677	23.839035
														DYFEEYGK	19	2	1	N-Term(iTRAQ8plex); K8(iTRAQ8plex)	1.329	0.653	0.645	3.69	46	0	1658.84245	2	829.925415	-5.219203376	23.36297167
														LTDCVVMRDPASK	2	2	1	N-Term(iTRAQ8plex); C4(Methylthio); M7(Oxidation); K13(iTRAQ8plex)	1.643	1.634	0.842	3.6	36	1	2105.09682	2	1045.05481	-1.976820039	30.283905
														TLETVPLER	1	1	1	N-Term(iTRAQ8plex)	2.031	0.687	0.968	3.18	-	0	1361.79009	2	681.3986816	-3.028835935	25.17943167
														GFGFVTFDDHDPVDKIVLQK	1	2	1	N-Term(iTRAQ8plex); K15(iTRAQ8plex); K20(iTRAQ8plex)	-	-	-	3.12	-	1	3189.76484	5	638.7587891	-1.994660366	49.29866667
														LTDCVVMR	6	2	1	N-Term(iTRAQ8plex); C4(Methylthio)	0.976	0.45	0.527	3.11	35	0	1286.65386	2	643.8305664	-2.655032263	37.41133667
														GGNFGFGDSR	4	2	1	N-Term(iTRAQ8plex)	1.753	0.964	0.885	2.84	31	0	1317.64714	2	659.3272095	-1.372882488	14.525955
														QEMQEVQSSR	1	2	1	N-Term(iTRAQ8plex); M3(Oxidation)	0.988	-	-	-	49	0	1541.74875	3	514.5877686	-2.856422573	8.452671667
														NMGGPYGGGNYGPGGSGGSGGYGGR	3	2	1	N-Term(iTRAQ8plex); M2(Oxidation); Y11(iTRAQ8plex)	2.102	0.546	0.476	-	27	0	2814.30507	3	837.37146	-2.354355442	20.67226667
														IFVGGIKEDTEEHHLR	17	4	2	N-Term(iTRAQ8plex); K7(iTRAQ8plex)	-	-	-	5.62	67	1	2488.37309	2	1244.690186	-1.525826757	26.84572333
														EDTEEHHLR	6	6	2	N-Term(iTRAQ8plex)	-	-	-	4.06	49	0	1469.72531	3	490.5799561	-2.263270493	8.073406667
														IFVGGIK	9	9	4	N-Term(iTRAQ8plex); K7(iTRAQ8plex)	-	-	-	3.12	27	0	1341.87183	3	447.9621277	0.282073617	31.44851833
														KLFVGGIK	9	9	4	N-Term(iTRAQ8plex); K1(iTRAQ8plex); K8(iTRAQ8plex)	-	-	-	3.5	30	1	1774.17019	2	887.5895996	0.079642084	21.37640667
14110414	HNRNPD	heterogeneous nuclear ribonucleoprotein D0 isoform c	NP_002129.2	20.92	5	6	34	306	32.8	8.16	0.975	0.805	0.761																
														EYFGGFGEVESIELPMDNK	6	4	1	N-Term(iTRAQ8plex); K19(iTRAQ8plex)	1.127	0.797	0.834	5.48	56	0	2769.38834	3	923.8009644	-0.686607785	50.16460333
														EYFGGFGEVESIELPMDNK	6	4	1	N-Term(iTRAQ8plex); M16(Oxidation); K19(iTRAQ8plex)	1.113	1.013	1.381	5.15	55	0	2785.37381	3	923.8009644	-0.686607785	50.16460333
														IFVGGLSPDTPEEK	5	4	1	N-Term(iTRAQ8plex); K14(iTRAQ8plex)	0.975	0.931	0.791	4.82	61	0	2097.15488	2	1049.081543	-6.218890576	26.942335
														GFCFITFK	4	4	1	N-Term(iTRAQ8plex); C3(Methylthio); K8(iTRAQ8plex)	0.777	0.713	0.704	3.6	45	0	1616.86748	2	808.9373779	-7.07730273	72.68760167
														IREYFGGFGEVESIELPMDNK	1	4	1	N-Term(iTRAQ8plex); K21(iTRAQ8plex)	-	-	-	3.55	-	1	3038.58291	4	760.4011841	2.460077067	52.388965
														HSEAATAQREEWK	2	2	1	N-Term(iTRAQ8plex); K13(iTRAQ8plex)	-	-	-	2.83	29	1	2151.1288	4	538.5376587	-5.358309716	12.17080833
														GFGFVLFK	10	6	2	N-Term(iTRAQ8plex); K8(iTRAQ8plex)	-	-	-	3.67	39	0	1522.91704	2	761.9621582	-4.715630308	69.43356
														FGEVVDCTIK	26	4	3	N-Term(iTRAQ8plex); C7(Methylthio); K10(iTRAQ8plex)	-	-	-	4.38	53	0	1764.93157	2	882.9761353	-1.970833198	34.73064167
14141170	MTA2	metastasis-associated protein MTA2	NP_004730.2	2.84	2	2	3	668	75.0	9.66	1.101	1.037	1.009																
														DITLFHAMDTLQR	2	2	1	N-Term(iTRAQ8plex)	1.127	0.979	1.329	3.27	25	0	1864.98282	3	622.3324585	-3.484783688	35.84948167
														VVCLFR	1	1	1	N-Term(iTRAQ8plex); C3(Methylthio)	1.075	1.097	0.766	-	28	0	1086.61077	2	543.809021	0.18688586	33.78200833
14149680	ESYT1	extended synaptotagmin-1 isoform 2	NP_056107.1	14.22	12	12	38	1104	122.8	5.83	0.700	0.450	0.747																
														FFLQDPQSQELDVQVK	4	2	1	N-Term(iTRAQ8plex); K16(iTRAQ8plex)	0.485	0.307	0.442	6.78	61	0	2529.38504	3	843.7998657	1.610121788	39.34427833
														IHVLEAQDLIAK	7	2	1	N-Term(iTRAQ8plex); K12(iTRAQ8plex)	0.766	0.624	0.888	5.43	45	0	1958.17746	3	653.3973389	-6.152633591	35.04370167
														AVYSTNCPVWEEAFR	3	2	1	N-Term(iTRAQ8plex); C7(Methylthio)	0.784	0.381	0.948	5.2	67	0	2122.00981	2	1061.508545	2.747830719	45.05457
														FEWELPLDEAQR	4	2	1	N-Term(iTRAQ8plex)	0.701	0.393	0.689	4.55	56	0	1836.93486	2	918.9710693	-4.659527293	47.586125
														ALTLGALTLPLAR	4	2	1	N-Term(iTRAQ8plex)	0.514	0.435	0.708	4.36	48	0	1614.02121	3	538.6785889	-2.737230713	52.679785
														LTLWYYSEER	2	2	1	N-Term(iTRAQ8plex)	0.701	0.45	0.747	4.27	39	0	1663.87248	2	832.4398804	5.50091565	40.26586333
														SAELAAALLSIYMER	4	2	1	N-Term(iTRAQ8plex)	0.701	0.387	0.649	3.89	60	0	1942.06839	3	648.0276489	3.209066871	66.29279
														LLAETVAPAVR	4	2	1	N-Term(iTRAQ8plex)	0.642	0.402	0.519	3.27	30	0	1443.8781	2	722.442688	-3.886715534	34.057535
														GSNPHLQTFTFTR	2	2	1	N-Term(iTRAQ8plex)	0.619	0.52	0.674	3.08	-	0	1809.9523	3	603.9889526	-1.467795498	21.993535
														KLVSIVHGCR	2	2	1	N-Term(iTRAQ8plex); K1(iTRAQ8plex); C9(Methylthio)	0.994	1.335	1.548	2.88	35	1	1766.03359	4	442.263855	-3.036328502	28.355005
														LTTVLNSGFLDEWLTLEDVPSGR	1	2	1	N-Term(iTRAQ8plex)	-	-	-	-	32	0	2866.52927	3	956.1812744	5.295146415	68.86172667
														IHLLAAR	1	2	1	N-Term(iTRAQ8plex)	0.538	0.371	0.999	-	32	0	1097.7092	3	366.574585	-0.436971091	26.67980333
14149734	CORO1B	coronin-1B	NP_065174.1	8.18	2	2	3	489	54.2	5.88	0.625	2.523	2.546																
														KSDLFQDDLYPDTAGPEAALEAEEWVSGR	2	1	1	N-Term(iTRAQ8plex); K1(iTRAQ8plex)	-	-	-	4.88	67	1	3817.91379	3	1273.309448	3.135266331	55.01347333
														KCEPIVMTVPR	1	1	1	N-Term(iTRAQ8plex); K1(iTRAQ8plex); C2(Methylthio)	0.625	2.523	2.546	3	-	1	1927.06986	3	643.0281372	-4.636017147	30.516255
14150017	TMEM126A	transmembrane protein 126A isoform 1	NP_115649.1	24.10	2	2	4	195	21.5	9.26	-	-	-																
														SGLTGLVIGGLYPVFLAIPVNGGLAAR	2	2	1	N-Term(iTRAQ8plex)	-	-	-	3.24	36	0	2929.72794	3	977.2474976	3.334140911	69.47113167
														IAAGLPMAGIPFLTTDLTYR	2	1	1	N-Term(iTRAQ8plex)	-	-	-	2.69	41	0	2425.34507	3	809.119873	-0.690792167	65.13245
14150100	NTPCR	cancer-related nucleoside-triphosphatase	NP_115700.1	12.63	2	2	3	190	20.7	9.54	0.835	0.849	1.341																
														HVFLTGPPGVGK	2	1	1	N-Term(iTRAQ8plex); K12(iTRAQ8plex)	0.905	0.956	1.316	4.76	39	0	1817.07639	3	606.3636475	-7.161045769	20.991135
														IGFDVVTLSGTR	1	1	1	N-Term(iTRAQ8plex)	0.77	0.754	1.368	2.76	-	0	1568.89531	2	784.9512939	0.197572536	37.200965
14150124	LLPH	protein LLP homolog	NP_115714.1	12.40	1	1	1	129	15.2	10.37	2.255	1.275	1.359																
														TLLDQHGQYPIWMNQR	1	1	1	N-Term(iTRAQ8plex)	2.255	1.275	1.359	3.23	-	0	2304.17777	3	768.7307739	-3.625055658	32.362545
14150155	VPS25	vacuolar protein-sorting-associated protein 25	NP_115729.1	8.52	1	1	2	176	20.7	6.34	0.907	1.451	1.759																
														KLPVESIQIVLEELR	2	2	1	N-Term(iTRAQ8plex); K1(iTRAQ8plex)	0.907	1.451	1.759	4.91	47	1	2374.44486	3	792.1531372	-3.437826268	61.48135
14165270	MRPL13	39S ribosomal protein L13, mitochondrial	NP_054797.2	8.43	1	1	6	178	20.7	9.16	1.335	1.543	1.216																
														RLDEYTQEEIDAFPR	6	1	1	N-Term(iTRAQ8plex)	1.335	1.543	1.216	4.81	44	1	2186.09873	3	729.3710938	-2.032524855	33.320215
14165466	PTBP1	polypyrimidine tract-binding protein 1 isoform c	NP_114368.1	38.98	10	11	44	531	57.2	9.17	1.170	0.914	0.931																
														IAIPGLAGAGNSVLLVSNLNPER	2	5	1	N-Term(iTRAQ8plex)	0.769	0.677	0.575	7.24	60	0	2579.48014	3	860.49823	-0.854671669	55.5129
														MALIQMGSVEEAVQALIDLHNHDLGENHHLR	3	5	1	N-Term(iTRAQ8plex)	-	-	-	7.16	38	0	3794.93618	4	949.489502	-0.306557856	64.93449
														NNQFQALLQYADPVSAQHAK	4	5	1	N-Term(iTRAQ8plex); K20(iTRAQ8plex)	1.078	0.685	0.824	6.1	61	0	2851.5322	3	951.182251	0.367654865	42.65269833
														GQPIYIQFSNHK	2	5	1	N-Term(iTRAQ8plex); K12(iTRAQ8plex)	1.352	1.033	1.132	5.6	49	0	2040.13565	3	680.7167358	-6.38537464	24.385235
														LSLDGQNIYNACCTLR	12	5	1	N-Term(iTRAQ8plex); C12(Methylthio); C13(Methylthio)	0.977	0.898	0.819	5.23	102	0	2180.0352	2	1090.516602	-0.691182147	59.994655
														NFQNIFPPSATLHLSNIPPSVSEEDLK	2	5	1	N-Term(iTRAQ8plex); K27(iTRAQ8plex)	3.742	0.93	0.919	5.11	-	0	3602.9103	4	901.4830322	-5.904191885	49.06297
														KLPIDVTEGEVISLGLPFGK	3	5	1	N-Term(iTRAQ8plex); K1(iTRAQ8plex); K20(iTRAQ8plex)	-	-	-	4.18	20	1	3024.7981	5	605.7654419	-4.356765938	54.09661167
														ENALVQMADGNQAQLAMSHLNGHK	1	5	1	N-Term(iTRAQ8plex); K24(iTRAQ8plex)	-	-	1.237	3.88	-	0	3185.6581	4	797.1699829	5.521766638	38.37619167
														VTPQSLFILFGVYGDVQR	4	5	1	N-Term(iTRAQ8plex)	-	1.091	1.67	3.87	70	0	2343.30832	3	781.774292	2.919558855	66.49116167
														HQNVQLPR	7	5	1	N-Term(iTRAQ8plex)	1.318	1.013	0.944	3.03	40	0	1295.73987	3	432.5848083	-6.721643257	13.43708667
														LSLDGQNIYNACCTLR	2	5	1	N-Term(iTRAQ8plex); Y9(iTRAQ8plex); C12(Methylthio); C13(Methylthio)	1.743	1.837	1.055	-	28	0	2484.23623	2	1090.516602	-0.691182147	59.994655
														VTNLLMLK	2	22	2	N-Term(iTRAQ8plex); K8(iTRAQ8plex)	-	-	-	3.82	37	0	1539.96221	2	770.4847412	-8.484297911	63.07715833
														VTNILMLK	2	22	2	N-Term(iTRAQ8plex); M6(Oxidation); K8(iTRAQ8plex)	-	-	-	3.48	26	0	1555.95536	2	770.4847412	-8.484297911	63.07715833
14165469	RPS15A	40S ribosomal protein S15a	NP_001010.2	39.23	4	4	61	130	14.8	10.13	1.089	1.402	1.267																
														QFGFIVLTTSAGIMDHEEAR	22	1	1	N-Term(iTRAQ8plex)	1.39	1.366	1.403	7.31	100	0	2526.30429	3	842.7729492	3.08478526	54.20336167
														QFGFIVLTTSAGIMDHEEAR	7	1	1	N-Term(iTRAQ8plex); M14(Oxidation)	0.987	1.234	1.164	7.04	58	0	2542.28501	3	842.7729492	3.08478526	54.20336167
														HGYIGEFEIIDDHR	25	1	1	N-Term(iTRAQ8plex)	1.067	1.527	1.177	5.46	73	0	2005.00266	3	669.0038452	-5.560559725	29.44696
														WQNNLLPSR	3	1	1	N-Term(iTRAQ8plex)	1.001	1.381	1.226	3.05	30	0	1431.79363	2	716.4004517	-5.164570892	22.46275
														HGYIGEFEIIDDHR	2	1	1	N-Term(iTRAQ8plex); Y3(iTRAQ8plex)	0.338	1.148	0.922	-	35	0	2309.20302	3	669.0038452	-5.560559725	29.44696
														IVVNLTGR	2	1	1	N-Term(iTRAQ8plex)	1.499	2.311	2.081	-	24	0	1175.7332	2	588.3702393	-6.956854125	21.53948667
14210522	MAP1LC3A	microtubule-associated proteins 1A/1B light chain 3A isoform a	NP_115903.1	5.79	1	1	1	121	14.3	8.68	1.274	0.370	2.536																
														IPVIIER	1	7	1	N-Term(iTRAQ8plex)	1.274	0.37	2.536	-	35	0	1143.7321	2	572.3696899	-7.185281557	31.304545
14210536	TUBB6	tubulin beta-6 chain isoform 1	NP_115914.1	33.63	4	13	222	446	49.8	4.88	0.760	1.536	2.001																
														EIVHIQAGQCGNQIGTK	2	3	1	N-Term(iTRAQ8plex); C10(Methylthio); K17(iTRAQ8plex)	0.207	1.674	2.092	6.18	45	0	2450.29978	3	817.4381104	-4.360484953	27.75918
														FWEVISDEHGIDPAGGYVGDSALQLER	8	3	1	N-Term(iTRAQ8plex)	0.581	1.41	3.002	5.01	51	0	3264.61997	3	1088.878174	2.565975353	48.34442833
														INVYYNESSSQK	2	2	1	N-Term(iTRAQ8plex); K12(iTRAQ8plex)	0.861	2.011	1.915	4.74	55	0	2040.0712	3	680.6952515	-7.151787379	31.580755
														AALVDLEPGTMDSVR	2	2	1	N-Term(iTRAQ8plex)	0.76	1.212	1.334	4.47	50	0	1877.99187	2	939.4995728	-1.396391662	37.63957167
														EVDEQMLAIQSK	4	7	2	N-Term(iTRAQ8plex); K12(iTRAQ8plex)	-	-	-	4.11	61	0	1999.0811	2	1000.044189	-8.978632097	23.45914833
														YLTVATVFR	8	7	2	N-Term(iTRAQ8plex)	-	-	-	3.4	35	0	1373.80332	3	458.605957	-4.464581043	44.41719167
														LHFFMPGFAPLTSR	105	21	4	N-Term(iTRAQ8plex)	-	-	-	5.26	73	0	1925.03666	3	642.350647	-1.861590873	50.37672667
														LHFFMPGFAPLTSR	9	21	4	N-Term(iTRAQ8plex); M5(Oxidation)	-	-	-	4.68	37	0	1941.02982	3	642.350647	-1.861590873	50.37672667
														NSSYFVEWIPNNVK	31	20	5	N-Term(iTRAQ8plex); K14(iTRAQ8plex)	-	-	-	4.24	49	0	2305.22641	2	1153.123413	-1.809680962	42.78834
														FPGQLNADLR	9	25	5	N-Term(iTRAQ8plex)	-	-	-	4.12	55	0	1434.7963	3	478.9369507	-3.068379269	31.15012333

														LAVNMVPFPR	13	25	5	N-Term(iTRAQ8plex)	-	-	-	4.01	51	0	1447.83405	3	483.2828674	-3.931565971	41.77579833
														LAVNMVPFPR	4	25	5	N-Term(iTRAQ8plex); M5(Oxidation)	-	-	-	3.45	44	0	1463.82685	3	483.2828674	-3.931565971	41.77579833
														KLAVNMVPFPR	15	25	5	N-Term(iTRAQ8plex); K1(iTRAQ8plex)	-	-	-	3.44	37	1	1880.12295	4	470.7861938	-9.108866745	31.42844
														FPGQLNADLRK	1	25	5	N-Term(iTRAQ8plex); K11(iTRAQ8plex)	-	-	-	3.07	-	1	1867.09755	4	467.5298462	-1.861935048	33.46670167
														NMMAACDPR	3	23	5	N-Term(iTRAQ8plex); C6(Methylthio)	-	-	-	-	55	0	1358.59062	2	679.7989502	-6.255401168	21.43194333
														NMMAACDPR	5	23	5	N-Term(iTRAQ8plex); M3(Oxidation); C6(Methylthio)	-	-	-	-	47	0	1374.58525	2	679.7989502	-6.255401168	21.43194333
														NMMAACDPR	1	23	5	N-Term(iTRAQ8plex); M2(Oxidation); M3(Oxidation); C6(Methylthio)	-	-	-	-	34	0	1390.5822	2	679.7989502	-6.255401168	21.43194333
14211923	HINT2	histidine triad nucleotide-binding protein 2, mitochondrial precursor	NP_115982.1	21.47	2	2	7	163	17.2	9.16	0.952	0.576	0.606																
														ISQAEEEDQQLLGHLLLVAK	4	1	1	N-Term(iTRAQ8plex); K20(iTRAQ8plex)	1.012	0.576	0.606	5.93	47	0	2842.62172	4	711.4108887	2.889412766	59.52837833
														DVAPQAPVHFLVIPK	3	1	1	N-Term(iTRAQ8plex); K15(iTRAQ8plex)	0.779	0.686	0.856	4.24	31	0	2239.33463	3	747.116394	-3.444068159	36.67688833
14249348	TXNDC17	thioredoxin domain-containing protein 17	NP_116120.1	19.51	2	2	6	123	13.9	5.52	0.757	1.504	1.552																
														YEEVSVSGFEEFHR	4	1	1	N-Term(iTRAQ8plex)	1.09	1.426	1.682	4.86	25	0	2018.96994	3	673.661499	-3.079530583	31.35052167
														TIFAYFTGSK	2	1	1	N-Term(iTRAQ8plex); K10(iTRAQ8plex)	0.334	1.663	1.552	3.17	37	0	1742.98276	3	581.6657715	-6.299601533	68.00107833
14249376	USMG5	up-regulated during skeletal muscle growth protein 5	NP_116136.1	43.10	2	2	3	58	6.5	9.76	0.727	0.588	0.624																
														YFNSYTLTGR	2	1	1	N-Term(iTRAQ8plex)	0.763	0.57	0.589	-	36	0	1525.78899	2	763.3981323	-4.098385564	29.33912833
														AGPESDAQYQFTGIK	1	1	1	N-Term(iTRAQ8plex); K15(iTRAQ8plex)	0.727	1.135	0.928	-	36	0	2220.17044	3	740.7283325	-2.372336511	36.16651333
14249382	ABHD14B	protein ABHD14B isoform 1	NP_116139.1	6.67	1	1	3	210	22.3	6.40	0.949	0.903	1.063																
														FSSETWQNLGTLHR	3	1	1	N-Term(iTRAQ8plex)	0.949	0.903	1.063	3.89	22	0	1980.01975	3	660.6781006	-2.203408421	31.300215
14249398	PHF5A	PHD finger-like domain-containing protein 5A	NP_116147.1	34.55	3	3	13	110	12.4	8.41	1.256	1.644	1.474																
														CVICGGPGVSDAYYCK	6	1	1	N-Term(iTRAQ8plex); C1(Methylthio); C4(Methylthio); C15(Methylthio); K16(iTRAQ8plex)	1.189	1.185	0.913	6.18	54	0	2381.06852	3	794.3610229	-2.762920219	49.57691333
														ICDECNYGSYQGR	2	1	1	N-Term(iTRAQ8plex); C2(Methylthio); C5(Methylthio)	1.043	0.974	0.843	3.82	18	0	1903.78813	2	952.3977051	6.959932157	37.39203667
														HHPDLIFCR	5	1	1	N-Term(iTRAQ8plex); C8(Methylthio)	1.439	2.032	1.711	2.79	38	0	1487.76125	4	372.6923828	-5.129182853	24.46707167
14251209	CLIC1	chloride intracellular channel protein 1	NP_001279.2	41.49	7	7	27	241	26.9	5.17	0.914	1.694	2.393																
														LAALNPESNTAGLDIFAK	10	1	1	N-Term(iTRAQ8plex); K18(iTRAQ8plex)	1.071	1.694	2.647	6.69	65	0	2453.37302	3	818.4608154	-7.409512853	41.11555167
														FLDGNELTLADCNLLPK	4	1	1	N-Term(iTRAQ8plex); C12(Methylthio); K17(iTRAQ8plex)	0.723	2.112	3.207	6.3	51	0	2530.35294	3	844.1224976	0.919684264	56.42069167
														GVTFNVTTVDTK	3	1	1	N-Term(iTRAQ8plex); K12(iTRAQ8plex)	0.842	1.657	2.046	4.47	38	0	1890.07413	3	630.696228	-2.725008445	29.09682333
														LHIVQVVCK	5	1	1	N-Term(iTRAQ8plex); C8(Methylthio); K9(iTRAQ8plex)	0.653	1.466	1.849	4.16	38	0	1692.99845	3	565.0036621	-8.800503996	37.55525833
														LFMVLWLK	2	1	1	N-Term(iTRAQ8plex); K8(iTRAQ8plex)	1.28	5.686	6.844	3.65	31	0	1658.01633	3	553.3436279	-9.692763962	84.07093333
														GFTIPEAFR	2	1	1	N-Term(iTRAQ8plex)	0.897	2.492	3.296	2.9	23	0	1341.74028	2	671.3737793	-4.911029607	37.93926167
														VLDNYLTSPLPEEVDETSAEDEGVSQR	1	1	1	N-Term(iTRAQ8plex)	-	-	-	2.89	-	0	3296.59677	3	1099.537109	0.219491509	45.72743167
14277700	RPS12	40S ribosomal protein S12	NP_001007.2	46.21	6	6	42	132	14.5	7.21	1.153	2.283	2.149																
														QAHLCVLASNCDEPMYVK	9	1	1	N-Term(iTRAQ8plex); C5(Methylthio); C11(Methylthio); K18(iTRAQ8plex)	0.326	2.589	2.092	7.71	63	0	2721.30594	3	907.7734985	-3.388426265	39.35213333
														LVEALCAEHQINLIK	8	1	1	N-Term(iTRAQ8plex); C6(Methylthio); K15(iTRAQ8plex)	-	-	-	4.47	32	0	2348.32436	4	587.8364868	-2.109978916	55.25329
														LGEWVGLCK	6	1	1	N-Term(iTRAQ8plex); C8(Methylthio); K9(iTRAQ8plex)	1.931	6.406	5.375	3.91	36	0	1658.90642	3	553.6438599	-2.915130823	46.211535
														DVIEEYFK	6	1	1	N-Term(iTRAQ8plex); K8(iTRAQ8plex)	1.052	4.459	3.534	3.51	44	0	1650.90581	2	825.956543	-8.541966731	53.63263333
														TALIHDGLAR	12	1	1	N-Term(iTRAQ8plex)	1.263	1.692	1.763	3.13	43	0	1370.79828	3	457.6046448	-4.668971927	24.52470167
														KLGEWVGLCK	1	1	1	N-Term(iTRAQ8plex); K1(iTRAQ8plex); C9(Methylthio); K10(iTRAQ8plex)	1.626	1.999	2.374	2.7	-	1	2091.20205	4	523.5559692	-9.633324003	42.07896
14327896	CCNB1	G2/mitotic-specific cyclin-B1	NP_114172.1	3.70	1	1	1	433	48.3	7.47	2.581	2.701	2.757																
														ALNFGLGRPLPLHFLR	1	1	1	N-Term(iTRAQ8plex)	2.581	2.701	2.757	3.68	-	0	2125.26455	4	532.0715942	-2.599312581	45.93483333
14591902	RECQL	ATP-dependent DNA helicase Q1	NP_116559.1	2.00	1	1	4	649	73.4	7.88	1.200	2.467	1.733																
														LYEMVSYCQNISK	4	1	1	N-Term(iTRAQ8plex); C8(Methylthio); K13(iTRAQ8plex)	1.2	2.467	1.733	4.96	43	0	2232.1135	3	744.7093506	-8.42868976	80.623385
14591909	RPL5	60S ribosomal protein L5	NP_000960.2	37.04	10	10	86	297	34.3	9.72	1.391	2.104	1.664																
														IEGDMIVCAAYAHELPK	10	1	1	N-Term(iTRAQ8plex); C8(Methylthio); K17(iTRAQ8plex)	1.158	1.779	1.403	6.12	46	0	2514.29165	4	629.3288574	-3.162913233	53.23475167
														VGLTNYAAAYCTGLLLAR	11	1	1	N-Term(iTRAQ8plex); C11(Methylthio)	2.006	2.91	2.803	5.5	73	0	2220.17851	3	740.7329712	0.935969218	67.58723833
														NSVTPDMMEEMYK	6	1	1	N-Term(iTRAQ8plex); K13(iTRAQ8plex)	2.977	3.085	2.276	5.23	48	0	2183.05889	2	1092.033081	-2.49457798	45.05066667
														HIMGQNVADYMR	12	1	1	N-Term(iTRAQ8plex)	1.227	1.679	1.703	4.62	74	0	1738.85923	3	580.2913208	-4.41792339	23.22818
														EFNAEVHR	6	1	1	N-Term(iTRAQ8plex)	1.398	1.722	1.377	4.2	53	0	1305.68003	3	435.8981934	-4.054953562	14.15003667
														HIMGQNVADYMR	11	1	1	N-Term(iTRAQ8plex); M11(Oxidation)	1.014	1.177	1.095	4.19	38	0	1754.8544	3	580.2913208	-4.41792339	23.22818
														HIMGQNVADYMR	6	1	1	N-Term(iTRAQ8plex); M3(Oxidation); M11(Oxidation)	1.012	1.347	1.086	4.12	33	0	1770.84995	3	580.2913208	-4.41792339	23.22818
														DIICQIAYAR	16	1	1	N-Term(iTRAQ8plex); C4(Methylthio)	1.413	2.762	2.029	3.69	43	0	1515.78923	3	505.9347839	-4.442399181	43.08461
														NSVTPDMMEEMYKK	1	1	1	N-Term(iTRAQ8plex); K13(iTRAQ8plex); K14(iTRAQ8plex)	1.61	2.739	2.276	3.66	-	1	2615.34647	3	872.4536743	-6.955713513	27.83885333
														GAVDGGLSIPHSTK	2	1	1	N-Term(iTRAQ8plex); K14(iTRAQ8plex)	1.385	1.778	1.511	3.45	34	0	1947.10288	3	649.7058105	-4.680240308	19.71527833
														QFSQYIK	2	1	1	N-Term(iTRAQ8plex); K7(iTRAQ8plex)	1.74	3.12	2.549	3.23	31	0	1521.87737	2	761.4423218	-7.335916517	22.08237167
														RFPGYDSESK	1	1	1	N-Term(iTRAQ8plex); K10(iTRAQ8plex)	1.854	2.971	2.541	2.87	-	1	1793.95098	2	897.479126	-7.394832444	14.88612333
														VGLTNYAAAYCTGLLLAR	1	1	1	N-Term(iTRAQ8plex); Y10(iTRAQ8plex); C11(Methylthio)	-	-	-	-	31	0	2524.37563	3	740.7329712	0.935969218	67.58723833
														HIMGQNVADYMR	1	1	1	N-Term(iTRAQ8plex); Y10(iTRAQ8plex)	1.035	3.075	2.874	-	23	0	2043.07534	3	580.2913208	-4.41792339	23.22818
14702180	EIF4H	eukaryotic translation initiation factor 4H isoform 2	NP_114381.1	21.93	3	3	4	228	25.2	8.09	2.032	2.604	2.646																
														EALTYDGALLGDR	2	4	1	N-Term(iTRAQ8plex)	2.158	2.108	2.185	3.89	31	0	1697.89531	2	849.4512939	-3.474907632	30.92211167
														TVATPLNQVANPNSAIFGGARPR	1	4	1	N-Term(iTRAQ8plex)	1.914	2.604	3.355	3.32	-	0	2655.45536	3	885.8233032	-3.003206968	34.52084167
														GFCYVEFDEVDSLK	1	4	1	N-Term(iTRAQ8plex); C3(Methylthio); K14(iTRAQ8plex)	-	3.021	2.646	-	32	0	2305.13047	3	769.0483398	-1.58169735	47.46901833
14719402	RRS1	ribosome biogenesis regulatory protein homolog	NP_055984.1	17.26	4	4	12	365	41.2	10.70	1.404	0.809	0.589																
														FQPLFGDFAAEK	4	1	1	N-Term(iTRAQ8plex); K12(iTRAQ8plex)	1.737	0.774	0.684	4.16	49	0	1978.0894	2	989.5483398	-0.034494144	36.84955833
														CAGPTPEAELQALAR	4	1	1	N-Term(iTRAQ8plex); C1(Methylthio)	1.213	0.82	0.571	4	54	0	1876.94712	3	626.3205566	-4.904575117	43.91242667
														TNLVWDEVSGQWR	2	1	1	N-Term(iTRAQ8plex)	1.404	0.906	0.697	3.8	38	0	1893.96648	2	947.4868774	-5.0861709	37.63535667
														ELELQFDLGNLLASDRNPPTGLR	2	1	1	N-Term(iTRAQ8plex)	-	-	-	3.02	39	1	2872.54775	4	718.892395	0.215452685	56.01460167
14916501	RPS24	40S ribosomal protein S24 isoform a	NP_148982.1	30.00	4	4	49	130	15.1	10.89	1.298	2.325	2.223																
														TTGFGMIYDSLDYAK	6	5	1	N-Term(iTRAQ8plex); M6(Oxidation); K15(iTRAQ8plex)	1.537	1.922	2.223	5.97	52	0	2306.16703	2	1145.592163	-5.03530857	37.33833
														TTGFGMIYDSLDYAK	17	5	1	N-Term(iTRAQ8plex); K15(iTRAQ8plex)	1.327	2.366	2.156	5.42	66	0	2290.17705	2	1145.592163	-5.03530857	37.33833
														TTPDVIFVFGFR	14	5	1	N-Term(iTRAQ8plex)	1.245	2.581	2.606	4.48	45	0	1702.94097	2	851.9741211	-3.567788093	54.98037333
														QMVIDVLHPGK	11	5	1	N-Term(iTRAQ8plex); K11(iTRAQ8plex)	1.268	2.478	2.201	4.43	48	0	1845.06951	2	923.0383911	-9.856373097	31.65047833
														KQMVIDVLHPGK	1	5	1	N-Term(iTRAQ8plex); K1(iTRAQ8plex); K12(iTRAQ8plex)	1.328	1.659	1.419	4.11	-	1	2277.36655	3	759.7937012	-9.428009897	30.989345
15011916	GAR1	H/ACA ribonucleoprotein complex subunit 1	NP_127460.1	8.29	1	1	3	217	22.3	10.92	1.258	0.649	0.723																
														VVLLGEFLHPCEDDIVCK	3	1	1	N-Term(iTRAQ8plex); C11(Methylthio); C17(Methylthio); K18(iTRAQ8plex)	1.258	0.649	0.723	5.15	45	0	2729.40122	3	910.4719238	0.606336058	67.106815
15011936	RPS26	40S ribosomal protein S26	NP_001020.2	31.30	3	3	21	115	13.0	11.00	1.377	2.699	2.271																
														DISEASVFDAYVLPK	18	1	1	N-Term(iTRAQ8plex); K15(iTRAQ8plex)	1.251	2.43	1.938	6.18	100	0	2262.26201	2	1131.626099	-1.282201741	42.14175
														LHYCVSCAIHSK	1	2	1	N-Term(iTRAQ8plex); C4(Methylthio); C7(Methylthio); K12(iTRAQ8plex)	4.427	8.239	5.268	3.01	-	0	2061.02456	4	516.0115967	-5.665367359	41.58284333
														NIVEAAAVR	2	2	1	N-Term(iTRAQ8plex)	1.377	2.825	2.468	2.7	31	0	1246.74053	2	623.8739014	-1.279484858	25.06449333
15055539	RPS2	40S ribosomal protein S2	NP_002943.2	38.57	11	12	93	293	31.3	10.24	1.250	2.706	2.343																
														ESEIIDFFLGASLKDEVLK	4	1	1	N-Term(iTRAQ8plex); K14(iTRAQ8plex); K19(iTRAQ8plex)	0.294	0.996	0.918	5.62	31	1	3065.75864	4	767.1984253	5.887584802	65.773195
														KLLMMAGIDDCYTSAR	17	1	1	N-Term(iTRAQ8plex); K1(iTRAQ8plex); C11(Methylthio)	1.574	2.796	2.434	5.24	55	1	2442.24186	3	814.7521362	-2.275310523	47.34739667
														TYSYLTPDLWK	12	1	1	N-Term(iTRAQ8plex); K11(iTRAQ8plex)	1.188	2.398	2.298	4.99	48	0	1995.08782	2	998.0475464	-8.483409314	66.06787833
														SLEEIYLFSLPIK	25	1	1	N-Term(iTRAQ8plex); K13(iTRAQ8plex)	1.181	2.89	3.2	4.73	43	0	2160.29009	2	1080.648682	5.756338617	53.52426
														GCTATLGNFAK	5	1	1	N-Term(iTRAQ8plex); C2(Methylthio); K11(iTRAQ8plex)	0.818	2.404	1.985	4.67	66	0	1736.91117	2	868.961792	-6.963453319	41.50626333
														SPYQEFTDHLVK	15	1	1	N-Term(iTRAQ8plex); K12(iTRAQ8plex)	1.298	2.554	2.158	4.59	31	0	2072.11016	3	691.3774414	-4.589667336	30.32340667
														ESEIIDFFLGASLK	2	1	1	N-Term(iTRAQ8plex); K14(iTRAQ8plex)	3.323	6.589	5.356	4.15	37	0	2177.2258	3	726.4134521	-2.594174972	58.50765167
														LLMMAGIDDCYTSAR	2	1	1	N-Term(iTRAQ8plex); C10(Methylthio)	1.652	3.086	2.311	3.83	40	0	2009.96379	2	1005.485535	8.313789531	53.53437333
														AFVAIGDYNGHVGLGVK	1	1	1	N-Term(iTRAQ8plex); K17(iTRAQ8plex)	1.459	1.76	1.219	2.81	-	0	2325.30027	3	775.7716064	-7.446442214	35.93762167
														LSIVPVRR	6	1	1	N-Term(iTRAQ8plex)	1.361	2.988	2.46	2.77	35	1	1243.80484	3	415.2731323	-8.343412872	28.28362
														LSIVPVR	2	1	1	N-Term(iTRAQ8plex)	1.06	2.337	2.197	-	21	0	1087.70622	2	544.3567505	-7.241793107	22.05051167
														LIPAPR	2	1	2	N-Term(iTRAQ8plex)	-	-	-	-	19	0	970.63127	2	485.8192749	-3.964549071	20.6673
15082258	CBX3	chromobox protein homolog 3	NP_009207.2	22.95	2	4	14	183	20.8	5.33	1.286	1.087	1.204																
														VEEAEPEEFVVEK	2	1	1	N-Term(iTRAQ8plex); K13(iTRAQ8plex)	1.286	1.415	1.204	5.09	61	0	2142.14165	2	1071.574463	-0.491162081	31.63983333
														KVEEAEPEEFVVEK	3	1	1	N-Term(iTRAQ8plex); K1(iTRAQ8plex); K14(iTRAQ8plex)	1.572	1.082	1.323	4.46	44	1	2574.42514	3	858.8132324	-6.948069326	27.76587333
														IIGATDSSGELMFLMK	4	2	2	N-Term(iTRAQ8plex); K16(iTRAQ8plex)	-	-	-	5.54	74	0	2321.27349	2	1161.140381	1.250950725	49.48972167
														CPQIVIAFYEER	5	2	2	N-Term(iTRAQ8plex); C1(Methylthio)	-	-	-	4.28	54	0	1817.91838	2	909.4628296	-2.651241309	57.362115
15088795	PIGS	GPI transamidase component PIG-S	NP_149975.1	2.52	1	1	1	555	61.6	6.49	3.017	1.799	2.041																
														AAAGAAATHLEVAR	1	1	1	N-Term(iTRAQ8plex)	3.017	1.799	2.041	-	47	0	1612.90464	3	538.3063965	-1.658558612	19.41718167
15149476	RARS	arginine--tRNA ligase, cytoplasmic	NP_002878.2	16.36	9	9	30	660	75.3	6.68	1.028	1.973	2.037																
														VEIAGPGFINVHLR	8	1	1	N-Term(iTRAQ8plex)	0.724	2.18	2.265	6.56	68	0	1826.05423	3	609.3562622	-2.643913208	37.86784167
														VFHAGFGVVLGEDK	3	1	1	N-Term(iTRAQ8plex); K14(iTRAQ8plex)	1.008	1.199	1.122	5.26	42	0	2083.17295	3	695.0625	-3.237633251	35.66517667
														MLLCEAVAAVMAK	5	1	1	N-Term(iTRAQ8plex); C4(Methylthio); K13(iTRAQ8plex)	0.337	2.833	2.363	4.7	41	0	2004.10599	3	668.7068481	4.235745829	63.75609833
														LNDYIFSFDK	3	1	1	N-Term(iTRAQ8plex); K10(iTRAQ8plex)	1.124	1.876	1.851	4.62	45	0	1870.0064	2	935.5068359	-7.644917705	69.18804833
														GNTAAYLLYAFTR	3	1	1	N-Term(iTRAQ8plex)	1.073	2.083	2.101	4.46	47	0	1764.96916	2	882.9882202	5.961866022	51.54745333
														LFEFAGYDVLR	4	1	1	N-Term(iTRAQ8plex)	1.36	2.53	2.453	4.37	37	0	1633.90032	2	817.4537964	6.814891537	51.41131833
														VIVDFSSPNIAK	2	1	1	N-Term(iTRAQ8plex); K12(iTRAQ8plex)	1.077	1.981	2.153	4.31	30	0	1898.12711	3	633.3805542	3.34437437	34.216545
														LMDLLGEGLKR	1	1	1	N-Term(iTRAQ8plex); K10(iTRAQ8plex)	1.231	3.29	2.739	2.69	-	1	1853.10514	3	618.37323	-4.740480408	47.47848167
														STIIGESISR	1	1	1	N-Term(iTRAQ8plex)	0.82	1.145	1.2	-	29	0	1366.778	2	683.8926392	-4.660303048	25.00566
15150811	MRPS36	28S ribosomal protein S36, mitochondrial	NP_150597.1	30.10	2	2	3	103	11.5	9.99	0.867	0.513	0.372																
														SPDLLMYQGPPDTAEIIK	2	1	1	N-Term(iTRAQ8plex); K18(iTRAQ8plex)	0.633	0.467	0.434	4.44	34	0	2596.43387	3	866.1494751	7.144700725	39.29446
														VVQVVKPHTPLIR	1	1	1	N-Term(iTRAQ8plex); K6(iTRAQ8plex)	1.188	0.563	0.319	-	19	0	2094.33584	4	524.3394165	-0.642286854	24.011465
15431288	RPL10A	60S ribosomal protein L10a	NP_009035.3	50.23	11	11	51	217	24.8	9.94	1.527	2.214	1.847																
														VLCLAVAVGHVK	14	2	1	N-Term(iTRAQ8plex); C3(Methylthio); K12(iTRAQ8plex)	1.411	2.278	2.124	5.42	54	0	1863.10947	3	621.7080078	-3.978355872	52.629525
														KYDAFLASESLIK	5	2	1	N-Term(iTRAQ8plex); K1(iTRAQ8plex); K13(iTRAQ8plex)	1.86	2.977	2.637	4.84	42	1	2397.40281	2	1199.200073	-9.506758743	36.67230167
														KVLCLAVAVGHVK	2	2	1	N-Term(iTRAQ8plex); K1(iTRAQ8plex); C4(Methylthio); K13(iTRAQ8plex)	-	-	-	4.83	40	1	2295.39594	5	459.8850098	-9.266367289	39.833175
														FSVCVLGDQQHCDEAK	8	1	1	N-Term(iTRAQ8plex); C4(Methylthio); C12(Methylthio); K16(iTRAQ8plex)	1.46	2.044	1.873	4.7	45	0	2479.16288	3	827.0591431	-2.822465135	34.01131167

														AVDIPHMDIEALK	7	1	1	N-Term(iTRAQ8plex); K13(iTRAQ8plex)	1.651	2.552	1.828	4.65	56	0	2060.15801	2	1030.582642	-4.404864504	34.324435
														AVDIPHMDIEALKK	5	1	1	N-Term(iTRAQ8plex); K13(iTRAQ8plex); K14(iTRAQ8plex)	2.102	2.841	2.386	4.6	23	1	2492.45743	3	831.4906616	-4.003954728	31.91287667
														AGKFPSLLTHNENMVAK	1	2	1	N-Term(iTRAQ8plex); K3(iTRAQ8plex); K17(iTRAQ8plex)	1.804	2.214	2.314	4.53	-	1	2769.5958	3	923.8701172	3.93451366	31.14053833
														FLETVELQISLK	2	1	1	N-Term(iTRAQ8plex); K12(iTRAQ8plex)	1.157	1.441	1.051	3.26	53	0	2028.2061	2	1014.606689	-6.921887147	49.45003333
														DTLYEAVR	4	1	1	N-Term(iTRAQ8plex)	1.384	1.905	1.798	2.88	28	0	1270.68938	2	635.8483276	-4.015652171	22.96396167
														EVLHGNQR	2	1	1	N-Term(iTRAQ8plex)	2.178	4.262	2.471	2.81	25	0	1256.69739	3	419.5706482	-3.111305831	10.34077333
														ILGPGLNK	1	2	1	N-Term(iTRAQ8plex); K8(iTRAQ8plex)	2.174	2.067	1.847	-	32	0	1419.90268	3	473.9724121	-8.238749046	21.36505333
15431293	RPL15	60S ribosomal protein L15 isoform 1	NP_002939.2	27.45	6	6	54	204	24.1	11.62	1.611	2.017	1.649																
														FFEVILIDPFHK	29	1	1	N-Term(iTRAQ8plex); K12(iTRAQ8plex)	1.935	2.352	2.117	5.32	52	0	2113.22696	3	705.0805054	-1.745404911	63.138445
														VLNSYWVGEDSTYK	14	1	1	N-Term(iTRAQ8plex); K14(iTRAQ8plex)	1.531	1.731	1.645	5.06	70	0	2269.17949	2	1135.093384	-7.315570206	58.12109833
														NTLQLHR	4	1	1	N-Term(iTRAQ8plex)	1.25	1.276	1.191	3.5	50	0	1185.69449	3	395.9030151	-5.118637123	13.71372667
														SLQSVAEER	3	2	1	N-Term(iTRAQ8plex)	1.397	1.875	1.642	3.14	31	0	1322.72099	2	661.8616333	-4.370793489	18.91080667
														YIQELWR	3	2	1	N-Term(iTRAQ8plex)	1.503	1.685	1.541	2.77	26	0	1311.72966	2	656.3684692	-5.0463473	57.200935
														QLSALHR	1	2	1	N-Term(iTRAQ8plex)	0.8	0.999	1.26	-	33	0	1128.67472	3	376.8964233	-3.884671468	14.75744167
15431295	RPL13	60S ribosomal protein L13 isoform 1	NP_150254.1	25.59	6	6	40	211	24.2	11.65	1.822	1.786	1.528																
														STESLQANVQR	6	2	1	N-Term(iTRAQ8plex)	1.771	4.635	0.071	6.15	58	0	1536.83182	3	512.9487915	2.251360019	18.74622167
														LATQLTGPVMPVR	5	3	1	N-Term(iTRAQ8plex)	1.632	1.636	0.873	4.26	49	0	1686.98197	2	843.9950562	-2.984983135	36.07612667
														TIGISVDPR	9	2	1	N-Term(iTRAQ8plex)	1.918	1.849	1.947	3.51	60	0	1261.74662	3	421.2537231	3.831897412	26.183525
														LATQLTGPVMPVR	3	3	1	N-Term(iTRAQ8plex); M10(Oxidation)	1.777	1.355	1.127	3.2	32	0	1702.97637	2	843.9950562	-2.984983135	36.07612667
														GFSLEELR	8	2	1	N-Term(iTRAQ8plex)	1.508	1.571	1.502	3.15	29	0	1254.69499	2	627.8511353	-3.656197092	33.25481333
														VITEEEK	1	3	1	N-Term(iTRAQ8plex); K7(iTRAQ8plex)	1.804	2.953	2.022	-	34	0	1455.83695	3	485.9505005	-9.996370249	23.63256333
														LILFPR	8	2	1	N-Term(iTRAQ8plex)	1.879	1.776	1.686	-	17	0	1062.68913	2	531.8482056	-8.080243703	28.25203167
15431301	RPL7	60S ribosomal protein L7	NP_000962.2	46.77	12	12	76	248	29.2	10.65	1.632	2.042	1.786																
														IVEPYIAWGYPNLK	22	2	1	N-Term(iTRAQ8plex); K14(iTRAQ8plex)	1.872	2.403	2.419	5.8	66	0	2271.30327	2	1136.155273	1.536102662	45.52150167
														IALTDNALIAR	9	2	1	N-Term(iTRAQ8plex)	1.444	1.528	1.459	5.08	48	0	1474.88184	3	492.2987976	-5.208368242	34.68945667
														FKEANNFLWPFK	5	2	1	N-Term(iTRAQ8plex); K2(iTRAQ8plex); K12(iTRAQ8plex)	2.179	2.537	2.602	4.8	35	1	2453.39638	3	818.4726563	-3.034886131	46.48527
														EVPAVPETLK	4	1	1	N-Term(iTRAQ8plex); K10(iTRAQ8plex)	1.439	1.783	1.645	4.24	35	0	1691.00542	2	846.0063477	-8.61180416	21.03052333
														YGIICMEDLIHEIYTVGK	6	2	1	N-Term(iTRAQ8plex); C5(Methylthio); K18(iTRAQ8plex)	-	-	-	3.9	36	0	2751.44106	4	688.6157227	1.100675949	69.896685
														TTHFVEGGDAGNREDQINR	4	2	1	N-Term(iTRAQ8plex)	0.888	2.58	2.225	3.55	42	1	2420.18144	2	1210.59436	-1.754916717	13.86244833
														KAGNFYVPAEPK	2	2	1	N-Term(iTRAQ8plex); K1(iTRAQ8plex); K12(iTRAQ8plex)	1.938	2.361	1.99	3.37	41	1	2233.29033	2	1117.148804	-9.166071174	19.36103333
														SVNELIYK	4	2	1	N-Term(iTRAQ8plex); K8(iTRAQ8plex)	1.356	1.58	1.491	3.36	39	0	1573.93083	2	787.4690552	-6.440923718	35.05069
														TTHFVEGGDAGNR	4	2	1	N-Term(iTRAQ8plex)	1.036	0.961	0.999	3.3	32	0	1664.82346	3	555.6126709	-3.593086494	13.69465667
														EANNFLWPFK	6	2	1	N-Term(iTRAQ8plex); K10(iTRAQ8plex)	1.345	1.472	1.332	3.07	34	0	1874.03032	3	625.3491211	-4.963236876	69.47928667
														LAFVIR	9	2	1	N-Term(iTRAQ8plex)	1.719	2.14	1.81	-	31	0	1022.66112	2	511.834198	-5.184438722	45.30588
														NFAELK	1	1	1	N-Term(iTRAQ8plex); K6(iTRAQ8plex)	2.045	2.17	2.971	-	30	0	1329.80234	3	443.9389648	2.759794547	17.50453
15431303	RPL9	60S ribosomal protein L9	NP_000652.2	34.38	7	7	20	192	21.8	9.95	1.296	1.624	1.528																
														TILSNQTVDIPENVDITLK	4	1	1	N-Term(iTRAQ8plex); K19(iTRAQ8plex)	2.117	3.017	3.017	6.37	61	0	2721.54032	3	907.8516235	-3.374539217	46.05768
														DFNHINVELSLLGK	8	1	1	N-Term(iTRAQ8plex); K14(iTRAQ8plex)	1.269	1.442	1.447	5.3	38	0	2207.25602	3	736.4221191	-5.738868624	40.996505
														RDFNHINVELSLLGK	3	1	1	N-Term(iTRAQ8plex); K15(iTRAQ8plex)	0.977	2.2	1.821	4.64	47	1	2363.3613	3	788.4586182	-1.815235703	36.97301
														TICSHVQNMIKGVTLGFR	2	1	1	N-Term(iTRAQ8plex); C3(Methylthio); K11(iTRAQ8plex)	2.14	2.833	1.587	3.21	-	1	2658.45441	5	532.4967041	1.554172902	59.82432667
														TICSHVQNMIK	1	1	1	N-Term(iTRAQ8plex); C3(Methylthio); K11(iTRAQ8plex)	1.475	1.624	1.422	2.82	-	0	1928.02018	3	643.3449097	-9.054130405	32.78223333
														MRPGVACSVSQAQK	1	1	1	N-Term(iTRAQ8plex); C7(Methylthio); K14(iTRAQ8plex)	1.2	1.604	1.394	2.75	-	0	2116.12076	3	706.045105	-3.705180128	19.33632833
														GVTLGFR	1	1	1	N-Term(iTRAQ8plex)	1.558	1.706	1.686	-	22	0	1053.63005	2	527.3186646	-5.503657023	31.31872167
15431306	RPL8	60S ribosomal protein L8	NP_150644.1	25.29	4	4	24	257	28.0	11.03	1.446	1.358	1.309																
														AQLNIGNVLPVGTMPEGTIVCCLEEKPGDR	5	2	1	N-Term(iTRAQ8plex); C21(Methylthio); C22(Methylthio); K26(iTRAQ8plex)	-	-	-	5.33	56	0	3853.96286	3	1285.325806	0.628039832	60.64794667
														ASGNYATVISHNPETK	4	2	1	N-Term(iTRAQ8plex); K16(iTRAQ8plex)	1.167	1.946	1.33	5.25	34	0	2297.22104	3	766.4118652	-5.906635288	21.02132333
														AVVGVVAGGGR	7	2	1	N-Term(iTRAQ8plex)	1.413	1.593	1.39	4.29	45	0	1245.76233	3	415.9256287	3.380609782	24.53079667
														AQLNIGNVLPVGTMPEGTIVCCLEEKPGDR	2	2	1	N-Term(iTRAQ8plex); M14(Oxidation); C21(Methylthio); C22(Methylthio); [illegible]	-	-	-	3.72	34	0	3869.95786	3	1285.325806	0.628039832	60.64794667
														DIIHDPGR	6	2	1	N-Term(iTRAQ8plex)	1.469	1.249	1.277	-	32	0	1226.67313	3	409.562439	-5.500778748	14.42682167
15431310	KRT14	keratin, type I cytoskeletal 14	NP_000517.2	74.36	13	42	812	472	51.6	5.16	0.178	0.130	0.140																
														ILTATVDNANVLLQIDNAR	18	1	1	N-Term(iTRAQ8plex)	0.2	0.136	0.27	6.91	124	0	2358.32842	2	1179.667847	-0.459885323	59.21418167
														ADLEMQIESLKEELAYLK	34	1	1	N-Term(iTRAQ8plex); K11(iTRAQ8plex); K18(iTRAQ8plex)	0.067	-	0.218	5.85	37	1	3035.73305	4	763.6856689	5.144507654	62.214555
														APSTYGGGLSVSSSR	45	1	1	N-Term(iTRAQ8plex)	0.149	0.092	0.092	5.81	86	0	1729.90775	2	865.4541626	-0.701037027	16.04228167
														ADLEMQIESLKEELAYLK	11	1	1	N-Term(iTRAQ8plex); M5(Oxidation); K11(iTRAQ8plex); K18(iTRAQ8plex)	0.085	0.01	0.087	5.71	38	1	3051.72085	4	763.6856689	5.144507654	62.214555
														TMQNLEIELQSQLSMK	9	1	1	N-Term(iTRAQ8plex); M2(Oxidation); M15(Oxidation); K16(iTRAQ8plex)	0.125	0.25	0.093	5.62	59	0	2533.34365	2	1259.189941	8.449242852	39.25414
														TMQNLEIELQSQLSMK	32	1	1	N-Term(iTRAQ8plex); K16(iTRAQ8plex)	0.264	0.063	0.084	5.61	60	0	2501.35161	2	1259.189941	8.449242852	39.25414
														NKILTATVDNANVLLQIDNAR	2	1	1	N-Term(iTRAQ8plex); K2(iTRAQ8plex)	0.319	0.22	0.197	5.58	74	1	2904.67838	3	968.897644	1.933976218	50.172085
														LLEGEDAHLSSSQFSSGSQSSR	10	1	1	N-Term(iTRAQ8plex)	0.34	0.241	0.23	5.43	38	0	2613.26804	3	871.7608643	-0.547887374	33.17399167
														GQVGGDVNVEMDAAPGVDLSR	3	1	1	N-Term(iTRAQ8plex)	0.383	0.853	0.697	5.39	128	0	2390.18999	2	1195.598633	-1.030167063	32.92327833
														TMQNLEIELQSQLSMK	36	1	1	N-Term(iTRAQ8plex); M15(Oxidation); K16(iTRAQ8plex)	0.279	0.239	0.215	5.2	83	0	2517.34697	2	1259.189941	8.449242852	39.25414
														GQVGGDVNVEMDAAPGVDLSR	5	1	1	N-Term(iTRAQ8plex); M11(Oxidation)	0.265	0.565	0.52	4.89	106	0	2406.18926	2	1195.598633	-1.030167063	32.92327833
														DAEEWFFTK	43	1	1	N-Term(iTRAQ8plex); K9(iTRAQ8plex)	0.15	0.078	0.093	4	46	0	1780.93108	2	890.9691772	-3.123887208	59.30878667
														VVSTHEQVLR	40	1	1	N-Term(iTRAQ8plex)	0.19	0.164	0.177	3.93	61	0	1471.85346	3	491.2890015	-0.679521557	21.66381667
														ADLEMQIESLK	5	1	1	N-Term(iTRAQ8plex); K11(iTRAQ8plex)	0.184	0.204	0.242	3.64	67	0	1885.0521	2	943.029541	-2.289533231	32.28555167
														TKYETELNLR	13	1	1	N-Term(iTRAQ8plex); K2(iTRAQ8plex)	0.123	0.026	0.031	3.48	36	1	1875.07668	4	469.5246277	-1.557503137	25.58123
														YETELNLR	5	1	1	N-Term(iTRAQ8plex)	0.185	0.08	0.095	2.94	41	0	1341.72832	2	671.3677979	-2.438807342	19.78731167
														VMDVHDGK	5	1	1	N-Term(iTRAQ8plex); K8(iTRAQ8plex)	0.151	0.098	0.125	2.79	41	0	1508.83061	2	754.9189453	-3.007159954	16.6058
														APSTYGGGLSVSSSR	1	1	1	N-Term(iTRAQ8plex); Y5(iTRAQ8plex)	0.115	0.043	0.051	-	28	0	2034.10198	2	865.4541626	-0.701037027	16.04228167
														VMDVHDGK	1	1	1	N-Term(iTRAQ8plex); M2(Oxidation); K8(iTRAQ8plex)	0.089	0.017	0.016	-	25	0	1524.83183	2	754.9189453	-3.007159954	16.6058
														ALEEANADLEVK	48	8	3	N-Term(iTRAQ8plex); K12(iTRAQ8plex)	-	-	-	5.48	82	0	1910.06462	2	955.5366211	-1.657394329	22.98732333
														LAADDFR	10	31	7	N-Term(iTRAQ8plex)	-	-	-	-	39	0	1111.60307	2	556.3051758	-1.68797437	21.86801333
														TRLEQEIATYR	39	9	4	N-Term(iTRAQ8plex)	-	-	-	3.84	45	1	1683.92827	2	842.4677734	-2.90454474	28.813565
														LEQEIATYR	22	11	5	N-Term(iTRAQ8plex)	-	-	-	3.33	37	0	1426.78179	2	713.894104	-2.403951519	24.59227833
														VTMQNLNDR	4	10	3	N-Term(iTRAQ8plex); M3(Oxidation)	-	-	-	3.55	61	0	1410.73212	3	470.9155579	0.584117972	12.82526667
														VTMQNLNDR	6	10	3	N-Term(iTRAQ8plex)	-	-	-	3	42	0	1394.73418	3	470.9155579	0.584117972	12.82526667
														GSCGIGGGIGGGSSR	51	2	2	N-Term(iTRAQ8plex); C3(Methylthio)	-	-	-	5.1	76	0	1571.76799	2	786.3823853	0.17279244	21.17306167
														ASLENSLEETK	18	2	2	N-Term(iTRAQ8plex); K11(iTRAQ8plex)	-	-	-	4.6	64	0	1829.00701	2	915.0089111	-0.391279524	21.283405
														QFTSSSSMK	10	2	2	N-Term(iTRAQ8plex); K9(iTRAQ8plex)	-	-	-	4.01	38	0	1610.86479	2	805.9360352	-1.264915285	14.47664
														ISSVLAGGSCR	30	2	2	N-Term(iTRAQ8plex); C10(Methylthio)	-	-	-	3.28	46	0	1399.73198	2	700.3696289	-1.401059293	43.93359
														DYSPYFK	17	3	2	N-Term(iTRAQ8plex); K7(iTRAQ8plex)	-	-	-	3.23	31	0	1527.82756	2	764.418335	-0.620486901	29.96665833
														QFTSSSSMK	5	2	2	N-Term(iTRAQ8plex); M8(Oxidation); K9(iTRAQ8plex)	-	-	-	3.1	32	0	1626.86443	2	805.9360352	-1.264915285	14.47664
														TIEDLR	4	2	2	N-Term(iTRAQ8plex)	-	-	-	-	28	0	1050.60954	2	525.8084106	-0.13956749	25.768515
														MSVEADINGLR	23	2	2	N-Term(iTRAQ8plex)	-	-	-	5.86	56	0	1508.81614	3	503.6102295	7.723887815	31.32573667
														MSVEADINGLR	11	2	2	N-Term(iTRAQ8plex); M1(Oxidation)	-	-	-	3.84	46	0	1524.80033	3	503.6102295	7.723887815	31.32573667
														MSVEADINGLRR	8	2	2	N-Term(iTRAQ8plex)	-	-	-	3.7	37	1	1664.9041	2	832.9556885	-0.901533533	33.59522
														LASYLDKVR	18	11	6	N-Term(iTRAQ8plex); K7(iTRAQ8plex)	-	-	-	3.04	34	1	1673.01726	4	419.0100403	-1.374788993	40.70226667
														VLDELTLAR	35	7	5	N-Term(iTRAQ8plex)	-	-	-	3.26	41	0	1333.80168	2	667.40448	1.785903382	36.03089333
														LASYLDK	45	13	7	N-Term(iTRAQ8plex); K7(iTRAQ8plex)	-	-	-	3.56	36	0	1417.84687	3	473.2871399	-2.979780374	31.776525
														EVATNSELVQSGK	15	2	2	N-Term(iTRAQ8plex); K13(iTRAQ8plex)	-	-	-	4.99	56	0	1970.09818	2	985.5529175	-1.483661338	20.27982833
														CEMEQQNQEYK	6	2	2	N-Term(iTRAQ8plex); C1(Methylthio); K11(iTRAQ8plex)	-	-	-	4.53	51	0	2083.96611	2	1042.486694	-2.217045708	29.56338833
														NHEEEMNALR	15	2	2	N-Term(iTRAQ8plex)	-	-	-	3.49	46	0	1546.75431	2	773.8811035	-2.360353717	12.883705
														NHEEEMNALR	7	2	2	N-Term(iTRAQ8plex); M6(Oxidation)	-	-	-	3.36	26	0	1562.75334	2	773.8811035	-2.360353717	12.883705
														CEMEQQNQEYK	6	2	2	N-Term(iTRAQ8plex); C1(Methylthio); M3(Oxidation); K11(iTRAQ8plex)	-	-	-	3.34	44	0	2099.96445	2	1042.486694	-2.217045708	29.56338833
														SEISELR	9	2	2	N-Term(iTRAQ8plex)	-	-	-	3.01	34	0	1137.64849	2	569.3234253	-1.887534604	15.25243333
														ILNEMRDQYEK	1	2	2	N-Term(iTRAQ8plex); K11(iTRAQ8plex)	-	-	-	2.98	-	1	2047.10805	4	512.5324707	-1.083659212	36.977935
														KNHEEEMNALR	2	2	2	N-Term(iTRAQ8plex); K1(iTRAQ8plex)	-	-	-	2.96	26	1	1979.05901	2	990.0331421	0.048639119	11.96262333
														TEELNREVATNSELVQSGK	1	2	2	N-Term(iTRAQ8plex); K19(iTRAQ8plex)	-	-	-	2.88	-	1	2712.45229	4	678.8685303	-3.756779901	28.37277167
														SEISELRR	2	2	2	N-Term(iTRAQ8plex)	-	-	-	2.84	-	1	1293.74082	3	431.918457	-1.564035255	14.41779667
														ILLDVK	16	2	2	N-Term(iTRAQ8plex); K6(iTRAQ8plex)	-	-	-	2.76	25	0	1308.86919	2	654.9382324	-1.469265429	39.06651667
														ILNEMR	2	3	3	N-Term(iTRAQ8plex)	-	-	-	-	25	0	1079.61772	2	540.3125	-0.711288805	26.970265
														IRDWYQR	1	4	3	N-Term(iTRAQ8plex)	-	-	-	-	23	1	1340.73529	3	447.5832825	-1.780469196	20.12547667
														EELAYLKK	2	3	3	N-Term(iTRAQ8plex); K7(iTRAQ8plex); K8(iTRAQ8plex)	-	-	-	3.68	25	1	1906.17815	2	953.5927124	0.265916744	21.585135
														EELAYLK	5	3	3	N-Term(iTRAQ8plex); K7(iTRAQ8plex)	-	-	-	3.16	33	0	1473.87773	2	737.4425049	0.286214803	35.20986333
15451856	CAV1	caveolin-1 isoform alpha	NP_001744.2	56.74	8	8	39	178	20.5	6.02	0.960	0.376	0.327																
														IDFEDVIAEPEGTHSFDGIWK	15	2	1	N-Term(iTRAQ8plex); K21(iTRAQ8plex)	0.627	0.393	0.428	5.79	50	0	3013.53165	2	1507.282227	5.559942626	50.14407333
														HLNDDVVK	5	2	1	N-Term(iTRAQ8plex); K8(iTRAQ8plex)	0.96	0.599	0.412	4.33	34	0	1547.89853	3	516.6376953	-1.065064967	16.40666167
														ASFTTFTVTK	3	2	1	N-Term(iTRAQ8plex); K10(iTRAQ8plex)	0.79	0.313	0.286	4.22	51	0	1710.9793	2	855.9932861	-5.473856054	38.80888167
														VYSIYVHTVCDPLFEAVGK	3	2	1	N-Term(iTRAQ8plex); C10(Methylthio); K19(iTRAQ8plex)	0.542	-	-	3.78	30	0	2794.47085	4	699.3731689	-2.1493868	65.47201333
														EQGNIYKPNNK	1	1	1	N-Term(iTRAQ8plex); K7(iTRAQ8plex); K11(iTRAQ8plex)	1.174	0.184	0.107	3.69	-	0	2217.28188	4	555.0759277	2.895327633	16.68688167
														YVDSEGHLYTVPIR	4	1	1	N-Term(iTRAQ8plex)	1.431	0.642	0.59	3.47	32	0	1953.03733	3	651.68396	-0.524653151	26.51653167
														EIDLVNRDPK	7	3	1	N-Term(iTRAQ8plex); K10(iTRAQ8plex)	1.156	0.353	0.26	3.44	29	1	1807.05459	4	452.519104	0.650138426	16.76949833
														QVYDAHTK	1	2	1	N-Term(iTRAQ8plex); K8(iTRAQ8plex)	1.348	0.55	0.325	3.15	-	0	1569.8862	3	523.9669189	1.077967265	12.95260333
15487670	NXF1	nuclear RNA export factor 1 isoform 1	NP_006353.2	1.94	1	1	3	619	70.1	8.51	1.196	0.645	0.653																
														LNVVAFLNELPK	3	1	1	N-Term(iTRAQ8plex); K12(iTRAQ8plex)	1.196	0.645	0.653	4.51	49	0	1965.19022	3	655.7349243	-4.646675263	49.93350667
15718687	RPS3	40S ribosomal protein S3 isoform 1	NP_000996.2	64.20	14	14	118	243	26.7	9.66	1.129	2.279	2.147																

														FVDGLMIHSGDPVNYYVD TAVR	30	3	1	N-Term(iTRAQ8plex)	0.852	1.929	1.822	5.73	59	0	2772.38138	3	924.8045654	0.797770793	49.42948667
														ELAEDGYSGVEVR	10	2	1	N-Term(iTRAQ8plex)	0.936	1.611	1.67	5.25	54	0	1727.87468	2	864.4395142	-2.106406152	25.63559833
														KPLPDHVSIVEPK	1	3	1	N-Term(iTRAQ8plex); K1(iTRAQ8plex); K13(iTRAQ8plex)	-	1.652	1.404	4.99	-	0	2371.45083	4	593.6181641	1.343089203	23.19153833
														FGFPEGSVELYAEK	10	1	1	N-Term(iTRAQ8plex); K14(iTRAQ8plex)	1.125	2.537	2.034	4.97	53	0	2181.15312	2	1091.0802	-7.20606417	33.32134
														FVDGLMIHSGDPVNYYVD TAVR	11	3	1	N-Term(iTRAQ8plex); M6(Oxidation)	0.891	2.066	1.831	4.78	42	0	2788.38993	3	924.8045654	0.797770793	49.42948667
														GLCAIAQAESLR	5	2	1	N-Term(iTRAQ8plex); C3(Methylthio)	1.569	2.221	1.986	4.52	70	0	1581.83367	3	527.9487915	-4.84314336	44.53874
														DEILPTTPISEQK	5	3	1	N-Term(iTRAQ8plex); K13(iTRAQ8plex)	1.283	2.552	2.377	4.38	41	0	2079.18047	2	1040.093872	0.512808119	29.44926167
														TEIIILATR	8	2	1	N-Term(iTRAQ8plex)	1.161	2.789	2.32	3.8	34	0	1333.82891	2	667.4174194	-6.087033318	29.207835
														IMLPWDPTGK	9	3	1	N-Term(iTRAQ8plex); K10(iTRAQ8plex)	1.061	2.778	2.282	3.62	41	0	1765.99932	2	883.5032959	-7.823402312	33.736445
														FVADGIFK	5	2	1	N-Term(iTRAQ8plex); K8(iTRAQ8plex)	1.513	2.261	2.27	3.6	37	0	1504.88404	3	502.29953	-9.545228574	46.87132667
														AELNEFLTR	10	2	1	N-Term(iTRAQ8plex)	1.172	2.078	1.886	3.38	48	0	1396.76529	3	466.2599487	-6.098632774	34.43144
														TQNVLGEK	5	2	1	N-Term(iTRAQ8plex); K8(iTRAQ8plex)	2.333	3.198	2.733	3.13	49	0	1496.87639	2	748.9418335	-8.61188532	18.13836833
														GGKPEPPAMPQPVPTA	3	3	1	N-Term(iTRAQ8plex); K3(iTRAQ8plex)	1.448	1.641	1.439	2.78	30	0	2182.21562	2	1091.61145	0.234839591	27.038745
														KFVADGIFK	1	2	1	N-Term(iTRAQ8plex); K1(iTRAQ8plex); K9(iTRAQ8plex)	0.73	1.744	1.636	-	31	1	1937.18186	3	646.3988037	-8.710290734	37.07530167
														GGKPEPPAMPQPVPTA	1	3	1	N-Term(iTRAQ8plex); K3(iTRAQ8plex); M9(Oxidation)	-	1.374	1.781	-	28	0	2198.19431	2	1091.61145	0.234839591	27.038745
														FVDGLMIHSGDPVNYYVD TAVR	1	3	1	N-Term(iTRAQ8plex); Y15(iTRAQ8plex)	0.49	2.692	2.332	-	27	0	3076.59519	3	924.8045654	0.797770793	49.42948667
														LLGGLAVR	3	2	1	N-Term(iTRAQ8plex)	1.158	2.626	2.36	-	25	0	1102.71672	2	551.8619995	-7.516795922	34.5101
15721937	MRPS24	28S ribosomal protein S24, mitochondrial precursor	NP_114403.1	4.79	1	1	2	167	19.0	9.38	1.153	0.799	0.950																
														TVEDVFLR	2	1	1	N-Term(iTRAQ8plex)	1.153	0.799	0.95	2.95	31	0	1282.72795	2	641.8676147	-2.282968735	33.319055
15826852	ACBD3	Golgi resident protein GCP60	NP_073572.2	5.30	2	2	3	528	60.6	5.06	0.689	1.215	1.112																
														IQQDADSVITVGR	2	1	1	N-Term(iTRAQ8plex)	0.651	1.237	1.209	4.42	48	0	1705.94658	2	853.4769287	4.64229183	30.30514333
														NANKPLLDEIVPVYR	1	1	1	N-Term(iTRAQ8plex); K4(iTRAQ8plex)	0.73	1.193	1.024	2.82	-	0	2349.37076	3	783.795105	-1.842880087	37.882625
16117787	RPL34	60S ribosomal protein L34	NP_000986.2	13.68	2	2	3	117	13.3	11.47	1.342	2.038	1.916																
														IVYLYTK	1	1	1	N-Term(iTRAQ8plex); K7(iTRAQ8plex)	1.547	2.038	1.916	-	28	0	1507.91936	2	754.4633179	-9.982084069	33.44682333
														SACGVCPGR	2	1	1	N-Term(iTRAQ8plex); C3(Methylthio); C6(Methylthio)	1.174	2.035	1.734	-	16	0	1245.54387	2	623.2755737	-6.063583339	24.71380833
16117791	RPL35A	60S ribosomal protein L35a	NP_000987.2	25.45	3	3	7	110	12.5	11.06	1.679	2.189	1.777																
														DETEFYLGK	4	1	1	N-Term(iTRAQ8plex); K9(iTRAQ8plex)	1.895	1.909	1.757	3.69	44	0	1709.90764	2	855.4574585	-7.603292427	35.01432667
														NNTVTPGGKPNK	1	1	1	N-Term(iTRAQ8plex); K9(iTRAQ8plex); K12(iTRAQ8plex)	1.679	2.435	2.202	3.21	-	0	2139.24599	4	535.5669556	-8.843730194	14.05584667
														EHTALLK	2	1	1	N-Term(iTRAQ8plex); K7(iTRAQ8plex)	0.699	1.342	1.194	2.91	37	0	1419.86565	2	710.4364624	-8.686114301	17.91669333
16117794	RPL36	60S ribosomal protein L36	NP_056229.2	29.52	4	4	19	105	12.2	11.59	1.524	1.653	1.732																
														EVCGFAPYER	11	1	1	N-Term(iTRAQ8plex); C3(Methylthio)	1.358	1.589	1.637	3.86	34	0	1520.71196	2	760.8596191	-3.939364522	34.3937
														REELSNVLAAMR	2	1	1	N-Term(iTRAQ8plex)	0.856	0.815	0.789	3.46	27	1	1692.92978	3	564.9814453	-4.204907122	37.11491667
														EELSNVLAAMR	4	1	1	N-Term(iTRAQ8plex)	2.489	2.041	1.85	3.16	43	0	1536.83086	2	768.9190674	-3.203445811	45.43369167
														YPMAVGLNK	2	1	1	N-Term(iTRAQ8plex); K9(iTRAQ8plex)	1.524	3.28	2.824	2.79	36	0	1600.91985	2	800.963562	-8.922305877	25.10968167
16306548	SARS	serine--tRNA ligase, cytoplasmic	NP_006504.2	14.98	6	6	15	514	58.7	6.43	1.024	2.161	2.361																
														EVMQEVAQLSQFDEELYK	2	2	1	N-Term(iTRAQ8plex); K18(iTRAQ8plex)	-	-	-	5.82	56	0	2794.44399	4	699.3664551	0.357542504	54.99983833
														YLIATSEQPIAALHR	2	2	1	N-Term(iTRAQ8plex)	1.024	1.017	0.952	5.57	44	0	1987.12149	3	663.0453491	-3.199193777	35.18841333
														KLDLEAWFPGSGAFR	6	2	1	N-Term(iTRAQ8plex); K1(iTRAQ8plex)	1.132	1.705	1.927	4.1	30	1	2302.26967	4	576.322876	-4.694182385	49.59153167
														DEWLRPEDLPIK	1	2	1	N-Term(iTRAQ8plex); K12(iTRAQ8plex)	1.175	3.106	2.361	2.89	-	0	2119.19199	3	707.0688477	-4.167428573	34.92039667
														LLIDEAILK	2	2	1	N-Term(iTRAQ8plex); K9(iTRAQ8plex)	0.701	1.731	1.428	2.75	38	0	1636.03818	3	546.0175781	-7.561055202	38.65821667
														VLDLDLFR	2	2	1	N-Term(iTRAQ8plex)	0.823	5.929	4.231	-	31	0	1294.75871	2	647.887146	-0.197663216	47.00495167
16332360	CDK11B	cyclin-dependent kinase 11B isoform 3	NP_277022.1	1.90	1	1	2	526	59.2	4.59	0.955	0.712	0.802																
														AQHPNIVTVR	2	19	1	N-Term(iTRAQ8plex)	0.955	0.712	0.802	3.18	34	0	1438.83643	3	480.2836609	-4.72040035	13.54268667
16445412	TRIM13	E3 ubiquitin-protein ligase TRIM13 isoform 1	NP_434698.1	3.44	1	1	2	407	47.0	6.01	1.022	0.669	0.646																
														DAFESLFQSFETWR	2	2	1	N-Term(iTRAQ8plex)	1.022	0.669	0.646	4.67	45	0	2067.02202	2	1034.014648	4.576126866	64.348055
16445419	SCAMP3	secretory carrier-associated membrane protein 3 isoform 1	NP_005689.2	16.43	3	3	9	347	38.3	7.64	1.003	0.859	0.856																
														AQQEFAAGVFSNPAVR	2	4	1	N-Term(iTRAQ8plex)	1.352	0.808	1.161	5.83	61	0	1996.05828	2	998.5327759	1.419716973	33.021415
														DGGNPFAEPSELDNPFQD PAVIQHRPSR	2	3	1	N-Term(iTRAQ8plex)	-	-	-	4.67	40	0	3394.66372	4	849.4213867	-2.413878276	36.41405667
														ELQHAALGGTATR	5	4	1	N-Term(iTRAQ8plex)	0.851	0.909	0.841	3.15	36	0	1628.89835	3	543.6376343	-2.370754424	16.61096167
16507237	HSPA5	78 kDa glucose-regulated protein precursor	NP_005338.1	51.38	29	31	389	654	72.3	5.16	1.058	0.798	0.753																
														LYGSAGPPPTGEEDTAEK DEL	8	1	1	N-Term(iTRAQ8plex); K18(iTRAQ8plex)	1.012	0.609	0.605	7.88	61	1	2784.39603	3	928.8035278	-2.733466728	30.46854
														DNHLLGTFDLTGIPPAPR	43	1	1	N-Term(iTRAQ8plex)	0.963	1.082	1.002	6.28	77	0	2238.21714	2	1119.615112	1.991173122	39.27652333
														IINEPTAAAIAYGLDKR	11	1	1	N-Term(iTRAQ8plex); K16(iTRAQ8plex)	1.025	0.77	0.874	6.12	82	1	2424.40312	2	1212.7052	-1.652996292	37.40571167
														TFAPEEISAMVLTK	14	1	1	N-Term(iTRAQ8plex); M10(Oxidation); K14(iTRAQ8plex)	1.095	0.861	0.748	6.06	48	0	2161.2166	2	1073.104858	-2.876708981	42.23885333
														IEWLESHQDADIEDFK	36	1	1	N-Term(iTRAQ8plex); K16(iTRAQ8plex)	1.045	0.752	0.73	5.94	90	0	2583.31767	2	1292.162476	-0.424550234	44.19695333
														VTHAVVTVPAYFNDAQR	33	1	1	N-Term(iTRAQ8plex)	0.974	0.963	0.678	5.91	76	0	2192.16924	2	1096.588257	-3.359342883	31.15895333
														ITPSYVAFTPEGER	16	1	1	N-Term(iTRAQ8plex)	1.207	0.89	0.817	5.45	68	0	1870.98955	2	935.9984131	2.286625212	32.01406333
														NELESYAYSLK	16	1	1	N-Term(iTRAQ8plex); K11(iTRAQ8plex)	1.057	0.79	0.761	5.37	48	0	1925.03923	2	963.0230713	-4.548137718	55.79188833
														ELEEIVQPIISK	21	1	1	N-Term(iTRAQ8plex); K12(iTRAQ8plex)	1.092	0.73	0.758	5.24	50	0	2006.19194	2	1003.603821	0.475367057	42.27527167
														IEIESFYEGEDFSETLTR	11	1	1	N-Term(iTRAQ8plex)	0.958	0.972	0.807	5.24	75	0	2469.19145	2	1235.099365	-2.461312281	51.10209
														SDIDEIVLVGGSTR	5	1	1	N-Term(iTRAQ8plex)	1.182	0.64	0.709	5.24	59	0	1764.95867	2	882.9829712	-3.33466007	35.61281833
														AKFEELNMDLFR	8	1	1	N-Term(iTRAQ8plex); K2(iTRAQ8plex); M8(Oxidation)	1.222	0.769	0.866	5.04	39	1	2137.15883	2	1061.082886	-1.808651091	41.12716667
														TKPYIQVDIGGGQTK	4	1	1	N-Term(iTRAQ8plex); K2(iTRAQ8plex); K15(iTRAQ8plex)	1.091	0.845	0.705	4.93	37	0	2517.47344	2	1259.240356	-2.762702189	23.4669
														TFAPEEISAMVLTK	8	1	1	N-Term(iTRAQ8plex); K14(iTRAQ8plex)	0.969	0.852	0.837	4.75	66	0	2145.20244	2	1073.104858	-2.876708981	42.23885333
														DAGTIAGLNVMR	11	1	1	N-Term(iTRAQ8plex)	1.197	0.875	0.743	4.65	66	0	1521.83521	3	507.9499207	-0.599662239	30.53191
														SQIFSTASDNQPTVTIK	10	1	1	N-Term(iTRAQ8plex); K17(iTRAQ8plex)	1.1	0.977	1.07	4.56	46	0	2445.33487	3	815.7831421	-3.966033681	44.95317667
														NQLTSNPENTVFDAK	4	1	1	N-Term(iTRAQ8plex); K15(iTRAQ8plex)	1.091	0.736	0.689	4.51	58	0	2286.21196	2	1143.609619	-2.921705801	36.79511667
														KVTHAVVTVPAYFNDAQ R	5	1	1	N-Term(iTRAQ8plex); K1(iTRAQ8plex)	1.081	1.049	1.033	4.46	39	1	2624.46146	3	875.4920044	-5.895063289	28.82996167
														AKFEELNMDLFR	19	1	1	N-Term(iTRAQ8plex); K2(iTRAQ8plex)	1.108	0.767	0.743	4.38	47	1	2121.15127	2	1061.082886	-1.808651091	41.12716667
														FEELNMDLFR	8	1	1	N-Term(iTRAQ8plex)	0.854	0.56	0.564	4.12	53	0	1617.82366	2	809.4154663	-0.757377238	53.014495
														KSDIDEIVLVGGSTR	20	1	1	N-Term(iTRAQ8plex); K1(iTRAQ8plex)	1.05	0.771	0.751	4.11	67	1	2197.25615	2	1099.131714	-3.973221611	32.77678167
														TWNDPSVQQDIK	4	1	1	N-Term(iTRAQ8plex); K12(iTRAQ8plex)	0.811	0.819	0.682	3.95	38	0	2039.08867	2	1020.047974	-6.444376139	33.06724333
														MVNDAEK	3	1	1	N-Term(iTRAQ8plex); M1(Oxidation); K7(iTRAQ8plex)	0.949	0.774	0.695	3.57	45	0	1430.78199	3	477.5988464	3.508400783	10.61969
														DAGTIAGLNVMR	5	1	1	N-Term(iTRAQ8plex); M11(Oxidation)	0.995	0.725	0.708	3.53	51	0	1537.82334	3	507.9499207	-0.599662239	30.53191
														VMEHFIK	7	1	1	N-Term(iTRAQ8plex); K7(iTRAQ8plex)	1.186	0.915	0.726	3.32	30	0	1511.87834	2	756.4428101	-5.362734936	23.42209667
														ITITNDQNR	10	1	1	N-Term(iTRAQ8plex)	1.06	0.756	0.687	3.29	38	0	1378.7609	3	460.2584839	1.2236423	20.62344667
														NTVVPTKK	1	1	1	N-Term(iTRAQ8plex); K7(iTRAQ8plex); K8(iTRAQ8plex)	1.456	0.813	0.778	2.99	-	1	1799.153	2	900.0801392	0.689435702	15.33238833
														VYEGERPLTK	5	1	1	N-Term(iTRAQ8plex); K10(iTRAQ8plex)	1.003	0.717	0.653	2.99	32	0	1800.04558	2	900.5264282	-1.111902226	23.248745
														MKETAEAYLGK	1	1	1	N-Term(iTRAQ8plex); M1(Oxidation); K2(iTRAQ8plex); K11(iTRAQ8plex)	0.916	0.707	0.509	2.91	-	1	2169.23728	3	718.4212646	4.133007524	31.78430667
														VMEHFIK	1	1	1	N-Term(iTRAQ8plex); M2(Oxidation); K7(iTRAQ8plex)	1.159	0.747	0.756	2.89	-	0	1527.88355	2	756.4428101	-5.362734936	23.42209667
														ETAEAYLGK	1	1	1	N-Term(iTRAQ8plex); K9(iTRAQ8plex)	0.684	0.525	0.463	2.87	-	0	1589.9011	3	530.6385498	0.996831857	31.18497667
														LTPEEIER	1	1	1	N-Term(iTRAQ8plex)	1.017	0.809	0.787	-	29	0	1290.71672	2	645.8619995	-3.090450827	21.64097167
														VLEDSDLKK	1	1	1	N-Term(iTRAQ8plex); K8(iTRAQ8plex); K9(iTRAQ8plex)	-	4.73	2.458	-	27	1	1959.19216	4	490.5534973	1.637449291	18.37259833
														MKETAEAYLGK	1	1	1	N-Term(iTRAQ8plex); K2(iTRAQ8plex); K11(iTRAQ8plex)	1.267	1.121	0.981	-	27	1	2153.24924	3	718.4212646	4.133007524	31.78430667
														FEELNMDLFR	1	1	1	N-Term(iTRAQ8plex); M6(Oxidation)	0.81	0.891	0.666	-	20	0	1633.82256	2	809.4154663	-0.757377238	53.014495
														IINEPTAAAIAYGLDK	12	5	2	N-Term(iTRAQ8plex); K16(iTRAQ8plex)	-	-	-	5.68	53	0	2268.30278	2	1134.655029	-1.423675126	44.57258167
														VEIIANDQGNR	24	6	3	N-Term(iTRAQ8plex)	-	-	-	4.22	66	0	1532.82414	3	511.616333	0.64225686	26.62443333
16554604	MRPS23	28S ribosomal protein S23, mitochondrial	NP_057154.2	24.21	4	4	6	190	21.8	8.90	1.467	0.652	0.559																
														EVPQDQHLEAPADQSK	2	1	1	N-Term(iTRAQ8plex); K16(iTRAQ8plex)	1.44	0.649	0.512	4.39	36	0	2400.26108	3	800.7585449	-0.208220838	17.637675
														AFDLFNPNFK	1	1	1	N-Term(iTRAQ8plex); K10(iTRAQ8plex)	1.658	0.655	0.467	3.15	-	0	1821.01975	3	607.6781006	2.29937529	68.569125
														LETVGSIFSR	2	1	1	N-Term(iTRAQ8plex)	1.495	0.847	0.686	2.89	24	0	1412.8012	2	706.9042358	-2.771968431	46.34025
														ALLAEGVILR	1	1	1	N-Term(iTRAQ8plex)	1.256	0.581	0.611	-	19	0	1358.86394	2	679.9356079	-2.496276183	43.36506833
16579885	RPL4	60S ribosomal protein L4	NP_000959.2	31.62	14	14	96	427	47.7	11.06	1.201	1.520	1.494																
														RGPCIIYNEDNGIIK	2	1	1	N-Term(iTRAQ8plex); C4(Methylthio); K15(iTRAQ8plex)	0.965	0.872	1.232	5.4	34	1	2359.26212	3	787.0922241	-4.307781927	29.27175333
														IEEVPELPLVVEDK	6	1	1	N-Term(iTRAQ8plex); K14(iTRAQ8plex)	1.378	1.269	1.246	5.25	49	0	2217.28415	3	739.7662354	0.117939186	43.28513
														NIPGITLLNVSK	13	1	1	N-Term(iTRAQ8plex); K12(iTRAQ8plex)	1.328	2.148	1.709	5.18	58	0	1877.16399	2	939.0856323	-2.159252782	35.96082333
														RYAICSALAASALPALVMS K	7	1	1	N-Term(iTRAQ8plex); C5(Methylthio); K20(iTRAQ8plex)	0.914	1.486	1.183	4.92	26	1	2690.49843	3	897.5059814	0.642551109	63.36889333
														IEEVPELPLVVEDKVEGYK	4	1	1	N-Term(iTRAQ8plex); K14(iTRAQ8plex); K19(iTRAQ8plex)	-	-	-	4.81	34	1	3097.76357	4	775.1963501	-5.31376556	54.92191667
														GPCIIYNEDNGIIK	8	1	1	N-Term(iTRAQ8plex); C3(Methylthio); K14(iTRAQ8plex)	2.115	2.19	2.026	4.78	56	0	2203.15006	3	735.0548706	-9.578890694	37.56313
														YAICSALAASALPALVMS K	4	1	1	N-Term(iTRAQ8plex); C4(Methylthio); M17(Oxidation); K19(iTRAQ8plex)	2.482	2.232	3.074	4.58	41	0	2550.40024	2	1267.70459	0.536918592	68.716165
														YAICSALAASALPALVMS K	11	1	1	N-Term(iTRAQ8plex); C4(Methylthio); K19(iTRAQ8plex)	1.532	2.239	2.201	4.26	74	0	2534.4019	2	1267.70459	0.536918592	68.716165
														SNYNLPMHK	4	1	1	N-Term(iTRAQ8plex); K9(iTRAQ8plex)	1.245	1.466	1.449	3.79	44	0	1711.92497	2	856.4661255	-9.356101276	16.65133
														FCIWTESAFR	18	1	1	N-Term(iTRAQ8plex); C2(Methylthio)	1.096	1.425	1.435	3.73	50	0	1609.77666	2	805.3919678	-2.629789109	48.86845333
														LAPGGHVGR	12	1	1	N-Term(iTRAQ8plex)	0.979	1.1	1.021	3.5	52	0	1167.68177	3	389.8987732	-7.069537403	12.54475167
														APIRPDIVNFVHTNLR	4	1	1	N-Term(iTRAQ8plex)	1.089	1.257	1.302	3.43	28	0	2166.23452	4	542.3140869	-4.825665764	35.09856
														KPTTEEK	1	1	1	N-Term(iTRAQ8plex); K1(iTRAQ8plex); K7(iTRAQ8plex)	1.366	1.222	1.137	2.89	-	0	1745.06057	4	437.0205994	1.934764329	12.47051
														KLDELYGTWR	1	1	1	N-Term(iTRAQ8plex); K1(iTRAQ8plex)	1.453	1.629	1.57	2.81	-	1	1889.05657	2	945.0319214	-9.29836982	33.04076
														MINTDLSR	1	1	1	N-Term(iTRAQ8plex)	1.033	1.483	1.388	-	28	0	1253.67729	2	627.3422852	-4.193748684	21.754565
16596694	MRPL53	39S ribosomal protein L53, mitochondrial	NP_444278.1	7.14	1	1	2	112	12.1	8.76	1.009	0.840	0.553																
														VQFCPFEK	2	1	1	N-Term(iTRAQ8plex); C4(Methylthio); K8(iTRAQ8plex)	1.009	0.84	0.553	3.57	26	0	1651.87773	2	826.4425049	-1.16180171	62.33392333
16751921	DCD	dermcidin isoform 1 preproprotein	NP_444513.1	12.73	1	1	2	110	11.3	6.54	0.403	0.484	0.599																
														GAVHDVKDVLDSVL	2	1	1	N-Term(iTRAQ8plex); K7(iTRAQ8plex)	0.403	0.484	0.599	3.14	-	1	2075.1912	3	692.4019165	-2.185359252	51.55453167
16753205	UBQLN1	ubiquilin-1 isoform 2	NP_444295.1	5.17	2	2	3	561	59.2	5.10	1.329	1.884	1.786																
														FQQQLEQLSAMGFLNR	2	2	1	N-Term(iTRAQ8plex)	2.341	3.343	2.879	6.44	69	0	2214.18193	2	1107.594604	7.944724017	55.09212667
														SHTDQLVLIFAGK	1	3	1	N-Term(iTRAQ8plex); K13(iTRAQ8plex)	0.754	1.062	1.108	4.19	-	0	2037.17777	3	679.7307739	-8.616220404	35.19993

16757970	FAM129A	protein Niban	NP_443198.1	2.59	2	2	8	928	103.1	4.78	0.995	2.228	3.289																
														EFPVYLWQPFFR	4	3	1	N-Term(iTRAQ8plex)	0.995	2.726	4.9	3.85	41	0	1933.0391	3	645.0178833	3.965440925	61.65130333
														FRFPHIDLVVQR	4	3	1	N-Term(iTRAQ8plex)	-	1.821	2.208	3.21	26	1	1831.05251	4	458.5185852	-6.531958869	38.67329333
16933546	RPLP0	60S acidic ribosomal protein P0	NP_444505.1	38.80	9	9	74	317	34.3	5.97	1.216	2.130	1.916																
														VLALSVETDYTFPLAEK	9	1	1	N-Term(iTRAQ8plex); K17(iTRAQ8plex)	0.96	1.476	1.36	5.29	74	0	2504.41509	2	1252.711182	1.68317196	50.69813
														GTIEILSDVQLIK	8	1	1	N-Term(iTRAQ8plex); K13(iTRAQ8plex)	1.498	2.453	2.013	5.21	85	0	2037.22917	2	1019.118225	-6.105216266	39.24654167
														AGAIAPCEVTVPAQNTGL GPEK	2	1	1	N-Term(iTRAQ8plex); C7(Methylthio); K22(iTRAQ8plex)	-	2.655	3.797	4.97	41	0	2777.46567	3	926.4934082	-4.688065368	38.81533
														CFIVGADNVGSK	10	1	1	N-Term(iTRAQ8plex); C1(Methylthio); K12(iTRAQ8plex)	1.467	2.627	2.183	4.92	64	0	1863.98015	2	932.4906006	-9.55901147	46.86501167
														TSFFQALGITTK	2	1	1	N-Term(iTRAQ8plex); K12(iTRAQ8plex)	1.788	2.054	1.916	4.9	31	0	1922.10917	3	641.3745728	-6.027903353	70.29314667
														IIQLLDDYPK	33	1	1	N-Term(iTRAQ8plex); K10(iTRAQ8plex)	1.216	2.035	1.834	4.71	59	0	1826.08939	3	609.3618774	-9.475110788	51.51957
														GHLENNPALEK	2	1	1	N-Term(iTRAQ8plex); K11(iTRAQ8plex)	0.278	0.407	0.2	3.58	26	0	1830.02616	3	610.6802368	-3.739892326	18.758455
														VEAKEESEESDEDMGFGL FD	2	1	1	N-Term(iTRAQ8plex); K4(iTRAQ8plex)	0.829	1.086	0.621	3.1	18	1	2871.32938	3	957.781311	-1.585134942	35.28289333
														LLPHIR	5	1	1	N-Term(iTRAQ8plex)	1.5	2.528	2.105	-	32	0	1052.67835	3	351.5643005	-9.369375448	27.14624833
														IIQLLDDYPK	1	1	1	N-Term(iTRAQ8plex); Y8(iTRAQ8plex); K10(iTRAQ8plex)	-	-	-	-	26	0	2130.27236	3	609.3618774	-9.475110788	51.51957
16950603	MRPS35	28S ribosomal protein S35, mitochondrial isoform 1 precursor	NP_068593.2	14.86	3	3	8	323	36.8	8.24	1.271	0.646	0.593																
														NILETLLQMK	4	1	1	N-Term(iTRAQ8plex); K10(iTRAQ8plex)	1.271	0.646	0.577	5.07	66	0	1811.08635	2	906.046814	-3.174884681	78.96472833
														HFPIEIDSTDYVSSGPSVR	2	3	1	N-Term(iTRAQ8plex)	1.564	1.184	1.252	4.95	39	0	2410.21689	3	804.0771484	-0.982997633	34.23792833
														SKTEADMEEYIWENSSSER	2	1	1	N-Term(iTRAQ8plex); K2(iTRAQ8plex)	1.089	0.538	0.551	4.37	35	1	2899.37546	3	967.1300049	-4.207768785	33.504645
17017988	COX5B	cytochrome c oxidase subunit 5B, mitochondrial precursor	NP_001853.2	21.71	4	4	10	129	13.7	8.81	1.135	0.554	0.598																
														EDPNLVPSISNKR	2	1	1	N-Term(iTRAQ8plex); K12(iTRAQ8plex)	1.28	0.568	0.633	3.67	-	1	2077.1854	3	693.0666504	-0.396391254	19.61466333
														EDPNLVPSISNK	2	1	1	N-Term(iTRAQ8plex); K12(iTRAQ8plex)	0.989	0.467	0.454	3.54	35	0	1921.07073	2	961.0390015	-7.482801188	20.72034
														LVPQQLAH	2	1	1	N-Term(iTRAQ8plex)	1.092	0.691	0.745	3.08	37	0	1209.72356	2	605.3654175	-1.787977962	23.38008833
														EIMLAAK	3	1	1	N-Term(iTRAQ8plex); K7(iTRAQ8plex)	1.099	0.552	0.564	3.05	26	0	1383.84894	3	467.2848816	-2.73943762	28.638745
														EIMLAAK	1	1	1	N-Term(iTRAQ8plex); M3(Oxidation); K7(iTRAQ8plex)	1.135	0.681	0.604	-	28	0	1399.84009	3	467.2848816	-2.73943762	28.638745
17105394	RPL23A	60S ribosomal protein L23a	NP_000975.2	19.87	3	3	15	156	17.7	10.45	1.486	2.047	1.611																
														LAPDYDALDVANK	11	1	1	N-Term(iTRAQ8plex); K13(iTRAQ8plex)	1.587	2.155	1.498	4.77	51	0	2013.09722	2	1007.054016	-5.248467275	31.000485
														LDHYAIIKFPLTTESAMK	1	1	1	N-Term(iTRAQ8plex); K8(iTRAQ8plex); K18(iTRAQ8plex)	-	-	-	4.25	-	1	2990.69619	4	748.4295044	-6.370958693	39.60449333
														FPLTTESAMK	2	1	1	N-Term(iTRAQ8plex); K10(iTRAQ8plex)	1.461	2.027	1.976	3	38	0	1732.96367	2	866.9854736	-7.346174989	23.44074833
														FPLTTESAMK	1	1	1	N-Term(iTRAQ8plex); M9(Oxidation); K10(iTRAQ8plex)	1.401	1.525	1.659	-	31	0	1748.95769	2	866.9854736	-7.346174989	23.44074833
17149836	FKBP1A	peptidyl-prolyl cis-trans isomerase FKBP1A isoform a	NP_463460.1	25.00	2	2	6	108	11.9	8.16	0.781	1.713	2.937																
														GWEEGVAQMSVGQR	4	1	1	N-Term(iTRAQ8plex)	1.313	1.061	1.795	5.64	61	0	1837.92203	3	613.3121948	2.79115358	30.83257167
														GVQVETISPGDGR	2	2	1	N-Term(iTRAQ8plex)	0.754	2.81	4.23	-	63	0	1618.86516	2	809.9362183	-3.145040543	20.180005
17149842	FKBP2	peptidyl-prolyl cis-trans isomerase FKBP2 precursor	NP_004461.2	29.58	4	4	7	142	15.6	9.13	1.479	0.598	0.785																
														GWDQGLLGMCEGEK	2	1	1	N-Term(iTRAQ8plex); C10(Methylthio); K14(iTRAQ8plex)	1.614	0.536	0.723	4.88	51	0	2177.06028	3	726.3582764	-2.175616583	72.27979
														IPGGATLVFEVELLK	2	1	1	N-Term(iTRAQ8plex); K15(iTRAQ8plex)	0.906	0.479	0.534	4.73	43	0	2194.33097	3	732.1151733	0.084522132	56.723385
														KLVIPSELGYGER	2	1	1	N-Term(iTRAQ8plex); K1(iTRAQ8plex)	1.366	0.668	1.151	3.48	25	1	2069.21097	3	690.4085083	-5.108057033	29.08276167
														LVIPSELGYGER	1	1	1	N-Term(iTRAQ8plex)	1.601	0.695	0.852	-	32	0	1636.91606	2	818.9616699	-3.144984869	37.35241833
17158044	RPS6	40S ribosomal protein S6	NP_001001.2	23.29	5	5	31	249	28.7	10.84	1.736	2.406	2.060																
														MATEVAADALGEEWK	4	1	1	N-Term(iTRAQ8plex); M1(Oxidation); K15(iTRAQ8plex)	1.41	1.578	1.843	6.5	64	0	2245.16973	2	1115.078369	-8.415603831	68.255525
														MATEVAADALGEEWK	8	1	1	N-Term(iTRAQ8plex); K15(iTRAQ8plex)	1.922	2.423	2.159	5.98	83	0	2229.14946	2	1115.078369	-8.415603831	68.255525
														LNISFPATGCQK	8	1	1	N-Term(iTRAQ8plex); C10(Methylthio); K12(iTRAQ8plex)	1.83	2.693	2.186	5.46	42	0	1933.03159	2	967.0220337	-6.617327331	30.142535
														DIPGLTDTTVPR	4	1	1	N-Term(iTRAQ8plex)	1.355	1.666	1.742	3.59	48	0	1588.87895	2	794.9433594	-3.391805481	29.80456167
														LFNLSKEDDVR	3	1	1	N-Term(iTRAQ8plex); K6(iTRAQ8plex)	1.386	1.381	1.309	3.06	25	1	1944.10769	4	486.7779236	-5.774858595	25.69000833
														LIEVDDER	4	1	1	N-Term(iTRAQ8plex)	1.726	2.573	2.21	2.9	34	0	1292.69341	2	646.8503418	-5.08575832	17.53062
17402873	BCCIP	BRCA2 and CDKN1A-interacting protein isoform C	NP_510869.1	5.14	1	1	2	292	33.2	4.60	1.169	4.433	1.730																
														KGTQCVEQIQELVLR	2	3	1	N-Term(iTRAQ8plex); K1(iTRAQ8plex); C5(Methylthio)	1.169	4.433	1.73	3.82	46	1	2398.33188	3	800.1154785	-3.677557385	49.915335
17402893	PSAT1	phosphoserine aminotransferase isoform 1	NP_478059.1	18.65	6	6	17	370	40.4	7.66	1.401	2.195	2.578																
														NVGSAGVTVVIVR	4	2	1	N-Term(iTRAQ8plex)	1.271	3.132	3.726	6.55	82	0	1574.95841	3	525.6576538	3.312389555	28.57217167
														GVGISVLEMSHR	6	2	1	N-Term(iTRAQ8plex)	0.759	1.826	2.55	5.33	48	0	1588.87223	3	530.2955933	-3.82961244	28.38575167
														CADYVVTGAWSAK	2	2	1	N-Term(iTRAQ8plex); C1(Methylthio); K13(iTRAQ8plex)	1.554	2.472	2.607	4.49	43	0	2025.02359	3	675.6793823	-7.811552119	65.71423333
														FGTINIVHPK	2	2	1	N-Term(iTRAQ8plex); K10(iTRAQ8plex)	1.56	4.535	6.004	3.72	31	0	1734.03495	3	578.6831665	-9.9911702	26.42617833
														LPHSVLLEIQK	1	2	1	N-Term(iTRAQ8plex); K11(iTRAQ8plex)	1.263	1.948	1.566	3.44	-	0	1885.15812	3	629.0575562	-7.960124325	32.64010167
														ALELNMLSLK	2	1	1	N-Term(iTRAQ8plex); K10(iTRAQ8plex)	-	-	-	3.07	35	0	1740.03861	3	580.6843872	-9.41526747	67.17891667
17402904	EXOSC6	exosome complex component MTR3	NP_478126.1	2.21	1	1	1	272	28.2	6.28	0.686	0.853	0.697																
														LLCDFR	1	1	1	N-Term(iTRAQ8plex); C3(Methylthio)	0.686	0.853	0.697	-	21	0	1116.58208	2	558.7946777	-2.385360916	32.72273667
17738287	SCAMP4	secretory carrier-associated membrane protein 4	NP_524558.1	10.92	1	1	1	229	25.7	8.82	-	-	-																
														FIPVKPCFYQNFSDEIPVEH QVLVK	1	1	1	N-Term(iTRAQ8plex); K5(iTRAQ8plex); C7(Methylthio); K25(iTRAQ8plex)	-	-	-	3.86	-	0	3935.12647	5	787.8311157	-2.60733595	52.69658167
17933772	S100A16	protein S100-A16	NP_525127.1	66.99	6	6	43	103	11.8	6.79	0.514	1.001	0.400																
														ISFDEYWTLIGGITGPIAK	10	1	1	N-Term(iTRAQ8plex); K19(iTRAQ8plex)	1.06	1.008	0.576	5.6	60	0	2689.5018	2	1345.266235	7.069196886	62.14216333
														LIQNLDANHDGR	10	1	1	N-Term(iTRAQ8plex)	0.403	0.89	0.324	4.82	57	0	1669.88945	3	557.302124	-0.330847592	21.72356
														AVIVLVENFYK	15	1	1	N-Term(iTRAQ8plex); K11(iTRAQ8plex)	0.284	0.948	0.384	4.3	57	0	1903.15665	3	635.0570679	2.800118046	59.51983667
														ELNHMLSDTGNR	4	1	1	N-Term(iTRAQ8plex); M5(Oxidation)	0.629	0.89	0.434	3.12	23	0	1706.84214	3	569.6188965	-0.726544563	12.93602
														AADKLIQNLDANHDGR	1	1	1	N-Term(iTRAQ8plex); K4(iTRAQ8plex)	0.688	1.187	-	2.98	-	1	2359.28676	4	590.5771484	-3.021010463	26.460205
														ELNHMLSDTGNR	2	1	1	N-Term(iTRAQ8plex)	1.21	1.223	0.81	2.78	19	0	1690.86179	3	569.6188965	-0.726544563	12.93602
														LIHEQEQQSSS	1	1	1	N-Term(iTRAQ8plex)	0.366	1.271	0.359	-	57	0	1589.80974	2	795.4085083	1.546525663	17.9762
17978489	ADGRE5	CD97 antigen isoform 2 preproprotein	NP_001775.2	2.16	1	1	6	742	81.7	7.05	4.571	1.771	2.230																
														HLIATQLLSNLEDIMR	6	4	1	N-Term(iTRAQ8plex)	4.571	1.771	2.23	5.91	69	0	2171.21775	3	724.4107666	0.785390913	65.004835
17999528	COX6A1	cytochrome c oxidase subunit 6A1, mitochondrial	NP_004364.2	26.61	1	1	1	109	12.1	9.32	-	-	-																
														TKPFPWGDGNHTLFHNP HVNPLPTGYEDE	1	1	1	N-Term(iTRAQ8plex); K2(iTRAQ8plex)	-	-	-	2.94	-	0	3924.94985	4	981.9929199	-1.437341295	37.87101167
18104948	RPL21	60S ribosomal protein L21	NP_000973.2	20.63	3	3	16	160	18.6	10.49	1.033	1.777	1.828																
														VYNVTQHAVGIVVNK	8	1	1	N-Term(iTRAQ8plex); K15(iTRAQ8plex)	1.092	2.654	2.659	4.97	52	0	2249.31021	3	750.4415894	-5.524575273	33.58448833
														HGVVPLATYMR	5	1	1	N-Term(iTRAQ8plex)	1.033	1.626	1.392	3.92	44	0	1547.861	3	516.6251831	-3.879698247	26.486635
														HGVVPLATYMR	1	1	1	N-Term(iTRAQ8plex); M10(Oxidation)	0.814	0.654	0.78	2.76	-	0	1563.85508	3	516.6251831	-3.879698247	26.486635
														GMGTVQK	1	1	1	N-Term(iTRAQ8plex); M2(Oxidation); K7(iTRAQ8plex)	1.546	1.777	1.86	2.71	-	0	1344.76453	3	448.9263611	-8.950487448	9.948886667
														HGVVPLATYMR	1	1	1	N-Term(iTRAQ8plex); Y9(iTRAQ8plex)	-	-	-	-	26	0	1852.06106	3	516.6251831	-3.879698247	26.486635
18105048	HIST1H2BK	histone H2B type 1-K	NP_542160.1	71.43	2	12	473	126	13.9	10.32	1.264	0.652	0.595																
														KESYSVYVYK	15	12	1	N-Term(iTRAQ8plex); K1(iTRAQ8plex); K10(iTRAQ8plex)	1.466	0.682	0.742	4.09	31	1	2178.25687	3	726.7571411	-0.207142767	27.8557
														ESYSVYVYK	25	12	1	N-Term(iTRAQ8plex); K9(iTRAQ8plex)	1.14	0.594	0.584	3.89	49	0	1745.95366	2	873.479126	-3.445667696	22.42496167
														ESYSVYVYK	1	12	1	N-Term(iTRAQ8plex); Y3(iTRAQ8plex); K9(iTRAQ8plex)	1.042	0.675	0.528	-	26	0	2050.16728	2	873.479126	-3.445667696	22.42496167
														AMGIMNSFVNDIFER	55	17	2	N-Term(iTRAQ8plex); M2(Oxidation); M5(Oxidation)	-	-	-	7.49	84	0	2080.00713	2	1024.521362	5.222902458	67.32255667
														AMGIMNSFVNDIFER	121	17	2	N-Term(iTRAQ8plex); M2(Oxidation)	-	-	-	6.73	92	0	2064.02519	2	1024.521362	5.222902458	67.32255667
														AMGIMNSFVNDIFER	107	17	2	N-Term(iTRAQ8plex)	-	-	-	6.11	103	0	2048.03545	2	1024.521362	5.222902458	67.32255667
														HAVSEGTK	28	18	2	N-Term(iTRAQ8plex); K8(iTRAQ8plex)	-	-	-	4.68	45	0	1436.83133	3	479.6161499	1.486015215	9.764976667
														QVHPDTGISSK	30	18	2	N-Term(iTRAQ8plex); K11(iTRAQ8plex)	-	-	-	4.06	50	0	1776.99167	2	889.0061646	-0.781550078	14.01388
														LLLPGELAK	73	18	2	N-Term(iTRAQ8plex); K9(iTRAQ8plex)	-	-	-	3.96	36	0	1562.01006	2	781.5079956	-3.237311684	35.57815
														YTSAK	1	8	2	N-Term(iTRAQ8plex); K5(iTRAQ8plex)	-	-	-	2.78	-	0	1177.70386	3	393.2394714	0.152633142	11.19466
														EIQTAVR	10	17	2	N-Term(iTRAQ8plex)	-	-	-	-	32	0	1120.65927	3	374.2246094	-3.137818139	14.11598833
														LAHYNK	1	15	2	N-Term(iTRAQ8plex); K6(iTRAQ8plex)	-	-	-	-	27	0	1353.80912	3	451.9412231	-0.565158261	18.01176667
														IAGEASR	1	15	2	N-Term(iTRAQ8plex)	-	-	-	-	26	0	1007.57665	2	504.2919617	-2.068904796	13.73985833
														SAPAPK	3	16	2	N-Term(iTRAQ8plex); K6(iTRAQ8plex)	-	-	-	-	23	0	1178.73804	3	393.584198	2.290113704	9.457145
														STITSR	2	17	2	N-Term(iTRAQ8plex)	-	-	-	-	20	0	968.56542	2	484.7863464	-2.472779707	9.323636667
18201905	GPI	glucose-6-phosphate isomerase isoform 2	NP_000166.2	43.01	17	17	103	558	63.1	8.32	1.197	2.517	3.864																
														LTPFMLGALVAMYEHK	6	4	1	N-Term(iTRAQ8plex); K16(iTRAQ8plex)	1.082	3.399	6.746	7.98	56	0	2429.3646	3	810.4597168	4.126939463	66.48480333
														TITDVINIGIGGSDLGPLM VTEALKPYSSGGPR	2	2	1	N-Term(iTRAQ8plex); K25(iTRAQ8plex)	-	-	-	5.62	-	0	3937.1288	4	985.0376587	-7.062612313	82.88284333
														ILLANFLAQTEALMR	6	4	1	N-Term(iTRAQ8plex)	-	-	-	5.49	81	0	2008.15666	2	1004.58197	-0.038587782	69.05770667
														ILLANFLAQTEALMR	3	4	1	N-Term(iTRAQ8plex); M14(Oxidation)	0.691	1.915	2.84	4.9	65	0	2024.15348	2	1004.58197	-0.038587782	69.05770667
														SNTPILVDGKDVMPEVNK	5	4	1	N-Term(iTRAQ8plex); K10(iTRAQ8plex); K18(iTRAQ8plex)	-	-	-	4.85	33	1	2868.60732	4	717.9072876	-6.804970912	31.62625333
														VKEFGIDPQNMFEFWDW VGGR	2	4	1	N-Term(iTRAQ8plex); K2(iTRAQ8plex)	-	-	-	4.39	31	1	3165.60952	4	792.1578369	0.558020893	66.57933167
														FAAYFQQGDMESNGK	2	4	1	N-Term(iTRAQ8plex); K15(iTRAQ8plex)	0.795	1.59	2.206	4.35	56	0	2301.12137	3	767.7119751	-9.416824899	56.35971333
														TLAQLNPESSLFIIASK	8	4	1	N-Term(iTRAQ8plex); K17(iTRAQ8plex)	1.969	2.535	3.388	4.16	36	0	2440.41684	3	814.1437988	-4.240286102	53.021565
														TFTTQETITNAETAK	2	4	1	N-Term(iTRAQ8plex); K15(iTRAQ8plex)	0.752	3.789	6.785	4.16	35	0	2264.20475	3	755.4064331	-8.08461939	34.52954833
														MIPCDFLIPVQTQHPIR	8	4	1	N-Term(iTRAQ8plex); M1(Oxidation); C4(Methylthio)	0.875	1.736	1.932	4.16	31	0	2374.24308	3	786.7542114	1.78892432	48.93771333
														MIPCDFLIPVQTQHPIR	10	4	1	N-Term(iTRAQ8plex); C4(Methylthio)	3.507	9.199	17.525	4.14	43	0	2358.24332	3	786.7542114	1.78892432	48.93771333
														VWYVSNIDGTHIAK	3	4	1	N-Term(iTRAQ8plex); K14(iTRAQ8plex)	1.065	1.781	2.771	4	32	0	2211.22109	4	553.56073	-7.756464608	33.10722833
														HFVALSTNTTK	5	4	1	N-Term(iTRAQ8plex); K11(iTRAQ8plex)	2.914	4.561	5.799	3.93	61	0	1827.04117	3	609.6852417	-9.468561853	16.15069833
														EWFLQAAK	11	4	1	N-Term(iTRAQ8plex); K8(iTRAQ8plex)	1.32	3.31	4.868	3.75	39	0	1600.91704	2	800.9621582	-8.571033607	34.33352667
														MIPCDFLIPVQTQHPIRK	3	4	1	N-Term(iTRAQ8plex); C4(Methylthio); K18(iTRAQ8plex)	1.267	3.274	3.859	3.58	-	1	2790.54287	4	698.3911743	-0.474787322	46.67880167
														VFEGNRPTNSIVFTK	2	4	1	N-Term(iTRAQ8plex); K15(iTRAQ8plex)	1.419	2.9	5.607	3.4	-	0	2317.2997	4	580.0803833	-5.518482075	30.268225
														AVLHVALR	21	4	1	N-Term(iTRAQ8plex)	0.768	1.127	2.407	3.34	50	0	1182.75409	3	394.9228821	-7.066819566	19.34796833
														NLVTEDVMR	1	4	1	N-Term(iTRAQ8plex)	1.31	1.805	2.159	3.16	-	0	1380.73479	2	690.8710327	-8.041001359	19.690625
														MIPCDFLIPVQTQHPIRK	1	4	1	N-Term(iTRAQ8plex); M1(Oxidation); C4(Methylthio); K18(iTRAQ8plex)	0.593	0.756	0.897	2.76	-	1	2806.52724	4	698.3911743	-0.474787322	46.67880167
														SPEDLER	2	5	1	N-Term(iTRAQ8plex)	1.186	3.168	4.462	-	21	0	1149.59868	2	575.3029785	-5.794099161	15.48122
18252047	SLC43A3	solute carrier family 43 member 3 isoform 1	NP_060081.1	1.83	1	1	1	491	54.5	8.53	8.037	0.914	0.837																
														SFWSYAFSR	1	2	1	N-Term(iTRAQ8plex)	8.037	0.914	0.837	3.26	-	0	1454.73235	2	727.869812	-3.206016949	64.90348667
18375501	APEX1	DNA-(apurinic or apyrimidinic site) lyase	NP_001632.2	36.48	7	7	28	318	35.5	8.12	1.685	3.015	1.723																

														KPLVLCGDLNVAHEEIDLR	4	1	1	N-Term(iTRAQ8plex); K1(iTRAQ8plex); C6(Methylthio)	-	-	-	6.77	63	0	2788.52085	3	930.1784668	-3.659703454	45.66285167
														QGFGELLQAVPLADSFR	2	1	1	N-Term(iTRAQ8plex)	2.661	4.744	3.788	5.8	75	0	2152.16582	2	1076.586548	-2.166290735	59.71201333
														VSYGIGDEEHDQEGR	10	1	1	N-Term(iTRAQ8plex)	1.343	2.007	1.577	4.72	59	0	1994.93022	2	997.96875	-2.775996842	17.14586333
														HLYPNTPYAYTFWTYMMNAR	1	1	1	N-Term(iTRAQ8plex)	0.754	0.677	0.711	3.96	-	0	2844.35135	4	787.8949585	-1.701475919	53.04084667
														GAVAEDGDELRTEPEAK	2	1	1	N-Term(iTRAQ8plex); K17(iTRAQ8plex)	3.229	6.178	4.718	3.88	38	1	2395.24224	2	1198.124756	-5.826634586	19.47916333
														ICSWNVDGLR	6	1	1	N-Term(iTRAQ8plex); C2(Methylthio)	2.269	5.753	4.638	3.7	38	0	1512.75176	2	756.8841553	0.358038621	42.61863333
														LDYFLLSHSLLPALCDSK	1	1	1	N-Term(iTRAQ8plex); C15(Methylthio); K18(iTRAQ8plex)	-	-	-	3.42	-	0	2689.44546	4	673.1168213	-3.694822563	66.74768333
														HLYPNTPYAYTFWTYMMNAR	2	1	1	N-Term(iTRAQ8plex); Y10(iTRAQ8plex)	-	-	1.737	-	30	0	3148.558	4	787.8949585	-1.701475919	53.04084667
18375623	DDX39B	spliceosome RNA helicase DDX39B	NP_542165.1	47.90	9	16	98	428	49.0	5.67	0.623	1.147	0.822																
														VNIAFNYDMPEDSDTYLHR	6	1	1	N-Term(iTRAQ8plex)	0.68	1.312	0.871	5.85	53	0	2604.22812	3	868.7475586	-2.357231571	37.91844333
														FMQDPMEIFVDDETK	4	1	1	N-Term(iTRAQ8plex); K15(iTRAQ8plex)	0.686	1.262	0.924	5.11	60	0	2453.23882	2	1227.123047	8.101873271	52.05582833
														CIALAQLLVEQNFPAIAIHR	4	1	1	N-Term(iTRAQ8plex); C1(Methylthio)	-	-	-	4.94	77	0	2570.42136	3	857.4786377	-1.549342892	71.42855833
														VAVFFGGLSIK	8	1	1	N-Term(iTRAQ8plex); K11(iTRAQ8plex)	0.643	1.108	0.814	4.76	63	0	1746.06645	2	873.5363159	-6.921896724	44.73715
														HFILDECDKMLEQLDMR	1	1	1	N-Term(iTRAQ8plex); C7(Methylthio); K9(iTRAQ8plex)	-	-	-	4.66	-	1	2790.39736	4	698.3547974	2.349942421	63.80969833
														HFILDECDK	4	1	1	N-Term(iTRAQ8plex); C7(Methylthio); K9(iTRAQ8plex)	0.608	0.857	0.72	4.4	39	0	1773.90396	3	591.9728394	-4.762086202	29.45848333
														FMQDPMEIFVDDETK	2	1	1	N-Term(iTRAQ8plex); M2(Oxidation); M6(Oxidation); K15(iTRAQ8plex)	0.29	1.147	0.714	3.97	31	0	2485.20151	2	1227.123047	8.101873271	52.05582833
														NCPHIVVGTPGR	14	1	1	N-Term(iTRAQ8plex); C2(Methylthio)	0.621	1.039	0.774	3.86	48	0	1599.83493	3	533.9505005	-1.996506681	22.209255
														QVMMFSATLSK	2	1	1	N-Term(iTRAQ8plex); K11(iTRAQ8plex)	1.01	0.765	0.822	2.96	27	0	1851.01609	3	617.6768799	-9.073341764	53.26581167
														VNIAFNYDMPEDSDTYLHR	2	1	1	N-Term(iTRAQ8plex); Y16(iTRAQ8plex)	0.417	1.556	1.102	-	33	0	2908.4291	3	868.7475586	-2.357231571	37.91844333
														ILALAR	2	10	1	N-Term(iTRAQ8plex)	0.643	1.254	0.868	-	27	0	960.65123	2	480.8292542	0.479238215	32.794705
														GLAITFVSDENDAK	14	3	2	N-Term(iTRAQ8plex); K14(iTRAQ8plex)	-	-	-	6.06	79	0	2088.12632	2	1044.567505	-7.484290413	26.62412
														LTLHGLQQYYVK	10	3	2	N-Term(iTRAQ8plex); K12(iTRAQ8plex)	-	-	-	4.75	41	0	2071.20659	2	1036.106934	-4.553255355	34.16349
														AIVDCGFEHPSEVQHECIPQAILGMDVLCQAK	1	2	2	N-Term(iTRAQ8plex); C5(Methylthio); C17(Methylthio); C29(Methylthio); [illegible]	-	-	-	4.54	-	0	4227.01772	4	1057.509888	0.425028086	64.3324
														DFLLKPELLR	8	2	2	N-Term(iTRAQ8plex); K5(iTRAQ8plex)	-	-	-	3.57	31	0	1852.14531	2	926.5745239	-5.345200131	36.26787833
														ILVATNLFGR	11	3	2	N-Term(iTRAQ8plex)	-	-	-	3.34	33	0	1407.85832	2	704.4328003	-3.016876052	49.87545833
														RDVQEIFR	3	3	2	N-Term(iTRAQ8plex)	-	-	-	3.21	37	1	1366.76714	2	683.887207	-5.373159259	19.86109167
														ELAFQISK	2	2	2	N-Term(iTRAQ8plex); K8(iTRAQ8plex)	-	-	-	3.06	40	0	1543.93393	3	515.3161621	2.274565025	38.51634333
18375673	UPF1	regulator of nonsense transcripts 1 isoform 2	NP_002902.2	1.88	2	2	4	1118	123.0	6.68	0.914	1.966	1.757																
														ADSVVVLLCR	2	4	1	N-Term(iTRAQ8plex); C9(Methylthio)	0.829	1.925	1.699	3.33	47	0	1424.79021	2	712.8987427	-0.367470547	48.870825
														ESQTQDNITVR	2	4	1	N-Term(iTRAQ8plex)	1.008	2.007	1.818	3.17	44	0	1594.83037	2	797.9188232	-2.17668016	17.24132
18379349	VAT1	synaptic vesicle membrane protein VAT-1 homolog	NP_006364.2	10.69	3	3	7	393	41.9	6.29	0.615	0.695	1.049																
														GVDIVMDPLGGSDTAK	2	1	1	N-Term(iTRAQ8plex); K16(iTRAQ8plex)	0.557	0.37	1.383	4.6	59	0	2183.18584	2	1092.096558	0.901120894	34.95867833
														VVTYGMANLLTGPK	4	1	1	N-Term(iTRAQ8plex); K14(iTRAQ8plex)	0.782	0.732	0.993	4.42	43	0	2072.18552	3	691.4000244	-8.653331296	34.90518333
														ACGLNFADLMAR	1	1	1	N-Term(iTRAQ8plex); C2(Methylthio)	-	-	-	-	24	0	1631.79343	3	544.6026611	-4.640386107	52.21833333
18379366	ZMPSTE24	CAAX prenyl protease 1 homolog	NP_005848.2	14.53	5	5	17	475	54.8	7.49	1.488	0.668	0.556																
														NEEVLAVLGHELGHWK	4	1	1	N-Term(iTRAQ8plex); K16(iTRAQ8plex)	1.271	0.471	-	5.78	34	0	2439.35512	4	610.5942383	-2.208803035	50.12188167
														IVLFDTLLEEYSVLNK	5	1	1	N-Term(iTRAQ8plex); K16(iTRAQ8plex)	1.562	0.658	0.427	4.91	54	0	2504.44333	3	835.491333	4.869103973	64.55980833
														DIQEDSGMEPR	2	1	1	N-Term(iTRAQ8plex)	0.83	0.331	0.31	3.8	31	0	1580.74639	2	790.8768311	-4.084026814	14.77975667
														LKEEIEVMAK	3	1	1	N-Term(iTRAQ8plex); K2(iTRAQ8plex); K10(iTRAQ8plex)	1.567	0.794	0.628	3.16	23	1	2102.277	4	526.324707	5.30707657	41.46686
														GMWASLDALWEMPAEK	1	1	1	N-Term(iTRAQ8plex); K16(iTRAQ8plex)	1.026	0.518	0.354	-	47	0	2443.26505	3	815.0932007	1.619759094	58.83811
														DIQEDSGMEPR	2	1	1	N-Term(iTRAQ8plex); M8(Oxidation)	1.894	0.964	0.673	-	19	0	1596.74712	2	790.8768311	-4.084026814	14.77975667
18390331	GFM1	elongation factor G, mitochondrial isoform 2	NP_079272.4	15.18	7	7	16	751	83.4	7.01	1.409	1.086	0.751																
														FVLQDGAHHMVDSNEISFIR	5	3	1	N-Term(iTRAQ8plex)	2.182	1.103	0.777	5.19	44	0	2619.32314	4	655.5862427	-2.309939076	37.32931167
														YNVPFLTFINKLDR	5	4	1	N-Term(iTRAQ8plex); K11(iTRAQ8plex)	1.336	1.118	0.633	3.71	44	1	2348.3613	3	783.4586182	1.1071304	60.41739667
														SFTPVFLGSALK	1	4	1	N-Term(iTRAQ8plex); K12(iTRAQ8plex)	1.409	1.182	0.83	3.54	-	0	1875.10801	3	625.7075195	-6.413473899	34.387225
														DNSHPFVGLAFK	2	4	1	N-Term(iTRAQ8plex); K12(iTRAQ8plex)	1.119	0.816	0.751	3.38	33	0	1940.06943	3	647.3613281	-8.046193992	36.82959167
														VIGVLEPLDPEDYTKLEFSDETFGSNIPK	1	4	1	N-Term(iTRAQ8plex); K15(iTRAQ8plex); K29(iTRAQ8plex)	-	-	-	3.2	-	1	4165.25503	4	1042.069214	4.552971819	57.14665833
														LNHNAAFMQIPMGLEGNFK	1	3	1	N-Term(iTRAQ8plex); K19(iTRAQ8plex)	-	-	-	3.18	-	0	2740.45424	4	685.8690186	0.668881356	44.34879
														IPSISDLK	1	4	1	N-Term(iTRAQ8plex); K8(iTRAQ8plex)	1.781	1.069	0.727	-	26	0	1480.91533	2	740.9613037	-2.829667597	44.22889167
18426973	DRAP1	dr1-associated corepressor	NP_006433.2	6.34	1	1	2	205	22.3	5.17	1.453	1.715	1.719																
														QCIELEQQFDFLK	2	1	1	N-Term(iTRAQ8plex); C2(Methylthio); K13(iTRAQ8plex)	1.453	1.715	1.719	3.93	32	0	2295.19663	3	765.7370605	-0.328472675	46.39051667
18482373	WDFY1	WD repeat and FYVE domain-containing protein 1	NP_065881.1	2.68	1	1	1	410	46.3	7.33	0.757	0.688	0.729																
														EDGVITASEDR	1	1	1	N-Term(iTRAQ8plex)	0.757	0.688	0.729	2.69	-	0	1495.75788	3	499.2574768	2.45084447	21.40778833
18490991	PBK	lymphokine-activated killer T-cell-originated protein kinase isoform 1	NP_060962.2	4.35	1	1	4	322	36.1	5.12	1.398	3.088	6.223																
														VIELFSVCTNEDPK	4	2	1	N-Term(iTRAQ8plex); C8(Methylthio); K14(iTRAQ8plex)	1.398	3.088	6.223	3.86	43	0	2248.1799	3	750.0648193	-0.666113145	61.41375167
18497304	CHST14	carbohydrate sulfotransferase 14	NP_569735.1	3.46	1	1	2	376	43.0	9.48	0.967	0.641	0.897																
														LEADANQVLEWVR	2	1	1	N-Term(iTRAQ8plex)	0.967	0.641	0.897	3.65	46	0	1847.00237	2	924.0048218	3.164930805	52.73804833
18644728	NOL6	nucleolar protein 6 alpha isoform	NP_075068.2	3.14	2	2	5	1146	127.5	7.64	1.402	0.840	0.990																
														LAELLTQQHGLQCR	4	2	1	N-Term(iTRAQ8plex); C13(Methylthio)	1.272	0.822	0.829	4.9	43	0	1960.02903	3	654.0145264	-6.125648283	42.06653
														LNLLAHSRPPVPEWDISQDPPK	1	2	1	N-Term(iTRAQ8plex); K22(iTRAQ8plex)	1.402	1.532	3.1	3.59	-	0	3117.72646	4	780.1870728	-1.334358362	41.54279
18644883	ATP5J	ATP synthase-coupling factor 6, mitochondrial isoform a precursor	NP_001676.2	26.85	2	2	4	108	12.6	9.52	0.779	0.505	0.536																
														QMFGNADMNTFPTFK	2	2	1	N-Term(iTRAQ8plex); K15(iTRAQ8plex)	0.779	0.505	0.536	4.74	37	0	2357.17984	3	786.3981323	-3.418312508	33.26178667
														FEDPKFEVIEKPQA	2	2	1	N-Term(iTRAQ8plex); K5(iTRAQ8plex); K11(iTRAQ8plex)	0.635	0.33	0.496	3.73	-	1	2589.45371	3	863.8227539	-5.975326456	31.47970833
18677735	PRRC1	protein PRRC1 isoform 2	NP_570721.1	6.07	2	2	9	445	46.7	5.83	0.503	1.386	1.770																
														WSGLLVTVGEVLEK	8	2	1	N-Term(iTRAQ8plex); K14(iTRAQ8plex)	0.304	1.274	1.47	5.53	65	0	2138.2692	2	1069.637573	-0.140971872	61.42254167
														SGGELDIVVTSNK	1	2	1	N-Term(iTRAQ8plex); K13(iTRAQ8plex)	0.834	1.62	2.132	2.78	-	0	1927.07663	3	643.0303955	-9.87922651	34.27258333
18702331	OTUD7A	OTU domain-containing protein 7A	NP_570971.1	1.51	1	1	3	926	100.6	8.40	-	0.024	0.484																
														LSLNILRAAMQGER	3	1	1	N-Term(iTRAQ8plex)	-	0.024	0.484	3.26	-	1	1876.07231	2	938.5397949	-0.937104736	56.600175
18765731	SNAP23	synaptosomal-associated protein 23 isoform SNAP23B	NP_570710.1	8.86	1	1	1	158	17.8	4.69	1.169	0.935	0.727																
														ILGLAIESQDAGIK	1	5	1	N-Term(iTRAQ8plex); K14(iTRAQ8plex)	1.169	0.935	0.727	-	28	0	2036.22099	2	1018.614136	-0.111203855	42.34578333
18860916	XRN2	5'-3' exoribonuclease 2 isoform 2	NP_036387.2	4.63	2	2	3	950	108.5	7.47	0.848	0.849	1.436																
														HDELADSLPCAEGEFIFLR	2	4	1	N-Term(iTRAQ8plex); C10(Methylthio)	0.848	0.849	1.436	5.08	36	0	2512.22678	3	838.0804443	4.500970177	57.69498667
														LMSDPDSSIIDFYPEDFAIDLNGKK	1	3	1	N-Term(iTRAQ8plex); K24(iTRAQ8plex); K25(iTRAQ8plex)	-	-	-	4.79	-	1	3742.95986	4	936.4954224	-1.472558371	55.90731
19224660	PACSIN3	protein kinase C and casein kinase substrate in neurons protein 3	NP_057307.2	12.03	4	4	9	424	48.5	6.18	0.808	0.619	0.555																
														DLHQGIEAASDEEDLR	4	1	1	N-Term(iTRAQ8plex)	0.737	0.413	0.555	4.71	55	0	2102.02585	3	701.3468018	-2.16899167	23.25103333
														AQYEQTLAELHR	2	1	1	N-Term(iTRAQ8plex)	0.994	0.615	0.359	4.04	41	0	1762.93613	3	588.3168945	-1.611491308	24.96533333
														AWHAFFTAAER	2	1	1	N-Term(iTRAQ8plex)	0.857	1.1	0.645	3.34	40	0	1610.83133	3	537.6152954	-4.232795446	35.972685
														GAFHRPVLGGFR	1	1	1	N-Term(iTRAQ8plex)	-	-	-	3.02	-	0	1617.93764	4	405.2398682	5.971476276	21.90194167
19718759	MYOF	myoferlin isoform b	NP_579899.1	34.18	55	55	196	2048	233.3	6.21	1.009	0.527	0.695																
														DIVIEMEDTKPLLASK	2	7	1	N-Term(iTRAQ8plex); K10(iTRAQ8plex); K16(iTRAQ8plex)	1.564	0.676	1.033	6.64	40	0	2714.5768	4	679.3996582	-0.353044901	40.08027
														SLLTEADAGHTEFTDEVYQNESR	9	7	1	N-Term(iTRAQ8plex)	0.914	0.431	0.717	5.79	47	0	2916.36911	3	972.7956543	-2.654551027	35.14677833
														GWEWEGEWIVDPER	4	7	1	N-Term(iTRAQ8plex)	1.165	0.567	0.73	5.69	80	0	2092.00054	2	1046.503906	-3.487293562	39.316215
														LEPISNDDLLVVEK	4	7	1	N-Term(iTRAQ8plex); K14(iTRAQ8plex)	1.021	0.505	0.65	5.66	52	0	2192.27739	2	1096.642334	6.345022215	38.00044167
														HSATTVFGANTPIVSCNFDR	8	7	1	N-Term(iTRAQ8plex); C16(Methylthio)	0.858	0.783	0.85	5.48	50	0	2487.20523	3	829.7399292	-0.421337302	41.02719333
														IDVGFVYDEPGHAVMR	4	7	1	N-Term(iTRAQ8plex)	0.874	1.002	1.277	5.37	52	0	2109.06736	3	703.6939697	-3.212495937	33.14075833
														KVDNELNPVWNEILEFDLR	8	6	1	N-Term(iTRAQ8plex); K1(iTRAQ8plex)	1.603	-	0.543	5.08	47	1	2951.61765	3	984.5440674	3.00770611	60.261375
														SLGPPGPPFNITPR	4	6	1	N-Term(iTRAQ8plex)	1.034	0.411	0.779	5.04	55	0	1753.98625	2	877.4967651	-2.313385612	35.00105833
														NLVDPFVEVSFAGK	4	23	1	N-Term(iTRAQ8plex); K14(iTRAQ8plex)	1.136	0.527	0.88	4.91	48	0	2130.19974	3	710.7380981	-2.736887201	37.28023167
														EELYMPPLVIK	2	7	1	N-Term(iTRAQ8plex); K11(iTRAQ8plex)	1.131	0.462	0.709	4.86	35	0	1940.14116	2	970.5742188	1.25742397	39.80108667
														AEDIPQMDDAFSQTVK	3	7	1	N-Term(iTRAQ8plex); K16(iTRAQ8plex)	1.128	0.674	0.882	4.83	63	0	2403.24272	2	1202.125	4.349157036	34.66965667
														LQMWVDVFPK	8	6	1	N-Term(iTRAQ8plex); K10(iTRAQ8plex)	1.048	0.51	0.677	4.8	52	0	1871.05864	3	624.3600464	-2.883592047	75.75499833
														DYSLDEFEANK	6	7	1	N-Term(iTRAQ8plex); K11(iTRAQ8plex)	0.974	0.496	0.643	4.77	66	0	1938.98398	2	969.9938965	-5.159902931	39.98578667
														FGSHCGIPEEYCVSGVNTWR	6	7	1	N-Term(iTRAQ8plex); C5(Methylthio); C12(Methylthio)	1.199	1.18	1.248	4.68	45	0	2637.16422	3	879.7262573	-0.703246817	48.929845
														TQGLVPEHVETR	14	7	1	N-Term(iTRAQ8plex)	0.863	0.342	0.636	4.63	56	0	1669.91191	3	557.3098145	-1.575643549	17.70783667
														DRDNDSDDVESNLLLPAGIALR	4	7	1	N-Term(iTRAQ8plex)	1.045	1.151	1.982	4.59	32	1	2702.39096	3	901.4685059	0.371163218	40.640475
														WLLLNDPEDTSSGSK	2	7	1	N-Term(iTRAQ8plex); K15(iTRAQ8plex)	0.946	0.73	0.669	4.58	57	0	2270.19797	3	757.4041748	-6.400382752	60.41108
														EYTGFPDPYDELNTGK	2	7	1	N-Term(iTRAQ8plex); K16(iTRAQ8plex)	0.903	0.347	0.593	4.45	48	0	2454.23125	2	1227.619263	1.107259402	35.42053
														ILHQHLGAPEER	5	7	1	N-Term(iTRAQ8plex)	0.833	0.673	0.819	4.41	54	0	1703.96099	2	852.4841309	6.745341502	17.16572
														STLAEIEDWLDK	2	7	1	N-Term(iTRAQ8plex); K12(iTRAQ8plex)	0.851	0.315	0.512	4.39	36	0	2028.09098	3	676.7018433	-9.868770718	76.27003167
														ISVYDYDTFTR	4	7	1	N-Term(iTRAQ8plex)	1.05	0.461	0.608	4.29	50	0	1683.84758	2	842.4274292	-3.313554993	33.35492167
														VGETIIDLENR	2	7	1	N-Term(iTRAQ8plex)	0.854	0.642	0.674	4.24	26	0	1562.87309	2	781.9401855	2.503833334	34.92146333
														VIEDRDHYIPNTLNPVFGR	1	7	1	N-Term(iTRAQ8plex)	0.716	1.571	1.29	4.21	-	1	2559.35268	4	640.5936279	-3.715572815	34.54195667
														AMEELQDQEGWEYASLIGWK	2	7	1	N-Term(iTRAQ8plex); K20(iTRAQ8plex)	0.807	0.32	0.728	4.14	75	0	2991.52812	2	1496.2677	8.758891624	53.80680833
														ILVELATFLEK	6	7	1	N-Term(iTRAQ8plex); K11(iTRAQ8plex)	1.051	0.382	0.583	4.08	71	0	1884.1613	2	942.5898438	3.056822171	65.63169833
														VIMELFDNDQVGKDEFLGR	3	7	1	N-Term(iTRAQ8plex); K13(iTRAQ8plex)	0.999	0.31	0.519	4.07	24	1	2833.50258	4	709.1311035	0.368054191	56.30322667
														LDLPNRPETSFLWFTNPCK	2	6	1	N-Term(iTRAQ8plex); C18(Methylthio); K19(iTRAQ8plex)	1.424	0.798	0.667	4.02	-	0	2932.53092	3	978.1818237	-0.042406576	56.79325833
														GIPLDFSSSLGIIVK	2	6	1	N-Term(iTRAQ8plex); K15(iTRAQ8plex)	1.2	0.663	0.847	4.01	44	0	2154.29111	3	718.7685547	-3.880853176	48.54378167
														VEMTLEILNEK	2	6	1	N-Term(iTRAQ8plex); K11(iTRAQ8plex)	0.666	0.252	0.543	3.93	28	0	1927.09586	3	643.0368042	-3.742394188	71.43008833
														DHYIPNTLNPVFGR	2	7	1	N-Term(iTRAQ8plex)	1.89	1.984	1.68	3.91	43	0	1947.03293	3	649.6824951	-3.132560491	32.391135
														AMEELQDQEGWEYASLIGWK	2	7	1	N-Term(iTRAQ8plex); M2(Oxidation); K20(iTRAQ8plex)	0.681	0.601	0.6	3.9	36	0	3007.51151	2	1496.2677	8.758891624	53.80680833
														LIDEVIEDTR	4	7	1	N-Term(iTRAQ8plex)	1.044	0.467	0.619	3.81	56	0	1506.84319	2	753.9252319	7.602614051	44.95307333
														MAPSETHGAAAIFK	2	7	1	N-Term(iTRAQ8plex); K14(iTRAQ8plex)	1	0.833	0.731	3.74	34	0	2039.1066	3	680.3737183	-6.796979046	22.63296333
														LTEKEEEIVDWWSK	1	2	1	N-Term(iTRAQ8plex); K4(iTRAQ8plex); K14(iTRAQ8plex)	1.317	0.257	0.609	3.71	-	1	2704.49892	4	676.880188	1.035597797	52.30913833
														MYELSCYLPQEK	4	7	1	N-Term(iTRAQ8plex); C6(Methylthio); K12(iTRAQ8plex)	1.099	1.147	1.131	3.68	34	0	2158.07378	2	1079.540527	-4.876678734	38.16426167
														KLEPISNDDLLVVEK	1	7	1	N-Term(iTRAQ8plex); K1(iTRAQ8plex); K15(iTRAQ8plex)	0.484	-	0.566	3.66	-	1	2624.54848	4	656.8925781	-5.840501748	34.712625

														LISLLNEK	2	6	1	N-Term(iTRAQ8plex); K8(iTRAQ8plex)	1.235	0.618	0.756	3.62	27	0	1537.97576	2	769.4915161	-1.050532449	66.52455
														WVTFLLK	6	7	1	N-Term(iTRAQ8plex); K7(iTRAQ8plex)	0.944	0.31	0.562	3.59	30	0	1514.95781	2	757.9825439	1.518174703	75.46323167
														DLTQTASSTAR	3	7	1	N-Term(iTRAQ8plex)	1.01	0.407	0.548	3.56	38	0	1454.77495	2	727.8911133	-0.214024513	17.07469
														GWIPGNEENK	1	6	1	N-Term(iTRAQ8plex); K10(iTRAQ8plex)	0.864	0.622	0.814	3.46	-	0	1751.94797	3	584.6541748	-3.265014733	23.18694667
														IPANQLAELWLK	2	7	1	N-Term(iTRAQ8plex); K12(iTRAQ8plex)	0.967	0.527	0.73	3.4	38	0	2004.2072	2	1002.607239	-1.521988812	41.48829833
														FVFPFDYLPAEQLCIVAK	1	6	1	N-Term(iTRAQ8plex); C14(Methylthio); K18(iTRAQ8plex)	1.12	0.573	0.547	3.35	-	0	2754.48795	3	918.8341675	0.714067893	68.52641
														SLSQIHEAAVR	1	7	1	N-Term(iTRAQ8plex)	0.605	0.254	0.46	3.32	-	0	1514.8555	3	505.6233521	-2.487628456	16.91760667
														EHFWSIDQTEFR	2	6	1	N-Term(iTRAQ8plex)	1.078	0.862	1.277	3.29	37	0	1898.92783	3	633.6474609	-3.197455641	32.90775667
														LDMIPDLK	2	6	1	N-Term(iTRAQ8plex); K8(iTRAQ8plex)	0.846	0.447	0.61	3.27	25	0	1552.92302	2	776.9651489	0.070790091	65.31997167
														FKGFPQPILSEDGSR	5	7	1	N-Term(iTRAQ8plex); K2(iTRAQ8plex)	2.477	1.164	1.317	3.25	32	1	2286.26694	3	762.760498	-1.469823718	30.71814333
														VYIYHLR	2	7	1	N-Term(iTRAQ8plex)	1.138	0.816	0.828	3.16	48	0	1267.75284	3	423.2557983	5.056560964	24.78106
														ANVTVLDTQIR	2	7	1	N-Term(iTRAQ8plex)	1.011	0.527	0.711	3.15	40	0	1533.8853	2	767.4462891	-3.220446141	27.929855
														SLDGEGNFNWR	3	6	1	N-Term(iTRAQ8plex)	1.006	0.463	0.657	3.11	28	0	1598.78325	2	799.8952637	-2.039411064	36.05614333
														GWWPCYAEK	4	6	1	N-Term(iTRAQ8plex); C5(Methylthio); K9(iTRAQ8plex)	0.935	0.54	0.651	3.09	35	0	1793.88249	2	897.4448853	-7.730947949	65.48061667
														LALHILR	11	7	1	N-Term(iTRAQ8plex)	1.201	0.419	0.712	3.08	48	0	1139.75873	3	380.591095	1.842812914	31.38321
														VSMFVLGTGDEPPPERR	1	7	1	N-Term(iTRAQ8plex)	1.356	0.651	0.775	2.88	-	1	2191.14621	3	731.0535889	-0.989412949	31.51124
														KEHFWSIDQTEFR	1	6	1	N-Term(iTRAQ8plex); K1(iTRAQ8plex)	0.789	0.521	0.672	2.86	-	1	2331.23124	3	777.7485962	-1.285147409	29.21230333
														IPAHQVLYSTSGENASGK	2	7	1	N-Term(iTRAQ8plex); K18(iTRAQ8plex)	0.774	-	0.554	2.76	24	0	2467.33144	4	617.5883179	-3.530006337	20.69057
														GFPQPILSEDGSR	2	7	1	N-Term(iTRAQ8plex)	1.135	0.88	0.791	2.76	26	0	1706.89873	2	853.9530029	-1.653326338	29.70597167
														ELPDSVPQECTVR	2	7	1	N-Term(iTRAQ8plex); C10(Methylthio)	1.017	1.022	1.181	2.73	28	0	1822.89324	2	911.9502563	-2.68047771	33.87716
19743813	ITGB1	integrin beta-1 isoform 1A precursor	NP_002202.2	29.70	17	17	81	798	88.4	5.39	0.340	0.281	0.865																
														FQGQTCEMCQTCLGVCAEHK	9	2	1	N-Term(iTRAQ8plex); C6(Methylthio); C9(Methylthio); C12(Methylthio); [illegible]	-	0.405	0.786	5.47	52	0	3008.2811	3	1003.431885	4.403094801	66.04252833
														DKLPQPVQPDPVSHCK	4	2	1	N-Term(iTRAQ8plex); K2(iTRAQ8plex); C15(Methylthio); K16(iTRAQ8plex)	0.31	0.102	0.822	5.33	25	1	2746.51334	3	916.1759644	-0.517923424	23.74015333
														HCECSTDEVNSEDMDAYCR	4	2	1	N-Term(iTRAQ8plex); C2(Methylthio); C4(Methylthio); C18(Methylthio)	0.926	0.504	0.944	5.04	57	0	2648.94449	3	883.6530151	-0.812451846	35.31507
														LLVFSTDAGFHFAGDGK	12	2	1	N-Term(iTRAQ8plex); K17(iTRAQ8plex)	0.304	0.444	1.065	4.95	32	0	2390.30068	4	598.3270264	-4.286395046	45.620065
														LSENNIQTIFAVTEEFQPVYK	4	2	1	N-Term(iTRAQ8plex); K21(iTRAQ8plex)	0.164	0.11	0.789	4.91	57	0	3078.6729	2	1539.840088	3.916577579	57.84303833
														LSEGVTISYK	4	2	1	N-Term(iTRAQ8plex); K10(iTRAQ8plex)	0.314	0.165	0.92	4.84	33	0	1704.98381	2	852.9955444	-9.037653438	35.92116167
														AEDYPIDLYYLMDLSYSMKDDLENVK	4	2	1	N-Term(iTRAQ8plex); K19(iTRAQ8plex); K26(iTRAQ8plex)	-	-	-	4.83	27	1	4056.09267	4	1014.778625	7.132670642	68.60943833
														TVMPYISTTPAK	4	2	1	N-Term(iTRAQ8plex); K12(iTRAQ8plex)	0.517	0.318	1.056	4.74	46	0	1917.08489	2	959.0460815	-6.616730512	22.285105
														SLGTDLMNEMR	4	2	1	N-Term(iTRAQ8plex)	0.285	0.212	0.864	4.72	41	0	1570.79363	2	785.9004517	4.141473585	34.72018833
														LKPEDITQIQPQQLVLR	6	2	1	N-Term(iTRAQ8plex); K2(iTRAQ8plex)	0.482	0.599	0.932	4.46	53	0	2627.56437	3	876.5263062	-2.339083697	39.21351
														NVLSLTNK	2	2	1	N-Term(iTRAQ8plex); K8(iTRAQ8plex)	0.611	0.497	0.816	3.69	30	0	1496.92778	2	748.9675293	1.416684075	20.71592333
														GEVFNELVGK	3	2	1	N-Term(iTRAQ8plex); K10(iTRAQ8plex)	0.377	0.164	0.528	3.59	30	0	1699.97515	2	850.4912109	-5.168337328	53.93809
														GICECGVCK	7	2	1	N-Term(iTRAQ8plex); C3(Methylthio); C5(Methylthio); C8(Methylthio); [illegible]	0.349	0.223	0.889	3.53	45	0	1657.74309	2	829.3751831	-5.552554627	63.13637333
														FCECDNFNCDR	10	2	1	N-Term(iTRAQ8plex); C2(Methylthio); C4(Methylthio); C9(Methylthio)	0.39	0.307	0.831	3.45	27	0	1807.62847	2	904.3178711	-3.202390652	39.42974167
														GCPPDDIENPR	2	2	1	N-Term(iTRAQ8plex); C2(Methylthio)	0.349	0.269	0.879	3.11	48	0	1562.7166	2	781.8619385	-5.062956952	15.92896167
														SGEPQTFTLK	1	2	1	N-Term(iTRAQ8plex); K10(iTRAQ8plex)	0.34	0.212	0.664	-	37	0	1715.9699	2	858.4885864	-5.207007685	18.260795
														IGFGSFVEK	1	2	1	N-Term(iTRAQ8plex); K9(iTRAQ8plex)	0.295	0.216	0.911	-	30	0	1591.91448	2	796.4608765	-10.02251726	46.78967333
19743875	FH	fumarate hydratase, mitochondrial	NP_000134.2	36.86	11	11	37	510	54.6	8.76	0.613	0.460	0.385																
														LNDHFPLVVWQTGSGTQTNMNVNEVISNR	4	2	1	N-Term(iTRAQ8plex)	-	-	-	7.02	80	0	3574.8021	3	1192.272217	0.15828586	47.75383333
														SGLGELILPENEPGSSIMPGK	6	2	1	N-Term(iTRAQ8plex); K21(iTRAQ8plex)	0.612	0.504	0.422	5.87	65	0	2733.49761	2	1367.252441	0.810277827	40.74557333
														LMNESLMLVTALNPHIGYDK	8	2	1	N-Term(iTRAQ8plex); K20(iTRAQ8plex)	0.372	0.355	0.217	5.54	42	0	2867.57279	3	956.5254517	-0.07308283	60.867255
														THTQDAVPLTLGQEFSGYVQQVK	2	2	1	N-Term(iTRAQ8plex); K23(iTRAQ8plex)	-	-	-	5.48	43	0	3154.70742	4	789.432312	2.557088758	41.86667667
														AIEMLGGELGSK	5	2	1	N-Term(iTRAQ8plex); K12(iTRAQ8plex)	0.726	0.473	0.42	5.11	62	0	1813.02774	3	605.0140991	-4.002799594	63.44848333
														IEYDTFGELK	4	1	1	N-Term(iTRAQ8plex); K10(iTRAQ8plex)	0.595	0.303	0.219	4.75	58	0	1822.99932	2	912.0032959	-2.960024768	51.99896167
														AAAEVNQDYGLDPK	2	2	1	N-Term(iTRAQ8plex); K14(iTRAQ8plex)	0.475	0.384	0.339	4.43	59	0	2099.11777	3	700.3774414	-2.467861176	32.56479167
														IYELAAGGTAVGTGLNTR	2	2	1	N-Term(iTRAQ8plex)	0.616	0.573	0.342	3.38	57	0	2068.13188	2	1034.56958	-1.062680657	43.16303833
														ETAIELGYLTAEQFDEWVKPK	1	2	1	N-Term(iTRAQ8plex); K19(iTRAQ8plex); K21(iTRAQ8plex)	0.815	0.732	0.335	3.31	-	0	3379.84055	3	1127.285034	-4.37062362	61.89895667
														IPVHPNDHVNK	1	2	1	N-Term(iTRAQ8plex); K11(iTRAQ8plex)	0.704	0.395	0.438	3.21	-	0	1878.082	3	626.6988525	0.741749926	13.52511667
														SGLGELILPENEPGSSIMPGK	1	2	1	N-Term(iTRAQ8plex); M18(Oxidation); K21(iTRAQ8plex)	0.538	1.218	0.742	3.04	-	0	2749.48661	2	1367.252441	0.810277827	40.74557333
														SKEFAQIIK	1	2	1	N-Term(iTRAQ8plex); K2(iTRAQ8plex); K9(iTRAQ8plex)	1.415	1.218	0.732	2.8	-	1	1976.23662	2	988.6219482	2.969565646	22.73175333
19913369	TBL3	transducin beta-like protein 3	NP_006444.2	8.91	5	5	13	808	89.0	6.90	2.253	0.893	0.588																
														ALLLAQWAWQEGSVTR	6	1	1	N-Term(iTRAQ8plex)	3.117	1.529	0.606	5.71	71	0	2133.19048	2	1067.098877	6.837638929	59.52092833
														VNILEVASGAVLR	2	1	1	N-Term(iTRAQ8plex)	0.612	-	0.426	4.4	64	0	1644.99186	3	549.0021362	-1.948859858	53.53975667
														EAPEELLAYEGVR	2	1	1	N-Term(iTRAQ8plex)	2.035	0.8	0.588	3.72	44	0	1779.9384	2	890.4728394	-2.628860109	37.26334167
														GSPGVVHLVAFHPDPTR	1	1	1	N-Term(iTRAQ8plex)	2.494	2.81	0.729	2.91	-	0	2090.13906	4	523.2902222	-2.80484119	25.40832333
														LDDHALTGASDSR	2	1	1	N-Term(iTRAQ8plex)	0.552	0.703	0.545	2.77	20	0	1661.83408	3	554.6162109	-3.36479891	15.596845
19913410	MVP	major vault protein isoform 1	NP_059447.2	9.97	6	6	21	893	99.3	5.48	0.312	0.413	1.002																
														DITPLQVVLPNTALHLK	2	3	1	N-Term(iTRAQ8plex); K17(iTRAQ8plex)	0.312	0.373	1.718	5.11	44	0	2480.49625	3	827.5036011	-3.975546161	45.14712667
														VPHNAAVQVYDYR	4	3	1	N-Term(iTRAQ8plex)	0.297	0.41	0.57	4.61	37	0	1835.96262	3	612.6590576	-4.347418816	22.53391333
														LFSVPDFVGDACK	9	3	1	N-Term(iTRAQ8plex); C12(Methylthio); K13(iTRAQ8plex)	0.138	0.374	1.002	4.04	48	0	2052.07834	3	684.6976318	1.393688415	54.17824833
														AQLELEVSK	4	2	1	N-Term(iTRAQ8plex); K9(iTRAQ8plex)	0.293	0.873	1.857	3.5	52	0	1624.95683	2	812.9820557	-9.961244219	33.55205333
														VVFGPELVSLGPEEQFTVLSLSAGRPK	1	2	1	N-Term(iTRAQ8plex); K27(iTRAQ8plex)	-	-	-	3.22	-	0	3464.96694	4	866.9971924	1.586840898	51.707085
														AQQLAEVEVK	1	2	1	N-Term(iTRAQ8plex); K10(iTRAQ8plex)	0.859	0.866	0.83	-	24	0	1723.00833	3	575.0076294	-7.374524051	32.61332833
19913416	AP2A1	AP-2 complex subunit alpha-1 isoform 2	NP_570603.2	16.13	6	11	25	955	105.3	7.66	1.032	0.684	1.155																
														LVECLETVLNK	2	4	1	N-Term(iTRAQ8plex); C4(Methylthio); K11(iTRAQ8plex)	1.293	-	1.274	3.92	53	0	1915.09439	3	639.0363159	4.747653135	64.84036667
														IVSSASTDLQDYTYYFVPAPWLSVK	2	4	1	N-Term(iTRAQ8plex); K25(iTRAQ8plex)	-	-	-	3.26	28	0	3458.84031	3	1153.618286	1.695322108	66.182225
														DFLTPPLLSVR	1	4	1	N-Term(iTRAQ8plex)	0.935	0.684	1.047	3.23	-	0	1561.91997	2	781.463623	-3.586500179	47.38712
														ANHPMDAEVTK	1	4	1	N-Term(iTRAQ8plex); M5(Oxidation); K11(iTRAQ8plex)	0.499	0.761	1.372	2.78	-	0	1836.96109	3	612.9918823	-6.724463238	12.00586
														LLGFGSALLDNVDPNPENFVGAGIIQTK	1	4	1	N-Term(iTRAQ8plex); K28(iTRAQ8plex)	-	-	-	-	39	0	3507.94626	3	1169.986938	4.387433141	67.12644333
														HLCELLAQQF	1	4	1	N-Term(iTRAQ8plex); C3(Methylthio)	1.138	0.564	0.578	-	21	0	1551.79265	2	776.3999634	-2.501727397	76.16804
														VGGYILGEFGNLIAGDPR	3	8	2	N-Term(iTRAQ8plex)	-	-	-	4.78	92	0	2152.16471	3	718.0597534	-2.683017742	65.11045167
														ACNQLGQFLQHR	2	8	2	N-Term(iTRAQ8plex); C2(Methylthio)	-	-	-	4.26	44	0	1764.8851	3	588.9665527	-5.018653537	38.94335333
														NADVELQQR	4	8	2	N-Term(iTRAQ8plex)	-	-	-	3.57	60	0	1376.74033	3	459.5849609	-2.35464701	18.121665
														GLAVFISDIR	6	5	2	N-Term(iTRAQ8plex)	-	-	-	3.55	51	0	1394.83354	2	697.9204102	1.865736963	45.26153833
														QSAALCLLR	2	8	2	N-Term(iTRAQ8plex); C6(Methylthio)	-	-	-	3.05	23	0	1324.72991	2	662.8685913	-6.330575595	37.09134
19913424	ATP6V1A	V-type proton ATPase catalytic subunit A	NP_001681.2	9.56	5	5	10	617	68.3	5.52	1.076	1.533	1.407																
														EILQEEEDLAEIVQLVGK	2	1	1	N-Term(iTRAQ8plex); K18(iTRAQ8plex)	1.089	0.636	1.108	5.7	60	0	2663.49442	3	888.5029907	-0.751787583	59.24144333
														IKSDYAQLLEDMQNAFR	2	1	1	N-Term(iTRAQ8plex); K2(iTRAQ8plex)	1.458	1.912	1.491	5.51	48	1	2650.40897	3	884.1411743	-1.12873176	61.987365
														SDYAQLLEDMQNAFR	2	1	1	N-Term(iTRAQ8plex)	1.076	1.64	1.54	5.25	60	0	2105.04399	2	1053.025635	7.805827957	56.883035
														VGHSELVGEIIR	3	1	1	N-Term(iTRAQ8plex)	0.946	1.027	0.873	2.86	37	0	1612.92607	3	538.3141479	-2.827592213	25.79544667
														LPANHPLLTGQR	1	1	1	N-Term(iTRAQ8plex)	0.924	1.533	1.407	2.83	-	0	1620.94388	3	540.986145	-3.010277815	18.25726167
19913428	ATP6V1B2	V-type proton ATPase subunit B, brain isoform	NP_001684.2	6.85	2	2	11	511	56.5	5.81	0.614	0.614	0.772																
														IPIFSAAGLPHNEIAAQICR	7	3	1	N-Term(iTRAQ8plex); C19(Methylthio)	0.548	0.614	0.772	5.32	43	0	2471.3223	3	824.4456177	0.71114858	53.26851333
														LALTTAEFLAYQCEK	4	3	1	N-Term(iTRAQ8plex); C13(Methylthio); K15(iTRAQ8plex)	0.67	0.572	0.762	4.42	34	0	2355.25522	3	785.7548828	-2.036774146	54.23937
19920317	CKAP4	cytoskeleton-associated protein 4	NP_006816.2	31.40	18	18	67	602	66.0	5.92	0.983	0.490	0.548																
														SVGELPSTVESLQK	4	1	1	N-Term(iTRAQ8plex); K14(iTRAQ8plex)	0.966	0.321	0.445	5.64	78	0	2082.18974	2	1041.598511	-0.267235792	31.22826667
														TAVDSLVAYSVK	3	1	1	N-Term(iTRAQ8plex); K12(iTRAQ8plex)	0.937	0.586	0.606	5.57	56	0	1861.0844	2	931.0458374	-2.532456517	26.73017
														HSEAFEALQQK	4	1	1	N-Term(iTRAQ8plex); K11(iTRAQ8plex)	1.019	0.699	0.767	5.26	41	0	1896.02849	2	948.5178833	-7.948888172	16.49135833
														VQSLQATFGTFESILR	6	1	1	N-Term(iTRAQ8plex)	1.112	0.167	0.52	5.06	78	0	2101.1624	2	1051.084839	1.351544447	65.888675
														DFTSLENTVEER	5	1	1	N-Term(iTRAQ8plex)	0.804	0.419	0.566	4.74	38	0	1743.86894	2	872.4381104	-0.77827917	33.94087
														VQEQVHTLLSQDQAQAAR	8	1	1	N-Term(iTRAQ8plex)	1.013	0.498	0.456	4.46	52	0	2326.23575	3	776.0846558	-0.996142026	30.73545667
														LALQALTEK	4	1	1	N-Term(iTRAQ8plex); K9(iTRAQ8plex)	0.976	0.408	0.543	4.25	38	0	1594.99912	3	532.3378906	0.173871569	46.09394667
														SINDNIAIFTEVQKR	2	1	1	N-Term(iTRAQ8plex); K14(iTRAQ8plex)	1.452	0.52	0.563	4.12	-	1	2356.33316	3	786.1159058	-4.819889232	34.860365
														SLGETQLVLYGDVEELKR	4	1	1	N-Term(iTRAQ8plex); K17(iTRAQ8plex)	0.786	0.117	0.308	4.11	28	1	2657.49717	3	886.5039063	0.035446886	42.89894333
														SINDNIAIFTEVQK	2	1	1	N-Term(iTRAQ8plex); K14(iTRAQ8plex)	0.678	0.196	0.385	3.87	38	0	2200.24674	3	734.0870972	1.516627135	37.82448333
														ERDFTSLENTVEER	8	1	1	N-Term(iTRAQ8plex)	1.035	0.638	0.706	3.76	48	1	2029.00614	3	677.006897	-3.885464851	26.941155
														QTESLESLLSK	3	1	1	N-Term(iTRAQ8plex); K11(iTRAQ8plex)	1.01	0.374	0.405	3.51	30	0	1843.06034	3	615.0249634	-1.602993251	54.10792
														DLSDGIHVVK	5	1	1	N-Term(iTRAQ8plex); K10(iTRAQ8plex)	0.977	0.247	0.313	3.43	48	0	1690.98931	2	845.998291	-3.261930003	20.90308833
														ASVSQVEADLK	2	1	1	N-Term(iTRAQ8plex); K11(iTRAQ8plex)	0.753	0.264	0.459	3.18	60	0	1755.00615	2	878.0067139	-2.689555032	22.784195
														LQNEILK	2	1	1	N-Term(iTRAQ8plex); K7(iTRAQ8plex)	0.97	0.248	0.414	2.98	38	0	1465.92436	2	733.4658203	3.078402345	20.60839667
														LSSLDNLK	2	1	1	N-Term(iTRAQ8plex); K8(iTRAQ8plex)	1.038	0.305	0.503	2.79	30	0	1497.91037	3	499.9749756	0.462115641	40.63318333
														RLEEELR	2	1	1	N-Term(iTRAQ8plex)	0.744	0.606	0.675	-	29	1	1248.71711	3	416.910553	-3.424005672	15.33088167
														EWDMEALR	1	1	1	N-Term(iTRAQ8plex)	0.555	0.271	0.415	-	22	0	1353.674	2	677.3406372	-2.57337139	23.620965
														IEEEIR	1	22	3	N-Term(iTRAQ8plex)	-	-	-	-	28	0	1092.61565	2	546.8114624	-4.222084244	20.37413667
19923142	KPNB1	importin subunit beta-1 isoform 1	NP_002256.2	23.17	14	14	48	876	97.1	4.78	0.846	1.366	1.696																
														ESTLEAIGYICQDIDPEQLQDK	2	2	1	N-Term(iTRAQ8plex); C11(Methylthio); K22(iTRAQ8plex)	0.475	1.632	2.271	6.97	82	0	3162.58847	3	1054.867676	2.814717698	63.91815
														LAATNALLNSLEFTK	8	2	1	N-Term(iTRAQ8plex); K15(iTRAQ8plex)	0.846	1.402	1.707	5.67	69	0	2214.28603	2	1107.64917	-1.97719095	46.46202667
														HFIMQVVCEATQCPDTR	15	2	1	N-Term(iTRAQ8plex); C8(Methylthio); C13(Methylthio)	0.645	0.654	1.046	5.52	64	0	2374.07993	3	792.0316772	0.688766997	59.32958667
														LQQVLQMESHIQSTSDR	2	2	1	N-Term(iTRAQ8plex)	1.693	1.783	1.486	5.41	36	0	2304.19278	3	768.7357788	0.339327415	39.82769167
														KVQHQDALQISDVVMASLLR	2	2	1	N-Term(iTRAQ8plex); K1(iTRAQ8plex)	1.071	1.361	1.492	4.53	-	1	2859.63125	4	715.663269	-0.80255387	49.62694667
														SNEILTAIIQGMR	2	2	1	N-Term(iTRAQ8plex)	0.711	1.461	1.847	4.5	44	0	1749.98516	2	875.4962158	0.939276896	53.88622833
														GALQYLVPILTQTLTK	6	2	1	N-Term(iTRAQ8plex); K16(iTRAQ8plex)	0.799	1.648	2.213	4.47	42	0	2367.44492	3	789.8198242	-0.955415575	64.9887
														SSAYESLMEIVK	1	2	1	N-Term(iTRAQ8plex); K12(iTRAQ8plex)	1.002	1.787	1.684	4.06	-	0	1965.07285	3	655.6958008	-4.820282492	45.29949667
														TTLVIMER	3	2	1	N-Term(iTRAQ8plex)	0.642	1.313	1.504	3.41	34	0	1266.74272	2	633.875	2.669902844	28.93341

														AAVENLPTFLVELSR	2	1	1	N-Term(iTRAQ8plex)	-	1.005	-	3.37	31	0	1963.11014	3	655.0415649	-3.310932812	61.26896
														VLANPGNSQVAR	2	1	1	N-Term(iTRAQ8plex)	0.92	1.356	2.126	3.31	35	0	1529.8654	2	765.4363403	-3.122664206	18.73202667
														SDYDMVDYLNELR	1	2	1	N-Term(iTRAQ8plex)	0.926	1.219	1.315	3.24	-	0	1936.92803	2	968.9676514	0.82337307	44.54702167
														TVSPDRLELEAAQK	1	1	1	N-Term(iTRAQ8plex); K14(iTRAQ8plex)	-	1.88	3.092	3.19	-	1	2165.22475	4	542.0616455	-6.423882203	24.82488
														WLAIDANAR	1	1	1	N-Term(iTRAQ8plex)	0.946	2.161	2.505	-	24	0	1333.74516	2	667.3762207	-5.890565872	40.568035
19923231	RAB6A	ras-related protein Rab-6A isoform a	NP_002860.2	25.96	5	5	10	208	23.5	5.54	1.037	0.432	0.593																
														LVFLGEQSVGK	2	4	1	N-Term(iTRAQ8plex); K11(iTRAQ8plex)	0.544	0.213	0.364	4.38	37	0	1785.06719	2	893.0372314	-3.302413164	58.52704167
														GSDVIIMLVGNK	2	5	1	N-Term(iTRAQ8plex); K12(iTRAQ8plex)	1.013	0.468	0.815	3.57	35	0	1854.08182	3	618.6987915	-8.688957573	68.24553667
														QVSIEEGER	4	4	1	N-Term(iTRAQ8plex)	1.012	0.359	0.491	3.55	41	0	1350.71939	3	450.9113159	2.001306798	18.83208
														VAAALPGMESTQDR	1	4	1	N-Term(iTRAQ8plex); M8(Oxidation)	1.09	0.399	0.671	-	30	0	1765.90117	2	883.4542236	-2.545813123	21.82344167
														SLIPSYIR	1	5	1	N-Term(iTRAQ8plex)	1.194	0.65	0.757	-	23	0	1252.75505	2	626.8811646	-1.300189079	33.05584
														IQIWDTAGQER	14	3	9	N-Term(iTRAQ8plex)	-	-	-	3.41	41	0	1620.85991	2	810.9328003	-3.971558752	26.96297167
19923483	RAB14	ras-related protein Rab-14	NP_057406.2	28.37	5	5	18	215	23.9	6.21	1.309	1.070	0.584																
														TGENVEDAFLEAAK	4	1	1	N-Term(iTRAQ8plex); K14(iTRAQ8plex)	0.998	0.785	0.425	4.96	44	0	2102.11356	3	701.3760376	-4.315540895	57.20436
														FMADCPHTIGVEFGTR	6	1	1	N-Term(iTRAQ8plex); C5(Methylthio)	1.388	1.143	0.665	4.79	55	0	2131.01128	2	1066.009277	1.673274814	45.63210167
														GAAGALMVYDITR	2	1	1	N-Term(iTRAQ8plex)	1.458	2.303	0.583	4.08	55	0	1641.89641	2	821.4518433	1.692313486	36.76047333
														IIEVSGQK	2	1	1	N-Term(iTRAQ8plex); K8(iTRAQ8plex)	1.42	0.937	0.586	4.05	28	0	1481.91428	3	494.6429443	-0.331694076	30.02571167
														SCLLHQFTEK	4	1	1	N-Term(iTRAQ8plex); C2(Methylthio); K10(iTRAQ8plex)	1.118	1.001	0.483	3.83	51	0	1859.99211	2	930.4996948	-2.521124434	33.40153333
														IQIWDTAGQER	14	3	9	N-Term(iTRAQ8plex)	-	-	-	3.41	41	0	1620.85991	2	810.9328003	-3.971558752	26.96297167
19923615	MYO19	unconventional myosin-XIX isoform 1	NP_079385.2	1.17	1	1	1	770	86.6	8.05	0.861	0.958	0.764																
														VACQASSER	1	8	1	N-Term(iTRAQ8plex); C3(Methylthio)	0.861	0.958	0.764	-	18	0	1300.63188	2	650.8195801	2.115307402	19.43636167
19923717	DTX3L	E3 ubiquitin-protein ligase DTX3L	NP_612144.1	1.89	1	1	2	740	83.5	8.06	0.744	0.936	0.762																
														GNQPEGSMVFTVSR	2	1	1	N-Term(iTRAQ8plex)	0.744	0.936	0.762	3.79	41	0	1812.92351	2	906.9653931	1.030497317	28.09415333
19923748	DLST	dihydrolipoyllysine-residue succinyltransferase component of 2-oxoglutarate dehydrogenase complex, mitochondrial isoform 1 precursor	NP_001924.2	19.65	8	8	23	453	48.7	8.95	0.928	0.797	0.722																
														NVEAMNFADIER	4	1	1	N-Term(iTRAQ8plex); M5(Oxidation)	1.125	1.09	0.973	6.1	52	0	1728.85453	3	576.9563599	0.942843842	27.972115
														ASAFALQEQPVVNAVIDDTTK	4	1	1	N-Term(iTRAQ8plex); K21(iTRAQ8plex)	0.833	0.837	0.838	5.8	84	0	2825.5706	2	1413.28894	7.086334826	45.48615167
														TPAFAESVTEGDVR	5	2	1	N-Term(iTRAQ8plex)	0.855	0.706	0.694	4.76	84	0	1782.91265	2	891.9599609	-2.779746396	28.87492667
														LGFMSAFVK	4	1	1	N-Term(iTRAQ8plex); K9(iTRAQ8plex)	1.148	0.757	0.699	3.86	38	0	1607.93181	2	804.4695435	-7.569354533	71.10320833
														VEGGTPLFTLR	2	2	1	N-Term(iTRAQ8plex)	0.819	0.685	0.644	2.88	23	0	1493.86162	2	747.4344482	-0.904659178	36.920285
														AAVEDPR	1	1	1	N-Term(iTRAQ8plex)	0.947	0.859	0.732	-	51	0	1061.58599	2	531.2966309	-3.12381785	10.85752167
														GLVVPVIR	2	1	1	N-Term(iTRAQ8plex)	0.783	0.72	0.643	-	27	0	1156.77216	3	386.2622375	0.171249353	31.89075167
														EAVTFLR	1	1	1	N-Term(iTRAQ8plex)	1.035	0.932	0.877	-	23	0	1139.66472	2	570.3359985	-6.941383326	20.11576833
19923821	DPYSL5	dihydropyrimidinase-related protein 5	NP_064519.2	1.24	1	1	3	564	61.4	7.20	1.351	0.564	0.562																
														DIGAIAR	3	1	1	N-Term(iTRAQ8plex)	1.351	0.564	0.562	-	35	0	1019.61101	2	510.3091431	-4.032701833	16.755635
19923855	SYAP1	synapse-associated protein 1	NP_116185.2	4.26	1	1	1	352	39.9	4.53	1.885	1.758	1.294																
														DFGNYLFNFASAATK	1	1	1	N-Term(iTRAQ8plex); K15(iTRAQ8plex)	1.885	1.758	1.294	3.32	-	0	2274.1829	3	758.7324829	-8.194512222	44.17271333
19923903	MYADM	myeloid-associated differentiation marker	NP_612382.1	6.52	2	2	2	322	35.3	8.15	0.406	0.644	0.790																
														ALTQPLGLLR	1	1	1	N-Term(iTRAQ8plex)	0.28	0.6	1.1	3.25	-	0	1385.8792	2	693.4432373	0.704743513	36.86409667
														SHAYYVCAWDR	2	1	1	N-Term(iTRAQ8plex); C7(Methylthio)	0.588	0.69	0.567	3.05	17	0	1720.77958	3	574.2647095	-4.74618141	29.81313
19923919	REEP6	receptor expression-enhancing protein 6	NP_612402.1	7.07	1	1	4	184	20.7	8.56	1.671	0.901	2.211																
														NLVTEVLGALEAK	4	1	1	N-Term(iTRAQ8plex); K13(iTRAQ8plex)	1.671	0.901	2.211	5	56	0	1965.19242	3	655.7356567	4.231476151	57.27890167
19923969	CCDC124	coiled-coil domain-containing protein 124	NP_612451.1	14.35	2	2	8	223	25.8	9.54	0.812	1.541	1.401																
														TIEDAIAVLSVAEEAADRHPER	6	1	1	N-Term(iTRAQ8plex)	-	-	-	5.18	42	1	2696.41982	4	674.8582153	-1.761545929	56.654695
														VLEEGSVEAR	2	1	1	N-Term(iTRAQ8plex)	0.812	1.541	1.401	3.66	60	0	1392.76508	3	464.9265442	1.02531172	24.78381833
19924129	RAD50	DNA repair protein RAD50	NP_005723.2	1.37	1	1	2	1312	153.8	6.89	-	-	-																
														VFQTEAELQEVISDLQSK	2	1	1	N-Term(iTRAQ8plex); K18(iTRAQ8plex)	-	-	-	4.76	63	0	2672.45615	3	891.4902344	-1.572121211	59.42402
20070125	P4HB	protein disulfide-isomerase precursor	NP_000909.2	51.38	26	26	185	508	57.1	4.87	0.744	0.918	0.802																
														QFLQAAEAIDDIPFGITSNSDVFSK	18	1	1	N-Term(iTRAQ8plex); K25(iTRAQ8plex)	1.051	0.775	0.776	6.64	80	0	3321.7529	3	1107.926636	5.70560366	61.61093167
														VDATEESDLAQQYGVR	10	1	1	N-Term(iTRAQ8plex)	0.761	0.767	0.816	6.15	94	0	2085.04009	2	1043.023682	-0.069369769	28.25554167
														ILFIFIDSDHTDNQR	13	1	1	N-Term(iTRAQ8plex)	0.861	1.158	1.021	5.8	58	0	2138.11942	3	713.37677	-1.246186694	54.95064167
														HNQLPLVIEFTEQTAPK	12	1	1	N-Term(iTRAQ8plex); K17(iTRAQ8plex)	0.753	0.946	0.884	5.75	57	0	2573.45176	2	1287.232788	1.359358291	38.11241333
														LITLEEEMTK	8	1	1	N-Term(iTRAQ8plex); K10(iTRAQ8plex)	0.892	1.183	0.863	4.82	45	0	1815.02214	3	605.682373	-3.765998199	69.73887167
														IFGGEIKTHILLFLPK	3	1	1	N-Term(iTRAQ8plex); K7(iTRAQ8plex); K16(iTRAQ8plex)	0.986	1.359	0.66	4.81	18	1	2738.69785	3	913.5708008	-4.444523244	54.98856
														THILLFLPK	14	1	1	N-Term(iTRAQ8plex); K9(iTRAQ8plex)	0.445	0.702	0.476	4.77	42	0	1690.07988	2	845.5430908	-5.139570318	46.33505667
														NNFEGEVTKENLLDFIK	3	1	1	N-Term(iTRAQ8plex); K9(iTRAQ8plex); K17(iTRAQ8plex)	1.018	1.454	0.421	4.66	27	1	2922.64326	5	585.3344727	3.159972135	47.976695
														EADDIVNWLK	6	1	1	N-Term(iTRAQ8plex); K10(iTRAQ8plex)	0.569	0.863	0.649	4.62	49	0	1811.00334	2	906.0049438	-7.37173133	66.63219667
														NFEDVAFDEK	6	1	1	N-Term(iTRAQ8plex); K10(iTRAQ8plex)	0.706	0.866	0.626	4.52	63	0	1821.93926	2	911.4732666	-4.766743372	38.596655
														ILEFFGLKK	2	1	1	N-Term(iTRAQ8plex); K8(iTRAQ8plex); K9(iTRAQ8plex)	0.496	1.073	0.648	4.33	28	1	2007.29287	4	502.5786743	7.914184265	52.26355333
														ILEFFGLK	14	1	1	N-Term(iTRAQ8plex); K8(iTRAQ8plex)	0.765	0.971	0.805	4.09	41	0	1574.96648	2	787.9868774	-6.459208706	81.64575
														MDSTANEVEAVK	4	1	1	N-Term(iTRAQ8plex); K12(iTRAQ8plex)	0.895	1.19	1.157	4.09	59	0	1901.99944	2	951.5033569	-5.501606682	31.91238
														TGPAATTLPDGAAAESLVESSEVAVIGFFK	7	1	1	N-Term(iTRAQ8plex); K30(iTRAQ8plex)	-	-	-	3.77	54	0	3543.9133	3	1181.975952	2.514704664	68.753315
														ENLLDFIK	6	1	1	N-Term(iTRAQ8plex); K8(iTRAQ8plex)	0.61	0.751	0.701	3.68	40	0	1599.95707	3	533.9901733	-0.421593146	40.126875
														NNFEGEVTK	4	1	1	N-Term(iTRAQ8plex); K9(iTRAQ8plex)	0.731	0.781	0.759	3.58	43	0	1645.89763	2	823.4524536	-1.792756355	16.20230333
														SVSDYDGK	6	1	1	N-Term(iTRAQ8plex); K8(iTRAQ8plex)	0.935	0.904	0.865	3.52	36	0	1478.79534	2	739.9013062	0.428987482	13.75176167
														ITEFCHR	33	1	1	N-Term(iTRAQ8plex); C5(Methylthio)	0.765	0.937	0.815	3.51	38	0	1255.61734	3	419.2102661	-5.309910717	25.362755
														TVIDYNGER	2	1	1	N-Term(iTRAQ8plex)	0.774	0.957	0.768	3.32	23	0	1370.7166	2	685.8619385	-3.765159679	18.45674333
														VHSFPTLK	3	1	1	N-Term(iTRAQ8plex); K8(iTRAQ8plex)	0.71	1.006	0.802	3.18	30	0	1536.92063	2	768.9653931	-8.017176594	26.18782333
														LITLEEEMTK	1	1	1	N-Term(iTRAQ8plex); M8(Oxidation); K10(iTRAQ8plex)	1.007	0.671	0.428	3.13	-	0	1831.01603	3	605.682373	-3.765998199	69.73887167
														IFGGEIK	2	1	1	N-Term(iTRAQ8plex); K7(iTRAQ8plex)	0.735	0.829	0.74	2.95	25	0	1371.84757	3	457.9540405	1.411987801	29.86652667
														TAAESFK	1	1	1	N-Term(iTRAQ8plex); K7(iTRAQ8plex)	0.724	0.858	0.772	2.88	-	0	1361.79151	3	454.6020203	2.207563345	17.182905
														MDSTANEVEAVK	1	1	1	N-Term(iTRAQ8plex); M1(Oxidation); K12(iTRAQ8plex)	0.681	0.868	0.74	-	41	0	1917.99455	2	951.5033569	-5.501606682	31.91238
														YKPESEELTAER	1	1	1	N-Term(iTRAQ8plex); K2(iTRAQ8plex)	0.621	0.744	0.631	-	28	0	2060.09941	2	1030.553345	-6.130027899	17.59555667
														DVESDSAK	1	1	1	N-Term(iTRAQ8plex); K8(iTRAQ8plex)	0.686	1.053	0.898	-	27	0	1458.77593	2	729.8916016	-9.394722487	10.91117833
														VDATEESDLAQQYGVR	1	1	1	N-Term(iTRAQ8plex); Y13(iTRAQ8plex)	0.692	1.02	0.885	-	27	0	2389.23745	2	1043.023682	-0.069369769	28.25554167
														FFPASADR	2	1	1	N-Term(iTRAQ8plex)	0.836	0.973	0.808	-	21	0	1214.64165	2	607.8244629	-4.529845287	18.69694
														EECPAVR	1	1	1	N-Term(iTRAQ8plex); C3(Methylthio)	0.805	1.044	0.918	-	21	0	1153.55864	2	577.282959	-5.288808936	18.23091333
20070130	BTF3	transcription factor BTF3 isoform B	NP_001198.2	11.73	1	1	1	162	17.7	7.50	1.392	1.277	2.509																
														VQASLAANTFTITGHAETK	1	2	1	N-Term(iTRAQ8plex); K19(iTRAQ8plex)	1.392	1.277	2.509	3.06	-	0	2568.40883	4	642.857666	-5.994807873	31.43418667
20070197	DDOST	dolichyl-diphosphooligosaccharide--protein glycosyltransferase 48 kDa subunit precursor	NP_005207.2	21.27	9	9	56	456	50.7	6.40	0.912	0.536	0.642																
														WVPFDGDDIQLEFVR	12	1	1	N-Term(iTRAQ8plex)	0.939	0.49	0.659	5.25	82	0	2140.09421	3	714.0362549	-3.513818	56.00186167
														YSQTGNYELAVALSR	2	1	1	N-Term(iTRAQ8plex)	0.885	0.643	0.779	4.62	63	0	1976.03301	2	988.5201416	-3.07419808	33.79811833
														TLVLLDNLNVR	10	1	1	N-Term(iTRAQ8plex)	0.868	0.492	0.533	4.12	79	0	1573.95499	3	525.3231812	-1.868600428	48.07549667
														LPDVYGVFQFK	8	1	1	N-Term(iTRAQ8plex); K11(iTRAQ8plex)	0.975	0.85	0.796	3.94	28	0	1921.09183	3	641.0383301	-2.047948951	50.61154
														ETHSLFFR	5	1	1	N-Term(iTRAQ8plex)	0.881	0.608	0.607	3.68	26	0	1340.71872	3	447.5777588	-5.764208912	25.35631833
														NTLLIAGLQAR	2	1	1	N-Term(iTRAQ8plex)	1.057	0.598	0.76	3.37	35	0	1473.90105	2	737.4541626	-3.021047235	26.13190333
														SSLNPILFR	6	1	1	N-Term(iTRAQ8plex)	0.903	0.549	0.626	3.22	34	0	1350.80388	2	675.9055786	-0.614962282	29.535455
														GFELTFK	6	1	1	N-Term(iTRAQ8plex); K7(iTRAQ8plex)	0.912	0.389	0.511	3.06	31	0	1449.85735	2	725.4299927	-2.402015333	42.41164
														WVFKEEGVLR	4	1	1	N-Term(iTRAQ8plex); K4(iTRAQ8plex)	0.957	0.538	0.672	2.99	-	1	1871.09292	4	468.5286865	-3.770569697	33.59285667
														YSQTGNYELAVALSR	1	1	1	N-Term(iTRAQ8plex); Y1(iTRAQ8plex)	0.904	0.426	0.693	-	24	0	2280.23929	2	988.5201416	-3.07419808	33.79811833
20070205	LAD1	ladinin-1	NP_005549.2	14.70	4	4	7	517	57.1	9.67	0.465	0.374	0.183																
														GLPCTELFVAPVGVASK	4	1	1	N-Term(iTRAQ8plex); C4(Methylthio); K17(iTRAQ8plex)	0.69	0.378	0.318	4.67	45	0	2342.31474	2	1171.661011	3.181709201	49.68479
														LPSVEEAEVPKPLPPASKDEDEDIQSILR	1	1	1	N-Term(iTRAQ8plex); K11(iTRAQ8plex); K18(iTRAQ8plex)	-	-	-	4.13	-	1	4114.25489	5	823.6567993	-3.376365427	40.51118333
														GPWALEEESLVGREPEER	1	1	1	N-Term(iTRAQ8plex)	0.425	0.128	0.061	3.2	-	1	2387.2128	3	796.4091187	-0.732483073	35.88568833
														TLEDEEEQERER	1	1	1	N-Term(iTRAQ8plex)	0.428	0.398	0.164	-	20	1	1866.89499	3	622.9698486	-1.782988104	13.25975167
20070349	VMP1	vacuole membrane protein 1	NP_112200.2	7.39	1	1	2	406	46.2	6.95	1.322	0.895	0.849																
														LSGAEPDDEEYQEFEEMLEHAESAQDFASR	2	1	1	N-Term(iTRAQ8plex)	1.322	0.895	0.849	6.79	69	0	3763.66184	3	1255.225464	3.23808357	52.40891333
20127408	HADHA	trifunctional enzyme subunit alpha, mitochondrial precursor	NP_000173.2	51.38	28	28	164	763	82.9	9.04	0.815	0.467	0.455																
														HLAILGAGLMGAGIAQVSVDK	2	1	1	N-Term(iTRAQ8plex); K21(iTRAQ8plex)	1.869	-	1.114	6.86	81	0	2629.52846	4	658.1375732	-1.364583536	47.46182167
														MGLVDQLVEPLGPGLKPPEER	6	1	1	N-Term(iTRAQ8plex); K16(iTRAQ8plex)	0.942	0.46	0.393	6.65	45	0	2882.63303	3	961.5491943	2.060081097	52.37665167
														DSIFSNLTGQLDYQGFEK	6	1	1	N-Term(iTRAQ8plex); K18(iTRAQ8plex)	0.828	0.358	0.557	6.5	48	0	2670.3779	3	890.7974854	-3.470855306	50.96399
														ELHSEFSEVMNEIWASDQIR	15	1	1	N-Term(iTRAQ8plex)	1.213	1.259	0.621	6.21	60	0	2724.33011	3	908.7815552	2.180711257	59.63124
														ADMVIEAVFEDLSLK	7	1	1	N-Term(iTRAQ8plex); M3(Oxidation); K15(iTRAQ8plex)	0.844	0.313	0.338	6.15	50	0	2304.26493	2	1144.636597	-0.417154588	65.92806167
														FGGGNPELLTQMVSK	4	1	1	N-Term(iTRAQ8plex); K15(iTRAQ8plex)	0.851	0.454	0.461	5.65	83	0	2186.21025	2	1093.608765	0.110408811	32.70766
														TLQEVTQLSQEAQR	3	1	1	N-Term(iTRAQ8plex)	1.527	0.585	0.48	5.56	53	0	1935.05071	2	968.0289917	2.995000812	32.65635333
														EVEAVIPDHCIFASNTSALPISEIAAVSK	9	1	1	N-Term(iTRAQ8plex); C10(Methylthio); K29(iTRAQ8plex)	-	0.622	0.209	5.47	52	0	3665.96445	3	1222.659668	7.214569755	56.93328667
														TGIEQGSDAGYLCESQK	2	1	1	N-Term(iTRAQ8plex); C13(Methylthio); K17(iTRAQ8plex)	0.741	0.529	0.405	5.4	54	0	2440.18833	3	814.067627	-2.522219517	27.606765
														KLDSLTTSFGFPVGAATLVDEVGVDVAK	4	1	1	N-Term(iTRAQ8plex); K1(iTRAQ8plex); K28(iTRAQ8plex)	-	-	-	5.31	28	1	3749.13222	4	938.0385132	4.833533015	61.67431167
														KMGLVDQLVEPLGPGLKPPEER	2	1	1	N-Term(iTRAQ8plex); K1(iTRAQ8plex); K17(iTRAQ8plex)	0.966	0.676	0.464	5.1	32	1	3314.926	3	1105.646851	-0.429207828	41.92873667
														DTSASAVAVGLK	4	1	1	N-Term(iTRAQ8plex); K12(iTRAQ8plex)	0.7	0.376	0.624	5.09	65	0	1727.01262	2	864.0099487	-1.934241257	18.04146833
														ADMVIEAVFEDLSLK	15	1	1	N-Term(iTRAQ8plex); K15(iTRAQ8plex)	0.742	0.249	0.337	5.03	75	0	2288.26592	2	1144.636597	-0.417154588	65.92806167
														MGLVDQLVEPLGPGLKPPEER	2	1	1	N-Term(iTRAQ8plex); M1(Oxidation); K16(iTRAQ8plex)	0.772	0.654	0.584	5.01	30	0	2898.61393	3	961.5491943	2.060081097	52.37665167
														MQLLEIITTEK	16	1	1	N-Term(iTRAQ8plex); K11(iTRAQ8plex)	0.772	0.447	0.44	4.99	51	0	1927.13518	2	964.071228	-2.216692092	76.73904
														TIEYLEEVAITFAK	26	1	1	N-Term(iTRAQ8plex); K14(iTRAQ8plex)	0.949	0.444	0.439	4.87	74	0	2235.27373	2	1118.140503	0.191448309	59.04136333
														DLNSDMDSILASLK	6	1	1	N-Term(iTRAQ8plex); K14(iTRAQ8plex)	0.844	0.511	0.479	4.84	75	0	2130.15215	2	1065.579712	-2.419588611	75.41892
														MVGVPAALDMMLTGR	2	1	1	N-Term(iTRAQ8plex); M1(Oxidation)	0.932	-	0.823	4.8	64	0	1881.98746	3	622.671936	3.147669624	62.45158

														SEVSSDEDIQFR	6	1	1	N-Term(iTRAQ8plex)	0.673	0.385	0.36	4.67	66	0	1715.84305	3	572.6192017	2.360606621	26.04179833
														MVGVPAALDMMLTGR	4	1	1	N-Term(iTRAQ8plex)	0.81	0.674	0.316	4.5	73	0	1866.00125	3	622.671936	3.147669624	62.45158
														LTAYAMTIPFVR	2	1	1	N-Term(iTRAQ8plex)	0.812	0.481	0.503	4.21	41	0	1686.95012	2	843.9786987	-3.183531627	53.519715
														ELHSEFSEVMNEIWASDQIR	2	1	1	N-Term(iTRAQ8plex); M10(Oxidation)	0.485	1.469	1.135	4.04	58	0	2740.32053	3	908.7815552	2.180711257	59.63124
														FVDLYGAQK	2	1	1	N-Term(iTRAQ8plex); K9(iTRAQ8plex)	0.709	0.37	0.455	3.95	38	0	1648.94975	2	824.9785156	-1.289711827	40.58742333
														SAVLISSKPGCFIAGADINMLAACK	1	1	1	N-Term(iTRAQ8plex); K8(iTRAQ8plex); C11(Methylthio); C24(Methylthio); K25(iTRAQ8plex)	0.572	-	-	3.62	-	0	3484.88857	4	871.9776001	7.437027067	60.24478
														HVAEDLGK	2	1	1	N-Term(iTRAQ8plex); K8(iTRAQ8plex)	0.906	0.651	0.55	3.47	30	0	1476.86515	3	492.9598999	1.405109354	17.62178667
														FGGGNPELLTQMVSK	1	1	1	N-Term(iTRAQ8plex); M12(Oxidation); K15(iTRAQ8plex)	0.885	0.615	0.464	3.41	-	0	2202.18821	2	1093.608765	0.110408811	32.70766
														CLAPMMSEVIR	2	1	1	N-Term(iTRAQ8plex); C1(Methylthio)	0.845	0.422	0.495	3.35	31	0	1599.80193	2	800.4046021	-0.865618683	51.133125
														FGELVMTK	2	1	1	N-Term(iTRAQ8plex); K8(iTRAQ8plex)	0.794	0.519	0.412	3.2	33	0	1532.898	2	766.9526367	0.851666258	46.56949833
														ALMGLYHGQVLCK	1	1	1	N-Term(iTRAQ8plex); C12(Methylthio); K13(iTRAQ8plex)	0.79	0.363	0.492	3.06	-	0	2087.1445	3	696.3863525	0.987984868	38.823055
														DATLTALDR	2	1	1	N-Term(iTRAQ8plex)	0.739	0.433	0.55	2.93	27	0	1279.71233	2	640.3598022	-2.839253583	17.53934667
														GFYIYQEGVK	1	1	1	N-Term(iTRAQ8plex); K10(iTRAQ8plex)	1.114	0.48	0.398	2.87	-	0	1812.01114	3	604.6752319	-2.239914051	40.07102667
														VIIVVK	3	1	1	N-Term(iTRAQ8plex); K6(iTRAQ8plex)	0.891	0.428	0.341	2.84	28	0	1278.89787	2	639.9525757	0.737703304	34.65799167
														LPPKSEVSSDEDIQFR	1	1	1	N-Term(iTRAQ8plex); K4(iTRAQ8plex)	0.741	0.762	1.18	2.78	-	1	2455.32021	4	614.5855103	-3.555611335	25.856725
														CLAPMMSEVIR	1	1	1	N-Term(iTRAQ8plex); C1(Methylthio); M5(Oxidation)	0.905	0.521	0.489	-	26	0	1615.79399	2	800.4046021	-0.865618683	51.133125
20127499	SRSF6	serine/arginine-rich splicing factor 6	NP_006266.2	12.50	5	6	24	344	39.6	11.43	0.869	0.955	0.818																
														CSWQDLKDFMR	5	1	1	N-Term(iTRAQ8plex); C1(Methylthio); K7(iTRAQ8plex)	1.542	1.884	0.972	4.18	27	1	2083.03481	4	521.5141602	-1.714592085	48.960395
														CSWQDLK	5	3	1	N-Term(iTRAQ8plex); C1(Methylthio); K7(iTRAQ8plex)	0.838	0.738	0.723	3.39	31	0	1533.80014	3	511.9382324	-0.813850735	33.42331
														VIVEHAR	8	3	1	N-Term(iTRAQ8plex)	0.841	0.928	0.738	3.33	44	0	1127.68448	3	376.5668335	1.849751065	13.99039
														TNEGVIEFR	4	1	1	N-Term(iTRAQ8plex)	1.015	1.085	0.94	2.95	40	0	1368.73528	2	684.8712769	-5.285323746	18.10006667
														FFSGYGR	1	1	1	N-Term(iTRAQ8plex)	0.951	0.962	0.837	-	22	0	1137.5916	2	569.2994385	-6.901393147	27.27339833
														LIVENLSSR	1	6	2	N-Term(iTRAQ8plex)	-	-	-	2.75	-	0	1334.79009	2	667.8986816	-3.337332809	30.127895
20149304	ISY1	pre-mRNA-splicing factor ISY1 homolog isoform 2	NP_065752.1	4.91	1	1	2	285	33.0	5.17	1.830	0.862	0.938																
														YASETLQAQSEEAR	2	3	1	N-Term(iTRAQ8plex)	1.83	0.862	0.938	5.05	61	0	1886.95171	2	943.9794922	6.325787221	23.87934167
20149498	FTL	ferritin light chain	NP_000137.2	19.43	2	2	12	175	20.0	5.78	0.290	0.224	0.614																
														TDPHLCDFLETHFLDEEVK	8	1	1	N-Term(iTRAQ8plex); C6(Methylthio); K19(iTRAQ8plex)	0.305	0.221	0.573	6.16	38	0	2942.43821	3	981.4842529	-4.862212879	52.63454667
														LGGPEAGLGEYLFER	4	1	1	N-Term(iTRAQ8plex)	0.287	0.224	0.72	4.53	53	0	1912.00664	2	956.5172119	8.005068415	43.16363667
20149560	STX4	syntaxin-4 isoform 3	NP_004595.2	13.80	3	3	7	297	34.2	6.28	0.985	0.703	0.902																
														LGSPDEEFFHK	2	1	1	N-Term(iTRAQ8plex); K11(iTRAQ8plex)	1.103	0.744	0.748	3.99	34	0	1914.01609	3	638.6768799	-2.970143043	26.94498833
														AIEPQKEEADENYNSVNTR	1	4	1	N-Term(iTRAQ8plex); K6(iTRAQ8plex)	0.875	1.232	1.912	3.44	-	1	2815.4269	4	704.6121826	-1.776182237	19.78913333
														VALVVHPGTAR	4	1	1	N-Term(iTRAQ8plex)	1.255	0.619	0.672	3.28	45	0	1423.86591	3	475.2934875	-1.974837305	21.85334167
20149572	OPTN	optineurin	NP_068815.2	1.91	1	1	2	577	65.9	5.17	-	-	4.430																
														MAEGEAEGSVK	2	1	1	N-Term(iTRAQ8plex); K11(iTRAQ8plex)	-	-	4.43	3.62	61	0	1715.89359	3	572.6360474	-9.250259025	22.69689333
20149675	EFHD2	EF-hand domain-containing protein D2	NP_077305.2	27.08	6	6	23	240	26.7	5.20	0.897	3.074	4.091																
														RADLNQGIGEPQSPSR	3	2	1	N-Term(iTRAQ8plex)	1.018	1.088	1.41	4.86	44	1	2029.06345	3	677.026001	-4.648262879	15.11273667
														LGAPQTHLGLK	7	5	1	N-Term(iTRAQ8plex); K11(iTRAQ8plex)	0.845	2.605	3.497	4.59	38	0	1743.0584	2	872.0328369	-8.802970629	23.67203167
														VFNPYTEFK	4	2	1	N-Term(iTRAQ8plex); K9(iTRAQ8plex)	0.959	4.125	6.036	4.02	39	0	1752.96257	2	876.9849243	-8.85317658	52.13619833
														DGFIDLMELK	4	4	1	N-Term(iTRAQ8plex); K10(iTRAQ8plex)	0.894	2.334	3.341	3.81	45	0	1788.99625	3	597.0036011	-3.566987546	42.12046167
														FEEEIKAEQEER	1	2	1	N-Term(iTRAQ8plex); K6(iTRAQ8plex)	0.737	1.37	1.679	3.6	-	1	2145.11342	4	537.0338135	-7.000689838	30.96577
														EFLLIFR	4	1	1	N-Term(iTRAQ8plex)	0.915	4.201	6.653	-	28	0	1241.74712	2	621.3771973	-7.129784013	41.01898833
20162550	PPP1R14B	protein phosphatase 1 regulatory subunit 14B	NP_619634.1	17.01	1	1	2	147	15.9	4.86	3.793	8.258	6.590																
														VYFQSPPGAAGEGPGGADDEGPVRR	2	1	1	N-Term(iTRAQ8plex)	3.793	8.258	6.59	4.15	36	1	2790.3682	3	930.7942505	-2.434476267	25.43047333
20162572	NSMCE3	non-structural maintenance of chromosomes element 3 homolog	NP_619649.1	3.62	1	1	2	304	34.3	9.28	1.240	0.575	0.756																
														VSELVQFLLIK	2	1	1	N-Term(iTRAQ8plex); K11(iTRAQ8plex)	1.24	0.575	0.756	3.66	40	0	1897.19034	3	633.0682983	-4.184888724	60.22810833
20270389	HIGD2A	HIG1 domain family member 2A, mitochondrial	NP_620175.1	23.58	1	1	6	106	11.5	10.20	-	-	-																
														ENPVVPIGCLATAAALTYGLYSFHR	4	1	1	N-Term(iTRAQ8plex); C9(Methylthio)	-	-	-	5.41	79	0	3013.55252	3	1005.189026	-1.884126205	70.85881
														ENPVVPIGCLATAAALTYGLYSFHR	2	1	1	N-Term(iTRAQ8plex); C9(Methylthio); Y18(iTRAQ8plex)	-	-	-	-	47	0	3317.75356	3	1005.189026	-1.884126205	70.85881
20336754	HIST1H2BJ	histone H2B type 1-J	NP_066402.2	71.43	2	12	456	126	13.9	10.32	1.386	0.652	0.821																
														KESYSIYVYK	5	5	1	N-Term(iTRAQ8plex); K1(iTRAQ8plex); K10(iTRAQ8plex)	1.386	0.588	0.821	4.38	32	1	2192.25547	3	731.4233398	-7.984935615	32.244215
														ESYSIYVYK	19	5	1	N-Term(iTRAQ8plex); K9(iTRAQ8plex)	1.368	0.653	0.851	4.04	46	0	1759.95781	2	880.4850464	-5.582587582	37.21028
														AMGIMNSFVNDIFER	55	17	2	N-Term(iTRAQ8plex); M2(Oxidation); M5(Oxidation)	-	-	-	7.49	84	0	2080.00713	2	1024.521362	5.222902458	67.32255667
														AMGIMNSFVNDIFER	121	17	2	N-Term(iTRAQ8plex); M2(Oxidation)	-	-	-	6.73	92	0	2064.02519	2	1024.521362	5.222902458	67.32255667
														AMGIMNSFVNDIFER	107	17	2	N-Term(iTRAQ8plex)	-	-	-	6.11	103	0	2048.03545	2	1024.521362	5.222902458	67.32255667
														HAVSEGTK	28	18	2	N-Term(iTRAQ8plex); K8(iTRAQ8plex)	-	-	-	4.68	45	0	1436.83133	3	479.6161499	1.486015215	9.764976667
														QVHPDTGISSK	30	18	2	N-Term(iTRAQ8plex); K11(iTRAQ8plex)	-	-	-	4.06	50	0	1776.99167	2	889.0061646	-0.781550078	14.01388
														LLLPGELAK	73	18	2	N-Term(iTRAQ8plex); K9(iTRAQ8plex)	-	-	-	3.96	36	0	1562.01006	2	781.5079956	-3.237311684	35.57815
														YTSAK	1	8	2	N-Term(iTRAQ8plex); K5(iTRAQ8plex)	-	-	-	2.78	-	0	1177.70386	3	393.2394714	0.152633142	11.19466
														EIQTAVR	10	17	2	N-Term(iTRAQ8plex)	-	-	-	-	32	0	1120.65927	3	374.2246094	-3.137818139	14.11598833
														LAHYNK	1	15	2	N-Term(iTRAQ8plex); K6(iTRAQ8plex)	-	-	-	-	27	0	1353.80912	3	451.9412231	-0.565158261	18.01176667
														IAGEASR	1	15	2	N-Term(iTRAQ8plex)	-	-	-	-	26	0	1007.57665	2	504.2919617	-2.068904796	13.73985833
														SAPAPK	3	16	2	N-Term(iTRAQ8plex); K6(iTRAQ8plex)	-	-	-	-	23	0	1178.73804	3	393.584198	2.290113704	9.457145
														STITSR	2	17	2	N-Term(iTRAQ8plex)	-	-	-	-	20	0	968.56542	2	484.7863464	-2.472779707	9.323636667
20336761	HEBP1	heme-binding protein 1	NP_057071.2	14.29	2	2	4	189	21.1	5.80	0.673	1.099	2.393																
														NSLFGSVETWPWQVLSK	2	1	1	N-Term(iTRAQ8plex); K17(iTRAQ8plex)	-	-	-	3.81	44	0	2586.40262	3	862.8057251	-5.826338445	52.70649
														AALEGTATYR	2	1	1	N-Term(iTRAQ8plex)	0.673	1.099	2.393	3.42	25	0	1356.74858	2	678.8779297	4.482374316	22.65845833
20357529	GNB2	guanine nucleotide-binding protein G(I)/G(S)/G(T) subunit beta-2	NP_005264.2	33.24	3	8	44	340	37.3	6.00	0.970	0.822	0.680																
														KACGDSTLTQITAGLDPVGR	2	1	1	N-Term(iTRAQ8plex); K1(iTRAQ8plex); C3(Methylthio)	0.73	0.606	0.824	5.87	53	1	2657.41239	3	886.475647	-3.301945935	39.34848
														ELPGHTGYLSCCR	10	2	1	N-Term(iTRAQ8plex); C11(Methylthio); C12(Methylthio)	0.983	0.855	0.68	3.43	26	0	1831.82224	2	916.4122925	-5.035960202	34.20467667
														LLLAGYDDFNCNIWDAMK	2	1	1	N-Term(iTRAQ8plex); C11(Methylthio); K18(iTRAQ8plex)	0.663	0.527	0.329	3.15	38	0	2756.37998	3	919.4648438	3.36517464	58.907865
														VSCLGVTDDGMAVATGSWDSFLK	10	3	2	N-Term(iTRAQ8plex); C3(Methylthio); K23(iTRAQ8plex)	-	-	-	7.93	81	0	3013.51975	3	1005.178101	8.865449721	61.94638667
														LIIWDSYTTNK	6	3	2	N-Term(iTRAQ8plex); K11(iTRAQ8plex)	-	-	-	4.88	49	0	1962.11052	2	981.5588989	-2.6145665	65.45539833
														AGVLAGHDNR	9	4	2	N-Term(iTRAQ8plex)	-	-	-	3.72	45	0	1313.72065	3	438.5796204	1.161989601	15.23490333
														VHAIPLR	4	5	2	N-Term(iTRAQ8plex)	-	-	-	2.89	40	0	1109.70444	3	370.572998	-4.722346944	20.27606833
														IYAMHWGTDSR	1	2	2	N-Term(iTRAQ8plex)	-	-	-	-	34	0	1640.8284	3	547.6143188	7.747301877	29.92760833
														IFDIR	2	11	3	N-Term(iTRAQ8plex)	-	-	-	-	25	0	967.58867	2	484.2963867	-2.412688602	25.13400667
														IWDVR	2	4	3	N-Term(iTRAQ8plex)	-	-	-	-	34	0	992.58318	2	496.7910767	-8.265975575	21.62371833
														IWDIR	1	4	3	N-Term(iTRAQ8plex)	-	-	-	-	33	0	1006.59648	2	503.8018799	-2.233421647	28.65469833
20357552	CTTN	src substrate cortactin isoform a	NP_005222.2	27.27	12	12	41	550	61.5	5.40	0.914	9.341	1.795																
														VDQSAVGFEYQGK	4	6	1	N-Term(iTRAQ8plex); K13(iTRAQ8plex)	1.584	21.512	2.967	5.82	74	0	2036.07781	2	1018.542542	-6.436351374	33.39107833
														LPSSPVYEDAASFK	2	4	1	N-Term(iTRAQ8plex); K14(iTRAQ8plex)	1.427	4.834	1.746	4.93	45	0	2119.14237	3	707.0523071	-5.102612946	33.93447
														QDSAAVGFDYK	4	2	1	N-Term(iTRAQ8plex); K11(iTRAQ8plex)	0.875	25.345	3.348	4.44	51	0	1808.95244	2	904.9798584	-6.341307195	30.91759667
														YGLFPANYVELR	14	3	1	N-Term(iTRAQ8plex)	0.897	9.414	1.917	4.4	48	0	1745.94768	2	873.477478	-2.950755841	45.11823667
														TQTPPVSPAPQPTEER	3	4	1	N-Term(iTRAQ8plex)	0.902	1.559	1.626	4.33	66	0	2039.0665	3	680.3603516	-2.275483657	17.02330667
														TVQGSGHQEHINIHK	1	6	1	N-Term(iTRAQ8plex); K15(iTRAQ8plex)	1.095	9.643	1.464	4.26	-	0	2293.24308	3	765.0858765	-8.322343003	15.17904333
														GPVSGTEPEPVYSMEAADYR	4	4	1	N-Term(iTRAQ8plex)	1.019	1.809	0.903	3.98	52	0	2459.16684	3	820.3937988	-1.39806115	32.24554333
														DYSSGFGGK	3	6	1	N-Term(iTRAQ8plex); K9(iTRAQ8plex)	0.663	5.935	1.399	3.65	33	0	1525.7979	2	763.4025879	-8.383901143	15.69745
														TVPVEAVTSK	2	4	1	N-Term(iTRAQ8plex); K10(iTRAQ8plex)	0.806	13.561	2.046	3.35	42	0	1638.97356	2	819.9904175	-9.220990165	16.90932167
														ENVFQEHQTLK	1	6	1	N-Term(iTRAQ8plex); K11(iTRAQ8plex)	0.899	5.067	1.533	2.98	-	0	1981.08377	3	661.0327759	-6.333358258	19.310315
														HCSQVDSVR	1	6	1	N-Term(iTRAQ8plex); C2(Methylthio)	0.892	1.695	1.076	-	29	0	1380.65778	3	460.8907776	-6.368548715	11.49195167
														YGLFPANYVELR	1	3	1	N-Term(iTRAQ8plex); Y1(iTRAQ8plex)	0.814	4.57	1.397	-	25	0	2050.15335	2	873.477478	-2.950755841	45.11823667
														CALGWDHQEK	1	5	1	N-Term(iTRAQ8plex); C1(Methylthio); K10(iTRAQ8plex)	1.644	16.131	2.138	-	20	0	1840.91397	3	614.3095093	-8.413059047	21.415355
20357568	AASDHPPT	L-aminoadipate-semialdehyde dehydrogenase-phosphopantetheinyl transferase	NP_056238.2	4.53	1	1	1	309	35.8	6.80	0.879	1.382	1.700																
														SFKDEWTQLDMFYR	1	1	1	N-Term(iTRAQ8plex); K3(iTRAQ8plex)	0.879	1.382	1.7	2.73	-	1	2474.25528	3	825.4232788	-3.317412021	48.13958167
20986531	MAPK1	mitogen-activated protein kinase 1	NP_620407.1	12.22	3	3	7	360	41.4	6.98	0.845	1.288	1.257																
														VADPDHDHTGFLTEYVATR	4	1	1	N-Term(iTRAQ8plex)	0.542	0.99	1.082	3.73	37	0	2448.20011	3	816.7382202	-3.9432665	28.56936667
														LKELIFEETAR	2	1	1	N-Term(iTRAQ8plex); K2(iTRAQ8plex)	0.849	1.335	1.543	3.04	-	1	1957.14859	3	653.0543823	-4.745463565	42.79633667
														FRHENIIGINDIIR	1	1	1	N-Term(iTRAQ8plex)	-	-	0.646	-	25	1	2014.16067	4	504.2956238	5.294481901	34.67495
21040371	DDX39A	ATP-dependent RNA helicase DDX39A	NP_005795.2	34.89	5	12	69	427	49.1	5.68	0.943	1.174	1.164																
														HFVLDECDKMLEQLDMR	2	1	1	N-Term(iTRAQ8plex); C7(Methylthio); K9(iTRAQ8plex)	-	-	-	5.21	30	1	2776.37832	4	694.8500366	1.139723775	60.468155
														CMALAQLLVEQNFPAIAIHR	4	2	1	N-Term(iTRAQ8plex); C1(Methylthio)	-	-	-	4.88	60	0	2588.38694	3	863.4671631	1.994952046	69.63166667
														NCPHVVVGTPGR	10	1	1	N-Term(iTRAQ8plex); C2(Methylthio)	0.964	1.219	1.305	4.35	48	0	1585.81926	2	793.4120483	-4.838036913	18.34010833
														HFVLDECDK	2	1	1	N-Term(iTRAQ8plex); C7(Methylthio); K9(iTRAQ8plex)	0.784	0.896	1.026	3.24	-	0	1759.88156	3	587.2987061	-8.635459477	26.12717833
														ILALVR	2	1	1	N-Term(iTRAQ8plex)	0.98	1.321	1.295	-	19	0	988.67656	2	494.8419189	-5.573136444	50.92614
														GLAITFVSDENDAK	14	3	2	N-Term(iTRAQ8plex); K14(iTRAQ8plex)	-	-	-	6.06	79	0	2088.12632	2	1044.567505	-7.484290413	26.62412
														LTLHGLQQYYVK	10	3	2	N-Term(iTRAQ8plex); K12(iTRAQ8plex)	-	-	-	4.75	41	0	2071.20659	2	1036.106934	-4.553255355	34.16349
														AIVDCGFEHPSEVQHECIPQAILGMDVLCQAK	1	2	2	N-Term(iTRAQ8plex); C5(Methylthio); C17(Methylthio); C29(Methylthio); K32(iTRAQ8plex)	-	-	-	4.54	-	0	4227.01772	4	1057.509888	0.425028086	64.3324
														DFLLKPELLR	8	2	2	N-Term(iTRAQ8plex); K5(iTRAQ8plex)	-	-	-	3.57	31	0	1852.14531	2	926.5745239	-5.345200131	36.26787833
														ILVATNLFGR	11	3	2	N-Term(iTRAQ8plex)	-	-	-	3.34	33	0	1407.85832	2	704.4328003	-3.016876052	49.87545833
														RDVQEIFR	3	3	2	N-Term(iTRAQ8plex)	-	-	-	3.21	37	1	1366.76714	2	683.887207	-5.373159259	19.86109167
														ELAFQISK	2	2	2	N-Term(iTRAQ8plex); K8(iTRAQ8plex)	-	-	-	3.06	40	0	1543.93393	3	515.3161621	2.274565025	38.51634333
21237748	CD151	CD151 antigen	NP_004348.2	3.95	1	1	2	253	28.3	7.47	0.772	0.470	0.868																
														LETFIQEHLR	2	1	1	N-Term(iTRAQ8plex)	0.772	0.47	0.868	3.5	28	0	1589.89359	3	530.6360474	-1.089670509	46.68753333
21264345	PRPH	peripherin	NP_006253.2	8.30	1	5	134	470	53.6	5.47	0.294	0.292	0.698																
														EYQELLNVK	1	2	1	N-Term(iTRAQ8plex); K9(iTRAQ8plex)	0.294	0.292	0.698	2.82	-	0	1744.00054	2	872.5039063	-5.496225375	49.174675

														KLLEGEESR	3	9	3	N-Term(iTRAQ8plex); K1(iTRAQ8plex)	-	-	-	3.13	26	1	1668.97317	4	417.9987488	-0.554869804	19.635685
														LLEGEESR	8	11	3	N-Term(iTRAQ8plex)	-	-	-	2.71	46	0	1236.67142	3	412.8953247	-1.892522837	20.19271
														IEEEIR	1	22	3	N-Term(iTRAQ8plex)	-	-	-	-	28	0	1092.61565	2	546.8114624	-4.222084244	20.37413667
														MALDIEIATYR	27	3	2	N-Term(iTRAQ8plex); M1(Oxidation)	-	-	-	4.78	75	0	1615.86435	3	533.9605103	-3.027619389	41.762285
														NLQEAEEWYK	30	3	2	N-Term(iTRAQ8plex); K10(iTRAQ8plex)	-	-	-	4.62	77	0	1918.01006	2	959.5106812	-1.348068991	32.387445
														MALDIEIATYR	61	3	2	N-Term(iTRAQ8plex)	-	-	-	4.57	79	0	1599.86698	3	533.9605103	-3.027619389	41.762285
														MALDIEIATYR	3	3	2	N-Term(iTRAQ8plex); Y10(iTRAQ8plex)	-	-	-	-	44	0	1904.06426	3	533.9605103	-3.027619389	41.762285
														MALDIEIATYR	1	3	2	N-Term(iTRAQ8plex); M1(Oxidation); Y10(iTRAQ8plex)	-	-	-	-	37	0	1920.0656	3	533.9605103	-3.027619389	41.762285
21264355	SMARCE1	SWI/SNF-related matrix-associated actin-dependent regulator of chromatin subfamily E member 1	NP_003070.3	2.43	1	1	2	411	46.6	4.88	1.075	1.010	0.961																
														AEAALEEESR	2	1	1	N-Term(iTRAQ8plex)	1.075	1.01	0.961	3.47	37	0	1408.73039	2	704.8688354	5.830056958	23.89951333
21264365	NUP98	nuclear pore complex protein Nup98-Nup96 isoform 1 proprotein	NP_057404.2	0.56	1	1	1	1800	195.7	6.37	0.974	0.754	0.850																
														LSWHLWEVLR	1	1	1	N-Term(iTRAQ8plex)	0.974	0.754	0.85	-	30	0	1642.9473	3	548.3206177	6.189168298	51.41357
21264578	TSPAN1	tetraspanin-1	NP_005718.2	5.39	1	1	2	241	26.3	5.25	1.483	2.040	0.895																
														VEGCFNQLLYDIR	2	2	1	N-Term(iTRAQ8plex); C4(Methylthio)	1.483	2.04	0.895	4.24	44	0	1919.96903	3	640.6611938	1.508354003	64.853505
21265040	MRPL22	39S ribosomal protein L22, mitochondrial isoform a	NP_054899.2	16.50	3	3	5	206	23.6	9.94	0.900	0.545	0.666																
														LVEGPPPPPEPPK	2	2	1	N-Term(iTRAQ8plex); K13(iTRAQ8plex)	0.857	0.702	0.666	5.55	32	0	1962.16532	3	654.7266235	6.745637257	27.07709667
														SNLYIAESTSGR	2	2	1	N-Term(iTRAQ8plex)	0.9	0.423	0.364	4.18	42	0	1601.84111	2	801.4241943	-1.597691622	25.10737833
														RPAEIYHCR	1	1	1	N-Term(iTRAQ8plex); C8(Methylthio)	1.146	-	0.788	-	23	0	1494.75759	4	374.4448547	-2.377131057	17.92025
21265080	MRPL18	39S ribosomal protein L18, mitochondrial isoform 1	NP_054880.2	12.78	2	2	2	180	20.6	9.54	0.851	0.702	0.701																
														LQSAMTEGGVVLR	1	1	1	N-Term(iTRAQ8plex)	0.736	0.62	0.637	3.77	-	0	1664.93291	2	832.9700928	1.295924216	33.69883167
														NVVACESIGR	1	1	1	N-Term(iTRAQ8plex); C5(Methylthio)	0.984	0.796	0.77	2.76	-	0	1397.71489	2	699.361084	-2.433222448	28.35984667
21265096	MRPL50	39S ribosomal protein L50, mitochondrial	NP_061924.1	36.71	4	4	5	158	18.3	7.88	0.984	0.584	0.609																
														VRDVLDFYNVPIQDR	1	1	1	N-Term(iTRAQ8plex)	0.665	0.558	0.62	4.78	-	1	2153.16025	3	718.3916016	-2.53574368	41.88621667
														EKEPVVVETVEEK	2	1	1	N-Term(iTRAQ8plex); K2(iTRAQ8plex); K13(iTRAQ8plex)	0.984	0.584	0.609	4.32	32	1	2427.40903	3	809.8078613	-0.807678311	24.923265
														FNLLAHLADDLGHVVPNSR	1	1	1	N-Term(iTRAQ8plex)	-	-	-	2.76	-	0	2392.31948	4	598.8353271	6.490526825	60.770215
														KEPILVCPPLR	1	1	1	N-Term(iTRAQ8plex); K1(iTRAQ8plex); C7(Methylthio)	1.013	0.602	0.451	-	30	1	1919.13993	3	640.3848267	-1.654683688	34.85357167
21281677	WDR36	WD repeat-containing protein 36	NP_644810.1	7.78	6	6	13	951	105.3	7.53	1.781	1.054	0.815																
														SAPFFIPTIPGLVPR	4	1	1	N-Term(iTRAQ8plex)	1.786	1.212	0.815	4.3	46	0	1916.12883	2	958.5680542	-1.220962559	58.34219333
														ALGLFSNDIPHVVR	2	1	1	N-Term(iTRAQ8plex)	1.707	2.018	1.756	4.14	36	0	1842.05179	3	614.6887817	-1.183101998	41.34199833
														GLQNTMSVR	2	1	1	N-Term(iTRAQ8plex)	2.096	0.938	0.658	3.36	33	0	1309.72759	2	655.3674316	5.791556448	22.68861667
														FRQDWGPITSISFR	2	1	1	N-Term(iTRAQ8plex)	1.804	0.93	0.714	3.11	30	1	2014.07572	3	672.0300903	-2.747440852	41.495795
														IWIFDGPTGEGR	2	1	1	N-Term(iTRAQ8plex)	1.781	1.054	0.793	2.75	55	0	1651.86626	2	826.4367676	-5.056561757	35.020885
														VVNLGVLAQK	1	1	1	N-Term(iTRAQ8plex); K10(iTRAQ8plex)	1.234	1.142	1.158	-	23	0	1649.05863	3	550.3577271	0.968190164	26.88611167
21327667	BOP1	ribosome biogenesis protein BOP1	NP_056016.1	19.57	9	9	15	746	83.6	6.19	2.002	0.867	1.130																
														LALPGHAESYNPPPEYLLSEEER	2	1	1	N-Term(iTRAQ8plex)	1.618	0.432	1.006	5.76	37	0	2915.4686	3	972.4943848	-1.485268311	37.48129667
														GDYLAVVLATQGHTQVLIHQLSR	1	1	1	N-Term(iTRAQ8plex)	-	-	-	5.76	-	0	2823.58095	4	706.6506958	0.928616239	52.24663833
														TTEEQVQASTPCPR	2	1	1	N-Term(iTRAQ8plex); C12(Methylthio)	3.646	0.854	1.13	5.12	58	0	1896.9185	2	948.9628906	4.619244305	24.97138333
														NTVGNVPLEWYDDFPHVGYDLDGR	2	1	1	N-Term(iTRAQ8plex)	1.028	0.449	0.703	3.88	30	0	3082.48606	3	1028.16687	0.374957596	51.532495
														DLGVLDVIFHPTQPWVFSSGADGTVR	1	1	1	N-Term(iTRAQ8plex)	3.215	-	1.394	3.39	-	0	3117.6489	3	1039.887817	5.676584828	64.38101667
														CLDLYLCPR	3	1	1	N-Term(iTRAQ8plex); C1(Methylthio); C7(Methylthio)	1.805	0.799	1.036	3.28	32	0	1491.70549	2	746.3563843	-5.296866811	54.73469
														LTDEQVALVR	2	1	1	N-Term(iTRAQ8plex)	2.002	0.902	0.912	3.05	40	0	1447.8394	2	724.4233398	-1.953415893	28.22731667
														TRDELDQFLDK	1	1	1	N-Term(iTRAQ8plex); K11(iTRAQ8plex)	1.525	1.09	1.199	-	34	1	1988.08573	3	663.3667603	-2.612456902	30.761565
														LYHLLR	1	1	1	N-Term(iTRAQ8plex)	2.486	1.245	1.878	-	27	0	1118.69565	3	373.5700684	-2.77955123	25.90457667
21359867	CYC1	cytochrome c1, heme protein, mitochondrial precursor	NP_001907.2	25.54	5	5	11	325	35.4	9.00	1.147	0.610	0.562																
														ELAAEVEVQDGPNEDGEMFMRPGK	2	2	1	N-Term(iTRAQ8plex); M18(Oxidation); M20(Oxidation); K24(iTRAQ8plex)	-	-	-	4.77	28	0	3288.59755	4	822.9048462	0.043661659	29.27897333
														HGGEDYVFSLLTGYCEPPTGVSLR	2	2	1	N-Term(iTRAQ8plex); C15(Methylthio)	0.434	-	0.466	4.63	27	0	2947.44229	3	983.1522827	5.107002284	62.40286
														GLLSSLDHTSIR	2	2	1	N-Term(iTRAQ8plex)	1.281	0.966	1.04	3.38	52	0	1602.90232	3	534.97229	-5.854650476	30.87478333
														LFDYFPKPYPNSEAAR	1	2	1	N-Term(iTRAQ8plex); K7(iTRAQ8plex)	0.829	0.661	0.409	2.91	-	0	2523.34165	3	841.7854004	-3.017180861	41.19143833
														DVCTFLR	4	2	1	N-Term(iTRAQ8plex); C3(Methylthio)	1.157	0.574	0.596	-	32	0	1203.61199	2	602.3096313	-3.9765512	41.60877333
21361091	UCHL1	ubiquitin carboxyl-terminal hydrolase isozyme L1	NP_004172.2	38.57	6	6	16	223	24.8	5.48	0.561	0.650	3.419																
														QTIGNSCGTIGLIHAVANNQDK	5	1	1	N-Term(iTRAQ8plex); C7(Methylthio); K22(iTRAQ8plex)	0.975	1.488	2.833	6.19	26	0	2908.51137	4	727.8833008	-3.994845991	35.86658333
														NEAIQAAHDAVAQEGQCR	6	1	1	N-Term(iTRAQ8plex); C17(Methylthio)	0.342	-	7.369	5.83	62	0	2261.06467	3	754.3597412	-2.588124641	28.37828167
														MPFPVNHGASSEDTLLK	1	1	1	N-Term(iTRAQ8plex); K17(iTRAQ8plex)	0.561	0.65	3.419	3.95	-	0	2451.30027	3	817.7716064	-6.529275211	30.32114
														MPFPVNHGASSEDTLLKDAAK	1	1	1	N-Term(iTRAQ8plex); K17(iTRAQ8plex); K21(iTRAQ8plex)	-	-	-	3.65	-	1	3140.69379	5	628.9445801	-7.640801985	31.583865
														MQLKPMEINPEMLNK	1	1	1	N-Term(iTRAQ8plex); K4(iTRAQ8plex); K15(iTRAQ8plex)	0.336	0.318	1.979	3.17	-	0	2728.50602	3	910.1735229	-9.798659849	37.33774
														MPFPVNHGASSEDTLLK	1	1	1	N-Term(iTRAQ8plex); M1(Oxidation); K17(iTRAQ8plex)	0.962	0.392	4.108	3.04	-	0	2467.29416	3	817.7716064	-6.529275211	30.32114
														LGFEDGSVLK	1	1	1	N-Term(iTRAQ8plex); K10(iTRAQ8plex)	1.638	0.935	2.972	-	41	0	1672.96067	3	558.3250732	-7.390982756	40.40660167
21361103	SLC25A12	calcium-binding mitochondrial carrier protein Aralar1	NP_003696.2	8.26	2	4	6	678	74.7	8.38	1.061	0.972	1.006																
														HLNYTEFTQFLQELQLEHAR	2	2	1	N-Term(iTRAQ8plex)	-	0.895	-	5.18	38	0	2821.4601	4	706.1204834	0.911731393	52.453525
														NIFLQYASTEVDGER	2	1	1	N-Term(iTRAQ8plex)	1.061	1.056	1.006	3.4	48	0	2046.03716	2	1023.522217	-3.628637058	45.76051833
														LTVNDFVR	1	8	2	N-Term(iTRAQ8plex)	-	-	-	-	25	0	1267.73064	2	634.3689575	-0.451894843	32.51188833
														GLIPQLIGVAPEK	2	8	2	N-Term(iTRAQ8plex); K13(iTRAQ8plex)	-	-	-	4.9	53	0	1943.21575	2	972.1115112	0.378002771	36.27524667
21361144	PSMC3	26S protease regulatory subunit 6A	NP_002795.2	27.11	8	8	22	439	49.2	5.24	0.919	1.920	1.777																
														TMLELLNQLDGFQPNTQVK	4	2	1	N-Term(iTRAQ8plex); K19(iTRAQ8plex)	0.671	1.739	1.624	7.15	79	0	2797.53861	3	933.1843872	0.252747573	51.68764667
														LAGPQLVQMFIGDGAK	2	2	1	N-Term(iTRAQ8plex); K16(iTRAQ8plex)	0.919	2.533	1.861	5.15	42	0	2253.275	3	751.7631836	-6.041769764	44.650775
														APSIIFIDELDAIGTK	2	2	1	N-Term(iTRAQ8plex); K16(iTRAQ8plex)	1.076	1.903	2.363	4.57	36	0	2311.33078	3	771.1151123	-2.685741038	49.80527
														KMNVSPDVNYEELAR	2	2	1	N-Term(iTRAQ8plex); K1(iTRAQ8plex)	0.881	3.145	2.33	4.3	39	1	2373.25705	3	791.7572021	-5.165097759	28.14305
														QIQELVEAIVLPMNHK	2	2	1	N-Term(iTRAQ8plex); K16(iTRAQ8plex)	-	-	-	4.03	39	0	2470.43862	4	618.3651123	2.986529879	61.13554833
														QTYFLPVIGLVDAEK	2	2	1	N-Term(iTRAQ8plex); K15(iTRAQ8plex)	-	-	-	3.43	34	0	2301.31675	3	767.7770996	-6.407515134	52.929665
														KIEFPMPNEEAR	3	2	1	N-Term(iTRAQ8plex); K1(iTRAQ8plex)	0.72	1.278	1.185	3.34	35	1	2069.11502	3	690.3758545	-8.709564605	25.59203167
														VDILDPALLR	4	2	1	N-Term(iTRAQ8plex)	0.795	2.506	1.931	3.08	28	0	1428.86894	2	714.9381104	-2.706488157	47.761895
														KMNVSPDVNYEELAR	1	2	1	N-Term(iTRAQ8plex); K1(iTRAQ8plex); M2(Oxidation)	0.95	1.92	1.652	3.05	-	1	2389.25241	3	791.7572021	-5.165097759	28.14305
21361176	ALDH1A1	retinal dehydrogenase 1	NP_000680.2	63.87	23	25	456	501	54.8	6.73	0.929	0.918	6.334																
														EAGFPPGVVNIVPGYGPTAGAAISSHMDIDK	9	1	1	N-Term(iTRAQ8plex); K31(iTRAQ8plex)	1.531	1.572	2.64	6.93	77	0	3675.93088	3	1225.981812	0.176831484	45.46269333
														FPVFNPATEEELCQVEEGDKEDVDK	7	1	1	N-Term(iTRAQ8plex); C13(Methylthio); K20(iTRAQ8plex); K25(iTRAQ8plex)	-	-	-	6.85	51	1	3825.89419	4	957.2290039	-0.660382875	44.51279333
														ANNTFYGLSAGVFTK	38	1	1	N-Term(iTRAQ8plex); K15(iTRAQ8plex)	1.114	1.132	3.977	6.39	79	0	2198.1895	2	1099.598389	-7.783940486	40.30260167
														IFVEESIYDEFVR	67	1	1	N-Term(iTRAQ8plex)	1.131	0.992	7.319	5.83	68	0	1950.02031	2	975.5137939	2.087142683	61.56432833
														LYSNAYLNDLAGCIK	65	1	1	N-Term(iTRAQ8plex); C13(Methylthio); K15(iTRAQ8plex)	2.022	0.739	11.196	5.58	81	0	2312.20766	2	1156.615112	-0.417049154	56.48538
														IFINNEWHDSVSGK	18	1	1	N-Term(iTRAQ8plex); K14(iTRAQ8plex)	1.383	2.116	6.36	5.51	70	0	2254.18682	2	1127.597046	-9.256607742	32.60154333
														IGPALSCGNTVVVKPAEQTPLTALHVASLIK	2	1	1	N-Term(iTRAQ8plex); C7(Methylthio); K14(iTRAQ8plex); K31(iTRAQ8plex)	-	-	-	5.46	-	0	4086.33741	5	818.0733032	-5.215598856	53.88884833
														IAKEEIFGPVQQIMK	26	1	1	N-Term(iTRAQ8plex); K3(iTRAQ8plex); K15(iTRAQ8plex)	0.988	1.012	7.203	5.45	50	1	2643.54841	2	1322.28833	0.851967898	41.65688833
														LLLATMESMNGGK	2	1	1	N-Term(iTRAQ8plex); K13(iTRAQ8plex)	-	-	-	5.31	44	0	1973.0839	3	658.3661499	-9.196184208	70.996995
														TIPIDGNFFTYTR	37	1	1	N-Term(iTRAQ8plex)	0.878	0.932	5.565	5.15	67	0	1848.96988	3	616.994812	-5.353529517	45.14330333
														EAGFPPGVVNIVPGYGPTAGAAISSHMDIDK	5	1	1	N-Term(iTRAQ8plex); M27(Oxidation); K31(iTRAQ8plex)	-	-	-	5.12	43	0	3691.91672	3	1225.981812	0.176831484	45.46269333
														YILGNPLTPGVTQGPQIDKEQYDK	5	1	1	N-Term(iTRAQ8plex); K19(iTRAQ8plex); K24(iTRAQ8plex)	-	-	-	5.08	-	1	3586.97036	4	897.4980469	-5.044279209	36.32122
														IAKEEIFGPVQQIMK	8	1	1	N-Term(iTRAQ8plex); K3(iTRAQ8plex); M14(Oxidation); K15(iTRAQ8plex)	0.874	0.918	5.321	4.97	39	1	2659.54264	2	1322.28833	0.851967898	41.65688833
														GYFVQPTVFSNVTDEMR	17	1	1	N-Term(iTRAQ8plex)	0.733	1.204	2.656	4.86	73	0	2294.13457	2	1147.570923	-3.645042008	45.30883333
														LECGGGPWGNK	29	1	1	N-Term(iTRAQ8plex); C3(Methylthio); K11(iTRAQ8plex)	0.813	0.861	5.545	4.78	59	0	1771.89116	2	886.4492188	-9.510414845	32.37854833
														ELGEYGFHEYTEVK	13	1	1	N-Term(iTRAQ8plex); K14(iTRAQ8plex)	0.92	0.776	9.979	4.34	29	0	2309.17765	3	770.3973999	-5.787706714	29.05688333
														VAFTGSTEVGK	8	1	1	N-Term(iTRAQ8plex); K11(iTRAQ8plex)	0.786	0.425	17.321	4.33	59	0	1703.96245	2	852.4848633	-9.619553236	17.99047167
														EEIFGPVQQIMK	2	1	1	N-Term(iTRAQ8plex); M11(Oxidation); K12(iTRAQ8plex)	0.617	1.966	4.664	4.14	30	0	2043.1232	3	676.3804932	-9.217507582	67.80481833
														QAFQIGSPWR	11	1	1	N-Term(iTRAQ8plex)	0.632	0.749	6.823	4.05	41	0	1493.8073	3	498.6115417	2.269814286	32.52315667
														EEIFGPVQQIMK	3	1	1	N-Term(iTRAQ8plex); K12(iTRAQ8plex)	0.984	1.708	5.106	3.86	35	0	2027.12693	3	676.3804932	-9.217507582	67.80481833
														GYFVQPTVFSNVTDEMR	6	1	1	N-Term(iTRAQ8plex); M16(Oxidation)	0.993	0.742	1.419	3.75	41	0	2310.13034	2	1147.570923	-3.645042008	45.30883333
														ILDLIESGK	4	1	1	N-Term(iTRAQ8plex); K9(iTRAQ8plex)	0.477	0.507	3.893	3.64	30	0	1595.97434	3	532.6629639	-5.340279121	43.300025
														ILDLIESGKK	15	1	1	N-Term(iTRAQ8plex); K9(iTRAQ8plex); K10(iTRAQ8plex)	0.549	0.272	13.914	3.5	42	1	2028.26995	2	1014.638611	-6.530807373	49.21442
														YCAGWADK	29	1	1	N-Term(iTRAQ8plex); C2(Methylthio); K8(iTRAQ8plex)	0.662	0.677	10.896	3.34	31	0	1567.77275	2	784.3900146	-8.285399209	31.50220333
														LYSNAYLNDLAGCIK	9	1	1	N-Term(iTRAQ8plex); Y2(iTRAQ8plex); C13(Methylthio); K15(iTRAQ8plex)	0.701	0.921	9.02	-	50	0	2616.41142	2	1156.615112	-0.417049154	56.48538
														GYFVQPTVFSNVTDEMR	4	1	1	N-Term(iTRAQ8plex); Y2(iTRAQ8plex)	1.134	-	11.982	-	43	0	2598.33315	2	1147.570923	-3.645042008	45.30883333
														GYFVQPTVFSNVTDEMR	2	1	1	N-Term(iTRAQ8plex); Y2(iTRAQ8plex); M16(Oxidation)	1.038	0.908	5.244	-	31	0	2614.32914	2	1147.570923	-3.645042008	45.30883333
														SLDDVIK	2	1	1	N-Term(iTRAQ8plex); K7(iTRAQ8plex)	1.11	0.93	6.156	-	30	0	1397.83415	2	699.4207153	-8.489745299	19.22553333
														SLDDVIKR	2	1	1	N-Term(iTRAQ8plex); K7(iTRAQ8plex)	1.017	0.919	8.869	-	30	1	1553.93352	2	777.4703979	-8.766154661	22.87644167
														TMDASER	1	1	1	N-Term(iTRAQ8plex)	-	0.228	22.68	-	27	0	1113.54436	2	557.2758179	-6.144504039	9.346673333
														ANNTFYGLSAGVFTK	1	1	1	N-Term(iTRAQ8plex); Y6(iTRAQ8plex); K15(iTRAQ8plex)	-	-	-	-	26	0	2502.39248	2	1099.598389	-7.783940486	40.30260167
														IFVEESIYDEFVR	1	1	1	N-Term(iTRAQ8plex); Y8(iTRAQ8plex)	-	-	-	-	24	0	2254.21767	2	975.5137939	2.087142683	61.56432833
														VTLELGGK	5	10	4	N-Term(iTRAQ8plex); K8(iTRAQ8plex)	-	-	-	3.03	32	0	1424.8792	2	712.9432373	-9.904917305	26.02754667
														LADLIER	3	2	2	N-Term(iTRAQ8plex)	-	-	-	2.74	31	0	1133.67644	2	567.3418579	-5.964752776	36.70049333
21361337	EIF5	eukaryotic translation initiation factor 5	NP_001960.2	6.50	2	2	6	431	49.2	5.58	1.340	1.954	4.347																
														FVLCPECENPETDLHVNPK	2	1	1	N-Term(iTRAQ8plex); C4(Methylthio); C7(Methylthio); K19(iTRAQ8plex)	1.47	1.786	4.347	5.86	30	0	2884.3945	3	962.1363525	-0.609810069	47.65216833
														VNILFDFVK	4	1	1	N-Term(iTRAQ8plex); K9(iTRAQ8plex)	1.25	2.361	3.288	3.42	40	0	1703.01963	3	568.3447266	-9.163054998	78.687085
21361370	PYGB	glycogen phosphorylase, brain form	NP_002853.2	8.90	3	5	10	843	96.6	6.86	1.386	1.056	1.432																
														IGEEFLTDLSQLKK	2	1	1	N-Term(iTRAQ8plex); K13(iTRAQ8plex); K14(iTRAQ8plex)	-	-	-	4.7	32	1	2533.48745	4	634.1273193	-5.140675263	54.10671
														HLDHVAALFPGDVDR	2	1	1	N-Term(iTRAQ8plex)	1.386	1.056	1.432	3.92	30	0	1966.03598	3	656.0168457	-4.520487264	27.78011833
														RWLLLCNPGLADTIVEK	2	1	1	N-Term(iTRAQ8plex); C6(Methylthio); K17(iTRAQ8plex)	-	-	-	3.7	26	1	2595.46718	4	649.6222534	2.316150215	55.26685667
														VLYPNDNFFEGK	2	5	2	N-Term(iTRAQ8plex); K12(iTRAQ8plex)	-	-	-	2.85	34	0	2051.09543	3	684.3699951	-5.070070497	60.630785

														LKQEYFVVAATLQDIIR	2	5	2	N-Term(iTRAQ8plex); K2(iTRAQ8plex)	-	-	-	2.75	25	1	2615.53763	3	872.517395	-0.194794141	67.903125
21361376	SF3A2	splicing factor 3A subunit 2	NP_009096.2	5.60	2	2	8	464	49.2	9.64	1.168	0.835	0.909																
														WQYLLMAAEPYETIAFK	6	1	1	N-Term(iTRAQ8plex); K17(iTRAQ8plex)	1.169	0.711	0.764	5	46	0	2682.44309	3	894.8192139	-1.164217966	60.646005
														QFFLQFHFK	2	1	1	N-Term(iTRAQ8plex); K9(iTRAQ8plex)	1.168	0.98	1.081	3.54	37	0	1850.05021	3	617.3549194	-3.862732589	42.20721
21361399	PPP2R1A	serine/threonine-protein phosphatase 2A 65 kDa regulatory subunit A alpha isoform	NP_055040.2	20.54	5	9	23	589	65.3	5.11	1.014	1.778	1.518																
														DNTIEHLLPLFLAQLKDECPEVR	1	2	1	N-Term(iTRAQ8plex); K16(iTRAQ8plex); C19(Methylthio)	-	-	-	4.19	-	1	3347.78669	5	670.3631592	-2.566744049	67.49207
														VLAMSGDPNYLHR	2	2	1	N-Term(iTRAQ8plex)	0.929	1.839	1.339	3.25	44	0	1776.93088	3	592.9818115	-3.376597115	26.45909833
														NLCSDDTPMVR	2	1	1	N-Term(iTRAQ8plex); C3(Methylthio)	0.99	1.885	1.829	3.02	26	0	1600.73454	2	800.8709106	-5.614691685	22.310065
														YFAQEALTVLSLA	1	2	1	N-Term(iTRAQ8plex)	1.396	1.322	0.782	-	40	0	1729.95806	2	865.482666	-5.645159268	85.75052333
														LAGGDWFTSR	1	1	1	N-Term(iTRAQ8plex)	1.038	1.719	1.722	-	21	0	1413.7332	2	707.3702393	-6.825477652	27.09787667
														QLSQSLLPAIVELAEDAK	2	11	2	N-Term(iTRAQ8plex); K18(iTRAQ8plex)	-	-	-	6.01	47	0	2533.4791	3	845.1645508	3.669970577	62.36273833
														TSACGLFSVCYPR	6	9	2	N-Term(iTRAQ8plex); C4(Methylthio); C10(Methylthio)	-	-	-	4.13	58	0	1799.83	2	900.4186401	2.517100742	53.112205
														KLSTIALALGVER	4	9	2	N-Term(iTRAQ8plex); K1(iTRAQ8plex)	-	-	-	3.48	59	1	1979.23734	3	660.4172974	-5.068851501	44.39236167
														DCEAEVR	4	11	2	N-Term(iTRAQ8plex); C2(Methylthio)	-	-	-	2.92	29	0	1171.5418	2	586.2703857	-4.632761802	21.63871833
21361416	RRAS2	ras-related protein R-Ras2 isoform a	NP_036382.2	11.27	2	2	10	204	23.4	6.01	1.154	0.642	0.794																
														MNVDQAFHELVR	6	4	1	N-Term(iTRAQ8plex)	1.249	0.69	0.794	5.31	75	0	1762.91764	3	588.31073	-2.044683494	36.24319667
														LVVVGGGGVGK	4	2	1	N-Term(iTRAQ8plex); K11(iTRAQ8plex)	0.873	0.33	0.725	4.04	54	0	1549.98625	2	775.4967651	-1.533979128	33.38502833
21361437	HTATSF1	HIV Tat-specific factor 1	NP_055315.2	4.50	2	2	3	755	85.8	4.40	0.861	1.888	1.614																
														NMFHPMDFEDDPLVLNEIR	2	1	1	N-Term(iTRAQ8plex)	0.74	2.593	1.634	5.86	56	0	2636.28385	3	879.4328003	1.781420051	49.593905
														LRGWEAFLNAPEANR	1	1	1	N-Term(iTRAQ8plex)	1.002	1.375	1.594	3.19	-	1	2048.09214	3	683.3688965	-2.853922815	44.690385
21361462	EHD2	EH domain-containing protein 2	NP_055416.2	4.24	2	2	5	543	61.1	6.46	0.404	0.453	0.709																
														LFELEEQDLFR	2	1	1	N-Term(iTRAQ8plex)	0.228	0.285	0.671	4.28	44	0	1742.93913	2	871.9732056	7.13347504	50.91021167
														LEGHGLPANLPR	3	1	1	N-Term(iTRAQ8plex)	0.405	0.722	0.93	2.82	37	0	1577.90238	3	526.6389771	-2.657577373	24.05257833
21361468	NOL11	nucleolar protein 11 isoform 1	NP_056277.2	5.70	4	4	11	719	81.1	6.07	1.518	0.618	0.716																
														QTPDFHTVIGDTVTGLLER	6	2	1	N-Term(iTRAQ8plex)	1.248	0.717	0.742	4.49	32	0	2403.28562	3	801.7667236	1.417371457	45.816195
														ATLSAEVYR	2	1	1	N-Term(iTRAQ8plex)	1.128	0.451	0.384	3.38	28	0	1313.74114	2	657.3742065	3.391205325	25.42660833
														HIEVEVR	2	2	1	N-Term(iTRAQ8plex)	1.917	0.727	0.7	-	37	0	1185.68616	3	395.9000854	-3.06843739	14.482585
														FFVVFR	1	1	1	N-Term(iTRAQ8plex)	1.628	0.618	0.716	-	21	0	1118.66069	2	559.8339844	-5.121454647	69.07098
21361497	ACAD9	acyl-CoA dehydrogenase family member 9, mitochondrial	NP_054768.2	16.75	7	7	18	621	68.7	7.96	0.912	0.619	0.524																
														IPVENILGEVGDGFK	4	2	1	N-Term(iTRAQ8plex); K15(iTRAQ8plex)	0.788	0.568	0.394	5.55	61	0	2195.26494	2	1098.136108	5.3200307	50.00348333
														MYIALTGLQHAGR	2	3	1	N-Term(iTRAQ8plex)	0.912	0.717	0.65	4.3	32	0	1734.95652	3	578.9903564	-3.565054228	36.42157167
														EVFPFPEVSQDELNEINQFLGPVEK	2	1	1	N-Term(iTRAQ8plex); K25(iTRAQ8plex)	-	-	-	4.06	39	0	3512.84365	4	878.9663696	0.742229341	66.21533333
														LASGEHIAAFCLTEPASGSDAASIR	1	2	1	N-Term(iTRAQ8plex); C11(Methylthio)	0.559	1.468	0.844	3.46	-	0	2824.3967	3	942.137085	1.940001137	41.89333
														ITAFIVER	5	2	1	N-Term(iTRAQ8plex)	0.92	0.619	0.476	3.13	32	0	1252.75224	2	626.8794556	-4.044526503	29.44399167
														TVETLLLR	2	3	1	N-Term(iTRAQ8plex)	0.96	0.593	0.524	2.9	24	0	1248.78069	2	624.8939819	-1.780979507	36.101765
														VSTVMDTVGR	2	3	1	N-Term(iTRAQ8plex)	1.448	0.891	0.654	2.81	23	0	1368.74919	2	684.8782349	2.412280189	30.02582667
21361565	ATP5F1	ATP synthase F(0) complex subunit B1, mitochondrial precursor	NP_001679.2	29.69	8	8	31	256	28.9	9.36	0.809	0.569	0.600																
														HVVQSISTQQEK	2	1	1	N-Term(iTRAQ8plex); K12(iTRAQ8plex)	0.67	0.42	0.506	5.92	56	0	1992.13144	3	664.715332	-0.998155145	13.20884333
														YGLIPEEFFQFLYPK	14	1	1	N-Term(iTRAQ8plex); K15(iTRAQ8plex)	0.665	0.405	0.38	5.43	56	0	2499.38376	3	833.7994385	2.132877619	65.72364667
														QASIQHIQNAIDTEK	2	1	1	N-Term(iTRAQ8plex); K15(iTRAQ8plex)	0.937	0.569	0.82	4.16	33	0	2304.26749	3	768.7606812	-4.049483408	27.38240667
														YGPFVADFADK	2	1	1	N-Term(iTRAQ8plex); K11(iTRAQ8plex)	0.981	1.179	0.807	3.52	29	0	1837.99546	3	613.3366699	0.525145817	61.68767333
														LAQLEEAK	4	1	1	N-Term(iTRAQ8plex); K8(iTRAQ8plex)	0.886	0.679	0.69	3.33	40	0	1509.909	2	755.4584961	0.016072153	24.07318167
														RHYLFDVQR	1	1	1	N-Term(iTRAQ8plex)	1.649	-	0.621	3.01	-	1	1537.84939	4	385.217804	-3.072706424	18.38896167
														HYLFDVQR	3	1	1	N-Term(iTRAQ8plex)	0.808	0.741	0.652	2.81	45	0	1381.74515	3	461.2532349	-5.67395525	22.19463667
														CIADLK	1	1	1	N-Term(iTRAQ8plex); C1(Methylthio); K6(iTRAQ8plex)	0.744	0.529	0.406	-	33	0	1316.7537	3	439.5894165	0.792829913	30.100315
														YGLIPEEFFQFLYPK	2	1	1	N-Term(iTRAQ8plex); Y13(iTRAQ8plex); K15(iTRAQ8plex)	2.847	1.349	1.208	-	31	0	2803.59145	3	833.7994385	2.132877619	65.72364667
21361621	PGM1	phosphoglucomutase-1 isoform 1	NP_002624.2	7.12	3	3	5	562	61.4	6.76	0.659	0.770	0.699																
														ADNFEYSDPVDGSISR	2	3	1	N-Term(iTRAQ8plex)	1.269	6.651	5.278	5.11	56	0	2075.99272	2	1038.5	4.981751566	29.278295
														QEATLVVGGDGR	2	1	1	N-Term(iTRAQ8plex)	0.126	0.77	0.699	3.62	52	0	1505.82207	2	753.4146729	-0.333516745	23.17131667
														LSGTGSAGATIR	1	3	1	N-Term(iTRAQ8plex)	0.659	0.551	0.508	2.77	-	0	1394.79643	2	697.9018555	4.232171344	21.468975
21361657	PDIA3	protein disulfide-isomerase A3 precursor	NP_005304.3	49.50	22	22	219	505	56.7	6.35	0.810	0.575	0.611																
														TFSHELSDFGLESTAGEIPVVAIR	39	1	1	N-Term(iTRAQ8plex)	0.767	0.658	0.749	7.18	98	0	2879.50064	2	1440.260864	1.774811591	51.07354833
														KTFSHELSDFGLESTAGEIPVVAIR	8	1	1	N-Term(iTRAQ8plex); K1(iTRAQ8plex)	1.602	1.427	0.992	6.48	87	1	3311.80075	3	1104.605103	-2.692472607	46.52356167
														FIQENIFGICPHMTEDNKDLIQGK	4	1	1	N-Term(iTRAQ8plex); C10(Methylthio); K18(iTRAQ8plex); K24(iTRAQ8plex)	0.557	0.648	0.573	5.63	27	1	3748.95041	3	1250.321655	-3.326192218	50.99389167
														FISDKDASIVGFFDDSFSEAHSEFLK	4	1	1	N-Term(iTRAQ8plex); K5(iTRAQ8plex); K26(iTRAQ8plex)	0.748	1.599	-	5.26	38	1	3850.99999	6	642.6727295	1.462610526	58.7434
														VVVAENFDEIVNNENK	2	1	1	N-Term(iTRAQ8plex); K16(iTRAQ8plex)	0.651	0.427	0.564	5.22	65	0	2441.31113	3	814.4418945	-0.885162167	33.74848667
														MDATANDVPSPYEVR	10	1	1	N-Term(iTRAQ8plex)	0.758	0.558	0.595	5.18	66	0	1968.9655	2	984.9863892	0.812812775	28.75855667
														ELSDFISYLQR	50	1	1	N-Term(iTRAQ8plex)	0.809	0.551	0.588	5.11	69	0	1674.88895	3	558.9678345	-6.872773284	54.63751333
														DASIVGFFDDSFSEAHSEFLK	4	1	1	N-Term(iTRAQ8plex); K21(iTRAQ8plex)	1.463	0.973	0.655	5.08	30	0	2956.48476	4	739.8766479	0.744335605	59.29130333
														DLLIAYYDVDYEK	16	1	1	N-Term(iTRAQ8plex); K13(iTRAQ8plex)	0.891	0.631	0.653	4.81	57	0	2228.17966	2	1114.598511	-2.219934652	36.65455667
														GFPTIYFSPANK	16	1	1	N-Term(iTRAQ8plex); K12(iTRAQ8plex)	0.989	0.647	0.603	4.59	62	0	1950.08599	2	975.5466309	-4.382474063	57.92472833
														FLQDYFDGNLK	2	1	1	N-Term(iTRAQ8plex); K11(iTRAQ8plex)	0.671	0.419	0.449	4.45	52	0	1968.05974	2	984.5335083	-4.558458392	69.87701333
														TADGIVSHLK	14	1	1	N-Term(iTRAQ8plex); K10(iTRAQ8plex)	0.62	0.526	0.564	4.4	61	0	1648.97905	2	824.9931641	-3.153342061	28.57246167
														GFPTIYFSPANKK	3	1	1	N-Term(iTRAQ8plex); K12(iTRAQ8plex); K13(iTRAQ8plex)	0.892	0.484	0.652	4.33	-	1	2382.39223	4	596.3535156	-1.103378345	30.77389333
														FLQDYFDGNLKR	9	1	1	N-Term(iTRAQ8plex); K11(iTRAQ8plex)	0.942	0.626	0.682	4.05	44	1	2124.16289	2	1062.585083	-3.268065933	39.00357
														VDCTANTNTCNK	2	1	1	N-Term(iTRAQ8plex); C3(Methylthio); C10(Methylthio); K12(iTRAQ8plex)	0.673	0.464	0.508	4.02	59	0	1983.90813	2	992.4577026	-6.827254935	31.57421167
														FVMQEEFSR	14	1	1	N-Term(iTRAQ8plex)	0.836	0.545	0.561	3.9	51	0	1476.74393	3	492.9199524	-0.397747847	31.58060333
														YGVSGYPTLK	4	1	1	N-Term(iTRAQ8plex); K10(iTRAQ8plex)	0.871	0.694	0.678	3.84	31	0	1692.97356	2	846.9885254	-4.906844101	23.31067667
														DGEEAGAYDGPR	7	1	1	N-Term(iTRAQ8plex)	0.751	0.663	0.712	3.63	59	0	1540.71733	2	770.8623047	-0.537765742	15.17168167
														EATNPPVIQEEKPK	2	1	1	N-Term(iTRAQ8plex); K12(iTRAQ8plex); K14(iTRAQ8plex)	0.658	0.32	0.324	3.12	24	0	2492.44059	3	831.4850464	-3.284086244	19.02089
														LKGIVPLAK	4	1	1	N-Term(iTRAQ8plex); K2(iTRAQ8plex); K9(iTRAQ8plex)	1.454	1.107	0.769	2.78	18	1	1851.25661	4	463.5696106	0.411181685	26.30584833
														LAPEYEAAATR	2	1	1	N-Term(iTRAQ8plex)	0.888	0.57	0.509	-	38	0	1495.8001	2	748.4036865	-3.840669043	19.67921333
														LNFAVASR	1	1	1	N-Term(iTRAQ8plex)	0.601	0.461	0.405	-	32	0	1181.69304	2	591.3501587	-1.176732992	26.87415667
														FVMQEEFSR	2	1	1	N-Term(iTRAQ8plex); M3(Oxidation)	0.788	0.464	0.484	-	31	0	1492.73454	3	492.9199524	-0.397747847	31.58060333
21361659	IPO9	importin-9	NP_060555.2	1.15	1	1	1	1041	115.9	4.81	1.032	1.642	1.989																
														LLQHGINADDKR	1	1	1	N-Term(iTRAQ8plex); K11(iTRAQ8plex)	1.032	1.642	1.989	3.25	-	1	1988.13022	3	663.3815918	-9.832937077	15.57629167
21361794	CAND1	cullin-associated NEDD8-dissociated protein 1	NP_060918.2	5.77	6	6	12	1230	136.3	5.78	0.817	2.831	2.513																
														ITSEALLVTQQLVK	2	1	1	N-Term(iTRAQ8plex); K14(iTRAQ8plex)	1.018	3.305	2.938	4.94	33	0	2151.33139	3	717.7819824	4.86801599	46.95544833
														HEMLPEFYK	2	1	1	N-Term(iTRAQ8plex); K9(iTRAQ8plex)	0.817	1.633	1.775	3.81	27	0	1801.9603	3	601.3249512	-9.112872449	25.540705
														HTVDDGLDIR	2	1	1	N-Term(iTRAQ8plex)	1.178	1.357	1.338	3.8	39	0	1444.76474	3	482.2597656	-3.493769503	21.99035667
														MLTFLMLVR	2	1	1	N-Term(iTRAQ8plex)	0.391	3.461	3.137	3.25	31	0	1427.84416	2	714.4257202	1.479509978	64.81316667
														TLEDPDLNVR	3	1	1	N-Term(iTRAQ8plex)	1.022	2.839	2.715	2.98	24	0	1475.78887	2	738.3980713	-8.0603277	18.66445333
														IDLRPVLGEGVPILASFLR	1	1	1	N-Term(iTRAQ8plex)	0.717	2.831	1.971	2.82	-	0	2369.42319	3	790.479248	0.37544607	64.94352
21389315	SLC25A1	tricarboxylate transport protein, mitochondrial isoform a precursor	NP_005975.1	3.86	1	1	10	311	34.0	9.89	0.661	0.609	0.590																
														FGMFEFLSNHMR	8	2	1	N-Term(iTRAQ8plex)	0.601	0.604	0.565	5.4	65	0	1819.9105	3	607.3083496	9.837999269	56.78373167
														FGMFEFLSNHMR	2	2	1	N-Term(iTRAQ8plex); M11(Oxidation)	0.841	1.16	1.179	4.16	30	0	1835.88553	3	607.3083496	9.837999269	56.78373167
21464101	YWHAG	14-3-3 protein gamma	NP_036611.2	42.51	5	8	76	247	28.3	4.89	1.078	1.634	2.031																
														LGLALNYSVFYYEIQNAPEQACHLAK	17	1	1	N-Term(iTRAQ8plex); C22(Methylthio); K26(iTRAQ8plex)	-	-	-	7.26	63	0	3609.86606	3	1203.962891	1.287115519	66.36009333
														TAFDDAIAELDTLNEDSYK	8	1	1	N-Term(iTRAQ8plex); K19(iTRAQ8plex)	0.688	1.634	2.638	5.75	71	0	2739.36655	2	1370.197388	1.94841753	53.63552167
														NVTELNEPLSNEER	4	1	1	N-Term(iTRAQ8plex)	1.124	1.256	1.351	4.79	65	0	1947.9926	2	974.499939	0.025672422	26.68431667
														EHMQPTHPIR	2	1	1	N-Term(iTRAQ8plex)	1.7	2.816	1.973	4.23	38	0	1549.81399	4	388.2089539	-4.597810046	10.90657667
														NCSETQYESK	1	1	1	N-Term(iTRAQ8plex); C2(Methylthio); K10(iTRAQ8plex)	1.465	1.447	1.435	3.49	-	0	1842.86405	3	614.9595337	-9.856655353	17.37772333
														DSTLIMQLLR	24	9	7	N-Term(iTRAQ8plex)	-	-	-	4.73	54	0	1493.87629	3	498.6302795	6.656557269	46.36715833
														NLLSVAYK	16	10	7	N-Term(iTRAQ8plex); K8(iTRAQ8plex)	-	-	-	4.01	39	0	1515.92546	2	758.4644165	-9.192370231	25.08285833
														DSTLIMQLLR	2	9	7	N-Term(iTRAQ8plex); M6(Oxidation)	-	-	-	3.14	48	0	1509.85791	3	498.6302795	6.656557269	46.36715833
														VISSIEQK	2	5	5	N-Term(iTRAQ8plex); K8(iTRAQ8plex)	-	-	-	3.44	29	0	1511.91423	2	756.4607544	-7.341111487	22.45532333
21536301	STAT1	signal transducer and activator of transcription 1-alpha/beta isoform beta	NP_644671.1	13.76	7	7	14	712	83.0	6.42	0.941	1.742	1.700																
														FSLENNFLLQHNIR	2	3	1	N-Term(iTRAQ8plex)	1.324	1.742	1.7	4.83	50	0	2049.1102	3	683.708252	-3.976697112	40.68465167
														FHDLLSQLDDQYSR	3	3	1	N-Term(iTRAQ8plex)	0.818	1.671	1.759	4.81	41	0	2041.0228	3	681.0124512	-3.16068707	44.91071333
														ELSAVTFPDIIR	2	3	1	N-Term(iTRAQ8plex)	1.016	2.214	1.954	4.46	41	0	1664.94609	2	832.9766846	-3.861253769	47.48020167
														SLEDLQDEYDFK	2	3	1	N-Term(iTRAQ8plex); K12(iTRAQ8plex)	2.262	3.995	2.51	3.82	46	0	2110.07114	3	704.0285645	-4.232649059	56.20321167
														QDWEHAANDVSFATIR	1	3	1	N-Term(iTRAQ8plex)	0.623	0.764	0.865	3.52	-	0	2164.06784	3	722.0274658	-2.170490669	31.87539
														WAQLSEVLSWQFSSVTK	2	3	1	N-Term(iTRAQ8plex); K17(iTRAQ8plex)	-	-	-	3.36	48	0	2604.41874	3	868.8110962	-3.653781035	58.99033167
														GLNVDQLNMLGEK	2	3	1	N-Term(iTRAQ8plex); K13(iTRAQ8plex)	0.941	1.44	1.013	3.26	58	0	2039.1303	2	1020.068787	-5.538968172	55.087295
21624607	COTL1	coactosin-like protein	NP_066972.1	54.93	6	6	39	142	15.9	5.67	1.473	4.837	5.860																
														YDGSTIVPGEQGAEYQHFIQQCTDDVR	8	1	1	N-Term(iTRAQ8plex); C22(Methylthio)	-	4.837	5.86	5.59	55	0	3406.55344	3	1136.189331	-2.58395651	50.742655
														SKFALITWIGENVSGLQR	24	1	1	N-Term(iTRAQ8plex); K2(iTRAQ8plex)	2.242	4.116	5.562	5.59	72	1	2627.49956	2	1314.253418	-5.115883827	61.32173667
														DDGSAVIWVTFK	4	1	1	N-Term(iTRAQ8plex); K12(iTRAQ8plex)	1.156	9.546	8.369	4.7	52	0	1946.06523	2	973.5362549	-9.839420616	66.14961667
														ELEEDFIK	1	1	1	N-Term(iTRAQ8plex); K8(iTRAQ8plex)	0.986	3.712	5.408	-	53	0	1630.8991	2	815.953186	-9.654705022	39.518825
														EFVISDR	1	1	1	N-Term(iTRAQ8plex)	1.473	3.366	4.606	-	24	0	1169.64067	2	585.3239746	-5.24836507	22.53588
														LFAFVR	1	1	1	N-Term(iTRAQ8plex)	1.833	10.432	14.533	-	22	0	1056.64263	2	528.8249512	-7.708922631	64.62946167
21626466	MATR3	matrin-3 isoform a	NP_061322.2	21.84	16	16	93	847	94.6	6.25	1.249	0.816	0.704																
														GDADQASNILASFGLSAR	4	1	1	N-Term(iTRAQ8plex)	1.149	0.721	0.521	6.07	80	0	2097.09453	2	1049.050903	3.175212433	46.72375333
														LCSLFYTNEEVAK	14	3	1	N-Term(iTRAQ8plex); C2(Methylthio); K13(iTRAQ8plex)	1.18	0.706	0.579	5.36	84	0	2171.12724	2	1086.067261	-2.974231768	45.36297333
														DLSAAGIGLLAAATQSLSMPASLGR	2	1	1	N-Term(iTRAQ8plex)	-	-	-	5.22	59	0	2675.47263	3	892.4957275	0.806611573	66.58042

														EDAMAMVDHCLK	16	3	1	N-Term(iTRAQ8plex); C10(Methylthio); K12(iTRAQ8plex)	1.245	0.833	0.719	5.08	43	0	2016.97757	3	672.9937744	-8.33544115	42.110465
														YQLLQLVEPFGVISNHLILNK	6	3	1	N-Term(iTRAQ8plex); K21(iTRAQ8plex)	0.971	-	0.342	5.02	35	0	3046.79789	4	762.4517822	-2.002856503	66.0752
														ITPENLPQILLQLK	9	1	1	N-Term(iTRAQ8plex); K14(iTRAQ8plex)	1.587	0.862	0.903	4.66	40	0	2228.39463	3	743.4683228	2.94100696	53.25123833
														GPSLNPVLDYDHGSR	2	1	1	N-Term(iTRAQ8plex)	1.197	1.357	1.592	4.22	39	0	1930.98886	3	644.3344727	-1.872916678	24.24491333
														TEEGPTLSYGR	6	1	1	N-Term(iTRAQ8plex)	1.508	0.787	0.734	3.93	44	0	1513.77568	2	757.3914795	-2.859694257	15.26738167
														GAPPSSNIEDFHGLLPK	5	1	1	N-Term(iTRAQ8plex); K17(iTRAQ8plex)	1.455	0.828	0.825	3.84	26	0	2387.31296	4	597.583252	-2.849803395	32.93231
														SQAFIEMETR	5	3	1	N-Term(iTRAQ8plex)	1.338	0.832	0.639	3.79	50	0	1515.78349	2	758.3953857	3.677018779	29.62623333
														DLDELSRYPEDK	3	1	1	N-Term(iTRAQ8plex); K12(iTRAQ8plex)	1.353	0.663	0.631	3.78	30	1	2088.11211	2	1044.559692	2.464832039	24.433685
														EDAMAMVDHCLKK	2	3	1	N-Term(iTRAQ8plex); C10(Methylthio); K12(iTRAQ8plex); K13(iTRAQ8plex)	2.976	1.111	1.578	3.62	25	1	2449.28213	4	613.0759888	-0.72964313	38.96987333
														DSFDDRGPSLNPVLDYDHGSR	2	1	1	N-Term(iTRAQ8plex)	0.43	-	0.661	3.59	21	1	2666.26821	4	667.3225098	-2.509919098	29.59791833
														VVHIMDFQR	10	3	1	N-Term(iTRAQ8plex)	1.19	0.828	0.763	3.58	46	0	1448.79114	3	483.6018982	-5.142197318	25.75640167
														ITPENLPQILLQLKR	1	1	1	N-Term(iTRAQ8plex); K14(iTRAQ8plex)	-	-	-	3.52	-	1	2384.48818	4	596.8775024	1.341413915	54.60736
														EDAMAMVDHCLK	1	3	1	N-Term(iTRAQ8plex); M4(Oxidation); C10(Methylthio); K12(iTRAQ8plex)	0.985	0.692	0.656	3.28	-	0	2032.96506	3	672.9937744	-8.33544115	42.110465
														VVHIMDFQR	4	3	1	N-Term(iTRAQ8plex); M5(Oxidation)	1.075	0.625	0.722	2.72	34	0	1464.78733	3	483.6018982	-5.142197318	25.75640167
														DLDELSR	1	1	1	N-Term(iTRAQ8plex)	0.672	0.468	0.483	-	24	0	1151.61907	2	576.3131714	-1.671686948	20.11903833
21700763	HN1L	hematological and neurological expressed 1-like protein	NP_653171.1	7.37	1	1	6	190	20.1	9.26	0.817	1.280	1.280																
														EQEPMPTVDSHEPR	4	1	1	N-Term(iTRAQ8plex)	0.89	0.95	1.183	4.17	52	0	1955.94065	3	652.6517334	-1.459325819	16.03778167
														EQEPMPTVDSHEPR	2	1	1	N-Term(iTRAQ8plex); M5(Oxidation)	0.817	1.28	1.28	3.72	38	0	1971.93399	3	652.6517334	-1.459325819	16.03778167
21735621	MDH2	malate dehydrogenase, mitochondrial isoform 1 precursor	NP_005909.2	61.24	16	16	218	338	35.5	8.68	0.861	0.823	0.794																
														LTLYDIAHTPGVAADLSHIETK	28	2	1	N-Term(iTRAQ8plex); K22(iTRAQ8plex)	0.813	0.837	0.784	6.44	55	0	2973.64487	3	991.8864746	-1.930533781	45.222955
														AGAGSATLSMAYAGAR	6	3	1	N-Term(iTRAQ8plex)	0.905	0.859	0.714	6.09	78	0	1758.91667	2	879.9619751	3.179386151	27.828765
														FVFSLVDAMNGK	14	3	1	N-Term(iTRAQ8plex); K12(iTRAQ8plex)	0.714	0.74	0.686	5.64	60	0	1936.07883	2	968.5423584	-2.500480107	48.995605
														VAVLGASGGIGQPLSLLLK	18	2	1	N-Term(iTRAQ8plex); K19(iTRAQ8plex)	1.17	0.793	0.646	5.53	75	0	2401.49649	2	1201.252319	-1.23225125	54.54948667
														VNVPVIGGHAGK	22	3	1	N-Term(iTRAQ8plex); K12(iTRAQ8plex)	0.888	0.799	0.761	5.37	62	0	1756.0623	2	878.53479	-3.814125422	22.24566
														TIIPLISQCTPK	14	3	1	N-Term(iTRAQ8plex); C9(Methylthio); K12(iTRAQ8plex)	0.845	0.806	0.786	5.11	49	0	1968.14408	3	656.7195435	-2.111216833	49.18309333
														GYLGPEQLPDCLK	25	2	1	N-Term(iTRAQ8plex); C11(Methylthio); K13(iTRAQ8plex)	0.803	0.804	0.842	4.55	53	0	2087.10478	2	1044.056641	-3.15157447	41.46245167
														SQETECTYFSTPLLLGK	13	3	1	N-Term(iTRAQ8plex); C6(Methylthio); K17(iTRAQ8plex)	0.796	1.331	1.032	4.53	60	0	2571.32646	2	1286.16687	-1.185647046	49.58037667
														EGVVECSFVK	18	3	1	N-Term(iTRAQ8plex); C6(Methylthio); K10(iTRAQ8plex)	0.781	0.83	0.76	4.47	63	0	1750.9207	2	875.9639893	-6.922303427	49.43594833
														GCDVVVIPAGVPR	14	2	1	N-Term(iTRAQ8plex); C2(Methylthio)	0.906	0.83	0.789	4.44	63	0	1631.88494	2	816.4508057	1.717273028	35.505215
														IFGVTTLDIVR	12	2	1	N-Term(iTRAQ8plex)	0.92	0.72	0.784	4.43	78	0	1537.92386	3	513.3148804	2.936780623	44.86988167
														ANTFVAELK	7	2	1	N-Term(iTRAQ8plex); K9(iTRAQ8plex)	0.911	0.854	0.908	4.39	40	0	1600.93668	3	534.3170776	-9.501712364	31.1682
														VDFPQDQLTALTGR	8	3	1	N-Term(iTRAQ8plex)	0.95	0.869	0.807	4.37	59	0	1865.002	2	933.0046387	-2.724166648	42.25693
														IQEAGTEVVK	4	3	1	N-Term(iTRAQ8plex); K10(iTRAQ8plex)	0.784	0.685	0.658	4.21	60	0	1681.98894	2	841.4981079	-3.300913317	17.14740667
														MISDAIPELK	8	3	1	N-Term(iTRAQ8plex); K10(iTRAQ8plex)	0.854	0.841	0.793	3.72	42	0	1725.00566	2	863.0064697	-1.187513131	57.46078
														MISDAIPELK	2	3	1	N-Term(iTRAQ8plex); M1(Oxidation); K10(iTRAQ8plex)	0.823	0.815	0.715	3.31	41	0	1740.99004	2	863.0064697	-1.187513131	57.46078
														AGAGSATLSMAYAGAR	2	3	1	N-Term(iTRAQ8plex); M10(Oxidation)	0.923	0.94	0.852	2.97	71	0	1774.903	2	879.9619751	3.179386151	27.828765
														VSSFEEK	3	3	1	N-Term(iTRAQ8plex); K7(iTRAQ8plex)	0.931	0.769	0.776	2.86	29	0	1433.81192	2	717.4024658	-8.352837887	15.5329
21914862	CECR5	cat eye syndrome critical region protein 5 isoform 1	NP_060299.4	10.18	2	2	10	393	43.6	6.90	1.084	0.616	0.754																
														AQELSALLGCEVDADQVILSHSPMK	2	2	1	N-Term(iTRAQ8plex); C10(Methylthio); K25(iTRAQ8plex)	-	-	-	5.29	34	0	3308.71792	4	827.9349365	0.883699918	65.739565
														ATHDGAPELGAGGTR	8	6	1	N-Term(iTRAQ8plex)	1.084	0.616	0.754	3.8	47	0	1713.88089	3	571.963562	-3.552482427	11.60918833
22027655	AP1S2	AP-1 complex subunit sigma-2 isoform 2	NP_003907.3	5.10	1	1	2	157	18.6	5.47	0.613	1.942	1.974																
														MCSFLEWR	2	6	1	N-Term(iTRAQ8plex); C2(Methylthio)	0.613	1.942	1.974	-	32	0	1421.65764	2	711.3324585	-7.408202589	78.49298
22035588	MRPL24	39S ribosomal protein L24, mitochondrial	NP_078816.2	6.02	1	1	2	216	24.9	9.29	0.923	0.505	0.585																
														TLQEEVMEAMGIK	2	1	1	N-Term(iTRAQ8plex); K13(iTRAQ8plex)	0.923	0.505	0.585	3.66	35	0	2087.12674	3	696.3804321	-3.353013086	43.90405667
22035594	MRPL35	39S ribosomal protein L35, mitochondrial isoform b	NP_663619.1	4.12	1	1	1	170	19.2	11.80	0.939	1.078	1.094																
														EFVFCNK	1	3	1	N-Term(iTRAQ8plex); C5(Methylthio); K7(iTRAQ8plex)	0.939	1.078	1.094	2.8	-	0	1540.80559	2	770.9064331	-3.66218107	31.54281167
22035634	MGST1	microsomal glutathione S-transferase 1 isoform a	NP_665707.1	27.10	4	4	34	155	17.6	9.39	0.331	0.643	0.657																
														IYHTIAYLTPLPQPNR	10	1	1	N-Term(iTRAQ8plex)	0.518	0.845	0.832	5.56	61	0	2201.23514	3	734.4234619	7.896034587	42.65047167
														VFANPEDCVAFGK	6	6	1	N-Term(iTRAQ8plex); C8(Methylthio); K13(iTRAQ8plex)	0.097	0.649	0.572	5.08	61	0	2051.03765	2	1026.022461	-8.501572382	36.24467167
														KVFANPEDCVAFGK	6	6	1	N-Term(iTRAQ8plex); K1(iTRAQ8plex); C9(Methylthio); K14(iTRAQ8plex)	0.434	0.802	0.658	5.06	51	1	2483.34006	3	828.4515381	-6.18171165	36.378775
														MMLMSTATAFYR	10	6	1	N-Term(iTRAQ8plex)	0.111	0.473	0.468	4.39	49	0	1726.86052	3	576.2945557	3.389047926	49.68564833
														IYHTIAYLTPLPQPNR	2	1	1	N-Term(iTRAQ8plex); Y2(iTRAQ8plex)	0.231	0.568	0.718	-	46	0	2505.45902	3	734.4234619	7.896034587	42.65047167
22202629	AIFM1	apoptosis-inducing factor 1, mitochondrial isoform AIF-exB precursor	NP_665811.1	18.56	8	8	21	609	66.3	8.94	0.934	0.715	0.819																
														KVETDHIVAAVGLEPNVELAK	1	3	1	N-Term(iTRAQ8plex); K1(iTRAQ8plex); K21(iTRAQ8plex)	-	-	-	4.81	-	1	3144.81885	5	629.7695923	-6.603364193	34.98786333
														APSHVPFLLIGGGTAAFAAAR	5	3	1	N-Term(iTRAQ8plex)	0.545	0.839	0.988	4.75	57	0	2328.31565	3	776.7767334	1.101931912	46.22465
														VLIVSEDPELPYMRPPLSK	4	3	1	N-Term(iTRAQ8plex); K19(iTRAQ8plex)	1.018	0.939	0.898	4.28	27	0	2791.58999	4	698.6529541	0.395912815	44.93051167
														VLIVSEDPELPYMRPPLSK	2	3	1	N-Term(iTRAQ8plex); M13(Oxidation); K19(iTRAQ8plex)	-	-	-	4.1	26	0	2807.57607	4	698.6529541	0.395912815	44.93051167
														TGGLEIDSDFGGFR	2	3	1	N-Term(iTRAQ8plex)	0.919	0.685	0.847	4.08	48	0	1774.88567	2	887.9464722	-3.224762116	39.04513833
														VNAELQAR	2	3	1	N-Term(iTRAQ8plex)	0.852	0.569	0.787	3.81	48	0	1204.69791	3	402.2374878	2.281757844	20.3842
														ELWFSDDPNVTK	2	3	1	N-Term(iTRAQ8plex); K12(iTRAQ8plex)	0.947	0.723	0.725	3.8	37	0	2059.08432	3	687.032959	-5.510991825	51.57353667
														GVIFYLR	2	3	1	N-Term(iTRAQ8plex)	0.79	0.519	0.625	2.69	27	0	1171.71184	2	586.3574219	-5.56808728	27.56150167
														SATEQSGTGIR	1	3	1	N-Term(iTRAQ8plex)	1.204	0.854	1.009	-	23	0	1410.74578	2	705.8765259	-2.322290979	12.789375
22202633	PFDN5	prefoldin subunit 5 isoform alpha	NP_002615.2	11.69	1	1	2	154	17.3	6.33	-	-	-																
														NQLDQEVEFLSTSIAQLK	2	1	1	N-Term(iTRAQ8plex); K18(iTRAQ8plex)	-	-	-	3.69	42	0	2671.47727	3	891.1639404	0.350644594	54.92074167
22212934	SIGMAR1	sigma non-opioid intracellular receptor 1 isoform 2	NP_671513.1	16.15	3	3	6	192	21.5	5.99	0.971	0.691	0.639																
														QYAGLDHELAFSR	2	5	1	N-Term(iTRAQ8plex)	1.129	1.106	1.117	4.15	44	0	1810.9354	3	604.3166504	-1.978744316	27.36275
														LELTTYLFGQDP	1	4	1	N-Term(iTRAQ8plex)	1.151	0.691	0.926	-	34	0	1700.89836	2	850.9528198	-3.83225224	81.68404
														LIVELR	3	6	1	N-Term(iTRAQ8plex)	0.953	0.368	0.374	-	33	0	1046.68682	2	523.8470459	-0.70328881	32.50573833
22219465	NUDT2	NUDT2 gene product	NP_671701.1	10.20	1	1	2	147	16.8	5.35	1.058	0.914	0.893																
														GHVEPGEDDLETALR	2	1	1	N-Term(iTRAQ8plex)	1.058	0.914	0.893	3.96	45	0	1941.97629	3	647.9969482	-2.940212742	23.28126
22267436	NIPSNAP3A	protein NipSnap homolog 3A	NP_056284.1	13.36	3	3	4	247	28.4	9.16	0.828	0.384	0.500																
														LVGVFHTEYGALNR	1	1	1	N-Term(iTRAQ8plex)	0.582	0.384	0.57	3.21	-	0	1880.02677	3	627.3471069	-3.432796453	36.453445
														MNEFLENFEK	2	1	1	N-Term(iTRAQ8plex); K10(iTRAQ8plex)	0.958	0.324	0.474	3.16	24	0	1908.99295	3	637.0025024	-2.958222433	69.61857
														MNTVFHIWK	1	1	1	N-Term(iTRAQ8plex); K9(iTRAQ8plex)	0.828	0.54	0.5	2.97	-	0	1784.01194	3	595.3421631	-1.034349687	37.48955667
22538442	CTSZ	cathepsin Z preproprotein	NP_001327.2	5.61	1	1	2	303	33.8	7.11	0.177	0.218	0.175																
														DQECDKFNQCGTCNEFK	2	1	1	N-Term(iTRAQ8plex); C4(Methylthio); K6(iTRAQ8plex); C10(Methylthio); [illegible]	0.177	0.218	0.175	4.06	36	1	3059.35415	4	765.5939941	-2.797197411	36.79158
22538465	PSMB3	proteasome subunit beta type-3	NP_002786.2	21.95	4	4	13	205	22.9	6.55	0.931	1.222	1.302																
														FGPYYTEPVIAGLDPK	7	1	1	N-Term(iTRAQ8plex); K16(iTRAQ8plex)	0.862	1.091	1.232	5.52	47	0	2375.31223	2	1188.165649	5.586988555	41.70433333
														QIKPYTLMSMVANLLYEKR	2	1	1	N-Term(iTRAQ8plex); K3(iTRAQ8plex); K18(iTRAQ8plex)	-	-	-	4.81	31	1	3210.8534	5	642.9765015	0.737970229	63.57945333
														NCVAIAADRR	2	1	1	N-Term(iTRAQ8plex); C2(Methylthio)	1.384	2.238	2.033	2.84	32	1	1438.74936	3	480.2546387	-4.670399549	22.58734
														NCVAIAADR	2	1	1	N-Term(iTRAQ8plex); C2(Methylthio)	1.006	1.392	1.164	-	25	0	1282.64885	2	641.828064	-4.76435176	24.29463667
22538467	PSMB4	proteasome subunit beta type-4	NP_002787.2	26.52	4	4	8	264	29.2	5.97	1.266	2.413	2.242																
														QVLGQMVIDEELLGDGHSYSPR	2	1	1	N-Term(iTRAQ8plex)	-	1.514	1.479	7.81	60	0	2747.3992	3	916.4712524	0.557900074	47.15545833
														VNNSTMLGASGDYADFQYLK	2	1	1	N-Term(iTRAQ8plex); K20(iTRAQ8plex)	0.861	3.65	3.354	4.01	81	0	2802.41875	2	1401.713013	-1.485334544	39.17137167
														FEGGVVIAADMLGSYGSLAR	3	1	1	N-Term(iTRAQ8plex)	2.864	5.37	5.843	3.47	52	0	2317.22129	3	773.0786133	2.082362085	65.83877333
														AIHSWLTR	1	1	1	N-Term(iTRAQ8plex)	1.266	1.595	1.499	2.84	-	0	1287.73935	3	429.9179688	-6.343131458	22.98402333
22547114	MRPL10	39S ribosomal protein L10, mitochondrial isoform a precursor	NP_660298.2	5.75	1	1	2	261	29.3	9.58	0.872	0.613	0.569																
														MIAVCQNVALSAEDK	2	2	1	N-Term(iTRAQ8plex); C5(Methylthio); K15(iTRAQ8plex)	0.872	0.613	0.569	4.76	38	0	2246.17417	3	749.3962402	-2.762406836	47.56680833
22547134	MRPL37	39S ribosomal protein L37, mitochondrial	NP_057575.2	15.13	4	4	9	423	48.1	8.59	1.055	0.719	0.669																
														NLAWVDSDQLLYQHFWCLPVIK	4	2	1	N-Term(iTRAQ8plex); C17(Methylthio); K22(iTRAQ8plex)	1.327	0.965	0.316	5.5	52	0	3342.77378	3	1114.929443	3.274085791	65.58663833
														VYEIPGLEPITFAGK	2	2	1	N-Term(iTRAQ8plex); K15(iTRAQ8plex)	0.966	0.513	0.669	4.56	62	0	2242.31059	2	1121.658936	7.230565556	45.79364667
														NHIENQDECVLNVISHAR	2	2	1	N-Term(iTRAQ8plex); C9(Methylthio)	-	-	-	3.14	26	0	2441.19301	4	611.0537109	-1.539587398	34.26801833
														VLSLVDDPR	1	2	1	N-Term(iTRAQ8plex)	0.885	0.719	0.879	-	47	0	1317.76067	2	659.3839722	-5.56518664	37.37318
22547140	MRPL4	39S ribosomal protein L4, mitochondrial isoform b	NP_666500.1	25.48	4	4	11	263	29.5	10.13	1.005	0.842	0.719																
														VGLADLHPDVFATAPR	6	3	1	N-Term(iTRAQ8plex)	1.228	0.842	0.678	4.07	39	0	1983.09482	3	661.703125	-0.887274769	35.7039
														LDILHQVAMWQK	2	3	1	N-Term(iTRAQ8plex); K12(iTRAQ8plex)	0.876	1.083	0.961	3.63	27	0	2090.189	3	697.4011841	-7.234897855	48.605865
														LAQDDLHIMDSLELPTGDPQYLTELAHYR	2	3	1	N-Term(iTRAQ8plex)	-	-	-	3.31	26	0	3658.84829	4	915.4675293	3.202102569	58.60309667
														KVELPVPTHR	1	3	1	N-Term(iTRAQ8plex); K1(iTRAQ8plex)	0.714	0.835	0.749	3.19	-	1	1784.0862	4	446.7770081	-7.897013843	17.45244833
22748853	C4orf32	uncharacterized protein C4orf32	NP_689613.1	12.88	1	1	1	132	14.7	4.65	0.948	0.576	0.452																
														GGDGGERDEDGDALAER	2	1	1	N-Term(iTRAQ8plex)	0.948	0.576	0.452	2.72	19	1	2022.93033	3	674.9816284	1.794774565	11.62025833
22748937	XPO5	exportin-5	NP_065801.1	1.41	1	1	2	1204	136.2	5.80	1.792	3.466	2.692																
														VLSDVDAFIAYVGTDQK	2	1	1	N-Term(iTRAQ8plex); K17(iTRAQ8plex)	1.792	3.466	2.692	5.49	38	0	2449.35294	3	817.1224976	3.8451485	64.224305
22749415	STT3A	dolichyl-diphosphooligosaccharide--protein glycosyltransferase subunit STT3A isoform a	NP_689926.1	13.33	8	8	28	705	80.5	8.07	0.932	0.484	0.694																
														DFELDVLEEAYTTEHWLVR	2	2	1	N-Term(iTRAQ8plex)	1.24	1.155	1.143	6.13	68	0	2669.34592	3	890.4534912	2.172473327	64.67708833
														NAEIGNKDFELDVLEEAYTTEHWLVR	4	2	1	N-Term(iTRAQ8plex); K7(iTRAQ8plex)	-	-	-	6.09	29	1	3699.92689	5	740.7911987	4.139498647	59.63693667
														FYSLLDPSYAK	2	2	1	N-Term(iTRAQ8plex); K11(iTRAQ8plex)	0.796	0.321	0.505	4.34	35	0	1912.05657	2	956.5319214	-5.787039337	69.63712167
														EGSPVLLNCLMYK	2	2	1	N-Term(iTRAQ8plex); C9(Methylthio); K13(iTRAQ8plex)	1.137	1.417	0.989	4.33	32	0	2121.12503	3	707.7131958	-5.496209544	42.42959
														FLAEEGFYK	6	1	1	N-Term(iTRAQ8plex); K9(iTRAQ8plex)	0.845	0.454	0.643	4.15	44	0	1711.94438	2	856.4758301	-4.186913908	54.93584333
														FESVIHEFDPYFNYR	4	1	1	N-Term(iTRAQ8plex)	1.23	0.652	0.713	3.79	27	0	2267.11411	3	756.3762207	2.914524394	63.35793
														ENDYYTPTGEFR	4	2	1	N-Term(iTRAQ8plex)	0.827	0.447	0.649	3.79	44	0	1795.83452	2	898.4208984	-5.312875375	19.16363167
														FHNWFDDR	4	1	1	N-Term(iTRAQ8plex)	0.867	0.793	0.837	3.72	60	0	1440.69257	3	480.9023743	-2.534716526	28.030805
														LFAVIR	3	3	2	N-Term(iTRAQ8plex)	-	-	-	-	23	0	1022.66417	2	511.8357239	-2.20029816	29.57904667
22749443	MICU2	calcium uptake protein 2, mitochondrial	NP_689939.1	3.00	1	1	2	434	49.6	9.09	0.490	0.448	0.826																
														DFLFSVMFEQMER	2	1	1	N-Term(iTRAQ8plex)	0.49	0.448	0.826	3.92	23	0	1982.97685	2	991.9920654	5.55878368	61.96664333
22902130	PTK7	inactive tyrosine-protein kinase 7 isoform d precursor	NP_690621.1	3.35	2	2	3	1014	112.2	7.05	0.672	1.112	0.627																

														ADGSSLPEWVTDNAGTLHFAR	2	3	1	N-Term(iTRAQ8plex)	0.458	1.008	0.516	5.82	58	0	2548.27439	3	850.0963135	0.367260171	42.77147333
														INSVEVYDGTWYR	1	5	1	N-Term(iTRAQ8plex)	0.986	1.227	0.761	3.31	-	0	1905.97698	2	953.4921265	6.361571959	36.45534333
22907039	APOBEC3C	DNA dC-_dU-editing enzyme APOBEC-3C	NP_055323.2	36.32	4	5	17	190	22.8	7.59	0.977	0.989	0.743																
														YCWENFVYNDNEPFKPWK	4	1	1	N-Term(iTRAQ8plex); C2(Methylthio); K15(iTRAQ8plex); K18(iTRAQ8plex)	0.458	0.576	0.541	4.31	31	0	3337.66543	4	835.171814	2.287131393	52.77818667
														NQVDSETHCHAER	2	1	1	N-Term(iTRAQ8plex); C9(Methylthio)	0.977	1.11	0.769	3.88	62	0	1875.8375	3	625.9506836	-0.247765998	11.10659167
														LYYFQYPCYQEGLR	4	1	1	N-Term(iTRAQ8plex); C8(Methylthio)	1.14	1.231	1.233	3.78	43	0	2193.04326	2	1097.025269	-0.826162738	53.10634
														AMYPGTFYFQFK	2	1	1	N-Term(iTRAQ8plex); K12(iTRAQ8plex)	0.624	0.881	0.718	3.32	37	0	2108.11382	2	1054.560547	0.126098505	69.77889167
														HSNVNLTIFTAR	5	6	2	N-Term(iTRAQ8plex)	-	-	-	3.9	42	0	1676.93277	3	559.6491089	-3.463400522	34.00310667
22907044	APOBEC3F	DNA dC-_dU-editing enzyme APOBEC-3F isoform a	NP_660341.2	8.04	1	2	7	373	45.0	7.23	-	-	-																
														YCWENFVYNDDEPFKPWK	2	2	1	N-Term(iTRAQ8plex); C2(Methylthio); K15(iTRAQ8plex); K18(iTRAQ8plex)	-	-	-	3.27	31	0	3338.65859	4	835.420105	5.025294268	54.89522833
														HSNVNLTIFTAR	5	6	2	N-Term(iTRAQ8plex)	-	-	-	3.9	42	0	1676.93277	3	559.6491089	-3.463400522	34.00310667
23110925	PSMB6	proteasome subunit beta type-6 isoform 1 precursor	NP_002789.1	13.81	2	2	6	239	25.3	4.92	0.613	2.222	2.421																
														EGMTKEECLQFTANALALAMER	4	1	1	N-Term(iTRAQ8plex); K5(iTRAQ8plex); C8(Methylthio)	0.69	3.757	4.588	4.06	45	1	3110.56601	3	1037.526855	1.800590705	64.56473
														LAAIAESGVER	2	1	1	N-Term(iTRAQ8plex)	0.546	1.314	1.277	2.69	60	0	1419.80803	2	710.4076538	-2.049786132	33.69795333
23110939	PSMA3	proteasome subunit alpha type-3 isoform 2	NP_687033.1	10.48	2	2	20	248	27.6	5.33	1.066	1.575	1.408																
														HVGMAVAGLLADAR	9	2	1	N-Term(iTRAQ8plex)	0.984	2.506	2.347	4.52	71	0	1684.93888	3	562.3178101	-4.869241449	33.894035
														HVGMAVAGLLADAR	6	2	1	N-Term(iTRAQ8plex); M4(Oxidation)	1.154	1.338	1.124	3.77	59	0	1700.93314	3	562.3178101	-4.869241449	33.894035
														AVENSSTAIGIR	5	2	1	N-Term(iTRAQ8plex)	1.01	3.296	3.263	3.64	59	0	1521.85599	2	761.4276733	-3.805399781	21.35851667
23110942	PSMA5	proteasome subunit alpha type-5 isoform 1	NP_002781.2	22.41	4	4	35	241	26.4	4.79	0.753	2.534	2.454																
														LFQVEYAIEAIK	24	1	1	N-Term(iTRAQ8plex); K12(iTRAQ8plex)	0.669	2.081	2.355	5.7	80	0	2032.18889	2	1016.598083	-2.475620173	52.14096833
														ITSPLMEPSSIEK	2	1	1	N-Term(iTRAQ8plex); K13(iTRAQ8plex)	0.825	3.903	2.891	5.26	52	0	2040.15019	2	1020.578735	-0.273125418	31.93581167
														GPQLFHMDPSGTFVQCDAR	4	2	1	N-Term(iTRAQ8plex); C16(Methylthio)	1.106	2.736	2.173	4.98	44	0	2456.13901	3	819.3845215	-2.984805356	40.89483333
														GPQLFHMDPSGTFVQCDAR	4	2	1	N-Term(iTRAQ8plex); M7(Oxidation); C16(Methylthio)	0.823	2.62	2.454	4.44	37	0	2472.13547	3	819.3845215	-2.984805356	40.89483333
														GVNTFSPEGR	1	1	1	N-Term(iTRAQ8plex)	0.408	0.754	0.931	2.91	-	0	1367.71465	2	684.3609619	-5.457345865	18.11711833
23110944	PSMA6	proteasome subunit alpha type-6 isoform a	NP_002782.1	30.49	6	6	27	246	27.4	6.76	0.925	2.662	2.184																
														ILTEAEIDAHLVALAERD	6	3	1	N-Term(iTRAQ8plex)	1.104	4.475	3.925	5.29	78	1	2283.25357	3	761.7560425	1.626140631	47.90859833
														LYQVEYAFK	4	1	1	N-Term(iTRAQ8plex); K9(iTRAQ8plex)	0.734	1.693	1.985	4.37	42	0	1769.00003	3	590.3381958	-5.283859512	40.84529
														LLDSSTVTHLFK	4	2	1	N-Term(iTRAQ8plex); K12(iTRAQ8plex)	1.259	7.024	5.63	4.23	29	0	1969.13918	4	493.0402527	-9.486601046	38.25009167
														AINQGGLTSVAVR	2	2	1	N-Term(iTRAQ8plex)	1.492	1.216	1.419	3.86	56	0	1589.9271	3	530.6472168	-0.379991873	26.64738167
														YGYEIPVDMLCK	6	3	1	N-Term(iTRAQ8plex); C11(Methylthio); K12(iTRAQ8plex)	0.925	2.662	2.423	3.73	35	0	2085.07083	2	1043.030151	-7.149067884	42.01193667
														CDPAGYYCGFK	5	3	1	N-Term(iTRAQ8plex); C1(Methylthio); C8(Methylthio); K11(iTRAQ8plex)	0.88	2.96	2.101	3.66	32	0	1923.8532	2	962.4302368	-9.874083541	67.40947
23199983	PIGK	GPI-anchor transamidase precursor	NP_005473.1	5.32	1	1	2	395	45.2	6.16	1.171	0.555	0.756																
														LGIPDSHIVLMLADDMACNPR	2	1	1	N-Term(iTRAQ8plex); C18(Methylthio)	1.171	0.555	0.756	5.18	26	0	2631.31327	3	877.7759399	2.392065703	61.45582167
23238188	TES	testin isoform 2	NP_690042.1	2.43	1	1	2	412	46.9	7.59	0.669	2.513	1.816																
														CEGFELHFWR	2	2	1	N-Term(iTRAQ8plex); C1(Methylthio)	0.669	2.513	1.816	4.22	37	0	1673.78684	3	558.6004639	-0.121848513	45.956675
23308577	PHGDH	D-3-phosphoglycerate dehydrogenase	NP_006614.2	29.08	12	12	45	533	56.6	6.71	0.984	7.039	3.424																
														ALVDHENVISCPHLGASTK	3	5	1	N-Term(iTRAQ8plex); C11(Methylthio); K19(iTRAQ8plex)	1.166	3.897	1.905	6.22	26	0	2645.38089	3	882.4651489	-7.227228677	31.382835
														AGTGVDNVDLEAATR	4	4	1	N-Term(iTRAQ8plex)	0.785	9.637	4.15	5.74	87	0	1792.93022	2	896.96875	-2.285599265	24.27499667
														NAGNCLSPAVIVGLLK	6	5	1	N-Term(iTRAQ8plex); C5(Methylthio); K16(iTRAQ8plex)	0.969	2.684	1.849	4.67	64	0	2223.27861	2	1112.142944	-1.255015053	50.62158333
														ALQSGQCAGAALDVFTEEPPRDR	5	5	1	N-Term(iTRAQ8plex); C7(Methylthio)	2.596	4.057	6.063	4.48	29	1	2781.35805	4	696.0949707	-0.794348465	42.47392667
														CGEEIAVQFVDMVK	6	5	1	N-Term(iTRAQ8plex); C1(Methylthio); K14(iTRAQ8plex)	1.055	9.071	4.146	4.4	45	0	2222.13718	2	1111.573853	-3.412854461	51.85057667
														KVLISDSLDPCCR	7	2	1	K1(iTRAQ8plex); N-Term(iTRAQ8plex); C11(Methylthio); C12(Methylthio)	0.837	6.516	2.93	3.94	37	1	2149.10263	3	717.0379639	-4.88341709	41.92834667
														VTADVINAAEK	1	4	1	N-Term(iTRAQ8plex); K11(iTRAQ8plex)	0.825	2.671	1.579	3.88	-	0	1738.99857	3	580.3377075	-10.00115337	36.36371
														GGIVDEGALLR	4	5	1	N-Term(iTRAQ8plex)	0.888	7.126	3.429	3.5	52	0	1403.81145	2	702.4093628	-3.264209069	30.636425
														VLISDSLDPCCR	2	2	1	N-Term(iTRAQ8plex); C10(Methylthio); C11(Methylthio)	0.817	7.022	3.473	3.07	26	0	1716.80327	2	858.9052734	-3.630611094	51.09535167
														DLPLLLFR	3	5	1	N-Term(iTRAQ8plex)	1.143	13.673	5.582	3	54	0	1290.79804	3	430.9375305	-8.291767533	41.27920333
														EELIAELQDCEGLIVR	2	2	1	N-Term(iTRAQ8plex); C10(Methylthio)	-	-	-	2.9	51	0	2180.11795	3	727.3775024	-3.012784592	67.948585
														TLGILGLGR	2	5	1	N-Term(iTRAQ8plex)	1.383	5.426	2.635	-	29	0	1203.76885	2	602.3880615	-3.194029432	31.90988833
23308607	HM13	minor histocompatibility antigen H13 isoform 1	NP_110416.1	5.84	2	2	7	377	41.5	6.43	1.147	0.675	1.085																
														LVFPQDLLEK	2	3	1	N-Term(iTRAQ8plex); K10(iTRAQ8plex)	1.21	0.715	1.097	3.65	28	0	1810.08306	2	905.545166	-5.759895226	36.66094667
														NASDMPETITSR	4	4	1	N-Term(iTRAQ8plex)	1.041	0.71	1.389	3.4	33	0	1625.8062	2	821.4048462	-1.93722287	14.72376333
														NASDMPETITSR	1	4	1	N-Term(iTRAQ8plex); M5(Oxidation)	1.125	0.56	0.897	-	36	0	1641.80242	2	821.4048462	-1.93722287	14.72376333
23510381	TNPO1	transportin-1 isoform 2	NP_694858.1	11.69	8	8	17	890	101.2	4.96	0.900	2.008	2.255																
														LEQLNQYPDFNNYLIFVLTK	2	4	1	N-Term(iTRAQ8plex); K20(iTRAQ8plex)	0.751	3.102	3.233	6.41	49	0	3080.69382	3	1027.569458	0.67824768	64.61919167
														SNILTLMYQCMQDK	4	4	1	N-Term(iTRAQ8plex); C10(Methylthio); K14(iTRAQ8plex)	1.711	2.42	2.792	4.57	38	0	2342.16776	3	781.394104	-6.815710882	84.05989
														YSDIDIILK	2	4	1	N-Term(iTRAQ8plex); K10(iTRAQ8plex)	0.996	2.409	2.669	3.73	39	0	1801.08452	2	901.0458984	-4.781033262	70.18251667
														TLLENTAITIGR	3	3	1	N-Term(iTRAQ8plex)	0.96	1.614	1.823	3.7	46	0	1605.94158	2	803.4744263	-3.851560374	36.75101667
														GDVEEDETIPDSEQDIRPR	2	4	1	N-Term(iTRAQ8plex)	0.806	5.953	4.669	3.65	33	0	2504.19779	3	835.4041138	-3.080124678	22.33188167
														SHAVACVNQFIISR	1	4	1	N-Term(iTRAQ8plex); C6(Methylthio)	1.368	1.036	1.172	3.24	-	0	1894.98319	3	632.3325806	-5.36355723	38.17177833
														ALVMLLEVR	2	6	1	N-Term(iTRAQ8plex)	0.682	2.02	2.076	3.16	29	0	1347.83354	2	674.4204102	-0.035321498	50.48778667
														FLQFFK	1	6	1	N-Term(iTRAQ8plex); K6(iTRAQ8plex)	0.627	1.997	1.493	2.74	-	0	1437.85718	3	479.9572449	-9.917487664	44.80371833
24233517	IKBIP	inhibitor of nuclear factor kappa-B kinase-interacting protein isoform 1	NP_710154.1	11.67	3	4	6	377	43.1	4.92	0.564	0.466	0.665																
														AVSEIMEMQK	1	1	1	N-Term(iTRAQ8plex); K10(iTRAQ8plex)	0.517	0.319	0.427	3.19	-	0	1773.96623	2	887.4867554	-2.095405054	47.89656333
														MQNELDILK	1	1	1	N-Term(iTRAQ8plex); K9(iTRAQ8plex)	0.6	0.713	0.93	3.02	-	0	1711.98301	3	571.3325195	-2.509345149	47.48723833
														TLEGIQYDNSILK	2	1	1	N-Term(iTRAQ8plex); K13(iTRAQ8plex)	0.564	0.466	0.665	2.87	27	0	2102.18406	3	701.3995361	-5.383045093	50.37918667
														LETNEFQQLQSK	2	2	2	N-Term(iTRAQ8plex); K12(iTRAQ8plex)	-	-	-	4.29	34	0	2073.14133	3	691.7186279	-1.129088935	40.45933167
														ILTER	2	27	5	N-Term(iTRAQ8plex)	-	-	-	-	28	0	935.57616	2	468.2917175	-7.036157716	17.75416
24234688	HSPA9	stress-70 protein, mitochondrial precursor	NP_004125.3	51.40	26	26	204	679	73.6	6.16	1.053	0.705	0.723																
														VINEPTAAALAYGLDK	18	1	1	N-Term(iTRAQ8plex); K16(iTRAQ8plex)	1.15	0.786	0.661	7.02	83	0	2254.28203	2	1127.654419	4.968266366	38.060875
														GAVVGIDLGTTNSCVAVMEGK	4	1	1	N-Term(iTRAQ8plex); C14(Methylthio); K21(iTRAQ8plex)	1.034	0.763	0.793	6.87	87	0	2675.40873	3	892.4744263	2.232652729	65.24643833
														SQVFSTAADGQTQVEIK	7	1	1	N-Term(iTRAQ8plex); K17(iTRAQ8plex)	1.133	0.945	0.866	6.57	51	0	2417.31095	3	806.4389648	-4.525713877	29.97757167
														VEAVNMAEGIIHDTETK	14	1	1	N-Term(iTRAQ8plex); K17(iTRAQ8plex)	1.334	1.059	1.115	6.23	53	0	2465.30106	3	822.4385376	-6.332596569	48.12783833
														AQFEGIVTDLIR	16	1	1	N-Term(iTRAQ8plex)	1.153	0.641	0.674	6.08	68	0	1665.94315	3	555.9872437	-0.356309598	50.013605
														LYSPSQIGAFVLMK	15	1	1	N-Term(iTRAQ8plex); K14(iTRAQ8plex)	1.01	0.681	0.74	5.68	71	0	2162.24809	2	1081.627686	-1.066864739	42.57732833
														LLGQFTLIGIPPAPR	22	1	1	N-Term(iTRAQ8plex)	1.055	0.68	0.68	5.5	64	0	1897.15312	2	949.0802002	-2.423383394	58.20239667
														EQQIVIQSSGGLSKDDIENMVK	2	1	1	N-Term(iTRAQ8plex); K14(iTRAQ8plex); K22(iTRAQ8plex)	1.065	-	-	5.48	-	1	3330.80337	4	837.4572754	-6.550905302	29.59170167
														TTPSVVAFTADGER	10	1	1	N-Term(iTRAQ8plex)	1.06	0.873	0.722	5.46	75	0	1754.92636	3	585.6469727	2.098902167	28.61009167
														CELSSSVQTDINLPYLTMDSSGPK	2	1	1	N-Term(iTRAQ8plex); C1(Methylthio); K24(iTRAQ8plex)	0.649	0.858	1.014	5.43	57	0	3239.61509	3	1080.543213	1.726477291	53.07698667
														EQQIVIQSSGGLSKDDIENMVK	2	1	1	N-Term(iTRAQ8plex); K14(iTRAQ8plex); M20(Oxidation); K22(iTRAQ8plex)	-	-	-	5.41	26	1	3346.80727	4	837.4572754	-6.550905302	29.59170167
														NAVITVPAYFNDSQR	14	1	1	N-Term(iTRAQ8plex)	1.103	0.712	0.795	5.18	49	0	1999.0512	2	1000.029236	-1.944042415	33.68680833
														EQQIVIQSSGGLSK	2	1	1	N-Term(iTRAQ8plex); K14(iTRAQ8plex)	1.048	0.583	0.606	4.99	60	0	2082.19829	2	1041.602783	-1.556774763	32.93968167
														QAVTNPNNTFYATK	2	1	1	N-Term(iTRAQ8plex); K14(iTRAQ8plex)	1.12	0.964	0.859	4.83	42	0	2177.17527	3	726.3966064	-2.684769643	17.85982667
														MEEFKDQLPADECNK	6	1	1	N-Term(iTRAQ8plex); M1(Oxidation); K5(iTRAQ8plex); C13(Methylthio); K15(iTRAQ8plex)	1.464	1.042	1.476	4.7	28	1	2771.36928	4	693.5988159	-3.010604065	25.14403667
														STNGDTFLGGEDFDQALLR	2	1	1	N-Term(iTRAQ8plex)	0.202	0.197	0.496	4.54	63	0	2360.16318	3	787.3925781	-1.724531182	46.21390333
														SDIGEVILVGGMTR	13	1	1	N-Term(iTRAQ8plex)	0.818	0.578	0.777	4.53	58	0	1750.96378	3	584.3261108	-2.148548424	43.45539167
														VQQTVQDLFGR	15	1	1	N-Term(iTRAQ8plex)	1.057	0.733	0.819	4.27	60	0	1594.88835	2	797.9478149	1.79445966	41.839685
														CELSSSVQTDINLPYLTMDSSGPK	2	1	1	N-Term(iTRAQ8plex); C1(Methylthio); M18(Oxidation); K24(iTRAQ8plex)	0.995	0.664	0.76	3.97	44	0	3255.61521	3	1080.543213	1.726477291	53.07698667
														DAGQISGLNVLR	9	1	1	N-Term(iTRAQ8plex)	1.135	0.794	0.802	3.86	60	0	1546.89072	3	516.3017578	3.373791976	30.36069833
														GAVVGIDLGTTNSCVAVMEGK	2	1	1	N-Term(iTRAQ8plex); C14(Methylthio); M18(Oxidation); K21(iTRAQ8plex)	-	-	-	3.48	27	0	2691.3937	3	892.4744263	2.232652729	65.24643833
														SDIGEVILVGGMTR	4	1	1	N-Term(iTRAQ8plex); M12(Oxidation)	0.957	0.548	0.634	3.47	31	0	1766.96184	3	584.3261108	-2.148548424	43.45539167
														MEEFKDQLPADECNK	2	1	1	N-Term(iTRAQ8plex); K5(iTRAQ8plex); C13(Methylthio); K15(iTRAQ8plex)	1.006	0.722	0.734	3.4	-	1	2755.38938	4	693.5988159	-3.010604065	25.14403667
														VINEPTAAALAYGLDKSEDK	1	1	1	N-Term(iTRAQ8plex); K16(iTRAQ8plex); K20(iTRAQ8plex)	0.976	0.369	0.433	3.34	-	1	3017.69693	3	1006.570496	1.544975029	36.529915
														ASNGDAWVEAHGK	2	1	1	N-Term(iTRAQ8plex); K13(iTRAQ8plex)	1.029	0.834	0.803	3.3	41	0	1950.02542	3	650.6799927	-1.828962131	15.691945
														LFEMAYK	7	1	1	N-Term(iTRAQ8plex); K7(iTRAQ8plex)	1.035	0.687	0.717	3.24	28	0	1509.86066	3	503.9585266	0.970476063	52.04402
														EMAGDNK	2	1	1	N-Term(iTRAQ8plex); K7(iTRAQ8plex)	0.815	0.694	0.644	2.88	23	0	1372.74057	3	458.251709	3.986480206	11.12691333
														GVPQIEVTFDIDANGIVHVSAK	1	1	1	N-Term(iTRAQ8plex); K22(iTRAQ8plex)	-	-	-	2.79	-	0	2917.62685	4	730.1621704	0.833026548	65.586255
														VLENAEGAR	4	1	1	N-Term(iTRAQ8plex)	0.945	0.577	0.59	2.71	37	0	1262.69634	2	631.8519897	-3.126980377	18.553645
														AMQDAEVSK	1	1	1	N-Term(iTRAQ8plex); K9(iTRAQ8plex)	1.368	0.53	0.714	-	39	0	1586.85381	2	793.930542	-8.219998316	22.46386
														GVFEVK	1	1	1	N-Term(iTRAQ8plex); K6(iTRAQ8plex)	1.211	0.704	0.682	-	29	0	1286.79766	2	643.9024658	3.725310352	23.26629667
24234690	MRE11A	double-strand break repair protein MRE11A isoform 2	NP_005581.2	8.24	4	4	8	680	77.6	5.82	0.836	1.640	1.561																
														GNDTFVTLDEILR	2	3	1	N-Term(iTRAQ8plex)	0.729	2.151	1.894	4.95	50	0	1796.97563	2	898.9914551	3.34018789	46.06253833
														VDYSGGFEPFSVLR	2	4	1	N-Term(iTRAQ8plex)	0.836	1.64	1.745	3.8	33	0	1876.97295	2	938.9901123	-0.939443814	48.21701333
														QFFMEDIVLANHPDIFNPDNPK	1	4	1	N-Term(iTRAQ8plex); K22(iTRAQ8plex)	0.913	1.131	1.402	3.08	-	0	3209.66892	3	1070.561157	4.311076949	54.29080667
														DIIHFFR	3	4	1	N-Term(iTRAQ8plex)	0.619	1.558	1.245	2.71	36	0	1251.70511	3	417.9065552	-8.028266848	33.01069333
24234699	KRT19	keratin, type I cytoskeletal 19	NP_002267.2	70.25	21	34	464	400	44.1	5.14	0.117	0.158	0.121																
														GQVGGQVSVEVDSAPGTDLAK	8	1	1	N-Term(iTRAQ8plex); K21(iTRAQ8plex)	0.104	0.135	0.084	6.72	99	0	2622.43608	2	1311.72229	6.76170908	32.83927167
														QSSATSSFGGLGGGSVR	18	1	1	N-Term(iTRAQ8plex)	0.206	0.109	0.175	6.71	84	0	1858.95879	2	929.9790649	-2.828497148	17.255025
														TDLEMQIEGLKEELAYLK	6	1	1	N-Term(iTRAQ8plex); K11(iTRAQ8plex); K18(iTRAQ8plex)	-	-	-	6.42	34	1	3035.7145	4	763.6827393	1.304466148	60.68224333
														TDLEMQIEGLKEELAYLK	7	1	1	N-Term(iTRAQ8plex); M5(Oxidation); K11(iTRAQ8plex); K18(iTRAQ8plex)	-	-	-	6.34	37	1	3051.71694	4	763.6827393	1.304466148	60.68224333
														SQYEVMAEQNR	11	1	1	N-Term(iTRAQ8plex)	0.035	0.102	0.042	5.45	53	0	1658.80735	3	553.6072998	-2.203472239	15.57153167
														AALEDTLAETEAR	12	1	1	N-Term(iTRAQ8plex)	0.07	0.154	0.07	5.32	75	0	1693.89104	2	847.4473267	-2.169329401	24.603595
														ALEAANGELEVK	14	1	1	N-Term(iTRAQ8plex); K12(iTRAQ8plex)	0.028	0.123	0.074	5.14	81	0	1852.05864	2	926.532959	-2.70507161	36.58666833
														FGAQLAHIQALISGIEAQLGDVR	9	1	1	N-Term(iTRAQ8plex)	0.863	-	-	5.13	105	0	2711.51396	2	1356.26062	-0.272056731	71.55970167
														DYSHYYTTIQDLR	36	1	1	N-Term(iTRAQ8plex)	0.152	0.316	0.23	4.48	69	0	1978.97576	2	989.9939575	-0.284429734	32.042665
														EVAGHTEQLQMSR	33	1	1	N-Term(iTRAQ8plex)	0.097	0.099	0.093	4.47	72	0	1789.91753	2	895.4624023	0.361339274	17.109605

														EVAGHTEQLQMSR	15	1	1	N-Term(iTRAQ8plex); M11(Oxidation)	0.09	0.124	0.104	4.29	49	0	1805.91227	2	895.4624023	0.361339274	17.109605
														DKILGATIENSR	7	1	1	N-Term(iTRAQ8plex); K2(iTRAQ8plex)	0.132	0.138	0.113	4.03	38	1	1925.12541	2	963.067627	0.174526709	20.22332167
														SRLEQEIATYR	8	1	1	N-Term(iTRAQ8plex)	0.196	0.35	0.205	3.62	39	1	1669.91313	2	835.4612427	-1.378853504	25.52895167
														NHEEEISTLR	27	1	1	N-Term(iTRAQ8plex)	0.21	0.284	0.206	3.47	46	0	1531.8001	2	766.4035034	-1.358587563	14.26240333
														FGPGVAFR	6	1	1	N-Term(iTRAQ8plex)	0.048	0.084	0.039	3.41	43	0	1154.66086	3	385.5584717	-1.341961946	28.380275
														DAEAWFTSR	15	1	1	N-Term(iTRAQ8plex)	0.136	0.189	0.134	3.27	42	0	1386.69219	2	693.8497314	-2.433870623	37.64308
														KNHEEEISTLR	3	1	1	N-Term(iTRAQ8plex); K1(iTRAQ8plex)	0.124	0.333	0.093	2.74	33	1	1964.1021	2	982.5546875	-0.021852224	12.31501
														ILGATIENSR	8	1	1	N-Term(iTRAQ8plex)	0.154	0.182	0.19	2.7	45	0	1377.80022	2	689.4037476	-0.103645514	29.566375
														DYSHYYTTIQDLR	1	1	1	N-Term(iTRAQ8plex); Y5(iTRAQ8plex)	0.068	0.137	0.065	-	39	0	2283.18535	2	989.9939575	-0.284429734	32.042665
														SQYEVMAEQNR	3	1	1	N-Term(iTRAQ8plex); M6(Oxidation)	0.062	0.089	0.035	-	36	0	1674.8062	3	553.6072998	-2.203472239	15.57153167
														DYSHYYTTIQDLR	1	1	1	N-Term(iTRAQ8plex); Y2(iTRAQ8plex); Y5(iTRAQ8plex); Y6(iTRAQ8plex)	-	0.065	-	-	34	0	2891.58647	2	989.9939575	-0.284429734	32.042665
														ILSDMR	2	1	1	N-Term(iTRAQ8plex)	0.102	0.169	0.065	-	29	0	1038.59014	2	519.7987061	-1.738713438	32.65933833
														SQYEVMAEQNR	1	1	1	N-Term(iTRAQ8plex); Y3(iTRAQ8plex)	0.066	0.181	0.092	-	27	0	1963.01042	3	553.6072998	-2.203472239	15.57153167
														SEVTDLR	2	2	1	N-Term(iTRAQ8plex)	0.103	0.161	0.101	-	27	0	1123.62297	2	562.3151245	-2.757950521	14.50495833
														GVSVSSAR	1	1	1	N-Term(iTRAQ8plex)	0.013	0.063	0.027	-	27	0	1066.61626	2	533.8117676	0.391177471	13.306645
														SEVTDLRR	1	2	1	N-Term(iTRAQ8plex)	-	0.121	-	-	26	1	1279.72565	3	427.2467346	-1.20392412	17.20285333
														APSIHGGSGGR	1	1	1	N-Term(iTRAQ8plex)	0.191	0.194	0.18	-	26	0	1299.70867	2	650.3579712	1.180620591	10.89244333
														TIQGLEIELQSQLSMK	34	1	2	N-Term(iTRAQ8plex); M15(Oxidation); K16(iTRAQ8plex)	-	-	-	7.27	98	0	2442.37571	2	1221.687256	-2.543107016	44.24184333
														TIQGLEIELQSQLSMK	53	1	2	N-Term(iTRAQ8plex); K16(iTRAQ8plex)	-	-	-	6.42	68	0	2426.39151	2	1221.687256	-2.543107016	44.24184333
														ITMQNLNDR	1	6	2	N-Term(iTRAQ8plex)	-	-	-	-	32	0	1408.74761	2	704.8774414	-3.141164166	16.806955
														ITMQNLNDR	1	6	2	N-Term(iTRAQ8plex); M3(Oxidation)	-	-	-	-	24	0	1424.74614	2	704.8774414	-3.141164166	16.806955
														LAADDFR	10	31	7	N-Term(iTRAQ8plex)	-	-	-	-	39	0	1111.60307	2	556.3051758	-1.68797437	21.86801333
														LEQEIATYR	22	11	5	N-Term(iTRAQ8plex)	-	-	-	3.33	37	0	1426.78179	2	713.894104	-2.403951519	24.59227833
														MSVEADINGLR	23	2	2	N-Term(iTRAQ8plex)	-	-	-	5.86	56	0	1508.81614	3	503.6102295	7.723887815	31.32573667
														MSVEADINGLR	11	2	2	N-Term(iTRAQ8plex); M1(Oxidation)	-	-	-	3.84	46	0	1524.80033	3	503.6102295	7.723887815	31.32573667
														MSVEADINGLRR	8	2	2	N-Term(iTRAQ8plex)	-	-	-	3.7	37	1	1664.9041	2	832.9556885	-0.901533533	33.59522
														TDLEMQIEGLK	4	2	2	N-Term(iTRAQ8plex); K11(iTRAQ8plex)	-	-	-	4.6	59	0	1885.05156	2	943.0294189	-2.419047557	60.82738667
														IVLQIDNAR	17	2	2	N-Term(iTRAQ8plex)	-	-	-	3.65	63	0	1345.80796	3	449.2741699	-1.913188552	31.11273333
														LASYLDKVR	18	11	6	N-Term(iTRAQ8plex); K7(iTRAQ8plex)	-	-	-	3.04	34	1	1673.01726	4	419.0100403	-1.374788993	40.70226667
														VLDELTLAR	35	7	5	N-Term(iTRAQ8plex)	-	-	-	3.26	41	0	1333.80168	2	667.40448	1.785903382	36.03089333
														LASYLDK	45	13	7	N-Term(iTRAQ8plex); K7(iTRAQ8plex)	-	-	-	3.56	36	0	1417.84687	3	473.2871399	-2.979780374	31.776525
														TKFETEQALR	6	2	2	N-Term(iTRAQ8plex); K2(iTRAQ8plex)	-	-	-	2.8	48	1	1831.05312	4	458.5187378	-0.152921901	24.78654833
														FETEQALR	2	2	2	N-Term(iTRAQ8plex)	-	-	-	-	29	0	1297.70098	2	649.354126	-3.402915586	16.7415
														EELAYLKK	2	3	3	N-Term(iTRAQ8plex); K7(iTRAQ8plex); K8(iTRAQ8plex)	-	-	-	3.68	25	1	1906.17815	2	953.5927124	0.265916744	21.585135
														EELAYLK	5	3	3	N-Term(iTRAQ8plex); K7(iTRAQ8plex)	-	-	-	3.16	33	0	1473.87773	2	737.4425049	0.286214803	35.20986333
24234756	ILF3	interleukin enhancer-binding factor 3 isoform c	NP_703194.1	35.80	18	18	91	690	74.6	8.24	1.318	0.876	0.847																
														VADNLAIQLAAVTEDKYEILQSVDDAAIVIK	2	7	1	N-Term(iTRAQ8plex); K16(iTRAQ8plex); K31(iTRAQ8plex)	-	-	-	6.06	45	1	4241.38198	4	1061.100952	-5.397435639	85.513635
														EDITQSAQHALR	14	8	1	N-Term(iTRAQ8plex)	1.381	1.194	0.984	5.44	69	0	1672.88633	3	558.300293	-3.410658371	18.40365833
														LFPDTPLALDANK	6	9	1	N-Term(iTRAQ8plex); K13(iTRAQ8plex)	1.566	0.527	0.719	5.15	75	0	2023.16228	2	1012.084778	-3.050796271	33.38155167
														EATDAIGHLDR	21	8	1	N-Term(iTRAQ8plex)	1.163	0.869	0.755	4.97	69	0	1501.78397	3	501.2671509	-2.904482176	17.46057833
														AYAALAALEK	8	9	1	N-Term(iTRAQ8plex); K10(iTRAQ8plex)	1.408	0.798	0.773	4.61	50	0	1628.97771	2	814.9924927	-3.365649762	63.27379167
														HGKNPVMELNEK	1	9	1	N-Term(iTRAQ8plex); K3(iTRAQ8plex); K12(iTRAQ8plex)	1.749	1.611	1.133	4.58	-	1	2308.32705	4	577.8372192	2.593121773	15.35615667
														AVSDWIDEQEK	6	7	1	N-Term(iTRAQ8plex); K11(iTRAQ8plex)	1.421	0.816	0.81	4.55	61	0	1928.01518	2	964.5112305	-3.623925038	42.157595
														YELISETGGSHDKR	1	14	1	N-Term(iTRAQ8plex); K13(iTRAQ8plex)	0.717	0.794	0.663	4.38	-	1	2200.16713	4	550.7972412	-6.688735604	26.49835333
														VLQDMGLPTGAEGR	2	9	1	N-Term(iTRAQ8plex)	1.116	0.63	0.591	4.09	68	0	1747.92436	2	874.4658203	-4.077576322	36.83550333
														GDLDLELVLLCK	2	7	1	N-Term(iTRAQ8plex); C11(Methylthio); K12(iTRAQ8plex)	1.319	0.938	1.316	4.05	28	0	1985.11448	3	662.3763428	-6.401257886	80.34278
														LAAFGQLHK	10	8	1	N-Term(iTRAQ8plex); K9(iTRAQ8plex)	1.428	0.882	0.95	4.05	39	0	1592.97269	3	531.6624146	-0.377217802	30.16165
														VLGMDPLPSK	6	8	1	N-Term(iTRAQ8plex); K10(iTRAQ8plex)	1.317	0.85	0.915	3.97	43	0	1664.97673	2	832.9920044	-5.915476623	27.06858833
														GWPLELICEK	4	12	1	N-Term(iTRAQ8plex); C8(Methylthio); K10(iTRAQ8plex)	0.944	0.665	0.67	3.73	42	0	1842.00041	2	921.5038452	-5.970948883	78.56988667
														CLAALASLR	5	7	1	N-Term(iTRAQ8plex); C1(Methylthio)	1.319	1.051	1.075	3.38	49	0	1267.70615	3	423.2402344	-8.426578195	44.39620167
														SIGTANRPMGAGEALR	2	7	1	N-Term(iTRAQ8plex)	1.31	0.901	0.897	3.23	30	0	1905.02605	2	953.0166626	-0.951548504	19.04454833
														SCVIVIR	1	7	1	N-Term(iTRAQ8plex); C2(Methylthio)	1.178	0.76	0.723	-	26	0	1139.6552	2	570.3312378	-2.670398964	37.24003
														HSSVYPTQEELEAVQNMVSHTER	1	7	1	N-Term(iTRAQ8plex); Y5(iTRAQ8plex)	-	-	-	-	24	0	3279.63392	5	656.732605	-5.660054786	44.59455667
														VLECLASGIVMPDGSGIYDPCEK	1	8	1	N-Term(iTRAQ8plex); C4(Methylthio); C21(Methylthio); K23(iTRAQ8plex)	-	-	-	-	21	0	3096.49868	4	774.880127	-1.887734538	67.51267167
24307939	CCT5	T-complex protein 1 subunit epsilon isoform a	NP_036205.1	37.15	15	16	70	541	59.6	5.66	1.077	3.313	2.085																
														ETGANLAICQWGFDDEANHLLLQNNLPAVR	2	5	1	N-Term(iTRAQ8plex); C9(Methylthio)	1.103	1.916	1.788	9.74	92	0	3672.83677	3	1224.950439	4.40880181	56.23119
														EMNPALGIDCLHK	8	5	1	N-Term(iTRAQ8plex); C10(Methylthio); K13(iTRAQ8plex)	1.06	4.623	2.563	6.07	76	0	2095.09018	2	1048.046997	-4.383961436	32.50200667
														LGFAGLVQEISFGTTK	14	5	1	N-Term(iTRAQ8plex); K16(iTRAQ8plex)	1.353	2.878	1.906	5.72	62	0	2276.31597	2	1138.661621	2.136903476	54.55266
														QQHVIETLIGK	3	5	1	N-Term(iTRAQ8plex); K11(iTRAQ8plex)	0.879	3.449	2.324	5.69	47	0	1874.11589	2	937.5615845	-8.584834456	28.21363667
														WVGGPEIELIAIATGGR	4	5	1	N-Term(iTRAQ8plex)	1.229	2.417	1.444	4.91	69	0	2043.1514	3	681.7219849	-1.32636287	64.44975333
														EMNPALGIDCLHK	3	5	1	N-Term(iTRAQ8plex); M2(Oxidation); C10(Methylthio); K13(iTRAQ8plex)	1.095	2.827	2.223	4.91	42	0	2111.08316	2	1048.046997	-4.383961436	32.50200667
														QMAEIAVNAVLTVADMER	2	5	1	N-Term(iTRAQ8plex)	-	-	-	4.74	71	0	2265.19315	3	755.7359009	2.034301896	68.45202667
														IAILTCPFEPPKPK	5	5	1	N-Term(iTRAQ8plex); C6(Methylthio); K12(iTRAQ8plex); K14(iTRAQ8plex)	1.745	7.407	4.422	4.55	29	0	2512.46046	5	503.2979126	-7.731792024	47.67257
														QQISLATQMVR	2	5	1	N-Term(iTRAQ8plex)	1.018	2.913	1.858	4.22	31	0	1578.89592	2	789.9515991	1.247911379	29.32867167
														FSELTAEK	1	5	1	N-Term(iTRAQ8plex); K8(iTRAQ8plex)	1.338	2.615	2.085	3.83	-	0	1532.86539	3	511.6266479	-8.259500424	35.53937667
														LMGLEALK	4	2	1	N-Term(iTRAQ8plex); K8(iTRAQ8plex)	1.029	4.63	3.098	3.7	42	0	1482.9036	3	494.9727173	-9.32811448	61.35231667
														GVIVDKDFSHPQMPK	1	5	1	N-Term(iTRAQ8plex); K6(iTRAQ8plex); K15(iTRAQ8plex)	0.922	3.382	1.78	3.41	-	1	2610.4711	3	870.8285522	-4.986767216	26.215165
														DVDFELIK	4	5	1	N-Term(iTRAQ8plex); K8(iTRAQ8plex)	0.875	2.09	1.366	3.23	36	0	1586.91142	2	793.9593506	-8.56175582	46.27650833
														MLVIEQCK	2	5	1	N-Term(iTRAQ8plex); C7(Methylthio); K8(iTRAQ8plex)	1.228	4.027	2.227	3.09	36	0	1617.89067	2	809.4489746	-4.950706117	63.53728
														LMVELSK	1	3	1	N-Term(iTRAQ8plex); K7(iTRAQ8plex)	0.905	3.908	3.013	2.78	-	0	1427.87894	3	476.6311646	2.604634216	39.75831167
														AVTIFIR	4	5	1	N-Term(iTRAQ8plex)	1.004	3.168	1.747	-	21	0	1123.70854	2	562.3579102	-4.945773111	34.20027667
														SLHDALCVIR	18	7	2	N-Term(iTRAQ8plex); C7(Methylthio)	-	-	-	3.54	55	0	1476.78952	3	492.9402161	6.244992752	32.50943833
24308007	ARL6IP1	ADP-ribosylation factor-like protein 6-interacting protein 1 isoform 1	NP_055976.1	21.67	2	3	10	203	23.3	9.32	1.363	1.748	1.344																
														FHEICSNLVK	8	2	1	N-Term(iTRAQ8plex); C5(Methylthio); K10(iTRAQ8plex)	1.363	1.748	1.344	4.22	44	0	1843.99289	2	922.4977417	-7.421696903	38.53452
														STNLLAAETASLEEQLQGWGEVMLMADK	1	1	1	N-Term(iTRAQ8plex); K28(iTRAQ8plex)	-	-	-	-	26	0	3643.89101	4	911.7282104	2.774675983	72.01379167
														IFGSNK	1	8	2	N-Term(iTRAQ8plex); K6(iTRAQ8plex)	-	-	-	2.74	-	0	1273.7717	3	425.262085	-0.588541248	18.094945
24308013	PMPCA	mitochondrial-processing peptidase subunit alpha isoform 1 precursor	NP_055975.1	8.38	4	4	10	525	58.2	6.92	1.260	0.934	0.712																
														FDSKDEILLTLEK	1	2	1	N-Term(iTRAQ8plex); K4(iTRAQ8plex); K13(iTRAQ8plex)	1.39	1.137	0.867	4.08	-	1	2463.44822	3	821.8209229	0.342611094	41.35916
														KLPHELCTLIR	6	6	1	N-Term(iTRAQ8plex); K1(iTRAQ8plex); C7(Methylthio)	1.276	0.925	0.591	3.52	36	1	1977.14565	4	495.043335	-4.194082349	36.33499333
														DTTMYAVSADSK	2	2	1	N-Term(iTRAQ8plex); M4(Oxidation); K12(iTRAQ8plex)	1.219	1.129	0.859	3.15	26	0	1912.97202	3	638.3288574	-3.255292975	14.74915167
														LYLNVLNR	1	6	1	N-Term(iTRAQ8plex)	1.185	0.8	0.558	-	28	0	1308.7896	2	654.8984375	-3.463444396	27.35641833
24308039	UFL1	E3 UFM1-protein ligase 1	NP_056138.1	7.05	4	4	7	794	89.5	6.79	0.987	0.735	0.474																
														VNIVDLQQVINVDLIHIENR	2	1	1	N-Term(iTRAQ8plex)	0.545	1.013	0.811	7	74	0	2648.51126	3	883.508606	2.825482071	62.05363
														SVFMSSTTSASGTGR	2	1	1	N-Term(iTRAQ8plex)	1.094	0.541	0.264	4.07	22	0	1779.88799	2	890.4476318	1.728059236	25.14277333
														FINDCTELFR	2	1	1	N-Term(iTRAQ8plex); C5(Methylthio)	1.029	0.647	0.326	3.73	39	0	1607.79924	2	804.4032593	8.004503518	57.314545
														QLEVVHTLDGK	1	1	1	N-Term(iTRAQ8plex); K11(iTRAQ8plex)	0.946	0.834	0.691	3.14	-	0	1847.08609	3	616.3668823	0.75384827	23.80241833
24308201	APMAP	adipocyte plasma membrane-associated protein	NP_065392.1	28.61	9	9	29	416	46.5	6.16	0.749	0.572	0.837																
														LFENQLVGPESIAHIGDVMFTGTADGR	10	2	1	N-Term(iTRAQ8plex)	0.789	0.356	0.899	6.54	61	0	3178.61057	3	1060.208374	-1.262337616	80.05529833
														RDYLLLVMEGTDDGR	7	2	1	N-Term(iTRAQ8plex)	0.799	1.283	1.187	4.21	41	1	2057.05881	3	686.3577881	-2.503716549	41.68589333
														DYLLLVMEGTDDGR	2	2	1	N-Term(iTRAQ8plex)	0.771	0.623	0.797	4.09	70	0	1900.96538	2	950.9863281	1.335284707	50.72809
														LLLSSETPIEGK	2	2	1	N-Term(iTRAQ8plex); K12(iTRAQ8plex)	0.609	0.261	0.318	3.83	34	0	1895.12712	2	948.0671997	-2.041290999	26.048585
														YSLVLELSDSGAFR	2	1	1	N-Term(iTRAQ8plex)	0.873	0.517	0.726	3.81	53	0	1861.00713	2	931.0072021	3.334955994	51.98259333
														RPLRPQVVTDDDGQAPEAK	2	2	1	N-Term(iTRAQ8plex); K19(iTRAQ8plex)	0.707	-	0.602	3.62	-	0	2700.48305	4	675.8762207	-2.19900195	15.83114333
														GLFEVNPWK	2	2	1	N-Term(iTRAQ8plex); K9(iTRAQ8plex)	0.54	0.422	0.539	3.15	28	0	1697.97855	3	566.6643677	-2.933795987	60.92041333
														TRDDEPVCGRPLGIR	1	2	1	N-Term(iTRAQ8plex); C8(Methylthio)	1	1.15	1.473	2.75	-	1	2034.04717	2	1017.527222	-2.71653515	19.35436167
														VYVSGLMK	1	1	1	N-Term(iTRAQ8plex); K8(iTRAQ8plex)	0.543	-	0.384	-	28	0	1504.88697	3	502.3005066	-9.831153395	37.42491333
24308213	SERINC1	serine incorporator 1 precursor	NP_065806.1	2.87	1	1	2	453	50.5	5.85	1.295	0.627	0.690																
														SDGSLEDGDDVHR	2	1	1	N-Term(iTRAQ8plex)	1.295	0.627	0.69	3.84	36	0	1705.78781	3	569.2674561	-3.11081243	11.44379
24308295	GRPEL1	grpE protein homolog 1, mitochondrial precursor	NP_079472.1	17.97	3	3	8	217	24.3	8.12	1.107	0.654	0.713																
														DLLEVADVLEK	5	1	1	N-Term(iTRAQ8plex); K11(iTRAQ8plex)	1.232	0.767	0.828	5.12	52	0	1852.09604	2	926.5457153	-2.509203444	39.086925
														EEIKDDNPHLK	1	1	1	N-Term(iTRAQ8plex); K4(iTRAQ8plex); K11(iTRAQ8plex)	1.011	0.551	0.534	4.07	-	1	2250.27976	3	750.7647705	-2.645460655	14.84825
														FDPYEHEALFHTPVEGK	2	1	1	N-Term(iTRAQ8plex); K17(iTRAQ8plex)	1.072	0.754	0.703	3.79	-	0	2624.34389	4	656.8414307	-6.351501339	31.76991833
24308324	FAR1	fatty acyl-CoA reductase 1	NP_115604.1	6.02	2	2	3	515	59.3	9.17	1.062	1.112	0.941																
														KLIDSLEWMDDGLVNDITPK	2	2	1	N-Term(iTRAQ8plex); K1(iTRAQ8plex); K20(iTRAQ8plex)	-	-	-	4.44	63	1	3214.77463	3	1072.263062	-1.586419041	58.47679833
														DAVQLNVIATR	1	2	1	N-Term(iTRAQ8plex)	1.062	1.112	0.941	3.14	-	0	1503.87688	2	752.4420776	-1.86361437	28.794335
24430132	WBP2	WW domain-binding protein 2	NP_036610.2	9.20	2	2	3	261	28.1	5.91	0.977	0.483	1.222																
														AEAGGGWEGSASYK	2	2	1	N-Term(iTRAQ8plex); K14(iTRAQ8plex)	0.609	0.315	0.829	3.99	43	0	1977.99808	3	660.0042114	-7.376282513	29.24396167
														MLQVASQASR	1	1	1	N-Term(iTRAQ8plex); M1(Oxidation)	1.568	0.742	1.8	-	25	0	1410.76177	2	705.8845215	-4.196279904	18.40633667
24430137	LAGE3	EKC/KEOPS complex subunit LAGE3	NP_006005.2	16.08	1	1	2	143	14.8	8.63	1.674	1.973	1.992																
														GGVDTAAAPAGGAPPAHAPGPGR	2	1	1	N-Term(iTRAQ8plex)	1.674	1.973	1.992	5.09	40	0	2256.1763	3	752.7302856	-1.102580681	12.77730667
24430151	PSMC1	26S protease regulatory subunit 4	NP_002793.2	22.50	7	7	31	440	49.2	6.21	1.338	2.608	2.104																
														VHAVIGVLMDDTDPLVTVMK	4	2	1	N-Term(iTRAQ8plex); K20(iTRAQ8plex)	0.453	1.51	1.204	5.86	58	0	2761.53676	4	691.1396484	-3.098792713	63.02743667
														TMLELLNQLDGFDSR	4	2	1	N-Term(iTRAQ8plex)	1.575	3.09	2.315	5.54	67	0	2056.08257	2	1028.544922	6.738946296	60.64678167
														AVANQTSATFLR	4	2	1	N-Term(iTRAQ8plex)	1.021	1.789	2.052	4.76	51	0	1582.88852	3	528.3010254	1.915246608	27.21002333
														MTLADDVTLDDLIMAK	4	2	1	N-Term(iTRAQ8plex); K16(iTRAQ8plex)	0.99	2.556	2.218	4.54	91	0	2373.28569	2	1187.146484	-0.39572564	51.73755333

														IKDYLLMEEEFIR	9	1	1	N-Term(iTRAQ8plex); K2(iTRAQ8plex)	1.461	3.11	2.998	3.9	39	1	2307.29422	4	577.5772705	-0.290015363	55.65192
														AICTEAGLMALR	2	2	1	N-Term(iTRAQ8plex); C3(Methylthio)	-	1.388	1.055	3.34	41	0	1598.82584	3	533.6134644	-7.012948699	48.463855
														LPLVTPHTQCR	4	1	1	N-Term(iTRAQ8plex); C10(Methylthio)	3.059	2.391	1.548	3.29	35	0	1614.86808	3	538.9608765	-5.019701354	26.96637
24430155	PSMC4	26S protease regulatory subunit 6B isoform 2	NP_694546.1	21.45	4	4	7	387	43.5	5.26	0.864	1.602	1.939																
														AQDEIPALSVSRPQTGLSF LGPEPEDLEDLYSR	1	2	1	N-Term(iTRAQ8plex)	0.89	2.877	3.518	4.45	-	0	3934.01694	3	1312.010498	3.672986376	49.66454667
														ILLELLNQMDGFDQNVNV K	2	2	1	N-Term(iTRAQ8plex); K19(iTRAQ8plex)	-	-	-	4.1	62	0	2811.56723	3	937.8605957	4.862967248	64.456365
														ADTLDPALLRPGR	2	2	1	N-Term(iTRAQ8plex)	0.864	1.088	1.296	2.9	-	0	1698.97563	2	849.9914551	-2.853345071	25.51701
														ENAPAIIFIDEIDAIATK	2	2	1	N-Term(iTRAQ8plex); K18(iTRAQ8plex)	0.766	2.086	2.852	2.86	28	0	2552.45806	2	1276.732666	5.796025215	62.21984333
24430192	KRT16	keratin, type I cytoskeletal 16	NP_005548.2	31.50	1	18	371	473	51.2	5.05	0.585	0.216	0.272																
														DAETWFLSK	2	1	1	N-Term(iTRAQ8plex); K9(iTRAQ8plex)	0.585	0.216	0.272	3.25	31	0	1704.93682	2	852.9720459	-2.877598208	52.768485
														ALEEANADLEVK	48	8	3	N-Term(iTRAQ8plex); K12(iTRAQ8plex)	-	-	-	5.48	82	0	1910.06462	2	955.5366211	-1.657394329	22.98732333
														LAADDFR	10	31	7	N-Term(iTRAQ8plex)	-	-	-	-	39	0	1111.60307	2	556.3051758	-1.68797437	21.86801333
														TRLEQEIATYR	39	9	4	N-Term(iTRAQ8plex)	-	-	-	3.84	45	1	1683.92827	2	842.4677734	-2.90454474	28.813565
														LEQEIATYR	22	11	5	N-Term(iTRAQ8plex)	-	-	-	3.33	37	0	1426.78179	2	713.894104	-2.403951519	24.59227833
														VTMQNLNDR	4	10	3	N-Term(iTRAQ8plex); M3(Oxidation)	-	-	-	3.55	61	0	1410.73212	3	470.9155579	0.584117972	12.82526667
														VTMQNLNDR	6	10	3	N-Term(iTRAQ8plex)	-	-	-	3	42	0	1394.73418	3	470.9155579	0.584117972	12.82526667
														GSCGIGGGIGGGSSR	51	2	2	N-Term(iTRAQ8plex); C3(Methylthio)	-	-	-	5.1	76	0	1571.76799	2	786.3823853	0.17279244	21.17306167
														ASLENSLEETK	18	2	2	N-Term(iTRAQ8plex); K11(iTRAQ8plex)	-	-	-	4.6	64	0	1829.00701	2	915.0089111	-0.391279524	21.283405
														QFTSSSSMK	10	2	2	N-Term(iTRAQ8plex); K9(iTRAQ8plex)	-	-	-	4.01	38	0	1610.86479	2	805.9360352	-1.264915285	14.47664
														ISSVLAGGSCR	30	2	2	N-Term(iTRAQ8plex); C10(Methylthio)	-	-	-	3.28	46	0	1399.73198	2	700.3696289	-1.401059293	43.93359
														DYSPYFK	17	3	2	N-Term(iTRAQ8plex); K7(iTRAQ8plex)	-	-	-	3.23	31	0	1527.82756	2	764.418335	-0.620486901	29.96665833
														QFTSSSSMK	5	2	2	N-Term(iTRAQ8plex); M8(Oxidation); K9(iTRAQ8plex)	-	-	-	3.1	32	0	1626.86443	2	805.9360352	-1.264915285	14.47664
														TIEDLR	4	2	2	N-Term(iTRAQ8plex)	-	-	-	-	28	0	1050.60954	2	525.8084106	-0.13956749	25.768515
														TDLEMQIEGLK	4	2	2	N-Term(iTRAQ8plex); K11(iTRAQ8plex)	-	-	-	4.6	59	0	1885.05156	2	943.0294189	-2.419047557	60.82738667
														LASYLDKVR	18	11	6	N-Term(iTRAQ8plex); K7(iTRAQ8plex)	-	-	-	3.04	34	1	1673.01726	4	419.0100403	-1.374788993	40.70226667
														VLDELTLAR	35	7	5	N-Term(iTRAQ8plex)	-	-	-	3.26	41	0	1333.80168	2	667.40448	1.785903382	36.03089333
														LASYLDK	45	13	7	N-Term(iTRAQ8plex); K7(iTRAQ8plex)	-	-	-	3.56	36	0	1417.84687	3	473.2871399	-2.979780374	31.776525
														ILNEMR	2	3	3	N-Term(iTRAQ8plex)	-	-	-	-	25	0	1079.61772	2	540.3125	-0.711288805	26.970265
														IRDWYQR	1	4	3	N-Term(iTRAQ8plex)	-	-	-	-	23	1	1340.73529	3	447.5832825	-1.780469196	20.12547667
24431933	RTN4	reticulon-4 isoform B	NP_722550.1	27.35	6	6	37	373	40.3	4.78	0.697	0.366	0.696																
														GSSGSVVVDLLYWR	15	1	1	N-Term(iTRAQ8plex)	0.595	0.297	0.662	5.44	83	0	1842.00078	2	921.5040283	-3.019151477	44.24951667
														AYLESEVAISEELVQK	8	5	1	N-Term(iTRAQ8plex); K16(iTRAQ8plex)	0.895	0.571	0.834	5.43	57	0	2416.33335	3	806.1159668	-4.069868666	40.68231167
														HQAQIDHYLGLANK	3	5	1	N-Term(iTRAQ8plex); K14(iTRAQ8plex)	0.7	0.354	0.699	4.4	29	0	2216.25942	4	554.8203125	8.928927643	27.099705
														LFLVDDLVDSLK	5	5	1	N-Term(iTRAQ8plex); K12(iTRAQ8plex)	0.725	0.492	0.731	4	52	0	1985.18644	3	662.4003296	4.27376461	64.47606333
														YQFVREPEDEEEEEEEEE DEDEDLEELEVLER	2	3	1	N-Term(iTRAQ8plex)	-	-	-	3.85	57	1	4490.9249	3	1497.646484	-1.621364232	47.91787833
														GPLPAAPPVAPER	4	3	1	N-Term(iTRAQ8plex)	0.633	0.335	0.632	3.56	47	0	1575.91545	2	788.4613647	-0.405115523	23.92935167
24475816	TECR	very-long-chain enoyl-CoA reductase isoform 1	NP_612510.1	6.17	2	2	4	308	36.0	9.45	1.139	0.556	0.677																
														HYEVEILDAK	2	1	1	N-Term(iTRAQ8plex); K10(iTRAQ8plex)	1.036	0.757	0.703	3.6	28	0	1825.02481	3	609.0131226	-3.713305247	23.87250833
														LLETLFVHR	2	4	1	N-Term(iTRAQ8plex)	1.251	0.409	0.652	3.22	50	0	1431.86432	3	477.9596252	1.229992531	45.54649167
24475861	PHPT1	14 kDa phosphohistidine phosphatase isoform 3	NP_054891.2	21.60	2	2	3	125	13.8	6.07	1.106	1.543	1.930																
														QGCDCECLGGGR	2	4	1	N-Term(iTRAQ8plex); C3(Methylthio); C5(Methylthio); C7(Methylthio)	1.331	2.384	2.649	3.88	32	0	1639.61479	2	820.3110352	1.01206241	34.13602
														AKYPDYEVTWANDGY	1	2	1	N-Term(iTRAQ8plex); K2(iTRAQ8plex)	0.919	0.998	1.407	2.84	-	1	2400.18418	3	800.7329102	-5.267962921	52.45956833
24497603	NUP62	nuclear pore glycoprotein p62	NP_036478.2	2.68	1	1	2	522	53.2	5.31	0.998	0.582	0.631																
														ELEDLLSPLEELVK	2	1	1	N-Term(iTRAQ8plex); K14(iTRAQ8plex)	0.998	0.582	0.631	5.05	64	0	2235.2918	2	1118.149536	-1.188057139	64.66677833
24638446	HIST2H2AC	histone H2A type 2-C	NP_003508.1	63.57	2	8	191	129	14.0	10.90	1.803	0.933	0.924																
														NDEELNKLLGK	3	7	1	N-Term(iTRAQ8plex); K7(iTRAQ8plex); K11(iTRAQ8plex)	1.803	0.933	0.924	4.54	33	1	2185.27422	2	1093.140747	-9.762518225	35.63651167
														VGAGAPVYMAAVLEYLT AEILELAGNAAR	2	2	1	N-Term(iTRAQ8plex); M9(Oxidation)	-	-	-	4.44	59	0	3253.72739	3	1085.247314	-5.269707191	86.542555
														VTIAQGGVLPNIQAVLLPK	39	10	2	N-Term(iTRAQ8plex); K19(iTRAQ8plex)	-	-	-	7.02	76	0	2539.56987	2	1270.292358	-0.862790435	49.553205
														HLQLAIRNDEELNK	3	12	2	N-Term(iTRAQ8plex); K14(iTRAQ8plex)	-	-	-	3.42	28	1	2301.30117	4	576.0807495	-5.380926798	26.793805
														NDEELNK	7	13	3	N-Term(iTRAQ8plex); K7(iTRAQ8plex)	-	-	-	3.69	35	0	1469.80754	2	735.4074097	1.314060434	12.628885
														KGNYAER	1	9	3	N-Term(iTRAQ8plex); K1(iTRAQ8plex)	-	-	-	2.94	-	1	1445.81789	4	362.2099304	-9.821084933	10.03500667
														AGLQFPVGR	34	17	4	N-Term(iTRAQ8plex)	-	-	-	3.56	51	0	1248.73304	3	416.91745	0.925177022	28.85471333
														HLQLAIR	102	17	3	N-Term(iTRAQ8plex)	-	-	-	3.4	39	0	1154.72541	3	385.5799866	-4.966404282	30.15787333
24638454	ATP2A2	sarcoplasmic/endoplasmic reticulum calcium ATPase 2 isoform b	NP_733765.1	31.19	26	26	140	1042	114.7	5.34	1.116	0.819	0.520																
														SLPSVETLGCTSVICSDK	2	21	1	N-Term(iTRAQ8plex); C10(Methylthio); C15(Methylthio); K18(iTRAQ8plex)	1.165	0.748	0.42	6.67	58	0	2539.27475	3	847.0964355	0.425353981	48.18188667
														EEMHLEDSANFIK	4	3	1	N-Term(iTRAQ8plex); K13(iTRAQ8plex)	1.217	0.872	0.582	5.97	36	0	2171.12052	3	724.3783569	-2.677764395	28.93223333
														DIVPGDIVEIAVGDKVPAD IR	14	3	1	N-Term(iTRAQ8plex); K15(iTRAQ8plex)	0.926	0.787	0.43	5.33	50	1	2799.61557	2	1400.306885	-0.442343862	49.66674
														TVEEVLGHFGVNESTGLSL EQVK	6	3	1	N-Term(iTRAQ8plex); K23(iTRAQ8plex)	1.883	0.683	0.361	5.32	35	0	3080.67788	3	1027.562622	0.266714726	49.16152833
														IVEFLQSFDEITAMTGDGV NDAPALK	2	3	1	N-Term(iTRAQ8plex); K26(iTRAQ8plex)	-	-	-	5.04	80	0	3389.77402	3	1130.596191	-0.587157503	71.17993333
														NAENAIEALKEYEPEMGK	2	5	1	N-Term(iTRAQ8plex); K10(iTRAQ8plex); M16(Oxidation); K18(iTRAQ8plex)	1.258	0.723	0.87	4.82	27	1	2964.55214	4	741.8934937	-7.77573458	37.63885333
														AMGVVVATGVNTEIGK	4	3	1	N-Term(iTRAQ8plex); M2(Oxidation); K16(iTRAQ8plex)	0.985	1.067	0.805	4.82	66	0	2170.2341	3	724.0828857	-0.982531531	28.22417833
														CLALATHDNPLR	21	3	1	N-Term(iTRAQ8plex); C1(Methylthio)	1.093	0.804	0.42	4.8	68	0	1673.87235	3	558.6289673	-2.732403421	35.876635
														IRDEMVATEQER	11	3	1	N-Term(iTRAQ8plex)	0.946	0.867	0.55	4.69	51	1	1780.90825	2	890.9577637	-4.666481974	15.64981833
														TVEEVLGHFGVNESTGLSL EQVKK	1	3	1	N-Term(iTRAQ8plex); K23(iTRAQ8plex); K24(iTRAQ8plex)	1.264	-	-	4.61	-	1	3512.96308	5	703.3984375	-2.772676449	46.85145
														VGEATETALTCLVEK	8	18	1	N-Term(iTRAQ8plex); C11(Methylthio); K15(iTRAQ8plex)	1.335	0.951	0.699	4.51	56	0	2218.18779	2	1109.597534	-1.884673895	45.29542667
														IGIFGQDEDVTSK	6	3	1	N-Term(iTRAQ8plex); K13(iTRAQ8plex)	1.228	0.963	0.4	4.5	57	0	2017.10015	2	1009.053711	-3.022182619	47.50672167
														CLALATHDNPLRR	12	3	1	N-Term(iTRAQ8plex); C1(Methylthio)	1.065	0.587	0.665	4.5	44	1	1829.97512	4	458.2487183	-2.731806976	37.38448333
														KAEIGIAMGSGTAVAK	2	20	1	N-Term(iTRAQ8plex); K1(iTRAQ8plex); K16(iTRAQ8plex)	1.512	0.895	0.671	4.39	28	1	2416.4313	4	604.8632813	-1.997234521	25.87826167
														NMLFSGTNIAAGK	2	6	1	N-Term(iTRAQ8plex); K13(iTRAQ8plex)	1.364	0.813	0.405	4.3	46	0	1932.07981	3	644.6981201	-1.829219696	43.15126
														ISLPVILMDETLK	10	3	1	N-Term(iTRAQ8plex); K13(iTRAQ8plex)	1.343	0.897	0.557	4.3	55	0	2080.25022	3	694.0882568	-2.218022692	52.94970833
														MNVFDTELK	11	3	1	N-Term(iTRAQ8plex); K9(iTRAQ8plex)	0.915	0.693	0.339	4.18	49	0	1704.93637	3	568.9862671	-0.500474118	49.33668333
														TGTLTTNQMSVCR	3	18	1	N-Term(iTRAQ8plex); C12(Methylthio)	0.982	0.869	0.571	3.91	46	0	1761.86052	2	881.4338989	0.322915211	29.20228333
														HTDPVPDPR	8	3	1	N-Term(iTRAQ8plex)	1.035	0.852	0.722	3.43	40	0	1337.71204	3	446.5734558	-4.281298847	12.47910667
														EFTLEFSR	3	21	1	N-Term(iTRAQ8plex)	1.1	0.862	0.466	3.43	26	0	1332.71514	2	666.8612061	3.747379498	32.44247667
														IEVASSVK	2	3	1	N-Term(iTRAQ8plex); K8(iTRAQ8plex)	1.146	0.972	0.4	3.37	30	0	1440.88996	3	480.9681702	1.204774895	30.72143833
														NYLEPGKECVQPATK	1	1	1	N-Term(iTRAQ8plex); K7(iTRAQ8plex); C9(Methylthio); K15(iTRAQ8plex)	1.283	3.032	-	3.16	-	1	2635.41103	4	659.6082153	-9.133623404	26.36525667
														NAENAIEALK	2	5	1	N-Term(iTRAQ8plex); K10(iTRAQ8plex)	1.139	0.746	0.881	3.08	37	0	1680.97281	3	560.9957886	-0.760766227	24.92924167
														GAPEGVIDR	1	6	1	N-Term(iTRAQ8plex)	1.061	0.759	0.366	2.72	-	0	1217.67681	2	609.342041	-1.959377677	16.40244
														LDEFGEQLSK	1	6	1	N-Term(iTRAQ8plex); K10(iTRAQ8plex)	0.988	0.986	0.81	-	26	0	1773.97922	3	591.9979248	-2.874969378	45.65246167
														SMSVYCTPNKPSR	1	3	1	N-Term(iTRAQ8plex); C6(Methylthio); K10(iTRAQ8plex)	1.434	0.925	0.555	-	22	0	2124.07358	3	708.6960449	-5.866456079	19.74701333
24797106	FAF2	FAS-associated factor 2	NP_055428.1	26.07	7	7	21	445	52.6	5.62	0.818	0.536	0.562																
														VTDPVGDIVSFMHSFEEK	8	2	1	N-Term(iTRAQ8plex); K18(iTRAQ8plex)	0.517	0.312	0.34	5.77	45	0	2645.39077	4	662.0983276	-1.024670125	66.63532
														NTLCAPEVISLINTR	2	2	1	N-Term(iTRAQ8plex); C4(Methylthio)	0.91	0.571	0.579	4.89	58	0	1994.0689	2	997.5380859	-1.392652484	53.43231333
														FTYYTILDIFR	3	2	1	N-Term(iTRAQ8plex)	0.695	0.312	0.313	4.49	47	0	1755.97685	2	878.4920654	8.276270465	65.14353833
														LNEQEGVPSVFNPPPSRPL QVNTADHR	1	1	1	N-Term(iTRAQ8plex)	-	-	-	3.99	-	0	3302.70742	4	826.432312	-3.34063292	29.92256667
														HTLEQHNWNIEAAVQDR	4	1	1	N-Term(iTRAQ8plex)	0.851	0.504	0.562	3.67	34	0	2365.19272	3	789.0690918	-1.010133066	25.51983167
														ENTYPFLAMIMLK	2	2	1	N-Term(iTRAQ8plex); K13(iTRAQ8plex)	0.68	0.687	0.647	3.61	26	0	2179.21415	3	727.0762329	1.176653737	53.17281667
														MLFWACSTNKPEGYR	1	2	1	N-Term(iTRAQ8plex); C6(Methylthio); K10(iTRAQ8plex)	0.818	0.654	0.641	2.82	-	0	2457.23655	3	819.7503662	1.12039796	45.73836
25188179	VDAC3	voltage-dependent anion-selective channel protein 3 isoform 1	NP_005653.3	24.38	5	6	22	283	30.6	8.66	0.834	0.902	0.911																
														WNTDNTLGTEISWENK	4	3	1	N-Term(iTRAQ8plex); K16(iTRAQ8plex)	0.77	1.031	0.911	6.09	45	0	2516.27451	3	839.4296875	-5.278458273	63.00386167
														LTLDTIFVPNTGK	2	3	1	N-Term(iTRAQ8plex); K13(iTRAQ8plex)	0.795	0.915	0.923	4.68	46	0	2027.18242	2	1014.094849	-8.544201152	34.27118667
														LSQNNFALGYK	2	3	1	N-Term(iTRAQ8plex); K11(iTRAQ8plex)	0.602	0.615	0.559	4.57	39	0	1863.04575	3	621.6867676	-6.828504146	39.299785
														VCNYGLTFTQK	1	3	1	N-Term(iTRAQ8plex); C2(Methylthio); K11(iTRAQ8plex)	0.903	0.746	0.801	2.76	-	0	1928.01853	3	643.3443604	-2.325960694	38.19239167
														GYGFGMVK	1	2	1	N-Term(iTRAQ8plex); K8(iTRAQ8plex)	0.876	1.12	0.999	-	31	0	1466.8344	2	733.9208374	3.951969421	22.33428
														LTLSALIDGK	12	3	2	N-Term(iTRAQ8plex); K10(iTRAQ8plex)	-	-	-	4.52	49	0	1639.0219	2	820.0123291	-4.685561582	66.41384333
25306272	MRPL11	39S ribosomal protein L11, mitochondrial isoform b	NP_733934.1	7.23	1	1	1	166	18.2	9.73	0.869	0.657	0.666																
														DLSSEELAAFQK	1	2	1	N-Term(iTRAQ8plex); K12(iTRAQ8plex)	0.869	0.657	0.666	3.46	-	0	1946.06339	3	649.359314	-2.945770165	44.14888333
25306283	GFM2	ribosome-releasing factor 2, mitochondrial isoform 3	NP_733781.1	3.51	1	1	2	513	57.0	7.09	0.936	0.695	0.580																
														VNLIDTPGHVDFTLEVER	2	5	1	N-Term(iTRAQ8plex)	0.936	0.695	0.58	4.81	41	0	2358.25797	3	786.7575073	-1.17992916	42.40608167
25777612	PSMD3	26S proteasome non-ATPase regulatory subunit 3	NP_002800.2	16.29	6	6	25	534	60.9	8.44	0.709	1.263	1.472																
														LQLDSPEDAEFIVAK	2	1	1	N-Term(iTRAQ8plex); K15(iTRAQ8plex)	0.501	1.2	1.575	5.65	42	0	2283.2761	3	761.7635498	2.975927473	38.19269167
														HDADGQATLLNLLLR	13	1	1	N-Term(iTRAQ8plex)	0.723	0.938	0.924	5.57	59	0	1954.09952	3	652.0371704	-2.775358464	49.11933333
														DFLLPFLEEPMDTEADLQ FRPR	2	1	1	N-Term(iTRAQ8plex)	-	1.811	2.844	4.34	27	0	2983.5062	3	995.173584	-3.895616801	80.81816167
														SLMPYFLLTQAVR	2	1	1	N-Term(iTRAQ8plex)	0.757	1.938	2.005	4.29	43	0	1843.03972	2	922.0234985	-3.066048781	59.37714167
														AIQLEYSEAR	2	1	1	N-Term(iTRAQ8plex)	0.633	1.385	1.526	4.1	35	0	1483.80779	2	742.4075317	1.31793119	27.02207333
														VYEFLDKLDVVR	4	1	1	N-Term(iTRAQ8plex); K7(iTRAQ8plex)	-	3.231	3.309	2.97	26	1	2104.21938	4	526.8103027	-3.283993372	58.420795
25777615	PSMD7	26S proteasome non-ATPase regulatory subunit 7	NP_002802.2	16.67	3	3	10	324	37.0	6.77	0.838	1.333	1.523																
														VVVHPLVLLSVVDHFNR	8	1	1	N-Term(iTRAQ8plex)	0.926	1.303	1.452	5.34	62	0	2247.32192	4	562.5858765	-2.801254797	52.38072667
														DLGLPTEAYISVEEVHDDG TPTSK	1	1	1	N-Term(iTRAQ8plex); K24(iTRAQ8plex)	-	-	-	3.39	-	0	3181.62197	4	796.1609497	-4.460728526	38.01419
														DIKDTTVGTLSQR	1	1	1	N-Term(iTRAQ8plex); K3(iTRAQ8plex)	0.758	1.363	1.596	3.08	-	1	2042.15696	3	681.3905029	-6.500852396	22.789235
25777713	SKP1	S-phase kinase-associated protein 1 isoform b	NP_733779.1	15.34	2	2	4	163	18.6	4.54	0.940	2.121	2.414																
														NDFTEEEEAQVR	2	1	1	N-Term(iTRAQ8plex)	0.848	1.959	2.198	4.23	44	0	1770.84819	2	885.9277344	1.903511557	24.67021667
														TDDIPVWDQEFLK	2	2	1	N-Term(iTRAQ8plex); K13(iTRAQ8plex)	1.042	2.297	2.65	3.78	30	0	2214.17319	3	738.729248	-7.732190709	35.883545
25777730	ALDH1B1	aldehyde dehydrogenase X, mitochondrial precursor	NP_000683.3	19.15	6	8	24	517	57.2	6.99	1.127	0.858	0.856																
														TFPTVNPTTGEVIGHVAE GDR	4	1	1	N-Term(iTRAQ8plex)	0.964	0.829	0.596	6.94	86	0	2501.28952	3	834.4346924	-1.730068239	33.61874167

														TIPMDGQHFCFTR	6	1	1	N-Term(iTRAQ8plex); C10(Methylthio)	1.127	0.858	0.856	4.56	46	0	1902.88913	3	634.9701538	-0.406813749	31.57918167
														VLGYIQLGQK	1	1	1	N-Term(iTRAQ8plex); K10(iTRAQ8plex)	1.18	1.114	0.968	3.73	-	0	1727.06815	3	576.3609009	0.328931269	63.23967667
														IAKEEIFGPVQPLFK	2	1	1	N-Term(iTRAQ8plex); K3(iTRAQ8plex); K15(iTRAQ8plex)	1.287	1.078	1.02	3.16	22	1	2628.57639	3	876.8636475	-4.889402203	44.10791167
														YGLAAAVFTR	2	1	1	N-Term(iTRAQ8plex)	1.075	0.605	0.484	3.08	36	0	1372.78813	2	686.8977051	-0.675824424	41.44966333
														TFVEESIYNEFLER	2	1	1	N-Term(iTRAQ8plex)	1.22	0.668	1.006	3.04	41	0	2080.04532	3	694.0199585	-4.210995609	54.50302333
														YFAGWADK	2	2	2	N-Term(iTRAQ8plex); K8(iTRAQ8plex)	-	-	-	3.34	25	0	1565.85759	2	783.4324341	0.217289276	29.965195
														VTLELGGK	5	10	4	N-Term(iTRAQ8plex); K8(iTRAQ8plex)	-	-	-	3.03	32	0	1424.8792	2	712.9432373	-9.904917305	26.02754667
25777732	ALDH2	aldehyde dehydrogenase, mitochondrial isoform 1 precursor	NP_000681.2	5.61	1	3	10	517	56.3	7.05	1.456	0.450	0.504																
														TFVQEDIYDEFVER	2	2	1	N-Term(iTRAQ8plex)	1.456	0.45	0.504	4.05	53	0	2094.02886	2	1047.518066	-2.146631116	48.68862167
														VTLELGGK	5	10	4	N-Term(iTRAQ8plex); K8(iTRAQ8plex)	-	-	-	3.03	32	0	1424.8792	2	712.9432373	-9.904917305	26.02754667
														LADLIER	3	2	2	N-Term(iTRAQ8plex)	-	-	-	2.74	31	0	1133.67644	2	567.3418579	-5.964752776	36.70049333
26051229	MRPL28	39S ribosomal protein L28, mitochondrial	NP_006419.2	21.09	3	3	9	256	30.1	8.29	0.983	0.769	0.624																
														GLWGGEGWILGQIYANNDK	4	1	1	N-Term(iTRAQ8plex); K19(iTRAQ8plex)	0.598	0.791	0.799	4.73	38	0	2699.43509	3	900.4832153	-1.903566387	47.10504167
														ERVEDVPIPIYFPPESQR	1	1	1	N-Term(iTRAQ8plex)	2.934	2.271	1.242	4.52	-	1	2475.32663	3	825.7803955	3.244813565	38.85325
														TLDLIDEAYGLDFYILK	4	1	1	N-Term(iTRAQ8plex); K17(iTRAQ8plex)	1.304	0.581	0.514	4.19	65	0	2610.47344	2	1305.740356	7.931500217	64.93676833
26051235	NUP133	nuclear pore complex protein Nup133	NP_060700.2	3.11	2	2	4	1156	128.9	5.10	1.281	0.807	0.805																
														ALDLLEYIDEEEDININDLK	2	1	1	N-Term(iTRAQ8plex); K20(iTRAQ8plex)	1.081	0.554	0.806	6.89	65	0	2985.60001	3	995.871521	7.867242164	65.153655
														YMTQFADQNFSDFLFR	2	1	1	N-Term(iTRAQ8plex)	1.519	1.175	0.804	4.42	42	0	2334.12009	3	778.7115479	1.447656743	57.624435
														ILIWK	1	17	2	N-Term(iTRAQ8plex); K5(iTRAQ8plex)	-	-	-	-	16	0	1280.85743	3	427.6239929	1.845802444	34.13876833
26051240	TNFAIP2	tumor necrosis factor alpha-induced protein 2	NP_006282.2	5.20	2	2	2	654	72.6	6.46	2.299	2.098	1.051																
														QAAAAGPGTSGLAATRPR	1	2	1	N-Term(iTRAQ8plex)	2.234	1.837	0.907	2.94	-	0	1957.08194	3	653.0321655	-3.172053163	15.35476333
														KGQPSSAEPEDAAGSR	1	2	1	N-Term(iTRAQ8plex); K1(iTRAQ8plex)	2.367	2.395	1.219	2.72	-	1	2195.1489	3	732.3878174	-1.098969443	10.36377167
26638657	MRPL20	39S ribosomal protein L20, mitochondrial isoform 1	NP_060441.2	13.42	1	1	2	149	17.4	10.86	1.282	1.100	1.219																
														HEGFAAALGDGKEPEGIFSR	2	1	1	N-Term(iTRAQ8plex); K12(iTRAQ8plex)	1.282	1.1	1.219	5.55	33	1	2696.42129	4	674.8607788	-1.5004872	30.71702167
26667177	MRPL46	39S ribosomal protein L46, mitochondrial	NP_071446.2	2.51	1	1	1	279	31.7	7.05	0.869	0.609	0.382																
														NLVLLVR	1	1	1	N-Term(iTRAQ8plex)	0.869	0.609	0.382	-	20	0	1130.7531	2	565.880188	-2.831691056	25.16334833
27151736	DNAJB2	dnaJ homolog subfamily B member 2 isoform b	NP_006727.2	11.73	2	3	8	324	35.6	5.95	0.570	0.400	0.480																
														SVSTSTTFVQGR	2	2	1	N-Term(iTRAQ8plex)	0.425	0.279	0.564	4.3	31	0	1573.85124	2	787.4292603	1.577396734	22.874845
														AQHQDPGLGGTQEGAR	2	1	1	N-Term(iTRAQ8plex)	0.764	0.574	0.408	3.38	26	0	1925.97159	3	642.6620483	-0.81492919	10.17385167
														VEVEEDGQLK	4	7	2	N-Term(iTRAQ8plex); K10(iTRAQ8plex)	-	-	-	4.05	52	0	1753.9625	3	585.3256836	-9.546252428	29.382285
27436889	POFUT1	GDP-fucose protein O-fucosyltransferase 1 isoform 1 precursor	NP_056167.1	12.11	4	4	7	388	43.9	8.53	0.975	0.741	1.152																
														YMVWSDEMVK	2	1	1	N-Term(iTRAQ8plex); K10(iTRAQ8plex)	1.044	1.109	1.261	4.22	42	0	1895.96701	3	632.6605225	-9.798254426	61.07264667
														VISLEDFMEK	2	2	1	N-Term(iTRAQ8plex); K10(iTRAQ8plex)	0.915	0.759	1.196	4.21	36	0	1819.00994	2	910.008606	-1.78998799	39.33090667
														VAYCFEVAAQR	2	2	1	N-Term(iTRAQ8plex); C4(Methylthio)	0.954	0.722	0.92	4.13	26	0	1606.8134	2	803.9103394	6.876835184	45.894425
														FGNQADHFLGSLAFAK	1	2	1	N-Term(iTRAQ8plex); K16(iTRAQ8plex)	0.997	0.413	1.109	2.74	-	0	2331.2643	4	583.5715332	-2.714564579	46.022865
27436946	LMNA	lamin isoform A	NP_733821.1	56.17	2	35	423	664	74.1	7.02	0.455	0.314	0.292																
														SVGGSGGGSFGDNLVTR	2	5	1	N-Term(iTRAQ8plex)	0.306	0.5	0.252	9.13	121	0	1870.9603	3	624.3249512	2.233593959	26.66578333
														TVLCGTCGQPADK	4	6	1	N-Term(iTRAQ8plex); C4(Methylthio); C7(Methylthio); K13(iTRAQ8plex)	0.484	0.252	0.299	4.1	42	0	1992.97721	3	664.9972534	-3.189347109	43.85702833
														AGQVVTIWAAGAGATHSPPTDLVWK	31	8	2	N-Term(iTRAQ8plex); K25(iTRAQ8plex)	-	-	-	6.34	57	0	3141.72915	4	786.1877441	-0.472547896	43.57742333
														IDSLSAQLSQLQK	6	7	2	N-Term(iTRAQ8plex); K13(iTRAQ8plex)	-	-	-	6.17	59	0	2039.19097	2	1020.099121	-2.327262419	33.64147833
														NSNLVGAAHEELQQSR	27	7	2	N-Term(iTRAQ8plex)	-	-	-	5.79	85	0	2057.06499	2	1029.036133	-1.357417006	22.37199667
														MQQQLDEYQELLDIK	16	8	2	N-Term(iTRAQ8plex); K15(iTRAQ8plex)	-	-	-	5.67	86	0	2502.32378	2	1251.665527	-5.304362535	73.416185
														ITESEEVVSR	13	4	2	N-Term(iTRAQ8plex)	-	-	-	5.34	67	0	1452.78806	3	484.9342041	2.263065605	24.38489333
														TLEGELHDLR	49	7	2	N-Term(iTRAQ8plex)	-	-	-	5.33	72	0	1486.81171	3	496.2755127	-3.199102584	23.654935
														LALDMEIHAYR	30	8	2	N-Term(iTRAQ8plex)	-	-	-	5.13	76	0	1635.87687	3	545.9638672	-3.669583546	32.87996833
														TALINSTGEEVAMR	10	7	2	N-Term(iTRAQ8plex)	-	-	-	5.03	62	0	1795.95865	3	604.651123	-4.807231513	29.81118
														MQQQLDEYQELLDIK	15	8	2	N-Term(iTRAQ8plex); M1(Oxidation); K15(iTRAQ8plex)	-	-	-	4.91	50	0	2518.33315	2	1251.665527	-5.304362535	73.416185
														VAVEEVDEEGK	6	8	2	N-Term(iTRAQ8plex); K11(iTRAQ8plex)	-	-	-	4.88	59	0	1811.98064	2	906.4939575	-2.25878845	29.31310667
														LQTMKEELDFQK	3	7	2	N-Term(iTRAQ8plex); K5(iTRAQ8plex); K12(iTRAQ8plex)	-	-	-	4.8	44	1	2422.37758	2	1211.690552	-1.686116457	36.74179333
														EAALSTALSEK	11	7	2	N-Term(iTRAQ8plex); K11(iTRAQ8plex)	-	-	-	4.77	51	0	1727.99419	2	864.5007324	-3.346698901	42.82938833
														LKDLEALLNSK	14	7	2	N-Term(iTRAQ8plex); K2(iTRAQ8plex); K11(iTRAQ8plex)	-	-	-	4.73	37	1	2156.34316	2	1078.674072	-0.418945243	50.611525
														LEAALGEAK	14	7	2	N-Term(iTRAQ8plex); K9(iTRAQ8plex)	-	-	-	4.38	51	0	1509.91047	3	503.9750061	0.50583168	38.65092667
														TALINSTGEEVAMR	5	7	2	N-Term(iTRAQ8plex); M13(Oxidation)	-	-	-	4.37	64	0	1811.93882	3	604.651123	-4.807231513	29.81118
														AAYEAELGDAR	16	4	2	N-Term(iTRAQ8plex)	-	-	-	4.34	58	0	1469.76225	2	735.3789673	-2.145499762	22.40932833
														SGAQASSTPLSPTR	6	4	2	N-Term(iTRAQ8plex)	-	-	-	4.33	54	0	1663.89067	2	832.4489746	-0.618250506	16.90638167
														LALDMEIHAYR	10	8	2	N-Term(iTRAQ8plex); M5(Oxidation)	-	-	-	4.31	55	0	1651.87461	3	545.9638672	-3.669583546	32.87996833
														QNGDDPLLTYR	2	8	2	N-Term(iTRAQ8plex)	-	-	-	3.7	42	0	1595.82915	2	798.4182129	-2.48280886	19.22656833
														LRDLEDSLAR	42	8	2	N-Term(iTRAQ8plex)	-	-	-	3.62	64	1	1491.84006	3	497.9511414	-2.969809824	38.74856833
														LQEKEDLQELNDR	4	4	2	N-Term(iTRAQ8plex); K4(iTRAQ8plex)	-	-	-	3.59	29	1	2238.20717	4	560.3076782	-4.364333217	22.5758
														LQLELSK	13	4	2	N-Term(iTRAQ8plex); K7(iTRAQ8plex)	-	-	-	3.55	35	0	1438.90959	2	719.9574585	-0.911057649	35.984405
														EGDLIAAQAR	8	7	2	N-Term(iTRAQ8plex)	-	-	-	3.54	49	0	1347.74952	3	449.9213562	-2.898124941	15.81620333
														SLETENAGLR	5	4	2	N-Term(iTRAQ8plex)	-	-	-	3.46	43	0	1393.75559	3	465.2567139	-2.369818973	17.995785
														LADALQELR	17	7	2	N-Term(iTRAQ8plex)	-	-	-	3.25	40	0	1332.77532	2	666.8912964	-2.689985716	44.88191167
														SNEDQSMGNWQIK	2	8	2	N-Term(iTRAQ8plex); K13(iTRAQ8plex)	-	-	-	3.19	26	0	2145.08622	3	715.7002563	0.323759933	21.69900833
														AQNTWGCGNSLR	3	8	2	N-Term(iTRAQ8plex); C7(Methylthio)	-	-	-	3.14	43	0	1656.78179	2	828.8945313	-4.253076694	20.70826167
														QLQDEMLR	1	7	2	N-Term(iTRAQ8plex)	-	-	-	2.96	-	0	1336.71367	2	668.8604736	-4.489109753	22.99892167
														LAVYIDR	10	4	2	N-Term(iTRAQ8plex)	-	-	-	2.89	62	0	1153.68875	3	385.234436	0.419138582	32.518365
														NIYSEELR	8	7	2	N-Term(iTRAQ8plex)	-	-	-	2.86	32	0	1327.70964	2	664.3584595	-4.744241125	16.52615333
														DLEDSLAR	3	8	2	N-Term(iTRAQ8plex)	-	-	-	2.8	31	0	1222.65495	2	611.8303833	-3.779745018	16.11086167
														VAVEEVDEEGKFVR	2	8	2	N-Term(iTRAQ8plex); K11(iTRAQ8plex)	-	-	-	2.71	35	1	2214.22353	4	554.3113403	0.379432389	26.61114167
														LALDMEIHAYR	5	8	2	N-Term(iTRAQ8plex); Y10(iTRAQ8plex)	-	-	-	-	51	0	1940.07561	3	545.9638672	-3.669583546	32.87996833
														AAYEAELGDAR	1	4	2	N-Term(iTRAQ8plex); Y3(iTRAQ8plex)	-	-	-	-	39	0	1773.95232	2	735.3789673	-2.145499762	22.40932833
														RVDAENR	1	7	2	N-Term(iTRAQ8plex)	-	-	-	-	38	1	1163.63999	3	388.5515137	-2.985059307	7.35876
														LVEIDNGK	1	7	2	N-Term(iTRAQ8plex); K8(iTRAQ8plex)	-	-	-	-	28	0	1495.89751	2	748.4523926	2.317830086	18.8729
														LALDMEIHAYR	1	8	2	N-Term(iTRAQ8plex); M5(Oxidation); Y10(iTRAQ8plex)	-	-	-	-	24	0	1956.07424	3	545.9638672	-3.669583546	32.87996833
														EMAEMR	1	8	2	N-Term(iTRAQ8plex)	-	-	-	-	17	0	1070.52471	2	535.7659912	-2.742111517	7.418713333
														LLEGEEER	5	11	4	N-Term(iTRAQ8plex)	-	-	-	2.96	37	0	1278.68534	3	426.8999634	0.786422343	21.17209167
27477134	NUP210	nuclear pore membrane glycoprotein 210 precursor	NP_079199.2	2.76	3	3	4	1887	205.0	6.81	1.582	3.762	3.958																
														VSPEEVTIYNHPGIQAELR	2	4	1	N-Term(iTRAQ8plex)	1.178	1.978	2.179	6.51	24	0	2456.29538	3	819.4366455	-5.442353006	32.26886
														WLSTRPEVASIEPLGLDEQQCSQK	1	4	1	N-Term(iTRAQ8plex); C21(Methylthio); K24(iTRAQ8plex)	-	-	-	3.94	-	0	3368.72939	4	842.9378052	-4.328479727	44.58166167
														DGAASLSYR	1	4	1	N-Term(iTRAQ8plex)	2.126	7.158	7.19	-	30	0	1243.65959	2	622.3334351	0.934497827	20.35400667
27477136	ZC3HAV1	zinc finger CCCH-type antiviral protein 1 isoform 1	NP_064504.2	7.65	5	5	9	902	101.4	8.40	1.445	0.951	1.407																
														ASLEDAPVDDLTR	2	4	1	N-Term(iTRAQ8plex)	1.323	2.006	1.475	4.71	45	0	1705.89556	2	853.451416	2.640320643	29.45675
														ATDLGGTSQAGTSQR	2	4	1	N-Term(iTRAQ8plex)	2.173	0.914	1.407	4.58	42	0	1753.88669	3	585.300415	-6.590752337	16.21307667
														LHICDHFTR	2	4	1	N-Term(iTRAQ8plex); C4(Methylthio)	3.265	7.924	2.917	4.31	33	0	1491.76308	4	373.696228	8.607217546	31.04297
														EHGLNPDVVQNIQDICNSK	2	4	1	N-Term(iTRAQ8plex); C16(Methylthio); K19(iTRAQ8plex)	0.654	0.951	0.803	4.27	40	0	2777.41623	3	926.4769287	-0.320231868	45.75541
														IENSELLDKFTWK	1	2	1	N-Term(iTRAQ8plex); K9(iTRAQ8plex); K13(iTRAQ8plex)	1.445	0.79	1.012	2.8	-	1	2535.4623	4	634.6210327	1.45917349	42.70285333
27501446	DENR	density-regulated protein	NP_003668.2	9.09	1	1	2	198	22.1	5.30	1.579	2.375	1.512																
														QEAGISEGQGTAGEEEEK	2	1	1	N-Term(iTRAQ8plex); K18(iTRAQ8plex)	1.579	2.375	1.512	2.79	39	0	2457.20212	3	819.7388916	-7.353788748	14.40086833
27545315	TACO1	translational activator of cytochrome c oxidase 1	NP_057444.2	10.10	2	2	4	297	32.5	8.13	1.305	0.769	0.827																
														EGGPNPEHNSNLANILEVCR	2	1	1	N-Term(iTRAQ8plex); C19(Methylthio)	1.192	0.785	0.966	4.81	37	0	2513.21372	3	838.4094238	-1.672784767	44.08131333
														GVIVVEVEDR	2	1	1	N-Term(iTRAQ8plex)	1.429	0.752	0.708	3.46	27	0	1418.81938	2	709.9133301	2.602330003	29.81320667
27597059	DNAJC9	dnaJ homolog subfamily C member 9	NP_056005.1	8.85	2	2	4	260	29.9	5.73	0.986	1.334	1.358																
														ISLEDIQAFEK	3	1	1	N-Term(iTRAQ8plex); K11(iTRAQ8plex)	0.954	1.299	1.23	3.03	28	0	1901.07108	3	634.3619995	-6.625745116	33.98389833
														ELGLDEGVDSLK	1	1	1	N-Term(iTRAQ8plex); K12(iTRAQ8plex)	0.988	1.628	1.709	-	24	0	1883.04178	3	628.3521118	-8.735388103	43.16288
27734984	HYPK	huntingtin-interacting protein K isoform 1	NP_057484.3	11.63	1	1	2	129	14.7	4.93	0.607	0.940	0.937																
														EHMGNVVEALIALTN	2	1	1	N-Term(iTRAQ8plex)	0.607	0.94	0.937	-	38	0	1915.02178	2	958.0145264	-2.263778762	52.66118
27735037	MMGT1	membrane magnesium transporter 1 precursor	NP_775741.1	16.03	1	1	4	131	14.7	9.16	-	-	-																
														GLVGIGLFALAHAAFSAAQHR	4	1	1	N-Term(iTRAQ8plex)	-	-	-	5.72	68	0	2411.36025	4	603.59552	-0.485660272	66.3469
27764882	P3H2	prolyl 3-hydroxylase 2 isoform a precursor	NP_060662.2	2.82	1	1	1	708	80.9	5.71	1.265	1.232	0.870																
														HPLPPPPGEGPGAELPLFR	1	1	1	N-Term(iTRAQ8plex)	1.265	1.232	0.87	2.82	-	0	2379.30869	3	793.7744141	-1.707370934	36.05232667
27777675	VN1R5	vomeronasal type-1 receptor 5	NP_776257.1	1.96	1	1	5	357	40.8	9.20	27.108	7.064	0.972																
														VIVFLNK	5	1	1	N-Term(iTRAQ8plex); K7(iTRAQ8plex)	27.108	7.064	0.972	3.22	26	0	[illegible]	2	[illegible]	[illegible]	[illegible]
28178825	IDH1	isocitrate dehydrogenase [NADP] cytoplasmic	NP_005887.2	24.64	7	8	17	414	46.6	7.01	[illegible]	[illegible]	[illegible]																
														[illegible]	2	1	1	N-Term(iTRAQ8plex)	[illegible]	[illegible]	[illegible]	[illegible]	64	0	[illegible]	2	[illegible]	[illegible]	[illegible]
														[illegible]	1	1	1	[illegible]	[illegible]	[illegible]	[illegible]	[illegible]	34	0	[illegible]	2	[illegible]	[illegible]	[illegible]
														TVEAEAAHGTVTR	2	1	1	N-Term(iTRAQ8plex)	[illegible]	[illegible]	[illegible]	[illegible]	36	0	[illegible]	3	[illegible]	[illegible]	[illegible]
														[illegible]	3	1	1	[illegible]	[illegible]	[illegible]	[illegible]	[illegible]	32	1	[illegible]	3	[illegible]	[illegible]	[illegible]
														[illegible]	2	1	1	[illegible]	[illegible]	[illegible]	[illegible]	[illegible]	28	0	[illegible]	3	[illegible]	[illegible]	[illegible]
														[illegible]	3	1	1	N-Term(iTRAQ8plex)	[illegible]	[illegible]	[illegible]	[illegible]	35	0	[illegible]	3	[illegible]	[illegible]	[illegible]
														[illegible]	3	1	1	[illegible]	[illegible]	[illegible]	[illegible]	[illegible]	[illegible]	0	[illegible]	3	[illegible]	[illegible]	[illegible]
														[illegible]	1	4	2	N-Term(iTRAQ8plex)	-	-	-	-	24	0	[illegible]	2	[illegible]	[illegible]	[illegible]
28178838	IDH3G	isocitrate dehydrogenase [NAD] subunit gamma, mitochondrial isoform b precursor	NP_777358.1	[illegible]	2	2	3	388	[illegible]	[illegible]	[illegible]	[illegible]	[illegible]																
														[illegible]	2	2	1	[illegible]	-	-	-	[illegible]	34	0	[illegible]	4	[illegible]	[illegible]	[illegible]
														[illegible]	1	2	1	[illegible]	[illegible]	[illegible]	[illegible]	[illegible]	-	0	[illegible]	4	[illegible]	[illegible]	[illegible]

28195394	HIST2H2AB	histone H2A type 2-B	NP_778235.1	40.77	3	6	56	130	14.0	10.89	1.216	1.049	1.294																
														LLGGVTIAQGGVLPNIQAVLLPK	2	3	1	N-Term(iTRAQ8plex); K23(iTRAQ8plex)	-	-	-	5.6	25	0	2879.77431	4	720.6990356	-5.694405219	65.308295
														NDEELNKLLGGVTIAQGGVLPNIQAVLLPK	1	3	1	N-Term(iTRAQ8plex); K7(iTRAQ8plex); K30(iTRAQ8plex)	-	-	-	3.57	-	1	4026.37204	3	1342.795532	-0.267702612	63.33524667
														HLQLAVR	11	1	1	N-Term(iTRAQ8plex)	1.216	1.049	1.294	3.22	34	0	1140.70978	3	380.9081116	-5.00550911	21.76283167
														NDEELNK	7	13	3	N-Term(iTRAQ8plex); K7(iTRAQ8plex)	-	-	-	3.69	35	0	1469.80754	2	735.4074097	1.314060434	12.628885
														KGNYAER	1	9	3	N-Term(iTRAQ8plex); K1(iTRAQ8plex)	-	-	-	2.94	-	1	1445.81789	4	362.2099304	-9.821084933	10.03500667
														AGLQFPVGR	34	17	4	N-Term(iTRAQ8plex)	-	-	-	3.56	51	0	1248.73304	3	416.91745	0.925177022	28.85471333
28269681	NDUFA11	NADH dehydrogenase [ubiquinone] 1 alpha subcomplex subunit 11 isoform 1	NP_783313.1	10.64	1	1	1	141	14.8	8.72	0.739	1.019	1.537																
														VTLNPPGTFLEGVAK	1	2	1	N-Term(iTRAQ8plex); K15(iTRAQ8plex)	0.739	1.019	1.537	2.99	-	0	2151.26859	3	717.7610474	2.402089572	37.892445
28302131	HBG1	hemoglobin subunit gamma-1	NP_000550.2	12.93	2	2	16	147	16.1	7.20	0.214	0.218	0.188																
														LLVVYPWTQR	13	6	1	N-Term(iTRAQ8plex)	0.12	0.08	0.135	3.53	48	0	1578.92905	3	526.9812012	-1.216669219	48.94020667
														LHVDPENFK	3	3	1	N-Term(iTRAQ8plex); K9(iTRAQ8plex)	0.26	0.229	0.229	3.35	36	0	1706.96701	4	427.4960327	-3.690954757	22.06481667
28372533	TMEM167A	protein kish-A precursor	NP_777569.1	27.78	2	2	2	72	8.1	8.95	0.565	0.667	0.572																
														NKTGLLGIFWK	2	1	1	N-Term(iTRAQ8plex); K2(iTRAQ8plex); K11(iTRAQ8plex)	0.493	0.583	0.505	2.75	19	1	2189.35952	4	548.0953369	0.984980417	42.27683667
														SLAPSLLDR	1	1	1	N-Term(iTRAQ8plex)	0.646	0.763	0.65	2.68	-	0	1275.75652	2	638.381897	-0.716417808	28.05994833
28416940	SBDS	ribosome maturation protein SBDS	NP_057122.2	3.60	1	1	1	250	28.7	8.75	1.318	1.446	1.046																
														DIATIVADK	1	1	1	N-Term(iTRAQ8plex); K9(iTRAQ8plex)	1.318	1.446	1.046	3.14	-	0	1553.92124	3	518.6452637	-9.442907413	39.76475667
28872725	PSMD11	26S proteasome non-ATPase regulatory subunit 11	NP_002806.2	17.54	5	5	11	422	47.4	6.48	0.869	1.371	1.338																
														DIQENDEEAVQVK	2	1	1	N-Term(iTRAQ8plex); K13(iTRAQ8plex)	0.871	1.536	1.579	5.52	50	0	2125.10776	3	709.0407715	-7.336144152	27.97833667
														EQSILELGSLLAK	4	1	1	N-Term(iTRAQ8plex); K13(iTRAQ8plex)	0.81	1.927	2.229	5.09	67	0	2009.2	2	1005.103638	-5.13265448	45.42188167
														YQEALHLGSQLLR	3	1	1	N-Term(iTRAQ8plex)	0.899	1.152	1.138	4.04	30	0	1832.02549	3	611.3466797	-4.216910496	46.99053333
														TGQAAELGGLLKYVRPFLNSISK	1	1	1	N-Term(iTRAQ8plex); K12(iTRAQ8plex); K23(iTRAQ8plex)	-	-	-	3.84	-	1	3375.00318	5	675.8064575	3.087341581	61.938795
														LYDNLLEQNLIR	1	1	1	N-Term(iTRAQ8plex)	0.546	0.777	0.847	3.08	-	0	1808.01641	2	904.5118408	-3.084197333	48.67143667
28872734	MRPL43	39S ribosomal protein L43, mitochondrial isoform a	NP_115488.2	7.55	1	1	2	159	17.8	9.88	0.920	0.469	0.502																
														FLASVLHNGLGR	2	6	1	N-Term(iTRAQ8plex)	0.92	0.469	0.502	4.14	43	0	1587.92319	3	529.979248	-2.588544206	46.53814333
28933465	STX12	syntaxin-12	NP_803173.1	5.43	1	1	1	276	31.6	5.59	1.037	0.749	0.978																
														DFSSIIQTCSGNIQR	1	1	1	N-Term(iTRAQ8plex); C9(Methylthio)	1.037	0.749	0.978	3.56	-	0	2018.99686	3	673.6704712	1.346225084	47.491925
29171702	PPA2	inorganic pyrophosphatase 2, mitochondrial isoform 1 precursor	NP_789845.1	19.46	5	5	12	334	37.9	7.39	0.264	0.262	0.358																
														NDEYENLFNMIVEIPR	4	2	1	N-Term(iTRAQ8plex)	0.198	0.236	0.315	5.49	101	0	2300.16509	2	1150.586182	5.036752065	60.47198
														ILSCGEVIHVK	3	1	1	N-Term(iTRAQ8plex); C4(Methylthio); K11(iTRAQ8plex)	0.526	0.485	0.478	3.74	33	0	1852.06162	4	463.7716064	0.050538869	37.81207333
														ILGILALIDEGETDWK	2	3	1	N-Term(iTRAQ8plex); K16(iTRAQ8plex)	-	-	-	3.42	49	0	2394.38651	3	798.800354	5.177719501	67.95420833
														AFALEVIK	2	4	1	N-Term(iTRAQ8plex); K8(iTRAQ8plex)	0.243	0.253	0.327	3.16	37	0	1498.94419	3	500.3195801	-0.776757157	49.86537333
														FKPGYLEATLNWFR	1	4	1	N-Term(iTRAQ8plex); K2(iTRAQ8plex)	0.288	0.272	0.391	3.06	-	0	2350.32602	3	784.1135254	3.906531173	55.96958833
29171745	PLPP2	phospholipid phosphatase 2 isoform 2	NP_803545.1	6.47	1	1	1	232	26.1	8.16	1.940	1.128	1.114																
														LRPNFLAVCDPDWSR	1	4	1	N-Term(iTRAQ8plex); C9(Methylthio)	1.94	1.128	1.114	2.91	-	0	2139.08127	3	713.6986084	1.449054486	52.98342167
29557855	PPM1A	protein phosphatase 1A isoform 2	NP_808820.1	3.70	1	1	3	324	35.9	5.78	0.378	0.714	0.706																
														IQNAGGSVMIQR	1	13	1	N-Term(iTRAQ8plex)	0.153	0.277	0.292	3.43	-	0	1577.87639	3	531.9621582	2.161440533	22.77916333
														IQNAGGSVMIQR	2	13	1	N-Term(iTRAQ8plex); M9(Oxidation)	0.932	1.839	1.707	3.27	32	0	1593.87192	3	531.9621582	2.161440533	22.77916333
29570791	CSNK2A1	casein kinase II subunit alpha isoform a	NP_808227.1	22.25	5	5	7	391	45.1	7.74	1.084	0.968	0.939																
														VLGTEDLYDYIDKYNIELDPR	2	2	1	N-Term(iTRAQ8plex); K13(iTRAQ8plex)	-	-	0.748	4.03	28	1	3152.65908	4	788.9202271	-0.682908244	55.46375667
														YSEVFEAINITNNEK	2	2	1	N-Term(iTRAQ8plex); K15(iTRAQ8plex)	0.97	0.968	1.005	3.57	38	0	2379.25248	3	793.7556763	-5.369645104	36.70775833
														EPFFHGHDNYDQLVR	1	2	1	N-Term(iTRAQ8plex)	1.368	0.904	0.877	3.56	-	0	2178.06314	3	726.6925659	-1.789110708	26.80252333
														DVKPHNVMIDHEHR	1	3	1	N-Term(iTRAQ8plex); K3(iTRAQ8plex); M8(Oxidation)	1.084	1.415	1.428	3.14	-	0	2351.2399	3	784.4181519	-4.254769402	11.853475
														EYWDYESHVVEWGNQDDYQLVR	1	2	1	N-Term(iTRAQ8plex); Y5(iTRAQ8plex)	-	-	-	-	20	0	3438.63613	4	860.4144897	-3.673606381	39.40552667
29725609	EGFR	epidermal growth factor receptor isoform a precursor	NP_005219.2	23.39	21	21	94	1210	134.2	6.68	1.894	0.827	0.753																
														EILDEAYVMASVDNPHVCR	2	1	1	N-Term(iTRAQ8plex); C18(Methylthio)	1.67	1.082	1.649	6.5	78	0	2511.20066	3	837.7384033	0.885795777	49.20518167
														FSNNPALCNVESIQWR	4	4	1	N-Term(iTRAQ8plex); C8(Methylthio)	1.852	0.821	0.716	6.15	91	0	2228.09551	2	1114.551392	2.717245786	44.27983667
														LTQLGTFEDHFLSLQR	6	4	1	N-Term(iTRAQ8plex)	2.421	0.603	0.54	6.05	66	0	2209.19748	3	737.0706787	2.517467477	52.60216667
														VLGSGAFGTVYK	2	16	1	N-Term(iTRAQ8plex); K12(iTRAQ8plex)	2.989	0.963	0.679	4.94	61	0	1807.04936	2	904.0283203	-4.458812892	46.48432167
														ATGQVCHALCSPEGCWGPEPR	10	3	1	N-Term(iTRAQ8plex); C6(Methylthio); C10(Methylthio); C15(Methylthio)	4.625	1.601	1.304	4.91	38	0	2640.13218	3	880.7155762	2.254040794	50.64966
														ACGADSYEMEEDGVR	2	4	1	N-Term(iTRAQ8plex); C2(Methylthio)	2.774	1.439	0.818	4.75	57	0	1981.82378	2	991.4155273	-0.304887098	28.43201333
														EISDGDVIISGNK	4	3	1	N-Term(iTRAQ8plex); K13(iTRAQ8plex)	2.608	0.769	0.548	4.73	48	0	1955.0855	2	978.0463867	-2.605759445	33.74400167
														TDLHAFENLEIIR	14	3	1	N-Term(iTRAQ8plex)	1.279	0.64	0.569	4.62	48	0	1875.02488	3	625.678833	-3.128244219	39.47366333
														NLQEILHGAVR	16	4	1	N-Term(iTRAQ8plex)	1.75	0.778	0.759	4.6	63	0	1553.90055	3	518.6384888	-3.634878749	28.416195
														NLCYANTINWK	2	3	1	N-Term(iTRAQ8plex); C3(Methylthio); K11(iTRAQ8plex)	1.659	1.216	1.183	4.45	36	0	1994.04087	3	665.3518066	-1.97870976	37.976525
														NYVVTDHGSCVR	2	4	1	N-Term(iTRAQ8plex); C10(Methylthio)	1.266	0.886	0.953	4.37	27	0	1699.80814	3	567.274231	-6.849407668	23.65594
														MHLPSPTDSNFYR	6	1	1	N-Term(iTRAQ8plex)	1.131	1.003	1.457	4.26	31	0	1868.91739	3	623.6439819	-4.98082433	28.84295
														YSFGATCVK	3	4	1	N-Term(iTRAQ8plex); C7(Methylthio); K9(iTRAQ8plex)	1.738	0.621	0.545	4.2	36	0	1629.85271	2	815.4299927	-3.800078623	38.467065
														SPSDCCHNQCAAGCTGPR	2	4	1	N-Term(iTRAQ8plex); C5(Methylthio); C6(Methylthio); C10(Methylthio); [illegible]	1.442	0.898	0.91	3.51	18	0	2294.82547	3	765.6133423	-3.626286455	31.543945
														MHLPSPTDSNFYR	1	1	1	N-Term(iTRAQ8plex); M1(Oxidation)	1.263	1.684	1.559	3.4	-	0	1884.91623	3	623.6439819	-4.98082433	28.84295
														GDSFTHTPPLDPQELDILK	1	4	1	N-Term(iTRAQ8plex); K19(iTRAQ8plex)	1.847	-	0.547	3.35	-	0	2731.46694	4	683.6221924	-3.441977867	41.68813
														YLVIQGDER	4	1	1	N-Term(iTRAQ8plex)	2.254	0.925	0.675	3.28	37	0	1396.77349	2	698.8903809	-0.226379763	28.39117
														ELIIEFSK	5	1	1	N-Term(iTRAQ8plex); K8(iTRAQ8plex)	2.943	0.762	0.696	3.23	37	0	1586.96573	3	529.6600952	2.735468349	55.75256
														GPDNCIQCAHYIDGPHCVK	2	3	1	N-Term(iTRAQ8plex); C5(Methylthio); C8(Methylthio); C17(Methylthio); [illegible]	2.288	0.771	0.801	3.15	19	0	2816.30068	4	704.8306274	9.837580846	40.78113833
														WMALESILHR	2	1	1	N-Term(iTRAQ8plex); M2(Oxidation)	1.942	2.22	-	2.95	52	0	1575.85874	3	525.9577637	-2.023363071	50.27973667
														CNLLEGEPR	2	3	1	N-Term(iTRAQ8plex); C1(Methylthio)	1.2	0.6	0.672	-	25	0	1380.68828	2	690.8477783	-2.5076474	34.21818167
														IICAQQCSGR	1	4	1	N-Term(iTRAQ8plex); C3(Methylthio); C7(Methylthio)	2.114	0.738	0.601	-	20	0	1474.68547	2	737.8463745	-5.825578893	25.208735
														ACGADSYEMEEDGVR	1	4	1	N-Term(iTRAQ8plex); C2(Methylthio); M9(Oxidation)	1.986	1.142	1.046	-	14	0	1997.8261	2	991.4155273	-0.304887098	28.43201333
29788785	TUBB	tubulin beta chain isoform b	NP_821133.1	70.50	4	22	545	444	49.6	4.89	0.824	1.465	2.206																
														ISVYYNEATGGK	18	3	1	N-Term(iTRAQ8plex); K12(iTRAQ8plex)	0.765	1.146	1.658	5.31	57	0	1910.03203	2	955.5203857	-7.573917245	41.42232667
														FWEVISDEHGIDPTGTYHGDSDLQLDR	16	3	1	N-Term(iTRAQ8plex)	-	1.481	2.674	4.96	36	0	3406.61386	3	1136.209473	0.244647261	47.845555
														ALTVPELTQQVFDAK	20	4	1	N-Term(iTRAQ8plex); K15(iTRAQ8plex)	1.051	2.36	3.542	4.85	78	0	2268.29441	2	1134.649902	-5.944221185	38.91148833
														MAVTFIGNSTAIQELFKR	1	5	1	N-Term(iTRAQ8plex); K17(iTRAQ8plex)	-	-	-	3.04	-	1	2634.49355	4	659.3788452	1.411914398	56.46140167
														AILVDLEPGTMDSVR	10	7	3	N-Term(iTRAQ8plex)	-	-	-	4.45	57	0	1920.02982	2	960.5213623	-3.121458321	44.53172
														AILVDLEPGTMDSVR	4	7	3	N-Term(iTRAQ8plex); M11(Oxidation)	-	-	-	2.95	45	0	1936.028	2	960.5213623	-3.121458321	44.53172
														EIVHIQAGQCGNQIGAK	24	9	4	N-Term(iTRAQ8plex); C10(Methylthio); K17(iTRAQ8plex)	-	-	-	6.74	73	0	2420.28691	2	1210.647095	-5.366911999	28.04771167
														EAESCDCLQGFQLTHSLGGGTGSGMGTLLISK	9	12	2	N-Term(iTRAQ8plex); C5(Methylthio); C7(Methylthio); K32(iTRAQ8plex)	-	-	-	5.55	34	0	3897.87046	3	1299.96167	-1.802723937	57.84804
														YLTVAAVFR	48	9	2	N-Term(iTRAQ8plex)	-	-	-	3.6	59	0	1343.80103	3	448.6051941	1.590804996	40.796475
														LHFFMPGFAPLTSR	105	21	4	N-Term(iTRAQ8plex)	-	-	-	5.26	73	0	1925.03666	3	642.350647	-1.861590873	50.37672667
														LHFFMPGFAPLTSR	9	21	4	N-Term(iTRAQ8plex); M5(Oxidation)	-	-	-	4.68	37	0	1941.02982	3	642.350647	-1.861590873	50.37672667
														NSSYFVEWIPNNVK	31	20	5	N-Term(iTRAQ8plex); K14(iTRAQ8plex)	-	-	-	4.24	49	0	2305.22641	2	1153.123413	-1.809680962	42.78834
														FPGQLNADLR	9	25	5	N-Term(iTRAQ8plex)	-	-	-	4.12	55	0	1434.7963	3	478.9369507	-3.068379269	31.15012333
														LAVNMVPFPR	13	25	5	N-Term(iTRAQ8plex)	-	-	-	4.01	51	0	1447.83405	3	483.2828674	-3.931565971	41.77579833
														LAVNMVPFPR	4	25	5	N-Term(iTRAQ8plex); M5(Oxidation)	-	-	-	3.45	44	0	1463.82685	3	483.2828674	-3.931565971	41.77579833
														KLAVNMVPFPR	15	25	5	N-Term(iTRAQ8plex); K1(iTRAQ8plex)	-	-	-	3.44	37	1	1880.12295	4	470.7861938	-9.108866745	31.42844
														FPGQLNADLRK	1	25	5	N-Term(iTRAQ8plex); K11(iTRAQ8plex)	-	-	-	3.07	-	1	1867.09755	4	467.5298462	-1.861935048	33.46670167
														NMMAACDPR	3	23	5	N-Term(iTRAQ8plex); C6(Methylthio)	-	-	-	-	55	0	1358.59062	2	679.7989502	-6.255401168	21.43194333
														NMMAACDPR	5	23	5	N-Term(iTRAQ8plex); M3(Oxidation); C6(Methylthio)	-	-	-	-	47	0	1374.58525	2	679.7989502	-6.255401168	21.43194333
														NMMAACDPR	1	23	5	N-Term(iTRAQ8plex); M2(Oxidation); M3(Oxidation); C6(Methylthio)	-	-	-	-	34	0	1390.5822	2	679.7989502	-6.255401168	21.43194333
														LTTPTYGDLNHLVSATMSGVTTCLR	95	12	3	N-Term(iTRAQ8plex); C23(Methylthio)	-	-	-	7.13	75	0	3001.51689	2	1501.262085	2.319161318	66.15877833
														SGPFGQIFRPDNFVFGQSGAGNNWAK	9	12	3	N-Term(iTRAQ8plex); K26(iTRAQ8plex)	-	-	-	5.17	30	0	3406.74965	4	852.442749	-1.486779919	46.41363167
														LTTPTYGDLNHLVSATMSGVTTCLR	14	12	3	N-Term(iTRAQ8plex); M17(Oxidation); C23(Methylthio)	-	-	-	5	31	0	3017.50541	2	1501.262085	2.319161318	66.15877833
														EVDEQMLNVQNK	4	9	3	N-Term(iTRAQ8plex); K12(iTRAQ8plex)	-	-	-	4.36	65	0	2055.08745	2	1028.047363	-6.160982347	33.78303333
														EVDEQMLNVQNK	2	9	3	N-Term(iTRAQ8plex); M6(Oxidation); K12(iTRAQ8plex)	-	-	-	4.18	46	0	2071.07792	2	1028.047363	-6.160982347	33.78303333
														TAVCDIPPR	12	17	3	N-Term(iTRAQ8plex); C4(Methylthio)	-	-	-	2.88	36	0	1321.68877	2	661.3473511	-2.713782013	26.90244667
														IREEYPDR	3	13	3	N-Term(iTRAQ8plex)	-	-	-	-	29	1	1381.73252	3	461.2490234	-3.788169878	16.81976833
														GHYTEGAELVDSVLDVVR	51	11	4	N-Term(iTRAQ8plex)	-	-	-	5.05	98	0	2263.18999	2	1132.098633	1.214084992	52.94817667
														GHYTEGAELVDSVLDVVR	10	11	4	N-Term(iTRAQ8plex); Y3(iTRAQ8plex)	-	-	-	-	64	0	2567.37212	2	1132.098633	1.214084992	52.94817667
														IMNTFSVVPSPK	18	11	3	N-Term(iTRAQ8plex); K12(iTRAQ8plex)	-	-	-	4.54	51	0	1928.10552	2	964.553772	-6.900042966	31.209235
														IMNTFSVVPSPK	2	11	3	N-Term(iTRAQ8plex); M2(Oxidation); K12(iTRAQ8plex)	-	-	-	3.64	37	0	1944.09331	2	964.553772	-6.900042966	31.209235
														EAESCDCLQGFQLTHSLGGGTGSGMGTLLLSK	5	12	2	N-Term(iTRAQ8plex); C5(Methylthio); C7(Methylthio); M25(Oxidation); [illegible]	-	-	-	4.96	30	0	3913.86538	4	975.2184448	-6.55075756	57.66156667
29789283	WDR75	WD repeat-containing protein 75 isoform 1	NP_115544.1	5.66	3	3	4	830	94.4	5.96	1.311	0.683	0.782																
														DVPESFTSEAYQWLNR	2	2	1	N-Term(iTRAQ8plex)	1.163	0.612	0.793	3.27	48	0	2246.10454	2	1123.555908	0.613723104	48.31274667
														ELSFVLDYINQSPK	1	2	1	N-Term(iTRAQ8plex); K14(iTRAQ8plex)	1.311	0.763	0.619	2.81	-	0	2261.26078	3	754.4251099	-1.333309359	46.55422667
														VYSTVTEECVHILHGHR	1	1	1	N-Term(iTRAQ8plex); Y2(iTRAQ8plex); C9(Methylthio)	1.439	-	0.782	-	29	0	2634.37818	5	527.6814575	1.548292377	47.65610667
29789409	NDUFAF2	mimitin, mitochondrial	NP_777549.1	20.12	2	2	5	169	19.8	8.97	0.729	0.460	0.353																
														EVDYEAGDIPTEWEAWIR	4	1	1	N-Term(iTRAQ8plex)	0.586	0.439	0.334	4.88	69	0	2483.20098	2	1242.104126	-0.93668088	56.74427167
														ETSEELLPPPVQTQIK	1	1	1	N-Term(iTRAQ8plex); K16(iTRAQ8plex)	1.278	0.763	0.864	4.49	-	0	2417.38218	3	806.4655762	3.042119571	31.92282333
29826282	PPM1G	protein phosphatase 1G	NP_817092.1	10.26	2	2	4	546	59.2	4.36	0.194	0.324	0.468																
														GTEAGQVGEPGIPTGEAGPSCSSASDK	2	1	1	N-Term(iTRAQ8plex); C21(Methylthio); K27(iTRAQ8plex)	0.194	0.324	0.468	6.97	77	0	3143.51511	3	1048.509888	2.187848856	27.71305167
														VADEDDVDNEEAALLHEEATMTIEELLTR	2	1	1	N-Term(iTRAQ8plex)	-	-	-	3.12	23	0	3575.75241	3	1192.588989	8.681591967	63.72153333
29826287	MRPL47	39S ribosomal protein L47, mitochondrial isoform a	NP_065142.2	12.00	3	3	13	250	29.4	10.37	0.904	0.605	0.481																
														NMLLTLEQEAK	1	1	1	N-Term(iTRAQ8plex); K11(iTRAQ8plex)	0.497	0.427	0.316	3.78	-	0	1898.08481	3	633.3664551	-1.548569172	64.20457167
														FFALPYVDHFLR	9	2	1	N-Term(iTRAQ8plex)	0.899	0.638	0.534	3.77	44	0	1829.01761	3	610.3440552	6.780130088	61.20842833
														LWYVLLK	2	1	1	N-Term(iTRAQ8plex); K7(iTRAQ8plex)	0.904	0.34	0.417	3.13	25	0	1542.99826	3	515.0042725	7.428934354	55.31351833

														FFALPYVDHFLR	1	2	1	N-Term(iTRAQ8plex); Y6(iTRAQ8plex)	0.987	0.609	0.493	-	30	0	2133.20913	3	610.3440552	6.780130088	61.20842833
30089997	ACTL6A	actin-like protein 6A isoform 2	NP_817126.1	4.91	1	1	2	387	43.2	6.14	1.064	1.796	1.598																
														ELFQEMNIELVPPYMIASK	2	2	1	N-Term(iTRAQ8plex); K19(iTRAQ8plex)	1.064	1.796	1.598	5.22	55	0	2860.56443	3	954.1929932	6.801085132	56.35542167
30240932	EHD1	EH domain-containing protein 1 isoform 1	NP_006786.2	11.24	3	3	4	534	60.6	6.83	1.028	0.948	1.539																
														IGPEPTTDSFIAVMHGPTEGVVPGNALVVDPR	1	2	1	N-Term(iTRAQ8plex)	1.719	0.743	1.847	5.04	-	0	3576.87265	3	1192.962402	1.432111721	44.48656667
														EHQISPGDFPSLR	1	2	1	N-Term(iTRAQ8plex)	0.615	1.209	1.283	4.07	-	0	1786.93064	3	596.3150635	-4.675122883	26.75276167
														LLDTVDDMLANDIAR	2	2	1	N-Term(iTRAQ8plex)	-	-	-	2.78	60	0	1979.04251	3	660.352356	0.173478382	64.79014167
30410792	PSME2	proteasome activator complex subunit 2	NP_002809.2	25.10	4	4	18	239	27.4	5.73	1.224	5.600	2.571																
														QNLFQEAEEFLYR	13	1	1	N-Term(iTRAQ8plex)	1.289	5.635	2.926	6.19	79	0	1991.02805	3	664.3475342	5.23246725	59.56130667
														IIYLNQLLQEDSLNVADLTSLR	2	1	1	N-Term(iTRAQ8plex)	-	-	-	3.61	63	0	2835.57969	3	945.8647461	0.946448892	69.52811833
														AFYAELYHIISSNLEK	2	2	1	N-Term(iTRAQ8plex); K16(iTRAQ8plex)	-	-	-	3.51	-	0	2506.37392	4	627.348938	-2.509293134	63.936025
														DEAAYGELR	1	2	1	N-Term(iTRAQ8plex)	1.097	2.3	1.621	-	28	0	1327.67778	2	664.3425293	-1.347711225	19.07166333
30410794	PSME3	proteasome activator complex subunit 3 isoform 1	NP_005780.2	11.02	2	2	10	254	29.5	5.95	1.595	4.491	3.883																
														ITSEAEDLVANFFPK	9	3	1	N-Term(iTRAQ8plex); K15(iTRAQ8plex)	1.877	7.619	6.268	5.04	72	0	2289.26438	2	1145.138916	5.165012594	61.80228667
														RLDECEEAFQGTK	1	4	1	N-Term(iTRAQ8plex); C5(Methylthio); K13(iTRAQ8plex)	1.355	2.019	1.8	3.89	-	1	2180.0784	3	727.3643188	-6.99470579	31.59672667
30794376	DOLPP1	dolichyldiphosphatase 1 isoform a	NP_065171.2	5.46	1	1	2	238	27.0	9.44	3.033	0.786	0.761																
														IAAWPVSEFFLIR	2	1	1	N-Term(iTRAQ8plex)	3.033	0.786	0.761	3.33	48	0	1853.05925	2	927.0332642	-1.888552503	67.446975
30795212	IGF2BP3	insulin-like growth factor 2 mRNA-binding protein 3	NP_006538.2	10.88	4	4	7	579	63.7	8.87	2.360	1.968	2.759																
														LYIGNLSENAAPSDLESIFK	2	2	1	N-Term(iTRAQ8plex); K20(iTRAQ8plex)	2.565	1.647	2.54	6.52	56	0	2789.5261	3	930.5135498	2.83018111	50.71844333
														MVIITGPPEAQFK	2	23	1	N-Term(iTRAQ8plex); K13(iTRAQ8plex)	0.897	1.08	1.278	5.73	37	0	2039.18351	3	680.399353	0.737811005	35.32564
														ILVPTQFVGAIIGK	2	19	1	N-Term(iTRAQ8plex); K14(iTRAQ8plex)	2.172	2.351	2.998	4.61	44	0	2064.30063	3	688.7717285	-1.709539332	57.42768333
														TGYAFVDCPDESWALK	2	2	1	N-Term(iTRAQ8plex); C8(Methylthio); K16(iTRAQ8plex)	4.343	2.878	3.703	3.53	44	0	2456.21147	2	1228.609375	1.136335381	44.34032833
30795231	BASP1	brain acid soluble protein 1	NP_006308.3	44.05	6	6	21	227	22.7	4.63	0.570	0.736	0.727																
														SDGAPASDSKPGSSEAAPSSK	2	1	1	N-Term(iTRAQ8plex); K10(iTRAQ8plex); K21(iTRAQ8plex)	-	-	1.071	5.24	29	0	2845.49404	4	712.1289673	-0.092089237	12.769205
														ESEPQAAAEPAEAK	8	1	1	N-Term(iTRAQ8plex); K14(iTRAQ8plex)	0.623	0.786	0.734	4.96	66	0	2036.06877	2	1018.538025	-3.392869339	13.83360833
														KTEAPAAPAAQETK	4	1	1	N-Term(iTRAQ8plex); K1(iTRAQ8plex); K14(iTRAQ8plex)	0.564	0.668	0.609	4.87	53	1	2325.34751	3	775.7873535	-2.852908266	17.07695
														ETPAATEAPSSTPK	3	1	1	N-Term(iTRAQ8plex); K14(iTRAQ8plex)	0.73	0.737	0.513	4.31	27	0	1995.08237	3	665.6989746	-1.569219357	15.579855
														AQGPAASAEEPKPVEAPAANSDQTVTVKE	2	1	1	N-Term(iTRAQ8plex); K12(iTRAQ8plex); K28(iTRAQ8plex)	-	-	-	3.72	31	1	3805.027	4	952.012207	-2.988955139	22.98726833
														GYNVNDEK	2	1	1	N-Term(iTRAQ8plex); K8(iTRAQ8plex)	0.72	0.823	0.745	2.93	34	0	1546.82781	2	773.9175415	-2.808917818	13.457265
31077094	CERS2	ceramide synthase 2	NP_071358.1	2.37	1	1	1	380	44.8	8.98	1.573	0.788	1.017																
														QVEVELLSR	1	2	1	N-Term(iTRAQ8plex)	1.573	0.788	1.017	3.53	-	0	1376.80278	2	688.9050293	-1.691837263	30.51321167
31317305	MPHOSPH10	U3 small nucleolar ribonucleoprotein protein MPP10	NP_005782.1	4.70	2	2	6	681	78.8	4.86	1.673	0.847	0.976																
														RPENSLLEETLHFDHAVR	2	1	1	N-Term(iTRAQ8plex)	-	-	-	5.13	28	0	2467.30268	5	494.2663574	1.252095821	34.67518333
														TAEEENPEHVEIQK	4	1	1	N-Term(iTRAQ8plex); K14(iTRAQ8plex)	1.673	0.847	0.976	4.26	31	0	2261.17812	4	566.0499878	-3.931582409	19.26129667
31377697	COLGALT1	procollagen galactosyltransferase 1 precursor	NP_078932.2	10.77	7	7	24	622	71.6	7.31	0.774	0.371	0.546																
														NAAHALPTTLGALER	10	1	1	N-Term(iTRAQ8plex)	0.849	0.608	0.936	4.73	50	0	1839.03423	2	920.0213623	-1.959348108	26.32652667
														EGLDWDLIYVGR	2	4	1	N-Term(iTRAQ8plex)	0.754	0.253	0.531	4.21	53	0	1739.94023	2	870.4737549	7.587528223	43.84132833
														ALQAQEIECR	2	4	1	N-Term(iTRAQ8plex); C9(Methylthio)	0.774	0.601	1.012	3.1	35	0	1510.75798	2	755.8826294	-5.288822586	20.52885667
														VLIALLAR	6	1	1	N-Term(iTRAQ8plex)	0.769	0.243	0.477	2.84	32	0	1172.80205	2	586.9046631	-1.033207714	53.921315
														FEIFFK	1	4	1	N-Term(iTRAQ8plex); K6(iTRAQ8plex)	0.736	0.271	0.501	2.77	-	0	1438.85198	2	719.9296265	-2.422063124	70.54919833
														TPAYIPIR	2	3	1	N-Term(iTRAQ8plex)	0.754	0.372	0.546	2.7	24	0	1234.74492	2	617.8760986	-0.972390617	25.71017667
														HPPAEPSR	1	4	1	N-Term(iTRAQ8plex)	0.879	0.434	0.518	-	23	0	1194.65458	3	398.8897095	1.074677146	9.638905
31542306	CHMP1B	charged multivesicular body protein 1b	NP_065145.2	3.02	1	1	1	199	22.1	8.10	1.461	2.016	1.721																
														HLFNLK	1	1	1	N-Term(iTRAQ8plex); K6(iTRAQ8plex)	1.461	2.016	1.721	2.98	-	0	1379.85505	3	460.6231995	-4.990496177	22.22805
31542331	CYR61	protein CYR61 precursor	NP_001545.2	15.75	2	2	3	381	42.0	8.21	-	-	-																
														HQCTCIDGAVGCIPLCPQESLPNLGCPNPR	2	1	1	N-Term(iTRAQ8plex); C3(Methylthio); C5(Methylthio); C12(Methylthio); [illegible]	-	-	-	3.87	43	0	3782.67514	3	1261.563232	4.456559177	65.26577333
														VTGQCCEEWVCDEDSIKDPMEDQDGLLGK	1	1	1	N-Term(iTRAQ8plex); C5(Methylthio); C6(Methylthio); C11(Methylthio); [illegible]	-	-	-	-	23	1	4292.93989	5	859.3937988	-0.646799035	56.639855
31542711	SLC35F6	solute carrier family 35 member F6 precursor	NP_060347.2	3.77	1	1	4	371	40.2	6.93	0.752	0.375	0.779																
														WADNFMAEGCGGSK	4	1	1	N-Term(iTRAQ8plex); C10(Methylthio); K14(iTRAQ8plex)	0.752	0.375	0.779	4.23	45	0	2126.9854	2	1063.996338	-3.029235063	65.24404167
31542947	HSPD1	60 kDa heat shock protein, mitochondrial	NP_002147.2	71.03	33	33	565	573	61.0	5.87	2.475	1.135	0.929																
														IMQSSSEVGYDAMAGDFVNMVEK	6	1	1	N-Term(iTRAQ8plex); K23(iTRAQ8plex)	3.288	1.408	0.938	8.83	98	0	3116.54917	3	1039.52124	9.364894169	51.79359833
														IMQSSSEVGYDAMAGDFVNMVEK	5	1	1	N-Term(iTRAQ8plex); M13(Oxidation); K23(iTRAQ8plex)	2.673	1.242	1.181	8.78	79	0	3132.53135	3	1039.52124	9.364894169	51.79359833
														DMAIATGGAVFGEEGLTLNLEDVQPHDLGK	16	1	1	N-Term(iTRAQ8plex); K30(iTRAQ8plex)	-	-	-	7.61	76	0	3705.92031	3	1241.312134	0.371264429	42.00310167
														ALMLQGVDLLADAVAVTMGPK	44	1	1	N-Term(iTRAQ8plex); K21(iTRAQ8plex)	3.406	1.205	1.192	6.96	98	0	2721.54961	2	1361.278442	-0.298783595	71.92074333
														KPLVIIAEDVDGEALSTLVLNR	39	1	1	N-Term(iTRAQ8plex); K1(iTRAQ8plex)	2.799	1.578	0.926	6.8	97	0	2973.73313	3	991.9190674	-0.639733086	59.51721167
														ISSIQSIVPALEIANAHR	57	1	1	N-Term(iTRAQ8plex)	1.619	1.132	1.076	6.74	79	0	2223.26822	2	1112.144409	2.343580548	49.07956
														ALMLQGVDLLADAVAVTMGPK	10	1	1	N-Term(iTRAQ8plex); M18(Oxidation); K21(iTRAQ8plex)	1.708	0.33	0.795	6.56	79	0	2737.54227	2	1361.278442	-0.298783595	71.92074333
														GVMLAVDAVIAELKK	6	1	1	N-Term(iTRAQ8plex); M3(Oxidation); K14(iTRAQ8plex); K15(iTRAQ8plex)	2.829	0.894	0.692	6.55	49	1	2485.5292	4	618.1396484	5.08296543	67.72831667
														QSKPVTTPEEIAQVATISANGDKEIGNIISDAMK	2	1	1	N-Term(iTRAQ8plex); K3(iTRAQ8plex); K23(iTRAQ8plex); K34(iTRAQ8plex)	-	-	-	6.39	-	1	4772.62708	6	798.9451904	-0.594503865	50.84216833
														GVMLAVDAVIAELKK	18	1	1	N-Term(iTRAQ8plex); K14(iTRAQ8plex); K15(iTRAQ8plex)	3.596	1.364	0.844	6.32	52	1	2469.51601	4	618.1396484	5.08296543	67.72831667
														QSKPVTTPEEIAQVATISANGDKEIGNIISDAMK	4	1	1	N-Term(iTRAQ8plex); K3(iTRAQ8plex); K23(iTRAQ8plex); M33(Oxidation); [illegible]	-	-	-	6.31	21	1	4788.63659	6	798.9451904	-0.594503865	50.84216833
														CIPALDSLTPANEDQK	14	1	1	N-Term(iTRAQ8plex); C1(Methylthio); K16(iTRAQ8plex)	3.377	1.664	1.231	6.21	61	0	2369.23491	2	1185.121094	2.008488047	38.37212167
														DMAIATGGAVFGEEGLTLNLEDVQPHDLGK	14	1	1	N-Term(iTRAQ8plex); M2(Oxidation); K30(iTRAQ8plex)	4.895	-	1.649	6.15	82	0	3721.94448	3	1241.312134	0.371264429	42.00310167
														GVMLAVDAVIAELK	20	1	1	N-Term(iTRAQ8plex); K14(iTRAQ8plex)	1.706	0.907	0.554	6.07	106	0	2037.22429	2	1019.115784	0.200990541	64.35522
														IQEIIEQLDVTTSEYEKEK	10	1	1	N-Term(iTRAQ8plex); K17(iTRAQ8plex); K19(iTRAQ8plex)	-	-	-	5.99	43	1	3207.756	4	802.694458	-6.353085689	78.57593833
														IQEIIEQLDVTTSEYEK	20	1	1	N-Term(iTRAQ8plex); K17(iTRAQ8plex)	2.496	1.081	0.839	5.88	82	0	2646.431	3	882.8151855	-0.924985148	65.0796
														TVIIEQSWGSPK	19	1	1	N-Term(iTRAQ8plex); K12(iTRAQ8plex)	2.18	1.027	0.898	5.88	60	0	1953.11614	2	977.0617065	-5.342659286	42.40523667
														VGEVIVTKDDAMLLK	4	1	1	N-Term(iTRAQ8plex); K8(iTRAQ8plex); K15(iTRAQ8plex)	5.629	2.578	1.64	5.79	42	1	2543.50625	4	636.632019	-7.21641952	38.89640333
														TLNDELEIIEGMKFDR	4	1	1	N-Term(iTRAQ8plex); K13(iTRAQ8plex)	7.083	3.403	2.429	5.54	49	1	2531.34926	4	633.5927734	-5.67579915	49.97167667
														TLNDELEIIEGMK	27	1	1	N-Term(iTRAQ8plex); K13(iTRAQ8plex)	2.682	1.148	1.085	5.51	73	0	2113.15019	2	1057.082153	-4.784916233	71.37882167
														GVMLAVDAVIAELK	6	1	1	N-Term(iTRAQ8plex); M3(Oxidation); K14(iTRAQ8plex)	2.19	1.68	1.319	5.5	38	0	2053.21909	2	1019.115784	0.200990541	64.35522
														AAVEEGIVLGGGCALLR	51	1	1	N-Term(iTRAQ8plex); C13(Methylthio)	2.087	1.224	0.949	5.37	79	0	1978.075	2	989.5411377	-0.906704435	57.53255
														NAGVEGSLIVEK	8	1	1	N-Term(iTRAQ8plex); K12(iTRAQ8plex)	2.171	0.882	0.778	5.35	62	0	1824.05657	2	912.5319214	-6.669281418	19.92123167
														KISSIQSIVPALEIANAHR	21	1	1	N-Term(iTRAQ8plex); K1(iTRAQ8plex)	1.957	0.858	0.909	5.28	48	1	2655.57046	4	664.6480713	-2.336983279	40.51846333
														CEFQDAYVLLSEKK	8	1	1	N-Term(iTRAQ8plex); C1(Methylthio); K13(iTRAQ8plex); K14(iTRAQ8plex)	3.149	1.712	1.128	5.22	32	1	2631.4125	4	658.6085815	-6.253678641	44.10720333
														CEFQDAYVLLSEK	45	1	1	N-Term(iTRAQ8plex); C1(Methylthio); K13(iTRAQ8plex)	2.563	1.137	0.923	5.09	79	0	2199.14702	2	1100.077148	8.365950448	47.22247333
														TLNDELEIIEGMKFDR	8	1	1	N-Term(iTRAQ8plex); M12(Oxidation); K13(iTRAQ8plex)	5.339	0.905	1.224	4.79	35	1	2547.36391	4	633.5927734	-5.67579915	49.97167667
														IMQSSSEVGYDAMAGDFVNMVEK	2	1	1	N-Term(iTRAQ8plex); M2(Oxidation); M13(Oxidation); M20(Oxidation); [illegible]	-	-	-	4.66	39	0	3164.50576	3	1039.52124	9.364894169	51.79359833
														GYISPYFINTSK	25	1	1	N-Term(iTRAQ8plex); K12(iTRAQ8plex)	2.581	1.166	1.047	4.63	55	0	1998.1043	2	999.5557861	-5.67820929	49.00323167
														TLNDELEIIEGMK	4	1	1	N-Term(iTRAQ8plex); M12(Oxidation); K13(iTRAQ8plex)	1.445	0.821	0.998	4.43	37	0	2129.15879	2	1057.082153	-4.784916233	71.37882167
														TALLDAAGVASLLTTAEVVVTEIPKEEK	5	1	1	N-Term(iTRAQ8plex); K25(iTRAQ8plex); K28(iTRAQ8plex)	-	-	-	4.42	45	1	3781.18299	5	757.0424194	-3.924867447	67.33561833
														ALMLQGVDLLADAVAVTMGPK	4	1	1	N-Term(iTRAQ8plex); M3(Oxidation); M18(Oxidation); K21(iTRAQ8plex)	-	-	-	4.4	59	0	2753.52701	2	1361.278442	-0.298783595	71.92074333
														VGGTSDVEVNEK	8	1	1	N-Term(iTRAQ8plex); K12(iTRAQ8plex)	1.993	0.773	0.688	4.29	66	0	1841.99993	2	921.5036011	-3.580766465	23.831745
														LKVGLQVVAVK	2	1	1	N-Term(iTRAQ8plex); K2(iTRAQ8plex); K11(iTRAQ8plex)	1.612	0.795	0.627	4.25	21	1	2066.38662	4	517.3521118	1.822546465	31.04716333
														VGLQVVAVK	8	1	1	N-Term(iTRAQ8plex); K9(iTRAQ8plex)	2.175	0.825	0.933	4.08	54	0	1520.9948	2	761.0010376	-2.401536607	21.81810667
														VGEVIVTK	8	1	1	N-Term(iTRAQ8plex); K8(iTRAQ8plex)	2.935	0.927	0.722	3.53	45	0	1452.92241	2	726.9648438	-1.51447867	32.15486833
														LVQDVANNTNEEAGDGTTTATVLAR	2	1	1	N-Term(iTRAQ8plex)	0.642	0.799	0.729	3.17	34	0	2864.43949	3	955.4846802	-5.070458887	36.36458667
														IGIEIIK	3	1	1	N-Term(iTRAQ8plex); K7(iTRAQ8plex)	2.519	0.928	0.723	3.06	24	0	1393.92569	3	465.3134155	1.299948799	42.20126
														TLKIPAMTIAK	1	1	1	N-Term(iTRAQ8plex); K3(iTRAQ8plex); K11(iTRAQ8plex)	3.566	1.472	1.108	2.96	-	1	2099.33548	3	700.4500122	-1.647154032	29.50382833
														IGIEIIKR	2	1	1	N-Term(iTRAQ8plex); K7(iTRAQ8plex)	3.031	1.321	0.89	2.92	19	1	1550.02675	4	388.262146	1.130822014	33.45968167
														GVITVK	1	1	1	N-Term(iTRAQ8plex); K6(iTRAQ8plex)	2.923	1.374	0.651	2.88	-	0	1224.81498	3	408.9431763	1.130177718	25.26530333
														KGVITVK	1	1	1	N-Term(iTRAQ8plex); K1(iTRAQ8plex); K7(iTRAQ8plex)	2.228	1.094	1.266	2.72	-	1	1657.1133	4	415.033783	-0.373748772	20.66251
														VTDALNATR	2	1	1	N-Term(iTRAQ8plex)	2.715	1.042	0.949	-	30	0	1264.71001	2	632.8586426	-4.967750478	16.63344833
														GYISPYFINTSK	1	1	1	N-Term(iTRAQ8plex); Y2(iTRAQ8plex); K12(iTRAQ8plex)	2.793	1.156	0.783	-	29	0	2302.32126	2	999.5557861	-5.67820929	49.00323167
31543415	GADD45GIP1	growth arrest and DNA damage-inducible proteins-interacting protein 1	NP_443082.2	4.95	1	1	2	222	25.4	10.02	-	-	-																
														FQELLQDLEKK	2	1	1	N-Term(iTRAQ8plex); K10(iTRAQ8plex); K11(iTRAQ8plex)	-	-	-	4	27	1	2303.38588	4	576.6019287	5.246044021	53.59223
31543831	TUBG1	tubulin gamma-1 chain	NP_001061.2	8.43	2	2	4	451	51.1	6.14	0.646	0.616	0.477																
														LHIQNPSFSQINQLVSTIMSASTTTLR	2	5	1	N-Term(iTRAQ8plex)	-	-	-	4.28	44	0	3291.78257	3	1097.932373	4.621991374	68.58954667
														IHEDIFDIIDR	2	3	1	N-Term(iTRAQ8plex)	0.646	0.616	0.477	3.38	33	0	1689.90635	3	563.9736328	-2.980113374	41.970385
31563378	EIF6	eukaryotic translation initiation factor 6 isoform a	NP_852133.1	21.63	3	3	19	245	26.6	4.68	1.720	1.411	1.407																
														HGLLVPNNTTDQELQHIR	10	1	1	N-Term(iTRAQ8plex)	2.056	1.893	1.407	6.52	86	0	2389.28464	3	797.0997314	-1.829088919	24.472815
														QTVADQVLVGSYCVFSNQGGLVHPK	7	2	1	N-Term(iTRAQ8plex); C13(Methylthio); K25(iTRAQ8plex)	1.071	-	-	5.41	46	0	3300.7311	4	825.9382324	-0.573355551	53.310905
														NSLPDTVQIR	2	1	1	N-Term(iTRAQ8plex)	1.69	1.135	1.41	2.97	-	0	1446.81267	2	723.9099731	-6.325373788	17.71462
31563512	NAA20	N-alpha-acetyltransferase 20 isoform b	NP_852668.1	9.04	1	1	2	166	18.8	5.00	1.241	2.456	1.973																
														SIIPLPHPVRPEDIE	2	2	1	N-Term(iTRAQ8plex)	1.241	2.456	1.973	3.01	27	0	2016.1362	3	672.7169189	-3.465893847	34.59658167
31652228	MRPL21	39S ribosomal protein L21, mitochondrial isoform a	NP_852616.1	8.33	1	1	6	120	13.7	10.07	1.217	0.555	0.637																
														LFAVVHFASR	6	3	1	N-Term(iTRAQ8plex)	1.217	0.555	0.637	4.39	61	0	1450.8433	3	484.287323	0.113343512	45.24049833
31712030	AUP1	ancient ubiquitous protein 1	NP_853553.1	6.59	2	2	4	410	45.8	8.65	1.340	0.756	0.683																
														QLGEANEEFALR	2	2	1	N-Term(iTRAQ8plex)	1.404	0.642	0.63	4.33	38	0	1680.89153	2	840.9494019	3.346908975	29.666665
														VKEVLPHVPLGVIQR	2	2	1	N-Term(iTRAQ8plex); K2(iTRAQ8plex)	1.278	0.891	0.74	4.2	24	1	2292.43247	3	764.8156738	-2.256583649	35.50579167
31982936	SGPL1	sphingosine-1-phosphate lyase 1	NP_003892.2	2.64	1	1	3	568	63.5	9.16	1.535	1.016	1.047																
														AFEPYLEILEVYSTK	3	1	1	N-Term(iTRAQ8plex); K15(iTRAQ8plex)	1.535	1.016	1.047	5.7	33	0	2410.34708	3	804.1205444	4.339105711	63.96657333
32189392	PRDX2	peroxiredoxin-2	NP_005800.3	10.61	1	2	10	198	21.9	5.97	0.962	4.696	0.880																
														ATAVVDGAFK	2	1	1	N-Term(iTRAQ8plex); K10(iTRAQ8plex)	0.962	4.696	0.88	4.53	47	0	1586.92851	2	793.9678955	-4.875395649	35.58892667
														QITVNDLPVGR	8	2	2	N-Term(iTRAQ8plex)	-	-	-	3.64	37	0	1515.87602	2	758.4416504	-2.412557948	28.69410333

32189394	ATP5B	ATP synthase subunit beta, mitochondrial precursor	NP_001677.2	72.21	23	23	487	529	56.5	5.40	0.697	0.474	0.517																
														GFQQILAGEYDHLPEQAFYMVGPIEEAVAK	19	1	1	N-Term(iTRAQ8plex); K30(iTRAQ8plex)	0.413	-	0.623	7.44	54	0	3959.02988	3	1320.348145	-5.350036976	83.57064
														FLSQPFQVAEVFTGHMGK	26	1	1	N-Term(iTRAQ8plex); K18(iTRAQ8plex)	0.697	0.488	0.552	6.87	73	0	2631.42826	3	877.81427	2.601442231	58.05099333
														TVLIMELINNVAK	78	1	1	N-Term(iTRAQ8plex); K13(iTRAQ8plex)	0.651	0.389	0.424	6.44	84	0	2066.24565	2	1033.626465	-2.302839691	85.35435
														QFAPIHAEAPEFMEMSVEQEILVTGIK	13	1	1	N-Term(iTRAQ8plex); K27(iTRAQ8plex)	-	0.697	0.736	6.3	56	0	3652.92441	3	1218.317261	4.267162446	50.42985833
														GFQQILAGEYDHLPEQAFYMVGPIEEAVAK	6	1	1	N-Term(iTRAQ8plex); M20(Oxidation); K30(iTRAQ8plex)	0.763	0.173	0.132	5.93	39	0	3975.03366	3	1320.348145	-5.350036976	83.57064
														DQEGQDVLLFIDNIFR	5	1	1	N-Term(iTRAQ8plex)	0.444	0.24	0.379	5.84	78	0	2226.19194	2	1113.599609	9.464965532	65.918395
														IMDPNIVGSEHYDVAR	8	1	1	N-Term(iTRAQ8plex)	0.704	0.451	0.63	5.83	79	0	2120.06797	3	707.3608398	-3.247546239	29.20971
														EGNDLYHEMIESGVINLK	14	1	1	N-Term(iTRAQ8plex); K18(iTRAQ8plex)	0.581	0.418	0.485	5.57	62	0	2669.40305	2	1335.203613	-2.46922815	43.92673667
														FTQAGSEVSALLGR	12	1	1	N-Term(iTRAQ8plex)	0.614	0.418	0.52	5.42	78	0	1739.95658	3	580.6570435	-1.623131832	48.52044333
														VALVYGQMNEPPGAR	17	1	1	N-Term(iTRAQ8plex)	0.764	0.597	0.611	5.33	54	0	1906.01291	2	953.5093384	-2.351067883	34.48544167
														FLSQPFQVAEVFTGHMGK	6	1	1	N-Term(iTRAQ8plex); M16(Oxidation); K18(iTRAQ8plex)	0.798	0.605	0.49	5.3	35	0	2647.42069	3	877.81427	2.601442231	58.05099333
														LVLEVAQHLGESTVR	91	1	1	N-Term(iTRAQ8plex)	0.788	0.568	0.618	5.27	55	0	1955.12436	3	652.3792114	0.153303568	44.74372333
														SLQDIIAILGMDELSEEDKLTVSR	18	1	1	N-Term(iTRAQ8plex); K19(iTRAQ8plex)	0.933	0.244	0.303	5.21	40	1	3283.78188	4	821.7008057	-3.123997161	66.63633167
														VLDSGAPIKIPVGPETLGR	6	1	1	N-Term(iTRAQ8plex); K9(iTRAQ8plex)	0.773	0.392	0.468	5.15	48	1	2527.50005	2	1264.253662	-2.699782837	38.94286667
														AHGGYSVFAGVGER	16	1	1	N-Term(iTRAQ8plex)	0.752	1.037	0.874	5.07	57	0	1710.88181	2	855.949646	3.187852967	23.07602667
														IGLFGGAGVGK	14	1	1	N-Term(iTRAQ8plex); K11(iTRAQ8plex)	0.666	0.406	0.395	4.92	52	0	1583.97361	3	528.6627197	0.394343495	48.69158167
														VALTGLTVAEYFR	13	1	1	N-Term(iTRAQ8plex)	0.555	0.31	0.423	4.83	71	0	1744.00115	2	872.5042114	3.689751911	49.86216667
														IMDPNIVGSEHYDVAR	10	1	1	N-Term(iTRAQ8plex); M2(Oxidation)	0.898	0.483	0.683	4.77	54	0	2136.0615	3	707.3608398	-3.247546239	29.20971
														GSITSVQAIYVPADDLTDPAPATTFAHLDATTVLSR	2	1	1	N-Term(iTRAQ8plex)	-	-	0.576	4.75	35	0	4019.10276	3	1340.372437	2.7680358	48.62506
														IMNVIGEPIDER	14	1	1	N-Term(iTRAQ8plex)	0.707	0.454	0.428	4.6	64	0	1689.91001	3	563.9748535	-2.819086877	42.40327
														QFAPIHAEAPEFMEMSVEQEILVTGIK	3	1	1	N-Term(iTRAQ8plex); M15(Oxidation); K27(iTRAQ8plex)	-	-	-	4.57	25	0	3668.9208	3	1218.317261	4.267162446	50.42985833
														IPSAVGYQPTLATDMGTMQER	2	1	1	N-Term(iTRAQ8plex)	0.983	-	0.791	4.5	60	0	2570.28879	3	857.4344482	-0.353977125	38.13951
														QFAPIHAEAPEFMEMSVEQEILVTGIK	2	1	1	N-Term(iTRAQ8plex); M13(Oxidation); M15(Oxidation); K27(iTRAQ8plex)	-	-	-	4.49	27	0	3684.91128	3	1218.317261	4.267162446	50.42985833
														VVDLLAPYAK	33	1	1	N-Term(iTRAQ8plex); K10(iTRAQ8plex)	0.648	0.552	0.584	4.38	43	0	1697.04595	2	849.0266113	0.091179765	44.06041
														TIAMDGTEGLVR	7	1	1	N-Term(iTRAQ8plex)	0.698	0.53	0.451	4.34	41	0	1566.84307	2	783.9251709	-2.097289255	28.66498833
														AIAELGIYPAVDPLDSTSR	8	1	1	N-Term(iTRAQ8plex)	0.658	0.371	0.413	4.32	82	0	2292.23784	2	1146.622559	-0.484562501	49.75221667
														TVLIMELINNVAK	23	1	1	N-Term(iTRAQ8plex); M5(Oxidation); K13(iTRAQ8plex)	1.047	0.612	0.543	4.25	49	0	2082.24515	2	1033.626465	-2.302839691	85.35435
														SLQDIIAILGMDELSEEDKLTVSR	4	1	1	N-Term(iTRAQ8plex); M11(Oxidation); K19(iTRAQ8plex)	-	-	-	4.09	34	1	3299.79848	4	821.7008057	-3.123997161	66.63633167
														IMNVIGEPIDER	3	1	1	N-Term(iTRAQ8plex); M2(Oxidation)	0.6	0.382	0.442	3.66	45	0	1705.92229	3	563.9748535	-2.819086877	42.40327
														IPSAVGYQPTLATDMGTMQER	2	1	1	N-Term(iTRAQ8plex); M15(Oxidation); M18(Oxidation)	-	-	-	3.23	31	0	2602.28281	3	857.4344482	-0.353977125	38.13951
														TIAMDGTEGLVR	5	1	1	N-Term(iTRAQ8plex); M4(Oxidation)	0.741	0.422	0.441	3.03	34	0	1582.8383	2	783.9251709	-2.097289255	28.66498833
														LVPLKETIK	1	1	1	N-Term(iTRAQ8plex); K5(iTRAQ8plex); K9(iTRAQ8plex)	0.823	0.489	0.492	2.77	-	1	1953.28899	2	977.1481323	0.740662773	24.74576333
														VALVYGQMNEPPGAR	1	1	1	N-Term(iTRAQ8plex); M8(Oxidation)	0.66	0.504	0.5	-	44	0	1922.00481	2	953.5093384	-2.351067883	34.48544167
														IPSAVGYQPTLATDMGTMQER	1	1	1	N-Term(iTRAQ8plex); Y7(iTRAQ8plex); M15(Oxidation); M18(Oxidation)	0.383	-	0.386	-	35	0	2906.49257	3	857.4344482	-0.353977125	38.13951
														SLQDIIAILGMDELSEEDK	1	1	1	N-Term(iTRAQ8plex); K19(iTRAQ8plex)	0.556	0.583	0.559	-	34	0	2727.46831	2	1364.237793	3.658344064	64.36860167
														IPSAVGYQPTLATDMGTMQER	1	1	1	N-Term(iTRAQ8plex); Y7(iTRAQ8plex); M15(Oxidation)	-	0.433	0.548	-	32	0	2890.4872	3	857.4344482	-0.353977125	38.13951
														EGNDLYHEMIESGVINLK	1	1	1	N-Term(iTRAQ8plex); Y6(iTRAQ8plex); K18(iTRAQ8plex)	0.629	0.459	0.47	-	27	0	2973.59708	2	1335.203613	-2.46922815	43.92673667
														GFQQILAGEYDHLPEQAFYMVGPIEEAVAK	1	1	1	N-Term(iTRAQ8plex); Y19(iTRAQ8plex); K30(iTRAQ8plex)	-	-	-	-	22	0	4263.26381	3	1320.348145	-5.350036976	83.57064
32307144	PLOD1	procollagen-lysine,2-oxoglutarate 5-dioxygenase 1 isoform 2 precursor	NP_000293.2	8.80	4	4	12	727	83.5	6.95	0.729	0.319	0.475																
														IFQNLDGALDEVVLK	5	2	1	N-Term(iTRAQ8plex); K15(iTRAQ8plex)	0.676	0.162	0.417	5.67	58	0	2282.31534	3	761.4432983	-2.773293573	51.09208833
														VGVWNVPYISNIYLIK	4	2	1	N-Term(iTRAQ8plex); K16(iTRAQ8plex)	0.584	0.362	0.469	5.55	49	0	2486.473	3	829.4958496	3.955111575	55.62001833
														IQGGYENVPTIDIHMNQIGFER	1	2	1	N-Term(iTRAQ8plex)	0.883	2.569	1.883	4.39	-	0	2835.44675	3	945.8204346	2.309745699	42.15885667
														LFIHNHEQHHK	2	2	1	N-Term(iTRAQ8plex); K11(iTRAQ8plex)	0.858	0.277	0.505	3.16	-	0	2048.13059	3	683.3817139	-4.51775316	12.39248167
32307161	CUL1	cullin-1	NP_003583.2	3.48	2	2	3	776	89.6	8.00	0.903	1.330	1.510																
														QIGLDQIWDDLR	2	1	1	N-Term(iTRAQ8plex)	0.904	2.464	1.922	4.6	54	0	1775.96843	2	888.487854	5.084599283	49.76853
														HQQLLGEVLTQLSSR	1	2	1	N-Term(iTRAQ8plex)	0.903	0.717	1.186	3.19	-	0	2013.13657	3	671.717041	-1.446252166	45.78983167
32454741	SERPINH1	serpin H1 precursor	NP_001226.2	48.09	14	14	135	418	46.4	8.69	1.075	0.800	0.614																
														SALQSINEWAAQTTDGKLPEVTK	2	1	1	N-Term(iTRAQ8plex); K17(iTRAQ8plex); K23(iTRAQ8plex)	-	-	-	6.71	40	1	3399.87745	5	680.781311	-3.324424009	45.81333
														HLAGLGLTEAIDK	12	1	1	N-Term(iTRAQ8plex); K13(iTRAQ8plex)	1.015	1.128	0.613	5.88	72	0	1946.15019	2	973.5787354	-1.509244705	30.35977833
														LSSLIILMPHHVEPLER	4	1	1	N-Term(iTRAQ8plex); M8(Oxidation)	0.897	0.942	0.932	5.83	43	0	2304.29935	3	763.4417725	0.210999668	47.59809333
														LSSLIILMPHHVEPLER	16	1	1	N-Term(iTRAQ8plex)	0.832	0.676	0.669	5.72	54	0	2288.31076	3	763.4417725	0.210999668	47.59809333
														SAGLAFSLYQAMAK	12	1	1	N-Term(iTRAQ8plex); K14(iTRAQ8plex)	1.019	0.543	0.636	5.25	50	0	2066.15055	3	689.387207	-4.565723514	50.43524667
														GVVEVTHDLQK	8	1	1	N-Term(iTRAQ8plex); K11(iTRAQ8plex)	1.03	0.73	0.465	5.11	62	0	1833.06072	2	917.0339966	-4.546906637	21.39730667
														TDGALLVNAMFFKPHWDEK	1	1	1	N-Term(iTRAQ8plex); K13(iTRAQ8plex); K19(iTRAQ8plex)	0.882	1.136	0.584	5.03	-	0	3131.70131	4	783.6807861	-3.278297551	47.29558
														LQIVEMPLAHK	2	1	1	N-Term(iTRAQ8plex); K11(iTRAQ8plex)	0.86	0.566	0.451	4.84	54	0	1887.13016	3	629.7149048	-2.374078176	33.06136167
														DQAVENILVSPVVVASSLGLVSLGGK	2	1	1	N-Term(iTRAQ8plex); K26(iTRAQ8plex)	0.497	0.598	-	4.79	59	0	3159.84958	3	1053.954712	1.449813375	69.264035
														LFYADHPFIFLVR	38	1	1	N-Term(iTRAQ8plex)	1.045	0.759	0.588	4.72	53	0	1942.09037	3	648.0349731	0.558616433	56.18167333
														SYTVGVMMMHR	6	1	1	N-Term(iTRAQ8plex)	2.341	1.544	0.972	4.58	54	0	1615.79807	3	539.270874	-4.95264838	25.63364333
														LYGPSSVSFADDFVR	19	1	1	N-Term(iTRAQ8plex)	1.2	0.874	0.614	4.49	74	0	1964.00127	2	982.5056152	-1.419269673	44.463095
														DTQSGSLLFIGR	5	1	1	N-Term(iTRAQ8plex)	1.003	0.784	0.63	3.81	62	0	1597.88005	2	799.4436646	-3.197247772	25.139685
														KAVAISLPK	2	1	1	N-Term(iTRAQ8plex); K1(iTRAQ8plex); K9(iTRAQ8plex)	1.123	0.849	0.75	3.77	27	1	1839.22869	2	920.117981	5.015142563	20.412385
														LQIVEMPLAHK	2	1	1	N-Term(iTRAQ8plex); M6(Oxidation); K11(iTRAQ8plex)	1.05	0.615	0.39	3.64	26	0	1903.12571	3	629.7149048	-2.374078176	33.06136167
														SAGLAFSLYQAMAK	2	1	1	N-Term(iTRAQ8plex); M12(Oxidation); K14(iTRAQ8plex)	0.842	0.653	0.727	3.5	24	0	2082.14499	3	689.387207	-4.565723514	50.43524667
														SYTVGVMMMHR	1	1	1	N-Term(iTRAQ8plex); M7(Oxidation); M8(Oxidation); M9(Oxidation)	1.135	0.791	0.989	3.26	-	0	1663.78983	3	539.270874	-4.95264838	25.63364333
														AATLAER	1	1	1	N-Term(iTRAQ8plex)	1.168	0.826	0.664	-	28	0	1035.60503	2	518.3061523	-4.831216887	13.76022167
32483374	NOP56	nucleolar protein 56	NP_006383.2	23.91	10	10	43	594	66.0	9.19	1.450	0.646	0.662																
														LVAFCPFASSQVALENANAVSEGVVHEDLR	11	1	1	N-Term(iTRAQ8plex); C5(Methylthio)	2.42	1.252	0.529	10.5	107	0	3522.77121	3	1174.928589	1.362862154	65.389265
														EAMVQAEEAAAEITR	4	1	1	N-Term(iTRAQ8plex)	1.243	0.606	0.698	5.3	60	0	1922.9832	2	961.9952393	1.888001737	39.36637667
														LSFYETGEIPR	6	1	1	N-Term(iTRAQ8plex)	1.252	0.595	0.662	4.56	50	0	1615.8637	2	808.4354858	0.212740523	36.16000667
														EETVNDPEEAGHR	10	1	1	N-Term(iTRAQ8plex)	1.486	0.525	0.549	4.37	58	0	1786.84989	2	893.9282227	-1.008819061	10.99460667
														LLLETHLPSK	1	1	1	N-Term(iTRAQ8plex); K10(iTRAQ8plex)	1.477	0.717	0.775	4.15	-	0	1759.09641	3	587.0369873	1.482217831	31.53641833
														YGLIFHSTFIGR	4	1	1	N-Term(iTRAQ8plex)	1.37	0.677	0.632	4.1	42	0	1714.95078	3	572.3217773	-4.357278846	43.23372667
														IDCFSEVPTSVFGEK	4	1	1	N-Term(iTRAQ8plex); C3(Methylthio); K15(iTRAQ8plex)	1.893	1.243	1.1	3.93	28	0	2312.1716	3	771.3953857	-2.040645871	44.63798167
														VDNMIIQSISLLDQLDKDINTFSMR	1	1	1	N-Term(iTRAQ8plex); M4(Oxidation); K17(iTRAQ8plex)	-	-	-	2.94	-	1	3533.89077	4	884.2281494	2.90939277	69.76164833
														LLLETHLPSKK	1	1	1	N-Term(iTRAQ8plex); K10(iTRAQ8plex); K11(iTRAQ8plex)	3.041	0.665	1.694	2.8	-	1	2191.39687	4	548.6046753	1.250340325	26.12989333
														IINDNATYCR	1	1	1	N-Term(iTRAQ8plex); C9(Methylthio)	1.27	0.951	1.328	-	29	0	1532.74541	2	766.8763428	-3.192554417	22.18260667
32528286	ACOT7	cytosolic acyl coenzyme A thioester hydrolase isoform hBACHd	NP_863656.1	38.30	8	9	16	329	36.5	7.78	0.366	0.621	0.788																
														TNIVTASVDAINFHDK	4	4	1	N-Term(iTRAQ8plex); K16(iTRAQ8plex)	0.19	0.292	0.707	7.64	71	0	2353.28922	3	785.1012573	-3.404841734	36.63858833
														HSVEVQVNVMSENILTGAK	2	4	1	N-Term(iTRAQ8plex); K19(iTRAQ8plex)	0.562	0.727	1.485	5.37	38	0	2663.46402	3	888.4928589	-0.265777888	38.543255
														AASAFFTYVSLSQEGR	2	4	1	N-Term(iTRAQ8plex)	0.57	0.629	0.925	4.3	48	0	2038.04258	3	680.019043	-5.974185179	52.20728333
														IMRPDDANVAGNVHGGTILK	1	3	1	N-Term(iTRAQ8plex); K20(iTRAQ8plex)	0.325	1.245	1.177	3.42	-	0	2686.48423	3	896.1662598	-2.883044333	25.67981333
														MIEEAGAIISTR	1	4	1	N-Term(iTRAQ8plex)	-	-	-	3.4	-	0	1594.8748	2	797.94104	-1.785620958	46.793835
														LMDEVAGIVAAR	2	4	1	N-Term(iTRAQ8plex)	0.307	0.44	0.807	3.32	38	0	1548.86931	2	774.9382935	-1.853614064	53.03487167
														VLEVPPVVYSR	1	4	1	N-Term(iTRAQ8plex)	0.366	0.651	0.851	3.04	-	0	1561.92388	2	781.4655762	-1.07916671	37.796095
														GCVITISGR	1	4	1	N-Term(iTRAQ8plex); C2(Methylthio)	0.182	0.383	0.69	3.03	-	0	1255.68376	2	628.34552	2.644869002	29.65203667
														ATLWYVPLSLK	2	4	1	N-Term(iTRAQ8plex); K11(iTRAQ8plex)	0.443	0.674	0.521	2.94	-	0	1899.15433	3	633.7229614	-1.09010238	44.69489667
32996709	TMED7	transmembrane emp24 domain-containing protein 7 precursor	NP_861974.1	35.71	6	6	32	224	25.2	6.89	0.839	0.848	0.623																
														VSALTQMESACVSIHEALK	10	3	1	N-Term(iTRAQ8plex); C11(Methylthio); K19(iTRAQ8plex)	0.781	1.222	0.707	6.61	42	0	2671.41543	4	668.609314	2.850054432	61.81817833
														FCFSNEFSTFTHK	10	3	1	N-Term(iTRAQ8plex); C2(Methylthio); K13(iTRAQ8plex)	0.712	0.707	0.552	5.36	45	0	2249.10708	4	563.0299683	0.027062793	45.81153
														QCFYEDIAQGTK	6	3	1	N-Term(iTRAQ8plex); C2(Methylthio); K12(iTRAQ8plex)	0.919	0.94	0.623	4.37	45	0	2057.02329	3	686.3459473	-2.889492383	50.042495
														SVIDYQTHFR	3	3	1	N-Term(iTRAQ8plex)	0.848	0.771	0.883	3.65	38	0	1569.82871	3	523.9477539	-2.543482119	27.65225
														TVYFDFQVGEDPPLFPSENR	2	3	1	N-Term(iTRAQ8plex)	1.411	1.362	0.983	3.11	36	0	2661.31125	3	887.7752686	-0.999088676	52.733305
														SFFSDK	1	1	1	N-Term(iTRAQ8plex); K6(iTRAQ8plex)	0.673	0.659	0.429	-	23	0	1338.75272	3	446.9224243	0.999735948	22.52302333
33188463	MRPS9	28S ribosomal protein S9, mitochondrial	NP_872578.1	6.31	2	2	11	396	45.8	9.51	1.486	0.566	0.584																
														AIAYLFPSGLFEK	8	1	1	N-Term(iTRAQ8plex); K13(iTRAQ8plex)	1.804	0.533	0.607	4.82	48	0	2064.19315	3	688.7359009	-2.839808514	50.30245167
														HDVTCTVSGGGR	3	2	1	N-Term(iTRAQ8plex); C5(Methylthio)	1.284	0.566	0.584	4.44	59	0	1538.73282	3	513.5824585	-1.891206632	14.77273
33239443	SYPL1	synaptophysin-like protein 1 isoform b	NP_874384.1	6.22	1	1	6	241	26.4	7.23	0.864	0.609	0.333																
														IATGHNIIDELPPCK	6	2	1	N-Term(iTRAQ8plex); C14(Methylthio); K15(iTRAQ8plex)	0.864	0.609	0.333	4.33	56	0	2275.23955	2	1138.123413	-0.163828079	37.484815
33239451	PCNA	proliferating cell nuclear antigen	NP_872590.1	44.06	7	7	44	261	28.8	4.69	1.292	4.254	3.989																
														AEDNADTLALVFEAPNQEK	9	1	1	N-Term(iTRAQ8plex); K19(iTRAQ8plex)	1.132	2.271	1.714	6.98	85	0	2683.40091	2	1342.206177	0.557349928	39.37490667
														LMDLDVEQLGIPEQEYSCVVK	5	1	1	N-Term(iTRAQ8plex); C18(Methylthio); K21(iTRAQ8plex)	1.462	6.017	4.798	6.08	72	0	3062.56656	3	1021.537415	8.737060838	55.10258833
														DLSHIGDAVVISCAK	10	1	1	N-Term(iTRAQ8plex); C13(Methylthio); K15(iTRAQ8plex)	2.83	8.511	9.673	5.14	71	0	2182.17241	2	1091.589844	-4.427432018	39.9069
														NLAMGVNLTSMSK	5	1	1	N-Term(iTRAQ8plex); K13(iTRAQ8plex)	0.606	1.541	1.646	4.53	53	0	1974.09244	2	987.5475464	-4.794693775	47.96383333
														LSQTSNVDKEEEAVTIEMNEPVQLTFALR	3	1	1	N-Term(iTRAQ8plex); K9(iTRAQ8plex)	-	-	-	4	29	1	3900.04538	4	975.7678833	-0.637934658	49.302545
														CAGNEDIITLR	7	1	1	N-Term(iTRAQ8plex); C1(Methylthio)	1.282	4.737	5.286	3.75	34	0	1554.78923	2	777.902832	3.984531081	32.24301667
														YLNFFTK	5	1	1	N-Term(iTRAQ8plex); K7(iTRAQ8plex)	1.594	4.254	3.989	3.15	29	0	1540.88907	3	514.3012085	-6.034867718	37.57737833
33286418	PKM	pyruvate kinase PKM isoform a	NP_002645.3	71.37	37	37	568	531	57.9	7.84	0.798	1.982	2.409																
														LAPITSDPTEATAVGAVEASFK	8	6	1	N-Term(iTRAQ8plex); K22(iTRAQ8plex)	1.021	5.032	6.056	7.5	82	0	2783.51836	2	1392.262817	-3.748189533	49.29973667
														AEGSDVANAVLDGADCIMLSGETAK	12	10	1	N-Term(iTRAQ8plex); C16(Methylthio); K25(iTRAQ8plex)	0.494	3.732	5.451	6.58	79	0	3091.50469	2	1546.255981	-5.187127629	61.627725
														GIFPVLCKDPVQEAWAEDVDLR	48	10	1	N-Term(iTRAQ8plex); C7(Methylthio); K8(iTRAQ8plex)	0.97	7.225	8.125	6.32	65	1	3154.66191	2	1577.834595	2.935507316	57.990585
														TATESFASDPILYRPVAVALDTK	32	9	1	N-Term(iTRAQ8plex); K23(iTRAQ8plex)	0.874	6.796	7.799	6.15	46	0	3073.69097	2	1537.349121	-3.935246788	45.380475
														IYVDDGLISLQVK	29	10	1	N-Term(iTRAQ8plex); K13(iTRAQ8plex)	0.96	3.218	4.176	5.9	69	0	2071.21123	2	1036.109131	-7.225821685	41.470775
														AGKPVICATQMLESMIK	5	10	1	N-Term(iTRAQ8plex); K3(iTRAQ8plex); C7(Methylthio); M11(Oxidation); K17(iTRAQ8plex)	-	2.05	2.899	5.83	41	0	2794.53383	2	1389.773682	-4.205675781	61.26629
														FGVEQDVDMVFASFIR	28	10	1	N-Term(iTRAQ8plex)	0.985	3.482	4.351	5.8	88	0	2164.08647	3	722.0410767	1.647528383	68.79961333

														RFDEILEASDGIMVAR	101	10	1	N-Term(iTRAQ8plex)	0.826	1.511	1.543	5.76	82	1	2126.11612	3	709.3776245	-1.651373214	44.99362833
														RFDEILEASDGIMVAR	30	10	1	N-Term(iTRAQ8plex); M13(Oxidation)	1.002	1.055	1.259	5.44	57	1	2142.11386	3	709.3776245	-1.651373214	44.99362833
														FDEILEASDGIMVAR	9	10	1	N-Term(iTRAQ8plex)	0.694	1.742	1.795	5.34	93	0	1970.02402	3	657.3461914	1.680014413	57.10334167
														AGKPVICATQMLESMIK	17	10	1	N-Term(iTRAQ8plex); K3(iTRAQ8plex); C7(Methylthio); K17(iTRAQ8plex)	0.405	2.968	3.859	5.34	48	0	2778.54009	2	1389.773682	-4.205675781	61.26629
														GADFLVTEVENGGSLGSK	6	10	1	N-Term(iTRAQ8plex); K18(iTRAQ8plex)	0.837	2.391	3.141	5.21	77	0	2388.27373	2	1194.640503	-5.452499211	34.55253833
														EAEAAIYHLQLFEELR	40	6	1	N-Term(iTRAQ8plex)	0.925	1.646	2.27	5.2	95	0	2236.18943	2	1118.598633	-0.71652901	52.59452
														EAEAAIYHLQLFEELRR	31	6	1	N-Term(iTRAQ8plex)	0.502	1.151	1.534	4.93	42	1	2392.28652	4	598.8272095	-2.384032248	46.816815
														AEGSDVANAVLDGADCIMLSGETAK	4	10	1	N-Term(iTRAQ8plex); C16(Methylthio); M18(Oxidation); K25(iTRAQ8plex)	-	1.137	1.435	4.9	27	0	3107.50625	2	1546.255981	-5.187127629	61.627725
														DPVQEAWAEDVDLR	4	10	1	N-Term(iTRAQ8plex)	0.572	1.755	2.494	4.86	56	0	1946.97014	2	973.9887085	-3.10786883	39.87586667
														GSGTAEVELKK	3	9	1	N-Term(iTRAQ8plex); K10(iTRAQ8plex); K11(iTRAQ8plex)	0.775	2.011	2.109	4.61	25	1	2031.22719	4	508.5622559	2.890989998	19.32376333
														VNFAMNVGK	10	10	1	N-Term(iTRAQ8plex); K9(iTRAQ8plex)	1.037	2.513	2.931	4.6	45	0	1587.89957	2	794.4527588	-9.761546096	28.56903
														AGKPVICATQMLESMIKKPRPTR	1	10	1	N-Term(iTRAQ8plex); K3(iTRAQ8plex); C7(Methylthio); K17(iTRAQ8plex); [illegible]	-	-	-	4.59	-	1	3818.17614	6	637.2020874	-8.229328552	52.21972833
														ITLDNAYMEK	7	10	1	N-Term(iTRAQ8plex); K10(iTRAQ8plex)	0.739	2.262	2.392	4.55	54	0	1805.97708	2	903.4920044	-8.881139304	38.92985167
														NTGIICTIGPASR	12	9	1	N-Term(iTRAQ8plex); C6(Methylthio)	0.679	2.421	2.839	4.54	53	0	1652.88443	3	551.6329956	4.743989505	32.24648
														CDENILWLDYK	23	10	1	N-Term(iTRAQ8plex); C1(Methylthio); K11(iTRAQ8plex)	0.445	2.183	3.194	4.12	56	0	2066.03794	2	1033.521729	-8.974619576	70.06611667
														AGKPVICATQMLESMIKKPRPTR	2	10	1	N-Term(iTRAQ8plex); K3(iTRAQ8plex); C7(Methylthio); M11(Oxidation); [illegible]	-	1.572	0.836	4.01	-	1	3834.17297	6	637.2020874	-8.229328552	52.21972833
														GIFPVLCK	3	10	1	N-Term(iTRAQ8plex); C7(Methylthio); K8(iTRAQ8plex)	0.872	1.786	2.206	3.85	27	0	1530.88657	3	510.967041	-8.552235929	66.59473
														FGVEQDVDMVFASFIR	4	10	1	N-Term(iTRAQ8plex); M9(Oxidation)	1.236	2.57	4.204	3.85	73	0	2180.10259	3	722.0410767	1.647528383	68.79961333
														ITLDNAYMEK	2	10	1	N-Term(iTRAQ8plex); M8(Oxidation); K10(iTRAQ8plex)	0.756	2.189	2.548	3.64	47	0	1821.97368	2	903.4920044	-8.881139304	38.92985167
														GDLGIEIPAEK	11	14	1	N-Term(iTRAQ8plex); K11(iTRAQ8plex)	0.741	2.73	3.419	3.59	57	0	1750.00505	2	875.5061646	-8.953584685	23.91388
														VNFAMNVGK	4	10	1	N-Term(iTRAQ8plex); M5(Oxidation); K9(iTRAQ8plex)	0.826	2.471	3.132	3.48	34	0	1603.89475	2	794.4527588	-9.761546096	28.56903
														AEGSDVANAVLDGADCIMLSGETAKGDYPLEAVR	2	10	1	N-Term(iTRAQ8plex); C16(Methylthio); K25(iTRAQ8plex)	-	-	-	3.45	30	1	4092.02236	4	1023.761047	0.927969789	56.58903833
														GSGTAEVELK	9	9	1	N-Term(iTRAQ8plex); K10(iTRAQ8plex)	0.684	2.332	3.184	3.45	50	0	1598.90593	2	799.9563599	-9.724273255	23.85138833
														LDIDSPPITAR	6	9	1	N-Term(iTRAQ8plex)	0.454	1.945	2.62	3.38	40	0	1501.84819	2	751.4277344	-3.068998601	28.94258167
														MQHLIAR	7	10	1	N-Term(iTRAQ8plex)	0.743	1.966	2.669	3.37	42	0	1172.67954	3	391.5646973	-6.849913264	14.77884333
														GDVVIVLTGWRPGSGFTNTMR	3	10	1	N-Term(iTRAQ8plex)	0.895	2.136	2.633	3.36	29	0	2567.3749	2	1284.196533	5.884774431	49.82443
														CCSGAIIVLTK	14	6	1	N-Term(iTRAQ8plex); C1(Methylthio); C2(Methylthio); K11(iTRAQ8plex)	0.788	2.764	2.815	3.25	36	0	1807.95829	3	603.3257446	-7.523752247	45.68555667
														IENHEGVR	8	10	1	N-Term(iTRAQ8plex)	0.693	1.518	1.493	3.2	33	0	1257.67975	3	419.8987732	-2.826605281	11.23417833
														LNFSHGTHEYHAETIK	1	8	1	N-Term(iTRAQ8plex); K16(iTRAQ8plex)	-	2.05	2.896	2.99	-	0	2492.30459	4	623.831604	-3.878002885	22.05380333
														GDYPLEAVR	11	10	1	N-Term(iTRAQ8plex)	0.732	1.741	2.301	2.97	35	0	1323.71354	3	441.9093628	-5.670540411	23.40469833
														KGDVVIVLTGWRPGSGFTNTMR	1	10	1	N-Term(iTRAQ8plex); K1(iTRAQ8plex)	-	-	-	2.96	-	1	2999.65742	5	600.7373047	-4.531975026	44.07563167
														APIIAVTR	4	10	1	N-Term(iTRAQ8plex)	0.611	2.205	3.434	2.85	62	0	1144.73041	3	382.2483215	-4.507568192	22.63764333
														DIQDLK	1	10	1	N-Term(iTRAQ8plex); K6(iTRAQ8plex)	1.072	1.14	1.366	2.79	-	0	1339.79468	3	447.2697449	-7.068195591	21.14767833
														QAHLYR	2	10	1	N-Term(iTRAQ8plex)	0.703	2.35	2.893	2.72	31	0	1091.62168	3	364.5454102	-4.254295556	10.841625
														ASDVHEVR	3	10	1	N-Term(iTRAQ8plex)	0.916	1.698	1.36	2.71	35	0	1216.65104	3	406.2218628	-6.358578483	10.883335
														MQHLIAR	7	10	1	N-Term(iTRAQ8plex); M1(Oxidation)	0.771	1.228	1.486	-	36	0	1188.67618	3	391.5646973	-6.849913264	14.77884333
														KASDVHEVR	1	10	1	N-Term(iTRAQ8plex); K1(iTRAQ8plex)	0.668	1.662	2.473	-	29	1	1648.94607	4	412.9919739	-7.905456982	12.36522833
														CDENILWLDYK	1	10	1	N-Term(iTRAQ8plex); C1(Methylthio); Y10(iTRAQ8plex); K11(iTRAQ8plex)	0.318	2.648	3.562	-	25	0	2370.24039	2	1033.521729	-8.974619576	70.06611667
														VFLAQK	1	14	1	N-Term(iTRAQ8plex); K6(iTRAQ8plex)	0.904	2.284	2.988	-	25	0	1313.82756	2	657.4174194	-9.574356007	19.49923833
33286446	OGFR	opioid growth factor receptor	NP_031372.2	2.22	1	1	2	677	73.3	4.84	-	-	-																
														DADAGDEDEESEEPR	2	1	1	N-Term(iTRAQ8plex)	-	-	-	3.52	77	0	1967.8198	3	656.6114502	-2.964684099	13.23766667
33300635	ZFPL1	zinc finger protein-like 1	NP_006773.2	12.90	4	4	6	310	34.1	8.07	1.511	0.470	1.058																
														VTNLFCFEHR	2	1	1	N-Term(iTRAQ8plex); C6(Methylthio)	1.511	0.969	1.058	3.82	41	0	1615.79898	3	539.2711792	-2.28862153	37.33656333
														CIVQSYLQWLQDSDYNPNCR	2	1	1	N-Term(iTRAQ8plex); C1(Methylthio); C19(Methylthio)	-	-	-	3.77	39	0	2841.29825	3	947.7709351	7.457374729	63.69119833
														KVTNLFCFEHR	1	1	1	N-Term(iTRAQ8plex); K1(iTRAQ8plex); C7(Methylthio)	1.463	0.456	1.126	3.16	-	1	2048.09023	4	512.7780151	-6.240756844	33.20619167
														RPALGWLAR	1	1	1	N-Term(iTRAQ8plex)	1.724	0.47	0.362	-	32	0	1343.81934	3	448.6112976	-1.51934689	26.16592333
33350932	DYNC1H1	cytoplasmic dynein 1 heavy chain 1	NP_001367.2	12.51	41	41	80	4646	532.1	6.40	0.840	1.346	2.046																
														FYFVGDEDLLEIIGNSK	4	1	1	N-Term(iTRAQ8plex); K17(iTRAQ8plex)	1.488	2.07	2.805	5.99	61	0	2567.38803	3	856.4675293	1.029583604	64.35115
														LDRDPASGTALQEISFWLNLER	2	1	1	N-Term(iTRAQ8plex)	-	-	-	5.62	59	1	2835.49399	3	945.8361816	-0.130357576	63.94020333
														HVPVVYVDYPGPASLTQIYGTFNR	6	1	1	N-Term(iTRAQ8plex)	0.391	0.569	1.309	5.6	55	0	2997.58316	3	999.8659058	1.839059954	50.63164333
														AKEALELTDTGLLSGSEER	2	1	1	N-Term(iTRAQ8plex); K2(iTRAQ8plex)	0.419	0.945	2.122	5.37	62	1	2627.41935	3	876.4779663	-5.912883394	38.787185
														WQASSLPADDLCTENAIMLK	2	1	1	N-Term(iTRAQ8plex); C12(Methylthio); K20(iTRAQ8plex)	1.128	3.394	4.387	5.18	53	0	2860.44907	3	954.154541	-0.472828948	48.25230333
														GTVGEPTYDAEFQHFLR	2	1	1	N-Term(iTRAQ8plex)	0.744	1.196	1.701	5.12	52	0	2271.12961	3	757.7147217	-2.284991095	36.03530333
														NVAQYNANHPDFPMQIEQLER	1	1	1	N-Term(iTRAQ8plex)	1.037	1.674	2.034	4.97	-	0	2818.38077	3	940.1317749	-2.739105747	37.05444167
														QWIVFDGDVDPEWVENLNSVLDDNK	1	1	1	N-Term(iTRAQ8plex); K25(iTRAQ8plex)	-	-	-	4.89	-	0	3554.79946	4	889.4553223	2.640903547	62.13210833
														INEWLTLVEK	2	1	1	N-Term(iTRAQ8plex); K10(iTRAQ8plex)	1.389	1.932	2.611	4.65	36	0	1853.08318	2	927.0452271	-8.695680061	76.34393333
														LLAESVTEVEIFGK	2	1	1	N-Term(iTRAQ8plex); K14(iTRAQ8plex)	0.682	1.031	1.565	4.61	26	0	2143.23081	3	715.0817871	-7.602177896	46.15676333
														VTFVNFTVTR	2	1	1	N-Term(iTRAQ8plex)	0.825	1.283	1.808	4.37	28	0	1487.8543	2	744.4307861	1.279927497	35.332905
														IQGLTVEQAEAVVR	2	1	1	N-Term(iTRAQ8plex)	0.713	1.624	2.018	4.35	39	0	1817.0363	2	909.0217896	-3.945335835	39.06598
														KLEHLITELVHQR	4	1	1	N-Term(iTRAQ8plex); K1(iTRAQ8plex)	-	1.514	3.079	4.32	32	1	2224.32143	4	556.8358154	-7.732453145	48.799425
														VPQIEVETHK	1	1	1	N-Term(iTRAQ8plex); K10(iTRAQ8plex)	0.556	1.123	1.763	4.22	-	0	1788.02964	3	596.6813965	-10.0362413	22.29350333
														NLHVVFTMNPSSEGLK	1	1	1	N-Term(iTRAQ8plex); K16(iTRAQ8plex)	1.199	1.574	1.659	4.2	-	0	2381.30783	3	794.4407959	-1.237494223	34.66476
														TTLSRFVAWMNGLSVYQIK	2	1	1	N-Term(iTRAQ8plex); K19(iTRAQ8plex)	-	-	-	4.17	29	1	2822.57534	4	706.399292	-3.345601133	64.21760333
														ILDDDTIITTLENLK	1	1	1	N-Term(iTRAQ8plex); K15(iTRAQ8plex)	1.54	1.288	1.38	4.09	-	0	2325.33982	3	775.78479	1.05170314	60.91758833
														LNTQEIFDDWAR	2	1	1	N-Term(iTRAQ8plex)	0.972	1.638	2.363	4.09	33	0	1811.91374	2	906.4605103	-5.114709361	37.44518333
														ESPEVLLTLDILK	2	1	1	N-Term(iTRAQ8plex); K13(iTRAQ8plex)	0.48	3.282	4.398	3.98	27	0	2078.25217	3	693.4222412	-2.290827483	51.12455167
														IMFEVQDLK	2	1	1	N-Term(iTRAQ8plex); K9(iTRAQ8plex)	1.028	1.242	1.588	3.93	37	0	1730.9876	2	865.9974365	-5.514119884	65.9207
														VNFLPEIITLSK	4	1	1	N-Term(iTRAQ8plex); K12(iTRAQ8plex)	0.549	1.555	2.807	3.93	36	0	1982.20476	2	991.6060181	-4.995340234	46.79298667
														ILDDDTIITTLENLKR	1	1	1	N-Term(iTRAQ8plex); K15(iTRAQ8plex)	-	-	-	3.92	-	1	2481.43252	4	621.1135864	-2.408075512	59.8253
														ALEHAFQLEHIMDLTR	3	1	1	N-Term(iTRAQ8plex)	-	1.619	3.073	3.9	-	0	2228.17544	4	557.7993164	-2.044522584	46.34117167
														VTDFGDKVEDPTFLNQLQSGVNR	1	1	1	N-Term(iTRAQ8plex); K7(iTRAQ8plex)	-	-	-	3.77	-	1	3187.67031	4	797.6730347	-4.439765331	43.07316833
														AHQANQLYPFAISLIESVR	2	1	1	N-Term(iTRAQ8plex)	-	-	-	3.74	42	0	2461.34836	3	821.1209717	-0.889557517	60.42596167
														IFVFEPPPGVK	1	1	1	N-Term(iTRAQ8plex); K11(iTRAQ8plex)	1.029	1.184	2.058	3.68	-	0	1838.08591	3	613.3668213	-9.657156235	35.96142
														LKVNFLPEIITLSK	1	1	1	N-Term(iTRAQ8plex); K2(iTRAQ8plex); K14(iTRAQ8plex)	-	2.382	3.214	3.64	-	1	2527.58901	4	632.65271	-3.976649768	52.21286667
														HLLPVETQR	2	1	1	N-Term(iTRAQ8plex)	0.898	0.833	1.131	3.62	26	0	1396.81253	3	466.2756958	-6.362448388	16.61368333
														TLINELVKGILPR	2	1	1	N-Term(iTRAQ8plex); K8(iTRAQ8plex)	-	-	-	3.32	17	1	2074.31338	4	519.3338013	-3.613775028	55.83639667
														FNALFVRPHIR	4	1	1	N-Term(iTRAQ8plex)	0.791	1.426	2.378	3.22	32	0	1673.99965	4	419.2553711	5.426043533	34.126965
														FGNPLLVQDVESYDPVLNPVLNR	2	1	1	N-Term(iTRAQ8plex)	-	-	-	3.19	25	0	2902.57871	3	968.1977539	5.859330774	62.11648667
														GIFEALRPLETLPVEGLIR	1	1	1	N-Term(iTRAQ8plex)	0.641	0.782	2.906	3.16	-	0	2427.4299	3	809.8148193	0.874776161	63.50881833
														ASVVTLPVYLNFTR	2	1	1	N-Term(iTRAQ8plex)	1.088	1.116	1.739	3.15	35	0	1884.08147	2	942.5443726	-4.35374104	51.23605667
														IWAHEALR	2	1	1	N-Term(iTRAQ8plex)	0.513	0.686	0.664	3	28	0	1299.73908	3	433.9178772	-6.503580868	21.12704833
														LLNTFLER	1	1	1	N-Term(iTRAQ8plex)	1.034	1.786	1.745	2.93	-	0	1309.76995	2	655.3886108	-6.273040807	36.17631
														EYQTQLIQR	2	1	1	N-Term(iTRAQ8plex)	0.818	1.313	1.817	2.91	29	0	1482.81194	2	741.9096069	-6.652261534	15.94736667
														LQGEFQLR	1	1	1	N-Term(iTRAQ8plex)	0.61	1.346	1.697	2.83	-	0	1294.74443	2	647.8758545	1.792681488	30.296245
														TVENIKDPLFR	1	1	1	N-Term(iTRAQ8plex); K6(iTRAQ8plex)	0.931	4.009	3.356	2.7	-	1	1940.13149	4	485.7883301	-5.706245971	29.30022333
														ENFIPTIVNFSAEEISDAIR	1	1	1	N-Term(iTRAQ8plex)	-	-	-	-	31	0	2569.35807	3	857.1242065	4.999333909	69.000855
														SIPLDEGEDEAQRR	2	1	1	N-Term(iTRAQ8plex)	0.895	1.224	1.968	-	29	1	1918.97367	3	640.3294067	-1.867839678	19.032715
														KYEFGESDLR	1	1	1	N-Term(iTRAQ8plex); K1(iTRAQ8plex)	0.856	1.182	1.183	-	27	1	1851.98929	3	618.0012817	-9.081766957	18.67063833
33356174	PNN	pinin	NP_002678.2	8.23	6	6	19	717	81.6	7.14	1.497	1.045	0.684																
														LTEVPVEPVLTVHPESK	4	1	1	N-Term(iTRAQ8plex); K17(iTRAQ8plex)	1.522	1.468	0.623	5.97	45	0	2482.42582	3	828.1467896	-4.812187169	35.48578667
														HVIADQEVMETNR	4	1	1	N-Term(iTRAQ8plex)	0.958	0.692	0.511	5.21	59	0	1845.94004	3	615.9848633	-1.660241497	16.85319333
														IFGLLMGTLQK	4	1	1	N-Term(iTRAQ8plex); K11(iTRAQ8plex)	1.808	1.238	0.798	3.99	40	0	1829.11118	3	610.3752441	-3.691480432	58.10092167
														RIFGLLMGTLQK	2	1	1	N-Term(iTRAQ8plex); K12(iTRAQ8plex)	1.224	1.247	0.992	3.61	25	1	1985.22434	3	662.4129639	2.66320106	50.93971833
														LEVQAEEER	3	1	1	N-Term(iTRAQ8plex)	1.681	0.98	0.721	3.43	44	0	1406.75298	2	703.8728638	-3.170725116	15.24668667
														DLEGAVSR	2	1	1	N-Term(iTRAQ8plex)	1.698	1.046	1.002	2.99	23	0	1150.63091	2	575.8190918	-5.279126619	16.90059667
33356547	MCM2	DNA replication licensing factor MCM2	NP_004517.2	8.85	7	7	16	904	101.8	5.52	1.191	2.522	1.609																
														GVCLIDEFDKMNDQDR	4	1	1	N-Term(iTRAQ8plex); C3(Methylthio); K10(iTRAQ8plex)	1.183	3.8	2.687	4.37	22	1	2552.22866	4	638.8161011	0.843225835	44.858605
														QLVAEQVTYQR	2	1	1	N-Term(iTRAQ8plex)	1.296	3.807	3.475	4.09	41	0	1638.91814	2	819.9627075	3.933765154	26.49069333
														ESLVVNYEDLAAR	2	1	1	N-Term(iTRAQ8plex)	1.191	1.56	1.449	3.72	27	0	1782.96221	2	891.9847412	4.618438825	36.23327167
														GLALALFGGEPK	2	1	1	N-Term(iTRAQ8plex); K12(iTRAQ8plex)	1.659	1.495	1.574	3.71	30	0	1781.06467	3	594.3597412	-7.585865585	67.20239333
														VMLESFIDTQK	2	1	1	N-Term(iTRAQ8plex); K11(iTRAQ8plex)	1.045	1.157	0.992	3.59	36	0	1919.06778	3	640.3607788	-4.725275625	33.75472
														QLHLNQLIR	3	1	1	N-Term(iTRAQ8plex)	0.693	1.919	2.126	2.76	34	0	1438.87168	3	480.2954102	-5.507149369	23.44866
														FDILCVVR	1	1	1	N-Term(iTRAQ8plex); C5(Methylthio)	1.825	5.354	4.28	-	22	0	1314.71465	2	657.8609619	-5.274978787	44.57132833
33413400	ESD	S-formylglutathione hydrolase	NP_001975.1	18.09	3	3	6	282	31.4	7.02	0.662	1.658	1.843																
														SVSAFAPICNPVLCPWGK	3	2	1	N-Term(iTRAQ8plex); C9(Methylthio); C14(Methylthio); K18(iTRAQ8plex)	0.726	1.592	2.202	4.84	43	0	2589.34641	3	863.7869873	5.918622557	60.5834
														VFEHDSVELNCK	2	1	1	N-Term(iTRAQ8plex); C11(Methylthio); K12(iTRAQ8plex)	0.604	1.728	1.543	3.84	38	0	2074.0402	3	692.0182495	-7.520112136	32.921885
														LQEGYDHSYYFIATFITDHIR	1	2	1	N-Term(iTRAQ8plex); Y10(iTRAQ8plex)	-	-	-	-	26	0	3197.66103	4	800.1707153	2.936567708	61.88882833
33457348	MYDGF	myeloid-derived growth factor precursor	NP_061980.1	20.81	3	3	12	173	18.8	6.68	0.773	0.585	0.849																
														ESDVPLKTEEFEVTK	4	1	1	N-Term(iTRAQ8plex); K7(iTRAQ8plex); K15(iTRAQ8plex)	0.507	0.366	0.806	5.4	26	1	2663.46474	4	666.6216431	-9.746295478	28.836975
														GAEIEYAMAYSK	4	1	1	N-Term(iTRAQ8plex); K12(iTRAQ8plex)	0.976	0.812	0.986	4.15	38	0	1941.01773	3	647.6774292	-3.640848931	48.05646333
														SYLYFTQFK	4	1	1	N-Term(iTRAQ8plex); K9(iTRAQ8plex)	0.898	0.54	0.621	4.01	25	0	1805.00388	3	602.3378906	-5.680155128	39.60292333
33469917	MCM4	DNA replication licensing factor MCM4	NP_877423.1	10.20	5	5	12	863	96.5	6.74	1.072	3.462	3.766																
														FIDPLAKEEENVGIDITEPLYMQR	6	1	1	N-Term(iTRAQ8plex); K7(iTRAQ8plex)	-	-	-	5.29	35	1	3428.82241	4	857.9610596	-0.254576833	55.34266667
														LGEINVIGEPFLNVNCEHIK	2	1	1	N-Term(iTRAQ8plex); C16(Methylthio); K20(iTRAQ8plex)	-	-	-	4.52	24	0	2892.55605	4	723.8944702	-0.418771704	55.04812667
														NLNPEDIDQLITISGMVIR	2	1	1	N-Term(iTRAQ8plex)	1.05	4.944	4.219	3.71	38	0	2445.33721	2	1223.172241	1.903419236	64.14632

														KTTIENIQLPHTLLSR	1	1	1	N-Term(iTRAQ8plex); K1(iTRAQ8plex)	1.093	2.424	3.361	2.75	-	1	2472.4601	4	618.8704834	-6.373241868	34.67689
														THIDVIHYR	1	1	1	N-Term(iTRAQ8plex)	-	-	-	-	47	0	1457.80947	4	365.2078247	-4.926811101	20.20061
33469968	MCM7	DNA replication licensing factor MCM7 isoform 1	NP_005907.3	13.21	7	7	19	719	81.3	6.46	0.933	2.205	2.308																
														QVVQGLLSETYLEAHR	2	3	1	N-Term(iTRAQ8plex)	0.846	2.143	1.581	5.65	72	0	2147.17765	3	716.3973999	0.645105929	42.633765
														LFADAVQELLPQYK	6	1	1	N-Term(iTRAQ8plex); K14(iTRAQ8plex)	1.496	4.3	4.606	4.08	37	0	2243.28879	3	748.4313354	-4.532739927	51.67666833
														MQEHSDQVPVGNIPR	2	3	1	N-Term(iTRAQ8plex)	0.942	1.496	2.195	4.03	41	0	2011.02719	3	671.013916	-3.046454638	20.61064
														ALLLLLVGGVDQSPR	2	3	1	N-Term(iTRAQ8plex)	-	-	-	4.02	68	0	1855.12809	3	619.0475464	-2.056671921	63.87577333
														MVDVVEKEDVNEAIR	2	3	1	N-Term(iTRAQ8plex); K7(iTRAQ8plex)	1.87	4.485	5.8	3.02	-	1	2354.27671	3	785.4304199	-3.374617925	32.36282
														TQRPADVIFATVR	1	3	1	N-Term(iTRAQ8plex)	0.587	0.749	0.933	2.75	-	0	1778.01616	2	889.5117188	-3.661620261	28.76819833
														QVVQGLLSETYLEAHR	1	3	1	N-Term(iTRAQ8plex); Y11(iTRAQ8plex)	0.908	0.85	0.926	-	29	0	2451.3666	3	716.3973999	0.645105929	42.633765
														MQEHSDQVPVGNIPR	1	3	1	N-Term(iTRAQ8plex); M1(Oxidation)	0.763	0.909	0.712	-	23	0	2027.02274	3	671.013916	-3.046454638	20.61064
														TLLAILR	2	3	1	N-Term(iTRAQ8plex)	1.546	4.929	4.781	-	22	0	1103.73723	2	552.3722534	-7.403106318	43.64309667
33519426	TXNRD1	thioredoxin reductase 1, cytoplasmic isoform 2	NP_877393.1	41.68	12	12	49	499	54.7	6.47	0.948	2.536	3.410																
														VVGFHVLGPNAGEVTQGFAAALK	12	5	1	N-Term(iTRAQ8plex); K23(iTRAQ8plex)	0.875	1.652	3.725	6.82	62	0	2890.64573	3	964.2200928	1.973182069	50.116755
														KLMHQAALLGQALQDSR	9	5	1	N-Term(iTRAQ8plex); K1(iTRAQ8plex)	1.632	3.026	2.776	5.59	51	1	2488.41153	3	830.1420288	-6.573721428	39.20866333
														FGEENIEVYHSYFWPLEWTIPSR	2	5	1	N-Term(iTRAQ8plex)	-	-	-	5.58	39	0	3203.60117	3	1068.538574	7.213165756	63.47218833
														VVYENAYGQFIGPHR	4	5	1	N-Term(iTRAQ8plex)	0.919	0.875	0.935	5.2	70	0	2054.06797	3	685.3608398	-3.979916382	32.63580667
														CDYENVPTTVFTPLEYGACGLSEEK	2	5	1	N-Term(iTRAQ8plex); C1(Methylthio); C19(Methylthio); K25(iTRAQ8plex)	-	-	-	4.97	42	0	3465.61155	3	1155.875366	-1.961534667	60.19918
														YLGIPGDKEYCISSDDLFSLPYCPGK	5	5	1	N-Term(iTRAQ8plex); K8(iTRAQ8plex); C11(Methylthio); C23(Methylthio); K26(iTRAQ8plex)	-	-	-	4.67	26	1	3884.9435	3	1295.645996	-3.922411601	57.153345
														VMVLDFVTPTPLGTR	2	4	1	N-Term(iTRAQ8plex)	0.96	5.694	6.32	4.27	47	0	1950.10295	2	975.5551147	-0.353021266	54.17548333
														WGLGGTCVNVGCIPK	2	7	1	N-Term(iTRAQ8plex); C7(Methylthio); C12(Methylthio); K15(iTRAQ8plex)	0.632	2.8	3.41	4.25	84	0	2204.11724	2	1102.562256	-6.205750326	40.96588833
														QFVPIKVEQIEAGTPGR	2	5	1	N-Term(iTRAQ8plex); K6(iTRAQ8plex)	0.663	5.155	6.594	3.31	-	1	2477.42246	3	826.4790039	-4.53407985	31.626875
														FLIATGERPR	3	5	1	N-Term(iTRAQ8plex)	1.63	4.933	5.044	3.28	-	0	1463.8544	3	488.6229858	-6.310074582	21.62213
														IGEHMEEHGIK	4	5	1	N-Term(iTRAQ8plex); K11(iTRAQ8plex)	0.844	2.506	2.602	3.27	32	0	1888.02665	3	630.0070801	-7.43870015	14.49516
														VEETVKHDWDR	1	5	1	N-Term(iTRAQ8plex); K6(iTRAQ8plex)	0.596	1.152	1.158	3.17	-	1	2022.09182	4	506.2784119	2.629099674	24.44004667
														VMVLDFVTPTPLGTR	1	4	1	N-Term(iTRAQ8plex); M2(Oxidation)	0.948	0.795	1.425	2.95	-	0	1966.08542	2	975.5551147	-0.353021266	54.17548333
33519455	MAT2B	methionine adenosyltransferase 2 subunit beta isoform 2	NP_877725.1	4.02	1	1	2	323	36.4	6.65	0.918	2.687	1.881																
														AVLENNLGAAVLR	2	2	1	N-Term(iTRAQ8plex)	0.918	2.687	1.881	3.8	56	0	1643.96782	2	822.4875488	-4.160825816	37.74962833
33519458	MCL1	induced myeloid leukemia cell differentiation protein Mcl-1 isoform 2	NP_877495.1	7.38	1	1	2	271	28.6	6.58	1.115	1.590	1.218																
														VARPPPIGAEVPDVTATPAR	2	2	1	N-Term(iTRAQ8plex)	1.115	1.59	1.218	3.57	28	0	2318.30692	3	773.4404907	-2.830678707	27.521215
33636719	TIMM44	mitochondrial import inner membrane translocase subunit TIM44	NP_006342.2	8.63	3	3	9	452	51.3	8.32	1.858	1.003	0.814																
														VFEPNEEALGVVLHK	2	1	1	N-Term(iTRAQ8plex); K15(iTRAQ8plex)	1.858	1.003	0.719	5.51	58	0	2289.30155	3	763.7720337	-2.098227639	39.08942167
														RLEESDVLQEAR	5	1	1	N-Term(iTRAQ8plex)	1.628	1.032	0.867	4.45	50	1	1748.9417	3	583.6508179	-3.762818976	16.98881167
														TEMSEVLTEILR	2	1	1	N-Term(iTRAQ8plex)	1.99	0.71	0.559	4.03	45	0	1724.95146	2	862.9793701	6.273981966	54.626105
33946329	RALA	ras-related protein Ral-A precursor	NP_005393.2	24.27	4	4	17	206	23.6	7.11	1.403	0.539	0.567																
														SALTLQFMYDEFVEDYEPTK	6	5	1	N-Term(iTRAQ8plex); K20(iTRAQ8plex)	1.403	0.523	0.606	7.13	57	0	3034.52103	3	1012.186829	8.008212887	61.75263833
														VKEDENVPFLLVGNK	5	1	1	N-Term(iTRAQ8plex); K2(iTRAQ8plex); K15(iTRAQ8plex)	1.69	0.669	0.555	5.23	35	1	2613.54233	3	871.8522949	1.683621334	34.89860167
														QVSVEEAK	1	1	1	N-Term(iTRAQ8plex); K8(iTRAQ8plex)	1.078	0.569	0.533	3.04	-	0	1497.87238	3	499.9623108	-0.615657893	20.16912
														VFFDLMR	5	6	1	N-Term(iTRAQ8plex)	1.377	0.393	0.462	2.93	31	0	1231.67314	2	616.348877	7.612290931	44.189915
34098946	YBX1	nuclease-sensitive element-binding protein 1	NP_004550.2	16.98	2	3	10	324	35.9	9.88	1.229	1.255	0.936																
														RFPPYYMR	1	1	1	N-Term(iTRAQ8plex)	1.229	1.255	0.936	3.23	-	1	1433.75983	3	478.5914612	-4.680703794	20.70742167
														RPQYSNPPVQGEVMEGADNQGAGEQGRPVR	1	1	1	N-Term(iTRAQ8plex)	-	-	-	2.9	-	0	3527.71865	4	882.6851196	-4.712015336	19.25091333
														SVGDGETVEFDVVEGEK	8	8	2	N-Term(iTRAQ8plex); K17(iTRAQ8plex)	-	-	-	4.7	94	0	2404.23648	2	1202.610962	-8.070865011	26.04200833
34101286	ZFR	zinc finger RNA-binding protein	NP_057191.2	4.56	3	3	11	1074	116.9	9.04	1.012	0.956	1.044																
														GSPGLLDPCEKDPFDTLATMTDQQR	5	1	1	N-Term(iTRAQ8plex); C9(Methylthio); K11(iTRAQ8plex)	1.71	0.961	1.451	5.51	33	1	3389.67299	4	848.1728516	1.735883061	46.32267
														EDITSSAQFALR	4	1	1	N-Term(iTRAQ8plex)	1.012	0.831	0.822	3.98	28	0	1641.88163	3	547.9653931	4.04088158	30.43114167
														LCECSFNDPNAK	2	1	1	N-Term(iTRAQ8plex); C2(Methylthio); C4(Methylthio); K12(iTRAQ8plex)	0.639	1.015	1.179	3.25	27	0	2040.9318	3	680.9821167	-7.534035369	63.48648833
34147513	RAB7A	ras-related protein Rab-7a	NP_004628.4	64.73	11	11	64	207	23.5	6.70	0.949	0.885	0.652																
														EAINVEQAFQTIAR	8	1	1	N-Term(iTRAQ8plex)	0.923	0.861	0.663	5.38	83	0	1894.0217	3	632.012085	-6.298590548	47.67179333
														DPENFPFVVLGNK	11	1	1	N-Term(iTRAQ8plex); K13(iTRAQ8plex)	1.019	0.976	0.661	5.29	57	0	2084.16582	2	1042.586548	1.016129861	41.51653
														LVTMQIWDTAGQER	6	1	1	N-Term(iTRAQ8plex)	0.762	0.52	0.39	4.84	75	0	1952.01738	2	976.5123291	-2.038771228	45.73944333
														ATIGADFLTK	4	1	1	N-Term(iTRAQ8plex); K10(iTRAQ8plex)	1.225	0.993	0.653	4.6	57	0	1644.98015	2	822.9937134	1.239430039	44.142615
														NALKQETEVELYNEFPEPIK	3	1	1	N-Term(iTRAQ8plex); K4(iTRAQ8plex); K20(iTRAQ8plex)	0.759	0.629	0.289	4.35	30	1	3303.82285	3	1101.945801	-0.351776201	43.88746833
														VIILGDSGVGK	4	1	1	N-Term(iTRAQ8plex); K11(iTRAQ8plex)	0.94	0.956	0.795	4.31	49	0	1666.03489	3	556.0164795	-0.651891174	40.447505
														NNIPYFETSAK	4	1	1	N-Term(iTRAQ8plex); K11(iTRAQ8plex)	1.007	0.845	0.669	4.25	39	0	1892.0302	2	946.5187378	-3.806669717	24.63863833
														FQSLGVAFYR	12	1	1	N-Term(iTRAQ8plex)	0.851	0.759	0.5	4.13	42	0	1491.82395	3	497.946167	-1.490506598	33.76761667
														TLDSWRDEFLIQASPR	5	1	1	N-Term(iTRAQ8plex)	1.451	1.128	0.729	3.92	41	1	2238.18242	2	1119.591187	-3.129725255	40.14442167
														AQAWCYSK	4	1	1	N-Term(iTRAQ8plex); C5(Methylthio); K8(iTRAQ8plex)	0.998	0.947	0.937	3.53	23	0	1610.81357	3	537.6099854	-7.778355788	24.16410667
														EVMVDDR	1	1	1	N-Term(iTRAQ8plex)	0.593	1.013	0.857	-	30	0	1167.59209	2	584.2996826	-5.20103355	19.67685667
														EVMVDDR	2	1	1	N-Term(iTRAQ8plex); M3(Oxidation)	0.705	0.6	0.58	-	28	0	1183.58928	2	584.2996826	-5.20103355	19.67685667
34147637	COPS6	COP9 signalosome complex subunit 6	NP_006824.2	8.56	2	2	3	327	36.1	5.73	0.603	1.024	1.246																
														QVCEIIESPLFLK	2	1	1	N-Term(iTRAQ8plex); C3(Methylthio); K13(iTRAQ8plex)	0.603	1.859	1.634	4.42	39	0	2173.21543	3	725.0766602	-3.089928944	51.940285
														ASEAGEVPFNHEILR	1	1	1	N-Term(iTRAQ8plex)	-	0.564	0.949	3.27	-	0	1973.0394	3	658.3513184	-0.025073942	25.79877667
34222121	NUP37	nucleoporin Nup37	NP_076962.2	4.91	1	1	2	326	36.7	5.92	1.749	1.393	1.153																
														VGAVAGNDWLIWDITR	2	1	1	N-Term(iTRAQ8plex)	1.749	1.393	1.153	4.53	73	0	2090.13701	2	1045.572144	1.583240409	55.27695
34577083	RSU1	ras suppressor protein 1 isoform 2	NP_689937.2	8.04	1	1	4	224	25.5	8.72	1.026	0.975	2.096																
														ALYLSDNDFEILPPDIGK	4	3	1	N-Term(iTRAQ8plex); K18(iTRAQ8plex)	1.026	0.975	2.096	4.99	80	0	2628.44097	2	1314.724121	1.06689385	47.97129
36029914	VCPIP1	deubiquitinating protein VCIP135	NP_079330.2	0.57	1	1	1	1222	134.2	7.20	10.144	2.513	0.620																
														ITIEILK	1	1	1	N-Term(iTRAQ8plex); K7(iTRAQ8plex)	10.144	2.513	0.62	2.98	-	0	1437.95047	3	479.9883423	0.265868315	63.09014667
37595530	PHC2	polyhomeotic-like protein 2 isoform b	NP_004418.2	3.41	1	1	1	323	35.7	8.62	0.996	0.781	1.554																
														WNVEDVYEFIR	1	6	1	N-Term(iTRAQ8plex)	0.996	0.781	1.554	-	26	0	1773.90703	2	887.4571533	-2.44729814	45.06685167
38016911	STOM	erythrocyte band 7 integral membrane protein isoform a	NP_004090.4	22.22	5	5	17	288	31.7	7.88	0.756	0.768	0.286																
														VIAAEGEMNASR	6	2	1	N-Term(iTRAQ8plex)	0.727	0.886	0.484	4.24	40	0	1551.81108	2	776.4091797	0.503576117	27.32043667
														AMAAEAEASR	5	1	1	N-Term(iTRAQ8plex)	0.734	0.735	0.253	4.18	64	0	1310.66654	3	437.5603638	-0.849370434	20.61340833
														TISFDIPPQEILTK	2	3	1	N-Term(iTRAQ8plex); K14(iTRAQ8plex)	0.756	0.768	0.286	4.18	35	0	2210.29399	2	1105.650635	2.127142935	40.39623333
														GPGLFFILPCTDSFIK	2	3	1	N-Term(iTRAQ8plex); C10(Methylthio); K16(iTRAQ8plex)	1.541	1.823	0.375	3.39	31	0	2409.32169	3	803.7787476	1.895606469	60.75558
														YLQTLTTIAAEK	2	2	1	N-Term(iTRAQ8plex); K12(iTRAQ8plex)	0.994	1.384	0.436	3.28	31	0	1960.15335	3	654.0559692	-2.125769979	70.17618833
38016943	ORAI1	calcium release-activated calcium channel protein 1	NP_116179.2	4.32	1	1	2	301	32.6	8.32	1.186	0.980	1.097																
														QFQELNELAEFAR	2	1	1	N-Term(iTRAQ8plex)	1.186	0.98	1.097	4.53	46	0	1898.99834	2	950.0028076	3.637346192	44.819175
														FIPLK	1	2	2	N-Term(iTRAQ8plex); K5(iTRAQ8plex)	-	-	-	-	21	0	1225.80924	3	409.2745972	-2.955636147	30.547225
38026892	ALG6	dolichyl pyrophosphate Man9GlcNAc2 alpha-1,3-glucosyltransferase precursor	NP_037471.2	3.94	2	2	3	507	58.1	8.46	1.051	0.970	0.811																
														FINPDWIALHTSR	1	1	1	N-Term(iTRAQ8plex)	0.973	0.973	1.08	2.99	-	0	1874.01523	3	625.3432617	-3.964425488	41.56173833
														SFSISVR	2	1	1	N-Term(iTRAQ8plex)	1.136	0.966	0.608	2.73	20	0	1099.64165	2	550.3244629	0.298152812	27.047245
38027914	COPS8	COP9 signalosome complex subunit 8 isoform 2	NP_937832.1	9.38	1	1	1	160	17.9	7.25	1.051	0.554	0.512																
														SANSELGGIWSVGQR	1	2	1	N-Term(iTRAQ8plex)	1.051	0.554	0.512	3.17	-	0	1864.98821	2	932.9977417	3.364889073	32.82915333
38201690	RAP2B	ras-related protein Rap-2b	NP_002877.2	12.02	2	2	4	183	20.5	4.81	0.946	0.800	0.446																
														ASVDELFAEIVR	2	1	1	N-Term(iTRAQ8plex)	0.936	0.792	0.361	4.63	50	0	1652.92058	2	826.9639282	2.685262297	50.65788833
														YDPTIEDFYR	2	3	1	N-Term(iTRAQ8plex)	0.957	0.808	0.552	3.96	24	0	1622.80657	2	811.9069214	3.798883715	33.791075
38201710	DDX17	probable ATP-dependent RNA helicase DDX17 isoform 1	NP_006377.2	26.34	13	18	77	729	80.2	8.27	0.765	0.982	0.915																
														LIQLMEEIMAEKENK	2	2	1	N-Term(iTRAQ8plex); K12(iTRAQ8plex); K15(iTRAQ8plex)	0.89	2.587	1.683	5.5	35	1	2731.54506	4	683.6417236	-1.869509244	64.66522833
														TLAYLLPAIVHINHQPYLER	4	2	1	N-Term(iTRAQ8plex)	-	-	-	4.9	24	0	2665.53872	4	667.1309204	-4.254185518	53.74483333
														LIQLMEEIMAEK	5	2	1	N-Term(iTRAQ8plex); K12(iTRAQ8plex)	0.831	0.922	0.866	4.48	72	0	2056.16233	2	1028.591797	5.838910749	64.87105
														NFYVEHPEVAR	11	2	1	N-Term(iTRAQ8plex)	0.779	0.926	0.92	4.33	47	0	1664.86338	2	832.9381714	-0.459583626	17.320185
														GTAYTFFTPGNLK	4	2	1	N-Term(iTRAQ8plex); K13(iTRAQ8plex)	0.813	0.956	0.872	4.19	40	0	2025.11223	2	1013.059753	-7.076686343	58.207145
														CTYLVLDEADR	9	2	1	N-Term(iTRAQ8plex); C1(Methylthio)	0.847	1.151	0.967	3.99	62	0	1647.80962	2	824.4084473	4.372842088	39.61209667
														LIDFLESGK	2	2	1	N-Term(iTRAQ8plex); K9(iTRAQ8plex)	0.717	0.972	0.962	3.97	49	0	1629.95683	2	815.4820557	-6.366175873	72.75463333
														LMQLVDHR	8	2	1	N-Term(iTRAQ8plex)	0.707	1.081	0.92	3.58	59	0	1315.73572	3	439.2512512	-5.03206706	28.19248833
														DGWPAMCIHGDK	2	2	1	N-Term(iTRAQ8plex); C7(Methylthio); K12(iTRAQ8plex)	1.068	1.184	1.171	3.37	26	0	1983.95652	3	661.9903564	-6.782514613	28.51372167
														QLAEDFLR	4	2	1	N-Term(iTRAQ8plex)	0.777	0.847	0.853	3.09	41	0	1295.71575	2	648.3615112	-8.015229494	35.28903833
														DWVLNEFR	6	2	1	N-Term(iTRAQ8plex)	0.72	0.937	0.877	3.08	32	0	1382.72881	2	691.868042	-5.947612852	62.18179833
														WDLSELPK	2	2	1	N-Term(iTRAQ8plex); K8(iTRAQ8plex)	0.646	0.925	0.846	2.8	25	0	1595.9135	2	798.4603882	-7.419921452	48.82454
														LTPYEVDELRR	1	2	1	N-Term(iTRAQ8plex)	0.77	1.186	0.722	2.7	-	1	1694.93241	3	565.6489868	-3.253286537	24.229615
														LMQLVDHR	1	2	1	N-Term(iTRAQ8plex); M2(Oxidation)	0.5	1.205	0.845	-	39	0	1331.73612	3	439.2512512	-5.03206706	28.19248833
														GVEICIATPGR	4	4	2	N-Term(iTRAQ8plex); C5(Methylthio)	-	-	-	4.2	39	0	1465.77471	2	733.3909912	-4.227456034	33.01195167
														GDGPICLVLAPTR	2	4	2	N-Term(iTRAQ8plex); C6(Methylthio)	-	-	-	3.62	43	0	1661.89824	2	831.4527588	-2.317435436	47.73994667
														APILIATDVASR	4	4	2	N-Term(iTRAQ8plex)	-	-	-	3.51	49	0	1530.91167	2	765.9594727	-2.660249826	35.94274
														QTLMWSATWPK	1	4	2	N-Term(iTRAQ8plex); K11(iTRAQ8plex)	-	-	-	3.23	-	0	1957.0662	3	653.0269165	-8.382464687	62.212765
														MLDMGFEPQIR	4	13	3	N-Term(iTRAQ8plex)	-	-	-	4.35	52	0	1640.84397	3	547.6195068	-0.173107372	38.69848833
														MLDMGFEPQIR	1	13	3	N-Term(iTRAQ8plex); M4(Oxidation)	-	-	-	-	27	0	1656.83379	3	547.6195068	-0.173107372	38.69848833
38201714	ELAVL1	ELAV-like protein 1	NP_001410.2	16.26	5	5	15	326	36.1	9.17	1.050	0.765	0.765																
														NVALLSQLYHSPAR	6	1	1	N-Term(iTRAQ8plex)	0.984	0.937	0.788	5.65	73	0	1873.05722	3	625.0239258	-1.354580211	31.59685833
														SLFSSIGEVESAK	2	1	1	N-Term(iTRAQ8plex); K13(iTRAQ8plex)	1.277	0.844	0.626	5.19	56	0	1962.08457	3	654.699707	-8.079904664	62.53659667
														VLVDQTTGLSR	4	1	1	N-Term(iTRAQ8plex)	1.205	0.556	0.771	3.93	41	0	1492.86101	2	746.9341431	-1.796407817	25.64747833
														ILQVSFK	1	1	1	N-Term(iTRAQ8plex); K7(iTRAQ8plex)	0.963	0.664	0.765	2.76	-	0	1442.91545	2	721.9613647	-2.542371977	46.59455333
														NVALLSQLYHSPAR	1	1	1	N-Term(iTRAQ8plex); Y9(iTRAQ8plex)	0.33	0.832	0.592	-	32	0	2177.26528	3	625.0239258	-1.354580211	31.59685833

														DVEDMFSR	1	1	1	N-Term(iTRAQ8plex)	1.205	0.765	0.803	-	24	0	1302.62444	2	651.8158569	-4.417276882	28.97300333
38202255	TARS	threonine--tRNA ligase, cytoplasmic isoform 1	NP_689508.3	24.76	13	13	31	723	83.4	6.67	1.373	4.481	2.948																
														FMADIDLDPGCTLNK	2	2	1	N-Term(iTRAQ8plex); C11(Methylthio); K15(iTRAQ8plex)	1.354	3.307	2.655	4.8	56	0	2307.15635	3	769.7236328	-3.487892148	47.95111
														VNNVVWDLDRPLEEDCTLELLK	3	2	1	N-Term(iTRAQ8plex); C16(Methylthio); K22(iTRAQ8plex)	-	-	-	4.74	36	0	3267.71963	4	817.6853638	-0.559136671	62.87364
														FLGDIEVWDQAEK	4	2	1	N-Term(iTRAQ8plex); K13(iTRAQ8plex)	1.484	4.995	3.366	4.69	62	0	2158.14433	2	1079.575806	-9.159068537	42.15945167
														LADFGVLHR	2	2	1	N-Term(iTRAQ8plex)	0.815	4.582	3.769	4.6	58	0	1331.771	3	444.5951843	-2.066099566	28.66745833
														DQELYFFHELSPGSCFFLPK	2	2	1	N-Term(iTRAQ8plex); C15(Methylthio); K20(iTRAQ8plex)	-	3.267	2.16	4.41	46	0	3058.54319	3	1020.185913	4.192750284	62.47170833
														QLENSLNEFGEKWELNSGDGAFYGPK	1	2	1	N-Term(iTRAQ8plex); K12(iTRAQ8plex); K26(iTRAQ8plex)	-	-	-	3.83	-	1	3841.98159	4	961.2508545	0.386984871	51.06470667
														FNLTYVSHDGDDK	2	2	1	N-Term(iTRAQ8plex); K13(iTRAQ8plex)	2.28	3.179	2.617	3.7	31	0	2119.0759	3	707.0301514	-7.423258615	24.67413
														AELNPWPEYIYTR	3	2	1	N-Term(iTRAQ8plex)	1.396	3.026	2.627	3.65	49	0	1956.00994	2	978.508606	-3.556222191	41.515405
														GAYIYNALIEFIR	2	2	1	N-Term(iTRAQ8plex)	1.391	6.391	4.849	3.49	28	0	1847.03874	2	924.0230103	0.997590234	68.69464667
														CGPLIDLCR	5	2	1	N-Term(iTRAQ8plex); C1(Methylthio); C8(Methylthio)	4.576	7.141	4.189	3	32	0	1385.66521	2	693.3362427	-4.571499954	56.89577333
														AEHDSILAEK	2	2	1	N-Term(iTRAQ8plex); K10(iTRAQ8plex)	0.922	1.236	1.314	2.92	27	0	1720.97202	3	574.3288574	1.753494881	16.97470833
														TISETIER	2	2	1	N-Term(iTRAQ8plex)	0.863	5.673	4.277	2.84	23	0	1252.70464	2	626.855957	-0.330371173	22.81678333
														ISGTVNIR	1	2	1	N-Term(iTRAQ8plex)	2.177	8.326	5.729	-	26	0	1163.69927	2	582.3532715	-4.919614041	28.14369
38202257	GANAB	neutral alpha-glucosidase AB isoform 2 precursor	NP_938148.1	33.16	21	21	110	944	106.8	6.14	0.908	0.775	0.847																
														YSLLPFWYTLLYQAHR	7	6	1	N-Term(iTRAQ8plex)	0.44	0.803	0.976	6.41	70	0	2375.30619	3	792.4311523	-2.731266101	66.1092
														LYNLDVFQYELYNPMALYGSVPVLLAHNPHR	2	6	1	N-Term(iTRAQ8plex)	-	-	-	6.01	29	0	3951.06826	4	988.522522	2.851560613	66.84118667
														FSFSGNTLVSSSADPEGHFETPIWIER	11	6	1	N-Term(iTRAQ8plex)	0.602	0.404	0.579	5.76	53	0	3314.62754	3	1105.547363	0.092104994	50.14991667
														ALLDSLQLGPDSLTVHLIHEVTK	1	2	1	N-Term(iTRAQ8plex); K23(iTRAQ8plex)	0.544	-	1.309	5.38	-	0	3107.79213	4	777.7034912	-0.130747564	57.02624333
														DENSVELTMAEGPYK	2	5	1	N-Term(iTRAQ8plex); K15(iTRAQ8plex)	0.81	0.558	0.496	5.25	41	0	2291.16025	3	764.3916016	-3.640017103	23.98825667
														WYQMGAYQPFFR	12	6	1	N-Term(iTRAQ8plex)	0.961	0.718	0.821	4.76	41	0	1897.93412	3	633.3162231	-1.067010909	49.34281167
														DVHNIYGLYVHMATADGLR	4	6	1	N-Term(iTRAQ8plex)	0.32	0.957	0.602	4.61	28	0	2449.28042	4	613.0755615	8.326900267	44.43218333
														YRVPDVLVADPPIAR	8	3	1	N-Term(iTRAQ8plex)	0.972	1.089	1.031	4.58	50	1	1985.13883	3	662.387207	-0.777502219	31.63005833
														HHGPQTLYLPVTLSSIPVFQR	9	6	1	N-Term(iTRAQ8plex)	0.791	1.293	0.998	4.47	37	0	2694.50571	3	898.8400879	0.871819223	48.25661667
														FGAVWTGDNTAEWDHLK	4	6	1	N-Term(iTRAQ8plex); K17(iTRAQ8plex)	0.794	0.564	0.595	4.2	24	0	2555.31264	4	639.5836182	-0.515993123	42.13589833
														LSFQHDPETSVLVLR	6	6	1	N-Term(iTRAQ8plex)	0.902	0.805	0.949	4.05	45	0	2045.12546	3	682.3800049	-3.853016214	37.157555
														LVAIVDPHIK	6	6	1	N-Term(iTRAQ8plex); K10(iTRAQ8plex)	0.955	0.483	0.65	4.03	41	0	1713.09389	3	571.6967163	-7.434362063	32.28736
														GLLEFEHQR	10	5	1	N-Term(iTRAQ8plex)	0.973	1.074	1.009	3.95	51	0	1432.78122	3	478.2652588	-2.664874965	31.47108
														EPWLLPSQHNDIIR	4	6	1	N-Term(iTRAQ8plex)	1.028	0.965	0.901	3.79	46	0	2022.09867	3	674.7044067	-4.349112781	35.16293333
														WYQMGAYQPFFR	2	6	1	N-Term(iTRAQ8plex); M4(Oxidation)	1.106	0.768	0.843	3.39	23	0	1913.92691	3	633.3162231	-1.067010909	49.34281167
														VTEGGEPYR	1	6	1	N-Term(iTRAQ8plex)	0.778	0.508	0.641	3.29	-	0	1311.68547	2	656.3463745	0.625991104	15.522515
														AEKDEPGAWEETFK	1	5	1	N-Term(iTRAQ8plex); K3(iTRAQ8plex); K14(iTRAQ8plex)	0.725	0.613	0.639	3.24	-	1	2549.3721	3	850.4622192	2.743093027	33.505835
														FRIDELEPR	6	3	1	N-Term(iTRAQ8plex)	1.146	0.879	1.186	3.22	35	1	1478.82328	3	493.6126099	-2.464770615	26.28694333
														SLLLSVNAR	3	5	1	N-Term(iTRAQ8plex)	0.912	0.631	0.73	3.22	25	0	1276.78667	2	638.8969727	-1.873928949	29.4059
														SGGMERPFVLAR	1	6	1	N-Term(iTRAQ8plex)	1.081	0.882	0.963	2.85	-	0	1623.88921	2	812.4482422	-3.142791377	23.75131667
														LDLLEDR	6	5	1	N-Term(iTRAQ8plex)	0.899	0.717	0.674	2.73	34	0	1177.67034	2	589.3388062	-2.288938789	37.00476167
														YSLLPFWYTLLYQAHR	2	6	1	N-Term(iTRAQ8plex); Y12(iTRAQ8plex)	-	-	-	-	29	0	2679.48916	3	792.4311523	-2.731266101	66.1092
														DVHNIYGLYVHMATADGLR	1	6	1	N-Term(iTRAQ8plex); Y9(iTRAQ8plex)	0.648	0.588	0.638	-	27	0	2753.47647	4	613.0755615	8.326900267	44.43218333
														IILTARPFR	1	5	1	N-Term(iTRAQ8plex)	0.942	0.661	0.845	-	22	0	1390.87845	3	464.2976685	-3.730864403	27.26224167
38327039	HSPA4	heat shock 70 kDa protein 4	NP_002145.3	19.52	10	12	48	840	94.3	5.19	0.787	2.517	2.309																
														AGGIETIANEYSDR	4	1	1	N-Term(iTRAQ8plex)	0.947	2.483	2.193	6.07	70	0	1799.91336	3	600.6426392	3.118444799	31.79608333
														EFSITDVVPYPISLR	3	1	1	N-Term(iTRAQ8plex)	0.901	1.979	1.935	4.89	58	0	2040.12749	2	1020.567383	-2.182361163	53.90035
														KFDEVLVNHFCEEFGK	3	1	1	N-Term(iTRAQ8plex); K1(iTRAQ8plex); C11(Methylthio); K16(iTRAQ8plex)	0.455	2.81	2.309	4.88	26	1	2899.51389	3	967.1761475	-3.832769082	58.15488167
														EMLNLYIENEGK	4	1	1	N-Term(iTRAQ8plex); K12(iTRAQ8plex)	1.371	2.885	2.982	4.44	40	0	2061.09702	3	687.7038574	-8.574212767	54.92319833
														SNLAYDIVQLPTGLTGIK	2	1	1	N-Term(iTRAQ8plex); K18(iTRAQ8plex)	-	1.123	0.856	4.25	39	0	2511.45243	3	837.8223267	-4.728168212	50.14085
														VLATAFDTTLGGR	6	1	1	N-Term(iTRAQ8plex)	0.875	2.044	1.949	3.96	66	0	1625.92021	2	813.4637451	2.301659297	42.25507167
														SVMDATQIAGLNCLR	2	1	1	N-Term(iTRAQ8plex); C13(Methylthio)	0.768	2.185	1.878	3.93	62	0	1941.98332	2	971.4953003	-1.502759589	48.55955667
														GKFLEMCNDLLAR	3	1	1	N-Term(iTRAQ8plex); K2(iTRAQ8plex); C7(Methylthio)	0.598	3.852	4.12	2.9	-	1	2164.14273	3	722.0551758	-1.288360324	58.78667167
														AFSDPFVEAEK	2	1	1	N-Term(iTRAQ8plex); K11(iTRAQ8plex)	0.395	1.125	1.362	2.82	34	0	1847.98313	3	616.6658936	-9.120151417	45.18654167
														LSQECEK	1	1	1	N-Term(iTRAQ8plex); C5(Methylthio); K7(iTRAQ8plex)	0.726	2.093	2.055	-	28	0	1490.76677	2	745.8870239	-9.089998985	16.29958833
														GCALQCAILSPAFK	16	17	3	N-Term(iTRAQ8plex); C2(Methylthio); C6(Methylthio); K14(iTRAQ8plex)	-	-	-	4.74	60	0	2122.09348	2	1061.553711	-6.61987844	44.91212167
														LMNETTAVALAYGIYK	2	6	2	N-Term(iTRAQ8plex); K16(iTRAQ8plex)	-	-	-	4.58	36	0	2366.31314	3	789.4425659	-5.019114279	43.39990333
38327625	CS	citrate synthase, mitochondrial precursor	NP_004068.2	39.91	13	13	79	466	51.7	8.32	1.316	0.639	0.680																
														AALPSHVVTMLDNFPTNLHPMSQLSAAVTALNSESNFAR	8	1	1	N-Term(iTRAQ8plex)	-	-	-	6.65	39	0	4456.28335	4	1114.82019	-2.407728414	65.57123833
														YWELIYEDSMDLIAK	6	1	1	N-Term(iTRAQ8plex); K15(iTRAQ8plex)	1.083	0.431	0.502	5.07	55	0	2497.30259	3	833.1057129	-4.779508176	85.38454333
														GLVYETSVLDPDEGIR	7	1	1	N-Term(iTRAQ8plex)	1.351	0.639	0.801	4.68	63	0	2067.0894	2	1034.04834	-0.874772275	39.82063833
														IVPNVLLEQGK	11	1	1	N-Term(iTRAQ8plex); K11(iTRAQ8plex)	1.585	0.794	0.774	4.67	56	0	1818.12432	2	909.5657959	-3.638982808	28.314565
														VVPGYGHAVLR	14	1	1	N-Term(iTRAQ8plex)	1.244	0.705	0.652	4.61	70	0	1471.8649	3	491.2937317	-1.406025235	19.225595
														EGSIGIGAIDSNLDWSHNFTNMLGYTDHQFTELTR	3	1	1	N-Term(iTRAQ8plex)	2.739	1.417	1.265	4.53	35	0	4130.94944	3	1377.654663	0.904079769	61.65236333
														HLPNDPMFK	4	1	1	N-Term(iTRAQ8plex); K9(iTRAQ8plex)	1.542	0.921	0.916	3.7	29	0	1706.94719	2	853.9772339	-2.144264674	19.95139667
														DYIWNTLNSGR	7	1	1	N-Term(iTRAQ8plex)	0.973	0.512	0.664	3.7	42	0	1642.84172	2	821.9244995	-4.485457401	55.5637
														TKYWELIYEDSMDLIAK	2	1	1	N-Term(iTRAQ8plex); K2(iTRAQ8plex); K17(iTRAQ8plex)	-	-	-	3.63	25	1	3030.68276	4	758.4261475	6.674622635	63.00195833
														LVAQLYK	2	1	1	N-Term(iTRAQ8plex); K7(iTRAQ8plex)	1.386	0.699	0.731	3.33	30	0	1442.9135	2	721.9603882	-3.889043115	39.41662333
														ALGVLAQLIWSR	6	1	1	N-Term(iTRAQ8plex)	1.386	0.6	0.67	3.29	63	0	1631.00383	2	816.0055542	5.622597727	64.17891333
														DILADLIPK	4	1	1	N-Term(iTRAQ8plex); K9(iTRAQ8plex)	1.095	0.557	0.624	3.26	43	0	1606.00066	2	803.5039673	-1.832720101	69.39559333
														ALGFPLERPK	5	1	1	N-Term(iTRAQ8plex); K10(iTRAQ8plex)	1.447	0.554	0.655	2.84	32	0	1736.0591	2	868.5357056	-2.192768753	27.39702167
38327634	DDX18	ATP-dependent RNA helicase DDX18	NP_006764.3	20.45	11	11	34	670	75.4	9.50	1.578	0.927	1.345																
														GLDIPEVDWIVQYDPPDDPKEYIHR	4	1	1	N-Term(iTRAQ8plex); K20(iTRAQ8plex)	-	-	-	6.03	26	1	3617.86635	4	905.2220459	-2.036445112	49.99427167
														LLDHMQNTPGFMYK	4	1	1	N-Term(iTRAQ8plex); K14(iTRAQ8plex)	1.913	1.194	1.29	5.66	33	0	2303.1976	3	768.4040527	-6.98869556	35.59167833
														EMGFTNMTEIQHK	4	1	1	N-Term(iTRAQ8plex); K13(iTRAQ8plex)	2.156	1.038	1.476	5.45	42	0	2174.10459	3	725.3730469	-6.846002297	25.74657667
														GLDIPEVDWIVQYDPPDDPK	2	1	1	N-Term(iTRAQ8plex); K20(iTRAQ8plex)	1.205	0.325	1.107	4.78	45	0	2919.52951	3	973.8480225	1.991016972	51.02358333
														NLQCLVIDEADR	6	1	1	N-Term(iTRAQ8plex); C4(Methylthio)	1.954	0.92	1.492	4.51	79	0	1738.87883	2	869.9430542	1.057853143	37.53777167
														ISDIQSQLEK	2	1	1	N-Term(iTRAQ8plex); K10(iTRAQ8plex)	1.589	0.745	1.379	4.18	31	0	1769.0123	3	590.3422852	-8.032211665	42.32484333
														VPLSEFDFSWSK	1	1	1	N-Term(iTRAQ8plex); K12(iTRAQ8plex)	0.857	0.365	0.545	3.68	-	0	2050.1063	3	684.0402832	-2.094984359	39.06087833
														FLLLFTFLKK	2	1	1	N-Term(iTRAQ8plex); K9(iTRAQ8plex); K10(iTRAQ8plex)	-	-	-	3.19	26	1	2182.43032	4	546.3630371	7.898193749	67.97556833
														GHALLILRPEELGFLR	7	1	1	N-Term(iTRAQ8plex)	1.172	1.113	1.388	2.89	42	0	2138.27138	2	1069.642944	1.585739041	44.90466667
														TLAFLIPAVELIVK	1	1	1	N-Term(iTRAQ8plex); K14(iTRAQ8plex)	-	-	-	-	36	0	2135.35892	3	712.4578247	-3.517174014	66.15563167
														QTMLFSATQTR	1	1	1	N-Term(iTRAQ8plex)	1.566	0.921	1.164	-	26	0	1587.83476	2	794.4210205	-7.492533003	21.49438833
38327642	WDR55	WD repeat-containing protein 55	NP_060176.2	3.66	1	1	3	383	42.1	4.94	0.463	0.618	0.994																
														AIHVLDVEQGQLER	3	2	1	N-Term(iTRAQ8plex)	0.463	0.618	0.994	4.35	27	0	1911.04546	3	637.6912231	-0.557343516	28.18656
38372921	BSG	basigin isoform 3	NP_940992.1	46.02	1	7	44	176	19.4	6.68	0.668	0.685	0.593																
														GTANIQLHGPPR	1	1	1	N-Term(iTRAQ8plex)	0.668	0.685	0.593	-	31	0	1564.88431	3	522.2996216	-1.182070708	15.41545333
														KPEDVLDDDDAGSAPLK	2	4	2	N-Term(iTRAQ8plex); K1(iTRAQ8plex); K17(iTRAQ8plex)	-	-	-	5.5	58	0	2697.46982	3	899.828125	-0.455816035	24.52907333
														SELHIENLNMEADPGQYR	11	4	2	N-Term(iTRAQ8plex)	-	-	-	5.46	49	0	2420.18094	3	807.3971558	-2.035911017	30.52970833
														SESVPPVTDWAWYK	17	4	2	N-Term(iTRAQ8plex); K14(iTRAQ8plex)	-	-	-	4.51	46	0	2273.19779	3	758.4041138	-3.736252715	33.719245
														SELHIENLNMEADPGQYR	2	4	2	N-Term(iTRAQ8plex); M10(Oxidation)	-	-	-	4.5	23	0	2436.17136	3	807.3971558	-2.035911017	30.52970833
														GSDQAIITLR	9	4	2	N-Term(iTRAQ8plex)	-	-	-	4.09	46	0	1377.79778	3	459.9390259	1.569954098	27.32681
														RKPEDVLDDDDAGSAPLK	1	4	2	N-Term(iTRAQ8plex); K2(iTRAQ8plex); K18(iTRAQ8plex)	-	-	-	3.97	-	1	2853.56655	4	714.1470947	-1.970262005	24.539505
														FFVSSSQGR	1	4	2	N-Term(iTRAQ8plex)	-	-	-	-	27	0	1318.70134	2	659.8543091	-3.321264489	21.84339667
38372933	CHMP2A	charged multivesicular body protein 2a	NP_940818.1	8.56	1	1	2	222	25.1	5.97	1.901	1.756	1.789																
														KAEAAASALADADLEER	2	2	1	N-Term(iTRAQ8plex); K1(iTRAQ8plex)	1.901	1.756	1.789	4.98	61	1	2525.32029	3	842.4449463	-4.017582589	35.85562333
38455427	CCT4	T-complex protein 1 subunit delta isoform a	NP_006421.2	29.50	14	16	79	539	57.9	7.83	1.140	2.842	2.017																
														ALIAGGGAPEIELALR	4	2	1	N-Term(iTRAQ8plex)	1.407	4.939	3.27	5.31	58	0	1855.0911	3	619.0352173	-2.389475393	46.98272667
														DALSDLALHFLNK	14	2	1	N-Term(iTRAQ8plex); K13(iTRAQ8plex)	1.105	2.186	1.75	4.86	62	0	2065.1832	2	1033.099609	0.823569938	51.87877333
														KLGGTIDDCELVEGLVLTQK	2	2	1	N-Term(iTRAQ8plex); K1(iTRAQ8plex); C9(Methylthio); K20(iTRAQ8plex)	-	-	-	4.61	25	1	3089.73891	4	773.1901855	1.132784225	57.50661167
														LGGTIDDCELVEGLVLTQK	4	2	1	N-Term(iTRAQ8plex); C8(Methylthio); K19(iTRAQ8plex)	1.256	2.429	2.577	4.55	82	0	2657.43828	2	1329.222778	1.203309461	61.39349667
														IGLIQFCLSAPK	17	2	1	N-Term(iTRAQ8plex); C7(Methylthio); K12(iTRAQ8plex)	1.448	4.044	2.895	4.39	54	0	1944.12724	2	972.5645142	-2.757337437	57.613795
														QMQVLHPAAR	2	1	1	N-Term(iTRAQ8plex)	0.872	0.954	0.945	3.93	30	0	1454.81559	3	490.9418335	-2.963713132	10.821505
														DIEREDIEFICK	4	2	1	N-Term(iTRAQ8plex); C11(Methylthio); K12(iTRAQ8plex)	1.096	4.375	2.553	3.91	34	1	2164.11051	2	1082.560303	-4.881191345	37.96622
														QMQVLHPAAR	5	1	1	N-Term(iTRAQ8plex); M2(Oxidation)	1.154	1.512	1.148	3.91	31	0	1470.80884	3	490.9418335	-2.963713132	10.821505
														TGCNVLLIQK	3	2	1	N-Term(iTRAQ8plex); C3(Methylthio); K10(iTRAQ8plex)	1.033	2.676	1.752	3.9	31	0	1743.00615	2	872.0067139	-3.202045857	51.88512833
														VIDPATATSVDLR	2	2	1	N-Term(iTRAQ8plex)	1.361	4.242	2.611	3.62	50	0	1661.93181	2	831.4695435	-3.484502735	31.008435
														AKIGLIQFCLSAPK	3	2	1	N-Term(iTRAQ8plex); K2(iTRAQ8plex); C9(Methylthio); K14(iTRAQ8plex)	0.481	2.08	1.685	3.61	40	1	2447.4448	3	816.4864502	-8.075754797	53.22899333
														TVTIVVR	5	2	1	N-Term(iTRAQ8plex)	1.136	4.596	2.58	3.49	40	0	1091.70929	3	364.5746155	0.258205686	24.62125
														TLSGMESYCVR	2	2	1	N-Term(iTRAQ8plex); M5(Oxidation); C9(Methylthio)	1.097	2.936	1.744	2.93	25	0	1611.74626	2	806.37677	-1.24329802	27.33272
														LVIEEAER	2	2	1	N-Term(iTRAQ8plex)	1.372	2.673	1.876	2.83	-	0	1262.7199	2	631.8635864	-4.676467406	25.18503333
														MLVELSK	1	2	1	N-Term(iTRAQ8plex); K7(iTRAQ8plex)	-	-	-	-	28	0	1427.87894	3	476.6311646	2.604634216	39.75831167
														IDDVVNTR	1	2	1	N-Term(iTRAQ8plex)	1.124	1.805	0.963	-	26	0	1235.68157	2	618.3444214	-6.615900719	17.04731833
														SLHDALCVIR	18	7	2	N-Term(iTRAQ8plex); C7(Methylthio)	-	-	-	3.54	55	0	1476.78952	3	492.9402161	6.244992752	32.50943833
38490688	IGSF10	immunoglobulin superfamily member 10 isoform 1 precursor	NP_849144.2	0.38	1	1	1	2623	290.7	9.13	2.567	1.155	0.716																
														NSTVNIPLFR	1	2	1	N-Term(iTRAQ8plex)	2.567	1.155	0.716	3.19	-	0	1464.83585	3	488.9501343	-8.049856216	26.12627
38569421	ACLY	ATP-citrate synthase isoform 1	NP_001087.2	29.88	23	23	52	1101	120.8	7.33	0.825	1.676	2.872																
														TTDGVYEGVAIGGDRYPGSTFMDHVLR	2	4	1	N-Term(iTRAQ8plex)	1.18	-	-	5.34	26	1	3217.57583	4	805.1494141	-4.116431349	40.78533167
														QHFPATPLLDYALEVEK	3	4	1	N-Term(iTRAQ8plex); K17(iTRAQ8plex)	1.797	4.913	7.086	5.14	42	0	2579.42557	3	860.4800415	-2.880501133	48.25663833

														TTDGVYEGVAIGGDR	4	4	1	N-Term(iTRAQ8plex)	0.917	0.995	1.659	5.06	63	0	1813.91983	3	605.3114624	-1.972161559	26.71168167
														GVTIIGPATVGGIKPGCFK	2	4	1	N-Term(iTRAQ8plex); K14(iTRAQ8plex); C17(Methylthio); K19(iTRAQ8plex)	-	-	-	4.82	31	0	2773.60903	4	694.1577148	-5.25770029	38.54545667
														DEPSVAAMVYPFTGDHK	2	4	1	N-Term(iTRAQ8plex); K17(iTRAQ8plex)	0.936	1.246	2.161	4.7	27	0	2472.26255	3	824.7590332	-2.602854877	36.61118
														EAYPEEAYIADLDAK	2	4	1	N-Term(iTRAQ8plex); K15(iTRAQ8plex)	0.793	1.294	2.225	4.69	48	0	2306.21294	2	1153.610107	5.063246137	36.978065
														MIVVLGEIGGTEEYK	2	4	1	N-Term(iTRAQ8plex); K15(iTRAQ8plex)	1.019	2.093	3.316	4.46	43	0	2246.23606	3	749.4168701	-9.008046315	43.58221333
														KHPEVDVLINFASLR	3	4	1	N-Term(iTRAQ8plex); K1(iTRAQ8plex)	0.874	2.426	3.78	4.38	28	1	2346.36051	3	782.791687	-6.360282145	39.39274833
														VDATADYICK	2	4	1	N-Term(iTRAQ8plex); C9(Methylthio); K10(iTRAQ8plex)	0.744	2.331	3.835	4.28	36	0	1752.89763	2	876.9524536	-8.238754611	44.45622667
														LGLVGVNLTLDGVKSWLKPR	1	4	1	N-Term(iTRAQ8plex); K14(iTRAQ8plex); K18(iTRAQ8plex)	-	-	-	4.21	-	1	3077.88435	5	616.3826904	-4.00783791	59.24615667
														AFDSGIIPMEFVNK	5	4	1	N-Term(iTRAQ8plex); K14(iTRAQ8plex)	0.963	1.841	2.961	4.12	34	0	2176.17851	3	726.0631714	-8.427810343	37.93408
														IGNTGGMLDNILASK	2	4	1	N-Term(iTRAQ8plex); K15(iTRAQ8plex)	0.95	1.645	2.496	3.98	58	0	2112.20024	2	1056.60376	2.784589799	41.76484
														DGVYVLDLAAK	2	4	1	N-Term(iTRAQ8plex); K11(iTRAQ8plex)	0.789	1.931	3.075	3.96	40	0	1772.03336	3	591.3493042	-4.560835326	54.87791
														AGKDLVSSLTSGLLTIGDR	3	4	1	N-Term(iTRAQ8plex); K3(iTRAQ8plex)	2.05	4.361	5.588	3.88	24	1	2511.45407	3	837.822876	-2.47928894	54.79567167
														LLQDHPWLLSQNLVVKPDQLIK	1	4	1	N-Term(iTRAQ8plex); K16(iTRAQ8plex); K22(iTRAQ8plex)	-	2.886	5.652	3.8	-	0	3510.08713	3	1170.700562	-2.971430423	49.62069
														FGGALDAAAK	2	4	1	N-Term(iTRAQ8plex); K10(iTRAQ8plex)	0.701	1.539	2.972	3.63	43	0	1528.87993	2	764.9436035	-9.458485563	33.576905
														WGDIEFPPPFGR	1	4	1	N-Term(iTRAQ8plex)	0.568	0.75	0.993	3.62	-	0	1721.8947	2	861.4509888	-0.377712375	48.60489667
														AIVWGMQTR	2	4	1	N-Term(iTRAQ8plex)	0.812	1.49	2.552	3.41	-	0	1365.76335	2	683.3853149	1.370634823	30.66712667
														AKPAMPQDSVPSPR	5	2	1	N-Term(iTRAQ8plex); K2(iTRAQ8plex)	0.724	1.498	2.85	3.31	28	0	2089.15189	3	697.055481	-7.941825096	19.09486167
														EGDYVLFHHEGGVDVGDVDAK	1	4	1	N-Term(iTRAQ8plex); K21(iTRAQ8plex)	-	-	-	3.3	-	0	2866.42714	4	717.3622437	-6.87147087	29.48600833
														FICTTSAIQNR	2	4	1	N-Term(iTRAQ8plex); C3(Methylthio)	0.832	1.328	1.986	2.8	23	0	1603.81865	2	802.4129639	-3.218060609	34.92386333
														LIMGIGHR	2	4	1	N-Term(iTRAQ8plex)	0.581	1.7	2.861	-	39	0	1200.71225	3	400.9089355	-5.519981458	20.72155167
														TASFSESR	1	4	1	N-Term(iTRAQ8plex)	0.446	1.651	3.34	-	24	0	1188.6093	2	594.8082886	-5.830992202	17.003245
38679892	PIN4	peptidyl-prolyl cis-trans isomerase NIMA-interacting 4 isoform 1	NP_006214.2	7.05	1	1	2	156	16.6	9.96	0.878	0.693	1.520																
														FGYHIIMVEGR	2	1	1	N-Term(iTRAQ8plex)	0.878	0.693	1.52	2.87	23	0	1625.86704	3	542.6271973	-6.478237688	32.85086667
38679974	ACACA	acetyl-CoA carboxylase 1 isoform 4	NP_942135.1	0.44	1	1	2	2268	257.1	6.61	0.945	2.497	1.648																
														IYVSANSGAR	2	8	1	N-Term(iTRAQ8plex)	0.945	2.497	1.648	3.36	34	0	1341.74724	2	671.3772583	3.285776509	21.47871167
38683855	PTCD3	pentatricopeptide repeat domain-containing protein 3, mitochondrial precursor	NP_060422.4	3.19	2	2	3	689	78.5	6.42	1.195	0.846	0.571																
														VEGTDVTGIEEVVIPK	2	1	1	N-Term(iTRAQ8plex); K16(iTRAQ8plex)	1.254	0.652	0.543	4.49	100	0	2293.32842	2	1147.167847	7.519829802	37.83195
														FHVFAR	1	1	1	N-Term(iTRAQ8plex)	1.139	1.099	0.6	-	20	0	1080.62619	3	360.880249	0.539058109	19.91164667
39725636	TMED9	transmembrane emp24 domain-containing protein 9 precursor	NP_059980.2	16.17	3	3	30	235	27.3	8.02	0.733	0.509	0.494																
														QLVEQVEQIQK	4	1	1	N-Term(iTRAQ8plex); K11(iTRAQ8plex)	0.748	0.504	0.596	4.78	41	0	1950.14005	3	650.7182007	-4.093295363	45.572965
														CFIEEIPDETMVIGNYR	12	4	1	N-Term(iTRAQ8plex); C1(Methylthio)	0.814	0.673	0.504	4.61	72	0	2379.127	2	1190.067139	-2.816821569	57.197335
														FSLFAGGMLR	11	1	1	N-Term(iTRAQ8plex)	0.519	0.445	0.364	3.85	40	0	1402.78069	2	701.8939819	-0.858332898	51.40922167
														CFIEEIPDETMVIGNYR	2	4	1	N-Term(iTRAQ8plex); C1(Methylthio); M11(Oxidation)	0.959	0.954	0.801	2.98	21	0	2395.12699	2	1190.067139	-2.816821569	57.197335
														CFIEEIPDETMVIGNYR	1	4	1	N-Term(iTRAQ8plex); C1(Methylthio); Y16(iTRAQ8plex)	0.783	0.614	0.521	-	37	0	2683.34049	2	1190.067139	-2.816821569	57.197335
39753957	TOR1AIP1	torsin-1A-interacting protein 1 isoform 2	NP_056417.2	10.46	4	4	7	583	66.2	8.18	0.727	0.436	0.518																
														CLSEQIADAYSSFR	2	4	1	N-Term(iTRAQ8plex); C1(Methylthio)	0.724	0.436	0.374	5.53	60	0	1939.924	2	970.4656372	2.281271878	45.633715
														SIQEAPVSEDLVIR	2	2	1	N-Term(iTRAQ8plex)	0.727	0.257	0.545	4.74	37	0	1860.03765	2	930.5224609	-0.218299345	33.12794667
														ATQDSDTVKLEVDQELSNGFK	1	4	1	N-Term(iTRAQ8plex); K9(iTRAQ8plex); K21(iTRAQ8plex)	-	-	-	3.61	-	1	3236.72182	4	809.9359131	-6.052060487	35.53893333
														SQPAILLLTAAR	2	4	1	N-Term(iTRAQ8plex)	0.85	0.549	0.518	3.56	38	0	1557.95781	2	779.4825439	-3.344140218	41.70721167
39777604	ARHGEF1	rho guanine nucleotide exchange factor 1 isoform 3	NP_945328.1	1.25	1	1	2	879	98.7	5.57	0.714	0.957	1.960																
														QLEELEEEFCR	2	3	1	N-Term(iTRAQ8plex); C10(Methylthio)	0.714	0.957	1.96	2.96	32	0	1774.83098	2	887.9191284	0.905954401	56.77390167
39995109	GM2A	ganglioside GM2 activator isoform 1 precursor	NP_000396.2	23.83	4	4	15	193	20.8	5.31	0.220	0.160	0.214																
														TYGLPCHCPFK	4	1	1	N-Term(iTRAQ8plex); C6(Methylthio); C8(Methylthio); K11(iTRAQ8plex)	0.171	0.157	0.209	3.57	27	0	1965.96842	3	655.9943237	0.841641906	41.832695
														EVAGLWIK	2	2	1	N-Term(iTRAQ8plex); K8(iTRAQ8plex)	0.232	0.127	0.198	3.37	43	0	1523.93779	2	762.4725342	-1.843618972	44.67317667
														SEFVVPDLELPSWLTTGNYR	6	1	1	N-Term(iTRAQ8plex)	0.092	0.033	0.109	3.36	42	0	2627.38327	3	876.4659424	6.596905221	63.12057167
														VDLVLEK	3	2	1	N-Term(iTRAQ8plex); K7(iTRAQ8plex)	0.301	0.248	0.241	3.18	39	0	1423.89336	2	712.4503174	-3.303024309	44.09031833
40217812	TBRG4	protein TBRG4 isoform 1	NP_004740.2	13.15	5	5	11	631	70.7	7.42	1.811	1.233	0.863																
														EAELQAVLHPEFHIQFLGGK	1	3	1	N-Term(iTRAQ8plex); K20(iTRAQ8plex)	-	-	-	6.27	-	0	2871.60139	5	575.1260986	1.248555624	49.748095
														CLELVEHFGPNELR	4	2	1	N-Term(iTRAQ8plex); C1(Methylthio)	1.494	1.233	0.863	4.12	33	0	2006.01706	3	669.3438721	1.445980192	57.886345
														LATDLLSLMPSLTSGEVAHCAK	3	2	1	N-Term(iTRAQ8plex); C20(Methylthio); K22(iTRAQ8plex)	-	-	-	3.99	25	0	2911.55556	4	728.6461182	2.548623252	64.49862333
														LNFHPDQEDQFFSLVHEK	1	3	1	N-Term(iTRAQ8plex); K18(iTRAQ8plex)	1.865	1.964	1.196	3.62	-	0	2838.47255	4	710.3735962	1.900171843	39.90908667
														WEFPNFNSR	2	3	1	N-Term(iTRAQ8plex)	1.811	0.749	0.695	2.81	19	0	1500.74016	2	750.8737183	-9.049798843	54.63458333
40254924	LRRC59	leucine-rich repeat-containing protein 59	NP_060979.2	25.41	7	7	29	307	34.9	9.57	1.419	0.398	0.667																
														LTTLPSDFCGLTHLVK	11	1	1	N-Term(iTRAQ8plex); C9(Methylthio); K16(iTRAQ8plex)	1.29	0.327	0.588	5.95	33	0	2399.32907	3	800.447876	0.142698095	57.70108
														ATILDLSCNK	6	1	1	N-Term(iTRAQ8plex); C8(Methylthio); K10(iTRAQ8plex)	1.462	0.586	0.767	4.51	53	0	1731.95469	3	577.9897461	-2.700572244	57.97228167
														WLDLKDNPLDPVLAK	6	1	1	N-Term(iTRAQ8plex); K5(iTRAQ8plex); K15(iTRAQ8plex)	2.797	0.493	0.984	4.37	30	1	2649.56522	3	883.8599243	-3.435129928	48.65118167
														LVNLQHLDLLNNK	2	1	1	N-Term(iTRAQ8plex); K13(iTRAQ8plex)	1.267	0.226	0.54	3.63	-	0	2142.27993	4	536.3254395	-2.615609392	40.918015
														LQQLPADFGR	2	1	1	N-Term(iTRAQ8plex)	1.587	0.4	0.668	3.19	31	0	1448.80864	2	724.907959	-5.321580111	25.38560167
														EYDALK	1	1	1	N-Term(iTRAQ8plex); K6(iTRAQ8plex)	1.003	0.633	0.829	2.71	-	0	1346.77837	2	673.8928223	0.576643692	16.93374667
														AVQADQER	1	1	1	N-Term(iTRAQ8plex)	1.531	0.183	0.577	-	23	0	1220.65068	2	610.8289795	-2.465025964	8.988331667
40316915	RNPEP	aminopeptidase B isoform a	NP_064601.3	7.85	4	4	10	650	72.5	5.74	0.820	1.725	1.498																
														LDSHPCLEVTAAALR	2	1	1	N-Term(iTRAQ8plex); C6(Methylthio)	0.82	1.322	1.149	4.16	30	0	1946.00699	3	649.3405151	-3.68453961	35.59717333
														AFFPCFDTPAVK	2	2	1	N-Term(iTRAQ8plex); C5(Methylthio); K12(iTRAQ8plex)	0.808	1.725	1.498	4.13	35	0	1997.03647	3	666.3503418	-6.039028102	40.64150833
														AFELLHLHLDLR	4	1	1	N-Term(iTRAQ8plex)	-	-	-	3.21	28	0	1781.02944	4	446.0128174	-4.571316211	47.47467333
														RVDIIPGFEFDR	2	3	1	N-Term(iTRAQ8plex)	1.292	1.754	1.567	2.71	37	1	1767.96146	3	589.9920044	-4.59363317	35.07146
40317626	THBS1	thrombospondin-1 precursor	NP_003237.2	8.55	8	8	21	1170	129.3	4.94	0.929	0.250	0.327																
														DCVGDVTENQICNK	2	1	1	N-Term(iTRAQ8plex); C2(Methylthio); C12(Methylthio); K14(iTRAQ8plex)	0.954	0.141	0.377	5.12	49	0	2238.04209	3	746.6855469	-2.799735106	52.28652167
														IEDANLIPPVPDDKFQDLVDAVR	4	2	1	N-Term(iTRAQ8plex); K14(iTRAQ8plex)	1.046	0.135	-	4.26	33	1	3187.7424	3	1063.252319	-1.134515192	55.62662167
														SITLFVQEDR	1	2	1	N-Term(iTRAQ8plex)	0.899	0.257	0.191	4.12	-	0	1511.84441	2	756.4258423	4.806480266	32.77658167
														TIVTTLQDSIR	4	2	1	N-Term(iTRAQ8plex)	0.833	0.239	0.252	3.8	37	0	1550.91289	2	775.960083	4.731468592	36.62400333
														FVFGTTPEDILR	2	2	1	N-Term(iTRAQ8plex)	0.54	0.088	0.075	3.33	30	0	1698.93498	2	849.9711304	-1.11080049	48.52408667
														NALWHTGNTPGQVR	1	3	1	N-Term(iTRAQ8plex)	0.929	0.679	0.36	3.25	-	0	1854.986	3	619.0001831	-0.903097704	18.58558167
														FYVVMWK	5	16	1	N-Term(iTRAQ8plex); K7(iTRAQ8plex)	1.025	0.243	0.211	3.09	28	0	1580.90714	3	527.640564	-3.031585696	45.26716667
														GFLLLASLR	2	2	1	N-Term(iTRAQ8plex)	1.118	0.349	0.529	2.84	36	0	1293.8178	3	431.9454346	1.630178915	43.54399167
40353734	NPM1	nucleophosmin isoform 2	NP_954654.1	38.49	10	10	152	265	29.4	4.61	1.168	0.683	0.774																
														MTDQEAIQDLWQWR	16	3	1	N-Term(iTRAQ8plex); M1(Oxidation)	1.124	0.547	0.849	7.63	69	0	2140.06473	2	1062.526489	-1.383870588	52.59252333
														DELHIVEAEAMNYEGSPIK	12	4	1	N-Term(iTRAQ8plex); K19(iTRAQ8plex)	1.149	0.609	0.688	7.12	69	0	2753.42348	3	918.4785156	-2.428860322	39.29288167
														TVSLGAGAKDELHIVEAEAMNYEGSPIK	6	4	1	N-Term(iTRAQ8plex); K9(iTRAQ8plex); K28(iTRAQ8plex)	2.107	-	-	6.5	32	1	3842.07314	3	1281.360107	-3.02500922	55.94864833
														MTDQEAIQDLWQWR	23	3	1	N-Term(iTRAQ8plex)	1.084	1.026	0.816	6.12	83	0	2124.0457	2	1062.526489	-1.383870588	52.59252333
														MSVQPTVSLGGFEITPPVVLR	4	5	1	N-Term(iTRAQ8plex); M1(Oxidation)	1.297	0.879	0.84	5.58	97	0	2547.40898	2	1274.20813	-2.713798381	53.17188167
														DELHIVEAEAMNYEGSPIK	2	4	1	N-Term(iTRAQ8plex); M11(Oxidation); K19(iTRAQ8plex)	-	-	-	5.57	52	0	2769.4164	3	918.4785156	-2.428860322	39.29288167
														MSVQPTVSLGGFEITPPVVLR	15	5	1	N-Term(iTRAQ8plex)	1.321	0.728	0.804	5.35	77	0	2531.41631	2	1274.20813	-2.713798381	53.17188167
														VDNDENEHQLSLR	54	4	1	N-Term(iTRAQ8plex)	1.117	0.636	0.717	5.22	79	0	1872.93381	3	624.9827881	-0.827734637	17.53305667
														TVSLGAGAK	4	4	1	N-Term(iTRAQ8plex); K9(iTRAQ8plex)	1.587	0.93	0.831	4.19	33	0	1411.87671	3	471.2970886	2.692387449	22.94215667
														MQASIEK	6	5	1	N-Term(iTRAQ8plex); M1(Oxidation); K7(iTRAQ8plex)	1.382	0.776	0.951	3.96	40	0	1430.81595	3	477.6101685	1.821213336	12.62289
														LISISGK	5	12	1	N-Term(iTRAQ8plex); K7(iTRAQ8plex)	2.127	1.217	1.233	3.53	29	0	1325.86301	3	442.6258545	1.31154316	28.93202
														TVSLGAGAKDELHIVEAEAMNYEGSPIK	1	4	1	N-Term(iTRAQ8plex); K9(iTRAQ8plex); M20(Oxidation); K28(iTRAQ8plex)	-	-	-	3.39	-	1	3858.05799	3	1281.360107	-3.02500922	55.94864833
														GPSSVEDIK	4	5	1	N-Term(iTRAQ8plex); K9(iTRAQ8plex)	1.553	0.843	0.905	3.12	39	0	1539.87407	2	770.4406738	-6.364328054	26.25423833
														FINYVKNCFR	1	3	1	N-Term(iTRAQ8plex); K6(iTRAQ8plex); C8(Methylthio)	-	-	-	2.85	-	1	1958.05129	4	490.26828	-4.48510356	56.19341167
														FINYVK	4	3	1	N-Term(iTRAQ8plex); K6(iTRAQ8plex)	1.123	0.668	0.772	2.7	26	0	1391.85198	2	696.4296265	0.923237319	29.52461333
40354195	KRT18	keratin, type I cytoskeletal 18	NP_954657.1	59.77	22	24	199	430	48.0	5.45	1.496	0.655	0.374																
														GGMGSGGLATGIAGGLAGMGGIQNEK	2	1	1	N-Term(iTRAQ8plex); M3(Oxidation); K26(iTRAQ8plex)	1.184	0.806	0.489	9.37	67	0	2885.52225	3	962.5122681	5.25889177	35.89046333
														YALQMEQLNGILLHLESELAQTR	26	1	1	N-Term(iTRAQ8plex)	1.587	0.697	-	9	67	0	2974.58347	3	992.2074585	3.493212855	69.957305
														LQLETEIEALKEELLFMK	5	1	1	N-Term(iTRAQ8plex); K11(iTRAQ8plex); K18(iTRAQ8plex)	2.609	0.283	-	7.44	46	1	3089.80068	4	773.2046509	1.033186277	67.07783
														LQLETEIEALKEELLFMK	8	1	1	N-Term(iTRAQ8plex); K11(iTRAQ8plex); M17(Oxidation); K18(iTRAQ8plex)	1.99	1.199	-	7.28	35	1	3105.79018	4	773.2046509	1.033186277	67.07783
														GLQAQIASSGLTVEVDAPK	6	1	1	N-Term(iTRAQ8plex); K19(iTRAQ8plex)	2.635	1.271	1.308	6.35	55	0	2492.41458	3	831.4763794	-1.410827247	35.55167
														TVQSLEIDLDSMR	10	1	1	N-Term(iTRAQ8plex)	1.683	0.66	0.268	5.73	63	0	1810.95	2	905.9874268	8.451612177	42.05515833
														YALQMEQLNGILLHLESELAQTR	10	1	1	N-Term(iTRAQ8plex); M5(Oxidation)	1.802	0.51	0.641	5.62	48	0	2990.60923	3	992.2074585	3.493212855	69.957305
														QAQEYEALLNIK	14	1	1	N-Term(iTRAQ8plex); K12(iTRAQ8plex)	1.65	0.755	0.571	5.23	67	0	2028.14885	2	1014.58136	-1.552244651	31.251545
														QSVENDIHGLR	22	1	1	N-Term(iTRAQ8plex)	1.233	0.651	0.456	4.97	63	0	1571.84258	3	524.6185913	-1.991692404	18.57982333
														VKLEAEIATYR	7	1	1	N-Term(iTRAQ8plex); K2(iTRAQ8plex)	1.642	0.754	0.299	4.39	29	1	1901.12224	4	476.0368042	-3.295092038	29.69949
														QSVENDIHGLRK	4	1	1	N-Term(iTRAQ8plex); K12(iTRAQ8plex)	2.309	1.189	0.708	4.2	32	1	2004.14028	4	501.7905273	-2.196355737	18.000405
														TVQSLEIDLDSMR	8	1	1	N-Term(iTRAQ8plex); M12(Oxidation)	1.646	0.59	0.347	4.12	52	0	1826.94585	2	905.9874268	8.451612177	42.05515833
														LEAEIATYR	10	1	1	N-Term(iTRAQ8plex)	1.282	0.582	0.318	3.52	50	0	1369.75833	3	457.2576294	-3.333702223	33.54503
														LLEDGEDFNLGDALDSSNSMQTIQK	2	1	1	N-Term(iTRAQ8plex); M20(Oxidation); K25(iTRAQ8plex)	-	-	-	3.47	30	0	3364.66054	4	841.9205933	-2.087322936	43.61158167
														NHEEEVK	1	1	1	N-Term(iTRAQ8plex); K7(iTRAQ8plex)	1.597	0.961	0.504	3.28	-	0	1492.8086	3	498.2743835	-8.704341104	9.64287
														YETELAMR	4	1	1	N-Term(iTRAQ8plex)	1.361	0.609	0.272	3.2	35	0	1316.67681	2	658.842041	-4.105706676	18.49647
														AQYDELAR	7	1	1	N-Term(iTRAQ8plex)	1.448	0.672	0.277	3.12	29	0	1269.67278	2	635.3382568	-3.815362288	14.941755
														LLEDGEDFNLGDALDSSNSMQTIQK	1	1	1	N-Term(iTRAQ8plex); K25(iTRAQ8plex)	1.17	0.374	0.33	3.11	-	0	3348.6489	4	841.9205933	-2.087322936	43.61158167
														VKYETELAMR	3	1	1	N-Term(iTRAQ8plex); K2(iTRAQ8plex)	1.633	0.774	0.294	3.08	-	1	1848.04656	2	924.5269165	-2.383550443	23.433425
														AQIFANTVDNAR	1	1	1	N-Term(iTRAQ8plex)	0.929	0.856	0.756	2.98	-	0	1623.86553	2	812.4364014	-6.241394661	24.442905
														VIDDTNITR	7	1	1	N-Term(iTRAQ8plex)	1.62	0.677	0.353	2.82	34	0	1350.74883	2	675.8779297	-3.323005522	16.434125
														STFSTNYR	1	1	1	N-Term(iTRAQ8plex)	1.201	0.583	0.514	2.73	-	0	1279.65801	2	640.3326416	-0.31626956	16.45575667
														SQDLAK	1	1	1	N-Term(iTRAQ8plex); K6(iTRAQ8plex)	1.273	0.64	0.281	2.69	-	0	1269.76482	2	635.3860474	1.99785545	11.26256
														LASYLDR	7	1	1	N-Term(iTRAQ8plex)	1.429	0.577	0.205	-	34	0	1141.6519	2	571.3295898	0.01906667	26.18362667

														ETMQSLNDR	1	3	1	N-Term(iTRAQ8plex)	1.275	0.557	0.328	-	32	0	1397.69548	2	699.3513794	-2.990015344	14.38244667
														YALQMEQLNGILLHLESELAQTR	3	1	1	N-Term(iTRAQ8plex); Y1(iTRAQ8plex)	0.913	-	-	-	27	0	3278.82461	3	992.2074585	3.493212855	69.957305
														IMADIR	1	1	1	N-Term(iTRAQ8plex)	1.32	0.726	0.401	-	26	0	1022.59667	2	511.8019714	-0.356982296	26.411485
														LAADDFR	10	31	7	N-Term(iTRAQ8plex)	-	-	-	-	39	0	1111.60307	2	556.3051758	-1.68797437	21.86801333
														IVLQIDNAR	17	2	2	N-Term(iTRAQ8plex)	-	-	-	3.65	63	0	1345.80796	3	449.2741699	-1.913188552	31.11273333
40354201	DNPH1	2'-deoxynucleoside 5'-phosphate N-hydrolase 1 isoform 2	NP_954653.1	11.49	1	1	2	148	16.2	6.79	0.622	1.940	2.517																
														FGTVLTEHVAAAELGAR	2	2	1	N-Term(iTRAQ8plex)	0.622	1.94	2.517	3.93	38	0	2046.12339	3	682.7126465	-2.553656788	39.77114
40538806	EBLN2	endogenous Bornavirus-like nucleoprotein 2	NP_060499.3	3.31	1	1	1	272	30.4	9.04	1.175	1.676	0.886																
														SFRPMILNK	1	1	1	N-Term(iTRAQ8plex); K9(iTRAQ8plex)	1.175	1.676	0.886	2.73	-	0	1714.02152	3	572.0120239	-4.622329749	37.13148
40789233	COQ9	ubiquinone biosynthesis protein COQ9, mitochondrial precursor	NP_064708.1	3.46	1	1	2	318	35.5	5.94	1.101	1.167	1.074																
														MLIPYIEHWPR	2	1	1	N-Term(iTRAQ8plex)	1.101	1.167	1.074	3.2	39	0	1758.96073	3	586.9917603	-3.407573681	49.35793333
40805827	COPE	coatomer subunit epsilon isoform c	NP_955476.1	14.45	2	2	4	256	28.8	5.29	0.823	1.146	1.585																
														NAFYIGSYQQCINEAQR	3	5	1	N-Term(iTRAQ8plex); C11(Methylthio)	0.709	0.767	0.897	3.53	35	0	2355.10837	2	1178.070557	7.415443224	41.38703833
														FGVVLDEIKPSSAPELQAVR	1	5	1	N-Term(iTRAQ8plex); K9(iTRAQ8plex)	0.857	1.722	1.665	3.01	-	0	2763.58371	3	921.8660889	-1.037764271	47.484585
40807504	AFAP1	actin filament-associated protein 1 isoform B	NP_940997.1	1.23	1	1	1	730	80.7	8.68	0.763	0.641	1.722																
														AAIEVNAGR	1	4	1	N-Term(iTRAQ8plex)	0.763	0.641	1.722	-	23	0	1204.69036	2	602.8488159	-3.997777555	19.814815
41152506	PTGFRN	prostaglandin F2 receptor negative regulator precursor	NP_065173.2	9.67	5	5	8	879	98.5	6.61	1.138	0.943	0.316																
														CSTPSTDATVQGNYEDTVQVK	2	2	1	N-Term(iTRAQ8plex); C1(Methylthio); K21(iTRAQ8plex)	0.913	0.82	0.27	8.15	58	0	2897.42905	3	966.4812012	5.970108439	28.97782333
														CIVSEWIAEQGNWQEIQEK	2	2	1	N-Term(iTRAQ8plex); C1(Methylthio); K19(iTRAQ8plex)	1.138	2.063	1.531	4.08	49	0	2944.47855	3	982.1643677	-0.289532643	48.28679333
														SDNVVTSELLAVMDGDWTLK	2	2	1	N-Term(iTRAQ8plex); K20(iTRAQ8plex)	3.542	2.129	0.603	3.62	40	0	2801.49332	3	934.5026245	2.894522395	59.263545
														TANDAVELHIK	1	2	1	N-Term(iTRAQ8plex); K11(iTRAQ8plex)	1.106	0.668	0.263	3.26	-	0	1819.05246	3	607.0223389	-0.515624731	19.40440667
														VPGFADDPTELACR	1	2	1	N-Term(iTRAQ8plex); C13(Methylthio)	1.267	0.943	0.316	-	51	0	1840.88652	2	920.9468994	-0.570427271	41.08395667
41281885	UQCR10	cytochrome b-c1 complex subunit 9 isoform a	NP_037519.2	38.10	2	2	6	63	7.3	9.47	0.951	0.469	0.518																
														AFDQGADAIYDHINEGK	4	1	1	N-Term(iTRAQ8plex); K17(iTRAQ8plex)	0.899	0.688	0.706	4.4	35	0	2472.26364	3	824.7593994	0.83473791	34.64442833
														LYSLLFR	2	2	1	N-Term(iTRAQ8plex)	0.951	0.329	0.387	-	21	0	1215.73552	2	608.375	1.992263621	50.23940833
41322910	PLEC	plectin isoform 1d	NP_958783.1	48.88	180	187	839	4515	512.3	5.77	0.629	0.336	0.360																
														DLLPSDMAVALLEAQAGTGHIIDPATSAR	10	22	1	N-Term(iTRAQ8plex)	0.56	0.169	0.166	8.05	75	0	3237.71115	3	1079.908569	0.597168098	50.70529167
														QTNLENLDQAFSVAER	2	22	1	N-Term(iTRAQ8plex)	0.673	0.656	1.109	7.7	51	0	2139.10929	3	713.7079468	5.079582468	37.04854
														LLEAQIATGGIIDPEESHR	8	22	1	N-Term(iTRAQ8plex)	0.903	0.571	0.671	7.47	73	0	2353.25681	3	785.0939331	0.286735987	38.68603167
														SEEMQTVQQEQLLQETQALQQSFLSEK	2	21	1	N-Term(iTRAQ8plex); K27(iTRAQ8plex)	0.681	0.238	0.146	7.31	107	0	3788.95456	3	1263.656372	1.900698681	51.01347833
														DALDGPAAEAEPEHSFDGLR	12	22	1	N-Term(iTRAQ8plex)	0.504	0.259	0.328	6.8	67	0	2401.15702	3	801.0571899	-0.172719143	27.90527833
														LEAQHQALVTLWHQLHVDMK	9	22	1	N-Term(iTRAQ8plex); K20(iTRAQ8plex)	0.52	0.257	0.398	6.64	42	0	3005.68613	3	1002.566895	8.55679623	50.933545
														TLLQGSGCLAGIYLEDTKEK	2	22	1	N-Term(iTRAQ8plex); C8(Methylthio); K18(iTRAQ8plex); K20(iTRAQ8plex)	-	0.324	0.35	6.57	49	1	3097.70522	4	775.1817627	0.364334619	46.07824333
														SQVMDEATALQLR	4	22	1	N-Term(iTRAQ8plex)	0.636	0.415	0.83	6.54	65	0	1765.95298	3	589.3225098	6.191980774	32.820265
														LLLEAQAATGFLLDPVKGER	12	22	1	N-Term(iTRAQ8plex); K17(iTRAQ8plex)	0.552	0.328	0.207	6.42	56	1	2749.60879	4	688.1559448	-1.953694834	57.37016167
														ATTAALLLEAQAATGFLVDPVR	6	22	1	N-Term(iTRAQ8plex)	0.699	0.185	0.36	6.3	62	0	2532.43888	3	844.8178101	1.929217651	66.848625
														CVEDPETGLCLLPLTDK	6	22	1	N-Term(iTRAQ8plex); C1(Methylthio); C10(Methylthio); K17(iTRAQ8plex)	0.563	0.233	0.196	6.15	62	0	2546.30644	2	1273.65686	9.001580771	50.89003
														GIYQSLEGAVQAGQLK	4	22	1	N-Term(iTRAQ8plex); K16(iTRAQ8plex)	0.699	0.519	0.691	5.86	67	0	2270.29374	3	757.4360962	-1.218333883	39.57831167
														LLEAQIATGGIIDPEESHRLPVEVAYK	4	22	1	N-Term(iTRAQ8plex); K27(iTRAQ8plex)	-	-	-	5.82	30	1	3556.99428	4	890.0040283	2.99750306	47.73865
														LSVAAQEAAR	5	21	1	N-Term(iTRAQ8plex)	0.532	0.244	0.266	5.78	69	0	1319.76633	3	440.5936279	5.932291862	24.98149333
														SSSVGSSSSYPISPAVSR	6	22	1	N-Term(iTRAQ8plex)	0.591	0.32	0.351	5.76	71	0	2059.05766	2	1030.032471	-1.596125163	19.273245
														EAQAVPATLPELEATK	2	22	1	N-Term(iTRAQ8plex); K16(iTRAQ8plex)	0.558	0.235	0.318	5.74	64	0	2276.31206	2	1138.659668	7.120296813	31.73714
														GFFDPNTEENLTYLQLMER	4	22	1	N-Term(iTRAQ8plex)	0.625	0.251	0.229	5.71	85	0	2621.28667	2	1311.146973	0.262234768	53.18107667
														VDGAEWGVDLPSVEAQLGSHR	9	22	1	N-Term(iTRAQ8plex)	0.91	0.956	0.861	5.71	77	0	2526.28965	3	842.7695313	1.951158464	37.82955
														LLDAQLSTGGIVDPSK	2	22	1	N-Term(iTRAQ8plex); K16(iTRAQ8plex)	1.034	0.3	0.52	5.71	63	0	2222.28312	3	741.4325562	-0.974715127	37.03492833
														GANVIAGVWLEEAGQK	4	22	1	N-Term(iTRAQ8plex); K16(iTRAQ8plex)	0.782	0.32	0.431	5.67	52	0	2250.26053	3	750.7583618	-4.345763809	68.55700167
														YLQDLLAWVEENQHR	2	22	1	N-Term(iTRAQ8plex)	0.957	0.65	0.425	5.6	63	0	2218.15097	3	740.0551758	-2.203279356	63.94851333
														FLEVQYLTGGLIEPDTPGR	4	22	1	N-Term(iTRAQ8plex)	0.569	0.276	0.331	5.57	84	0	2409.29643	2	1205.151855	-0.152319364	60.504585
														DPYTEQTISLFQAMK	4	22	1	N-Term(iTRAQ8plex); K15(iTRAQ8plex)	0.615	0.3	0.321	5.46	65	0	2380.29106	2	1190.64917	9.730710714	44.43364667
														LQEAGILSAEELQR	8	22	1	N-Term(iTRAQ8plex)	0.645	0.367	0.373	5.4	70	0	1861.02544	2	931.0163574	-4.225130989	40.91064833
														FLEGTSCIAGVFVDATK	2	22	1	N-Term(iTRAQ8plex); C7(Methylthio); K17(iTRAQ8plex)	0.786	0.436	0.347	5.39	57	0	2412.28232	3	804.765625	2.462170347	55.50444833
														LLDAQLATGGIVDPR	6	22	1	N-Term(iTRAQ8plex)	0.66	0.468	0.388	5.38	88	0	1843.05303	2	922.0301514	-3.312539324	43.539755
														QYINAIKDYELQLVTYK	4	22	1	N-Term(iTRAQ8plex); K7(iTRAQ8plex); K17(iTRAQ8plex)	0.317	0.348	0.398	5.32	35	1	3014.73574	4	754.4393921	0.921201597	51.01763333
														GLFDEEMNEILTDPSDDTK	6	22	1	N-Term(iTRAQ8plex); K19(iTRAQ8plex)	0.505	0.56	0.626	5.29	46	0	2777.36222	3	931.7889404	-2.673583644	35.026155
														AALAHSEEVTASQVAATK	5	22	1	N-Term(iTRAQ8plex); K18(iTRAQ8plex)	0.665	0.334	0.384	5.22	51	0	2392.3295	3	798.1146851	0.096402181	26.371105
														ESADPLGAWLQDAR	6	22	1	N-Term(iTRAQ8plex)	0.713	0.381	0.316	5.19	64	0	1832.9498	2	916.9810791	5.668597318	42.11319167
														GYYSPYSVSGSGSTAGSR	4	22	1	N-Term(iTRAQ8plex)	0.878	0.394	0.377	5.17	75	0	2087.01055	2	1044.008911	5.852555777	24.89093667
														LQMEAGLCEEQLNQADALLQSDVR	2	22	1	N-Term(iTRAQ8plex); C8(Methylthio)	-	-	-	5.09	96	0	3024.47495	3	1008.829834	0.210417721	66.83051
														RDDGTGQLLLPLSDAR	8	22	1	N-Term(iTRAQ8plex)	0.867	0.712	0.626	5.06	60	1	2031.1124	3	677.7089844	-0.635821533	27.913345
														AGVVGPELHEQLLSAEK	6	22	1	N-Term(iTRAQ8plex); K17(iTRAQ8plex)	0.631	0.343	0.427	5.05	34	0	2385.36454	3	795.7930298	1.963978838	30.32923667
														LQEDKEQMAQQLAEETQGFQR	3	21	1	N-Term(iTRAQ8plex); K5(iTRAQ8plex)	0.796	-	0.158	5.05	42	1	3115.59243	4	779.6535645	-0.48253022	43.01603667
														SLQEEHVAVAQLR	8	21	1	N-Term(iTRAQ8plex)	0.524	0.272	0.413	5.01	50	0	1783.9948	2	892.5008545	-1.350304452	22.27345833
														IITVVEEQEQK	4	22	1	N-Term(iTRAQ8plex); K12(iTRAQ8plex)	0.484	0.203	0.316	4.92	47	0	2037.19919	3	679.737915	-2.959667782	35.66365333
														LLFNDVQTLK	6	22	1	N-Term(iTRAQ8plex); K10(iTRAQ8plex)	0.597	0.398	0.458	4.92	45	0	1799.08171	2	900.0444946	-3.895688639	63.37283
														FLEVQYLTGGLIEPDTPGRVPLDEALQR	3	22	1	N-Term(iTRAQ8plex)	0.622	0.168	0.211	4.91	31	1	3430.88156	3	1144.298706	8.471179641	56.59043167
														SQVEEELFSVR	10	21	1	N-Term(iTRAQ8plex)	0.724	0.406	0.391	4.91	43	0	1626.86936	3	542.9613037	3.23724999	36.07196
														RQEQIQAMPLADSQAVR	6	22	1	N-Term(iTRAQ8plex)	0.639	0.717	0.58	4.9	44	1	2245.19345	3	749.0693359	-4.042741832	20.198
														APVPASELLASGVLSR	6	22	1	N-Term(iTRAQ8plex)	0.626	0.264	0.3	4.88	64	0	1871.08708	2	936.0471802	-1.789103493	44.31727333
														DTHDQLSEPSEVR	8	22	1	N-Term(iTRAQ8plex)	0.378	0.317	0.303	4.82	57	0	1816.89629	2	908.9517822	-0.89353855	13.835955
														LQNVQIALDYLR	14	22	1	N-Term(iTRAQ8plex)	0.576	0.309	0.339	4.77	55	0	1750.01531	2	875.5112915	-0.682800992	55.134455
														CITDPQTGLCLLPLK	5	22	1	N-Term(iTRAQ8plex); C1(Methylthio); C10(Methylthio); K15(iTRAQ8plex)	0.51	0.123	0.59	4.76	55	0	2315.25542	2	1158.131348	4.239442621	51.66702667
														LKAEAELLQQQK	4	21	1	N-Term(iTRAQ8plex); K2(iTRAQ8plex); K12(iTRAQ8plex)	0.737	0.36	0.343	4.74	36	1	2311.41318	3	771.1425781	0.834911307	28.08801
														LEDLLQDAQDEKEQLNEYK	2	22	1	N-Term(iTRAQ8plex); K12(iTRAQ8plex); K19(iTRAQ8plex)	0.66	-	0.508	4.71	-	1	3233.71694	4	809.1846924	-4.193826795	58.94287167
														QQEELLAEENQR	2	22	1	N-Term(iTRAQ8plex)	0.6	0.247	0.277	4.66	63	0	1790.91399	2	895.9606323	-2.603850098	15.70119333
														LLEAQACTGGIIDPSTGER	2	22	1	N-Term(iTRAQ8plex); C7(Methylthio)	0.677	0.784	0.797	4.66	34	0	2281.14859	3	761.0543823	0.675722161	48.03042667
														QALHSLELHYQAFLR	6	22	1	N-Term(iTRAQ8plex)	0.507	0.311	0.58	4.65	32	0	2130.17275	4	533.2990723	-0.813134104	43.83475333
														EGLTSIEEVTK	6	22	1	N-Term(iTRAQ8plex); K11(iTRAQ8plex)	0.785	0.366	0.383	4.65	51	0	1814.03606	2	907.5216675	-0.381998505	37.06908
														GLFDEEMNEILTDPSDDTK	2	22	1	N-Term(iTRAQ8plex); M7(Oxidation); K19(iTRAQ8plex)	0.673	0.362	0.217	4.62	57	0	2793.35227	3	931.7889404	-2.673583644	35.026155
														AGTLSITEFADMLSGNAGGFR	4	22	1	N-Term(iTRAQ8plex)	0.675	0.237	0.235	4.55	78	0	2419.22124	2	1210.114258	-0.732589054	63.769115
														QLEMSAEAER	11	21	1	N-Term(iTRAQ8plex)	0.694	0.295	0.288	4.53	53	0	1467.74549	3	489.9200134	2.688295238	22.81348333
														DGHNLISLLEVLSGDSLPR	6	22	1	N-Term(iTRAQ8plex)	-	0.796	-	4.5	42	0	2339.29384	3	780.43573	2.280771256	65.172555
														GGELVYTDSEAR	6	22	1	N-Term(iTRAQ8plex)	0.599	0.212	0.202	4.49	56	0	1600.81987	2	800.9135742	4.885320102	23.29751167
														QTLSIYQALK	2	22	1	N-Term(iTRAQ8plex); K10(iTRAQ8plex)	0.585	0.35	0.487	4.45	47	0	1773.06597	2	887.0366211	-3.996316346	47.168165
														CRPDQLTGLSLLPLSEK	8	22	1	N-Term(iTRAQ8plex); C1(Methylthio); K17(iTRAQ8plex)	0.656	0.304	0.424	4.45	38	0	2524.40311	3	842.1392212	-2.250819514	46.61400667
														SIITYVSSLYDAMPR	6	22	1	N-Term(iTRAQ8plex)	0.568	0.32	0.306	4.45	53	0	2020.07956	3	674.0313721	3.396728061	64.55360833
														LFNAIIHR	42	22	1	N-Term(iTRAQ8plex)	0.577	0.279	0.3	4.45	62	0	1287.77845	3	429.9320679	-1.755683285	34.77273167
														DSQDAGGFGPEDR	6	22	1	N-Term(iTRAQ8plex)	0.568	0.277	0.287	4.44	71	0	1654.75774	2	827.8825073	-2.032485774	14.24123833
														SELELTLGK	5	22	1	N-Term(iTRAQ8plex); K9(iTRAQ8plex)	0.697	0.434	0.342	4.42	30	0	1597.96012	2	799.4831543	-1.933295027	37.99904833
														VTLVQTLEIQR	8	21	1	N-Term(iTRAQ8plex)	0.733	0.328	0.398	4.37	58	0	1603.97495	3	535.329834	4.025875298	37.70822667
														IISLETYNLLR	13	22	1	N-Term(iTRAQ8plex)	0.573	0.241	0.276	4.37	64	0	1638.97647	3	546.9970093	1.978845994	47.51218833
														AKLEQLFQDEVAK	8	22	1	N-Term(iTRAQ8plex); K2(iTRAQ8plex); K13(iTRAQ8plex)	0.843	0.339	0.272	4.33	41	1	2431.42885	3	811.1488647	-0.144076294	39.59052333
														GLVGPELHDR	2	22	1	N-Term(iTRAQ8plex)	0.364	0.537	0.487	4.3	45	0	1396.78305	3	466.2658691	-1.429805523	16.22996333
														RQEQIQAMPLADSQAVR	6	22	1	N-Term(iTRAQ8plex); M8(Oxidation)	0.645	0.594	0.548	4.3	37	1	2261.19815	3	749.0693359	-4.042741832	20.198
														TVTIWEIINSEYFTAEQR	2	22	1	N-Term(iTRAQ8plex)	0.635	0.226	0.24	4.28	54	0	2504.31597	2	1252.661621	7.368985285	62.52004833
														LISLFQAMK	6	22	1	N-Term(iTRAQ8plex); K9(iTRAQ8plex)	0.433	0.139	0.201	4.27	35	0	1659.0159	3	553.6768188	2.116913383	50.33185667
														TVTVWELISSEYFTAEQR	2	22	1	N-Term(iTRAQ8plex)	1.57	1.442	1.613	4.26	37	0	2463.29276	3	821.769104	8.848912352	63.58775833
														QVEEEILALK	6	21	1	N-Term(iTRAQ8plex); K10(iTRAQ8plex)	0.604	0.439	0.586	4.22	55	0	1780.06389	2	890.5355835	-2.118422733	66.46592333
														LQAEEVAQQK	2	21	1	N-Term(iTRAQ8plex); K10(iTRAQ8plex)	0.736	0.311	0.377	4.14	45	0	1752.00766	3	584.6740723	-2.01925665	27.76408667
														AQLEPVASPAK	4	22	1	N-Term(iTRAQ8plex); K11(iTRAQ8plex)	0.598	0.25	0.313	4.11	51	0	1719.02141	2	860.0143433	-2.746560651	16.13900667
														AGLVGPEFHEK	10	22	1	N-Term(iTRAQ8plex); K11(iTRAQ8plex)	0.684	0.33	0.343	4.1	39	0	1792.02079	3	598.0117798	-0.332135953	21.30361333
														QEELYSELQAR	6	22	1	N-Term(iTRAQ8plex)	0.57	0.288	0.266	4.05	40	0	1669.87479	3	557.2964478	2.933580259	30.587885
														ELMWLNEKEEEEVGFDWSDR	1	22	1	N-Term(iTRAQ8plex); M3(Oxidation); K8(iTRAQ8plex)	0.88	0.889	0.816	4.05	-	1	3165.5353	4	792.1392822	1.795186505	46.60558167
														SLAAEEEAAR	7	21	1	N-Term(iTRAQ8plex)	0.689	0.395	0.39	4.04	54	0	1350.72306	3	450.9125366	4.705119418	21.16151
														LPLLAVCDYK	3	22	1	N-Term(iTRAQ8plex); C7(Methylthio); K10(iTRAQ8plex)	0.683	0.344	0.314	4.01	41	0	1789.01482	2	895.0110474	-3.585321582	47.04547833
														EQLQLLEEQHR	9	22	1	N-Term(iTRAQ8plex)	0.556	0.484	0.443	3.99	81	0	1726.93694	3	576.3170776	-1.33278763	22.70101333
														GFFDPNTHENLTYR	12	22	1	N-Term(iTRAQ8plex)	0.669	0.352	0.391	3.98	52	0	2014.9865	2	1007.996887	-2.964538941	28.31324
														VLALPEPSPAAPTLR	4	22	1	N-Term(iTRAQ8plex)	0.535	0.281	0.391	3.94	60	0	1836.0855	2	918.5463867	-2.289916514	38.45442833
														LTVDEAVR	5	22	1	N-Term(iTRAQ8plex)	0.623	0.226	0.253	3.91	51	0	1206.70539	3	402.9066467	4.811145794	24.50237333
														LAQGHTTVDELAR	10	22	1	N-Term(iTRAQ8plex)	0.64	0.478	0.489	3.9	60	0	1714.93523	2	857.9721069	-1.197742475	18.68550333
														VVIVDPETGKEMSVYEAYR	3	22	1	N-Term(iTRAQ8plex); K10(iTRAQ8plex)	0.555	0.193	0.338	3.9	-	1	2793.49453	4	699.1290894	-0.298464206	36.95261333
														TPVEVPVGGFK	2	22	1	N-Term(iTRAQ8plex); K11(iTRAQ8plex)	0.536	0.205	0.18	3.89	44	0	1738.03154	2	869.5194092	-2.548607732	23.17482833
														LHVAILER	13	22	1	N-Term(iTRAQ8plex)	0.515	0.507	0.556	3.87	50	0	1254.779	3	418.9316711	-2.487385136	20.43246
														SLSAIYLEK	4	22	1	N-Term(iTRAQ8plex); K9(iTRAQ8plex)	0.528	0.272	0.279	3.87	53	0	1631.98076	2	816.4940186	-1.275021542	41.57268
														QVEVTVHK	2	22	1	N-Term(iTRAQ8plex); K8(iTRAQ8plex)	0.601	0.463	0.508	3.84	31	0	1547.93955	3	516.6513672	1.929669995	19.98349
														SSIAGLLLK	6	22	1	N-Term(iTRAQ8plex); K9(iTRAQ8plex)	0.686	0.278	0.247	3.83	47	0	1509.98271	2	755.4949951	0.167097526	57.16389

														LLEAQACTGGIIDPSTGERFPVTDAVNK	2	22	1	N-Term(iTRAQ8plex); C7(Methylthio); K28(iTRAQ8plex)	0.511	-	0.207	3.83	31	1	3556.86582	3	1186.293457	1.609715981	42.22087833
														LTAEDLFEAR	11	22	1	N-Term(iTRAQ8plex)	0.526	0.273	0.31	3.82	65	0	1468.79253	3	490.2683411	-3.051052367	37.456465
														AVTGYRDPYTEQTISLFQAMK	3	22	1	N-Term(iTRAQ8plex); K21(iTRAQ8plex)	0.869	0.507	0.353	3.81	25	1	3027.61118	3	1009.875244	1.370014718	45.093825
														AYSDPSTGEPATYGELQQR	2	22	1	N-Term(iTRAQ8plex)	0.527	0.353	0.602	3.8	50	0	2374.1453	3	792.0532837	-0.498484984	22.44526167
														NDDIADGNPK	2	22	1	N-Term(iTRAQ8plex); K10(iTRAQ8plex)	0.602	0.336	0.33	3.8	41	0	1666.8853	2	833.9462891	-0.221847898	13.253105
														SIQEELQQLR	5	21	1	N-Term(iTRAQ8plex)	0.76	0.318	0.341	3.79	40	0	1547.87749	2	774.4351196	-4.224315941	26.98666
														EQLRQEQALLEEIER	3	22	1	N-Term(iTRAQ8plex)	0.578	0.542	0.33	3.77	30	1	2188.1821	3	730.0655518	-2.504031341	37.765735
														GRLPLLAVCDYK	15	22	1	N-Term(iTRAQ8plex); C9(Methylthio); K12(iTRAQ8plex)	0.712	0.377	0.344	3.7	31	1	2002.14219	3	668.0522461	-0.81775848	45.94291667
														LSIYNALKK	3	22	1	N-Term(iTRAQ8plex); K8(iTRAQ8plex); K9(iTRAQ8plex)	0.832	0.329	0.452	3.7	19	1	1962.25405	4	491.3193054	1.997955875	37.33577167
														AQAEVEGLGK	2	22	1	N-Term(iTRAQ8plex); K10(iTRAQ8plex)	0.569	0.249	0.336	3.63	45	0	1609.93814	3	537.3175659	0.722514657	29.08549833
														NLLDEELQR	3	21	1	N-Term(iTRAQ8plex)	0.622	0.388	0.503	3.62	41	0	1433.78838	2	717.3978271	-1.264916883	29.284795
														LYVHEAVK	1	22	1	N-Term(iTRAQ8plex); K8(iTRAQ8plex)	0.641	0.371	0.401	3.6	-	0	1566.94791	3	522.9874878	0.969291255	23.79990833
														SLVPAAELLESR	6	22	1	N-Term(iTRAQ8plex)	0.647	0.32	0.398	3.59	43	0	1588.91802	2	794.9626465	-2.017065209	40.79958
														ALQALEELR	12	21	1	N-Term(iTRAQ8plex)	0.615	0.298	0.275	3.59	48	0	1346.79228	2	673.8984375	-3.67757518	34.46689333
														HKPLLIDMNK	2	22	1	N-Term(iTRAQ8plex); K2(iTRAQ8plex); K10(iTRAQ8plex)	0.265	0.602	0.269	3.58	-	0	2121.30214	4	531.0809937	1.891964983	19.66967833
														RPELEDSTLR	7	22	1	N-Term(iTRAQ8plex)	0.652	0.475	0.373	3.57	35	0	1519.83623	2	760.4216919	-1.384445946	15.256925
														AQAEAQQPTFDALRDELR	4	22	1	N-Term(iTRAQ8plex)	0.696	0.255	0.433	3.57	-	1	2363.22379	3	788.4127808	-0.838796923	32.03998
														QQGLASYDYVR	2	22	1	N-Term(iTRAQ8plex)	0.585	0.29	0.312	3.56	36	0	1603.83354	2	802.4204102	-2.891576572	18.76542333
														LGFHLPLEVAYQR	8	22	1	N-Term(iTRAQ8plex)	0.683	0.814	0.908	3.55	36	0	1847.04984	3	616.3547974	0.919054705	41.73308667
														KGLVGPELHDR	3	22	1	N-Term(iTRAQ8plex); K1(iTRAQ8plex)	0.483	-	0.223	3.55	44	1	1829.08962	4	458.0278625	2.317554736	16.02055167
														DYELQLVTYK	2	22	1	N-Term(iTRAQ8plex); K10(iTRAQ8plex)	0.759	0.714	0.663	3.51	25	0	1880.05515	3	627.3565674	-3.932013147	49.14153833
														HISDLYEDLR	2	22	1	N-Term(iTRAQ8plex)	0.547	0.462	0.676	3.49	59	0	1564.83817	3	522.2842407	6.951273419	30.42702167
														LSVYAALQR	2	22	1	N-Term(iTRAQ8plex)	0.763	0.39	0.369	3.49	-	0	1324.79363	2	662.9004517	3.453670232	32.64380833
														ELMWLNEKEEEEVGFDWSDR	1	22	1	N-Term(iTRAQ8plex); K8(iTRAQ8plex)	0.515	0.308	0.296	3.45	-	1	3149.53049	4	792.1392822	1.795186505	46.60558167
														GLHQSIEEFR	5	22	1	N-Term(iTRAQ8plex)	0.484	0.548	0.492	3.38	44	0	1519.83013	2	760.4187012	8.589396624	21.18502333
														FLQEEAEK	5	21	1	N-Term(iTRAQ8plex); K8(iTRAQ8plex)	0.764	0.468	0.491	3.38	43	0	1601.89653	2	801.4519043	-1.866116937	25.98842
														GWLYYEAGQR	5	22	1	N-Term(iTRAQ8plex)	0.662	0.484	0.493	3.36	32	0	1546.78874	2	773.8980103	-4.426856747	39.192865
														AELELELGR	2	21	1	N-Term(iTRAQ8plex)	0.658	0.336	0.374	3.36	28	0	1333.77263	2	667.3899536	7.279581497	30.64393167
														QLAEAHAQAK	2	21	1	N-Term(iTRAQ8plex); K10(iTRAQ8plex)	0.543	0.296	0.336	3.36	-	0	1674.97666	3	558.9970703	1.138157652	13.96005
														NLVDNITGQR	2	22	1	N-Term(iTRAQ8plex)	0.596	0.271	0.213	3.35	29	0	1433.79582	2	717.4015503	-3.903857139	19.42880333
														VPPGYHPLDVEK	1	22	1	N-Term(iTRAQ8plex); K12(iTRAQ8plex)	0.506	0.183	0.182	3.35	-	0	1959.11679	3	653.7104492	0.404828468	23.13247
														LQLEACETR	6	22	1	N-Term(iTRAQ8plex); C6(Methylthio)	0.582	0.322	0.406	3.34	37	0	1412.7144	2	706.8604126	-3.11720992	23.18068167
														LREQLQLLEEQHR	1	22	1	N-Term(iTRAQ8plex)	0.608	0.424	0.333	3.34	-	1	1996.11904	4	499.7852173	-2.571008679	36.74418167
														LQAEEAER	2	21	1	N-Term(iTRAQ8plex)	0.584	0.263	0.294	3.34	33	0	1249.67119	2	625.3392334	1.74356014	17.98627333
														KQEELQQLEQQR	2	22	1	N-Term(iTRAQ8plex); K1(iTRAQ8plex)	0.408	0.336	0.263	3.33	35	1	2165.20456	3	722.4063721	-4.12355452	21.36461833
														LEEQAAQHK	2	21	1	N-Term(iTRAQ8plex); K9(iTRAQ8plex)	0.752	0.429	0.451	3.32	31	0	1661.94303	3	554.6525269	-0.050533377	14.91489167
														AQLMADFQAGR	5	22	1	N-Term(iTRAQ8plex)	0.691	0.421	0.478	3.31	34	0	1511.79473	2	756.401001	0.313556541	48.50101667
														VSITEAMHR	2	22	1	N-Term(iTRAQ8plex); M7(Oxidation)	0.659	0.462	0.567	3.31	33	0	1363.72574	3	455.2467651	-3.537470691	13.07062167
														LSIYNALK	1	22	1	N-Term(iTRAQ8plex); K8(iTRAQ8plex)	0.523	0.429	0.574	3.3	-	0	1529.95053	3	510.6550293	-0.396522215	52.162795
														LSYTQLLR	5	22	1	N-Term(iTRAQ8plex)	0.603	0.307	0.335	3.24	35	0	1297.78435	3	433.2640076	-0.517688847	34.33631167
														CITDPQTGLCLLPLKEK	1	22	1	N-Term(iTRAQ8plex); C1(Methylthio); C10(Methylthio); K15(iTRAQ8plex); K17(iTRAQ8plex)	1.167	0.528	0.923	3.15	-	1	2876.59121	4	719.9032593	0.929547921	47.11838167
														FRELAEEAAR	2	21	1	N-Term(iTRAQ8plex)	0.859	0.693	0.733	3.14	30	1	1495.81043	3	499.2749939	-4.454348238	28.02298333
														KGWLYYEAGQR	1	22	1	N-Term(iTRAQ8plex); K1(iTRAQ8plex)	0.657	0.321	0.307	3.13	-	1	1979.08829	3	660.3676147	-3.855468072	28.02704
														CDNFTSSWR	3	22	1	N-Term(iTRAQ8plex); C1(Methylthio)	0.431	0.297	0.312	3.06	20	0	1465.64763	2	733.3274536	-2.02074044	43.80229833
														RFPSSFEEIEILWSQFLK	3	22	1	N-Term(iTRAQ8plex); K18(iTRAQ8plex)	-	-	-	3.06	33	1	2864.58193	4	716.9009399	0.411433729	68.56101
														AALEEVER	2	21	1	N-Term(iTRAQ8plex)	0.623	0.405	0.42	3.04	36	0	1220.67705	2	610.8421631	-1.476023593	20.56026167
														GGAEGELQALR	4	21	1	N-Term(iTRAQ8plex)	0.619	0.256	0.28	3.03	47	0	1404.77287	2	702.8900757	-1.435500962	24.348345
														GCLDEETSR	2	22	1	N-Term(iTRAQ8plex); C2(Methylthio)	0.544	0.225	0.237	3.02	32	0	1359.61736	2	680.3123169	-0.937860149	18.439115
														DVAEVDTVR	2	22	1	N-Term(iTRAQ8plex)	0.624	0.402	0.241	3.02	32	0	1307.70757	2	654.3574219	-2.534335721	18.328195
														QAEVELASR	7	21	1	N-Term(iTRAQ8plex)	0.653	0.328	0.326	3	34	0	1306.72417	2	653.8657227	-2.060578326	23.66665
														VSIYEAMR	3	22	1	N-Term(iTRAQ8plex)	0.649	0.308	0.274	2.98	28	0	1272.69072	2	636.8479004	-3.030701288	36.456485
														AFCGFEDPR	3	22	1	N-Term(iTRAQ8plex); C3(Methylthio)	0.58	0.349	0.406	2.94	36	0	1391.64458	2	696.3259277	4.021535984	33.94248667
														RQELEAELAK	1	21	1	N-Term(iTRAQ8plex); K10(iTRAQ8plex)	0.644	0.423	0.515	2.92	-	1	1795.0477	3	599.020752	-3.180244523	23.43811333
														QSSEAEIQAK	2	21	1	N-Term(iTRAQ8plex); K10(iTRAQ8plex)	-	-	-	2.91	28	0	1698.94754	3	566.9873657	-0.422250842	17.59424333
														SLLAWQSLR	1	22	1	N-Term(iTRAQ8plex)	0.53	0.385	0.37	2.91	-	0	1377.81182	2	689.4095459	-2.75518259	55.44319833
														MVEGYQGLR	2	22	1	N-Term(iTRAQ8plex); M1(Oxidation)	0.694	0.668	0.485	2.9	30	0	1372.72593	3	458.2468262	4.559515858	22.74906833
														DLSELGSVR	2	22	1	N-Term(iTRAQ8plex)	0.666	0.416	0.49	2.85	25	0	1279.71147	2	640.359375	-3.506978013	25.86000667
														RQQEELLAEENQR	1	22	1	N-Term(iTRAQ8plex)	1.504	2.214	1.817	2.83	-	1	1947.01114	3	649.6752319	-4.431792418	14.42886667
														QLAEEDAAR	1	21	1	N-Term(iTRAQ8plex)	0.666	0.441	0.425	2.83	-	0	1306.68511	2	653.8461914	-4.113544629	13.375215
														TEAEIALK	1	21	1	N-Term(iTRAQ8plex); K8(iTRAQ8plex)	1.108	0.571	0.466	2.83	-	0	1482.90104	3	494.9718628	1.513134628	29.823635
														LQLEATER	3	21	1	N-Term(iTRAQ8plex)	0.664	0.356	0.325	2.8	25	0	1263.72429	2	632.3657837	2.571338413	24.66415
														AEVVETTQVYTEEETRR	2	22	1	N-Term(iTRAQ8plex)	0.618	0.212	0.46	2.79	30	1	2344.19162	3	782.0689087	-0.541114246	27.51766
														ELIPTEEALR	3	22	1	N-Term(iTRAQ8plex)	0.723	0.33	0.363	2.76	27	0	1474.8405	2	737.9238892	-0.948983762	30.50080333
														FIEQEK	1	22	1	N-Term(iTRAQ8plex); K6(iTRAQ8plex)	0.625	0.376	0.396	2.74	-	0	1401.81681	3	467.9437866	-2.136289638	21.38904167
														LQLEETDHQK	1	21	1	N-Term(iTRAQ8plex); K10(iTRAQ8plex)	0.553	0.246	0.251	2.73	-	0	1849.02756	3	617.0140381	-0.00556627	16.60781167
														LVASMEEAR	2	22	1	N-Term(iTRAQ8plex)	0.837	0.457	0.516	2.72	23	0	1309.70659	2	655.3569336	-1.672689529	29.050825
														QLLEEELAR	2	21	1	N-Term(iTRAQ8plex)	0.664	0.307	0.304	2.69	32	0	1404.79717	2	702.9022217	-2.039127564	24.59792667
														SLAQAEAEK	1	21	1	N-Term(iTRAQ8plex); K9(iTRAQ8plex)	0.812	0.712	0.846	-	42	0	1554.90141	2	777.9543457	4.272609303	14.33258833
														YLQDLLAWVEENQHR	1	22	1	N-Term(iTRAQ8plex); Y1(iTRAQ8plex)	0.819	-	-	-	38	0	2522.36587	3	740.0551758	-2.203279356	63.94851333
														AQFEQLK	1	22	1	N-Term(iTRAQ8plex); K7(iTRAQ8plex)	0.605	0.271	0.504	-	36	0	1471.87827	3	491.2976074	3.646847527	17.34323333
														AQVEQELTTLR	1	21	1	N-Term(iTRAQ8plex)	0.726	0.39	0.339	-	35	0	1591.8897	2	796.4484863	-3.784975658	26.119375
														SYVDPSTDER	2	22	1	N-Term(iTRAQ8plex)	0.664	0.247	0.243	-	35	0	1472.71269	2	736.8599854	-2.97221372	13.84011333
														GLLSAEVAR	3	22	1	N-Term(iTRAQ8plex)	0.595	0.363	0.435	-	34	0	1219.72869	2	610.367981	-2.079155773	17.97921
														SNAEDTLR	1	21	1	N-Term(iTRAQ8plex)	0.498	0.293	0.351	-	33	0	1209.63664	2	605.3219604	-0.873833946	12.14575333
														VPDVQDGVR	1	21	1	N-Term(iTRAQ8plex)	0.599	0.214	0.277	-	30	0	1288.71135	2	644.859314	-3.841038808	15.582705
														LAAIGEATR	1	21	1	N-Term(iTRAQ8plex)	0.685	0.275	0.313	-	30	0	1205.71391	2	603.3605957	-1.373886234	28.11365
														AQAQAAAEK	1	21	1	N-Term(iTRAQ8plex); K9(iTRAQ8plex)	0.764	0.497	0.504	-	30	0	1495.87248	2	748.4398804	2.401823836	11.285005
														VPLDEALQR	1	22	1	N-Term(iTRAQ8plex)	0.466	0.233	0.277	-	29	0	1344.77458	2	672.8909302	-3.210626145	27.712785
														AAEEAEEAR	2	21	1	N-Term(iTRAQ8plex)	0.855	0.376	0.485	-	28	0	1279.6425	2	640.3238525	-2.166891428	13.75830667
														GLVEDTLR	2	21	1	N-Term(iTRAQ8plex)	0.745	0.295	0.262	-	28	0	1206.69512	2	603.8511963	-3.70047232	18.31939667
														EAIAELER	2	21	1	N-Term(iTRAQ8plex)	0.612	0.265	0.299	-	28	0	1234.69231	2	617.8497925	-1.776117862	25.39333833
														ELAEEAAR	1	21	1	N-Term(iTRAQ8plex)	0.618	0.363	0.41	-	28	0	1192.64446	2	596.8258667	-2.594672322	16.32301333
														QLAEEDLAQQR	1	21	1	N-Term(iTRAQ8plex)	0.599	0.291	0.367	-	28	0	1604.84745	3	535.6206665	-4.45216042	16.32958833
														AVVQLKPR	1	22	1	N-Term(iTRAQ8plex); K6(iTRAQ8plex)	0.693	0.424	0.431	-	28	0	1518.997	2	760.0021362	1.951643713	23.121105
														SMVEEGTGLR	1	22	1	N-Term(iTRAQ8plex)	0.535	0.346	0.281	-	27	0	1382.71977	2	691.8635254	-3.896045208	16.00029333
														TISLVIR	3	22	1	N-Term(iTRAQ8plex)	0.559	0.324	0.329	-	27	0	1105.72112	2	553.3641968	-3.205478541	30.14047667
														QVAEEAAR	1	21	1	N-Term(iTRAQ8plex)	0.53	0.198	0.307	-	27	0	1177.64665	2	589.3269653	-1.04212578	11.68273667
														SDEGQLSPATR	1	22	1	N-Term(iTRAQ8plex)	0.662	0.283	0.304	-	27	0	1464.7592	2	732.8832397	-0.285663208	12.63467
														AQLMADFQAGR	1	22	1	N-Term(iTRAQ8plex); M4(Oxidation)	0.489	0.152	0.176	-	26	0	1527.79619	2	756.401001	0.313556541	48.50101667
														SAEAELQSK	1	21	1	N-Term(iTRAQ8plex); K9(iTRAQ8plex)	0.731	0.486	0.372	-	26	0	1570.89511	3	524.3032227	3.458804282	13.38190833
														QVEEAER	1	21	1	N-Term(iTRAQ8plex)	0.573	0.252	0.266	-	22	0	1164.61443	2	582.8108521	-1.557441154	9.919848333
														LSFSGLR	1	22	1	N-Term(iTRAQ8plex)	0.628	0.275	0.348	-	22	0	1083.64324	2	542.3252563	-2.930138427	28.02910833
														LCFEGLR	2	22	1	N-Term(iTRAQ8plex); C2(Methylthio)	0.586	0.272	0.295	-	22	0	1187.61797	2	594.3126221	-3.279488423	49.60778333
														LLLEAQAATGFLLDPVK	1	22	1	N-Term(iTRAQ8plex); K17(iTRAQ8plex)	-	-	-	-	21	0	2407.44065	3	803.1517334	-0.616569588	62.95066167
														EYGSCSHHYQQLLQSLEQGAQEESR	1	22	1	N-Term(iTRAQ8plex); Y2(iTRAQ8plex); C5(Methylthio); Y9(iTRAQ8plex)	-	-	-	-	21	0	3865.8832	4	967.2262573	-4.372321214	41.90532667
														QLEMSAEAER	1	21	1	N-Term(iTRAQ8plex); M4(Oxidation)	0.729	0.336	0.309	-	20	0	1483.73931	3	489.9200134	2.688295238	22.81348333
														GYFSEEMNR	1	22	1	N-Term(iTRAQ8plex)	0.769	0.658	0.474	-	19	0	1436.67388	2	718.8405762	-3.003866307	21.90841
														LLEAQIATGGVIDPVHSHR	16	26	2	N-Term(iTRAQ8plex)	-	-	-	7.69	79	0	2317.28611	3	773.1002197	-2.997200171	34.71904833
														LLEAQIATGGIIDPVHSHR	18	25	2	N-Term(iTRAQ8plex)	-	-	-	7.69	85	0	2331.30246	3	777.7723389	-2.675735361	36.190225
														GFFDPNTHENLTYLQLLER	17	26	2	N-Term(iTRAQ8plex)	-	-	-	5.53	53	0	2611.34983	3	871.1209717	0.957547105	48.89956833
														VLADPSDDTK	2	26	2	N-Term(iTRAQ8plex); K10(iTRAQ8plex)	-	-	-	3.69	47	0	1668.92375	2	834.9655151	-1.628382697	15.67851
														GYFDEEMNR	2	26	2	N-Term(iTRAQ8plex)	-	-	-	3.17	22	0	1464.66948	2	732.8383789	-2.478448241	18.047615
														IEEEIR	1	22	3	N-Term(iTRAQ8plex)	-	-	-	-	28	0	1092.61565	2	546.8114624	-4.222084244	20.37413667
														LTVEEAVR	6	48	2	N-Term(iTRAQ8plex)	-	-	-	4.02	50	0	1220.72028	3	407.5782776	4.135438525	26.11625667
41327712	CRK	adapter molecule crk isoform a	NP_058431.2	8.55	2	2	6	304	33.8	5.55	1.306	2.229	3.275																
														IGDQEFDSLPALLEFYK	4	2	1	N-Term(iTRAQ8plex); K17(iTRAQ8plex)	1.225	1.514	2.578	4.97	35	0	2593.4	3	865.1381836	-0.402471177	58.40824667
														GHFPFTHVR	2	1	1	N-Term(iTRAQ8plex)	1.392	3.281	4.161	3.9	33	0	1401.76369	4	351.1963806	-4.015455024	17.23227
41327730	CNN2	calponin-2 isoform b	NP_958434.1	4.81	1	1	2	270	29.5	6.99	0.590	0.323	0.897																
														CASQVGMTAPGTR	2	4	1	N-Term(iTRAQ8plex); C1(Methylthio)	0.59	0.323	0.897	4.4	48	0	1628.78862	2	814.8979492	1.565284449	25.81201833
41327771	DDX23	probable ATP-dependent RNA helicase DDX23	NP_004809.2	5.73	4	4	4	820	95.5	9.55	1.031	0.967	0.814																
														GFIAGIDLK	1	1	1	N-Term(iTRAQ8plex); K9(iTRAQ8plex)	1.073	0.894	0.828	3.9	-	0	1541.9401	3	514.6515503	-7.181648049	52.06934333
														TVAVIGGISR	1	1	1	N-Term(iTRAQ8plex)	2.345	1.047	0.801	3.57	-	0	1276.79216	2	638.8997192	2.420563546	27.64431667
														IDRIEESDQGPYAIILAPTR	1	1	1	N-Term(iTRAQ8plex)	0.88	1.683	1.091	3.38	-	1	2561.38877	3	854.4677734	0.396567094	39.6353
														LIDVLENR	1	1	1	N-Term(iTRAQ8plex)	0.991	0.874	0.759	-	40	0	1275.74907	2	638.3781738	-6.553219547	32.84280167
41327773	DDX46	probable ATP-dependent RNA helicase DDX46 isoform 2	NP_055644.2	3.20	3	3	5	1031	117.3	9.29	0.731	1.137	1.110																

														GYAYTFITEDQAR	2	2	1	N-Term(iTRAQ8plex)	0.731	1.137	1.11	4.63	46	0	1838.92497	2	919.9661255	1.263268809	33.02952167
														TIAFLLPMFR	2	3	1	N-Term(iTRAQ8plex)	0.737	1.734	1.615	3.01	28	0	1512.88774	2	756.9475098	-2.44458901	63.95683333
														LLVATSVAAR	1	2	1	N-Term(iTRAQ8plex)	0.58	0.886	0.974	2.76	-	0	1304.82329	2	652.9152832	2.236690802	31.057255
41327776	DDX47	probable ATP-dependent RNA helicase DDX47 isoform 2	NP_957518.1	2.71	1	1	2	406	45.1	9.25	1.503	1.184	1.004																
														SILLATDVASR	2	2	1	N-Term(iTRAQ8plex)	1.503	1.184	1.004	3.04	44	0	1449.85466	2	725.4309692	-2.22052839	32.37507333
41349456	PREP	prolyl endopeptidase	NP_002717.3	10.28	4	4	7	710	80.6	5.86	0.872	2.263	1.408																
														ICDPYAWLEDPDSEQTK	3	3	1	N-Term(iTRAQ8plex); C2(Methylthio); K17(iTRAQ8plex)	0.855	2.307	1.789	5	64	0	2664.29294	3	888.769165	5.524755539	54.45909167
														LIDNFEGEYDYVTNEGTVFTFK	2	3	1	N-Term(iTRAQ8plex); K22(iTRAQ8plex)	0.872	2.263	0.879	3.75	39	0	3209.61948	3	1070.544678	1.722526038	58.61420833
														QNCFDDFQCAAEYLIK	1	1	1	N-Term(iTRAQ8plex); C3(Methylthio); C9(Methylthio); K16(iTRAQ8plex)	-	-	-	-	27	0	2608.20847	3	870.0743408	-3.0726413	56.47997667
														LWYCDLQQESSGIAGILK	1	3	1	N-Term(iTRAQ8plex); C4(Methylthio); K18(iTRAQ8plex)	-	-	-	-	23	0	2678.41184	3	893.4754639	-0.90499467	52.10149833
41393545	RAB5C	ras-related protein Rab-5C isoform a	NP_004574.2	37.50	4	6	31	216	23.5	8.41	1.375	0.994	0.929																
														TAMNVNEIFMAIAK	5	2	1	N-Term(iTRAQ8plex); K14(iTRAQ8plex)	1.868	1.155	0.962	5.28	67	0	2161.18064	2	1081.096069	-5.621568214	44.45975167
														GVDLQENNPASR	4	2	1	N-Term(iTRAQ8plex)	1.332	0.905	1.001	4.32	42	0	1603.8303	3	535.2816162	-2.429133057	17.33244167
														GAQAAIVVYDITNTDTFAR	2	2	1	N-Term(iTRAQ8plex)	-	0.316	0.525	4.14	44	0	2330.22397	3	777.4128418	-2.34705309	51.11766
														TAMNVNEIFMAIAK	2	2	1	N-Term(iTRAQ8plex); M3(Oxidation); K14(iTRAQ8plex)	0.89	0.688	0.957	3.91	30	0	2177.19138	2	1081.096069	-5.621568214	44.45975167
														QASPNIVIALAGNK	1	2	1	N-Term(iTRAQ8plex); K14(iTRAQ8plex)	3.366	1.856	0.902	3.8	-	0	2004.21538	2	1002.611328	4.564544356	33.62829167
														LVLLGESAVGK	8	6	3	N-Term(iTRAQ8plex); K11(iTRAQ8plex)	-	-	-	4.74	53	0	1694.05156	2	847.5296631	-8.985407579	34.19193833
														FEIWDTAGQER	9	6	3	N-Term(iTRAQ8plex)	-	-	-	3.48	38	0	1655.82427	2	828.4157715	-5.353799573	28.77746167
41393561	LAP3	cytosol aminopeptidase	NP_056991.2	3.85	2	2	8	519	56.1	7.93	0.604	1.287	1.045																
														TIQVDNTDAEGR	6	1	1	N-Term(iTRAQ8plex)	0.659	1.118	1.034	4.2	58	0	1622.82903	2	811.9161987	-2.249446977	16.357245
														TLIEFLLR	2	1	1	N-Term(iTRAQ8plex)	0.554	1.748	1.147	3.17	24	0	1308.82378	2	654.9155273	3.420450923	53.64570333
41406084	GPX1	glutathione peroxidase 1 isoform 1	NP_000572.2	14.29	2	2	5	203	22.1	6.55	0.550	0.379	0.324																
														YVRPGGGFEPNFMLFEK	1	1	1	N-Term(iTRAQ8plex); K17(iTRAQ8plex)	0.291	0.26	0.341	3.56	-	0	2596.3837	3	866.1327515	-0.23115053	43.239015
														FLVGPDGVPLRR	4	1	1	N-Term(iTRAQ8plex)	0.616	0.4	0.307	3.1	33	1	1629.97428	3	543.9962769	0.003691105	28.18567333
41872557	NRP2	neuropilin-2 isoform 6 precursor	NP_957716.1	2.88	1	1	1	555	62.4	6.48	1.119	0.598	0.944																
														IANEQISASSTYSDGR	1	8	1	N-Term(iTRAQ8plex)	1.119	0.598	0.944	3.09	-	0	2003.01311	2	1002.010193	7.367811085	25.02165333
41872631	FASN	fatty acid synthase	NP_004095.4	32.22	56	56	290	2511	273.3	6.44	0.701	3.248	1.485																
														TLLEGSGLESIISIIHSSLAEPR	20	1	1	N-Term(iTRAQ8plex)	0.952	3.563	1.606	6.85	69	0	2726.52573	3	909.5134277	0.542321354	68.70796833
														TGGAYGEDLGADYNLSQVCDGK	4	1	1	N-Term(iTRAQ8plex); C19(Methylthio); K22(iTRAQ8plex)	0.326	6.213	2.059	6.16	100	0	2887.38017	2	1444.193726	3.557637462	40.36663167
														GVDLVLNSLAEEK	11	1	1	N-Term(iTRAQ8plex); K13(iTRAQ8plex)	0.804	2.512	1.213	6	57	0	1995.15471	2	998.0809937	-1.789643326	43.38237333
														WTSQDSLLGMEFSGR	4	1	1	N-Term(iTRAQ8plex)	0.59	4.406	1.767	5.99	68	0	2017.99736	2	1009.502319	0.902256816	46.45256167
														AAPLDSIHSLAAYYIDCIR	16	1	1	N-Term(iTRAQ8plex); C17(Methylthio)	0.589	5.136	2.017	5.82	49	0	2442.24295	3	814.7525024	-1.387341818	63.016515
														EAHLPPGAMAAVGLSWEECK	2	1	1	N-Term(iTRAQ8plex); C19(Methylthio); K20(iTRAQ8plex)	-	2.987	1.514	5.81	29	0	2750.38442	4	688.3515625	-2.945835477	45.60126667
														HSQDLAFLSMLNDIAAVPATAMPFR	9	1	1	N-Term(iTRAQ8plex)	-	6.256	2.074	5.58	70	0	3020.56157	2	1510.784424	-0.824437341	66.39565667
														AALQEELQLCK	4	1	1	N-Term(iTRAQ8plex); C10(Methylthio); K11(iTRAQ8plex)	0.442	4.851	1.823	5.45	65	0	1900.04228	2	950.5247803	-3.667483184	65.78993833
														DLVEAVAHILGIR	27	1	1	N-Term(iTRAQ8plex)	0.551	4.73	1.905	5.38	69	0	1710.02103	3	570.6785278	-0.339793427	68.36058167
														LQLNGNLQLELAQVLAQERPK	2	1	1	N-Term(iTRAQ8plex); K21(iTRAQ8plex)	-	-	-	5.34	43	0	2983.75356	4	746.6938477	0.740491785	53.749895
														LPEDPLLSGLLDSPALK	2	1	1	N-Term(iTRAQ8plex); K17(iTRAQ8plex)	1.669	4.074	4.138	5.22	59	0	2386.40091	3	796.1384888	-1.884947907	58.51426
														RPTPQDSPIFLPVDDTSFR	3	1	1	N-Term(iTRAQ8plex)	1.431	0.859	0.887	5.05	38	0	2492.30655	3	831.4403687	-0.890176598	38.22236333
														DNLEFFLAGIGR	7	1	1	N-Term(iTRAQ8plex)	0.647	4.343	1.787	5.02	54	0	1655.89934	2	828.4533081	-3.968664583	43.48857
														RVYATILNAGTNTDGFK	2	1	1	N-Term(iTRAQ8plex); K17(iTRAQ8plex)	0.816	1.499	1.13	4.99	24	1	2449.34781	3	817.1207886	-7.417822512	33.74745167
														LHLSGIDANPNALFPPVEFPAPR	13	1	1	N-Term(iTRAQ8plex)	0.503	3.79	2.103	4.96	84	0	2776.51041	2	1388.758423	0.244113376	50.43753833
														HFLLEEDKPEEPTAHAFVSTLTR	4	1	1	N-Term(iTRAQ8plex); K8(iTRAQ8plex)	-	6.365	2.923	4.91	25	0	3275.77512	3	1092.596558	7.014615083	35.84386167
														CTVFHGAQVEDAFR	19	1	1	N-Term(iTRAQ8plex); C1(Methylthio)	0.871	2.711	1.713	4.89	58	0	1929.91825	2	965.4612427	-5.250784279	34.15564
														LFDHPESPTPNPTEPLFLAQAEVYK	4	1	1	N-Term(iTRAQ8plex); K25(iTRAQ8plex)	-	-	-	4.88	28	0	3448.81972	4	862.9597778	-2.229706954	46.23965167
														CVLLSNLSSTSHVPEVDPGSAELQK	2	1	1	N-Term(iTRAQ8plex); C1(Methylthio); K25(iTRAQ8plex)	-	-	-	4.84	28	0	3264.68886	4	816.9276733	-5.405973237	49.967845
														DTVTISGPQAPVFEFVEQLRK	3	1	1	N-Term(iTRAQ8plex); K21(iTRAQ8plex)	-	4.307	1.441	4.82	32	1	2969.65299	3	990.5558472	-0.92032507	54.91634833
														EDGLAQQQTQLNLR	4	1	1	N-Term(iTRAQ8plex)	0.618	1.402	1.056	4.76	64	0	1918.02959	2	959.5184326	-0.006613884	26.64134
														VLQGDLVMNVYR	2	1	1	N-Term(iTRAQ8plex)	0.51	4.444	1.756	4.72	46	0	1710.96306	2	855.9851685	6.773374792	37.771545
														VSVHVIEGDHR	5	1	1	N-Term(iTRAQ8plex)	0.806	1.402	1.311	4.64	39	0	1551.84658	4	388.7171021	-5.118403446	19.716525
														HSQDLAFLSMLNDIAAVPATAMPFR	8	1	1	N-Term(iTRAQ8plex); M22(Oxidation)	-	-	-	4.5	48	0	3036.57364	2	1510.784424	-0.824437341	66.39565667
														IPGLLSPHPLLQLSYTATDR	12	1	1	N-Term(iTRAQ8plex)	0.84	2.933	1.187	4.48	39	0	2496.4061	3	832.8068848	-2.691971119	49.73043833
														VLFPATGYLSIVWK	5	1	1	N-Term(iTRAQ8plex); K14(iTRAQ8plex)	0.699	4.301	2.068	4.42	43	0	2202.31499	2	1101.661133	0.126096858	57.43769833
														TGTVSLEVR	4	1	1	N-Term(iTRAQ8plex)	0.485	4.059	1.485	4.34	38	0	1265.73658	3	422.5837097	-0.091171901	24.138175
														LPESENLQEFWDNLIGGVDMVTDDDRR	2	1	1	N-Term(iTRAQ8plex)	-	-	-	4.33	35	1	3467.67739	4	867.6748047	2.358226872	68.88486333
														SFYGSTLFLCR	4	1	1	N-Term(iTRAQ8plex); C10(Methylthio)	0.882	3.647	1.41	4.32	38	0	1643.81572	2	822.411499	-4.271082843	41.34664667
														AINCATSGVVGLVNCLR	2	1	1	N-Term(iTRAQ8plex); C4(Methylthio); C15(Methylthio)	-	-	-	4.3	63	0	2086.05594	3	696.0234985	-1.160536725	66.10627667
														FDASFFGVHPK	9	1	1	N-Term(iTRAQ8plex); K11(iTRAQ8plex)	0.773	3.963	1.771	4.28	29	0	1860.00803	4	465.7604675	-3.452147113	32.47041333
														GGPEVQQVPAGERPLWFICSGMGTQWR	3	1	1	N-Term(iTRAQ8plex); C19(Methylthio)	-	-	-	4.26	26	0	3336.64785	4	834.9172363	2.635319887	54.755035
														GYDYGPHFQGILEASLEGDSGR	8	1	1	N-Term(iTRAQ8plex)	0.851	1.862	1.405	4.2	37	0	2672.28897	3	891.4343872	-0.326659667	43.49134667
														SLLVNPEGPTLMR	5	1	1	N-Term(iTRAQ8plex)	0.636	1.879	1.39	4.1	46	0	1730.97368	2	865.9904785	-2.32290577	35.624545
														GLKPSCTIIPLMK	1	1	1	N-Term(iTRAQ8plex); K3(iTRAQ8plex); C6(Methylthio); K13(iTRAQ8plex)	0.515	7.185	2.148	4	-	0	2359.39414	3	787.1362305	-4.295231384	41.15206
														VTAIHIDPATHR	8	1	1	N-Term(iTRAQ8plex)	0.593	3.693	1.503	3.99	39	0	1634.92055	4	409.4855957	-4.568030836	17.31859167
														GTPLISPLIK	2	1	1	N-Term(iTRAQ8plex); K10(iTRAQ8plex)	0.776	2.847	1.212	3.91	35	0	1647.05425	2	824.0307617	-7.458456529	26.02372333
														DGLLENQTPEFFQDVCKPK	1	1	1	N-Term(iTRAQ8plex); C16(Methylthio); K17(iTRAQ8plex); K19(iTRAQ8plex)	-	-	-	3.8	-	0	3166.64638	4	792.4170532	-6.842977319	44.89632833
														FPQLDSTSFANSR	4	1	1	N-Term(iTRAQ8plex)	0.717	3.571	1.808	3.79	54	0	1773.9102	2	887.4587402	1.608057017	32.66317833
														VAAAVDLIIK	4	1	1	N-Term(iTRAQ8plex); K10(iTRAQ8plex)	-	5.314	2.579	3.63	54	0	1621.03489	3	541.0164795	-9.873984566	46.69246333
														DHRDNLEFFLAGIGR	3	1	1	N-Term(iTRAQ8plex)	1.017	2.82	1.648	3.61	33	1	2064.10869	2	1032.557983	7.654091546	41.328675
														ELNLVLSVR	6	1	1	N-Term(iTRAQ8plex)	0.611	2.739	1.122	3.6	30	0	1346.82817	2	673.9168091	-3.407830539	38.792455
														YHGNVMLLR	2	1	1	N-Term(iTRAQ8plex)	0.778	3.849	1.561	3.39	36	0	1406.79654	3	469.6036987	6.064587981	22.76112
														LQVVDQPLPVR	2	1	1	N-Term(iTRAQ8plex)	0.53	2.954	1.277	3.32	35	0	1567.94097	2	784.4741211	-4.085442399	34.670295
														QELSFAAR	1	1	1	N-Term(iTRAQ8plex)	0.522	2.865	1.402	3.3	-	0	1225.68413	2	613.3457031	-0.10742572	25.291265
														MVVPGLDGAQIPR	2	1	1	N-Term(iTRAQ8plex)	0.593	2.514	1.354	3.26	27	0	1656.9373	2	828.97229	-2.189486511	36.65435167
														VLEALLPLK	2	1	1	N-Term(iTRAQ8plex); K9(iTRAQ8plex)	0.378	3.272	1.485	3.23	34	0	1604.06211	3	535.3588867	0.863810849	74.848865
														LYTLQDK	2	1	1	N-Term(iTRAQ8plex); K7(iTRAQ8plex)	0.929	3.005	1.532	3.13	27	0	1488.877	2	744.9421387	-7.52283516	22.36548833
														LPESENLQEFWDNLIGGVDMVTDDDRR	2	1	1	N-Term(iTRAQ8plex); M20(Oxidation)	-	-	-	3.09	21	1	3483.68447	4	867.6748047	2.358226872	68.88486333
														AQVADVVVSR	1	1	1	N-Term(iTRAQ8plex)	0.652	1.612	0.805	3.06	-	0	1347.78227	2	674.3947754	-5.584832873	23.99994667
														QAHTMDPQLR	2	1	1	N-Term(iTRAQ8plex)	0.2	1.228	0.662	2.97	47	0	1500.78449	3	500.9330139	-3.326794747	12.374605
														YSGTLNLDR	2	1	1	N-Term(iTRAQ8plex)	0.838	2.963	1.379	2.89	30	0	1342.71855	2	671.862915	-6.172434166	29.84776333
														VYQWDDPDPR	2	1	1	N-Term(iTRAQ8plex)	0.907	2.154	1.095	2.84	25	0	1594.77349	2	797.8903809	-4.305439809	20.87299333
														LSIPTYGLQCTR	2	1	1	N-Term(iTRAQ8plex); C10(Methylthio)	0.551	2.605	1.082	2.82	36	0	1701.89751	2	851.4523926	0.3150814	42.66679167
														LSFFFDFR	1	1	1	N-Term(iTRAQ8plex)	0.525	3.246	1.487	2.76	-	0	1382.73052	2	691.8688965	-7.618947529	81.382485
														AINCATSGVVGLVNCLRR	1	1	1	N-Term(iTRAQ8plex); C4(Methylthio); C15(Methylthio)	0.675	4.136	1.645	2.73	-	1	2242.16452	3	748.0596924	2.248094814	63.77086
														HGLYLPTR	1	1	1	N-Term(iTRAQ8plex)	0.49	1.076	0.712	-	32	0	1260.73917	3	420.9179077	2.029440024	17.92694333
														DPSQQELPR	1	1	1	N-Term(iTRAQ8plex)	0.869	3.236	1.372	-	29	0	1373.72795	2	687.3676147	-3.434761448	15.63297167
														SLLVNPEGPTLMR	1	1	1	N-Term(iTRAQ8plex); M12(Oxidation)	0.413	2.448	1.06	-	29	0	1746.96697	2	865.9904785	-2.32290577	35.624545
														YHGNVMLLR	1	1	1	N-Term(iTRAQ8plex); Y1(iTRAQ8plex)	-	-	-	-	28	0	1710.99148	3	469.6036987	6.064587981	22.76112
														QAHTMDPQLR	2	1	1	N-Term(iTRAQ8plex); M5(Oxidation)	0.64	1.067	0.814	-	28	0	1516.77591	3	500.9330139	-3.326794747	12.374605
42475534	CLSTN3	calsyntenin-3 precursor	NP_055533.2	0.73	1	1	1	956	106.0	5.44	1.215	1.591	1.269																
														AAVTEGK	1	2	1	N-Term(iTRAQ8plex); K7(iTRAQ8plex)	1.215	1.591	1.269	2.76	-	0	1283.77851	3	428.5976868	0.440693415	13.278925
42491362	IKBIP	inhibitor of nuclear factor kappa-B kinase-interacting protein isoform 2	NP_963906.1	6.86	1	2	4	350	39.3	9.17	0.341	0.362	0.835																
														NIGDLLSSSIDR	2	1	1	N-Term(iTRAQ8plex)	0.341	0.362	0.835	3.9	36	0	1593.87627	2	797.4417725	0.801192602	37.56920167
														LETNEFQQLQSK	2	2	2	N-Term(iTRAQ8plex); K12(iTRAQ8plex)	-	-	-	4.29	34	0	2073.14133	3	691.7186279	-1.129088935	40.45933167
42516576	GLRX5	glutaredoxin-related protein 5, mitochondrial precursor	NP_057501.2	8.92	1	1	2	157	16.6	6.79	1.326	1.638	1.143																
														DYAAYNVLDDPELR	2	1	1	N-Term(iTRAQ8plex)	1.326	1.638	1.143	4.68	62	0	1957.97734	2	979.4923096	-1.822686758	36.43730667
42542392	CEBPZ	CCAAT/enhancer-binding protein zeta	NP_005751.2	5.60	3	3	6	1054	120.9	5.94	0.969	0.782	1.180																
														RPWRPEEAVEDPDEEDEDNTSEAENGFSLEEVLR	2	1	1	N-Term(iTRAQ8plex)	-	-	-	4.32	13	0	4292.9562	4	1073.994507	-1.980881206	40.96200167
														ASHLLETLLCK	2	1	1	N-Term(iTRAQ8plex); C10(Methylthio); K11(iTRAQ8plex)	1.649	1.216	1.287	4.12	41	0	1882.05979	3	628.0247803	-8.115964109	50.51381333
														EESQIPVDEVFFHR	2	1	1	N-Term(iTRAQ8plex)	0.57	0.503	1.082	3.78	40	0	2036.0372	3	679.3505859	-0.936494429	32.67917333
42558283	GJB2	gap junction beta-2 protein	NP_003995.2	15.49	2	2	4	226	26.2	8.88	0.510	0.337	0.552																
														EVWGDEQADFVCNTLQPGCK	2	1	1	N-Term(iTRAQ8plex); C12(Methylthio); C19(Methylthio); K20(iTRAQ8plex)	0.436	0.298	0.329	5.9	58	0	2939.3721	3	980.4622192	2.177732052	48.86072667
														MDWGTLQTILGGVNK	2	1	1	N-Term(iTRAQ8plex); K15(iTRAQ8plex)	0.598	0.38	0.926	4.06	26	0	2241.24314	3	747.7525635	-4.048699653	75.65966333
42734430	PTRF	polymerase I and transcript release factor	NP_036364.2	31.54	10	10	46	390	43.4	5.60	0.728	0.264	0.469																
														ATEMVEVGADDDEGGAER	2	1	1	N-Term(iTRAQ8plex)	0.776	0.545	0.89	6.84	46	0	2154.98929	3	719.0012817	6.000338328	26.47569167
														IIGAVDQIQLTQAQLEER	2	2	1	N-Term(iTRAQ8plex)	1.366	0.415	0.533	5.87	74	0	2329.30075	3	777.1051025	-0.94317267	53.156115
														QAEMEGAVQSIQGELSK	2	2	1	N-Term(iTRAQ8plex); M4(Oxidation); K17(iTRAQ8plex)	0.634	0.169	0.397	5.13	52	0	2429.27982	3	810.4314575	-0.192234506	30.22756333
														SDQVNGVLVLSLLDK	4	2	1	N-Term(iTRAQ8plex); K15(iTRAQ8plex)	0.543	0.234	0.525	5.09	48	0	2208.31253	3	736.7756958	2.953772278	47.88055167
														KLEVNEAELLR	5	2	1	N-Term(iTRAQ8plex); K1(iTRAQ8plex)	0.567	0.194	0.517	4.65	65	1	1922.1542	4	481.2940063	0.553655179	35.26474167
														VPPFTFHVK	9	1	1	N-Term(iTRAQ8plex); K9(iTRAQ8plex)	0.671	0.255	0.4	4.31	35	0	1680.00913	3	560.6745605	-0.126731082	29.19021667
														SFTPDHVVYAR	12	1	1	N-Term(iTRAQ8plex)	0.896	0.429	0.519	4.23	52	0	1595.84159	3	532.6187134	-4.245041198	21.03700667
														VMIYQDEVK	2	1	1	N-Term(iTRAQ8plex); K9(iTRAQ8plex)	0.81	0.236	0.357	3.68	36	0	1732.97202	3	578.3288574	-2.522997675	36.15149
														QAEMEGAVQSIQGELSK	1	2	1	N-Term(iTRAQ8plex); K17(iTRAQ8plex)	0.908	0.692	0.657	3.47	-	0	2413.28684	3	810.4314575	-0.192234506	30.22756333
														EGQVEVLK	4	1	1	N-Term(iTRAQ8plex); K8(iTRAQ8plex)	0.734	0.236	0.384	3.38	36	0	1509.91008	2	755.4586792	0.258610392	27.74559
														ATEMVEVGADDDEGGAERGEAGDLR	2	1	1	N-Term(iTRAQ8plex)	0.806	0.227	0.174	3.36	30	1	2853.3129	3	951.7758179	0.60948916	25.89454667
														ATEMVEVGADDDEGGAER	1	1	1	N-Term(iTRAQ8plex); M4(Oxidation)	0.566	-	0.395	-	18	0	2170.96782	3	719.0012817	6.000338328	26.47569167

42734503	CAPRIN1	caprin-1 isoform 2	NP_976240.1	1.59	1	1	2	694	76.8	5.12	0.485	1.036	1.161																
														SFMALSQDIQK	2	2	1	N-Term(iTRAQ8plex); K11(iTRAQ8plex)	0.485	1.036	1.161	3.74	37	0	1876.02848	3	626.0143433	-9.277452374	52.800685
42794771	TXNDC5	thioredoxin domain-containing protein 5 isoform 1 precursor	NP_110437.2	25.23	8	8	36	432	47.6	5.97	1.031	0.640	0.870																
														ALAPTWEQLALGLEHSETVK	7	2	1	N-Term(iTRAQ8plex); K20(iTRAQ8plex)	1.031	0.869	0.555	5.22	30	0	2801.57705	3	934.5252075	-1.699763556	50.704805
														LQPTWNDLGDKYNSMEDAK	1	1	1	N-Term(iTRAQ8plex); K11(iTRAQ8plex); K19(iTRAQ8plex)	3.146	-	1.058	4.48	-	1	3137.61928	4	785.1602783	-4.722656706	34.06150667
														FVLSQAKDEL	2	2	1	N-Term(iTRAQ8plex); K7(iTRAQ8plex)	1.102	0.659	0.892	4.18	27	1	1758.02121	3	586.6785889	-2.604034811	57.59558833
														VDCTAHSDVCSAQGVR	7	2	1	N-Term(iTRAQ8plex); C3(Methylthio); C10(Methylthio)	1.08	0.607	0.948	4	68	0	2043.89958	2	1022.45343	-1.301222652	25.06183
														VDCTQHYELCSGNQVR	7	2	1	N-Term(iTRAQ8plex); C3(Methylthio); C10(Methylthio)	0.986	0.79	0.763	3.66	21	0	2247.98691	3	750.0011597	-1.413506115	29.50872333
														IAEVDCTAER	2	2	1	N-Term(iTRAQ8plex); C6(Methylthio)	0.928	0.458	0.858	3.11	37	0	1456.70488	2	728.8560791	-1.998151384	28.023085
														GYPTLLWFR	8	2	1	N-Term(iTRAQ8plex)	0.992	0.544	0.837	3.09	38	0	1456.82012	2	728.9136963	-3.655456028	39.55420833
														GYPTLLLFR	2	2	1	N-Term(iTRAQ8plex)	1.086	0.584	0.87	3.04	-	0	1383.82659	2	692.4169312	-2.605539849	45.131125
44680114	PSMG1	proteasome assembly chaperone 1 isoform b	NP_982257.1	5.24	1	1	2	267	30.3	7.77	2.333	4.936	6.961																
														AGTEDEEEEEEGRR	2	3	1	N-Term(iTRAQ8plex)	2.333	4.936	6.961	2.92	33	1	1939.87662	3	647.2970581	-0.881619777	9.907433333
44889481	MYO1B	unconventional myosin-Ib isoform 2	NP_036355.2	25.60	23	23	111	1078	124.9	9.20	1.070	0.692	0.424																
														AAHIFNEALVCHQIR	12	5	1	N-Term(iTRAQ8plex); C11(Methylthio)	0.732	0.567	0.658	5.28	62	0	2072.0784	3	691.3643188	-2.494600119	37.052365
														EICELTGIDQSVLER	4	5	1	N-Term(iTRAQ8plex); C3(Methylthio)	0.895	0.588	0.481	5.23	82	0	2055.03496	2	1028.021118	-2.667882534	51.32376667
														IFLLTNNNLLLADQK	6	5	1	N-Term(iTRAQ8plex); K15(iTRAQ8plex)	1.075	1.337	0.48	5.06	47	0	2338.39322	3	780.1359253	-0.970183745	47.45767667
														NFYELSPHIFALSDEAYR	12	4	1	N-Term(iTRAQ8plex)	1.222	0.898	0.606	5.04	61	0	2476.24289	3	826.0858154	-0.879663681	47.045775
														IIIAEVVNK	12	5	1	N-Term(iTRAQ8plex); K9(iTRAQ8plex)	1.17	0.709	0.381	4.67	55	0	1607.03336	2	804.0222168	1.198275809	56.17144167
														NFHVFYQLLSGASEELLNKLK	2	5	1	N-Term(iTRAQ8plex); K19(iTRAQ8plex); K21(iTRAQ8plex)	-	-	-	4.66	48	1	3362.91761	5	673.3893433	-1.897608609	68.99724667
														SSALVIQSYIR	8	5	1	N-Term(iTRAQ8plex)	1.121	0.669	0.499	4.61	48	0	1540.91233	3	514.30896	7.959786291	33.87169333
														EQLLQSNPVLEAFGNAK	2	7	1	N-Term(iTRAQ8plex); K17(iTRAQ8plex)	1.245	1.385	1.306	4.57	34	0	2466.37204	3	822.7955322	-3.757693547	42.65432167
														VSTTLNVAQAYYAR	6	5	1	N-Term(iTRAQ8plex)	0.949	0.739	0.566	4.5	56	0	1861.00041	2	931.0038452	-6.296339445	35.30324333
														LVMSYVAAVCGK	11	6	1	N-Term(iTRAQ8plex); C10(Methylthio); K12(iTRAQ8plex)	1.059	0.519	0.249	4.21	66	0	1895.03006	2	948.0263672	2.176968886	55.81867833
														VLYQVEGFVDKNNDLLYR	3	5	1	N-Term(iTRAQ8plex); K11(iTRAQ8plex)	0.823	0.591	0.598	4.17	-	1	2793.5602	4	699.1455078	7.377707482	57.62743
														FLNDTSLPHSCFR	12	5	1	N-Term(iTRAQ8plex); C11(Methylthio)	1.219	0.913	0.425	4.11	42	0	1886.91849	3	629.6431885	-2.38490885	42.48837
														YNYLSLDSAK	2	5	1	N-Term(iTRAQ8plex); K10(iTRAQ8plex)	1.045	0.668	0.433	4.06	26	0	1781.99138	2	891.4993286	1.127562448	29.06295167
														SLPIYSPEKVEEYR	2	4	1	N-Term(iTRAQ8plex); K9(iTRAQ8plex)	1.343	0.45	0.331	4.04	-	1	2318.27407	4	580.3239746	-4.82373618	31.15435167
														LEDLATLIQK	1	5	1	N-Term(iTRAQ8plex); K10(iTRAQ8plex)	0.663	0.402	0.337	4.04	-	0	1752.07358	3	584.6960449	0.479554988	74.722365
														DLSQAMWK	1	5	1	N-Term(iTRAQ8plex); K8(iTRAQ8plex)	1.139	1.248	1.057	3.72	-	0	1586.88755	3	529.6340332	3.431282442	28.29157667
														SQIVIAAWYR	1	5	1	N-Term(iTRAQ8plex)	1.07	0.936	0.341	3.66	-	0	1510.86284	2	755.9350586	-3.660644998	31.16181833
														LEASELFK	2	5	1	N-Term(iTRAQ8plex); K8(iTRAQ8plex)	1.105	0.589	0.296	3.37	47	0	1544.91374	2	772.9605103	-0.451424677	44.218555
														LKDAIEEK	1	5	1	N-Term(iTRAQ8plex); K2(iTRAQ8plex); K8(iTRAQ8plex)	1.318	0.923	0.27	3.21	-	1	1858.14641	2	929.5768433	2.765445936	20.66194667
														QTWPHWK	1	5	1	N-Term(iTRAQ8plex); K7(iTRAQ8plex)	1.059	0.805	0.499	2.95	-	0	1590.89719	3	530.9705811	-1.836313469	22.58776167
														IFHLWR	8	5	1	N-Term(iTRAQ8plex)	0.583	0.594	0.226	2.84	31	0	1175.69034	3	392.5708008	-1.073580471	33.47522833
														LFSWIVNR	2	12	1	N-Term(iTRAQ8plex)	0.832	0.473	0.327	2.75	24	0	1338.77995	2	669.8936157	-2.708790697	77.26717
														YLGLLENVR	1	7	1	N-Term(iTRAQ8plex)	1.125	0.536	0.305	-	37	0	1380.81474	2	690.9110107	-0.381250002	47.22544
44890050	STMN1	stathmin isoform a	NP_981944.1	28.86	4	4	8	149	17.3	5.97	0.923	1.778	2.889																
														ASGQAFELILSPR	2	2	1	N-Term(iTRAQ8plex)	1.007	2.035	3.627	4.36	63	0	1692.95207	2	846.9796753	-3.896940831	43.13079333
														ESVPEFPLSPPK	3	2	1	N-Term(iTRAQ8plex); K12(iTRAQ8plex)	0.885	1.726	2.396	3.75	36	0	1935.09429	2	968.0507813	-5.423725385	25.728475
														AIEENNNFSK	4	5	1	N-Term(iTRAQ8plex); K10(iTRAQ8plex)	0.801	4.262	9.63	3.64	53	0	1773.9448	3	591.9864502	-8.097187739	26.84584167
														SHEAEVLK	1	2	1	N-Term(iTRAQ8plex); K8(iTRAQ8plex)	0.962	1.688	2.566	-	32	0	1520.87418	3	507.6295776	-9.92957869	17.69969
45359859	GRB2	growth factor receptor-bound protein 2 isoform 2	NP_987102.1	6.82	1	1	2	176	20.5	5.85	1.769	2.598	2.701																
														FNSLNELVDYHR	2	2	1	N-Term(iTRAQ8plex)	1.769	2.598	2.701	4.58	33	0	1810.9332	3	604.315918	-3.18655804	38.96804
45387945	ESYT2	extended synaptotagmin-2	NP_065779.1	9.29	6	6	17	893	98.8	8.68	0.850	0.711	1.001																
														ACDLPAWVHFPDTER	2	1	1	N-Term(iTRAQ8plex); C2(Methylthio)	0.977	0.901	1.226	4.84	48	0	2106.99826	3	703.0042725	-2.880005784	48.69684833
														LLDEWFTLDEVPK	7	1	1	N-Term(iTRAQ8plex); K13(iTRAQ8plex)	0.841	0.644	0.941	4.5	74	0	2213.24906	3	738.4212036	7.954951922	57.100065
														TNEPVWEENFTFFIHNPK	3	1	1	N-Term(iTRAQ8plex); K18(iTRAQ8plex)	1.395	0.964	1.001	4.27	30	0	2857.47013	3	953.1615601	-2.405755411	50.00347333
														AQPPEAGPQGLHDLGR	2	1	1	N-Term(iTRAQ8plex)	0.335	0.269	0.37	4.09	56	0	1947.03366	3	649.6827393	-0.707114924	20.043285
														LIVVVHACR	2	1	1	N-Term(iTRAQ8plex); C8(Methylthio)	0.899	0.839	1.756	3.81	40	0	1359.7898	3	453.9347839	-0.635203588	35.293575
														IHFIEAQDLQGK	1	1	1	N-Term(iTRAQ8plex); K12(iTRAQ8plex)	0.796	0.657	0.869	3.37	-	0	2007.13419	3	669.7162476	-7.06573297	29.34273167
45387955	C19orf70	MICOS complex subunit MIC13 isoform 2 precursor	NP_991330.1	22.03	1	1	2	118	13.1	9.42	-	-	-																
														IYFPIRDSWNAGIMTVMSALSVAPSK	2	2	1	N-Term(iTRAQ8plex); K26(iTRAQ8plex)	-	-	-	4.12	24	1	3462.88254	3	1154.965698	2.500857727	67.39525833
45439306	DARS	aspartate--tRNA ligase, cytoplasmic isoform 1	NP_001340.2	26.55	11	11	38	501	57.1	6.55	1.066	1.583	1.907																
														VTMLFLGLHNVR	14	3	1	N-Term(iTRAQ8plex)	1.531	1.631	1.984	5.32	56	0	1703.98423	3	568.6673584	-3.379927736	49.75595167
														LQSGICHLFR	4	3	1	N-Term(iTRAQ8plex); C6(Methylthio)	1.143	2.01	2.354	5.19	69	0	1523.80835	3	508.6076355	-2.952507676	45.40401
														FGAPPHAGGGIGLER	4	3	1	N-Term(iTRAQ8plex)	0.73	1.242	1.442	4.08	43	0	1739.94229	3	580.6522827	-4.153244329	23.03584
														EAGVEMGDEDDLSTPNEK	2	3	1	N-Term(iTRAQ8plex); K18(iTRAQ8plex)	1.017	1.155	0.931	3.62	49	0	2544.20835	3	848.7409668	-5.854142956	19.23258833
														EIMDAAEDYAK	2	1	1	N-Term(iTRAQ8plex); K11(iTRAQ8plex)	0.897	1.278	1.249	3.44	46	0	1863.94352	3	621.9860229	-9.853143183	42.83992333
														IHDPQLLTER	4	3	1	N-Term(iTRAQ8plex)	1.294	2.119	2.512	3.31	40	0	1525.85419	3	509.2895813	-6.442721445	21.706115
														ESIVDVEGVVR	2	3	1	N-Term(iTRAQ8plex)	1.058	2.196	2.416	3.29	35	0	1505.84221	2	753.4247437	-3.659490532	32.15607833
														IYVISLAEPR	2	3	1	N-Term(iTRAQ8plex)	1.159	1.687	1.883	3.28	28	0	1464.86675	2	732.9370117	-4.126301941	44.638115
														LPLQLDDAVRPEAEGEEEGR	1	3	1	N-Term(iTRAQ8plex)	1.073	1.478	1.592	2.84	-	0	2527.28556	3	843.1000366	-3.44823983	33.47541667
														EAGVEMGDEDDLSTPNEK	1	3	1	N-Term(iTRAQ8plex); M6(Oxidation); K18(iTRAQ8plex)	0.759	0.772	0.809	-	32	0	2560.20517	3	848.7409668	-5.854142956	19.23258833
														LLGHLVK	1	3	1	N-Term(iTRAQ8plex); K7(iTRAQ8plex)	1.336	2.708	2.93	-	28	0	1387.91178	3	463.3087769	-9.196538139	35.51938833
														VFSIGPVFR	1	3	1	N-Term(iTRAQ8plex)	1.513	3.879	4.392	-	28	0	1325.77959	2	663.3934326	-6.601916111	34.89354333
45439327	PPL	periplakin	NP_002696.3	1.08	2	2	3	1756	204.6	5.58	1.337	1.851	0.385																
														SIVVIHPDTGR	2	4	1	N-Term(iTRAQ8plex)	1.039	1.353	0.844	4.08	35	0	1497.86496	3	499.9598389	-2.769240579	19.37279167
														ELDVEVSR	1	4	1	N-Term(iTRAQ8plex)	1.72	2.531	0.175	3.4	-	0	1250.69194	2	625.8496094	2.02354386	24.37086167
45504382	DNAJC5	dnaJ homolog subfamily C member 5	NP_079495.1	7.58	1	1	1	198	22.1	5.07	-	-	-																
														EINNAHAILTDATKR	1	1	1	N-Term(iTRAQ8plex); K14(iTRAQ8plex)	-	-	-	2.77	-	1	2275.28359	4	569.576355	-6.289878847	23.87362
45643127	GNL3	guanine nucleotide-binding protein-like 3 isoform 2	NP_996561.1	10.80	4	4	6	537	60.5	8.79	3.231	1.839	1.119																
														KVIEASDVVLEVLDAR	2	2	1	N-Term(iTRAQ8plex); K1(iTRAQ8plex)	1.685	0.882	1.119	4.26	66	1	2364.39676	3	788.803772	0.360078301	60.71846
														NSLEFFTVLAQR	1	2	1	N-Term(iTRAQ8plex)	3.402	2.282	0.979	4.09	-	0	1728.96086	2	864.9840698	1.273440298	45.362505
														SPASIEVVKPMEAASAILSQADAR	2	2	1	N-Term(iTRAQ8plex); K9(iTRAQ8plex)	3.337	1.935	1.959	3.06	-	0	3049.68692	3	1017.233826	1.840353099	51.88687
														EFGLCK	1	2	1	N-Term(iTRAQ8plex); C5(Methylthio); K6(iTRAQ8plex)	1.471	1.019	1.049	-	24	0	1350.7404	2	675.8738403	2.518429191	25.30037167
45827771	EDC4	enhancer of mRNA-decapping protein 4	NP_055144.3	1.00	1	1	1	1401	151.6	5.86	0.808	1.438	1.653																
														SCQAMFQQINDSFR	1	1	1	N-Term(iTRAQ8plex); C2(Methylthio)	0.808	1.438	1.653	-	20	0	2024.93254	2	1012.96991	1.531120604	50.829265
46276863	PTMS	parathymosin	NP_002815.3	22.55	2	2	3	102	11.5	4.16	0.570	1.659	3.917																
														SVEAAAELSAK	2	2	1	N-Term(iTRAQ8plex); K11(iTRAQ8plex)	0.38	2.029	6.975	4.02	43	0	1683.9578	3	561.9907837	-9.479736251	31.851145
														AAEEEDEADPKR	1	1	1	N-Term(iTRAQ8plex); K11(iTRAQ8plex)	0.856	1.357	2.2	2.88	-	1	1968.00405	4	492.7564697	-4.589560552	15.28649667
46367787	PABPC1	polyadenylate-binding protein 1	NP_002559.2	27.36	8	14	106	636	70.6	9.50	1.018	2.145	1.529																
														LFPLIQAMHPTLAGK	2	2	1	N-Term(iTRAQ8plex); K15(iTRAQ8plex)	-	-	-	4.64	32	0	2245.33364	4	562.0888672	-0.673588091	50.30777667
														GYGFVHFETQEAAER	14	1	1	N-Term(iTRAQ8plex)	1.054	1.749	1.253	4.16	38	0	2044.99509	3	682.3359375	-4.788304292	27.742085
														NLDDGIDDERLR	4	2	1	N-Term(iTRAQ8plex)	2.331	3.49	2.456	3.68	36	1	1734.88529	3	578.9666138	-4.128942389	16.56925167
														YQGVNLYVK	2	2	1	N-Term(iTRAQ8plex); K9(iTRAQ8plex)	1.161	2.739	1.987	3.67	45	0	1691.97905	2	846.4931641	-8.871147067	25.809565
														NLDDGIDDER	2	2	1	N-Term(iTRAQ8plex)	0.869	2.375	1.752	3.34	57	0	1465.70317	2	733.3552246	-2.789583407	17.76016667
														NFGEDMDDER	4	2	1	N-Term(iTRAQ8plex)	0.875	1.904	1.645	3.33	43	0	1531.65618	2	766.3317261	-4.906957372	15.40832667
														GFGFVSFER	10	2	1	N-Term(iTRAQ8plex)	0.895	2.194	1.523	3.32	38	0	1349.70696	2	675.3571167	-6.382512529	52.49694333
														FSPAGPILSIR	4	4	1	N-Term(iTRAQ8plex)	0.999	2.14	1.539	3.24	32	0	1461.8678	3	487.9607849	-3.652733624	38.59170667
														GYGFVHFETQEAAER	1	1	1	N-Term(iTRAQ8plex); Y2(iTRAQ8plex)	0.879	2.455	1.682	-	37	0	2349.1959	3	682.3359375	-4.788304292	27.742085
														ALYDTFSAFGNILSCK	40	4	2	N-Term(iTRAQ8plex); C15(Methylthio); K16(iTRAQ8plex)	-	-	-	6.61	79	0	2404.26738	2	1202.637329	7.170285326	65.123275
														SLGYAYVNFQQPADAER	6	4	2	N-Term(iTRAQ8plex)	-	-	-	5.78	90	0	2233.11455	2	1117.060913	-2.056208055	31.477775
														ALDTMNFDVIK	4	4	2	N-Term(iTRAQ8plex); K11(iTRAQ8plex)	-	-	-	4.97	71	0	1875.03594	2	938.0216064	-7.842532023	63.45430333
														ITGMLLEIDNSELLHMLESPESLR	2	4	2	N-Term(iTRAQ8plex)	-	-	-	3.75	46	0	3044.61618	3	1015.543579	6.931170766	69.39083
														AKEFTNVYIK	1	4	2	N-Term(iTRAQ8plex); K2(iTRAQ8plex); K10(iTRAQ8plex)	-	-	-	2.86	-	1	2125.2582	4	532.0700073	-9.515024165	29.22272833
														SLGYAYVNFQQPADAER	1	4	2	N-Term(iTRAQ8plex); Y4(iTRAQ8plex)	-	-	-	-	29	0	2537.31801	2	1117.060913	-2.056208055	31.477775
														GFGFVCFSSPEEATK	9	6	2	N-Term(iTRAQ8plex); C6(Methylthio); K15(iTRAQ8plex)	-	-	-	5.73	66	0	2260.11406	2	1130.560669	-4.349793465	37.29285167
46593007	UQCRC1	cytochrome b-c1 complex subunit 1, mitochondrial precursor	NP_003356.2	41.25	13	13	87	480	52.6	6.37	1.069	0.916	0.760																
														DVVFNYLHATAFQGTPLAQAVEGPSENVR	8	1	1	N-Term(iTRAQ8plex)	0.656	-	-	6.76	78	0	3434.78684	3	1145.595093	1.745027774	54.76894667
														HLGGIPWTYAEDAVPTLTPCR	14	1	1	N-Term(iTRAQ8plex); C20(Methylthio)	1.124	1.568	2.291	6.41	51	0	2647.3273	3	883.1139526	-1.581836835	52.11969333
														MVLAAAGGVEHQQLLDLAQK	2	1	1	N-Term(iTRAQ8plex); M1(Oxidation); K20(iTRAQ8plex)	-	-	-	6.1	54	0	2716.52407	4	679.8864746	-1.33175426	36.596805
														IDDMMFVLQGQWMR	4	1	1	N-Term(iTRAQ8plex)	1.017	0.561	0.757	5.76	53	0	2074.03232	3	692.015625	4.662632254	64.584545
														MVLAAAGGVEHQQLLDLAQK	6	1	1	N-Term(iTRAQ8plex); K20(iTRAQ8plex)	1.02	1.152	0.523	5.74	49	0	2700.53481	4	679.8864746	-1.33175426	36.596805
														NALVSHLDGTTPVCEDIGR	18	1	1	N-Term(iTRAQ8plex); C14(Methylthio)	0.83	0.851	0.759	5.36	49	0	2347.1669	3	783.0604858	-0.825689245	39.09592667
														YIYDQCPAVAGYGPIEQLPDYNR	8	1	1	N-Term(iTRAQ8plex); C6(Methylthio)	0.706	0.419	0.737	4.71	57	0	2995.43955	3	999.144104	-3.159338377	46.01592667
														RIPLAEWESR	9	1	1	N-Term(iTRAQ8plex)	1.874	1.412	0.956	4.39	45	1	1560.87432	3	520.9639893	-1.670071293	29.57654167
														LCTSATESEVAR	4	1	1	N-Term(iTRAQ8plex); C2(Methylthio)	1	0.593	0.48	4.12	61	0	1616.78911	2	808.8981934	-2.147590696	27.29153667
														MKIDDMMFVLQGQWMR	2	1	1	N-Term(iTRAQ8plex); K2(iTRAQ8plex)	1.311	0.706	0.761	3.79	41	1	2637.36124	3	879.7919312	-0.849748201	61.00021667
														AVELLGDIVQNCSLEDSQIEKER	5	1	1	N-Term(iTRAQ8plex); C12(Methylthio); K21(iTRAQ8plex)	2.803	1.469	0.758	3.71	39	1	3242.70216	5	649.3462524	5.04622264	68.01580667
														AVELLGDIVQNCSLEDSQIEK	2	1	1	N-Term(iTRAQ8plex); C12(Methylthio); K21(iTRAQ8plex)	-	-	-	3.57	49	0	2957.54898	3	986.5211792	2.33708645	69.99996
														IAEVDASVVR	4	1	1	N-Term(iTRAQ8plex)	1.044	0.968	0.716	2.87	30	0	1362.78984	2	681.8985596	0.272397558	29.140685
														EVESMGAHLNAYSTR	1	1	1	N-Term(iTRAQ8plex)	1.07	1.03	1.213	-	25	0	1968.97226	3	656.9956055	-1.451591677	24.44370167
46852147	IARS2	isoleucine--tRNA ligase, mitochondrial precursor	NP_060530.3	8.50	6	6	17	1012	113.7	7.20	0.741	0.654	0.757																
														CGFSELYSWQR	2	1	1	N-Term(iTRAQ8plex); C1(Methylthio)	0.734	0.656	0.558	4.59	40	0	1725.7957	2	863.4014893	-4.270728202	35.68576667

														IHFVPGWDCHGLPIEIK	3	1	1	N-Term(iTRAQ8plex); C9(Methylthio); K17(iTRAQ8plex)	0.589	0.518	1.307	4.37	-	0	2615.40053	4	654.6055908	-3.134853972	47.99198833
														KPGLEEAVESACAMR	5	1	1	N-Term(iTRAQ8plex); K1(iTRAQ8plex); C12(Methylthio)	0.74	0.691	0.736	4.12	33	0	2245.15445	3	749.0563354	-2.462726934	45.85780833
														FLINLEGGDIR	3	1	1	N-Term(iTRAQ8plex)	0.998	0.615	0.548	3.76	31	0	1550.88957	2	775.9484253	3.309497996	41.59714333
														LVEQHGSDIWWTLPPEQLLPK	2	1	1	N-Term(iTRAQ8plex); K21(iTRAQ8plex)	1.699	0.98	1.12	3.16	-	0	3094.72702	3	1032.247192	2.705440051	52.38169167
														ELSNFYFSIIK	2	1	1	N-Term(iTRAQ8plex); K11(iTRAQ8plex)	0.549	0.583	0.582	2.7	32	0	1969.12041	2	985.0638428	-2.58103725	42.38539667
47132587	PRKCDBP	protein kinase C delta-binding protein	NP_659477.2	4.21	1	1	5	261	27.7	6.43	0.322	0.214	0.141																
														LEANHGLLVAR	5	1	1	N-Term(iTRAQ8plex)	0.322	0.214	0.141	3.75	53	0	1496.88288	3	499.6324768	-1.485841085	22.48938667
47132600	DFFA	DNA fragmentation factor subunit alpha isoform 2	NP_998731.1	10.82	2	2	4	268	29.4	4.61	0.782	1.271	1.190																
														ETSSDVALASHILTALR	2	2	1	N-Term(iTRAQ8plex)	0.674	1.52	1.187	4.72	37	0	2088.16458	3	696.7263794	2.056175852	44.180635
														LQHTLQQVLDQR	2	2	1	N-Term(iTRAQ8plex)	0.908	1.062	1.192	4.14	36	0	1783.00669	3	595.0070801	-3.434824821	37.098765
47132620	KRT2	keratin, type II cytoskeletal 2 epidermal	NP_000414.2	23.47	4	11	320	639	65.4	8.00	0.713	1.057	0.849																
														GGGFGGGSSFGGGSGFSGGGFGGGGFGGGR	2	1	1	N-Term(iTRAQ8plex)	0.51	1.119	1.508	8.29	75	0	2703.2305	3	901.7483521	2.427318846	35.56198167
														GFSSGSAVVSGGSR	6	2	1	N-Term(iTRAQ8plex)	0.75	0.923	0.752	4.77	58	0	1558.82109	2	779.9141846	5.363810296	21.45600833
														VDLLNQEIEFLK	4	2	1	N-Term(iTRAQ8plex); K12(iTRAQ8plex)	0.741	1.126	1.061	4.61	45	0	2069.19101	3	690.4018555	-9.326419662	77.14794833
														FGGFGGPGGVGGLGGPGGFGPGGYPGGIHEVSVNQ[illegible]	1	2	1	N-Term(iTRAQ8plex); K44(iTRAQ8plex)	-	-	-	-	32	0	4700.48598	4	1175.876953	0.512993765	55.88697833
														LALDVEIATYR	117	10	7	N-Term(iTRAQ8plex)	-	-	-	4.83	67	0	1567.90409	3	523.3062134	2.771589637	39.12087
														LALDVEIATYR	8	10	7	N-Term(iTRAQ8plex); Y10(iTRAQ8plex)	-	-	-	-	60	0	1872.106	3	523.3062134	2.771589637	39.12087
														FASFIDKVR	16	27	8	N-Term(iTRAQ8plex); K7(iTRAQ8plex)	-	-	-	3.33	31	1	1691.01076	4	423.5080261	0.114317571	39.05999667
														FASFIDK	57	28	8	N-Term(iTRAQ8plex); K7(iTRAQ8plex)	-	-	-	3.23	35	0	1435.83818	2	718.4240723	0.227470368	33.40336167
														NLDLDSIIAEVK	64	8	5	N-Term(iTRAQ8plex); K12(iTRAQ8plex)	-	-	-	5.73	89	0	1938.13909	2	969.5731812	1.173488892	50.31108
														KLLEGEECR	13	13	6	N-Term(iTRAQ8plex); K1(iTRAQ8plex); C8(Methylthio)	-	-	-	2.79	31	1	1730.93911	3	577.6514282	0.43855829	29.74912
														LLEGEECR	16	16	6	N-Term(iTRAQ8plex); C7(Methylthio)	-	-	-	2.75	36	0	1298.63898	3	433.551178	0.260569375	30.10390833
														AQYEEIAQR	16	7	4	N-Term(iTRAQ8plex)	-	-	-	3.65	42	0	1411.75541	3	471.2566528	5.024911587	22.42886333
47174861	PPAN	suppressor of SWI4 1 homolog	NP_064615.3	10.78	4	4	8	473	53.2	10.13	1.668	0.851	0.909																
														NLEAYAANPHSFVFTR	3	3	1	N-Term(iTRAQ8plex)	1.456	0.809	1.002	5.32	45	0	2141.10147	3	714.3720093	-3.132368105	32.31068667
														CLLIDYNPDSQELDFR	2	3	1	N-Term(iTRAQ8plex); C1(Methylthio)	1.764	0.94	0.909	3.79	45	0	2291.09502	2	1146.051147	-1.743270398	53.80184167
														TEEELQAILEAK	2	3	1	N-Term(iTRAQ8plex); K12(iTRAQ8plex)	1.373	0.807	0.642	3.34	26	0	1982.11429	3	661.3762817	-6.230385001	35.91370833
														QLSLDVR	1	3	1	N-Term(iTRAQ8plex)	1.938	0.851	1.052	-	24	0	1134.67241	2	567.8398438	-5.314679323	29.69422833
47174864	EXOSC5	exosome complex component RRP46	NP_064543.3	5.96	1	1	4	235	25.2	7.59	1.814	1.310	1.253																
														NTCEAVVLGTLHPR	4	1	1	N-Term(iTRAQ8plex); C3(Methylthio)	1.814	1.31	1.253	4.43	52	0	1859.97013	3	620.6615601	-3.895425047	39.962275
47271406	MEPCE	7SK snRNA methylphosphate capping enzyme isoform A	NP_062552.2	1.45	1	1	2	689	74.3	9.57	0.946	1.108	1.132																
														AAPPDVGEER	2	1	1	N-Term(iTRAQ8plex)	0.946	1.108	1.132	2.7	26	0	1344.69988	2	672.8535767	-4.658734485	15.04456833
47419918	WARS	tryptophan--tRNA ligase, cytoplasmic isoform b	NP_998810.1	14.19	4	4	15	430	48.8	6.48	1.775	2.721	2.792																
														ALIEVLQPLIAEHQAR	10	2	1	N-Term(iTRAQ8plex)	-	2.721	2.792	5.11	60	0	2105.24576	3	702.4193115	2.322781468	47.34563833
														DMNQVLDAYENK	2	2	1	N-Term(iTRAQ8plex); K12(iTRAQ8plex)	2.986	10.093	8.635	4.77	53	0	2048.04325	3	683.3526001	-7.155247846	51.471475
														DRTDIQCLIPCAIDQDPYFR	2	2	1	N-Term(iTRAQ8plex); C7(Methylthio); C11(Methylthio)	-	-	-	3.21	33	1	2778.30539	3	926.7733154	1.011313972	52.43434167
														GIFGFTDSDCIGK	1	2	1	N-Term(iTRAQ8plex); C10(Methylthio); K13(iTRAQ8plex)	1.056	2.227	1.699	-	29	0	2014.00559	3	672.0067139	-8.864895182	68.39008833
47419936	SRPK1	SRSF protein kinase 1	NP_003128.3	5.04	2	2	4	655	74.3	6.16	0.941	1.882	1.267																
														IADLGNACWVHK	3	15	1	N-Term(iTRAQ8plex); C8(Methylthio); K12(iTRAQ8plex)	0.941	1.882	1.267	5.25	41	0	1981.05027	3	661.0216064	-5.328594758	39.312835
														IIHTDIKPENILLSVNEQYIR	1	1	1	N-Term(iTRAQ8plex); K7(iTRAQ8plex)	-	-	-	4.38	-	0	3116.79189	4	779.9534302	-0.311370444	44.98509
48255891	PRKCSH	glucosidase 2 subunit beta isoform 2 precursor	NP_001001329.1	26.10	11	11	54	525	59.1	4.42	1.090	0.739	0.708																
														VNDGVCDCCDGTDEYNSGVICENTCK	13	3	1	N-Term(iTRAQ8plex); C6(Methylthio); C8(Methylthio); C9(Methylthio); [illegible]	1.056	0.817	0.704	6.82	95	0	3594.40683	3	1198.807129	9.043820095	54.25182
														ESLQQMAEVTR	6	3	1	N-Term(iTRAQ8plex); M6(Oxidation)	1.224	0.667	0.721	5.26	52	0	1611.83805	3	537.9508667	4.113656251	20.33547333
														LWEEQLAAAK	4	3	1	N-Term(iTRAQ8plex); K10(iTRAQ8plex)	1.07	0.692	0.797	5.08	41	0	1767.02434	2	884.0158081	-1.013969656	46.47179833
														SLEDQVEMLR	8	3	1	N-Term(iTRAQ8plex)	1.071	0.738	0.704	4.62	44	0	1523.80826	3	508.607605	2.7040091	31.856565
														AQQEQELAADAFK	2	3	1	N-Term(iTRAQ8plex); K13(iTRAQ8plex)	0.816	0.654	0.686	4.15	52	0	2057.10733	3	686.3739624	-2.458473063	37.60785
														YEQGTGCWQGPNR	4	3	1	N-Term(iTRAQ8plex); C7(Methylthio)	1.191	0.868	0.864	4.04	48	0	1845.8239	2	923.4155884	-4.075232847	19.63552167
														ETMVTSTTEPSR	5	3	1	N-Term(iTRAQ8plex)	0.808	0.671	0.639	4.03	46	0	1642.82353	2	821.9154053	-1.495822938	17.64814833
														ESLQQMAEVTR	3	3	1	N-Term(iTRAQ8plex)	1.004	1.17	0.843	3.88	41	0	1595.83122	3	537.9508667	4.113656251	20.33547333
														LIELQAGK	1	3	1	N-Term(iTRAQ8plex); K8(iTRAQ8plex)	0.92	0.977	0.993	3.6	-	0	1479.93811	3	493.9842224	1.758645381	35.74787833
														LGGSPTSLGTWGSWIGPDHDKFSAMK	1	3	1	N-Term(iTRAQ8plex); K21(iTRAQ8plex); K26(iTRAQ8plex)	1.167	0.842	0.631	3.4	-	1	3644.91465	3	1215.643066	-4.193604716	43.470775
														ILIEDWK	3	3	1	N-Term(iTRAQ8plex); K7(iTRAQ8plex)	1.444	0.781	0.63	3.14	31	0	1524.92548	3	508.980011	0.569476342	57.54294833
														KLIELQAGK	1	3	1	N-Term(iTRAQ8plex); K1(iTRAQ8plex); K9(iTRAQ8plex)	1.112	0.612	0.727	2.86	-	1	1912.2363	3	638.0836182	0.240525811	20.375835
														ETMVTSTTEPSR	3	3	1	N-Term(iTRAQ8plex); M3(Oxidation)	1.38	0.671	0.729	-	34	0	1658.8176	2	821.9154053	-1.495822938	17.64814833
48255959	ATP2B4	plasma membrane calcium-transporting ATPase 4 isoform 4a	NP_001001396.1	3.85	2	3	6	1170	129.3	7.52	0.383	0.536	0.708																
														GIIDSTVGEHR	2	2	1	N-Term(iTRAQ8plex)	0.613	0.485	0.637	3.92	39	0	1487.80936	3	496.6079712	-1.768355443	16.94802167
														CGILTPGDDFLCLEGK	2	2	1	N-Term(iTRAQ8plex); C1(Methylthio); C12(Methylthio); K16(iTRAQ8plex)	0.24	0.594	0.786	3.87	32	0	2381.19999	3	794.4048462	6.959526424	56.375645
														CGILHPGEDFLCLEGK	2	6	2	N-Term(iTRAQ8plex); C1(Methylthio); C12(Methylthio); K16(iTRAQ8plex)	-	-	-	4.23	-	0	2431.2103	3	811.0749512	0.002865085	50.68653333
														HLDACETMGNATAICSDK	2	26	2	N-Term(iTRAQ8plex); C5(Methylthio); C15(Methylthio); K18(iTRAQ8plex)	-	-	-	4.32	24	0	2580.18601	3	860.7335205	0.563902538	39.61552167
48762926	PWP2	periodic tryptophan protein 2 homolog	NP_005040.2	9.03	6	6	14	919	102.4	6.15	1.827	0.910	0.687																
														GEIEVVTSSLPELYVEK	2	2	1	N-Term(iTRAQ8plex); K17(iTRAQ8plex)	1.036	0.73	0.512	5.68	30	0	2500.41245	3	834.142334	4.698186146	44.674995
														LLDVLSGHEGPISGLCFNPMK	2	3	1	N-Term(iTRAQ8plex); C16(Methylthio); K21(iTRAQ8plex)	2.545	0.958	0.661	4.97	43	0	2881.53	3	961.1815186	2.245374526	63.859045
														VLEFLASSFEVSR	2	2	1	N-Term(iTRAQ8plex)	2.219	0.885	0.743	3.32	39	0	1787.98332	2	894.4953003	-0.686434849	67.649865
														LWDMFDSWR	4	3	1	N-Term(iTRAQ8plex)	1.827	0.959	0.733	3.16	28	0	1559.75298	2	780.380127	-5.708605067	81.06252833
														VLFDPFELDTSVTPGR	2	2	1	N-Term(iTRAQ8plex)	2.063	1.154	0.894	3.04	45	0	2097.11455	2	1049.060913	-1.188179313	55.08046
														AILMALR	2	2	1	N-Term(iTRAQ8plex)	1.668	0.775	0.624	-	27	0	1091.68425	2	546.3457642	-6.430064075	27.69118167
48762932	CCT8	T-complex protein 1 subunit theta isoform 1	NP_006576.2	37.59	16	16	70	548	59.6	5.60	1.168	2.590	1.980																
														QITSYGETCPGLEQYAIK	2	4	1	N-Term(iTRAQ8plex); C9(Methylthio); K18(iTRAQ8plex)	0.878	2.183	1.471	6.94	51	0	2655.36051	3	885.791687	-0.513871785	39.01576
														LIAQACVSIFPDSGHFNVDNIR	9	4	1	N-Term(iTRAQ8plex); C6(Methylthio)	1.834	5.488	4.655	6.54	79	0	2766.41489	3	922.8098145	5.027376561	63.19257833
														AIADTGANVVVTGGK	6	4	1	N-Term(iTRAQ8plex); K15(iTRAQ8plex)	1.028	1.836	1.655	6.27	51	0	1981.16555	3	661.0556641	-0.722994592	26.88389
														NVGLDIEAEVPAVK	6	4	1	N-Term(iTRAQ8plex); K14(iTRAQ8plex)	1.026	2.012	2.128	5.66	69	0	2062.18877	2	1031.598022	-5.684735707	29.60521
														DMLEAGILDTYLGK	4	4	1	N-Term(iTRAQ8plex); K14(iTRAQ8plex)	1.219	2.85	1.983	4.85	67	0	2147.179	2	1074.09314	-4.1303604	42.21900667
														HFSGLEEAVYR	3	2	1	N-Term(iTRAQ8plex)	0.787	1.521	1.262	4.75	51	0	1611.85526	3	537.956604	7.430854386	27.13512667
														GSTDNLMDDIER	6	4	1	N-Term(iTRAQ8plex)	0.809	2.121	1.48	4.75	62	0	1669.80351	2	835.4013672	-3.032434288	22.60140667
														LFVTNDAATILR	8	3	1	N-Term(iTRAQ8plex)	1.114	3.067	2.185	4.71	56	0	1637.94695	3	546.6536255	-3.985530578	45.08657
														ELEVQHPAAK	4	3	1	N-Term(iTRAQ8plex); K10(iTRAQ8plex)	1.29	3.1	2.268	4.49	44	0	1729.99289	2	865.4996948	-7.866088787	14.75043667
														TAEELMNFSK	2	4	1	N-Term(iTRAQ8plex); K10(iTRAQ8plex)	1.153	2.358	1.991	4.01	29	0	1777.96025	2	889.4837646	-0.691029579	43.49159167
														FAEAFEAIPR	8	4	1	N-Term(iTRAQ8plex)	1.23	3.664	2.45	3.85	63	0	1454.79123	3	485.6019287	-2.287900224	47.70832
														LATNAAVTVLR	2	4	1	N-Term(iTRAQ8plex)	0.94	1.268	1.037	3.63	68	0	1432.88303	3	478.2991943	2.846329901	30.33286167
														HEKEDGAISTIVLR	1	4	1	N-Term(iTRAQ8plex); K3(iTRAQ8plex)	0.774	0.942	0.786	3.47	-	1	2176.23769	4	544.8148804	-7.788531414	20.83984833
														LVPGGGATEIELAK	3	4	1	N-Term(iTRAQ8plex); K14(iTRAQ8plex)	0.831	1.571	1.288	3.18	67	0	1963.15215	2	982.0797119	-8.310151558	34.07156
														DIDEVSSLLR	5	4	1	N-Term(iTRAQ8plex)	1.184	2.147	1.969	3.17	57	0	1450.7999	3	484.2714844	-3.867814576	40.261975
														KDWDDDQND	1	4	1	N-Term(iTRAQ8plex); K1(iTRAQ8plex)	1.33	3.867	2.514	-	17	1	1758.82549	2	879.9163818	-7.746219409	14.00631333
49472822	EIF3G	eukaryotic translation initiation factor 3 subunit G	NP_003746.2	14.06	4	4	8	320	35.6	6.13	1.332	1.944	2.319																
														ELAEQLGLSTGEK	2	1	1	N-Term(iTRAQ8plex); K13(iTRAQ8plex)	0.931	1.151	1.403	4.63	44	0	1983.10782	3	661.7074585	-7.094451225	43.712065
														EDLNCQEEEDPMNK	2	1	1	N-Term(iTRAQ8plex); C5(Methylthio); K14(iTRAQ8plex)	1.442	1.781	2.133	3.34	34	0	2348.0535	3	783.3560181	-5.545593734	35.47489833
														GFAFISFHR	2	1	1	N-Term(iTRAQ8plex)	1.584	3.085	4.125	2.91	37	0	1385.77347	3	462.5960083	7.425710301	35.37001833
														VTNLSEDTR	2	1	1	N-Term(iTRAQ8plex)	1.23	2.123	2.522	2.75	24	0	1338.71269	2	669.8599854	-2.978396255	17.62917667
49574502	CYB5R1	NADH-cytochrome b5 reductase 1	NP_057327.2	10.16	1	1	1	305	34.1	9.38	-	-	-																
														EHLPAPGDDVLVLLCGPPPMVQLACHPNLDK	1	1	1	N-Term(iTRAQ8plex); C15(Methylthio); C25(Methylthio); K31(iTRAQ8plex)	-	-	-	3.17	-	0	3989.06582	4	998.0219116	5.440698493	66.59250167
50053795	EIF4B	eukaryotic translation initiation factor 4B isoform 2	NP_001408.2	11.46	5	5	7	611	69.1	5.73	1.154	3.227	2.961																
														AASIFGGAKPVDTAAR	1	2	1	N-Term(iTRAQ8plex); K9(iTRAQ8plex)	-	-	-	3.6	-	0	2140.21694	4	535.8096924	-7.752321011	26.35293333
														SPPYTAFLGNLPYDVTEESIKEFFR	3	2	1	N-Term(iTRAQ8plex); K21(iTRAQ8plex)	-	-	-	3.5	-	1	3528.8644	4	882.9715576	3.746046107	67.82659
														VAPAQPSEEGPGR	1	2	1	N-Term(iTRAQ8plex)	1.11	2.546	2.755	-	37	0	1598.83672	2	799.9219971	-4.580692509	15.02692167
														GLNISAVR	1	2	1	N-Term(iTRAQ8plex)	1.154	3.227	3.479	-	28	0	1133.68816	2	567.3477173	-5.533576093	22.13859
														SILPTAPR	1	2	1	N-Term(iTRAQ8plex)	1.273	3.549	2.961	-	21	0	1158.70757	2	579.8574219	-6.269200449	21.64654833
50083289	EPT1	ethanolaminephosphotransferase 1	NP_277040.1	5.79	1	1	3	397	45.2	6.60	1.298	0.617	0.317																
														YSAVDTNPLSLYVMHPFWNTIVK	3	1	1	N-Term(iTRAQ8plex); K23(iTRAQ8plex)	1.298	0.617	0.317	5.33	24	0	3303.7822	3	1101.932251	3.789105616	61.224455
50345292	HN1	hematological and neurological expressed 1 protein isoform 3	NP_001002033.1	13.89	1	1	1	108	11.0	4.84	1.102	6.899	4.528																
														SSGGREDLESSGLQR	1	4	1	N-Term(iTRAQ8plex)	1.102	6.899	4.528	-	30	1	1881.95317	2	941.4802246	-1.949411549	12.94842833
50428938	ASNA1	ATPase ASNA1	NP_004308.2	14.08	4	4	10	348	38.8	4.91	0.555	0.963	1.281																
														ESVLIISTDPAHNISDAFDQK	4	1	1	N-Term(iTRAQ8plex); K21(iTRAQ8plex)	0.555	0.87	1.355	4.97	33	0	2908.54245	3	970.1856689	-3.041975217	41.81385167
														LPLLPHEVR	3	1	1	N-Term(iTRAQ8plex)	0.52	1.311	1.502	3.6	57	0	1377.84381	3	459.9527893	-5.941624146	28.09419167
														LLNFPTIVER	2	1	1	N-Term(iTRAQ8plex)	0.855	0.963	1.23	3.59	38	0	1505.89519	2	753.4512329	-2.763870764	47.988285
														LEETLPVIR	1	1	1	N-Term(iTRAQ8plex)	0.796	0.815	0.915	2.9	-	0	1373.82707	2	687.4171753	-2.567524057	36.25534333
50592988	UQCRC2	cytochrome b-c1 complex subunit 2, mitochondrial precursor	NP_003357.2	29.58	10	10	38	453	48.4	8.63	1.109	0.655	0.565																
														YEDFSNLGTTHLLR	8	1	1	N-Term(iTRAQ8plex)	1.101	1.041	1.044	5.87	70	0	1970.02494	3	657.3464966	-1.819674381	36.11263
														AVAFQNPQTHVIENLHAAAYR	4	1	1	N-Term(iTRAQ8plex)	1.049	0.594	0.566	5.4	59	0	2654.43308	3	885.4825439	8.494200182	32.61512333
														NALANPLYCPDYR	8	1	1	N-Term(iTRAQ8plex); C9(Methylthio)	0.995	0.573	0.504	5.22	57	0	1859.90911	2	930.4581909	0.270398127	38.16072
														TIAQGNLSNTDVQAAK	2	1	1	N-Term(iTRAQ8plex); K16(iTRAQ8plex)	0.959	0.618	0.456	5.18	70	0	2239.24179	3	747.0854492	-3.790074735	17.360215
														ENMAYTVECLR	4	1	1	N-Term(iTRAQ8plex); C9(Methylthio)	1.149	0.688	0.519	4.63	29	0	1678.79802	2	839.8955688	-3.938103553	28.84268
														MALIGLGVSHPVLKQVAEQFLNMR	2	1	1	N-Term(iTRAQ8plex); K14(iTRAQ8plex)	-	-	-	4.46	-	1	3259.89027	5	652.7838745	8.283944056	65.14394667
														RWEVADLQPQLK	1	1	1	N-Term(iTRAQ8plex); K12(iTRAQ8plex)	1.235	0.458	0.451	3.96	-	1	2091.20798	3	697.7408447	-4.375638576	29.11634833
														SMAASGNLGHTPFVDEL	2	1	1	N-Term(iTRAQ8plex)	1.224	0.832	0.471	3.16	58	0	2050.01933	2	1025.513306	-1.18369219	38.65080833
														WEVADLQPQLK	2	1	1	N-Term(iTRAQ8plex); K11(iTRAQ8plex)	1.658	0.553	0.591	3.06	38	0	1935.10654	3	645.7070313	-4.894200811	57.70339333

														AVAFQNPQTHVIENLHAAAYR	1	1	1	N-Term(iTRAQ8plex); Y20(iTRAQ8plex)	1.252	0.556	0.413	-	52	0	2958.63626	3	885.4825439	8.494200182	32.61512333
														IGLFIK	3	1	1	N-Term(iTRAQ8plex); K6(iTRAQ8plex)	1.132	0.693	0.601	-	23	0	1298.86979	3	433.6281128	3.198033362	37.07580833
														ENMAYTVECLR	1	1	1	N-Term(iTRAQ8plex); M3(Oxidation); C9(Methylthio)	1.063	0.876	0.655	-	21	0	1694.78948	2	839.8955688	-3.938103553	28.84268
50592996	TUBB3	tubulin beta-3 chain isoform 1	NP_006077.2	52.00	4	17	245	450	50.4	4.93	0.832	0.942	3.151																
														FWEVISDEHGIDPSGNYVGDSDLQLER	9	1	1	N-Term(iTRAQ8plex)	-	-	6.926	5.66	55	0	3381.61667	3	1127.873047	-3.90463304	51.592065
														LHFFMPGFAPLTAR	16	2	1	N-Term(iTRAQ8plex)	0.872	0.744	3.242	4.56	68	0	1909.04386	3	637.0194702	-1.154455127	51.21864833
														LATPTYGDLNHLVSATMSGVTTSLR	4	2	1	N-Term(iTRAQ8plex)	0.376	0.814	3.23	4.52	33	0	2909.53159	3	970.5153809	-0.997401742	59.92193833
														LHFFMPGFAPLTAR	4	2	1	N-Term(iTRAQ8plex); M5(Oxidation)	0.959	0.942	1.729	4.35	30	0	1925.03464	3	637.0194702	-1.154455127	51.21864833
														SGAFGHLFRPDNFIFGQSGAGNNWAK	1	2	1	N-Term(iTRAQ8plex); K26(iTRAQ8plex)	-	1.187	1.157	4.17	-	0	3403.77895	4	851.7001953	7.165585765	47.40837833
														EVDEQMLAIQSK	4	7	2	N-Term(iTRAQ8plex); K12(iTRAQ8plex)	-	-	-	4.11	61	0	1999.0811	2	1000.044189	-8.978632097	23.45914833
														YLTVATVFR	8	7	2	N-Term(iTRAQ8plex)	-	-	-	3.4	35	0	1373.80332	3	458.605957	-4.464581043	44.41719167
														AILVDLEPGTMDSVR	10	7	3	N-Term(iTRAQ8plex)	-	-	-	4.45	57	0	1920.02982	2	960.5213623	-3.121458321	44.53172
														AILVDLEPGTMDSVR	4	7	3	N-Term(iTRAQ8plex); M11(Oxidation)	-	-	-	2.95	45	0	1936.028	2	960.5213623	-3.121458321	44.53172
														EIVHIQAGQCGNQIGAK	24	9	4	N-Term(iTRAQ8plex); C10(Methylthio); K17(iTRAQ8plex)	-	-	-	6.74	73	0	2420.28691	2	1210.647095	-5.366911999	28.04771167
														NSSYFVEWIPNNVK	31	20	5	N-Term(iTRAQ8plex); K14(iTRAQ8plex)	-	-	-	4.24	49	0	2305.22641	2	1153.123413	-1.809680962	42.78834
														FPGQLNADLR	9	25	5	N-Term(iTRAQ8plex)	-	-	-	4.12	55	0	1434.7963	3	478.9369507	-3.068379269	31.15012333
														LAVNMVPFPR	13	25	5	N-Term(iTRAQ8plex)	-	-	-	4.01	51	0	1447.83405	3	483.2828674	-3.931565971	41.77579833
														LAVNMVPFPR	4	25	5	N-Term(iTRAQ8plex); M5(Oxidation)	-	-	-	3.45	44	0	1463.82685	3	483.2828674	-3.931565971	41.77579833
														KLAVNMVPFPR	15	25	5	N-Term(iTRAQ8plex); K1(iTRAQ8plex)	-	-	-	3.44	37	1	1880.12295	4	470.7861938	-9.108866745	31.42844
														FPGQLNADLRK	1	25	5	N-Term(iTRAQ8plex); K11(iTRAQ8plex)	-	-	-	3.07	-	1	1867.09755	4	467.5298462	-1.861935048	33.46670167
														NMMAACDPR	3	23	5	N-Term(iTRAQ8plex); C6(Methylthio)	-	-	-	-	55	0	1358.59062	2	679.7989502	-6.255401168	21.43194333
														NMMAACDPR	5	23	5	N-Term(iTRAQ8plex); M3(Oxidation); C6(Methylthio)	-	-	-	-	47	0	1374.58525	2	679.7989502	-6.255401168	21.43194333
														NMMAACDPR	1	23	5	N-Term(iTRAQ8plex); M2(Oxidation); M3(Oxidation); C6(Methylthio)	-	-	-	-	34	0	1390.5822	2	679.7989502	-6.255401168	21.43194333
														GHYTEGAELVDSVLDVVR	51	11	4	N-Term(iTRAQ8plex)	-	-	-	5.05	98	0	2263.18999	2	1132.098633	1.214084992	52.94817667
														GHYTEGAELVDSVLDVVR	10	11	4	N-Term(iTRAQ8plex); Y3(iTRAQ8plex)	-	-	-	-	64	0	2567.37212	2	1132.098633	1.214084992	52.94817667
														IMNTFSVVPSPK	18	11	3	N-Term(iTRAQ8plex); K12(iTRAQ8plex)	-	-	-	4.54	51	0	1928.10552	2	964.553772	-6.900042966	31.209235
														IMNTFSVVPSPK	2	11	3	N-Term(iTRAQ8plex); M2(Oxidation); K12(iTRAQ8plex)	-	-	-	3.64	37	0	1944.09331	2	964.553772	-6.900042966	31.209235
														ALTVPELTQQMFDAK	10	7	2	N-Term(iTRAQ8plex); K15(iTRAQ8plex)	-	-	-	5.83	52	0	2300.27726	3	772.7599487	-3.33008426	35.96597833
														ALTVPELTQQMFDAK	2	7	2	N-Term(iTRAQ8plex); M11(Oxidation); K15(iTRAQ8plex)	-	-	-	4.01	60	0	2316.26529	3	772.7599487	-3.33008426	35.96597833
50593002	SNRPA1	U2 small nuclear ribonucleoprotein A'	NP_003081.2	26.27	4	4	6	255	28.4	8.62	1.132	1.069	0.858																
														NAIANASTLAEVER	2	1	1	N-Term(iTRAQ8plex)	1.132	0.956	0.859	3.49	59	0	1762.95647	2	881.9818726	-2.077647904	30.0068
														SLTYLSILR	1	1	1	N-Term(iTRAQ8plex)	1.197	1.069	0.858	3.44	-	0	1369.83232	2	685.4197998	-2.437021356	45.26636667
														VKLTAELIEQAAQYTNAVR	2	1	1	N-Term(iTRAQ8plex); K2(iTRAQ8plex)	1.053	1.177	0.538	-	55	1	2726.56949	3	909.5280151	1.225298004	61.82083833
														IPVIENLGATLDQFDAIDFSDNEIR	1	1	1	N-Term(iTRAQ8plex)	-	-	-	-	25	0	3109.61203	3	1037.208862	3.988395957	68.17689333
50659095	DDX21	nucleolar RNA helicase 2 isoform 1	NP_004719.2	34.61	18	19	97	783	87.3	9.28	2.926	1.547	1.167																
														LGVCFDVPTASVTEIQEK	6	4	1	N-Term(iTRAQ8plex); C4(Methylthio); K18(iTRAQ8plex)	2.613	1.492	0.972	6.53	67	0	2590.36772	2	1295.6875	-1.551486286	48.61496667
														GAVEALAAALAHISGATSVDQR	24	4	1	N-Term(iTRAQ8plex)	4.117	1.357	1.116	6.51	63	0	2412.31491	3	804.7758789	-0.765999763	58.41789333
														WQLSVATEQPELEGPR	5	4	1	N-Term(iTRAQ8plex)	0.907	0.598	0.776	6.25	47	0	2144.11686	2	1072.573608	5.1017551	35.11242667
														TAITVEHLAIK	7	4	1	N-Term(iTRAQ8plex); K11(iTRAQ8plex)	3.493	1.734	1.22	5.76	60	0	1804.11455	2	902.5609131	-0.400056707	27.81283833
														DSEDNPQTLLFSATCPHWVFNVAK	8	4	1	N-Term(iTRAQ8plex); C15(Methylthio); K24(iTRAQ8plex)	-	-	-	5.45	41	0	3373.68276	4	844.1761475	0.630665033	59.39106667
														GLDIPEVDLVIQSSPPKDVESYIHR	3	2	1	N-Term(iTRAQ8plex); K17(iTRAQ8plex)	6.551	3.969	2.072	5.23	-	1	3414.85976	4	854.4703979	-3.874063372	49.71778333
														KLSVACFYGGTPYGGQFER	6	4	1	N-Term(iTRAQ8plex); K1(iTRAQ8plex); C6(Methylthio)	4.767	3.28	3.006	5.13	53	1	2734.39011	3	912.1348877	-1.493007178	41.45839333
														SDKTEEIAEEEETVFPK	2	2	1	N-Term(iTRAQ8plex); K3(iTRAQ8plex); K17(iTRAQ8plex)	4.496	2.27	1.655	5.06	44	1	2893.52304	3	965.1791992	-7.450050827	31.58550667
														LLDSVPPTAISHFK	2	4	1	N-Term(iTRAQ8plex); K14(iTRAQ8plex)	1.573	1.365	0.96	4.77	46	0	2133.24021	3	711.7515869	-5.922355939	35.83704
														EEYQLVQVEQK	2	4	1	N-Term(iTRAQ8plex); K11(iTRAQ8plex)	3.128	1.528	1.188	4.66	64	0	2001.09661	2	1001.051941	-7.344015289	34.56115
														RWQLSVATEQPELEGPR	2	4	1	N-Term(iTRAQ8plex)	0.763	0.738	0.501	4.42	36	1	2300.22562	3	767.4133911	-1.956855935	30.52253833
														TFSFAIPLIEK	6	4	1	N-Term(iTRAQ8plex); K11(iTRAQ8plex)	4.17	1.981	1.278	4.41	57	0	1874.1248	2	937.559082	-7.413572801	41.69722667
														GVTFLFPIQAK	7	4	1	N-Term(iTRAQ8plex); K11(iTRAQ8plex)	2.321	1.183	0.942	4.29	74	0	1829.11191	2	915.055481	-5.940617815	35.95531167
														QDAQSLHGDIPQK	2	2	1	N-Term(iTRAQ8plex); K13(iTRAQ8plex)	1.598	0.935	0.77	3.92	33	0	2045.11374	3	682.3760986	-4.825478093	16.140055
														TIIFCETK	6	4	1	N-Term(iTRAQ8plex); C5(Methylthio); K8(iTRAQ8plex)	2.245	1.349	1.278	3.66	35	0	1608.88089	3	536.9651489	-8.74444635	49.20851
														NGSFGVLVATNVAAR	1	2	1	N-Term(iTRAQ8plex)	1.624	1.11	1.297	3.55	-	0	1780.00896	2	890.5081177	3.942357441	32.51417833
														APQVLVLAPTR	3	4	1	N-Term(iTRAQ8plex)	2.343	1.595	0.998	3.42	52	0	1468.91155	2	734.9594116	-2.583326287	30.55192833
														AAVIGDVIR	2	4	1	N-Term(iTRAQ8plex)	3.059	1.624	1.48	3.02	31	0	1217.74639	2	609.3756104	-6.583663925	25.77389
														EGAFSNFPISEETIK	3	6	2	N-Term(iTRAQ8plex); K15(iTRAQ8plex)	-	-	-	3.55	62	0	2277.21367	2	1139.110474	-3.807571721	34.28320833
50959085	THOC5	THO complex subunit 5 homolog	NP_003669.4	7.32	3	3	6	683	78.5	6.87	1.254	0.992	1.073																
														ELCGPWPSHQLLTNQLQR	2	3	1	N-Term(iTRAQ8plex); C3(Methylthio)	1.232	1.05	1.171	5.65	34	0	2470.25534	3	824.0899658	-3.39563644	41.39173833
														QFASLEHGIVPVTSDCQYLFPAK	2	3	1	N-Term(iTRAQ8plex); C16(Methylthio); K23(iTRAQ8plex)	-	-	-	4.05	28	0	3204.65078	4	801.9181519	-5.456057255	55.62088167
														DVAIEIEER	2	2	1	N-Term(iTRAQ8plex)	1.277	0.937	0.983	3.22	29	0	1377.75676	2	689.382019	2.910648708	27.87869
50959176	ERGIC2	endoplasmic reticulum-Golgi intermediate compartment protein 2	NP_057654.2	5.84	1	1	2	377	42.5	6.77	-	-	-																
														IDHLSFGELVPAIINPLDGTEK	2	1	1	N-Term(iTRAQ8plex); K22(iTRAQ8plex)	-	-	-	3.87	29	0	2986.68056	4	747.4255981	3.18795329	58.48185
51094101	DDX54	ATP-dependent RNA helicase DDX54 isoform 2	NP_076977.3	6.13	3	3	6	881	98.5	10.02	2.022	1.198	1.837																
														GLDIPLLDNVINYSFPAK	2	2	1	N-Term(iTRAQ8plex); K18(iTRAQ8plex)	1.971	1.476	1.941	5.53	42	0	2597.49771	3	866.5040894	6.834853523	62.15232833
														KLGPGRPLPTFPTSECTSDVEPDTR	2	2	1	N-Term(iTRAQ8plex); K1(iTRAQ8plex); C16(Methylthio)	-	-	-	4.76	31	1	3354.71963	4	839.4353638	-2.595478266	33.18066167
														TACFLLPMFER	2	2	1	N-Term(iTRAQ8plex); C3(Methylthio)	2.074	0.973	1.738	4.14	26	0	1677.85588	2	839.4315796	5.364143477	57.883575
51100974	MYO1D	unconventional myosin-Id isoform 1	NP_056009.1	3.48	3	3	5	1006	116.1	9.39	0.571	0.261	0.148																
														GIIAILDDACMNVGK	2	3	1	N-Term(iTRAQ8plex); C10(Methylthio); K15(iTRAQ8plex)	0.638	0.195	-	4.39	52	0	2187.18052	3	729.7316895	0.394484757	60.56792
														VVSIIAELLSTK	2	4	1	N-Term(iTRAQ8plex); K12(iTRAQ8plex)	0.436	-	0.092	3.49	40	0	1881.19004	3	627.7348633	1.023444582	66.71241833
														CTVSVETR	1	1	1	N-Term(iTRAQ8plex); C1(Methylthio)	0.571	0.349	0.241	-	19	0	1244.62505	2	622.8161621	-2.422176266	27.914585
51173724	BYSL	bystin	NP_004044.3	9.38	3	3	5	437	49.6	8.12	1.273	0.496	1.037																
														ELPVLWHQCLLTLVQR	2	1	1	N-Term(iTRAQ8plex); C9(Methylthio)	1.273	0.458	1.072	4.76	39	0	2298.28232	3	766.765625	2.375447937	67.314295
														GTGEAEEEYVGPR	2	1	1	N-Term(iTRAQ8plex)	1.691	0.911	1.037	3.96	21	0	1697.8178	2	849.4125366	-6.26962318	14.979395
														LQPHPQLSPEIR	1	1	1	N-Term(iTRAQ8plex)	1.107	0.496	0.81	3.89	-	0	1718.98075	3	573.6651001	-2.789011476	21.34950333
51243032	OSBPL8	oxysterol-binding protein-related protein 8 isoform b	NP_001003712.1	4.37	3	3	4	847	96.9	7.55	1.473	0.744	1.291																
														CLWIHPR	2	7	1	N-Term(iTRAQ8plex); C1(Methylthio)	1.482	1.33	1.291	3.12	36	0	1274.67112	3	425.5618896	-7.261955318	30.45275667
														KPYNPILGETFR	1	10	1	N-Term(iTRAQ8plex); K1(iTRAQ8plex)	0.807	0.658	0.726	2.95	-	0	2043.1818	3	681.7321167	-1.446005612	26.02558667
														FADTRPWDPLNDMIQFEK	1	7	1	N-Term(iTRAQ8plex); K18(iTRAQ8plex)	1.473	0.744	1.664	2.82	-	0	2831.46108	4	708.6207275	-1.29651217	52.20507167
51243055	MTFP1	mitochondrial fission process protein 1 isoform a	NP_057582.2	12.65	1	1	2	166	18.0	9.31	-	-	-																
														KWTTTALGLLTIPIIHPIDR	2	1	1	N-Term(iTRAQ8plex); K1(iTRAQ8plex)	-	-	-	4.4	35	1	2980.82412	4	745.9614868	2.306773347	59.548765
51317370	NDUFA6	NADH dehydrogenase [ubiquinone] 1 alpha subcomplex subunit 6	NP_002481.2	15.58	2	2	5	154	17.9	10.14	1.254	1.192	0.492																
														VVDLLVIK	4	1	1	N-Term(iTRAQ8plex); K8(iTRAQ8plex)	1.257	1.228	0.5	3.16	38	0	1507.00367	3	503.006073	-2.843759413	36.26766833
														QATSTASTFVKPIFSR	1	1	1	N-Term(iTRAQ8plex); K11(iTRAQ8plex)	1.254	1	0.492	3.04	-	0	2349.33249	3	783.7823486	-2.642730044	29.50243167
51317376	SNU13	NHP2-like protein 1	NP_001003796.1	18.75	2	2	19	128	14.2	8.46	1.492	1.419	1.021																
														LLDLVQQSCNYK	17	1	1	N-Term(iTRAQ8plex); C9(Methylthio); K12(iTRAQ8plex)	1.527	1.472	1.065	4.62	50	0	2078.10294	3	693.3724976	-9.875773634	77.65308833
														QQIQSIQQSIER	2	1	1	N-Term(iTRAQ8plex)	1.302	0.722	0.618	4.14	42	0	1761.96807	2	881.4876709	-4.555203516	21.66229
51479152	ATP5H	ATP synthase subunit d, mitochondrial isoform b	NP_001003785.1	69.34	7	7	35	137	15.8	7.18	0.776	0.545	0.502																
														LAALPENPPAIDWAYYK	10	2	1	N-Term(iTRAQ8plex); K17(iTRAQ8plex)	0.79	0.676	0.521	4.96	56	0	2540.4207	2	1270.713989	7.766664636	46.34934167
														TIDWVAFAEIIPQNQK	12	2	1	N-Term(iTRAQ8plex); K16(iTRAQ8plex)	0.633	0.393	0.437	4.84	65	0	2481.392	2	1241.211548	7.893793122	52.602645
														NLIPFDQMTIEDLNEAFPETK	2	2	1	N-Term(iTRAQ8plex); K21(iTRAQ8plex)	1.061	0.506	0.542	4.49	26	0	3073.59775	3	1025.204102	-1.158207023	59.22711
														SCAEWVSLSK	5	2	1	N-Term(iTRAQ8plex); C2(Methylthio); K10(iTRAQ8plex)	0.795	0.586	0.42	4.06	39	0	1763.91692	2	882.4620972	-6.318088993	58.39920833
														AGLVDDFEK	4	2	1	N-Term(iTRAQ8plex); K9(iTRAQ8plex)	0.653	0.51	0.514	3.7	48	0	1601.89849	2	801.4528809	-0.653100842	41.33489167
														SWNETLTSR	1	2	1	N-Term(iTRAQ8plex)	0.522	0.301	0.39	3.4	-	0	1397.73528	2	699.3712769	1.871449466	25.75899
														KYPYWPHQPIENL	1	2	1	N-Term(iTRAQ8plex); K1(iTRAQ8plex)	1.07	0.668	0.859	2.71	-	1	2293.24948	3	765.0880127	-4.132330183	35.42776667
51479156	ATP5L	ATP synthase subunit g, mitochondrial	NP_006467.4	35.92	3	3	18	103	11.4	9.64	0.691	0.402	0.454																
														LATFWYYAK	12	1	1	N-Term(iTRAQ8plex); K9(iTRAQ8plex)	0.676	0.401	0.436	4.71	42	0	1770.99675	2	886.0020142	-4.037048448	72.50235833
														TPALVNAAVTYSKPR	2	1	1	N-Term(iTRAQ8plex); K13(iTRAQ8plex)	0.537	0.287	0.343	4.62	29	0	2196.29624	3	732.7702637	0.062134435	28.23255
														VELVPPTPAEIPR	4	1	1	N-Term(iTRAQ8plex)	0.887	0.642	0.633	3.15	41	0	1722.00652	2	861.506897	-2.243879247	37.15062333
51944953	NOMO1	nodal modulator 1 precursor	NP_055102.3	12.93	10	10	20	1222	134.2	5.81	1.106	0.586	0.627																
														VLPGDYEILATHPTWALK	2	7	1	N-Term(iTRAQ8plex); K18(iTRAQ8plex)	0.678	0.543	0.596	5.33	27	0	2632.48305	4	658.8762207	-4.895897615	47.32290667
														EGYVLTAVEGTIGDFK	4	5	1	N-Term(iTRAQ8plex); K16(iTRAQ8plex)	1.572	0.694	0.587	5.06	39	0	2307.27042	3	769.7616577	0.489147055	50.207645
														SENLDNPIQTVSLGQSLFFHFPPLLR	2	6	1	N-Term(iTRAQ8plex)	0.853	-	-	4.32	44	0	3273.77817	3	1091.930908	6.32909693	67.02834833
														MTKPPVQEMVDELQGPFSYDFSYWAR	2	6	1	N-Term(iTRAQ8plex); K3(iTRAQ8plex)	-	-	-	4.29	43	0	3729.85341	4	933.218811	-0.227097229	64.500265
														GQPLGPAGVQVSLR	2	7	1	N-Term(iTRAQ8plex)	1.106	0.58	0.808	3.78	46	0	1682.9937	2	842.0004883	4.835812814	28.94730167
														IEPVFHVMGFSVTGR	2	7	1	N-Term(iTRAQ8plex)	1.244	0.824	0.819	3.42	25	0	1980.06424	3	660.6929321	-1.857597951	46.18156833
														ALGQAASDNSGPEDAKR	2	1	1	N-Term(iTRAQ8plex); K16(iTRAQ8plex)	1.193	0.098	0.558	3.4	26	1	2295.21494	3	765.7431641	-0.018456877	14.81108
														FLLFSSLVTK	2	7	1	N-Term(iTRAQ8plex); K10(iTRAQ8plex)	1.019	0.435	0.675	3.06	25	0	1763.09433	3	588.3696289	0.903053637	51.474095
														ITFDVAPSR	1	7	1	N-Term(iTRAQ8plex)	1.032	0.586	0.484	-	28	0	1309.74016	2	655.3737183	-1.237952849	27.90054167
														LIEIHGK	1	5	1	N-Term(iTRAQ8plex); K7(iTRAQ8plex)	1.107	0.731	0.627	-	19	0	1417.89942	3	473.304657	0.491147155	26.89937
51988900	ERCC5	DNA repair protein complementing XP-G cells	NP_000114.2	1.52	1	1	1	1186	133.2	5.26	1.393	1.115	0.768																
														EKEAAASEIEAVSVAMEK	1	2	1	N-Term(iTRAQ8plex); K2(iTRAQ8plex); M16(Oxidation); K18(iTRAQ8plex)	1.393	1.115	0.768	2.98	-	1	2820.52164	3	940.8453979	-7.518361866	39.05947167
52352803	SLC30A1	zinc transporter 1	NP_067017.2	2.17	1	1	1	507	55.3	6.48	0.564	0.472	0.455																
														EPDHMELEEDR	2	1	1	N-Term(iTRAQ8plex)	0.564	0.472	0.455	3.29	19	0	1703.78574	3	568.6000977	0.509699506	14.82591333
52487191	ERP44	endoplasmic reticulum resident protein 44 precursor	NP_055866.1	19.21	5	5	21	406	46.9	5.26	1.148	0.431	0.567																
														VANILHDDCAFLSAFGDVSKPER	10	1	1	N-Term(iTRAQ8plex); C9(Methylthio); K20(iTRAQ8plex)	1.346	0.575	0.532	6.27	41	0	3158.62973	3	1053.548096	2.32112023	60.53897
														EDTISLKPQNEYSR	2	1	1	N-Term(iTRAQ8plex); K7(iTRAQ8plex)	[illegible]	[illegible]	[illegible]	[illegible]	54	0	[illegible]	2	[illegible]	[illegible]	[illegible]
														VDCEQHSDAQR	4	1	1	N-Term(iTRAQ8plex); C3(Methylthio)	[illegible]	[illegible]	[illegible]	[illegible]	41	0	[illegible]	2	[illegible]	[illegible]	[illegible]

														TPADCPVIAIDSFR	2	1	1	N-Term(iTRAQ8plex); C5(Methylthio)	0.854	0.756	0.694	3.81	28	0	1854.93766	3	618.9840698	-1.049133558	50.24259333
														GTINFLHADCDKFR	1	1	1	N-Term(iTRAQ8plex); C10(Methylthio); K12(iTRAQ8plex)	2.022	0.424	1.206	3.05	-	1	2291.18227	4	573.5510254	-2.741212507	34.33413333
53759151	SCD	acyl-CoA desaturase	NP_005054.3	7.24	2	2	7	359	41.5	9.00	1.702	0.578	0.607																
														GSTLDLSDLEAEK	5	1	1	N-Term(iTRAQ8plex); K13(iTRAQ8plex)	1.603	0.578	0.591	5.31	74	0	1986.09136	2	993.5493164	3.114102723	32.50605
														GFFFSHVGWLLVR	2	1	1	N-Term(iTRAQ8plex)	3.559	-	2.327	3.47	43	0	1869.04245	3	623.6856689	-2.839803907	59.42944667
54112117	SF3B1	splicing factor 3B subunit 1 isoform 1	NP_036565.2	15.18	11	11	26	1304	145.7	7.09	1.299	1.026	0.909																
														SLVEIIEHGLVDEQQK	2	1	1	N-Term(iTRAQ8plex); K16(iTRAQ8plex)	1.299	1.026	1.103	5.15	45	0	2445.37735	3	815.7973022	-1.479449261	51.09973
														AIGPHDVLATLLNNLK	8	1	1	N-Term(iTRAQ8plex); K16(iTRAQ8plex)	1.311	1.04	1.006	4.91	65	0	2297.37627	2	1149.191772	-1.703246516	58.18349167
														ATVNTFGYIAK	4	1	1	N-Term(iTRAQ8plex); K11(iTRAQ8plex)	1.145	0.974	0.784	4.39	62	0	1793.0302	2	897.0187378	-6.44848819	37.151325
														AAGLATMISTMRPDIDNMDEYVR	2	1	1	N-Term(iTRAQ8plex)	1.173	1.027	0.999	4.27	-	0	2874.41013	3	958.8082275	-0.045899091	76.81586
														LLVDVDESTLSPEEQKER	1	1	1	N-Term(iTRAQ8plex); K16(iTRAQ8plex)	-	1.557	0.764	4.26	-	1	2695.46157	4	674.6208496	0.177059657	33.71391
														VKPYLPQICGTVLWR	3	1	1	N-Term(iTRAQ8plex); K2(iTRAQ8plex); C9(Methylthio)	-	-	0.446	4.23	30	0	2427.39052	4	607.6013184	-1.268337933	56.98223667
														NRPLSDEELDAMFPEGYK	1	1	1	N-Term(iTRAQ8plex); K18(iTRAQ8plex)	-	-	-	3.68	-	0	2719.37172	4	680.5983887	-5.183731364	35.35468333
														EFGAGPLFNQILPLLMSPTLEDQER	2	1	1	N-Term(iTRAQ8plex)	-	-	-	3.28	53	0	3119.65573	3	1040.556763	5.363904661	68.9788
														EVMLILIR	1	1	1	N-Term(iTRAQ8plex)	1.037	0.778	0.909	2.92	-	0	1290.81365	2	645.9104614	1.181372359	43.352235
														ILVVIEPLLIDEDYYAR	1	1	1	N-Term(iTRAQ8plex)	-	-	-	-	30	0	2338.3209	3	780.1118164	-0.134853711	70.087395
														TISALAIAALAEAATPYGIESFDSVLKPLWK	1	1	1	N-Term(iTRAQ8plex); K27(iTRAQ8plex); K31(iTRAQ8plex)	-	-	-	-	30	0	4159.37124	4	1040.598267	-2.65539504	71.78906833
54112121	SF3B3	splicing factor 3B subunit 3	NP_036558.3	12.41	11	11	30	1217	135.5	5.26	1.259	1.072	1.008																
														HGLEVSEMAVSELPGNPNAVWTVR	2	1	1	N-Term(iTRAQ8plex)	1.302	-	0.287	5.62	31	0	2896.49228	3	966.1689453	-0.24464505	44.61550667
														SMFFFLAQTEQGDIFK	8	1	1	N-Term(iTRAQ8plex); K16(iTRAQ8plex)	1.411	1.272	0.934	5.58	55	0	2517.33908	3	839.7845459	3.266602169	56.94606667
														FLAVGLVDNTVR	6	1	1	N-Term(iTRAQ8plex)	1.191	0.928	0.973	4.87	54	0	1607.93845	3	536.651001	-2.38915881	47.61133
														NVIDGDLCEQFNSMEPNK	2	1	1	N-Term(iTRAQ8plex); C8(Methylthio); M14(Oxidation); K18(iTRAQ8plex)	3.984	3.174	1.2	4.84	68	0	2723.28366	3	908.4327393	-3.640008822	38.41318333
														LKYFDTVPVAAAMCVLK	1	1	1	N-Term(iTRAQ8plex); K2(iTRAQ8plex); C14(Methylthio); K17(iTRAQ8plex)	2.398	1.081	1.28	4.75	-	1	2827.58779	4	707.6524048	-6.147656826	61.72493833
														LTISSPLEAHK	2	1	1	N-Term(iTRAQ8plex); K11(iTRAQ8plex)	1.338	1.236	1.169	4.39	26	0	1804.08725	3	602.0339355	4.64062401	24.14829833
														MFLYNLTLQR	3	1	1	N-Term(iTRAQ8plex)	1.068	1.052	1.088	4.11	27	0	1602.89128	2	801.9492798	-4.166439582	37.01968
														IVPGQFLAVDPK	2	1	1	N-Term(iTRAQ8plex); K12(iTRAQ8plex)	0.941	0.801	0.685	3.62	36	0	1892.13066	2	946.5689697	-8.418270042	31.09181667
														NVIDGDLCEQFNSMEPNK	1	1	1	N-Term(iTRAQ8plex); C8(Methylthio); K18(iTRAQ8plex)	1.177	0.867	1.085	3.53	-	0	2707.28739	3	908.4327393	-3.640008822	38.41318333
														QQEIVVSR	1	1	1	N-Term(iTRAQ8plex)	1.047	0.816	0.735	3.16	-	0	1262.73931	2	631.873291	1.808854164	18.50535833
														NVSEELDRTPPEVSK	1	1	1	N-Term(iTRAQ8plex); K15(iTRAQ8plex)	1.674	1.932	1.836	3.04	-	1	2308.25846	3	770.0910034	-0.894308905	23.63709667
														FGNICVVR	1	1	1	N-Term(iTRAQ8plex); C5(Methylthio)	1.259	1.209	0.986	-	21	0	1257.66838	2	629.3378296	-5.231697325	29.36901
54607053	GCN1	eIF-2-alpha kinase activator GCN1	NP_006827.1	2.96	6	6	18	2671	292.5	7.43	0.679	2.348	1.865																
														FSSVQLLGDLLFHISGVTGK	4	1	1	N-Term(iTRAQ8plex); K20(iTRAQ8plex)	-	-	-	5.22	56	0	2726.57436	4	682.3990479	1.533142213	69.97317
														IKNPFLSLAACVMPSR	4	1	1	N-Term(iTRAQ8plex); K2(iTRAQ8plex); C11(Methylthio)	0.813	2.149	1.213	5	42	1	2401.32924	4	601.0877686	-3.597158228	61.23184
														LSSFWQLIVDEK	2	1	1	N-Term(iTRAQ8plex); K12(iTRAQ8plex)	0.507	2.258	1.886	4.13	30	0	2073.17649	3	691.7303467	-3.668048034	82.75593333
														IIIEDLLEATR	4	1	1	N-Term(iTRAQ8plex)	0.638	2.853	2.127	3.76	41	0	1589.94243	2	795.4748535	0.508893426	57.41001333
														LTEVLTDSHVK	1	1	1	N-Term(iTRAQ8plex); K11(iTRAQ8plex)	0.933	2.348	1.694	3.53	-	0	1850.07254	3	617.3623657	-6.387523597	32.369675
														ALVAVLLSR	3	1	1	N-Term(iTRAQ8plex)	0.801	2.804	1.98	2.83	38	0	1245.81804	2	623.4126587	-1.284727894	43.71979333
54607135	TOMM70	mitochondrial import receptor subunit TOM70	NP_055635.3	20.23	9	9	31	608	67.4	7.12	1.681	0.784	0.513																
														CAEGYALYAQALTDQQQFGK	4	1	1	N-Term(iTRAQ8plex); C1(Methylthio); K20(iTRAQ8plex)	1.826	1.005	0.662	6.68	57	0	2859.40775	3	953.8074341	-6.596926903	43.78249
														YEQAIQCYTEAISLCPTEK	4	1	1	N-Term(iTRAQ8plex); C7(Methylthio); C15(Methylthio); K19(iTRAQ8plex)	1.625	0.212	0.227	6.59	35	0	2890.40371	4	723.3563843	2.817575052	64.31785
														SYFSSFTDDIISQPMLK	5	1	1	N-Term(iTRAQ8plex); K17(iTRAQ8plex)	1.734	1.19	0.827	5.58	58	0	2587.36333	2	1294.185303	2.271636412	49.89238667
														NREPLMPSPQFIK	2	1	1	N-Term(iTRAQ8plex); K13(iTRAQ8plex)	1.638	1.55	0.794	4.1	-	1	2165.22745	3	722.4140015	-4.022743876	25.653415
														GLLQLQWK	8	1	1	N-Term(iTRAQ8plex); K8(iTRAQ8plex)	1.684	0.796	0.526	3.68	49	0	1593.99479	2	797.5026855	2.76234568	34.56177333
														SEMEMAHLYSLCDAAHAQTEVAK	1	1	1	N-Term(iTRAQ8plex); C12(Methylthio); K23(iTRAQ8plex)	0.863	-	0.216	3.57	-	0	3189.52529	4	798.1367798	-1.418091711	42.308105
														YMAEALLLR	4	1	1	N-Term(iTRAQ8plex)	1.72	0.743	0.418	3.47	35	0	1383.7935	2	692.4003906	-2.664176402	46.267885
														NVDLSTFYQNR	2	1	1	N-Term(iTRAQ8plex)	1.643	0.706	0.425	3.21	33	0	1660.85417	2	830.9307251	-3.298137052	24.09862667
														AGKYEQAIQCYTEAISLCPTEK	1	1	1	N-Term(iTRAQ8plex); K3(iTRAQ8plex); C10(Methylthio); C18(Methylthio); [illegible]	-	-	-	3	-	1	3450.74087	4	863.4406738	-3.946360515	58.84763667
54859722	NUP160	nuclear pore complex protein Nup160 isoform 1	NP_056046.1	2.51	2	2	4	1436	162.0	5.50	0.818	0.867	0.787																
														NPIHAIGLLIR	2	1	1	N-Term(iTRAQ8plex)	0.818	0.867	1.018	4.02	44	0	1520.95749	3	507.6573486	-0.248956045	37.64090667
														SQLQDLVEFPYVNLHNEVVGIIESR	2	1	1	N-Term(iTRAQ8plex)	-	-	0.609	2.75	41	0	3202.71201	3	1068.242188	2.166273452	58.36684333
55741641	KIDINS220	kinase D-interacting substrate of 220 kDa	NP_065789.1	0.68	1	1	1	1771	196.4	6.62	0.774	0.743	0.563																
														NAESHVVPEDPR	1	1	1	N-Term(iTRAQ8plex)	0.774	0.743	0.563	3.68	-	0	1653.84317	3	551.9525757	-4.03151842	12.29958667
55741709	RBM25	RNA-binding protein 25	NP_067062.1	7.71	5	5	13	843	100.1	6.32	1.076	1.136	0.922																
														LQAFGFCEYKEPESTLR	2	1	1	N-Term(iTRAQ8plex); C7(Methylthio); K10(iTRAQ8plex)	1.196	1.063	0.939	5.02	33	1	2672.37197	4	668.8484497	1.742514956	45.95930333
														RFPVAPLIPYPLITK	4	2	1	N-Term(iTRAQ8plex); K15(iTRAQ8plex)	1.221	1.297	0.664	3.83	29	1	2333.45633	3	778.4902954	-0.273551093	47.09977333
														LLAEGHPDPDAELQR	4	2	1	N-Term(iTRAQ8plex)	1.004	0.952	0.92	3.54	26	0	1965.0322	3	655.6820679	-1.384678317	25.43103333
														GAIEVLIR	2	2	1	N-Term(iTRAQ8plex)	1.526	1.482	0.969	3.09	27	0	1174.7448	2	587.8760376	-1.142992664	31.59449167
														LLHDLQIGEK	1	1	1	N-Term(iTRAQ8plex); K10(iTRAQ8plex)	0.967	1.728	1.351	2.79	-	0	1774.05594	3	592.0234985	-6.977764475	29.88221167
55743086	ALG3	dol-P-Man:Man(5)GlcNAc(2)-PP-Dol alpha-1,3-mannosyltransferase isoform d	NP_001006942.1	2.56	1	1	2	390	44.3	8.94	-	-	-																
														FLPEALFLHR	2	4	1	N-Term(iTRAQ8plex)	-	-	-	3.3	51	0	1546.90592	3	516.3068237	0.741248316	47.84344333
55749577	SLC25A4	ADP/ATP translocase 1	NP_001142.2	45.97	1	15	158	298	33.0	9.76	1.223	1.137	0.422																
														EQGFLSFWR	2	1	1	N-Term(iTRAQ8plex)	1.223	1.137	0.422	3.23	26	0	1473.77007	2	737.3886719	-6.218181658	69.96224167
														GMGGAFVLVLYDEIKK	10	3	3	N-Term(iTRAQ8plex); K15(iTRAQ8plex); K16(iTRAQ8plex)	-	-	-	4.73	31	1	2652.56606	4	667.8931274	-0.174781906	56.38034
														YFPTQALNFAFKDK	3	6	3	N-Term(iTRAQ8plex); K12(iTRAQ8plex); K14(iTRAQ8plex)	-	-	-	5.78	33	1	2602.4811	4	651.3757324	0.552744648	44.38119833
														YFPTQALNFAFK	30	6	3	N-Term(iTRAQ8plex); K12(iTRAQ8plex)	-	-	-	5.17	57	0	2055.14353	2	1028.076172	-3.558955578	40.728655
														AFFKGAWSNVLR	17	3	3	N-Term(iTRAQ8plex); K4(iTRAQ8plex)	-	-	-	4.99	36	1	2004.15639	4	501.7945557	-3.781403376	40.955375
														GMGGAFVLVLYDELKK	7	3	3	N-Term(iTRAQ8plex); M2(Oxidation); K15(iTRAQ8plex); K16(iTRAQ8plex)	-	-	-	4.6	32	1	2668.55068	4	663.8932495	-1.908778683	55.97575833
														LLLQVQHASK	42	3	3	N-Term(iTRAQ8plex); K10(iTRAQ8plex)	-	-	-	4.59	48	0	1745.0822	2	873.0447388	-4.114637656	25.33012667
														GMGGAFVLVLYDELK	8	3	3	N-Term(iTRAQ8plex); K15(iTRAQ8plex)	-	-	-	4.59	41	0	2220.24057	3	740.751709	-6.9035748	81.47813167
														TAVAPIER	6	6	3	N-Term(iTRAQ8plex)	-	-	-	3.35	63	0	1160.69382	3	387.569458	-0.241688684	16.48437333
														LAADVGK	2	3	3	N-Term(iTRAQ8plex); K7(iTRAQ8plex)	-	-	-	2.94	-	0	1281.80051	3	427.9383545	1.426841812	20.00134333
														GNLANVIR	5	6	3	N-Term(iTRAQ8plex)	-	-	-	2.75	37	0	1160.69975	2	580.8535156	-4.804575841	17.04167167
														GAWSNVLR	2	3	3	N-Term(iTRAQ8plex)	-	-	-	2.73	30	0	1206.68608	2	603.8466797	-2.982171628	22.68380667
														GADIMYTGTVDCWR	2	2	2	N-Term(iTRAQ8plex); C12(Methylthio)	-	-	-	5.12	55	0	1937.89519	2	969.4512329	4.647774742	41.15287167
														YFAGNLASGGAAGATSLCFVYPLDFAR	18	2	2	N-Term(iTRAQ8plex); C18(Methylthio)	-	-	-	4.94	61	0	3089.52536	3	1131.912842	0.54802354	62.85787
														AAYFGVYDTAK	4	2	2	N-Term(iTRAQ8plex); K11(iTRAQ8plex)	-	-	-	4.63	54	0	1813.98674	2	907.4970093	-4.260982252	40.51684667
														KGADIMYTGTVDCWR	6	2	2	N-Term(iTRAQ8plex); K1(iTRAQ8plex); C13(Methylthio)	-	-	-	3.77	34	1	2370.17484	3	790.7297974	-4.925147278	36.04928
														YFAGNLASGGAAGATSLCFVYPLDFAR	8	2	2	N-Term(iTRAQ8plex); Y1(iTRAQ8plex); C18(Methylthio)	-	-	-	-	67	0	3393.72397	3	1131.912842	0.54802354	62.85787
														KGADIMYTGTVDCWR	1	2	2	K1(iTRAQ8plex); N-Term(iTRAQ8plex); M6(Oxidation); C13(Methylthio)	-	-	-	3.48	-	1	2386.17716	3	790.7297974	-4.925147278	36.04928
55770836	SCCPDH	saccharopine dehydrogenase-like oxidoreductase	NP_057086.2	6.76	1	1	2	429	47.1	9.14	2.482	1.510	0.775																
														GPEAGYVATPIAMVQAAMTLLSDASHLPK	2	1	1	N-Term(iTRAQ8plex); K29(iTRAQ8plex)	2.482	1.51	0.775	7.31	76	0	3547.89511	3	1183.303223	-4.590491185	86.37695167
55770900	BRIX1	ribosome biogenesis protein BRX1 homolog	NP_060791.3	15.58	5	5	24	353	41.4	9.92	1.609	0.761	0.666																
														LFVINEVCEMK	6	1	1	N-Term(iTRAQ8plex); C8(Methylthio); K11(iTRAQ8plex)	1.382	0.58	0.553	4.86	48	0	1979.04837	3	660.3600464	1.573272851	54.20869667
														FLVQNIHTLAELK	11	1	1	N-Term(iTRAQ8plex); K13(iTRAQ8plex)	1.655	0.887	0.749	4.6	32	0	2134.28208	4	534.3237305	-5.331700541	46.03284167
														SQPFVDHVFTFTILDNR	4	1	1	N-Term(iTRAQ8plex)	1.465	0.761	0.671	4.53	45	0	2340.23417	3	780.7495728	2.189401298	49.58609
														FVLNLIK	2	1	1	N-Term(iTRAQ8plex); K7(iTRAQ8plex)	1.662	0.606	0.646	2.99	30	0	1454.95073	2	727.9790039	-3.2854085	68.07807167
														ILIFSSR	1	1	1	N-Term(iTRAQ8plex)	1.995	0.667	0.652	-	24	0	1139.70342	2	570.3553467	-4.908786394	38.395725
55770906	XAB2	pre-mRNA-splicing factor SYF1	NP_064581.2	6.32	3	3	3	855	99.9	6.23	1.034	0.982	0.893																
														LSRPERPDLVFEEEDLPYEEEIMR	1	1	1	N-Term(iTRAQ8plex)	-	-	-	3.45	-	0	3295.64174	4	824.6658936	-1.273488314	42.730215
														SHPLPETAVR	1	1	1	N-Term(iTRAQ8plex)	0.79	1.018	0.894	3.01	-	0	1410.79639	3	470.9369812	-3.048594761	13.42295833
														METASELGREEEDDVDLELR	1	1	1	N-Term(iTRAQ8plex)	1.353	0.948	0.892	2.77	-	1	2640.25614	3	880.756897	-1.963306498	39.89787333
55953087	GTPBP4	nucleolar GTP-binding protein 1	NP_036473.2	6.15	3	3	6	634	73.9	9.50	1.406	1.047	1.268																
														WQVVDTPGILDHPLEDR	4	2	1	N-Term(iTRAQ8plex)	1.649	1.038	1.234	7.37	69	0	2294.21262	3	765.4090576	1.873541069	41.45743833
														TLLLCGYPNVGK	1	2	1	N-Term(iTRAQ8plex); C5(Methylthio); K12(iTRAQ8plex)	1.511	0.992	1.082	3.38	-	0	1932.07157	3	644.6953735	-9.909024518	34.74344167
														YWDLMNLSEK	1	1	1	N-Term(iTRAQ8plex); K10(iTRAQ8plex)	1.308	1.104	1.486	3.3	-	0	1907.0004	3	636.3383179	-9.92183154	68.829525
55956779	SCP2	non-specific lipid-transfer protein isoform 4	NP_001007101.1	32.86	4	4	18	140	15.1	8.92	0.811	0.748	0.404																
														ADCTITMADSDFLALMTGK	3	6	1	N-Term(iTRAQ8plex); C3(Methylthio); K19(iTRAQ8plex)	1.06	0.919	0.469	7	49	0	2658.33304	3	886.7825317	8.358495528	64.72280333
														EATWVVDVK	6	6	1	N-Term(iTRAQ8plex); K9(iTRAQ8plex)	0.795	0.677	0.438	4.09	46	0	1654.96074	2	827.9840088	-1.039506154	38.88430167
														ITGNMGLAMK	3	6	1	N-Term(iTRAQ8plex); K10(iTRAQ8plex)	0.957	0.902	0.46	4.03	50	0	1643.94097	2	822.4741211	-1.445144655	37.30372
														IGGIFAFK	6	6	1	N-Term(iTRAQ8plex); K8(iTRAQ8plex)	0.833	0.779	0.325	3.35	31	0	1460.91062	3	487.6434021	4.847472757	39.02254667
55956788	NCL	nucleolin	NP_005372.2	30.00	21	21	163	710	76.6	4.70	1.141	1.371	1.164																
														TLVLSNLSYSATEETLQEVFEK	30	1	1	N-Term(iTRAQ8plex); K22(iTRAQ8plex)	1.07	1.137	1.193	7.21	79	0	3109.70229	3	1037.227661	-2.611581185	65.65155
														VEGTEPTTAFNLFVGNLNFNK	10	1	1	N-Term(iTRAQ8plex); K21(iTRAQ8plex)	0.977	0.802	0.997	6.96	60	0	2920.5604	3	974.1916504	-2.110340923	48.17518833
														VTQDELKEVFEDAAEIR	5	1	1	N-Term(iTRAQ8plex); K7(iTRAQ8plex)	1.804	2.655	1.556	5.77	63	1	2600.39141	2	1300.699341	-4.401752503	52.03623833
														FGYVDFESAEDLEK	18	1	1	N-Term(iTRAQ8plex); K14(iTRAQ8plex)	1.209	1.623	1.405	5.76	81	0	2257.12627	2	1129.066772	-9.850134854	66.95845167
														GFGFVDFNSEEDAK	15	1	1	N-Term(iTRAQ8plex); K14(iTRAQ8plex)	1.246	1.298	1.262	5.47	82	0	2170.0728	2	1085.542969	-5.838691578	55.72232833
														GYAFIEFASFEDAK	21	1	1	N-Term(iTRAQ8plex); K14(iTRAQ8plex)	1.158	1.316	1.144	5.46	54	0	2203.14043	2	1102.073853	-5.79302241	73.70045667
														KFGYVDFESAEDLEK	6	1	1	N-Term(iTRAQ8plex); K1(iTRAQ8plex); K15(iTRAQ8plex)	1.043	1.388	1.136	5.4	56	1	2689.44358	3	897.15271	-1.95381202	34.21123
														QKVEGTEPTTAFNLFVGNLNFNK	1	1	1	N-Term(iTRAQ8plex); K2(iTRAQ8plex); K23(iTRAQ8plex)	-	-	-	5	-	1	3480.92714	4	870.9872437	0.48074243	50.66709667
														SISLYYTGEK	6	1	1	N-Term(iTRAQ8plex); K10(iTRAQ8plex)	1.067	1.452	1.191	4.72	46	0	1768.98332	2	884.9953003	-6.103694657	35.97253333
														GLSEDTTEETLKESFDGSVR	5	1	1	N-Term(iTRAQ8plex); K12(iTRAQ8plex)	1.32	1.691	1.755	4.56	33	1	2808.43349	4	702.8638306	-0.893350112	41.80986667
														EVFEDAAEIR	9	1	1	N-Term(iTRAQ8plex)	1.131	1.158	1.164	3.7	55	0	1482.77433	3	494.9296265	0.070708354	31.66902167
														NSTWSGESK	2	1	1	N-Term(iTRAQ8plex); K9(iTRAQ8plex)	1.144	0.998	1.086	3.55	35	0	1603.85088	2	802.4290771	-1.71064727	13.47191667
														GIAYIEFK	11	1	1	N-Term(iTRAQ8plex); K8(iTRAQ8plex)	1.177	1.668	1.406	3.49	43	0	1548.91191	2	774.9595947	-8.191858278	46.07329833
														ALELTGLK	2	1	1	N-Term(iTRAQ8plex); K8(iTRAQ8plex)	1.425	1.592	1.531	3.48	42	0	1452.93169	2	726.9694824	4.870789045	27.62293167

														NDLAVVDVR	13	1	1	N-Term(iTRAQ8plex)	1.078	1.141	1.09	3.37	52	0	1304.74321	3	435.5869446	-1.012311578	26.73328667
														EAMEDGEIDGNK	2	1	1	N-Term(iTRAQ8plex); M3(Oxidation); K12(iTRAQ8plex)	0.949	-	0.895	3.29	51	0	1931.93497	3	644.6498413	-6.592159887	16.87040333
														ESFDGSVR	2	1	1	N-Term(iTRAQ8plex)	1.088	1.143	1.09	2.89	26	0	1200.61638	2	600.8118286	0.116029204	19.060145
														QGTEIDGR	1	1	1	N-Term(iTRAQ8plex)	1.199	1.199	1.155	-	25	0	1179.622	2	590.3146362	-4.361955847	10.19289833
														VTLDWAKPK	1	1	1	N-Term(iTRAQ8plex); K7(iTRAQ8plex); K9(iTRAQ8plex)	0.753	0.939	0.536	-	25	0	1970.22634	4	493.3120422	3.120334548	23.45511667
														LELQGPR	1	1	1	N-Term(iTRAQ8plex)	-	-	-	-	24	0	1116.66181	3	372.8921204	-5.438948456	20.40588333
														GIAYIEFK	1	1	1	N-Term(iTRAQ8plex); Y4(iTRAQ8plex); K8(iTRAQ8plex)	1.141	1.467	1.356	-	23	0	1853.12954	2	774.9595947	-8.191858278	46.07329833
														EALNSCNK	1	1	1	N-Term(iTRAQ8plex); C6(Methylthio); K8(iTRAQ8plex)	1.607	1.251	1.229	-	20	0	1532.78968	3	511.6014099	-8.119713919	14.71029
55956801	SMARCB1	SWI/SNF-related matrix-associated actin-dependent regulator of chromatin subfamily B member 1 isoform b	NP_001007469.1	13.30	2	2	5	376	43.1	5.64	0.452	1.352	2.518																
														TFPLCFDDHDPAVIHENASQPEVLVPIR	3	2	1	N-Term(iTRAQ8plex); C5(Methylthio)	-	-	-	5.22	23	0	3509.75576	4	878.194397	1.634219308	48.32925333
														NTGDADQWCPLLETLTDAEMEK	2	4	1	N-Term(iTRAQ8plex); C9(Methylthio); K22(iTRAQ8plex)	0.452	1.352	2.518	3.25	29	0	3134.50412	3	1045.506226	3.181111723	64.65669167
55956899	KRT9	keratin, type I cytoskeletal 9	NP_000217.2	9.47	3	4	51	623	62.0	5.24	0.722	0.793	0.726																
														SDLEMQYETLQEELMALK	2	2	1	N-Term(iTRAQ8plex); K18(iTRAQ8plex)	0.743	1.194	0.789	5.9	42	0	2779.452	3	927.1555176	5.946562264	57.08996667
														GGSGGSYGGGGSGGGYGGGSGSR	2	2	1	N-Term(iTRAQ8plex)	-	0.793	0.57	5.3	60	0	2095.92899	3	699.3145142	-1.990558627	12.93665833
														QGVDADINGLR	2	2	1	N-Term(iTRAQ8plex)	0.701	0.595	0.726	3.12	38	0	1461.78996	2	731.3986206	-4.369101729	17.68495167
														LASYLDK	45	13	7	N-Term(iTRAQ8plex); K7(iTRAQ8plex)	-	-	-	3.56	36	0	1417.84687	3	473.2871399	-2.979780374	31.776525
55956921	HNRNPAB	heterogeneous nuclear ribonucleoprotein A/B isoform b	NP_004490.2	27.37	6	7	67	285	30.6	7.91	1.041	0.660	0.778																
														EYFGEFGEIEAIELPMDPK	6	2	1	N-Term(iTRAQ8plex); M16(Oxidation); K19(iTRAQ8plex)	0.936	0.923	1.065	6.67	46	0	2838.43691	2	1411.734253	6.830187489	52.97084833
														EYFGEFGEIEAIELPMDPK	12	2	1	N-Term(iTRAQ8plex); K19(iTRAQ8plex)	0.919	0.679	0.776	6.58	97	0	2822.46823	2	1411.734253	6.830187489	52.97084833
														IFVGGLNPEATEEK	8	2	1	N-Term(iTRAQ8plex); K14(iTRAQ8plex)	1.192	1.087	1.087	5.61	60	0	2112.16728	2	1056.58728	-5.902644748	27.11561667
														MFVGGLSWDTSK	2	2	1	N-Term(iTRAQ8plex); K12(iTRAQ8plex)	1.053	0.514	0.591	4.08	41	0	1936.03269	3	646.0157471	-6.819791393	64.47358
														EVYQQQQYGSGGR	4	2	1	N-Term(iTRAQ8plex)	1.022	0.56	0.714	4	44	0	1803.88591	2	902.4465942	-3.785961957	13.876935
														IREYFGEFGEIEAIELPMDPK	3	2	1	N-Term(iTRAQ8plex); K21(iTRAQ8plex)	-	-	-	3.64	30	1	3091.62221	3	1031.219238	5.181805568	59.71715167
														GFGFILFK	6	2	1	N-Term(iTRAQ8plex); K8(iTRAQ8plex)	1.118	0.62	0.778	3.53	42	0	1536.92546	2	768.9663696	-9.374990875	71.25458333
														FGEVVDCTIK	26	4	3	N-Term(iTRAQ8plex); C7(Methylthio); K10(iTRAQ8plex)	-	-	-	4.38	53	0	1764.93157	2	882.9761353	-1.970833198	34.73064167
56118210	NTRK1	high affinity nerve growth factor receptor isoform 3	NP_001007793.1	1.18	1	1	1	760	83.9	6.73	1.759	3.897	2.129																
														DIYSTDYYR	1	3	1	N-Term(iTRAQ8plex)	1.759	3.897	2.129	-	21	0	1499.72925	3	500.5812683	-1.806738354	23.65779833
56118310	NUCKS1	nuclear ubiquitous casein and cyclin-dependent kinase substrate 1	NP_073568.2	9.88	1	1	2	243	27.3	5.08	1.293	4.483	1.698																
														EMLMEDVGSEEEQEEEDEAPFQEK	2	1	1	N-Term(iTRAQ8plex); M4(Oxidation); K24(iTRAQ8plex)	1.293	4.483	1.698	6.7	80	0	3481.58493	3	1161.199829	6.930106588	33.8011
56243533	SDF2L1	stromal cell-derived factor 2-like protein 1 precursor	NP_071327.2	9.05	1	1	2	221	23.6	7.03	1.130	0.849	1.154																
														AMEGIFIKPSVEPSAGHDEL	2	1	1	N-Term(iTRAQ8plex); K8(iTRAQ8plex)	1.13	0.849	1.154	3.33	-	0	2735.44155	4	684.6158447	-4.376724392	38.00117833
56243583	WDR18	WD repeat-containing protein 18	NP_077005.2	13.43	4	4	6	432	47.4	6.70	1.166	0.925	0.785																
														VTELEDEVR	2	2	1	N-Term(iTRAQ8plex)	1.117	0.925	0.944	3.64	43	0	1393.75005	2	697.3786621	1.712142542	30.93504333
														GPVTNAAILLAPVSMLSSDFRPSLPLPHFNK	1	2	1	N-Term(iTRAQ8plex); K31(iTRAQ8plex)	-	-	-	3.32	-	0	3898.19253	4	975.3035889	1.30170296	65.61999667
														NYISAWELQR	1	2	1	N-Term(iTRAQ8plex)	1.166	1.018	0.785	3.09	-	0	1583.86345	2	792.4353638	9.527095946	33.271875
														DLFDFSTR	2	2	1	N-Term(iTRAQ8plex)	1.346	0.483	0.631	2.7	24	0	1304.68181	2	652.8445435	2.275646092	34.16630667
56549119	DNM2	dynamin-2 isoform 3	NP_004936.2	1.73	1	1	6	866	97.6	7.64	1.159	1.271	1.992																
														ALLQMVQQFGVDFEK	6	5	1	N-Term(iTRAQ8plex); K15(iTRAQ8plex)	1.159	1.271	1.992	4.85	54	0	2361.31821	3	787.7775879	3.590842538	63.62794667
56549687	GTPBP8	GTP-binding protein 8 isoform 2	NP_612494.1	5.58	1	1	2	251	28.6	9.13	1.561	1.338	1.242																
														APEDFVDMVETYLK	2	2	1	N-Term(iTRAQ8plex); K14(iTRAQ8plex)	1.561	1.338	1.242	3.16	35	0	2265.19571	3	755.7367554	1.042542712	58.1341
56550081	BUB3	mitotic checkpoint protein BUB3 isoform b	NP_001007794.1	6.13	1	1	8	326	36.9	6.84	0.938	0.582	0.850																
														MHDLNTDQENLVGTHDAPIR	6	2	1	N-Term(iTRAQ8plex)	0.731	0.522	0.869	6.27	43	0	2580.2717	3	860.762085	-2.394967624	23.76879333
														MHDLNTDQENLVGTHDAPIR	2	2	1	N-Term(iTRAQ8plex); M1(Oxidation)	3.281	1.271	0.831	3.96	30	0	2596.26357	3	860.762085	-2.394967624	23.76879333
56605994	CISD2	CDGSH iron-sulfur domain-containing protein 2	NP_001008389.1	20.00	2	2	14	135	15.3	9.61	1.127	0.994	0.950																
														VVNEINIEDLCLTK	6	1	1	N-Term(iTRAQ8plex); C11(Methylthio); K14(iTRAQ8plex)	1.147	0.978	0.966	6.38	82	0	2257.24346	2	1129.125366	1.86222796	50.42731
														RLPVPESITGFAR	8	1	1	N-Term(iTRAQ8plex)	1.107	1.011	0.946	4.04	61	1	1747.012	3	583.0088501	-2.784329495	30.64349667
56682959	FTH1	ferritin heavy chain	NP_002023.2	21.86	3	3	7	183	21.2	5.55	0.598	0.428	0.597																
														KPDCDDWESGLNAMECALHLEK	4	1	1	N-Term(iTRAQ8plex); K1(iTRAQ8plex); C4(Methylthio); C16(Methylthio); K22(iTRAQ8plex)	0.323	0.231	0.515	5.97	51	0	3508.67905	3	1170.231201	-0.473977343	54.600255
														NVNQSLLELHK	2	1	1	N-Term(iTRAQ8plex); K11(iTRAQ8plex)	0.598	0.467	0.645	4.55	32	0	1903.1138	3	635.0427856	-4.381039321	24.04529333
														IFLQDIK	1	1	1	N-Term(iTRAQ8plex); K7(iTRAQ8plex)	0.644	0.428	0.597	2.91	-	0	1484.92583	2	742.9665527	-2.60110719	48.494275
56699409	RBMX	RNA-binding motif protein, X chromosome isoform 1	NP_002130.2	21.74	8	8	56	391	42.3	10.05	1.218	0.648	0.677																
														GFAFVTFESPADAK	14	5	1	N-Term(iTRAQ8plex); K14(iTRAQ8plex)	1.494	0.823	0.737	5.66	83	0	2095.12676	2	1048.064819	-4.634215309	33.22619833
														GGHMDDGGYSMNFNMSSSR	2	2	1	N-Term(iTRAQ8plex)	0.308	0.805	0.656	5.13	50	0	2353.98172	3	785.3320923	-3.614171872	24.04544333
														LFIGGLNTETNEK	10	3	1	N-Term(iTRAQ8plex); K13(iTRAQ8plex)	1.226	0.741	0.785	5.05	59	0	2044.14787	2	1022.577576	-2.762301535	45.82673667
														IVEVLLMK	10	3	1	N-Term(iTRAQ8plex); K8(iTRAQ8plex)	1.127	0.551	0.625	3.94	38	0	1552.99407	3	518.3372192	0.916398904	44.282085
														ALEAVFGK	13	2	1	N-Term(iTRAQ8plex); K8(iTRAQ8plex)	1.182	0.585	0.718	3.83	34	0	1442.88205	3	481.6322021	-0.483826579	53.30571667
														RGPPPPPR	5	1	1	N-Term(iTRAQ8plex)	1.204	0.694	0.667	2.89	42	1	1177.70615	3	393.2402344	-3.915598981	9.266723333
														DYAPPPR	1	2	1	N-Term(iTRAQ8plex)	1.21	0.628	0.717	-	21	0	1119.60991	2	560.3085938	-0.098623636	12.29901333
														YDDYSSSR	1	2	1	N-Term(iTRAQ8plex)	1.242	0.647	0.59	-	20	0	1296.60063	2	648.8039551	-0.245074498	13.044575
57165424	CUL4A	cullin-4A isoform 1	NP_001008895.1	3.82	2	3	5	759	87.6	8.13	1.440	2.144	1.671																
														ETVEEQVSTTER	2	3	1	N-Term(iTRAQ8plex)	1.644	2.568	1.901	4.96	60	0	1711.86687	2	856.4370728	0.968292478	21.99006833
														AVQSSTSIR	1	1	1	N-Term(iTRAQ8plex)	1.261	1.79	1.47	-	21	0	1252.71977	2	626.8635254	2.788222332	15.77128333
														SIFLFLDR	2	11	2	N-Term(iTRAQ8plex)	-	-	-	3.12	26	0	1314.77239	2	657.8898315	0.026746787	47.66894167
57222563	CKAP5	cytoskeleton-associated protein 5 isoform b	NP_055571.2	1.83	3	3	4	1972	218.4	8.06	0.597	0.820	1.068																
														LDDIFEPVLIPEPK	2	2	1	N-Term(iTRAQ8plex); K14(iTRAQ8plex)	0.202	0.697	0.66	3.28	32	0	2233.30167	3	745.1054077	3.406881674	51.104385
														TAASGAAGDK	1	2	1	N-Term(iTRAQ8plex); K10(iTRAQ8plex)	0.909	1.371	1.338	3.11	-	0	1456.80878	3	486.2744446	-8.807476957	26.77443333
														IEDLEEGQQVIR	1	2	1	N-Term(iTRAQ8plex)	0.597	0.82	1.068	3.04	-	0	1732.94878	2	866.9780273	6.028318686	33.48846167
57634534	NUP205	nuclear pore complex protein Nup205	NP_055950.1	2.68	3	3	6	2012	227.8	6.21	1.393	0.800	0.934																
														CQDVSAGSLQELALLTGIISK	2	2	1	N-Term(iTRAQ8plex); C1(Methylthio); K21(iTRAQ8plex)	1.393	0.695	0.934	6.3	58	0	2800.55436	3	934.1896362	4.789459409	64.98951833
														ASTEGVAIQGQQGTR	2	1	1	N-Term(iTRAQ8plex)	1.421	0.8	0.782	5.84	68	0	1806.96384	3	602.9927979	1.465997619	17.97185333
														QSLAESLFAWACQSPLGK	2	1	1	N-Term(iTRAQ8plex); C12(Methylthio); K18(iTRAQ8plex)	0.856	2.097	1.945	3.42	27	0	2590.37912	3	864.1312256	6.671304465	63.35481667
57863257	TCP1	T-complex protein 1 subunit alpha isoform a	NP_110379.2	32.55	15	15	60	556	60.3	6.11	1.053	2.509	1.861																
														IACLDFSLQK	2	2	1	N-Term(iTRAQ8plex); C3(Methylthio); K10(iTRAQ8plex)	1.086	3.773	2.244	5.25	42	0	1791.9854	2	896.4963379	-5.777468251	72.773195
														QAGVFEPTIVK	2	2	1	N-Term(iTRAQ8plex); K11(iTRAQ8plex)	1.169	3.331	2.265	5.04	43	0	1797.06059	2	899.0339355	-6.948472572	22.291415
														LGVQVVITDPEKLDQIR	5	2	1	N-Term(iTRAQ8plex); K12(iTRAQ8plex)	0.641	2.11	1.188	4.9	33	1	2531.48966	3	844.5014038	-4.784978749	39.76402167
														YINENLIVNTDELGR	2	1	1	N-Term(iTRAQ8plex)	1.41	3.833	2.29	4.88	79	0	2067.09673	2	1034.052002	-2.759432432	38.12198
														ILATGANVILTTGGIDDMCLK	2	2	1	N-Term(iTRAQ8plex); C19(Methylthio); K21(iTRAQ8plex)	-	1.661	1.908	4.76	54	0	2773.52121	3	925.1785889	3.212901895	61.71169333
														SQNVMAAASIANIVK	1	1	1	N-Term(iTRAQ8plex); K15(iTRAQ8plex)	0.977	1.049	0.745	4.42	-	0	2125.21744	3	709.0773315	-4.023080616	60.31046333
														AFHNEAQVNPER	8	2	1	N-Term(iTRAQ8plex)	0.97	1.907	1.313	4.38	48	0	1715.87015	3	572.6282349	-3.828937843	14.633905
														FATEAAITILR	8	2	1	N-Term(iTRAQ8plex)	1.203	3.461	2.397	4.35	60	0	1509.8873	3	503.9664307	-6.313434111	43.40886833
														ICDDELILIK	15	2	1	N-Term(iTRAQ8plex); C2(Methylthio); K10(iTRAQ8plex)	0.963	2.955	1.866	4.31	45	0	1829.02019	2	915.0137329	-9.350962766	40.42902833
														LLEVEHPAAK	2	1	1	N-Term(iTRAQ8plex); K10(iTRAQ8plex)	1.218	3.005	2.003	3.87	41	0	1715.01885	2	858.0130615	-7.209759327	23.62398333
														EQLAIAEFAR	3	2	1	N-Term(iTRAQ8plex)	1.335	2.368	1.478	3.77	23	0	1451.81902	2	726.413147	2.063635534	33.92861
														YFVEAGAMAVR	1	2	1	N-Term(iTRAQ8plex)	0.945	2.228	1.709	3.57	-	0	1517.81352	2	759.4104004	3.091856263	33.577985
														TSASIILR	5	2	1	N-Term(iTRAQ8plex)	1.1	2.509	1.874	3.33	41	0	1164.73084	3	388.9151306	4.676870847	26.14608
														YPVNSVNILK	2	2	1	N-Term(iTRAQ8plex); K10(iTRAQ8plex)	1.361	1.829	2.141	3.05	40	0	1755.0582	3	585.690918	-2.444730179	40.86746833
														SLHDALCVVK	1	2	1	N-Term(iTRAQ8plex); C7(Methylthio); K10(iTRAQ8plex)	1.745	2.457	1.94	-	31	0	1738.96506	3	580.3265381	-8.839333539	29.59879833
														YINENLIVNTDELGR	1	1	1	N-Term(iTRAQ8plex); Y1(iTRAQ8plex)	1.03	1.477	1.092	-	31	0	2371.29728	2	1034.052002	-2.759432432	38.12198
57863269	RRP1B	ribosomal RNA processing protein 1 homolog B	NP_055871.1	4.35	2	2	5	758	84.4	9.76	1.933	0.607	0.381																
														GRDDCGTFEDTGPLLQFDYK	3	1	1	N-Term(iTRAQ8plex); C5(Methylthio); K20(iTRAQ8plex)	1.933	0.607	0.381	6.22	47	1	2931.41617	3	977.8102417	1.633868197	43.57577167
														FHFIDIYLDELSK	2	1	1	N-Term(iTRAQ8plex); K13(iTRAQ8plex)	-	-	-	3.03	27	0	2248.2455	4	562.8168335	-0.848303987	60.27210667
58197556	IBA57	putative transferase CAF17, mitochondrial isoform 1 precursor	NP_001010867.1	2.81	1	1	1	356	38.1	9.83	0.804	0.495	0.524																
														VTVEPHPELR	1	1	1	N-Term(iTRAQ8plex)	0.804	0.495	0.524	-	30	0	1480.84064	3	494.2850647	-1.296870271	18.289695
58219792	RAP1B	ras-related protein Rap-1b isoform 1 precursor	NP_001010942.1	60.87	3	9	70	184	20.8	5.78	1.073	0.649	1.033																
														VKDTDDVPMILVGNK	2	3	1	N-Term(iTRAQ8plex); K2(iTRAQ8plex); K15(iTRAQ8plex)	1.117	1.111	1.067	5.93	41	1	2556.47037	3	852.8283081	-5.120432351	32.90902833
														KQVEVDAQQCMLEILDTAGTEQFTAMR	4	1	1	N-Term(iTRAQ8plex); K1(iTRAQ8plex); C10(Methylthio)	-	-	2.136	5.8	54	1	3709.87424	3	1237.296265	6.015943901	66.72058667
														QWNNCAFLESSAK	4	4	1	N-Term(iTRAQ8plex); C5(Methylthio); K13(iTRAQ8plex)	0.995	0.575	0.902	4.19	54	0	2152.06157	2	1076.534424	-7.444612164	63.49254833
														IQIWDTAGQER	14	3	9	N-Term(iTRAQ8plex)	-	-	-	3.41	41	0	1620.85991	2	810.9328003	-3.971558752	26.96297167
														SALTVQFVQGIFVEK	16	5	2	N-Term(iTRAQ8plex); K15(iTRAQ8plex)	-	-	-	5.35	81	0	2274.32915	2	1137.668213	-1.179309842	45.240645
														INVNEIFYDLVR	22	5	2	N-Term(iTRAQ8plex)	-	-	-	4.99	71	0	1798.99614	2	900.001709	-2.43466406	63.03819
														LVVLGSGGVGK	4	6	2	N-Term(iTRAQ8plex); K11(iTRAQ8plex)	-	-	-	4.62	52	0	1594.01255	3	532.0090332	-1.439725572	38.77282667
														SKINVNEIFYDLVR	11	5	2	N-Term(iTRAQ8plex); K2(iTRAQ8plex)	-	-	-	4.46	43	1	2318.33731	4	580.3383179	-0.62100509	54.51473
														YDPTIEDSYR	6	5	2	N-Term(iTRAQ8plex)	-	-	-	3.02	41	0	1562.76238	2	781.8848267	-1.04596816	23.15122
														CDLEDER	1	7	2	N-Term(iTRAQ8plex); C1(Methylthio)	-	-	-	-	35	0	1229.53996	2	615.2736206	-3.609243449	21.03867
58331150	MRPS30	28S ribosomal protein S30, mitochondrial	NP_057724.2	4.78	2	2	4	439	50.3	7.97	0.908	0.554	0.554																
														QNCADQIEVVFR	2	1	1	N-Term(iTRAQ8plex); C3(Methylthio)	0.669	0.554	0.454	4.67	38	0	1771.87749	2	886.4423828	0.094083818	40.50713833
														CPVHFYWVR	2	1	1	N-Term(iTRAQ8plex); C1(Methylthio)	0.997	0.543	0.561	3.25	-	0	1556.77622	3	519.5969238	-2.951012208	40.39410833
58530840	DSP	desmoplakin isoform I	NP_004406.2	15.12	31	31	77	2871	331.6	6.81	0.995	0.945	0.458																
														TMIQSPSGVILQEAADVHAR	2	3	1	N-Term(iTRAQ8plex)	1.461	0.87	0.692	6.26	75	0	2427.2966	3	809.7703857	-0.093652929	37.7209
														HQNQNTIQELLQNCSDCLMR	4	3	1	N-Term(iTRAQ8plex); C14(Methylthio); C17(Methylthio)	0.98	1.385	1.601	5.96	35	0	2784.27207	3	934.0933228	1.534394453	65.79036333
														TMIQSPSGVILQEAADVHAR	4	3	1	N-Term(iTRAQ8plex); M2(Oxidation)	0.964	0.818	0.89	5.62	59	0	2443.29178	3	809.7703857	-0.093652929	37.7209
														SAIYQLEEEYENLLK	6	3	1	N-Term(iTRAQ8plex); K15(iTRAQ8plex)	0.765	1.012	0.192	5.56	53	0	2450.31296	3	817.4494629	2.58092256	50.74740667
														IKDFLQGSSCIAGIYNETTK	4	3	1	N-Term(iTRAQ8plex); K2(iTRAQ8plex); C10(Methylthio); K20(iTRAQ8plex)	0.872	1.052	0.294	5.45	72	1	3146.69675	3	1049.570435	-0.820491858	56.06689
														[illegible]	[illegible]	[illegible]	[illegible]	N-Term(iTRAQ8plex)	-	-	-	[illegible]	[illegible]	[illegible]	[illegible]	[illegible]	[illegible]	[illegible]	[illegible]
														ALLQAVLQTEEMLK	[illegible]	[illegible]	[illegible]	N-Term(iTRAQ8plex); K14(iTRAQ8plex)	[illegible]	[illegible]	[illegible]	[illegible]	[illegible]	[illegible]	[illegible]	[illegible]	[illegible]	[illegible]	[illegible]

														LLEAQIATGGIIDPK	2	3	1	N-Term(iTRAQ8plex); K15(iTRAQ8plex)	1.243	1.063	0.902	5.07	54	0	2147.29264	3	716.43573	1.394961896	41.40560833
														GFFDPNTEENLTYLQLK	2	3	1	N-Term(iTRAQ8plex); K17(iTRAQ8plex)	1.342	0.72	0.492	5.04	46	0	2637.39603	3	879.8035278	-2.298120508	37.86914833
														GYFNEELSEILSDPSDDTK	2	3	1	N-Term(iTRAQ8plex); K19(iTRAQ8plex)	1.131	0.945	0.207	4.88	76	0	2767.3831	2	1384.19519	2.180015975	47.610745
														IGLVRPGTALELLEAQAATGFIVDPVSNLR	4	3	1	N-Term(iTRAQ8plex)	-	-	-	4.37	50	0	3424.98008	3	1142.331543	9.630525002	59.60567333
														HQNQNTIQELLQNCSDCLMR	2	3	1	N-Term(iTRAQ8plex); C14(Methylthio); C17(Methylthio); M19(Oxidation)	-	2.106	0.85	4.35	50	0	2800.26542	3	934.0933228	1.534394453	65.79036333
														SVEEVASEIQPFLR	2	3	1	N-Term(iTRAQ8plex)	1.026	1.054	0.477	4.13	50	0	1908.03557	2	954.5214233	-1.300416172	53.77976833
														TIADLELHYQEFIR	3	3	1	N-Term(iTRAQ8plex)	1.133	1.111	0.57	4.07	26	0	2052.10288	3	684.7088623	2.559491868	48.006365
														AFIGFEGVK	4	3	1	N-Term(iTRAQ8plex); K9(iTRAQ8plex)	0.921	0.729	0.264	4.04	48	0	1575.93694	2	788.4721069	0.898479599	45.2569
														LLEAQACTGGIIHPTTGQK	2	3	1	N-Term(iTRAQ8plex); C7(Methylthio); K19(iTRAQ8plex)	0.996	0.803	0.463	3.96	31	0	2592.38857	4	648.8526001	-8.209336215	39.56218333
														TVSVSEAIK	2	3	1	N-Term(iTRAQ8plex); K9(iTRAQ8plex)	1.08	0.949	0.355	3.89	35	0	1541.93596	2	771.4716187	0.03851307	29.72419
														IEPHTGLLLLSVQK	2	3	1	N-Term(iTRAQ8plex); K14(iTRAQ8plex)	0.442	0.421	0.555	3.79	29	0	2156.31882	3	719.444458	-3.487669119	33.70572167
														ELDECFAQANDQMEILDSLIR	2	3	1	N-Term(iTRAQ8plex); C5(Methylthio)	-	-	-	3.68	67	0	2803.33683	3	935.1171265	4.035849145	69.806735
														AELIVQPELK	2	3	1	N-Term(iTRAQ8plex); K10(iTRAQ8plex)	0.766	0.863	0.316	3.66	33	0	1748.07781	2	874.5425415	-0.014251189	25.83962
														LTEEETVCLDLDKVEAYR	1	3	1	N-Term(iTRAQ8plex); C8(Methylthio); K13(iTRAQ8plex)	-	-	-	3.52	-	1	2780.43764	4	695.8648682	2.492584672	52.56461333
														GLVGIEFK	4	3	1	N-Term(iTRAQ8plex); K8(iTRAQ8plex)	0.678	0.658	0.297	3.45	45	0	1470.91504	3	490.976532	0.674000358	41.76277833
														QQIQNDLNQWK	2	3	1	N-Term(iTRAQ8plex); K11(iTRAQ8plex)	0.855	0.934	0.905	3.34	33	0	2023.12149	3	675.0453491	1.667133334	45.26407167
														CIKDEETGLCLLPLK	1	3	1	N-Term(iTRAQ8plex); C1(Methylthio); K3(iTRAQ8plex); C10(Methylthio); K15(iTRAQ8plex)	0.725	0.484	0.299	3.3	-	1	2679.47548	4	670.6243286	1.261916052	46.97626833
														SGSLSLTQFADMISLK	2	3	1	N-Term(iTRAQ8plex); K16(iTRAQ8plex)	0.972	0.53	0.48	2.99	27	0	2306.28861	3	769.4343872	-0.018613848	57.86766
														ISTEEAIR	1	3	1	N-Term(iTRAQ8plex)	0.881	0.855	0.453	2.97	-	0	1222.69316	2	611.8502197	-1.086508795	18.70617833
														EHLMLEEELR	1	1	1	N-Term(iTRAQ8plex)	1.113	1.06	1.21	2.84	-	0	1602.84189	3	534.9521484	-2.775843033	23.37802667
														ENLGWQK	1	3	1	N-Term(iTRAQ8plex); K7(iTRAQ8plex)	1.251	1.306	0.78	2.83	-	0	1482.8537	3	494.9560852	0.8099688	19.65565333
														LWDLEK	1	3	1	N-Term(iTRAQ8plex); K6(iTRAQ8plex)	0.906	1.227	0.629	2.77	-	0	1411.84661	2	706.4269409	4.295024626	30.64806
														LISPESTVMLLEAQAATGGIIDPHR	1	3	1	N-Term(iTRAQ8plex)	-	-	-	-	33	0	2923.60288	3	975.2058105	5.581158353	53.87987
														LVFDGLR	1	3	1	N-Term(iTRAQ8plex)	0.926	0.974	0.233	-	22	0	1123.67668	2	562.34198	-0.923716829	33.83269167
														YEVTSGGGGTSR	1	3	1	N-Term(iTRAQ8plex)	0.995	0.809	0.109	-	20	0	1474.75334	2	737.8803101	6.361875516	16.09510333
														DLIDFDDR	1	3	1	N-Term(iTRAQ8plex)	0.892	0.96	0.547	-	19	0	1312.66338	2	656.8353271	-4.040352778	22.65842167
58530848	DSTN	destrin isoform b	NP_001011546.1	32.43	3	4	12	148	16.6	8.34	0.657	1.162	0.909																
														YALYDASFETK	2	2	1	N-Term(iTRAQ8plex); K11(iTRAQ8plex)	0.265	1.951	1.867	3.92	43	0	1916.00992	3	639.3414917	-8.475661925	45.22240667
														HECQANGPEDLNR	4	2	1	N-Term(iTRAQ8plex); C3(Methylthio)	0.981	0.935	0.669	3.87	17	0	1832.81394	3	611.6094971	-9.9433656	19.28333
														KEELMFFLWAPELAPLK	1	2	1	N-Term(iTRAQ8plex); K1(iTRAQ8plex); K17(iTRAQ8plex)	-	-	-	3.24	-	1	2974.71402	3	992.2428589	-3.481978356	64.72903
														MIYASSK	4	5	2	N-Term(iTRAQ8plex); M1(Oxidation); K7(iTRAQ8plex)	-	-	-	3.65	43	0	1423.79551	3	475.2700195	-8.435871728	15.36087
														MIYASSK	1	5	2	N-Term(iTRAQ8plex); K7(iTRAQ8plex)	-	-	-	-	26	0	1407.79914	3	475.2700195	-8.435871728	15.36087
58761500	OLA1	obg-like ATPase 1 isoform 1	NP_037473.3	25.76	7	7	19	396	44.7	7.81	1.260	3.910	2.777																
														IPAFLNVVDIAGLVK	11	1	1	N-Term(iTRAQ8plex); K15(iTRAQ8plex)	1.257	6.004	3.248	4.58	60	0	2177.35332	2	1089.180298	0.674113435	64.17118
														FYHDWNDKEIEVLNK	1	2	1	N-Term(iTRAQ8plex); K8(iTRAQ8plex); K15(iTRAQ8plex)	1.48	1.826	1.692	4.2	-	1	2862.53628	4	716.3895264	-6.66391948	36.42596
														NYIVEDGDIIFFK	2	2	1	N-Term(iTRAQ8plex); K13(iTRAQ8plex)	2.006	7.297	4.077	3.93	47	0	2181.18912	3	727.7345581	-7.381401933	69.38977333
														IGIVGLPNVGK	2	1	1	N-Term(iTRAQ8plex); K11(iTRAQ8plex)	0.882	4.981	2.777	3.3	31	0	1675.05649	3	559.0236816	-9.671093605	56.20942
														SWVIDQK	1	2	1	N-Term(iTRAQ8plex); K7(iTRAQ8plex)	1.263	2.099	1.473	3.05	-	0	1483.85979	2	742.4335327	-8.836744827	19.97941167
														YLEANMTQSALPK	1	2	1	N-Term(iTRAQ8plex); K13(iTRAQ8plex)	1.175	0.995	0.865	3.03	-	0	2074.1442	3	692.0529175	-1.023124908	42.74645167
														STFFNVLTNSQASAENFPFCTIDPNESR	1	1	1	N-Term(iTRAQ8plex); C20(Methylthio)	-	-	-	-	28	0	3486.61411	3	1162.876221	-3.084207282	65.08910333
59710109	TIMMDC1	complex I assembly factor TIMMDC1, mitochondrial precursor	NP_057673.2	7.72	1	1	2	285	32.2	8.50	-	-	-																
														TAATAGIIGWVYGGIPAFIHAK	2	1	1	N-Term(iTRAQ8plex); K22(iTRAQ8plex)	-	-	-	3.88	23	0	2822.63564	4	706.4143677	6.316622261	63.24237333
59814146	BTBD1	BTB/POZ domain-containing protein 1 isoform 2	NP_001011885.1	1.56	1	1	1	385	42.1	6.98	0.735	0.097	0.219																
														ALSLIR	1	2	1	N-Term(iTRAQ8plex)	0.735	0.097	0.219	-	24	0	976.64763	2	488.8274536	1.995915929	27.03367833
60279265	SEC61G	protein transport protein Sec61 subunit gamma	NP_001012474.1	19.12	1	1	2	68	7.7	9.99	0.809	0.945	0.969																
														LIHIPINNIIVGG	2	1	1	N-Term(iTRAQ8plex)	0.809	0.945	0.969	-	40	0	1677.03423	2	839.020752	-1.367899183	50.51203833
61742775	LNPEP	leucyl-cystinyl aminopeptidase isoform 2	NP_787116.2	3.07	2	2	4	1011	115.6	5.73	0.307	0.406	0.534																
														KYFAATQFEPLAAR	2	2	1	N-Term(iTRAQ8plex); K1(iTRAQ8plex)	0.582	0.469	0.52	3.84	30	1	2221.2526	3	741.0890503	-2.878407931	32.73227333
														EPCLHPLEPDEVEYEPR	2	2	1	N-Term(iTRAQ8plex); C3(Methylthio)	0.162	0.352	0.547	3.68	27	0	2402.12455	3	801.3796997	-2.708614305	33.77729667
														ISFLLR	1	6	1	N-Term(iTRAQ8plex)	0.54	0.296	0.424	-	21	0	1052.67449	2	526.8408813	-2.370366833	54.06817833
61743952	AGPAT5	1-acyl-sn-glycerol-3-phosphate acyltransferase epsilon	NP_060831.2	1.92	1	1	1	364	42.0	9.10	0.863	0.946	0.767																
														IHIHIDR	2	1	1	N-Term(iTRAQ8plex)	0.863	0.946	0.767	2.81	25	0	1207.71558	3	403.2433777	-4.737450982	15.47830667
61744483	SLC3A2	4F2 cell-surface antigen heavy chain isoform f	NP_001013269.1	46.31	19	20	137	529	57.9	5.35	1.326	0.558	0.403																
														SLLHGDFHAFSAGPGLFSYIR	12	4	1	N-Term(iTRAQ8plex)	1.682	1.491	0.804	7.09	49	0	2596.36245	4	649.8460693	0.379825148	51.40480833
														DDVAQTDLLQIDPNFGSKEDFDSLLQSAK	3	4	1	N-Term(iTRAQ8plex); K18(iTRAQ8plex); K29(iTRAQ8plex)	-	-	-	6.3	30	1	4122.15493	4	1031.294189	-2.368510565	53.74454833
														GQSEDPGSLLSLFR	10	4	1	N-Term(iTRAQ8plex)	1.307	0.673	0.578	6.29	63	0	1809.96897	3	603.9945068	2.25747396	45.60353333
														VAEDEAEAAAAAK	6	4	1	N-Term(iTRAQ8plex); K13(iTRAQ8plex)	1.153	0.505	0.279	5.58	77	0	1853.99639	2	927.5038452	-3.305010218	19.82711167
														IGDLQAFQGHGAGNLAGLK	9	4	1	N-Term(iTRAQ8plex); K19(iTRAQ8plex)	1.322	0.526	0.403	5.56	41	0	2475.38515	3	825.8009644	-1.83869477	35.7265
														IKVAEDEAEAAAAAK	4	4	1	N-Term(iTRAQ8plex); K2(iTRAQ8plex); K15(iTRAQ8plex)	2.443	0.976	0.513	5.48	29	1	2399.38906	4	600.6027222	-0.784578106	32.37699833
														EDFDSLLQSAK	10	4	1	N-Term(iTRAQ8plex); K11(iTRAQ8plex)	1.128	0.504	0.297	5.22	43	0	1861.00945	2	931.0089722	-3.054030888	53.56944333
														LLTSFLPAQLLR	4	4	1	N-Term(iTRAQ8plex)	1.289	0.481	0.278	4.06	37	0	1676.05779	2	838.5325317	9.853810944	64.269955
														GLVLGPIHK	19	4	1	N-Term(iTRAQ8plex); K9(iTRAQ8plex)	1.343	0.629	0.454	4.03	41	0	1541.99968	2	771.5028687	-0.207921941	21.41029333
														VILDLTPNYR	19	4	1	N-Term(iTRAQ8plex)	1.327	0.551	0.435	3.8	38	0	1507.88623	3	503.3002625	5.061922672	36.46547667
														VKDALEFWLQAGVDGFQVR	9	4	1	N-Term(iTRAQ8plex); K2(iTRAQ8plex)	0.843	-	-	3.77	44	1	2786.54953	3	929.5205688	0.754336049	67.70478833
														LDYLSSLK	6	4	1	N-Term(iTRAQ8plex); K8(iTRAQ8plex)	1.814	0.837	0.258	3.49	28	0	1546.92917	3	516.3155518	1.31274245	36.98552167
														ADLLLSTQPGREEGSPLELER	9	4	1	N-Term(iTRAQ8plex)	1.555	0.31	0.266	3.45	56	1	2614.39678	2	1307.698853	-3.30204769	34.46080667
														LKLEPHEGLLLR	3	4	1	N-Term(iTRAQ8plex); K2(iTRAQ8plex)	1.472	0.654	0.407	3.4	-	1	2026.25882	3	676.0911255	-2.234702252	31.943815
														EVELNELEPEKQPMNAASGAAMSLAGAEK	2	4	1	N-Term(iTRAQ8plex); K11(iTRAQ8plex); K29(iTRAQ8plex)	-	-	-	3.35	26	1	3927.05854	5	786.2175293	-0.573285476	41.14493167
														GRLDYLSSLK	1	4	1	N-Term(iTRAQ8plex); K10(iTRAQ8plex)	1.723	0.808	0.543	2.99	-	1	1760.04897	4	440.7677002	-2.096759131	25.56008333
														WCSWSLSQAR	6	4	1	N-Term(iTRAQ8plex); C2(Methylthio)	0.785	0.524	0.386	2.97	40	0	1573.74785	2	787.3775635	-5.008405173	68.34534333
														LEPHEGLLLR	3	4	1	N-Term(iTRAQ8plex)	1.413	0.419	0.473	2.93	36	0	1480.87378	3	494.2961121	-3.490080261	25.73462667
														WWHTGALYR	1	4	1	N-Term(iTRAQ8plex)	1.155	1.057	0.739	2.84	-	0	1493.78968	3	498.6014099	-3.919436427	33.97402
														DIENLK	1	4	2	N-Term(iTRAQ8plex); K6(iTRAQ8plex)	-	-	-	-	28	0	1339.80632	2	670.4067993	1.620169107	18.610635
61835164	FXR1	fragile X mental retardation syndrome-related protein 1 isoform b	NP_001013456.1	3.90	1	2	3	539	60.8	6.39	0.960	1.605	1.340																
														EISEGDEVEVYSR	2	6	1	N-Term(iTRAQ8plex)	0.96	1.605	1.34	4.24	31	0	1815.90264	2	908.4549561	6.170038611	27.24010333
														LQIDEQLR	1	16	3	N-Term(iTRAQ8plex)	-	-	-	3.01	-	0	1318.7614	2	659.8843384	-1.396129874	28.05334833
62198232	HMGCL	hydroxymethylglutaryl-CoA lyase, mitochondrial isoform 1 precursor	NP_000182.2	6.46	2	2	8	325	34.3	8.54	0.373	0.300	0.479																
														GFEAAVAAGAK	4	1	1	N-Term(iTRAQ8plex); K11(iTRAQ8plex)	0.4	0.3	0.479	4.39	66	0	1599.92363	2	800.4654541	-4.925058121	32.45303
														AAQSANISVR	4	1	1	N-Term(iTRAQ8plex)	0.311	0.28	0.497	3.59	62	0	1320.75217	3	440.9222412	-1.189411021	17.78478
62241042	EPRS	bifunctional glutamate/proline--tRNA ligase	NP_004437.2	21.03	22	22	76	1512	170.5	7.33	1.045	2.134	2.550																
														LSSCDSFTSTINELNHCLSLR	12	1	1	N-Term(iTRAQ8plex); C4(Methylthio); C17(Methylthio)	1.26	1.31	3.165	7.23	85	0	2736.27408	3	912.7628784	-0.972598509	67.37727167
														GFFICDQPYEPVSPYSCK	6	2	1	N-Term(iTRAQ8plex); C5(Methylthio); C17(Methylthio); K18(iTRAQ8plex)	0.94	2.602	2.583	6.28	47	0	2780.32427	2	1390.665771	6.824041706	50.59676667
														ERPTPSLNNNCTTSEDSLVLYNR	2	2	1	N-Term(iTRAQ8plex); C11(Methylthio)	-	1.344	1.423	5.98	61	0	2973.42752	3	991.8140259	-2.461103632	32.69256
														WFGFLEAQQAFQSVGTK	3	1	1	N-Term(iTRAQ8plex); K17(iTRAQ8plex)	1.159	1.626	1.972	5.42	42	0	2552.37009	3	851.4615479	-2.249280124	51.32463167
														KLTWFVNEGLVDGWDDPR	7	1	1	N-Term(iTRAQ8plex); K1(iTRAQ8plex)	1.168	1.686	2.759	5.05	61	1	2755.47049	3	919.1616821	1.46321172	52.22168
														THVADFAPEVAWVTR	6	2	1	N-Term(iTRAQ8plex)	0.899	1.889	1.628	4.88	56	0	2003.05625	3	668.3569336	-4.505000177	39.13712
														DLPIKLNQWCNVVR	5	2	1	N-Term(iTRAQ8plex); K5(iTRAQ8plex); C10(Methylthio)	1.233	2.405	2.428	4.87	47	1	2352.31484	4	588.8341675	0.317874913	50.75412833
														TIGVMTMVHGDNMGLVLPPR	2	2	1	N-Term(iTRAQ8plex)	2.014	2.134	2.422	4.83	42	0	2442.29294	3	814.769165	-1.821826013	43.24102
														YNVYPTYDFACPIVDSIEGVTHALR	6	1	1	N-Term(iTRAQ8plex); C11(Methylthio)	-	-	-	4.66	50	0	3193.57138	3	1065.196045	2.980740973	68.65442333
														MFEIVFEDPKIPGEK	3	2	1	N-Term(iTRAQ8plex); K10(iTRAQ8plex); K15(iTRAQ8plex)	-	1.306	2.55	4.65	33	1	2691.51406	4	673.6339722	-2.02952985	51.64586833
														NSEPAGLETPEAK	2	2	1	N-Term(iTRAQ8plex); K13(iTRAQ8plex)	1.466	1.462	1.684	4.58	34	0	1951.04551	3	651.0200195	-7.065932707	15.17033833
														SLYDEVAAQGEVVR	2	2	1	N-Term(iTRAQ8plex)	0.886	1.658	1.804	4.54	44	0	1839.98088	2	920.4940796	2.957237077	32.10318333
														APKDQVDIAVQELLQLK	2	2	1	N-Term(iTRAQ8plex); K3(iTRAQ8plex); K17(iTRAQ8plex)	-	-	-	4.5	24	1	2820.6852	4	705.9267578	-3.910372556	56.66997
														INEAVECLLSLK	4	2	1	N-Term(iTRAQ8plex); C7(Methylthio); K12(iTRAQ8plex)	0.848	3.029	3.217	4.14	59	0	1986.12151	2	993.5643921	-0.460566138	62.69522333
														SLCIPFKPLCELQPGAK	2	2	1	N-Term(iTRAQ8plex); C3(Methylthio); K7(iTRAQ8plex); C10(Methylthio); K17(iTRAQ8plex)	1.332	4.292	5.805	3.67	-	0	2848.56131	3	950.1919556	-3.924735876	54.49291833
														DQVDIAVQELLQLK	2	2	1	N-Term(iTRAQ8plex); K14(iTRAQ8plex)	-	-	-	3.54	34	0	2220.30338	3	740.772644	-1.185613245	52.399505
														VAVQGDVVR	2	2	1	N-Term(iTRAQ8plex)	1.483	3.97	4.801	3.37	27	0	1246.74968	2	623.878479	6.063837774	21.65419
														LTWFVNEGLVDGWDDPR	2	1	1	N-Term(iTRAQ8plex)	1.258	2.398	2.766	3.33	59	0	2323.17827	2	1162.092773	5.225150027	59.57131667
														VACVQVVIIPCGITNALSEEDKEALIAK	2	2	1	N-Term(iTRAQ8plex); C3(Methylthio); C11(Methylthio); K22(iTRAQ8plex); K28(iTRAQ8plex)	-	-	-	2.76	30	1	3931.15976	3	1311.058105	1.345622302	65.07074167
														LNLENK	1	2	1	N-Term(iTRAQ8plex); K6(iTRAQ8plex)	0.85	1.874	1.494	2.7	-	0	1338.80912	3	446.9412231	-8.227548105	20.03980167
														VASQGEVVR	1	2	1	N-Term(iTRAQ8plex)	1.028	1.546	1.829	-	30	0	1248.72612	2	624.8666992	3.796282801	16.94998333
														YNVYPTYDFACPIVDSIEGVTHALR	1	1	1	N-Term(iTRAQ8plex); Y1(iTRAQ8plex); C11(Methylthio)	-	-	-	-	24	0	3497.75844	3	1065.196045	2.980740973	68.65442333
														LLSVNIR	1	2	1	N-Term(iTRAQ8plex)	1.004	1.402	1.629	-	17	0	1118.71184	2	559.8595581	-7.215181584	32.399795
62244044	OCIAD2	OCIA domain-containing protein 2 isoform 1	NP_001014446.1	33.12	5	5	79	154	16.9	9.03	0.296	0.216	0.223																
														VALAGLLGFGLGK	24	2	1	N-Term(iTRAQ8plex); K13(iTRAQ8plex)	0.258	0.171	0.183	5.43	73	0	1824.15715	2	912.5822144	0.208748976	49.12390333
														HCLLTCEECK	15	2	1	N-Term(iTRAQ8plex); C2(Methylthio); C6(Methylthio); C9(Methylthio); K10(iTRAQ8plex)	0.364	0.347	0.198	5.11	37	0	1924.87058	3	642.2950439	-1.878419309	36.31649333
														ECQEESFWK	6	2	1	N-Term(iTRAQ8plex); C2(Methylthio); K9(iTRAQ8plex)	0.276	0.151	0.166	4.21	44	0	1839.88494	2	920.4454346	-1.630418026	27.91555667
														VSYIGVCQSK	8	2	1	N-Term(iTRAQ8plex); C7(Methylthio); K10(iTRAQ8plex)	0.472	0.281	0.258	3.83	57	0	1737.94451	2	869.4758911	-2.460776753	32.38014667
														FHFFEDQLR	26	2	1	N-Term(iTRAQ8plex)	0.343	0.343	0.381	3.81	41	0	1542.7985	3	514.9376831	-1.422890675	37.154055
62460637	IPO4	importin-4	NP_078934.3	4.72	4	4	10	1081	118.6	4.96	1.183	2.960	1.882																
														KAGLLVLAVLSDGAGDHIR	2	1	1	N-Term(iTRAQ8plex); K1(iTRAQ8plex)	0.379	0.97	0.461	4.09	26	1	2513.4916	4	629.1283569	-4.318075156	50.60713
														LLECPHLNVR	4	1	1	N-Term(iTRAQ8plex); C4(Methylthio)	1.334	2.96	1.882	3.88	36	0	1543.83469	3	515.2830811	-2.838636783	35.39141833
														EVMPLLLAYLK	2	1	1	N-Term(iTRAQ8plex); K11(iTRAQ8plex)	1.085	3.338	2.071	3.38	35	0	1898.1608	3	633.3917847	-1.970616026	52.23953667
														LLPPLLQIVCK	2	1	1	N-Term(iTRAQ8plex); C10(Methylthio); K11(iTRAQ8plex)	-	-	-	3.15	33	0	1891.17496	3	631.0631714	0.861163134	60.98622167
62526022	CUTA	protein CutA isoform 2 precursor	NP_001014837.1	17.31	1	1	4	156	16.8	5.21	-	-	-																

														SVHPYEVAEVIALPVEQGNFPYLQWVR	4	3	1	N-Term(iTRAQ8plex)	-	-	-	4.56	26	0	3444.81289	4	861.9586792	-3.766353515	64.38013833
62865612	AK6	adenylate kinase isoenzyme 6 isoform c	NP_001015891.1	18.34	1	1	1	169	19.8	4.74	-	-	-															
														EEQLYDGYDEEYDCPILDEDRVVDELDNQMR	2	2	1	N-Term(iTRAQ8plex); C14(Methylthio)	-	-	-	2.71	16	1	4158.82065	3	1386.945068	3.880794455	54.939355
62955803	NUP188	nucleoporin NUP188 homolog	NP_056169.1	2.80	4	4	6	1749	195.9	6.73	1.032	0.760	0.828															
														HLVDETMDPFVDR	2	1	1	N-Term(iTRAQ8plex)	1.42	1.134	1.956	4.74	26	0	1877.94193	3	626.6521606	2.645120979	32.666875
														AAIAFLHALWQDRR	2	1	1	N-Term(iTRAQ8plex)	-	-	-	4.62	39	1	1972.11696	4	493.7846985	-0.683141371	55.56925167
														HTLNPEETSSVVR	1	1	1	N-Term(iTRAQ8plex)	0.285	0.248	0.171	3.33	-	0	1772.9401	3	591.6515503	-2.461262566	16.79081833
														ELWTILLGR	1	1	1	N-Term(iTRAQ8plex)	1.032	0.76	0.828	2.91	-	0	1404.85393	2	702.930603	1.607380412	53.94671167
63055057	ACTBL2	beta-actin-like protein 2	NP_001017992.1	17.29	1	8	244	376	42.0	5.59	0.474	0.874	1.017															
														IWYHTFYNELR	2	1	1	N-Term(iTRAQ8plex)	0.474	0.874	1.017	2.86	22	0	1845.94375	4	462.241394	-8.242400753	32.45384833
														ILTER	2	27	5	N-Term(iTRAQ8plex)	-	-	-	-	28	0	935.57616	2	468.2917175	-7.036157716	17.75416
														DLTDYLMK	15	8	3	N-Term(iTRAQ8plex); K8(iTRAQ8plex)	-	-	-	3.55	36	0	1606.88591	2	803.9469604	-6.488959006	34.11513833
														IIAPPER	20	8	3	N-Term(iTRAQ8plex)	-	-	-	3.2	50	0	1099.67974	3	367.2290039	-4.785817094	19.22718
														DLTDYLMK	11	8	3	N-Term(iTRAQ8plex); M7(Oxidation); K8(iTRAQ8plex)	-	-	-	2.96	35	0	1622.8792	2	803.9469604	-6.488959006	34.11513833
														SYELPDGQVITIGNER	114	16	4	N-Term(iTRAQ8plex)	-	-	-	5.5	87	0	2095.09258	2	1048.049927	-2.278851268	43.30549667
														SYELPDGQVITIGNER	10	16	4	N-Term(iTRAQ8plex); Y2(iTRAQ8plex)	-	-	-	-	61	0	2399.29204	2	1048.049927	-2.278851268	43.30549667
														HQGVMVGMGQK	10	7	3	N-Term(iTRAQ8plex); K11(iTRAQ8plex)	-	-	-	6.08	64	0	1779.96953	2	890.4884033	-6.922185558	14.76235333
														HQGVMVGMGQK	15	7	3	N-Term(iTRAQ8plex); M5(Oxidation); K11(iTRAQ8plex)	-	-	-	4.94	41	0	1795.96048	2	890.4884033	-6.922185558	14.76235333
														HQGVMVGMGQK	30	7	3	N-Term(iTRAQ8plex); M5(Oxidation); M8(Oxidation); K11(iTRAQ8plex)	-	-	-	4.79	44	0	1811.96592	2	890.4884033	-6.922185558	14.76235333
														CDVDIR	14	3	2	N-Term(iTRAQ8plex); C1(Methylthio)	-	-	-	-	34	0	1070.51923	2	535.7648315	-4.890915061	22.84930333
														CDVDIRK	1	3	2	N-Term(iTRAQ8plex); C1(Methylthio); K7(iTRAQ8plex)	-	-	-	-	29	1	1502.81584	2	751.9115601	-8.057409605	24.068925
63162572	CCT3	T-complex protein 1 subunit gamma isoform a	NP_005989.3	41.65	19	19	71	545	60.5	6.49	0.970	2.311	1.666															
														GISDLAQHYLMR	12	2	1	N-Term(iTRAQ8plex)	1.052	1.868	1.633	4.66	57	0	1707.90799	3	569.9741821	-4.343420905	35.09556667
														NVLLDPQLVPGGGASEMAVAHALTEK	2	2	1	N-Term(iTRAQ8plex); M17(Oxidation); K26(iTRAQ8plex)	2.213	8.316	6.23	4.48	30	0	3241.775	3	1081.263184	1.172577139	40.64455
														WSSLACNIALDAVK	14	2	1	N-Term(iTRAQ8plex); C6(Methylthio); K14(iTRAQ8plex)	1.608	4.549	3.265	4.46	72	0	2145.16216	2	1073.084717	-1.648046157	51.35887833
														IVLLDSSLEYK	5	2	1	N-Term(iTRAQ8plex); K11(iTRAQ8plex)	0.932	1.966	1.392	4.39	38	0	1888.1138	3	630.0427856	-6.015324068	69.8442
														EIQVQHPAAK	6	1	1	N-Term(iTRAQ8plex); K10(iTRAQ8plex)	0.993	2.613	1.923	4.31	46	0	1729.00908	2	865.0081787	-7.299266537	15.20138667
														IGDEYFTFITDCKDPK	1	2	1	N-Term(iTRAQ8plex); C12(Methylthio); K13(iTRAQ8plex); K16(iTRAQ8plex)	-	-	-	4.28	-	1	2850.46987	4	713.3729248	-4.298575958	50.22125167
														NLQDAMQVCR	4	2	1	N-Term(iTRAQ8plex); C9(Methylthio)	0.935	2.002	1.875	3.87	43	0	1527.73003	2	764.3686523	-5.46839582	27.26768
														AMTGVEQWPYR	4	2	1	N-Term(iTRAQ8plex)	0.782	1.27	1.166	3.74	29	0	1641.8344	2	821.4208374	-1.037324662	29.92891833
														NVLLDPQLVPGGGASEMAVAHALTEK	2	2	1	N-Term(iTRAQ8plex); K26(iTRAQ8plex)	-	-	-	3.66	-	0	3225.77309	3	1081.263184	1.172577139	40.64455
														TAVETAVLLLR	2	2	1	N-Term(iTRAQ8plex)	1.081	2.975	2.503	3.57	38	0	1489.92046	2	745.4638672	-3.432092615	49.28442
														AVAQALEVIPR	4	2	1	N-Term(iTRAQ8plex)	0.896	2.186	1.574	3.5	43	0	1470.88481	2	735.9460449	-6.661348436	44.45320167
														TLIQNCGASTIR	2	2	1	N-Term(iTRAQ8plex); C6(Methylthio)	0.924	2.551	1.539	3.37	46	0	1626.85552	2	813.9313965	-3.329091733	32.57655833
														IVSRPEELREDDVGTGAGLLEIK	4	2	1	N-Term(iTRAQ8plex); K23(iTRAQ8plex)	-	-	-	3.3	-	1	3104.72791	5	621.7514038	-4.30271747	34.78143333
														GESQTDIEITR	2	2	1	N-Term(iTRAQ8plex)	0.888	1.857	1.478	3.22	42	0	1552.81865	2	776.9129639	4.250215806	21.347345
														IPGGIIEDSCVLR	2	2	1	N-Term(iTRAQ8plex); C10(Methylthio)	1.077	3.014	2.033	2.85	39	0	1721.91606	2	861.4616699	-4.157622083	48.986575
														VEKIPGGIIEDSCVLR	1	2	1	N-Term(iTRAQ8plex); K3(iTRAQ8plex); C13(Methylthio)	1.349	2.741	2.276	2.7	-	1	2382.32865	3	794.7810669	-2.487006321	44.11117167
														EILSEVER	1	2	1	N-Term(iTRAQ8plex)	0.708	1.602	1.132	-	35	0	1278.71562	2	639.8614502	-3.978624552	28.44115833
														ELGIWEPLAVK	1	2	1	N-Term(iTRAQ8plex); K11(iTRAQ8plex)	2.331	2.972	2.868	-	29	0	1863.1091	3	621.7078857	-5.87046857	35.246455
														TIADIIR	1	1	1	N-Term(iTRAQ8plex)	0.637	1.409	0.962	-	28	0	1105.68254	2	553.3449097	-5.190082904	30.66560833
														ACTILLR	1	2	1	N-Term(iTRAQ8plex); C2(Methylthio)	0.93	2.026	1.385	-	21	0	1139.65605	2	570.331665	-1.920616197	35.86488
63253298	SRM	spermidine synthase	NP_003123.2	3.31	1	1	4	302	33.8	5.49	0.906	1.687	2.323															
														AAFVLPEFAR	4	1	1	N-Term(iTRAQ8plex)	0.906	1.687	2.323	3.57	33	0	1424.81499	2	712.9111328	-3.784558024	45.27355667
66346685	SERBP1	plasminogen activator inhibitor 1 RNA-binding protein isoform 4	NP_056455.3	10.85	3	3	20	387	42.4	8.44	1.383	4.042	2.769															
														FDQLFDDESDPFEVLK	11	4	1	N-Term(iTRAQ8plex); K16(iTRAQ8plex)	2.682	7.062	5.816	4.94	50	0	2552.30802	3	851.4408569	2.462967141	52.089135
														RPDQQLQGEGK	2	4	1	N-Term(iTRAQ8plex); K11(iTRAQ8plex)	2.796	4.522	3.642	3.87	-	0	1864.03664	4	466.7646179	-7.016761376	13.257725
														PGHLQEGFGCVVTNR	7	4	1	N-Term(iTRAQ8plex); C10(Methylthio)	1.221	2.594	1.662	-	49	0	1963.97184	3	655.3287964	-3.36379052	27.47022667
66529396	PON2	serum paraoxonase/arylesterase 2 isoform 2	NP_001018171.1	17.54	4	4	5	342	38.0	5.72	0.826	0.880	0.942															
														FPGLHSFAPDKPGGILMMDLK	1	2	1	N-Term(iTRAQ8plex); K11(iTRAQ8plex); K21(iTRAQ8plex)	0.341	-	0.668	4.2	-	0	3183.77241	5	637.5603027	-3.220750988	46.41577667
														EVESVDLPHCHLIK	1	2	1	N-Term(iTRAQ8plex); C10(Methylthio); K14(iTRAQ8plex)	0.696	0.751	0.851	3.67	-	0	2273.21132	4	569.0582886	-5.691456393	32.02932667
														LFVYDPNNPPSSEVLR	1	5	1	N-Term(iTRAQ8plex)	0.982	1.52	1.758	3.31	-	0	2151.13382	3	717.7161255	-2.323465468	43.235175
														LLIGTLYHR	2	5	1	N-Term(iTRAQ8plex)	1.156	0.88	1.042	2.99	51	0	1389.84775	3	463.9541016	-3.043399894	32.93719667
66933016	IMPDH2	inosine-5'-monophosphate dehydrogenase 2	NP_000875.2	19.65	7	7	34	514	55.8	6.90	1.000	1.530	1.409															
														REDLVVAPAGITLK	13	4	1	N-Term(iTRAQ8plex); K14(iTRAQ8plex)	0.93	1.68	1.256	5.19	50	1	2090.27396	3	697.4260254	-7.601136388	28.87004167
														DIDFLKEEEHDCFLEEIMTK	2	4	1	N-Term(iTRAQ8plex); K6(iTRAQ8plex); C12(Methylthio); K20(iTRAQ8plex)	-	-	-	4.56	-	1	3442.72133	4	861.435791	-3.920769702	53.11079167
														EDLVVAPAGITLK	2	4	1	N-Term(iTRAQ8plex); K13(iTRAQ8plex)	1.219	1.754	1.728	4.39	46	0	1934.18266	2	967.5949707	2.261154714	35.25426
														FVPYLIAGIQHSCQDIGAK	2	4	1	N-Term(iTRAQ8plex); C13(Methylthio); K19(iTRAQ8plex)	-	-	-	4.16	32	0	2714.4562	4	679.3695068	-2.094281814	68.59564833
														HGFCGIPITDTGR	11	4	1	N-Term(iTRAQ8plex); C4(Methylthio)	1.1	1.464	1.367	3.79	37	0	1723.84714	3	575.2873535	-5.038150467	29.61690667
														FGVPVIADGGIQNVGHIAK	2	4	1	N-Term(iTRAQ8plex); K19(iTRAQ8plex)	0.866	1.675	1.791	3.12	27	0	2500.44229	3	834.1522827	-2.954039565	37.30046833
														LPIVNEDDELVAIIAR	2	4	1	N-Term(iTRAQ8plex)	0.635	1.467	1.534	3.07	73	0	2084.18755	2	1042.597412	-1.441185681	58.71867333
68051721	NCEH1	neutral cholesterol ester hydrolase 1 isoform b	NP_065843.3	10.23	4	4	12	440	49.0	7.93	0.972	0.802	0.641															
														TYILTCEHDVLR	2	3	1	N-Term(iTRAQ8plex); C6(Methylthio)	0.838	0.831	0.713	4.58	47	0	1812.92173	3	604.9787598	-4.013550624	45.2128
														VYFPEQIHDVVR	6	3	1	N-Term(iTRAQ8plex)	1.147	0.947	0.791	4.51	43	0	1805.9802	3	602.664917	-2.770558306	33.26411333
														LMLLDATFR	2	2	1	N-Term(iTRAQ8plex)	0.901	0.538	0.356	3.05	31	0	1383.79704	2	692.4021606	-0.113189241	49.32076333
														VTDTDFDGVEVR	2	2	1	N-Term(iTRAQ8plex)	0.881	0.637	0.493	3	47	0	1656.83599	2	828.9216309	-1.385895327	29.15836
68160937	TRIM25	E3 ubiquitin/ISG15 ligase TRIM25	NP_005073.2	2.06	1	1	2	630	70.9	8.09	0.958	1.224	1.999															
														NSASWCVEWFNTK	2	1	1	N-Term(iTRAQ8plex); C6(Methylthio); K13(iTRAQ8plex)	0.958	1.224	1.999	3.9	44	0	2226.07212	3	742.6955566	-9.490091524	73.91519167
68303549	ASL	argininosuccinate lyase isoform 3	NP_001020117.1	3.20	1	1	2	438	48.7	6.13	0.829	1.272	0.983															
														LNSNDEDIHTANER	2	3	1	N-Term(iTRAQ8plex)	0.829	1.272	0.983	3.17	22	0	1931.93094	3	644.6484985	-2.665180426	14.37607333
68303572	CSNK1A1	casein kinase I isoform alpha isoform 2	NP_001883.4	12.76	3	3	5	337	38.9	9.57	0.732	0.554	0.486															
														AAQQAASSSGQGQQAQTPTGK	1	3	1	N-Term(iTRAQ8plex); K21(iTRAQ8plex)	-	-	-	3.67	-	0	2610.36318	4	653.3462524	-2.322057874	12.71064833
														TVLMLADQMISR	2	5	1	N-Term(iTRAQ8plex)	0.946	0.613	0.506	3.66	46	0	1681.93205	2	841.4696655	2.225496968	48.90106667
														LFLIDFGLAK	2	3	1	N-Term(iTRAQ8plex); K10(iTRAQ8plex)	0.567	0.501	0.467	3.01	37	0	1745.08738	3	582.3673096	2.970717118	49.28183
68509926	DHX15	pre-mRNA-splicing factor ATP-dependent RNA helicase DHX15	NP_001349.2	21.38	14	14	56	795	90.9	7.46	1.085	0.854	1.037															
														ALVTGYFMQVAHLER	6	1	1	N-Term(iTRAQ8plex)	1.024	0.725	0.939	6.9	82	0	2039.1099	3	680.3748169	2.391304951	50.305965
														IAPQYYDMSNFPQCEAK	6	1	1	N-Term(iTRAQ8plex); C14(Methylthio); K17(iTRAQ8plex)	1.122	0.848	1.035	5.93	51	0	2659.30156	2	1330.154419	7.535426187	39.62215
														LGIDDLVHFDFMDPPAPETLMR	4	1	1	N-Term(iTRAQ8plex)	1.436	0.84	1.32	5.79	32	0	2833.40897	3	945.1411743	-4.158101039	80.29259833
														TLATDILMGVLK	10	1	1	N-Term(iTRAQ8plex); K12(iTRAQ8plex)	1.154	0.861	1.056	4.92	59	0	1883.13506	2	942.071167	-7.739187674	48.89015333
														THPVEIFYTPEPERDYLEAAIR	4	1	1	N-Term(iTRAQ8plex)	-	-	-	4.8	31	1	2950.52578	4	738.3869019	0.161409156	40.82634333
														YGVIILDEAHER	8	1	1	N-Term(iTRAQ8plex)	1.112	0.936	0.947	4.66	57	0	1718.93131	3	573.6486206	-3.847002821	32.24209333
														HQSFVLVGETGSGK	2	1	1	N-Term(iTRAQ8plex); K14(iTRAQ8plex)	1.025	1.757	1.839	4.55	37	0	2054.13205	3	685.3822021	-8.299420021	19.04431167
														IRVESLLVTAISK	4	1	1	N-Term(iTRAQ8plex); K13(iTRAQ8plex)	1.338	1.648	1.929	4.48	33	1	2037.27756	3	679.7640381	-5.728376148	45.69337833
														TTQIPQWCVEYMR	2	1	1	N-Term(iTRAQ8plex); C8(Methylthio)	1.229	0.829	0.958	4.18	55	0	2004.9793	2	1002.993286	7.253613869	46.203305
														VESLLVTAISK	2	1	1	N-Term(iTRAQ8plex); K11(iTRAQ8plex)	0.918	0.573	0.819	3.82	32	0	1768.10721	3	590.0405884	1.793209539	41.92684333
														KLGIDDLVHFDFMDPPAPETLMR	2	1	1	N-Term(iTRAQ8plex); K1(iTRAQ8plex)	-	-	-	3.55	-	1	3265.71816	4	817.1849976	-0.894426733	54.41743833
														VAAMSVAQR	2	1	1	N-Term(iTRAQ8plex)	1	0.718	0.841	3.31	23	0	1236.70671	2	618.8569946	2.491567144	23.68796167
														EVDDLGPEVGDIK	2	1	1	N-Term(iTRAQ8plex); K13(iTRAQ8plex)	0.916	1.206	1.046	3.19	32	0	1994.07657	3	665.3637085	-6.869877471	24.15088333
														ALVTGYFMQVAHLER	1	1	1	N-Term(iTRAQ8plex); Y6(iTRAQ8plex)	0.442	0.475	0.697	-	37	0	2343.29873	3	680.3748169	2.391304951	50.305965
														FTDILVR	1	1	1	N-Term(iTRAQ8plex)	1.028	0.854	1.012	-	26	0	1167.69744	2	584.352356	-5.56309001	36.744845
68799814	AP2M1	AP-2 complex subunit mu isoform b	NP_001020376.1	17.09	6	6	8	433	49.4	9.54	1.000	0.481	0.846															
														QSIAIDDCTFHQCVR	2	3	1	N-Term(iTRAQ8plex); C8(Methylthio); C13(Methylthio)	1.504	1.33	1.475	5.87	46	0	2131.96433	3	711.3262939	-2.624206225	34.49661833
														ESQISAEIELLPTNDK	1	3	1	N-Term(iTRAQ8plex); K16(iTRAQ8plex)	0.973	0.955	1.113	5.15	-	0	2395.31632	3	799.1102905	-0.577363398	37.21101
														WARPPISMNFEVPFAPSGLK	1	3	1	N-Term(iTRAQ8plex); K20(iTRAQ8plex)	1.24	0.282	0.75	3.15	-	0	2852.56923	4	713.8977661	-1.758669628	52.19068
														SYLSGMPECK	1	3	1	N-Term(iTRAQ8plex); C9(Methylthio); K10(iTRAQ8plex)	0.949	0.703	0.846	2.78	-	0	1768.88213	2	884.9447021	-4.016448023	43.90288333
														DIILPFR	1	3	1	N-Term(iTRAQ8plex)	1	0.402	0.828	-	23	0	1177.72038	2	589.3638306	-3.639902343	38.261485
														VIPLVR	2	3	1	N-Term(iTRAQ8plex)	0.987	0.42	0.853	-	23	0	1000.67388	2	500.8405762	-8.190057169	20.16178
70906444	DUT	deoxyuridine 5'-triphosphate nucleotidohydrolase, mitochondrial isoform 3	NP_001020420.1	46.81	5	5	20	141	15.4	6.57	0.860	3.025	1.318															
														HFIDVGAGVIDEDYR	10	4	1	N-Term(iTRAQ8plex)	0.732	0.847	0.88	5.35	67	0	2010.01847	3	670.6776733	-2.4848182	33.934285
														TDIQIALPSGCYGR	2	5	1	N-Term(iTRAQ8plex); C11(Methylthio)	0.724	3.025	1.318	4.79	38	0	1843.92534	2	922.4663086	-5.147568771	42.51405167
														IFYPEIEEVQALDDTER	4	4	1	N-Term(iTRAQ8plex)	1.182	4.268	2.059	4.77	96	0	2371.19805	2	1186.102661	0.381387639	57.651215
														GNVGVVLFNFGK	2	4	1	N-Term(iTRAQ8plex); K12(iTRAQ8plex)	6.817	7.454	2.401	4.18	36	0	1859.08232	2	930.0447998	-9.493022663	67.05624333
														IAQLICER	2	4	1	N-Term(iTRAQ8plex); C6(Methylthio)	1.167	2.78	1.222	2.71	-	0	1295.70391	2	648.3555908	-6.048665397	47.85523167
70980549	PDCD11	protein RRP5 homolog	NP_055791.1	4.49	5	5	9	1871	208.6	8.87	1.208	0.846	0.918															
														VTPNEGLTVSFPFGK	2	3	1	N-Term(iTRAQ8plex); K15(iTRAQ8plex)	1.757	0.858	1.73	5.69	49	0	2201.2542	2	1101.130737	5.231876167	39.422615
														LSCQNLGAVLDDVPVQGFFK	2	3	1	N-Term(iTRAQ8plex); C3(Methylthio); K20(iTRAQ8plex)	1.118	0.466	0.879	3.09	64	0	2804.50688	2	1402.75708	4.735676111	62.17243167
														EKFEILSVESLCEGMR	2	2	1	N-Term(iTRAQ8plex); K2(iTRAQ8plex); C12(Methylthio)	1.853	1.125	0.632	2.78	31	1	2524.30899	3	842.1090088	2.15140449	59.96622333
														IEEALMDPGRQPESADDFDR	2	3	1	N-Term(iTRAQ8plex)	1.156	0.804	0.917	-	25	1	2595.2208	3	865.7451172	-3.530715963	34.823645
														GRIPLLLTSLSFK	1	3	1	N-Term(iTRAQ8plex); K13(iTRAQ8plex)	-	-	-	-	21	1	2053.28359	4	514.076355	-7.700474597	48.94829333
70995396	NQO1	NAD(P)H dehydrogenase [quinone] 1 isoform b	NP_001020604.1	56.25	14	14	143	240	27.3	8.72	0.966	1.577	3.692															
														VFIGEFAYTYAAMYDKGPFR	8	2	1	N-Term(iTRAQ8plex); K16(iTRAQ8plex)	-	-	-	6.38	49	1	2954.5375	4	739.3887939	-1.291371023	64.69695333
														EGHLSPDIVAEQK	19	4	1	N-Term(iTRAQ8plex); K13(iTRAQ8plex)	0.763	1.436	2.63	5.89	58	0	2031.12016	2	1016.063721	-6.378999123	23.18048167
														KGWEVVESDLYAMNFNPIISR	17	4	1	N-Term(iTRAQ8plex); K1(iTRAQ8plex)	-	-	-	5.82	64	1	3076.62961	3	1026.21582	-1.879851342	60.33990333
														LKDPANFQYPAESVLAYK	11	4	1	N-Term(iTRAQ8plex); K2(iTRAQ8plex); K18(iTRAQ8plex)	0.762	1.634	2.393	5.58	37	1	2966.64736	3	989.5563965	-7.023094842	39.52845333
														GWEVVESDLYAMNFNPIISR	2	4	1	N-Term(iTRAQ8plex)	-	-	-	4.84	76	0	2644.3447	3	882.119751	2.396956678	64.917835

														ALIVLAHSER	55	4	1	N-Term(iTRAQ8plex)	1.268	1.577	3.659	4.59	57	0	1412.84159	3	471.6187134	-7.887942558	27.56129667
														FGLSVGHHLGK	5	4	1	N-Term(iTRAQ8plex); K11(iTRAQ8plex)	0.88	2.952	10.081	4.51	66	0	1760.02886	2	880.5180664	-7.906181453	28.24737833
														VFIGEFAYTYAAMYDK	2	2	1	N-Term(iTRAQ8plex); K16(iTRAQ8plex)	-	-	-	4.34	54	0	2497.28354	3	833.0993652	-3.939819691	61.65784833
														IQILEGWK	6	4	1	N-Term(iTRAQ8plex); K8(iTRAQ8plex)	0.528	1.504	4.521	3.73	39	0	1594.96891	3	532.3255615	-9.768244262	42.591615
														EAAAAALKK	3	4	1	N-Term(iTRAQ8plex); K8(iTRAQ8plex); K9(iTRAQ8plex)	1.37	4.839	16.196	3.56	33	1	1785.12126	2	893.06427	-8.32877925	21.31693
														TSFNYAMK	3	4	1	N-Term(iTRAQ8plex); K8(iTRAQ8plex)	0.901	2.819	7.255	3.36	27	0	1569.84153	2	785.4249878	-8.174280182	18.51622333
														KGWEVVESDLYAMNFNPIISR	3	4	1	N-Term(iTRAQ8plex); K1(iTRAQ8plex); M13(Oxidation)	-	-	-	3.35	42	1	3092.61513	3	1026.21582	-1.879851342	60.33990333
														IQILEGWKK	2	4	1	N-Term(iTRAQ8plex); K8(iTRAQ8plex); K9(iTRAQ8plex)	-	-	-	3.24	34	1	2027.25979	4	507.5704041	-9.004750741	38.602065
														EVQDEEK	3	4	1	N-Term(iTRAQ8plex); K7(iTRAQ8plex)	1.122	2.159	5.704	2.99	32	0	1484.79389	3	495.6028137	-7.673380112	11.73584167
														SIPTDNQIK	3	4	1	N-Term(iTRAQ8plex); K9(iTRAQ8plex)	1.319	2.231	8.284	2.74	41	0	1623.93967	3	541.9847412	-7.968841099	26.42782167
														TSFNYAMK	1	4	1	N-Term(iTRAQ8plex); M7(Oxidation); K8(iTRAQ8plex)	1.052	1.203	1.794	2.73	-	0	1585.83634	2	785.4249878	-8.174280182	18.51622333
71565154	ADH5	alcohol dehydrogenase class-3	NP_000662.3	8.82	2	2	5	374	39.7	7.49	0.825	1.779	1.983																
														AGDTVIPLYIPQCGECK	3	1	1	N-Term(iTRAQ8plex); C13(Methylthio); C16(Methylthio); K17(iTRAQ8plex)	0.396	1.031	1.056	3.47	57	0	2507.27007	2	1254.138672	2.933002749	48.92110167
														VDEFVTHNLSFDEINK	2	1	1	N-Term(iTRAQ8plex); K16(iTRAQ8plex)	0.91	2.83	2.996	3.25	-	0	2515.31655	4	629.5845947	-4.922749685	42.341375
71772942	AP1G1	AP-1 complex subunit gamma-1 isoform b	NP_001119.3	2.92	2	2	10	822	91.3	6.80	1.087	1.582	1.967																
														ELLYFLDSCEPEFK	8	2	1	N-Term(iTRAQ8plex); C9(Methylthio); K14(iTRAQ8plex)	1.133	1.92	2.002	5.11	50	0	2387.21299	3	796.4091797	0.261188467	58.40892333
														AALCAVHVIR	2	2	1	N-Term(iTRAQ8plex); C4(Methylthio)	1.017	0.941	0.996	3.8	56	0	1402.79212	3	468.2688904	-3.111228931	27.58965333
71773329	ANXA6	annexin A6 isoform 1	NP_001146.2	27.49	13	13	37	673	75.8	5.60	0.483	0.414	2.131																
														WGTDEAQFIYILGNR	4	3	1	N-Term(iTRAQ8plex)	0.467	1.052	2.383	6.01	55	0	2087.09726	3	696.3706055	5.209079827	50.34304667
														GSIHDFPGFDPNQDAEALYTAMK	2	2	1	N-Term(iTRAQ8plex); K23(iTRAQ8plex)	-	-	-	5.28	35	0	3132.55117	4	783.8932495	-1.463067283	49.15733
														GFGSDKEAILDIITSR	8	3	1	N-Term(iTRAQ8plex); K6(iTRAQ8plex)	0.139	0.088	2.762	5.12	38	1	2330.31645	4	583.3323364	-4.349416035	53.15356833
														GLGTDEDTIIDIITHR	6	3	1	N-Term(iTRAQ8plex)	-	-	3.503	5.08	71	0	2073.11661	3	691.7103882	1.736516297	52.71364833
														SLEDALSSDTSGHFR	2	3	1	N-Term(iTRAQ8plex)	1.05	0.831	1.455	4.6	45	0	1925.94156	3	642.6520386	-4.734737295	28.74584167
														EDAQVAAEILEIADTPSGDKTSLETR	2	2	1	N-Term(iTRAQ8plex); K20(iTRAQ8plex)	-	-	-	4.52	32	1	3367.76797	4	842.6974487	-0.307698789	46.456075
														DAFVAIVQSVK	2	3	1	N-Term(iTRAQ8plex); K11(iTRAQ8plex)	0.638	0.414	2.131	4.29	34	0	1785.05948	3	595.6913452	-7.618180572	35.45813
														ALIEILATR	4	3	1	N-Term(iTRAQ8plex)	0.304	0.248	2.069	3.45	27	0	1303.81841	2	652.418457	3.463751989	42.42913
														TTGKPIEASIR	2	3	1	N-Term(iTRAQ8plex); K4(iTRAQ8plex)	0.081	0.1	2.106	3.08	27	0	1781.0645	2	891.0358887	-5.412817024	15.87819333
														SELDMLDIR	1	3	1	N-Term(iTRAQ8plex)	0.499	0.428	1.245	3	-	0	1395.74138	2	698.3743286	-2.995320978	36.05752167
														SEIDLLNIR	2	3	1	N-Term(iTRAQ8plex)	0.553	0.613	1.73	2.98	26	0	1376.79863	2	688.9029541	-4.706364988	36.95012167
														GTVRPANDFNPDADAK	1	3	1	N-Term(iTRAQ8plex); K16(iTRAQ8plex)	-	-	2.846	2.98	-	0	2296.19785	3	766.0708008	-7.143202515	17.00104833
														CLIEILASR	1	3	1	N-Term(iTRAQ8plex); C1(Methylthio)	0.139	0.27	1.924	-	24	0	1367.76555	2	684.3864136	-2.713748391	53.50254667
71897229	SLC9A3R2	Na(+)/H(+) exchange regulatory cofactor NHE-RF2 isoform b	NP_004776.3	4.29	1	1	2	326	36.1	8.21	0.697	0.506	0.805																
														RVEPGSPAEAAALR	2	2	1	N-Term(iTRAQ8plex)	0.697	0.506	0.805	3.1	27	1	1727.96738	3	576.6606445	-1.89295023	17.90486833
71979932	SLC7A5	large neutral amino acids transporter small subunit 1	NP_003477.4	10.26	3	4	29	507	55.0	7.72	1.363	0.531	0.258																
														SADGSAPAGEGEGVTLQR	6	3	1	N-Term(iTRAQ8plex)	1.416	0.668	0.486	5.72	88	0	2006.01798	2	1003.506897	-1.382834334	18.007405
														WLLQGIFSTTVLCQK	16	2	1	N-Term(iTRAQ8plex); C13(Methylthio); K15(iTRAQ8plex)	1.314	0.516	0.258	5.47	57	0	2391.32444	3	797.7796631	-6.049489551	85.48323833
														ALAAPAAEEKEEAR	6	3	1	N-Term(iTRAQ8plex); K10(iTRAQ8plex)	1.491	0.527	0.197	4.49	60	1	2064.148	2	1032.578125	-2.731353247	17.07203167
														MLAAK	1	8	2	N-Term(iTRAQ8plex); M1(Oxidation); K5(iTRAQ8plex)	-	-	-	-	22	0	1157.719	3	386.5778503	1.504229778	12.19833333
73486658	GOT2	aspartate aminotransferase, mitochondrial isoform 1 precursor	NP_002071.2	47.21	14	14	78	430	47.5	9.01	1.133	0.932	0.702																
														ASAELALGENSEVLK	6	1	1	N-Term(iTRAQ8plex); K15(iTRAQ8plex)	1.157	0.886	0.635	7.45	78	0	2139.22246	2	1070.114868	4.991724015	31.096145
														ISVAGVTSSNVGYLAHAIHQVTK	6	2	1	N-Term(iTRAQ8plex); K23(iTRAQ8plex)	-	-	1.523	6.56	50	0	2960.66999	4	740.9221191	-3.77435856	48.06328667
														NLFAFFDMAYQGFASGDGDKDAWAVR	2	2	1	N-Term(iTRAQ8plex); K20(iTRAQ8plex)	-	-	0.736	6.56	57	1	3507.7333	4	877.6887817	2.276619244	67.11754833
														NLFAFFDMAYQGFASGDGDKDAWAVR	2	2	1	N-Term(iTRAQ8plex); M8(Oxidation); K20(iTRAQ8plex)	-	-	-	5.27	33	1	3523.72451	4	877.6887817	2.276619244	67.11754833
														HFIEQGINVCLCQSYAK	4	2	1	N-Term(iTRAQ8plex); C10(Methylthio); C12(Methylthio); K17(iTRAQ8plex)	0.861	0.77	0.659	5.06	46	0	2653.3176	3	885.1107178	-1.636905317	50.181735
														FVTVQTISGTGALR	8	2	1	N-Term(iTRAQ8plex)	1.209	0.979	0.807	5.05	61	0	1754.02019	2	877.5137329	4.993044189	34.42994333
														EYLPIGGLAEFCK	2	1	1	N-Term(iTRAQ8plex); C12(Methylthio); K13(iTRAQ8plex)	1.173	1.036	0.891	5.01	42	0	2094.11734	3	698.7106323	-2.425163689	47.75308833
														TCGFDFTGAVEDISK	10	2	1	N-Term(iTRAQ8plex); C2(Methylthio); K15(iTRAQ8plex)	0.611	0.882	0.759	4.95	56	0	2244.10324	3	748.710083	0.879399151	44.74847667
														NLDKEYLPIGGLAEFCK	18	1	1	N-Term(iTRAQ8plex); K4(iTRAQ8plex); C16(Methylthio); K17(iTRAQ8plex)	1.174	1.056	0.762	4.83	52	1	2868.57793	2	1434.792603	0.427444593	52.80663667
														VGAFTMVCK	4	2	1	N-Term(iTRAQ8plex); C8(Methylthio); K9(iTRAQ8plex)	1.098	1.071	0.653	4.53	44	0	1609.87278	2	805.4318848	-9.92597093	64.88806333
														EFSIYMTK	3	2	1	N-Term(iTRAQ8plex); K8(iTRAQ8plex)	0.784	0.624	0.557	3.71	28	0	1626.90232	3	542.97229	0.101768364	42.50156
														DAGMQLQGYR	6	2	1	N-Term(iTRAQ8plex)	1.118	0.972	0.698	3.62	39	0	1442.72942	2	721.8683472	-4.826673649	27.81189
														DDNGKPYVLPSVR	1	2	1	N-Term(iTRAQ8plex); K5(iTRAQ8plex)	0.958	0.723	0.854	3.25	-	0	2068.14663	4	517.7921143	-8.763473785	23.19223167
														KAEAQIAAK	4	1	1	N-Term(iTRAQ8plex); K1(iTRAQ8plex); K9(iTRAQ8plex)	0.953	0.712	0.615	3.19	30	1	1842.16677	2	921.5889893	7.116927661	17.99244
														IPEQSVLLLHACAHNPTGVDPRPEQWK	2	2	1	N-Term(iTRAQ8plex); C12(Methylthio); K27(iTRAQ8plex)	-	-	-	2.9	-	0	3689.94611	5	738.7950439	-1.205731276	49.60760667
73622130	BOLA2	bolA-like protein 2 isoform 1	NP_001026997.1	23.03	2	2	16	152	16.9	8.19	0.829	2.879	1.631																
														LVNACLAEELPHIHAFEQK	10	1	1	N-Term(iTRAQ8plex); C5(Methylthio); K19(iTRAQ8plex)	-	3.567	1.782	7.29	43	0	2816.49306	4	704.8776245	-5.735557649	51.57764833
														DLEAEHVEVEDTTLNR	6	2	1	N-Term(iTRAQ8plex)	0.829	2.324	1.493	4.07	47	0	2174.0817	3	725.3657837	-2.356614838	23.46839
73623028	CPT1A	carnitine O-palmitoyltransferase 1, liver isoform isoform 2	NP_001027017.1	7.01	4	4	23	756	86.2	8.44	1.205	1.367	0.685																
														IPGEETDTIQHMR	4	4	1	N-Term(iTRAQ8plex)	0.922	1.137	0.412	4.96	85	0	1830.92942	3	610.9813232	-1.520988965	22.27026833
														TSPDAFVQLALQLAHYK	4	4	1	N-Term(iTRAQ8plex); K17(iTRAQ8plex)	-	-	-	4.91	34	0	2510.42348	4	628.3613281	0.287330008	62.109685
														IPGEETDTIQHMR	4	4	1	N-Term(iTRAQ8plex); M12(Oxidation)	1.139	0.943	0.518	4.16	38	0	1846.92496	3	610.9813232	-1.520988965	22.27026833
														AGNAIHAILLYR	9	4	1	N-Term(iTRAQ8plex)	1.507	1.585	0.693	3.99	45	0	1615.9581	3	539.3242188	-0.302173676	33.21057
														SCTTESCDFVR	2	4	1	N-Term(iTRAQ8plex); C2(Methylthio); C7(Methylthio)	1.183	1.43	0.695	2.75	29	0	1643.68804	2	822.3476563	2.48379249	32.67066
73695475	HEATR1	HEAT repeat-containing protein 1	NP_060542.4	7.00	11	11	28	2144	242.2	6.54	1.950	0.837	1.055																
														MIELLADNINLGDPSSMLK	2	1	1	N-Term(iTRAQ8plex); K19(iTRAQ8plex)	2.29	0.851	1.058	7.29	62	0	2682.48264	3	894.8323975	5.930264965	63.41670167
														MVEDLISVGEEESFNLK	2	1	1	N-Term(iTRAQ8plex); K17(iTRAQ8plex)	2.07	1.13	1.055	6	46	0	2547.36655	3	849.7937012	7.552492052	59.98486833
														ITSEMGSASQANIR	2	1	1	N-Term(iTRAQ8plex)	1.685	0.713	0.807	4.85	49	0	1768.92803	2	884.9676514	6.486874653	26.50759667
														GWEEALENVIK	4	1	1	N-Term(iTRAQ8plex); K11(iTRAQ8plex)	2.932	1.228	1.659	4.5	70	0	1896.05901	2	948.5331421	-5.128395411	70.821785
														IISVFVDALPHVPEHR	7	1	1	N-Term(iTRAQ8plex)	0.923	-	-	4.42	52	0	2133.21035	4	534.0567017	-3.419521075	58.61216667
														LDDTYSFQVINK	1	1	1	N-Term(iTRAQ8plex); K12(iTRAQ8plex)	1.655	0.935	1.069	4.3	-	0	2051.11338	3	684.3759766	-6.618289684	53.43471333
														TLDVVLEEHLK	1	1	1	N-Term(iTRAQ8plex); K11(iTRAQ8plex)	1.418	0.753	0.978	3.49	-	0	1904.11887	3	635.3778076	-6.539349321	32.87066667
														GIPVELWAHYVEELNSTQR	4	1	1	N-Term(iTRAQ8plex)	2.624	0.807	0.658	3.35	35	0	2545.33957	3	849.118042	1.678136233	50.80665333
														GISVNAEQVR	2	1	1	N-Term(iTRAQ8plex)	1.071	0.331	0.397	3.29	29	0	1376.78337	2	688.8953247	2.477872794	22.16714167
														HRWFWLLPVK	1	1	1	N-Term(iTRAQ8plex); K10(iTRAQ8plex)	1.837	0.837	1.105	3.01	-	1	1990.18239	4	498.3010559	-8.822079445	46.556885
														SGICSLHPLLR	2	1	1	N-Term(iTRAQ8plex); C4(Methylthio)	2.873	3.665	2.885	2.87	24	0	1545.85068	3	515.9550781	-2.614207859	36.871925
74099697	HNRNPH2	heterogeneous nuclear ribonucleoprotein H2	NP_001027565.1	34.52	5	12	118	449	49.2	6.30	0.819	0.738	0.959																
														VTGEADVEFATHEDAVAAMAK	3	1	1	N-Term(iTRAQ8plex); K21(iTRAQ8plex)	0.747	0.666	0.767	6.24	43	0	2770.41446	3	924.1430054	-1.230298486	36.19631
														EGRPSGEAFVELESEEEVK	2	1	1	N-Term(iTRAQ8plex); K19(iTRAQ8plex)	0.826	0.619	0.914	4.55	40	0	2729.39994	3	910.4714966	-3.346817659	29.97742667
														ATENDIYNFFSPLNPMR	5	1	1	N-Term(iTRAQ8plex)	0.818	1.16	0.797	4.24	55	0	2333.15434	2	1167.080811	0.219948479	58.09009833
														SNSVEMDWVLK	2	1	1	N-Term(iTRAQ8plex); K11(iTRAQ8plex)	0.637	0.669	1.007	4.2	57	0	1916.02825	2	958.5177612	-6.552856979	53.01419333
														GLPWSCSADEVMR	4	1	1	N-Term(iTRAQ8plex); C6(Methylthio)	0.795	0.885	1.188	3.9	49	0	1800.83037	2	900.9188232	-4.521489509	44.567595
														VTGEADVEFATHEDAVAAMAK	2	1	1	N-Term(iTRAQ8plex); M19(Oxidation); K21(iTRAQ8plex)	-	-	-	3.54	36	0	2786.40493	3	924.1430054	-1.230298486	36.19631
														GLPWSCSADEVMR	1	1	1	N-Term(iTRAQ8plex); C6(Methylthio); M12(Oxidation)	0.819	0.728	1.047	-	34	0	1816.84172	2	900.9188232	-4.521489509	44.567595
														VHIEIGPDGR	30	14	3	N-Term(iTRAQ8plex)	-	-	-	3.86	57	0	1396.78022	3	466.2649231	-3.461720631	18.23122667
														HTGPNSPDTANDGFVR	28	7	2	N-Term(iTRAQ8plex)	-	-	-	5.81	74	0	1988.96738	3	663.6606445	-2.735568368	13.74724167
														STGEAFVQFASQEIAEK	12	7	2	N-Term(iTRAQ8plex); K17(iTRAQ8plex)	-	-	-	5.53	82	0	2450.30198	2	1225.649902	-4.025328563	38.89856333
														DLNYCFSGMSDHR	10	13	2	N-Term(iTRAQ8plex); C5(Methylthio)	-	-	-	4.44	56	0	1894.81633	2	947.9118042	-1.309109237	36.276525
														DLNYCFSGMSDHR	4	13	2	N-Term(iTRAQ8plex); C5(Methylthio); M9(Oxidation)	-	-	-	3.97	41	0	1910.80661	2	947.9118042	-1.309109237	36.276525
														YIEIFK	8	8	2	N-Term(iTRAQ8plex); K6(iTRAQ8plex)	-	-	-	2.87	27	0	1420.86783	3	474.2941284	1.281402721	48.86860667
														YVEVFK	2	8	2	N-Term(iTRAQ8plex); K6(iTRAQ8plex)	-	-	-	2.83	-	0	1392.82268	2	696.914978	-8.638476017	24.23172
														FFSDCK	5	7	2	N-Term(iTRAQ8plex); C5(Methylthio); K6(iTRAQ8plex)	-	-	-	2.8	23	0	1400.72019	3	467.5782471	2.802601813	31.19859
77404397	SND1	staphylococcal nuclease domain-containing protein 1	NP_055205.2	30.55	19	19	57	910	101.9	7.17	1.033	1.010	1.148																
														VWAHYEEQPVEEVMPVLEEK	4	1	1	N-Term(iTRAQ8plex); K20(iTRAQ8plex)	1.533	1.271	1.075	5.1	45	0	3049.56388	3	1017.19281	-5.343181804	45.77190167
														VLPAQATEYAFAFIQVPQDDDARTDAVDSVVR	2	1	1	N-Term(iTRAQ8plex)	-	-	-	4.86	28	1	3811.93667	4	953.7379624	-2.076395004	49.116825
														VITEYLNAQESAK	2	1	1	N-Term(iTRAQ8plex); K13(iTRAQ8plex)	1.063	1.074	1.177	4.83	50	0	2074.14713	3	692.053894	-8.168379599	55.94222167
														SEAVVEYVFSGSR	8	2	1	N-Term(iTRAQ8plex)	0.905	0.994	1.089	4.69	43	0	1733.89231	3	578.6356201	-5.129614106	45.92605833
														NLPGLVQEGEPFSEEATLFTK	2	2	1	N-Term(iTRAQ8plex); K21(iTRAQ8plex)	1.04	1.013	1.505	4.58	67	0	2914.57134	2	1457.789307	1.864892165	47.08187
														EVCFTIENK	2	2	1	N-Term(iTRAQ8plex); C3(Methylthio); K9(iTRAQ8plex)	1.051	1.258	1.417	3.85	27	0	1736.90458	3	579.6397095	-7.246875972	49.979545
														EVEVEVESMDK	2	1	1	N-Term(iTRAQ8plex); M9(Oxidation); K11(iTRAQ8plex)	0.992	0.57	1.107	3.8	44	0	1917.98435	3	639.9996338	-4.816610115	30.20212333
														LRPLYDIPYMFEAR	9	2	1	N-Term(iTRAQ8plex)	1.108	1.309	1.383	3.77	32	0	2088.12613	3	696.7131348	-0.273093405	51.44310667
														EYGMIYLGK	2	2	1	N-Term(iTRAQ8plex); K9(iTRAQ8plex)	0.936	0.975	1.189	3.75	33	0	1681.93132	2	841.4692993	-7.746622598	43.80856167
														TCATVTIGGINIAEALVSK	2	2	1	N-Term(iTRAQ8plex); C2(Methylthio); K19(iTRAQ8plex)	-	-	-	3.69	42	0	2515.40531	3	839.1399536	-1.2541558	58.55617167
														ALLLPDYYLVTVMLSGIK	2	2	1	N-Term(iTRAQ8plex); K18(iTRAQ8plex)	-	-	-	3.65	52	0	2617.55545	3	873.1900024	2.102256978	71.58210833
														SSHYDELLAAEAR	2	2	1	N-Term(iTRAQ8plex)	1.203	0.53	0.642	3.64	41	0	1765.90178	2	883.4545288	-0.271987274	19.040755
														EADGSETPEPFAAEAK	2	2	1	N-Term(iTRAQ8plex); K16(iTRAQ8plex)	1.268	0.936	0.958	3.6	25	0	2257.12516	3	753.0465698	-8.570671099	18.25485333
														VNVTVDYIRPASPATETVPAFSER	2	2	1	N-Term(iTRAQ8plex)	1.559	1.279	1.734	3.4	-	0	2923.52121	3	975.1785889	-8.745600449	62.06713167
														IHVFYIDYGNR	7	1	1	N-Term(iTRAQ8plex)	0.974	0.923	1.109	3.25	36	0	1700.90055	3	567.6383667	-3.324387398	39.87884
														NDIASHPPVEGSYAPR	3	1	1	N-Term(iTRAQ8plex)	0.835	0.801	0.95	3.1	26	0	2014.02115	3	672.0119019	-4.195081856	18.85637667
														KLRPLYDIPYMFEAR	1	2	1	N-Term(iTRAQ8plex); K1(iTRAQ8plex)	1.792	0.667	0.803	3.03	-	1	2520.42788	4	630.8624268	0.843448555	46.213925
														AKQFLPFLQR	1	2	1	N-Term(iTRAQ8plex); K2(iTRAQ8plex)	1.072	1.118	1.371	2.95	-	1	1856.12107	3	619.37854	-8.412399973	35.241075
														FVDGEWYR	2	1	1	N-Term(iTRAQ8plex)	0.972	1.064	1.267	2.88	18	0	1375.68633	2	688.3468018	-6.174666571	41.53581167
78000181	RPL14	60S ribosomal protein L14	NP_003964.3	24.65	5	5	60	215	23.4	10.93	1.647	2.149	1.892																
														VAYVSFGPHAGK	10	1	1	N-Term(iTRAQ8plex); K12(iTRAQ8plex)	1.813	2.356	1.888	4.64	65	0	1841.0427	2	921.0268555	-3.566983634	23.79088333
														LVAIVDVIDQNR	25	1	1	N-Term(iTRAQ8plex)	1.823	2.342	1.938	4.44	81	0	1658.98825	3	553.6676025	8.398532751	58.15856333
														CMQLTDFILK	20	1	1	N-Term(iTRAQ8plex); C1(Methylthio); K10(iTRAQ8plex)	1.45	2.105	1.846	4.17	63	0	1865.99626	2	933.506958	-4.363813184	49.36697167

														FPHSAHQK	1	1	1	N-Term(iTRAQ8plex); K8(iTRAQ8plex)	1.775	1.987	1.896	3.69	-	0	1559.87514	4	390.7242432	-9.698547874	11.06803333
														CMQLTDFILK	2	1	1	N-Term(iTRAQ8plex); C1(Methylthio); M2(Oxidation); K10(iTRAQ8plex)	1.334	1.333	1.535	3.25	39	0	1881.99497	2	933.506958	-4.363813184	49.36697167
														ALVDGPCTQVR	2	1	1	N-Term(iTRAQ8plex); C7(Methylthio)	1.18	1.404	1.233	-	29	0	1508.78349	2	754.8953857	-2.132470057	31.70925
78711824	SDHC	succinate dehydrogenase cytochrome b560 subunit, mitochondrial isoform 4 precursor	NP_001030590.1	6.90	1	1	2	116	12.5	8.98	0.791	0.390	0.527																
														HLMWDLGK	2	3	1	N-Term(iTRAQ8plex); K8(iTRAQ8plex)	0.791	0.39	0.527	3.62	28	0	1607.91666	3	536.6437378	-1.344081536	28.87428167
79750824	FAM129B	niban-like protein 1 isoform 2	NP_001030611.1	3.68	2	2	11	733	82.6	6.15	0.669	0.658	1.375																
														FQELIFEDFAR	8	4	1	N-Term(iTRAQ8plex)	1.201	1.808	1.606	4.22	53	0	1718.90787	3	573.6408081	1.337923018	55.49359333
														HEIEGTGLPQAQLLWR	3	4	1	N-Term(iTRAQ8plex)	0.342	0.262	0.576	3.77	-	0	2152.1724	3	718.0623169	-4.323011677	35.21755167
81230485	ACOT1	acyl-coenzyme A thioesterase 1	NP_001032238.1	8.55	2	2	5	421	46.2	7.34	1.353	3.171	1.998																
														TPLAVELEVLDGHDPDPG R	4	3	1	N-Term(iTRAQ8plex)	1.353	3.171	1.998	5.78	60	0	2334.2186	3	778.7443848	-2.476311825	43.07745333
														AESTFLFLVGQDDHNWK	1	6	1	N-Term(iTRAQ8plex); K17(iTRAQ8plex)	-	-	-	4.01	-	0	2615.36904	4	654.5977173	-0.929902946	49.734815
81295798	HHLA3	HERV-H LTR-associating protein 3 isoform 3	NP_001031723.1	9.21	1	1	1	76	8.6	7.25	0.428	0.410	0.452																
														GPYPDPK	1	1	1	N-Term(iTRAQ8plex); K7(iTRAQ8plex)	0.428	0.41	0.452	-	23	0	1381.78223	3	461.2655945	-8.214827551	13.37618833
82880645	USP14	ubiquitin carboxyl-terminal hydrolase 14 isoform b	NP_001032411.1	11.76	4	4	11	459	52.4	5.78	0.888	1.077	1.252																
														SLIDQFFGVEFETTMK	2	2	1	N-Term(iTRAQ8plex); K16(iTRAQ8plex)	0.341	0.82	1.086	4.92	35	0	2500.30753	3	834.1073608	-7.16393094	52.41737333
														RVEIMEEESEQ	5	2	1	N-Term(iTRAQ8plex)	0.923	1.03	0.984	3.73	60	1	1682.81243	2	841.9098511	-5.048555284	28.15853167
														FDDDKVSIVTPEDILR	2	2	1	N-Term(iTRAQ8plex); K5(iTRAQ8plex)	1.338	2.327	2.434	3.62	28	1	2470.35715	3	824.1239014	-3.177859701	39.95535167
														CTESEEEEVTK	2	2	1	N-Term(iTRAQ8plex); C1(Methylthio); K11(iTRAQ8plex)	0.747	2.383	1.659	3.22	28	0	1937.91538	3	646.6433105	-7.159192568	28.12652833
83267879	EIF2B5	translation initiation factor eIF-2B subunit epsilon	NP_003898.2	2.22	1	1	1	721	80.3	5.08	0.696	1.020	0.948																
														CHEDNVVVAVDSTTNR	2	1	1	N-Term(iTRAQ8plex); C1(Methylthio)	0.696	1.02	0.948	3.25	18	0	2108.98935	3	703.6679688	-5.376651136	23.41570667
83367083	UQCRQ	cytochrome b-c1 complex subunit 8	NP_055217.2	34.15	3	3	13	82	9.9	10.08	1.070	0.708	0.716																
														HVISYSLSPFEQR	10	1	1	N-Term(iTRAQ8plex)	1.137	1.005	0.722	5.15	72	0	1866.99761	2	934.0024414	-2.123788208	30.953495
														AYPHVFTK	1	1	1	N-Term(iTRAQ8plex); K8(iTRAQ8plex)	1.117	0.665	0.778	-	31	0	1570.91983	3	524.3114624	-0.214742319	18.43776833
														GIPNVLR	2	1	1	N-Term(iTRAQ8plex)	1.01	0.542	0.535	-	27	0	1072.67107	2	536.8391724	-6.510097685	19.02425333
83700235	EIF4A2	eukaryotic initiation factor 4A-II	NP_001958.2	18.43	1	6	53	407	46.4	5.48	0.872	1.364	1.203																
														LQAEAPHIVVGTPGR	1	1	1	N-Term(iTRAQ8plex)	0.872	1.364	1.203	2.79	-	0	1849.05503	3	617.0231934	-2.576706375	25.66689
														GYDVIAQAQSGTGK	6	3	2	N-Term(iTRAQ8plex); K14(iTRAQ8plex)	-	-	-	5.39	72	0	2003.09026	2	1002.048767	-5.767957918	33.57006667
														MFVLDEADEMLSR	16	3	2	N-Term(iTRAQ8plex)	-	-	-	4.67	75	0	1859.91391	3	620.6419067	-3.970262283	58.32259167
														MFVLDEADEMLSR	12	3	2	N-Term(iTRAQ8plex); M1(Oxidation)	-	-	-	3.85	58	0	1875.90799	3	620.6419067	-3.970262283	58.32259167
														VLITTDLLAR	8	2	2	N-Term(iTRAQ8plex)	-	-	-	3.36	41	0	1418.88054	2	709.9452515	-3.682466757	44.96066
														GIYAYGFEKPSAIQQR	9	5	3	N-Term(iTRAQ8plex); K9(iTRAQ8plex)	-	-	-	4.36	33	0	2436.33755	4	609.8397827	-5.044589113	29.05811333
														VDWLTEK	1	5	3	N-Term(iTRAQ8plex); K7(iTRAQ8plex)	-	-	-	3.27	-	0	1498.85893	2	749.9331055	-9.098242795	30.08201167
83715985	HSD17B10	3-hydroxyacyl-CoA dehydrogenase type-2 isoform 2	NP_001032900.1	34.52	5	5	15	252	26.0	7.21	1.009	0.708	0.907																
														LGNNCVFAPADVTSEKDV QTALALAK	1	2	1	N-Term(iTRAQ8plex); C5(Methylthio); K16(iTRAQ8plex); K26(iTRAQ8plex)	-	0.449	0.747	5.3	-	1	3633.99087	4	909.2531738	4.411990314	51.61021333
														GLVAVITGGASGLGLATA ER	2	2	1	N-Term(iTRAQ8plex)	1.009	0.669	0.836	4.7	52	0	2117.21482	3	706.40979	-3.98846293	54.18659667
														LGDPAEYAHLVQAIIENPF LNGEVIR	9	2	1	N-Term(iTRAQ8plex)	-	-	-	4.25	41	0	3182.72348	3	1061.579346	2.583497881	69.98179167
														VDVAVNCAGIAVASK	2	2	1	N-Term(iTRAQ8plex); C7(Methylthio); K15(iTRAQ8plex)	1.216	1.245	1.613	4.01	27	0	2071.14676	3	691.053772	-1.588314305	42.58811667
														DVQTALALAK	1	2	1	N-Term(iTRAQ8plex); K10(iTRAQ8plex)	0.848	0.749	0.984	3.17	-	0	1638.00583	3	546.6734619	0.715011	41.34197167
83921614	CYB5B	cytochrome b5 type B	NP_085056.2	43.33	5	5	27	150	16.7	4.97	1.097	0.674	0.776																
														FLNEHPGGEEVLLEQAGV DASESFEDVGHSSDAR	2	1	1	N-Term(iTRAQ8plex)	-	-	-	6.06	49	0	3931.83925	4	983.715271	-3.379384244	53.44269333
														NSLKELWLVIHGR	12	1	1	N-Term(iTRAQ8plex); K4(iTRAQ8plex)	1.682	0.894	0.88	5.52	45	1	2173.30678	4	544.0821533	0.079188659	44.310685
														GQEVETSVTYYR	6	1	1	N-Term(iTRAQ8plex)	1.109	0.48	0.664	4.81	56	0	1735.87749	2	868.4423828	-1.700750784	20.18736167
														ELWLVIHGR	6	1	1	N-Term(iTRAQ8plex)	1.066	1.034	0.904	4.26	61	0	1426.84394	3	476.286499	-1.617190219	34.29881667
														LEEVAK	1	1	1	N-Term(iTRAQ8plex); K6(iTRAQ8plex)	0.658	0.556	0.629	2.89	-	0	1296.8006	3	432.938385	1.743120179	21.45462333
83977459	PHLDA1	pleckstrin homology-like domain family A member 1	NP_031376.3	3.74	1	1	1	401	45.0	9.89	0.614	1.144	1.117																
														CCILTEEGLLLIPPK	1	1	1	N-Term(iTRAQ8plex); C1(Methylthio); C2(Methylthio); K15(iTRAQ8plex)	0.614	1.144	1.117	3.74	-	0	2342.29264	3	781.43573	4.684893439	63.22677833
84043963	EIF5B	eukaryotic translation initiation factor 5B	NP_056988.3	4.92	3	3	6	1220	138.7	5.49	1.268	1.162	1.321																
														AIIVEFAQQGLNAALFYEN KDPR	2	1	1	N-Term(iTRAQ8plex); K20(iTRAQ8plex)	-	-	-	5.91	32	1	3215.75429	4	804.6940308	-4.07622347	65.30787167
														IPGMLIIDTPGHESFSNLR	3	1	1	N-Term(iTRAQ8plex)	1.268	1.162	1.321	3.65	-	0	2401.27933	3	801.0979614	-2.444643811	44.883015
														ETVAVKPTENNEEEFTSK	1	1	1	N-Term(iTRAQ8plex); K6(iTRAQ8plex); K18(iTRAQ8plex)	-	-	-	2.8	-	0	2964.57729	4	741.8997803	-5.279027906	22.09115833
87162455	CLUH	clustered mitochondria protein homolog	NP_056044.3	1.22	2	2	3	1309	146.6	6.13	1.055	2.201	2.833																
														QVSITASTR	2	2	1	N-Term(iTRAQ8plex)	1.174	2.106	2.573	-	61	0	1266.73093	3	422.9151611	-0.790342715	18.89246833
														VLELVLR	1	2	1	N-Term(iTRAQ8plex)	1.055	2.201	2.833	-	18	0	1145.74553	2	573.3764038	-9.112235248	39.61860833
														DIENLK	1	4	2	N-Term(iTRAQ8plex); K6(iTRAQ8plex)	-	-	-	-	28	0	1339.80632	2	670.4067993	1.620169107	18.610635
88703043	SNX6	sorting nexin-6 isoform a	NP_067072.3	17.24	4	4	7	290	33.5	8.57	0.642	0.950	1.356																
														TVAMHEVFLCR	2	3	1	N-Term(iTRAQ8plex); C10(Methylthio)	0.988	1.471	1.565	4.18	40	0	1655.82914	3	552.614563	-4.97429655	35.68265167
														NKDVLQAETSQQLCCQK	1	3	1	N-Term(iTRAQ8plex); K2(iTRAQ8plex); C14(Methylthio); C15(Methylthio); [illegible]	-	0.95	2.477	3.85	-	1	2940.49013	4	735.8779907	-9.433899833	30.22990167
														VAAHPILR	2	3	1	N-Term(iTRAQ8plex)	0.417	0.702	1.175	3.54	48	0	1180.73764	3	394.2507324	-7.755562314	14.63289667
														MKQELEAEYLAIFK	2	8	1	N-Term(iTRAQ8plex); K2(iTRAQ8plex); K14(iTRAQ8plex)	-	-	0.689	2.95	30	1	2625.48574	4	657.1268921	-8.833725617	57.36245333
88853069	VTN	vitronectin precursor	NP_000629.3	3.14	1	1	4	478	54.3	5.80	4.797	2.467	5.140																
														DVWGIEGPIDAAFTR	4	1	1	N-Term(iTRAQ8plex)	4.797	2.467	5.14	4.75	61	0	1951.01421	2	976.0107422	-4.379027567	51.00510167
88999583	MYL6	myosin light polypeptide 6 isoform 2	NP_524147.2	70.20	9	9	54	151	17.0	4.55	0.853	1.575	1.633																
														VLDFEHFLPMLQTVAK	14	2	1	N-Term(iTRAQ8plex); K16(iTRAQ8plex)	0.969	2.219	2.098	5.59	42	0	2496.41617	2	1248.717163	5.014467327	62.36849167
														DQGTYEDYVEGLR	2	2	1	N-Term(iTRAQ8plex)	0.727	1.409	1.633	5.12	47	0	1848.90044	2	924.9538574	4.698427006	31.439625
														ILYSQCGDVMR	10	3	1	N-Term(iTRAQ8plex); C6(Methylthio)	0.878	1.326	1.252	4.18	39	0	1634.79753	2	817.9024048	-1.901978456	40.79935833
														MTEEEVEMLVAGHEDSN GCINYEELVR	1	1	1	N-Term(iTRAQ8plex); C19(Methylthio)	1.41	3.328	3.586	4	-	0	3446.55173	3	1149.522095	-0.28473976	53.23428833
														VLDFEHFLPMLQTVAK	6	2	1	N-Term(iTRAQ8plex); M10(Oxidation); K16(iTRAQ8plex)	-	-	-	3.64	25	0	2512.4103	2	1248.717163	5.014467327	62.36849167
														HVLVTLGEK	5	2	1	N-Term(iTRAQ8plex); K9(iTRAQ8plex)	0.699	1.402	1.536	3.6	41	0	1603.99138	4	401.7514954	-9.364016891	18.79390667
														NKDQGTYEDYVEGLR	5	2	1	N-Term(iTRAQ8plex); K2(iTRAQ8plex)	0.721	1.904	1.999	3.48	35	1	2395.22348	3	799.0771484	-7.564758565	25.18543
														ALGQNPTNAEVLK	2	3	1	N-Term(iTRAQ8plex); K13(iTRAQ8plex)	0.475	1.139	1.213	3.45	45	0	1963.12589	3	655.046814	-8.864767955	19.20049833
														EAFQLFDR	7	2	1	N-Term(iTRAQ8plex)	0.799	1.291	1.41	3.23	29	0	1329.71648	2	665.3551025	-5.680097196	27.24718167
														SDEMNVK	1	2	1	N-Term(iTRAQ8plex); K7(iTRAQ8plex)	1.004	2.081	2.1	-	28	0	1430.76286	2	715.8850708	-9.85230941	13.52225
														SDEMNVK	1	2	1	N-Term(iTRAQ8plex); M4(Oxidation); K7(iTRAQ8plex)	0.955	1.575	1.281	-	23	0	1446.76673	2	715.8850708	-9.85230941	13.52225
90819233	MLLT4	afadin isoform 2	NP_001035089.1	0.73	1	1	1	1651	187.6	6.52	1.094	0.935	0.572																
														SMDAETYVEGQR	1	18	1	N-Term(iTRAQ8plex)	1.094	0.935	0.572	3.35	-	0	1689.80815	2	845.4077148	1.516010853	25.82205667
91208426	PRPF8	pre-mRNA-processing-splicing factor 8	NP_006436.3	18.67	32	32	95	2335	273.4	8.84	1.264	0.885	0.935																
														EELGLIEQAYDNPHEALSR	2	1	1	N-Term(iTRAQ8plex)	0.636	0.786	0.655	7.33	78	0	2488.25846	3	830.0910034	-1.504785841	38.74495
														TEDPDLPAFYFDPLINPISH R	16	1	1	N-Term(iTRAQ8plex)	1.586	1.106	0.866	6.69	67	0	2761.40531	3	921.1399536	-3.133806592	75.473695
														KGMLDPLEVHLLDFPNIVI K	2	1	1	N-Term(iTRAQ8plex); K1(iTRAQ8plex); K20(iTRAQ8plex)	0.343	0.738	1.306	5.39	38	1	3203.8945	3	1068.636353	-1.525310152	55.21997333
														ELGGLGMLSMGHVLIPQS DLR	4	1	1	N-Term(iTRAQ8plex)	1.341	0.973	0.989	5.25	55	0	2527.36429	3	843.1262817	-1.432059149	50.34709833
														DGPYITAEEAVAVYTTTV HWLESR	6	1	1	N-Term(iTRAQ8plex)	-	-	-	5.1	63	0	3012.5524	3	1004.855652	8.866573204	70.151545
														DNPNLLFNMCGFECR	5	1	1	N-Term(iTRAQ8plex); C10(Methylthio); C14(Methylthio)	1.427	1.014	1.249	4.97	75	0	2168.95053	2	1084.980835	8.386530363	60.30522667
														LTLEDLEDSWDR	2	1	1	N-Term(iTRAQ8plex)	1.515	0.885	0.935	4.69	57	0	1795.91521	2	898.4612427	7.571310436	44.94294667
														GMLDPLEVHLLDFPNIVIK	2	1	1	N-Term(iTRAQ8plex); K19(iTRAQ8plex)	1.269	0.425	0.721	4.6	43	0	2771.6041	3	924.5395508	1.817645511	61.787175
														DINLQDEDWNEFNDINK	2	1	1	N-Term(iTRAQ8plex); K17(iTRAQ8plex)	0.854	0.898	0.797	4.53	45	0	2730.33121	3	910.7819214	-5.702747631	32.26608333
														TDMIQALGGVEGILEHTL FK	2	1	1	N-Term(iTRAQ8plex); K20(iTRAQ8plex)	0.959	1.158	1.349	4.42	50	0	2780.55979	3	927.5247803	4.328721996	69.15443667
														ITDAYLDQYLWYEADK	1	1	1	N-Term(iTRAQ8plex); K16(iTRAQ8plex)	0.794	0.895	0.966	4.16	-	0	2615.35123	3	872.4552612	0.863800388	48.343115
														FGNAFHLCR	4	1	1	N-Term(iTRAQ8plex); C8(Methylthio)	1.351	0.838	0.958	4.15	51	0	1414.69214	3	472.2355652	-7.372624084	35.34618333
														FTLWWSPTINR	8	1	1	N-Term(iTRAQ8plex)	1.232	1.065	1.224	4.06	39	0	1724.93589	3	575.6501465	-3.898751791	42.98104833
														YIQPWESEFIDSQR	2	1	1	N-Term(iTRAQ8plex)	1.686	1.13	1.13	4.06	52	0	2102.05693	2	1051.532104	3.459023523	42.55801
														ATEPQMVLFNLYDDWLK	2	1	1	N-Term(iTRAQ8plex); K17(iTRAQ8plex)	0.817	0.806	0.862	4.05	41	0	2691.43576	3	897.8167725	1.658160626	59.52206833
														TNHIYVSSDDIK	2	1	1	N-Term(iTRAQ8plex); K12(iTRAQ8plex)	1.082	1.126	0.863	3.83	27	0	2000.07547	3	667.3633423	-7.708556369	17.43699333
														VESHFDLELR	5	1	1	N-Term(iTRAQ8plex)	1.456	1.092	1.036	3.82	53	0	1548.82578	3	516.9467773	-4.269375446	29.46895
														TAEEVAALIR	3	1	1	N-Term(iTRAQ8plex)	0.989	0.596	1.001	3.75	37	0	1376.79949	2	688.9033813	-4.092988663	37.52390833
														SGMSHEEDQLIPNLYR	2	1	1	N-Term(iTRAQ8plex)	1.333	1.084	1.06	3.7	21	0	2193.08255	3	731.6990356	-3.952252683	32.95748833
														ELGGLGMLSMGHVLIPQS DLR	1	1	1	N-Term(iTRAQ8plex); M7(Oxidation)	0.953	1.046	0.857	3.64	-	0	2543.36203	3	843.1262817	-1.432059149	50.34709833
														QNPFWWTHQR	2	1	1	N-Term(iTRAQ8plex)	0.612	0.647	0.471	3.63	27	0	1703.86649	3	568.6270142	-2.548362538	29.91942667
														TTITEPHHIWPTLTDEEWI K	1	1	1	N-Term(iTRAQ8plex); K20(iTRAQ8plex)	-	-	-	3.31	-	0	3055.61953	4	764.6603394	-5.050942366	39.19106333
														VWAEYALK	2	1	1	N-Term(iTRAQ8plex); K8(iTRAQ8plex)	0.995	0.862	1.193	3.23	27	0	1587.93784	3	529.9841309	1.472367537	35.26214667
														ISLIQIFR	3	1	1	N-Term(iTRAQ8plex)	1.959	0.591	1.288	3.09	51	0	1293.81818	3	431.9442444	-1.121792633	43.01778333
														GSELQLPFQACLK	2	1	1	N-Term(iTRAQ8plex); C11(Methylthio); K13(iTRAQ8plex)	1.323	0.697	0.883	2.99	28	0	2088.14263	2	1044.574951	-0.764591571	42.82596667
														SQEPLPDDDEEFELPEFVEP FLK	1	1	1	N-Term(iTRAQ8plex); K23(iTRAQ8plex)	-	-	-	2.96	-	0	3357.70107	4	840.1807251	4.023351198	63.136435
														LLILALER	2	1	1	N-Term(iTRAQ8plex)	1.304	0.572	0.795	2.83	33	0	1244.82402	2	622.9156494	-0.296501247	47.421465
														YIDRIHIFFR	1	1	1	N-Term(iTRAQ8plex)	1.364	0.98	1.378	2.82	-	1	1683.97194	4	421.7484436	4.91292868	39.98983
														EQSQLTATQTR	2	1	1	N-Term(iTRAQ8plex)	2.035	1.24	0.984	2.82	23	0	1566.83696	2	783.9221191	-1.250697295	15.282495
														EVGIEFMDLYSHLVPVYDV EPLEK	1	1	1	N-Term(iTRAQ8plex); K24(iTRAQ8plex)	-	-	-	2.79	-	0	3429.81655	4	858.2095947	1.530031177	65.745025
														DLILADYGK	2	1	1	N-Term(iTRAQ8plex); K9(iTRAQ8plex)	0.893	0.559	0.693	2.75	31	0	1615.9365	3	539.3170166	-9.316421372	41.85322
														DGPYITAEEAVAVYTTTV HWLESR	1	1	1	N-Term(iTRAQ8plex); Y14(iTRAQ8plex)	-	-	-	-	37	0	3316.73745	3	1004.855652	8.866573204	70.151545
														AVFWDIK	1	1	1	N-Term(iTRAQ8plex); K7(iTRAQ8plex)	1.258	0.802	0.815	-	28	0	1486.88807	3	496.3008728	0.157280301	52.91366667
														RFTLWWSPTINR	1	1	1	N-Term(iTRAQ8plex)	0.752	0.592	0.626	-	28	1	1881.04227	3	627.6856079	-0.776601118	41.197735
														ILLLLR	3	0	1	N-Term(iTRAQ8plex)	-	-	-	-	20	0	1044.74285	2	522.875061	-1.747654646	42.30550667
93588500	AGTRAP	type-1 angiotensin II receptor-associated protein isoform a	NP_065083.3	10.69	1	1	2	159	17.4	6.14	1.325	1.366	1.021																
														GGELLVHTGFLGSSQDR	2	2	1	N-Term(iTRAQ8plex)	1.325	1.366	1.021	3.57	35	0	2077.09494	3	693.036499	-1.48644129	29.06261667
94536771	COA3	cytochrome c oxidase assembly factor 3 homolog, mitochondrial	NP_001035521.1	18.87	2	2	5	106	11.7	9.60	0.666	0.301	0.310																
														FLDELEDEAK	2	1	1	N-Term(iTRAQ8plex); K10(iTRAQ8plex)	0.794	0.356	0.419	4.65	54	0	1816.97515	2	908.9912109	-2.072700339	67.33283333

														LTPEQLHSMR	3	1	1	N-Term(iTRAQ8plex)	0.473	0.238	0.27	3.89	44	0	1515.82221	3	505.9455872	-2.192259338	18.07343167
94538354	PMPCB	mitochondrial-processing peptidase subunit beta precursor	NP_004270.2	12.47	4	4	6	489	54.3	6.83	1.213	0.811	0.610																
														IVLAAAGGVSHDELLDLAK	1	3	1	N-Term(iTRAQ8plex); K19(iTRAQ8plex)	1.213	0.78	0.536	4.02	-	0	2500.45328	3	834.1559448	-2.519574107	48.18365333
														VTCLESGLR	2	3	1	N-Term(iTRAQ8plex); C3(Methylthio)	1.162	0.811	0.61	2.99	25	0	1327.70049	2	664.3538818	-0.817389265	37.36593333
														LCTSVTESEVAR	2	3	1	N-Term(iTRAQ8plex); C2(Methylthio)	1.271	0.845	0.776	2.91	33	0	1644.82195	2	822.9146118	-1.176593729	33.11444333
														AVEILADIIQNSTLGEAEIER	1	3	1	N-Term(iTRAQ8plex)	-	-	-	-	44	0	2588.41568	3	863.4767456	2.751592973	71.7183
94557305	MRPL19	39S ribosomal protein L19, mitochondrial	NP_055578.2	19.18	5	5	11	292	33.5	9.50	1.008	0.757	0.634																
														FDLCLTEQQMK	2	1	1	N-Term(iTRAQ8plex); C4(Methylthio); K11(iTRAQ8plex)	1.046	0.753	0.666	3.82	21	0	2010.0192	3	670.6779175	-6.314919081	67.68522
														WNQPWLEFDMMR	4	1	1	N-Term(iTRAQ8plex)	1.083	0.727	0.591	3.61	35	0	1956.93877	2	978.9730225	-0.773145358	47.29070667
														VLHIPEFYVGSILR	2	1	1	N-Term(iTRAQ8plex)	0.913	1.869	2.059	3.5	23	0	1947.13016	3	649.7149048	-3.492419083	55.07616833
														GLGATFILR	2	1	1	N-Term(iTRAQ8plex)	1.097	0.583	0.359	3.15	27	0	1251.77812	2	626.3927002	4.339803134	33.61825833
														RLDDSLLYLR	1	1	1	N-Term(iTRAQ8plex)	0.738	0.815	1.438	2.98	-	1	1567.90976	3	523.3081055	-0.770544185	30.58598833
94681057	YARS2	tyrosine--tRNA ligase, mitochondrial precursor	NP_001035526.1	18.87	6	6	17	477	53.2	8.98	0.985	0.909	0.682																
														LGLEALAANHQQLFTDGR	4	1	1	N-Term(iTRAQ8plex)	1.125	0.919	0.7	6.37	60	0	2258.21903	3	753.4111938	-0.231601552	35.83843667
														AGHNVIALVGGATAR	4	1	1	N-Term(iTRAQ8plex)	0.785	1.443	0.77	4.18	63	0	1710.98943	2	855.9983521	-1.334794009	25.22186333
														QHLVDFLAAVGGHFR	2	1	1	N-Term(iTRAQ8plex)	-	-	-	4.07	50	0	1971.07888	4	493.525177	-3.953759676	53.321895
														LFTFLPLPEIDHIMQLHVK	1	1	1	N-Term(iTRAQ8plex); K19(iTRAQ8plex)	-	-	-	3.57	-	0	2899.6913	4	725.6782837	6.353636883	66.61101333
														DKTSPFELYQFFVR	2	1	1	N-Term(iTRAQ8plex); K2(iTRAQ8plex)	0.959	1.04	0.645	3.46	-	1	2385.31089	3	795.7751465	1.909563718	54.86507
														IELPELFDR	4	1	1	N-Term(iTRAQ8plex)	0.917	0.881	0.567	3.32	35	0	1435.80449	2	718.4058838	-3.747266364	47.95053667
94721239	IARS	isoleucine--tRNA ligase, cytoplasmic	NP_038203.2	8.32	8	8	22	1262	144.4	6.15	1.083	1.623	1.981																
														FGWDCHGLPVEYEIDK	5	1	1	N-Term(iTRAQ8plex); C5(Methylthio); K16(iTRAQ8plex)	0.991	1.579	1.981	4.8	33	0	2562.25156	3	854.7548828	-4.561269223	45.35627833
														TGTIVVEGHELHDEDIR	2	1	1	N-Term(iTRAQ8plex)	1.144	1.594	1.85	4.68	56	0	2224.14164	3	742.052063	-4.296302449	21.97745667
														KTESAVSQMQSVIELGR	2	1	1	N-Term(iTRAQ8plex); K1(iTRAQ8plex)	1.19	1.369	1.673	4.55	51	1	2471.36734	3	824.4606323	-3.037823318	38.29312333
														LQKEEEIEFLYNENTVR	6	1	1	N-Term(iTRAQ8plex); K3(iTRAQ8plex)	2.161	2.853	5.055	4.51	44	1	2762.46384	3	921.4927979	-6.6248814	37.5407
														GSELEITLTR	2	1	1	N-Term(iTRAQ8plex)	0.996	1.652	2.514	3.91	41	0	1422.81157	2	711.9094238	0.688591708	30.12736667
														LESDYEILER	2	1	1	N-Term(iTRAQ8plex)	0.85	1.539	1.933	3.84	42	0	1570.81658	2	785.9119263	-6.401371803	35.42887333
														VENMVDQLLR	2	1	1	N-Term(iTRAQ8plex)	1.02	0.944	1.725	3.54	24	0	1520.84343	2	760.925354	1.689909533	49.09735333
														LLILMEAR	1	1	1	N-Term(iTRAQ8plex)	1.155	1.943	3.163	3.02	-	0	1262.77898	2	631.8931274	-1.459508924	47.41140333
94721252	VAPA	vesicle-associated membrane protein-associated protein A isoform 2	NP_919415.2	34.54	5	6	20	249	27.9	8.62	1.288	0.543	0.571																
														FMVQTIFAPPNTSDMEAVWK	2	4	1	N-Term(iTRAQ8plex); M2(Oxidation); K20(iTRAQ8plex)	1.27	0.64	0.841	5.07	48	0	2936.52921	3	979.5145874	4.925085365	50.35310167
														HEQILVLDPPTDLK	10	2	1	N-Term(iTRAQ8plex); K14(iTRAQ8plex)	1.261	0.432	0.545	4.98	31	0	2226.28543	3	742.7667847	-4.330327261	30.12026167
														FKGPFTDVVTTNLK	1	2	1	N-Term(iTRAQ8plex); K2(iTRAQ8plex); K14(iTRAQ8plex)	1.815	0.876	0.512	3.82	-	1	2479.47622	4	620.6245117	3.002930598	36.30256167
														CVFEMPNENDKLNDMEPSK	1	2	1	C1(Methylthio); N-Term(iTRAQ8plex); K11(iTRAQ8plex); M15(Oxidation); K19(iTRAQ8plex)	3.447	0.968	2.055	3.7	-	1	3214.54995	4	804.3929443	-4.887102862	31.41772333
														QDGPMPKPHSVSLNDTETR	1	4	1	N-Term(iTRAQ8plex); M5(Oxidation); K7(iTRAQ8plex)	-	-	-	3.61	-	0	2733.39858	4	680.1067505	-3.160819359	17.31529167
														QDGPMPKPHSVSLNDTETR	2	4	1	N-Term(iTRAQ8plex); K7(iTRAQ8plex)	0.764	-	-	3.37	26	0	2717.40517	4	680.1067505	-3.160819359	17.31529167
														CVFEMPNENDKLNDMEPSK	1	2	1	N-Term(iTRAQ8plex); C1(Methylthio); K11(iTRAQ8plex); K19(iTRAQ8plex)	2.278	1.302	0.253	3.36	-	1	3198.5676	4	804.3929443	-4.887102862	31.41772333
														GPFTDVVTTNLK	2	4	2	N-Term(iTRAQ8plex); K12(iTRAQ8plex)	-	-	-	3.86	51	0	1900.09343	2	950.550354	-3.468204289	26.99260833
98986464	TMED10	transmembrane emp24 domain-containing protein 10 precursor	NP_006818.3	25.11	5	5	50	219	25.0	7.44	0.822	0.802	0.568																
														RLEDLSESIVNDFAYMK	27	1	1	N-Term(iTRAQ8plex); K17(iTRAQ8plex)	0.783	0.908	0.719	5.28	45	1	2638.40421	3	880.1397705	1.526291	59.06466833
														RLEDLSESIVNDFAYMK	5	1	1	N-Term(iTRAQ8plex); M16(Oxidation); K17(iTRAQ8plex)	0.99	0.543	0.737	4.91	36	1	2654.39719	3	880.1397705	1.526291	59.06466833
														IPDQLVILDMK	10	1	1	N-Term(iTRAQ8plex); K11(iTRAQ8plex)	0.868	0.652	0.501	4.63	42	0	1893.14861	2	947.0779419	7.725684612	42.78126
														ITDSAGHILYSK	2	1	1	N-Term(iTRAQ8plex); K12(iTRAQ8plex)	0.895	1.017	0.661	3.27	26	0	1913.09543	4	479.0284424	-1.731045268	24.436735
														HGVEAK	2	1	1	N-Term(iTRAQ8plex); K6(iTRAQ8plex)	0.826	0.791	0.594	2.92	25	0	1248.75437	3	416.9229736	1.854927196	9.312546667
														LKPLEVELR	3	1	1	N-Term(iTRAQ8plex); K2(iTRAQ8plex)	0.716	0.66	0.516	2.77	25	0	1705.08828	4	427.0275269	2.947789825	37.02704167
														IPDQLVILDMK	1	1	1	N-Term(iTRAQ8plex); M10(Oxidation); K11(iTRAQ8plex)	0.598	0.632	0.665	2.74	-	0	1909.11435	2	947.0779419	7.725684612	42.78126
100913206	DHX9	ATP-dependent RNA helicase A	NP_001348.2	35.04	35	35	248	1270	140.9	6.84	1.276	0.821	0.817																
														NELMYQLEQDHDLQAILQER	18	1	1	N-Term(iTRAQ8plex)	1.255	1.072	0.939	6.86	47	0	2790.41635	3	936.1389771	1.422320041	38.53405
														NELMYQLEQDHDLQAILQER	6	1	1	N-Term(iTRAQ8plex); M4(Oxidation)	1.7	1.024	0.831	6.72	64	0	2806.40238	3	936.1389771	1.422320041	38.53405
														AIEPPPLDAVIEAEHTLR	36	1	1	N-Term(iTRAQ8plex)	1.532	1.063	0.989	6.56	81	0	2275.24912	2	1138.135986	2.050194977	41.51135833
														VQSDGQIVLVDDWIK	2	1	1	N-Term(iTRAQ8plex); K15(iTRAQ8plex)	1.092	0.696	0.671	6.09	49	0	2323.32218	3	775.1122437	4.453281224	42.44933333
														KFESEILEAISQNSVVIIR	6	1	1	N-Term(iTRAQ8plex); K1(iTRAQ8plex)	1.669	0.706	1.092	6.01	69	1	2783.61197	3	928.5423584	-0.093171979	62.25008333
														KEEQEVQATLESEEVDLNAGLHGNWTLENAK	2	1	1	N-Term(iTRAQ8plex); K1(iTRAQ8plex); K31(iTRAQ8plex)	-	-	-	5.91	-	1	4394.29395	5	879.6646118	1.354318144	51.26984833
														FSDHVALLSVFQAWDDAR	11	1	1	N-Term(iTRAQ8plex)	0.851	1.166	1.383	5.83	67	0	2381.21354	3	794.4093628	-2.38792994	59.66839
														LQISHEAAACITGLR	20	1	1	N-Term(iTRAQ8plex); C10(Methylthio)	1.337	0.707	0.706	5.04	63	0	1933.03411	2	967.0181274	-0.602182999	43.60511333
														GISHVIVDEIHER	19	1	1	N-Term(iTRAQ8plex)	1.118	0.929	0.876	5.02	54	0	1807.99224	3	603.335022	-3.500367943	25.95132167
														TPLHEIALSIK	10	1	1	N-Term(iTRAQ8plex); K11(iTRAQ8plex)	1.448	0.921	0.715	4.95	48	0	1830.1237	2	915.5654907	-3.94316224	29.97921167
														AAMEALVVEVTK	7	1	1	N-Term(iTRAQ8plex); K12(iTRAQ8plex)	1.126	0.712	0.804	4.89	63	0	1869.09136	2	935.0493164	-3.341253213	37.45997833
														ELDALDANDELTPLGR	4	1	1	N-Term(iTRAQ8plex)	1.28	1.11	1.025	4.85	59	0	2046.06914	2	1023.538208	1.66079218	37.637605
														QLYHLGVVEAYSGLTK	2	1	1	N-Term(iTRAQ8plex); K16(iTRAQ8plex)	1.127	0.671	1.385	4.67	28	0	2386.35495	3	796.1231689	-1.729715043	36.02525333
														AAECNIVVTQPR	6	1	1	N-Term(iTRAQ8plex); C4(Methylthio)	1.348	0.777	0.821	4.66	63	0	1650.85698	3	550.9597168	2.213894847	30.13376
														LGGIGQFLAK	4	1	1	N-Term(iTRAQ8plex); K10(iTRAQ8plex)	1.336	0.602	0.626	4.63	45	0	1611.99675	2	806.5020142	-4.664773474	66.12827667
														KVQSDGQIVLVDDWIK	2	1	1	N-Term(iTRAQ8plex); K1(iTRAQ8plex); K16(iTRAQ8plex)	1.164	0.889	0.872	4.48	40	1	2755.61423	3	919.2095947	0.751059297	39.31022833
														GMTLVTPLQLLFASK	13	1	1	N-Term(iTRAQ8plex); K16(iTRAQ8plex)	1.345	0.709	0.911	4.46	44	0	2340.42423	2	1170.717285	3.735383075	62.706295
														DINTDFLLVVLR	4	1	1	N-Term(iTRAQ8plex)	1.15	0.681	0.724	4.26	59	0	1722.017	3	574.6771851	3.850499602	56.48684333
														YQILPLHSQIPR	8	1	1	N-Term(iTRAQ8plex)	0.854	0.654	0.715	4.16	55	0	1769.03501	3	590.3498535	-1.443726729	29.316475
														HLEMNPHFGSHR	2	1	1	N-Term(iTRAQ8plex)	1.78	0.969	1.093	4.16	29	0	1765.87522	5	353.9808655	-6.014251345	16.44912833
														LAAQSCALSLVR	4	1	1	N-Term(iTRAQ8plex); C6(Methylthio)	1.008	0.782	1.255	4.15	54	0	1581.88016	3	527.9649048	2.717529162	39.452925
														TTQVPQFILDDFIQNDR	4	1	1	N-Term(iTRAQ8plex)	0.623	0.97	0.5	3.99	71	0	2354.22599	3	785.4135132	-1.467573308	56.16691333
														GMTLVTPLQLLFASK	4	1	1	N-Term(iTRAQ8plex); M2(Oxidation); K16(iTRAQ8plex)	1.743	0.668	0.788	3.87	39	0	2356.4152	2	1170.717285	3.735383075	62.706295
														VFDPVPVGVTK	2	1	1	N-Term(iTRAQ8plex); K11(iTRAQ8plex)	1.27	0.806	1.226	3.84	37	0	1766.05657	2	883.5319214	-6.061610238	26.35113833
														YPSPFFVFGEK	9	1	1	N-Term(iTRAQ8plex); K11(iTRAQ8plex)	1.348	0.8	0.741	3.75	47	0	1926.04936	2	963.5283203	-6.649515448	69.96847333
														HLEMNPHFGSHR	3	1	1	N-Term(iTRAQ8plex); M4(Oxidation)	1.327	0.634	0.872	3.57	23	0	1781.87589	5	353.9808655	-6.014251345	16.44912833
														LNQYFQK	5	1	1	N-Term(iTRAQ8plex); K7(iTRAQ8plex)	1.579	0.954	0.764	3.46	40	0	1548.88835	2	774.9478149	-7.152250299	29.10902833
														TYPVQEYFLEDCIQMTHFVPPPK	2	1	1	N-Term(iTRAQ8plex); C12(Methylthio); K23(iTRAQ8plex)	1.581	0.788	0.413	3.45	-	0	3436.72402	4	859.9364624	-0.012386978	66.95677167
														VRPGFCFHLCSR	7	1	1	N-Term(iTRAQ8plex); C6(Methylthio); C10(Methylthio)	0.949	0.883	0.767	3.36	34	0	1817.87002	4	455.2229614	-2.066966748	40.45080333
														LETHMTPEMFR	3	1	1	N-Term(iTRAQ8plex)	1.218	0.956	1.065	3.35	36	0	1695.84416	2	848.4257202	-3.471716587	29.51297167
														ISAVSVAER	3	1	1	N-Term(iTRAQ8plex)	1.213	0.77	0.713	3.09	38	0	1235.71929	2	618.3632813	-5.539623827	16.80139833
														KLAAQSCALSLVR	3	1	1	N-Term(iTRAQ8plex); K1(iTRAQ8plex); C7(Methylthio)	1.067	0.724	0.57	3.06	28	1	2014.168	3	672.062439	-1.702264836	35.88431167
														MGGEEAEIR	1	1	1	N-Term(iTRAQ8plex)	1.415	0.801	0.759	3.03	-	0	1295.65386	2	656.3284912	-1.494681176	14.460095
														DVVQAYPEVR	1	1	1	N-Term(iTRAQ8plex)	0.82	0.614	0.654	3.01	-	0	1479.80803	2	740.4076538	-1.953160648	22.58165667
														YTQVGPDHNR	1	1	1	N-Term(iTRAQ8plex)	1.093	0.824	0.926	2.95	-	0	1490.76029	3	497.5916138	-3.396415538	13.77400333
														DFVNYLVR	9	1	1	N-Term(iTRAQ8plex)	1.394	0.806	0.815	2.91	44	0	1329.74126	2	665.3730469	-6.041915823	28.83697833
														LAQFEPSQR	1	1	1	N-Term(iTRAQ8plex)	1.208	1.463	0.966	2.72	-	0	1379.75249	2	690.3798828	-4.350269376	19.53078833
														MGGEEAEIR	3	1	1	N-Term(iTRAQ8plex); M1(Oxidation)	1.441	0.646	0.674	-	25	0	1311.64971	2	656.3284912	-1.494681176	14.460095
														LPIEPR	1	1	1	N-Term(iTRAQ8plex)	1.33	0.681	0.586	-	14	0	1028.63774	2	514.8225098	-2.778809906	22.086795
102467242	INCENP	inner centromere protein isoform 2	NP_064623.2	1.64	1	1	1	914	104.9	9.45	3.678	2.534	1.938																
														TDQADGPREPPQSAR	1	7	1	N-Term(iTRAQ8plex)	3.678	2.534	1.938	-	21	1	1928.96463	3	643.659729	-4.249708957	9.448136667
103471997	POLR1A	DNA-directed RNA polymerase I subunit RPA1	NP_056240.2	0.64	1	1	6	1720	194.7	7.03	0.891	0.908	2.525																
														AVIHLLLCQLR	6	1	1	N-Term(iTRAQ8plex); C8(Methylthio)	0.891	0.908	2.525	2.86	39	0	1628.9683	3	543.6609497	2.261290845	57.47211
105990539	NEFL	neurofilament light polypeptide	NP_006149.2	5.71	2	3	33	543	61.5	4.65	0.127	0.083	0.067																
														IDSLMDEISFLK	3	1	1	N-Term(iTRAQ8plex); K12(iTRAQ8plex)	0.159	-	0.052	4.03	43	0	2019.13547	3	673.7166748	3.060648555	53.9497
														VLEAELLVLR	2	1	1	N-Term(iTRAQ8plex)	0.101	0.083	0.086	3.07	29	0	1458.92607	2	729.9666748	4.326257164	49.50984
														EYQDLLNVK	27	8	2	N-Term(iTRAQ8plex); K9(iTRAQ8plex)	-	-	-	4.3	64	0	1729.99065	2	865.4995117	-1.574852889	41.383425
														EYQDLLNVK	1	8	2	N-Term(iTRAQ8plex); Y2(iTRAQ8plex); K9(iTRAQ8plex)	-	-	-	-	25	0	2034.20366	2	865.4995117	-1.574852889	41.383425
106879206	C14orf169	bifunctional lysine-specific demethylase and histidyl-hydroxylase NO66	NP_078920.2	2.18	1	1	3	641	71.0	6.46	1.056	0.629	0.832																
														NEEVQFGQHLDAAR	3	1	1	N-Term(iTRAQ8plex)	1.056	0.629	0.832	4.6	37	0	1917.9664	3	639.9936523	-2.965390186	21.27094833
108773782	ABCE1	ATP-binding cassette sub-family E member 1	NP_002931.2	1.67	1	1	2	599	67.3	8.34	1.036	1.379	1.065																
														VIVFDGVPSK	2	1	1	N-Term(iTRAQ8plex); K10(iTRAQ8plex)	1.036	1.379	1.065	3.55	28	0	1669.002	2	835.0046387	-7.489850968	26.22822
108773793	G6PD	glucose-6-phosphate 1-dehydrogenase isoform b	NP_001035810.1	25.63	11	11	31	515	59.2	6.84	0.622	2.120	2.658																
														KPGMFFNPEESELDLTYGNR	2	2	1	N-Term(iTRAQ8plex); K1(iTRAQ8plex)	-	2.47	2.508	4.79	41	0	2952.48905	3	984.8345337	-4.47198129	40.04695333
														VGFQYEGTYK	5	2	1	N-Term(iTRAQ8plex); K10(iTRAQ8plex)	0.529	1.953	2.658	4.46	45	0	1799.96599	2	900.4866333	-7.123089275	34.01754667
														LFYLALPPTVYEAVTK	8	2	1	N-Term(iTRAQ8plex); K16(iTRAQ8plex)	0.625	1.705	1.97	4.36	50	0	2433.44052	3	811.8183594	6.22311285	60.192415
														KPGMFFNPEESELDLTYGNR	2	2	1	N-Term(iTRAQ8plex); K1(iTRAQ8plex); M4(Oxidation)	-	1.64	1.826	4.05	31	0	2968.49265	3	984.8345337	-4.47198129	40.04695333
														EMVQNLMVLR	2	2	1	N-Term(iTRAQ8plex)	0.619	2.277	3.139	3.94	36	0	1536.8593	2	768.9332886	3.181311676	37.94171167
														LQFHDVAGDIFHQQCK	3	2	1	N-Term(iTRAQ8plex); C15(Methylthio); K16(iTRAQ8plex)	1.031	1.643	1.778	3.33	-	0	2540.28164	4	635.8258667	-7.186361882	41.40307667
														LNSHMNALHLGSQANR	1	2	1	N-Term(iTRAQ8plex)	0.971	1.034	1.661	3.3	-	0	2067.06753	4	517.5223389	-6.998180307	26.71523667
														IIVEKPFGR	1	2	1	N-Term(iTRAQ8plex); K5(iTRAQ8plex)	0.598	2.327	2.911	2.91	-	0	1667.04326	2	834.0252686	-1.926051172	22.9163
														LKLEDFFAR	4	2	1	N-Term(iTRAQ8plex); K2(iTRAQ8plex)	1.235	4.816	7.54	2.84	46	1	1747.03042	4	437.5130615	-3.368622661	47.23814333
														WDGVPFILR	1	2	1	N-Term(iTRAQ8plex)	0.581	2.366	3.397	2.81	-	0	1406.79912	2	703.9031982	-7.599831045	70.44096833
														GYLDDPTVPR	1	2	1	N-Term(iTRAQ8plex)	0.554	0.969	1.166	-	27	0	1436.76274	2	718.8850098	-4.161013208	23.85687333
														LPDAYER	1	2	1	N-Term(iTRAQ8plex)	0.894	2.12	2.832	-	20	0	1167.62248	2	584.3148804	-7.422928156	19.61008667
109148508	OTUB1	ubiquitin thioesterase OTUB1	NP_060140.2	9.23	2	2	21	271	31.3	4.94	0.912	2.514	2.194																
														AFGFSHLEALLDDSK	6	1	1	N-Term(iTRAQ8plex); K15(iTRAQ8plex)	0.873	1.919	1.525	4.82	36	0	2258.22207	3	753.4113159	-3.705041288	49.89455833
														FFEHFIEGGR	15	1	1	N-Term(iTRAQ8plex)	0.987	2.699	2.622	3.85	45	0	1542.79813	3	514.9358521	-4.989909626	40.90275333
109148542	AARS	alanine--tRNA ligase, cytoplasmic	NP_001596.2	19.63	13	13	37	968	106.7	5.53	0.945	2.919	4.118																

														DNFWEMGDTGPCGPCSEIHYDR	2	1	1	N-Term(iTRAQ8plex); C12(Methylthio); C15(Methylthio)	-	1.82	3.773	6.55	79	0	2925.16495	3	975.7265015	-2.178391843	51.14033167
														ASEWVQQVSGLMDGK	2	1	1	N-Term(iTRAQ8plex); K15(iTRAQ8plex)	0.915	3.686	4.447	5.72	63	0	2243.17472	3	748.3964233	-9.08289948	74.023185
														GLVVDMDGFEEER	2	1	1	N-Term(iTRAQ8plex)	1.11	5.315	8.694	5.29	50	0	1799.88494	2	900.446106	3.413548245	35.68703167
														VGDQVWLFIDEPR	6	1	1	N-Term(iTRAQ8plex)	0.954	3.831	6.704	5.24	73	0	1878.00334	2	939.5053101	-1.606920895	52.29433667
														VGAEDADGIDMAYR	2	1	1	N-Term(iTRAQ8plex)	1.326	4.904	7.609	4.71	65	0	1786.86553	2	893.9364014	3.998518227	28.54458833
														IVAVTGAEAQK	2	1	1	N-Term(iTRAQ8plex); K11(iTRAQ8plex)	0.927	1.429	2.279	4.69	44	0	1695.02048	3	565.6783447	-3.333511527	27.51049667
														EIADLGEALATAVIPQWQK	4	1	1	N-Term(iTRAQ8plex); K19(iTRAQ8plex)	1.196	2.169	4.118	4.64	94	0	2661.5269	2	1331.26709	7.37988689	63.711385
														TITVALADGGRPDNTGR	2	1	1	N-Term(iTRAQ8plex)	0.868	2.919	4.19	3.97	44	0	2018.0833	2	1009.545288	-4.951107879	22.14828167
														QIWQNLGLDDTK	2	1	1	N-Term(iTRAQ8plex); K12(iTRAQ8plex)	5.686	3.596	3.965	3.94	44	0	2039.12546	3	680.3800049	-6.247728158	60.35269333
														AVFDETYPDPVR	6	1	1	N-Term(iTRAQ8plex)	0.811	2.035	2.712	3.94	42	0	1712.88152	2	856.944397	1.036864076	30.851825
														MSNYDTDLFVPYFEAIQK	2	1	1	N-Term(iTRAQ8plex); K18(iTRAQ8plex)	0.464	6.355	12.21	3.82	65	0	2789.44048	2	1395.223877	3.153315628	53.54449833
														SIDTGMGLER	2	1	1	N-Term(iTRAQ8plex)	1.506	3.257	5.383	3.81	34	0	1382.72771	2	691.8674927	1.842323363	25.91381667
														ITCLCQVPQNAANR	2	1	1	N-Term(iTRAQ8plex); C3(Methylthio); C5(Methylthio)	1.342	1.224	1.498	3.42	49	0	1926.92722	3	642.9805908	-2.695529447	48.70796333
														MSNYDTDLFVPYFEAIQK	1	1	1	N-Term(iTRAQ8plex); M1(Oxidation); K18(iTRAQ8plex)	-	-	-	3.12	-	0	2805.42276	2	1395.223877	3.153315628	53.54449833
109148552	KRT3	keratin, type II cytoskeletal 3	NP_476429.2	8.76	1	8	240	628	64.4	6.48	0.185	0.077	0.138																
														NMEDLVEDFK	13	1	1	N-Term(iTRAQ8plex); K10(iTRAQ8plex)	0.162	0.032	0.075	4.07	60	0	1847.9616	2	924.4844971	-2.843013102	66.18254
														NMEDLVEDFK	3	1	1	N-Term(iTRAQ8plex); M2(Oxidation); K10(iTRAQ8plex)	0.421	0.216	0.191	3.55	25	0	1863.95745	2	924.4844971	-2.843013102	66.18254
														LALDVEIATYR	117	10	7	N-Term(iTRAQ8plex)	-	-	-	4.83	67	0	1567.90409	3	523.3062134	2.771589637	39.12087
														LALDVEIATYR	8	10	7	N-Term(iTRAQ8plex); Y10(iTRAQ8plex)	-	-	-	-	60	0	1872.106	3	523.3062134	2.771589637	39.12087
														TAAENEFVTLK	10	4	3	N-Term(iTRAQ8plex); K11(iTRAQ8plex)	-	-	-	4.72	51	0	1831.03826	2	916.0227661	-2.1385099	38.95932833
														TAAENEFVTLKK	2	4	3	N-Term(iTRAQ8plex); K11(iTRAQ8plex); K12(iTRAQ8plex)	-	-	-	4.09	34	1	2263.34414	4	566.5914917	0.722654964	26.116015
														FLEQQNK	13	31	8	N-Term(iTRAQ8plex); K7(iTRAQ8plex)	-	-	-	-	33	0	1514.87885	3	505.631134	0.090799082	15.97476833
														FASFIDKVR	16	27	8	N-Term(iTRAQ8plex); K7(iTRAQ8plex)	-	-	-	3.33	31	1	1691.01076	4	423.5080261	0.114317571	39.05999667
														FASFIDK	57	28	8	N-Term(iTRAQ8plex); K7(iTRAQ8plex)	-	-	-	3.23	35	0	1435.83818	2	718.4240723	0.227470368	33.40336167
														VELQAK	1	16	4	N-Term(iTRAQ8plex); K6(iTRAQ8plex)	-	-	-	2.75	-	0	1295.81623	3	432.61026	1.470485751	22.94674667
109288010	SLC16A3	monocarboxylate transporter 4	NP_001035888.1	11.61	5	5	9	465	49.4	7.96	0.471	1.021	0.402																
														LHKPPADSGVDLR	2	1	1	N-Term(iTRAQ8plex); K3(iTRAQ8plex)	0.388	1.176	0.707	3.44	-	0	2013.16994	4	504.0479431	-0.118578694	15.26543333
														EVEHFLK	2	1	1	N-Term(iTRAQ8plex); K7(iTRAQ8plex)	0.565	1.063	0.323	3.13	36	0	1509.89006	2	755.4486694	1.000700134	19.74633167
														EPQPEVAAAEEEK	2	1	1	N-Term(iTRAQ8plex); K13(iTRAQ8plex)	0.545	1.092	0.817	3.1	30	0	2035.0748	3	679.0297852	-2.766031619	16.48551833
														LLDLSVFR	2	1	1	N-Term(iTRAQ8plex)	0.385	0.746	0.216	2.7	24	0	1266.77178	2	633.8895264	-0.454056236	51.99992333
														GGAVVDEGPTGVK	1	1	1	N-Term(iTRAQ8plex); K13(iTRAQ8plex)	0.343	0.915	0.162	-	47	0	1794.02048	3	598.6783447	-0.730409896	15.36768
109452587	FAM98B	protein FAM98B	NP_775882.2	15.01	4	4	7	433	45.5	8.69	1.115	1.250	1.269																
														GGRPNEIEPPPPEMPPWQK	2	3	1	N-Term(iTRAQ8plex); K19(iTRAQ8plex)	1.158	1.029	1.099	4.76	27	0	2764.46793	3	922.1608276	-0.814493024	28.01348167
														MDLNSEQAEQLER	2	1	1	N-Term(iTRAQ8plex)	1.073	1.545	1.065	3.98	47	0	1866.92302	2	933.9651489	3.251302541	27.1898
														NSEVYQEVQAMFDTLGIPK	2	1	1	N-Term(iTRAQ8plex); K19(iTRAQ8plex)	0.662	1.275	1.467	3.9	34	0	2777.46146	3	926.4920044	-0.936598561	58.23933333
														RLDVTVQSFGWSDR	1	3	1	N-Term(iTRAQ8plex)	1.167	1.225	1.714	-	26	1	1970.03629	3	657.3502808	-1.772690617	32.20392167
109452591	SUCLG1	succinyl-CoA ligase [ADP/GDP-forming] subunit alpha, mitochondrial precursor	NP_003840.2	19.36	5	5	15	346	36.2	8.79	0.796	0.657	0.745																
														LIGPNCPGVINPGECK	6	1	1	N-Term(iTRAQ8plex); C6(Methylthio); C15(Methylthio); K16(iTRAQ8plex)	0.693	0.505	0.68	5.57	57	0	2311.21025	2	1156.108765	9.124819713	40.875645
														GGQTHLGLPVFNTVK	4	1	1	N-Term(iTRAQ8plex); K15(iTRAQ8plex)	0.983	0.616	0.638	4.37	44	0	2176.26871	3	726.0944214	-0.542391589	29.71005
														QGTFHSQQALEYGTK	2	1	1	N-Term(iTRAQ8plex); K15(iTRAQ8plex)	0.78	0.697	0.884	3.62	28	0	2303.21262	3	768.4090576	-4.959029114	16.35571167
														IICQGFTGK	1	1	1	N-Term(iTRAQ8plex); C3(Methylthio); K9(iTRAQ8plex)	0.812	0.619	0.833	3.06	-	0	1620.90581	2	810.956543	-0.242446228	52.70717667
														MGHAGAIIAGGK	2	1	1	N-Term(iTRAQ8plex); M1(Oxidation); K12(iTRAQ8plex)	1.362	1.272	0.725	2.68	42	0	1706.97124	2	853.9892578	-7.028410746	14.82263
109637788	PML	protein PML isoform 5	NP_150247.2	23.57	10	10	22	560	62.0	6.42	2.172	0.497	0.381																
														CYASDQEVLDMHGFLR	2	9	1	N-Term(iTRAQ8plex); C1(Methylthio)	2.304	1.069	0.928	6.05	67	0	2234.03446	3	745.3496704	-0.082505232	40.95217
														QEELDAMTQALQEQDSAFGAVHAQMHAAVGQLGR	2	9	1	N-Term(iTRAQ8plex)	-	-	-	4.43	22	0	3941.94546	4	986.2418213	7.027995837	54.365935
														APASEEEFQFLR	3	9	1	N-Term(iTRAQ8plex)	2.233	0.505	0.337	4.25	58	0	1727.8958	2	864.4515381	2.979492095	33.90935167
														SSPEQPRPSTSK	1	7	1	N-Term(iTRAQ8plex); K12(iTRAQ8plex)	2.758	0.796	0.295	3.98	-	0	1909.05495	4	478.0191956	-2.613310545	9.801941667
														CFEAHQWFLK	5	9	1	N-Term(iTRAQ8plex); C1(Methylthio); K10(iTRAQ8plex)	2.012	0.364	0.278	3.79	27	0	1963.01528	3	655.0106201	-0.293017507	41.28962667
														TPTLTSIYCR	2	9	1	N-Term(iTRAQ8plex); C9(Methylthio)	2.311	0.662	0.553	3.71	22	0	1504.78594	2	752.8966064	3.591188949	33.18619667
														ELLEAVDAR	2	9	1	N-Term(iTRAQ8plex)	1.99	0.625	0.605	3.38	26	0	1319.7487	2	660.3779907	1.086210812	31.73300167
														AETEELIR	2	9	1	N-Term(iTRAQ8plex)	1.833	0.641	0.469	2.69	29	0	1264.70207	2	632.8546753	-2.362085209	21.32939167
														LRQEEPQSLQAAVR	2	9	1	N-Term(iTRAQ8plex)	2.339	0.391	0.544	-	28	1	1929.07779	3	643.6974487	-2.171171432	22.19663833
														LDAVLQR	1	9	1	N-Term(iTRAQ8plex)	2.065	0.469	0.385	-	23	0	1118.6812	2	559.8442383	-2.084023134	30.49421667
109948283	PUM3	pumilio homolog 3	NP_055693.4	5.56	2	2	2	648	73.5	9.64	-	-	-																
														QIIISEIISSLPSIVNDKYGR	1	1	1	N-Term(iTRAQ8plex); K18(iTRAQ8plex)	-	-	-	2.89	-	1	2953.72011	4	739.1854858	0.613524405	67.626645
														QAFEELRDDLVELSK	1	1	1	N-Term(iTRAQ8plex); K15(iTRAQ8plex)	-	-	-	-	26	1	2400.31533	4	600.8342896	-3.250813251	54.65059167
109948304	SMU1	WD40 repeat-containing protein SMU1	NP_060695.2	7.80	3	3	5	513	57.5	7.18	1.139	1.092	0.952																
														DSSQILSASFDQTIR	2	2	1	N-Term(iTRAQ8plex)	1.397	1.05	0.977	4.63	36	0	1972.03435	2	986.520813	2.74744533	34.00249667
														SNTVVIMNMQGQIVR	1	2	1	N-Term(iTRAQ8plex)	0.706	0.718	0.928	3.85	-	0	1994.08928	2	997.5482788	3.189274045	32.55991167
														YIHLENLLAR	1	1	1	N-Term(iTRAQ8plex)	1.205	2.596	2.161	3.26	-	0	1545.89811	4	463.2863464	6.872277339	33.86899167
														YIHLENLLAR	1	1	1	N-Term(iTRAQ8plex); Y1(iTRAQ8plex)	1.077	1.136	0.909	-	32	0	1850.12356	4	463.2863464	6.872277339	33.86899167
110227846	C14orf142	uncharacterized protein C14orf142	NP_115879.2	18.00	1	1	2	100	10.9	4.27	0.612	0.759	0.884																
														DMVTELFDPLVQGEVQHR	2	1	1	N-Term(iTRAQ8plex)	0.612	0.759	0.884	4.98	52	0	2417.24631	3	806.4202881	1.067209329	50.01180333
110347427	USP34	ubiquitin carboxyl-terminal hydrolase 34	NP_055524.3	0.28	1	1	1	3546	404.0	5.82	0.250	0.197	0.258																
														VAVDDSESLR	1	1	1	N-Term(iTRAQ8plex)	0.25	0.197	0.258	3.04	-	0	1394.74077	2	697.8740234	-1.535084544	22.83743333
110611218	RRBP1	ribosome-binding protein 1	NP_001036041.1	7.68	6	6	16	977	108.6	5.54	0.691	0.504	0.797																
														TAGPLESSETEEASQLK	2	1	1	N-Term(iTRAQ8plex); K17(iTRAQ8plex)	0.465	0.308	0.754	7.42	97	0	2385.2625	2	1193.134888	0.803882435	27.58931833
														QLLLESQSQLDAAK	2	1	1	N-Term(iTRAQ8plex); K14(iTRAQ8plex)	0.691	0.782	1.114	6.41	48	0	2152.25809	3	718.0908813	6.825154024	32.17058
														QVLQLQASHR	2	1	1	N-Term(iTRAQ8plex)	0.551	0.504	0.78	4.24	65	0	1483.86527	3	495.2932739	0.395741686	16.077655
														SILAETEGMLR	4	1	1	N-Term(iTRAQ8plex)	0.616	0.453	0.903	4.03	43	0	1523.84087	2	762.4240723	0.220896589	39.71030167
														IPDHDPAPNVTVLLR	5	1	1	N-Term(iTRAQ8plex)	1.192	0.624	0.656	3.97	40	0	1961.10733	3	654.3739624	-2.492134359	34.63695667
														HLEEIVEK	1	1	1	N-Term(iTRAQ8plex); K8(iTRAQ8plex)	0.843	0.713	1.004	3.37	-	0	1604.93181	2	802.9695435	-9.340587458	17.98720167
														IQEEIEK	2	1	1	N-Term(iTRAQ8plex); K7(iTRAQ8plex)	0.649	0.5	0.349	2.91	37	0	1496.88042	2	748.9438477	1.581550851	19.18573
112293277	DNAJC8	dnaJ homolog subfamily C member 8	NP_055095.2	22.13	3	3	5	253	29.8	9.06	1.597	2.719	3.543																
														EGKPTIVEEDDPELFK	2	1	1	N-Term(iTRAQ8plex); K3(iTRAQ8plex); K16(iTRAQ8plex)	1.372	2.162	2.73	4.75	44	0	2758.50345	3	920.1726685	-8.837827949	30.52902333
														QREEEIEAQEK	2	1	1	N-Term(iTRAQ8plex); K11(iTRAQ8plex)	1.859	3.42	4.599	3.62	39	1	1997.05735	3	666.3572998	-9.341312093	12.89909167
														LTRPGSSYFNLNPFEVLQIDPEVTDEEIK	1	1	1	N-Term(iTRAQ8plex); K29(iTRAQ8plex)	-	-	-	2.84	-	0	3959.10268	4	990.5311279	3.218036958	54.143915
112382252	SPTBN1	spectrin beta chain, non-erythrocytic 1 isoform 2	NP_842565.2	8.86	15	15	40	2155	251.2	5.53	0.727	0.665	1.118																
														DVSSVELLMNNHQGIK	2	3	1	N-Term(iTRAQ8plex); K16(iTRAQ8plex)	0.944	0.98	1.47	5.17	41	0	2392.29764	3	798.1040649	-5.797658929	31.22767333
														HTLVEADIGIQAER	6	3	1	N-Term(iTRAQ8plex)	0.727	0.592	0.859	4.95	82	0	1856.00992	3	619.3422852	-3.05982953	23.91470833
														HQILEQAVEDYAETVHQLSK	2	3	1	N-Term(iTRAQ8plex); K20(iTRAQ8plex)	-	-	-	4.72	38	0	2946.56484	4	737.3966675	-4.520945185	53.90046333
														ALVADSHPESER	8	3	1	N-Term(iTRAQ8plex)	0.764	0.791	1.184	4.55	44	0	1614.83438	3	538.949646	-2.821689804	13.24769
														EQWANLEQLSAIR	2	3	1	N-Term(iTRAQ8plex)	0.641	0.482	1.091	4.53	43	0	1862.0114	2	931.5093384	2.142219866	39.46291333
														FANSLVGVQQQLQAFNTYR	2	3	1	N-Term(iTRAQ8plex)	0.556	0.508	0.828	4.52	55	0	2488.32419	3	830.112915	-0.349404959	56.18507833
														QALQDTLALYK	1	3	1	N-Term(iTRAQ8plex); K11(iTRAQ8plex)	0.895	0.6	0.832	4.34	-	0	1872.10306	3	624.7058716	-1.084237267	57.50188667
														MWEVLESTTQTK	2	3	1	N-Term(iTRAQ8plex); K12(iTRAQ8plex)	1.26	0.991	1.458	4.29	29	0	2061.11094	3	687.7084961	-1.822448606	68.645735
														EVDDLEQWIAER	2	3	1	N-Term(iTRAQ8plex)	0.666	0.506	1.064	3.86	26	0	1806.93071	2	903.9689941	7.25559711	46.42759833
														DLDDFQSWLSR	2	3	1	N-Term(iTRAQ8plex)	0.717	0.747	1.2	3.77	28	0	1685.83599	2	843.4216309	-4.571145306	37.451965
														ENEVLEAWK	2	3	1	N-Term(iTRAQ8plex); K9(iTRAQ8plex)	0.574	0.634	1.1	3.73	37	0	1725.95391	2	863.4805908	-5.380375077	28.62149167
														LTTLELLEVR	4	3	1	N-Term(iTRAQ8plex)	0.742	0.716	1.324	3.51	39	0	1490.90557	2	745.9564209	-2.693747491	50.21593167
														FLQDCQELSLWINEK	2	3	1	N-Term(iTRAQ8plex); C5(Methylthio); K15(iTRAQ8plex)	-	-	1.19	3.48	48	0	2520.31711	3	840.7772217	3.324775689	61.999815
														IDDIFER	2	3	1	N-Term(iTRAQ8plex)	0.71	0.685	0.961	2.77	26	0	1211.65056	2	606.3289185	-5.62951577	35.73317833
														HRPDLIDFDK	1	7	1	N-Term(iTRAQ8plex); K10(iTRAQ8plex)	0.742	0.43	0.944	-	27	0	1864.064	3	622.0261841	5.502866908	22.58849
112382377	UBE2S	ubiquitin-conjugating enzyme E2 S	NP_055316.2	8.11	1	1	2	222	23.8	8.38	0.778	1.293	2.082																
														CLLIHPNPESALNEEAGR	2	1	1	N-Term(iTRAQ8plex); C1(Methylthio)	0.778	1.293	2.082	3.01	43	0	2313.15818	3	771.7242432	-2.241981489	37.47284833
112421122	DNAJC13	dnaJ homolog subfamily C member 13	NP_056083.3	2.23	3	3	6	2243	254.3	6.74	0.619	0.469	0.636																
														ILPPGLLAYLESSDLVPEKDADR	2	2	1	N-Term(iTRAQ8plex); K19(iTRAQ8plex)	-	-	-	3.8	28	1	3119.74941	4	780.6928101	1.432504278	61.44468833
														QLVLETLYALTSSTK	2	3	1	N-Term(iTRAQ8plex); K15(iTRAQ8plex)	0.628	0.491	0.857	3.53	38	0	2275.32547	3	759.1133423	-5.044418772	56.46898667
														DSLLASLLDGVR	2	2	1	N-Term(iTRAQ8plex)	0.611	0.447	0.472	3.29	28	0	1562.90947	2	781.958374	2.495427244	54.98978667
114155142	TPR	nucleoprotein TPR	NP_003283.2	6.35	9	9	18	2363	267.1	5.02	0.957	0.979	1.081																
														RPSTSQTVSTPAPVPVIESTEAIEAK	4	1	1	N-Term(iTRAQ8plex); K26(iTRAQ8plex)	2.2	1.621	0.981	7.51	48	0	3303.81772	3	1101.944092	-2.418159852	33.631975
														LTELYNAYVETQDQLLLEK	2	1	1	N-Term(iTRAQ8plex); K19(iTRAQ8plex)	0.734	0.324	0.313	6.22	42	0	2891.60978	3	964.5414429	8.131824523	63.75748833
														KLELDILPLQEANAELSEK	1	1	1	N-Term(iTRAQ8plex); K1(iTRAQ8plex); K19(iTRAQ8plex)	0.729	-	0.593	5.79	-	1	3065.7748	4	767.1991577	-3.712053678	46.27147
														TDGFAEAIHSPQVAGVPR	1	1	1	N-Term(iTRAQ8plex)	0.948	0.981	1.14	3.93	-	0	2156.13718	3	719.3839111	-1.419738777	28.92904667
														LTIHAPPQELGPPVQR	2	1	1	N-Term(iTRAQ8plex)	0.901	1.329	1.083	3.76	29	0	2057.17636	3	686.3969727	-2.239273856	26.94866667
														LKESAEFQTQLEK	1	1	1	N-Term(iTRAQ8plex); K2(iTRAQ8plex); K13(iTRAQ8plex)	0.957	0.846	0.743	3.49	-	1	2463.40273	4	616.6061401	-7.908017996	32.99649
														ASTALSNEQQAR	3	1	1	N-Term(iTRAQ8plex)	1.253	1.017	1.188	3.44	36	0	1579.82952	2	790.418396	-2.953437549	14.875775
														LSQELEYLTEDVKR	1	1	1	N-Term(iTRAQ8plex); K13(iTRAQ8plex)	1.125	0.509	1.411	3.26	-	1	2331.3041	4	583.5814819	1.053725414	48.28004833
														QLDTETNLHLNTK	3	1	1	N-Term(iTRAQ8plex); K13(iTRAQ8plex)	0.89	0.849	0.954	3.05	28	0	2135.18058	3	712.3983765	-5.201038073	23.131365
114796642	RCC1	regulator of chromosome condensation isoform c	NP_001041664.1	19.00	4	4	12	421	44.9	7.52	2.330	1.020	0.952																
														VFAWGMGTNYQLGTGQDEDAWSPVEMMGK	2	3	1	N-Term(iTRAQ8plex); K29(iTRAQ8plex)	3.006	-	1.251	6.1	83	0	3813.82541	3	1271.946655	2.137295086	58.553585
														SGQVYSFGCNDEGALGR	2	3	1	N-Term(iTRAQ8plex); C9(Methylthio)	2.965	1.201	1.081	5.87	80	0	2109.97417	2	1055.490723	5.017309054	32.769915
														VVQVSAGDSHTAALTDDGR	6	3	1	N-Term(iTRAQ8plex)	1.963	1.02	0.838	5.26	59	0	2203.11911	3	735.0445557	-2.994644171	21.019225
														SMVPVQVQLDVPVVK	2	3	1	N-Term(iTRAQ8plex); M2(Oxidation); K15(iTRAQ8plex)	1.15	0.923	0.79	4.7	46	0	2262.32425	3	754.7796021	-4.846077056	35.70694167
115298668	SNRNP40	U5 small nuclear ribonucleoprotein 40 kDa protein	NP_004805.2	3.36	1	1	2	357	39.3	8.10	1.425	0.848	0.865																

														TVAVWDSETGER	2	1	1	N-Term(iTRAQ8plex)	1.425	0.848	0.865	4.31	42	0	1653.83953	2	827.4234009	0.552555311	28.032365
														IWDIR	1	4	3	N-Term(iTRAQ8plex)	-	-	-	-	33	0	1006.59648	2	503.8018799	-2.233421647	28.65469833
115298678	C3	complement C3 preproprotein	NP_000055.2	0.54	1	1	1	1663	187.0	6.40	2.324	0.781	0.730																
														GLEVTITAR	1	1	1	N-Term(iTRAQ8plex)	2.324	0.781	0.73	3.04	-	0	1263.75859	2	632.3829346	0.918862694	27.22003
115387094	SDHB	succinate dehydrogenase [ubiquinone] iron-sulfur subunit, mitochondrial precursor	NP_002991.2	3.21	1	1	2	280	31.6	8.76	0.804	0.351	0.426																
														LQDPFSLYR	2	1	1	N-Term(iTRAQ8plex)	0.804	0.351	0.426	-	25	0	1442.78972	2	721.8984985	-3.33440807	39.63527333
115430112	REEP5	receptor expression-enhancing protein 5	NP_005660.4	14.81	3	3	6	189	21.5	8.10	0.736	0.522	0.451																
														KATVNLLGEEK	2	1	1	N-Term(iTRAQ8plex); K1(iTRAQ8plex); K11(iTRAQ8plex)	0.997	0.86	0.703	3.14	27	1	2114.2985	3	705.4376831	1.738054685	29.50873167
														NCMTDLLAK	3	2	1	N-Term(iTRAQ8plex); C2(Methylthio); K9(iTRAQ8plex)	0.489	0.463	0.451	3.13	46	0	1662.87906	3	554.9645386	-2.826319394	58.18845333
														IIRPFFLK	1	2	1	N-Term(iTRAQ8plex); K8(iTRAQ8plex)	0.827	0.581	0.328	2.75	-	0	1642.07192	4	411.2734375	3.318259044	37.400515
115430223	LGALS3	galectin-3 isoform 1	NP_002297.2	21.20	4	4	13	250	26.1	8.56	0.541	1.308	0.244																
														IQVLVEPDHFK	6	2	1	N-Term(iTRAQ8plex); K11(iTRAQ8plex)	0.525	1.276	0.152	4.94	47	0	1933.14116	2	967.0742188	2.286216904	31.79385333
														QSVFPFESGKPFK	1	2	1	N-Term(iTRAQ8plex); K10(iTRAQ8plex); K13(iTRAQ8plex)	0.584	1.178	0.244	4.45	-	0	2410.38418	4	603.3515015	-2.329637193	31.848165
														MLITILGTVKPNANR	4	3	1	N-Term(iTRAQ8plex); K10(iTRAQ8plex)	0.525	1.45	0.247	4.2	21	0	2249.37229	4	563.0963135	0.441336531	48.40110167
														VAVNDAHLLQYNHR	2	2	1	N-Term(iTRAQ8plex)	1.093	1.341	0.814	2.74	28	0	1954.05228	3	652.0222778	-1.940098504	19.56243167
115583685	SLC16A1	monocarboxylate transporter 1	NP_003042.3	6.00	2	2	20	500	53.9	8.66	0.671	0.925	0.370																
														SVFQTINQFLDLTLFTHR	2	1	1	N-Term(iTRAQ8plex)	-	-	-	4.4	49	0	2484.34897	3	828.7878418	-2.547612854	70.82942167
														DLHDANTDLIGR	18	1	1	N-Term(iTRAQ8plex)	0.671	0.925	0.37	4.16	53	0	1643.86026	2	822.4331055	-3.98877444	20.23008667
116063573	FLNA	filamin-A isoform 1	NP_001447.2	44.30	73	76	368	2639	279.8	6.05	0.582	1.032	2.142																
														VQVQDNEGCPVEALVK	7	7	1	N-Term(iTRAQ8plex); C9(Methylthio); K16(iTRAQ8plex)	0.597	0.939	1.969	7.92	104	0	2382.24607	2	1191.639404	4.088397825	36.3748
														FGGEHVPNSPFQVTALAGDQPSVQPPLR	14	5	1	N-Term(iTRAQ8plex)	-	-	1.246	7.5	71	0	3249.68845	3	1083.901001	-2.302257717	67.24708
														FNEEHIPDSPFVVPVASPSGDAR	20	7	1	N-Term(iTRAQ8plex)	0.982	0.87	1.265	7.03	69	0	2771.38346	3	924.4696045	-0.018428788	38.78429667
														GQHVPGSPFQFTVGPLGEGGAHK	2	9	1	N-Term(iTRAQ8plex); K23(iTRAQ8plex)	-	-	-	6.57	42	0	2912.54506	4	728.8917236	-6.096609102	32.49176
														YWPQEAGEYAVHVLCNSEDIR	20	7	1	N-Term(iTRAQ8plex); C15(Methylthio)	0.612	0.933	2.071	6.55	59	0	2829.33487	3	943.7831421	2.481402819	62.71986
														DAGEGGLSLAIEGPSK	4	5	1	N-Term(iTRAQ8plex); K16(iTRAQ8plex)	0.765	1.155	2.455	6.46	66	0	2109.16557	2	1055.086426	0.347835423	30.12131333
														VHSPSGALEECYVTEIDQDKYAVR	6	7	1	N-Term(iTRAQ8plex); C11(Methylthio); K20(iTRAQ8plex)	-	-	-	6.39	64	1	3363.67031	4	841.6730347	-3.182091924	40.134575
														VTYTPMAPGSYLISIK	4	7	1	N-Term(iTRAQ8plex); K16(iTRAQ8plex)	0.433	0.737	1.871	6.07	74	0	2349.34526	2	1175.17627	4.431508074	41.34665167
														SAGQGEVLVYVEDPAGHQEEAK	5	7	1	N-Term(iTRAQ8plex); K22(iTRAQ8plex)	0.317	0.821	2.052	5.97	55	0	2921.4968	3	974.5037842	-4.577109563	28.57107667
														HTAMVSWGGVSIPNSPFR	10	7	1	N-Term(iTRAQ8plex)	1.113	1.46	1.758	5.9	63	0	2247.16568	3	749.7267456	0.446013578	38.97058
														AGNNMLLVGVHGPR	12	7	1	N-Term(iTRAQ8plex)	0.556	0.89	2.177	5.39	72	0	1738.9616	2	869.984436	-4.184019766	22.21961167
														GAGTGGLGLAVEGPSEAK	2	7	1	N-Term(iTRAQ8plex); K18(iTRAQ8plex)	1.124	1.551	2.319	5.31	33	0	2179.20316	3	727.0725708	-6.787869958	20.84032
														VANPSGNLTETYVQDR	6	7	1	N-Term(iTRAQ8plex)	0.625	0.97	2.066	5.28	81	0	2068.06011	2	1034.533691	-0.573052735	27.34498667
														ATCAPQHGAPGPGPADASK	2	7	1	N-Term(iTRAQ8plex); C3(Methylthio); K19(iTRAQ8plex)	0.547	0.935	2.555	5.26	35	0	2387.19468	3	796.4030762	-4.610865837	17.19500833
														EGPYSISVLYGDEEVPR	6	7	1	N-Term(iTRAQ8plex)	0.573	0.787	2.815	5.21	97	0	2214.11797	2	1107.562622	-2.377370787	37.06368667
														LIALLEVLSQKK	14	7	1	N-Term(iTRAQ8plex); K11(iTRAQ8plex); K12(iTRAQ8plex)	0.442	1.301	3.198	5.09	44	1	2267.47402	4	567.6279297	3.055171286	66.17326167
														TFSVWYVPEVTGTHK	7	7	1	N-Term(iTRAQ8plex); K15(iTRAQ8plex)	0.411	0.873	1.732	5.08	41	0	2359.27164	3	787.0967407	-6.35889321	35.70851167
														CSYQPTMEGVHTVHVTFAGVPIPR	1	6	1	N-Term(iTRAQ8plex); C1(Methylthio)	0.633	-	1.068	5.06	-	0	2976.4789	4	744.8751831	-1.599123757	42.600635
														NGQHVASSPIPVVISQSEIGDASR	2	7	1	N-Term(iTRAQ8plex)	1.318	1.571	1.65	5.05	54	0	2752.44321	3	918.1525879	-3.633454847	31.44141667
														SPFSVAVSPSLDLSK	4	7	1	N-Term(iTRAQ8plex); K15(iTRAQ8plex)	0.546	0.911	1.584	5.02	49	0	2142.21604	3	714.7435303	-4.973228423	38.142075
														IANLQTDLSDGLR	6	7	1	N-Term(iTRAQ8plex)	0.557	0.844	2.141	4.94	63	0	1719.95012	2	860.4786987	-2.436384062	43.156295
														EATTEFSVDAR	12	7	1	N-Term(iTRAQ8plex)	0.559	0.88	2.145	4.87	58	0	1529.78362	2	765.3876953	-4.462960718	21.77808667
														VDINTEDLEDGTCR	2	7	1	N-Term(iTRAQ8plex); C13(Methylthio)	0.589	0.69	2.187	4.71	37	0	1929.88884	2	965.4480591	2.714790588	32.34484333
														LQVEPAVDTSGVQCYGPGIEGQGVFR	4	7	1	N-Term(iTRAQ8plex); C14(Methylthio)	-	2.972	0.791	4.67	76	0	3056.50998	3	1019.508179	-0.79156201	45.44237167
														CAPGVVGPAEADIDFDIIR	2	7	1	N-Term(iTRAQ8plex); C1(Methylthio)	1.225	1.062	1.33	4.63	52	0	2308.15866	3	770.0577393	-1.441745151	52.103125
														VSGQGLHEGHTFEPAEFIIDTR	5	7	1	N-Term(iTRAQ8plex)	0.793	1.291	1.401	4.62	26	0	2744.38272	4	686.8521118	-2.920111301	35.24157333
														AWGPGLEGGVVGK	4	7	1	N-Term(iTRAQ8plex); K13(iTRAQ8plex)	0.726	0.972	1.887	4.57	55	0	1835.05522	2	918.03125	-4.565486582	39.47789
														HTAMVSWGGVSIPNSPFR	2	7	1	N-Term(iTRAQ8plex); M4(Oxidation)	1.298	0.885	1.596	4.56	40	0	2263.15427	3	749.7267456	0.446013578	38.97058
														DAPQDFHPDR	8	7	1	N-Term(iTRAQ8plex)	0.725	0.916	1.191	4.55	51	0	1501.72931	3	501.2479553	-2.948988986	14.17999833
														CAPGVVGPAEADIDFDIIRNDNDTFTVK	1	7	1	N-Term(iTRAQ8plex); C1(Methylthio); K28(iTRAQ8plex)	-	3.485	6.896	4.53	-	1	3646.82663	3	1216.280396	-2.097447269	44.51460667
														APSVANVGSHCDLSLK	2	7	1	N-Term(iTRAQ8plex); C11(Methylthio); K16(iTRAQ8plex)	0.769	1.556	3.256	4.53	44	0	2252.1901	3	751.4015503	-3.856100381	30.48882
														DVDIIDHHDNTYTVK	4	7	1	N-Term(iTRAQ8plex); K15(iTRAQ8plex)	0.315	1.046	2.453	4.43	29	0	2393.24503	3	798.418396	-6.312178349	21.22827
														YAPSEAGLHEMDIR	2	7	1	N-Term(iTRAQ8plex); M11(Oxidation)	0.543	0.613	1.336	4.4	31	0	1908.93601	3	631.6516113	-3.999368444	24.671655
														LLGWIQNKLPQLPITNFSR	2	7	1	N-Term(iTRAQ8plex); K8(iTRAQ8plex)	-	-	-	4.37	23	1	2846.68838	4	712.4275513	0.640708741	65.97103167
														AEAGVPAEFSIWTR	8	7	1	N-Term(iTRAQ8plex)	0.722	1.216	2.449	4.33	50	0	1837.96501	2	919.486145	-5.466851036	44.65259333
														VTVLFAGQHIAK	9	7	1	N-Term(iTRAQ8plex); K12(iTRAQ8plex)	0.422	0.894	2.629	4.28	42	0	1892.14231	3	631.3856201	-8.193991741	31.75599167
														EAMQQADDWLGIPQVITPEEIVDPNVDEHSVMTYLSQFPK	2	7	1	N-Term(iTRAQ8plex); K40(iTRAQ8plex)	-	-	-	4.22	27	0	5178.58706	4	1295.402222	-0.605273449	60.50426
														GKLDVQFSGLTK	2	7	1	N-Term(iTRAQ8plex); K2(iTRAQ8plex); K12(iTRAQ8plex)	1.096	1.408	2.461	4.2	27	1	2205.31899	4	552.0852051	-8.176081364	34.74983667
														AFGPGLQGGSAGSPAR	4	7	1	N-Term(iTRAQ8plex)	0.406	0.775	1.993	4.2	75	0	1733.91631	2	867.461792	-4.264297875	16.34122
														YGGDEIPFSPYR	9	7	1	N-Term(iTRAQ8plex)	0.562	0.98	2.319	4.13	42	0	1704.84697	2	852.9334717	3.610525544	32.76572667
														YAPSEAGLHEMDIR	4	7	1	N-Term(iTRAQ8plex)	0.934	0.938	1.878	4.12	42	0	1892.94028	3	631.6516113	-3.999368444	24.671655
														MDCQECPEGYR	4	7	1	N-Term(iTRAQ8plex); C3(Methylthio); C6(Methylthio)	0.52	0.862	1.706	4.08	38	0	1726.67253	2	863.8399048	3.243029703	30.32274833
														SPYTVTVGQACNPSACR	2	6	1	N-Term(iTRAQ8plex); C11(Methylthio); C16(Methylthio)	0.473	1.022	2.44	4.03	63	0	2149.97319	2	1075.490234	-3.391284146	37.68787833
														LYSVSYLLK	8	7	1	N-Term(iTRAQ8plex); K9(iTRAQ8plex)	0.571	0.868	2.249	4.01	35	0	1694.02019	3	565.3450928	-8.344779658	54.73604833
														AHVVPCFDASK	9	7	1	N-Term(iTRAQ8plex); C6(Methylthio); K11(iTRAQ8plex)	0.668	1.468	2.357	3.98	47	0	1827.95757	2	914.4837036	-5.728622448	23.98744
														TGVAVNKPAEFTVDAK	1	7	1	N-Term(iTRAQ8plex); K7(iTRAQ8plex); K16(iTRAQ8plex)	0.892	1.055	2.395	3.97	-	0	2559.4684	4	640.6225586	-8.820896152	27.06016167
														YNEQHVPGSPFTAR	10	5	1	N-Term(iTRAQ8plex)	0.802	1.29	2.54	3.96	62	0	1906.96196	2	953.9846191	-4.91862918	21.72921
														DGSCGVAYVVQEPGDYEVSVK	1	7	1	N-Term(iTRAQ8plex); C4(Methylthio); K21(iTRAQ8plex)	-	-	-	3.92	-	0	2855.39736	4	714.6047974	-2.753636669	36.327725
														SPFEVYVDK	4	7	1	N-Term(iTRAQ8plex); K9(iTRAQ8plex)	0.494	0.994	2.624	3.9	41	0	1691.93083	2	846.4690552	-9.236529133	42.04549333
														VTYTPMAPGSYLISIK	2	7	1	N-Term(iTRAQ8plex); M6(Oxidation); K16(iTRAQ8plex)	1.094	1.139	1.57	3.9	25	0	2365.31614	2	1175.17627	4.431508074	41.34665167
														YGGPYHIGGSPFK	1	7	1	N-Term(iTRAQ8plex); K13(iTRAQ8plex)	0.446	0.537	1.121	3.86	-	0	1988.07053	3	663.3616943	-7.289249537	26.91545
														NDNDTFTVK	3	9	1	N-Term(iTRAQ8plex); K9(iTRAQ8plex)	0.4	0.957	1.9	3.83	37	0	1661.8853	2	831.4462891	-6.13146707	13.79479833
														FVPAEMGTHTVSVK	4	7	1	N-Term(iTRAQ8plex); K14(iTRAQ8plex)	0.624	1.055	2.113	3.74	27	0	2111.15957	3	704.3927612	-6.745501729	25.98663333
														YTPVQQGPVGVNVTYGGDPIPK	2	7	1	N-Term(iTRAQ8plex); K22(iTRAQ8plex)	-	-	-	3.73	90	0	2894.58084	3	965.5317993	-2.223672831	33.448175
														VPVHDVTDASK	5	7	1	N-Term(iTRAQ8plex); K11(iTRAQ8plex)	0.431	0.966	2.988	3.72	58	0	1775.99944	2	888.5040283	-5.867834909	17.75791667
														GTVEPQLEAR	4	6	1	N-Term(iTRAQ8plex)	0.226	0.778	2.142	3.69	36	0	1403.77397	2	702.390625	-4.030506411	17.53550833
														ANLPQSFQVDTSK	2	7	1	N-Term(iTRAQ8plex); K13(iTRAQ8plex)	0.89	1.007	1.403	3.62	26	0	2043.12394	3	681.7128296	-4.491054267	35.532765
														GLVEPVDVVDNADGTQTVNYVPSR	2	7	1	N-Term(iTRAQ8plex)	0.599	1.064	1.014	3.51	48	0	2848.45237	3	950.1556396	-3.772436154	36.73598167
														VLPTHDASK	4	7	1	N-Term(iTRAQ8plex); K9(iTRAQ8plex)	0.556	0.763	2.33	3.47	35	0	1575.91605	2	788.4625854	-8.621655742	14.28256667
														EAGAGGLAIAVEGPSK	2	7	1	N-Term(iTRAQ8plex); K16(iTRAQ8plex)	1.484	1.655	1.284	3.47	33	0	2035.14841	3	679.0543213	-7.881824602	21.93374333
														AEFTVETR	9	7	1	N-Term(iTRAQ8plex)	0.578	0.928	2.069	3.46	38	0	1256.6799	2	628.8442383	1.87688214	23.31448333
														AGNNMLLVGVHGPR	4	7	1	N-Term(iTRAQ8plex); M5(Oxidation)	0.984	1.252	2.184	3.45	53	0	1754.95854	2	869.984436	-4.184019766	22.21961167
														VAQPTITDNKDGTVTVR	1	7	1	N-Term(iTRAQ8plex); K10(iTRAQ8plex)	0.293	1.23	3.4	3.42	-	1	2423.36258	3	808.4590454	-3.667762964	21.75428667
														VEYTPYEEGLHSVDVTYDGSPVPSSPFQVPVTEGCDPSR	1	7	1	N-Term(iTRAQ8plex); C35(Methylthio)	-	-	-	3.28	-	0	4590.1124	3	1530.708984	-3.21613584	50.90003333
														AGQSAAGAAPGGGVDTR	4	7	1	N-Term(iTRAQ8plex)	0.765	0.922	1.742	3.27	49	0	1746.90019	2	873.9537354	-2.007680168	13.83354333
														VTYCPTEPGNYIINIK	2	7	1	N-Term(iTRAQ8plex); C4(Methylthio); K16(iTRAQ8plex)	1.152	0.88	1.134	3.25	31	0	2479.31925	3	827.1112671	0.284642461	41.95584167
														CSGPGLSPGMVR	2	7	1	N-Term(iTRAQ8plex); C1(Methylthio)	0.498	1.225	3.066	3.13	33	0	1510.74162	2	755.8744507	-4.373714674	27.79619167
														FVPAEMGTHTVSVK	1	7	1	N-Term(iTRAQ8plex); M6(Oxidation); K14(iTRAQ8plex)	0.32	0.744	1.217	3.01	-	0	2127.15273	3	704.3927612	-6.745501729	25.98663333
														YGGQPVPNFPSK	1	7	1	N-Term(iTRAQ8plex); K12(iTRAQ8plex)	1.005	1.165	0.981	2.95	-	0	1899.0474	3	633.6873169	-5.836540163	24.43546833
														VNVGAGSHPNK	3	7	1	N-Term(iTRAQ8plex); K11(iTRAQ8plex)	0.55	0.845	1.642	2.93	-	0	1687.95554	3	563.3233643	-8.567628841	15.67534667
														ALTQTGGPHVK	1	7	1	N-Term(iTRAQ8plex); K11(iTRAQ8plex)	0.453	0.581	1.164	2.79	-	0	1717.00541	3	573.0066528	-9.490974144	15.15762167
														VEPGLGADNSVVR	2	7	1	N-Term(iTRAQ8plex)	0.865	1.325	2.142	2.79	25	0	1616.88701	2	808.9471436	-2.462021526	22.63191333
														FADQHVPGSPFSVK	1	7	1	N-Term(iTRAQ8plex); K14(iTRAQ8plex)	0.381	0.542	0.71	2.76	-	0	2124.15927	3	708.7246094	-4.974403344	29.87577167
														DKGEYTLVVK	2	7	1	N-Term(iTRAQ8plex); K2(iTRAQ8plex); K10(iTRAQ8plex)	0.435	1.105	2.692	2.75	29	1	2064.2271	2	1032.617188	-9.540093731	17.749535
														FNGTHIPGSPFK	1	7	1	N-Term(iTRAQ8plex); K12(iTRAQ8plex)	0.808	0.675	1.195	2.69	-	0	1910.05716	3	637.3572388	-9.061635296	25.20735
														THEAEIVEGENHTYCIR	1	7	1	N-Term(iTRAQ8plex); Y14(iTRAQ8plex); C15(Methylthio)	-	-	-	-	40	0	2655.29824	4	664.5800171	-5.021590581	23.29347
														EGPYSISVLYGDEEVPR	1	7	1	N-Term(iTRAQ8plex); Y4(iTRAQ8plex)	0.623	0.948	2.355	-	34	0	2518.3198	2	1107.562622	-2.377370787	37.06368667
														VTGDDSMR	1	5	1	N-Term(iTRAQ8plex); M7(Oxidation)	0.61	1.652	3.052	-	24	0	1200.57561	2	600.7914429	-6.352934172	8.501173333
														SPYTVTVGQACNPSACR	1	6	1	N-Term(iTRAQ8plex); Y3(iTRAQ8plex); C11(Methylthio); C16(Methylthio)	0.764	1.833	3.579	-	23	0	2454.17081	2	1075.490234	-3.391284146	37.68787833
														VGTECGNQK	1	7	1	N-Term(iTRAQ8plex); C5(Methylthio); K9(iTRAQ8plex)	1.242	1.158	1.65	-	20	0	1589.81357	3	530.609375	-6.302971731	13.169
														LIALLEVLSQK	14	14	2	N-Term(iTRAQ8plex); K11(iTRAQ8plex)	-	-	-	4.46	46	0	1835.19065	3	612.3999634	1.474285275	66.43766667
														AGGPGLER	1	12	2	N-Term(iTRAQ8plex)	-	-	-	-	26	0	1060.59892	2	530.8031006	-6.002974336	11.45919167
														WCNEHLK	1	14	2	N-Term(iTRAQ8plex); C2(Methylthio); K7(iTRAQ8plex)	-	-	-	-	22	0	1583.81247	3	528.6090088	-9.975412545	21.57536167
116256327	MME	neprilysin	NP_009218.2	1.47	1	1	2	750	85.5	5.73	-	0.150	0.143																
														HVVEDLIAQIR	2	1	1	N-Term(iTRAQ8plex)	-	0.15	0.143	3.68	50	0	1596.93601	3	532.9835205	-0.953717967	39.78061333
116295258	ITGA2	integrin alpha-2 precursor	NP_002194.2	13.29	9	9	27	1181	129.2	5.23	0.436	0.424	0.411																
														IFSGPSSEQFGYAVQQFINPK	2	1	1	N-Term(iTRAQ8plex); K21(iTRAQ8plex)	0.396	0.189	0.432	5.82	56	0	2952.58902	3	984.8678589	5.890460887	54.71605167
														STNINFYEISSDGNVPSIVHSFEDVGPK	2	1	1	N-Term(iTRAQ8plex); K28(iTRAQ8plex)	-	-	-	5.09	33	0	3660.86342	4	915.9713135	-0.231440961	51.16537833
														AVIDQCNHDNILR	6	1	1	N-Term(iTRAQ8plex); C6(Methylthio)	0.323	0.439	0.414	4.97	48	0	1860.93161	3	620.9820557	-2.481852519	27.76910167
														IPLLYDAEIHLTR	2	1	1	N-Term(iTRAQ8plex)	0.697	0.908	0.529	4.54	63	0	1858.07334	3	620.0292969	-0.368082888	51.876255
														EGILGQHQFLEGPEGIENTR	4	1	1	N-Term(iTRAQ8plex)	0.621	0.517	0.615	4.44	32	0	2528.30362	3	843.4393921	-0.446259879	33.802585
														GNWLLVGSPWSGFPENR	2	1	1	N-Term(iTRAQ8plex)	0.522	0.315	0.156	4.12	78	0	2220.15215	2	1110.579712	0.78188701	49.37107167
														NMVVNQAQSCPEHIIYIQEPSDVVNSLDLR	2	1	1	N-Term(iTRAQ8plex); C10(Methylthio)	-	-	-	3.18	24	0	3761.86484	3	1254.626465	1.187969648	51.55756667
														VFSIPFHK	5	1	1	N-Term(iTRAQ8plex); K8(iTRAQ8plex)	0.388	0.342	0.294	3.12	28	0	1582.95664	3	528.3237305	0.042235048	34.98053833
														IPLLYDAEIHLTR	1	1	1	N-Term(iTRAQ8plex); Y5(iTRAQ8plex)	-	0.522	0.147	-	38	0	2162.28066	3	620.0292969	-0.368082888	51.876255
														VYLFTIK	1	1	1	N-Term(iTRAQ8plex); K7(iTRAQ8plex)	-	-	-	-	34	0	1491.93537	3	497.9833069	-2.77753031	64.29653

116534898	DSG2	desmoglein-2 preproprotein	NP_001934.2	5.81	5	5	11	1118	122.2	5.24	0.880	0.794	0.572																
														GQIIGNFQAFDEDTGLPAHAR	5	1	1	N-Term(iTRAQ8plex)	1.007	0.767	1.003	6.11	72	0	2561.30509	3	854.4400635	0.213125556	37.4391
														GITEPPFGIFVFNK	1	1	1	N-Term(iTRAQ8plex); K14(iTRAQ8plex)	1.039	0.839	0.369	4.37	-	0	2174.24192	3	725.4188232	-2.357062201	43.97882
														FLDDLGLK	2	1	1	N-Term(iTRAQ8plex); K8(iTRAQ8plex)	0.875	0.769	0.545	3.51	40	0	1528.91777	2	764.9625244	-1.150547078	67.62203333
														IVAISEDYPR	2	1	1	N-Term(iTRAQ8plex)	0.88	1.159	0.572	2.98	27	0	1466.81413	2	733.9107056	-1.047704103	34.04556667
														VATPLPDPMASR	1	1	1	N-Term(iTRAQ8plex)	0.849	0.794	0.522	-	30	0	1558.85625	2	779.9317627	-0.174826496	30.25979333
117168248	HACD3	very-long-chain (3R)-3-hydroxyacyl-CoA dehydratase 3	NP_057479.2	18.78	7	7	26	362	43.1	8.94	1.728	1.037	1.280																
														VELSDVQNPAISITENVLHFK	6	1	1	N-Term(iTRAQ8plex); K21(iTRAQ8plex)	0.68	-	0.255	5.11	43	0	2961.64785	4	741.1674194	-0.936453545	56.04805833
														WLDESDAEMELR	2	1	1	N-Term(iTRAQ8plex)	2.853	1.282	2.413	4.57	60	0	1797.87541	2	899.4413452	6.8316801	37.33132667
														RPLFLAPDFDR	10	1	1	N-Term(iTRAQ8plex)	1.551	0.955	1.28	4.49	43	0	1650.91989	3	550.9781494	-4.292338977	33.19215667
														LESEGSPETLTNLR	2	2	1	N-Term(iTRAQ8plex)	1.509	0.763	1.256	4.32	45	0	1849.98613	2	925.4967041	2.81639037	30.15270833
														QVNITVQK	2	1	1	N-Term(iTRAQ8plex); K8(iTRAQ8plex)	1.946	0.657	1.256	3.42	44	0	1537.93779	2	769.4725342	-9.375906296	28.28584833
														WLDESDAEMELR	2	1	1	N-Term(iTRAQ8plex); M9(Oxidation)	2.622	1.324	2.321	3.31	30	0	1813.86467	2	899.4413452	6.8316801	37.33132667
														LRLESEGSPETLTNLR	1	2	1	N-Term(iTRAQ8plex)	1.728	1.297	1.433	2.92	-	1	2119.16324	3	707.0592651	-1.353902232	28.43781667
														RPLFLAPDFDRWLDESDAEMELR	1	1	1	N-Term(iTRAQ8plex)	-	-	-	2.84	-	1	3125.56784	3	1042.527466	0.298477264	55.54083667
117189975	HNRNPC	heterogeneous nuclear ribonucleoproteins C1/C2 isoform a	NP_112604.2	47.71	1	15	160	306	33.6	5.08	2.407	0.621	1.215																
														SAAEMYGSVTEHPSPSPLLSSSFDLDYDFQR	1	1	1	N-Term(iTRAQ8plex)	2.407	0.621	1.215	3.54	-	0	3737.76633	3	1246.593628	2.126735157	50.0565
														VFIGNLNTLVVK	27	5	2	N-Term(iTRAQ8plex); K12(iTRAQ8plex)	-	-	-	5.89	94	0	1925.20659	2	963.1069336	1.121577035	41.85852667
														MIAGQVLDINLAAEPK	12	2	2	N-Term(iTRAQ8plex); K16(iTRAQ8plex)	-	-	-	5.51	107	0	2291.31401	2	1146.160645	-4.966083845	35.92067333
														MIAGQVLDINLAAEPK	9	2	2	N-Term(iTRAQ8plex); M1(Oxidation); K16(iTRAQ8plex)	-	-	-	5.36	73	0	2307.3019	2	1146.160645	-4.966083845	35.92067333
														QKVDSLLENLEK	4	5	2	N-Term(iTRAQ8plex); K2(iTRAQ8plex); K12(iTRAQ8plex)	-	-	-	5.27	38	1	2328.38555	2	1164.696411	-1.990930747	35.18612
														GFAFVQYVNER	66	2	2	N-Term(iTRAQ8plex)	-	-	-	4.83	57	0	1633.86344	3	545.2926636	-0.35732934	35.70433333
														VDSLLENLEK	8	5	2	N-Term(iTRAQ8plex); K10(iTRAQ8plex)	-	-	-	4.68	57	0	1768.02068	2	884.5139771	-5.991908676	68.441755
														KSDVEAIFSK	7	5	2	N-Term(iTRAQ8plex); K1(iTRAQ8plex); K10(iTRAQ8plex)	-	-	-	4.33	34	1	2036.22141	3	679.4119873	2.901140337	37.95227833
														SDVEAIFSK	6	5	2	N-Term(iTRAQ8plex); K9(iTRAQ8plex)	-	-	-	3.67	50	0	1603.91033	2	802.4589233	-2.868763632	41.84024667
														IVGCSVHK	8	13	2	N-Term(iTRAQ8plex); C4(Methylthio); K8(iTRAQ8plex)	-	-	-	3.26	28	0	1496.85569	3	499.6234131	1.285165852	28.958285
														LKGDDLQAIK	1	5	2	N-Term(iTRAQ8plex); K2(iTRAQ8plex); K10(iTRAQ8plex)	-	-	-	3.08	-	1	2013.25138	3	671.7553101	2.103873282	29.013175
														ELTQIK	1	19	2	N-Term(iTRAQ8plex); K6(iTRAQ8plex)	-	-	-	2.84	-	0	1339.84403	3	447.2861938	2.610000041	20.842695
														AAVAGEDGR	4	5	2	N-Term(iTRAQ8plex)	-	-	-	-	40	0	1149.61301	3	383.8758545	-3.115025148	10.423885
														VPPPPPIAR	1	2	2	N-Term(iTRAQ8plex)	-	-	-	-	37	0	1247.77411	3	416.5962219	-2.94025856	21.56077833
														GFAFVQYVNER	3	2	2	N-Term(iTRAQ8plex); Y7(iTRAQ8plex)	-	-	-	-	30	0	1938.06462	3	545.2926636	-0.35732934	35.70433333
														MYSYPAR	1	2	2	N-Term(iTRAQ8plex); M1(Oxidation)	-	-	-	-	22	0	1207.60576	2	604.3065186	-2.099866288	13.67385
														DYYDR	1	4	4	N-Term(iTRAQ8plex)	-	-	-	-	14	0	1035.50249	2	518.2548828	-2.29096021	11.38908833
117647226	TRMT10C	mitochondrial ribonuclease P protein 1 precursor	NP_060289.2	2.98	1	1	3	403	47.3	9.36	2.056	1.087	0.565																
														EVPEHITEEELK	3	1	1	N-Term(iTRAQ8plex); K12(iTRAQ8plex)	2.056	1.087	0.565	3.12	25	0	2061.12522	4	516.0357056	-5.559554445	21.86522833
117676403	KRR1	KRR1 small subunit processome component homolog	NP_008974.5	2.36	1	1	2	381	43.6	9.77	0.721	0.671	0.142																
														SVSFEQAVR	2	1	1	N-Term(iTRAQ8plex)	0.721	0.671	0.142	4.17	53	0	1326.73569	3	442.916748	2.826177551	25.84217833
117938251	BCLAF1	bcl-2-associated transcription factor 1 isoform 2	NP_001070908.1	4.83	4	4	8	869	100.2	9.95	1.524	1.011	0.724																
														FNDSEGDDTEETEDYR	2	4	1	N-Term(iTRAQ8plex)	1.583	1.615	0.53	5.05	101	0	2225.94145	2	1113.474365	6.919565977	23.86919667
														SAAMTLNER	2	9	1	N-Term(iTRAQ8plex)	1.334	0.621	0.398	3.98	32	0	1296.69292	2	648.8500977	3.506915504	20.92053
														LLASTLVHSVK	3	8	1	N-Term(iTRAQ8plex); K11(iTRAQ8plex)	1.521	1.092	0.732	3.18	23	0	1776.1124	3	592.7120972	0.780888988	31.64754
														VFLLDR	1	4	1	N-Term(iTRAQ8plex)	1.645	0.914	0.831	-	21	0	1066.65019	2	533.8287354	-5.678728517	26.71215833
118150660	ZC3H15	zinc finger CCCH domain-containing protein 15	NP_060941.2	9.86	3	3	5	426	48.6	5.31	1.066	2.122	2.089																
														DVDETGITVASLER	2	2	1	N-Term(iTRAQ8plex)	0.849	1.48	1.456	4.5	52	0	1808.95073	2	904.9790039	-2.017803712	32.00728
														EVFEFRPELVNDDDEEADDTR	2	2	1	N-Term(iTRAQ8plex)	-	-	-	3.09	27	0	2844.30393	3	948.7728271	-2.641456355	36.02776167
														SVYIDAR	1	1	1	N-Term(iTRAQ8plex)	1.339	3.043	2.997	-	22	0	1127.62969	2	564.3184814	-5.804236492	23.351145
118200356	GHITM	growth hormone-inducible transmembrane protein	NP_055209.2	3.19	1	1	1	345	37.2	9.94	1.287	0.664	0.668																
														VATMLATGGNR	1	1	1	N-Term(iTRAQ8plex)	1.287	0.664	0.668	-	30	0	1394.76567	2	697.8864746	-5.096699316	20.02905
118343647	HSPB11	intraflagellar transport protein 25 homolog	NP_057210.2	8.33	1	1	1	144	16.3	5.03	0.645	1.420	1.104																
														LVIQSYFVQTLK	1	1	1	N-Term(iTRAQ8plex); K12(iTRAQ8plex)	0.645	1.42	1.104	3.46	-	0	2047.24161	3	683.0853882	0.205154476	45.12515833
118402586	GLO1	lactoylglutathione lyase	NP_006699.2	34.24	4	4	19	184	20.8	5.31	1.020	3.944	2.544																
														FSLYFLAYEDKNDIPK	3	1	1	N-Term(iTRAQ8plex); K11(iTRAQ8plex); K16(iTRAQ8plex)	-	4.89	2.544	5.97	28	1	2875.59292	4	719.6536865	-2.77684863	50.54522667
														GLAFIQDPDGYWIEILNPNK	9	1	1	N-Term(iTRAQ8plex); K20(iTRAQ8plex)	1.33	9.75	5.201	5.28	77	0	2911.57065	2	1456.29834	2.720770824	57.50843667
														GFGHIGIAVPDVYSACK	5	1	1	N-Term(iTRAQ8plex); C16(Methylthio); K17(iTRAQ8plex)	0.5	2.862	2.218	4.35	40	0	2388.25724	4	597.8209229	-1.930598074	43.59828833
														DFLLQQTMLR	2	1	1	N-Term(iTRAQ8plex)	1.02	2.66	1.946	3.56	-	0	1568.88469	2	784.9459839	4.748502857	37.99039833
118498359	RSL1D1	ribosomal L1 domain-containing protein 1	NP_056474.2	24.90	11	11	42	490	54.9	10.13	1.901	0.866	0.613																
														TVSQIISLQTLK	2	1	1	N-Term(iTRAQ8plex); K12(iTRAQ8plex)	1.389	0.568	0.474	5.77	45	0	1939.2072	2	970.1072388	1.23226598	37.60737667
														DDVAPESGDTTVK	2	1	1	N-Term(iTRAQ8plex); K13(iTRAQ8plex)	1.314	0.481	0.369	5.14	41	0	1942.01726	2	971.5122681	-2.735190515	14.43585667
														LLSSFDFFLTDAR	8	1	1	N-Term(iTRAQ8plex)	2.139	0.777	0.553	4.81	74	0	1835.98223	2	918.494751	-1.266878754	67.24858167
														EINDCIGGTVLNISK	8	1	1	N-Term(iTRAQ8plex); C5(Methylthio); K15(iTRAQ8plex)	1.871	1.58	1.253	4.6	58	0	2230.21318	2	1115.610229	4.474937398	38.58413667
														SAALPIFSSFVSNWDEATKR	2	1	1	N-Term(iTRAQ8plex); K19(iTRAQ8plex)	2.002	0.919	0.447	4.44	35	1	2834.52738	3	945.5139771	-0.847900668	61.10560167
														SDSEDICLFTK	4	1	1	N-Term(iTRAQ8plex); C7(Methylthio); K11(iTRAQ8plex)	2.236	0.871	0.645	4.22	58	0	1911.95525	2	956.4812622	-5.227374411	53.79826667
														SAALPIFSSFVSNWDEATK	2	1	1	N-Term(iTRAQ8plex); K19(iTRAQ8plex)	-	-	-	3.92	61	0	2678.42777	3	893.4807739	-0.333319669	54.29212
														KAVDALLTHCK	1	1	1	N-Term(iTRAQ8plex); K1(iTRAQ8plex); C10(Methylthio); K11(iTRAQ8plex)	1.298	0.402	0.338	3.3	-	1	2157.26821	4	540.0725098	1.435557474	31.63264667
														LTLPHSIR	7	1	1	N-Term(iTRAQ8plex)	1.659	0.998	0.742	3.19	38	0	1240.76502	3	414.2581787	-6.398028061	19.63398667
														KVPVSVNLLSK	2	1	1	N-Term(iTRAQ8plex); K1(iTRAQ8plex); K11(iTRAQ8plex)	2.118	0.839	0.791	3.14	19	1	2096.3622	4	524.8460083	2.471912114	35.38849333
														LLPSLIGR	4	1	1	N-Term(iTRAQ8plex)	2.004	0.735	0.558	-	23	0	1172.76152	2	586.8843994	-4.561107927	29.43752167
118498362	KTN1	kinectin isoform b	NP_001072990.1	17.99	18	18	39	1306	150.3	5.74	1.138	0.995	0.778																
														QMQSSFTSSEQELER	2	10	1	N-Term(iTRAQ8plex)	1.207	1.028	0.708	5.92	69	0	2090.99614	2	1046.001709	-0.243879575	28.47921
														LQTLVSEQPNKDVVEQMEK	2	10	1	N-Term(iTRAQ8plex); K11(iTRAQ8plex); K19(iTRAQ8plex)	-	2.038	0.864	5.29	26	1	3127.72548	4	782.6868286	-5.811484085	35.77925333
														TVEELLETGLIQVATK	3	10	1	N-Term(iTRAQ8plex); K16(iTRAQ8plex)	0.942	0.848	0.667	4.98	29	0	2352.37864	3	784.7977295	-2.561693661	52.545825
														SVEELLEAELLK	6	10	1	N-Term(iTRAQ8plex); K12(iTRAQ8plex)	1.292	1.301	0.925	4.93	58	0	1981.15459	2	991.0809326	-6.659089453	75.07599833
														QPTPPSEAAASK	2	10	1	N-Term(iTRAQ8plex); K12(iTRAQ8plex)	1.527	1.065	0.955	4.87	44	0	1792.00504	3	598.0065308	-0.603940737	13.51913833
														VSVPSNLSYGEWLHGFEK	1	8	1	N-Term(iTRAQ8plex); K18(iTRAQ8plex)	0.896	0.865	0.866	4.74	-	0	2657.42301	3	886.479187	1.72967764	44.125905
														EVIDLLKPDQVEGIQK	1	10	1	N-Term(iTRAQ8plex); K7(iTRAQ8plex); K16(iTRAQ8plex)	0.457	1.337	0.648	4.73	-	0	2736.60317	4	684.90625	-8.88370673	34.94077833
														GELTTLIHQLQEK	5	10	1	N-Term(iTRAQ8plex); K13(iTRAQ8plex)	1.32	0.713	0.746	4.52	39	0	2118.24184	4	530.315918	1.855640334	44.43678333
														ECMAGTSGSEEVK	2	10	1	N-Term(iTRAQ8plex); C2(Methylthio); K13(iTRAQ8plex)	1.293	1.217	0.835	4.5	43	0	1981.94254	3	666.6499023	-4.36626721	22.66713333
														LLEEQLQHEISNK	2	10	1	N-Term(iTRAQ8plex); K13(iTRAQ8plex)	1.594	1.432	1.02	4.16	29	0	2189.22983	3	730.4147949	-4.024147784	36.04346333
														NWEAMEALASTEK	2	4	1	N-Term(iTRAQ8plex); K13(iTRAQ8plex)	1.096	0.848	0.701	4.01	36	0	2088.08384	3	696.6994629	-2.579339983	61.065885
														TAEHEAAQQDLQSK	2	10	1	N-Term(iTRAQ8plex); K14(iTRAQ8plex)	0.84	0.879	0.657	3.98	29	0	2164.13662	4	541.7896118	-4.091199374	13.64176667
														SVLAETEGILQK	2	10	1	N-Term(iTRAQ8plex); K12(iTRAQ8plex)	0.931	0.753	0.699	3.89	44	0	1896.11313	3	632.7092285	-6.913618656	64.37761667
														QEALPLHQETK	1	10	1	N-Term(iTRAQ8plex); K11(iTRAQ8plex)	0.96	0.799	0.709	3.61	-	0	1902.08329	3	634.6992798	-3.798568961	16.92354333
														EQMEAEIAHLK	1	10	1	N-Term(iTRAQ8plex); M3(Oxidation); K11(iTRAQ8plex)	1.192	0.956	0.802	3.28	-	0	1923.03531	3	641.6832886	-5.878117407	17.08160333
														LVNELTEK	1	10	1	N-Term(iTRAQ8plex); K8(iTRAQ8plex)	1.121	0.888	0.632	3.18	-	0	1553.9312	2	777.4692383	-3.025460522	39.43082333
														STYVTEVR	1	10	1	N-Term(iTRAQ8plex)	1.075	0.964	0.569	3.15	-	0	1258.69194	2	629.8496094	-2.009363781	18.23735
														ECMAGTSGSEEVK	2	10	1	N-Term(iTRAQ8plex); C2(Methylthio); M3(Oxidation); K13(iTRAQ8plex)	1.791	1.692	-	3.15	46	0	1997.93515	3	666.6499023	-4.36626721	22.66713333
														EREHLEMELEK	1	10	1	N-Term(iTRAQ8plex); K11(iTRAQ8plex)	1.154	1.125	0.782	-	28	1	2051.1082	2	1026.057739	1.462914352	21.04941333
118600975	STXBP3	syntaxin-binding protein 3	NP_009200.2	7.43	2	2	4	592	67.7	7.80	1.363	1.039	0.941																
														AAYIYFTDFCPDNLFNK	3	1	1	N-Term(iTRAQ8plex); C10(Methylthio); K17(iTRAQ8plex)	1.363	1.039	0.941	4.07	55	0	2696.33109	3	899.4485474	-1.42544073	58.07877
														GKDAIMETMADQIVTVCATLDENPGVR	1	1	1	N-Term(iTRAQ8plex); K2(iTRAQ8plex); C17(Methylthio)	-	-	-	-	21	1	3531.79026	3	1177.934937	3.554576971	69.29746667
118601081	HNRNPUL2	heterogeneous nuclear ribonucleoprotein U-like protein 2	NP_001073027.1	18.47	8	8	28	747	85.1	4.91	0.815	0.438	0.466																
														EEPFFPPPEEFVFIHAVPVEER	8	1	1	N-Term(iTRAQ8plex)	0.859	0.465	0.59	7.57	56	0	2945.49332	3	982.5026245	-3.222215708	75.92068
														LQEALDAEMLEDEAGGGGAGPGGACK	2	1	1	N-Term(iTRAQ8plex); C25(Methylthio); K26(iTRAQ8plex)	-	0.55	0.458	5.5	28	0	3100.49941	4	775.8803101	4.746994735	43.18382833
														NFILDQCNVYNSGQR	2	1	1	N-Term(iTRAQ8plex); C7(Methylthio)	0.78	0.402	0.466	5.27	69	0	2121.02056	2	1061.013916	2.179663577	40.05480333
														YNVLGAETVLNQMR	2	1	1	N-Term(iTRAQ8plex)	0.926	0.508	0.55	5.01	48	0	1912.04338	2	956.5253296	8.865520439	40.946155
														ALLPHVLCK	6	1	1	N-Term(iTRAQ8plex); C8(Methylthio); K9(iTRAQ8plex)	0.72	0.379	0.408	3.71	25	0	1647.98948	3	550.0013428	-0.241038971	35.09885833
														VGWSVDFSRPQLGEDEFSYGFDGR	3	1	1	N-Term(iTRAQ8plex)	0.872	0.173	0.368	3.62	21	0	3054.45669	4	764.3696289	1.003270079	47.38202833
														TIEECEVILMVGLPGSGK	2	1	1	N-Term(iTRAQ8plex); C5(Methylthio); K18(iTRAQ8plex)	0.723	0.249	0.622	3.09	40	0	2529.34165	3	843.7854004	-6.76238346	64.00685167
														EGCTEVSLLR	3	1	1	N-Term(iTRAQ8plex); C3(Methylthio)	0.958	0.57	0.557	2.96	30	0	1456.74138	2	728.8743286	-1.916396057	33.37945167
														ILLFK	2	1	1	N-Term(iTRAQ8plex); K5(iTRAQ8plex)	0.919	0.401	0.559	-	21	0	1241.84259	3	414.6190491	-1.262102951	42.73066167
119220557	PAICS	multifunctional protein ADE2 isoform 2	NP_001072992.1	19.53	5	5	12	425	47.0	7.23	0.920	4.050	3.772																
														IKAEYEGDGIPTVFVAVAGR	4	2	1	N-Term(iTRAQ8plex); K2(iTRAQ8plex)	1.587	4.607	4.095	5.24	45	1	2700.49948	3	900.8380127	-6.915929504	40.84629333
														EIVLADVIDNDSWR	4	3	1	N-Term(iTRAQ8plex)	1.109	4.885	4.666	5.03	60	0	1949.03299	3	650.3491821	2.448309872	49.98325833
														VVVLMGSTSDLGHCEK	1	3	1	N-Term(iTRAQ8plex); C14(Methylthio); K16(iTRAQ8plex)	1.078	1.489	1.767	4.84	-	0	2329.21506	3	777.0765381	-1.039531397	40.93119833
														ACGNFGIPCELR	2	3	1	N-Term(iTRAQ8plex); C2(Methylthio); C9(Methylthio)	0.592	2.36	2.21	3.91	36	0	1675.76775	2	838.3875122	-3.171982481	50.094605
														CGETAFIAPQCEMIPIEWVCR	1	3	1	N-Term(iTRAQ8plex); C1(Methylthio); C11(Methylthio); C20(Methylthio)	-	-	-	-	22	0	2838.26273	3	946.7590942	1.23144514	70.55374667
119226260	CHERP	calcium homeostasis endoplasmic reticulum protein	NP_006378.3	3.93	2	2	5	916	103.6	9.04	-	-	-																
														LLAAVEAFYSPPSHDRPR	4	1	1	N-Term(iTRAQ8plex)	-	-	-	5.47	43	0	2330.25893	4	583.3201904	1.287641776	53.826175
														QEQVTAAVAHAVEQQMQK	1	1	1	N-Term(iTRAQ8plex); K18(iTRAQ8plex)	-	-	-	2.75	-	0	2604.39077	4	651.8531494	-4.48992674	39.59373
119393891	GAA	lysosomal alpha-glucosidase preproprotein	NP_000143.2	1.89	1	1	1	952	105.3	6.00	0.777	0.988	0.866																
														AHFPLDVQWNDLDYMDSR	2	1	1	N-Term(iTRAQ8plex)	0.777	0.988	0.866	4.23	20	0	2526.20139	3	842.7386475	-0.471920882	46.44463833
119395754	KRT5	keratin, type II cytoskeletal 5	NP_000415.2	60.68	28	44	1417	590	62.3	7.74	0.218	0.132	0.142																
														QCANLQNAIADAEQR	28	1	1	N-Term(iTRAQ8plex); C2(Methylthio)	0.199	0.163	0.151	6.76	84	0	1994.9644	2	997.9858398	-2.305420835	32.18758833
														GLGVGFGSGGGSSSSVK	14	1	1	N-Term(iTRAQ8plex); K17(iTRAQ8plex)	0.138	0.116	0.215	6.56	97	0	2048.12162	3	683.3787231	-0.822761887	29.06181167
														MFFDAELSQMQTHVSDTSVVLSMDNNR	85	1	1	N-Term(iTRAQ8plex)	0.159	0.131	0.091	6.49	58	0	3406.6124	3	1141.536621	-0.481000118	50.792955

														MFFDAELSQMQTHVSDTSVVLSMDNNR	26	1	1	N-Term(iTRAQ8plex); M1(Oxidation)	0.218	0.109	0.22	5.93	75	0	3422.59897	3	1141.536621	-0.481000118	50.792955
														SFSTASAITPSVSR	43	1	1	N-Term(iTRAQ8plex)	0.16	0.067	0.076	5.7	88	0	1714.92742	2	857.9673462	-0.195656787	25.59052
														VSLAGACGVGGYGSR	135	1	1	N-Term(iTRAQ8plex); C7(Methylthio)	0.264	0.179	0.186	5.3	70	0	1703.84953	2	852.4354248	7.313196651	34.704555
														MFFDAELSQMQTHVSDTSVVLSMDNNR	9	1	1	N-Term(iTRAQ8plex); M1(Oxidation); M23(Oxidation)	1.271	-	1.441	5.17	42	0	3438.5914	3	1141.536621	-0.481000118	50.792955
														VDALMDEINFMK	96	1	1	N-Term(iTRAQ8plex); M11(Oxidation); K12(iTRAQ8plex)	0.234	0.151	0.154	5.17	77	0	2050.07451	2	1025.540894	-3.153939329	71.53923667
														VDALMDEINFMK	110	1	1	N-Term(iTRAQ8plex); K12(iTRAQ8plex)	0.156	0.121	0.086	5.1	72	0	2034.08237	2	1025.540894	-3.153939329	71.53923667
														TEAESWYQTK	21	1	1	N-Term(iTRAQ8plex); K10(iTRAQ8plex)	0.236	0.121	0.202	5.02	48	0	1850.97014	2	925.9912109	0.369510634	25.53017167
														TTAENEFVMLK	27	1	1	N-Term(iTRAQ8plex); K11(iTRAQ8plex)	0.181	0.067	0.064	5.02	47	0	1891.04241	2	946.0239868	-2.566992448	44.43880833
														YEELQQTAGR	48	1	1	N-Term(iTRAQ8plex)	0.233	0.128	0.123	5.02	48	0	1498.77825	3	500.2674255	4.925354814	25.78864833
														VDALMDEINFMK	14	1	1	N-Term(iTRAQ8plex); M5(Oxidation); M11(Oxidation); K12(iTRAQ8plex)	0.379	0.206	0.278	4.96	47	0	2066.06938	2	1025.540894	-3.153939329	71.53923667
														ISISTSGGSFR	29	1	1	N-Term(iTRAQ8plex)	0.189	0.081	0.076	4.85	50	0	1415.77849	3	472.5996399	3.351589301	23.09418833
														LAELEEALQK	45	1	1	N-Term(iTRAQ8plex); K10(iTRAQ8plex)	0.144	0.128	0.114	4.47	61	0	1752.03032	2	876.5253906	4.07714191	43.24912333
														NMQDLVEDFK	25	1	1	N-Term(iTRAQ8plex); K10(iTRAQ8plex)	0.23	0.161	0.152	4.45	58	0	1846.97637	2	923.993042	-2.243629222	31.98679
														TTAENEFVMLKK	4	1	1	N-Term(iTRAQ8plex); M9(Oxidation); K11(iTRAQ8plex); K12(iTRAQ8plex)	0.388	0.093	0.068	4.33	27	1	2339.33999	3	775.1196899	-0.587397078	34.95341167
														QCANLQNAIADAEQRGELALK	4	1	1	N-Term(iTRAQ8plex); C2(Methylthio); K21(iTRAQ8plex)	-	-	-	4.27	33	1	2910.54995	4	728.3929443	3.877330386	53.22361667
														NKLAELEEALQK	8	1	1	N-Term(iTRAQ8plex); K2(iTRAQ8plex); K12(iTRAQ8plex)	0.236	0.146	0.155	4.16	35	1	2298.3749	4	575.3501587	-0.35521315	38.96119667
														KQCANLQNAIADAEQR	2	1	1	N-Term(iTRAQ8plex); K1(iTRAQ8plex); C3(Methylthio)	0.493	0.276	0.263	4.02	42	1	2427.26108	3	809.7585449	-3.399215864	34.87185
														NMQDLVEDFK	8	1	1	N-Term(iTRAQ8plex); M2(Oxidation); K10(iTRAQ8plex)	0.242	0.122	0.144	3.97	39	0	1862.97562	2	923.993042	-2.243629222	31.98679
														SLYNLGGSK	55	1	1	N-Term(iTRAQ8plex); K9(iTRAQ8plex)	0.238	0.149	0.147	3.91	51	0	1546.90337	2	773.954895	-1.556582213	19.58279
														EYQELMNTK	20	1	1	N-Term(iTRAQ8plex); K9(iTRAQ8plex)	0.329	0.194	0.205	3.69	58	0	1763.93901	2	882.4780273	1.681889824	27.58457833
														AQYEEIANR	22	1	1	N-Term(iTRAQ8plex)	0.199	0.11	0.109	3.58	39	0	1397.74079	3	466.582489	0.18064325	18.493545
														EYQELMNTK	7	1	1	N-Term(iTRAQ8plex); M6(Oxidation); K9(iTRAQ8plex)	0.362	0.223	0.238	3.53	48	0	1779.94158	2	882.4780273	1.681889824	27.58457833
														TTAENEFVMLKK	2	1	1	N-Term(iTRAQ8plex); K11(iTRAQ8plex); K12(iTRAQ8plex)	0.393	0.194	0.179	3.32	27	1	2323.34452	3	775.1196899	-0.587397078	34.95341167
														NTKHEISEMNR	2	1	1	N-Term(iTRAQ8plex); K3(iTRAQ8plex)	0.188	0.015	-	3.21	33	1	1967.05913	2	984.0332031	0.116076836	12.09965
														HEISEMNR	9	1	1	N-Term(iTRAQ8plex)	0.384	0.33	0.278	3.01	32	0	1319.66516	3	440.559906	-2.119122711	12.06033333
														LLREYQELMNTK	2	1	1	N-Term(iTRAQ8plex); K12(iTRAQ8plex)	0.374	-	0.141	2.86	-	1	2146.20791	4	537.3074341	-3.338377355	44.98190167
														FVSTTSSSR	3	1	1	N-Term(iTRAQ8plex)	0.109	0.025	0.016	2.74	25	0	1275.68828	2	638.3477783	2.852358748	19.05276667
														AEIDNVKK	4	1	1	N-Term(iTRAQ8plex); K7(iTRAQ8plex); K8(iTRAQ8plex)	0.141	0.085	0.054	2.73	26	1	1829.13064	4	458.0381165	2.561129435	17.63281167
														VSLAGACGVGGYGSR	8	1	1	N-Term(iTRAQ8plex); C7(Methylthio); Y12(iTRAQ8plex)	0.211	0.114	0.136	-	41	0	2008.05303	2	852.4354248	7.313196651	34.704555
														AEIDNVK	4	1	1	N-Term(iTRAQ8plex); K7(iTRAQ8plex)	0.241	0.117	0.121	-	37	0	1396.82663	3	466.2803955	0.724480343	16.996245
														SLYNLGGSKR	1	1	1	N-Term(iTRAQ8plex); K9(iTRAQ8plex)	0.535	0.271	0.259	-	34	1	1702.99944	2	852.0033569	-3.877894888	17.15041667
														YEELQQTAGR	1	1	1	N-Term(iTRAQ8plex); Y1(iTRAQ8plex)	0.169	0.059	0.041	-	30	0	1802.98345	3	500.2674255	4.925354814	25.78864833
														SLYNLGGSK	1	1	1	N-Term(iTRAQ8plex); Y3(iTRAQ8plex); K9(iTRAQ8plex)	0.209	0.066	0.069	-	29	0	1851.10442	2	773.954895	-1.556582213	19.58279
														QSSVSFR	2	1	1	N-Term(iTRAQ8plex)	0.152	0.155	0.141	-	22	0	1114.61577	2	557.8115234	-0.054767752	14.575165
														TSFTSVSR	2	1	1	N-Term(iTRAQ8plex)	0.15	0.028	0.016	-	22	0	1188.64995	2	594.8286133	-2.238974982	14.48448833
														LALDVEIATYR	117	10	7	N-Term(iTRAQ8plex)	-	-	-	4.83	67	0	1567.90409	3	523.3062134	2.771589637	39.12087
														LALDVEIATYR	8	10	7	N-Term(iTRAQ8plex); Y10(iTRAQ8plex)	-	-	-	-	60	0	1872.106	3	523.3062134	2.771589637	39.12087
														DVDAAYMNKVELEAK	10	2	2	N-Term(iTRAQ8plex); K9(iTRAQ8plex); K15(iTRAQ8plex)	-	-	-	6.03	48	1	2608.43657	2	1304.721924	-2.074140429	31.19943833
														DVDAAYMNKVELEAK	3	2	2	N-Term(iTRAQ8plex); M7(Oxidation); K9(iTRAQ8plex); K15(iTRAQ8plex)	-	-	-	5.55	-	1	2624.43545	2	1304.721924	-2.074140429	31.19943833
														FLEQQNK	13	31	8	N-Term(iTRAQ8plex); K7(iTRAQ8plex)	-	-	-	-	33	0	1514.87885	3	505.631134	0.090799082	15.97476833
														VEIEAK	5	1	3	N-Term(iTRAQ8plex); K6(iTRAQ8plex)	-	-	-	2.98	27	0	1296.79612	3	432.9381104	1.107730516	13.75300333
														GELAIK	4	2	6	N-Term(iTRAQ8plex); K6(iTRAQ8plex)	-	-	-	-	28	0	1238.78995	3	413.6025085	0.089671776	17.83946167
														FASFIDKVR	16	27	8	N-Term(iTRAQ8plex); K7(iTRAQ8plex)	-	-	-	3.33	31	1	1691.01076	4	423.5080261	0.114317571	39.05999667
														FASFIDK	57	28	8	N-Term(iTRAQ8plex); K7(iTRAQ8plex)	-	-	-	3.23	35	0	1435.83818	2	718.4240723	0.227470368	33.40336167
														NKYEDEINK	1	9	5	N-Term(iTRAQ8plex); K2(iTRAQ8plex); K9(iTRAQ8plex)	-	-	-	-	27	1	2065.16616	4	517.0469971	-1.50249351	14.99920833
														QNLEPLFEQYINNLR	50	4	3	N-Term(iTRAQ8plex)	-	-	-	6.54	76	0	2195.17461	2	1098.090942	-0.753085595	58.40959667
														WTLLQEQGTK	50	4	3	N-Term(iTRAQ8plex); K10(iTRAQ8plex)	-	-	-	4.48	49	0	1812.04447	2	906.5276489	0.242781404	32.60840167
														QLDSIVGER	26	3	3	N-Term(iTRAQ8plex)	-	-	-	3.72	66	0	1320.74237	3	440.9189758	-0.103774792	25.30154833
														QNLEPLFEQYINNLRR	5	4	3	N-Term(iTRAQ8plex)	-	-	-	3.15	27	1	2351.28554	4	588.5768433	3.471235937	52.94203
														NLDLDSIIAEVK	64	8	5	N-Term(iTRAQ8plex); K12(iTRAQ8plex)	-	-	-	5.73	89	0	1938.13909	2	969.5731812	1.173488892	50.31108
														KLLEGEECR	13	13	6	N-Term(iTRAQ8plex); K1(iTRAQ8plex); C8(Methylthio)	-	-	-	2.79	31	1	1730.93911	3	577.6514282	0.43855829	29.74912
														LLEGEECR	16	16	6	N-Term(iTRAQ8plex); C7(Methylthio)	-	-	-	2.75	36	0	1298.63898	3	433.551178	0.260569375	30.10390833
														LDSELR	1	16	3	N-Term(iTRAQ8plex)	-	-	-	-	26	0	1036.59331	2	518.800293	-0.706142412	18.550555
														DVDAAYMNK	25	11	4	N-Term(iTRAQ8plex); K9(iTRAQ8plex)	-	-	-	4.2	50	0	1634.86492	2	817.9366455	-0.50579336	22.28227833
														DVDAAYMNK	13	11	4	N-Term(iTRAQ8plex); M7(Oxidation); K9(iTRAQ8plex)	-	-	-	3.52	47	0	1650.86186	2	817.9366455	-0.50579336	22.28227833
														KDVDAAYMNK	3	11	4	N-Term(iTRAQ8plex); K1(iTRAQ8plex); K10(iTRAQ8plex)	-	-	-	3	31	1	2067.16056	3	689.7250366	-3.199890157	21.25677833
119829187	COL17A1	collagen alpha-1(XVII) chain	NP_000485.3	4.81	5	5	8	1497	150.3	8.79	0.624	0.455	0.396																
														EMELLMTK	3	1	1	N-Term(iTRAQ8plex); K9(iTRAQ8plex)	0.723	0.358	0.324	3.8	48	0	1715.98807	2	858.4932861	-6.017353707	65.60491667
														AHSPASTLPNSPGSTFER	2	1	1	N-Term(iTRAQ8plex)	0.624	0.502	0.71	3.36	30	0	2160.09446	3	720.7030029	-1.988691247	20.445645
														VFTASPASIAATSFSEDTLKK	1	1	1	N-Term(iTRAQ8plex); K20(iTRAQ8plex); K21(iTRAQ8plex)	-	-	-	3.35	-	1	3083.74428	4	771.6915283	1.641543781	40.450815
														SELISYLTSPDVR	1	1	1	N-Term(iTRAQ8plex)	0.51	0.735	0.472	2.94	-	0	1783.97063	2	892.4889526	-2.092325122	49.30797833
														SILPYGDSMDR	1	1	1	N-Term(iTRAQ8plex)	0.414	0.336	0.359	-	29	0	1557.78813	2	779.3977051	-0.223242007	28.79611
														FILLEK	4	4	1	N-Term(iTRAQ8plex); K6(iTRAQ8plex)	0.642	0.182	0.164	-	24	0	1370.88727	3	457.6329346	-1.831547092	55.23515833
119943112	DHCR7	7-dehydrocholesterol reductase	NP_001351.2	8.21	4	4	15	475	54.5	8.70	0.925	0.323	0.253																
														VIECSYTSADGQR	2	1	1	N-Term(iTRAQ8plex); C4(Methylthio)	0.954	0.206	0.17	4.22	66	0	1778.84148	2	889.9243774	3.359469006	29.794495
														LLVSGFWGVAR	7	1	1	N-Term(iTRAQ8plex)	0.901	0.279	0.231	3.78	69	0	1508.88132	3	503.631958	-5.176369046	40.65075
														LSDIWAK	4	2	1	N-Term(iTRAQ8plex); K7(iTRAQ8plex)	0.777	0.306	0.274	2.92	31	0	1440.87041	2	720.9388428	2.302515795	30.48415667
														YTAAVPYR	2	1	1	N-Term(iTRAQ8plex)	1.024	0.389	0.353	-	23	0	1244.69121	2	622.8492432	-2.299037587	21.13295667
119964726	IGF2R	cation-independent mannose-6-phosphate receptor precursor	NP_000867.2	2.45	6	6	13	2491	274.1	5.91	0.673	0.579	0.569																
														ETSDCSYLFEWR	2	1	1	N-Term(iTRAQ8plex); C5(Methylthio)	0.765	0.5	0.471	4.73	45	0	1885.84538	2	943.4263306	2.721438807	47.45157333
														HFFINICHR	4	1	1	N-Term(iTRAQ8plex); C7(Methylthio)	0.683	0.594	0.591	4.35	48	0	1536.80105	4	384.955719	9.164359042	33.712875
														YVDGDLCPDGIR	2	1	1	N-Term(iTRAQ8plex); C7(Methylthio)	0.652	0.579	0.538	3.75	22	0	1672.80144	2	836.9043579	2.25185143	35.02751667
														ATLITFLCDR	2	1	1	N-Term(iTRAQ8plex); C8(Methylthio)	0.673	0.444	0.45	3.41	31	0	1502.81401	2	751.9106445	8.46421606	59.85784167
														YVDQVLQLVYK	2	1	1	N-Term(iTRAQ8plex); K11(iTRAQ8plex)	0.707	0.948	0.925	3.32	35	0	1976.16336	3	659.3926392	-2.184571179	47.50481833
														IHLVCSR	1	1	1	N-Term(iTRAQ8plex); C5(Methylthio)	0.649	0.912	0.795	-	22	0	1177.64518	3	393.2199097	-3.020048965	24.86196333
120433590	DBI	acyl-CoA-binding protein isoform 3	NP_001073331.1	50.57	3	3	9	87	10.0	6.57	0.522	1.294	1.972																
														QATVGDINTERPGMLDFTGK	2	7	1	N-Term(iTRAQ8plex); M14(Oxidation); K20(iTRAQ8plex)	-	1.295	3.833	4.65	27	0	2774.44814	4	694.3674927	-4.417266517	27.800675
														QATVGDINTERPGMLDFTGK	2	7	1	N-Term(iTRAQ8plex); K20(iTRAQ8plex)	0.351	0.948	1.476	3.84	-	0	2758.45522	4	694.3674927	-4.417266517	27.800675
														TKPSDEEMLFIYGHYK	1	6	1	N-Term(iTRAQ8plex); K2(iTRAQ8plex); K16(iTRAQ8plex)	0.483	1.152	1.219	3.22	-	0	2870.54311	4	718.3912354	-3.295464789	32.62042667
														WDAWNELK	4	7	1	N-Term(iTRAQ8plex); K8(iTRAQ8plex)	0.746	1.544	1.972	3.2	37	0	1669.90715	2	835.4572144	-5.203408496	35.35898667
120587023	UTP20	small subunit processome component 20 homolog	NP_055318.2	1.97	4	4	8	2785	318.2	7.39	1.986	0.984	1.124																
														NIQGTITGDILPR	2	1	1	N-Term(iTRAQ8plex)	1.502	0.877	1.212	4.2	27	0	1701.99114	2	851.4992065	6.466053613	33.458245
														ILNHFDVQLPESMEDDGLSER	2	1	1	N-Term(iTRAQ8plex)	1.313	1.026	1.045	3.64	27	0	2748.34641	3	916.7869873	0.399281568	41.223455
														LAVSAQSEPAR	2	1	1	N-Term(iTRAQ8plex)	2.661	0.944	1.124	3.51	46	0	1432.81487	2	716.9110718	6.06193786	20.746335
														LLETYLILVK	2	1	1	N-Term(iTRAQ8plex); K10(iTRAQ8plex)	2.626	1.038	-	2.96	29	0	1813.16379	3	605.0594482	-1.163614367	52.72618667
122056623	PRNP	major prion protein preproprotein	NP_001073590.1	4.74	1	1	2	253	27.6	9.00	0.677	0.750	0.635																
														VVEQMCITQYER	2	1	1	N-Term(iTRAQ8plex); C6(Methylthio)	0.677	0.75	0.635	3.93	21	0	1848.88835	2	924.9478149	-4.137100639	43.76234667
122937345	MYO5B	unconventional myosin-Vb	NP_001073936.1	1.14	1	1	1	1848	213.5	7.20	-	-	-																
														YAMRYFATVGGSASETNIEEK	1	1	1	N-Term(iTRAQ8plex); K21(iTRAQ8plex)	-	-	-	2.83	-	1	2932.49257	4	733.8786011	-1.560748411	50.84449
123173757	RAVER1	ribonucleoprotein PTB-binding 1	NP_597709.2	8.73	4	4	5	756	79.5	8.92	0.947	1.327	2.173																
														SGAEVEAGDAAER	2	2	1	N-Term(iTRAQ8plex)	0.947	2.235	2.173	3.62	48	0	1565.77715	2	783.3922119	3.963468324	15.845015
														RPAEGPPTNPPAPGGGSSSK	1	1	1	N-Term(iTRAQ8plex); K21(iTRAQ8plex)	2.137	1.327	2.904	3.43	-	0	2556.34731	4	639.8422852	-6.028349487	13.727985
														GLPGDVTNQEVHDLLSDYELK	1	2	1	N-Term(iTRAQ8plex); K21(iTRAQ8plex)	-	-	-	3.43	-	0	2950.56386	4	738.3964233	0.67857517	48.629475
														EPALGPHGPSR	1	2	1	N-Term(iTRAQ8plex)	0.461	0.316	0.241	3.28	-	0	1421.77878	3	474.5977783	-1.068794303	13.06293333
124028525	FARSB	phenylalanine--tRNA ligase beta subunit	NP_005678.3	12.22	5	6	12	589	66.1	6.84	1.669	3.413	3.908																
														DRYDSFIELQEK	1	2	1	N-Term(iTRAQ8plex); K12(iTRAQ8plex)	0.845	0.948	0.824	4.41	-	1	2151.14096	3	717.7185059	-6.17206036	26.81817667
														LFEISDIVIK	2	2	1	N-Term(iTRAQ8plex); K10(iTRAQ8plex)	1.669	3.413	3.644	4.37	39	0	1785.0842	3	595.699585	-7.859386787	44.2656
														ALVAIGTHDLDTLSGPFTYTAK	2	2	1	N-Term(iTRAQ8plex); K22(iTRAQ8plex)	-	-	-	3.83	-	0	2899.61245	4	725.6585693	3.395682674	53.01044833
														YDLLCLEGLVR	2	1	1	N-Term(iTRAQ8plex); C5(Methylthio)	1.142	3.352	3.908	3.76	33	0	1643.88603	2	822.4466553	3.504882185	54.66563333
														IRPFAVAAVLR	4	2	1	N-Term(iTRAQ8plex)	2.241	3.787	5.405	3.13	44	0	1516.95627	3	506.3236084	-4.416313648	32.484085
														LTELLR	1	4	2	N-Term(iTRAQ8plex)	-	-	-	-	20	0	1048.65776	2	524.8325195	-8.628990147	27.20188333
124028529	SYMPK	symplekin	NP_004810.2	1.73	2	2	3	1274	141.1	6.13	1.482	1.185	1.274																
														AVACSGAAQVR	2	1	1	N-Term(iTRAQ8plex); C4(Methylthio)	1.237	0.926	1.058	4.37	50	0	1382.72074	3	461.5784302	1.522344525	24.55083167
														LGGFVMNILSR	1	1	1	N-Term(iTRAQ8plex)	1.776	1.518	1.534	-	29	0	1510.87248	2	755.9398804	0.471795069	58.26126667
124494247	MYO1C	unconventional myosin-Ic isoform b	NP_001074419.1	16.38	14	14	48	1044	119.6	9.45	0.811	0.570	0.936																
														GVSFYEVPPHLFAVADTVYR	12	3	1	N-Term(iTRAQ8plex)	1.147	0.648	1.16	6.03	45	0	2571.35904	3	857.7901001	0.301686365	51.28011
														LLQSNPVLEAFGNAK	4	3	1	N-Term(iTRAQ8plex); K15(iTRAQ8plex)	0.969	0.607	1.203	5.71	46	0	2209.26908	3	737.0945435	-5.003545752	41.71028
														TLFATEDALEVR	3	3	1	N-Term(iTRAQ8plex)	0.825	0.443	0.879	4.92	46	0	1668.9146	2	834.9609375	2.125381373	35.380335
														NFHIFYQLLEGGEEETLR	6	3	1	N-Term(iTRAQ8plex)	0.468	0.679	0.746	4.79	63	0	2499.29093	3	833.7650757	-0.611175541	59.48222667
														CPENAFFLDHVR	8	3	1	N-Term(iTRAQ8plex); C1(Methylthio)	0.88	0.663	0.716	4.71	41	0	1797.86606	3	599.9603882	-2.909559133	33.65296833
														GIISILDEECLRPGEATDLTFLEK	1	3	1	N-Term(iTRAQ8plex); C10(Methylthio); K24(iTRAQ8plex)	-	-	-	4	-	0	3316.78725	4	829.9522705	7.314262087	64.85929333
														HHPHFLTHK	3	3	1	N-Term(iTRAQ8plex); K9(iTRAQ8plex)	0.634	0.542	1.105	3.82	29	0	1761.99845	3	588.0043335	-7.754865925	10.65626667
														YLGLLENLR	2	3	1	N-Term(iTRAQ8plex)	0.882	0.378	0.983	3.2	26	0	1394.83318	2	697.9202271	1.617527887	47.64871333
														YMDVQFDFK	2	3	1	N-Term(iTRAQ8plex); K9(iTRAQ8plex)	0.806	0.516	0.706	3.16	23	0	1800.9296	3	600.9813843	-8.602067495	64.43384167
														SAICIQSWWR	2	3	1	N-Term(iTRAQ8plex); C4(Methylthio)	0.797	0.564	1.62	2.91	25	0	1599.79668	2	800.4019775	-6.934532384	71.05298

														FDEVLIR	1	3	1	N-Term(iTRAQ8plex)	0.606	0.314	0.821	2.83	-	0	1195.69377	2	598.3505249	-4.247003226	34.59704667
														GAPVGGHILSYLLEK	1	3	1	N-Term(iTRAQ8plex); K15(iTRAQ8plex)	0.467	-	0.424	2.73	-	0	2162.27993	4	541.3254395	0.248179014	44.735625
														NFHIFYQLLEGGEEETLRR	2	3	1	N-Term(iTRAQ8plex)	-	-	-	2.7	31	1	2655.37929	4	664.6002808	-1.516970903	54.485455
														VTMESALTAR	1	2	1	N-Term(iTRAQ8plex); M3(Oxidation)	0.816	0.457	0.796	-	26	0	1398.75994	2	699.883606	2.487198822	20.97075667
124494254	PA2G4	proliferation-associated protein 2G4	NP_006182.2	37.82	12	12	77	394	43.8	6.55	1.092	2.046	1.886																
														VAHSFNCTPIEGMLSHQLK	7	1	1	N-Term(iTRAQ8plex); C7(Methylthio); K19(iTRAQ8plex)	0.853	2.376	2.631	6.27	32	0	2766.42295	3	922.8125	-4.36504115	44.61578667
														HELLQPFNVLYEK	15	1	1	N-Term(iTRAQ8plex); K13(iTRAQ8plex)	1.107	1.691	1.584	5.48	39	0	2238.26145	3	746.758667	-5.738191778	39.29129333
														SLVEASSSGVSVLSLCEK	6	1	1	N-Term(iTRAQ8plex); C16(Methylthio); K18(iTRAQ8plex)	0.846	1.661	1.59	5.48	52	0	2449.30423	3	817.1062622	-3.939457081	48.3701
														GIAFPTSISVNNCVCHFSPLK	3	1	1	N-Term(iTRAQ8plex); C13(Methylthio); C15(Methylthio); K21(iTRAQ8plex)	-	-	-	5.03	25	0	2934.4977	4	734.3798828	0.60932745	56.67563667
														ITSGPFEPDLYK	5	1	1	N-Term(iTRAQ8plex); K12(iTRAQ8plex)	0.91	3.44	1.886	4.41	41	0	1975.08341	2	988.0449829	-8.603234579	27.885675
														EGEFVAQFK	3	1	1	N-Term(iTRAQ8plex); K9(iTRAQ8plex)	1.829	3.866	3.384	3.62	35	0	1662.92009	2	831.9636841	-6.656817723	26.572425
														AAHLCAEAALR	19	1	1	N-Term(iTRAQ8plex); C5(Methylthio)	0.886	1.636	1.43	3.54	53	0	1475.76679	3	492.5937805	-6.56878671	34.55761
														GDAMIMEETGK	4	1	1	N-Term(iTRAQ8plex); K11(iTRAQ8plex)	1.563	5.498	3.822	3.28	32	0	1789.91233	3	597.30896	-9.030982662	32.51782833
														RFDAMPFTLR	8	1	1	N-Term(iTRAQ8plex)	1.115	2.678	2.581	2.94	35	1	1557.845	3	519.953186	-4.086699583	29.63262667
														SDQDYILK	2	1	1	N-Term(iTRAQ8plex); K8(iTRAQ8plex)	1.176	1.302	1.941	2.75	34	0	1589.88738	2	795.4473267	-7.626074567	16.69745833
														AFFSEVER	4	1	1	N-Term(iTRAQ8plex)	1.204	2.568	2.075	2.75	31	0	1288.67583	2	644.8415527	-6.287423934	22.72947833
														ALLQSSASR	1	1	1	N-Term(iTRAQ8plex)	1.123	2.436	2.133	-	27	0	1236.71782	2	618.8625488	-2.870714475	17.28579833
125656165	THOC2	THO complex subunit 2	NP_001075019.1	0.50	1	1	2	1593	182.7	8.44	1.453	1.333	1.506																
														GEFLHLCR	2	16	1	N-Term(iTRAQ8plex); C7(Methylthio)	1.453	1.333	1.506	2.9	37	0	1324.67612	3	442.2302246	-3.511591855	33.76704333
125988409	IK	protein Red	NP_006074.2	2.51	1	1	1	557	65.6	6.64	1.107	1.977	1.763																
														FAGSAGWEGTESLK	1	1	1	N-Term(iTRAQ8plex); K14(iTRAQ8plex)	1.107	1.977	1.763	-	31	0	2048.08023	3	683.3649292	-5.222916644	43.43033333
126012562	LRP1	prolow-density lipoprotein receptor-related protein 1 preproprotein	NP_002323.2	0.88	2	2	3	4544	504.3	5.39	0.597	0.722	0.721																
														VPDEHMIPIENLMNPR	2	2	1	N-Term(iTRAQ8plex)	0.597	0.722	0.721	3.73	33	0	2209.14145	3	737.052002	0.122812133	39.879555
														AIVVDPLNGWMYWTDWEEDPKDSR	1	2	1	N-Term(iTRAQ8plex); K21(iTRAQ8plex)	-	-	-	-	25	1	3530.75014	4	883.4429932	-0.294136857	61.25807333
127139033	POR	NADPH--cytochrome P450 reductase	NP_000932.3	13.24	5	5	17	680	77.0	5.58	1.304	1.273	1.394																
														DVQNTFYDIVAELGAMEHAQAVDYIK	5	1	1	N-Term(iTRAQ8plex); K26(iTRAQ8plex)	-	0.956	2.271	6.08	39	0	3548.81679	4	887.9596558	-0.689844888	68.36685833
														DGALTQLNVAFSR	2	1	1	N-Term(iTRAQ8plex)	1.487	0.976	1.288	5.23	44	0	1695.94133	2	848.4743042	4.805872871	34.87211667
														EQFWPAVCEHFGVEATGEESSIR	4	1	1	N-Term(iTRAQ8plex); C8(Methylthio)	1.152	1.878	1.618	5.2	35	0	2958.37625	3	986.796936	1.951258046	56.70279667
														EVGETLLYYGCR	4	1	1	N-Term(iTRAQ8plex); C11(Methylthio)	1.323	1.278	1.394	4.55	60	0	1752.86394	2	876.9356079	2.103357956	42.98309833
														SDEDYLYREELAQFHR	2	1	1	N-Term(iTRAQ8plex)	-	-	-	3.61	24	1	2375.14492	4	594.541687	-5.078904042	29.02508
132566680	SCARB1	scavenger receptor class B member 1 isoform 2	NP_001076428.1	2.37	1	1	2	506	56.1	7.05	0.699	0.549	0.469																
														IDPSSLSFNMWK	2	2	1	N-Term(iTRAQ8plex); K12(iTRAQ8plex)	0.699	0.549	0.469	4.28	39	0	2033.09104	3	678.3685303	-3.744903473	69.44269833
140972063	PPP1R9B	neurabin-2	NP_115984.3	2.45	1	1	2	817	89.3	4.97	-	-	-																
														LEGYWGEAQSLCQAVDEHLR	2	1	1	N-Term(iTRAQ8plex); C12(Methylthio)	-	-	-	4.05	57	0	2654.27231	3	885.4289551	2.931004607	67.4376
143770802	MRPL1	39S ribosomal protein L1, mitochondrial precursor	NP_064621.3	8.00	2	2	4	325	36.9	8.78	0.640	0.379	0.437																
														AYPYMEGEPEDDVYLK	2	1	1	N-Term(iTRAQ8plex); K16(iTRAQ8plex)	0.805	0.373	0.407	6.12	51	0	2527.26358	3	843.0927124	4.460419999	34.20642167
														FQILDFTSPK	2	1	1	N-Term(iTRAQ8plex); K10(iTRAQ8plex)	0.509	0.386	0.469	3.82	34	0	1804.03435	2	902.520813	-6.747068559	68.46490333
145275185	C12orf10	UPF0160 protein MYG1, mitochondrial precursor	NP_067653.3	3.72	1	1	1	376	42.4	6.67	0.974	1.247	1.119																
														FQVDPSGEIVELAK	1	1	1	N-Term(iTRAQ8plex); K14(iTRAQ8plex)	0.974	1.247	1.119	3.21	-	0	2140.20755	3	714.0740356	-1.634241688	38.08101667
145309311	USP9X	probable ubiquitin carboxyl-terminal hydrolase FAF-X isoform 4	NP_001034680.2	0.47	1	1	2	2554	290.3	5.80	1.030	1.000	1.135																
														VVIQSNDDIASR	2	4	1	N-Term(iTRAQ8plex)	1.03	1	1.135	3.15	55	0	1620.88712	3	540.9672241	0.757494625	24.54072667
145309326	LAMC1	laminin subunit gamma-1 precursor	NP_002284.3	3.29	4	4	7	1609	177.5	5.12	0.603	0.420	0.523																
														DGSEASLEWSSER	3	1	1	N-Term(iTRAQ8plex)	0.522	0.357	0.458	4.76	59	0	1756.84038	2	878.9238281	6.379822624	25.932705
														TFAEVTDLDNEVNNMLK	2	1	1	N-Term(iTRAQ8plex); K17(iTRAQ8plex)	1.02	0.926	0.645	3.37	25	0	2561.33585	3	854.4501343	-0.761992212	45.59466333
														SQECYFDPELYR	1	1	1	N-Term(iTRAQ8plex); C4(Methylthio)	0.719	0.913	1.297	3.14	-	0	1899.86614	2	950.4367065	5.392046177	38.05083
														CLPFFNDRPWR	1	1	1	N-Term(iTRAQ8plex); C1(Methylthio)	0.321	0.306	0.325	2.85	-	0	1800.89811	3	600.9708862	0.057272205	46.67098
145386531	DDT	D-dopachrome decarboxylase	NP_001077861.1	28.81	3	3	11	118	12.7	7.30	1.102	3.199	3.320																
														FFPLESWQIGK	6	1	1	N-Term(iTRAQ8plex); K11(iTRAQ8plex)	1.384	4.519	4.741	5.31	44	0	1960.10282	2	980.5574341	-3.923129511	73.00267833
														LCAAAASILGKPADR	4	3	1	N-Term(iTRAQ8plex); C2(Methylthio); K11(iTRAQ8plex)	-	-	-	4.25	43	0	2111.18007	4	528.5504761	-5.929329893	51.869965
														ELALGQDR	1	3	1	N-Term(iTRAQ8plex)	0.737	0.903	0.869	-	24	0	1205.67449	2	603.3408813	-3.894272245	18.50617333
146219841	PNMAL2	PNMA-like protein 2	NP_065760.1	1.26	1	1	2	635	68.6	5.45	0.791	1.916	1.796																
														QVLSVMSK	2	1	1	N-Term(iTRAQ8plex); M6(Oxidation); K8(iTRAQ8plex)	0.791	1.916	1.796	2.83	-	0	1515.91715	3	505.9772339	9.665508548	29.01881667
146231948	CTNND1	catenin delta-1 isoform 3A	NP_001078937.1	19.23	10	10	66	832	92.6	7.15	1.328	0.783	0.507																
														DMDLTEVITGTLWNLSSHDSIK	5	15	1	N-Term(iTRAQ8plex); K22(iTRAQ8plex)	1.382	0.757	0.416	7.55	49	0	3083.61106	3	1028.54187	-2.255212577	67.58847333
														ALSAIADLLTNEHER	28	15	1	N-Term(iTRAQ8plex)	1.316	0.635	0.457	5.9	75	0	1957.06418	3	653.0262451	-0.755825205	55.35902833
														GIPVLVGLLDHPK	2	15	1	N-Term(iTRAQ8plex); K13(iTRAQ8plex)	0.821	0.611	0.338	5.74	49	0	1966.22623	3	656.0802612	-2.421317812	47.15578667
														HIEWESVLTNTAGCLR	9	15	1	N-Term(iTRAQ8plex); C14(Methylthio)	0.954	0.828	0.796	5.56	46	0	2179.09385	3	727.0361328	-0.163881193	50.71981667
														AAALVLQTIWGYK	12	15	1	N-Term(iTRAQ8plex); K13(iTRAQ8plex)	1.972	1.243	0.498	4.87	43	0	2042.21793	3	681.4130249	-0.67076022	64.14645167
														SYEDMIGEEVPSDQYYWAPLAQHER	4	15	1	N-Term(iTRAQ8plex)	1.073	0.658	0.516	4.83	38	0	3317.53891	3	1106.517822	0.775347914	44.716
														MEIVDHALHALTDEVIIPHSGWER	1	15	1	N-Term(iTRAQ8plex)	-	0.993	1.769	4.11	-	0	3072.57973	4	768.9003906	-2.674846778	65.71912333
														GYELLFQPEVVR	2	15	1	N-Term(iTRAQ8plex)	1.388	0.919	0.421	3.68	45	0	1753.97502	2	877.4911499	-2.302265003	45.88787167
														FHPEPYGLEDDQR	2	15	1	N-Term(iTRAQ8plex)	1.079	1.278	0.955	3.59	26	0	1906.91727	3	636.3106079	-3.388096396	20.58669667
														NLAVDAR	1	15	1	N-Term(iTRAQ8plex)	1.189	0.783	0.377	2.74	-	0	1062.62126	2	531.81427	0.312546976	17.85647667
148224884	PTPMT1	phosphatidylglycerophosphatase and protein-tyrosine phosphatase 1 isoform 1	NP_783859.1	5.97	1	1	2	201	22.8	9.77	1.061	0.879	0.566																
														GVITMNEEYETR	2	1	1	N-Term(iTRAQ8plex)	1.061	0.879	0.566	4.38	38	0	1745.87444	2	873.4408569	3.577546618	26.901405
148225659	ENDOD1	endonuclease domain-containing 1 protein precursor	NP_055851.1	2.60	1	1	2	500	55.0	5.71	0.543	0.485	0.739																
														ILEVVNQIQDEER	2	1	1	N-Term(iTRAQ8plex)	0.543	0.485	0.739	4.87	62	0	1889.04228	2	945.0247803	7.449609252	50.04627833
148470397	HNRNPF	heterogeneous nuclear ribonucleoprotein F	NP_001091678.1	36.14	7	9	98	415	45.6	5.58	0.873	1.172	1.122																
														ITGEAFVQFASQELAEK	12	1	1	N-Term(iTRAQ8plex); K17(iTRAQ8plex)	0.825	1.322	1.321	6.88	65	0	2476.35879	4	619.8451538	1.758248603	51.72750833
														MRPGAYSTGYGGYEEYSGLSDGYGFTTDLFGR	6	1	1	N-Term(iTRAQ8plex)	0.975	1.535	0.76	5.79	77	0	3778.74533	3	1260.253296	4.74579357	50.07732333
														VTGEADVEFATHEEAVAAMSK	4	1	1	N-Term(iTRAQ8plex); K21(iTRAQ8plex)	1.002	1.372	1.771	5.6	35	0	2800.41593	3	934.1434937	-4.464905675	37.31275333
														HSGPNSADSANDGFVR	6	1	1	N-Term(iTRAQ8plex)	0.661	0.818	0.869	5.05	58	0	1934.91867	3	645.6444092	-3.719541825	12.26823333
														QSGEAFVELGSEDDVK	4	1	1	N-Term(iTRAQ8plex); K16(iTRAQ8plex)	0.635	0.948	1.335	4.5	41	0	2318.18314	3	773.399231	-6.091701154	24.21125667
														DLSYCLSGMYDHR	10	1	1	N-Term(iTRAQ8plex); C5(Methylthio)	1.055	1.209	1.091	4.42	57	0	1909.84824	3	637.2875977	-3.462359528	43.805365
														TEMDWVLK	2	1	1	N-Term(iTRAQ8plex); K8(iTRAQ8plex)	0.776	1.487	1.785	3.36	39	0	1629.90019	2	815.4537354	-7.900616812	47.24826
														DLSYCLSGMYDHR	2	1	1	N-Term(iTRAQ8plex); Y4(iTRAQ8plex); C5(Methylthio)	0.452	1.623	1.511	-	32	0	2214.05044	3	637.2875977	-3.462359528	43.805365
														ATENDIYNFFSPLNPVR	22	13	2	N-Term(iTRAQ8plex)	-	-	-	5.86	67	0	2301.18462	2	1151.095947	1.245711112	55.62111333
														VHIEIGPDGR	30	14	3	N-Term(iTRAQ8plex)	-	-	-	3.86	57	0	1396.78022	3	466.2649231	-3.461720631	18.23122667
148491070	CTPS1	CTP synthase 1 isoform a	NP_001896.2	14.04	7	7	18	591	66.6	6.46	1.258	2.024	2.552																
														LYGDADYLEER	2	2	1	N-Term(iTRAQ8plex)	1.998	1.506	1.574	4.26	32	0	1647.81267	2	824.4099731	-2.50127397	31.62393667
														VPLLLEEQGVVDYFLR	2	2	1	N-Term(iTRAQ8plex)	-	-	-	4.13	87	0	2194.2471	3	732.0872192	2.065931788	68.37849167
														GLGLSPDLVVCR	4	2	1	N-Term(iTRAQ8plex); C11(Methylthio)	1.258	3.194	6.542	4.01	56	0	1578.86057	3	526.958374	-2.78902379	46.22736833
														LLETCSIALVGK	3	2	1	N-Term(iTRAQ8plex); C5(Methylthio); K12(iTRAQ8plex)	1.028	2.898	3.884	3.79	35	0	1901.08847	3	634.3676758	-9.241130273	51.78561
														LCSAHGVLVPGGFGVR	2	2	1	N-Term(iTRAQ8plex); C2(Methylthio)	0.941	0.9	0.996	3.7	30	0	1919.0178	3	640.3441162	-6.235959466	42.59707
														IYQYVINK	2	1	1	N-Term(iTRAQ8plex); K8(iTRAQ8plex)	4.978	2.721	2.642	3.2	24	0	1648.97367	3	550.3294067	-8.838428077	35.24504167
														RLDLPIER	3	2	1	N-Term(iTRAQ8plex)	0.984	1.347	1.869	3.01	42	1	1315.79404	3	439.2695313	-4.507392356	25.57444167
148491091	SLC25A24	calcium-binding mitochondrial carrier protein SCaMC-1 isoform 1	NP_037518.3	12.79	5	5	9	477	53.3	6.33	0.657	0.468	0.493																
														VLPAVGISYVVYENMK	2	3	1	N-Term(iTRAQ8plex); K16(iTRAQ8plex)	0.732	0.588	0.628	5.35	50	0	2390.3613	3	797.4586182	-0.046042334	58.16142333
														QLLAGGIAGAVSR	1	2	1	N-Term(iTRAQ8plex)	1.426	0.724	0.591	3.71	-	0	1516.91606	2	758.9616699	3.119436774	32.09631
														FWAYEQYK	4	3	1	N-Term(iTRAQ8plex); K8(iTRAQ8plex)	0.537	0.346	0.361	3.43	29	0	1742.93987	2	871.9735718	2.09739576	33.17449333
														NGDGVVDIGELQEGLR	1	1	1	N-Term(iTRAQ8plex)	0.87	1.25	1.215	3.25	-	0	1975.04997	2	988.0286255	5.120405405	35.20397167
														MNIFGGFR	1	3	1	N-Term(iTRAQ8plex)	0.558	0.372	0.411	-	24	0	1245.6657	2	623.3364868	-4.739864959	60.252545
148529014	DDB1	DNA damage-binding protein 1	NP_001914.3	7.37	6	6	9	1140	126.9	5.26	0.914	1.355	1.300																
														QSGESIDIITR	2	1	1	N-Term(iTRAQ8plex)	0.835	1.446	1.34	4.4	39	0	1522.84563	2	761.9264526	5.093985426	26.51715833
														ALYYLQIHPQELR	2	1	1	N-Term(iTRAQ8plex)	1.263	1.113	1.375	4.28	47	0	1948.08927	3	650.0346069	-3.353927013	38.753335
														LEELHVIDVK	2	1	1	N-Term(iTRAQ8plex); K10(iTRAQ8plex)	1.045	1.143	1.185	3.59	-	0	1803.069	3	601.694519	-8.118059299	33.84485167
														KTEPATGFIDGDLIESFLDISRPK	1	1	1	N-Term(iTRAQ8plex); K1(iTRAQ8plex); K24(iTRAQ8plex)	-	-	-	3.33	-	1	3561.98743	5	713.2033081	-1.630349001	56.79131
														SVLLLAYKPMEGNFEEIAR	1	1	1	N-Term(iTRAQ8plex); K8(iTRAQ8plex)	0.739	1.585	1.136	3.29	-	0	2788.55564	3	930.1900635	1.006212553	49.869805
														IEHSFWR	1	1	1	N-Term(iTRAQ8plex)	0.619	1.471	1.384	2.98	-	0	1278.68268	3	426.8990784	-5.466127506	22.37196
148536853	COPA	coatomer subunit alpha isoform 2	NP_004362.2	12.99	11	11	26	1224	138.3	7.66	0.922	1.415	1.465																
														GVNWAAFHPTMPLIVSGADDR	4	2	1	N-Term(iTRAQ8plex)	0.853	1.145	1.472	5.35	50	0	2558.30747	3	853.4406738	-2.09633349	46.09246
														LLHDQVGVIQFGPYKQLFLQTYAR	2	2	1	N-Term(iTRAQ8plex); K15(iTRAQ8plex)	-	-	-	5.01	28	1	3442.94179	4	861.4909058	-1.225153772	53.04374667
														MCTLIDKFDEHDGPVR	6	2	1	N-Term(iTRAQ8plex); C2(Methylthio); K7(iTRAQ8plex)	1.227	1.504	1.739	4.83	24	1	2530.25991	4	633.3204346	-4.510959017	47.404925
														VTTVTEIGKDVIGLR	2	2	1	N-Term(iTRAQ8plex); K9(iTRAQ8plex)	-	1.103	1.721	4.52	30	1	2209.32363	4	553.0863647	-6.346827346	47.19948833
														YAVTTGDHGIIR	2	2	1	N-Term(iTRAQ8plex)	0.442	0.481	0.756	3.99	40	0	1606.87845	3	536.2976685	-4.374430995	20.47722333
														GFFEGTIASK	1	2	1	N-Term(iTRAQ8plex); K10(iTRAQ8plex)	1.501	2.424	1.861	3.67	-	0	1664.93149	3	555.6486816	-9.201356453	39.74639167
														NLSPGAVESDVR	4	2	1	N-Term(iTRAQ8plex)	0.702	1.176	1.283	3.4	31	0	1547.82219	2	774.4147339	-7.068090007	18.33662
														SSGLTAVWVAR	2	2	1	N-Term(iTRAQ8plex)	0.922	1.311	1.321	3.35	24	0	1450.82353	2	725.9154053	-5.836252947	24.10475833
														LLELGPKPEVAQQTR	1	2	1	N-Term(iTRAQ8plex); K7(iTRAQ8plex)	0.893	1.415	1.445	3.21	-	0	2287.34983	3	763.12146	-4.20778102	28.07988833
														DADSITLFDVQQK	1	2	1	N-Term(iTRAQ8plex); K13(iTRAQ8plex)	1.418	1.644	1.343	3.11	-	0	2088.12503	3	696.7131958	-8.772055005	48.56072333
														TLDLPIYVTR	1	2	1	N-Term(iTRAQ8plex)	0.946	1.429	1.58	2.86	-	0	1494.89177	2	747.9495239	5.632469002	38.98618167
148539848	DSC3	desmocollin-3 isoform Dsc3b preproprotein	NP_077741.2	2.62	2	2	2	839	93.4	6.32	0.367	0.357	0.238																
														GVDKEPLNLFYIER	1	2	1	N-Term(iTRAQ8plex); K4(iTRAQ8plex)	0.367	0.357	0.238	2.8	-	1	2301.3032	3	767.772583	-1.36684578	39.59053167
														ALVTVHVR	1	2	1	N-Term(iTRAQ8plex)	-	-	-	-	44	0	1198.75907	3	400.2578735	1.423184621	15.74599667
148539872	ACAT2	acetyl-CoA acetyltransferase, cytosolic isoform 1	NP_005882.2	9.57	2	2	5	397	41.3	6.92	-	2.071	2.327																

														ILVTLLHTLER	3	2	1	N-Term(iTRAQ8plex)	-	2.071	2.327	3.42	37	0	1612.01603	3	538.0101929	3.76178846	66.30987333
														ATVAPEDVSEVIFGHVLAAGCGQNPVR	2	2	1	N-Term(iTRAQ8plex); C21(Methylthio)	-	-	-	2.96	45	0	3086.55722	3	1029.523926	-4.33434577	51.80752167
148596949	NOLC1	nucleolar and coiled-body phosphoprotein 1 isoform 2	NP_004732.2	5.15	4	4	10	699	73.6	9.47	1.476	1.145	0.901																
														EEEIEVDSR	2	4	1	N-Term(iTRAQ8plex)	1.373	1.145	0.612	3.61	24	0	1409.70793	2	705.357605	1.235745999	21.54904167
														VVPSDLYPLVLGFLRDNQLSEVANK	1	4	1	N-Term(iTRAQ8plex); K25(iTRAQ8plex)	-	-	-	3.17	-	1	3394.91762	4	849.4848633	-0.567923942	61.34796667
														VVPSDLYPLVLGFLR	3	4	1	N-Term(iTRAQ8plex)	1.771	1.306	0.881	2.89	40	0	1992.18564	3	664.7333984	1.029403313	68.55970167
														VREEEIEVDSR	4	4	1	N-Term(iTRAQ8plex)	1.446	0.999	0.945	-	28	1	1664.86797	2	832.9376221	-4.66330035	21.26153833
148664186	PKP2	plakophilin-2 isoform 2a	NP_001005242.2	10.04	6	6	19	837	92.7	9.29	1.041	1.212	0.439																
														TSSVPEYVYNLHLVENDFVGGR	6	2	1	N-Term(iTRAQ8plex)	1.164	1.168	0.444	6.17	62	0	2799.42331	3	933.8126221	-0.806715816	50.844035
														ISAAATFIQHECFQK	6	2	1	N-Term(iTRAQ8plex); C12(Methylthio); K15(iTRAQ8plex)	1.041	1.212	0.165	5.04	36	0	2348.23794	3	783.4152222	-1.724296246	62.91636833
														AVSMLEADHMLPSR	2	2	1	N-Term(iTRAQ8plex)	1.542	1.367	0.871	4.76	51	0	1860.95688	3	620.9904785	-2.433695895	35.984385
														EAGPSVAVDSSGR	3	2	1	N-Term(iTRAQ8plex)	0.947	1.083	0.244	3.13	41	0	1535.79692	2	768.4020996	0.11153737	15.47358333
														GVEWLWHSIVIR	1	2	1	N-Term(iTRAQ8plex)	1.209	0.935	0.607	2.91	-	0	1799.03739	3	600.350647	5.762174839	55.347415
														AALLVPPR	1	2	1	N-Term(iTRAQ8plex)	0.884	1.278	0.669	-	31	0	1140.73979	2	570.8735352	-0.760565648	29.09383
148727247	USP5	ubiquitin carboxyl-terminal hydrolase 5 isoform 2	NP_003472.2	10.42	6	7	17	835	93.2	5.08	0.952	2.029	2.398																
														TMTELEIDMNQR	2	2	1	N-Term(iTRAQ8plex)	0.929	2.21	2.977	5.26	52	0	1784.88884	2	892.9480591	3.563383845	32.31865167
														ENLWLNLTDGSILCGR	4	2	1	N-Term(iTRAQ8plex); C14(Methylthio)	1.065	5.168	6.206	5.11	89	0	2154.11894	2	1077.56311	9.276081611	60.21250667
														FASFPDYLVIQIK	4	2	1	N-Term(iTRAQ8plex); K13(iTRAQ8plex)	0.909	1.284	1.297	3.75	40	0	2149.24289	3	717.0858154	-4.130878351	48.01245167
														DGLGGLPDIVR	2	2	1	N-Term(iTRAQ8plex)	0.976	1.637	1.932	3.42	33	0	1415.81536	2	708.4113159	-0.477522765	33.05587833
														IVILPDYLEIAR	2	2	1	N-Term(iTRAQ8plex)	0.803	2.516	3.094	3.26	40	0	1719.03032	2	860.0187988	-3.217083307	54.096945
														QQDAQEFFLHLINMVER	2	2	1	N-Term(iTRAQ8plex)	-	-	-	2.69	36	0	2422.2515	3	808.0886841	0.973349008	67.73681
														VYLHLR	1	6	2	N-Term(iTRAQ8plex)	-	-	-	-	23	0	1104.68808	3	368.9008789	4.500970749	20.21731
148727341	STRAP	serine-threonine kinase receptor-associated protein	NP_009109.3	15.43	5	5	17	350	38.4	5.12	0.964	1.863	2.054																
														QGDTGDWIGTFLGHK	2	1	1	N-Term(iTRAQ8plex); K15(iTRAQ8plex)	0.792	1.73	2.054	5.13	41	0	2240.18442	3	747.3996582	-3.401479161	39.58716833
														GHFGPIHCVR	11	2	1	N-Term(iTRAQ8plex); C8(Methylthio)	0.964	2.058	2.118	4.83	50	0	1472.76247	4	368.9460754	4.616886934	20.82201833
														YDYNSGEELESYK	2	2	1	N-Term(iTRAQ8plex); K13(iTRAQ8plex)	1.383	4.017	3.058	4.67	67	0	2205.06571	3	735.6934204	-6.831233861	35.978415
														LWQTVVGK	1	2	1	N-Term(iTRAQ8plex); K8(iTRAQ8plex)	1.139	1.863	1.855	3.28	-	0	1538.93901	2	769.9731445	-8.108740762	31.66627167
														ALWCSEDK	1	2	1	N-Term(iTRAQ8plex); C4(Methylthio); K8(iTRAQ8plex)	0.746	1.091	1.155	-	30	0	1605.80692	3	535.9404907	-9.723075575	26.03003167
148833484	PCBP2	poly(rC)-binding protein 2 isoform c	NP_001092090.1	22.05	3	5	16	331	34.9	8.00	0.989	2.527	2.647																
														GYWAGLDASAQTTSHELTIPNDLIGCIIGR	3	4	1	N-Term(iTRAQ8plex); C26(Methylthio)	-	-	-	4.72	46	0	3522.74961	3	1174.935791	7.501859527	63.31053
														IITLAGPTNAIFK	3	7	1	N-Term(iTRAQ8plex); K13(iTRAQ8plex)	0.967	2.68	2.693	4.6	38	0	1967.20476	2	984.1060181	-5.206263933	36.70086833
														IANPVEGSTDR	1	7	1	N-Term(iTRAQ8plex)	0.989	2.358	2.599	-	28	0	1462.77519	2	731.8912354	-3.532475049	18.09412333
														INISEGNCPER	7	10	2	N-Term(iTRAQ8plex); C8(Methylthio)	-	-	-	4	39	0	1581.76238	2	791.3848267	-2.734670992	25.71615
														QMSGAQIK	2	10	2	N-Term(iTRAQ8plex); M2(Oxidation); K8(iTRAQ8plex)	-	-	-	3.29	30	0	1486.83918	3	496.2845764	-7.81543972	11.07629333
149193319	GRSF1	G-rich sequence factor 1 isoform 2	NP_001091947.1	10.38	2	2	7	318	36.6	5.69	1.263	1.048	0.542																
														GLPFQANAQDIINFFAPLKPVR	6	3	1	N-Term(iTRAQ8plex); K19(iTRAQ8plex)	1.214	1.159	0.512	6.48	40	0	3064.76072	3	1022.258423	1.629492529	64.35269167
														YIELFLNSCPK	1	3	1	N-Term(iTRAQ8plex); C9(Methylthio); K11(iTRAQ8plex)	1.314	0.948	0.575	3.8	-	0	1981.06272	3	661.0257568	-6.059900115	82.04759833
149944524	C17orf102	uncharacterized protein C17orf102	NP_997337.2	11.38	1	1	2	167	17.8	10.61	1.047	2.002	2.742																
														AGWVRGAAVGNLFILLLGK	2	1	1	N-Term(iTRAQ8plex); K19(iTRAQ8plex)	1.047	2.002	2.742	3	-	1	2563.59121	4	641.6532593	8.369481575	57.32336667
149999378	INF2	inverted formin-2 isoform 2	NP_001026884.3	2.66	3	3	8	1240	134.5	5.53	0.968	0.994	1.338																
														SVQANLDQSQR	2	4	1	N-Term(iTRAQ8plex)	1.32	1.241	1.07	4.4	33	0	1549.8248	3	517.2797852	0.768421533	18.114
														ALDELFEAIEQK	2	4	1	N-Term(iTRAQ8plex); K12(iTRAQ8plex)	1.035	0.681	1.717	4.19	38	0	2014.11472	3	672.0430908	-8.446435002	80.49494833
														SHPDLLQLPR	4	4	1	N-Term(iTRAQ8plex)	0.826	0.994	1.255	3.47	32	0	1479.85242	3	493.955658	-4.136002321	25.20314667
149999611	SRP14	signal recognition particle 14 kDa protein isoform 1	NP_003125.3	27.94	4	4	12	136	14.6	10.04	1.375	2.663	1.696																
														TSGSVYITLK	2	1	1	N-Term(iTRAQ8plex); K10(iTRAQ8plex)	1.245	2.386	1.528	4.18	50	0	1676.99175	2	838.9995117	-7.486311441	34.73472333
														FQMAYSNLLR	4	2	1	N-Term(iTRAQ8plex)	1.193	1.946	1.417	3.8	33	0	1546.83196	2	773.9196167	-2.200964072	45.83217167
														EVNKFQMAYSNLLR	1	2	1	N-Term(iTRAQ8plex); K4(iTRAQ8plex)	1.39	3.832	2.468	2.78	-	1	2321.27817	3	774.4309082	-4.94121763	41.38595333
														VLLESEQFLTELTR	5	1	1	N-Term(iTRAQ8plex)	1.8	2.711	1.81	-	72	0	1982.11931	2	991.5632935	4.086027527	68.14406
150010589	IFITM1	interferon-induced transmembrane protein 1	NP_003632.3	13.60	2	2	6	125	13.9	7.93	0.685	0.442	0.556																
														MVGDVTGAQAYASTAK	2	3	1	N-Term(iTRAQ8plex); K16(iTRAQ8plex)	0.685	0.249	0.477	4.69	46	0	2178.17129	2	1097.581543	-3.480601727	18.43327167
														MVGDVTGAQAYASTAK	3	3	1	N-Term(iTRAQ8plex); M1(Oxidation); K16(iTRAQ8plex)	0.678	0.442	0.427	3.68	81	0	2194.15581	2	1097.581543	-3.480601727	18.43327167
														KMVGDVTGAQAYASTAK	1	3	1	N-Term(iTRAQ8plex); K1(iTRAQ8plex); K17(iTRAQ8plex)	-	0.959	0.647	2.75	-	1	2610.46621	4	653.3720093	-1.01670369	23.65164167
150010661	SEC14L5	SEC14-like protein 5	NP_055507.1	1.15	1	1	1	696	78.9	6.54	0.886	0.555	0.430																
														LLIVRAPR	1	1	1	N-Term(iTRAQ8plex)	0.886	0.555	0.43	-	20	1	1241.8274	3	414.6139832	-6.897163918	57.05199667
151101292	TMX1	thioredoxin-related transmembrane protein 1 precursor	NP_110382.3	8.21	2	2	5	280	31.8	4.98	1.315	1.048	0.859																
														FIITALPTIYHCK	4	1	1	N-Term(iTRAQ8plex); C12(Methylthio); K13(iTRAQ8plex)	1.255	0.736	0.838	5.79	48	0	2174.2269	3	725.4138184	-2.631037207	51.43156333
														LLSESAQPLK	1	1	1	N-Term(iTRAQ8plex); K10(iTRAQ8plex)	1.636	1.176	0.978	-	26	0	1694.0186	2	847.5129395	-7.2425496	21.88194667
151101404	PTMA	prothymosin alpha isoform 2	NP_002814.3	21.82	2	2	4	110	12.1	3.79	0.984	2.780	3.168																
														AAEDDEDDDVDTKK	2	7	1	N-Term(iTRAQ8plex); K13(iTRAQ8plex); K14(iTRAQ8plex)	-	-	-	3.47	30	1	2478.24039	3	826.7516479	-8.357019126	13.40529167
														EVVEEAENGR	2	8	1	N-Term(iTRAQ8plex)	0.984	2.78	3.168	2.82	44	0	1435.72527	2	718.366272	-5.442527805	19.41350833
151101459	PPP6R1	serine/threonine-protein phosphatase 6 regulatory subunit 1	NP_055746.3	2.16	1	1	2	881	96.7	4.55	0.760	1.040	0.888																
														EDLSLPELLDEEDVLQECK	2	1	1	N-Term(iTRAQ8plex); C18(Methylthio); K19(iTRAQ8plex)	0.76	1.04	0.888	3.91	37	0	2871.46268	3	957.8257446	5.655074965	56.291845
151108473	FIS1	mitochondrial fission 1 protein	NP_057152.2	15.13	2	2	4	152	16.9	8.79	0.688	0.656	0.713																
														STQFEYAWCLVR	2	1	1	N-Term(iTRAQ8plex); C9(Methylthio)	0.677	0.685	0.74	4.32	44	0	1852.90605	2	926.956665	1.760023461	47.870395
														GIVLLEELLPK	2	1	1	N-Term(iTRAQ8plex); K11(iTRAQ8plex)	0.7	0.629	0.688	4.02	44	0	1832.16227	3	611.3922729	-5.171868519	54.75815333
153252132	RPL31	60S ribosomal protein L31 isoform 3	NP_001093163.1	39.67	5	5	21	121	14.0	10.80	1.628	2.104	1.774																
														LYTLVTYVPVTTFK	12	3	1	N-Term(iTRAQ8plex); K14(iTRAQ8plex)	2.516	2.582	1.887	6.03	61	0	2253.35185	2	1127.179565	7.266037634	55.70602667
														EYTINIHK	4	3	1	N-Term(iTRAQ8plex); K8(iTRAQ8plex)	0.976	1.138	1.27	3.34	33	0	1625.94121	2	813.4742432	-3.629646533	17.56084667
														NEDEDSPNKLYTLVTYVPVTTFK	1	3	1	N-Term(iTRAQ8plex); K9(iTRAQ8plex); K23(iTRAQ8plex)	-	0.788	1.548	3.11	-	1	3585.93203	4	897.2384644	-3.776914348	50.351775
														SAINEVVTR	3	3	1	N-Term(iTRAQ8plex)	1.414	1.793	1.834	2.97	33	0	1292.74211	2	646.8741455	-5.087565337	20.001545
														EMGTPDVR	1	3	1	N-Term(iTRAQ8plex)	1.428	1.885	1.753	-	27	0	1208.61992	2	604.8135986	-3.96373938	15.72022667
153266822	ALDH1A3	aldehyde dehydrogenase family 1 member A3 isoform 1	NP_000684.2	24.80	10	12	60	512	56.1	7.25	0.480	4.349	0.611																
														ELGEYALAEYTEVK	2	2	1	N-Term(iTRAQ8plex); K14(iTRAQ8plex)	0.437	3.976	0.637	6.03	41	0	2223.21384	3	741.7427979	5.990878811	39.64241
														GLFIKPTVFSEVTDNMR	2	2	1	N-Term(iTRAQ8plex); K5(iTRAQ8plex)	-	-	-	4.87	34	0	2562.42544	4	641.3618164	1.699345058	46.18723833
														LECGGSAMEDK	4	2	1	N-Term(iTRAQ8plex); C3(Methylthio); K11(iTRAQ8plex)	0.487	3.01	0.489	4.35	40	0	1793.8571	2	897.4321899	-6.772578019	34.45213667
														LLHQLADLVER	27	2	1	N-Term(iTRAQ8plex)	-	4.747	0.387	4.11	49	0	1610.95768	3	537.6569824	1.994290868	53.87848667
														IAFTGSTEVGK	4	5	1	N-Term(iTRAQ8plex); K11(iTRAQ8plex)	0.293	4.854	0.98	4.06	46	0	1717.98418	2	859.4957275	-6.002873603	33.10729833
														ILELIESGK	2	2	1	N-Term(iTRAQ8plex); K9(iTRAQ8plex)	0.481	3.477	0.522	3.99	35	0	1609.98992	2	805.4985962	-5.338876414	72.84431667
														VFVEEQVYSEFVR	4	2	1	N-Term(iTRAQ8plex)	0.591	3.57	1.19	3.66	45	0	1935.01384	2	968.0105591	-1.410716492	53.61483333
														ILELIESGKK	4	2	1	N-Term(iTRAQ8plex); K9(iTRAQ8plex); K10(iTRAQ8plex)	-	8.774	0.351	3.59	23	1	2042.30141	4	511.3337708	7.05661964	50.28924
														IFINNEWHESK	1	2	1	N-Term(iTRAQ8plex); K11(iTRAQ8plex)	0.5	4.378	1.047	3.56	-	0	2025.09171	3	675.7020874	-4.791063499	28.343335
														TIPTDDNVVCFTR	2	1	1	N-Term(iTRAQ8plex); C10(Methylthio)	0.388	3.599	0.965	3.21	21	0	1830.89922	2	915.9532471	-2.176405635	35.860525
														LECGGSAMEDK	1	2	1	N-Term(iTRAQ8plex); C3(Methylthio); M8(Oxidation); K11(iTRAQ8plex)	0.791	1.527	0.329	-	20	0	1809.86423	2	897.4321899	-6.772578019	34.45213667
														YFAGWADK	2	2	2	N-Term(iTRAQ8plex); K8(iTRAQ8plex)	-	-	-	3.34	25	0	1565.85759	2	783.4324341	0.217289276	29.965195
														VTLELGGK	5	10	4	N-Term(iTRAQ8plex); K8(iTRAQ8plex)	-	-	-	3.03	32	0	1424.8792	2	712.9432373	-9.904917305	26.02754667
153285461	FGF2	fibroblast growth factor 2	NP_001997.5	3.82	1	1	2	288	30.8	11.18	0.957	0.498	3.726																
														CVTDECFFFER	2	1	1	N-Term(iTRAQ8plex); C1(Methylthio); C6(Methylthio)	0.957	0.498	3.726	3.72	49	0	1791.76006	2	896.383667	4.690396094	52.59248167
153791158	KRT75	keratin, type II cytoskeletal 75	NP_004684.2	18.69	1	14	440	551	59.5	7.74	0.284	0.184	0.157																
														LVDLEEALQK	9	1	1	N-Term(iTRAQ8plex); K10(iTRAQ8plex)	0.284	0.184	0.157	4.12	42	0	1766.04424	2	883.5257568	-4.402068738	75.26014333
														LALDVEIATYR	117	10	7	N-Term(iTRAQ8plex)	-	-	-	4.83	67	0	1567.90409	3	523.3062134	2.771589637	39.12087
														LALDVEIATYR	8	10	7	N-Term(iTRAQ8plex); Y10(iTRAQ8plex)	-	-	-	-	60	0	1872.106	3	523.3062134	2.771589637	39.12087
														DVDAAYMNKVELEAK	10	2	2	N-Term(iTRAQ8plex); K9(iTRAQ8plex); K15(iTRAQ8plex)	-	-	-	6.03	48	1	2608.43657	2	1304.721924	-2.074140429	31.19943833
														DVDAAYMNKVELEAK	3	2	2	N-Term(iTRAQ8plex); M7(Oxidation); K9(iTRAQ8plex); K15(iTRAQ8plex)	-	-	-	5.55	-	1	2624.43545	2	1304.721924	-2.074140429	31.19943833
														FLEQQNK	13	31	8	N-Term(iTRAQ8plex); K7(iTRAQ8plex)	-	-	-	-	33	0	1514.87885	3	505.631134	0.090799082	15.97476833
														VEIEAK	5	1	3	N-Term(iTRAQ8plex); K6(iTRAQ8plex)	-	-	-	2.98	27	0	1296.79612	3	432.9381104	1.107730516	13.75300333
														GELAIK	4	2	6	N-Term(iTRAQ8plex); K6(iTRAQ8plex)	-	-	-	-	28	0	1238.78995	3	413.6025085	0.089671776	17.83946167
														AQYEDIANR	8	3	2	N-Term(iTRAQ8plex)	-	-	-	2.94	31	0	1383.72576	2	692.3607788	-1.973169497	18.09094667
														FASFIDKVR	16	27	8	N-Term(iTRAQ8plex); K7(iTRAQ8plex)	-	-	-	3.33	31	1	1691.01076	4	423.5080261	0.114317571	39.05999667
														FASFIDK	57	28	8	N-Term(iTRAQ8plex); K7(iTRAQ8plex)	-	-	-	3.23	35	0	1435.83818	2	718.4240723	0.227470368	33.40336167
														NLDLDSIIAEVK	64	8	5	N-Term(iTRAQ8plex); K12(iTRAQ8plex)	-	-	-	5.73	89	0	1938.13909	2	969.5731812	1.173488892	50.31108
														KLLEGEECR	13	13	6	N-Term(iTRAQ8plex); K1(iTRAQ8plex); C8(Methylthio)	-	-	-	2.79	31	1	1730.93911	3	577.6514282	0.43855829	29.74912
														LLEGEECR	16	16	6	N-Term(iTRAQ8plex); C7(Methylthio)	-	-	-	2.75	36	0	1298.63898	3	433.551178	0.260569375	30.10390833
														AEAESWYQTK	19	4	3	N-Term(iTRAQ8plex); K10(iTRAQ8plex)	-	-	-	4.56	66	0	1820.96013	2	910.9831543	-2.67404492	21.62443167
														YEELQVTAGR	43	3	3	N-Term(iTRAQ8plex)	-	-	-	4.27	46	0	1469.78899	2	735.4003296	2.178033814	27.29477167
														YEELQVTAGR	3	3	3	N-Term(iTRAQ8plex); Y1(iTRAQ8plex)	-	-	-	-	32	0	1773.99126	2	735.4003296	2.178033814	27.29477167
														DVDAAYMNK	25	11	4	N-Term(iTRAQ8plex); K9(iTRAQ8plex)	-	-	-	4.2	50	0	1634.86492	2	817.9366455	-0.50579336	22.28227833
														DVDAAYMNK	13	11	4	N-Term(iTRAQ8plex); M7(Oxidation); K9(iTRAQ8plex)	-	-	-	3.52	47	0	1650.86186	2	817.9366455	-0.50579336	22.28227833
														KDVDAAYMNK	3	11	4	N-Term(iTRAQ8plex); K1(iTRAQ8plex); K10(iTRAQ8plex)	-	-	-	3	31	1	2067.16056	3	689.7250366	-3.199890157	21.25677833
153791670	KRT76	keratin, type II cytoskeletal 2 oral	NP_056932.2	14.58	2	11	262	638	65.8	8.12	0.397	0.271	0.255																
														QLDSLLGER	1	1	1	N-Term(iTRAQ8plex)	0.397	0.271	0.255	2.89	-	0	1334.75774	2	667.8825073	-0.317114897	28.55038
														TLYEMELSQMQSHASDTSVVLSMDNNR	3	1	1	N-Term(iTRAQ8plex); M5(Oxidation)	-	-	-	-	27	0	3406.60805	4	852.4064331	5.030300665	53.52389167
														LALDVEIATYR	117	10	7	N-Term(iTRAQ8plex)	-	-	-	4.83	67	0	1567.90409	3	523.3062134	2.771589637	39.12087
														LALDVEIATYR	8	10	7	N-Term(iTRAQ8plex); Y10(iTRAQ8plex)	-	-	-	-	60	0	1872.106	3	523.3062134	2.771589637	39.12087
														FLEQQNK	13	31	8	N-Term(iTRAQ8plex); K7(iTRAQ8plex)	-	-	-	-	33	0	1514.87885	3	505.631134	0.090799082	15.97476833
														GELAIK	4	2	6	N-Term(iTRAQ8plex); K6(iTRAQ8plex)	-	-	-	-	28	0	1238.78995	3	413.6025085	0.089671776	17.83946167

														FASFIDKVR	16	27	8	N-Term(iTRAQ8plex); K7(iTRAQ8plex)	-	-	-	3.33	31	1	1691.01076	4	423.5080261	0.114317571	39.05999667
														FASFIDK	57	28	8	N-Term(iTRAQ8plex); K7(iTRAQ8plex)	-	-	-	3.23	35	0	1435.83818	2	718.4240723	0.227470368	33.40336167
														KLLEGEECR	13	13	6	N-Term(iTRAQ8plex); K1(iTRAQ8plex); C8(Methylthio)	-	-	-	2.79	31	1	1730.93911	3	577.6514282	0.43855829	29.74912
														LLEGEECR	16	16	6	N-Term(iTRAQ8plex); C7(Methylthio)	-	-	-	2.75	36	0	1298.63898	3	433.551178	0.260569375	30.10390833
														GEMALK	1	4	3	N-Term(iTRAQ8plex); M3(Oxidation); K6(iTRAQ8plex)	-	-	-	-	30	0	1272.74594	3	424.920166	1.365941791	12.0309
														VELQAK	1	16	4	N-Term(iTRAQ8plex); K6(iTRAQ8plex)	-	-	-	2.75	-	0	1295.81623	3	432.61026	1.470485751	22.94674667
														AQYEEIAQR	16	7	4	N-Term(iTRAQ8plex)	-	-	-	3.65	42	0	1411.75541	3	471.2566528	5.024911587	22.42886333
153945728	MAP1B	microtubule-associated protein 1B isoform 1	NP_005900.2	5.06	8	8	21	2468	270.5	4.81	0.778	0.639	2.679																
														NLISPDLGVVFLNVPENLKNPEPNIK	2	2	1	N-Term(iTRAQ8plex); K19(iTRAQ8plex); K26(iTRAQ8plex)	-	-	-	5.86	37	1	3786.19448	4	947.3040771	0.30041081	54.01584333
														SVGNTIDPVILFQK	3	2	1	N-Term(iTRAQ8plex); K14(iTRAQ8plex)	0.84	0.708	2.02	5.09	49	0	2139.2598	3	713.7581177	-1.688211341	40.386295
														SWDTNLIECNLDQELK	2	1	1	N-Term(iTRAQ8plex); C9(Methylthio); K16(iTRAQ8plex)	0.772	2.41	7.596	4.82	72	0	2575.28574	3	859.1000977	-5.26255438	41.42562
														HNLQDFINIK	3	2	1	N-Term(iTRAQ8plex); K10(iTRAQ8plex)	1.969	1.104	3.279	4.65	42	0	1850.05803	2	925.5326538	-8.880966184	29.681725
														ESSPLYSPTFSDSTSAVK	2	2	1	N-Term(iTRAQ8plex); K18(iTRAQ8plex)	0.734	0.966	1.453	4.49	29	0	2511.30191	3	837.7721558	-2.22882992	30.06472833
														ASLTLFCPEEGDWK	2	2	1	N-Term(iTRAQ8plex); C7(Methylthio); K14(iTRAQ8plex)	0.986	0.639	5.479	4.09	32	0	2250.12339	3	750.7126465	-7.180093756	41.33381333
														HMDPPPAPVQDR	4	2	1	N-Term(iTRAQ8plex); M2(Oxidation)	0.706	1.004	4.094	3.59	31	0	1679.8414	3	560.6191406	-2.903649556	10.16715833
														HMDPPPAPVQDR	2	2	1	N-Term(iTRAQ8plex)	0.409	0.441	0.944	3.56	23	0	1663.84623	3	560.6191406	-2.903649556	10.16715833
														STDFAPIKEDFGQEK	1	2	1	N-Term(iTRAQ8plex); K8(iTRAQ8plex); K15(iTRAQ8plex)	1.699	0.345	1.907	3.54	-	1	2624.41738	4	656.8598022	-6.150773026	28.54183667
154146191	HSP90AA1	heat shock protein HSP 90-alpha isoform 2	NP_005339.3	51.91	25	37	526	732	84.6	5.02	1.065	3.127	3.724																
														HGLEVIYMIEPIDEYCVQQLK	38	3	1	N-Term(iTRAQ8plex); C16(Methylthio); K21(iTRAQ8plex)	-	-	-	7.37	59	0	3174.64194	3	1058.895996	7.434695799	64.361635
														HLEINPDHSIIETLR	27	3	1	N-Term(iTRAQ8plex)	1.168	2.996	3.954	5.9	70	0	2091.14189	2	1046.074585	-3.901216914	28.61405333
														KHGLEVIYMIEPIDEYCVQQLK	14	3	1	N-Term(iTRAQ8plex); K1(iTRAQ8plex); C17(Methylthio); K22(iTRAQ8plex)	-	-	-	5.69	38	1	3606.94516	3	1202.986572	-1.385868899	59.74453667
														DQVANSAFVER	11	3	1	N-Term(iTRAQ8plex)	1.176	3.147	3.776	5.47	94	0	1539.81267	3	513.9416504	2.264318958	24.09792
														HGLEVIYMIEPIDEYCVQQLK	10	3	1	N-Term(iTRAQ8plex); M8(Oxidation); C16(Methylthio); K21(iTRAQ8plex)	-	3.917	4.151	5.38	49	0	3190.64426	3	1058.895996	7.434695799	64.361635
														HSQFIGYPITLFVEK	33	3	1	N-Term(iTRAQ8plex); K15(iTRAQ8plex)	1.27	4.32	3.636	5.29	67	0	2387.35356	2	1194.18042	-2.009788715	48.38265333
														ELISNSSDALDKIR	11	3	1	N-Term(iTRAQ8plex); K12(iTRAQ8plex)	1.54	4.777	5.396	5.24	55	1	2169.22441	2	1085.115845	-4.222000647	29.22255333
														KHGLEVIYMIEPIDEYCVQQLK	1	3	1	N-Term(iTRAQ8plex); K1(iTRAQ8plex); M9(Oxidation); C17(Methylthio); [illegible]	-	-	-	4.97	-	1	3622.93374	3	1202.986572	-1.385868899	59.74453667
														DLVILLYETALLSSGFSLEDPQTHANR	21	3	1	N-Term(iTRAQ8plex)	-	-	-	4.94	61	0	3306.75327	3	1102.922607	0.256358898	70.17788833
														NPDDITNEEYGEFYK	30	3	1	N-Term(iTRAQ8plex); K15(iTRAQ8plex)	1.035	5.419	6.821	4.9	73	0	2442.17484	2	1221.591675	-6.579505386	28.96848333
														KHLEINPDHSIIETLR	11	3	1	N-Term(iTRAQ8plex); K1(iTRAQ8plex)	1.572	3.265	3.924	4.66	76	1	2523.43741	2	1262.222168	-5.279300488	28.10402
														HIYYITGETK	21	3	1	N-Term(iTRAQ8plex); K10(iTRAQ8plex)	0.796	2.246	2.393	4.59	50	0	1833.0258	3	611.6784668	-8.621920149	18.97976667
														ELHINLIPNKQDR	6	3	1	N-Term(iTRAQ8plex); K10(iTRAQ8plex)	0.793	2.203	2.447	4.56	46	1	2198.27129	2	1099.639282	-6.966140603	24.60357
														LVTSPCCIVTSTYGWTANMER	7	3	1	N-Term(iTRAQ8plex); C6(Methylthio); C7(Methylthio)	0.44	6.799	6.39	4.53	71	0	2728.26432	3	910.0929565	2.339611624	64.53971
														ELHINLIPNK	10	3	1	N-Term(iTRAQ8plex); K10(iTRAQ8plex)	0.906	1.555	2.162	4.5	36	0	1799.08207	2	900.0460815	-8.37360937	27.04742667
														YYTSASGDEMVSLK	6	3	1	N-Term(iTRAQ8plex); K14(iTRAQ8plex)	1.463	2.023	3.343	4.41	47	0	2159.09635	3	720.3703003	-8.671994242	34.81426167
														ELISNSSDALDK	2	3	1	N-Term(iTRAQ8plex); K12(iTRAQ8plex)	-	-	-	4.21	68	0	1900.03012	3	634.0148926	-9.608697983	33.88961667
														RAPFDLFENR	22	3	1	N-Term(iTRAQ8plex)	0.871	1.965	2.522	3.9	47	1	1568.83847	3	523.6176758	-6.531715291	37.706835
														HSQFIGYPITLFVEKER	1	3	1	N-Term(iTRAQ8plex); K15(iTRAQ8plex)	1.467	3.127	2.629	3.67	-	1	2672.48379	4	668.8764038	-6.846045512	42.82841167
														APFDLFENR	21	3	1	N-Term(iTRAQ8plex)	1.092	3.42	4.095	3.39	62	0	1412.74224	3	471.5849609	-5.147256964	40.92483833
														YYTSASGDEMVSLKDYCTR	3	3	1	N-Term(iTRAQ8plex); K14(iTRAQ8plex); C17(Methylthio)	1.247	4.105	4.271	3.38	21	1	2843.33713	3	948.4505615	-4.896000384	40.85315833
														LGIHEDSQNR	7	3	1	N-Term(iTRAQ8plex)	1.053	1.465	1.608	3.22	39	0	1472.77201	2	736.8887329	-3.898842962	13.705585
														LVTSPCCIVTSTYGWTANMER	2	3	1	N-Term(iTRAQ8plex); C6(Methylthio); C7(Methylthio); M19(Oxidation)	0.769	-	0.276	3.02	52	0	2744.26242	3	910.0929565	2.339611624	64.53971
														FYEQFSK	6	3	1	N-Term(iTRAQ8plex); K7(iTRAQ8plex)	1.125	3.221	3.202	2.98	29	0	1556.8414	3	519.619873	-7.602175473	24.68346
														IMKDILEK	1	3	1	N-Term(iTRAQ8plex); K3(iTRAQ8plex); K8(iTRAQ8plex)	3.149	7.174	9.835	2.81	-	1	1902.16921	4	476.29776	-8.890450359	41.11091667
														DNSTMGYMAAK	1	3	1	N-Term(iTRAQ8plex); M8(Oxidation); K11(iTRAQ8plex)	1.614	3.166	4.291	-	57	0	1812.90166	2	906.9544678	-3.534319191	12.86375167
														AILFVPR	16	1	1	N-Term(iTRAQ8plex)	1.278	4.724	5.906	-	43	0	1119.71216	3	373.9083252	-7.831457034	27.73520833
														VFIMDNCEELIPEYLNFIR	1	3	1	N-Term(iTRAQ8plex); C7(Methylthio); Y14(iTRAQ8plex)	-	-	-	-	32	0	3012.5709	3	1004.861816	7.134659894	68.43389
														HGLEVIYMIEPIDEYCVQQLK	2	3	1	N-Term(iTRAQ8plex); Y7(iTRAQ8plex); C16(Methylthio); K21(iTRAQ8plex)	-	-	-	-	31	0	3478.84348	3	1058.895996	7.434695799	64.361635
														TDTGEPMGR	1	3	1	N-Term(iTRAQ8plex)	0.96	4.651	5.901	-	18	0	1267.62102	2	634.3141479	-3.488441336	13.32315833
														ISEILR	1	1	3	N-Term(iTRAQ8plex)	-	-	-	-	29	0	1034.64141	2	517.8243408	-9.429623952	36.16632333
														TLTIVDTGIGMTK	24	3	2	N-Term(iTRAQ8plex); K13(iTRAQ8plex)	-	-	-	6.24	73	0	1958.14018	2	979.5737305	-2.597864311	34.38798333
														ADLINNLGTIAK	30	5	2	N-Term(iTRAQ8plex); K12(iTRAQ8plex)	-	-	-	5.52	92	0	1851.09868	2	926.0541382	-8.109526608	30.785565
														HNDDEQYAWESSAGGSFTVR	24	6	2	N-Term(iTRAQ8plex)	-	-	-	5.2	48	0	2560.16581	3	854.0576172	-2.333053977	33.96651167
														SLTNDWEDHLAVK	29	6	2	N-Term(iTRAQ8plex); K13(iTRAQ8plex)	-	-	-	5.15	76	0	2136.14507	2	1068.573975	-6.511135446	31.08030833
														TLTIVDTGIGMTK	11	3	2	N-Term(iTRAQ8plex); M11(Oxidation); K13(iTRAQ8plex)	-	-	-	4.88	57	0	1974.12615	2	979.5737305	-2.597864311	34.38798333
														YIDQEELNK	9	6	2	N-Term(iTRAQ8plex); K9(iTRAQ8plex)	-	-	-	4.4	45	0	1759.96037	2	880.4838257	-4.702692703	18.77699667
														VILHLKEDQTEYLEER	20	6	2	N-Term(iTRAQ8plex); K6(iTRAQ8plex)	-	-	-	4.4	-	1	2623.4413	4	656.6157837	-5.293205664	36.60168
														EDQTEYLEER	14	6	2	N-Term(iTRAQ8plex)	-	-	-	4.15	48	0	1615.76946	2	808.3883667	-3.629553423	18.78577833
														EGLELPEDEEEK	6	6	2	N-Term(iTRAQ8plex); K12(iTRAQ8plex)	-	-	-	3.96	57	0	2025.03191	2	1013.019592	-8.174364869	19.69937667
														HFSVEGQLEFR	17	6	2	N-Term(iTRAQ8plex)	-	-	-	3.75	60	0	1652.88022	3	551.6312866	5.726560459	29.77204667
														YESLTDPSK	6	6	2	N-Term(iTRAQ8plex); K9(iTRAQ8plex)	-	-	-	3.36	30	0	1647.88913	3	549.9678955	-9.617167953	26.7403
														VILHLK	4	6	2	N-Term(iTRAQ8plex); K6(iTRAQ8plex)	-	-	-	2.96	29	0	1330.8898	3	444.3014526	-9.968248158	32.28242
														HNDDEQYAWESSAGGSFTVR	4	6	2	N-Term(iTRAQ8plex); Y7(iTRAQ8plex)	-	-	-	-	28	0	2864.35903	3	854.0576172	-2.333053977	33.96651167
														FENLCK	1	6	2	N-Term(iTRAQ8plex); C5(Methylthio); K6(iTRAQ8plex)	-	-	-	-	26	0	1407.74485	3	469.9197998	-9.67230388	34.797355
														GVVDSEDIPLNLSR	8	4	3	N-Term(iTRAQ8plex)	-	-	-	4.64	70	0	1817.98552	2	909.4963989	-3.06936902	34.48323833
154354966	IMMT	MICOS complex subunit MIC60 isoform 3	NP_001093640.1	23.96	15	15	47	747	82.6	6.57	0.955	0.654	0.658																
														GIEQAVQSHAVAEEEAR	10	7	1	N-Term(iTRAQ8plex)	0.867	0.703	0.758	6.33	73	0	2128.08561	3	710.0349121	-1.643831593	25.51456833
														TDHPEIGEGKPTPALSEEASSSSIR	2	2	1	N-Term(iTRAQ8plex); K10(iTRAQ8plex)	-	-	-	5.52	31	0	3203.64785	4	801.6674194	-5.079661185	21.95373833
														LHNMIVDLDNVVK	4	7	1	N-Term(iTRAQ8plex); K13(iTRAQ8plex)	0.469	0.683	0.555	5.39	53	0	2118.22098	3	706.7451782	0.382452056	48.33254833
														LFEMVLGPAAYNVPLPK	2	6	1	N-Term(iTRAQ8plex); K17(iTRAQ8plex)	0.707	0.219	0.624	5.04	44	0	2467.43351	3	823.149353	3.702037769	61.30452167
														TIPYSDKLFEMVLGPAAYNVPLPK	3	6	1	N-Term(iTRAQ8plex); K7(iTRAQ8plex); K24(iTRAQ8plex)	1.643	1.067	0.776	5.01	42	1	3576.03476	3	1192.683105	0.906550541	61.99814
														AVDEAADALLK	2	6	1	N-Term(iTRAQ8plex); K11(iTRAQ8plex)	1.101	0.87	0.981	4.53	43	0	1724.00406	3	575.3395386	-0.585165294	53.99939333
														ELDSITPEVLPGWK	4	7	1	N-Term(iTRAQ8plex); K14(iTRAQ8plex)	1.293	0.877	0.929	4.46	56	0	2192.23198	2	1096.619629	-4.730387837	36.025075
														QAAAHTDHLR	2	7	1	N-Term(iTRAQ8plex)	1.25	0.494	0.547	3.61	59	0	1423.76841	3	475.2609863	-1.668505647	9.10284
														GVYSEETLR	7	7	1	N-Term(iTRAQ8plex)	0.986	0.697	0.658	3.36	42	0	1357.73371	3	453.2494202	5.299970096	23.02694
														LSTDDLNSLIAHAHR	2	7	1	N-Term(iTRAQ8plex)	1.058	-	0.718	3.34	64	0	1967.05556	4	492.5193481	-2.881770084	31.16260167
														ERPPEEVAAR	3	4	1	N-Term(iTRAQ8plex)	0.905	0.552	0.524	3.21	29	0	1457.79814	2	729.40271	-2.26231467	10.721445
														VAQDWLK	2	7	1	N-Term(iTRAQ8plex); K7(iTRAQ8plex)	1.082	0.682	0.644	3.1	26	0	1467.88237	2	734.4448242	2.984242869	25.00322
														LSEQELQFR	2	7	1	N-Term(iTRAQ8plex)	0.971	0.57	0.638	2.95	36	0	1453.79289	2	727.4000854	-1.635048274	29.58093167
														VAMIDETR	1	7	1	N-Term(iTRAQ8plex)	0.722	0.498	0.523	-	41	0	1238.66826	2	619.8377686	-2.745537876	25.02384833
														SLEDALR	1	6	1	N-Term(iTRAQ8plex)	0.938	0.569	0.609	-	29	0	1107.63054	2	554.3189087	-0.560238423	22.64319
154816184	TMEM55B	type 1 phosphatidylinositol 4,5-bisphosphate 4-phosphatase isoform 2	NP_653169.2	11.55	2	2	5	277	29.4	8.91	1.125	0.694	0.611																
														IINLGPVHPGPLSPEPQPMGVR	2	2	1	N-Term(iTRAQ8plex)	1.34	1.107	0.864	4.71	34	0	2609.45835	3	870.4909668	1.657754235	38.33114833
														CPCNCLLICK	3	4	1	N-Term(iTRAQ8plex); C1(Methylthio); C3(Methylthio); C5(Methylthio); [illegible]	1.122	0.668	0.533	3.44	30	0	1901.84616	3	634.6202393	-6.793807409	83.08793333
155030202	RAP1GDS1	rap1 GTPase-GDP dissociation stimulator 1 isoform 6	NP_001093900.1	2.33	1	1	4	516	56.5	5.44	0.967	1.729	1.305																
														SVAQQASLTEQR	4	6	1	N-Term(iTRAQ8plex)	0.967	1.729	1.305	4.68	71	0	1621.88445	2	811.4458618	2.044358227	19.877855
155969697	KRT6C	keratin, type II cytoskeletal 6C	NP_775109.2	54.43	1	35	803	564	60.0	8.00	0.709	0.075	0.082																
														SGFSSISVSR	2	2	1	N-Term(iTRAQ8plex)	0.709	0.075	0.082	3.85	43	0	1330.73491	2	665.8710938	6.056487986	24.93122167
														LALDVEIATYR	117	10	7	N-Term(iTRAQ8plex)	-	-	-	4.83	67	0	1567.90409	3	523.3062134	2.771589637	39.12087
														LALDVEIATYR	8	10	7	N-Term(iTRAQ8plex); Y10(iTRAQ8plex)	-	-	-	-	60	0	1872.106	3	523.3062134	2.771589637	39.12087
														TAAENEFVTLK	10	4	3	N-Term(iTRAQ8plex); K11(iTRAQ8plex)	-	-	-	4.72	51	0	1831.03826	2	916.0227661	-2.1385099	38.95932833
														TAAENEFVTLKK	2	4	3	N-Term(iTRAQ8plex); K11(iTRAQ8plex); K12(iTRAQ8plex)	-	-	-	4.09	34	1	2263.34414	4	566.5914917	0.722654964	26.116015
														FLEQQNK	13	31	8	N-Term(iTRAQ8plex); K7(iTRAQ8plex)	-	-	-	-	33	0	1514.87885	3	505.631134	0.090799082	15.97476833
														GELAIK	4	2	6	N-Term(iTRAQ8plex); K6(iTRAQ8plex)	-	-	-	-	28	0	1238.78995	3	413.6025085	0.089671776	17.83946167
														EYQELMNVK	14	5	3	N-Term(iTRAQ8plex); K9(iTRAQ8plex)	-	-	-	4.08	56	0	1761.96379	2	881.4855347	-1.565630391	28.72846833
														FASFIDKVR	16	27	8	N-Term(iTRAQ8plex); K7(iTRAQ8plex)	-	-	-	3.33	31	1	1691.01076	4	423.5080261	0.114317571	39.05999667
														FASFIDK	57	28	8	N-Term(iTRAQ8plex); K7(iTRAQ8plex)	-	-	-	3.23	35	0	1435.83818	2	718.4240723	0.227470368	33.40336167
														NKYEDEINK	1	9	5	N-Term(iTRAQ8plex); K2(iTRAQ8plex); K9(iTRAQ8plex)	-	-	-	-	27	1	2065.16616	4	517.0469971	-1.50249351	14.99920833
														QNLEPLFEQYINNLR	50	4	3	N-Term(iTRAQ8plex)	-	-	-	6.54	76	0	2195.17461	2	1098.090942	-0.753085595	58.40959667
														WTLLQEQGTK	50	4	3	N-Term(iTRAQ8plex); K10(iTRAQ8plex)	-	-	-	4.48	49	0	1812.04447	2	906.5276489	0.242781404	32.60840167
														QLDSIVGER	26	3	3	N-Term(iTRAQ8plex)	-	-	-	3.72	66	0	1320.74237	3	440.9189758	-0.103774792	25.30154833
														QNLEPLFEQYINNLRR	5	4	3	N-Term(iTRAQ8plex)	-	-	-	3.15	27	1	2351.28554	4	588.5768433	3.471235937	52.94203
														NLDLDSIIAEVK	64	8	5	N-Term(iTRAQ8plex); K12(iTRAQ8plex)	-	-	-	5.73	89	0	1938.13909	2	969.5731812	1.173488892	50.31108
														KLLEGEECR	13	13	6	N-Term(iTRAQ8plex); K1(iTRAQ8plex); C8(Methylthio)	-	-	-	2.79	31	1	1730.93911	3	577.6514282	0.43855829	29.74912
														LLEGEECR	16	16	6	N-Term(iTRAQ8plex); C7(Methylthio)	-	-	-	2.75	36	0	1298.63898	3	433.551178	0.260569375	30.10390833
														AEAESWYQTK	19	4	3	N-Term(iTRAQ8plex); K10(iTRAQ8plex)	-	-	-	4.56	66	0	1820.96013	2	910.9831543	-2.67404492	21.62443167
														YEELQVTAGR	43	3	3	N-Term(iTRAQ8plex)	-	-	-	4.27	46	0	1469.78899	2	735.4003296	2.178033814	27.29477167
														YEELQVTAGR	3	3	3	N-Term(iTRAQ8plex); Y1(iTRAQ8plex)	-	-	-	-	32	0	1773.99126	2	735.4003296	2.178033814	27.29477167
														GEMALK	1	4	3	N-Term(iTRAQ8plex); M3(Oxidation); K6(iTRAQ8plex)	-	-	-	-	30	0	1272.74594	3	424.920166	1.365941791	12.0309
														ALYDAELSQMQTHISDTSVVLSMDNNR	37	3	2	N-Term(iTRAQ8plex)	-	-	-	8.02	71	0	3342.63169	3	1114.88208	2.042009116	47.06352
														GSGGLGGACGGAGFGSR	22	3	2	N-Term(iTRAQ8plex); C9(Methylthio)	-	-	-	7.23	107	0	1717.81766	2	859.4060059	-0.301082678	27.15323167
														AIGGGLSSVGGGSSTIK	29	2	2	N-Term(iTRAQ8plex); K17(iTRAQ8plex)	-	-	-	7.14	100	0	2056.18364	2	1028.595459	-1.099099908	27.14610667
														ADTLTDEINFLR	22	3	2	N-Term(iTRAQ8plex)	-	-	-	6.31	70	0	1711.91397	3	571.3095703	-1.579292097	42.44845667
														ISIGGGSCAISGGYGSR	40	3	2	N-Term(iTRAQ8plex); C8(Methylthio)	-	-	-	5.5	76	0	1891.93132	2	946.4692993	0.26412841	34.20610167
														ALYDAELSQMQTHISDTSVVLSMDNNR	15	3	2	N-Term(iTRAQ8plex); M23(Oxidation)	-	-	-	5.22	44	0	3358.62156	3	1114.88208	2.042009116	47.06352
														SLYGLGGSK	26	3	2	N-Term(iTRAQ8plex); K9(iTRAQ8plex)	-	-	-	4.12	48	0	1489.88294	3	497.2991638	-0.351098001	22.94869333

														NKLEGLEDALQK	4	3	2	N-Term(iTRAQ8plex); K2(iTRAQ8plex); K12(iTRAQ8plex)	-	-	-	4.07	33	1	2270.34477	2	1135.676025	-1.562378891	31.82945
														DVDAAYMNKVELQAK	1	9	2	N-Term(iTRAQ8plex); M7(Oxidation); K9(iTRAQ8plex); K15(iTRAQ8plex)	-	-	-	4.03	-	1	2623.44716	4	656.6172485	-2.177302998	24.033175
														LLKEYQELMNVK	1	3	2	N-Term(iTRAQ8plex); K3(iTRAQ8plex); M9(Oxidation); K12(iTRAQ8plex)	-	-	-	3.34	-	1	2436.42789	3	807.4837036	0.634716014	54.63243167
														QEIAEINR	9	3	2	N-Term(iTRAQ8plex)	-	-	-	3.24	37	0	1276.72034	3	426.2434082	-0.48557454	23.91620167
														LLKEYQELMNVK	6	3	2	N-Term(iTRAQ8plex); K3(iTRAQ8plex); K12(iTRAQ8plex)	-	-	-	3.22	29	1	2420.44155	3	807.4837036	0.634716014	54.63243167
														LPGVSR	2	3	2	N-Term(iTRAQ8plex)	-	-	-	-	16	0	932.58293	2	466.795105	-0.158655126	16.53337333
														VELQAK	1	16	4	N-Term(iTRAQ8plex); K6(iTRAQ8plex)	-	-	-	2.75	-	0	1295.81623	3	432.61026	1.470485751	22.94674667
														LDSELR	1	16	3	N-Term(iTRAQ8plex)	-	-	-	-	26	0	1036.59331	2	518.800293	-0.706142412	18.550555
														DVDAAYMNK	25	11	4	N-Term(iTRAQ8plex); K9(iTRAQ8plex)	-	-	-	4.2	50	0	1634.86492	2	817.9366455	-0.50579336	22.28227833
														DVDAAYMNK	13	11	4	N-Term(iTRAQ8plex); M7(Oxidation); K9(iTRAQ8plex)	-	-	-	3.52	47	0	1650.86186	2	817.9366455	-0.50579336	22.28227833
														KDVDAAYMNK	3	11	4	N-Term(iTRAQ8plex); K1(iTRAQ8plex); K10(iTRAQ8plex)	-	-	-	3	31	1	2067.16056	3	689.7250366	-3.199890157	21.25677833
														AQYEEIAQR	16	7	4	N-Term(iTRAQ8plex)	-	-	-	3.65	42	0	1411.75541	3	471.2566528	5.024911587	22.42886333
156071459	SLC25A5	ADP/ATP translocase 2	NP_001143.2	47.32	5	17	258	298	32.8	9.69	0.997	1.210	0.735																
														DFLAGGVAAAISK	30	1	1	N-Term(iTRAQ8plex); K13(iTRAQ8plex)	0.86	1.19	0.748	5.61	83	0	1828.06291	2	914.5393066	-4.143513767	30.92870833
														GTDIMYTGTLDCWR	20	1	1	N-Term(iTRAQ8plex); C12(Methylthio)	0.928	1.231	0.697	5.55	55	0	1981.90752	2	991.4573975	-2.459245994	47.400485
														AAYFGIYDTAK	24	1	1	N-Term(iTRAQ8plex); K11(iTRAQ8plex)	1.06	1.265	0.738	5.37	72	0	1828.00188	2	914.5045776	-4.509101801	33.41265167
														KGTDIMYTGTLDCWR	17	1	1	N-Term(iTRAQ8plex); K1(iTRAQ8plex); C13(Methylthio)	1.211	1.589	0.842	4.19	58	1	2414.21196	2	1207.609619	-0.315067095	40.28715
														KGTDIMYTGTLDCWR	5	1	1	K1(iTRAQ8plex); N-Term(iTRAQ8plex); M6(Oxidation); C13(Methylthio)	0.89	0.871	0.605	3.45	25	1	2430.19553	2	1207.609619	-0.315067095	40.28715
														QIFLGGVDKR	9	1	1	N-Term(iTRAQ8plex); K9(iTRAQ8plex)	1.032	1.169	0.691	3.22	28	1	1741.04595	2	871.0305176	-2.494330648	20.101585
														GTDIMYTGTLDCWR	2	1	1	N-Term(iTRAQ8plex); M5(Oxidation); C12(Methylthio)	0.571	0.661	0.476	2.98	25	0	1997.92241	2	991.4573975	-2.459245994	47.400485
														GMGGAFVLVLYDEIKK	10	3	3	N-Term(iTRAQ8plex); K15(iTRAQ8plex); K16(iTRAQ8plex)	-	-	-	4.73	31	1	2652.56606	4	667.8931274	-0.174781906	56.38034
														EQGVLSFWR	29	2	2	N-Term(iTRAQ8plex)	-	-	-	3.78	55	0	1425.77396	3	475.9295044	-3.697196438	32.06432
														GLGDCLVK	5	2	2	N-Term(iTRAQ8plex); C5(Methylthio); K8(iTRAQ8plex)	-	-	-	2.89	35	0	1458.82927	3	486.9468384	-0.637782234	33.167455
														YFPTQALNFAFKDK	3	6	3	N-Term(iTRAQ8plex); K12(iTRAQ8plex); K14(iTRAQ8plex)	-	-	-	5.78	33	1	2602.4811	4	651.3757324	0.552744648	44.38119833
														YFPTQALNFAFK	30	6	3	N-Term(iTRAQ8plex); K12(iTRAQ8plex)	-	-	-	5.17	57	0	2055.14353	2	1028.076172	-3.558955578	40.728655
														AFFKGAWSNVLR	17	3	3	N-Term(iTRAQ8plex); K4(iTRAQ8plex)	-	-	-	4.99	36	1	2004.15639	4	501.7945557	-3.781403376	40.955375
														GMGGAFVLVLYDELKK	7	3	3	N-Term(iTRAQ8plex); M2(Oxidation); K15(iTRAQ8plex); K16(iTRAQ8plex)	-	-	-	4.6	32	1	2668.55068	4	663.8932495	-1.908778683	55.97575833
														LLLQVQHASK	42	3	3	N-Term(iTRAQ8plex); K10(iTRAQ8plex)	-	-	-	4.59	48	0	1745.0822	2	873.0447388	-4.114637656	25.33012667
														GMGGAFVLVLYDELK	8	3	3	N-Term(iTRAQ8plex); K15(iTRAQ8plex)	-	-	-	4.59	41	0	2220.24057	3	740.751709	-6.9035748	81.47813167
														TAVAPIER	6	6	3	N-Term(iTRAQ8plex)	-	-	-	3.35	63	0	1160.69382	3	387.569458	-0.241688684	16.48437333
														LAADVGK	2	3	3	N-Term(iTRAQ8plex); K7(iTRAQ8plex)	-	-	-	2.94	-	0	1281.80051	3	427.9383545	1.426841812	20.00134333
														GNLANVIR	5	6	3	N-Term(iTRAQ8plex)	-	-	-	2.75	37	0	1160.69975	2	580.8535156	-4.804575841	17.04167167
														GAWSNVLR	2	3	3	N-Term(iTRAQ8plex)	-	-	-	2.73	30	0	1206.68608	2	603.8466797	-2.982171628	22.68380667
156071462	SLC25A6	ADP/ATP translocase 3	NP_001627.2	58.05	2	18	204	298	32.8	9.74	1.238	0.931	0.740																
														DFLAGGIAAAISK	12	1	1	N-Term(iTRAQ8plex); K13(iTRAQ8plex)	1.23	0.885	0.658	5.75	78	0	1842.09153	2	921.5471191	-4.125559166	67.81415833
														QIFLGGVDKHTQFWR	1	1	1	N-Term(iTRAQ8plex); K9(iTRAQ8plex)	2.504	2.584	3.015	3.33	-	1	2440.36342	4	610.8463135	-3.109075343	33.77707333
														GMGGAFVLVLYDEIKK	10	3	3	N-Term(iTRAQ8plex); K15(iTRAQ8plex); K16(iTRAQ8plex)	-	-	-	4.73	31	1	2652.56606	4	667.8931274	-0.174781906	56.38034
														EQGVLSFWR	29	2	2	N-Term(iTRAQ8plex)	-	-	-	3.78	55	0	1425.77396	3	475.9295044	-3.697196438	32.06432
														GLGDCLVK	5	2	2	N-Term(iTRAQ8plex); C5(Methylthio); K8(iTRAQ8plex)	-	-	-	2.89	35	0	1458.82927	3	486.9468384	-0.637782234	33.167455
														YFPTQALNFAFKDK	3	6	3	N-Term(iTRAQ8plex); K12(iTRAQ8plex); K14(iTRAQ8plex)	-	-	-	5.78	33	1	2602.4811	4	651.3757324	0.552744648	44.38119833
														YFPTQALNFAFK	30	6	3	N-Term(iTRAQ8plex); K12(iTRAQ8plex)	-	-	-	5.17	57	0	2055.14353	2	1028.076172	-3.558955578	40.728655
														AFFKGAWSNVLR	17	3	3	N-Term(iTRAQ8plex); K4(iTRAQ8plex)	-	-	-	4.99	36	1	2004.15639	4	501.7945557	-3.781403376	40.955375
														GMGGAFVLVLYDELKK	7	3	3	N-Term(iTRAQ8plex); M2(Oxidation); K15(iTRAQ8plex); K16(iTRAQ8plex)	-	-	-	4.6	32	1	2668.55068	4	663.8932495	-1.908778683	55.97575833
														LLLQVQHASK	42	3	3	N-Term(iTRAQ8plex); K10(iTRAQ8plex)	-	-	-	4.59	48	0	1745.0822	2	873.0447388	-4.114637656	25.33012667
														GMGGAFVLVLYDELK	8	3	3	N-Term(iTRAQ8plex); K15(iTRAQ8plex)	-	-	-	4.59	41	0	2220.24057	3	740.751709	-6.9035748	81.47813167
														TAVAPIER	6	6	3	N-Term(iTRAQ8plex)	-	-	-	3.35	63	0	1160.69382	3	387.569458	-0.241688684	16.48437333
														LAADVGK	2	3	3	N-Term(iTRAQ8plex); K7(iTRAQ8plex)	-	-	-	2.94	-	0	1281.80051	3	427.9383545	1.426841812	20.00134333
														GNLANVIR	5	6	3	N-Term(iTRAQ8plex)	-	-	-	2.75	37	0	1160.69975	2	580.8535156	-4.804575841	17.04167167
														GAWSNVLR	2	3	3	N-Term(iTRAQ8plex)	-	-	-	2.73	30	0	1206.68608	2	603.8466797	-2.982171628	22.68380667
														GADIMYTGTVDCWR	2	2	2	N-Term(iTRAQ8plex); C12(Methylthio)	-	-	-	5.12	55	0	1937.89519	2	969.4512329	4.647774742	41.15287167
														YFAGNLASGGAAGATSLCFVYPLDFAR	18	2	2	N-Term(iTRAQ8plex); C18(Methylthio)	-	-	-	4.94	61	0	3089.52536	3	1131.912842	0.54802354	62.85787
														AAYFGVYDTAK	4	2	2	N-Term(iTRAQ8plex); K11(iTRAQ8plex)	-	-	-	4.63	54	0	1813.98674	2	907.4970093	-4.260982252	40.51684667
														KGADIMYTGTVDCWR	6	2	2	N-Term(iTRAQ8plex); K1(iTRAQ8plex); C13(Methylthio)	-	-	-	3.77	34	1	2370.17484	3	790.7297974	-4.925147278	36.04928
														YFAGNLASGGAAGATSLCFVYPLDFAR	8	2	2	N-Term(iTRAQ8plex); Y1(iTRAQ8plex); C18(Methylthio)	-	-	-	-	67	0	3393.72397	3	1131.912842	0.54802354	62.85787
														KGADIMYTGTVDCWR	1	2	2	K1(iTRAQ8plex); N-Term(iTRAQ8plex); M6(Oxidation); C13(Methylthio)	-	-	-	3.48	-	1	2386.17716	3	790.7297974	-4.925147278	36.04928
156104891	GTF2F1	general transcription factor IIF subunit 1	NP_002087.2	4.84	2	2	3	517	58.2	7.49	1.107	1.898	1.972																
														TLTAEEAEEEWER	2	1	1	N-Term(iTRAQ8plex)	1.341	2.424	2.498	4.84	43	0	1896.91094	2	948.9591064	-1.041181922	31.66458667
														EFRPEDQPWLLR	1	1	1	N-Term(iTRAQ8plex)	0.913	1.486	1.556	2.99	-	0	1890.01389	3	630.6761475	-1.958804223	32.88539
156105689	MUT	methylmalonyl-CoA mutase, mitochondrial precursor	NP_000246.2	1.47	1	1	2	750	83.1	6.93	0.657	0.212	0.373																
														IIADIFEYTAK	2	1	1	N-Term(iTRAQ8plex); K11(iTRAQ8plex)	0.657	0.212	0.373	2.76	32	0	1892.10422	3	631.3729248	2.785186991	50.46074167
156105695	HELZ2	helicase with zinc finger domain 2 isoform 2	NP_208384.3	0.48	1	1	1	2080	231.5	7.69	1.402	0.856	1.270																
														VVVTTTSQAR	1	2	1	N-Term(iTRAQ8plex)	1.402	0.856	1.27	2.84	-	0	1365.79753	2	683.4024048	-2.063519387	19.27755667
156139137	C14orf159	UPF0317 protein C14orf159, mitochondrial isoform c	NP_001095839.1	1.60	1	1	1	564	61.0	7.09	0.701	0.630	0.254																
														LVDVTTAQV	1	4	1	N-Term(iTRAQ8plex)	0.701	0.63	0.254	-	26	0	1249.74077	2	625.3740234	8.184861408	23.22113833
156523968	PARP1	poly [ADP-ribose] polymerase 1	NP_001609.2	18.44	15	15	52	1014	113.0	8.88	1.549	1.588	1.490																
														HPDVEVDGFSELR	18	1	1	N-Term(iTRAQ8plex)	1.46	1.512	1.498	4.91	64	0	1803.91166	3	601.9754028	-3.479135959	27.26757
														VVDRDSEEAEIIR	4	1	1	N-Term(iTRAQ8plex)	1.464	1.516	1.242	4.65	63	1	1834.97844	2	917.9928589	-1.542332323	21.50975
														VVSEDFLQDVSASTK	2	1	1	N-Term(iTRAQ8plex); K15(iTRAQ8plex)	1.989	1.592	1.423	4.64	48	0	2233.20444	3	745.072998	-5.736335259	32.49950333
														AMVEYEIDLQK	2	1	1	N-Term(iTRAQ8plex); K11(iTRAQ8plex)	1.454	1.685	1.483	4.22	44	0	1947.05326	3	649.68927	-9.50384451	52.920055
														VFSATLGLVDIVK	11	1	1	N-Term(iTRAQ8plex); K13(iTRAQ8plex)	1.842	2.042	1.868	4.16	73	0	1970.21518	2	985.6099243	-1.06551304	50.86449333
														GGAAVDPDSGLEHSAHVLEK	3	1	1	N-Term(iTRAQ8plex); K20(iTRAQ8plex)	1.475	1.728	1.617	3.78	32	0	2597.38968	3	866.4680786	4.485768894	24.185405
														MAIMVQSPMFDGK	1	1	1	N-Term(iTRAQ8plex); K13(iTRAQ8plex)	0.916	1.162	1.51	3.69	-	0	2063.08097	3	688.3651733	-6.734844968	33.84075167
														GIYFADMVSK	2	1	1	N-Term(iTRAQ8plex); K10(iTRAQ8plex)	2.372	2.651	2.145	3.52	25	0	1738.95517	3	580.3232422	-6.128960461	54.72415833
														GGSDDSSKDPIDVNYEK	2	1	1	N-Term(iTRAQ8plex); K8(iTRAQ8plex); K17(iTRAQ8plex)	2.185	1.795	1.501	3.37	24	1	2738.40543	3	913.4733276	-7.074058368	19.25442667
														FYTLIPHDFGMK	1	1	1	N-Term(iTRAQ8plex); K12(iTRAQ8plex)	1.836	1.249	1.217	3.04	-	0	2077.13431	3	693.0496216	-2.792214193	48.271325
														LQLLEDDKENR	1	1	1	N-Term(iTRAQ8plex); K8(iTRAQ8plex)	1.841	1.755	1.345	2.97	-	1	1981.10007	3	661.038208	-8.783264823	29.76702833
														AEPVEVVAPR	2	1	1	N-Term(iTRAQ8plex)	1.007	1.052	0.856	-	65	0	1370.78913	3	457.6012268	-3.964234885	20.821525
														LLWHGSR	1	1	1	N-Term(iTRAQ8plex)	1.601	1.204	1.447	-	32	0	1172.67459	3	391.5630493	-8.18351555	18.32547833
														IAPPEAPVTGYMFGK	1	1	1	N-Term(iTRAQ8plex); M12(Oxidation); K15(iTRAQ8plex)	1.485	1.162	1.497	-	32	0	2202.21684	2	1101.612061	3.586465052	32.17961667
														ELLIFNK	1	1	1	N-Term(iTRAQ8plex); K7(iTRAQ8plex)	0.698	0.584	0.818	-	25	0	1484.93022	2	742.96875	0.358319867	49.13507
156523970	AHSG	alpha-2-HS-glycoprotein preproprotein	NP_001613.2	1.91	1	1	2	367	39.3	5.72	2.530	1.878	2.881																
														CNLLAEK	2	1	1	N-Term(iTRAQ8plex); C1(Methylthio); K7(iTRAQ8plex)	2.53	1.878	2.881	-	30	0	1444.79743	3	482.2706604	-9.554522012	28.77919
156564401	NSF	vesicle-fusing ATPase	NP_006169.2	1.34	1	1	2	744	82.5	6.95	0.802	1.844	1.936																
														LQILHIHTAR	2	1	1	N-Term(iTRAQ8plex)	0.802	1.844	1.936	2.78	25	0	1505.93227	4	377.2385254	6.952757901	26.87408167
156627571	SORD	sorbitol dehydrogenase	NP_003095.2	6.72	1	1	2	357	38.3	7.97	-	-	-																
														LPDNVTFEEGALIEPLSVGIHACR	2	1	1	N-Term(iTRAQ8plex); C23(Methylthio)	-	-	-	2.96	27	0	2930.52463	3	977.5130615	6.404662193	51.67441
156631005	PSMD8	26S proteasome non-ATPase regulatory subunit 8	NP_002803.2	7.14	2	2	4	350	39.6	9.70	0.419	1.713	1.477																
														LVLLELNFLPTTGTK	2	1	1	N-Term(iTRAQ8plex); K15(iTRAQ8plex)	-	-	-	3.69	37	0	2267.36771	3	756.4607544	-6.977856453	59.01962667
														VAEFHTELER	2	1	1	N-Term(iTRAQ8plex)	0.419	1.713	1.477	2.87	27	0	1534.80997	3	512.2748413	-4.411376228	21.97605167
156671217	EPN2	epsin-2 isoform a	NP_683723.2	1.71	1	1	3	584	62.3	7.53	-	-	-																
														ALTLLDYLIK	6	3	1	N-Term(iTRAQ8plex); K10(iTRAQ8plex)	1.124	0.519	1.662	3.39	47	0	1771.12351	3	591.0460205	2.572928097	60.77770167
156713442	PSMA4	proteasome subunit alpha type-4 isoform 1	NP_001096137.1	16.48	4	4	14	261	29.5	7.72	0.905	2.354	2.485																
														ATCIGNNSAAAVSMLK	2	2	1	N-Term(iTRAQ8plex); C3(Methylthio); K16(iTRAQ8plex)	0.712	0.994	0.953	4.85	57	0	2205.14829	3	735.7209473	-7.602097878	29.66415333
														LLDEVFFSEK	8	1	1	N-Term(iTRAQ8plex); K10(iTRAQ8plex)	0.885	2.506	2.579	4.55	48	0	1835.02544	2	918.0163574	-8.541067564	73.53715
														TTIFSPEGR	2	1	1	N-Term(iTRAQ8plex)	0.903	2.001	1.681	2.91	28	0	1311.71489	2	656.361084	-4.68848169	23.15712
														VEIATLTR	2	2	1	N-Term(iTRAQ8plex)	0.96	2.545	2.706	-	25	0	1206.731	2	603.8691406	-4.107518561	26.87972
157168362	PNP	purine nucleoside phosphorylase	NP_000261.2	8.65	2	2	5	289	32.1	6.95	0.951	2.235	1.388																
														FEVGDIMLIRDHINLPGFSGQNPLR	2	1	1	N-Term(iTRAQ8plex)	-	-	-	4.8	35	1	3142.69228	4	786.4285278	4.71893103	56.08937167
														DHINLPGFSGQNPLR	3	1	1	N-Term(iTRAQ8plex)	0.951	2.235	1.388	4.5	38	0	1969.04844	3	657.0209961	-3.720715357	31.13590167
157266300	ANPEP	aminopeptidase N precursor	NP_001141.2	25.34	18	18	77	967	109.5	5.48	0.192	0.214	0.220																
														ENSLLFDPLSSSSSNK	6	1	1	N-Term(iTRAQ8plex); K16(iTRAQ8plex)	0.224	0.403	0.3	5.67	54	0	2333.23606	3	778.4177856	-2.450506308	29.31223
														QQQDYWLIDVR	8	1	1	N-Term(iTRAQ8plex)	0.253	0.235	0.263	5.29	59	0	1767.9459	3	589.9868164	7.203087368	36.41514833
														QWMENPNNNPIHPNLR	5	1	1	N-Term(iTRAQ8plex)	0.398	0.065	0.273	4.89	33	0	2278.14383	3	760.0527954	-0.657693122	27.50701167
														QYMPWEAALSSLSYFK	12	1	1	N-Term(iTRAQ8plex); K16(iTRAQ8plex)	0.251	-	0.029	4.83	48	0	2529.33579	2	1265.18103	9.469856513	63.775225
														FSTEYELQQLEQFKK	2	1	1	N-Term(iTRAQ8plex); K14(iTRAQ8plex); K15(iTRAQ8plex)	-	-	0.083	4.83	26	1	2830.58217	4	708.401001	2.389164431	42.32250833
														EATDVIIIHSK	10	1	1	N-Term(iTRAQ8plex); K11(iTRAQ8plex)	0.076	0.09	0.073	4.53	49	0	1834.08733	2	917.5473022	-1.157757202	23.66407333
														QYMPWEAALSSLSYFK	2	1	1	N-Term(iTRAQ8plex); M3(Oxidation); K16(iTRAQ8plex)	0.093	-	-	4.48	47	0	2545.3295	2	1265.18103	9.469856513	63.775225
														VVATTQMQAADAR	2	1	1	N-Term(iTRAQ8plex)	0.025	0.06	0.046	4.43	75	0	1665.90007	2	833.4536743	6.279315902	23.77861
														QVTPLFIHFR	9	1	1	N-Term(iTRAQ8plex)	0.429	0.267	0.257	4.34	56	0	1561.91288	3	521.3094482	-1.197136134	31.97442667
														YLSYTLNPDLIR	8	1	1	N-Term(iTRAQ8plex)	0.112	0.147	0.112	4.24	57	0	1771.98808	2	886.4985352	0.108574271	44.32748
														AQIINDAFNLASAHK	2	1	1	N-Term(iTRAQ8plex); K15(iTRAQ8plex)	-	-	-	3.87	40	0	2221.24917	4	556.067749	-2.61343317	46.62345
														KVVATTQMQAADAR	2	1	1	N-Term(iTRAQ8plex); K1(iTRAQ8plex)	0.101	-	0.074	3.82	40	1	2098.18741	3	700.0673218	-1.20542996	19.24267333
														VMAVDALASSHPLSTPASEINTPAQISELFDAISYSK	2	1	1	N-Term(iTRAQ8plex); K37(iTRAQ8plex)	-	-	-	3.77	24	0	4469.33852	4	1118.090088	0.30661864	65.15431
														VNYDEENWR	2	1	1	N-Term(iTRAQ8plex)	0.394	0.343	0.279	3.3	24	0	1528.72844	2	764.8678589	-3.238081028	21.23360833
														DHSAIPVINR	1	1	1	N-Term(iTRAQ8plex)	0.513	0.401	0.491	2.99	-	0	1425.8115	3	475.9420166	-0.066450097	15.77291833

														GVGGSQPPDIDKTELVEPTEYLVVHLK	1	1	1	N-Term(iTRAQ8plex); K12(iTRAQ8plex); K27(iTRAQ8plex)	-	-	-	2.82	-	1	3833.14802	5	767.4354248	0.411205993	47.015515
														DLTALSNMLPK	1	1	1	N-Term(iTRAQ8plex); K11(iTRAQ8plex)	0.431	0.281	0.243	2.74	-	0	1811.0557	3	604.3567505	-0.012746795	37.87358333
														GLYVFK	1	1	1	N-Term(iTRAQ8plex); K6(iTRAQ8plex)	0.108	0.155	0.177	-	31	0	1334.82829	2	667.9177856	-0.701686457	25.09741667
														SEYMEGNVR	1	1	1	N-Term(iTRAQ8plex)	0.142	0.209	0.23	-	24	0	1388.67742	2	694.8423462	-0.558471808	17.655005
157311635	FAM83H	protein FAM83H	NP_940890.3	6.87	5	5	13	1179	127.1	6.99	1.020	1.015	0.983																
														HSFATEGAGAVENFAAAR	6	6	1	N-Term(iTRAQ8plex)	1.02	1.222	0.989	6.4	68	0	2110.05851	3	704.024353	-1.651834752	31.70676333
														DPGGGAGAITVASHSK	2	6	1	N-Term(iTRAQ8plex); K16(iTRAQ8plex)	0.847	0.434	0.353	4.83	39	0	2033.13095	3	678.3818359	3.601963013	17.47934
														FLATEGAPDFLCPEELEHVSR	2	6	1	N-Term(iTRAQ8plex); C12(Methylthio)	1.429	1.711	-	4.61	57	0	2710.31668	3	904.1104126	0.281237604	54.04948833
														RLEAEAGPAGELAGAR	2	6	1	N-Term(iTRAQ8plex)	0.724	0.63	0.983	4.46	37	1	1872.01975	3	624.6781006	-2.357235916	22.90760667
														VNLHHVDFLR	1	1	1	N-Term(iTRAQ8plex)	1.331	0.281	-	-	34	0	1553.89382	4	389.2289124	5.404638307	25.19315167
157388900	IMPAD1	inositol monophosphatase 3	NP_060283.3	7.24	1	1	2	359	38.7	6.86	-	1.414	-																
														EVPAESVTVWIDPLDATQEYTEDLRK	2	1	1	N-Term(iTRAQ8plex); K26(iTRAQ8plex)	-	1.414	-	2.69	30	1	3612.89536	3	1204.969971	1.637899465	47.66462833
157388904	DNAAF5	dynein assembly factor 5, axonemal	NP_060272.3	1.29	1	1	1	855	93.5	6.42	1.325	0.658	1.502																
														ELLVHLDDPER	1	1	1	N-Term(iTRAQ8plex)	1.325	0.658	1.502	2.76	-	0	1639.89273	3	547.3024292	-1.827529497	26.666325
157388993	SYNJ2BP	synaptojanin-2-binding protein	NP_060843.2	8.28	1	1	6	145	15.9	6.30	0.916	0.736	0.738																
														NLLHQDAVDLFR	6	4	1	N-Term(iTRAQ8plex)	0.916	0.736	0.738	3.77	41	0	1744.96213	3	582.3249512	-2.5851788	34.449005
157389005	CAPN2	calpain-2 catalytic subunit isoform 1	NP_001739.2	19.43	9	9	39	700	80.0	4.98	0.520	0.891	1.802																
														WNDNCPSWNTIDPEER	4	3	1	N-Term(iTRAQ8plex); C5(Methylthio)	0.337	0.853	1.544	5.98	91	0	2326.03594	2	1163.521606	8.105623104	40.18534
														FADDQLIIDFDNFVR	6	2	1	N-Term(iTRAQ8plex)	0.33	0.583	1.64	5.39	77	0	2132.11016	2	1066.558716	6.338190639	63.350845
														LEEEDEDEEDGESGCTFLVGLIQK	2	3	1	N-Term(iTRAQ8plex); C15(Methylthio); K24(iTRAQ8plex)	-	1.196	2.759	4.74	79	0	3338.60337	3	1113.539307	8.403026294	59.44820833
														LGLKEFYILWTK	5	2	1	N-Term(iTRAQ8plex); K4(iTRAQ8plex); K12(iTRAQ8plex)	0.345	0.85	3.25	4.26	20	1	2423.46897	3	808.4945068	-5.770258409	55.70140667
														NYPNTFWMNPQYLIK	2	3	1	N-Term(iTRAQ8plex); K15(iTRAQ8plex)	1.034	1.092	1.458	4.23	40	0	2537.33475	3	846.4497681	-4.883214868	41.56163167
														RPTEICADPQFIIGGATR	6	2	1	N-Term(iTRAQ8plex); C6(Methylthio)	0.615	0.914	1.892	4.17	51	0	2295.18491	3	765.7331543	-1.865932177	37.68663167
														MPCQLHQVIVAR	6	2	1	N-Term(iTRAQ8plex); C3(Methylthio)	0.533	0.95	1.9	3.82	66	0	1744.92771	3	582.3140869	-2.845279945	29.32770667
														IMVDMLDSDGSGK	2	2	1	N-Term(iTRAQ8plex); K13(iTRAQ8plex)	0.925	0.992	1.11	3.7	32	0	1976.01157	3	659.342041	-8.791195047	57.24399333
														NPWGEVEWTGR	4	3	1	N-Term(iTRAQ8plex)	0.604	0.884	1.475	3.65	39	0	1634.81304	2	817.9101563	-6.034586086	45.88840333
														MPCQLHQVIVAR	2	2	1	N-Term(iTRAQ8plex); M1(Oxidation); C3(Methylthio)	0.184	0.503	0.985	3.2	37	0	1760.92453	3	582.3140869	-2.845279945	29.32770667
157412270	HNRNPM	heterogeneous nuclear ribonucleoprotein M isoform b	NP_112480.2	44.86	25	25	112	691	73.6	8.82	1.091	0.799	0.717																
														GIGMGNIGPAGMGMEGIGFGINK	2	9	1	N-Term(iTRAQ8plex); M4(Oxidation); K23(iTRAQ8plex)	1.103	1.014	1.006	8.19	48	0	2802.48172	3	945.4887695	2.010633534	32.69848333
														GNFGGSFAGSFGGAGGHAPGVAR	2	18	1	N-Term(iTRAQ8plex)	1.122	1.023	1.588	6.87	74	0	2339.14927	3	780.3879395	-3.897224842	27.06519333
														GIGTVTFEQSIEAVQAISMFNGQLLFDRPMHVK	3	16	1	N-Term(iTRAQ8plex); M30(Oxidation); K33(iTRAQ8plex)	-	-	-	6.36	25	0	4288.29653	4	1072.82959	5.718633222	67.50159833
														VGEVTYVELLMDAEGK	12	8	1	N-Term(iTRAQ8plex); K16(iTRAQ8plex)	1.172	0.799	0.732	6.12	82	0	2361.27666	2	1181.141968	-2.787633164	45.75661833
														MGGMEGPFGGGMENMGR	4	9	1	N-Term(iTRAQ8plex)	1.055	0.692	0.592	5.92	73	0	2018.89286	3	673.6358032	3.43015626	34.82173833
														MGLAMGGGGGASFDR	3	18	1	N-Term(iTRAQ8plex); M1(Oxidation); M5(Oxidation)	0.972	0.688	0.741	5.89	58	0	1719.81919	2	844.4157715	2.615822534	30.19721
														VGEVTYVELLMDAEGK	2	8	1	N-Term(iTRAQ8plex); M11(Oxidation); K16(iTRAQ8plex)	1.367	0.82	0.796	5.85	49	0	2377.29911	2	1181.141968	-2.787633164	45.75661833
														MGLAMGGGGGASFDR	2	18	1	N-Term(iTRAQ8plex)	0.907	0.732	0.605	5.24	68	0	1687.82427	2	844.4157715	2.615822534	30.19721
														GIGTVTFEQSIEAVQAISMFNGQLLFDRPMHVK	2	16	1	N-Term(iTRAQ8plex); K33(iTRAQ8plex)	-	-	-	4.55	25	0	4272.30678	4	1072.82959	5.718633222	67.50159833
														GIGMGNIGPAGMGMEGIGFGINK	2	9	1	N-Term(iTRAQ8plex); M4(Oxidation); M12(Oxidation); M14(Oxidation); [illegible]	1.177	-	0.597	4.43	88	0	2834.45176	3	945.4887695	2.010633534	32.69848333
														MVPAGMGAGLER	2	18	1	N-Term(iTRAQ8plex); M1(Oxidation); M6(Oxidation)	0.834	0.627	0.815	4.39	53	0	1524.77872	3	508.9310913	-1.928558012	16.713525
														MGPLGLDHMASSIER	12	18	1	N-Term(iTRAQ8plex)	1.236	0.987	0.735	4.36	60	0	1917.98124	3	639.9985962	-0.858181379	31.11748333
														MGPAMGPALGAGIER	2	18	1	N-Term(iTRAQ8plex)	0.881	0.529	0.532	4.34	47	0	1731.91631	2	866.461792	-1.463082029	31.408715
														ADILEDKDGK	3	16	1	N-Term(iTRAQ8plex); K7(iTRAQ8plex); K10(iTRAQ8plex)	1.142	0.771	0.603	4.12	35	1	2016.17314	2	1008.59021	-0.445397325	19.72700667
														NLPFDFTWK	6	18	1	N-Term(iTRAQ8plex); K9(iTRAQ8plex)	0.92	0.848	0.795	4.03	33	0	1775.98491	2	888.4987183	-2.213636455	40.86719
														LKEVFSMAGVVVR	3	16	1	N-Term(iTRAQ8plex); K2(iTRAQ8plex)	1.091	0.737	0.953	3.98	48	1	2043.23	4	511.5629578	2.67217821	55.12513333
														MGPAMGPALGAGIER	3	18	1	N-Term(iTRAQ8plex); M1(Oxidation)	1.021	0.791	0.8	3.95	47	0	1747.91141	2	866.461792	-1.463082029	31.408715
														MGPLGLDHMASSIER	2	18	1	N-Term(iTRAQ8plex); M1(Oxidation)	1.564	1.231	0.93	3.59	38	0	1933.97641	3	639.9985962	-0.858181379	31.11748333
														AFITNIPFDVK	1	8	1	N-Term(iTRAQ8plex); K11(iTRAQ8plex)	1.182	0.542	0.654	3.56	-	0	1873.10154	3	625.0386963	-1.519235748	35.57201
														MGAGLGHGMDR	5	18	1	N-Term(iTRAQ8plex); M1(Oxidation); M9(Oxidation)	0.945	0.709	0.575	3.51	46	0	1437.68561	3	479.900177	-1.463145759	8.6168
														GCAVVEFK	6	8	1	N-Term(iTRAQ8plex); C2(Methylthio); K8(iTRAQ8plex)	1.289	0.94	0.734	3.46	39	0	1506.82866	2	753.9179688	1.173710088	40.19890667
														QGGGGGGGSVPGIER	2	10	1	N-Term(iTRAQ8plex)	0.95	0.566	0.905	3.32	34	0	1588.83249	3	530.2823486	-1.295700819	18.28537
														MGLAMGGGGGASFDR	2	18	1	N-Term(iTRAQ8plex); M5(Oxidation)	0.69	0.456	0.419	3.25	60	0	1703.82317	2	844.4157715	2.615822534	30.19721
														FNECGHVLYADIK	1	18	1	N-Term(iTRAQ8plex); C4(Methylthio); K13(iTRAQ8plex)	0.921	1.181	1.317	3.17	-	0	2163.10691	3	721.7071533	-5.46232321	41.59259
														DKFNECGHVLYADIK	1	18	1	N-Term(iTRAQ8plex); K2(iTRAQ8plex); C6(Methylthio); K15(iTRAQ8plex)	0.885	1.43	1.374	3.09	-	1	2710.44887	4	678.3676758	1.059213855	35.81929167
														MGAGMGFGLER	5	18	1	N-Term(iTRAQ8plex)	1.213	0.939	0.64	2.95	46	0	1429.71965	2	715.3623047	-4.251571175	39.11532333
														FESPEVAER	4	18	1	N-Term(iTRAQ8plex)	0.969	0.687	0.628	2.92	31	0	1367.70574	2	684.3565063	-3.76193837	15.80484333
														MGAGMGFGLER	5	18	1	N-Term(iTRAQ8plex); M1(Oxidation)	1.028	0.666	0.648	2.9	36	0	1445.71416	2	715.3623047	-4.251571175	39.11532333
														EKVGEVTYVELLMDAEGK	1	8	1	N-Term(iTRAQ8plex); K2(iTRAQ8plex); K18(iTRAQ8plex)	-	-	-	2.83	-	1	2922.63588	4	731.4144287	3.323596963	49.374445
														MVPAGMGAGLER	1	18	1	N-Term(iTRAQ8plex)	1.04	0.775	0.815	2.81	-	0	1492.78752	3	508.9310913	-1.928558012	16.713525
														AAEVLNK	3	16	1	N-Term(iTRAQ8plex); K7(iTRAQ8plex)	1.355	0.973	0.828	2.78	32	0	1352.84075	2	676.9240112	3.662323857	15.42805667
														ACQIFVR	5	18	1	N-Term(iTRAQ8plex); C2(Methylthio)	0.937	0.703	0.537	2.77	27	0	1186.63249	2	593.8198853	-4.507218718	44.444545
														MVPAGMGAGLER	1	18	1	N-Term(iTRAQ8plex); M1(Oxidation)	1.203	0.793	0.717	-	33	0	1508.78484	3	508.9310913	-1.928558012	16.713525
														VGQTIER	1	18	1	N-Term(iTRAQ8plex)	1.105	0.755	0.544	-	24	0	1106.64263	2	553.8249512	-4.080428627	13.41375667
														MEESMK	1	16	1	N-Term(iTRAQ8plex); M1(Oxidation); M5(Oxidation); K6(iTRAQ8plex)	0.632	0.72	0.427	-	21	0	1394.71149	3	465.5753479	-0.072308021	8.160981667
157419138	LAMC2	laminin subunit gamma-2 isoform a precursor	NP_005553.2	26.66	25	25	80	1193	130.9	6.19	0.271	0.215	0.210																
														MQQAEQALQDILR	6	3	1	N-Term(iTRAQ8plex)	0.197	0.131	0.14	6.6	81	0	1848.01096	3	616.6751709	8.55955168	55.546485
														LNEHPSNNWSPQLSYFEYR	2	3	1	N-Term(iTRAQ8plex)	0.328	0.354	0.327	6.27	36	0	2685.30869	3	895.7744141	3.253867064	38.33496333
														CLPGFHMLTDAGCTQDQR	6	3	1	N-Term(iTRAQ8plex); C1(Methylthio); C13(Methylthio)	0.367	0.194	0.163	5.02	46	0	2389.05521	3	797.0232544	0.772955596	43.33119667
														HPSAHDVILEGAGLR	3	3	1	N-Term(iTRAQ8plex)	0.44	0.444	0.261	4.86	58	0	1876.02958	3	626.0147095	-2.519220429	27.42658667
														EATQAEIEADR	8	2	1	N-Term(iTRAQ8plex)	0.225	0.188	0.086	4.71	64	0	1536.79044	3	512.9349976	6.297622326	20.33140833
														LAESHVESASNMEQLTR	2	2	1	N-Term(iTRAQ8plex)	0.442	0.35	0.366	4.67	42	0	2206.10355	3	736.0393677	-1.841038992	24.05544833
														CDCDPAGIAGPCDAGR	4	3	1	N-Term(iTRAQ8plex); C1(Methylthio); C3(Methylthio); C12(Methylthio)	0.273	0.175	0.109	4.59	56	0	1962.76494	2	981.8861084	1.862615742	44.82618167
														CLNCNDNTDGIHCEK	5	3	1	N-Term(iTRAQ8plex); C1(Methylthio); C4(Methylthio); C13(Methylthio); [illegible]	0.178	0.132	0.253	4.53	41	0	2425.03066	4	607.0131226	-2.100569575	34.08369667
														AGYFGDPLAPNPADK	3	3	1	N-Term(iTRAQ8plex); K15(iTRAQ8plex)	0.357	0.253	0.212	4.5	50	0	2141.16265	2	1071.084961	6.474965847	32.292545
														IQMDQFMQQLQR	2	3	1	N-Term(iTRAQ8plex)	0.273	0.319	0.267	4.4	30	0	1869.96648	2	935.4868774	2.538528293	40.0281
														LDPVYFVAPAK	4	3	1	N-Term(iTRAQ8plex); K11(iTRAQ8plex)	0.319	0.239	0.228	4.37	42	0	1828.09293	3	610.0358276	5.480224771	36.18296333
														CLPGFHMLTDAGCTQDQR	2	3	1	N-Term(iTRAQ8plex); C1(Methylthio); M7(Oxidation); C13(Methylthio)	0.532	0.247	0.231	4.32	29	0	2405.04728	3	797.0232544	0.772955596	43.33119667
														EFDLQVDNR	4	2	1	N-Term(iTRAQ8plex)	0.137	0.058	0.045	4.11	49	0	1439.74077	2	720.3740234	-1.716312444	31.79876
														MQQAEQALQDILR	2	3	1	N-Term(iTRAQ8plex); M1(Oxidation)	0.22	0.213	0.402	3.7	49	0	1863.98636	3	616.6751709	8.55955168	55.546485
														VSDASDKTQQAER	1	2	1	N-Term(iTRAQ8plex); K7(iTRAQ8plex)	0.585	0.341	0.299	3.57	-	1	2043.0945	4	511.5290833	0.877016609	15.84154667
														GHLHLLETSIDGILADVK	3	1	1	N-Term(iTRAQ8plex); K18(iTRAQ8plex)	0.248	0.277	0.21	3.53	-	0	2539.46914	4	635.6227417	-0.521519677	52.713625
														EVEGELER	3	2	1	N-Term(iTRAQ8plex)	0.239	0.296	0.235	3.51	23	0	1264.67522	2	632.8412476	5.169071942	22.23719
														CIHNTAGIYCDQCK	2	3	1	N-Term(iTRAQ8plex); C1(Methylthio); C10(Methylthio); C13(Methylthio); [illegible]	0.45	0.418	0.585	3.34	36	0	2315.03904	3	772.3511963	-0.141977222	35.36617167
														LQGVSDQSFQVEEAKR	2	2	1	N-Term(iTRAQ8plex); K15(iTRAQ8plex)	-	-	-	3.3	47	1	2429.32412	4	608.0864868	-0.166266153	25.49303833
														DAQISEGASR	4	3	1	N-Term(iTRAQ8plex)	0.138	0.031	0.034	3.27	36	0	1337.69585	2	669.3515625	-0.331106657	13.78119
														SLGLQLAK	2	3	1	N-Term(iTRAQ8plex); K8(iTRAQ8plex)	0.213	0.247	0.168	3.05	29	0	1437.92204	2	719.4646606	-2.014456172	34.32409833
														IQMDQFMQQLQR	2	3	1	N-Term(iTRAQ8plex); M7(Oxidation)	0.346	0.216	0.264	3.03	26	0	1885.97185	2	935.4868774	2.538528293	40.0281
														ALGSAAADAQR	2	2	1	N-Term(iTRAQ8plex)	0.347	0.184	0.183	2.99	54	0	1334.73161	2	667.8694458	-1.053633871	16.68637667
														CLPCNCNSK	2	3	1	N-Term(iTRAQ8plex); C1(Methylthio); C4(Methylthio); C6(Methylthio); [illegible]	0.32	0.27	0.226	2.93	16	0	1727.76743	3	576.5939941	-0.905867363	32.68382
														ITSTFHQDVDGWK	1	3	1	N-Term(iTRAQ8plex); K13(iTRAQ8plex)	0.33	0.262	0.272	2.88	-	0	2142.14042	3	714.7183228	-1.678829388	32.307415
														ITAPLMPLGK	1	3	1	N-Term(iTRAQ8plex); M6(Oxidation); K10(iTRAQ8plex)	0.221	0.096	0.078	2.73	-	0	1665.02202	2	833.0146484	-0.568187681	23.65807667
														ADSLSSLVTR	1	2	1	N-Term(iTRAQ8plex)	0.21	0.16	0.169	-	43	0	1352.7647	2	676.8859863	-2.975583087	22.48562
														QALSLVR	1	2	1	N-Term(iTRAQ8plex)	0.237	0.108	0.107	-	18	0	1090.68718	2	545.847229	-1.311019522	22.77046
157649073	CAP1	adenylyl cyclase-associated protein 1	NP_001099000.1	25.68	8	8	22	475	51.6	8.02	0.671	1.869	1.835																
														LSDLLAPISEQIK	6	3	1	N-Term(iTRAQ8plex); K13(iTRAQ8plex)	0.423	1.346	1.205	5.7	49	0	2035.23868	3	679.0844116	6.250035142	49.037025
														SALFAQINQGESITHALK	4	1	1	N-Term(iTRAQ8plex); K18(iTRAQ8plex)	1.501	4.116	3.586	5.5	61	0	2536.42051	3	846.1450195	-5.478569591	41.62211833
														AGAAPYVQAFDSLLAGPVAEYLK	2	2	1	N-Term(iTRAQ8plex); K23(iTRAQ8plex)	-	-	-	5.39	56	0	2959.63089	3	987.2151489	-2.739453989	67.87511333
														NSLDCEIVSAK	4	3	1	N-Term(iTRAQ8plex); C5(Methylthio); K11(iTRAQ8plex)	0.514	1.158	0.98	4.75	50	0	1832.95769	2	916.9824829	-7.077686389	40.58059333
														CVNTTLQIK	2	3	1	N-Term(iTRAQ8plex); C1(Methylthio); K9(iTRAQ8plex)	0.774	3.62	3.096	3.93	39	0	1673.93987	2	837.4735718	-8.366707544	38.05318
														ALLVTASQCQQPAENK	2	3	1	N-Term(iTRAQ8plex); C9(Methylthio); K16(iTRAQ8plex)	0.732	2.221	2.447	3.55	58	0	2355.26641	2	1178.136841	1.822605127	32.15148833
														QVAYIYK	1	3	1	N-Term(iTRAQ8plex); K7(iTRAQ8plex)	0.897	1.572	1.376	3.1	-	0	1492.88738	2	746.9473267	-7.357956038	19.79155167
														SSEMNVLIPTEGGDFNEFPVPEQFK	1	3	1	N-Term(iTRAQ8plex); K25(iTRAQ8plex)	-	-	-	-	25	0	3419.72531	3	1140.579956	-1.092598263	53.48882667
157694492	MYBBP1A	myb-binding protein 1A isoform 2	NP_055335.2	16.79	16	16	55	1328	148.8	9.28	1.470	0.829	0.797																
														LVSIVDSLHLEMEEALTEQVAR	6	3	1	N-Term(iTRAQ8plex)	1.262	-	0.573	6.03	63	0	2786.47757	3	929.4973755	-4.908736854	85.79361333
														AQHQQALSSLELLNVLFR	4	3	1	N-Term(iTRAQ8plex)	0.924	1.215	0.856	5.39	70	0	2371.34331	2	1186.175293	1.399214313	61.13301667
														EAVSSAFFSLLQTLSTQFK	2	3	1	N-Term(iTRAQ8plex); K19(iTRAQ8plex)	-	-	-	4.97	48	0	2712.50058	3	904.8383789	-2.329153877	68.45362
														HPVLCQSLLPILVQHITGPVRPR	2	3	1	N-Term(iTRAQ8plex); C5(Methylthio)	-	-	-	4.51	35	0	2923.66712	5	585.5392456	-4.156007558	59.391615
														EFLDFFWDIAKPEQETR	2	2	1	N-Term(iTRAQ8plex); K11(iTRAQ8plex)	2.756	1.404	1.35	4.28	25	0	2779.45456	3	927.1563721	-0.236859084	61.80010833
														VLDLVEVLVTK	8	3	1	N-Term(iTRAQ8plex); K11(iTRAQ8plex)	1.738	0.713	0.784	4.21	64	0	1836.17827	2	918.5927734	6.327792453	62.28691167
														FCLFHSFFVTK	9	3	1	N-Term(iTRAQ8plex); C2(Methylthio); K11(iTRAQ8plex)	1.474	0.785	0.731	4.1	36	0	2030.0914	3	677.3686523	3.040001666	60.40656333
														HPFSFPLENQAR	2	3	1	N-Term(iTRAQ8plex)	1.366	1.027	1.015	4.06	31	0	1746.91697	3	582.9771729	-3.420881147	27.31337333
														ELEAHSAEAR	4	3	1	N-Term(iTRAQ8plex)	1.213	0.962	0.815	3.87	45	0	1416.73761	3	472.9173889	-0.619593879	12.84251
														EQLHLVMQGDVIR	5	3	1	N-Term(iTRAQ8plex)	1.229	1.234	1.095	3.52	36	0	1842.01938	3	614.6779785	-0.857213089	28.783825
														KLPAIALDLLR	2	3	1	N-Term(iTRAQ8plex); K1(iTRAQ8plex)	1.371	0.766	0.774	3.33	19	1	1831.20169	3	611.0720825	1.49246727	47.70477333
														FLSPPALQGYVAWLR	2	3	1	N-Term(iTRAQ8plex)	1.857	1.082	1.012	3.23	27	0	2022.15752	2	1011.582397	4.770648076	63.61696167
														NSPLTVPMFLSLFSR	2	3	1	N-Term(iTRAQ8plex)	-	-	-	2.9	36	0	2013.11789	3	671.7108154	1.669916637	66.46908

														IFTHHLCR	2	3	1	N-Term(iTRAQ8plex); C7(Methylthio)	1.704	0.742	0.459	2.85	37	0	1376.71872	3	459.5777588	-3.318893494	23.23978333
														MQFWPASYLCFR	2	3	1	N-Term(iTRAQ8plex); C10(Methylthio)	1.732	0.831	0.75	2.71	20	0	1898.91484	3	633.6410522	1.485689346	61.46414667
														LLEYLR	1	2	1	N-Term(iTRAQ8plex)	1.467	0.736	0.754	-	18	0	1110.68059	2	555.8439331	-1.676187628	42.85830333
157694511	NOC2L	nucleolar complex protein 2 homolog	NP_056473.2	4.41	3	3	6	749	84.9	5.62	1.595	0.898	1.251																
														EEGTPLTLYYSHWR	2	1	1	N-Term(iTRAQ8plex)	1.45	1.028	1.251	4.71	40	0	2056.0391	3	686.0178833	-2.463303186	37.97277167
														LFHEVVQAFR	2	1	1	N-Term(iTRAQ8plex)	1.857	0.898	1.47	3.34	36	0	1549.87607	3	517.296875	-2.070842991	44.39912333
														VLAFLVLSR	2	1	1	N-Term(iTRAQ8plex)	1.595	0.725	1.088	3.1	26	0	1321.85808	2	661.4326782	5.400371962	56.580395
157743245	WDR43	WD repeat-containing protein 43	NP_055946.1	4.58	3	3	12	677	74.8	5.57	1.643	0.600	0.705																
														LILLITQVTASEK	2	1	1	N-Term(iTRAQ8plex); K13(iTRAQ8plex)	0.851	0.265	0.572	4.74	65	0	2037.27335	3	679.7626343	-2.278397236	58.023695
														SGGNEVSIEER	6	1	1	N-Term(iTRAQ8plex)	2.011	0.314	0.629	4.5	61	0	1480.75347	2	740.8803711	-0.726476436	17.73462667
														LWVLETK	4	1	1	N-Term(iTRAQ8plex); K7(iTRAQ8plex)	1.149	0.87	0.835	3.3	29	0	1496.92792	3	499.6507568	5.361584333	35.982675
158303333	SYNPO	synaptopodin isoform B	NP_001103444.1	3.80	1	1	2	685	73.6	9.33	-	1.433	0.869																
														RPLGNFTAPPTYTETLSTAPLASWVR	2	4	1	N-Term(iTRAQ8plex)	-	1.433	0.869	4.71	40	0	3150.69687	3	1050.903809	2.488729182	49.61299833
161702986	EZR	ezrin	NP_001104547.1	32.08	8	17	73	586	69.4	6.27	1.274	1.966	1.399																
														SQEQLAAELAEYTAK	2	2	1	N-Term(iTRAQ8plex); K15(iTRAQ8plex)	1.567	3.003	1.897	5.93	51	0	2260.21781	3	754.0774536	-4.571997496	64.47030667
														IAQDLEMYGINYFEIK	6	2	1	N-Term(iTRAQ8plex); K16(iTRAQ8plex)	1.402	3.128	1.874	5.78	77	0	2555.38359	2	1278.195435	6.250028634	58.50195667
														FYPEDVAEELIQDITQK	11	1	1	N-Term(iTRAQ8plex); K17(iTRAQ8plex)	2.707	6.723	2.95	5.57	58	0	2646.41397	3	882.8095093	0.620946703	68.95554
														IAQDLEMYGINYFEIK	2	2	1	N-Term(iTRAQ8plex); M7(Oxidation); K16(iTRAQ8plex)	0.982	1.309	1.043	4.81	44	0	2571.35117	2	1278.195435	6.250028634	58.50195667
														IQVWHAEHR	2	2	1	N-Term(iTRAQ8plex)	1.112	1.618	1.537	3.78	45	0	1479.80426	3	493.9396057	-5.397613403	15.78603167
														EGILSDEIYCPPETAVLLGSYAVQAK	2	1	1	N-Term(iTRAQ8plex); C10(Methylthio); K26(iTRAQ8plex)	-	-	-	3.71	44	0	3420.7855	3	1140.93335	-1.07774132	64.46030167
														ILQLCMGNHELYMR	2	2	1	N-Term(iTRAQ8plex); C5(Methylthio)	1.814	1.822	1.253	3.47	39	0	2071.02646	3	691.0136719	0.067635543	59.40649333
														DNAMLEYLK	2	2	1	N-Term(iTRAQ8plex); K9(iTRAQ8plex)	1.235	2.005	1.226	3.37	56	0	1704.93547	2	852.9713745	-5.641795081	46.87726167
														ELSEQIQR	2	2	1	N-Term(iTRAQ8plex)	1.048	1.392	0.9	3.3	28	0	1306.73271	2	653.8699951	4.486238321	21.48879167
														FYPEDVAEELIQDITQK	2	1	1	N-Term(iTRAQ8plex); Y2(iTRAQ8plex); K17(iTRAQ8plex)	0.367	1.696	1.273	-	44	0	2950.62705	3	882.8095093	0.620946703	68.95554
														IALLEEAR	3	2	2	N-Term(iTRAQ8plex)	-	-	-	-	37	0	1218.72893	2	609.868103	-5.778040664	39.12274333
														VTTMDAELEFAIQPNTTGK	4	7	3	N-Term(iTRAQ8plex); M4(Oxidation); K19(iTRAQ8plex)	-	-	-	5.01	62	0	2690.42168	2	1345.714478	1.818361101	34.020625
														APDFVFYAPR	7	9	3	N-Term(iTRAQ8plex)	-	-	-	3.69	38	0	1486.79473	2	743.906311	3.843240361	35.09177167
														KPDTIEVQQMK	2	9	3	N-Term(iTRAQ8plex); K1(iTRAQ8plex); K11(iTRAQ8plex)	-	-	-	3.47	34	0	2229.28287	3	743.7658081	-9.482974937	17.16221
														RKPDTIEVQQMK	1	9	3	N-Term(iTRAQ8plex); K2(iTRAQ8plex); K12(iTRAQ8plex)	-	-	-	3.34	-	1	2385.38759	4	597.102356	-7.351992573	15.829755
														QLFDQVVK	2	7	3	N-Term(iTRAQ8plex); K8(iTRAQ8plex)	-	-	-	3.26	37	0	1584.94194	2	792.9746094	-9.482473522	25.558705
														IGFPWSEIR	11	9	3	N-Term(iTRAQ8plex)	-	-	-	3.21	41	0	1408.79267	3	470.2690735	2.552475654	39.48728
														LFFLQVK	3	8	3	N-Term(iTRAQ8plex); K7(iTRAQ8plex)	-	-	-	3.06	28	0	1502.9406	2	751.973938	-9.921844845	69.32707
														KAPDFVFYAPR	7	9	3	N-Term(iTRAQ8plex); K1(iTRAQ8plex)	-	-	-	2.98	29	1	1919.08756	3	640.3673706	-6.46282841	28.32321167
162329583	CPSF6	cleavage and polyadenylation specificity factor subunit 6 isoform 1	NP_008938.2	2.54	1	1	2	551	59.2	7.15	0.677	0.800	0.808																
														TPLSEAEFEEIMNR	2	4	1	N-Term(iTRAQ8plex)	0.677	0.8	0.808	4.14	59	0	1969.99309	2	985.5001831	4.456000878	40.7358
162417971	SPCS2	signal peptidase complex subunit 2	NP_055567.2	21.68	3	3	17	226	25.0	8.47	1.020	0.650	0.607																
														SGGSGGCSGAGGASNCGTGSGR	3	1	1	N-Term(iTRAQ8plex); C7(Methylthio); C16(Methylthio)	1.508	0.262	0.44	7.04	73	0	2139.85599	3	713.9568481	-0.865533201	18.43671167
														FFDHSGTLVMDAYEPEISR	10	1	1	N-Term(iTRAQ8plex)	0.703	0.852	0.624	5.94	50	0	2518.22257	3	840.0790405	-0.032947036	42.91759833
														SIFLVAHR	4	1	1	N-Term(iTRAQ8plex)	1.051	0.841	0.592	3.31	38	0	1246.75751	3	416.2573547	0.120038567	21.19104333
163644321	UQCRFS1	cytochrome b-c1 complex subunit Rieske, mitochondrial	NP_005994.2	14.23	3	3	17	274	29.6	8.32	0.941	0.517	0.534																
														EIEQEAAVELSQLRDPQHDLDR	14	1	1	N-Term(iTRAQ8plex)	0.991	1.6	1.046	6.96	51	1	2895.47036	4	724.6230469	-1.631403336	41.83087333
														RLEVLDSTK	1	1	1	N-Term(iTRAQ8plex); K9(iTRAQ8plex)	0.941	0.459	0.564	2.91	-	1	1669.00907	3	557.0078735	-0.841184484	16.29326
														VPDFSEYR	2	1	1	N-Term(iTRAQ8plex)	0.897	0.464	0.415	-	21	0	1316.6751	2	658.8411865	-2.836594339	28.93667
163965362	NACA	nascent polypeptide-associated complex subunit alpha isoform b	NP_001106672.1	25.58	4	4	15	215	23.4	4.56	1.290	3.442	3.209																
														IEDLSQQAQLAAAEK	4	6	1	N-Term(iTRAQ8plex); K15(iTRAQ8plex)	0.762	2.227	1.645	5.27	49	0	2223.22647	3	741.7470093	-7.946431986	35.86748167
														SPASDTYIVFGEAK	3	6	1	N-Term(iTRAQ8plex); K14(iTRAQ8plex)	1.671	3.647	3.253	4.98	47	0	2093.11772	2	1047.0625	-9.463356876	25.82719333
														DIELVMSQANVSR	4	6	1	N-Term(iTRAQ8plex)	1.053	2.202	2.41	4.33	47	0	1765.94475	2	883.4760132	1.533710037	34.950495
														NILFVITKPDVYK	4	6	1	N-Term(iTRAQ8plex); K8(iTRAQ8plex); K13(iTRAQ8plex)	1.93	7.862	7.169	4.24	39	0	2462.49785	2	1231.752563	-6.95674389	42.41801
166795236	TACSTD2	tumor-associated calcium signal transducer 2 precursor	NP_002344.2	26.01	5	5	36	323	35.7	8.87	2.366	1.615	0.494																
														ALGSGMAVDCSTLTSK	4	1	1	N-Term(iTRAQ8plex); C10(Methylthio); K16(iTRAQ8plex)	1.731	1.076	0.317	6.59	71	0	2195.14116	2	1098.074219	3.680209794	37.50314333
														AAGDVDIGDAAYYFER	10	1	1	N-Term(iTRAQ8plex)	3.072	1.373	0.367	4.69	100	0	2036.98203	2	1018.993958	-2.996042683	41.48904
														FVAAVHYEQPTIQIELR	12	1	1	N-Term(iTRAQ8plex)	1.989	1.648	0.54	4.6	64	0	2318.28432	2	1159.64563	1.256034915	42.121255
														TLVRPSEHALVDNDGLYDPDCDPEGR	5	1	1	N-Term(iTRAQ8plex); C21(Methylthio)	-	-	-	4.56	27	0	3233.50551	4	809.1324463	-2.052576904	33.859465
														THHILIDLR	5	1	1	N-Term(iTRAQ8plex)	2.049	3.328	0.878	3.35	38	0	1421.86123	4	356.2207642	5.749830202	19.34181167
166795268	RDH11	retinol dehydrogenase 11 isoform 1 precursor	NP_057110.3	11.64	3	3	6	318	35.4	8.82	0.932	0.682	0.793																
														LANILFTQELAR	4	1	1	N-Term(iTRAQ8plex)	1.126	0.682	0.819	4.29	50	0	1692.99431	2	847.0007935	-0.437689562	52.28624333
														IHFHNLQGEK	1	1	1	N-Term(iTRAQ8plex); K10(iTRAQ8plex)	0.511	0.549	0.646	3.43	-	0	1831.05063	3	611.0217285	3.899945064	17.98815333
														RLWDVSCDLLGLPID	1	2	1	N-Term(iTRAQ8plex); C7(Methylthio)	0.807	0.745	0.828	-	46	1	2065.06572	2	1033.036499	-5.191540886	84.89587
														VYIACR	1	0	1	N-Term(iTRAQ8plex); C5(Methylthio)	-	-	-	-	17	0	1074.56828	2	537.7877808	-5.469542183	29.952205
166795299	SLC2A1	solute carrier family 2, facilitated glucose transporter member 1	NP_006507.2	6.50	4	4	17	492	54.0	8.72	0.776	0.730	0.621																
														TFDEIASGFR	4	1	1	N-Term(iTRAQ8plex)	0.689	0.984	0.687	3.43	29	0	1446.74578	2	723.8765259	-5.050066372	28.83364833
														VTILELFR	5	1	1	N-Term(iTRAQ8plex)	0.802	0.657	0.665	3.24	27	0	1294.80071	2	647.9039917	-2.274111797	56.32319333
														GTADVTHDLQEMK	1	1	1	N-Term(iTRAQ8plex); M12(Oxidation); K13(iTRAQ8plex)	0.927	0.902	0.621	3.03	-	0	2069.07383	3	690.362793	-2.682670711	19.757245
														KVTILELFR	7	1	1	N-Term(iTRAQ8plex); K1(iTRAQ8plex)	0.776	0.73	0.567	-	27	1	1727.098	3	576.3708496	-3.465768526	43.67042167
166795301	PCYOX1	prenylcysteine oxidase 1 precursor	NP_057381.3	9.31	4	4	12	505	56.6	6.18	0.652	0.567	0.519																
														MHMWVEDVLDK	3	1	1	N-Term(iTRAQ8plex); K11(iTRAQ8plex)	0.292	0.532	0.588	5.05	44	0	2011.05686	3	671.0238037	-1.647599954	44.01223167
														SNLISGSVMYIEEK	2	1	1	N-Term(iTRAQ8plex); K14(iTRAQ8plex)	0.74	0.62	0.508	4.78	40	0	2178.17575	3	726.7301025	-8.225659833	35.96746667
														MYEVVYQIGTETR	5	1	1	N-Term(iTRAQ8plex)	0.66	0.435	0.519	4.25	50	0	1892.97185	2	946.9891357	-1.049374571	48.29101333
														CPSIILHDR	2	1	1	N-Term(iTRAQ8plex); C1(Methylthio)	0.652	0.782	0.669	3.59	60	0	1403.75635	3	468.5903015	8.725866853	29.40727
167466198	ICAM1	intercellular adhesion molecule 1 precursor	NP_000192.2	8.65	3	3	11	532	57.8	7.99	2.447	0.774	0.525																
														ASVSVTAEDEGTQR	5	1	1	N-Term(iTRAQ8plex)	3.133	0.606	0.357	5.27	62	0	1753.8897	2	877.4484863	1.525031053	19.02779
														REPAVGEPAEVTTTVLVR	3	1	1	N-Term(iTRAQ8plex)	1.348	0.426	0.458	4.37	47	1	2228.25052	3	743.4216919	-2.135696305	27.09367
														VELAPLPSWQPVGK	3	1	1	N-Term(iTRAQ8plex); K14(iTRAQ8plex)	1.637	0.996	0.583	4.2	35	0	2129.25932	2	1065.133301	0.649823384	38.73863667
167614485	ACAA2	3-ketoacyl-CoA thiolase, mitochondrial	NP_006102.2	7.30	2	2	4	397	41.9	8.09	-	-	0.790																
														LCGSGFQSIVNGCQEICVK	2	1	1	N-Term(iTRAQ8plex); C2(Methylthio); C13(Methylthio); C17(Methylthio); [illegible]	-	-	1.051	4.51	43	0	2731.30698	3	911.1071777	1.495860097	60.92079167
														ITAHLVHELR	2	1	1	N-Term(iTRAQ8plex)	-	-	0.594	2.87	36	0	1492.90297	4	373.9812012	8.576248903	25.43126
167614504	LAMB1	laminin subunit beta-1 precursor	NP_002282.2	1.12	1	1	6	1786	197.9	4.94	0.617	-	0.490																
														VESLSQVEVILQHSAADIAR	6	2	1	N-Term(iTRAQ8plex)	0.617	-	0.49	5.95	72	0	2469.3682	3	823.7942505	2.699846402	59.22800667
167860126	SERPINB5	serpin B5	NP_002630.2	4.00	1	1	2	375	42.1	6.05	0.452	0.454	0.629																
														DVPFGFQTVTSDVNK	2	1	1	N-Term(iTRAQ8plex); K15(iTRAQ8plex)	0.452	0.454	0.629	3.57	29	0	2262.21128	3	754.7419434	-5.036873431	29.14353333
169404009	SSR1	translocon-associated protein subunit alpha isoform 1 precursor	NP_003135.2	8.04	2	2	14	286	32.2	4.49	1.045	0.646	0.490																
														GTEDFIVESLDASFR	8	1	1	N-Term(iTRAQ8plex)	0.896	0.62	0.47	5.27	71	0	1990.0103	2	995.5087891	1.583009393	51.24040833
														FLVGFTNK	6	1	1	N-Term(iTRAQ8plex); K8(iTRAQ8plex)	1.154	0.713	0.568	4.03	35	0	1533.92539	3	511.9799805	0.291315506	53.57353333
169636418	MRPL38	39S ribosomal protein L38, mitochondrial	NP_115867.2	2.89	1	1	7	380	44.6	7.53	0.736	0.790	0.618																
														DLFHGATFVPR	7	1	1	N-Term(iTRAQ8plex)	0.736	0.79	0.618	3.52	44	0	1563.85398	3	521.9561768	-2.925002353	29.88852333
169790849	HCCS	cytochrome c-type heme lyase	NP_005324.3	6.34	1	1	7	268	30.6	6.68	0.773	1.020	0.539																
														WEALHAAECPCGPSLIR	7	1	1	N-Term(iTRAQ8plex); C9(Methylthio); C11(Methylthio)	0.773	1.02	0.539	3.67	34	0	2249.06412	3	750.3609009	1.763435546	51.871435
169881252	GTF2I	general transcription factor II-I isoform 4	NP_001509.3	6.90	3	3	14	957	107.9	7.94	1.044	1.780	2.090																
														DQSAVVVQGLPEGVAFK	2	6	1	N-Term(iTRAQ8plex); K17(iTRAQ8plex)	0.725	1.663	2.176	5.47	87	0	2352.34697	2	1176.677124	3.647475045	34.851955
														GPVTIPYPLFQSHVEDLYVEGLPEGIPFR	4	5	1	N-Term(iTRAQ8plex)	0.408	1.905	2.007	5.46	40	0	3573.89389	3	1191.969482	0.076307701	62.86922667
														FEAHPNDLYVEGLPENIPFR	8	5	1	N-Term(iTRAQ8plex)	1.27	1.689	1.753	4.91	55	0	2661.36032	3	887.791626	-0.453384798	44.84519167
170016077	ATP13A1	manganese-transporting ATPase 13A1	NP_065143.2	3.41	3	3	5	1204	132.9	8.13	0.810	0.742	0.771																
														WRPIASDEIVPGDIVSIGR	1	1	1	N-Term(iTRAQ8plex)	1.059	0.742	0.771	3.62	-	0	2384.31845	3	795.4443359	-2.343117166	41.65486
														GFQEDSEIR	2	1	1	N-Term(iTRAQ8plex)	0.734	0.525	0.551	3.33	28	0	1384.71135	2	692.859314	7.445602058	22.668915
														AEMVVPDFSELFK	2	1	1	N-Term(iTRAQ8plex); K13(iTRAQ8plex)	0.81	0.859	1.012	3.26	26	0	2120.14359	3	707.3860474	-5.78378758	47.57516167
170763533	ESRP1	epithelial splicing regulatory protein 1 isoform 5	NP_001116299.1	3.97	1	1	1	604	67.2	6.64	-	-	-																
														YGASQVEDMGNIILAMISEPYNHR	1	7	1	N-Term(iTRAQ8plex)	-	-	-	-	32	0	3012.4896	3	1004.834717	1.137212433	69.070165
														YIEIFR	2	3	2	N-Term(iTRAQ8plex)	-	-	-	-	28	0	1144.66175	3	382.2254333	-4.415715984	34.92836833
171846268	TSFM	elongation factor Ts, mitochondrial isoform 2 precursor	NP_005717.3	8.31	1	1	2	325	35.4	8.38	-	-	-																
														LGQHVVGMAPLSVGSLDDEPGGEAETK	2	2	1	N-Term(iTRAQ8plex); K27(iTRAQ8plex)	-	-	-	5.34	58	0	3301.7101	4	826.1829834	-2.864450831	36.837425
171906582	CCDC47	coiled-coil domain-containing protein 47 precursor	NP_064583.2	12.63	4	4	14	483	55.8	4.87	1.822	1.122	0.929																
														TLLFTFNVPGSGNTYPK	5	1	1	N-Term(iTRAQ8plex); K17(iTRAQ8plex)	1.836	1.122	0.777	4.45	48	0	2464.36393	2	1232.688599	0.105238107	43.41281167
														YGLPDSLAILSEMGEVTDGMMDTK	2	1	1	N-Term(iTRAQ8plex); K24(iTRAQ8plex)	2.039	-	-	4.09	45	0	3181.59262	3	1061.202393	-0.061860725	66.43593667
														LAQAWFNTHR	4	1	1	N-Term(iTRAQ8plex)	1.631	1.15	1.075	3.65	44	0	1547.8328	3	516.6157837	-3.665431944	29.862555
														RQDLLNVLAR	3	1	1	N-Term(iTRAQ8plex)	1.651	1.127	0.982	2.92	47	1	1501.90583	3	501.3067932	-3.878346582	26.95784167
183076548	HIST2H3D	histone H3.2	NP_001116847.1	51.47	2	6	74	136	15.4	11.27	0.549	0.515	0.448																
														FQSSAVMALQEASEAYLVGLFEDTNLCAIHAK	4	1	1	N-Term(iTRAQ8plex); M7(Oxidation); C27(Methylthio); K32(iTRAQ8plex)	-	-	-	8.69	44	0	4127.05581	5	823.0192261	-3.156951427	72.668735
														FQSSAVMALQEASEAYLVGLFEDTNLCAIHAK	7	1	1	N-Term(iTRAQ8plex); C27(Methylthio); K32(iTRAQ8plex)	-	-	-	7.28	50	0	4111.0519	5	823.0192261	-3.156951427	72.668735
														SAPATGGVK	5	3	1	N-Term(iTRAQ8plex); K9(iTRAQ8plex)	0.549	0.515	0.448	3.92	36	0	1395.84381	2	698.4259033	2.091006958	10.08947333
														EIAQDFK	26	4	2	N-Term(iTRAQ8plex); K7(iTRAQ8plex)	-	-	-	3.43	35	0	1458.84208	2	729.9260864	2.484795726	28.75155667
														STELLIR	30	5	2	N-Term(iTRAQ8plex)	-	-	-	3.14	41	0	1135.69231	2	568.3497925	-5.752402314	28.27890167
														VTIMPK	1	5	2	N-Term(iTRAQ8plex); M4(Oxidation); K6(iTRAQ8plex)	-	-	-	-	31	0	1312.81675	3	438.2770996	3.701413881	23.42421
														YRPGTVALR	1	5	2	N-Term(iTRAQ8plex)	-	-	-	-	26	0	1336.79551	3	446.2700195	-3.580170812	20.97728333
183227678	PARK7	protein deglycase DJ-1	NP_001116849.1	51.32	7	7	17	189	19.9	6.79	1.037	2.632	2.965																
														GAEEMETVIPVDVMR	2	1	1	N-Term(iTRAQ8plex)	1.037	2.573	2.965	5.66	59	0	1980.00371	2	990.5054932	-2.389688389	43.29247667

														GLIAAICAGPTALLAHEIGFGSK	2	1	1	N-Term(iTRAQ8plex); C7(Methylthio); K23(iTRAQ8plex)	-	-	-	5.38	45	0	2864.60097	4	716.9057007	0.775170572	69.90399667
														GPGTSFEFALAIVEALNGK	4	1	1	N-Term(iTRAQ8plex); K19(iTRAQ8plex)	0.948	2.334	4.149	5.32	82	0	2529.41655	2	1265.211914	-0.328057868	64.66831333
														KGLIAAICAGPTALLAHEIGFGSK	2	1	1	N-Term(iTRAQ8plex); K1(iTRAQ8plex); C8(Methylthio); K24(iTRAQ8plex)	0.763	4.396	2.482	4.55	56	1	3296.88095	3	1099.631836	-5.498450732	68.07241
														VTVAGLAGKDPVQCSR	2	1	1	N-Term(iTRAQ8plex); K9(iTRAQ8plex); C14(Methylthio)	1.862	3.212	2.971	4.3	-	1	2255.22548	4	564.5618286	-9.13333368	30.97432
														DVVICPDASLEDAKK	1	1	1	N-Term(iTRAQ8plex); C5(Methylthio); K14(iTRAQ8plex); K15(iTRAQ8plex)	2.291	2.632	4.949	3.93	-	1	2561.38881	4	641.1026611	-7.58481049	31.68403167
														VTTHPLAK	2	1	1	N-Term(iTRAQ8plex); K8(iTRAQ8plex)	1.082	2.122	2.618	3.18	36	0	1474.90581	2	737.956543	-9.744339204	18.67672833
														GAEEMETVIPVDVMR	2	1	1	N-Term(iTRAQ8plex); M14(Oxidation)	0.91	1.395	1.717	2.82	44	0	1996.00334	2	990.5054932	-2.389688389	43.29247667
183227688	ADSL	adenylosuccinate lyase isoform b	NP_001116850.1	10.59	3	3	4	425	48.3	7.78	0.964	2.977	2.057																
														YASPEMCFVFSDR	2	7	1	N-Term(iTRAQ8plex); C7(Methylthio)	0.936	6.148	3.675	4.56	43	0	1901.86699	2	951.4371338	6.929326913	47.56519167
														AFIITGQTYTR	1	10	1	N-Term(iTRAQ8plex)	0.993	1.441	1.152	3.55	-	0	1574.89067	2	787.9489746	3.975703748	31.07615667
														ASLPTLGFTHFQPAQLTTVGK	1	8	1	N-Term(iTRAQ8plex); K21(iTRAQ8plex)	-	-	-	3.54	-	0	2822.58584	4	706.4019165	-5.918390209	43.20208333
183603931	PPP6C	serine/threonine-protein phosphatase 6 catalytic subunit isoform c	NP_001116841.1	4.59	1	1	1	283	32.5	6.04	0.795	1.188	1.042																
														LVTVWSAPNYCYR	1	4	1	N-Term(iTRAQ8plex); C11(Methylthio)	0.795	1.188	1.042	-	37	0	1921.96379	2	961.4855347	1.644368094	50.20148
186928847	UTP4	U3 small nucleolar RNA-associated protein 4 homolog isoform 1	NP_116219.2	6.56	4	4	8	686	76.8	8.85	1.398	0.805	0.707																
														ATEALCWAEGQR	4	1	1	N-Term(iTRAQ8plex); C6(Methylthio)	1.567	0.698	0.789	4.57	39	0	1684.81047	2	842.9088745	0.925981565	37.597225
														IYKPLLFMDLLDER	2	3	1	N-Term(iTRAQ8plex); K3(iTRAQ8plex)	1.649	0.882	0.57	3.72	26	0	2374.3685	3	792.1276855	0.838998084	61.88234167
														NADHLLHLK	1	3	1	N-Term(iTRAQ8plex); K9(iTRAQ8plex)	1.398	0.222	0.645	3.14	-	0	1668.99575	4	418.0043945	-2.883803032	20.111775
														FFNYVPSGIR	1	1	1	N-Term(iTRAQ8plex)	1.364	0.997	0.941	2.76	-	0	1503.81902	2	752.413147	-4.757204561	43.69455
186928852	MRPS7	28S ribosomal protein S7, mitochondrial	NP_057055.2	11.16	2	2	4	242	28.1	9.99	1.112	1.045	0.959																
														YHAASAEEQATIER	2	1	1	N-Term(iTRAQ8plex)	0.88	1.341	1.232	4.29	48	0	1879.93631	3	627.3169556	-4.72242103	15.40698167
														NCEPMIGLVPILK	2	1	1	N-Term(iTRAQ8plex); C2(Methylthio); K13(iTRAQ8plex)	1.405	0.814	0.747	3.59	45	0	2081.16387	2	1041.085571	-6.879007516	43.940705
186928854	MRPS31	28S ribosomal protein S31, mitochondrial	NP_005821.2	12.41	2	2	3	395	45.3	9.29	0.547	0.282	0.557																
														QLATVNEQPLQNGFEELIQWTK	2	1	1	N-Term(iTRAQ8plex); K22(iTRAQ8plex)	0.547	0.282	0.557	5.92	96	0	3194.74143	3	1065.585327	3.367062892	57.66462333
														LWEFPINNEAGFDDDGSEFHEHIFLEK	1	1	1	N-Term(iTRAQ8plex); K27(iTRAQ8plex)	-	-	-	5.37	-	0	3843.87856	4	961.7250977	0.87397272	51.31256167
186972143	TPP2	tripeptidyl-peptidase 2	NP_003282.2	0.88	1	1	2	1249	138.3	6.32	0.745	1.280	1.139																
														LKDLPFIVSHR	2	4	1	N-Term(iTRAQ8plex); K2(iTRAQ8plex)	0.745	1.28	1.139	4.34	30	1	1933.17275	4	484.048645	-6.006241229	33.30724667
187281616	NDUFS7	NADH dehydrogenase [ubiquinone] iron-sulfur protein 7, mitochondrial	NP_077718.3	14.08	3	3	8	213	23.5	9.99	0.922	0.539	0.463																
														QSDVMIVAGTLTNK	2	2	1	N-Term(iTRAQ8plex); K14(iTRAQ8plex)	0.632	0.182	0.73	3.58	41	0	2085.16843	3	695.7276611	-7.203807302	30.31304167
														LDDLVNWAR	5	3	1	N-Term(iTRAQ8plex)	0.922	0.539	0.432	3.26	40	0	1405.76933	2	703.3883057	-3.426303132	37.75041
														RLQIWYR	1	1	1	N-Term(iTRAQ8plex)	1.139	0.742	0.656	-	35	1	1338.78943	3	446.9346619	-4.010305256	23.46280333
187608744	CNBP	cellular nucleic acid-binding protein isoform 5	NP_001120667.1	22.22	3	3	6	171	18.9	7.58	1.069	3.667	2.753																
														DCDLQEDEACYNCGR	2	2	1	N-Term(iTRAQ8plex); C2(Methylthio); C10(Methylthio); C13(Methylthio)	1.6	4.131	4.021	4.5	58	0	2175.80498	2	1088.406128	7.530518374	37.35149833
														EQCCYNCGKPGHLAR	2	6	1	N-Term(iTRAQ8plex); C3(Methylthio); C4(Methylthio); C7(Methylthio); K9(iTRAQ8plex)	1.069	3.667	2.753	3.9	29	0	2425.08266	4	607.026123	-6.582040583	27.88845667
														TSEVNCYR	2	6	1	N-Term(iTRAQ8plex); C6(Methylthio)	0.961	1.629	1.557	2.69	17	0	1321.60991	2	661.3085938	-6.281293695	16.69317167
187960037	CBX1	chromobox protein homolog 1	NP_001120700.1	15.14	1	2	6	185	21.4	4.93	0.775	0.989	1.181																
														CPQVVISFYEER	2	1	1	N-Term(iTRAQ8plex); C1(Methylthio)	0.775	0.989	1.181	3.83	41	0	1819.91472	2	910.4609985	6.735563032	46.62991667
														IIGATDSSGELMFLMK	4	2	2	N-Term(iTRAQ8plex); K16(iTRAQ8plex)	-	-	-	5.54	74	0	2321.27349	2	1161.140381	1.250950725	49.48972167
188035908	CBX5	chromobox protein homolog 5	NP_001120793.1	21.47	2	3	11	191	22.2	5.86	0.298	1.147	0.497																
														IIGATDSCGDLMFLMK	4	1	1	N-Term(iTRAQ8plex); C8(Methylthio); K16(iTRAQ8plex)	0.279	1.161	0.479	5.47	64	0	2369.23917	3	790.4179077	8.170384306	61.26423
														NLDCPELISEFMK	2	1	1	N-Term(iTRAQ8plex); C4(Methylthio); K13(iTRAQ8plex)	0.54	1.144	0.536	2.7	38	0	2193.13042	2	1097.068848	4.088399132	58.42843833
														CPQIVIAFYEER	5	2	2	N-Term(iTRAQ8plex); C1(Methylthio)	-	-	-	4.28	54	0	1817.91838	2	909.4628296	-2.651241309	57.362115
188528628	PNPT1	polyribonucleotide nucleotidyltransferase 1, mitochondrial precursor	NP_149100.2	8.56	5	5	13	783	85.9	7.77	1.398	0.894	0.750																
														LLTDILGIEDYNGDMDFK	2	2	1	N-Term(iTRAQ8plex); K18(iTRAQ8plex)	1.372	1.072	0.709	6.64	75	0	2680.41941	3	894.1446533	7.213576685	61.78207667
														FPEADPYEIIESFNVVAK	4	2	1	N-Term(iTRAQ8plex); K18(iTRAQ8plex)	1.781	0.818	0.779	6.14	48	0	2676.44693	3	892.8204956	3.277579053	58.95607167
														LFTPSPEIVK	2	2	1	N-Term(iTRAQ8plex); K10(iTRAQ8plex)	1.233	0.703	0.477	3.68	24	0	1739.05229	2	870.0297852	-2.338979406	26.39596833
														KVLQSPATTVVR	3	2	1	N-Term(iTRAQ8plex); K1(iTRAQ8plex)	1.336	0.897	0.761	3.43	21	1	1907.18863	3	636.401062	-0.633662986	20.92562333
														AVAVDLGNR	2	1	1	N-Term(iTRAQ8plex)	1.398	0.894	0.733	2.71	26	0	1218.71355	2	609.8604126	2.23783129	24.10790667
189083772	GSN	gelsolin isoform b	NP_001121134.1	1.50	1	1	6	731	80.6	5.85	0.386	0.334	0.778																
														HVVPNEVVVQR	6	9	1	N-Term(iTRAQ8plex)	0.386	0.334	0.778	3.96	41	0	1579.91435	3	527.3106079	-3.127264714	16.35399667
189083844	CTSC	dipeptidyl peptidase 1 isoform a preproprotein	NP_001805.3	21.81	9	9	51	463	51.8	6.99	0.361	0.543	0.387																
														YAQDFGLVEEACFPYTGTDSPCK	11	1	1	N-Term(iTRAQ8plex); C12(Methylthio); C22(Methylthio); K23(iTRAQ8plex)	0.195	0.331	0.292	8.69	85	0	3241.49009	2	1621.259399	9.381089013	62.29891167
														GTDECAIESIAVAATPIPK	2	1	1	N-Term(iTRAQ8plex); C5(Methylthio); K19(iTRAQ8plex)	0.226	0.291	0.243	5.88	85	0	2540.37993	2	1270.693604	9.37974315	42.841655
														NSWGTGWGENGYFR	2	1	1	N-Term(iTRAQ8plex)	0.371	0.32	0.221	3.86	60	0	1934.8991	2	967.953186	-4.99036343	53.0955
														NWACFTGK	4	1	1	N-Term(iTRAQ8plex); C4(Methylthio); K8(iTRAQ8plex)	0.499	0.558	0.518	3.77	28	0	1580.82053	3	527.6116943	1.991027921	34.03946833
														ILHLPTSWDWR	17	1	1	N-Term(iTRAQ8plex)	0.399	0.601	0.498	3.68	38	0	1727.94889	3	576.65448	-2.676357287	46.53138333
														NVHGINFVSPVR	8	1	1	N-Term(iTRAQ8plex)	0.432	0.622	0.438	3.66	48	0	1642.92551	3	548.3133545	-4.62654782	22.928625
														KVVVYLQK	3	3	1	N-Term(iTRAQ8plex); K1(iTRAQ8plex); K8(iTRAQ8plex)	0.296	0.246	0.193	3.43	23	1	1889.23357	2	945.1204224	-0.806249404	21.95770667
														WFAFFK	2	3	1	N-Term(iTRAQ8plex); K6(iTRAQ8plex)	0.135	0.182	0.158	3.02	-	0	1453.85208	3	485.2888794	4.714120277	54.95667167
														VVVYLQK	1	3	1	N-Term(iTRAQ8plex); K7(iTRAQ8plex)	1.517	0.543	0.239	2.87	-	0	1456.93531	3	486.3166199	0.374120346	38.25277833
														YAQDFGLVEEACFPYTGTDSPCK	1	1	1	N-Term(iTRAQ8plex); Y1(iTRAQ8plex); C12(Methylthio); C22(Methylthio); K23(iTRAQ8plex)	-	-	-	-	26	0	3545.69448	2	1621.259399	9.381089013	62.29891167
189163485	CTSA	lysosomal protective protein isoform b preproprotein	NP_001121167.1	13.33	4	4	7	480	54.4	6.61	0.856	0.805	1.654																
														GAGHMVPTDKPLAAFTMFSR	2	3	1	N-Term(iTRAQ8plex); K10(iTRAQ8plex)	-	0.613	2.065	4.34	-	0	2742.4601	4	686.3704834	-2.905269702	39.20194
														YGDSGEQIAGFVK	2	3	1	N-Term(iTRAQ8plex); K13(iTRAQ8plex)	0.549	0.312	0.882	4.02	38	0	1979.05368	3	660.3560791	-7.967062965	39.597075
														EFSHIAFLTIK	2	3	1	N-Term(iTRAQ8plex); K11(iTRAQ8plex)	0.856	1.058	1.459	3.9	24	0	1914.12082	3	638.711792	-5.27582863	39.92991833
														CNFYDNKDLECVTNLQEVAR	1	3	1	N-Term(iTRAQ8plex); C1(Methylthio); K7(iTRAQ8plex); C11(Methylthio)	1.12	1.558	1.877	2.79	-	1	3074.46201	3	1025.492188	-1.448725008	48.33715833
189181666	HEXA	beta-hexosaminidase subunit alpha isoform 2 preproprotein	NP_000511.2	4.16	2	2	4	529	60.7	5.16	0.515	0.470	1.067																
														LTSDLTFAYER	2	2	1	N-Term(iTRAQ8plex)	0.491	0.386	1.06	4.51	38	0	1619.86064	2	810.43396	1.470498024	35.60871
														ALLSAPWYLNR	2	2	1	N-Term(iTRAQ8plex)	0.541	0.573	1.074	2.9	42	0	1607.91423	2	804.4607544	-4.296952412	42.88623833
189458817	TFRC	transferrin receptor protein 1 isoform 1	NP_003225.2	21.84	13	13	66	760	84.8	6.61	2.453	1.122	1.461																
														LVYLVENPGGYVAYSK	8	2	1	N-Term(iTRAQ8plex); K16(iTRAQ8plex)	3.135	0.979	1.37	6.5	79	0	2380.34453	2	1190.675903	3.045240104	46.05240833
														SAFSNLFGGEPLSYTR	12	1	1	N-Term(iTRAQ8plex)	1.623	1.022	0.804	6	62	0	2050.05303	2	1025.530151	-0.841532196	46.70764333
														VSASPLLYTLIEK	11	3	1	N-Term(iTRAQ8plex); K13(iTRAQ8plex)	3.132	1.194	1.579	5.58	61	0	2042.21648	2	1021.611877	-9.452003445	40.15430167
														LFGNMEGDCPSDWK	4	3	1	N-Term(iTRAQ8plex); C9(Methylthio); K14(iTRAQ8plex)	4.939	1.639	2.329	5.16	46	0	2253.04685	3	751.6871338	-5.802889906	70.01564
														VEYHFLSPYVSPK	4	3	1	N-Term(iTRAQ8plex); K13(iTRAQ8plex)	1.228	0.989	1.103	4.69	32	0	2174.20566	3	725.4067383	-2.27655844	31.58505667
														AFTYINLDK	6	3	1	N-Term(iTRAQ8plex); K9(iTRAQ8plex)	2.952	1.132	1.503	4.43	58	0	1692.96843	2	846.987854	-5.705902135	35.3376
														LTHDVELNLDYER	5	3	1	N-Term(iTRAQ8plex)	1.303	1.143	1.27	4.27	46	0	1920.99094	3	641.0018311	-3.098081211	28.78063167
														GFVEPDHYVVVGAQR	4	3	1	N-Term(iTRAQ8plex)	1.949	1.075	0.937	4.25	44	0	1977.04245	3	659.6856689	-3.620414953	25.65109333
														ILNIFGVIK	2	3	1	N-Term(iTRAQ8plex); K9(iTRAQ8plex)	3.308	0.99	1.461	3.84	50	0	1625.04766	2	813.0274658	-8.243622477	77.55153833
														LAVDEEENADNNTK	2	1	1	N-Term(iTRAQ8plex); K14(iTRAQ8plex)	2.795	1.757	2.041	3.74	49	0	2170.09696	3	724.0371704	-5.287007794	15.68406
														SSGLPNIPVQTISR	3	3	1	N-Term(iTRAQ8plex)	2.219	1.103	1.296	3.38	48	0	1773.01225	2	887.0097656	-2.812541192	31.83965333
														YNSQLLSFVR	2	3	1	N-Term(iTRAQ8plex)	2.96	1.342	1.464	2.84	30	0	1530.84734	2	765.9273071	-7.089970319	30.810905
														LLNENSYVPR	2	2	1	N-Term(iTRAQ8plex)	1.714	0.739	0.779	-	49	0	1508.83245	2	754.9198608	-3.324584071	23.0781
														LTHDVELNLDYER	1	3	1	N-Term(iTRAQ8plex); Y11(iTRAQ8plex)	2.237	1.397	1.533	-	39	0	2225.19423	3	641.0018311	-3.098081211	28.78063167
189571677	NUDCD1	nudC domain-containing protein 1 isoform 1	NP_116258.2	3.60	1	1	2	583	66.7	5.11	-	1.855	2.372																
														LSLEPLPCYQLELDAAVAEVK	2	1	1	N-Term(iTRAQ8plex); C8(Methylthio); K21(iTRAQ8plex)	-	1.855	2.372	3.69	37	0	2955.60129	3	985.8719482	-0.64989261	65.61904333
190014588	PSIP1	PC4 and SFRS1-interacting protein isoform 2	NP_001121689.1	12.45	4	4	7	530	60.1	9.13	2.893	2.956	1.703																
														QPCPSESDIITEEDKSK	2	4	1	N-Term(iTRAQ8plex); C3(Methylthio); K15(iTRAQ8plex); K17(iTRAQ8plex)	-	-	-	5.65	43	1	2864.45327	4	716.8687744	-8.809542831	26.89347833
														GFNEGLWEIDNNPK	2	4	1	N-Term(iTRAQ8plex); K14(iTRAQ8plex)	0.763	0.498	0.331	3.9	41	0	2241.16495	3	747.7265015	-4.957312697	53.68514333
														VDEVPDGAVKPPTNK	1	4	1	N-Term(iTRAQ8plex); K10(iTRAQ8plex); K15(iTRAQ8plex)	10.973	17.553	8.77	3.82	-	0	2478.41965	3	826.8114014	-5.439130711	22.03815167
														CIEALDELASLQVTMQQAQK	2	1	1	N-Term(iTRAQ8plex); C1(Methylthio); K20(iTRAQ8plex)	-	-	-	3.54	42	0	2873.52701	3	958.513855	8.292732697	62.49129167
190014601	JAGN1	protein jagunal homolog 1	NP_115881.3	6.56	1	1	2	183	21.1	9.73	1.068	0.661	0.704																
														AAGTDGSDFQHR	2	1	1	N-Term(iTRAQ8plex)	1.068	0.661	0.704	4.16	30	0	1565.75589	3	522.5901489	-3.274950251	12.97661333
190194386	TM9SF3	transmembrane 9 superfamily member 3 precursor	NP_064508.3	4.92	3	3	11	589	67.8	7.21	1.083	0.899	0.917																
														YLDPSFFQHR	5	3	1	N-Term(iTRAQ8plex)	1.188	0.918	0.956	3.94	42	0	1613.83097	3	538.6151733	-4.228776501	32.99311833
														YFSLPFCVGSK	4	3	1	N-Term(iTRAQ8plex); C7(Methylthio); K11(iTRAQ8plex)	1.08	0.914	0.892	3.94	32	0	1902.00627	2	951.5067749	-2.691429248	75.05310167
														DAFVYAIK	2	3	1	N-Term(iTRAQ8plex); K8(iTRAQ8plex)	1.035	0.813	0.736	3.69	23	0	1534.91031	3	512.3082886	0.886809246	42.618245
190341022	TSPAN14	tetraspanin-14 isoform 2	NP_001121781.1	7.48	1	1	2	147	16.9	7.84	1.326	0.708	1.536																
														VVNTQCGYDVR	2	4	1	N-Term(iTRAQ8plex); C6(Methylthio)	1.326	0.708	1.536	4.29	47	0	1603.78813	2	802.3977051	0.443472698	27.85443
190358517	RAB11B	ras-related protein Rab-11B	NP_004209.2	25.69	6	6	19	218	24.5	5.94	1.060	0.639	0.949																
														VVLIGDSGVGK	4	3	1	N-Term(iTRAQ8plex); K11(iTRAQ8plex)	1.213	0.553	1.061	4.5	45	0	1652.01377	3	551.3427734	-3.967423051	23.16919167
														STIGVEFATR	7	3	1	N-Term(iTRAQ8plex)	0.998	0.694	0.791	4.35	57	0	1384.76933	3	462.2638855	2.377237559	27.84087167
														NEFNLESK	2	3	1	N-Term(iTRAQ8plex); K8(iTRAQ8plex)	1.27	0.827	1.265	3.55	38	0	1588.86516	2	794.9362183	-8.780730369	21.296075
														NILTEIYR	3	1	1	N-Term(iTRAQ8plex)	1.046	0.606	0.966	3.27	32	0	1325.76824	2	663.3877563	-3.639568784	36.73267333
														AQIWDTAGQER	2	3	1	N-Term(iTRAQ8plex)	0.954	0.566	0.75	3.22	22	0	1578.81206	2	789.909668	-3.64323446	19.07688
														AITSAYYR	1	4	1	N-Term(iTRAQ8plex)	1.384	1.2	1.127	-	22	0	1248.68486	2	624.8460693	-3.298869275	18.70678167
190885499	COX5A	cytochrome c oxidase subunit 5A, mitochondrial precursor	NP_004246.2	58.00	8	8	34	150	16.8	6.79	1.572	0.779	0.662																
														EIYPYVIQELRPTLNELGISTPEELGLDKV	7	1	1	N-Term(iTRAQ8plex); K29(iTRAQ8plex)	1.269	1.173	0.716	5.94	23	1	4037.20717	4	1010.058594	-4.51949948	84.124925
														WVTYFNKPDIDAWELR	6	1	1	N-Term(iTRAQ8plex); K7(iTRAQ8plex)	1.653	0.741	0.595	5.75	25	0	2661.42837	4	666.1107178	-2.836862764	53.457835
														GINTLVTYDMVPEPK	7	1	1	N-Term(iTRAQ8plex); K15(iTRAQ8plex)	1.41	0.787	0.865	4.69	71	0	2285.25345	2	1143.137695	0.408138106	37.134925
														WVTYFNKPDIDAWELRK	2	1	1	N-Term(iTRAQ8plex); K7(iTRAQ8plex); K17(iTRAQ8plex)	2.49	1.036	0.769	4.47	-	1	3093.72785	5	619.5513916	-0.345797122	46.174675
														ILEVVKDK	5	1	1	N-Term(iTRAQ8plex); K6(iTRAQ8plex); K8(iTRAQ8plex)	1.743	0.816	0.679	3.64	26	1	1856.1957	2	928.6032104	0.405656063	28.79271833
														RLNDFASTVR	2	1	1	N-Term(iTRAQ8plex)	1.552	0.988	0.975	2.89	33	1	1482.82317	2	741.9152222	-6.672126709	19.02962333
														IIDAALR	4	1	1	N-Term(iTRAQ8plex)	1.572	0.672	0.547	-	34	0	1075.67583	2	538.3415527	-1.759313726	32.46363833
														LNDFASTVR	1	1	1	N-Term(iTRAQ8plex)	1.067	0.661	0.617	-	33	0	1326.72917	2	663.8682251	-2.086126349	29.49235333
193083120	TOMM40	mitochondrial import receptor subunit TOM40 homolog	NP_001122388.1	40.17	11	11	52	361	37.9	7.25	1.388	0.733	0.756																

														QLSPTEAFPVLVGDMDNS GSLNAQVIHQLGPGLR	5	2	1	N-Term(iTRAQ8plex)	1.193	0.764	-	7.08	66	0	3865.02719	3	1289.017578	4.152565705	52.88666
														TPGAATASASGAAEDGA CGCLPNPGTFEECHR	4	2	1	N-Term(iTRAQ8plex); C18(Methylthio); C20(Methylthio); C30(Methylthio)	1.234	0.701	0.743	6.18	101	0	3490.46701	3	1164.160522	2.945116734	39.45613
														KLPPLPLTLALGAFLNHR	10	1	1	N-Term(iTRAQ8plex); K1(iTRAQ8plex)	0.972	-	0.714	5.14	48	1	2579.60586	4	645.6569214	1.87019535	60.65221
														LPPLPLTLALGAFLNHR	2	1	1	N-Term(iTRAQ8plex)	1.314	-	0.919	5.11	61	0	2147.30765	3	716.4407349	3.236629758	67.76538667
														LPPLPLTLALGAFLNHRK	6	1	1	N-Term(iTRAQ8plex); K18(iTRAQ8plex)	-	-	-	4.85	30	1	2579.59218	4	645.6535034	-3.429808859	63.81121667
														MQDTSVSFGYQLDLPK	1	2	1	N-Term(iTRAQ8plex); K16(iTRAQ8plex)	1.248	0.818	0.817	4.58	-	0	2437.27274	3	818.4302979	-3.238932028	36.80220333
														ELFPIQMEGVK	3	2	1	N-Term(iTRAQ8plex); K11(iTRAQ8plex)	1.698	0.793	0.804	4.57	50	0	1899.07695	2	950.0421143	-5.307528147	31.46052833
														FVNWQVDGEYR	10	2	1	N-Term(iTRAQ8plex)	1.446	0.637	0.756	4.24	53	0	1716.85747	2	858.9393921	3.935215686	34.52057
														MQDTSVSFGYQLDLPK	2	2	1	N-Term(iTRAQ8plex); M1(Oxidation); K16(iTRAQ8plex)	1.244	0.73	1.01	3.96	30	0	2453.27634	3	818.4302979	-3.238932028	36.80220333
														RPGEEGTVMSLAGK	3	2	1	N-Term(iTRAQ8plex); K14(iTRAQ8plex)	1.061	0.54	0.565	3.85	41	0	2040.12908	2	1020.568176	-3.811220098	21.76791667
														CKELFPIQMEGVK	1	2	1	N-Term(iTRAQ8plex); C1(Methylthio); K2(iTRAQ8plex); K13(iTRAQ8plex)	2.27	0.792	0.786	3.71	-	1	2480.39701	3	827.47052	5.13406062	43.233665
														RPGEEGTVMSLAGK	1	2	1	N-Term(iTRAQ8plex); M9(Oxidation); K14(iTRAQ8plex)	1.928	0.845	0.871	3.31	-	0	2056.12442	2	1020.568176	-3.811220098	21.76791667
														ANLLFK	4	2	1	N-Term(iTRAQ8plex); K6(iTRAQ8plex)	1.987	0.756	0.682	2.74	34	0	1313.84323	3	438.6192627	2.354570055	25.811895
193211480	SKIV2L2	superkiller viralicidic activity 2-like 2	NP_056175.3	5.09	4	4	7	1042	117.7	6.52	1.224	0.776	1.193																
														DVDFEGTDEPIFGK	2	1	1	N-Term(iTRAQ8plex); K14(iTRAQ8plex)	1.501	0.737	1.193	6.03	79	0	2177.13384	2	1089.070557	5.298416228	34.59929333
														QFAEWICHLHK	1	1	1	N-Term(iTRAQ8plex); C7(Methylthio); K11(iTRAQ8plex)	1.223	-	1.834	3.98	-	0	2066.07607	4	517.2744751	-7.924098057	44.98229333
														EYPFILDAFQR	2	1	1	N-Term(iTRAQ8plex)	1.224	0.817	0.936	3.35	34	0	1702.92192	2	851.9645996	6.624671748	46.31417667
														IEESITEDLSLADLMPR	2	1	1	N-Term(iTRAQ8plex)	-	-	-	3.1	44	0	2236.17221	3	746.0622559	1.660439255	64.77913667
193211614	NSDHL	sterol-4-alpha-carboxylate 3-dehydrogenase, decarboxylating	NP_001123237.1	25.47	5	5	26	373	41.9	8.06	0.601	0.256	0.352																
														NLVDFTFVENVVHGHILA AEQLSR	16	2	1	N-Term(iTRAQ8plex)	-	-	-	7.64	34	0	3012.62197	4	753.9127197	2.687926764	60.35749
														GVNTVFHCASPPPSSNNK	2	2	1	N-Term(iTRAQ8plex); C8(Methylthio); K18(iTRAQ8plex)	0.639	0.607	0.609	4.66	31	0	2510.2703	3	837.4282837	-1.50361329	25.374345
														AVLGANDPEKNFLTTAIR PHGIFGPR	1	2	1	N-Term(iTRAQ8plex); K10(iTRAQ8plex)	-	-	-	4.58	-	1	3399.90308	5	680.786438	-2.349440086	42.35748167
														AFHITNDEPIPFWTFLSR	2	2	1	N-Term(iTRAQ8plex)	-	-	-	3.43	30	0	2495.30216	3	832.4389038	-0.157545738	54.20429
														FFLGDLCSR	5	2	1	N-Term(iTRAQ8plex); C7(Methylthio)	0.565	0.237	0.343	3.06	20	0	1407.70183	2	704.3545532	-3.432882295	74.70764833
193794814	ALDOA	fructose-bisphosphate aldolase A isoform 1	NP_001121089.1	52.47	12	15	237	364	39.4	8.09	1.130	3.949	3.057																
														IGEHTPSALAIMENANVL AR	15	3	1	N-Term(iTRAQ8plex); M12(Oxidation)	0.784	1.729	1.402	7.28	86	0	2427.28818	2	1206.156372	1.470664199	44.6667
														IGEHTPSALAIMENANVL AR	69	3	1	N-Term(iTRAQ8plex)	1.062	2.473	1.956	6.16	121	0	2411.28952	2	1206.156372	1.470664199	44.6667
														GILAADESTGSIAK	6	3	1	N-Term(iTRAQ8plex); K14(iTRAQ8plex)	1.586	13.75	7.915	6.08	70	0	1941.09819	2	971.0527344	-6.768936292	33.95788667
														VNPCIGGVILFHETLYQK	2	3	1	N-Term(iTRAQ8plex); C4(Methylthio); K18(iTRAQ8plex)	-	-	-	5.81	41	0	2685.46938	4	672.1228027	-0.868094984	56.32976167
														FSHEEIAMATVTALR	11	2	1	N-Term(iTRAQ8plex); M8(Oxidation)	0.687	1.215	1.36	5.05	53	0	1996.04056	2	990.5297241	-0.24730551	36.35476667
														ALANSLACQGK	7	3	1	N-Term(iTRAQ8plex); C8(Methylthio); K11(iTRAQ8plex)	1.22	2.593	1.639	4.98	71	0	1729.93877	2	865.4730225	-9.355243331	23.41034667
														FSHEEIAMATVTALR	54	2	1	N-Term(iTRAQ8plex)	1.119	3.929	3.187	4.68	69	0	1980.04888	2	990.5297241	-0.24730551	36.35476667
														ELSDIAHR	14	3	1	N-Term(iTRAQ8plex)	0.878	3.033	2.198	4.63	62	0	1244.68357	3	415.56604	-5.225772225	21.361105
														LQSIGTENTEENRR	8	3	1	N-Term(iTRAQ8plex)	1.482	8.941	5.814	4.32	34	1	1951.00713	2	976.0072021	-3.87165327	17.69001167
														ADDGRPFPQVIK	6	3	1	N-Term(iTRAQ8plex); K12(iTRAQ8plex)	1.312	9.827	6.648	3.98	-	0	1951.10991	2	976.0585938	-6.283818216	24.04457833
														CPLLKPWALTFSYGR	14	3	1	N-Term(iTRAQ8plex); C1(Methylthio); K5(iTRAQ8plex)	1.098	16.442	10.965	3.95	51	0	2406.32891	2	1203.668091	0.096711894	59.67601333
														QLLLTADDR	5	3	1	N-Term(iTRAQ8plex)	1.446	4.795	3.715	3.55	36	0	1348.77849	2	674.8928833	3.46882872	28.20576333
														ALQASALK	2	3	1	N-Term(iTRAQ8plex); K8(iTRAQ8plex)	1.789	7.291	4.806	3.42	35	0	1409.88005	2	705.4436646	-9.638279805	15.338415
														VNPCIGGVILFHETLYQK	3	3	1	N-Term(iTRAQ8plex); C4(Methylthio); Y16(iTRAQ8plex); K18(iTRAQ8plex)	-	1.353	1.095	-	35	0	2989.70058	4	672.1228027	-0.868094984	56.32976167
														PYQYPALTPEQK	2	1	1	N-Term(iTRAQ8plex); K12(iTRAQ8plex)	1.837	13.653	9.467	-	32	0	2043.11955	2	1022.063416	-8.593165913	23.76701333
														YASICQQNGIVPIVEPEILP DGDHDLK	4	4	2	N-Term(iTRAQ8plex); C5(Methylthio); K27(iTRAQ8plex)	-	-	-	5.14	43	0	3617.88205	3	1206.632202	0.436967068	45.759295
														YASICQQNGIVPIVEPEILP DGDHDLKR	13	4	2	N-Term(iTRAQ8plex); C5(Methylthio); K27(iTRAQ8plex)	-	-	-	5.07	34	1	3773.97573	4	944.2493896	-1.553689444	48.23959167
														DGADFAK	2	4	2	N-Term(iTRAQ8plex); K7(iTRAQ8plex)	-	-	-	2.98	24	0	1331.72998	3	444.5815125	-8.693351222	16.29871167
193794826	CYB5R3	NADH-cytochrome b5 reductase 3 isoform 2	NP_001123291.1	43.17	8	8	22	278	31.6	7.58	0.552	0.753	0.538																
														STPAITLESPDIK	2	3	1	N-Term(iTRAQ8plex); K13(iTRAQ8plex)	0.482	0.392	0.262	5.8	52	0	1980.15312	2	990.5802002	2.905063531	29.94963
														MSQYLESMQIGDTIEFR	2	3	1	N-Term(iTRAQ8plex)	0.537	0.753	0.411	5.76	82	0	2352.16313	2	1176.585205	4.822044904	49.84205833
														FALPSPQHILGLPVGQHIY LSAR	2	3	1	N-Term(iTRAQ8plex)	0.565	0.782	0.698	4.53	25	0	2818.59574	3	940.2034302	-2.729736143	50.36621833
														AIMKDPDDHTVCHLLFA NQTEK	4	3	1	N-Term(iTRAQ8plex); K4(iTRAQ8plex); C12(Methylthio); K22(iTRAQ8plex)	-	1.109	-	4.44	-	1	3484.81	5	697.7678223	-1.568946906	39.22418667
														STPAITLESPDIKYPLR	2	3	1	N-Term(iTRAQ8plex); K13(iTRAQ8plex)	0.806	0.899	0.479	4.35	29	1	2509.43808	3	837.1508789	-4.211234108	33.45965833
														APEAWDYGQGFVNEEMI R	2	3	1	N-Term(iTRAQ8plex); M16(Oxidation)	1.1	1.173	0.688	4.29	40	0	2432.14341	3	811.3859863	-2.504196755	42.35484
														GPSGLLVYQGK	2	3	1	N-Term(iTRAQ8plex); K11(iTRAQ8plex)	0.529	0.476	0.35	3.77	34	0	1727.03069	2	864.0189819	-0.297651232	21.562915
														DILLRPELEELR	4	3	1	N-Term(iTRAQ8plex)	0.539	0.547	0.604	3.58	35	0	1800.04959	2	900.5288696	-1.582545738	37.76595667
														APEAWDYGQGFVNEEMI R	2	3	1	N-Term(iTRAQ8plex)	0.79	0.8	0.888	2.71	18	0	2416.15372	3	811.3859863	-2.504196755	42.35484
194097323	ECHS1	enoyl-CoA hydratase, mitochondrial	NP_004083.3	31.03	7	7	33	290	31.4	8.07	1.107	0.985	0.416																
														ICPVETLVEEAIQCAEK	14	1	1	N-Term(iTRAQ8plex); C2(Methylthio); C14(Methylthio); K17(iTRAQ8plex)	1.191	1.061	0.253	7.17	83	0	2575.31352	2	1288.1604	1.33997714	66.07758667
														EMQNLSFQDCYSSK	6	1	1	N-Term(iTRAQ8plex); C10(Methylthio); K14(iTRAQ8plex)	0.997	0.785	0.425	5.92	66	0	2334.08603	3	778.7001953	-7.047560488	59.35081833
														ALNALCDGLIDELNQALK	2	1	1	N-Term(iTRAQ8plex); C6(Methylthio); K18(iTRAQ8plex)	1.072	1.328	0.59	5.15	57	0	2568.39164	3	856.802063	-2.724111486	86.38555167
														SLAMEMVLTGDR	2	1	1	N-Term(iTRAQ8plex)	0.968	0.736	0.366	4.8	39	0	1626.85332	2	813.9302979	2.205351326	38.63760667
														AFAAGADIK	4	1	1	N-Term(iTRAQ8plex); K9(iTRAQ8plex)	1.177	0.877	0.237	3.76	38	0	1471.87627	2	736.4417725	2.27396959	31.056765
														NNTVGLIQLNRPK	2	1	1	N-Term(iTRAQ8plex); K13(iTRAQ8plex)	1.219	1.021	0.341	3.11	28	0	2075.2518	3	692.4221191	-1.333393914	21.91780167
														SLAMEMVLTGDR	1	1	1	N-Term(iTRAQ8plex); M6(Oxidation)	1.178	1.849	1.315	2.91	-	0	1642.85332	2	813.9302979	2.205351326	38.63760667
														QAGLVSK	1	1	1	N-Term(iTRAQ8plex); K7(iTRAQ8plex)	1.12	1.167	0.823	2.7	-	0	1310.82831	3	437.6142883	2.357623959	15.60500333
														SLAMEMVLTGDR	1	1	1	N-Term(iTRAQ8plex); M4(Oxidation); M6(Oxidation)	1.106	0.985	0.44	-	33	0	1658.83965	2	813.9302979	2.205351326	38.63760667
194097354	GSPT1	eukaryotic peptide chain release factor GTP-binding subunit ERF3A isoform 2	NP_001123478.1	12.26	5	5	12	636	68.5	5.33	0.902	1.850	1.723																
														GIEEEEILPGFILCDPNNLC HSGR	4	3	1	N-Term(iTRAQ8plex); C14(Methylthio); C20(Methylthio)	-	1.85	0.507	5.47	51	0	3051.44071	3	1017.81842	1.84328683	63.14746
														ETWYLSWALDTNQEER	2	4	1	N-Term(iTRAQ8plex)	0.949	3.953	2.385	5.34	75	0	2345.15434	2	1173.080811	8.167127164	47.809045
														LKGIEEEEILPGFILCDPNN LCHSGR	3	3	1	N-Term(iTRAQ8plex); K2(iTRAQ8plex); C16(Methylthio); C22(Methylthio)	-	-	-	4.58	37	1	3596.83027	4	899.9627075	2.645913421	60.53382167
														TFDAQIVIIEHK	2	3	1	N-Term(iTRAQ8plex); K12(iTRAQ8plex)	0.858	1.67	1.723	4.26	28	0	2022.17832	3	674.730957	-3.01825818	31.45910667
														QLNVNAKPFVPNVHAAEF VPSFLR	1	2	1	N-Term(iTRAQ8plex); K7(iTRAQ8plex)	-	-	-	3.27	-	0	3302.87246	4	826.4735718	3.071850187	47.46005333
194097365	FTSJ3	pre-rRNA processing protein FTSJ3	NP_060117.3	12.28	6	6	14	847	96.5	8.40	1.378	0.668	0.734																
														ASEIMVDDEELAQHPATT EDIR	4	1	1	N-Term(iTRAQ8plex)	1.402	0.698	0.553	6.48	52	0	2774.34317	3	925.4525757	-0.91463062	29.19202833
														ALLDLCAAPGGWLQVAA K	4	1	1	N-Term(iTRAQ8plex); C6(Methylthio); K18(iTRAQ8plex)	1.508	0.729	0.941	6.46	61	0	2451.38628	2	1226.196777	6.109101464	62.70640167
														ILDPEGLALGAVIASSK	2	1	1	N-Term(iTRAQ8plex); K17(iTRAQ8plex)	1.29	0.628	0.538	5.38	25	0	2262.34128	3	754.7852783	-5.171451875	57.08439667
														GHQLLEEVTQGDMSAAD TFLSDLPR	2	1	1	N-Term(iTRAQ8plex)	-	-	-	4.8	74	0	3034.5192	3	1012.177917	3.218925558	57.03021833
														KAEAVVNTVDISER	1	1	1	N-Term(iTRAQ8plex); K1(iTRAQ8plex)	0.707	0.664	0.893	3.41	-	1	2139.21439	3	713.742981	-4.030301473	26.13424833
														TSVTDFLR	1	1	1	N-Term(iTRAQ8plex)	1.594	0.668	0.767	-	31	0	1242.69634	2	621.8518066	-2.602919679	32.27514333
194239727	EEF1D	elongation factor 1-delta isoform 2	NP_001123527.1	24.20	5	5	19	281	31.1	5.01	1.522	3.206	1.924																
														SSILLDVKPWDDETDMAQ LEACVR	11	10	1	N-Term(iTRAQ8plex); K8(iTRAQ8plex); C22(Methylthio)	-	-	-	4.8	43	0	3388.72085	4	847.9356689	4.725559283	58.219465
														QENGASVILR	2	7	1	N-Term(iTRAQ8plex)	1.52	3.423	2.484	3.62	27	0	1390.79936	2	695.9033203	2.698236063	23.34395667
														IASLEVENQSLR	4	9	1	N-Term(iTRAQ8plex)	1.681	3.179	1.89	3.39	51	0	1662.92717	2	831.9672241	-3.409456475	32.86842167
														SIQLDGLVWGASK	1	10	1	N-Term(iTRAQ8plex); K13(iTRAQ8plex)	0.859	1.433	1.132	2.7	-	0	1982.13883	3	661.3844604	-7.216044679	32.94570333
														LVPVGYGIR	1	10	1	N-Term(iTRAQ8plex)	2.149	5.009	2.093	-	23	0	1277.78006	3	426.5982056	-6.470480341	31.87689833
194248072	HSPA1A	heat shock 70 kDa protein 1A	NP_005336.3	48.52	19	23	235	641	70.0	5.66	1.855	4.706	1.343																
														ELEQVCNPIISGLYQGAGG PGPGGFGAQGPK	5	1	1	N-Term(iTRAQ8plex); C6(Methylthio); K31(iTRAQ8plex)	-	-	-	8.19	112	0	3652.89731	3	1218.30542	8.315092512	57.30442333
														CQEVISWLDANTLAEKDE FEHK	9	1	1	N-Term(iTRAQ8plex); C1(Methylthio); K16(iTRAQ8plex); K22(iTRAQ8plex)	-	-	-	5.71	27	1	3563.85603	4	891.7119141	-0.557246649	59.66525
														AAAIGIDLGTTYSCVGVFQ HGK	2	1	1	N-Term(iTRAQ8plex); C14(Methylthio); K22(iTRAQ8plex)	-	-	-	5.55	34	0	2862.50942	4	716.3828125	-0.308184139	53.67105167
														IINEPTAAAIAYGLDR	8	2	1	N-Term(iTRAQ8plex)	1.64	3.704	1.536	5.14	70	0	1992.10271	2	996.5549927	-2.054396302	46.19798667
														LDKAQIHDLVLVGGSTR	4	1	1	N-Term(iTRAQ8plex); K3(iTRAQ8plex)	2.228	8.934	1.964	5.07	35	1	2430.42533	3	810.8132935	-1.482941634	30.75300833
														LVNHFVEEFK	36	1	1	N-Term(iTRAQ8plex); K10(iTRAQ8plex)	1.615	4.25	1.315	4.88	54	0	1870.05327	2	935.5302734	-8.048457891	41.76030167
														NALESYAFNMK	6	2	1	N-Term(iTRAQ8plex); K11(iTRAQ8plex)	2.489	7.61	1.594	4.69	65	0	1896.00078	2	948.5040283	-7.273878534	52.59203667
														ATAGDTHLGGEDFDNR	26	3	1	N-Term(iTRAQ8plex)	1.759	4.005	1.512	4.68	92	0	1979.93291	2	990.4691772	-2.542223752	16.44848833
														DAGVIAGLNVLR	12	2	1	N-Term(iTRAQ8plex)	1.652	3.794	1.416	4.5	55	0	1501.89702	2	751.4497681	-5.440994276	30.94942167
														AQIHDLVLVGGSTR	18	1	1	N-Term(iTRAQ8plex)	1.644	4.706	1.257	4.43	50	0	1770.01286	2	885.510498	-2.181805829	25.43166833
														QTQIFTTYSDNQPGVLIQV YEGER	3	2	1	N-Term(iTRAQ8plex)	-	-	-	4.36	43	0	3090.5687	3	1030.861084	0.028280596	48.40317
														GVPQIEVTFDIDANGILNV TATDK	2	2	1	N-Term(iTRAQ8plex); K24(iTRAQ8plex)	2.287	2.204	1.185	4.04	47	0	3138.69504	3	1046.903198	-6.158554171	65.96892167
														FEELCSDLFR	20	2	1	N-Term(iTRAQ8plex); C5(Methylthio)	2.085	6.186	1.35	3.93	51	0	1608.76079	2	804.8840332	-5.968888483	64.23093333
														MKEIAEAYLGYPVTNAVI TVPAYFNDSQR	1	1	1	N-Term(iTRAQ8plex); K2(iTRAQ8plex)	-	-	-	3.51	-	1	3869.05556	4	968.0193481	3.900537969	62.46603
														SAVEDEGLK	3	1	1	N-Term(iTRAQ8plex); K9(iTRAQ8plex)	1.231	2.264	1.353	3.41	47	0	1555.87114	2	778.439209	-4.916828294	13.64165167
														LVNHFVEEFKR	6	1	1	N-Term(iTRAQ8plex); K10(iTRAQ8plex)	1.966	6.094	1.664	3.25	-	1	2026.15432	4	507.2940369	-7.46424837	45.03737
														LLQDFFNGR	6	1	1	N-Term(iTRAQ8plex)	2.083	5.341	1.354	3.1	31	0	1413.77178	2	707.3895264	-5.273259624	72.88418333
														ARFEELCSDLFR	9	2	1	N-Term(iTRAQ8plex); C7(Methylthio)	2.63	5.934	2.348	2.95	38	1	1835.90769	3	612.6407471	-0.514371757	55.71121667
														STLEPVEK	2	2	1	N-Term(iTRAQ8plex); K8(iTRAQ8plex)	1.442	2.494	1.199	2.86	27	0	1510.89165	2	755.9494629	-1.358230255	13.78242333
														VEIIANDQGNR	24	6	3	N-Term(iTRAQ8plex)	-	-	-	4.22	66	0	1532.82414	3	511.616333	0.64225686	26.62443333
														TTPSYVAFTDTER	27	6	2	N-Term(iTRAQ8plex)	-	-	-	4.46	84	0	1791.8958	2	896.4515381	-6.067176522	26.16447333
														MVQEAEK	2	2	2	N-Term(iTRAQ8plex); K7(iTRAQ8plex)	-	-	-	3.4	27	0	1442.79975	3	486.9385986	-4.814673416	11.32049167
														MVQEAEK	2	2	2	N-Term(iTRAQ8plex); M1(Oxidation); K7(iTRAQ8plex)	-	-	-	3.39	35	0	1458.80124	3	486.9385986	-4.814673416	11.32049167
														TTPSYVAFTDTER	1	6	2	N-Term(iTRAQ8plex); Y5(iTRAQ8plex)	-	-	-	-	52	0	2096.106	2	896.4515381	-6.067176522	26.16447333
														ITITNDK	1	5	2	N-Term(iTRAQ8plex); K7(iTRAQ8plex)	-	-	-	-	27	0	1412.84452	3	471.6196899	-8.772887346	14.269095
194328699	EMG1	ribosomal RNA small subunit methyltransferase NEP1 isoform 1	NP_006322.4	18.44	3	3	10	244	26.7	9.17	2.011	1.261	1.098																
														NVLIEVNPQTR	4	2	1	N-Term(iTRAQ8plex)	1.494	1.014	0.857	5.29	51	0	1586.9227	3	529.645752	3.725014057	28.295875
														ELVPSSDPIVFVVGAFAHG K	4	1	1	N-Term(iTRAQ8plex); K20(iTRAQ8plex)	2.78	1.239	2.036	4.45	44	0	2677.50364	3	893.1727295	-5.152588126	57.37185333
														VGTSFSIPVVSDVR	2	1	1	N-Term(iTRAQ8plex)	2.08	1.322	1.149	4.26	43	0	1766.99468	2	884.0009766	-0.438481796	42.49907
194363762	HRAS	GTPase HRas isoform 1	NP_001123914.1	25.40	3	3	13	189	21.3	5.31	0.955	1.171	0.807																
														SALTIQLIQNHFVDEYDPTI EDSYR	5	6	1	N-Term(iTRAQ8plex)	0.931	0.801	0.389	7	58	0	3271.62229	3	1091.21228	-6.181560067	74.17076167
														QGVEDAFYTLVR	4	2	1	N-Term(iTRAQ8plex)	1.002	1.159	0.753	4.93	38	0	1701.91362	2	851.4604492	1.322345312	41.96558167

														LVVVGAGGVGK	4	7	1	N-Term(iTRAQ8plex); K11(iTRAQ8plex)	1.37	1.381	0.892	4	33	0	1564.00724	3	522.0072632	1.889377947	35.14503167
194473681	MPST	3-mercaptopyruvate sulfurtransferase isoform 2	NP_001123989.1	3.70	1	1	1	297	33.2	6.60	0.674	0.564	0.602																
														ARPEDVISEGR	1	2	1	N-Term(iTRAQ8plex)	0.674	0.564	0.602	-	33	0	1532.82859	3	511.6143799	-3.186854424	14.421485
194688149	PPP3CA	serine/threonine-protein phosphatase 2B catalytic subunit alpha isoform isoform 3	NP_001124164.1	5.12	1	1	1	469	52.6	6.23	0.623	1.001	0.236																
														NLLDIDAPVTVCGDIHGQFFDLMK	1	4	1	N-Term(iTRAQ8plex); C12(Methylthio); K24(iTRAQ8plex)	0.623	1.001	0.236	3.46	-	0	3315.70969	3	1105.908081	1.80583182	64.42755833
														TLFILR	2	2	2	N-Term(iTRAQ8plex)	-	-	-	-	20	0	1066.68999	2	533.8486328	-2.477097402	26.89852167
195539395	PSMC6	26S protease regulatory subunit 10B	NP_002797.3	13.65	4	4	13	403	45.8	7.78	0.814	1.267	1.136																
														DHQPCIIFMDEIDAIGGR	4	2	1	N-Term(iTRAQ8plex); C5(Methylthio)	1.377	1.191	0.723	5.17	53	0	2380.15024	3	794.0549316	4.217957211	63.13653333
														ALQSVGQIVGEVLK	4	1	1	N-Term(iTRAQ8plex); K14(iTRAQ8plex)	0.475	0.822	0.75	5.06	41	0	2049.24717	3	683.7539063	-2.774579902	50.158885
														NVCTEAGMFAIR	4	2	1	N-Term(iTRAQ8plex); C3(Methylthio)	0.814	2.187	1.567	4.17	50	0	1661.81499	2	831.4111328	2.06202557	40.70525667
														IHIDLPNEQAR	1	2	1	N-Term(iTRAQ8plex)	1.256	1.317	1.148	3.2	-	0	1609.88596	3	537.3001709	-6.479871836	24.904665
195546902	SUPT5H	transcription elongation factor SPT5 isoform b	NP_001124297.1	4.43	3	3	5	1083	120.4	5.10	0.836	0.817	0.886																
														HEDLKDMLEFPAQELR	2	3	1	N-Term(iTRAQ8plex); K5(iTRAQ8plex)	-	0.623	0.973	4.39	26	1	2579.36416	4	645.5964966	-4.150190212	40.00321667
														VEENFVILFSDLTMHELK	2	3	1	N-Term(iTRAQ8plex); K18(iTRAQ8plex)	-	-	-	3.12	29	0	2772.51504	4	693.8842163	1.711101406	68.29366167
														SSDGEEAEVDEERR	1	2	1	N-Term(iTRAQ8plex)	0.836	1.07	0.807	-	23	1	1911.87687	3	637.9638062	-3.423909294	10.76321667
195947407	ERCC2	TFIIH basal transcription factor complex helicase XPD subunit isoform 2	NP_001124339.1	4.69	1	1	1	405	46.2	8.63	0.978	0.833	0.740																
														VQHVVQESPPAFLSGLAQR	1	4	1	N-Term(iTRAQ8plex)	0.978	0.833	0.74	3.51	-	0	2367.31253	3	789.7756958	1.619073227	35.442695
195972866	KRT10	keratin, type I cytoskeletal 10	NP_000412.3	27.74	9	13	113	584	58.8	5.21	0.877	1.341	0.998																
														GSLGGGFSSGGFSGGSFSR	2	3	1	N-Term(iTRAQ8plex)	0.839	1.165	0.688	6.24	68	0	2011.99077	2	1006.499023	6.550206489	31.34144
														QSLEASLAETEGR	2	3	1	N-Term(iTRAQ8plex)	0.903	1.332	0.998	5.39	52	0	1694.89446	2	847.9508667	4.817691394	28.58004333
														NVQALEIELQSQLALK	4	3	1	N-Term(iTRAQ8plex); K16(iTRAQ8plex)	1.156	2.61	1.59	4.91	53	0	2405.42063	3	802.4783936	-0.75408264	49.17257333
														ADLEMQIESLTEELAYLK	6	3	1	N-Term(iTRAQ8plex); K18(iTRAQ8plex)	0.41	1.079	0.803	4.79	60	0	2704.46921	3	902.1560059	-1.52229348	60.60093
														TIDDLKNQILNLTTDNANILLQIDNAR	3	3	1	N-Term(iTRAQ8plex); K6(iTRAQ8plex)	-	-	-	4.66	31	1	3661.04287	3	1221.020874	2.703466365	66.774535
														DAEAWFNEK	6	7	1	N-Term(iTRAQ8plex); K9(iTRAQ8plex)	0.955	1.341	0.873	3.79	33	0	1717.89116	2	859.4492188	-5.489532873	25.84880667
														VLDELTLTK	4	4	1	N-Term(iTRAQ8plex); K9(iTRAQ8plex)	0.752	1.29	1.287	3.74	36	0	1640.0031	2	820.505188	-3.641422044	60.82017
														ALEESNYELEGK	2	3	1	N-Term(iTRAQ8plex); K12(iTRAQ8plex)	0.892	1.36	1.134	3.5	45	0	1990.04404	3	664.0195313	-7.487673487	33.73185
														SLLEGEGSSGGGGR	1	3	1	N-Term(iTRAQ8plex)	0.943	1.129	0.914	-	30	0	1566.80034	2	783.9038086	-1.423750329	17.783815
														LAADDFR	10	31	7	N-Term(iTRAQ8plex)	-	-	-	-	39	0	1111.60307	2	556.3051758	-1.68797437	21.86801333
														VTMQNLNDR	4	10	3	N-Term(iTRAQ8plex); M3(Oxidation)	-	-	-	3.55	61	0	1410.73212	3	470.9155579	0.584117972	12.82526667
														VTMQNLNDR	6	10	3	N-Term(iTRAQ8plex)	-	-	-	3	42	0	1394.73418	3	470.9155579	0.584117972	12.82526667
														LASYLDKVR	18	11	6	N-Term(iTRAQ8plex); K7(iTRAQ8plex)	-	-	-	3.04	34	1	1673.01726	4	419.0100403	-1.374788993	40.70226667
														LASYLDK	45	13	7	N-Term(iTRAQ8plex); K7(iTRAQ8plex)	-	-	-	3.56	36	0	1417.84687	3	473.2871399	-2.979780374	31.776525
196114970	PTPN12	tyrosine-protein phosphatase non-receptor type 12 isoform 3	NP_001124481.1	2.46	1	1	1	650	72.7	5.22	0.747	3.316	2.333																
														YWPLYGEDPITFAPFK	1	5	1	N-Term(iTRAQ8plex); K16(iTRAQ8plex)	0.747	3.316	2.333	3.16	-	0	2552.36386	3	851.4594727	-1.855762541	56.24130667
197116389	TMEM106B	transmembrane protein 106B	NP_001127704.1	3.65	1	1	4	274	31.1	6.99	1.054	0.518	0.522																
														SAYVSYDVQK	4	1	1	N-Term(iTRAQ8plex); K10(iTRAQ8plex)	1.054	0.518	0.522	4.34	55	0	1767.97331	2	884.4902954	-0.23026348	30.27498167
197927278	DERL1	derlin-1 isoform b	NP_001128143.1	10.82	2	2	4	231	26.4	9.54	1.274	1.431	0.665																
														YWFAATVAVPLVGK	2	2	1	N-Term(iTRAQ8plex); K14(iTRAQ8plex)	1.215	1.652	0.931	3.99	34	0	2130.2587	3	710.7577515	0.704066849	50.52329167
														NFLSTPQFLYR	2	3	1	N-Term(iTRAQ8plex)	1.336	1.239	0.474	3.44	30	0	1689.92107	2	845.4641724	-3.280149317	33.17927167
197927454	DEK	protein DEK isoform 2	NP_001128181.1	10.26	3	3	8	341	38.7	8.15	2.307	1.740	1.179																
														VYENYPTYDLTER	2	3	1	N-Term(iTRAQ8plex)	2.04	1.69	1.244	4.6	48	0	1966.96831	2	983.987793	-0.850030218	31.26211333
														NVGQFSGFPFEK	4	3	1	N-Term(iTRAQ8plex); K12(iTRAQ8plex)	2.405	2.08	1.186	4.43	38	0	1965.0571	3	655.6905518	-6.085968978	59.95406667
														SICEVLDLER	2	3	1	N-Term(iTRAQ8plex); C3(Methylthio)	2.237	1.777	0.917	3.72	52	0	1526.78545	2	763.8963623	-0.38925615	51.31454333
203098013	RETSAT	all-trans-retinol 13,14-reductase precursor	NP_060220.3	2.95	1	1	1	610	66.8	8.28	-	-	-																
														EEAAEHIPLLFFAFPSAK	1	1	1	N-Term(iTRAQ8plex); K18(iTRAQ8plex)	-	-	-	3.19	-	0	2625.44814	4	657.1174927	-2.140768575	57.15771
205277396	DCTN1	dynactin subunit 1 isoform 4	NP_001128513.1	1.05	1	1	4	1139	126.7	5.43	0.740	1.204	2.488																
														ELTNQQEASVER	4	6	1	N-Term(iTRAQ8plex)	0.74	1.204	2.488	5.27	66	0	1707.88565	3	569.9667358	2.414078203	19.42636
205277463	TKT	transketolase isoform 1	NP_001128527.1	41.57	18	18	172	623	67.8	7.66	0.949	3.086	4.770																
														SVPTSTVFYPSDGVATEK	8	3	1	N-Term(iTRAQ8plex); K18(iTRAQ8plex)	0.809	4.52	7.586	7.48	60	0	2493.32207	2	1247.168335	-1.58342421	30.24109667
														NMAEQIIQEIYSQIQSK	37	3	1	N-Term(iTRAQ8plex); K17(iTRAQ8plex)	0.603	9.282	20.144	6.56	78	0	2631.41313	2	1316.210205	-5.368888515	85.14358833
														ILTVEDHYYEGGIGEAVSSAVVGEPGITVTHLAVNR	6	3	1	N-Term(iTRAQ8plex)	-	-	-	6.11	44	0	4057.10634	4	1015.032043	-2.999022171	54.17780333
														LGQSDPAPLQHQMDIYQK	2	3	1	N-Term(iTRAQ8plex); M13(Oxidation); K18(iTRAQ8plex)	0.984	5.332	11.099	6.05	31	0	2693.40671	3	893.1412354	-5.116324973	28.46943167
														LGQSDPAPLQHQMDIYQK	6	3	1	N-Term(iTRAQ8plex); K18(iTRAQ8plex)	1.146	2.848	4.14	5.78	35	0	2677.40915	3	893.1412354	-5.116324973	28.46943167
														NMAEQIIQEIYSQIQSK	20	3	1	N-Term(iTRAQ8plex); M2(Oxidation); K17(iTRAQ8plex)	1.265	3.289	3.825	5.49	57	0	2647.413	2	1316.210205	-5.368888515	85.14358833
														CEAFGWHAIIVDGHSVEELCK	11	3	1	N-Term(iTRAQ8plex); C1(Methylthio); C20(Methylthio); K21(iTRAQ8plex)	3.681	4.869	9.55	5.02	41	0	3043.46427	3	1015.159607	-3.82976378	56.45752
														TSRPENAIIYNNNEDFQVGQAK	4	3	1	N-Term(iTRAQ8plex); K22(iTRAQ8plex)	-	-	-	4.87	37	0	3116.6083	4	779.9075317	-4.451706969	25.54721833
														HQPTAIIAK	8	3	1	N-Term(iTRAQ8plex); K9(iTRAQ8plex)	0.499	2.946	4.999	4.49	38	0	1586.96994	3	529.661499	-8.763543217	20.445095
														ISSDLDGHPVPK	15	2	1	N-Term(iTRAQ8plex); K12(iTRAQ8plex)	0.867	2.947	4.057	4.33	46	0	1873.05217	2	937.0297241	-6.299705898	21.55614667
														LDNLVAILDINR	12	3	1	N-Term(iTRAQ8plex)	0.381	3.326	8.169	4.26	80	0	1672.99912	3	558.3378906	5.467620932	62.59793333
														NSTFSEIFKK	2	3	1	N-Term(iTRAQ8plex); K9(iTRAQ8plex); K10(iTRAQ8plex)	-	-	-	4.1	23	1	2113.22353	4	529.0613403	-8.75906008	36.79299
														VLDPFTIKPLDR	15	3	1	N-Term(iTRAQ8plex); K8(iTRAQ8plex)	1.055	4.17	9.125	4.04	42	0	2022.20818	2	1011.607727	-6.252481114	36.44631833
														AVELAANTK	5	3	1	N-Term(iTRAQ8plex); K9(iTRAQ8plex)	0.425	1.728	2.273	3.92	47	0	1524.90589	3	508.9734802	-9.642481082	28.503285
														MFGIDRDAIAQAVR	5	3	1	N-Term(iTRAQ8plex)	1.607	1.274	2.008	3.81	39	1	1867.01042	2	934.0088501	-3.110708229	35.79169333
														TVPFCSTFAAFFTR	5	3	1	N-Term(iTRAQ8plex); C5(Methylthio)	0.663	8.188	18.54	3.78	51	0	1944.96018	3	648.9915771	-2.691111492	69.34079833
														NSTFSEIFK	5	3	1	N-Term(iTRAQ8plex); K9(iTRAQ8plex)	0.897	2.578	4.629	3.69	31	0	1680.92759	2	840.9675293	-8.286689291	28.88710167
														ILATPPQEDAPSVDIANIR	3	3	1	N-Term(iTRAQ8plex)	1.236	0.551	0.939	3.48	38	0	2324.27011	3	775.4282227	-2.709057441	42.48639667
														IIALDGDTK	1	3	1	N-Term(iTRAQ8plex); K9(iTRAQ8plex)	0.124	0.364	0.575	3.09	-	0	1553.92266	2	777.4649658	-8.530848902	35.038375
														NMAEQIIQEIYSQIQSK	1	3	1	N-Term(iTRAQ8plex); Y11(iTRAQ8plex); K17(iTRAQ8plex)	-	-	-	-	30	0	2935.62177	2	1316.210205	-5.368888515	85.14358833
														LILDSAR	1	3	1	N-Term(iTRAQ8plex)	0.599	3.082	5.784	-	24	0	1091.66619	2	546.336731	-5.904740867	23.92042
205360840	DNAJA3	dnaJ homolog subfamily A member 3, mitochondrial isoform 2	NP_001128582.1	15.67	6	6	15	453	49.6	9.39	1.492	0.886	0.580																
														FSQLAEAYEVLSDEVK	4	2	1	N-Term(iTRAQ8plex); K16(iTRAQ8plex)	1.477	0.801	0.605	4.89	57	0	2436.31963	2	1218.663452	3.179540479	59.33425833
														EFTVNIMDTCER	2	3	1	N-Term(iTRAQ8plex); C10(Methylthio)	1.609	0.886	0.582	4.53	45	0	1807.83623	2	904.4217529	1.74072149	41.90629667
														DGADIHSDLFISIAQALLGGTAR	4	3	1	N-Term(iTRAQ8plex)	2.078	0.888	-	3.86	53	0	2645.4285	3	882.4810181	3.163816076	67.09897833
														FSQLAEAYEVLSDEVKR	3	2	1	N-Term(iTRAQ8plex); K16(iTRAQ8plex)	1.492	0.887	0.473	3.82	39	1	2592.42031	4	648.8605347	2.818791324	60.68780667
														GGPTVDPEELFR	1	2	1	N-Term(iTRAQ8plex)	1.133	0.7	0.578	3.01	-	0	1620.85686	2	810.9320679	2.046955701	34.99158333
														EIFITFR	1	3	1	N-Term(iTRAQ8plex)	1.363	0.624	0.403	-	26	0	1229.71745	2	615.3623657	-1.729238435	29.43149333
207028748	NDRG1	protein NDRG1 isoform 1	NP_001128714.1	7.61	2	2	4	394	42.8	5.82	0.299	2.773	1.613																
														TASGSSVTSLDGTR	2	5	1	N-Term(iTRAQ8plex)	0.212	4.22	2.143	4.96	65	0	1642.85784	2	821.9325562	1.737450816	20.04889333
														EMQDVDLAEVKPLVEK	2	2	1	N-Term(iTRAQ8plex); K11(iTRAQ8plex); K16(iTRAQ8plex)	0.422	1.822	1.215	2.89	-	0	2755.54721	3	919.1872559	-7.518278681	33.53911833
207029439	RBBP4	histone-binding protein RBBP4 isoform c	NP_001128728.1	20.26	2	6	13	390	43.5	4.96	-	2.651	1.521																
														IGEEQSPEDAEDGPPELLFIHGGHTAK	2	3	1	N-Term(iTRAQ8plex); K27(iTRAQ8plex)	-	-	-	5.98	44	0	3481.76699	4	871.1972046	-0.770534224	32.68867667
														HPSKPDPSGECNPDLR	2	3	1	N-Term(iTRAQ8plex); K4(iTRAQ8plex); C11(Methylthio)	-	2.651	1.521	5.09	24	0	2403.18813	4	601.5524902	-5.181135151	18.239405
														TPSSDVLVFDYTK	2	5	2	N-Term(iTRAQ8plex); K13(iTRAQ8plex)	-	-	-	3.97	29	0	2080.13047	3	694.0483398	-5.675133816	27.72360667
														TVALWDLR	4	5	2	N-Term(iTRAQ8plex)	-	-	-	3.49	31	0	1277.74541	2	639.3763428	-5.120247805	29.29529
														LNVWDLSK	2	5	2	N-Term(iTRAQ8plex); K8(iTRAQ8plex)	-	-	-	3.05	29	0	1582.93888	3	528.3178101	-1.544200003	32.90782667
														LMIWDTR	1	5	2	N-Term(iTRAQ8plex)	-	-	-	-	25	0	1238.68205	2	619.8446655	-3.92099433	51.24503
208022622	RPS27A	ubiquitin-40S ribosomal protein S27a precursor	NP_001129064.1	58.33	9	9	168	156	18.0	9.64	0.855	1.305	1.325																
														ECPSDECGAGVFMASHFDR	12	1	1	N-Term(iTRAQ8plex); C2(Methylthio); C7(Methylthio)	1.39	1.78	1.806	7.08	87	0	2453.9943	3	818.6677246	-2.975858084	42.31819333
														TITLEVEPSDTIENVK	25	8	1	N-Term(iTRAQ8plex); K16(iTRAQ8plex)	0.98	1.093	1.185	6.08	64	0	2396.32646	2	1198.66687	-4.856469184	36.52759333
														ECPSDECGAGVFMASHFDR	6	1	1	N-Term(iTRAQ8plex); C2(Methylthio); C7(Methylthio); M13(Oxidation)	1.087	1.523	1.4	6.07	80	0	2469.98435	3	818.6677246	-2.975858084	42.31819333
														CCLTYCFNKPEDK	8	1	1	N-Term(iTRAQ8plex); C1(Methylthio); C2(Methylthio); C6(Methylthio); [illegible]	1.254	4.418	3.371	5.4	27	0	2614.22866	4	654.3126221	-5.613854283	46.92801833
														IQDKEGIPPDQQR	12	7	1	N-Term(iTRAQ8plex); K4(iTRAQ8plex)	0.733	1.26	1.378	4.34	52	1	2132.18047	2	1066.593872	-5.428142482	15.74789833
														TLSDYNIQK	14	7	1	N-Term(iTRAQ8plex); K9(iTRAQ8plex)	0.851	1.304	1.247	4.05	49	0	1689.95122	2	845.479248	-7.066373344	24.887445
														ESTLHLVLR	80	7	1	N-Term(iTRAQ8plex)	0.845	1.29	1.254	3.79	69	0	1371.82239	3	457.9450073	-4.156941432	22.96753833
														MQIFVK	1	6	1	N-Term(iTRAQ8plex); M1(Oxidation); K6(iTRAQ8plex)	1.121	1.575	1.614	2.88	-	0	1389.82596	3	458.6191101	-0.541712448	33.100395
														MQIFVK	7	6	1	N-Term(iTRAQ8plex); K6(iTRAQ8plex)	0.96	1.262	1.351	2.79	30	0	1373.84278	3	458.6191101	-0.541712448	33.100395
														EGIPPDQQR	2	7	1	N-Term(iTRAQ8plex)	0.659	0.877	0.962	-	28	0	1343.71807	2	672.3626709	-3.011139933	11.69135
														QLEDGR	1	7	1	N-Term(iTRAQ8plex)	0.898	1.31	1.22	-	23	0	1021.55272	2	511.2799988	-5.159167313	9.29024
208022658	GLB1	beta-galactosidase isoform c precursor	NP_001129074.1	2.56	1	1	2	546	60.5	6.99	0.296	0.374	0.515																
														TEAVASSLYDILAR	2	4	1	N-Term(iTRAQ8plex)	0.296	0.374	0.515	4.14	69	0	1812.99956	2	907.003418	-0.751231567	58.80720667
208431836	PABPC4	polyadenylate-binding protein 4 isoform 3	NP_001129126.1	19.49	2	8	67	631	69.5	9.52	1.334	2.684	1.937																
														NFGEEVDDESLKELFSQFGK	3	3	1	N-Term(iTRAQ8plex); K12(iTRAQ8plex); K20(iTRAQ8plex)	1.347	3.481	2.405	4.86	33	1	3230.67714	4	808.4247437	-6.607460192	56.04474667
														KSGVGNVFIK	1	4	1	N-Term(iTRAQ8plex); K1(iTRAQ8plex); K10(iTRAQ8plex)	1.322	2.069	1.559	3.39	-	1	1961.21379	2	981.1105347	-8.820345914	18.95226667
														ALYDTFSAFGNILSCK	40	4	2	N-Term(iTRAQ8plex); C15(Methylthio); K16(iTRAQ8plex)	-	-	-	6.61	79	0	2404.26738	2	1202.637329	7.170285326	65.123275
														SLGYAYVNFQQPADAER	6	4	2	N-Term(iTRAQ8plex)	-	-	-	5.78	90	0	2233.11455	2	1117.060913	-2.056208055	31.477775
														ALDTMNFDVIK	4	4	2	N-Term(iTRAQ8plex); K11(iTRAQ8plex)	-	-	-	4.97	71	0	1875.03594	2	938.0216064	-7.842532023	63.45430333
														ITGMLLEIDNSELLHMLESPESLR	2	4	2	N-Term(iTRAQ8plex)	-	-	-	3.75	46	0	3044.61618	3	1015.543579	6.931170766	69.39083
														AKEFTNVYIK	1	4	2	N-Term(iTRAQ8plex); K2(iTRAQ8plex); K10(iTRAQ8plex)	-	-	-	2.86	-	1	2125.2582	4	532.0700073	-9.515024165	29.22272833
														SLGYAYVNFQQPADAER	1	4	2	N-Term(iTRAQ8plex); Y4(iTRAQ8plex)	-	-	-	-	29	0	2537.31801	2	1117.060913	-2.056208055	31.477775
														GFGFVCFSSPEEATK	9	6	2	N-Term(iTRAQ8plex); C6(Methylthio); K15(iTRAQ8plex)	-	-	-	5.73	66	0	2260.11406	2	1130.560669	-4.349793465	37.29285167
208609990	NUP93	nuclear pore complex protein Nup93 isoform 1	NP_055484.3	10.50	7	7	13	819	93.4	5.72	1.143	0.939	1.157																
														SSGQSAQLLSHEPGDPPCLR	2	2	1	N-Term(iTRAQ8plex); C18(Methylthio)	0.95	0.635	0.946	4.4	29	0	2429.1771	3	810.3972168	-3.480668159	28.86563333
														TLITFAGMIPYR	4	2	1	N-Term(iTRAQ8plex)	1.199	1.339	1.44	3.87	34	0	1686.95671	2	843.9819946	0.723988828	47.70914
														SSLDNIEMAYAR	2	2	1	N-Term(iTRAQ8plex)	1.067	0.867	1.039	3.75	38	0	1673.84709	2	837.4271851	0.019235441	24.17615667
														NLQEIQQAGER	2	1	1	N-Term(iTRAQ8plex)	1.129	0.888	1.258	3.39	25	0	1589.85027	2	795.428772	-2.927303774	16.37711333

														NEKDNALLSAIEESR	1	1	1	N-Term(iTRAQ8plex); K3(iTRAQ8plex)	1.294	1.493	1.533	3.05	-	1	2297.25528	3	766.4232788	-0.212481487	30.77116667
														ASVLLGSR	1	1	1	N-Term(iTRAQ8plex)	1.153	0.803	1.063	-	25	0	1106.68205	2	553.8446655	-1.334498293	25.45395667
														AVYCIIGR	1	2	1	N-Term(iTRAQ8plex); C4(Methylthio)	1.397	0.992	1.002	-	25	0	1244.67339	2	622.840332	-5.073505679	29.386455
208973246	QDPR	dihydropteridine reductase isoform 1	NP_000311.2	7.38	1	1	2	244	25.8	7.37	0.613	0.849	1.452																
														MTDSFTEQADQVTAEVGK	2	1	1	N-Term(iTRAQ8plex); K18(iTRAQ8plex)	0.613	0.849	1.452	6.15	48	0	2565.30576	3	855.7734375	3.675175938	36.15276167
209364550	ECD	protein ecdysoneless homolog isoform 3	NP_001129225.1	3.16	1	1	2	601	67.7	4.75	0.726	0.913	1.876																
														IEDNDGEFLLIEAADFLPK	2	3	1	N-Term(iTRAQ8plex); K19(iTRAQ8plex)	0.726	0.913	1.876	3.91	31	0	2757.49491	3	919.8364868	5.122729033	63.81559
209413730	PVR	poliovirus receptor isoform gamma precursor	NP_001129241.1	2.47	1	1	4	364	39.3	6.21	3.212	1.155	2.058																
														QAELTVQVK	4	4	1	N-Term(iTRAQ8plex); K9(iTRAQ8plex)	3.212	1.155	2.058	3.69	53	0	1623.976	2	812.4916382	-8.00600059	29.73226333
209413738	RPN2	dolichyl-diphosphooligosaccharide--protein glycosyltransferase subunit 2 isoform 2 precursor	NP_001129243.1	29.27	12	12	63	615	67.7	6.06	0.909	0.500	0.642																
														NFESLSEAFSVASAAAVLSHNR	8	11	1	N-Term(iTRAQ8plex)	0.753	-	0.348	7.66	78	0	2611.35129	3	871.119751	1.086941792	49.64106
														TSFTPVGDVFELNFMNVK	8	11	1	N-Term(iTRAQ8plex); K18(iTRAQ8plex)	0.973	0.513	0.712	7.53	63	0	2653.40384	3	885.1394653	-4.454110496	59.59206
														KNFESLSEAFSVASAAAVLSHNR	4	11	1	N-Term(iTRAQ8plex); K1(iTRAQ8plex)	0.723	0.498	0.648	7.16	80	1	3043.63547	3	1015.216675	-2.207930615	49.679835
														FSSGYYDFLVEVEGDNR	8	11	1	N-Term(iTRAQ8plex)	0.899	0.504	0.676	6.28	110	0	2301.10674	2	1151.054443	1.681281145	52.15555333
														YHVPVVVVPEGSASDTHEQAILR	3	11	1	N-Term(iTRAQ8plex)	-	-	-	5.35	44	0	2807.49845	3	936.5043335	-0.346950047	31.179855
														ASLDRPFTNLESAFYSIVGLSSLGAQVPDAK	4	10	1	N-Term(iTRAQ8plex); K31(iTRAQ8plex)	-	-	0.532	5.33	24	0	3862.10146	3	1288.036865	2.910635314	68.49179
														SIVEEIEDLVAR	10	11	1	N-Term(iTRAQ8plex)	0.818	0.431	0.563	5.27	70	0	1676.93863	3	559.651062	0.811887286	64.72901167
														LQVTNVLSQPLTQATVK	2	11	1	N-Term(iTRAQ8plex); K17(iTRAQ8plex)	-	-	-	4.69	35	0	2448.46206	4	612.8709717	-1.051460545	39.35427667
														TSFTPVGDVFELNFMNVK	2	11	1	N-Term(iTRAQ8plex); M15(Oxidation); K18(iTRAQ8plex)	1.005	0.218	0.741	4.45	43	0	2669.41458	3	885.1394653	-4.454110496	59.59206
														QEIQHLFR	8	7	1	N-Term(iTRAQ8plex)	1.116	0.452	0.675	3.98	57	0	1374.77854	3	458.9310303	-0.737376719	24.80658667
														EDQVIQLMNAIFSK	2	11	1	N-Term(iTRAQ8plex); K14(iTRAQ8plex)	1.281	0.719	0.725	3.6	38	0	2244.23929	3	748.7512817	-5.605166615	83.970985
														YIANTVELR	2	11	1	N-Term(iTRAQ8plex)	0.896	0.632	0.688	2.69	25	0	1382.79155	2	691.8994141	-2.154912645	28.93324167
														FELDTSER	2	11	1	N-Term(iTRAQ8plex)	0.951	0.43	0.434	2.68	27	0	1300.66313	2	650.8352051	-4.257646432	17.03039333
209571584	FDPS	farnesyl pyrophosphate synthase isoform b	NP_001129294.1	15.30	4	4	26	353	40.5	5.17	0.729	1.442	1.738																
														VLTEDEMGHPEIGDAIAR	10	2	1	N-Term(iTRAQ8plex)	0.667	1.48	2.131	4.77	87	0	2257.13651	2	1129.071899	-3.176188859	32.32102667
														CSWLVVQCLQR	6	3	1	N-Term(iTRAQ8plex); C1(Methylthio); C8(Methylthio)	0.443	1.483	1.902	4.22	50	0	1730.84562	3	577.6235352	2.553024987	63.143365
														QDFVQHFSQIVR	6	2	1	N-Term(iTRAQ8plex)	0.816	1.442	1.411	3.43	40	0	1807.96416	2	904.4857178	-6.38418249	38.2965
														VLTEDEMGHPEIGDAIAR	2	2	1	N-Term(iTRAQ8plex); M7(Oxidation)	0.59	0.91	1.096	3.35	20	0	2273.12839	2	1129.071899	-3.176188859	32.32102667
														LKEVLEYNAIGGK	2	2	1	N-Term(iTRAQ8plex); K2(iTRAQ8plex); K13(iTRAQ8plex)	0.722	1.384	1.738	2.7	29	1	2346.39786	3	782.8041382	-7.730998872	45.13043667
209862837	EPHX1	epoxide hydrolase 1	NP_001129490.1	16.92	5	5	22	455	52.9	7.25	0.601	0.895	0.788																
														GGHFAAFEEPELLAQDIR	6	1	1	N-Term(iTRAQ8plex)	0.609	0.99	0.806	6.21	78	0	2304.19516	3	768.7365723	1.077272843	41.27073167
														VYVPTGFSAFPFELLHTPEK	9	1	1	N-Term(iTRAQ8plex); K20(iTRAQ8plex)	0.473	0.984	0.793	5.2	38	0	2887.58677	3	963.2004395	0.441132849	57.53919833
														VETSDEEIHDLHQR	2	1	1	N-Term(iTRAQ8plex)	0.839	0.789	0.788	3.7	30	0	2011.99271	3	671.3357544	-2.972856898	14.35724333
														DKEETLPLEDGWWGPGTR	2	1	1	N-Term(iTRAQ8plex); K2(iTRAQ8plex)	0.619	0.689	0.842	3.62	28	1	2694.39585	3	898.8034668	-0.962798164	39.05254167
														FLSVLER	3	1	1	N-Term(iTRAQ8plex)	0.482	0.678	0.729	2.77	30	0	1167.69451	2	584.3508911	-8.072054285	35.00209667
209869993	GART	trifunctional purine biosynthetic protein adenosine-3 isoform 1	NP_001129477.1	16.53	11	11	30	1010	107.7	6.70	0.993	3.041	1.811																
														SAGVQCFGPTAEAAQLESSK	2	2	1	N-Term(iTRAQ8plex); C6(Methylthio); K20(iTRAQ8plex)	0.891	1.143	0.791	5.99	43	0	2635.3328	3	879.1157837	0.427089975	35.03711667
														KIEFVVVGPEAPLAAGIVGNLR	4	2	1	N-Term(iTRAQ8plex); K1(iTRAQ8plex)	-	2.49	1.452	5.65	40	1	2857.7067	3	953.2404175	-2.019839403	53.06685833
														VDLGGFAGLFDLK	6	1	1	N-Term(iTRAQ8plex); K13(iTRAQ8plex)	0.962	3.483	1.623	4.75	66	0	1960.12297	2	980.5651245	-6.866353159	49.17561667
														AAGFKDPLLASGTDGVGTK	4	1	1	N-Term(iTRAQ8plex); K5(iTRAQ8plex); K19(iTRAQ8plex)	1.108	2.508	1.312	4.53	31	1	2717.53798	4	680.1399536	-8.153235392	28.53637333
														AFAHITGGGLLENIPR	2	1	1	N-Term(iTRAQ8plex)	0.977	1.329	1.309	4.49	45	0	1970.11631	3	657.3769531	1.900814174	39.16959333
														TVAPMPPAQDHK	2	2	1	N-Term(iTRAQ8plex); K12(iTRAQ8plex)	0.979	7.177	2.79	4.1	41	0	1900.04668	2	950.5269775	-5.49609754	15.549215
														HGIPTAQWK	2	2	1	N-Term(iTRAQ8plex); K9(iTRAQ8plex)	1.2	2.471	1.921	3.59	31	0	1645.94712	3	549.3205566	-9.924151244	18.70093
														IYSHSLLPVLR	2	1	1	N-Term(iTRAQ8plex)	1.22	2.32	1.16	3.54	-	0	1601.95952	2	801.4833984	-5.344108195	36.91103
														GVEITGFPEAQALGLEVFHAGTALK	1	2	1	N-Term(iTRAQ8plex); K25(iTRAQ8plex)	-	-	-	3.23	-	0	3163.77285	4	791.6986694	3.659097834	52.19927333
														MLNIHPSLLPSFK	1	1	1	N-Term(iTRAQ8plex); K13(iTRAQ8plex)	1.534	2.64	3.72	3.15	-	0	2105.22085	4	527.0606689	-9.179748903	43.122895
														TVAPMPPAQDHK	1	2	1	N-Term(iTRAQ8plex); M5(Oxidation); K12(iTRAQ8plex)	1.868	5.246	2.647	-	32	0	1916.05205	2	950.5269775	-5.49609754	15.549215
														VLEFNCR	3	2	1	N-Term(iTRAQ8plex); C6(Methylthio)	1.104	4.085	1.811	-	23	0	1230.61907	2	615.8131714	-6.993347332	45.98168833
209915573	APP	amyloid beta A4 protein isoform f precursor	NP_001129602.1	5.32	3	3	8	714	80.8	4.82	1.084	0.469	0.411																
														CLVGEFVSDALLVPDK	4	10	1	N-Term(iTRAQ8plex); C1(Methylthio); K16(iTRAQ8plex)	1.084	0.42	0.411	4.77	61	0	2359.29375	2	1180.150513	3.190137146	60.46189833
														EVCSEQAETGPCR	2	6	1	N-Term(iTRAQ8plex); C3(Methylthio); C12(Methylthio)	0.971	0.491	0.354	4.46	37	0	1804.76189	2	902.8845825	-1.177374673	28.50195667
														WYFDVTEGK	2	6	1	N-Term(iTRAQ8plex); K9(iTRAQ8plex)	1.217	0.678	0.611	3.82	24	0	1752.94077	3	584.9851074	-0.537544903	33.12339167
209969703	RCC2	protein RCC2	NP_001129676.1	13.03	5	5	13	522	56.0	8.78	1.321	4.036	1.886																
														AGGAAVVITEPEHTK	4	1	1	N-Term(iTRAQ8plex); K15(iTRAQ8plex)	0.829	3.788	1.404	4.77	41	0	2088.17923	3	696.7312622	-5.625919805	20.12538333
														IEYDCELVPR	1	1	1	N-Term(iTRAQ8plex); C5(Methylthio)	1.321	2.146	1.306	3.58	-	0	1586.78838	2	793.8978271	1.478065322	39.284465
														LIEGLSHEVIVSAACGR	5	1	1	N-Term(iTRAQ8plex); C15(Methylthio)	1.189	4.483	2.913	3.36	34	0	2104.12058	3	702.0450439	0.416461845	54.76203
														VFSWGFGGYGR	2	1	1	N-Term(iTRAQ8plex)	2.429	7.318	3.264	3.11	43	0	1536.78142	2	768.8943481	-5.668750823	69.38382667
														TKDGQILPVPNVVVR	1	1	1	N-Term(iTRAQ8plex); K2(iTRAQ8plex)	1.555	4.436	1.886	2.84	-	1	2243.35855	3	748.4577026	-4.933067797	28.42158167
210032390	SEC13	protein SEC13 homolog isoform 2	NP_001129704.1	11.04	2	2	6	308	34.0	5.72	1.476	2.013	2.087																
														VFIWTCDDASSNTWSPK	5	6	1	N-Term(iTRAQ8plex); C6(Methylthio); K17(iTRAQ8plex)	1.476	2.013	2.087	5.04	68	0	2611.26798	3	871.0941772	-3.899948915	43.49235
														GQGSVSASVTEGQQNEQ	1	4	1	N-Term(iTRAQ8plex)	-	-	-	-	23	0	2009.96189	3	670.6588135	-2.937428988	16.151025
210147571	ANP32E	acidic leucine-rich nuclear phosphoprotein 32 family member E isoform 3	NP_001129951.1	12.73	2	2	3	220	25.1	3.74	0.927	2.149	2.008																
														SLDLFNCEITNLEDYR	2	7	1	N-Term(iTRAQ8plex); C7(Methylthio)	0.659	3.429	3.439	3.22	46	0	2295.10259	2	1148.054932	3.775152057	61.23364833
														DLSTVEALQNLK	1	6	1	N-Term(iTRAQ8plex); K12(iTRAQ8plex)	1.303	1.348	1.173	2.88	-	0	1939.11765	3	647.0440674	-7.427296415	61.904005
211971074	AKAP2	A-kinase anchor protein 2 isoform 2	NP_001130034.1	1.86	1	1	1	859	94.6	5.11	0.459	0.378	1.252																
														AKEDIVTEQIDFSAAR	1	5	1	N-Term(iTRAQ8plex); K2(iTRAQ8plex)	0.459	0.378	1.252	3.22	-	1	2401.31229	3	801.1089478	-2.536649775	31.25143667
212274337	LANCL1	lanC-like protein 1	NP_001130046.1	11.78	3	3	6	399	45.3	7.75	0.515	1.080	1.271																
														IPQSHIQQICETILTSGENLAR	2	2	1	N-Term(iTRAQ8plex); C10(Methylthio)	-	2.253	5.046	5.57	49	0	2801.46805	3	934.4942017	3.155285124	65.48705667
														SLAEGYFDAAGR	2	2	1	N-Term(iTRAQ8plex)	0.503	0.787	1.252	4.77	30	0	1560.79595	2	780.9016113	-0.041814402	30.60619
														IDPHAPNEMLYGR	2	2	1	N-Term(iTRAQ8plex)	0.527	1.08	1.271	3.38	24	0	1816.92954	3	606.3146973	-1.245371973	23.92661833
212276104	LRRFIP1	leucine-rich repeat flightless-interacting protein 1 isoform 5	NP_001131025.1	3.99	2	2	4	752	82.6	4.61	0.672	0.803	1.069																
														SAVEAQNEVTENPK	2	3	1	N-Term(iTRAQ8plex); K14(iTRAQ8plex)	1.032	1.433	1.762	5.24	51	0	2124.1406	3	708.7183838	0.59639046	20.99086167
														IAAESSENVDCPENPK	2	3	1	N-Term(iTRAQ8plex); C11(Methylthio); K16(iTRAQ8plex)	0.438	0.45	0.648	4.04	41	0	2357.14157	3	786.385376	-6.706266812	20.88534333
213511012	BCAP31	B-cell receptor-associated protein 31 isoform b	NP_001132913.1	18.29	3	3	11	246	28.0	8.44	0.751	0.612	0.754																
														LVTLISQQATLLASNEAFKK	2	2	1	N-Term(iTRAQ8plex); K19(iTRAQ8plex); K20(iTRAQ8plex)	-	-	-	6.09	42	1	3087.85976	4	772.7203979	1.697168032	61.69039833
														VNLQNNPGAMEHFHMK	3	2	1	N-Term(iTRAQ8plex); K16(iTRAQ8plex)	0.708	0.502	0.525	3.81	40	0	2475.26836	2	1238.137817	-6.55813917	24.23874
														AENQVLAMR	4	2	1	N-Term(iTRAQ8plex)	0.754	0.639	0.814	3.13	33	0	1335.72869	2	668.367981	-5.22262194	16.82379667
														VNLQNNPGAMEHFHMK	2	2	1	N-Term(iTRAQ8plex); M10(Oxidation); K16(iTRAQ8plex)	0.677	0.558	0.7	3.01	-	0	2491.26601	2	1238.137817	-6.55813917	24.23874
214010185	APLP2	amyloid-like protein 2 isoform 4 precursor	NP_001135750.1	4.02	1	1	3	522	59.1	5.59	0.966	1.163	1.106																
														QYGTISHGIVEVDPMLTPEER	3	5	1	N-Term(iTRAQ8plex)	0.966	1.163	1.106	4.78	53	0	2675.3516	3	892.4553833	-5.12255947	37.24002333
214830079	SPG20	spartin	NP_001135766.1	2.70	1	1	2	666	72.8	5.91	1.424	1.127	2.123																
														VSQFLVDGVCTVANCVGK	2	1	1	N-Term(iTRAQ8plex); C10(Methylthio); C15(Methylthio); K18(iTRAQ8plex)	1.424	1.127	2.123	5.34	56	0	2539.30716	3	847.1072388	2.752624297	65.345365
215276969	ALG5	dolichyl-phosphate beta-glucosyltransferase isoform 2	NP_001135836.1	5.10	1	1	4	294	33.5	9.44	1.539	0.871	0.935																
														RLPVMMDEALSYLEK	4	2	1	N-Term(iTRAQ8plex); K15(iTRAQ8plex)	1.539	0.871	0.935	4.96	34	1	2403.32737	3	801.7817383	2.908585931	59.38477667
215490098	SPNS1	protein spinster homolog 1 isoform 4	NP_001135921.1	3.74	1	1	6	454	49.0	7.75	2.016	1.320	1.274																
														HSDLPPLNPTSWWADLR	6	8	1	N-Term(iTRAQ8plex)	2.016	1.32	1.274	4.75	56	0	2309.19687	3	770.4038086	-0.523458508	53.79108333
215983058	MAGT1	magnesium transporter protein 1	NP_115497.4	3.81	1	1	2	367	41.5	9.94	0.713	0.433	0.546																
														QADEEFQILANSWR	2	1	1	N-Term(iTRAQ8plex)	0.713	0.433	0.546	5.22	69	0	2011.02776	2	1006.017517	4.498326616	46.00240833
217035105	DNAJC11	dnaJ homolog subfamily C member 11	NP_060668.2	11.99	4	4	14	559	63.2	8.40	1.364	0.739	0.558																
														GWGELEFGAGDLQGPLFGLK	4	2	1	N-Term(iTRAQ8plex); K20(iTRAQ8plex)	1.37	0.762	0.494	5.24	50	0	2699.45926	3	900.491272	-2.27772729	50.32073167
														LFNLVHQAYEVLSDPQTR	4	1	1	N-Term(iTRAQ8plex)	1.053	0.278	0.61	5.07	44	0	2434.31119	3	812.1085815	3.257627429	67.20002667
														AGFFGTVVEYGAER	2	2	1	N-Term(iTRAQ8plex)	1.175	0.769	0.558	4.83	47	0	1806.93376	2	903.97052	0.504788322	40.46678
														GVLHQVMVLDSEALR	4	2	1	N-Term(iTRAQ8plex)	1.414	0.85	0.64	4.44	42	0	1971.0961	3	657.7035522	-1.952117764	42.07060167
217272851	P4HA1	prolyl 4-hydroxylase subunit alpha-1 isoform 3 precursor	NP_001136068.1	25.97	9	9	18	516	58.9	6.02	0.994	0.847	1.118																
														VSVLDYLSYAVYQQGDLDK	2	3	1	N-Term(iTRAQ8plex); K19(iTRAQ8plex)	0.74	0.796	0.933	6.08	51	0	2784.50077	3	928.8384399	3.284349686	58.67949167
														SAWLSGYENPVVSR	2	3	1	N-Term(iTRAQ8plex)	1.069	0.779	0.933	5.07	38	0	1868.98943	2	934.9983521	4.588673128	35.16346333
														LNTEWSELENLVLK	6	3	1	N-Term(iTRAQ8plex); K14(iTRAQ8plex)	1.423	1.354	1.698	4.97	52	0	2296.3035	3	766.1060181	1.123392103	57.63452667
														GVAVDYLPER	2	3	1	N-Term(iTRAQ8plex)	0.795	0.847	0.897	4.14	41	0	1422.78325	2	711.8952637	-4.365089515	27.840615
														VAYTEADYYHTELWMEQALR	1	3	1	N-Term(iTRAQ8plex)	0.726	0.474	0.794	4.09	-	0	2793.34934	3	931.7879639	-0.101293609	52.87233333
														FHDIISDAEIEIVK	2	3	1	N-Term(iTRAQ8plex); K14(iTRAQ8plex)	-	-	-	3.75	35	0	2237.26137	4	560.0708008	-1.08974974	46.34781167
														DPEGFVGHPVNAFK	1	3	1	N-Term(iTRAQ8plex); K14(iTRAQ8plex)	0.995	0.905	1.118	3.17	-	0	2122.14109	3	708.0518799	-6.18003282	26.68489833
														YFEYIMAK	1	3	1	N-Term(iTRAQ8plex); K8(iTRAQ8plex)	0.746	0.591	1.12	-	27	0	1672.91619	2	836.961731	-4.002594987	67.69815
														KGTAVFWYNLFASGEGDYSTR	1	3	1	N-Term(iTRAQ8plex); K1(iTRAQ8plex)	-	-	-	-	22	1	2977.52652	3	993.1803589	-1.332612762	55.21217333
217272894	EFTUD2	116 kDa U5 small nuclear ribonucleoprotein component isoform b	NP_001136077.1	28.82	17	18	67	937	105.3	5.22	1.368	0.924	1.133																
														GLAEDIENEVVQITWNR	2	3	1	N-Term(iTRAQ8plex)	1.285	0.509	0.83	6.01	61	0	2290.22026	2	1145.61377	9.654592121	60.36886
														SYLFNIMDTPGHVNFSDEVTAGLR	8	3	1	N-Term(iTRAQ8plex)	1.289	1.303	1.104	5.91	53	0	2987.49057	3	996.501709	1.011686247	53.27820167
														ALLGSVKDSIVQGFQWGTR	3	3	1	N-Term(iTRAQ8plex); K7(iTRAQ8plex)	1.263	1.231	1.015	5.84	42	1	2670.51528	4	668.3842773	-1.326262773	57.09440333
														ILDAVVAQEPLHR	6	3	1	N-Term(iTRAQ8plex)	1.312	1.113	1.172	5.5	62	0	1765.02152	3	589.0130615	-1.580702976	35.97159333
														TCFVDCLIEQTHPEIR	4	2	1	N-Term(iTRAQ8plex); C2(Methylthio); C6(Methylthio)	3.308	3.149	2.962	5.17	85	0	2300.08756	3	767.3673706	1.128333744	56.19456833
														IYADTFGDINYQEFAK	5	3	1	N-Term(iTRAQ8plex); K16(iTRAQ8plex)	1.225	0.72	1.25	4.8	61	0	2503.29068	3	835.0991211	-5.489299369	37.438875
														VADPVVTFCETVVETSSLK	4	3	1	N-Term(iTRAQ8plex); C9(Methylthio); K19(iTRAQ8plex)	1.865	1.195	1.317	4.52	49	0	2678.44004	3	893.4848633	5.922960206	65.96149167
														MYSTDDGVQFHAFGR	2	3	1	N-Term(iTRAQ8plex)	1.17	1.421	1.316	4.47	33	0	2034.96054	3	678.9916992	-1.973941834	28.25736333
														FFDDPMLLELAK	6	3	1	N-Term(iTRAQ8plex); K12(iTRAQ8plex)	0.987	0.781	0.814	4.44	75	0	2047.13042	2	1024.068848	-4.422096129	49.24865833
														SFVEFILEPLYK	10	3	1	N-Term(iTRAQ8plex); K12(iTRAQ8plex)	1.721	0.639	1.132	4.36	66	0	2093.21299	2	1047.109497	-1.243915918	55.796565
														AFIPAIDSFGFETDLR	4	3	1	N-Term(iTRAQ8plex)	1.202	0.618	0.808	4.22	71	0	2103.09722	2	1052.052246	-4.405755678	57.18110833

														GLSEDVSISK	2	3	1	N-Term(iTRAQ8plex); K10(iTRAQ8plex)	1.979	0.971	1.133	4.1	59	0	1642.94927	2	821.9782715	1.256915714	31.12406167
														RLWGDIYFNPK	2	3	1	N-Term(iTRAQ8plex); K11(iTRAQ8plex)	1.27	0.805	0.961	3.77	27	1	2017.13541	3	673.0499878	-6.227232456	35.561395
														GHVTQDAPIPGSPLYTIK	2	3	1	N-Term(iTRAQ8plex); K18(iTRAQ8plex)	1.955	2.524	1.455	3.76	30	0	2502.40702	3	834.8071899	-4.261787036	28.11572333
														ILAQVVGDVDTSLPR	2	3	1	N-Term(iTRAQ8plex)	-	-	-	3.62	84	0	1887.09043	3	629.7016602	2.695111308	61.807575
														EGPLCDELIR	2	3	1	N-Term(iTRAQ8plex); C5(Methylthio)	1.83	0.879	1.074	3.6	25	0	1494.76848	2	747.8878784	5.785455107	37.76151167
														SIVIRPLEPQPAPHLAR	2	3	1	N-Term(iTRAQ8plex)	-	-	-	2.96	24	0	2198.2958	4	550.3294067	-5.360619299	26.34345667
														GGGQIIPTAR	1	4	2	N-Term(iTRAQ8plex)	-	-	-	2.95	-	0	1273.74748	2	637.3773804	-4.347155054	17.59469667
217416369	SAR1A	GTP-binding protein SAR1a	NP_001136120.1	25.76	3	4	7	198	22.4	6.68	1.216	1.635	2.084																
														NYLPAINGIVFLVDCADHSR	2	1	1	N-Term(iTRAQ8plex); C15(Methylthio)	0.91	-	2.084	4.19	45	0	2567.30417	3	856.4395752	-0.428451009	69.39886833
														EIFGLYGQTTGK	1	1	1	N-Term(iTRAQ8plex); K12(iTRAQ8plex)	1.216	1.522	1.655	3.41	-	0	1922.06632	3	641.3602905	-9.387276493	48.39793167
														TDAISEEK	2	1	1	N-Term(iTRAQ8plex); K8(iTRAQ8plex)	1.298	1.756	2.234	3.2	26	0	1500.82634	2	750.9168091	-6.822745346	13.08266833
														LVFLGLDNAGK	2	2	2	N-Term(iTRAQ8plex); K11(iTRAQ8plex)	-	-	-	3.94	49	0	1755.05315	2	878.0302124	-5.340644711	66.90018167
218505670	EPCAM	epithelial cell adhesion molecule precursor	NP_002345.2	16.56	3	3	8	314	34.9	7.46	2.983	2.049	1.204																
														AKPEGALQNNDGLYDPDCDESGLFK	2	1	1	N-Term(iTRAQ8plex); K2(iTRAQ8plex); C18(Methylthio); K25(iTRAQ8plex)	-	-	-	4.98	49	0	3654.81313	4	914.4587402	-1.436801913	35.378075
														TQNDVDIADVAYYFEK	2	1	1	N-Term(iTRAQ8plex); K16(iTRAQ8plex)	1.532	2.049	1.204	4.25	35	0	2499.27298	3	833.7625122	-5.360730499	50.46825
														LAVNCFVNNNR	4	1	1	N-Term(iTRAQ8plex); C5(Methylthio)	3.514	2.407	1.213	3.98	22	0	1613.81792	2	807.4125977	-0.919315298	48.97181833
218931253	DAGLB	sn1-specific diacylglycerol lipase beta isoform 2	NP_001136408.1	1.47	1	1	1	543	59.8	8.07	1.648	0.442	1.187																
														ESVVVAVR	1	2	1	N-Term(iTRAQ8plex)	1.648	0.442	1.187	3.04	-	0	1162.70989	2	581.8585815	0.116250785	23.97469333
219283155	API5	apoptosis inhibitor 5 isoform c	NP_001136403.1	4.00	1	1	2	450	50.7	6.18	-	-	-																
														GTLGGLFSQILQGEDIVR	2	6	1	N-Term(iTRAQ8plex)	-	-	-	4.27	53	0	2207.23606	3	736.4168701	1.017472307	64.60890167
219555710	EIF5A	eukaryotic translation initiation factor 5A-1 isoform B	NP_001137233.1	35.06	5	5	56	154	16.8	5.24	0.913	4.878	4.829																
														RNDFQLIGIQDGYLSLLQDSGEVR	6	2	1	N-Term(iTRAQ8plex)	2.58	1.301	0.868	6.15	71	1	3040.60837	3	1014.207642	2.552204605	57.27936
														NDFQLIGIQDGYLSLLQDSGEVR	6	2	1	N-Term(iTRAQ8plex)	-	-	-	6.09	71	0	2884.48979	2	1442.748535	-3.361983641	61.70561667
														VHLVGIDIFTGK	40	4	1	N-Term(iTRAQ8plex); K12(iTRAQ8plex)	0.913	5.448	5.906	5.26	60	0	1907.14775	2	954.0751953	-7.533412094	39.36798667
														YEDICPSTHNMDVPNIK	1	4	1	N-Term(iTRAQ8plex); C5(Methylthio); K17(iTRAQ8plex)	0.375	2.297	2.353	3.6	-	0	2630.26554	3	877.4266968	-8.290120393	36.934705
														KYEDICPSTHNMDVPNIK	3	4	1	N-Term(iTRAQ8plex); K1(iTRAQ8plex); C6(Methylthio); K18(iTRAQ8plex)	-	4.878	4.829	3.51	31	1	3062.59439	3	1021.536316	2.194937703	34.08671667
219842220	FKBP11	peptidyl-prolyl cis-trans isomerase FKBP11 isoform 3 precursor	NP_001137254.1	32.19	3	3	8	146	15.8	6.16	2.191	0.783	0.161																
														QVIPGLEQSLLDMCVGEK	4	2	1	N-Term(iTRAQ8plex); C14(Methylthio); K18(iTRAQ8plex)	2.627	0.728	0.061	6.24	59	0	2613.41178	3	871.8087769	7.907294436	64.216675
														AIIPSHLAYGK	2	3	1	N-Term(iTRAQ8plex); K11(iTRAQ8plex)	1.869	0.74	0.338	3.68	34	0	1778.07566	3	593.3634033	-1.588565172	24.05726667
														IIDTSLTRDPLVIELGQK	2	2	1	N-Term(iTRAQ8plex); K18(iTRAQ8plex)	1.085	0.838	0.247	3.64	27	1	2619.55092	4	655.6431885	-1.251082283	47.00639333
219842250	PPHLN1	periphilin-1 isoform 8	NP_001137261.1	2.39	1	1	1	293	33.9	6.64	1.840	0.860	0.727																
														SIQFALR	1	32	1	N-Term(iTRAQ8plex)	1.84	0.86	0.727	-	21	0	1138.6845	2	569.8458862	-3.614222445	34.445825
219842335	GSTK1	glutathione S-transferase kappa 1 isoform d	NP_001137153.1	13.11	2	2	3	183	20.5	6.54	0.524	0.269	0.449																
														NEDITEPQSILAAAEK	2	2	1	N-Term(iTRAQ8plex); K16(iTRAQ8plex)	0.481	0.271	0.435	7.17	64	0	2337.29009	2	1169.148682	6.094819523	34.33933833
														ETTEAACR	1	2	1	N-Term(iTRAQ8plex); C7(Methylthio)	0.57	0.267	0.462	-	19	0	1230.57634	2	615.7918091	0.251300185	17.271485
221136993	TMX2	thioredoxin-related transmembrane protein 2 isoform 2	NP_001137484.1	17.83	4	4	6	258	29.6	8.65	2.495	1.046	0.910																
														AVSWTFSEENVIR	2	2	1	N-Term(iTRAQ8plex)	2.561	1.046	0.91	3.91	39	0	1841.97026	2	921.4887695	0.163489339	37.61740833
														TIDEELER	1	2	1	N-Term(iTRAQ8plex)	2.008	1.192	1.194	3.41	-	0	1308.68926	2	654.8482666	-4.305618654	21.15180667
														EFNLNELYQR	2	2	1	N-Term(iTRAQ8plex)	2.495	0.8	0.855	2.72	36	0	1629.84209	2	815.4246826	-7.210935162	28.66124667
														AVLAPLIALVYSVPR	1	2	1	N-Term(iTRAQ8plex)	-	-	-	-	28	0	1886.1812	2	943.5942383	1.63751102	69.86045667
221139815	LEMD2	LEM domain-containing protein 2 isoform 2	NP_001137416.1	4.98	1	1	1	201	23.5	6.23	1.114	1.099	0.994																
														YPYVGILHVR	1	2	1	N-Term(iTRAQ8plex)	1.114	1.099	0.994	-	28	0	1520.88663	3	507.633728	-1.617785257	32.92783167
221307584	PHB2	prohibitin-2 isoform 1	NP_001138303.1	56.86	14	14	126	299	33.3	9.83	1.189	0.779	0.712																
														IGGVQQDTILAEGLHFR	24	2	1	N-Term(iTRAQ8plex)	1.064	0.833	0.828	6.48	91	0	2158.18979	2	1079.599854	0.073724311	39.474565
														GMGTALKLLLGAGAVAYGVR	9	2	1	N-Term(iTRAQ8plex); K7(iTRAQ8plex)	1.715	0.978	0.777	6.18	44	1	2526.50991	4	632.3829346	1.902664713	63.34494
														IVQAEGEAEAAK	6	1	1	N-Term(iTRAQ8plex); K12(iTRAQ8plex)	1.37	0.739	0.645	5.56	62	0	1824.02495	2	912.5165405	-3.589239416	21.099315
														IYLTADNLVLNLQDESFTR	12	2	1	N-Term(iTRAQ8plex)	1.281	0.663	0.702	5.43	70	0	2529.34659	3	843.7955933	8.676158822	62.53909667
														LLLGAGAVAYGVR	6	2	1	N-Term(iTRAQ8plex)	0.816	0.701	0.703	5.38	71	0	1563.95084	3	521.9884644	-1.036786804	40.81036
														AKDFSLILDDVAITELSFSR	20	2	1	N-Term(iTRAQ8plex); K2(iTRAQ8plex)	1.836	0.973	0.824	4.4	69	1	2848.59775	3	950.2016602	-0.454288281	68.65432
														ESVFTVEGGHR	10	2	1	N-Term(iTRAQ8plex)	0.95	0.71	0.721	4.2	42	0	1521.79163	3	507.9353943	-3.09614574	17.71607
														EYTAAVEAK	4	2	1	N-Term(iTRAQ8plex); K9(iTRAQ8plex)	1.143	0.701	0.628	4.12	50	0	1589.89922	2	795.4532471	-0.184778615	27.10679167
														IPWFQYPIIYDIR	19	2	1	N-Term(iTRAQ8plex)	1.236	0.828	0.731	3.96	45	0	2028.13419	3	676.7160034	3.65148747	59.76431833
														DLQMVNISLR	4	2	1	N-Term(iTRAQ8plex)	1.031	0.575	0.552	3.81	38	0	1492.85039	2	746.928833	2.979599065	34.085165
														VLSRPNAQELPSMYQR	3	2	1	N-Term(iTRAQ8plex)	1.065	0.757	0.599	3.34	32	0	2193.17461	2	1097.090942	-0.284133499	26.25468167
														AQVSLLIR	6	2	1	N-Term(iTRAQ8plex)	1.189	0.794	0.742	3.33	32	0	1203.76824	2	602.3877563	-3.69275792	35.83455167
														AAQNISK	1	2	1	N-Term(iTRAQ8plex); K7(iTRAQ8plex)	1.114	0.856	0.85	3.05	-	0	1339.81885	3	447.2778015	2.589895651	12.799215
														LGLDYEER	2	2	1	N-Term(iTRAQ8plex)	0.895	0.553	0.537	2.75	24	0	1298.68364	2	649.845459	-4.435222765	18.10582667
221316566	CHORDC1	cysteine and histidine-rich domain-containing protein 1 isoform a	NP_036256.2	11.14	3	3	8	332	37.5	7.87	1.566	6.358	2.898																
														TTDFSDFLSIVGCTK	6	1	1	N-Term(iTRAQ8plex); C13(Methylthio); K15(iTRAQ8plex)	1.566	6.358	2.79	5.17	68	0	2288.16941	2	1144.598267	5.656998471	59.21477333
														FQEHIIQAPKPVEAIK	1	2	1	N-Term(iTRAQ8plex); K10(iTRAQ8plex); K16(iTRAQ8plex)	0.901	5.714	2.981	2.81	-	0	2760.63369	4	690.9138794	-7.343821806	29.52224833
														YWSCCR	1	1	1	N-Term(iTRAQ8plex); C4(Methylthio); C5(Methylthio)	1.886	6.638	2.898	-	13	0	1213.48577	2	607.246521	-5.80800205	38.42337833
221316630	COPB1	coatomer subunit beta	NP_001137533.1	13.54	8	8	21	953	107.1	6.05	0.490	1.506	1.623																
														NFEHLIPDAPELIHDFLVNEK	4	1	1	N-Term(iTRAQ8plex); K21(iTRAQ8plex)	0.351	1.654	1.859	5.97	43	0	3098.69016	3	1033.568237	4.188016032	61.014535
														NAFMMLIHADQDR	2	1	1	N-Term(iTRAQ8plex)	1.122	1.451	1.623	5.62	44	0	1865.92417	3	622.6462402	-3.362326821	37.63074167
														VTVNTNMVDLNDYLQHILK	4	1	1	N-Term(iTRAQ8plex); K19(iTRAQ8plex)	0.49	0.797	0.979	5.59	36	0	2838.5685	4	710.397583	1.434121194	56.71872333
														GALWILGEYCSTK	2	1	1	N-Term(iTRAQ8plex); C10(Methylthio); K13(iTRAQ8plex)	-	1.859	3.508	5.28	55	0	2095.09824	3	699.0375977	-9.270487122	79.82847333
														LKEAELLEPLMPAIR	2	1	1	N-Term(iTRAQ8plex); K2(iTRAQ8plex)	0.37	0.711	0.965	4.43	27	1	2331.38614	3	777.8002319	-2.976233363	49.158315
														VLSECSPLMNDIFNK	4	1	1	N-Term(iTRAQ8plex); C5(Methylthio); K15(iTRAQ8plex)	0.649	2.08	2.977	4.35	42	0	2364.2164	3	788.7436523	-2.465306789	56.21179333
														LVEKPSPLTLAPHDFANIK	1	1	1	N-Term(iTRAQ8plex); K4(iTRAQ8plex); K19(iTRAQ8plex)	-	-	-	3.48	-	0	3002.75373	5	601.3565674	-8.958741992	37.32296
														SLGEIPIVESEIKK	2	1	1	N-Term(iTRAQ8plex); K13(iTRAQ8plex); K14(iTRAQ8plex)	0.936	1.564	1.544	3.35	23	1	2454.47939	4	614.3753052	-6.221472844	32.93544667
221316723	NAT10	RNA cytidine acetyltransferase isoform a	NP_078938.2	16.10	11	11	22	1025	115.6	8.27	1.254	1.077	0.762																
														ALQLLQMYYEGRFPCLEEK	2	2	1	N-Term(iTRAQ8plex); C15(Methylthio); K19(iTRAQ8plex)	-	-	-	5.56	33	1	2985.56655	4	747.1470947	5.636024511	63.999435
														ELKESLQDTQPVGVLVDCCK	1	2	1	N-Term(iTRAQ8plex); K3(iTRAQ8plex); C18(Methylthio); C19(Methylthio); K20(iTRAQ8plex)	-	-	-	4.41	-	1	3208.69814	4	802.9299927	3.982151815	53.03972
														QDDPFELFIAATNR	2	2	1	N-Term(iTRAQ8plex)	1.758	0.724	0.646	4.14	61	0	2054.09355	2	1027.550415	3.642559567	59.75419333
														NMVDYHLIMDMIPAISR	1	2	1	N-Term(iTRAQ8plex)	1.254	0.99	0.545	3.92	-	0	2323.19126	3	775.0686035	-0.105029289	58.26859167
														IAVHPDYQGMGYGSR	2	2	1	N-Term(iTRAQ8plex)	1.888	1.172	0.69	3.58	31	0	1954.96854	3	652.3276978	-3.161835168	22.82403333
														TEAHQDVVGR	2	2	1	N-Term(iTRAQ8plex)	1.03	0.963	0.805	3.54	31	0	1415.75571	3	472.5900879	0.875237067	11.36659667
														IVSGCPLPEACELYYVNR	2	2	1	N-Term(iTRAQ8plex); C5(Methylthio); C11(Methylthio)	-	2.061	2.049	3.53	31	0	2422.16452	3	808.0596924	2.642511842	62.40733833
														STVALTAAR	2	2	1	N-Term(iTRAQ8plex)	2.016	0.664	0.655	3.39	53	0	1193.71831	2	597.362793	2.302065302	20.36409167
														TLHEVSLQESIR	4	2	1	N-Term(iTRAQ8plex)	1.215	1.79	1.359	3.16	25	0	1715.9542	3	572.6558228	-3.768336663	21.03471667
														LGQAELVVIDEAAAIPLPLVK	2	2	1	N-Term(iTRAQ8plex); K21(iTRAQ8plex)	-	-	-	3.07	27	0	2767.6874	4	692.6773071	2.8786978	67.17047333
														SLFVVVGDR	2	1	1	N-Term(iTRAQ8plex)	1.831	0.59	0.722	2.84	25	0	1295.75908	2	648.3831787	-2.65519893	34.01578
222136639	MTHFD1	C-1-tetrahydrofolate synthase, cytoplasmic	NP_005947.3	27.59	19	19	68	935	101.5	7.30	1.051	5.790	3.549																
														STTTIGLVQALGAHLYQNVFACVR	6	1	1	N-Term(iTRAQ8plex); C22(Methylthio)	2.367	23.969	13.612	8.26	59	0	2912.53031	3	971.5149536	-4.317452122	85.80338833
														IVGAPMHDLLLWNNATVTTCHSK	5	1	1	N-Term(iTRAQ8plex); C20(Methylthio); K23(iTRAQ8plex)	-	-	-	6.81	50	0	3175.65781	3	1059.224121	-3.070944457	47.849925
														YVVVTGITPTPLGEGK	2	1	1	N-Term(iTRAQ8plex); K16(iTRAQ8plex)	1.076	8.37	5.166	5.93	56	0	2239.31865	2	1120.162964	1.254763083	34.75145333
														AYIQENLELVEK	7	1	1	N-Term(iTRAQ8plex); K12(iTRAQ8plex)	1.061	1.702	1.551	5.12	49	0	2057.16281	3	686.3924561	-5.394024166	57.59951167
														AAQAPSSFQLLYDLK	2	1	1	N-Term(iTRAQ8plex); K15(iTRAQ8plex)	1.004	3.004	1.77	5.03	37	0	2260.28008	2	1130.643677	0.130778317	43.62938833
														MFGIPVVVAVNAFK	10	1	1	N-Term(iTRAQ8plex); K14(iTRAQ8plex)	0.951	8.76	4.358	4.76	51	0	2100.24479	3	700.7531128	-2.497885547	51.54698667
														IYGADDIELLPEAQHK	2	1	1	N-Term(iTRAQ8plex); K16(iTRAQ8plex)	0.89	2.831	1.732	4.71	49	0	2420.32248	3	807.4456787	-2.362667466	34.12779667
														MFGIPVVVAVNAFK	2	1	1	N-Term(iTRAQ8plex); M1(Oxidation); K14(iTRAQ8plex)	2.38	13.046	4.833	4.37	36	0	2116.23374	3	700.7531128	-2.497885547	51.54698667
														EIGLLSEEVELYGETK	2	1	1	N-Term(iTRAQ8plex); K16(iTRAQ8plex)	1.265	1.923	1.661	4.26	37	0	2417.30729	3	806.4406128	-8.238990225	42.49192
														LDIDPETITWQR	2	1	1	N-Term(iTRAQ8plex)	0.795	7.378	3.999	4	41	0	1790.95891	2	895.9830933	-0.0845338	38.65173667
														IFHELTQTDK	2	1	1	N-Term(iTRAQ8plex); K10(iTRAQ8plex)	1.212	1.747	1.381	3.87	-	0	1840.02829	3	614.0142822	-7.716115216	36.50950833
														GVPTGFILPIR	6	1	1	N-Term(iTRAQ8plex)	1.012	5.349	2.976	3.81	43	0	1473.89911	3	491.9712219	-7.068854596	45.18933167
														MHGGGPTVTAGLPLPK	1	1	1	N-Term(iTRAQ8plex); M1(Oxidation); K16(iTRAQ8plex)	1.081	1.372	1.136	3.74	-	0	2157.22098	3	719.7451782	-4.684212753	25.302385
														WMIQYNNLNLK	2	1	1	N-Term(iTRAQ8plex); K11(iTRAQ8plex)	0.471	3.056	1.603	3.71	53	0	2045.13187	3	682.3821411	-7.031923157	66.06442
														GDLNDCFIPCTPK	4	1	1	N-Term(iTRAQ8plex); C6(Methylthio); C10(Methylthio); K13(iTRAQ8plex)	1.119	7.823	4.366	3.7	50	0	2123.01079	2	1062.009033	-6.897522057	37.62713667
														TDTESELDLISR	4	1	1	N-Term(iTRAQ8plex)	0.84	3.184	2.048	3.7	45	0	1682.86968	2	841.9384766	-3.193827232	30.788975
														VLLSALER	6	1	1	N-Term(iTRAQ8plex)	1.281	9.15	5.33	3.09	31	0	1204.75627	2	602.8817749	-0.355354292	35.58867833
														TPVPSDIDISR	1	1	1	N-Term(iTRAQ8plex)	0.751	7.131	3.587	-	40	0	1503.82756	2	752.4174194	-2.991734508	25.06746667
														EISAQIR	1	1	1	N-Term(iTRAQ8plex)	0.939	5.79	3.601	-	36	0	1120.6644	3	374.2263184	1.437119018	21.52905333
														GALALAQAVQR	1	1	1	N-Term(iTRAQ8plex)	0.521	2.268	1.576	-	31	0	1401.84014	2	701.4237061	-5.603927599	23.23677
222352111	NIFK	MKI67 FHA domain-interacting nucleolar phosphoprotein	NP_115766.3	23.21	4	4	14	293	34.2	9.88	1.636	0.953	0.832																
														HLPNLLDETQIFSYFSQFGTVTR	8	1	1	N-Term(iTRAQ8plex)	1.815	1.201	1.317	5.97	65	0	3017.577	3	1006.530518	3.150933473	64.66297
														EEIQETQTPTHSR	4	1	1	N-Term(iTRAQ8plex)	1.225	0.874	0.739	5.87	46	0	1859.9357	3	620.6500854	-2.36470666	11.57094
														SQVAELNDDDKDDEIVFK	1	1	1	N-Term(iTRAQ8plex); K11(iTRAQ8plex); K18(iTRAQ8plex)	1.703	0.667	0.656	4.56	-	1	2992.58091	3	998.1984863	-2.327611465	28.19911833
														IVAETMNNYLFGER	1	1	1	N-Term(iTRAQ8plex)	1.636	0.965	0.927	2.94	-	0	1961.01335	2	981.0103149	1.479828175	53.673365
222352151	PCBP1	poly(rC)-binding protein 1	NP_006187.2	29.21	5	7	25	356	37.5	7.09	0.736	1.632	1.886																
														GYWASLDASTQTTHELTIPNNLIGCIIGR	6	1	1	N-Term(iTRAQ8plex); C25(Methylthio)	0.736	3.2	3.555	6.26	52	0	3494.75425	3	1165.591797	-3.040243809	64.53914833
														IITLTGPTNAIFK	4	1	1	N-Term(iTRAQ8plex); K13(iTRAQ8plex)	0.447	0.908	0.931	5.28	43	0	1997.21208	2	999.1096802	-6.748190914	36.08015167
														VMTIPYQPMPASSPVICAGGQDR	2	1	1	N-Term(iTRAQ8plex); C17(Methylthio)	-	-	-	3.74	45	0	2768.35892	3	923.4578247	1.545526146	45.08094
														IANPVEGSSGR	2	1	1	N-Term(iTRAQ8plex)	0.817	1.967	2.207	2.74	28	0	1390.75347	2	695.8803711	-4.145761735	17.00539333
														GYWASLDASTQTTHELTIPNNLIGCIIGR	1	1	1	N-Term(iTRAQ8plex); Y2(iTRAQ8plex); C25(Methylthio)	-	-	-	-	31	0	3798.973	3	1165.591797	-3.040243809	64.53914833
														QGANINEIR	1	1	1	N-Term(iTRAQ8plex)	0.775	1.353	1.612	-	30	0	1318.73296	2	659.8701172	-3.892785852	16.78303333

														INISEGNCPER	7	10	2	N-Term(iTRAQ8plex); C8(Methylthio)	-	-	-	4	39	0	1581.76238	2	791.3848267	-2.734670992	25.71615
														QMSGAQIK	2	10	2	N-Term(iTRAQ8plex); M2(Oxidation); K8(iTRAQ8plex)	-	-	-	3.29	30	0	1486.83918	3	496.2845764	-7.81543972	11.07629333
222831610	SLC44A2	choline transporter-like protein 2 isoform 2	NP_001138528.1	3.13	1	2	3	704	79.8	8.53	1.590	0.975	0.723																
														KPDDLPAFPLFSAFGR	2	4	1	N-Term(iTRAQ8plex); K1(iTRAQ8plex)	1.59	0.975	0.723	4.4	32	0	2386.33939	3	796.117981	0.58177856	52.67815333
														VYLHLR	1	6	2	N-Term(iTRAQ8plex)	-	-	-	-	23	0	1104.68808	3	368.9008789	4.500970749	20.21731
223029410	TLN1	talin-1	NP_006280.3	5.04	9	9	20	2541	269.6	6.07	0.578	1.157	1.570																
														VAGSVTELIQAAEAMK	2	1	1	N-Term(iTRAQ8plex); K16(iTRAQ8plex)	0.646	1.365	2.389	4.96	39	0	2226.27115	3	742.7619019	3.908336322	61.926085
														QEDVIATANLSR	2	4	1	N-Term(iTRAQ8plex)	0.552	1.157	1.463	4.92	43	0	1620.88291	3	540.9658203	-1.840732903	26.70488167
														GVGAAATAVTQALNELLQHVK	2	1	1	N-Term(iTRAQ8plex); K21(iTRAQ8plex)	-	-	-	4.41	51	0	2699.56386	4	675.6464233	-0.977128439	68.895925
														ILAQATSDLVNAIK	2	1	1	N-Term(iTRAQ8plex); K14(iTRAQ8plex)	0.605	0.781	1.666	4.33	44	0	2065.23355	3	689.0827026	-6.878950718	52.09055333
														LHTDDELNWLDHGR	1	1	1	N-Term(iTRAQ8plex)	0.976	2	1.012	3.74	-	0	2025.00785	3	675.6741333	-0.668959213	28.91960167
														EAAYHPEVAPDVR	2	1	1	N-Term(iTRAQ8plex)	0.519	0.684	0.918	3.68	33	0	1757.90879	3	586.6411133	-2.079007897	18.26384833
														TLAESALQLLYTAK	4	1	1	N-Term(iTRAQ8plex); K14(iTRAQ8plex)	-	0.597	1.553	3.48	28	0	2130.25962	3	710.7580566	-1.616999214	50.57815667
														FLPSELRDEH	4	1	1	N-Term(iTRAQ8plex)	0.452	1.384	2.171	3.42	29	1	1546.81015	3	516.2749023	-4.258776511	26.835275
														GAAAHPDSEEQQQR	1	1	1	N-Term(iTRAQ8plex)	0.87	0.876	1.553	-	20	0	1827.88218	3	609.9655762	-3.597624871	8.455583333
223468595	ITGAV	integrin alpha-V isoform 2 precursor	NP_001138471.1	7.58	6	6	14	1002	111.1	5.62	0.504	0.352	0.384																
														IYIGDDNPLTLIVK	2	3	1	N-Term(iTRAQ8plex); K14(iTRAQ8plex)	0.324	0.112	0.312	6.01	77	0	2182.31011	2	1091.658691	7.210200079	48.778945
														ILACAPLYHWR	6	3	1	N-Term(iTRAQ8plex); C4(Methylthio)	0.504	0.352	0.354	4.64	45	0	1692.90342	3	564.9726563	0.83477887	51.80175167
														LEVSVDSDQK	2	3	1	N-Term(iTRAQ8plex); K10(iTRAQ8plex)	0.547	0.37	0.416	4.41	46	0	1727.96335	3	576.6593018	-0.13268601	29.35028333
														VSPVVSHK	2	3	1	N-Term(iTRAQ8plex); K8(iTRAQ8plex)	0.723	0.556	0.53	3.26	28	0	1460.90403	3	487.6395264	-0.345983328	17.13574333
														DLALSEGDIHTLGCGVAQCLK	1	3	1	N-Term(iTRAQ8plex); C14(Methylthio); C19(Methylthio); K21(iTRAQ8plex)	-	-	0.296	3.18	-	0	2843.43838	4	711.6150513	-0.034501143	54.403995
														CQPIEFDATGNR	1	3	1	N-Term(iTRAQ8plex); C1(Methylthio)	0.343	0.238	0.477	-	36	0	1700.79753	2	850.9024048	-3.69788265	23.77042333
223717983	SLC37A2	sugar phosphate exchanger 2 isoform 2	NP_001138762.1	2.20	1	1	2	501	54.4	6.81	0.079	0.086	0.113																
														SSLAPGVWFFR	2	2	1	N-Term(iTRAQ8plex)	0.079	0.086	0.113	3.4	31	0	1570.86345	2	785.9353638	-3.145009794	38.49031167
223718097	OXA1L	mitochondrial inner membrane protein OXA1L	NP_005006.3	4.44	2	2	4	495	55.2	9.69	0.870	0.575	0.558																
														CLIFPLIVTGQR	2	1	1	N-Term(iTRAQ8plex); C1(Methylthio)	0.937	0.522	0.414	3.47	37	0	1709.98076	2	855.4940186	3.45512038	58.10634667
														LAGDHIEYYK	2	1	1	N-Term(iTRAQ8plex); K10(iTRAQ8plex)	0.808	0.633	0.752	3.06	25	0	1817.00077	3	606.3384399	-2.528741796	21.76485
223718134	CDK6	cyclin-dependent kinase 6	NP_001138778.1	4.60	1	1	2	326	36.9	6.46	-	-	-																
														FVTDIDELGKDLLLK	2	1	1	N-Term(iTRAQ8plex); K10(iTRAQ8plex); K15(iTRAQ8plex)	-	-	-	4.31	21	1	2631.57583	4	658.6494141	0.82652758	63.70332
223941782	SIN3A	paired amphipathic helix protein Sin3a	NP_001138829.1	1.18	1	1	4	1273	145.1	7.25	-	-	-																
														QILEDAAALIIHHVK	4	1	1	N-Term(iTRAQ8plex); K15(iTRAQ8plex)	-	-	-	5.95	50	0	2279.36147	4	570.5958252	-3.571350509	55.70815333
223972653	ALDH16A1	aldehyde dehydrogenase family 16 member A1 isoform 2	NP_001138868.1	3.33	2	2	4	751	79.9	7.58	0.594	1.445	1.999																
														VQAQGHTLQVAGLR	2	3	1	N-Term(iTRAQ8plex)	0.232	1.049	1.325	4.51	37	0	1782.02359	3	594.6793823	-2.927894649	21.55030333
														AAEAEVELSAR	2	3	1	N-Term(iTRAQ8plex)	1.522	1.992	3.015	3.78	29	0	1449.78655	2	725.3969116	0.983125544	26.808115
224028244	NONO	non-POU domain-containing octamer-binding protein isoform 1	NP_001138880.1	32.06	12	14	81	471	54.2	8.95	1.304	0.767	0.931																
														MGQMAMGGAMGINNR	4	2	1	N-Term(iTRAQ8plex); M1(Oxidation)	1.354	0.399	0.542	6.67	50	0	1858.87864	3	620.2977295	4.717320645	25.35164833
														NLPQYVSNELLEEAFSVFGQVER	9	2	1	N-Term(iTRAQ8plex)	-	-	-	5.03	87	0	2972.52665	3	991.5137329	-1.394712319	72.44835333
														FAQPGSFEYEYAMR	14	2	1	N-Term(iTRAQ8plex)	1.274	0.767	0.931	4.99	55	0	1999.95183	2	1000.479065	-0.869039949	35.79203333
														MGQMAMGGAMGINNR	2	2	1	N-Term(iTRAQ8plex)	0.382	0.108	0.148	4.42	24	0	1842.88298	3	620.2977295	4.717320645	25.35164833
														LFVGNLPPDITEEEMR	7	1	1	N-Term(iTRAQ8plex)	1.875	0.68	0.887	4.38	75	0	2164.1209	2	1082.564087	-2.469405421	46.71883333
														FACHSASLTVR	12	2	1	N-Term(iTRAQ8plex); C3(Methylthio)	1.157	0.727	0.827	3.97	32	0	1541.78446	3	514.5996704	-1.669701311	32.29101
														MEELHNQEVQK	2	2	1	N-Term(iTRAQ8plex); M1(Oxidation); K11(iTRAQ8plex)	1.171	0.637	0.947	3.67	32	0	2009.05533	3	670.3566284	-1.44560284	14.71956
														GIVEFSGKPAAR	2	2	1	N-Term(iTRAQ8plex); K8(iTRAQ8plex)	1.618	0.712	0.937	3.61	36	0	1840.07768	2	920.5424805	-6.769813365	19.140985
														AGEVFIHK	6	2	1	N-Term(iTRAQ8plex); K8(iTRAQ8plex)	1.754	0.846	1.084	3.27	37	0	1508.90407	2	754.9521484	-4.984465407	23.83486
														VELDNMPLR	5	2	1	N-Term(iTRAQ8plex)	1.257	0.748	0.892	3.03	42	0	1390.76164	3	464.2563171	-8.796041918	22.95714167
														RMEELHNQEVQK	1	2	1	N-Term(iTRAQ8plex); K12(iTRAQ8plex)	1.506	1.025	1.417	2.83	-	1	2149.17496	3	717.0631714	4.894720229	13.41245
														EKLEMEMEAAR	1	2	1	N-Term(iTRAQ8plex); K2(iTRAQ8plex)	1.559	0.879	0.988	2.8	-	1	1945.0217	3	649.012085	-6.508753695	22.70132667
														TFNLEK	2	1	1	N-Term(iTRAQ8plex); K6(iTRAQ8plex)	1.584	0.935	1.081	2.73	-	0	1359.81206	2	680.409668	2.079712031	21.681965
														FAQPGSFEYEYAMR	1	2	1	N-Term(iTRAQ8plex); M13(Oxidation)	1.572	0.713	0.926	-	40	0	2015.94451	2	1000.479065	-0.869039949	35.79203333
														LFVGNLPPDITEEEMR	1	1	1	N-Term(iTRAQ8plex); M15(Oxidation)	0.878	1.315	1.048	-	31	0	2180.11814	2	1082.564087	-2.469405421	46.71883333
														GFGFIR	1	9	2	N-Term(iTRAQ8plex)	-	-	-	-	25	0	1000.58647	2	500.796875	-1.696924799	23.23995667
														AVVIVDDR	11	5	2	N-Term(iTRAQ8plex)	-	-	-	3.22	53	0	1190.706	3	397.5735168	1.113614202	25.177715
224465192	SCRN1	secernin-1 isoform c	NP_001138987.1	8.38	2	2	3	346	38.8	4.70	0.951	0.875	2.085																
														MDAEHPELR	2	3	1	N-Term(iTRAQ8plex)	0.626	0.739	1.315	3.12	26	0	1401.70099	3	467.9051819	-6.312969564	14.35703333
														DSLEKQEESITVQTMMNTLR	1	3	1	N-Term(iTRAQ8plex); K5(iTRAQ8plex)	1.445	1.036	3.306	3.1	-	1	2961.53904	3	987.8511963	-3.10165853	46.22264167
224586817	GLG1	Golgi apparatus protein 1 isoform 3 precursor	NP_001139139.1	8.31	7	7	13	1179	134.5	6.90	0.239	0.278	0.576																
														ALNEACESVIQTACK	4	3	1	N-Term(iTRAQ8plex); C6(Methylthio); C14(Methylthio); K15(iTRAQ8plex)	0.216	0.179	0.461	4.89	50	0	2280.13089	3	760.7151489	-0.350757344	53.15321333
														VAELSSDDFHLDR	2	3	1	N-Term(iTRAQ8plex)	0.615	0.501	0.683	4.84	52	0	1807.90683	3	603.3071289	-3.323086753	32.55167833
														ADIFVDPVLHTACALDIK	1	3	1	N-Term(iTRAQ8plex); C13(Methylthio); K18(iTRAQ8plex)	-	-	0.636	4.4	-	0	2595.41787	4	649.6099243	1.662578377	59.30215167
														QVSSECQGEMLDYR	2	3	1	N-Term(iTRAQ8plex); C6(Methylthio)	0.563	0.5	0.602	4.35	25	0	1994.89482	2	997.9510498	1.223467697	31.64778667
														SGDPMILSCLMEHLYTEK	1	3	1	N-Term(iTRAQ8plex); C9(Methylthio); K18(iTRAQ8plex)	-	-	0.593	3.59	-	0	2721.35341	4	681.093811	-1.715337431	60.39289
														LLELQYFISR	1	3	1	N-Term(iTRAQ8plex)	0.25	0.247	0.413	3.47	-	0	1585.93791	2	793.4725952	7.795077966	57.72805333
														LICGFMDDCK	2	3	1	N-Term(iTRAQ8plex); C3(Methylthio); C9(Methylthio); K10(iTRAQ8plex)	0.229	0.253	0.432	3.43	37	0	1844.85514	3	615.62323	-7.9391646	79.386545
224589127	TMEM33	transmembrane protein 33	NP_060596.2	19.84	5	5	21	247	28.0	9.70	3.292	0.901	0.698																
														IVVEHIIMKPACPLFVR	3	1	1	N-Term(iTRAQ8plex); K9(iTRAQ8plex); C12(Methylthio)	3.466	0.862	0.894	4.56	-	0	2619.51853	3	873.8443604	0.891577846	50.53352667
														LCLQSIAFISR	4	1	1	N-Term(iTRAQ8plex); C2(Methylthio)	2.544	0.554	0.522	4.11	41	0	1600.89678	2	800.9520264	6.923503464	52.07600167
														ALLANALTSALR	5	1	1	N-Term(iTRAQ8plex)	4.998	0.996	0.766	3.96	45	0	1517.92925	2	759.4758301	8.341699727	45.78356667
														LPHFQLSR	8	1	1	N-Term(iTRAQ8plex)	3.128	1.091	0.717	3.27	32	0	1301.75837	3	434.5913696	-2.777520062	29.62508333
														RLCLQSIAFISR	1	1	1	N-Term(iTRAQ8plex); C3(Methylthio)	0.68	0.229	0.577	3.1	-	1	1756.98191	3	586.3321533	-2.792054399	45.40293833
224591430	LYAR	cell growth-regulating nucleolar protein	NP_001139197.1	3.17	1	1	2	379	43.6	9.57	0.828	0.943	0.469																
														NCECLSCIDCGK	2	1	1	N-Term(iTRAQ8plex); C2(Methylthio); C4(Methylthio); C7(Methylthio); C10(Methylthio); K12(iTRAQ8plex)	0.828	0.943	0.469	3.61	37	0	2079.83672	2	1040.421997	-4.124256151	74.09920667
224593278	DDX27	probable ATP-dependent RNA helicase DDX27	NP_060365.7	8.54	6	6	14	796	89.8	9.28	1.928	0.838	1.013																
														ELGIQVHSVTR	7	2	1	N-Term(iTRAQ8plex)	1.997	0.773	0.793	4.48	46	0	1542.88346	3	514.9660034	-4.611039195	20.78056167
														IFVNSNTDVAPFLR	2	2	1	N-Term(iTRAQ8plex)	1.928	0.894	1.15	4.47	29	0	1897.04741	2	949.0273438	-0.601155245	48.22812167
														TFTDHVMLFTQTK	1	2	1	N-Term(iTRAQ8plex); K13(iTRAQ8plex)	1.763	1.7	1.37	3.83	-	0	2177.189	3	726.4011841	0.219409566	34.72510833
														ALQEFDLALR	2	2	1	N-Term(iTRAQ8plex)	2.393	0.917	1.181	3.29	26	0	1479.83989	2	740.423584	-5.020780487	46.42941667
														AAPDILIATPGR	1	2	1	N-Term(iTRAQ8plex)	1.194	0.917	0.869	-	26	0	1498.88445	2	749.9458618	-3.392047464	30.23463167
														VLVLVPTR	1	2	1	N-Term(iTRAQ8plex)	2.607	0.617	1.02	-	20	0	1200.7968	2	600.9020386	-1.141551645	36.81066
224994203	WFS1	wolframin	NP_005996.2	2.70	2	2	4	890	100.2	8.05	0.753	0.499	0.951																
														DAAAPAEPQAQHTR	2	2	1	N-Term(iTRAQ8plex)	1.003	0.552	0.952	3.7	44	0	1766.90769	3	589.6407471	-0.607467538	9.612051667
														QLSSETDLER	2	2	1	N-Term(iTRAQ8plex)	0.565	0.45	0.949	3.49	28	0	1481.77666	2	741.3919678	1.165915838	22.50798333
225007648	POP1	ribonucleases P/MRP protein subunit POP1	NP_055844.2	4.00	2	2	3	1024	114.6	9.22	0.869	0.524	0.405																
														VNPHSLPDPEVNEQSSSK	2	2	1	N-Term(iTRAQ8plex); K18(iTRAQ8plex)	0.869	0.524	0.405	5.5	38	0	2572.33481	3	858.1164551	-4.493705707	21.127385
														QLLLEGVPVECTHSFIWNQDICK	1	1	1	N-Term(iTRAQ8plex); C11(Methylthio); C22(Methylthio); K23(iTRAQ8plex)	-	-	-	4.4	-	0	3372.71035	4	843.9330444	0.878982797	62.89722
225543097	PAFAH1B3	platelet-activating factor acetylhydrolase IB subunit gamma	NP_001139411.1	3.90	1	1	3	231	25.7	6.84	0.585	1.395	3.611																
														VVVLGLLPR	3	1	1	N-Term(iTRAQ8plex)	0.585	1.395	3.611	2.79	26	0	1269.85515	2	635.4312134	-0.693907638	42.70932833
225543166	SAMM50	sorting and assembly machinery component 50 homolog	NP_056195.3	4.48	2	2	7	469	51.9	6.90	1.170	0.832	0.852																
														DVVVQHVHFDGLGR	6	1	1	N-Term(iTRAQ8plex)	1.186	0.915	1.067	3.11	38	0	1882.0143	4	471.2590332	-5.009721491	25.85067833
														TASFAVR	1	1	1	N-Term(iTRAQ8plex)	1.154	0.756	0.68	-	22	0	1055.60893	2	528.3081055	-5.851582247	23.52829
225703079	MBOAT7	lysophospholipid acyltransferase 7 isoform 1	NP_077274.3	15.25	5	5	15	472	52.7	8.97	1.298	0.779	0.739																
														LVSLASEVQDLHLAQR	2	3	1	N-Term(iTRAQ8plex)	1.763	1.404	1.087	5.97	88	0	2083.1865	3	695.0670166	2.465095994	44.628645
														NIDCYSTDFCVR	2	4	1	N-Term(iTRAQ8plex); C4(Methylthio); C10(Methylthio)	1.072	0.772	0.753	4.97	35	0	1831.79314	2	916.4002075	7.765679849	39.894225
														TYLDWLEQPFPGAVPSLRPLLR	3	4	1	N-Term(iTRAQ8plex)	1.133	0.59	0.595	3.71	29	0	2872.6083	3	958.2076416	1.94605528	61.72449167
														AWDWVHWFLK	4	2	1	N-Term(iTRAQ8plex); K10(iTRAQ8plex)	1.338	0.786	0.726	3.62	24	0	1996.10048	3	666.0419922	3.0268914	61.568715
														AASLEYDYETIR	4	4	1	N-Term(iTRAQ8plex)	1.259	0.864	0.815	3.44	38	0	1734.87932	2	867.9432983	-3.389999664	32.27045667
225703102	MFSD10	major facilitator superfamily domain-containing protein 10	NP_001111.3	2.86	1	1	2	455	48.3	9.60	0.526	0.263	0.490																
														DAADLLSPLALLR	2	2	1	N-Term(iTRAQ8plex)	0.526	0.263	0.49	4.24	41	0	1671.9904	2	836.4988403	-2.588086586	58.69864
225903399	CYP51A1	lanosterol 14-alpha demethylase isoform 2	NP_001139624.1	15.10	3	3	6	404	46.3	8.50	0.677	0.365	0.520																
														TFTYLLGSDAAALLFNSK	2	2	1	N-Term(iTRAQ8plex); K18(iTRAQ8plex)	0.677	0.365	0.638	7.1	45	0	2540.43637	3	847.4836426	5.618398681	61.91608
														IDDILQTLLDATYK	2	2	1	N-Term(iTRAQ8plex); K14(iTRAQ8plex)	-	-	0.424	3.46	32	0	2230.29563	3	744.1033936	7.405347462	66.39326667
														LYEFDLIDGYFPTVNYTTMIHTPENPVIR	2	2	1	N-Term(iTRAQ8plex)	-	-	-	2.92	41	0	3762.92026	3	1254.978271	4.539002142	64.68034333
226371643	P3H1	prolyl 3-hydroxylase 1 isoform 2 precursor	NP_001139761.1	1.43	1	1	2	697	78.9	5.58	0.420	0.239	0.338																
														DLSFFGGLLR	2	3	1	N-Term(iTRAQ8plex)	0.42	0.239	0.338	3.07	45	0	1428.81731	2	714.9122925	1.411685683	49.34089833
226371718	PARP9	poly [ADP-ribose] polymerase 9 isoform c	NP_001139578.1	2.25	1	1	2	710	80.2	8.06	1.340	-	-																
														ETAAEILFDEVLTFAK	2	4	1	N-Term(iTRAQ8plex); K16(iTRAQ8plex)	1.34	-	-	3.22	32	0	2405.35013	3	802.454895	3.184779003	65.826155
226437577	SUMF2	sulfatase-modifying factor 2 isoform g	NP_001139805.1	10.33	2	2	5	213	24.1	6.93	1.121	1.005	1.494																
														SVLWWLPVEK	2	5	1	N-Term(iTRAQ8plex); K10(iTRAQ8plex)	1.009	0.834	1.494	3.85	42	0	1865.0988	2	933.0530396	-8.443959332	75.24565
														LPTEEEWEFAAR	3	7	1	N-Term(iTRAQ8plex)	1.141	1.178	1.599	3.09	29	0	1781.89995	2	891.4536133	-0.713484222	38.30724
226437606	ANKRD34C	ankyrin repeat domain-containing protein 34C	NP_001139813.1	2.80	1	1	1	535	58.2	9.03	9.306	2.823	1.904																
														TGASALVYAINADDK	1	1	1	N-Term(iTRAQ8plex); K15(iTRAQ8plex)	9.306	2.823	1.904	3.16	-	0	2117.17215	3	706.3955688	1.063679666	29.95603333
227430301	CD109	CD109 antigen isoform 2 preproprotein	NP_001153059.1	7.63	7	7	14	1428	159.6	5.80	0.738	0.292	0.312																
														CFLEADPYIDIDQNVLHR	2	5	1	N-Term(iTRAQ8plex); C1(Methylthio)	0.912	0.325	0.897	5.9	77	0	2511.23655	3	837.7503662	2.036081715	44.68384833
														ENKDDLNHVDLNVCTSFSGPGR	2	5	1	N-Term(iTRAQ8plex); K3(iTRAQ8plex); C14(Methylthio)	0.738	-	-	4.47	28	1	3071.50405	4	768.6314697	-3.097268829	35.34056667
														GDVTLTFLPLSFWGK	3	5	1	N-Term(iTRAQ8plex); K15(iTRAQ8plex)	0.804	0.333	0.311	4.23	52	0	2289.30997	2	1145.166626	6.815994442	59.90085167
														IVTLFSDFKPYK	2	3	1	N-Term(iTRAQ8plex); K9(iTRAQ8plex); K12(iTRAQ8plex)	0.372	0.114	0.098	4.17	30	0	2370.41958	4	593.3603516	-0.183482285	52.23263833

														NNVVITVTQR	2	5	1	N-Term(iTRAQ8plex)	0.612	0.263	0.254	3.31	28	0	1447.8532	2	724.4302368	-0.175630504	25.11404
														VGSPFELVVSGNKR	1	5	1	N-Term(iTRAQ8plex); K13(iTRAQ8plex)	0.814	0.611	0.699	2.81	-	1	2097.2219	3	699.7454834	-2.767639751	30.18908
														SPVTLTAYIVTSLLGYRK	2	5	1	N-Term(iTRAQ8plex); K18(iTRAQ8plex)	-	-	-	2.75	35	1	2590.54848	4	648.3925781	2.166074111	70.66508333
229577398	BZW2	basic leucine zipper and W2 domain-containing protein 2	NP_001153239.1	2.15	1	1	1	419	48.1	6.68	2.357	4.035	3.012																
														LLELFPVNR	2	1	1	N-Term(iTRAQ8plex)	2.357	4.035	3.012	3.29	23	0	1404.85845	2	702.9328613	4.822362188	49.25188667
229892270	DCAF13	DDB1- and CUL4-associated factor 13	NP_056235.4	4.19	2	2	4	597	67.5	9.29	1.462	0.862	0.763																
														EFVSASFDK	2	1	1	N-Term(iTRAQ8plex); K9(iTRAQ8plex)	1.462	0.862	0.763	3.69	32	0	1637.9008	2	819.4540405	0.783401952	35.54640167
														FNPIETFLLGSCASDR	2	1	1	N-Term(iTRAQ8plex); C12(Methylthio)	-	-	-	2.91	27	0	2120.05387	3	707.3561401	3.775781493	67.137385
237649015	EBNA1BP2	probable rRNA-processing protein EBP2 isoform 2	NP_006815.2	15.69	5	5	9	306	34.8	10.10	1.577	0.727	0.721																
														GLLKPGLNVVLEGPK	2	2	1	N-Term(iTRAQ8plex); K4(iTRAQ8plex); K15(iTRAQ8plex)	2.156	0.684	0.558	4.09	50	0	2446.54228	2	1223.77478	-4.159900767	37.03722
														QCLAEFK	3	2	1	N-Term(iTRAQ8plex); C2(Methylthio); K7(iTRAQ8plex)	0.771	0.543	0.651	3.52	26	0	1492.81134	3	498.2752991	0.07480302	31.62625667
														ESYDDVSSFR	2	2	1	N-Term(iTRAQ8plex)	1.59	0.773	0.794	2.78	37	0	1508.71111	2	754.8591919	-3.953129866	17.37169333
														FGFGGK	1	2	1	N-Term(iTRAQ8plex); K6(iTRAQ8plex)	1.565	1.221	1.017	-	24	0	1220.72486	3	407.5798035	0.062135534	22.61625833
														AVDPEDDFQR	1	2	1	N-Term(iTRAQ8plex)	1.809	1.048	1.023	-	21	0	1495.73052	2	748.3688965	-1.714848512	21.44973167
237649049	SNRPD2	small nuclear ribonucleoprotein Sm D2 isoform 2	NP_808210.2	44.44	5	5	27	108	12.4	9.89	0.981	0.900	0.882																
														REEEEFNTGPLSVLTQSVK	12	2	1	N-Term(iTRAQ8plex); K19(iTRAQ8plex)	0.842	0.918	0.882	5.9	53	1	2771.49442	2	1386.250854	-3.315516538	41.04457667
														EEEEFNTGPLSVLTQSVK	2	2	1	N-Term(iTRAQ8plex); K18(iTRAQ8plex)	1.237	1.589	1.162	5.3	26	0	2615.39627	3	872.4702759	-2.380867994	37.88912833
														HCNMVLENVK	2	2	1	N-Term(iTRAQ8plex); C2(Methylthio); K10(iTRAQ8plex)	1.507	1.338	1.053	4.44	43	0	1840.96306	2	920.9851685	-3.347164357	23.36971667
														NNTQVLINCR	5	2	1	N-Term(iTRAQ8plex); C9(Methylthio)	1.075	0.912	0.964	3.82	49	0	1524.78777	2	762.897522	-3.328315452	33.37576333
														GDSVIVVLR	6	2	1	N-Term(iTRAQ8plex)	0.868	0.651	0.709	3.5	40	0	1261.78215	2	631.3947144	3.154895582	29.774645
238624149	NPTN	neuroplastin isoform d precursor	NP_001154836.1	6.83	2	2	6	278	30.8	7.84	0.993	0.502	0.524																
														IVTSEEVIIR	4	5	1	N-Term(iTRAQ8plex)	1.012	0.485	0.51	3.61	48	0	1462.87786	2	731.9425659	-0.291264278	32.499405
														AAPDITGHK	2	5	1	N-Term(iTRAQ8plex); K9(iTRAQ8plex)	0.899	0.726	0.599	3.33	31	0	1517.89139	3	506.6353149	1.166271993	17.32786
238776833	ALYREF	THO complex subunit 4	NP_005773.3	18.18	3	3	21	264	27.5	11.05	1.126	1.260	0.857																
														SLGTADVHFER	18	1	1	N-Term(iTRAQ8plex)	1.119	1.349	0.918	4.75	69	0	1535.80735	3	512.6072998	-3.0245564	25.800455
														MDMSLDDIIK	2	1	1	N-Term(iTRAQ8plex); K10(iTRAQ8plex)	1.132	0.957	0.708	3.89	52	0	1788.95354	2	894.9804077	-8.989887084	65.03226333
														LLVSNLDFGVSDADIQELFAEFGTLKK	1	1	1	N-Term(iTRAQ8plex); K26(iTRAQ8plex); K27(iTRAQ8plex)	-	-	-	-	50	1	3882.17451	5	777.2407227	1.969378563	68.07658333
238859541	ALDH4A1	delta-1-pyrroline-5-carboxylate dehydrogenase, mitochondrial isoform b	NP_001154976.1	3.78	1	1	2	503	55.1	6.80	0.330	0.103	0.475																
														VGDPAEDFGTFFSAVIDAK	2	3	1	N-Term(iTRAQ8plex); K19(iTRAQ8plex)	0.33	0.103	0.475	5.87	54	0	2594.38053	3	865.4650269	7.938702973	63.80552167
239985428	PDP1	pyruvate dehyrogenase phosphatase catalytic subunit 1 isoform 3	NP_001155253.1	6.89	3	3	6	537	61.0	6.67	1.246	0.741	0.711																
														FLVLATDGLWETMHR	2	5	1	N-Term(iTRAQ8plex)	0.956	2.099	1.302	4.46	70	0	2093.12162	3	698.3787231	2.873642672	66.30510667
														VTLGQMHGLLTER	2	5	1	N-Term(iTRAQ8plex)	1.246	0.741	0.711	3.35	29	0	1758.98453	3	586.998291	-2.002013943	30.239315
														LLGLLMPFR	2	5	1	N-Term(iTRAQ8plex)	1.446	0.569	0.401	3.21	27	0	1363.8477	2	682.4274902	2.890754399	64.78545167
251831109	COX1	cytochrome c oxidase subunit I (mitochondrion)	YP_003024028.1	7.60	2	2	3	513	57.0	6.70	0.831	0.530	0.731																
														VLMVEEPSMNLEWLYGCPPPYHTFEEPVYMK	1	1	1	N-Term(iTRAQ8plex); C17(Methylthio); K31(iTRAQ8plex)	-	-	-	4.07	-	0	4383.12221	4	1096.536011	1.193393315	64.42227833
														WLFSTNHK	2	1	1	N-Term(iTRAQ8plex); K8(iTRAQ8plex)	0.831	0.53	0.731	2.98	27	0	1640.95182	3	547.6554565	9.095379132	28.31592833
251831110	COX2	cytochrome c oxidase subunit II (mitochondrion)	YP_003024029.1	20.26	3	3	18	227	25.5	4.82	1.407	0.755	0.859																
														MMITSQDVLHSWAVPTLGLK	6	1	1	N-Term(iTRAQ8plex); K20(iTRAQ8plex)	1.254	1.065	0.872	5.69	47	0	2835.56723	3	945.8605957	-1.7483065	55.22847833
														MMITSQDVLHSWAVPTLGLK	2	1	1	N-Term(iTRAQ8plex); M2(Oxidation); K20(iTRAQ8plex)	1.601	1.112	1.319	5.04	37	0	2851.55985	3	945.8605957	-1.7483065	55.22847833
														MMITSQDVLHSWAVPTLGLK	3	1	1	N-Term(iTRAQ8plex); M1(Oxidation); M2(Oxidation); K20(iTRAQ8plex)	1.256	0.662	0.749	4.24	-	0	2867.5499	3	945.8605957	-1.7483065	55.22847833
														ILYMTDEVNDPSLTIK	2	1	1	N-Term(iTRAQ8plex); M4(Oxidation); K16(iTRAQ8plex)	1.349	0.293	0.49	4.18	53	0	2476.34177	3	826.1187744	-1.932813908	36.74098333
														ILYMTDEVNDPSLTIK	2	1	1	N-Term(iTRAQ8plex); K16(iTRAQ8plex)	1.04	1.182	1.411	3.9	49	0	2460.36142	3	826.1187744	-1.932813908	36.74098333
														VVLPIEAPIR	3	1	1	N-Term(iTRAQ8plex)	1.672	0.558	0.788	2.79	42	0	1410.89884	3	470.9711304	0.146405451	38.17356
251831112	ATP6	ATP synthase F0 subunit 6 (mitochondrion)	YP_003024031.1	4.42	1	1	4	226	24.8	10.10	0.581	0.473	0.430																
														LITTQQWLIK	4	1	1	N-Term(iTRAQ8plex); K10(iTRAQ8plex)	0.581	0.473	0.43	4.57	59	0	1852.14336	2	926.5753174	-4.477598528	33.82842833
251831116	ND4	NADH dehydrogenase subunit 4 (mitochondrion)	YP_003024035.1	4.79	2	2	4	459	51.5	9.35	1.019	0.787	0.590																
														MPLYGLHLWLPK	2	1	1	N-Term(iTRAQ8plex); K12(iTRAQ8plex)	0.9	0.619	0.401	3.65	-	0	2076.2206	4	519.8106079	-3.982977227	51.64021667
														LTLILNPLTK	2	1	1	N-Term(iTRAQ8plex); K10(iTRAQ8plex)	1.019	0.787	0.59	3.53	38	0	1734.13675	3	578.7171021	1.044670982	41.05344333
251831117	ND5	NADH dehydrogenase subunit 5 (mitochondrion)	YP_003024036.1	3.32	1	1	1	603	67.0	9.03	-	-	-																
														FPTLTNINENNPTLLNPIKR	1	1	1	N-Term(iTRAQ8plex); K19(iTRAQ8plex)	-	-	-	3.39	-	1	2917.68203	4	730.1759644	3.429517604	41.67716333
254540132	COPS9	myeloma-overexpressed gene 2 protein isoform 2	NP_001156896.1	35.09	1	1	2	57	6.2	3.83	0.493	1.100	0.483																
														AVHADFFNDFEDLFDDDDIQ	2	1	1	N-Term(iTRAQ8plex)	0.493	1.1	0.483	4.43	29	0	2692.21396	3	898.0761719	5.427159656	63.05264333
255653002	PYGL	glycogen phosphorylase, liver form isoform 2	NP_001157412.1	13.41	5	7	23	813	93.1	7.30	0.881	2.536	1.908																
														DGWQVEEADDWLR	4	2	1	N-Term(iTRAQ8plex)	0.924	2.355	1.848	5.32	48	0	1922.9229	2	961.9650879	2.198663946	39.74056833
														DFNVGDYIQAVLDR	4	2	1	N-Term(iTRAQ8plex)	0.757	5.731	3.934	4.93	83	0	1929.00034	3	643.6716309	-0.85638755	58.251285
														LHSFLGDDVFLR	8	2	1	N-Term(iTRAQ8plex)	0.993	2.536	1.908	4.77	45	0	1722.94937	3	574.9879761	0.739239745	45.02208833
														GAGTVFDAFPDQVAIQLNDTHPALAIPELMR	1	2	1	N-Term(iTRAQ8plex)	-	-	-	3.27	-	0	3611.881	4	903.725708	-0.694693083	52.78158
														FSQFLETEYK	2	2	1	N-Term(iTRAQ8plex); K10(iTRAQ8plex)	0.89	1.969	1.682	3.08	32	0	1900.02188	3	634.012146	-4.929712544	65.0791
														VLYPNDNFFEGK	2	5	2	N-Term(iTRAQ8plex); K12(iTRAQ8plex)	-	-	-	2.85	34	0	2051.09543	3	684.3699951	-5.070070497	60.630785
														LKQEYFVVAATLQDIIR	2	5	2	N-Term(iTRAQ8plex); K2(iTRAQ8plex)	-	-	-	2.75	25	1	2615.53763	3	872.517395	-0.194794141	67.903125
255958306	PLIN3	perilipin-3 isoform 3	NP_001157666.1	18.48	4	4	22	422	45.8	5.43	0.587	1.121	2.234																
														LGQMVLSGVDTVLGK	8	3	1	N-Term(iTRAQ8plex); K15(iTRAQ8plex)	0.241	1.176	4.009	5.05	60	0	2125.24736	2	1063.127319	-1.782968041	56.16569667
														EALDHMVEYVAQNTPVTWLVGPFAPGITEK	4	3	1	N-Term(iTRAQ8plex); K30(iTRAQ8plex)	-	-	-	4.82	28	0	3921.07322	3	1307.695923	0.360616752	68.25933667
														LGQMVLSGVDTVLGK	2	3	1	N-Term(iTRAQ8plex); M4(Oxidation); K15(iTRAQ8plex)	0.786	1.069	1.4	4.52	28	0	2141.22818	2	1063.127319	-1.782968041	56.16569667
														DTVATQLSEAVDATR	2	3	1	N-Term(iTRAQ8plex)	0.806	0.763	1.234	4.08	48	0	1880.97893	2	940.993103	-4.153084966	37.11916333
														SEEWADNHLPLTDAELAR	6	3	1	N-Term(iTRAQ8plex)	0.587	1.266	2.166	3.67	59	0	2371.1834	2	1186.095337	0.069968491	33.54314
256222019	RAB10	ras-related protein Rab-10	NP_057215.3	39.00	7	7	28	200	22.5	8.38	1.124	0.492	0.937																
														NIDEHANEDVER	12	1	1	N-Term(iTRAQ8plex)	1.208	0.464	0.938	4.58	65	0	1744.84111	2	872.9241943	0.407344755	13.44404833
														AFLTLAEDILR	10	1	1	N-Term(iTRAQ8plex)	1.028	0.427	0.807	4.5	69	0	1565.91716	3	522.6471558	4.101554867	65.47115667
														GAMGIMLVYDITNGK	2	1	1	N-Term(iTRAQ8plex); K15(iTRAQ8plex)	1.403	1.527	2.776	4.02	34	0	2191.20591	3	731.0734863	-0.760113698	49.42844833
														FSDDAFNTTFISTIGIDFK	1	1	1	N-Term(iTRAQ8plex); K19(iTRAQ8plex)	0.549	0.859	1.198	3.57	-	0	2747.46384	3	916.4927979	9.080739962	60.790235
														ANINIEK	1	1	1	N-Term(iTRAQ8plex); K7(iTRAQ8plex)	1.337	1.369	1.316	-	30	0	1409.86365	3	470.6260681	4.539301157	14.59539
														KTYDLLFK	1	1	1	N-Term(iTRAQ8plex); K1(iTRAQ8plex); K8(iTRAQ8plex)	0.994	0.628	0.946	-	21	1	1940.1998	3	647.4047852	0.737511028	35.57952667
														TCVLFR	1	3	1	N-Term(iTRAQ8plex); C2(Methylthio)	1.108	0.577	0.879	-	18	0	1088.58183	2	544.7945557	-7.337612681	25.71588833
														IQIWDTAGQER	14	3	9	N-Term(iTRAQ8plex)	-	-	-	3.41	41	0	1620.85991	2	810.9328003	-3.971558752	26.96297167
														LLIIGDSGVGK	22	2	5	N-Term(iTRAQ8plex); K11(iTRAQ8plex)	-	-	-	4.71	52	0	1680.04277	3	560.6833496	-9.598441174	57.819735
256222415	FLNB	filamin-B isoform 4	NP_001157791.1	26.42	42	45	129	2578	275.5	5.78	0.526	0.723	1.754																
														GAGTGGLGLTVEGPCEAK	2	7	1	N-Term(iTRAQ8plex); C15(Methylthio); K18(iTRAQ8plex)	0.301	0.502	2.007	7.73	101	0	2271.19292	2	1136.100098	-0.208528081	33.07548167
														SPFTVGVAAPLDLSK	2	5	1	N-Term(iTRAQ8plex); K15(iTRAQ8plex)	0.229	0.784	1.842	6.82	54	0	2110.23978	3	704.0847778	1.378490786	38.34193333
														FNDEHIPESPYLVPVIAPSDDAR	10	5	1	N-Term(iTRAQ8plex)	1.164	1.282	2.359	6.35	50	0	2885.46347	3	962.4926758	0.37872382	37.14226333
														QKDFLDGVYAFEYYPSTPGR	2	5	1	N-Term(iTRAQ8plex); K2(iTRAQ8plex)	0.353	0.816	2.476	6.22	57	1	2961.51212	3	987.8422241	-4.123632194	43.984265
														APSVATVGSICDLNLK	2	5	1	N-Term(iTRAQ8plex); C11(Methylthio); K16(iTRAQ8plex)	0.379	0.48	1.774	5.48	55	0	2242.23355	3	748.0827026	-2.692689988	45.99740833
														TFEMSDFIVDTR	4	5	1	N-Term(iTRAQ8plex)	0.492	0.894	1.629	5.45	52	0	1764.89226	2	882.9497681	8.056136422	40.86635
														CLATGPGIASTVK	2	5	1	N-Term(iTRAQ8plex); C1(Methylthio); K13(iTRAQ8plex)	0.319	0.581	1.788	5.32	51	0	1872.03691	2	936.5220947	-9.31042906	25.15709
														GQHVTGSPFQFTVGPLGEGGAHK	2	5	1	N-Term(iTRAQ8plex); K23(iTRAQ8plex)	0.524	-	1.24	5.27	51	0	2916.57119	4	729.8982544	4.613773249	33.13327833
														GLHVVEVTYDDVPIPNSPFK	2	5	1	N-Term(iTRAQ8plex); K20(iTRAQ8plex)	1.388	1.124	1.951	5.1	39	0	2834.5369	3	945.5171509	-6.357974484	43.55709167
														VDIQTEDLEDGTCK	2	5	1	N-Term(iTRAQ8plex); C13(Methylthio); K14(iTRAQ8plex)	0.502	0.971	1.844	4.99	57	0	2220.10307	2	1110.555176	2.077670425	31.389155
														VSYFPTVPGVYIVSTK	6	5	1	N-Term(iTRAQ8plex); K16(iTRAQ8plex)	0.595	0.628	1.727	4.98	66	0	2365.3604	2	1183.183838	-1.002865674	46.92621
														AWGPGLHGGIVGR	3	5	1	N-Term(iTRAQ8plex)	0.488	0.895	1.748	4.87	53	0	1580.88718	3	527.6339111	-5.801268856	21.91198333
														VMYTPMAPGNYLISVK	2	5	1	N-Term(iTRAQ8plex); K16(iTRAQ8plex)	0.436	0.611	2.108	4.8	61	0	2392.33599	2	1196.671631	5.460687308	42.08300833
														GAGIGGLGITVEGPSESK	2	5	1	N-Term(iTRAQ8plex); K18(iTRAQ8plex)	1.034	0.937	1.631	4.79	37	0	2237.24118	3	746.4185791	-8.325988921	25.14540833
														AGSNMLLIGVHGPTTPCEEVSMK	4	5	1	N-Term(iTRAQ8plex); C17(Methylthio); K23(iTRAQ8plex)	-	0.935	2.479	4.75	29	0	3025.53774	4	765.1379395	-1.283509105	35.30877
														GEAGVPAEFSIWTR	2	5	1	N-Term(iTRAQ8plex)	0.319	0.76	2.044	4.72	47	0	1823.96037	2	912.4838257	0.52821717	39.00151167
														IGNLQTDLSDGLR	4	5	1	N-Term(iTRAQ8plex)	0.327	0.285	0.834	4.47	45	0	1705.93926	2	853.4732666	0.348947434	36.75419667
														EAFTNKPNVFTVVTR	3	5	1	N-Term(iTRAQ8plex); K6(iTRAQ8plex)	0.481	0.393	1.581	4.41	53	0	2331.31963	2	1166.163452	-3.65184723	31.19008333
														VLFASQEIPASPFR	4	5	1	N-Term(iTRAQ8plex)	0.503	0.714	1.616	4.23	57	0	1866.03421	3	622.6829224	-4.569830549	47.93114333
														IEYNDQNDGSCDVK	2	5	1	N-Term(iTRAQ8plex); C11(Methylthio); K14(iTRAQ8plex)	0.733	0.833	1.756	4.18	60	0	2254.04258	3	752.019043	-6.684276469	32.43419833
														EVGEHLVSIK	2	5	1	N-Term(iTRAQ8plex); K10(iTRAQ8plex)	0.463	0.486	1.891	4.13	39	0	1719.01958	2	860.0134277	-3.805920148	21.19730667
														VTASGPGLSSYGVPASLPVDFAIDAR	5	5	1	N-Term(iTRAQ8plex)	1.401	0.789	1.925	4.13	55	0	2851.52634	2	1426.261353	0.349694101	45.481675
														NTVELLVEDK	2	5	1	N-Term(iTRAQ8plex); K10(iTRAQ8plex)	0.618	0.628	1.491	4.12	56	0	1768.0147	2	884.5109863	-9.375071252	45.671405
														SGCIVNNLAEFTVDPK	2	5	1	N-Term(iTRAQ8plex); C3(Methylthio); K16(iTRAQ8plex)	0.678	0.495	0.782	4.11	38	0	2361.22922	3	787.7479248	-4.701422394	37.74692833
														AGSNMLLIGVHGPTTPCEEVSMK	2	5	1	N-Term(iTRAQ8plex); M5(Oxidation); C17(Methylthio); M22(Oxidation); [illegible]	-	-	-	3.99	42	0	3057.52993	4	765.1379395	-1.283509105	35.30877
														DGSCSAEYIPFAPGDYDVNITYGGAHIPGSPFR	2	5	1	N-Term(iTRAQ8plex); C4(Methylthio)	-	-	-	3.95	40	0	3823.78049	3	1275.265015	3.394126662	52.98604833
														RLPNNHIGISFIPR	1	5	1	N-Term(iTRAQ8plex)	0.738	0.517	0.914	3.94	-	1	1938.12746	4	485.287323	-3.348173915	35.63729333
														VLSEDEEDVDFDIIHNANDTFTVK	1	5	1	N-Term(iTRAQ8plex); K24(iTRAQ8plex)	-	-	-	3.9	-	0	3373.6852	4	844.1767578	-1.32493986	44.09871
														KVVPCLVTPVTGR	5	5	1	N-Term(iTRAQ8plex); K1(iTRAQ8plex); C5(Methylthio)	0.452	0.66	1.994	3.86	35	1	2023.19291	3	675.0678711	-6.238798439	29.75496167
														LIALLEVLSQKR	4	7	1	N-Term(iTRAQ8plex); K11(iTRAQ8plex)	-	-	3.71	3.84	43	1	1991.27578	4	498.5744019	-4.004346467	66.724875
														VAVTEGCQPSR	2	5	1	N-Term(iTRAQ8plex); C7(Methylthio)	0.427	0.509	1.754	3.77	32	0	1496.74748	2	748.8773804	-1.896230221	25.13508
														ALGALVDSCAPGLCPDWESWDPQKPVDNAR	1	5	1	N-Term(iTRAQ8plex); C9(Methylthio); C14(Methylthio); K24(iTRAQ8plex)	-	-	-	3.72	-	0	3910.90493	4	978.4816895	5.1892421	65.37879833
														YGGPNHIVGSPFK	2	5	1	N-Term(iTRAQ8plex); K13(iTRAQ8plex)	0.784	0.801	1.423	3.71	-	0	1981.09348	3	661.0360107	-9.137541277	20.92249667
														YAPTEVGLHEMHIK	2	5	1	N-Term(iTRAQ8plex); K14(iTRAQ8plex)	-	-	2.435	3.69	32	0	2233.21425	4	559.059021	-5.246785257	25.241575
														VTVLFAGQHISK	3	5	1	N-Term(iTRAQ8plex); K12(iTRAQ8plex)	0.746	0.913	1.469	3.4	40	0	1908.1351	3	636.7165527	-9.232232471	33.75213833
														VVPCLVTPVTGR	2	5	1	N-Term(iTRAQ8plex); C4(Methylthio)	0.445	0.725	1.588	3.3	51	0	1590.89531	2	795.9512939	-3.797251354	43.35140833
														YGGELVPHFPAR	5	5	1	N-Term(iTRAQ8plex)	0.737	0.735	0.916	3.24	32	0	1646.88896	3	549.6339111	-5.149807263	29.40320167
														EAGAGGLSIAVEGPSK	1	7	1	N-Term(iTRAQ8plex); K16(iTRAQ8plex)	1.035	1.213	0.931	3.16	-	0	2051.14322	3	684.3859253	-7.868134665	27.57359

														VKVEPAVDTSR	2	5	1	N-Term(iTRAQ8plex); K2(iTRAQ8plex)	0.232	0.393	1.624	3	33	1	1809.05803	2	905.0326538	-6.097263119	14.86612333
														YADEEIPR	2	5	1	N-Term(iTRAQ8plex)	0.897	0.755	1.585	2.7	32	0	1296.66692	2	648.8370972	-5.270115223	15.68962667
														AGLAPLEVR	1	5	1	N-Term(iTRAQ8plex)	0.292	0.557	1.856	-	39	0	1229.74614	2	615.376709	-4.732644449	26.755555
														AHIANPSGASTECFVTDN ADGTYQVEYTPFEK	1	5	1	N-Term(iTRAQ8plex); C13(Methylthio); K32(iTRAQ8plex)	-	-	-	-	29	0	4116.93715	4	1029.989746	-1.085225456	39.39189
														VNQPASFAIR	1	5	1	N-Term(iTRAQ8plex)	0.528	0.485	0.984	-	26	0	1406.79717	2	703.9022217	-6.123527136	18.47937
														LIALLEVLSQK	14	14	2	N-Term(iTRAQ8plex); K11(iTRAQ8plex)	-	-	-	4.46	46	0	1835.19065	3	612.3999634	1.474285275	66.43766667
														AGGPGLER	1	12	2	N-Term(iTRAQ8plex)	-	-	-	-	26	0	1060.59892	2	530.8031006	-6.002974336	11.45919167
														WCNEHLK	1	14	2	N-Term(iTRAQ8plex); C2(Methylthio); K7(iTRAQ8plex)	-	-	-	-	22	0	1583.81247	3	528.6090088	-9.975412545	21.57536167
258614015	BCAT2	branched-chain-amino-acid aminotransferase, mitochondrial isoform b	NP_001158245.1	8.00	2	2	3	300	33.8	7.72	0.796	1.110	1.265																
														NLHIPTMENGPELILR	1	3	1	N-Term(iTRAQ8plex); M7(Oxidation)	0.593	1.733	1.341	3.49	-	0	2167.17734	3	723.0639648	-3.416895613	34.63362833
														AHEWMFPV	2	3	1	N-Term(iTRAQ8plex)	0.809	0.916	1.207	-	43	0	1320.6624	2	660.8348389	-6.708895186	58.618695
258645172	BCKDHA	2-oxoisovalerate dehydrogenase subunit alpha, mitochondrial isoform 2 precursor	NP_001158255.1	3.38	1	1	1	444	50.4	8.27	0.777	0.646	0.644																
														QGQIINPSEDPHLPK	1	2	1	N-Term(iTRAQ8plex); K15(iTRAQ8plex)	0.777	0.646	0.644	3.61	-	0	2281.2786	3	761.0977173	1.094695523	23.35828333
259013553	OGDH	2-oxoglutarate dehydrogenase, mitochondrial isoform 3 precursor	NP_001158508.1	16.39	12	12	63	1019	115.4	7.02	1.214	0.565	0.431																
														FLQMCNDDPDVLPDLK	6	4	1	N-Term(iTRAQ8plex); C5(Methylthio); K16(iTRAQ8plex)	1.235	0.555	0.396	5.64	64	0	2517.27495	2	1259.141113	4.01531128	55.15807167
														VIPEDGPAAQNPENVK	2	4	1	N-Term(iTRAQ8plex); K16(iTRAQ8plex)	0.735	0.647	0.586	5.5	32	0	2286.26072	3	762.7584229	2.477409794	26.21146333
														VVNAPIFHVNSDDPEAVM YVCK	6	4	1	N-Term(iTRAQ8plex); C21(Methylthio); K22(iTRAQ8plex)	1.17	0.716	0.428	5.23	65	0	3101.5792	3	1034.529419	0.571092647	44.76067667
														IEQLSPFPFDLLLK	6	4	1	N-Term(iTRAQ8plex); K14(iTRAQ8plex)	1.31	0.504	0.347	5	46	0	2268.35625	2	1134.681763	4.332419403	64.33803
														TVDWALAEYMAFGSLLK	4	8	1	N-Term(iTRAQ8plex); K17(iTRAQ8plex)	1.211	0.317	0.225	4.97	54	0	2523.38628	2	1262.196777	3.355319625	63.23552333
														DMVGQVAITR	4	4	1	N-Term(iTRAQ8plex)	1.212	0.527	0.385	4.87	54	0	1393.78452	3	465.2663574	5.013483454	26.13596333
														LLDTAFDLDVFK	20	4	1	N-Term(iTRAQ8plex); K12(iTRAQ8plex)	1.492	0.692	0.47	4.83	73	0	2005.14035	2	1003.078552	1.584206897	51.92913167
														HWLDSPWPGFFTLDGQPR	4	4	1	N-Term(iTRAQ8plex)	0.675	0.713	1.327	4.62	41	0	2460.24112	3	820.7518921	0.338064234	54.16816667
														FGLEGCEVLIPALK	5	5	1	N-Term(iTRAQ8plex); C6(Methylthio); K14(iTRAQ8plex)	1.171	0.518	0.458	4.23	56	0	2143.20755	3	715.0740356	-1.884379513	53.16178833
														ILHIKHWLDSPWPGFFTLD GQPR	2	4	1	N-Term(iTRAQ8plex); K5(iTRAQ8plex)	-	-	-	3.86	39	1	3368.8832	4	842.9762573	9.337847296	59.52587333
														LVEDHLAVQSLIR	2	6	1	N-Term(iTRAQ8plex)	0.789	0.707	0.501	3.49	36	0	1797.04795	3	599.6875	-3.163271192	36.92459333
														SSPYPTDVAR	2	8	1	N-Term(iTRAQ8plex)	1.012	0.539	0.434	3.01	30	0	1396.73491	2	698.8710938	-1.790189377	16.56056167
259013556	FXR2	fragile X mental retardation syndrome-related protein 2	NP_004851.2	3.12	1	2	3	673	74.2	6.23	0.743	0.892	1.503																
														EITEGDEVEVYSR	2	1	1	N-Term(iTRAQ8plex)	0.743	0.892	1.503	4.74	38	0	1829.9146	2	915.4609375	4.107885694	27.179705
														LQIDEQLR	1	16	3	N-Term(iTRAQ8plex)	-	-	-	3.01	-	0	1318.7614	2	659.8843384	-1.396129874	28.05334833
259089438	TMEM14C	transmembrane protein 14C	NP_001158730.1	34.82	2	2	2	112	11.6	9.88	0.527	0.568	0.486																
														VGVSMFNRPH	1	1	1	N-Term(iTRAQ8plex); M5(Oxidation)	0.527	0.568	0.486	-	29	0	1463.77127	3	488.5952759	-1.251502413	17.02513167
														AGSVPSLAAGLLFGSLAGL GAYQLSQDPR	1	1	1	N-Term(iTRAQ8plex)	-	-	-	-	28	0	3120.7042	3	1040.90625	1.466159467	70.49859667
259155315	SLC25A11	mitochondrial 2-oxoglutarate/malate carrier protein isoform 2	NP_001158889.1	20.13	5	5	13	303	33.0	9.88	0.865	0.564	0.588																
														LGIYTVLFER	4	3	1	N-Term(iTRAQ8plex)	0.971	0.449	0.508	4.14	37	0	1514.88445	2	757.9458618	-2.636694301	55.415085
														LGPHTVLTFIFLEQMNK	5	3	1	N-Term(iTRAQ8plex); K17(iTRAQ8plex)	1.347	0.943	0.658	3.79	30	0	2596.47671	3	866.1646118	0.420145551	60.6373
														LTGADGTPPGFLLK	2	3	1	N-Term(iTRAQ8plex); K14(iTRAQ8plex)	0.641	0.318	0.424	3.74	37	0	1995.1801	2	998.093689	3.287928585	35.79771833
														GIYTGLSAGLLR	1	2	1	N-Term(iTRAQ8plex)	0.798	0.675	0.685	3.16	-	0	1524.903	2	762.9551392	-1.416221022	40.59773667
														NVFNALIR	1	3	1	N-Term(iTRAQ8plex)	0.824	0.934	0.874	2.77	-	0	1250.74797	2	625.8776245	-3.445055805	47.59272167
259906018	ACIN1	apoptotic chromatin condensation inducer in the nucleus isoform 2	NP_001158286.1	2.79	3	3	11	1328	150.5	6.43	1.049	0.764	0.523																
														AAPCIYWLPLTDSQIVQK	6	7	1	N-Term(iTRAQ8plex); C4(Methylthio); K18(iTRAQ8plex)	1.161	0.81	0.467	5.52	66	0	2700.47641	3	900.8303223	1.863096026	55.16574667
														EAVVDLHADDSR	2	2	1	N-Term(iTRAQ8plex)	0.928	1.073	0.791	3.62	34	0	1630.82755	3	544.2807007	-3.870733741	18.89827167
														LLDDLFR	3	7	1	N-Term(iTRAQ8plex)	1.004	0.669	0.51	2.92	36	0	1195.70659	2	598.3569336	6.47254733	47.55516
259906403	CD320	CD320 antigen isoform 2 precursor	NP_001159367.1	5.42	1	1	2	240	24.4	4.81	2.502	0.821	1.408																
														CTLSDDCIPLTWR	2	2	1	N-Term(iTRAQ8plex); C1(Methylthio); C7(Methylthio)	2.502	0.821	1.408	4.22	44	0	1918.88945	2	959.9483643	2.980158977	53.071775
259906438	TNFRSF10A	tumor necrosis factor receptor superfamily member 10A	NP_003835.3	2.78	1	1	2	468	50.1	7.03	1.452	1.123	1.566																
														NASHTLLDALER	2	1	1	N-Term(iTRAQ8plex)	1.452	1.123	1.566	3.95	33	0	1756.98301	3	586.3325195	-1.671021428	45.93119167
260166619	ELAC2	zinc phosphodiesterase ELAC protein 2 isoform 3	NP_001159434.1	2.80	1	1	2	786	87.5	7.97	-	-	-																
														DATLLIHEATLEDGLEEEA VEK	2	3	1	N-Term(iTRAQ8plex); K22(iTRAQ8plex)	-	-	-	3.75	28	0	3033.59633	4	759.154541	-4.142646587	44.64139333
260436864	AP1B1	AP-1 complex subunit beta-1 isoform c	NP_001159491.1	10.99	2	6	13	919	101.3	5.11	-	2.749	0.880																
														KPTETQELVQQVLSLATQ DSDNPDLR	2	3	1	N-Term(iTRAQ8plex); K1(iTRAQ8plex)	-	-	0.844	4.92	54	0	3533.89377	3	1178.636108	0.83212612	60.68179
														YPNKYESVIATLCENLDSL DEPEAR	2	3	1	N-Term(iTRAQ8plex); K4(iTRAQ8plex); C13(Methylthio)	-	2.749	0.918	4.01	42	1	3523.76328	3	1175.259277	2.467952743	67.150505
														LASQANIAQVLAELK	2	11	2	N-Term(iTRAQ8plex); K15(iTRAQ8plex)	-	-	-	5.11	32	0	2177.31406	3	726.4428711	1.20732755	51.074175
														AAMIWIVGEYAER	4	11	2	N-Term(iTRAQ8plex)	-	-	-	4.38	50	0	1812.96428	2	906.9857788	1.229862723	52.74818333
														MEPLNNLQVAVK	2	7	2	N-Term(iTRAQ8plex); K12(iTRAQ8plex)	-	-	-	3.87	42	0	1964.14249	3	655.3856812	-1.75704817	48.76226667
														DCEDPNPLIR	1	11	2	N-Term(iTRAQ8plex); C2(Methylthio)	-	-	-	-	23	0	1521.73052	2	761.3688965	-2.514210768	28.01158
260593665	PEPD	xaa-Pro dipeptidase isoform 3	NP_001159529.1	3.73	1	1	2	429	47.2	6.07	0.640	1.258	3.318																
														FEVNNTILHPEIVECR	2	2	1	N-Term(iTRAQ8plex); C15(Methylthio)	0.64	1.258	3.318	4.07	41	0	2263.15061	3	755.0550537	-0.492418482	41.74922833
260656005	NDUFV1	NADH dehydrogenase [ubiquinone] flavoprotein 1, mitochondrial isoform 2 precursor	NP_001159574.1	20.88	6	6	15	455	49.8	8.21	1.074	0.803	0.561																
														QIEGHTICALGDGAAWPV QGLIR	6	2	1	N-Term(iTRAQ8plex); C8(Methylthio)	0.882	0.473	0.27	5.89	72	0	2755.44486	3	919.1516113	2.775757627	46.53918167
														GAGAYICGEETALIESIEG K	2	2	1	N-Term(iTRAQ8plex); C7(Methylthio); K20(iTRAQ8plex)	-	1.451	2.141	3.99	27	0	2665.36722	3	889.1272583	-0.064024069	53.81271167
														GPDWILGEIK	2	2	1	N-Term(iTRAQ8plex); K10(iTRAQ8plex)	1.488	0.928	0.515	3.87	39	0	1736.01201	2	868.5096436	-4.787300188	67.06278667
														GDARPAEIDSLWEISK	2	2	1	N-Term(iTRAQ8plex); K16(iTRAQ8plex)	1.344	1.024	0.72	3.81	-	0	2395.30204	3	799.1055298	-2.415225489	36.12892333
														YLVVNADEGEPGTCKDR	1	2	1	N-Term(iTRAQ8plex); C14(Methylthio); K15(iTRAQ8plex)	0.834	0.803	0.561	3.25	-	1	2520.2598	3	840.7581177	-3.385572653	27.35564
														HFRPELEER	2	2	1	N-Term(iTRAQ8plex)	0.826	0.45	0.435	2.78	24	0	1516.81279	2	758.9100342	-3.045570719	13.20884833
260656008	SPINT2	kunitz-type protease inhibitor 2 isoform b precursor	NP_001159575.1	3.59	1	1	1	195	21.8	8.38	4.346	1.488	0.337																
														WYFDVER	1	2	1	N-Term(iTRAQ8plex)	4.346	1.488	0.337	-	30	0	1318.66606	2	659.8366699	-5.526855095	45.23795667
260656039	PNPLA6	neuropathy target esterase isoform c	NP_001159584.1	0.92	1	1	2	1300	143.3	7.74	0.519	0.200	0.463																
														GDLIGVVEALTR	2	4	1	N-Term(iTRAQ8plex)	0.519	0.2	0.463	2.96	52	0	1546.91252	2	773.9598999	1.20361343	60.26877333
260763955	NDUFA13	NADH dehydrogenase [ubiquinone] 1 alpha subcomplex subunit 13	NP_057049.5	18.75	2	2	8	144	16.7	8.43	1.149	0.761	0.452																
														IALLPLLQAETDRR	5	1	1	N-Term(iTRAQ8plex)	1.213	0.761	0.452	3.51	30	1	1913.14762	3	638.3873901	-0.519635722	49.15135167
														WVPPLIGELYGLR	3	1	1	N-Term(iTRAQ8plex)	1.122	0.703	0.473	2.86	33	0	1817.0645	2	909.0358887	0.968278093	65.27521833
260898743	NDUFS2	NADH dehydrogenase [ubiquinone] iron-sulfur protein 2, mitochondrial isoform 2 precursor	NP_001159631.1	13.35	5	5	11	457	51.8	8.15	1.136	0.644	0.502																
														NITLNFGPQHPAAHGVLR	2	2	1	N-Term(iTRAQ8plex)	-	-	1.436	4.25	-	0	2246.23623	4	562.3145142	-4.364874566	27.27744167
														TSMESLIHHFK	3	3	1	N-Term(iTRAQ8plex); K11(iTRAQ8plex)	1.177	0.768	0.55	4.12	38	0	1938.06728	4	485.2722778	-2.822323006	33.104455
														LDELEELLTNNR	3	3	1	N-Term(iTRAQ8plex)	-	-	-	3.42	55	0	1762.96085	3	588.3251343	6.783067267	57.8936
														IIAQCLNK	2	3	1	N-Term(iTRAQ8plex); C5(Methylthio); K8(iTRAQ8plex)	1.136	0.627	0.457	3.23	33	0	1556.91062	3	519.6417236	-0.426904123	67.33921
														APGFAHLAGLDK	1	3	1	N-Term(iTRAQ8plex); K12(iTRAQ8plex)	0.899	0.644	0.454	3.2	-	0	1805.05204	3	602.3555298	-0.54579841	27.71618167
261278315	UTP14A	U3 small nucleolar RNA-associated protein 14 homolog A isoform 2	NP_001159693.1	8.62	4	4	6	719	82.0	7.85	1.751	0.786	0.993																
														VQTLEELEELGKEECFQNK	2	4	1	N-Term(iTRAQ8plex); K12(iTRAQ8plex); C15(Methylthio); K19(iTRAQ8plex)	3.512	-	1.469	7.11	43	1	3224.69912	4	806.9302368	1.381550794	63.06119
														LVLADLLEPVK	2	2	1	N-Term(iTRAQ8plex); K11(iTRAQ8plex)	2.121	1.057	1.118	3.78	33	0	1818.14847	3	606.7210083	-4.190883074	44.18003833
														EEPAPEEEEPLLLQRPER	1	4	1	N-Term(iTRAQ8plex)	1.445	0.703	0.691	3.33	-	0	2465.27304	3	822.4291992	-3.888775635	27.12213333
														ELPRPVLEGQQSER	1	5	1	N-Term(iTRAQ8plex)	1.171	0.786	0.882	2.95	-	0	1942.06034	3	648.0249634	-2.916703036	21.07557167
261337161	TRIP13	pachytene checkpoint protein 2 homolog isoform 2	NP_001159732.1	6.92	1	1	2	289	32.4	5.52	0.702	0.936	1.185																
														HNIVFGDYTWTEFDEPFL TR	2	2	1	N-Term(iTRAQ8plex)	0.702	0.936	1.185	4.81	36	0	2791.37235	3	931.1289673	1.915654668	55.39275333
261399877	CCT7	T-complex protein 1 subunit eta isoform d	NP_001159757.1	40.68	15	15	56	499	54.8	7.68	1.186	2.384	1.600																
														VHTVEDYQAIVDAEWNIL YDKLEK	2	4	1	N-Term(iTRAQ8plex); K21(iTRAQ8plex); K24(iTRAQ8plex)	-	-	-	6.33	31	1	3804.07545	5	761.6209106	3.442469225	63.26619167
														QLCDNAGFDATNILNK	4	4	1	N-Term(iTRAQ8plex); C3(Methylthio); K16(iTRAQ8plex)	0.783	1.482	1.33	6.3	45	0	2391.21695	3	797.7438354	-3.671436978	35.163795
														VQGGALEDSQLVAGVAFK	4	3	1	N-Term(iTRAQ8plex); K18(iTRAQ8plex)	1.325	2.273	1.728	6.07	115	0	2397.37261	2	1199.189941	5.316191762	38.10108667
														CQVFEETQIGGER	4	4	1	N-Term(iTRAQ8plex); C1(Methylthio)	1.119	2.475	1.482	5.2	53	0	1845.88677	2	923.4470215	4.899568552	35.91046333
														INALTAASEAACLIVSVDE TIKNPR	10	4	1	N-Term(iTRAQ8plex); C12(Methylthio); K22(iTRAQ8plex)	-	-	-	4.56	60	1	3253.77236	4	814.1985474	-0.673902975	69.10791333
														LLDVVHPAAK	10	2	1	N-Term(iTRAQ8plex); K10(iTRAQ8plex)	1.098	3.253	2.119	4.38	41	0	1671.02934	2	836.0183105	-7.203240499	28.8897
														IALLNVELELK	2	4	1	N-Term(iTRAQ8plex); K11(iTRAQ8plex)	1.145	2.098	1.811	4.38	31	0	1863.16001	3	621.7248535	-9.416262187	75.38396
														GGAEQFMEETER	2	4	1	N-Term(iTRAQ8plex)	1.338	4.55	2.634	4.14	67	0	1687.78166	2	844.3944702	-4.916210722	19.54256167
														QVKPYVEEGLHPQIIIR	1	3	1	N-Term(iTRAQ8plex); K3(iTRAQ8plex)	1.186	2.384	1.477	4.07	-	0	2627.53397	3	876.5161743	-5.86161814	32.655255
														YNFFTGCPK	5	4	1	N-Term(iTRAQ8plex); C7(Methylthio); K9(iTRAQ8plex)	1.604	3.376	1.818	4.02	45	0	1730.87345	3	577.6293335	-6.935840247	35.84670833
														SLHDAIMIVR	4	4	1	N-Term(iTRAQ8plex)	0.48	1.788	1.323	3.49	45	0	1458.83347	3	486.9493408	-4.79293162	27.11579167
														LPIGDVATQYFADRDMFC AGR	3	4	1	N-Term(iTRAQ8plex); C18(Methylthio)	1.569	5.482	3.291	3.18	22	1	2696.29091	4	674.828186	-1.05257165	55.77282333
														QQLLIGAYAK	2	4	1	N-Term(iTRAQ8plex); K10(iTRAQ8plex)	1.276	1.594	0.368	3.08	34	0	1713.03641	3	571.6836548	-9.060049413	39.64379
														INALTAASEAACLIVSVDE TIK	2	4	1	N-Term(iTRAQ8plex); C12(Methylthio); K22(iTRAQ8plex)	-	-	-	2.75	26	0	2886.59397	3	962.8695068	5.624260979	67.70473167
														ALEIIPR	1	4	1	N-Term(iTRAQ8plex)	0.816	2	1.403	-	18	0	1115.70378	2	558.3555298	-4.695107938	31.499725
261862348	SHMT2	serine hydroxymethyltransferase, mitochondrial isoform 3	NP_001159829.1	47.62	15	16	111	483	53.4	8.15	1.219	1.182	0.832																
														ALEAFDLDPAQWGVNVQ PYSGSPANLAVYTALLQP HDR	4	3	1	N-Term(iTRAQ8plex)	-	-	-	6.54	54	0	4428.26914	3	1476.76123	2.797222993	57.96381833
														VVDFIDEGVNIGLEVK	18	4	1	N-Term(iTRAQ8plex); K16(iTRAQ8plex)	1.274	1.177	0.656	5.93	82	0	2354.35031	2	1177.677246	1.870695822	61.37339667
														AHLLADMAHISGLVAAK	8	4	1	N-Term(iTRAQ8plex); K17(iTRAQ8plex)	0.828	1.429	1.25	5.88	49	0	2326.36611	4	582.3469849	5.80659814	49.49072
														AALEALGSCLNNK	6	3	1	N-Term(iTRAQ8plex); C9(Methylthio); K13(iTRAQ8plex)	1.047	1.235	0.771	5.76	66	0	1958.04973	2	979.5285034	-8.295031435	70.93233333
														VLELVSITANK	14	4	1	N-Term(iTRAQ8plex); K11(iTRAQ8plex)	1.206	1.032	0.736	5.38	71	0	1795.10539	2	898.0563354	-5.318362695	39.68321333
														GLELIASENFCSR	14	3	1	N-Term(iTRAQ8plex); C11(Methylthio)	1.424	1.36	0.877	5.33	64	0	1788.89115	3	596.9736328	7.659924477	45.82393833
														AFPMPGFDEH	4	4	1	N-Term(iTRAQ8plex)	1.173	1.192	0.906	4.72	45	0	1451.69621	2	726.3517456	2.123922971	34.10914333
														YYGGAEVVDEIELLCQR	8	3	1	N-Term(iTRAQ8plex); C15(Methylthio)	1.221	1.265	0.93	4.69	92	0	2307.14141	3	769.7183228	4.378888971	67.7462
														TGLIDYNQLALTAR	8	[illegible]	1	N-Term(iTRAQ8plex)	0.964	1.063	0.794	4.18	57	0	[illegible]	2	[illegible]	[illegible]	[illegible]
														IMGLDLPDGGHLTHGYMSDVK	3	3	1	N-Term(iTRAQ8plex); K21(iTRAQ8plex)	1.346	1.02	1.325	3.88	-	0	[illegible]	4	[illegible]	[illegible]	[illegible]
														SATPGLLR	1	4	1	N-Term(iTRAQ8plex)	1.229	1.132	0.877	3.81	-	0	[illegible]	2	[illegible]	[illegible]	[illegible]
														AMADALLER	2	4	1	N-Term(iTRAQ8plex)	1.218	1.244	0.864	2.83	32	0	[illegible]	2	[illegible]	[illegible]	[illegible]
														[illegible]	1	3	1	N-Term(iTRAQ8plex); K8(iTRAQ8plex)	1.137	1.352	0.8	2.8	-	0	[illegible]	3	[illegible]	[illegible]	[illegible]
														DIPYTEDR	4	4	1	N-Term(iTRAQ8plex)	1.21	1.156	0.796	2.76	41	0	[illegible]	2	[illegible]	[illegible]	[illegible]

														YYGGAEVVDEIELLCQR	2	3	1	N-Term(iTRAQ8plex); Y1(iTRAQ8plex); C15(Methylthio)	1.438	1.455	0.956	-	62	0	2611.33792	3	769.7183228	4.378888971	67.7462
														ALEAFDLDPAQWGVNVQPYSGSPANLAVYTALLQPHDR	1	3	1	N-Term(iTRAQ8plex); Y19(iTRAQ8plex)	-	-	-	-	30	0	4732.46743	3	1476.76123	2.797222993	57.96381833
														LIIAGTSAYAR	1	4	1	N-Term(iTRAQ8plex)	1.273	1.444	0.926	-	25	0	1439.84868	2	720.4279785	-2.577276904	31.93821667
														AMADALLER	1	4	1	N-Term(iTRAQ8plex); M2(Oxidation)	1.104	1.181	0.884	-	25	0	1309.70659	2	647.3588257	-2.702732297	30.89876333
														ISATSIFFESMPYK	14	9	2	N-Term(iTRAQ8plex); K14(iTRAQ8plex)	-	-	-	4.24	40	0	2229.20414	3	743.7380371	-4.052226658	43.11548833
														ISATSIFFESMPYK	3	9	2	N-Term(iTRAQ8plex); M11(Oxidation); K14(iTRAQ8plex)	-	-	-	3.71	31	0	2245.19657	3	743.7380371	-4.052226658	43.11548833
262118227	COX7A2	cytochrome c oxidase subunit 7A2, mitochondrial precursor	NP_001856.2	20.00	2	2	10	115	12.8	9.13	1.249	0.850	0.638																
														LFQEDDEIPLYLK	5	1	1	N-Term(iTRAQ8plex); K13(iTRAQ8plex)	1.721	1.047	0.659	3.89	63	0	2231.23833	2	1116.122803	-1.645925136	39.37473167
														GGVADALLYR	5	1	1	N-Term(iTRAQ8plex)	1.217	0.848	0.603	3.72	41	0	1338.76555	2	669.8864136	-2.077116162	31.82443833
262527293	RAB35	ras-related protein Rab-35 isoform 2	NP_001161078.1	16.45	1	2	32	152	17.0	6.79	1.063	1.107	0.784																
														WLHEINQNCDDVCR	10	2	1	N-Term(iTRAQ8plex); C9(Methylthio); C13(Methylthio)	1.063	1.107	0.784	4.75	43	0	2140.9332	3	714.3153687	-1.083917403	50.68792833
														IQIWDTAGQER	14	3	9	N-Term(iTRAQ8plex)	-	-	-	3.41	41	0	1620.85991	2	810.9328003	-3.971558752	26.96297167
														LLIIGDSGVGK	22	2	5	N-Term(iTRAQ8plex); K11(iTRAQ8plex)	-	-	-	4.71	52	0	1680.04277	3	560.6833496	-9.598441174	57.819735
266453278	ATXN10	ataxin-10 isoform 2	NP_001161093.1	15.57	4	4	9	411	46.3	5.08	1.067	3.695	4.737																
														LASEEPPDDEEALATIR	2	2	1	N-Term(iTRAQ8plex)	1.067	3.695	4.737	4.55	84	0	2160.08306	2	1080.545166	-6.659877598	33.968445
														MKELEENLNIAIDVIDAYQK	2	2	1	N-Term(iTRAQ8plex); K2(iTRAQ8plex); K20(iTRAQ8plex)	-	-	-	4.01	28	1	3261.80752	4	816.2073364	-2.855924769	68.02170833
														VTLLDLMIAK	3	2	1	N-Term(iTRAQ8plex); K10(iTRAQ8plex)	-	-	-	3.31	36	0	1725.06845	3	575.6948242	-6.12258879	47.17599833
														LSGVMVPAPIQDLEALR	2	2	1	N-Term(iTRAQ8plex)	-	-	-	3.07	38	0	2113.19309	3	705.0692139	-2.96373695	58.53365
														VIDILR	1	2	1	N-Term(iTRAQ8plex)	0.937	4.59	2.807	-	29	0	1032.66179	2	516.8345337	-9.790753641	22.377055
266453748	PFKM	ATP-dependent 6-phosphofructokinase, muscle type isoform 2	NP_001160159.1	7.69	2	5	9	780	85.1	7.99	1.304	2.488	2.095																
														IMEIVDAITTTAQSHQR	1	5	1	N-Term(iTRAQ8plex)	1.502	1.708	1.851	7.2	-	0	2218.1843	3	740.0662842	1.766304569	64.22862667
														ALVFQPVAELKDQTDFEHR	1	5	1	N-Term(iTRAQ8plex); K11(iTRAQ8plex)	1.132	3.623	2.372	3.65	-	1	2851.55263	4	713.6436157	-1.296938844	40.98670667
														VTVLGHVQR	2	10	2	N-Term(iTRAQ8plex)	-	-	-	3.78	48	0	1312.79285	3	438.2691345	-5.668057642	17.74227333
														EQWWLK	2	16	2	N-Term(iTRAQ8plex); K6(iTRAQ8plex)	-	-	-	2.74	-	0	1497.85271	2	749.4299927	-9.822440052	32.76727167
														TFVLEVMGR	3	20	3	N-Term(iTRAQ8plex)	-	-	-	3.75	27	0	1355.75701	2	678.3821411	-6.561421701	43.95479333
268832194	ATP5SL	ATP synthase subunit s-like protein isoform 5	NP_001161343.1	5.22	1	1	2	230	26.3	5.86	0.813	0.425	0.796																
														GLACLHHLQNLR	2	5	1	N-Term(iTRAQ8plex); C4(Methylthio)	0.813	0.425	0.796	2.97	32	0	1724.94765	4	431.9923706	7.084413175	34.77925833
269847750	CMSS1	protein CMSS1 isoform 2	NP_001161396.1	4.21	1	1	1	261	29.9	9.54	0.802	0.307	0.418																
														VVHLGVGTPGR	1	2	1	N-Term(iTRAQ8plex)	0.802	0.307	0.418	-	28	0	1395.82743	3	465.9473267	-7.160318919	17.76043167
270265879	ME2	NAD-dependent malic enzyme, mitochondrial isoform 2 precursor	NP_001161807.1	9.60	3	3	10	479	53.6	8.54	0.739	0.414	0.670																
														NTLIQFEDFGNHNAFR	6	2	1	N-Term(iTRAQ8plex)	0.935	0.425	0.532	6.19	35	0	2227.11673	3	743.0437622	-1.386514169	39.67598667
														ILGLGDLGVYGMGIPVGK	2	2	1	N-Term(iTRAQ8plex); K18(iTRAQ8plex)	0.665	0.414	1.199	4.67	67	0	2367.40592	3	789.8068237	5.4222393	54.94950667
														SIVDNWPENHVK	2	2	1	N-Term(iTRAQ8plex); K12(iTRAQ8plex)	0.694	0.387	0.67	2.94	25	0	2046.11368	3	682.7094116	-4.496175751	23.225775
271398379	NDC1	nucleoporin NDC1 isoform 2	NP_001162023.1	1.42	1	1	1	634	71.9	9.06	1.379	1.170	0.723																
														LQQFLEFKE	1	3	1	N-Term(iTRAQ8plex); K8(iTRAQ8plex)	1.379	1.17	0.723	3.71	-	1	1790.02312	2	895.5151978	-4.330900593	70.02907
281427278	CDK1	cyclin-dependent kinase 1 isoform 4	NP_001163877.1	14.68	1	1	1	109	12.5	7.40	1.077	1.962	1.681																
														IRLESEEEGVPSTAIR	1	3	1	N-Term(iTRAQ8plex)	1.077	1.962	1.681	2.81	-	1	2090.13022	3	697.3815918	-4.473003297	25.54063333
281604138	PGAM5	serine/threonine-protein phosphatase PGAM5, mitochondrial isoform 2	NP_001164015.1	11.11	2	2	4	288	31.9	8.68	1.646	0.960	0.517																
														EGAPIEPDPPVSHWKPEAVYYEDGAR	1	1	1	N-Term(iTRAQ8plex); K15(iTRAQ8plex)	-	-	-	2.92	-	0	3517.77602	4	880.1994629	-2.527056142	32.03058
														HIFLIR	3	3	1	N-Term(iTRAQ8plex)	1.646	0.96	0.517	-	25	0	1102.70313	3	368.2392273	-0.664426549	22.06088667
282165706	TJP2	tight junction protein ZO-2 isoform 6	NP_001164101.1	1.11	1	1	2	993	111.6	7.46	1.320	0.997	0.668																
														AFEVMDEFDGR	2	12	1	N-Term(iTRAQ8plex)	1.32	0.997	0.668	4.22	35	0	1619.78081	2	810.394043	8.049248037	42.87204
282165814	TMED5	transmembrane emp24 domain-containing protein 5 isoform 1 precursor	NP_057124.3	5.24	1	1	3	229	26.0	4.84	0.682	0.549	0.449																
														LEDILESINSIK	3	1	1	N-Term(iTRAQ8plex); K12(iTRAQ8plex)	0.682	0.549	0.449	4.63	24	0	1982.16996	3	661.3948364	3.498553352	54.86548
282396088	COA7	cytochrome c oxidase assembly factor 7	NP_075565.2	6.49	1	1	4	231	25.7	6.02	1.084	1.221	1.920																
														ACDLGHIWACANASR	4	1	1	N-Term(iTRAQ8plex); C2(Methylthio); C10(Methylthio)	1.084	1.221	1.92	2.96	26	0	1983.89389	3	661.9694824	-1.249706137	47.07744667
283436222	ATAD3A	ATPase family AAA domain-containing protein 3A isoform 2	NP_001164006.1	21.67	5	10	26	586	66.2	9.25	1.154	0.646	0.534																
														LLSRPQDALEGVVLSPSLEAR	5	4	1	N-Term(iTRAQ8plex)	1.154	0.794	0.543	4.21	41	0	2554.45182	3	852.1554565	0.436523758	45.34449333
														VQDAVQQHQQK	1	3	1	N-Term(iTRAQ8plex); K11(iTRAQ8plex)	1.112	0.728	0.589	3.73	-	0	1917.07413	3	639.696228	-1.106095969	12.98710833
														DALNLAQMQEQTLQLEQQSK	2	4	1	N-Term(iTRAQ8plex); M8(Oxidation); K20(iTRAQ8plex)	0.985	-	-	3.08	27	0	2940.55312	4	735.8937378	-0.889291238	33.32577333
														GLLLFVDEADAFLR	2	5	1	N-Term(iTRAQ8plex)	2.298	0.265	0.435	2.79	36	0	1883.05791	2	942.0325928	-0.086228429	70.82703
														ATLNAFLYR	1	5	1	N-Term(iTRAQ8plex)	1.36	0.481	0.526	-	27	0	1372.78447	2	686.895874	-3.336192403	27.3929
														TAGTLFGEGFR	4	10	2	N-Term(iTRAQ8plex)	-	-	-	3.8	33	0	1459.78166	2	730.3944702	-2.094484667	34.03944667
														QQQLLNEENLR	2	9	2	N-Term(iTRAQ8plex)	-	-	-	3.55	42	0	1688.91728	2	844.9622803	-3.580621255	18.342975
														DKWSNFDPTGLER	1	7	2	N-Term(iTRAQ8plex); K2(iTRAQ8plex)	-	-	-	3.02	-	1	2173.14896	3	725.0545044	-0.405571096	27.33976667
														ITVLEALR	6	11	2	N-Term(iTRAQ8plex)	-	-	-	2.91	37	0	1218.76738	2	609.8873291	-4.077658255	46.50683667
														LFDWANTSR	2	11	2	N-Term(iTRAQ8plex)	-	-	-	2.84	22	0	1413.73747	2	707.3723755	-3.796280098	54.490165
284795266	SRPRB	signal recognition particle receptor subunit beta	NP_067026.3	16.97	4	4	7	271	29.7	9.04	1.348	0.794	0.625																
														GNSLTLIDLPGHESLR	1	1	1	N-Term(iTRAQ8plex)	1.201	1.588	1.311	4.01	-	0	2026.11875	3	676.0444336	-2.351179444	32.43073667
														NTPSFLIACNK	1	1	1	N-Term(iTRAQ8plex); C9(Methylthio); K11(iTRAQ8plex)	1.682	0.789	0.603	3.57	-	0	1862.00608	3	621.34021	-3.429455611	56.70177333
														AIVFVVDSAAFQR	4	1	1	N-Term(iTRAQ8plex)	1.435	0.799	0.648	3.57	59	0	1726.97477	3	576.3297729	-2.690081196	52.49823
														TLLFVR	1	1	1	N-Term(iTRAQ8plex)	1.267	0.514	0.545	-	17	0	1052.67204	2	526.8396606	-4.68961911	32.19412167
284807150	GBA	glucosylceramidase isoform 2	NP_001165282.1	13.14	3	3	7	449	50.3	7.15	0.651	0.831	0.753																
														NDLDAVALMHPDGSAVVVVLNR	2	3	1	N-Term(iTRAQ8plex)	0.635	0.683	0.688	5.29	50	0	2609.40561	3	870.4733887	1.238391459	38.47405167
														ALQLAQRPVSLLASPWTSPTWLK	3	3	1	N-Term(iTRAQ8plex); K23(iTRAQ8plex)	-	-	-	4.58	-	0	3171.84707	4	793.7172241	-1.032425447	57.85434
														SYFSEEGIGYNIIR	2	2	1	N-Term(iTRAQ8plex)	0.668	1.01	0.824	4.47	39	0	1952.00762	2	976.5074463	0.458327169	37.62754167
284925165	SUN1	SUN domain-containing protein 1 isoform c	NP_001165415.1	1.32	1	1	1	682	76.4	6.64	1.158	0.568	0.767																
														GSQGYLVVR	1	3	1	N-Term(iTRAQ8plex)	1.158	0.568	0.767	3.4	-	0	1282.74639	2	641.8768311	3.339856907	24.41363
289547194	VANGL1	vang-like protein 1 isoform 2	NP_001165882.1	2.68	1	1	2	522	59.7	8.81	-	-	-																
														LVVAVEEAFIHIQR	2	2	1	N-Term(iTRAQ8plex)	-	-	-	4.98	73	0	1928.13187	3	643.3821411	2.452524082	65.658305
289577114	EIF4G2	eukaryotic translation initiation factor 4 gamma 2 isoform 2	NP_001036024.3	2.53	2	2	4	869	98.1	6.99	1.164	1.240	1.738																
														DMGEDLECLCQIMR	1	2	1	N-Term(iTRAQ8plex); C8(Methylthio); C10(Methylthio)	1.164	1.049	1.339	2.94	-	0	2051.87773	2	1026.442505	3.523526528	65.46103
														VIILSLDR	3	2	1	N-Term(iTRAQ8plex)	1.03	1.399	1.937	2.7	29	0	1232.78325	2	616.8952637	-3.853537309	39.24072833
291045307	AAAS	aladin isoform 2	NP_001166937.1	6.24	2	2	5	513	55.8	7.33	1.039	0.911	0.738																
														VWDVSTETCVPLPWFR	4	4	1	N-Term(iTRAQ8plex); C9(Methylthio)	1.24	0.957	0.815	4.58	68	0	2285.14922	2	1143.078247	3.988895147	64.12087
														LGGEAHSMVWDPSGER	1	4	1	N-Term(iTRAQ8plex); M8(Oxidation)	0.937	0.512	0.546	3.05	-	0	2047.97586	3	683.3301392	-2.491274248	21.96858833
291084757	PDHA1	pyruvate dehydrogenase E1 component subunit alpha, somatic form, mitochondrial isoform 4	NP_001166927.1	12.26	5	5	9	359	40.2	8.28	0.554	0.365	0.465																
														LPCIFICENNR	2	5	1	N-Term(iTRAQ8plex); C3(Methylthio); C7(Methylthio)	0.453	0.455	0.52	3.12	20	0	1717.83061	2	859.4189453	6.17680516	56.55260833
														VDGMDILCVR	2	5	1	N-Term(iTRAQ8plex); C8(Methylthio)	0.578	0.376	0.495	3.08	38	0	1470.73674	2	735.8720093	-3.631081729	46.536455
														GANQWIK	2	5	1	N-Term(iTRAQ8plex); K7(iTRAQ8plex)	0.575	0.297	0.35	2.88	31	0	1424.84734	2	712.9273071	0.222015232	18.19658
														EILAELTGR	1	5	1	N-Term(iTRAQ8plex)	0.533	0.583	0.816	2.83	-	0	1305.76604	2	653.3866577	-1.502941763	41.97404833
														EIDVEVR	2	5	1	N-Term(iTRAQ8plex)	0.509	0.316	0.405	-	33	0	1163.6648	3	388.5597839	6.374049168	24.43380167
291219876	TFCP2	alpha-globin transcription factor CP2 isoform 3	NP_001166924.1	3.78	1	1	1	450	51.3	5.44	0.634	1.370	1.556																
														IAQLFSISPCQISQIYK	1	3	1	N-Term(iTRAQ8plex); C10(Methylthio); K17(iTRAQ8plex)	0.634	1.37	1.556	4.03	-	0	2593.44632	3	865.1536255	4.654391624	62.9566
291575128	LDHB	L-lactate dehydrogenase B chain isoform LDHB	NP_001167568.1	47.31	13	15	153	334	36.6	6.05	0.993	5.039	4.003																
														LIAPVAEEEATVPNNK	12	3	1	N-Term(iTRAQ8plex); K16(iTRAQ8plex)	1.606	9.639	8.309	6.74	58	0	2303.31059	2	1152.158936	1.668543426	30.94506833
														SLADELALVDVLEDK	21	3	1	N-Term(iTRAQ8plex); K15(iTRAQ8plex)	1.367	7.799	7.48	6.18	95	0	2238.2581	2	1119.63269	-4.855141864	58.42866333
														MVVESAYEVIK	31	3	1	N-Term(iTRAQ8plex); K11(iTRAQ8plex)	0.938	4.569	3.808	5.29	82	0	1876.05522	2	938.53125	-8.42614855	62.36904667
														SLADELALVDVLEDKLK	8	3	1	N-Term(iTRAQ8plex); K15(iTRAQ8plex); K17(iTRAQ8plex)	1.05	11.846	10.766	5.22	34	1	2783.64933	4	696.6643677	-6.369539395	54.98866667
														GMYGIENEVFLSLPCILNAR	3	2	1	N-Term(iTRAQ8plex); M2(Oxidation); C15(Methylthio)	1.262	-	0.986	5.09	43	0	2605.32035	3	869.1116333	2.790195207	66.48008667
														SADTLWDIQK	8	2	1	N-Term(iTRAQ8plex); K10(iTRAQ8plex)	1.305	9.396	7.104	4.34	52	0	1784.9843	2	892.9957886	-8.964096523	41.30456833
														GEMMDLQHGSLFLQTPK	1	3	1	N-Term(iTRAQ8plex); K17(iTRAQ8plex)	0.804	3.176	2.346	4.01	-	0	2540.33567	3	847.4500732	-4.147020245	36.51248167
														IVVVTAGVR	12	3	1	N-Term(iTRAQ8plex)	0.895	5.068	4.255	3.92	60	0	1217.7808	3	406.5984497	-6.196048527	29.062815
														GLTSVINQK	8	2	1	N-Term(iTRAQ8plex); K9(iTRAQ8plex)	0.928	2.942	2.387	3.89	49	0	1567.9478	2	784.4780273	-8.930924719	20.026325
														GMYGIENEVFLSLPCILNAR	3	2	1	N-Term(iTRAQ8plex); C15(Methylthio)	-	-	1	3.79	39	0	2589.31876	3	869.1116333	2.790195207	66.48008667
														IVADKDYSVTANSK	2	3	1	N-Term(iTRAQ8plex); K5(iTRAQ8plex); K14(iTRAQ8plex)	-	0.799	0.821	3.78	-	1	2423.36734	3	808.4606323	-9.721009654	20.35147
														IHPVSTMVK	2	3	1	N-Term(iTRAQ8plex); K9(iTRAQ8plex)	-	11.853	9.304	3.75	43	0	1619.96244	3	540.6589966	-8.577238332	18.05871667
														IHPVSTMVK	2	3	1	N-Term(iTRAQ8plex); M7(Oxidation); K9(iTRAQ8plex)	0.955	3.346	3.912	3.48	-	0	1635.9578	3	540.6589966	-8.577238332	18.05871667
														FIIPQIVK	3	3	1	N-Term(iTRAQ8plex); K8(iTRAQ8plex)	0.516	3.152	2.544	2.95	35	0	1566.01151	3	522.675354	-7.932597054	41.35769
														LKGEMMDLQHGSLFLQTPK	1	3	1	N-Term(iTRAQ8plex); K2(iTRAQ8plex); K19(iTRAQ8plex)	-	-	-	2.86	-	1	3085.73745	4	772.1898193	2.218647525	43.96036833
														GMYGIENEVFLSLPCILNAR	1	2	1	N-Term(iTRAQ8plex); Y3(iTRAQ8plex); C15(Methylthio)	-	-	-	-	34	0	2893.52561	3	869.1116333	2.790195207	66.48008667
														GMYGIENEVFLSLPCILNAR	1	2	1	N-Term(iTRAQ8plex); M2(Oxidation); Y3(iTRAQ8plex); C15(Methylthio)	2.037	6.901	5.873	-	31	0	2909.51474	3	869.1116333	2.790195207	66.48008667
														VIGSGCNLDSAR	32	14	2	N-Term(iTRAQ8plex); C6(Methylthio)	-	-	-	5.17	104	0	1541.77402	3	514.5961914	1.445522314	30.11560667
														LNLVQR	2	8	2	N-Term(iTRAQ8plex)	-	-	-	-	18	0	1046.65569	2	523.8314819	-6.405213189	23.27319
291621647	PGRMC2	membrane-associated progesterone receptor component 2	NP_006311.2	17.00	2	3	13	247	26.2	5.31	0.712	0.791	0.905																
														GLGAGAGAGEESPATSLPR	2	2	1	N-Term(iTRAQ8plex)	0.481	0.44	0.576	5.41	88	0	2002.04082	2	1001.524048	-4.971035945	24.720495
														FYGPAGPYGIFAGR	9	1	1	N-Term(iTRAQ8plex)	0.716	0.794	0.911	4.15	37	0	1776.92949	2	888.9683838	-4.524851389	43.932675
														GLATFCLDK	2	2	2	N-Term(iTRAQ8plex); C6(Methylthio); K9(iTRAQ8plex)	-	-	-	3.75	44	0	1621.87712	2	811.4421997	-8.076756095	66.02182333
294774574	PPP2R1B	serine/threonine-protein phosphatase 2A 65 kDa regulatory subunit A beta isoform isoform d	NP_001171033.1	11.87	1	5	17	556	61.3	4.93	1.278	1.580	2.538																
														ITLNDLIPAFQNLLK	1	9	1	N-Term(iTRAQ8plex); K15(iTRAQ8plex)	1.278	1.58	2.538	3.45	-	0	2321.40451	3	774.4730225	-0.352014129	65.26657167
														QLSQSLLPAIVELAEDAK	2	11	2	N-Term(iTRAQ8plex); K18(iTRAQ8plex)	-	-	-	6.01	47	0	2533.4791	3	845.1645508	3.669970577	62.36273833
														TSACGLFSVCYPR	6	9	2	N-Term(iTRAQ8plex); C4(Methylthio); C10(Methylthio)	-	-	-	4.13	58	0	1799.83	2	900.4186401	2.517100742	53.112205
														KLSTIALALGVER	4	9	2	N-Term(iTRAQ8plex); K1(iTRAQ8plex)	-	-	-	3.48	59	1	1979.23734	3	660.4172974	-5.068851501	44.39236167
														DCEAEVR	4	11	2	N-Term(iTRAQ8plex); C2(Methylthio)	-	-	-	2.92	29	0	1171.5418	2	586.2703857	-4.632761802	21.63871833
294997282	DAG1	dystroglycan preproprotein	NP_004384.4	1.45	1	1	2	895	97.5	8.56	0.843	1.623	1.618																

														ATSITVTGSGSCR	2	1	1	N-Term(iTRAQ8plex); C12(Methylthio)	0.843	1.623	1.618	4.34	39	0	1589.80058	2	795.4039307	4.826019187	24.91103667
295842266	ECI1	enoyl-CoA delta isomerase 1, mitochondrial isoform 2 precursor	NP_001171500.1	13.33	3	3	11	285	30.9	8.90	1.011	0.920	0.829																
														VLVEPDAGAGVAVMK	2	2	1	N-Term(iTRAQ8plex); K15(iTRAQ8plex)	0.986	1.042	0.743	6.73	65	0	2064.1937	3	688.736084	-2.27817616	32.13423667
														VLVEPDAGAGVAVMK	2	2	1	N-Term(iTRAQ8plex); M14(Oxidation); K15(iTRAQ8plex)	1.037	0.738	0.785	4.92	42	0	2080.1854	3	688.736084	-2.27817616	32.13423667
														DADVQNFVSFISK	5	2	1	N-Term(iTRAQ8plex); K13(iTRAQ8plex)	1.358	1.491	0.966	4.67	42	0	2078.12967	3	693.3814087	-3.945122332	70.48359
														DTLENTIGHR	2	2	1	N-Term(iTRAQ8plex)	0.833	0.812	0.571	3.57	49	0	1459.77683	3	487.2637939	-2.639248676	16.466805
296010848	ASNS	asparagine synthetase [glutamine-hydrolyzing] isoform b	NP_001171546.1	16.48	7	7	14	540	62.1	7.06	1.043	3.522	3.549																
														FSSYYLSLPPEMR	1	3	1	N-Term(iTRAQ8plex)	1.223	1.405	1.655	4.17	-	0	1893.98796	2	947.4976196	8.295373636	43.19385833
														AMTEDGFLAVCSEAK	2	3	1	N-Term(iTRAQ8plex); C11(Methylthio); K15(iTRAQ8plex)	1.043	1.439	1.769	3.91	24	0	2226.09098	3	742.7018433	-6.992420798	39.13425667
														TTAAHGLELR	2	3	1	N-Term(iTRAQ8plex)	0.767	8.874	8.505	3.39	51	0	1372.77976	3	458.2647705	-3.848415254	15.58514167
														LAVVDPLFGMQPIR	2	2	1	N-Term(iTRAQ8plex)	1.039	2.755	2.834	3.32	28	0	1860.06755	2	930.5374146	-2.364870261	57.20818167
														ELYLFDVLR	5	3	1	N-Term(iTRAQ8plex)	0.873	4.438	4.444	3.18	39	0	1471.84502	3	491.2836609	-6.666422836	44.60008167
														ILQEYVEHQVDDAMMANAAQK	1	3	1	N-Term(iTRAQ8plex); K21(iTRAQ8plex)	-	-	-	3.12	-	0	3012.53701	4	753.8897095	-0.326399102	51.23551667
														VPFLDHR	1	3	1	N-Term(iTRAQ8plex)	1.685	5.777	6.205	-	34	0	1187.67405	3	396.5628662	-8.264824196	30.35643167
296040443	UGDH	UDP-glucose 6-dehydrogenase isoform 2	NP_001171629.1	17.56	6	6	18	427	47.6	5.92	0.773	3.334	3.532																
														AVQALCAVYEHWVPR	4	4	1	N-Term(iTRAQ8plex); C6(Methylthio)	0.881	2.917	2.9	7.12	64	0	2092.07645	3	698.0303345	-0.464673282	67.05313833
														EQIVVDLSHPGVSEDDQVSR	4	4	1	N-Term(iTRAQ8plex)	2.97	5.541	6.236	4.87	38	0	2513.28238	3	838.432312	1.508579053	29.92747333
														IIDSLFNTVTDKK	2	4	1	N-Term(iTRAQ8plex); K12(iTRAQ8plex); K13(iTRAQ8plex)	-	-	-	4.26	31	1	2406.42055	4	602.3605957	-6.889222867	51.97679167
														IAILGFAFKK	2	4	1	N-Term(iTRAQ8plex); K9(iTRAQ8plex); K10(iTRAQ8plex)	0.773	7.266	5.465	3.88	23	1	2020.2914	4	505.8283081	-8.522908182	46.676855
														VTVVDVNESR	3	3	1	N-Term(iTRAQ8plex)	0.767	2.626	2.983	2.85	60	0	1421.78411	2	711.3956909	-4.280596499	20.93801667
														AVQALCAVYEHWVPR	2	4	1	N-Term(iTRAQ8plex); C6(Methylthio); Y9(iTRAQ8plex)	-	-	-	-	31	0	2396.27138	3	698.0303345	-0.464673282	67.05313833
														EVVESCR	1	3	1	N-Term(iTRAQ8plex); C6(Methylthio)	0.756	2.051	2.129	-	17	0	1171.56877	2	586.2880249	-5.572968025	20.02477833
296179429	HADH	hydroxyacyl-coenzyme A dehydrogenase, mitochondrial isoform 2 precursor	NP_005318.3	18.15	4	4	8	314	34.3	8.85	0.474	0.338	0.342																
														TFESLVDFSK	4	3	1	N-Term(iTRAQ8plex); K10(iTRAQ8plex)	0.517	0.357	0.366	5.01	38	0	1780.99802	3	594.3375244	2.172204654	64.89171833
														LGAGYPMGPFELLDYVGLDTTK	2	3	1	N-Term(iTRAQ8plex); M7(Oxidation); K22(iTRAQ8plex)	0.247	0.27	0.193	3.64	52	0	2981.59696	3	994.5371704	5.987288262	64.17952667
														FAGLHFFNPVPVMK	1	3	1	N-Term(iTRAQ8plex); K14(iTRAQ8plex)	-	-	0.371	2.99	-	0	2212.25429	4	553.8190308	-0.84896801	48.83905
														LLVPYLMEAIR	1	3	1	N-Term(iTRAQ8plex)	-	-	-	-	28	0	1621.97385	3	541.3294678	5.252100897	63.25233
296317239	PEX11B	peroxisomal membrane protein 11B isoform 2	NP_001171724.1	4.49	1	1	6	245	26.7	9.91	0.441	0.472	0.478																
														GHPPLLLDVVR	6	2	1	N-Term(iTRAQ8plex)	0.441	0.472	0.478	3.61	47	0	1519.92432	3	507.3122559	-2.649459188	28.636645
296923771	NCKAP1L	nck-associated protein 1-like isoform 2	NP_001171905.1	1.21	1	1	1	1077	122.4	6.62	4.130	1.139	1.061																
														LCLQGSLYITLIR	1	2	1	N-Term(iTRAQ8plex); C2(Methylthio)	4.13	1.139	1.061	2.73	-	0	1843.04862	3	615.0210571	-0.061942631	42.97011
297206813	CLDN7	claudin-7 isoform 2 precursor	NP_001171952.1	11.03	1	1	2	145	15.1	8.62	1.358	5.314	0.705																
														MYDSVLALSAALQATR	2	2	1	N-Term(iTRAQ8plex)	1.358	5.314	0.705	2.88	33	0	2014.09368	2	1007.550476	-0.419926626	63.46260833
297206823	NDUFS5	NADH dehydrogenase [ubiquinone] iron-sulfur protein 5	NP_001171908.1	34.91	3	3	7	106	12.5	9.14	0.902	0.538	0.428																
														WLTIQSGEQPYK	2	1	1	N-Term(iTRAQ8plex); K12(iTRAQ8plex)	0.947	0.538	0.402	4.84	38	0	2058.13987	3	686.7181396	-3.96260955	48.54733667
														IEYDDFVECLLR	3	1	1	N-Term(iTRAQ8plex); C9(Methylthio)	1.511	0.446	0.526	4.61	56	0	1864.92522	2	932.9662476	6.711366796	60.28509667
														EWIECAHGIGYTR	2	1	1	N-Term(iTRAQ8plex); C5(Methylthio)	0.766	0.729	0.456	4.21	51	0	1884.89902	3	628.9711914	-2.568483084	37.17169
297374782	ARHGDIA	rho GDP-dissociation inhibitor 1 isoform a	NP_001172006.1	25.49	4	4	9	204	23.2	5.11	0.978	2.994	2.615																
														SIQEIQELDKDDESLR	2	5	1	N-Term(iTRAQ8plex); K10(iTRAQ8plex)	-	-	-	4.56	36	1	2526.33828	4	632.3400269	-4.957276797	29.32205167
														AEEYEFLTPVEEAPK	4	3	1	N-Term(iTRAQ8plex); K15(iTRAQ8plex)	-	1.831	1.944	4.02	52	0	2360.2399	3	787.4181519	-3.524634757	36.10885833
														IDKTDYMVGSYGPR	1	3	1	N-Term(iTRAQ8plex); K3(iTRAQ8plex)	0.772	2.414	1.909	3	-	1	2210.15641	3	737.3903198	-7.780027375	24.77887333
														EIVSGMK	2	5	1	N-Term(iTRAQ8plex); K7(iTRAQ8plex)	1.239	8.73	6.936	2.91	33	0	1371.80179	3	457.9387817	-7.894241879	28.18707333
297374791	FMR1	fragile X mental retardation protein 1 isoform ISO9	NP_001172011.1	3.07	1	2	3	586	66.2	8.47	-	-	-																
														AHMLIDMHFR	2	5	1	N-Term(iTRAQ8plex)	-	-	-	3.8	35	0	1574.82619	4	394.4620056	1.537490898	31.14713
														LQIDEQLR	1	16	3	N-Term(iTRAQ8plex)	-	-	-	3.01	-	0	1318.7614	2	659.8843384	-1.396129874	28.05334833
297374821	INS	insulin preproprotein	NP_001172026.1	6.36	1	1	3	110	12.0	5.30	0.109	0.104	0.185																
														GFFYTPK	3	2	1	N-Term(iTRAQ8plex); K7(iTRAQ8plex)	0.109	0.104	0.185	2.74	27	0	1467.84629	3	489.9535522	0.335313273	23.349585
297747286	PFDN6	prefoldin subunit 6	NP_001172110.1	9.30	1	1	2	129	14.6	8.88	0.627	1.340	1.804																
														ETLAQLQQEFQR	2	1	1	N-Term(iTRAQ8plex)	0.627	1.34	1.804	3.93	35	0	1794.96622	3	598.9935913	0.567908058	36.42520333
298358756	ATP5G3	ATP synthase F(0) complex subunit C3, mitochondrial isoform B precursor	NP_001177258.1	6.67	1	1	1	105	10.6	9.99	0.367	0.261	0.308																
														DIDTAAK	1	6	1	N-Term(iTRAQ8plex); K7(iTRAQ8plex)	0.367	0.261	0.308	3.01	-	0	1341.78513	3	447.9332275	1.272981158	11.87845167
300360487	CNPY2	protein canopy homolog 2 isoform 2 precursor	NP_001177920.1	19.05	1	1	10	84	9.1	7.30	1.545	1.880	1.128																
														ALVDELEWEIAQVDPK	10	2	1	N-Term(iTRAQ8plex); K16(iTRAQ8plex)	1.545	1.88	1.128	4.19	61	0	2463.3552	3	821.7900391	-1.472528998	52.84886833
300360532	GSTO1	glutathione S-transferase omega-1 isoform 2	NP_001177931.1	15.38	3	3	6	208	23.7	7.17	0.832	1.963	3.490																
														HEVININLK	3	3	1	N-Term(iTRAQ8plex); K9(iTRAQ8plex)	0.832	1.963	3.248	4.28	31	0	1688.02422	3	563.345459	-5.750871664	27.22948833
														GSAPPGPVPEGSIR	2	2	1	N-Term(iTRAQ8plex)	0.801	2.645	7.037	3.18	44	0	1624.88994	2	812.9486084	-3.779408668	19.057885
														LLPDDPYEK	1	3	1	N-Term(iTRAQ8plex); K9(iTRAQ8plex)	0.916	1.681	2.968	-	29	0	1697.94585	2	849.4765625	-6.586146437	22.77039167
300360554	SRSF10	serine/arginine-rich splicing factor 10 isoform 6	NP_001177938.1	29.70	5	5	20	165	20.1	10.05	1.029	0.724	0.811																
														GFAYVQFEDVRDAEDALHNLDR	3	8	1	N-Term(iTRAQ8plex)	1.669	-	1.156	5.31	23	1	2884.41005	4	721.8579712	-2.34932421	51.08529833
														YGPIVDVYVPLDFYTR	6	8	1	N-Term(iTRAQ8plex)	1.208	0.778	0.7	4.81	50	0	2221.17998	2	1111.093628	-2.121422248	58.209165
														GFAYVQFEDVR	2	10	1	N-Term(iTRAQ8plex)	0.955	0.545	0.529	4.67	46	0	1634.85576	2	817.9315186	4.721633285	35.11976667
														QIEIQFAQGDR	6	10	1	N-Term(iTRAQ8plex)	1.027	0.687	0.811	3.74	63	0	1608.86716	3	536.9605713	1.491308448	29.15068167
														DAEDALHNLDR	2	8	1	N-Term(iTRAQ8plex)	0.813	0.797	0.923	3.42	25	0	1572.78788	3	524.9341431	-2.616596201	20.02251
														YGPIVDVYVPLDFYTR	1	8	1	N-Term(iTRAQ8plex); Y1(iTRAQ8plex)	1.455	0.703	0.853	-	29	0	2525.3851	2	1111.093628	-2.121422248	58.209165
300388183	PSMD1	26S proteasome non-ATPase regulatory subunit 1 isoform 2	NP_001177966.1	8.13	4	4	7	922	102.2	5.27	0.963	1.834	2.221																
														TPEQCPSVVSLLSESYNPHVR	2	2	1	N-Term(iTRAQ8plex); C5(Methylthio)	1.24	1.11	1.082	5.07	27	0	2692.33023	3	898.1149292	-2.766359137	47.57872333
														NSVCHTATVIANSFMHCGTTSDQFLR	1	3	1	N-Term(iTRAQ8plex); C4(Methylthio); C17(Methylthio)	-	-	-	4.12	-	0	3236.45058	4	809.868103	-6.677398612	65.346335
														YQPFKPLSIGGIIILK	2	2	1	N-Term(iTRAQ8plex); K5(iTRAQ8plex); K16(iTRAQ8plex)	0.963	2.631	3.753	4.01	22	0	2699.69594	4	675.6794434	-1.1733701	53.74287667
														AAVESLGFILFR	2	3	1	N-Term(iTRAQ8plex)	0.703	1.834	2.221	3.72	42	0	1626.95647	2	813.9818726	2.66583481	56.01697333
301336155	NSUN2	tRNA (cytosine(34)-C(5))-methyltransferase isoform 2	NP_001180384.1	8.33	3	3	5	732	82.3	7.01	0.926	1.891	1.483																
														LSSPCIMVVNHDASSIPR	2	2	1	N-Term(iTRAQ8plex); C5(Methylthio)	0.926	1.891	1.483	5.99	52	0	2276.14798	3	759.3875122	-1.040762254	41.74066333
														LQQQQRPEDAEDGAEGGGK	2	2	1	N-Term(iTRAQ8plex); K19(iTRAQ8plex)	-	-	-	4.24	35	0	2621.32729	4	656.0872803	-3.944599472	14.10780667
														LFGFKEDPFVFIPEDDPLFPPIEK	1	2	1	N-Term(iTRAQ8plex); K5(iTRAQ8plex); K24(iTRAQ8plex)	-	-	-	3.07	-	1	3749.05898	4	938.0202026	-1.507213718	64.93553667
302191611	CSRP1	cysteine and glycine-rich protein 1 isoform 3	NP_001180499.1	19.79	3	3	7	187	20.0	8.48	0.865	1.035	2.067																
														GLESTTLADKDGEIYCK	3	2	1	N-Term(iTRAQ8plex); K10(iTRAQ8plex); C16(Methylthio); K17(iTRAQ8plex)	-	-	-	4.96	28	1	2801.46206	4	701.1209717	-7.42307439	32.23870333
														SCFLCMVCKK	1	2	1	N-Term(iTRAQ8plex); C2(Methylthio); C5(Methylthio); M6(Oxidation); [illegible]	-	-	-	3.38	-	1	2228.08437	4	553.7775269	-8.931270205	53.04987333
														SCFLCMVCKK	2	2	1	N-Term(iTRAQ8plex); C2(Methylthio); C5(Methylthio); C8(Methylthio); [illegible]	-	-	-	2.92	21	1	2212.08828	4	553.7775269	-8.931270205	53.04987333
														CSQAVYAAEK	1	2	1	N-Term(iTRAQ8plex); C1(Methylthio); K10(iTRAQ8plex)	0.865	1.035	2.067	-	45	0	1723.88445	2	862.4458618	-7.13811602	20.45773167
302370946	PEX19	peroxisomal biogenesis factor 19 isoform c	NP_001180573.1	7.17	1	1	2	279	30.8	4.37	1.716	1.894	2.325																
														FFQELFDSELASQATAEFEK	2	2	1	N-Term(iTRAQ8plex); K20(iTRAQ8plex)	1.716	1.894	2.325	5.27	63	0	2945.53031	3	982.5149536	9.283995406	63.1534
302699239	EIF4G1	eukaryotic translation initiation factor 4 gamma 1 isoform 4	NP_004944.3	6.98	8	8	15	1404	154.8	5.16	1.016	2.894	2.570																
														VEYTLGEESEAPGQR	2	7	1	N-Term(iTRAQ8plex)	0.864	3.069	2.642	5.95	58	0	1968.98528	2	984.9962769	1.841451523	27.46851667
														QVTQLAIDTEER	2	7	1	N-Term(iTRAQ8plex)	0.611	1.093	1.064	4.28	55	0	1706.9251	2	853.9661865	1.420757642	28.3141
														FMLQDVIDLR	2	45	1	N-Term(iTRAQ8plex)	0.983	3.5	2.876	3.91	39	0	1553.86492	2	777.4360962	-0.923849411	51.25824667
														EMDEAATAEER	4	7	1	N-Term(iTRAQ8plex)	0.92	1.921	1.946	3.68	52	0	1555.71855	2	778.362915	-1.708433617	19.61434
														NHDEESLECLCR	2	45	1	N-Term(iTRAQ8plex); C9(Methylthio); C11(Methylthio)	1.289	2.894	2.57	3.58	25	0	1843.76731	3	615.2606201	-4.11993336	31.94640333
														FSALQQAVPTESTDNRR	1	7	1	N-Term(iTRAQ8plex)	1.346	1.305	1.281	3.23	-	1	2224.1572	3	742.057251	-2.347684918	29.35755667
														LKGVIDLIFEK	2	7	1	N-Term(iTRAQ8plex); K2(iTRAQ8plex); K11(iTRAQ8plex)	1.206	3.49	2.902	2.98	26	1	2187.3682	3	729.7942505	-9.053384415	63.71041167
														GGPPGPPISR	1	7	1	N-Term(iTRAQ8plex)	1.108	1.667	1.572	-	24	0	1238.70976	2	619.8585205	-4.962344329	15.78604
303523511	YAP1	transcriptional coactivator YAP1 isoform 2	NP_006097.2	8.00	2	2	6	450	48.2	5.05	0.522	0.948	1.208																
														QASTDAGTAGALTPQHVR	2	12	1	N-Term(iTRAQ8plex)	0.652	0.548	0.646	5.93	58	0	2085.09427	3	695.7029419	-2.311102414	16.23261333
														GDSETDLEALFNAVMNPK	4	12	1	N-Term(iTRAQ8plex); K18(iTRAQ8plex)	0.418	1.64	2.26	5.22	53	0	2559.30796	3	853.7741699	-5.550244291	54.766705
304571975	DCXR	L-xylulose reductase isoform 2	NP_001182147.1	2.89	1	1	1	242	25.7	8.10	0.830	2.049	0.946																
														SFEVNLR	1	2	1	N-Term(iTRAQ8plex)	0.83	2.049	0.946	2.96	-	0	1168.66448	2	584.8358765	1.441825025	27.669845
305410791	GSR	glutathione reductase, mitochondrial isoform 3 precursor	NP_001182032.1	9.81	3	3	5	469	50.5	8.34	1.251	4.004	2.946																
														SFDSMISTNCTEELENAGVEVLK	2	2	1	N-Term(iTRAQ8plex); C10(Methylthio); K23(iTRAQ8plex)	-	-	-	6.67	80	0	3170.5737	3	1057.529419	6.951828911	58.31501
														GIYAVGDVCGK	1	4	1	N-Term(iTRAQ8plex); C9(Methylthio); K11(iTRAQ8plex)	1.138	4.761	2.471	3.52	-	0	1735.91782	3	579.310791	-8.832505174	52.37278833
														ALLTPVAIAAGR	2	4	1	N-Term(iTRAQ8plex)	1.375	3.367	3.511	3.08	31	0	1456.90947	2	728.958374	-4.035853456	37.50775167
306966132	SPTAN1	spectrin alpha chain, non-erythrocytic 1 isoform 3	NP_001182461.1	13.38	23	23	52	2452	282.1	5.34	0.751	0.358	1.064																
														IAALQAFADQLIAAGHYAK	4	16	1	N-Term(iTRAQ8plex); K19(iTRAQ8plex)	-	-	-	5.35	52	0	2580.47866	4	645.8751221	1.076196171	68.00323833
														LIQSHPESAEDLQEK	2	16	1	N-Term(iTRAQ8plex); K15(iTRAQ8plex)	0.703	0.236	1.119	5.23	37	0	2332.2482	3	778.0875854	-5.277398146	21.62659333
														RQDLEDSLQAQQYFADANEAESWMR	2	16	1	N-Term(iTRAQ8plex)	-	-	-	5.02	50	1	3305.56015	3	1102.524902	5.013516766	49.86229833
														HQAFEAELHANADR	2	16	1	N-Term(iTRAQ8plex)	0.544	0.326	0.91	5	55	0	1912.97019	3	638.3282471	7.013523275	17.64194
														LAALADQWQFLVQK	6	16	1	N-Term(iTRAQ8plex); K14(iTRAQ8plex)	0.832	0.275	1.34	4.97	54	0	2239.28501	2	1120.156494	-0.107014754	54.57363
														HALLEADVAAHQDR	2	16	1	N-Term(iTRAQ8plex)	0.672	0.419	0.539	4.93	45	0	1849.97531	3	617.3299561	-3.754853311	15.83890333
														REELITNWEQIR	2	16	1	N-Term(iTRAQ8plex)	0.894	0.619	0.885	4.81	35	1	1891.02719	3	631.013916	-3.578215662	32.16220333
														VLETAEDIQER	2	16	1	N-Term(iTRAQ8plex)	0.896	0.316	1.105	4.57	46	0	1606.86199	2	803.9346313	1.851294846	30.908205
														GVIDMGNSLIER	1	16	1	N-Term(iTRAQ8plex)	0.855	0.521	1.098	4.5	-	0	1607.87676	2	804.4420166	2.397747905	33.31964667
														ADVVESWIGEK	2	16	1	N-Term(iTRAQ8plex); K11(iTRAQ8plex)	0.74	0.4	0.909	4.44	55	0	1841.01018	2	921.008728	-8.882007006	66.60110167
														KSEQLGAMEEALRDNK	[illegible]	[illegible]	[illegible]	[illegible]	[illegible]	[illegible]	[illegible]	[illegible]	[illegible]	[illegible]	[illegible]	[illegible]	[illegible]	[illegible]	[illegible]
														DVEQIKEYHR	[illegible]	[illegible]	[illegible]	[illegible]	[illegible]	[illegible]	[illegible]	[illegible]	[illegible]	[illegible]	[illegible]	[illegible]	[illegible]	[illegible]	[illegible]
														QWAMVEEAIRAAEDVK	[illegible]	[illegible]	[illegible]	[illegible]	[illegible]	[illegible]	[illegible]	[illegible]	[illegible]	[illegible]	[illegible]	[illegible]	[illegible]	[illegible]	[illegible]
														DCEQAENWMAAR	[illegible]	[illegible]	[illegible]	[illegible]	[illegible]	[illegible]	[illegible]	[illegible]	[illegible]	[illegible]	[illegible]	[illegible]	[illegible]	[illegible]	[illegible]
														DVHDLAAWHK	[illegible]	[illegible]	[illegible]	[illegible]	[illegible]	[illegible]	[illegible]	[illegible]	[illegible]	[illegible]	[illegible]	[illegible]	[illegible]	[illegible]	[illegible]
														LSQFVEHWK	[illegible]	[illegible]	[illegible]	[illegible]	[illegible]	[illegible]	[illegible]	[illegible]	[illegible]	[illegible]	[illegible]	[illegible]	[illegible]	[illegible]	[illegible]

														ALINADELASDVAGAEALLDR	2	16	1	N-Term(iTRAQ8plex)	-	-	-	4	67	0	2431.30222	3	811.1055908	1.615558488	65.75062833
														DVDETISWIK	1	16	1	N-Term(iTRAQ8plex); K10(iTRAQ8plex)	0.359	0.121	0.318	3.08	-	0	1814.00994	2	907.508606	-3.134495477	55.67521667
														ELPTAFDYVEFTR	2	16	1	N-Term(iTRAQ8plex)	0.71	0.358	0.909	2.99	35	0	1891.96745	2	946.4873657	-3.655704896	47.99308333
														LIQNNHYAMEDVATR	1	16	1	N-Term(iTRAQ8plex)	0.873	0.815	0.855	2.96	-	0	2079.05326	3	693.68927	-3.013146193	24.31109
														LGESQTLQQFSR	2	16	1	N-Term(iTRAQ8plex)	0.8	0.574	0.948	2.92	26	0	1697.90654	2	849.4569092	-3.468718967	22.188775
														AALLELWELRR	1	16	1	N-Term(iTRAQ8plex)	0.846	0.081	1.144	-	37	1	1673.99759	3	558.6707153	-1.725542449	48.24316667
														YTEHSTVGLAQQWDQLDQLGMR	1	16	1	N-Term(iTRAQ8plex); Y1(iTRAQ8plex)	-	-	-	-	25	0	3184.61513	4	796.9092407	-4.86661299	37.70552333
307775422	LDLR	low-density lipoprotein receptor isoform 6 precursor	NP_001182732.1	12.61	5	5	13	682	75.8	5.21	0.644	0.254	0.430																
														NINSINFDNPVYQK	2	6	1	N-Term(iTRAQ8plex); K14(iTRAQ8plex)	0.652	0.145	0.395	4.98	45	0	2274.22263	3	758.7457275	-4.947595133	47.868575
														AIVVDPVHGFMYWTDWGTPAK	3	6	1	N-Term(iTRAQ8plex); K21(iTRAQ8plex)	0.644	0.173	0.418	4.8	42	0	2998.5864	3	1000.200317	3.924381983	54.25535167
														NVVALDTEVASNR	4	6	1	N-Term(iTRAQ8plex)	0.699	0.276	0.453	4.42	50	0	1691.9157	2	846.4614868	-4.323080163	26.38516333
														AVGSIAYLFFTNR	2	6	1	N-Term(iTRAQ8plex)	0.549	0.254	0.46	3.94	44	0	1762.98528	2	881.9962769	3.338536637	55.65025333
														CEDIDECQDPDTCSQLCVNLEGGYK	2	6	1	N-Term(iTRAQ8plex); C1(Methylthio); C7(Methylthio); C13(Methylthio); [illegible]	-	-	0.429	3.89	56	0	3569.48508	3	1190.499878	5.279539944	59.734015
309747091	ERAP1	endoplasmic reticulum aminopeptidase 1 isoform b precursor	NP_001185470.1	9.88	6	6	10	941	107.2	6.46	0.877	0.756	0.954																
														CVQQTIETIEENIGWMDK	4	4	1	N-Term(iTRAQ8plex); C1(Methylthio); K18(iTRAQ8plex)	1.181	0.784	-	4.27	46	0	2791.40604	3	931.1401978	4.828287797	64.86002833
														ASLINNAFQLVSIGK	1	4	1	N-Term(iTRAQ8plex); K15(iTRAQ8plex)	0.804	1.122	1.276	4.22	-	0	2183.30252	3	728.4390259	0.761729857	46.95045833
														GSDGAPDTGYLWHVPLTFITSK	1	4	1	N-Term(iTRAQ8plex); K22(iTRAQ8plex)	0.484	0.287	-	3.44	-	0	2970.58242	4	743.401062	0.073004833	53.16639167
														MAFPCFDEPAFK	1	3	1	N-Term(iTRAQ8plex); C5(Methylthio); K12(iTRAQ8plex)	0.692	0.709	0.662	3.07	-	0	2057.00772	3	686.3407593	-3.795667792	42.27640833
														TDVLILPEEVEWIK	2	4	1	N-Term(iTRAQ8plex); K14(iTRAQ8plex)	0.956	0.728	0.713	3.04	30	0	2292.33432	3	764.782959	1.37519344	50.76886
														QTWTDEGSVSER	1	4	1	N-Term(iTRAQ8plex)	1.637	1.183	1.544	-	22	0	1698.83061	2	849.9189453	4.080868889	22.34703667
311771540	RBM4	RNA-binding protein 4 isoform 2	NP_001185772.1	16.78	2	2	3	143	16.2	6.02	0.717	1.230	1.029																
														AKFEEYGPVIECDIVK	2	5	1	N-Term(iTRAQ8plex); K2(iTRAQ8plex); C12(Methylthio); K16(iTRAQ8plex)	0.682	1.273	1.108	3.26	32	1	2798.52427	3	933.5129395	0.236842461	44.18602667
														LFIGNLPR	1	5	1	N-Term(iTRAQ8plex)	0.754	1.188	0.955	-	27	0	1233.75505	2	617.3811646	-5.72951529	27.493005
311893345	CPNE1	copine-1 isoform c	NP_001185792.1	3.36	2	2	4	536	58.9	5.83	1.918	1.519	2.232																
														VVTMEVEAR	2	3	1	N-Term(iTRAQ8plex)	2.045	1.665	2.402	3.26	36	0	1337.7426	2	669.374939	1.883777705	26.97797167
														SDPFLEFFR	2	3	1	N-Term(iTRAQ8plex)	1.799	1.386	2.073	3.11	29	0	1461.77641	2	731.3918457	5.755658776	48.49354333
311893396	ATP5J2-PTCD1	ATP5J2-PTCD1 fusion protein	NP_001185808.1	3.87	2	3	26	749	84.1	8.72	0.759	0.405	0.431																
														DFSPSGIFGAFQR	16	5	1	N-Term(iTRAQ8plex)	0.759	0.423	0.438	4.81	53	0	1732.89177	2	866.9495239	-2.481440855	37.75645333
														LGELPSWILMR	8	5	1	N-Term(iTRAQ8plex)	0.736	0.343	0.377	3.85	39	0	1618.92551	3	540.3133545	-2.335545035	59.95512167
														LGELPSWILMR	1	5	1	N-Term(iTRAQ8plex); M10(Oxidation)	-	-	-	-	27	0	1634.91867	3	540.3133545	-2.335545035	59.95512167
														EIIHK	1	2	2	N-Term(iTRAQ8plex); K5(iTRAQ8plex)	-	-	-	-	20	0	1247.79375	2	624.4005127	0.454779186	17.22253333
312222769	NFS1	cysteine desulfurase, mitochondrial isoform b precursor	NP_001185918.1	3.20	1	1	2	406	44.3	7.55	1.020	1.086	0.933																
														FTTEEEVDYTVEK	2	2	1	N-Term(iTRAQ8plex); K13(iTRAQ8plex)	1.02	1.086	0.933	4.42	24	0	2198.14652	3	733.3870239	6.376839408	30.67721833
312596881	PSMC5	26S protease regulatory subunit 8 isoform 2	NP_001186092.1	25.38	6	6	25	398	44.8	8.18	0.569	1.447	1.555																
														EHAPSIIFMDEIDSIGSSR	10	2	1	N-Term(iTRAQ8plex)	0.807	1.163	1.41	5.82	59	0	2408.20383	3	803.4066772	-0.636495404	48.19610667
														EVIELPVKHPELFEALGIAQPK	2	2	1	N-Term(iTRAQ8plex); K8(iTRAQ8plex); K22(iTRAQ8plex)	0.433	1.734	1.555	4.99	24	1	3369.98069	3	1123.998413	-3.173695396	46.77557167
														TMLELLNQLDGFEATK	7	2	1	N-Term(iTRAQ8plex); K16(iTRAQ8plex)	0.645	2.136	2.499	4.97	57	0	2431.3284	3	811.1143188	-3.264884752	53.32791333
														LLREELQLLQEQGSYVGEVVR	4	2	1	N-Term(iTRAQ8plex)	0.569	0.76	1.629	4.77	30	1	2762.53397	3	921.5161743	-0.547179075	62.08932167
														IEELQLIVNDK	1	2	1	N-Term(iTRAQ8plex); K11(iTRAQ8plex)	1.016	1.297	1.554	3.95	-	0	1922.12931	3	641.3812866	-6.547263327	72.71444167
														KIEFPPPNEEAR	1	2	1	N-Term(iTRAQ8plex); K1(iTRAQ8plex)	0.565	0.505	0.909	3.69	-	1	2035.13047	3	679.0483398	-6.306730189	21.92379667
313569778	RPL17	60S ribosomal protein L17 isoform b	NP_001186274.1	45.89	5	5	18	146	17.1	10.10	1.052	1.694	1.780																
														GLDVDSLVIEHIQVNK	6	4	1	N-Term(iTRAQ8plex); K16(iTRAQ8plex)	1.059	1.574	2.064	5.77	41	0	2387.36733	4	597.5971069	-3.72521155	38.66186333
														INPYMSSPCHIEMILTEK	4	4	1	N-Term(iTRAQ8plex); C9(Methylthio); K18(iTRAQ8plex)	0.813	1.089	1.208	5.14	27	0	2760.40097	3	920.8051758	-1.589698848	50.77070667
														EQIVPKPEEEVAQK	2	4	1	N-Term(iTRAQ8plex); K6(iTRAQ8plex); K14(iTRAQ8plex)	3.142	2.001	1.935	4.29	-	0	2536.45839	4	634.8700562	-6.54320633	22.70424667
														SAEFLLHMLK	3	4	1	N-Term(iTRAQ8plex); K10(iTRAQ8plex)	1.161	1.943	1.839	3.55	-	0	1797.04154	3	599.6853638	-7.66996261	46.03874167
														NAESNAELK	2	4	1	N-Term(iTRAQ8plex); K9(iTRAQ8plex)	1.611	2.181	1.78	3.02	30	0	1583.87993	2	792.4436035	-3.157366069	12.31725167
														INPYMSSPCHIEMILTEK	1	4	1	N-Term(iTRAQ8plex); M5(Oxidation); C9(Methylthio); K18(iTRAQ8plex)	0.755	1.235	0.8	2.92	-	0	2776.37636	3	920.8051758	-1.589698848	50.77070667
314122231	BAG6	large proline-rich protein BAG6 isoform c	NP_001186626.1	1.88	1	1	3	903	96.7	6.21	1.233	1.607	2.011																
														LQVVLEHMPVGPDAILR	3	21	1	N-Term(iTRAQ8plex)	1.233	1.607	2.011	3.97	35	0	2191.27201	3	731.09552	6.605984355	58.17639833
315075338	ARL2	ADP-ribosylation factor-like protein 2 isoform 2	NP_001186674.1	7.01	1	1	2	157	18.0	5.94	1.347	0.687	1.202																
														LLMLGLDNAGK	2	2	1	N-Term(iTRAQ8plex); K11(iTRAQ8plex)	1.347	0.687	1.202	3.83	42	0	1753.03508	2	877.0211792	-8.653290353	67.92027833
315139004	PSMB2	proteasome subunit beta type-2 isoform 3	NP_001186709.1	13.10	1	1	2	84	9.6	8.78	0.762	2.146	1.987																
														FILNLPTFSVR	2	3	1	N-Term(iTRAQ8plex)	0.762	2.146	1.987	3.2	26	0	1610.95207	2	805.9796753	-3.182797394	55.53315833
315221152	RPL11	60S ribosomal protein L11 isoform 2	NP_001186731.1	33.33	5	5	25	177	20.1	9.60	1.605	1.875	1.695																
														VLEQLTGQTPVFSK	10	2	1	N-Term(iTRAQ8plex); K14(iTRAQ8plex)	1.636	1.762	1.455	5.04	69	0	2155.25469	2	1078.125244	-7.010642079	35.893535
														LCLNICVGESGDR	10	2	1	N-Term(iTRAQ8plex); C2(Methylthio); C6(Methylthio)	1.523	1.993	1.795	4.64	56	0	1774.82744	2	887.9173584	0.685631092	48.222085
														KLCLNICVGESGDR	2	2	1	K1(iTRAQ8plex); N-Term(iTRAQ8plex); C3(Methylthio); C7(Methylthio)	1.524	2.221	2.109	4.01	23	1	2207.11777	3	736.3774414	-3.979094983	41.45470167
														KNNFSDTGNFGFGIQEHIDLGIK	1	2	1	N-Term(iTRAQ8plex); K1(iTRAQ8plex); K23(iTRAQ8plex)	1.984	1.875	1.695	3.05	-	1	3463.85488	4	866.7191772	-5.454095591	40.09274833
														IAVHCTVR	2	2	1	N-Term(iTRAQ8plex); C5(Methylthio)	1.451	1.947	1.5	-	41	0	1248.67682	3	416.8971252	-7.231508919	17.04089833
315360622	GGCT	gamma-glutamylcyclotransferase isoform 4	NP_001186746.1	11.70	1	1	1	94	10.5	4.84	1.975	1.940	1.618																
														NPSAAFFCVAR	1	4	1	N-Term(iTRAQ8plex); C8(Methylthio)	1.975	1.94	1.618	3.23	-	0	1532.76518	2	766.8862305	-0.259650013	37.19096
315434223	AK3	GTP:AMP phosphotransferase AK3, mitochondrial isoform c	NP_001186782.1	20.38	2	2	6	157	18.2	8.78	1.217	1.897	1.376																
														AYQIDTVINLNVPFEVIK	4	3	1	N-Term(iTRAQ8plex); K18(iTRAQ8plex)	1.444	1.744	1.408	5.87	55	0	2684.56052	3	895.5250244	4.525393299	54.72616
														NLTQYSWLLDGFPR	2	2	1	N-Term(iTRAQ8plex)	1.167	1.897	0.843	5.13	71	0	2014.0772	2	1007.542236	3.572224672	53.09197
315467838	GLRX3	glutaredoxin-3 isoform 1	NP_001186797.1	20.90	4	4	10	335	37.4	5.39	0.925	1.387	1.265																
														HNIQFSSFDIFSDEEVR	6	2	1	N-Term(iTRAQ8plex)	0.877	1.353	1.077	5.73	60	0	2374.15665	3	792.0570679	-2.148525759	43.78619333
														ELKENGELLPILR	1	2	1	N-Term(iTRAQ8plex); K3(iTRAQ8plex)	1.11	3.735	2.031	3.29	-	1	2132.28202	3	711.4321899	-3.730616175	34.11870833
														YEISSVPTFLFFK	1	1	1	N-Term(iTRAQ8plex); K13(iTRAQ8plex)	-	-	-	3.07	-	0	2186.24686	3	729.4204712	5.06759983	59.60378167
														SLLVVHFWAPWAPQCAQMNEVMAELAK	2	1	1	N-Term(iTRAQ8plex); C15(Methylthio); K27(iTRAQ8plex)	-	-	-	2.97	36	0	3723.9247	4	931.7366333	3.050819775	68.97981833
316659409	ACTG1	actin, cytoplasmic 2	NP_001186883.1	68.53	6	21	1263	375	41.8	5.48	0.800	0.774	1.136																
														VAPEEHPVLLTEAPLNPK	105	2	1	N-Term(iTRAQ8plex); K18(iTRAQ8plex)	0.843	0.779	1.183	6.73	59	0	2562.46927	2	1281.737793	-2.697392383	27.48439
														DLYANTVLSGGTTMYPGIADR	39	2	1	N-Term(iTRAQ8plex)	0.743	0.73	1.019	6.68	111	0	2519.27056	2	1260.142944	1.270503334	41.44637333
														TTGIVMDSGDGVTHTVPIYEGYALPHAILR	93	2	1	N-Term(iTRAQ8plex)	0.947	1.111	1.388	6.66	55	0	3487.82041	3	1163.27832	0.173858579	42.94665667
														KDLYANTVLSGGTTMYPGIADR	22	2	1	N-Term(iTRAQ8plex); K1(iTRAQ8plex)	0.959	0.802	1.386	6.08	83	1	2951.56473	3	984.5264282	-3.730184845	34.36076
														TTGIVMDSGDGVTHTVPIYEGYALPHAILR	37	2	1	N-Term(iTRAQ8plex); M6(Oxidation)	1.51	0.913	1.579	5.84	37	0	3503.80507	3	1163.27832	0.173858579	42.94665667
														DLYANTVLSGGTTMYPGIADR	24	2	1	N-Term(iTRAQ8plex); M14(Oxidation)	0.992	0.74	1.268	5.77	92	0	2535.26456	2	1260.142944	1.270503334	41.44637333
														KDLYANTVLSGGTTMYPGIADR	4	2	1	N-Term(iTRAQ8plex); K1(iTRAQ8plex); M15(Oxidation)	0.84	0.799	1.126	5.64	50	1	2967.56841	3	984.5264282	-3.730184845	34.36076
														CPEALFQPSFLGMESCGIHETTFNSIMK	7	2	1	C1(Methylthio); N-Term(iTRAQ8plex); M13(Oxidation); C16(Methylthio); [illegible]	-	-	-	4.96	26	0	3833.80776	4	959.2073975	2.003664302	61.31902167
														CPEALFQPSFLGMESCGIHETTFNSIMK	3	2	1	N-Term(iTRAQ8plex); C1(Methylthio); C16(Methylthio); K28(iTRAQ8plex)	-	-	0.617	4.64	20	0	3817.82924	4	959.2073975	2.003664302	61.31902167
														CPEALFQPSFLGMESCGIHETTFNSIMK	4	2	1	N-Term(iTRAQ8plex); C1(Methylthio); M13(Oxidation); C16(Methylthio); [illegible]	-	-	1.124	4.34	18	0	3849.80044	4	959.2073975	2.003664302	61.31902167
														GYSFTTTAER	38	2	1	N-Term(iTRAQ8plex)	0.73	0.721	1.116	4.03	54	0	1436.72934	3	479.5812988	-2.072809078	26.76051667
														DLYANTVLSGGTTMYPGIADR	2	2	1	N-Term(iTRAQ8plex); Y3(iTRAQ8plex); M14(Oxidation)	0.91	0.724	0.789	-	51	0	2839.46921	2	1260.142944	1.270503334	41.44637333
														DLYANTVLSGGTTMYPGIADR	8	2	1	N-Term(iTRAQ8plex); Y3(iTRAQ8plex)	0.984	0.842	1.42	-	49	0	2823.48447	2	1260.142944	1.270503334	41.44637333
														KDLYANTVLSGGTTMYPGIADR	2	2	1	N-Term(iTRAQ8plex); K1(iTRAQ8plex); Y4(iTRAQ8plex)	0.372	-	0.308	-	24	1	3255.75307	3	984.5264282	-3.730184845	34.36076
														LCYVALDFEQEMATAASSSLEK	185	6	2	N-Term(iTRAQ8plex); C2(Methylthio); K23(iTRAQ8plex)	-	-	-	8.08	99	0	3147.53098	3	1055.183716	-2.921758225	59.33140333
														LCYVALDFEQEMATAASSSLEK	49	6	2	N-Term(iTRAQ8plex); C2(Methylthio); M12(Oxidation); K23(iTRAQ8plex)	-	-	-	7.89	111	0	3163.53916	3	1055.183716	-2.921758225	59.33140333
														QEYDESGPSIVHR	63	21	2	N-Term(iTRAQ8plex)	-	-	-	5.52	85	0	1820.90531	3	607.6399536	-1.517767093	16.41818
														LCYVALDFEQEMATAASSSLEK	13	6	2	N-Term(iTRAQ8plex); C2(Methylthio); Y3(iTRAQ8plex); K23(iTRAQ8plex)	-	-	-	-	47	0	3451.76845	3	1055.183716	-2.921758225	59.33140333
														QEYDESGPSIVHR	1	21	2	N-Term(iTRAQ8plex); Y3(iTRAQ8plex)	-	-	-	-	44	0	2125.10112	3	607.6399536	-1.517767093	16.41818
														IWHHTFYNELR	59	23	3	N-Term(iTRAQ8plex)	-	-	-	5.27	56	0	1819.94927	2	910.4796143	-1.428281869	32.213985
														IWHHTFYNELR	4	23	3	N-Term(iTRAQ8plex); Y7(iTRAQ8plex)	-	-	-	-	50	0	2124.14629	2	910.4796143	-1.428281869	32.213985
														AGFAGDDAPR	34	24	3	N-Term(iTRAQ8plex)	-	-	-	4.35	73	0	1280.65605	3	427.5570984	2.366454583	19.34597667
														AVFPSIVGRPR	138	20	3	N-Term(iTRAQ8plex)	-	-	-	4.24	42	0	1502.91629	3	501.639679	-4.290149398	23.05970333
														GILTLK	1	23	3	N-Term(iTRAQ8plex); K6(iTRAQ8plex)	-	-	-	-	26	0	1252.84558	3	418.2867126	0.553451648	23.60428667
														ILTER	2	27	5	N-Term(iTRAQ8plex)	-	-	-	-	28	0	935.57616	2	468.2917175	-7.036157716	17.75416
														DSYVGDEAQSK	34	6	2	N-Term(iTRAQ8plex); K11(iTRAQ8plex)	-	-	-	4.93	59	0	1806.92607	2	903.9666748	-3.834307739	16.92340333
														EITALAPSTMK	20	7	2	N-Term(iTRAQ8plex); M10(Oxidation); K11(iTRAQ8plex)	-	-	-	4.57	40	0	1786.0087	2	885.5181885	-0.04009362	30.370905
														EITALAPSTMK	32	7	2	N-Term(iTRAQ8plex); K11(iTRAQ8plex)	-	-	-	4.52	50	0	1770.0291	2	885.5181885	-0.04009362	30.370905
														DLTDYLMK	15	8	3	N-Term(iTRAQ8plex); K8(iTRAQ8plex)	-	-	-	3.55	36	0	1606.88591	2	803.9469604	-6.488959006	34.11513833
														IIAPPER	20	8	3	N-Term(iTRAQ8plex)	-	-	-	3.2	50	0	1099.67974	3	367.2290039	-4.785817094	19.22718
														DLTDYLMK	11	8	3	N-Term(iTRAQ8plex); M7(Oxidation); K8(iTRAQ8plex)	-	-	-	2.96	35	0	1622.8792	2	803.9469604	-6.488959006	34.11513833
														SYELPDGQVITIGNER	114	16	4	N-Term(iTRAQ8plex)	-	-	-	5.5	87	0	2095.09258	2	1048.049927	-2.278851268	43.30549667
														SYELPDGQVITIGNER	10	16	4	N-Term(iTRAQ8plex); Y2(iTRAQ8plex)	-	-	-	-	61	0	2399.29204	2	1048.049927	-2.278851268	43.30549667
														HQGVMVGMGQK	10	7	3	N-Term(iTRAQ8plex); K11(iTRAQ8plex)	-	-	-	6.08	64	0	1779.96953	2	890.4884033	-6.922185558	14.76235333
														HQGVMVGMGQK	15	7	3	N-Term(iTRAQ8plex); M5(Oxidation); K11(iTRAQ8plex)	-	-	-	4.94	41	0	1795.96048	2	890.4884033	-6.922185558	14.76235333
														HQGVMVGMGQK	30	7	3	N-Term(iTRAQ8plex); M5(Oxidation); M8(Oxidation); K11(iTRAQ8plex)	-	-	-	4.79	44	0	1811.96592	2	890.4884033	-6.922185558	14.76235333
														CDVDIR	14	3	2	N-Term(iTRAQ8plex); C1(Methylthio)	-	-	-	-	34	0	1070.51923	2	535.7648315	-4.890915061	22.84930333
														CDVDIRK	1	3	2	N-Term(iTRAQ8plex); C1(Methylthio); K7(iTRAQ8plex)	-	-	-	-	29	1	1502.81584	2	751.9115601	-8.057409605	24.068925
316983136	UQCRB	cytochrome b-c1 complex subunit 7 isoform 2	NP_001186904.1	48.10	3	3	13	79	10.0	5.63	1.130	0.460	0.614																
														DDTIYEDEDVKEAIR	4	3	1	N-Term(iTRAQ8plex); K11(iTRAQ8plex)	1.135	0.407	0.422	4.71	76	1	2419.23882	2	1210.123047	-2.527311465	25.76483333
														YEEENFYLEPYLK	4	2	1	N-Term(iTRAQ8plex); K13(iTRAQ8plex)	1.052	0.284	0.506	3.65	50	0	2345.20586	2	1173.106567	-4.389019556	34.69088833
														RLPENLYNDR	5	3	1	N-Term(iTRAQ8plex)	1.328	0.685	0.921	3.4	33	1	1593.8604	3	531.9579468	-3.635338462	16.46768167
316983156	NDUFS1	NADH-ubiquinone oxidoreductase 75 kDa subunit, mitochondrial isoform 4	NP_001186912.1	16.57	9	9	21	670	73.5	5.87	1.091	0.637	0.552																
														MHEDINEEWISDK	2	6	1	N-Term(iTRAQ8plex); K13(iTRAQ8plex)	0.67	0.825	0.583	5.08	25	0	2254.11747	3	752.0440063	-4.256879766	26.41435667
														NDGAAILAAVSSIAQK	2	6	1	N-Term(iTRAQ8plex); K16(iTRAQ8plex)	0.898	0.771	0.616	4.42	30	0	2137.23319	3	713.0825806	-4.942301494	42.26279833

														DFYMTDSISR	2	6	1	N-Term(iTRAQ8plex)	1.125	0.536	0.416	4.33	40	0	1538.75163	2	769.8794556	3.485431107	29.442325
														VLFLLGADGGCITR	4	6	1	N-Term(iTRAQ8plex); C11(Methylthio)	1.116	0.541	0.475	4.3	37	0	1784.98308	2	892.9951782	7.040697167	57.832965
														FASEIAGVDDLGTTGR	2	5	1	N-Term(iTRAQ8plex)	0.907	0.719	0.492	4.17	52	0	1913.00395	2	957.0056152	6.33169039	37.84204
														KPMVVLGSSALQR	2	6	1	N-Term(iTRAQ8plex); K1(iTRAQ8plex)	1.337	0.637	0.562	3.71	24	0	1994.19999	3	665.4048462	-2.079017055	25.76807833
														KPMVVLGSSALQR	1	6	1	N-Term(iTRAQ8plex); K1(iTRAQ8plex); M3(Oxidation)	0.989	0.579	0.618	3.24	-	0	2010.19883	3	665.4048462	-2.079017055	25.76807833
														MCLVEIEK	2	3	1	N-Term(iTRAQ8plex); C2(Methylthio); K8(iTRAQ8plex)	0.916	0.624	0.552	3.19	34	0	1618.88115	2	809.9442139	-0.958183758	59.5124
														FEAPLFNAR	2	6	1	N-Term(iTRAQ8plex)	1.242	0.696	0.531	2.85	26	0	1368.7564	2	684.8818359	-1.005324983	35.96939833
														NRLEEVSPNLVR	2	6	1	N-Term(iTRAQ8plex)	1.091	0.862	0.581	2.8	30	1	1729.98246	3	577.3323364	-2.211162687	17.73294167
316983171	NDUFB6	NADH dehydrogenase [ubiquinone] 1 beta subcomplex subunit 6 isoform 3	NP_001186916.1	25.77	2	2	4	97	11.7	9.48	1.065	0.513	0.567																
														IFPGDTILETGEVIPPMKEFPDQHH	2	2	1	N-Term(iTRAQ8plex); K18(iTRAQ8plex)	-	-	-	5.04	25	1	3455.80654	4	864.7070923	-1.884376136	47.76948667
														IFPGDTILETGEVIPPMK	2	2	1	N-Term(iTRAQ8plex); K18(iTRAQ8plex)	1.065	0.513	0.567	4.72	50	0	2565.44565	3	855.8200684	-0.101141053	47.67894667
319655561	ALDH7A1	alpha-aminoadipic semialdehyde dehydrogenase isoform 2	NP_001188306.1	16.44	7	7	12	511	55.3	6.86	0.336	0.155	0.290																
														QAVSMFLGAVEEAK	2	3	1	N-Term(iTRAQ8plex); K14(iTRAQ8plex)	0.366	0.23	0.281	4.57	29	0	2088.16092	3	696.7251587	-0.516471585	43.57812333
														QVGLMVQER	2	4	1	N-Term(iTRAQ8plex)	0.209	0.049	0.249	3.7	40	0	1363.77056	2	682.388916	2.635422239	25.74558
														FKNEEEVFAWNNEVK	1	4	1	N-Term(iTRAQ8plex); K2(iTRAQ8plex); K15(iTRAQ8plex)	0.336	-	0.223	3.53	-	1	2795.50161	4	699.6308594	-4.136531336	41.41347
														IWADIPAPK	4	3	1	N-Term(iTRAQ8plex); K9(iTRAQ8plex)	0.345	0.225	0.375	3.27	51	0	1618.97417	2	809.9907227	-2.194357124	56.90427833
														MIGGPILPSER	1	4	1	N-Term(iTRAQ8plex)	0.361	0.205	0.28	3.13	-	0	1473.8394	2	737.4233398	-0.500890734	31.390535
														VGNPWDPNVLYGPLHTK	1	3	1	N-Term(iTRAQ8plex); K17(iTRAQ8plex)	-	-	0.303	2.98	-	0	2515.39443	4	629.6040649	1.044999382	36.67216667
														STCTINYSK	1	4	1	N-Term(iTRAQ8plex); C3(Methylthio); K9(iTRAQ8plex)	0.108	0.079	0.476	-	22	0	1670.86905	3	557.6278687	-0.676051164	30.86646
320118865	PLRG1	pleiotropic regulator 1 isoform 2	NP_001188493.1	8.32	2	3	10	505	56.3	9.17	0.703	0.594	1.169																
														VHAAVQPGSLDSESGIFACAFDQSESR	6	2	1	N-Term(iTRAQ8plex); C19(Methylthio)	0.41	0.403	1.328	5.48	56	0	3158.48435	3	1053.499634	0.56540321	46.28278167
														VISGHLGWVR	2	2	1	N-Term(iTRAQ8plex)	1.207	1.079	1.091	3.45	38	0	1427.85193	3	476.6221619	6.605058613	30.459635
														IWDVR	2	4	3	N-Term(iTRAQ8plex)	-	-	-	-	34	0	992.58318	2	496.7910767	-8.265975575	21.62371833
320118883	NUBPL	iron-sulfur protein NUBPL isoform 3	NP_001188503.1	14.71	1	1	2	136	14.7	7.03	0.507	0.445	0.389																
														EASDTGQPIVFSQPESDEAK	2	6	1	N-Term(iTRAQ8plex); K20(iTRAQ8plex)	0.507	0.445	0.389	6.03	40	0	2743.38791	3	915.1341553	-0.148987201	27.5288
320461711	PRDX1	peroxiredoxin-1	NP_001189360.1	57.79	9	11	71	199	22.1	8.13	1.090	4.309	3.190																
														LVQAFQFTDK	15	1	1	N-Term(iTRAQ8plex); K10(iTRAQ8plex)	1.171	6.614	5.433	4.99	57	0	1805.02532	2	903.0162964	-9.116296827	63.16656
														TIAQDYGVLK	7	1	1	N-Term(iTRAQ8plex); K10(iTRAQ8plex)	1.031	2.592	2.034	4.26	42	0	1715.99906	2	858.5043335	-8.054147211	25.527895
														QGGLGPMNIPLVSDPKR	1	1	1	N-Term(iTRAQ8plex); K16(iTRAQ8plex)	1.319	2.345	1.577	4.14	-	1	2387.36057	3	796.458374	-3.527946217	30.68331
														KQGGLGPMNIPLVSDPK	2	1	1	N-Term(iTRAQ8plex); K1(iTRAQ8plex); M8(Oxidation); K17(iTRAQ8plex)	-	3.518	2.525	4.02	25	1	2679.53823	4	670.6400146	-9.28801313	27.86245833
														ADEGISFR	5	1	1	N-Term(iTRAQ8plex)	1.052	5.991	4.731	3.7	60	0	1198.64104	3	400.2158813	-3.245786439	24.42372667
														DISLSDYK	12	1	1	N-Term(iTRAQ8plex); K8(iTRAQ8plex)	1.057	4.696	3.404	3.69	37	0	1548.86003	2	774.9336548	-8.340553318	26.03591833
														IGHPAPNFK	9	1	1	N-Term(iTRAQ8plex); K9(iTRAQ8plex)	1.078	3.425	3.171	3.44	37	0	1588.92917	3	530.3145752	-8.067674938	23.39782
														ATAVMPDGQFK	2	1	1	N-Term(iTRAQ8plex); K11(iTRAQ8plex)	1.138	6.677	4.37	3.14	42	0	1772.96575	2	886.9865112	-9.484367705	20.04070167
														KQGGLGPMNIPLVSDPK	1	1	1	N-Term(iTRAQ8plex); K1(iTRAQ8plex); K17(iTRAQ8plex)	-	-	-	3.08	-	1	2663.54433	4	670.6400146	-9.28801313	27.86245833
														HGEVCPAGWKPGSDTIKPDVQK	2	1	1	N-Term(iTRAQ8plex); C5(Methylthio); K10(iTRAQ8plex); K17(iTRAQ8plex); [illegible]	-	-	-	3	-	0	3611.97228	6	602.8347778	-0.716630032	28.09148667
														QITVNDLPVGR	8	2	2	N-Term(iTRAQ8plex)	-	-	-	3.64	37	0	1515.87602	2	758.4416504	-2.412557948	28.69410333
														GLFIIDDK	7	5	2	N-Term(iTRAQ8plex); K8(iTRAQ8plex)	-	-	-	3.47	44	0	1528.90427	3	510.3062744	-9.981859631	46.92072167
321267477	GBAS	protein NipSnap homolog 2 isoform 2	NP_001189398.1	14.98	2	2	8	247	29.0	10.35	0.782	1.034	0.767																
														KNQLLLEFSFWNEPVPR	2	2	1	N-Term(iTRAQ8plex); K1(iTRAQ8plex)	0.217	0.762	0.475	4.23	41	1	2725.52616	3	909.1802368	-0.915089652	55.14951167
														HGWEELVYYTVPLIQEMESR	6	2	1	N-Term(iTRAQ8plex)	1.535	1.403	1.238	3.91	29	0	2783.41202	3	928.4755249	3.718514166	65.84254333
321267511	NIPSNAP1	protein NipSnap homolog 1 isoform 2	NP_001189431.1	17.05	3	3	5	264	31.4	9.11	0.506	0.393	0.661																
														NQLLLEFSFWNEPQPR	2	2	1	N-Term(iTRAQ8plex)	0.429	0.393	0.662	4.68	75	0	2322.23979	2	1161.623535	9.177515844	54.84716333
														GWDENVYYTVPLVR	2	2	1	N-Term(iTRAQ8plex)	0.506	0.403	0.595	4.42	29	0	2015.07036	2	1008.038818	8.113264466	39.531255
														LKPGTMIEWGNNWAR	1	2	1	N-Term(iTRAQ8plex); K2(iTRAQ8plex)	0.507	0.318	0.661	3.13	-	0	2381.29245	3	794.4356689	-3.54753321	37.08464667
321400140	CD44	CD44 antigen isoform 7 precursor	NP_001189485.1	17.94	7	7	79	340	37.3	5.52	0.997	0.429	1.034																
														YGFIEGHVVIPR	36	28	1	N-Term(iTRAQ8plex)	0.939	0.524	1.058	4.54	67	0	1690.95334	3	564.3226318	-2.909034841	35.77621
														LVINSGNGAVEDR	4	24	1	N-Term(iTRAQ8plex)	1.588	0.173	0.534	4.05	66	0	1647.89507	2	824.4511719	-1.052354628	25.07596333
														KLVINSGNGAVEDR	1	24	1	N-Term(iTRAQ8plex); K1(iTRAQ8plex)	1.394	0.792	1.317	3.74	-	1	2080.1865	3	694.0670166	-5.112399936	24.27826333
														NLQNVDMK	6	24	1	N-Term(iTRAQ8plex); K8(iTRAQ8plex)	1.937	0.346	0.864	3.59	41	0	1569.88382	3	523.9661255	-2.604068591	19.98593833
														ALSIGFETCR	16	28	1	N-Term(iTRAQ8plex); C9(Methylthio)	1.01	0.462	1.08	3.39	42	0	1446.73357	2	723.8723755	-0.84185518	35.32292833
														FAGVFHVEK	9	29	1	N-Term(iTRAQ8plex); K9(iTRAQ8plex)	0.872	0.284	1.047	3.28	44	0	1641.94536	2	821.4746094	-9.360361415	28.96795333
														TEAADLCK	2	29	1	N-Term(iTRAQ8plex); C7(Methylthio); K8(iTRAQ8plex)	1.062	0.603	1.46	3.19	37	0	1504.7824	2	752.8948364	-9.028593418	20.60258
														YGFIEGHVVIPR	4	28	1	N-Term(iTRAQ8plex); Y1(iTRAQ8plex)	0.865	0.447	0.882	-	54	0	1995.15398	3	564.3226318	-2.909034841	35.77621
														NLQNVDMK	1	24	1	N-Term(iTRAQ8plex); M7(Oxidation); K8(iTRAQ8plex)	1.979	0.371	0.954	-	30	0	1585.87236	3	523.9661255	-2.604068591	19.98593833
322302755	SHC1	SHC-transforming protein 1 isoform 5 precursor	NP_001189788.1	2.80	1	1	5	428	46.6	6.54	0.814	0.908	0.883																
														HLLLVDPEGVVR	5	14	1	N-Term(iTRAQ8plex)	0.814	0.908	0.883	4.41	51	0	1650.97885	3	550.9988403	-1.52851694	29.520105
322303127	RPS10	40S ribosomal protein S10	NP_001190174.1	24.24	5	5	45	165	18.9	10.15	1.432	3.344	2.720																
														AEAGAGSATEFQFR	4	1	1	N-Term(iTRAQ8plex)	1.55	3.219	2.548	6.77	78	0	1745.88274	2	873.4450073	3.818543074	28.21456833
														IAIYELLFK	30	2	1	N-Term(iTRAQ8plex); K9(iTRAQ8plex)	1.782	4.121	3.656	4.24	45	0	1718.0573	2	859.5328979	-7.422344128	54.91290333
														KAEAGAGSATEFQFR	7	1	1	N-Term(iTRAQ8plex); K1(iTRAQ8plex)	0.7	1.522	1.26	4.01	54	1	2178.16404	3	726.7261963	-5.677112747	20.95731167
														NVPNLHVMK	3	2	1	N-Term(iTRAQ8plex); M8(Oxidation); K9(iTRAQ8plex)	1.357	2.404	1.902	3.89	29	0	1675.9688	2	838.4880371	-5.130585523	24.486835
														HPELADKNVPNLHVMK	1	2	1	N-Term(iTRAQ8plex); K7(iTRAQ8plex); K16(iTRAQ8plex)	-	-	-	2.73	-	1	2754.58631	5	551.7230835	0.391953763	24.77171
322812163	NDUFC2-KCTD14	NADH dehydrogenase [ubiquinone] 1 subunit C2, isoform 2 isoform 3	NP_001190191.1	8.14	1	1	1	86	9.1	8.35	1.083	0.615	0.524																
														RNPEPLR	1	6	1	N-Term(iTRAQ8plex)	1.083	0.615	0.524	-	33	1	1185.69971	3	395.9047546	-0.734278651	10.3918
323276668	MINOS1	MICOS complex subunit MIC10 isoform b	NP_001191011.1	12.90	1	1	4	62	7.0	7.28	0.989	0.453	0.395																
														CLADAVVK	4	3	1	N-Term(iTRAQ8plex); C1(Methylthio); K8(iTRAQ8plex)	0.989	0.453	0.395	3.36	34	0	1472.8447	3	491.619751	1.46477065	38.06628833
323714253	SCARB2	lysosome membrane protein 2 isoform 2 precursor	NP_001191184.1	10.45	3	3	8	335	37.7	5.14	0.552	0.471	0.641																
														DEVLYVFPSDFCR	4	2	1	N-Term(iTRAQ8plex); C12(Methylthio)	0.466	0.352	0.553	4.44	49	0	1939.91814	2	970.4627075	-2.816552322	58.4502
														KLDDFVETGDIR	2	2	1	N-Term(iTRAQ8plex); K1(iTRAQ8plex)	0.564	0.461	0.605	3.11	30	1	2016.12009	3	672.7115479	-1.061938385	27.52043833
														VEEVGPYTYR	2	2	1	N-Term(iTRAQ8plex)	0.596	0.597	0.715	2.69	24	0	1516.78496	2	758.8961182	-6.567570315	21.881445
														IDIIR	1	0	2	N-Term(iTRAQ8plex)	-	-	-	-	22	0	933.601	2	467.3041382	-2.658044296	28.15645333
324021716	PPP5C	serine/threonine-protein phosphatase 5 isoform 2	NP_001191213.1	4.19	1	1	2	477	54.4	6.27	0.856	1.040	1.573																
														VLIMHGGLFSEDGVTLDDIR	2	3	1	N-Term(iTRAQ8plex)	0.856	1.04	1.573	5.14	62	0	2491.31888	3	831.111145	0.79094997	49.46303667
325651836	SMARCA5	SWI/SNF-related matrix-associated actin-dependent regulator of chromatin subfamily A member 5	NP_003592.3	5.13	5	5	12	1052	121.8	8.09	1.517	1.213	1.098																
														NIPGPHMVLVPK	3	7	1	N-Term(iTRAQ8plex); K12(iTRAQ8plex)	1.347	1.06	0.975	4.12	35	0	1910.14189	2	955.574585	-4.574528288	28.4826
														CNTLITLIER	2	1	1	N-Term(iTRAQ8plex); C1(Methylthio)	1.482	1.301	0.806	3.82	37	0	1525.84673	2	763.427002	5.455978049	54.07691167
														IAFTEWIEPPKR	1	1	1	N-Term(iTRAQ8plex); K11(iTRAQ8plex)	2.042	1.626	1.098	3.58	-	1	2095.2092	3	699.074585	-3.278750008	38.99681167
														YLVIDEAHR	3	7	1	N-Term(iTRAQ8plex)	1.672	1.711	1.22	3.3	50	0	1419.78687	3	473.9338074	-2.051315534	20.71512167
														KANYAVDAYFR	2	7	1	N-Term(iTRAQ8plex); K1(iTRAQ8plex)	1.552	1.018	1.097	2.73	24	1	1926.05875	3	642.6911011	-5.514539649	24.84539167
														YLVIDEAHR	1	7	1	N-Term(iTRAQ8plex); Y1(iTRAQ8plex)	2.177	-	1.51	-	36	0	1724.0021	3	473.9338074	-2.051315534	20.71512167
325651886	NT5E	5'-nucleotidase isoform 2 preproprotein	NP_001191742.1	19.27	6	6	17	524	57.9	6.98	1.019	0.320	0.533																
														YDAMALGNHEFDNGVEGLIEPLLK	4	2	1	N-Term(iTRAQ8plex); K24(iTRAQ8plex)	0.754	0.152	0.431	5.58	43	0	3253.70424	3	1085.236694	-2.121139524	58.05033167
														FRECNMGNLICDAMINNNLR	2	2	1	N-Term(iTRAQ8plex); C4(Methylthio); C11(Methylthio)	0.32	-	0.624	4.99	44	1	2737.25003	3	913.0881958	0.833264158	63.61646333
														IIALGHSGFEMDK	5	2	1	N-Term(iTRAQ8plex); K13(iTRAQ8plex)	1.019	0.326	0.564	4.48	38	0	2026.12241	3	676.0459595	-0.930906825	32.29296833
														VILPNFLANGGDGFQMIKDELLR	3	2	1	N-Term(iTRAQ8plex); K18(iTRAQ8plex)	1.084	-	0.444	3.94	29	1	3168.78286	3	1056.930786	2.440640813	65.38779833
														GAEVAHFMNALR	2	2	1	N-Term(iTRAQ8plex)	1.147	0.658	0.446	3.27	40	0	1619.85532	3	540.623291	-4.742092106	39.30268333
														VIYPAVEGR	1	2	1	N-Term(iTRAQ8plex)	0.978	0.314	0.506	-	26	0	1307.76018	2	654.383728	-1.783098575	23.53026833
325652046	TCEB1	transcription elongation factor B polypeptide 1 isoform b	NP_001191792.1	8.33	1	1	2	96	10.8	5.36	0.906	2.804	3.162																
														VCMYFTYK	2	2	1	N-Term(iTRAQ8plex); C2(Methylthio); K8(iTRAQ8plex)	0.906	2.804	3.162	2.79	26	0	1708.85794	3	570.2908325	-8.285060472	46.80685667
330864681	CAPZB	F-actin-capping protein subunit beta isoform 3	NP_001193470.1	26.92	5	5	28	260	29.2	5.99	0.782	0.929	1.246																
														SGSGTMNLGGSLTR	3	8	1	N-Term(iTRAQ8plex)	0.862	0.905	1.036	4.79	60	0	1641.85991	2	821.4335938	4.07439146	25.63186833
														LTSTVMLWLQTNK	12	8	1	N-Term(iTRAQ8plex); K13(iTRAQ8plex)	0.562	0.891	1.236	4.5	56	0	2143.23539	2	1072.116821	-6.623351644	48.93370667
														KLEVEANNAFDQYR	4	11	1	N-Term(iTRAQ8plex); K1(iTRAQ8plex)	1.029	1.163	1.292	4.13	25	1	2305.23062	3	769.0817261	-3.941613149	31.91863333
														SGSGTMNLGGSLTR	1	8	1	N-Term(iTRAQ8plex); M6(Oxidation)	0.793	0.726	0.825	4.12	-	0	1657.85007	2	821.4335938	4.07439146	25.63186833
														DYLLCDYNR	6	10	1	N-Term(iTRAQ8plex); C5(Methylthio)	0.671	0.89	1.024	3.07	29	0	1524.70488	2	762.8560791	-5.221152612	27.96813167
														SPWSNKYDPPLEDGAMPSAR	1	10	1	N-Term(iTRAQ8plex); K6(iTRAQ8plex); M16(Oxidation)	1.054	1.147	1.388	2.94	-	1	2842.41849	3	948.1443481	-3.727500521	25.81646667
														SPWSNKYDPPLEDGAMPSAR	1	10	1	N-Term(iTRAQ8plex); K6(iTRAQ8plex)	0.851	0.814	1.204	2.88	-	1	2826.42954	3	948.1443481	-3.727500521	25.81646667
332164729	GTF3C3	general transcription factor 3C polypeptide 3 isoform 2	NP_001193703.1	8.72	2	2	4	413	47.2	4.64	0.932	1.254	1.211																
														ALEIITDFSGIVLEK	2	2	1	N-Term(iTRAQ8plex); K15(iTRAQ8plex)	-	-	-	4.51	66	0	2256.34037	3	752.7849731	4.075131453	63.84615
														SLQFELIAAHLNPSDTEEWVR	2	2	1	N-Term(iTRAQ8plex)	0.932	1.254	1.211	4.13	47	0	2759.43894	3	920.4844971	2.992152073	52.13737833
332205961	TGOLN2	trans-Golgi network integral membrane protein 2 isoform 4 precursor	NP_001193773.1	2.37	1	1	2	379	39.5	5.59	2.542	1.776	2.294																
														IIAFVLEGK	2	4	1	N-Term(iTRAQ8plex); K9(iTRAQ8plex)	2.542	1.776	2.294	3.49	51	0	1598.00827	3	533.3409424	-3.440158385	79.24013833
332801090	HNRNPDL	heterogeneous nuclear ribonucleoprotein D-like isoform b	NP_001193929.1	20.94	4	6	46	363	40.0	9.96	1.067	0.415	0.393																
														EYFGAFGEIENIELPMDTK	2	2	1	N-Term(iTRAQ8plex); M16(Oxidation); K19(iTRAQ8plex)	1.103	0.694	0.466	5.76	68	0	2827.4368	2	1406.226929	3.339740452	52.894335
														GFCFITYTDEEPVKK	4	2	1	N-Term(iTRAQ8plex); C3(Methylthio); K14(iTRAQ8plex); K15(iTRAQ8plex)	1.248	0.399	0.559	4.96	28	1	2735.44883	3	912.487793	-2.320147133	38.89846
														EYFGAFGEIENIELPMDTK	2	2	1	N-Term(iTRAQ8plex); K19(iTRAQ8plex)	0.862	0.388	0.315	4.54	77	0	2811.44658	2	1406.226929	3.339740452	52.894335
														VFVGGLSPDTSEEQIKEYFGAFGEIENIELPMDTK	1	2	1	N-Term(iTRAQ8plex); K16(iTRAQ8plex); K35(iTRAQ8plex)	-	-	-	-	29	1	4802.51936	5	961.3096924	6.299631886	64.84996333
														DLTEYLSR	1	2	1	N-Term(iTRAQ8plex)	0.724	0.43	0.331	-	23	0	1300.7022	2	650.8547363	-2.187482852	31.07800667
														GFGFVLFK	10	6	2	N-Term(iTRAQ8plex); K8(iTRAQ8plex)	-	-	-	3.67	39	0	1522.91704	2	761.9621582	-4.715630308	69.43356
														FGEVVDCTIK	26	4	3	N-Term(iTRAQ8plex); C7(Methylthio); K10(iTRAQ8plex)	-	-	-	4.38	53	0	1764.93157	2	882.9761353	-1.970833198	34.73064167
333805628	PPFIA2	liprin-alpha-2 isoform i	NP_001207409.1	2.03	1	1	2	443	50.2	9.44	0.928	3.121	0.689																
														KHELLEEAR	2	50	1	N-Term(iTRAQ8plex); K1(iTRAQ8plex)	0.928	3.121	0.689	2.83	27	1	1733.03354	3	578.3493652	9.763892453	16.84147667
334724463	TWF1	twinfilin-1 isoform 2	NP_002813.3	3.71	1	1	2	350	40.3	6.96	0.614	0.927	0.780																
														HQTLQGVAFPISR	2	2	1	N-Term(iTRAQ8plex)	0.614	0.927	0.78	3.41	31	0	1757.98935	3	586.6679688	-4.032049448	24.96320833

336285443	NCAM1	neural cell adhesion molecule 1 isoform 4 precursor	NP_001229537.1	1.52	1	1	1	726	80.3	4.87	0.467	0.673	0.919																
														FFLCQVAGDAK	1	5	1	N-Term(iTRAQ8plex); C4(Methylthio); K11(iTRAQ8plex)	0.467	0.673	0.919	3.42	-	0	1852.98454	2	926.9959106	-3.490385797	71.29341667
338753391	TRIM26	tripartite motif-containing protein 26	NP_001229712.1	2.04	1	1	1	539	62.1	5.03	0.541	0.820	0.632																
														LALVISELEGK	1	1	1	N-Term(iTRAQ8plex); K11(iTRAQ8plex)	0.541	0.82	0.632	3.98	-	0	1780.08707	3	594.0338745	-9.53942935	82.58554
338827663	ARFGAP2	ADP-ribosylation factor GTPase-activating protein 2 isoform 2	NP_001229761.1	4.67	2	2	4	493	53.4	8.50	0.838	2.204	2.089																
														EVDAEYEAR	2	10	1	N-Term(iTRAQ8plex)	0.749	3.416	3.17	3.32	29	0	1385.6845	2	693.3458862	-0.400855364	19.774525
														STELDSNWNWFQLR	2	8	1	N-Term(iTRAQ8plex)	0.938	1.422	1.377	3.16	39	0	2100.05132	2	1050.529297	2.888419893	47.94089667
338968934	CSDE1	cold shock domain-containing protein E1 isoform 2	NP_001229822.1	5.22	4	4	7	767	85.7	6.20	1.248	2.490	2.296																
														VTLLEGDHVR	2	4	1	N-Term(iTRAQ8plex)	0.998	2.547	2.327	4.5	45	0	1442.81916	3	481.6112366	-5.374778376	25.22513
														LLTSYGFIQCSER	2	4	1	N-Term(iTRAQ8plex); C10(Methylthio)	1.301	2.434	2.264	3.49	45	0	1866.94463	2	933.9759521	2.707834394	45.69414833
														NITLDDASAPR	2	4	1	N-Term(iTRAQ8plex)	1.197	1.376	1.312	2.8	25	0	1476.79167	2	738.8994751	-2.937262422	21.14543333
														LFFHVK	1	4	1	N-Term(iTRAQ8plex); K6(iTRAQ8plex)	1.465	3.233	3.207	2.77	-	0	1398.85837	3	466.9576416	-9.579057884	25.38127
339882741	HLA-C	HLA class I histocompatibility antigen, Cw-1 alpha chain precursor	NP_001229971.1	7.10	1	2	5	366	40.7	5.87	1.332	0.742	0.771																
														APWVEQEGPEYWDR	4	2	1	N-Term(iTRAQ8plex)	1.332	0.742	0.771	3.79	67	0	2066.00444	2	1033.505859	5.944241817	36.10638
														AYLEGTCVEWLR	1	4	2	N-Term(iTRAQ8plex); C7(Methylthio)	-	-	-	3.26	-	0	1789.89861	2	895.4529419	3.745949092	51.517215
341865579	NVL	nuclear valosin-containing protein-like isoform 3	NP_001230075.1	2.70	1	1	1	667	73.2	6.51	1.188	0.989	0.922																
														VLVIGATNRPDSLDPALR	1	15	1	N-Term(iTRAQ8plex)	1.188	0.989	0.922	2.86	-	0	2211.2695	3	737.7613525	-3.09638097	35.78413833
342307096	HIP1	huntingtin-interacting protein 1 isoform 2	NP_001230127.1	1.12	1	1	2	986	110.6	5.27	0.450	0.526	0.693																
														SVSVTAAGQCR	2	5	1	N-Term(iTRAQ8plex); C10(Methylthio)	0.45	0.526	0.693	3.43	30	0	1428.72722	2	714.8672485	2.182467211	23.5331
343168772	ALCAM	CD166 antigen isoform 3 precursor	NP_001230210.1	20.36	6	6	17	555	61.9	6.19	1.079	0.643	0.457																
														EMDPVTQLYTMTSTLEYK	2	3	1	N-Term(iTRAQ8plex); K18(iTRAQ8plex)	1.112	0.419	0.29	5.62	55	0	2758.40934	3	920.1412964	-1.684082846	56.64393167
														TIHSEQAVFDIYYPTEQVTIQVLPPK	4	3	1	N-Term(iTRAQ8plex); K26(iTRAQ8plex)	-	1.038	-	4.52	33	0	3624.98476	4	907.0016479	2.022247639	48.572915
														ALFLETEQLK	6	3	1	N-Term(iTRAQ8plex); K10(iTRAQ8plex)	1.014	0.493	0.463	4.14	44	0	1800.06699	3	600.6938477	-3.195204002	59.80419833
														SMIASTAITVHYLDLSLNPSGEVTR	2	3	1	N-Term(iTRAQ8plex)	-	-	-	3.62	29	0	2979.58829	3	993.8676147	4.003804256	47.54597333
														ESLTLIVEGKPQIK	2	3	1	N-Term(iTRAQ8plex); K10(iTRAQ8plex); K14(iTRAQ8plex)	2.372	0.764	0.451	3.62	28	0	2467.52689	3	823.180481	0.242478044	34.07066667
														ADIQMPFTCSVTYYGPSGQK	1	3	1	N-Term(iTRAQ8plex); C9(Methylthio); K20(iTRAQ8plex)	1.411	0.687	0.802	3.32	-	0	2847.41379	3	949.8094482	5.678548236	44.10651667
344925834	FAM213A	redox-regulatory protein FAM213A isoform 2 precursor	NP_001230710.1	4.13	1	1	2	218	24.4	9.01	1.217	1.996	0.394																
														LGVWYNFFR	2	4	1	N-Term(iTRAQ8plex)	1.217	1.996	0.394	2.96	28	0	1505.81279	2	753.4100342	-5.246044308	80.14857333
345197264	TPD52L2	tumor protein D54 isoform h	NP_001230821.1	8.59	1	1	2	163	17.5	6.60	1.123	2.644	2.489																
														TSAALSTVGSAISR	2	12	1	N-Term(iTRAQ8plex)	1.123	2.644	2.489	3.95	38	0	1624.90996	2	812.9586182	-4.450456982	31.96200833
345498499	LRBA	lipopolysaccharide-responsive and beige-like anchor protein isoform 1	NP_001186211.2	0.42	1	1	2	2851	317.5	5.57	1.543	0.952	1.161																
														ASPNVEAPQPHR	2	5	1	N-Term(iTRAQ8plex)	1.543	0.952	1.161	4.16	23	0	1606.86069	3	536.291748	0.217561253	11.47977
345842445	RELA	transcription factor p65 isoform 4	NP_001230914.1	3.79	1	1	2	448	49.5	7.40	0.719	1.663	0.902																
														NSGSCLGGDEIFLLCDK	2	5	1	N-Term(iTRAQ8plex); C5(Methylthio); C15(Methylthio); K17(iTRAQ8plex)	0.719	1.663	0.902	4.11	43	0	2471.18113	3	824.3985596	-3.572315672	49.40667833
347300410	BID	BH3-interacting domain death agonist isoform 2	NP_001231496.1	12.31	2	2	4	195	22.0	5.44	1.792	4.161	5.332																
														IEADSESQEDIIR	2	3	1	N-Term(iTRAQ8plex)	1.792	4.161	5.332	4.78	49	0	1808.92375	2	904.9655151	3.185514827	27.121165
														TMLVLALLLAK	2	4	1	N-Term(iTRAQ8plex); K11(iTRAQ8plex)	-	-	-	2.92	25	0	1794.18656	3	598.7337036	6.59702292	62.52443333
347446678	LUC7L2	putative RNA-binding protein Luc7-like 2 isoform 3	NP_001231514.1	10.28	3	3	10	389	46.2	10.05	0.601	1.668	1.270																
														LAETQEEISAEVAAK	2	4	1	N-Term(iTRAQ8plex); K15(iTRAQ8plex)	0.72	1.007	0.714	5.18	62	0	2197.22856	2	1099.11792	5.143717836	36.63409167
														LHLGFIEIR	6	4	1	N-Term(iTRAQ8plex)	0.493	1.812	1.68	3.66	58	0	1401.84739	3	467.9539795	-3.292850196	38.95844167
														SHLLNCCPHDVLSGTR	2	4	1	N-Term(iTRAQ8plex); C6(Methylthio); C7(Methylthio)	0.502	1.261	1.27	3.6	22	0	2148.00747	4	537.7573242	-2.321362973	35.24104
347543829	DPYSL2	dihydropyrimidinase-related protein 2 isoform 3	NP_001231533.1	18.47	6	6	22	536	58.1	6.15	0.440	0.556	1.046																
														MDENQFVAVTSTNAAK	2	10	1	N-Term(iTRAQ8plex); K16(iTRAQ8plex)	0.197	0.097	0.609	6.19	41	0	2334.22171	3	778.7454224	-0.136002572	29.11562667
														AALAGGTTMIIDHVVPEPGTSLLAAFDQWR	2	3	1	N-Term(iTRAQ8plex)	0.579	0.556	1.164	5.2	29	0	3441.8342	3	1147.949585	5.764465006	64.10069167
														GLYDGPVCEVSVTPK	2	3	1	N-Term(iTRAQ8plex); C8(Methylthio); K15(iTRAQ8plex)	0.108	0.184	0.969	4.72	77	0	2218.18071	2	1109.593994	4.453812621	35.77753833
														MVIPGGIDVHTR	8	3	1	N-Term(iTRAQ8plex)	0.398	0.518	0.943	3.9	47	0	1598.89311	3	533.6370239	-1.584075409	25.22796167
														SSAEVIAQAR	2	3	1	N-Term(iTRAQ8plex)	0.605	0.758	1.896	3.81	53	0	1335.75647	3	445.9236755	2.292379928	20.46382167
														AITIANQTNCPLYITK	2	3	1	N-Term(iTRAQ8plex); C10(Methylthio); K16(iTRAQ8plex)	1.286	0.947	1.132	3.32	26	0	2418.32499	3	806.7798462	-3.947763778	40.09240833
														MVIPGGIDVHTR	4	3	1	N-Term(iTRAQ8plex); M1(Oxidation)	0.71	0.687	0.741	2.85	34	0	1614.88877	3	533.6370239	-1.584075409	25.22796167
347800665	RAD23B	UV excision repair protein RAD23 homolog B isoform 3	NP_001231653.1	3.86	1	1	2	337	35.0	4.68	0.831	2.153	1.695																
														AVEYLLMGIPGDR	2	3	1	N-Term(iTRAQ8plex)	0.831	2.153	1.695	4.12	46	0	1737.9467	2	869.4769897	-2.559588165	54.89133167
351721324	SET	protein SET isoform 4	NP_001234930.1	13.91	3	3	30	266	31.1	4.23	1.667	8.032	3.084																
														IDFYFDENPYFENK	16	7	1	N-Term(iTRAQ8plex); K14(iTRAQ8plex)	1.669	11.844	4.598	4.73	67	0	2449.20488	2	1225.106079	-5.042337196	72.14753833
														EFHLNESGDPSSK	6	7	1	N-Term(iTRAQ8plex); K13(iTRAQ8plex)	1.519	7.594	3.004	4.69	50	0	2055.04447	2	1028.028809	-4.866417286	16.56227833
														VEVTEFEDIK	8	7	1	N-Term(iTRAQ8plex); K10(iTRAQ8plex)	1.582	5.451	2.265	4.59	67	0	1817.0009	2	909.0040894	-7.919208541	49.29440167
354681991	PAPOLA	poly(A) polymerase alpha isoform 2	NP_001238935.1	5.61	1	1	1	285	32.6	7.72	-	-	-																
														VTDEILHLVPNIDNFR	1	8	1	N-Term(iTRAQ8plex)	-	-	-	2.89	-	0	2199.21988	3	733.744812	5.593567634	58.11044833
354725902	RAB5B	ras-related protein Rab-5B isoform 1	NP_001238965.1	25.58	2	4	19	215	23.7	8.13	0.764	0.956	0.800																
														TAMNVNDLFLAIAK	1	2	1	N-Term(iTRAQ8plex); K14(iTRAQ8plex)	1.072	0.74	0.645	4.98	-	0	2129.21683	3	710.4104614	-3.804343709	43.83082167
														GAQAAIVVYDITNQETFAR	1	1	1	N-Term(iTRAQ8plex)	0.545	1.235	0.993	2.94	-	0	2371.25461	3	791.0897217	-0.581764156	50.36905
														LVLLGESAVGK	8	6	3	N-Term(iTRAQ8plex); K11(iTRAQ8plex)	-	-	-	4.74	53	0	1694.05156	2	847.5296631	-8.985407579	34.19193833
														FEIWDTAGQER	9	6	3	N-Term(iTRAQ8plex)	-	-	-	3.48	38	0	1655.82427	2	828.4157715	-5.353799573	28.77746167
356461016	GEMIN5	gem-associated protein 5 isoform 2	NP_001239085.1	2.59	2	2	3	1507	168.4	6.65	0.707	0.911	0.953																
														IVFNLCPLQTEDDKQLLLSTSMDR	1	2	1	N-Term(iTRAQ8plex); C6(Methylthio); K14(iTRAQ8plex); M22(Oxidation)	-	-	-	3.42	-	1	3449.80849	4	863.2075806	4.210403464	50.28896667
														CSDAVPGGLFGFAAR	2	2	1	N-Term(iTRAQ8plex); C1(Methylthio)	0.707	0.911	0.953	2.95	29	0	1817.90093	2	909.4541016	1.564554459	45.115095
358438328	ISYNA1	inositol-3-phosphate synthase 1 isoform 4	NP_001240318.1	6.98	2	2	7	430	47.1	5.38	-	-	-																
														SVLVDFLIGSGLK	6	4	1	N-Term(iTRAQ8plex); K13(iTRAQ8plex)	-	-	-	4.62	52	0	1956.18674	3	652.7337646	-6.277828406	53.73658333
														FCEVIPGLNDTAENLLR	1	4	1	N-Term(iTRAQ8plex); C2(Methylthio)	-	-	-	-	32	0	2254.15189	3	752.055481	0.216188224	65.40331333
359338964	SRA1	steroid receptor RNA activator 1 isoform 2	NP_001240693.1	5.77	1	1	2	208	23.4	10.24	1.079	1.661	1.463																
														MALLVQELSSHR	2	2	1	N-Term(iTRAQ8plex)	1.079	1.661	1.463	4.24	57	0	1687.94126	3	563.3186035	-3.237081327	51.76145333
359718987	YME1L1	ATP-dependent zinc metalloprotease YME1L1 isoform 4	NP_001240795.1	7.32	3	3	11	683	75.9	8.57	0.895	0.346	0.469																
														ANAPCVIFIDELDSVGGK	7	4	1	N-Term(iTRAQ8plex); C5(Methylthio); K18(iTRAQ8plex)	0.877	0.321	0.453	6.28	57	0	2502.33927	3	834.7846069	7.970642127	64.73044
														NLAEALLTYETLDAK	2	4	1	N-Term(iTRAQ8plex); K15(iTRAQ8plex)	1.71	1.015	1.046	5.28	40	0	2273.27579	3	758.4301147	-4.020766752	42.81861
														GVEEAKQELQEVVEFLK	2	4	1	N-Term(iTRAQ8plex); K6(iTRAQ8plex); K17(iTRAQ8plex)	-	-	-	2.8	24	1	2887.66157	5	578.3381348	2.471209994	64.23593333
371123783	ARL8A	ADP-ribosylation factor-like protein 8A isoform 2	NP_001243058.1	10.88	1	1	2	147	16.9	8.44	0.600	0.922	1.001																
														GVSAIVYMVDAADQEK	2	2	1	N-Term(iTRAQ8plex); K16(iTRAQ8plex)	0.6	0.922	1.001	5.04	35	0	2304.23233	3	768.7489624	-1.860638316	43.14038333
371502112	CSE1L	exportin-2 isoform 2	NP_001243064.1	11.37	8	8	16	915	103.8	6.11	1.198	2.396	3.261																
														EHDPVGQMVNNPK	2	3	1	N-Term(iTRAQ8plex); M8(Oxidation); K13(iTRAQ8plex)	1.188	1.144	1.557	4.96	38	0	2089.0878	3	697.0341187	-3.778487893	14.130725
														LLTECPPMMDTEYTK	2	3	1	N-Term(iTRAQ8plex); C5(Methylthio); K15(iTRAQ8plex)	1.281	3.106	4.068	4.64	63	0	2426.20683	2	1213.607056	5.441583931	45.81377667
														YGALALQEIFDGIQPK	6	3	1	N-Term(iTRAQ8plex); K16(iTRAQ8plex)	0.838	2.579	2.614	4.51	57	0	2371.34433	2	1186.177979	0.197824662	55.87996333
														NLFEDQNTLTSICEK	2	3	1	N-Term(iTRAQ8plex); C13(Methylthio); K15(iTRAQ8plex)	0.688	1.676	2.17	4.24	48	0	2409.20725	3	803.7406006	-7.389896489	38.48394667
														HKDAAIYLVTSLASK	1	3	1	N-Term(iTRAQ8plex); K2(iTRAQ8plex); K15(iTRAQ8plex)	0.833	1.069	1.314	3.16	-	1	2529.49184	4	633.128418	-9.855459735	38.99872667
														KQIFILLFQR	1	3	1	N-Term(iTRAQ8plex); K1(iTRAQ8plex)	1.345	3.045	4.988	-	33	1	1914.2034	3	638.7393188	-6.022352176	52.84641
														YIMIFR	1	3	1	N-Term(iTRAQ8plex)	1.208	2.226	4.273	-	23	0	1146.65593	2	573.831604	-7.648250838	54.49714833
														IVEDEPNKICEADR	1	3	1	N-Term(iTRAQ8plex); K8(iTRAQ8plex); C10(Methylthio)	1.695	5.199	8.115	-	23	1	2285.15897	3	762.3911743	-5.983216469	27.47626667
371872799	TMCO1	transmembrane and coiled-coil domain-containing protein 1 isoform c	NP_001243094.1	13.07	2	2	7	176	19.9	9.82	1.543	0.718	0.883																
														LPFTPLSYIQGLSHR	4	3	1	N-Term(iTRAQ8plex)	1.08	0.762	0.95	4.42	59	0	2033.14634	3	678.3869629	-1.104631814	54.52425
														ILGLAPSR	3	3	1	N-Term(iTRAQ8plex)	1.638	0.718	0.806	-	19	0	1130.71648	2	565.8618774	-3.045004874	22.38234167
371874679	SNX12	sorting nexin-12 isoform 4	NP_001243117.1	15.19	2	2	3	158	18.3	7.30	0.573	1.398	2.135																
														CLHMFLQEEAIDR	2	2	1	N-Term(iTRAQ8plex); C1(Methylthio); M4(Oxidation)	0.554	1.783	1.848	3.43	27	0	1970.93644	3	657.6503296	-3.856938424	39.77349
														IAGHPLAQNER	1	2	1	N-Term(iTRAQ8plex)	0.593	1.096	2.466	2.91	-	0	1509.83713	3	503.9505615	-4.523653277	12.81279667
372266129	FLII	protein flightless-1 homolog isoform 3	NP_001243194.1	4.70	4	4	10	1214	138.4	6.07	0.548	1.119	0.922																
														QLPAMTALQTLHLR	2	6	1	N-Term(iTRAQ8plex)	0.882	1.219	0.878	6.42	70	0	1897.09549	3	633.0366821	-2.133874281	43.48383833
														LAEDILNTMFDTSYSK	6	6	1	N-Term(iTRAQ8plex); K16(iTRAQ8plex)	0.515	1.027	0.968	5.58	69	0	2456.28019	3	819.4304199	-2.949498465	57.62488333
														FKNWDDVLTVDYTR	1	6	1	N-Term(iTRAQ8plex); K2(iTRAQ8plex)	0.853	1.361	1.172	3.09	-	1	2380.26303	3	794.0925293	-5.346744956	40.79114667
														LGQELCGMLHRPR	1	6	1	N-Term(iTRAQ8plex); C6(Methylthio)	-	0.954	0.822	2.95	-	0	1859.9706	4	465.7481079	-0.140077147	42.45867833
372466577	KRT8	keratin, type II cytoskeletal 8 isoform 2	NP_001243222.1	74.95	25	35	386	483	53.7	5.59	1.902	1.827	0.362																
														SNMDNMFESYINNLR	14	2	1	N-Term(iTRAQ8plex)	1.814	1.521	0.366	6.36	74	0	2152.01567	2	1076.511475	2.389061946	48.57234667
														LEAELGNMQGLVEDFK	6	2	1	N-Term(iTRAQ8plex); K16(iTRAQ8plex)	1.571	1.527	-	5.69	67	0	2401.28647	3	801.1003418	-1.219829208	52.94123
														LEGLTDEINFLR	33	2	1	N-Term(iTRAQ8plex)	2.134	2.032	0.208	5.6	83	0	1723.94693	3	575.3204956	-3.664691453	56.85785833
														SLDMDSIIAEVK	14	2	1	N-Term(iTRAQ8plex); K12(iTRAQ8plex)	2.184	2.065	0.258	5.34	78	0	1929.07268	2	965.0449829	0.180344946	39.389525
														LESGMQNMSIHTK	4	2	1	N-Term(iTRAQ8plex); K13(iTRAQ8plex)	1.315	1.44	0.339	5.14	60	0	2084.10845	2	1042.557861	-0.218445326	23.85833667
														LKLEAELGNMQGLVEDFK	11	2	1	N-Term(iTRAQ8plex); K2(iTRAQ8plex); K18(iTRAQ8plex)	2.181	1.833	0.048	5.08	47	1	2946.67095	2	1473.839111	-0.968979503	59.01227833
														TEMENEFVLIKK	2	2	1	N-Term(iTRAQ8plex); K11(iTRAQ8plex); K12(iTRAQ8plex)	2.591	2.653	0.436	5.07	27	1	2393.38271	4	599.1011353	-2.105874815	32.05437167
														ASLEAAIADAEQR	15	2	1	N-Term(iTRAQ8plex)	1.757	1.634	0.567	5.05	71	0	1648.88982	2	824.9485474	5.456281229	34.50498
														DVDEAYMNKVELESR	6	2	1	N-Term(iTRAQ8plex); K9(iTRAQ8plex)	3.007	2.89	0.213	4.82	54	1	2406.23662	2	1203.621948	-2.718533818	29.6179
														LSELEAALQR	24	2	1	N-Term(iTRAQ8plex)	2.033	1.895	0.208	4.81	77	0	1433.82007	3	478.6115417	-4.539834521	38.30793833
														YEELQSLAGK	23	2	1	N-Term(iTRAQ8plex); K10(iTRAQ8plex)	1.862	1.75	0.362	4.78	56	0	1745.98467	2	873.500061	1.978330164	31.99212833
														TEMENEFVLIK	16	2	1	N-Term(iTRAQ8plex); K11(iTRAQ8plex)	1.634	1.487	0.465	4.72	70	0	1961.07781	2	981.0425415	-4.902871348	61.278845
														ASLEAAIADAEQRGELAIK	1	2	1	N-Term(iTRAQ8plex); K19(iTRAQ8plex)	4.789	3.374	1.927	4.59	-	1	2564.45083	4	641.8681641	0.134559024	47.20872333
														AEAESMYQIK	6	2	1	N-Term(iTRAQ8plex); K10(iTRAQ8plex)	1.354	1.395	0.545	4.55	48	0	1777.94975	2	889.4785156	-6.589989379	32.065905
														SLDMDSIIAEVK	5	2	1	N-Term(iTRAQ8plex); M4(Oxidation); K12(iTRAQ8plex)	2.004	1.821	0.328	4.38	43	0	1945.0656	2	965.0449829	0.180344946	39.389525
														ELQSQISDTSVVLSMDNSR	2	2	1	N-Term(iTRAQ8plex)	0.495	0.416	0.524	4.36	45	0	2413.21616	3	805.0769043	-0.883327159	40.33585167
														LQAEIEGLK	15	2	1	N-Term(iTRAQ8plex); K9(iTRAQ8plex)	2.382	2.142	0.258	4.29	52	0	1608.97637	2	804.9918213	-1.08475296	31.75447
														LVSESSDVLPK	4	2	1	N-Term(iTRAQ8plex); K11(iTRAQ8plex)	1.661	1.801	0.369	4.2	36	0	1782.03435	2	891.520813	-7.05482666	37.24643

														SNMDNMFESYINNLR	6	2	1	N-Term(iTRAQ8plex); M3(Oxidation)	1.932	1.541	0.212	4.11	60	0	2168.02129	2	1076.511475	2.389061946	48.57234667
														QLETLGQEK	2	2	1	N-Term(iTRAQ8plex); K9(iTRAQ8plex)	1.992	2.261	0.533	3.96	48	0	1653.955	2	827.4811401	-4.944298088	17.36245
														WSLLQQQK	18	2	1	N-Term(iTRAQ8plex); K8(iTRAQ8plex)	1.867	1.711	0.391	3.92	33	0	1638.97746	2	819.9923706	-0.79116955	33.62508167
														QLYEEEIRELQSQISDTSVVLSMDNSR	3	2	1	N-Term(iTRAQ8plex)	2.108	2.174	-	3.86	-	1	3473.72788	3	1158.580811	-2.69822787	78.36961
														SNMDNMFESYINNLR	4	2	1	N-Term(iTRAQ8plex); M3(Oxidation); M6(Oxidation)	1.574	1.412	0.449	3.59	29	0	2183.99687	2	1076.511475	2.389061946	48.57234667
														DVDEAYMNK	2	2	1	N-Term(iTRAQ8plex); K9(iTRAQ8plex)	1.427	1.45	0.34	3.56	37	0	1692.87322	2	854.9372559	-0.000702951	13.56633333
														LESGMQNMSIHTK	1	2	1	N-Term(iTRAQ8plex); M5(Oxidation); K13(iTRAQ8plex)	2.252	1.923	0.204	3.48	-	0	2100.0972	2	1042.557861	-0.218445326	23.85833667
														TEMENEFVLIKK	2	2	1	N-Term(iTRAQ8plex); M3(Oxidation); K11(iTRAQ8plex); K12(iTRAQ8plex)	4.587	6.096	-	3.41	25	1	2409.38833	4	599.1011353	-2.105874815	32.05437167
														QLYEEEIR	8	2	1	N-Term(iTRAQ8plex)	1.561	1.312	0.562	3.01	29	0	1383.74138	2	692.3737793	-1.358149556	24.10787167
														GGLGGGYGGASGMGGITAVTVNQSLLSPLVLEVDPN[illegible]	1	2	1	N-Term(iTRAQ8plex)	-	-	-	-	59	0	4229.24082	3	1410.418457	-3.199972958	82.07851667
														DVDEAYMNK	1	2	1	N-Term(iTRAQ8plex); M7(Oxidation); K9(iTRAQ8plex)	1.886	1.575	0.204	-	42	0	1708.86724	2	854.9372559	-0.000702951	13.56633333
														ISSSSFSR	2	2	1	N-Term(iTRAQ8plex)	1.643	1.884	0.143	-	24	0	1174.63457	2	587.8209229	-2.036562637	17.62577333
														TTSGYAGGLSSAYGGLTSPGLSYSLGSSFGSGAGSSSFS[illegible]	1	2	1	N-Term(iTRAQ8plex)	-	-	-	-	21	0	4029.92099	3	1343.978516	-1.158254717	44.136775
														FLEQQNK	13	31	8	N-Term(iTRAQ8plex); K7(iTRAQ8plex)	-	-	-	-	33	0	1514.87885	3	505.631134	0.090799082	15.97476833
														VEIEAK	5	1	3	N-Term(iTRAQ8plex); K6(iTRAQ8plex)	-	-	-	2.98	27	0	1296.79612	3	432.9381104	1.107730516	13.75300333
														GELAIK	4	2	6	N-Term(iTRAQ8plex); K6(iTRAQ8plex)	-	-	-	-	28	0	1238.78995	3	413.6025085	0.089671776	17.83946167
														AQYEDIANR	8	3	2	N-Term(iTRAQ8plex)	-	-	-	2.94	31	0	1383.72576	2	692.3607788	-1.973169497	18.09094667
														EYQELMNVK	14	5	3	N-Term(iTRAQ8plex); K9(iTRAQ8plex)	-	-	-	4.08	56	0	1761.96379	2	881.4855347	-1.565630391	28.72846833
														FASFIDKVR	16	27	8	N-Term(iTRAQ8plex); K7(iTRAQ8plex)	-	-	-	3.33	31	1	1691.01076	4	423.5080261	0.114317571	39.05999667
														FASFIDK	57	28	8	N-Term(iTRAQ8plex); K7(iTRAQ8plex)	-	-	-	3.23	35	0	1435.83818	2	718.4240723	0.227470368	33.40336167
														KLLEGEESR	3	9	3	N-Term(iTRAQ8plex); K1(iTRAQ8plex)	-	-	-	3.13	26	1	1668.97317	4	417.9987488	-0.554869804	19.635685
														LLEGEESR	8	11	3	N-Term(iTRAQ8plex)	-	-	-	2.71	46	0	1236.67142	3	412.8953247	-1.892522837	20.19271
														LALDIEIATYR	21	15	2	N-Term(iTRAQ8plex)	-	-	-	4.5	64	0	1581.91385	3	527.9749146	-3.28773257	44.71627667
														NKYEDEINK	1	9	5	N-Term(iTRAQ8plex); K2(iTRAQ8plex); K9(iTRAQ8plex)	-	-	-	-	27	1	2065.16616	4	517.0469971	-1.50249351	14.99920833
373251164	GLS	glutaminase kidney isoform, mitochondrial isoform 2	NP_001243239.1	22.58	10	10	20	598	65.4	7.88	1.160	0.807	1.435																
														HSFGPLDYESLQQELALK	1	1	1	N-Term(iTRAQ8plex); K18(iTRAQ8plex)	-	-	-	4.99	-	0	2683.44765	4	671.6173706	-2.809355389	44.174515
														CVQSNIVLLTQAFR	6	5	1	N-Term(iTRAQ8plex); C1(Methylthio)	1.167	0.807	1.519	4.58	64	0	1942.05303	2	971.5301514	-1.336825303	59.29140667
														FSPDLWGVSVCTVDGQR	2	5	1	N-Term(iTRAQ8plex); C11(Methylthio)	0.757	0.474	0.864	4.43	54	0	2216.07053	3	739.3616943	-3.475765813	54.15388
														YAIAVNDLGTEYVHR	2	5	1	N-Term(iTRAQ8plex)	0.79	1.196	1.095	4.27	60	0	2025.06296	3	675.6925049	-3.831931012	33.14694333
														VPFCLQSCVKPLK	2	5	1	N-Term(iTRAQ8plex); C4(Methylthio); C8(Methylthio); K10(iTRAQ8plex); [illegible]	1.037	0.703	1.445	4.07	36	0	2466.37685	4	617.3496704	-4.209226732	52.61587333
														QGVNNAEKFDYVMQFLNK	1	4	1	N-Term(iTRAQ8plex); K8(iTRAQ8plex); K18(iTRAQ8plex)	-	-	-	3.69	-	1	3057.63857	4	765.1651001	-6.851950906	50.31984167
														FVIPDFMSFTSHIDELYESAK	1	5	1	N-Term(iTRAQ8plex); K21(iTRAQ8plex)	-	-	-	3.56	-	0	3084.5934	4	771.9038086	2.753424483	66.094635
														FDYVMQFLNK	2	4	1	N-Term(iTRAQ8plex); K10(iTRAQ8plex)	1.182	0.434	1.345	3.55	27	0	1913.03098	3	638.3485107	-7.403081076	80.38488833
														VADYIPQLAK	1	5	1	N-Term(iTRAQ8plex); K10(iTRAQ8plex)	1.16	0.904	1.677	2.89	-	0	1726.0206	3	576.0117188	-8.892304051	59.82446
														ELVASGENK	2	5	1	N-Term(iTRAQ8plex); K9(iTRAQ8plex)	1.45	1.192	1.435	2.76	39	0	1554.88176	2	777.944519	-8.367088987	13.27805333
373432682	UBE2L3	ubiquitin-conjugating enzyme E2 L3 isoform 3	NP_001243285.1	45.08	3	3	19	122	14.1	8.92	1.103	2.499	2.478																
														TDQVIQSLIALVNDPQPEHPLR	13	3	1	N-Term(iTRAQ8plex)	-	-	-	7.69	64	0	2787.53958	3	929.8513794	3.17614632	56.32239833
														GQVCLPVISAENWKPATK	2	4	1	N-Term(iTRAQ8plex); C4(Methylthio); K14(iTRAQ8plex); K18(iTRAQ8plex)	-	-	-	5.68	44	0	2899.60415	4	725.6564941	-8.965229799	38.67971333
														IEINFPAEYPFKPPK	4	4	1	N-Term(iTRAQ8plex); K12(iTRAQ8plex); K15(iTRAQ8plex)	1.103	2.499	2.478	5.51	30	0	2702.54458	3	901.5228271	-5.388645361	39.90358167
373938452	ELOVL1	elongation of very long chain fatty acids protein 1 isoform 3	NP_001243331.1	7.07	1	1	2	198	23.3	9.66	1.077	0.877	1.183																
														CDPVDYSNSPEALR	2	2	1	N-Term(iTRAQ8plex); C1(Methylthio)	1.077	0.877	1.183	4.03	39	0	1915.89446	2	958.4508667	5.879487984	31.00884667
374081856	RAB18	ras-related protein Rab-18 isoform 3	NP_001243340.1	16.24	2	2	4	197	22.4	8.97	0.587	0.496	0.802																
														FTDDTFDPELAATIGVDFK	2	4	1	N-Term(iTRAQ8plex); K19(iTRAQ8plex)	0.53	0.584	0.837	5.77	48	0	2710.42197	3	904.1455078	5.423099304	52.62284167
														GAQGVILVYDVTR	2	4	1	N-Term(iTRAQ8plex)	0.65	0.421	0.769	4.67	47	0	1694.98052	2	847.9938965	3.660837803	35.71722667
374671775	SSBP1	single-stranded DNA-binding protein, mitochondrial precursor	NP_001243439.1	49.32	6	6	21	148	17.2	9.60	0.987	0.657	0.473																
														SGDSEVYQLGDVSQK	4	2	1	N-Term(iTRAQ8plex); K15(iTRAQ8plex)	0.876	0.795	0.619	6	58	0	2220.15726	3	740.723938	-1.437098719	27.67755167
														NPVTIFSLATNEMWR	8	2	1	N-Term(iTRAQ8plex)	1.117	0.592	0.444	5.45	84	0	2083.10483	2	1042.05603	4.763383461	62.702755
														IDYGEYMDK	3	2	1	N-Term(iTRAQ8plex); K9(iTRAQ8plex)	0.837	0.728	0.686	3.74	32	0	1741.8897	2	871.4484863	-1.731030246	37.76285167
														DVAYQYVK	2	2	1	N-Term(iTRAQ8plex); K8(iTRAQ8plex)	0.924	0.494	0.454	3.27	30	0	1593.90898	2	797.4581299	-0.425466185	19.84826667
														QATTIIADNIIFLSDQTK	2	1	1	N-Term(iTRAQ8plex); K18(iTRAQ8plex)	1.3	0.716	0.658	2.77	62	0	2600.47881	2	1300.743042	1.232874641	46.08879167
														VGQDPVLR	2	2	1	N-Term(iTRAQ8plex)	0.855	0.493	0.434	-	30	0	1187.70036	2	594.3538208	-3.903609508	15.15843333
375065825	TSPO	translocator protein	NP_001243459.1	23.08	3	3	3	169	18.8	9.36	0.733	0.729	0.488																
														ELGGFTEK	1	1	1	N-Term(iTRAQ8plex); K8(iTRAQ8plex)	0.936	0.885	0.552	-	27	0	1488.85764	3	496.9573975	3.894148547	18.78894333
														APPWVPAMGFTLAPSLGCFVGSR	1	1	1	N-Term(iTRAQ8plex); C18(Methylthio)	-	-	-	-	27	0	2711.39243	3	904.4689941	4.044737957	66.92805333
														FVHGEGLR	1	1	1	N-Term(iTRAQ8plex)	0.574	0.6	0.433	-	25	0	1218.68369	3	406.8994141	-4.91707779	17.59779
375331941	DPP3	dipeptidyl peptidase 3 isoform 2	NP_001243599.1	2.26	1	1	2	707	79.3	5.03	1.261	1.835	1.163																
														VILGSEAAQQHPEEVR	2	2	1	N-Term(iTRAQ8plex)	1.261	1.835	1.163	5.24	39	0	2067.10416	3	689.7062378	-4.606043216	21.40598833
378744165	PAF1	RNA polymerase II-associated factor 1 homolog isoform 2	NP_001243755.1	5.36	1	1	1	485	55.4	5.36	-	-	-																
														IDPNVLLDPADEKLLEEEIQAPTSSK	1	2	1	N-Term(iTRAQ8plex); K13(iTRAQ8plex); K26(iTRAQ8plex)	-	-	-	4.8	-	1	3777.10415	4	945.0314941	2.752209655	51.06243333
379643013	CLCN4	H(+)/Cl(-) exchange transporter 4 isoform 2	NP_001243873.1	0.90	1	1	2	666	74.3	7.69	1.698	3.994	2.334																
														WTLLIK	2	2	1	N-Term(iTRAQ8plex); K6(iTRAQ8plex)	1.698	3.994	2.334	-	24	0	1381.90555	3	461.3067017	2.033735566	37.22188833
379698839	CCDC51	coiled-coil domain-containing protein 51 isoform 1	NP_001243893.1	2.92	1	1	1	411	45.8	8.19	0.665	0.925	0.703																
														RPEEVALGLHHR	1	2	1	N-Term(iTRAQ8plex)	0.665	0.925	0.703	3.29	-	0	1717.97158	4	430.2483521	-2.795725456	17.24375333
380692328	DDX56	probable ATP-dependent RNA helicase DDX56 isoform 2	NP_001244118.1	6.90	3	3	5	507	57.2	9.22	1.386	0.456	0.878																
														LLQAVTDLGWSRPTLIQEK	2	2	1	N-Term(iTRAQ8plex); K19(iTRAQ8plex)	-	-	-	6.19	47	0	2776.62099	4	694.9107056	1.005209437	57.41300833
														TAAYAIPMLQLLLHR	2	2	1	N-Term(iTRAQ8plex)	1.386	0.456	0.878	4.38	47	0	2015.17472	3	672.3964233	-1.525689283	61.827685
														TAAYAIPMLQLLLHRK	1	2	1	N-Term(iTRAQ8plex); K16(iTRAQ8plex)	-	-	-	3.14	-	1	2447.4623	4	612.6210327	-6.463969075	53.55233167
383087728	SLC16A2	monocarboxylate transporter 8	NP_006508.2	2.41	1	1	2	539	59.5	5.59	0.553	0.389	0.322																
														VHEPEPTPTVETR	2	1	1	N-Term(iTRAQ8plex)	0.553	0.389	0.322	3.96	-	0	1795.94529	3	599.3199463	-2.191426901	15.193275
383872538	CD63	CD63 antigen isoform D precursor	NP_001244329.1	10.26	2	2	2	156	17.3	7.88	0.673	0.598	0.776																
														MQADFK	1	3	1	N-Term(iTRAQ8plex); M1(Oxidation); K6(iTRAQ8plex)	0.74	0.485	0.707	2.86	-	0	1363.75092	3	455.2551575	0.662043264	14.481
														VMSEFNNNFR	1	3	1	N-Term(iTRAQ8plex)	0.611	0.737	0.851	2.7	-	0	1561.76518	2	781.3862305	-5.325373227	44.03091667
385198059	NOP2	probable 28S rRNA (cytosine(4447)-C(5))-methyltransferase isoform 2	NP_001245237.1	11.21	7	7	20	812	89.2	9.23	1.572	1.201	1.237																
														ELLLSAIDSVNATSK	4	4	1	N-Term(iTRAQ8plex); K15(iTRAQ8plex)	1.47	1.288	1.414	5.59	52	0	2169.26456	3	723.7597046	2.691796859	44.30005167
														LGVTNTIISHYDGR	4	4	1	N-Term(iTRAQ8plex)	1.598	1.079	1.046	5.16	47	0	1850.00187	3	617.3388062	-2.987074269	34.53345667
														ILDMCCAPGGK	6	4	1	N-Term(iTRAQ8plex); C5(Methylthio); C6(Methylthio); K11(iTRAQ8plex)	2.229	1.498	1.344	3.96	58	0	1807.87566	2	904.4413452	-5.730201447	44.0437
														VLLDAPCSGTGVISK	2	4	1	N-Term(iTRAQ8plex); C7(Methylthio); K15(iTRAQ8plex)	2.503	1.595	1.237	3.32	49	0	2114.17929	3	705.3979492	-0.812558213	33.38772333
														GISAGAVQTAGK	1	2	1	N-Term(iTRAQ8plex); K12(iTRAQ8plex)	1.434	1.201	1.237	2.86	-	0	1667.97507	3	556.663208	-8.999251143	24.16356833
														IQDIVGILR	2	4	1	N-Term(iTRAQ8plex)	1.897	1.186	0.967	2.7	30	0	1330.83391	2	665.9205933	-1.586521464	52.44939833
														LVPTGLDFGQEGFTR	1	4	1	N-Term(iTRAQ8plex)	1.572	0.894	0.705	-	46	0	1941.03533	2	971.0213013	-1.579250463	45.75283
385719196	CLPB	caseinolytic peptidase B protein homolog isoform 3	NP_001245322.1	5.71	3	3	6	648	72.2	8.87	1.219	0.976	1.147																
														GCTALHYAVLADDYR	2	6	1	N-Term(iTRAQ8plex); C2(Methylthio)	1.071	0.974	0.991	4.04	27	0	2017.97348	3	673.3293457	-2.115211453	38.338665
														EQGIHSLEVLITR	2	2	1	N-Term(iTRAQ8plex)	1.838	0.976	1.147	3.41	30	0	1799.02823	3	600.3475952	-2.589678908	42.39901333
														HNITLLWDR	2	5	1	N-Term(iTRAQ8plex)	1.219	1.166	1.364	2.96	48	0	1471.82581	3	491.2801208	-4.42443741	29.4354
386643030	SMS	spermine synthase isoform 2	NP_001245352.1	10.54	3	3	6	313	35.3	5.02	-	-	-																
														MVTMVEIDQMVIDGCKK	2	5	1	N-Term(iTRAQ8plex); C15(Methylthio); K16(iTRAQ8plex); K17(iTRAQ8plex)	-	-	-	5.64	41	1	2898.51577	4	725.3843994	-8.326511112	61.12205333
														GDCYQVLIEDCIPVLK	2	5	1	N-Term(iTRAQ8plex); C3(Methylthio); C11(Methylthio); K16(iTRAQ8plex)	-	-	-	4.67	35	0	2508.29789	3	836.770813	5.88941805	62.948065
														MVTMVEIDQMVIDGCK	2	5	1	N-Term(iTRAQ8plex); C15(Methylthio); K16(iTRAQ8plex)	-	-	-	3.77	52	0	2466.23655	3	822.7503662	-1.227342605	57.20148
386781688	SRP68	signal recognition particle subunit SRP68 isoform 2	NP_001247431.1	7.30	3	3	4	589	66.1	7.20	0.876	2.386	1.720																
														QVPGGGGGGGSGGGGGSGGGGSGGGR	2	2	1	N-Term(iTRAQ8plex)	-	-	-	3.54	43	0	2148.00272	3	716.6724243	-2.510053051	10.61992
														FHLLSR	1	2	1	N-Term(iTRAQ8plex)	0.86	3.979	1.947	-	29	0	1076.64237	3	359.5523071	-8.78022597	21.91751
														QAATMSEVEWR	1	2	1	N-Term(iTRAQ8plex); M5(Oxidation)	0.893	1.431	1.519	-	23	0	1627.81182	2	814.4095459	4.059975983	24.11794167
386869309	NOL10	nucleolar protein 10 isoform 3	NP_001248323.1	5.49	2	2	3	638	74.5	8.38	0.646	0.527	0.589																
														CLDSEVVTFEILSDDYSK	1	4	1	N-Term(iTRAQ8plex); C1(Methylthio); K18(iTRAQ8plex)	0.371	0.257	0.356	3.96	-	0	2717.35892	3	906.4578247	2.895668671	59.16099167
														DLENLGLTHLIGSPFLR	2	5	1	N-Term(iTRAQ8plex)	1.122	1.08	0.975	3.82	27	0	2199.25266	3	733.7557373	3.950143713	53.17495667
386869374	PSMD9	26S proteasome non-ATPase regulatory subunit 9 isoform 2	NP_001248329.1	10.17	1	1	1	118	13.0	5.85	0.671	1.695	1.643																
														GLLGCNIIPLQR	1	2	1	N-Term(iTRAQ8plex); C5(Methylthio)	0.671	1.695	1.643	3.32	-	0	1646.94255	2	823.9749146	2.277056693	45.09495167
387527991	OS9	protein OS-9 isoform 7 precursor	NP_001248351.1	5.36	2	2	3	560	63.1	4.72	1.489	1.170	1.012																
														IIEELQDLGPQVWSETK	2	11	1	N-Term(iTRAQ8plex); K17(iTRAQ8plex)	1.592	1.513	1.243	5.18	69	0	2593.44376	3	865.152771	3.990230458	59.35442167
														TKDWWTYEFCYGR	1	10	1	N-Term(iTRAQ8plex); K2(iTRAQ8plex); C10(Methylthio)	1.394	0.904	0.824	3.54	-	1	2409.15635	3	803.7236328	-2.198741692	45.95459167
387528025	AARSD1	alanyl-tRNA editing protein Aarsd1	NP_001248363.1	2.67	1	1	1	412	45.5	6.42	0.359	0.742	1.721																
														GLPDDHAGPIR	1	3	1	N-Term(iTRAQ8plex)	0.359	0.742	1.721	2.81	-	0	1451.78308	3	484.5992126	-5.363480694	15.175805
388240801	LMNB2	lamin-B2	NP_116126.3	40.48	21	23	108	620	69.9	5.59	1.405	0.516	0.607																
														AGQMVTVWAAGAGVAHSPPSTLVWK	6	1	1	N-Term(iTRAQ8plex); K25(iTRAQ8plex)	1.017	0.729	0.685	6.03	52	0	3129.71328	4	783.1837769	0.126606446	47.74269833
														VKDLESLFHR	12	1	1	N-Term(iTRAQ8plex); K2(iTRAQ8plex)	1.602	0.664	0.492	5.14	47	1	1852.08938	4	463.7761536	-3.961443975	36.30326167
														LESLSYQLSGLQK	5	1	1	N-Term(iTRAQ8plex); K13(iTRAQ8plex)	1.372	0.463	0.587	4.95	34	0	2074.19236	3	692.0689697	-3.902902394	68.56297667
														TVLVNADGEEVAMR	4	1	1	N-Term(iTRAQ8plex)	1.515	0.367	0.488	4.91	58	0	1807.95134	2	904.4793091	-0.707848662	28.59417833
														DVMQQQLAEYQELLDVK	2	1	1	N-Term(iTRAQ8plex); K17(iTRAQ8plex)	1.8	0.385	0.444	4.79	58	0	2658.42534	2	1329.716309	-0.602135586	48.71583
														SEVELAAALSDKR	4	1	1	N-Term(iTRAQ8plex); K12(iTRAQ8plex)	1.475	0.564	0.678	4.57	46	1	1997.13828	4	500.0426636	0.021589215	27.95043667
														ATSSSGSLSATGR	9	1	1	N-Term(iTRAQ8plex)	1.608	0.433	0.568	4.31	71	0	1572.81072	2	786.9089966	-1.522596402	13.31308333
														ALYESELADAR	8	1	1	N-Term(iTRAQ8plex)	1.326	0.428	0.588	4.15	52	0	1541.80681	2	771.4058838	-4.430095978	21.30882333
														CQSLQEELDFR	6	1	1	N-Term(iTRAQ8plex); C1(Methylthio)	1.323	0.392	0.528	4.09	58	0	1717.81059	2	859.4105835	-3.051510901	35.61729333
														LYKLELEQTYQAK	2	1	1	N-Term(iTRAQ8plex); K3(iTRAQ8plex); K13(iTRAQ8plex)	2.548	0.734	0.756	4.03	23	1	2539.49192	3	847.1688232	0.805348719	31.31878167
														LALDMEINAYR	5	1	1	N-Term(iTRAQ8plex)	1.322	0.649	0.813	3.96	53	0	1612.87981	3	538.2937012	-0.323048658	37.27152
														EGELTVAQGR	2	1	1	N-Term(iTRAQ8plex)	1.514	0.368	0.461	3.74	38	0	1363.75273	2	682.3800049	3.227744677	21.13301667

														MAQALEELR	10	1	1	N-Term(iTRAQ8plex)	1.38	0.551	0.607	3.59	31	0	1364.74468	2	682.8782349	-1.310272573	39.57604333
														TVLVNADGEVAMR	2	1	1	N-Term(iTRAQ8plex); M13(Oxidation)	1.758	0.395	0.529	3.46	36	0	1823.9583	2	904.4793091	-0.707848662	28.59417833
														SEVELAAALSDK	2	1	1	N-Term(iTRAQ8plex); K12(iTRAQ8plex)	1.484	0.834	1.105	3.44	30	0	1841.04087	3	614.3518066	-3.690644921	47.87486167
														DVMQQQLAEYQELLDVK	2	1	1	N-Term(iTRAQ8plex); M3(Oxidation); K17(iTRAQ8plex)	1.724	1.074	0.749	3.42	33	0	2674.4152	2	1329.716309	-0.602135586	48.71583
														GLESDVAELR	4	1	1	N-Term(iTRAQ8plex)	1.412	0.523	0.595	3.2	37	0	1392.75981	2	696.8835449	-2.755734487	29.569525
														AELDEVNK	2	1	1	N-Term(iTRAQ8plex); K8(iTRAQ8plex)	1.384	0.644	0.629	3.17	50	0	1525.87383	2	763.4405518	3.673695378	14.09346833
														SVFEEVR	3	1	1	N-Term(iTRAQ8plex)	1.124	0.656	0.878	3.07	24	0	1298.68498	2	649.8461304	-3.40896974	23.14539167
														IRELEEAMAGER	2	1	1	N-Term(iTRAQ8plex)	1.234	0.71	0.737	-	31	1	1707.89994	3	569.9714966	-0.149191355	38.47317833
														LAHYIDR	1	1	1	N-Term(iTRAQ8plex)	1.263	0.808	0.796	-	31	0	1191.67133	3	397.8952942	-6.236603548	24.433745
														AGGPATPLSPTR	1	1	1	N-Term(iTRAQ8plex)	1.345	0.447	0.482	-	27	0	1428.80803	2	714.9076538	-2.267836366	16.767765
														LVEVDSSR	1	1	1	N-Term(iTRAQ8plex)	1.354	0.511	0.555	-	24	0	1208.6751	2	604.8411865	-3.098329018	19.232685
														LQIEIGK	8	1	2	N-Term(iTRAQ8plex); K7(iTRAQ8plex)	-	-	-	3.45	35	0	1408.90134	3	470.3052979	2.09312363	28.58012833
														LLEGEEER	5	11	4	N-Term(iTRAQ8plex)	-	-	-	2.96	37	0	1278.68534	3	426.8999634	0.786422343	21.17209167
388454220	CNOT1	CCR4-NOT transcription complex subunit 1 isoform c	NP_001252541.1	1.14	2	2	4	2371	266.2	7.05	0.826	1.494	1.637																
														SLDLIESLLR	2	3	1	N-Term(iTRAQ8plex)	0.875	2.43	2.575	3.76	27	0	1462.8781	2	731.942688	-0.124373615	57.41764
														AQAEQQHNPAANPTMIR	2	2	1	N-Term(iTRAQ8plex)	0.779	0.919	1.041	3.33	27	0	2181.10892	3	727.7078247	-2.191742403	16.37774333
389565487	PDCD6	programmed cell death protein 6 isoform 4	NP_001254487.1	9.09	1	1	8	121	14.4	5.29	0.847	1.041	1.000																
														LSDQFHDILIR	8	4	1	N-Term(iTRAQ8plex)	0.847	1.041	1	3.41	47	0	1660.92771	3	554.3140869	-2.850098647	35.92340333
389565511	TBC1D2	TBC1 domain family member 2A isoform 3	NP_001254501.1	1.28	1	1	2	468	54.4	7.14	0.677	0.508	0.742																
														WLVHLR	2	4	1	N-Term(iTRAQ8plex)	0.677	0.508	0.742	2.79	24	0	1127.7005	3	376.5716858	1.235100137	28.71601833
390517020	ITPA	inosine triphosphate pyrophosphatase isoform c	NP_001254552.1	16.34	1	1	4	153	16.8	7.18	-	-	-																
														GCQDFGWDPCFQPDGYEQTYAEMPK	4	3	1	N-Term(iTRAQ8plex); C2(Methylthio); C10(Methylthio); K25(iTRAQ8plex)	-	-	-	5.05	58	0	3612.57236	3	1204.862305	6.199879272	53.75632333
392513662	ILF2	interleukin enhancer-binding factor 2 isoform 2	NP_001254738.1	43.18	10	10	60	352	38.9	4.94	1.254	0.714	0.776																
														NQDLAPNSAEQASILSLVTK	2	2	1	N-Term(iTRAQ8plex); K20(iTRAQ8plex)	1.313	0.941	0.745	6.79	52	0	2707.5214	3	903.1786499	4.688848276	41.06285167
														AQDPSEVLTMLTNETGFEISSSDATVK	2	2	1	N-Term(iTRAQ8plex); K27(iTRAQ8plex)	-	-	-	5.63	64	0	3478.76877	3	1160.261108	-0.936927661	60.262125
														ILPTLEAVAALGNK	24	2	1	N-Term(iTRAQ8plex); K14(iTRAQ8plex)	1.232	0.673	0.729	5.51	70	0	2018.24089	2	1009.624084	-3.04670008	49.51331667
														VKPAPDETSFSEALLK	2	2	1	N-Term(iTRAQ8plex); K2(iTRAQ8plex); K16(iTRAQ8plex)	0.987	0.615	0.6	5.12	41	0	2644.51719	3	882.1772461	-5.795488431	29.84478333
														KLDPELHLDIK	1	2	1	N-Term(iTRAQ8plex); K1(iTRAQ8plex); K11(iTRAQ8plex)	1.516	0.74	0.926	4.63	-	1	2233.37247	5	447.4803162	1.859519894	26.90183167
														INNVIDNLIVAPGTFEVQIEEVR	2	2	1	N-Term(iTRAQ8plex)	-	-	-	4.39	29	0	2886.5969	3	962.8704834	3.105956818	66.932255
														WFEENASQSTVK	2	2	1	N-Term(iTRAQ8plex); K12(iTRAQ8plex)	0.941	0.693	0.698	4.2	24	0	2034.06699	3	678.6938477	-4.066523517	36.30392
														VLQSALAAIR	11	2	1	N-Term(iTRAQ8plex)	1.348	0.825	0.854	3.79	37	0	1345.84209	2	673.4246826	-3.59082664	49.38628333
														ILITTVPPNLR	11	2	1	N-Term(iTRAQ8plex)	1.198	0.695	0.781	3.41	37	0	1540.97502	2	770.9911499	1.409422382	35.54587167
														ILSHGGFR	3	2	1	N-Term(iTRAQ8plex)	1.166	0.822	0.89	2.83	42	0	1190.69199	3	397.5688477	-2.327706304	22.636405
392513684	OSTC	oligosaccharyltransferase complex subunit OSTC isoform 2	NP_001254746.1	14.46	1	1	12	83	9.4	7.43	0.800	0.446	0.525																
														VPFLVLECPNLK	12	3	1	N-Term(iTRAQ8plex); C8(Methylthio); K12(iTRAQ8plex)	0.8	0.446	0.525	5.42	55	0	2026.16434	3	676.0615234	0.510698753	52.27466833
392583868	SLIRP	SRA stem-loop-interacting RNA-binding protein, mitochondrial isoform 3 precursor	NP_001254793.1	55.43	4	4	12	92	10.3	10.23	1.295	0.923	0.889																
														GLGWVQFSSEEGLR	6	3	1	N-Term(iTRAQ8plex)	1.664	1.19	1.031	5.3	52	0	1868.98577	2	934.996521	2.618571141	39.33986833
														NALQQENHIIDGVK	2	3	1	N-Term(iTRAQ8plex); K14(iTRAQ8plex)	1.509	0.969	0.863	3.33	34	0	2187.22831	3	729.7476196	-2.709211135	23.76920167
														SINQPVAFVR	3	3	1	N-Term(iTRAQ8plex)	1.263	0.87	0.84	3.3	33	0	1434.83403	2	717.9206543	-2.125211824	26.652065
														CILPFDKETGFHR	1	3	1	N-Term(iTRAQ8plex); C1(Methylthio); K7(iTRAQ8plex)	1.246	0.543	1.049	3	-	1	2217.16323	4	555.0462646	-6.176174164	32.16211
393715095	TUBA1A	tubulin alpha-1A chain isoform 2	NP_001257329.1	62.74	1	19	428	416	46.3	5.08	0.882	2.274	2.887																
														EDMAALEKDYEEVGVDSVEGEGEEEGEEY	7	3	1	N-Term(iTRAQ8plex); K8(iTRAQ8plex)	0.882	2.274	2.887	3.84	34	1	3844.72446	3	1282.246338	0.743648057	56.45669833
														AYHEQLSVAEITNACFEPANQMVK	31	8	2	N-Term(iTRAQ8plex); C15(Methylthio); K24(iTRAQ8plex)	-	-	-	7.09	58	0	3347.65224	3	1116.55896	-1.79996266	51.00859333
														AVCMLSNTTAIAEAWAR	62	10	2	N-Term(iTRAQ8plex); C3(Methylthio)	-	-	-	6.73	96	0	2158.06858	3	720.0307617	0.741218088	64.75706667
														AYHEQLSVAEITNACFEPANQMVK	11	8	2	N-Term(iTRAQ8plex); C15(Methylthio); M22(Oxidation); K24(iTRAQ8plex)	-	-	-	5.67	50	0	3363.64157	3	1116.55896	-1.79996266	51.00859333
														AVCMLSNTTAIAEAWAR	4	10	2	N-Term(iTRAQ8plex); C3(Methylthio); M4(Oxidation)	-	-	-	4.95	81	0	2174.06987	3	720.0307617	0.741218088	64.75706667
														AYHEQLSVAEITNACFEPANQMVK	2	8	2	N-Term(iTRAQ8plex); Y2(iTRAQ8plex); C15(Methylthio); K24(iTRAQ8plex)	-	-	-	-	53	0	3651.85251	3	1116.55896	-1.79996266	51.00859333
														AFVHWYVGEGMEEGEFSEAR	63	12	3	N-Term(iTRAQ8plex)	-	-	-	5.85	74	0	2634.21878	2	1317.612793	-2.058288973	42.39526167
														IHFPLATYAPVISAEK	59	11	3	N-Term(iTRAQ8plex); K16(iTRAQ8plex)	-	-	-	5.36	61	0	2365.36284	2	1183.18103	-8.132975761	42.23089833
														AFVHWYVGEGMEEGEFSEAR	9	12	3	N-Term(iTRAQ8plex); M11(Oxidation)	-	-	-	5.26	51	0	2650.21103	2	1317.612793	-2.058288973	42.39526167
														FDGALNVDLTEFQTNLVPYPR	9	13	3	N-Term(iTRAQ8plex)	-	-	-	4.9	86	0	2713.41611	2	1357.209961	-0.485014693	60.02287167
														QLFHPEQLITGK	34	12	3	N-Term(iTRAQ8plex); K12(iTRAQ8plex)	-	-	-	4.6	61	0	2019.17363	2	1010.090454	-5.507063237	33.54091833
														VGINYQPPTVVPGGDLAK	12	13	3	N-Term(iTRAQ8plex); K18(iTRAQ8plex)	-	-	-	4.23	54	0	2433.38237	2	1217.194458	-5.996553988	32.76091333
														HGKYMACCLLYR	2	9	3	N-Term(iTRAQ8plex); K3(iTRAQ8plex); C7(Methylthio); C8(Methylthio)	-	-	-	3.64	30	1	2158.07802	4	540.2749634	3.137206139	39.16075667
														LDHKFDLMYAK	1	14	3	N-Term(iTRAQ8plex); K4(iTRAQ8plex); K11(iTRAQ8plex)	-	-	-	3.35	-	1	2293.29328	5	459.4644775	-9.109220726	30.17153333
														YMACCLLYR	21	11	3	N-Term(iTRAQ8plex); C4(Methylthio); C5(Methylthio)	-	-	-	3.28	56	0	1531.6823	3	511.2360229	1.940530286	56.21411833
														QLFHPEQLITGKEDAANNYAR	2	11	3	N-Term(iTRAQ8plex); K12(iTRAQ8plex)	-	-	-	3.06	-	1	3023.60024	4	756.6555176	-5.199386906	33.902205
														FDLMYAK	7	15	3	N-Term(iTRAQ8plex); K7(iTRAQ8plex)	-	-	-	2.97	26	0	1495.82952	2	748.4181519	-9.950474816	33.88415667
														EDAANNYAR	5	13	3	N-Term(iTRAQ8plex)	-	-	-	2.88	49	0	1327.64682	3	443.220459	-5.715071764	12.21192333
														FDGALNVDLTEFQTNLVPYPR	1	13	3	N-Term(iTRAQ8plex); Y19(iTRAQ8plex)	-	-	-	-	52	0	3017.62424	2	1357.209961	-0.485014693	60.02287167
														VGINYQPPTVVPGGDLAK	1	13	3	N-Term(iTRAQ8plex); Y5(iTRAQ8plex); K18(iTRAQ8plex)	-	-	-	-	38	0	2737.57944	2	1217.194458	-5.996553988	32.76091333
														NLDIERPTYTNLNR	1	13	3	N-Term(iTRAQ8plex)	-	-	-	-	33	0	2023.07659	2	1012.041931	-5.365894395	24.47497167
														IHFPLATYAPVISAEK	2	11	3	N-Term(iTRAQ8plex); Y8(iTRAQ8plex); K16(iTRAQ8plex)	-	-	-	-	32	0	2669.55459	2	1183.18103	-8.132975761	42.23089833
														AFVHWYVGEGMEEGEFSEAR	9	12	3	N-Term(iTRAQ8plex); Y6(iTRAQ8plex)	-	-	-	-	31	0	2938.41794	2	1317.612793	-2.058288973	42.39526167
														YMACCLLYR	2	11	3	N-Term(iTRAQ8plex); M2(Oxidation); C4(Methylthio); C5(Methylthio)	-	-	-	-	28	0	1547.67998	3	511.2360229	1.940530286	56.21411833
														YMACCLLYR	1	11	3	Y1(iTRAQ8plex); N-Term(iTRAQ8plex); C4(Methylthio); C5(Methylthio)	-	-	-	-	23	0	1835.88645	3	511.2360229	1.940530286	56.21411833
														TIGGGDDSFNTFFSETGAGK	16	8	2	N-Term(iTRAQ8plex); K20(iTRAQ8plex)	-	-	-	5.54	81	0	2616.29961	2	1308.653442	-1.625637355	42.13857333
														TIQFVDWCPTGFK	40	9	2	N-Term(iTRAQ8plex); C8(Methylthio); K13(iTRAQ8plex)	-	-	-	5.26	54	0	2196.13335	2	1098.570313	-4.951393324	45.72812167
														AVFVDLEPTVIDEVR	8	6	2	N-Term(iTRAQ8plex)	-	-	-	4.5	75	0	2006.10771	2	1003.557495	-1.753487912	50.28440167
														DVNAAIATIK	6	8	2	N-Term(iTRAQ8plex); K10(iTRAQ8plex)	-	-	-	3.78	42	0	1623.97629	3	541.9969482	-7.83251794	28.63752667
393715119	APOBEC3B	DNA dC-_dU-editing enzyme APOBEC-3B isoform b	NP_001257340.1	5.88	1	1	2	357	43.1	5.91	1.312	1.935	1.724																
														QGCPFQPWDGLEEHSQALSGR	2	2	1	N-Term(iTRAQ8plex); C3(Methylthio)	1.312	1.935	1.724	5.59	40	0	2692.25736	3	898.0906372	0.860271061	42.28880833
394025725	ACADVL	very long-chain specific acyl-CoA dehydrogenase, mitochondrial isoform 4	NP_001257377.1	31.78	14	14	35	579	62.5	7.25	1.275	0.669	0.622																
														AGLGSGLSLSGLVHPELSR	2	5	1	N-Term(iTRAQ8plex)	0.878	0.751	0.611	6.22	61	0	2154.21073	3	718.7417603	-3.604005951	40.17030667
														ASNTAEVFFDGVR	4	7	1	N-Term(iTRAQ8plex)	1.298	0.498	0.532	5.16	59	0	1716.89104	2	858.9491577	2.992332985	34.08844167
														ALEQFATVVEAK	4	7	1	N-Term(iTRAQ8plex); K12(iTRAQ8plex)	1.062	0.681	0.643	5.13	64	0	1914.11003	2	957.5586548	-2.950901249	36.92503333
														EGMAALQSDPWQQELYR	2	7	1	N-Term(iTRAQ8plex)	2.58	2.238	1.32	5.03	40	0	2326.142	3	776.0521851	-0.859523716	38.77891667
														IHNFGLIQEK	2	7	1	N-Term(iTRAQ8plex); K10(iTRAQ8plex)	0.895	0.823	0.792	4.89	39	0	1807.06304	2	904.0351563	-3.107484293	24.26771167
														LWISNGGLADIFTVFAK	2	7	1	N-Term(iTRAQ8plex); K17(iTRAQ8plex)	1.229	0.522	0.635	4.79	33	0	2460.41265	2	1230.709961	0.603132569	65.75811167
														VPSENVLGEVGSGFK	2	7	1	N-Term(iTRAQ8plex); K15(iTRAQ8plex)	1.318	0.878	0.68	4.64	42	0	2127.1959	3	709.7368164	2.465531623	29.59159
														VTDECIQIMGGMGFMK	4	7	1	N-Term(iTRAQ8plex); C5(Methylthio); K16(iTRAQ8plex)	1.343	0.7	0.534	4.31	41	0	2414.17508	3	805.3965454	-4.99521332	54.99323667
														IFGSEAAWK	2	7	1	N-Term(iTRAQ8plex); K9(iTRAQ8plex)	1.279	0.719	0.706	3.9	33	0	1616.90952	3	539.6413574	-9.995787757	31.296205
														TPVTDPATGAVK	2	7	1	N-Term(iTRAQ8plex); K12(iTRAQ8plex)	1.321	0.504	0.525	3.84	65	0	1765.02104	2	883.0141602	-5.987241647	15.39646833
														MLCDTWCIEAAAR	2	7	1	N-Term(iTRAQ8plex); C3(Methylthio); C7(Methylthio)	1.09	0.669	0.622	3.78	36	0	1878.84111	2	939.9241943	3.416335652	54.95957
														GFGGITHGPPEK	1	7	1	N-Term(iTRAQ8plex); K12(iTRAQ8plex)	0.686	0.557	0.552	3.67	-	0	1805.01395	3	602.3428345	-1.485380227	18.42106833
														GIVNEQFLLQR	2	5	1	N-Term(iTRAQ8plex)	0.999	0.82	0.678	3.44	42	0	1620.94059	3	540.9850464	1.884467687	33.68882667
														ITAFVVER	4	7	1	N-Term(iTRAQ8plex)	1.497	0.661	0.623	3.27	43	0	1238.74285	2	619.871521	-4.267195604	25.89065
394582093	MCM3	DNA replication licensing factor MCM3 isoform 1	NP_002379.3	13.60	8	8	20	853	95.8	5.77	1.138	2.969	2.599																
[truncated: 826,827 more chars]
